# Supplementary material for: Mosquito genomes are frequently invaded by transposable elements through horizontal transfer
Source: PLoS Genet. 2020 Nov 30;16(11):e1008946. doi: 10.1371/journal.pgen.1008946 (PMC7728395; doi:10.1371/journal.pgen.1008946)
Supplement: S1 File — (DOCX) [file pgen.1008946.s001.docx]

**Supplementary methods**

**REPET execution details**

Through the use of this pipeline, we performed auto-alignment with BLASTER and a structural search with LTRharvest (--struct option). The whole-genome assembly was used as input of pipeline for all genomes of the *Anopheles* genus. However, due to the high density of TEs and the large genome size of the *C. quinquefasciatus*, *Ae. aegypti* e *Ae. albopictus*, we needed to perform a random sub-sampling of the contigs/scaffolds of these genomes corresponding to one-third of these genomes. In the second step, we use the algorithms RECON, Piler, Grouper, and MCL. After that, the clusters were aligned by MAP. After getting a consensus, the PASTEC module of TEdenovo was used to remove redundancy and classify TEs. Through this tool, we performed blastx and tblastx against the Repbase REPET version (RepBase20.05_REPET) and ribosomal mosquito sequences. We also search for domains using Pfam. At the end of the pipeline, a filtering step that eliminated potential multigene mosquito families and microsatellite regions was performed.

**Resolving HTT signals in low-copy number structural variants of TEs and removing redundancy between TEs involved in HTTs and identified by different methodologies**

Since low-copy number TEs may not be identified by the *de novo* method, we performed a clustering with all copies of TEs that were characterized by the homology approach. This process followed the same clustering methodology used in TEdenovo data. Redundancy was removed between the new homology sequences and the *de novo* derived sequences using the cd-hit-est-2d software. Only copies that had no more than 80% identity at 80% of minor sequence coverage and 50% major sequence coverage (allows small fragments to map to better-assembled consensus) relative to TEdenovo consensus were selected for HTT analysis. These copies were clustered individually for each species with cd-hit-est following parameter 80-80. The representative copy of each cluster was then clustered among all species with the same previous parameters. The same ORF extraction and alignment procedure was performed, each alignment was subjected to analysis with VHICA.

**Evolutionary rate calculations**

The dS value between the transferring TEs of a pair of species was calculated by VHICA. The calculation of the evolutionary rate of culicids was performed using the same formula used to date the transfers. For this we used as time **T** fossil dating of some ancestors of each group:  *Anopheles dominicanus* (ancestor of subfamily Anophelinae, used to calculate the evolutionary rate to date transfers within the genus Anopheles, age = 34 million years) [1], *Paleoculicis minutus* (ancestor of subfamily Culicinae, age = 79 million years) [2], *Burmaculex antiquus* (ancestor from the Culicidae family, used to calculate the evolutionary rate to date transfers that occur from the genus *Anopheles* to the genus *Aedes* or *Culex*, age = 99 million years) [3]. The **k** value was considered the central value among all the values of all dS obtained from each species pair. This information was extracted from the files generated by the VHICA package using pairwise comparisons between species. Additionally, we calculate the interquartile range as the error rate of the evolution rate estimates.

**REFERENCES**

1. Zavortink TJ, Poinar GO. Anopheles ( Nyssorhynchus ) dominicanus sp . n . ( Diptera : Culicidae ) from Dominican Amber. Ann Entomol Soc Am. 2000;93: 1230–1235.

2. Poinar J, Zavortink TJ, Pike T, Johnston PA. Paleoculicis minutus (Diptera: Culicidae) n. gen., n. sp., from Cretaceous Canadian amber, with a summary of described fossil mosquitoes. Acta Geol Hisp. 2000;35: 119–128.

3. Borkent A, Grimaldi DA. The Earliest Fossil Mosquito (Diptera: Culicidae), in Mid-Cretaceous Burmese Amber. Ann Entomol Soc Am. 2006;97: 882–888. doi:10.1603/0013-8746(2004)097[0882:tefmdc]2.0.co;2
