# Supplementary material for: Mosquito genomes are frequently invaded by transposable elements through horizontal transfer
Source: PLoS Genet. 2020 Nov 30;16(11):e1008946. doi: 10.1371/journal.pgen.1008946 (PMC7728395; doi:10.1371/journal.pgen.1008946)
Supplement: S2 File — Figures of VHICA graphs for each case of horizontal transposon transfer detected. (PDF) [file pgen.1008946.s002.pdf]

Bel-Pao .... 2
Copia .... 15
Gypsy .... 22
I .... 46
Jockey .... 49
L1 .... 60
R2 .... 61
RTE .... 62
CACTA .... 66
PHIS .... 67
hAT .... 68
P .... 69
PiggyBac .... 70
Sola .... 72
Tc1-Mariner .... 72
Transib .... 87

## Bel-Pao

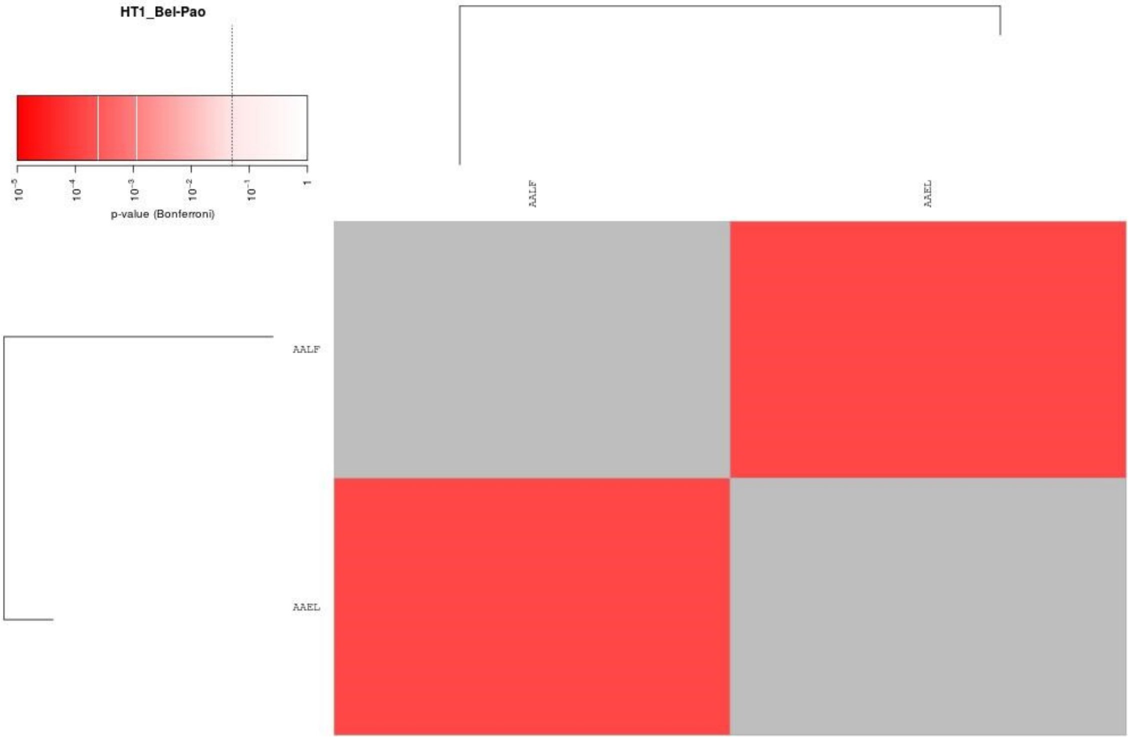
HT1_Bel-Pao
p-value (Bonferroni)
AALF
AAEL
AALF
AAEL

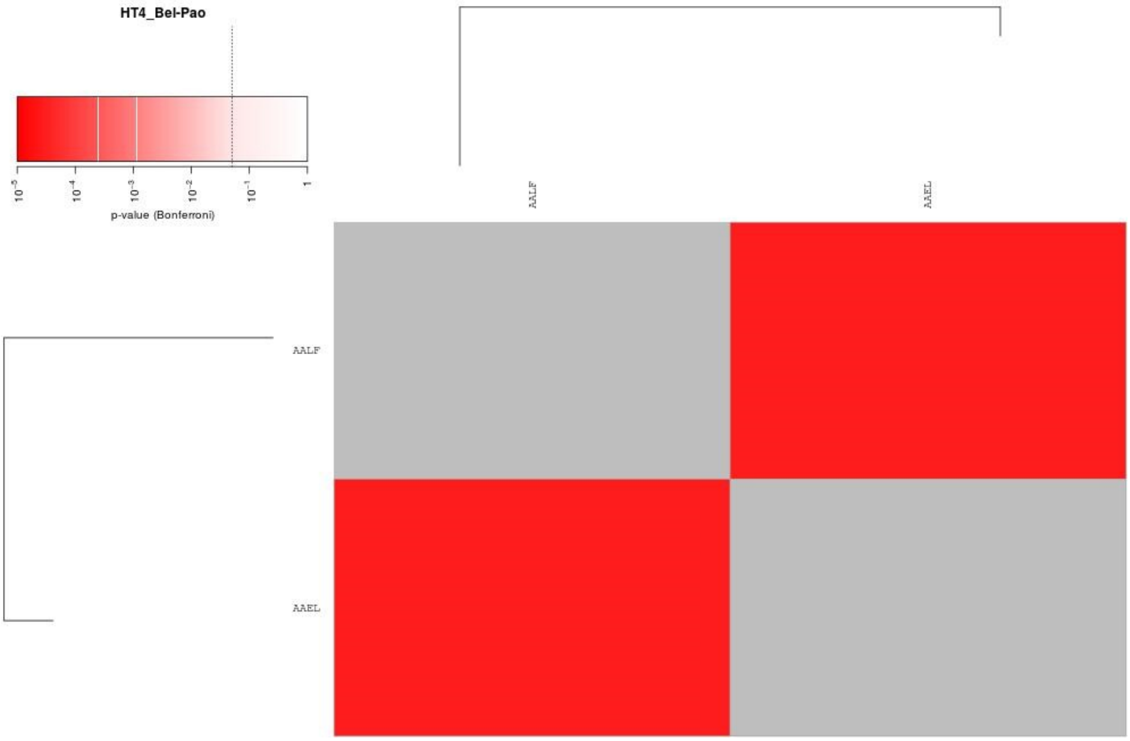
HT4_Bel-Pao
p-value (Bonferroni)
AALF
AAEL
AALF
AAEL

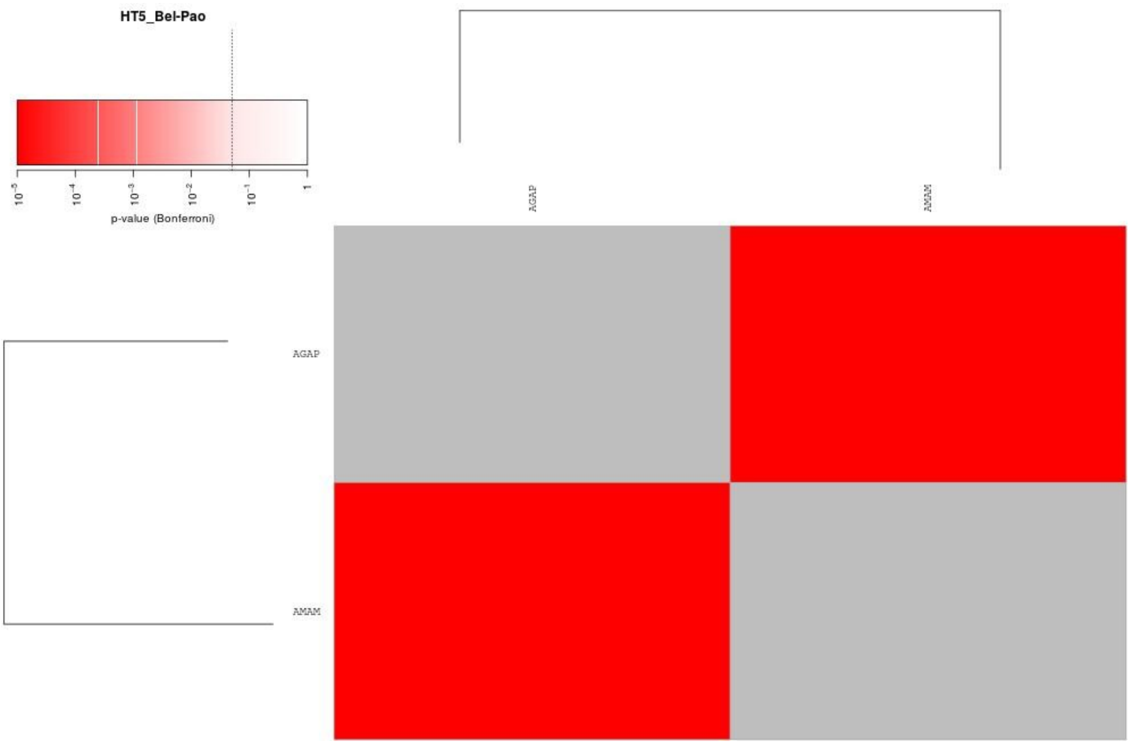
HT5_Bel-Pao
p-value (Bonferroni)
AGAP
AMAM
AGAP
AMAM

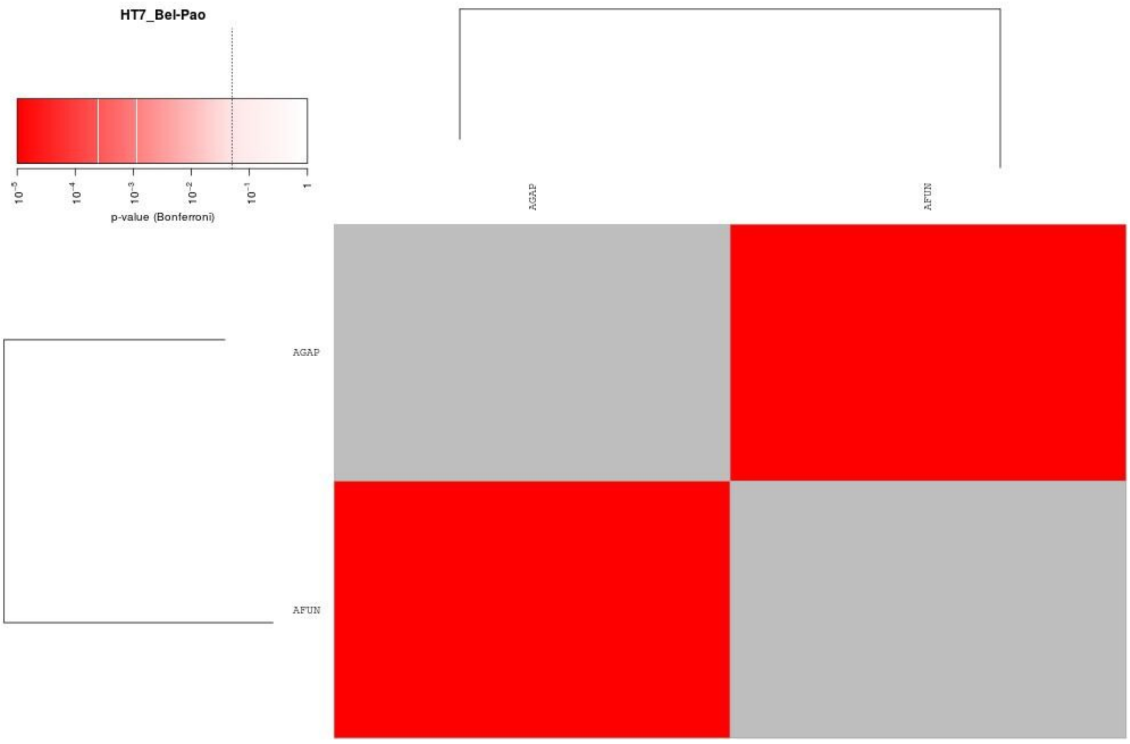
HT7_Bel-Pao
p-value (Bonferroni)
AGAP
AFUN
AGAP
AFUN

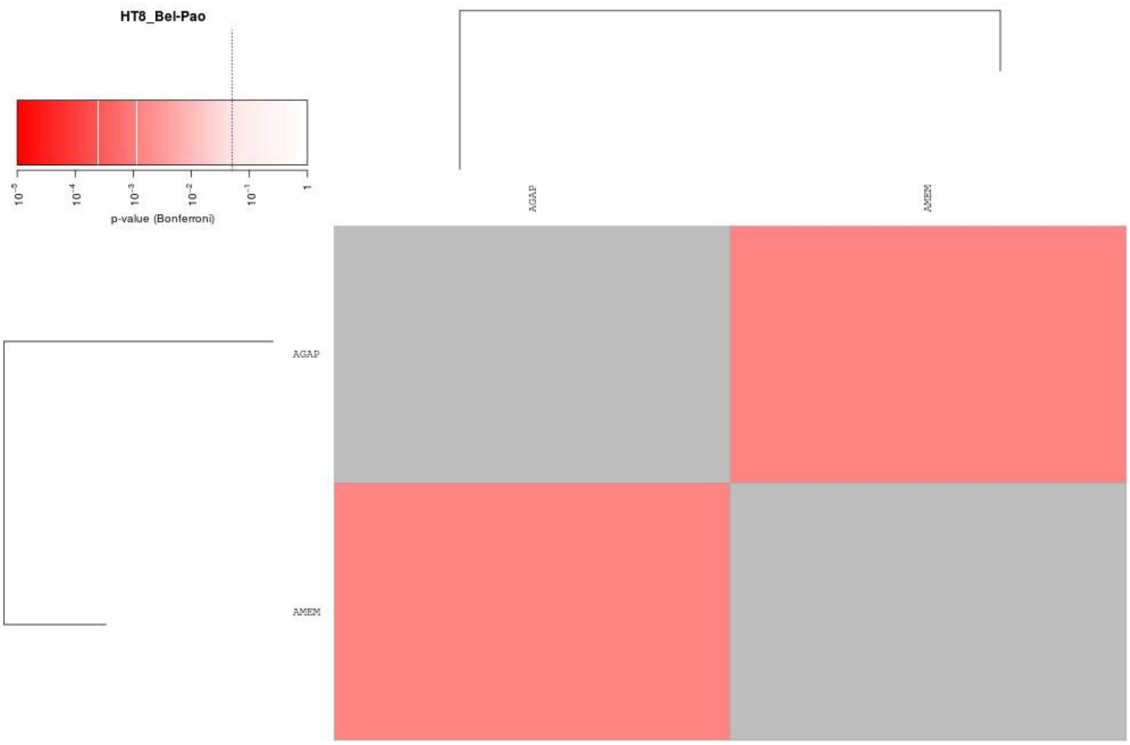
HT8_Bel-Pao
p-value (Bonferroni)
AGAP
AMEM
AGAP
AMEM

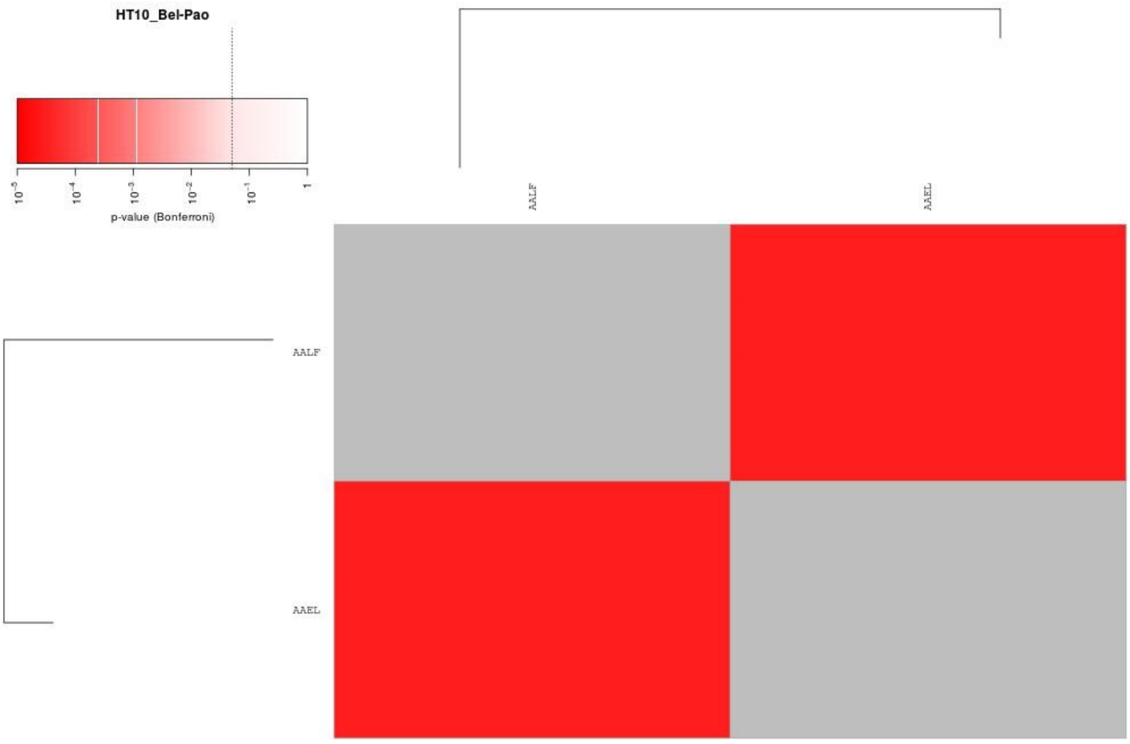
HT10_Bel-Pao
p-value (Bonferroni)
AALF
AAEL
AALF
AAEL

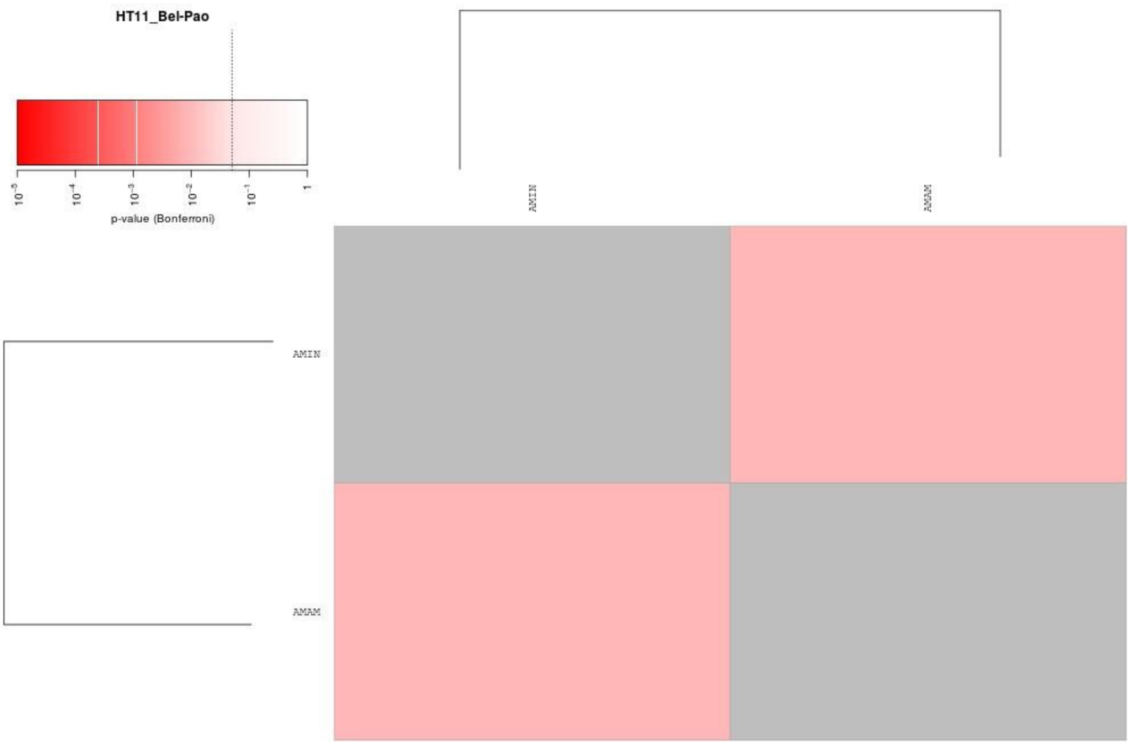
HT11_Bel-Pao
p-value (Bonferroni)
AMIN
AMAM
AMIN
AMAM

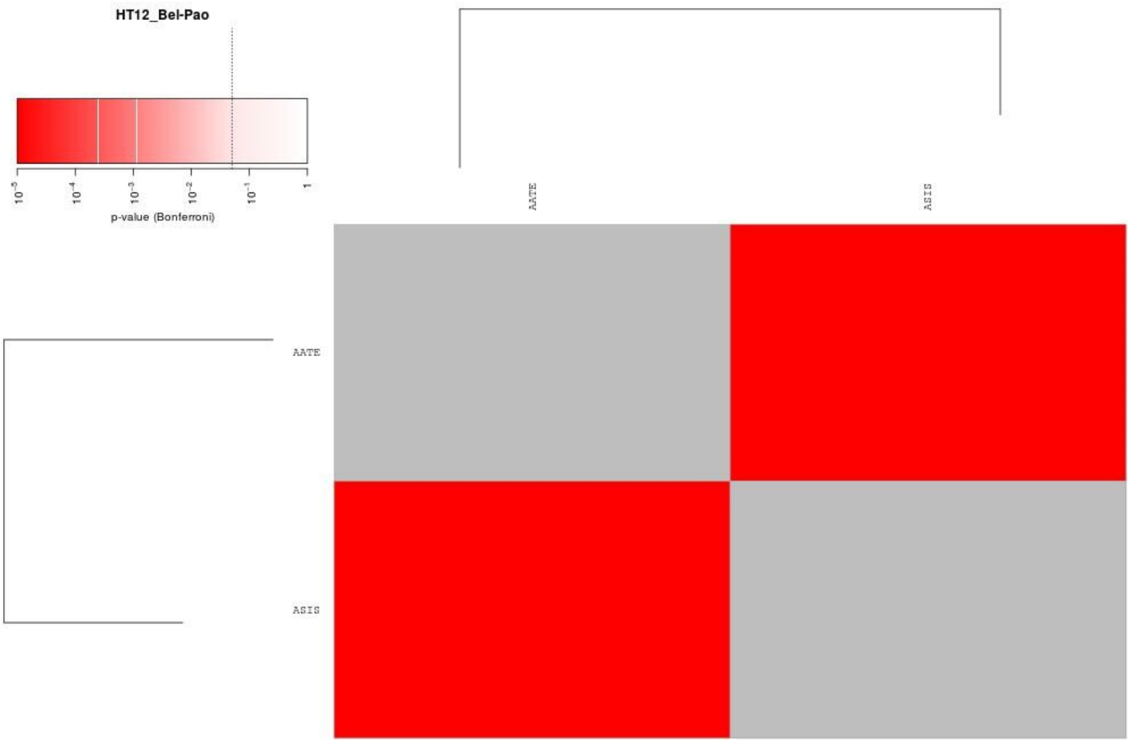
HT12_Bel-Pao
p-value (Bonferroni)
AATE
ASIS
AATE
ASIS

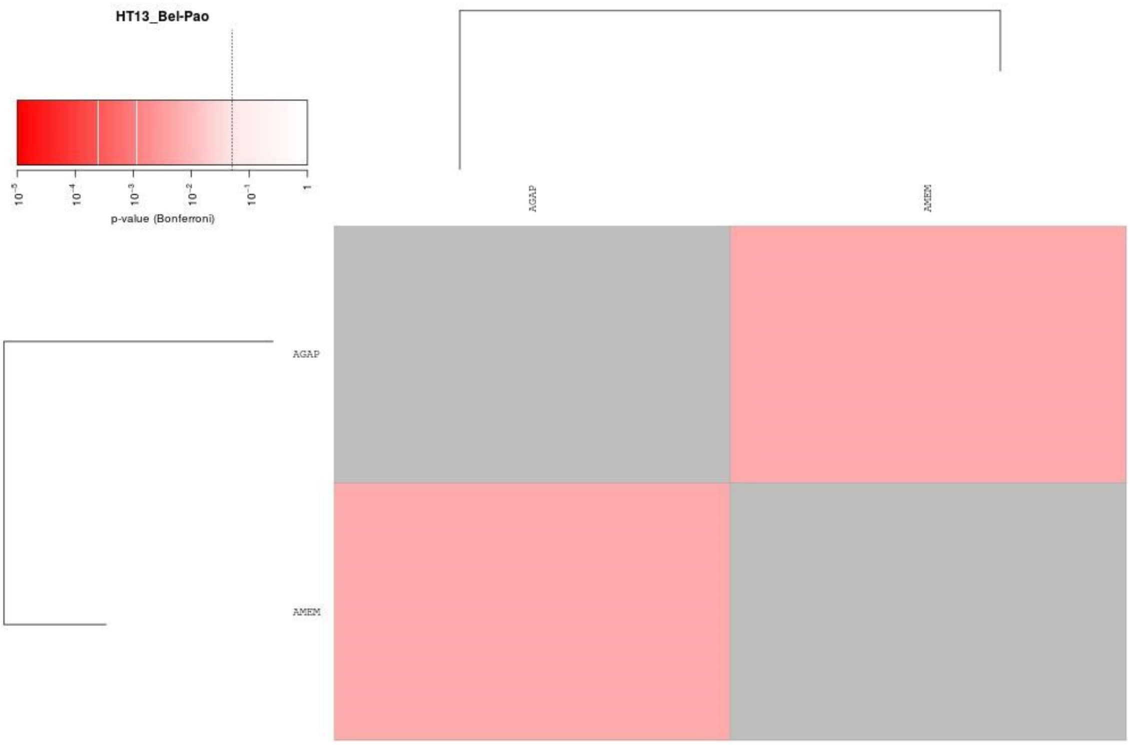
HT13_Bel-Pao
p-value (Bonferroni)
AGAP
AMEM
AGAP
AMEM

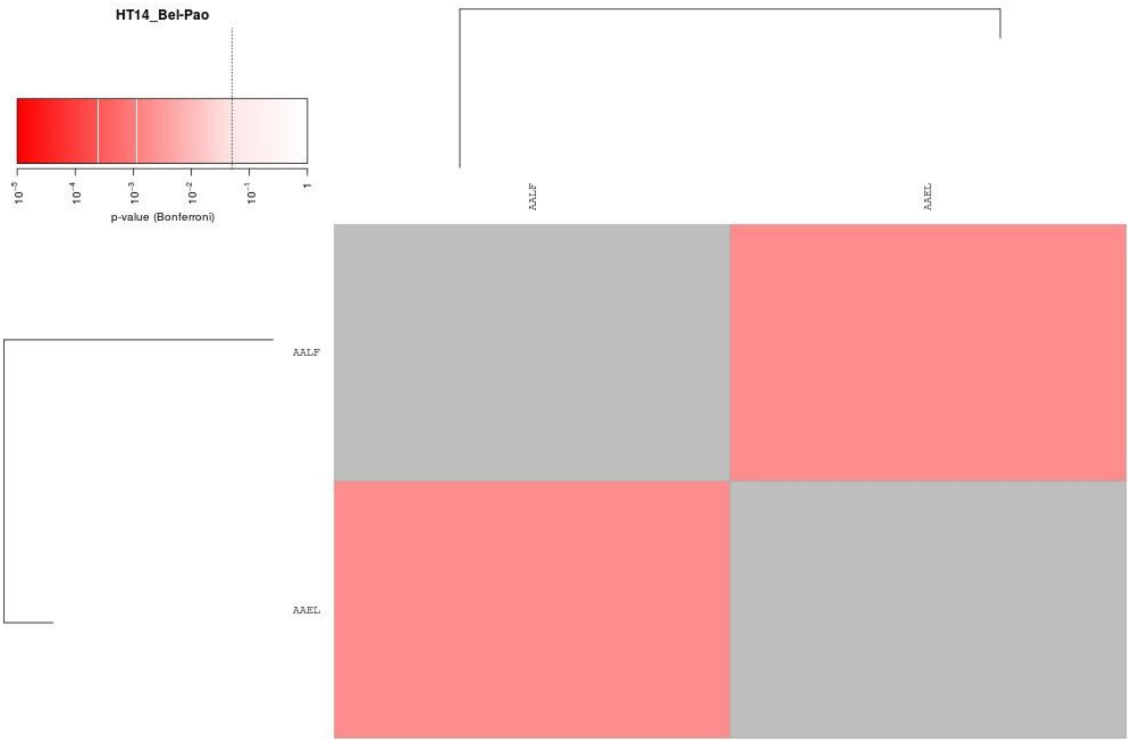
HT14_Bel-Pao
p-value (Bonferroni)
AALF
AAEL
AALF
AAEL

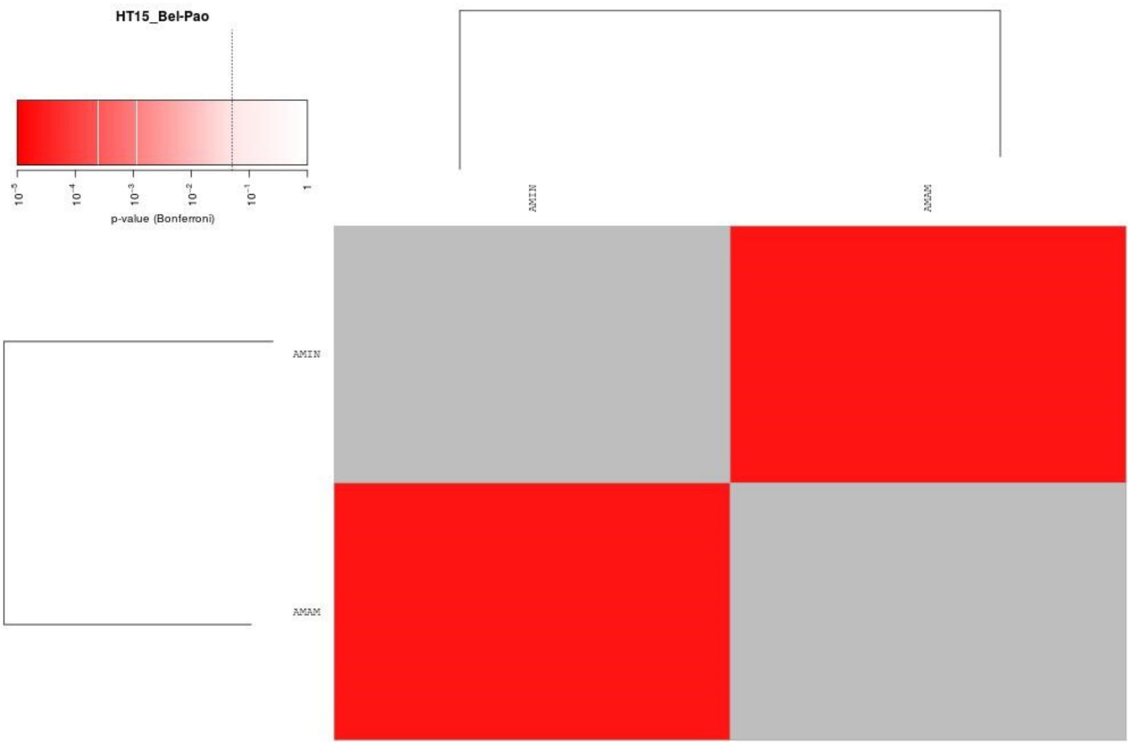
HT15_Bel-Pao
p-value (Bonferroni)
AMIN
AMAM
AMIN
AMAM

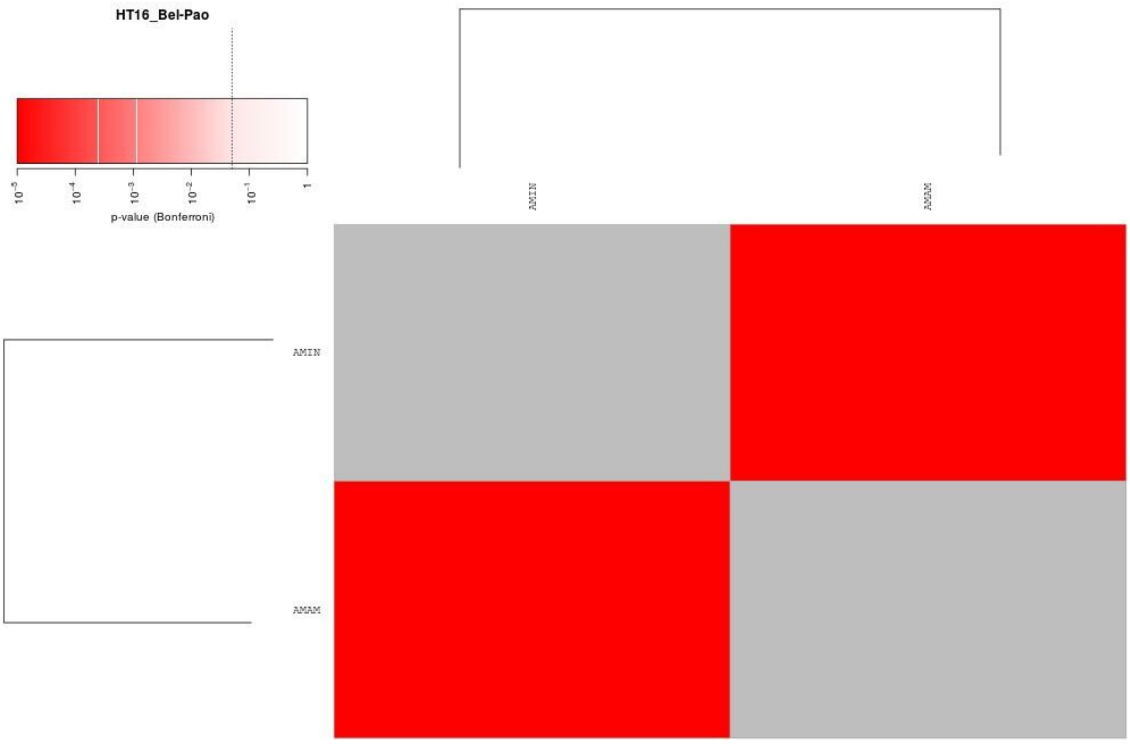
HT16_Bel-Pao
p-value (Bonferroni)
AMIN
AMAM
AMIN
AMAM

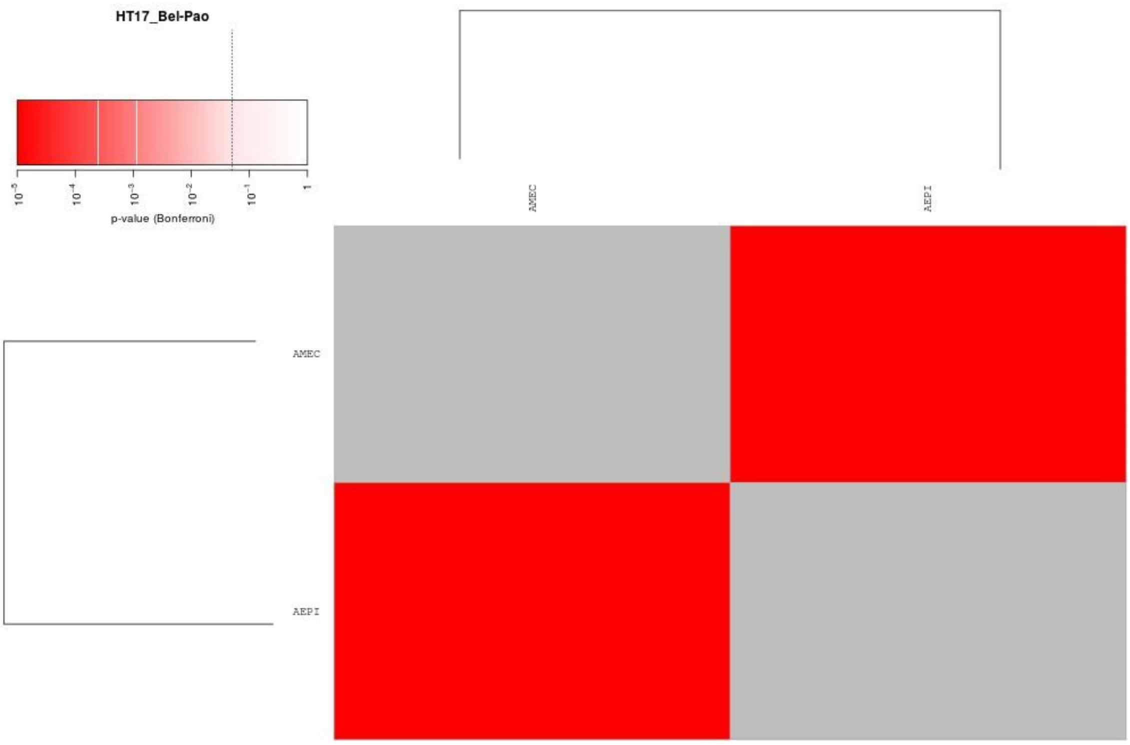
HT17_Bel-Pao
p-value (Bonferroni)
AMEC
AEPI

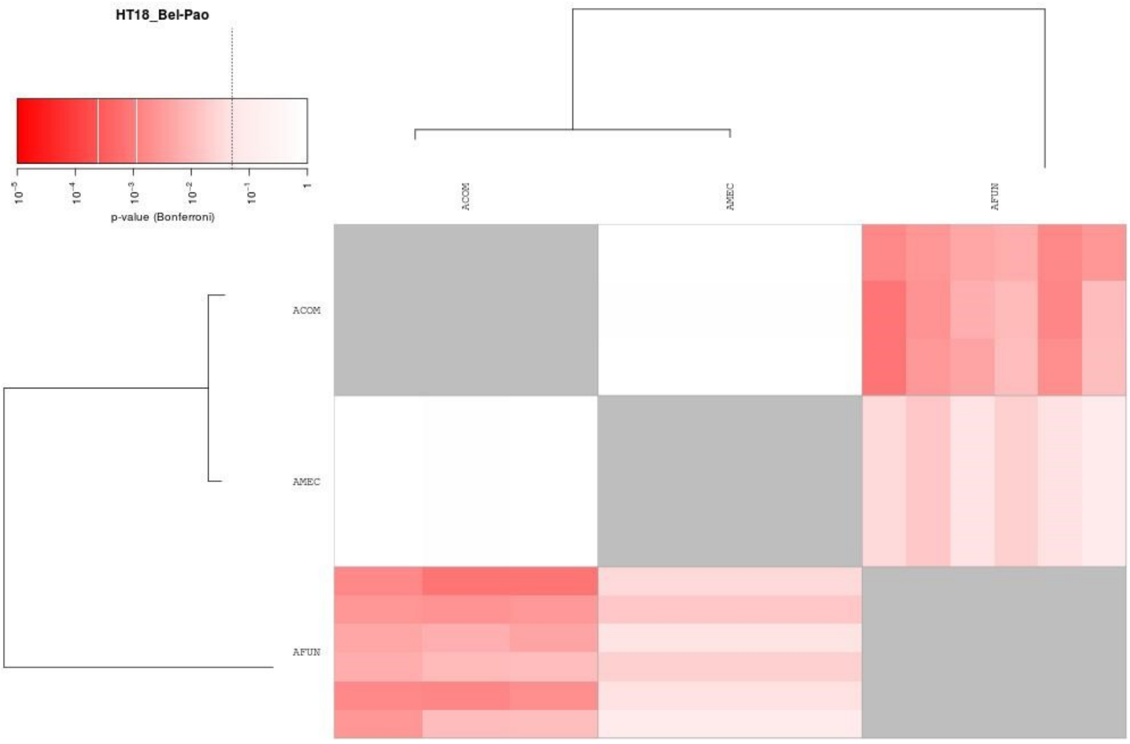
HT18_Bel-Pao
p-value (Bonferroni)
ACOM
AMEC
AFUN

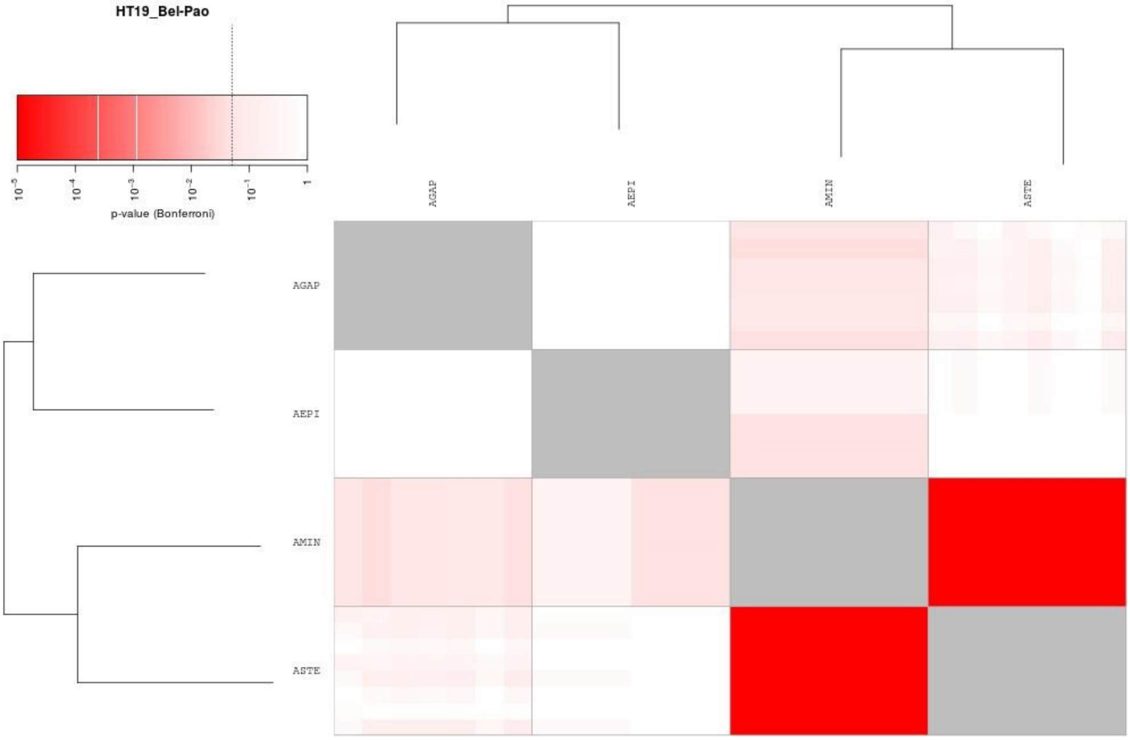
HT19_Bel-Pao
p-value (Bonferroni)
AGAP
AEPI
AMIN
ASTE

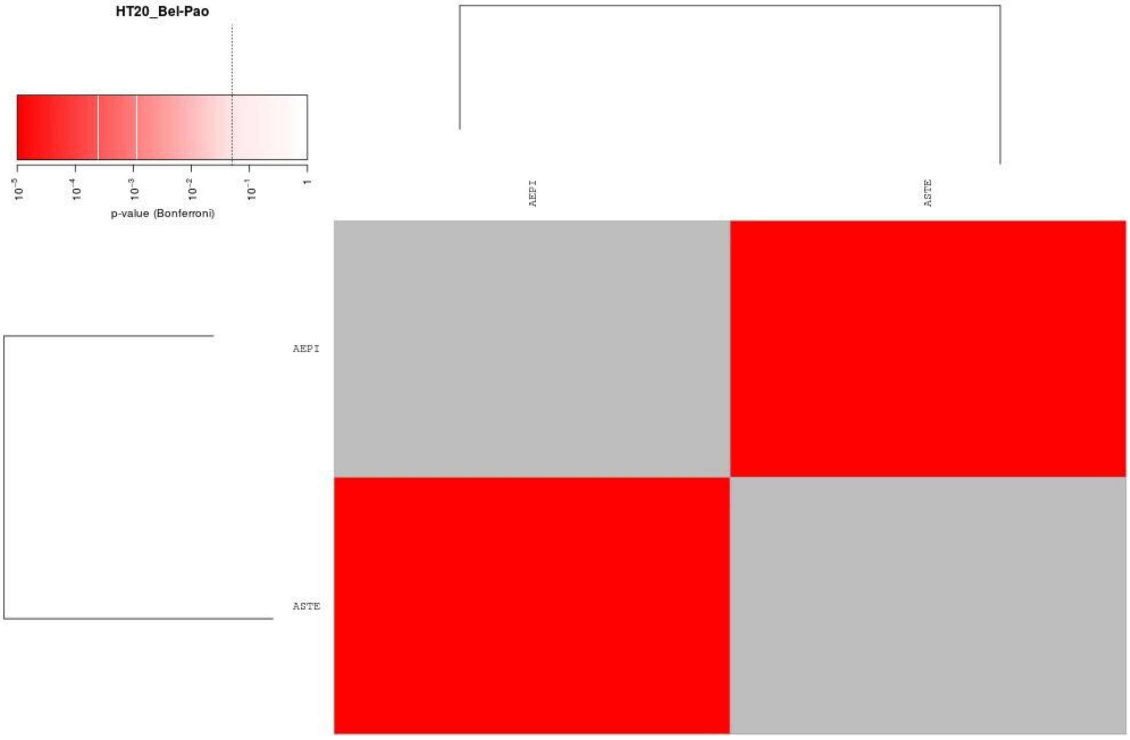
HT20_Bel-Pao
p-value (Bonferroni)
AEPI
ASTE

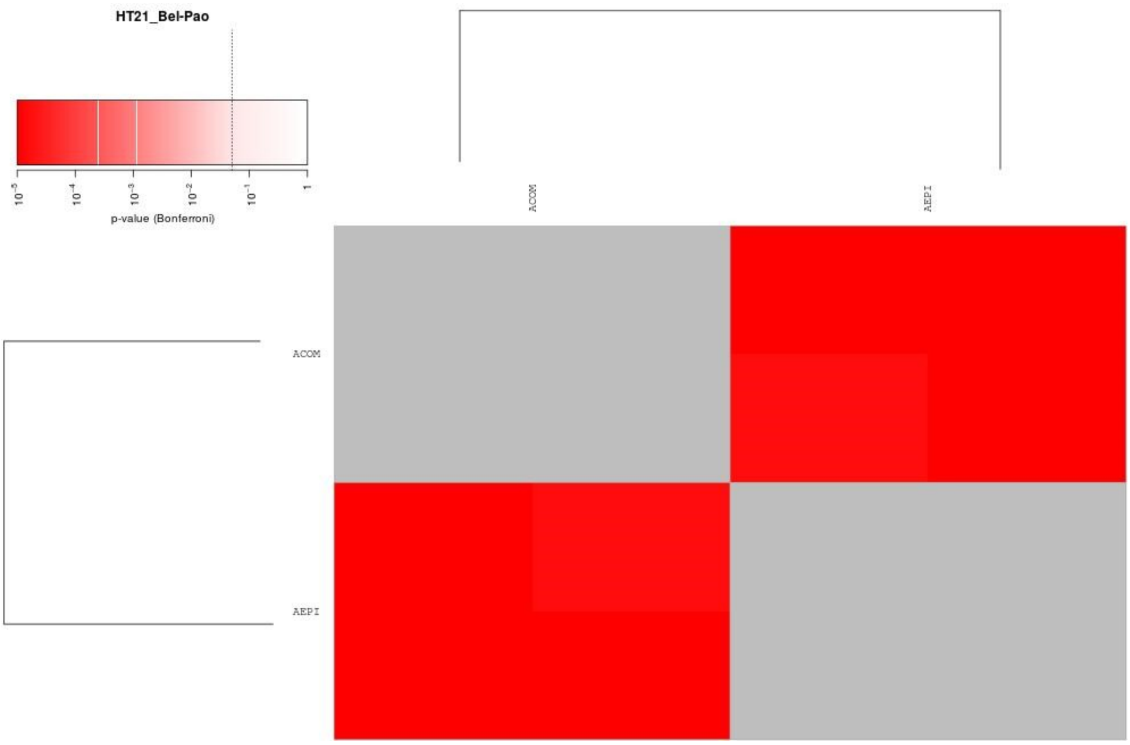
HT21_Bel-Pao
p-value (Bonferroni)
ACOM
AEPI
ACOM
AEPI

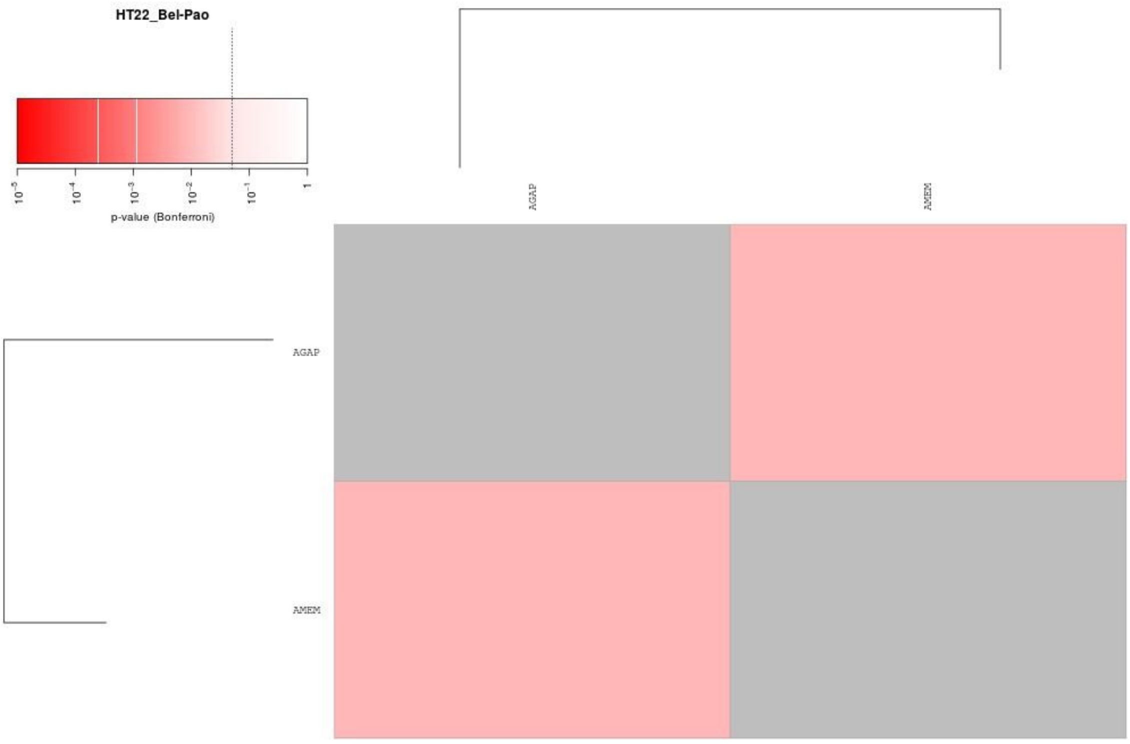
HT22_Bel-Pao
p-value (Bonferroni)
AGAP
AMEM
AGAP
AMEM

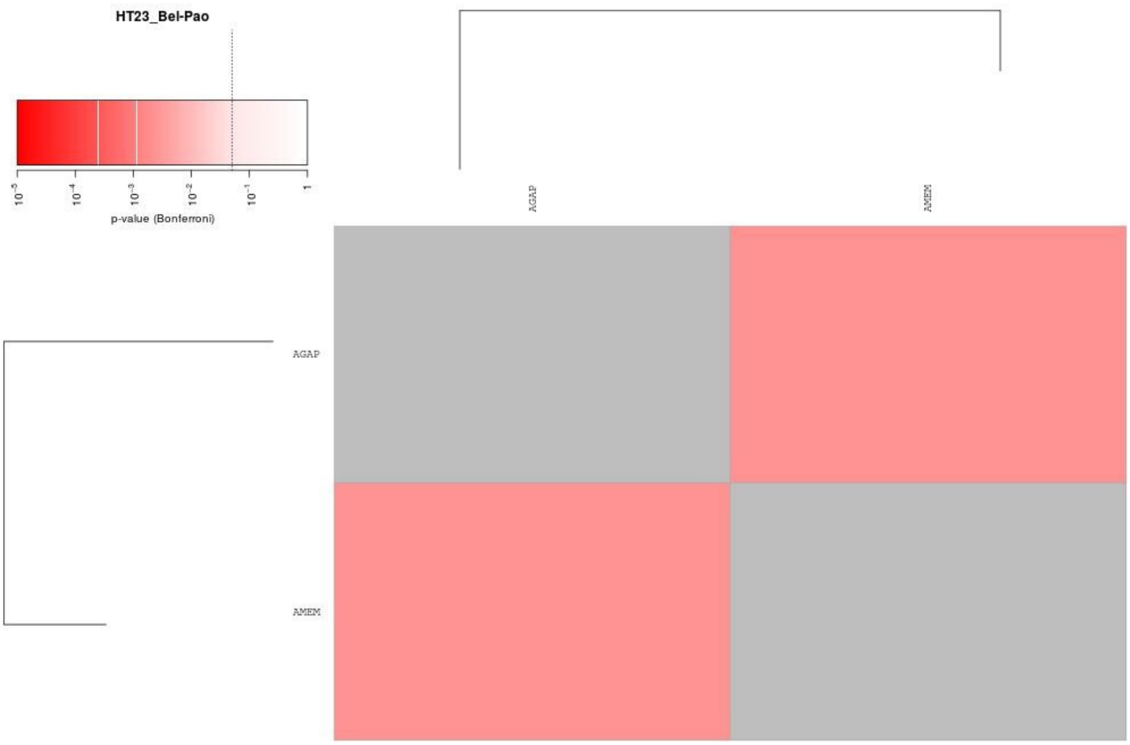
HT23_Bel-Pao
p-value (Bonferroni)
AGAP
AMEM
AGAP
AMEM

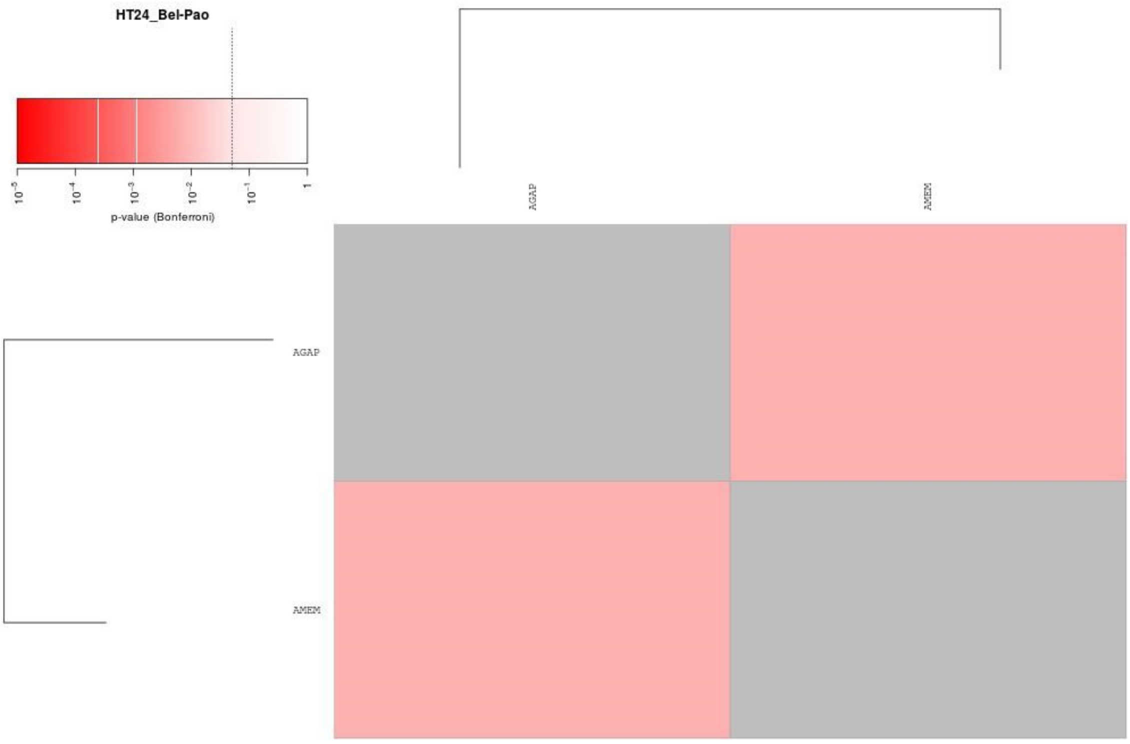
HT24_Bel-Pao
p-value (Bonferroni)
AGAP
AMEM
AGAP
AMEM

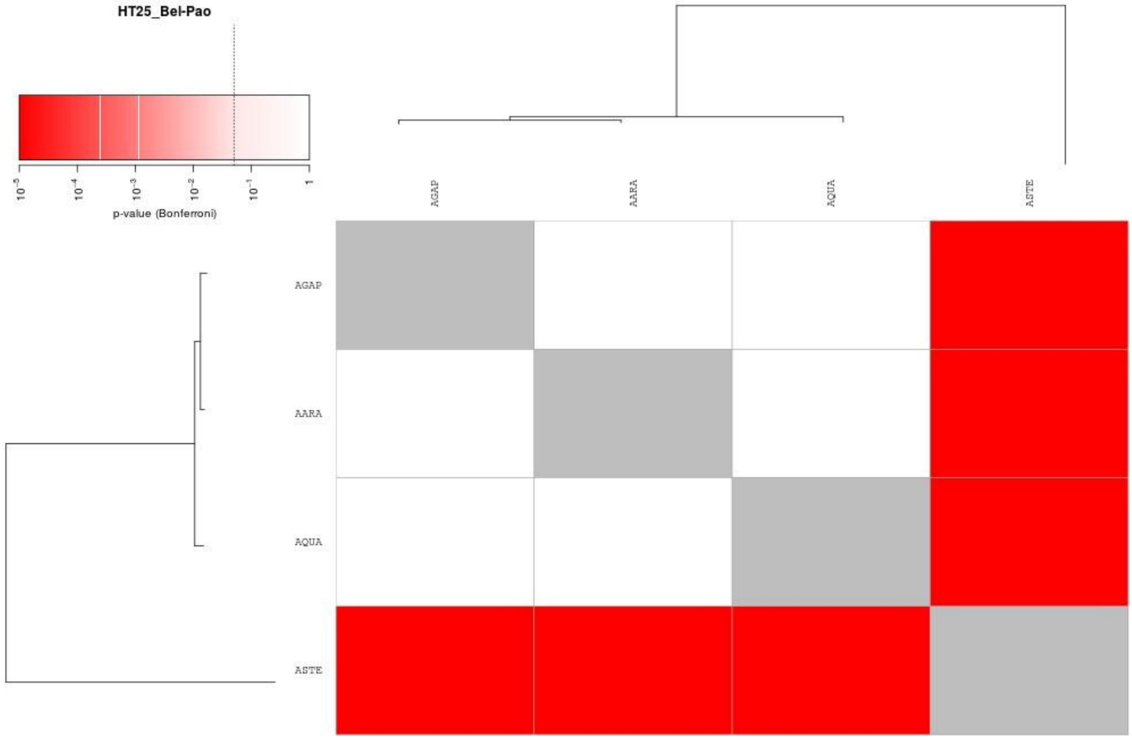
HT25_Bel-Pao
p-value (Bonferroni)
AGAP
AARA
AQUA
ASTE

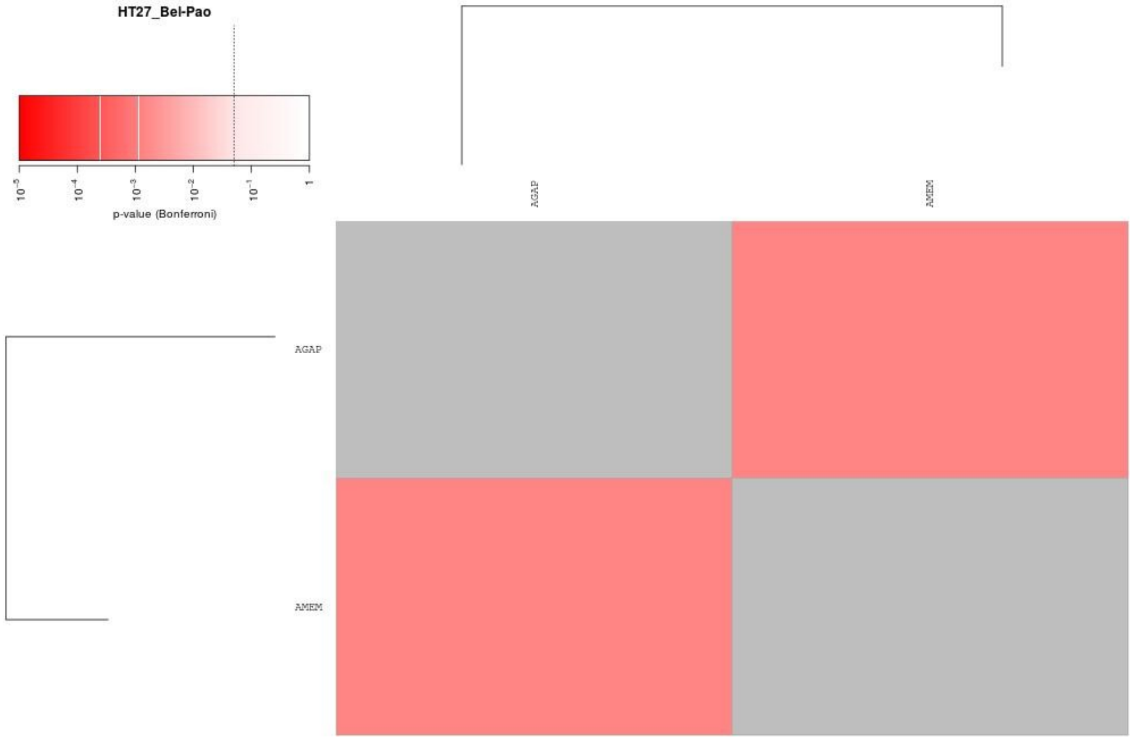
HT27_Bel-Pao
p-value (Bonferroni)
AGAP
AMEM

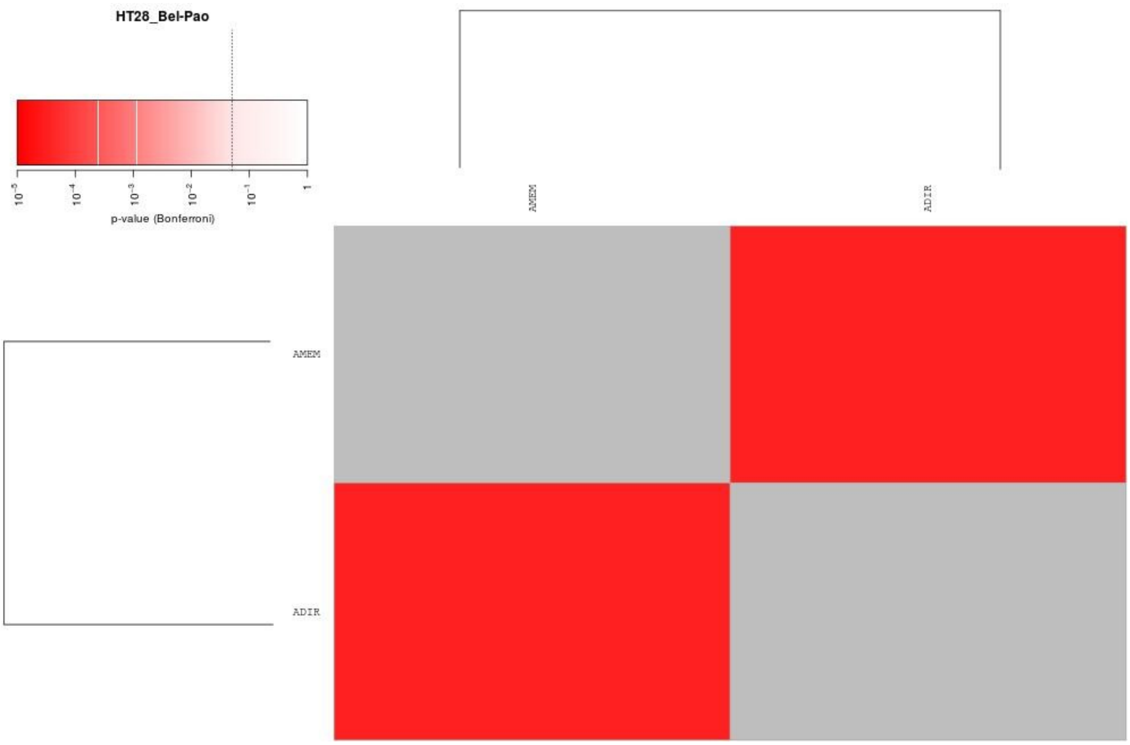
HT28_Bel-Pao
p-value (Bonferroni)
AMEM
ADIR
AMEM
ADIR

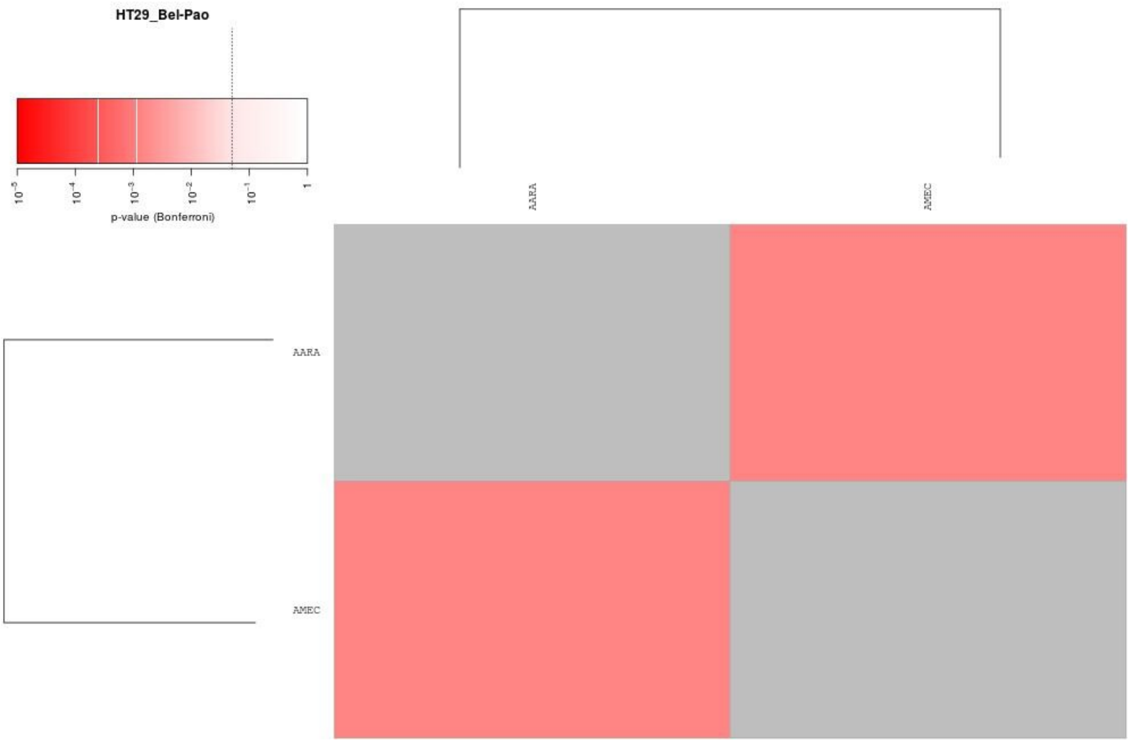
HT29_Bel-Pao
p-value (Bonferroni)
AARA
AMEC
AARA
AMEC

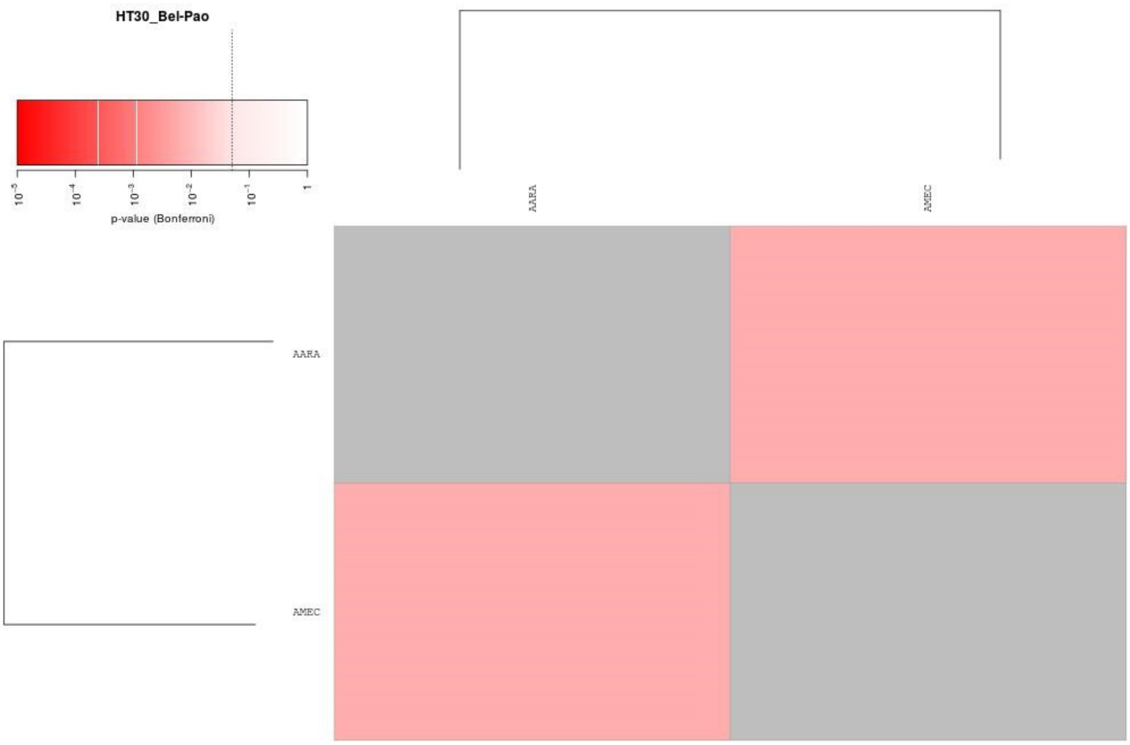
HT30_Bel-Pao
p-value (Bonferroni)
AARA
AMEC
AARA
AMEC

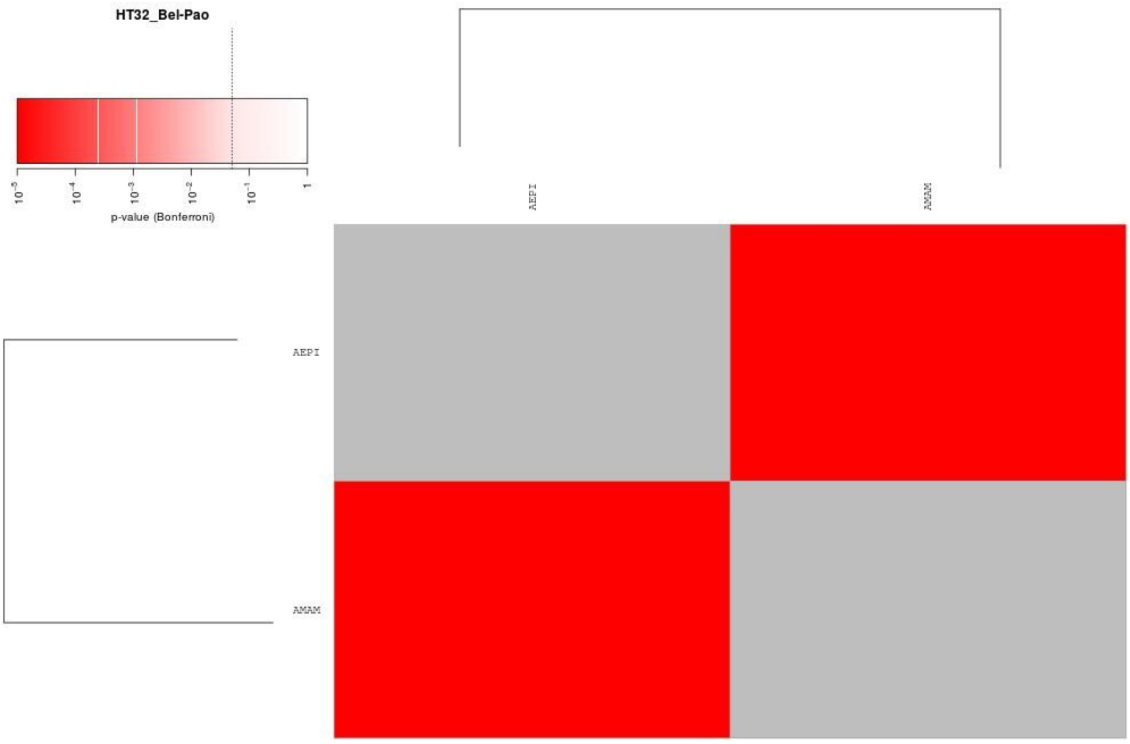
HT32_Bel-Pao
p-value (Bonferroni)
AEPI
AMAM
AEPI
AMAM

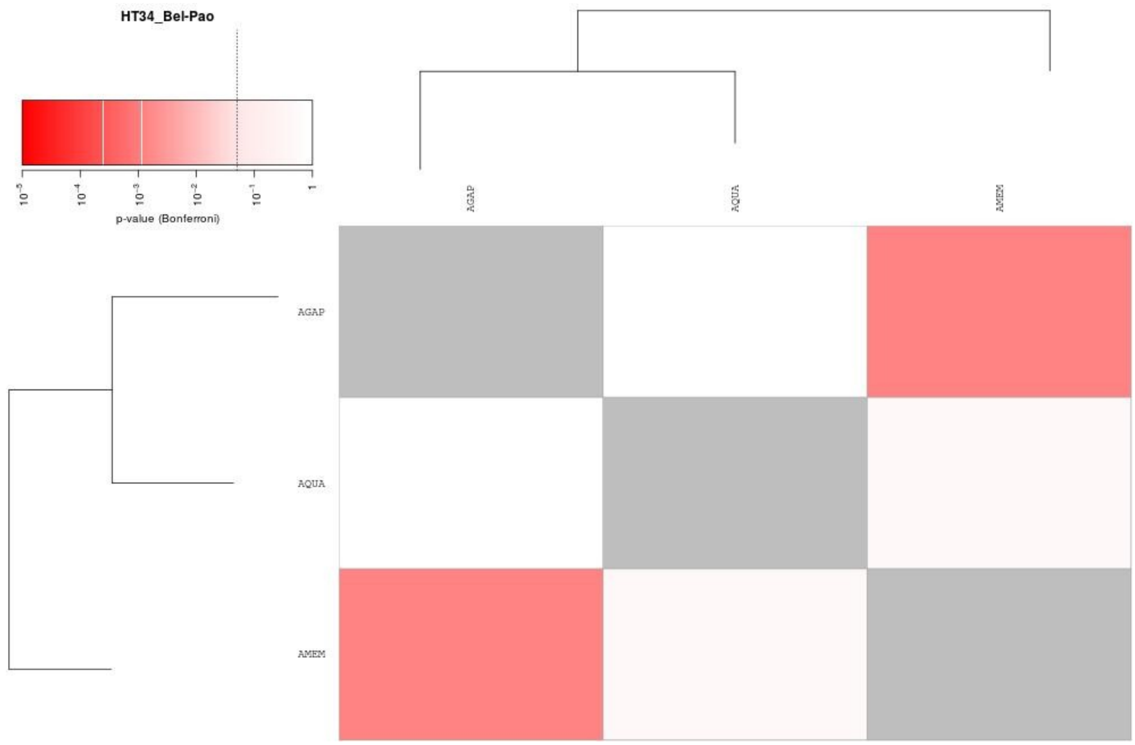
HT34_Bel-Pao
p-value (Bonferroni)
AGAP
AQUA
AMEM

## Copia

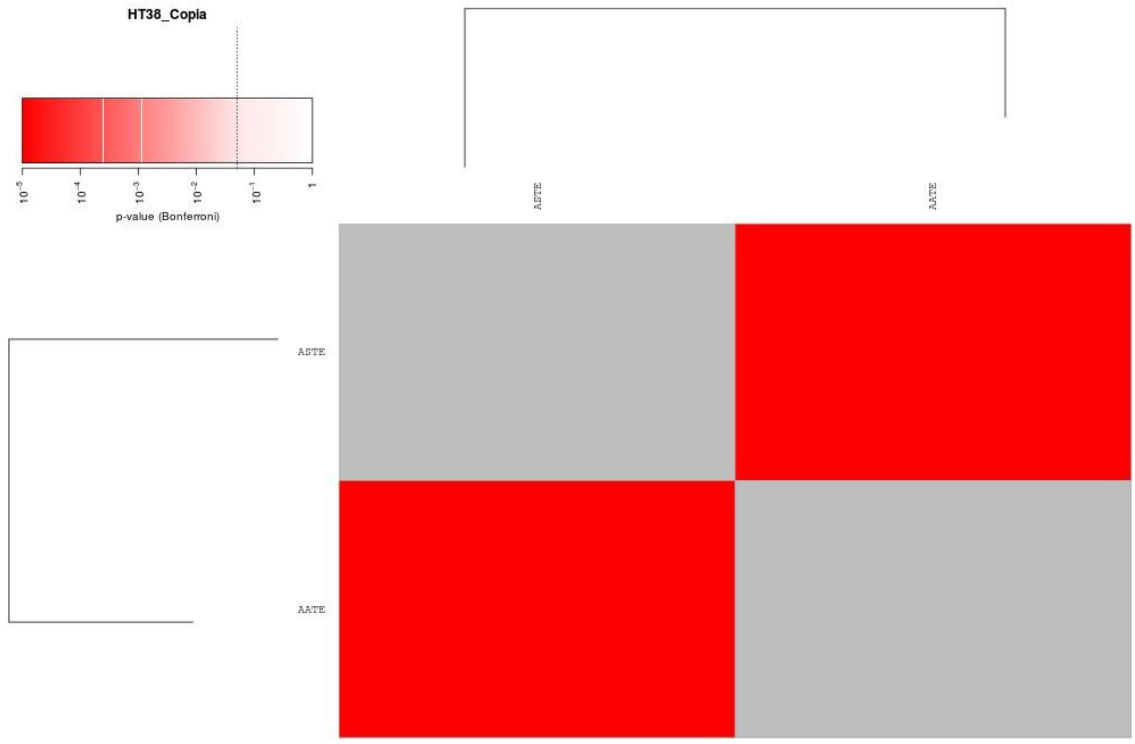
HT38_Copia
p-value (Bonferroni)
ASTE
AATE

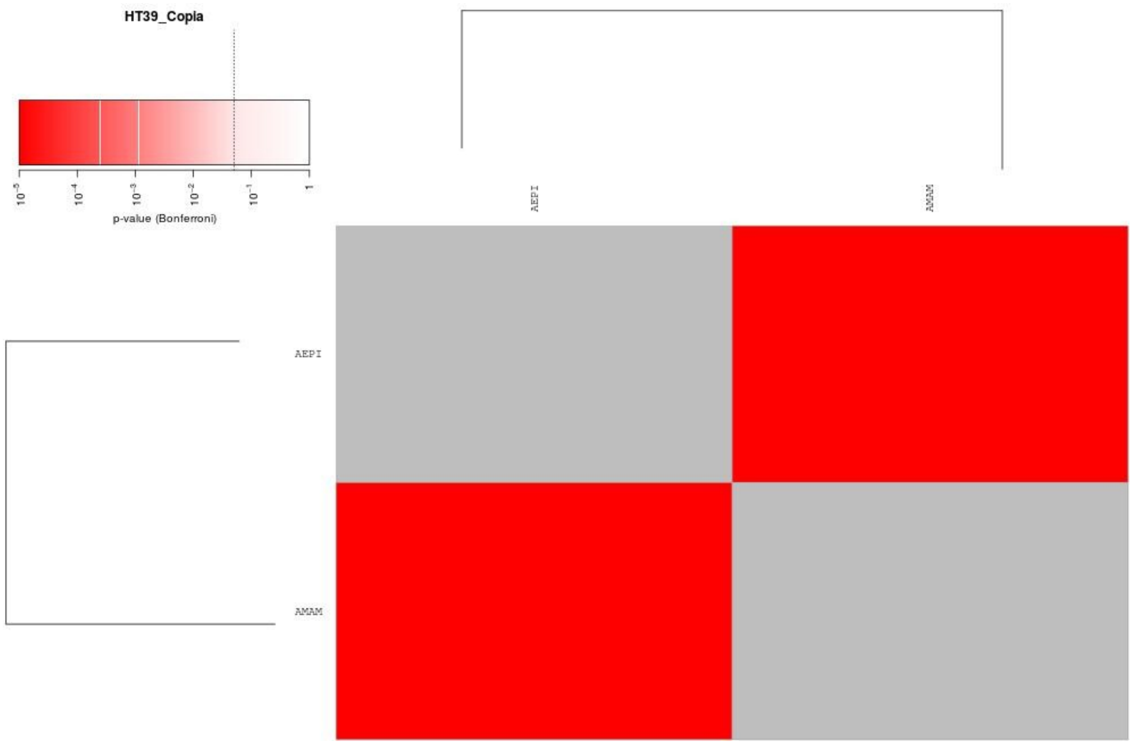
HT39_Copia
p-value (Bonferroni)
AEPI
AMAM
AEPI
AMAM

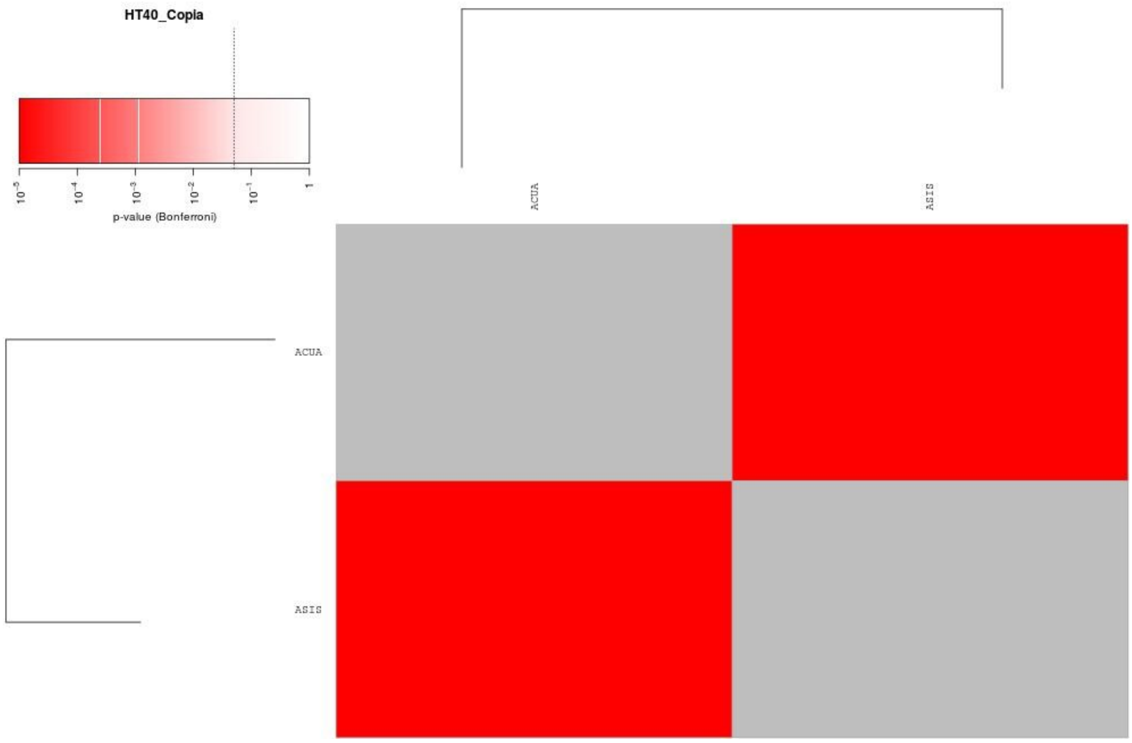
HT40_Copia
p-value (Bonferroni)
ACUA
ASIS
ACUA
ASIS

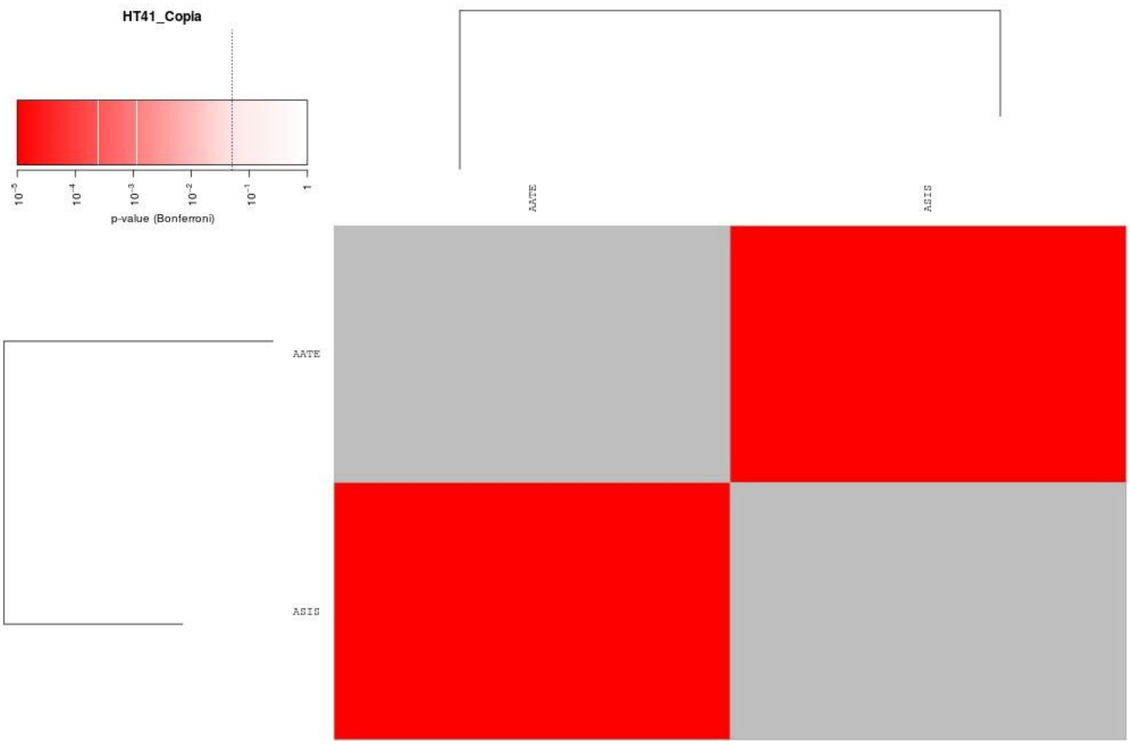
HT41_Copia
p-value (Bonferroni)
AATE
ASIS
AATE
ASIS

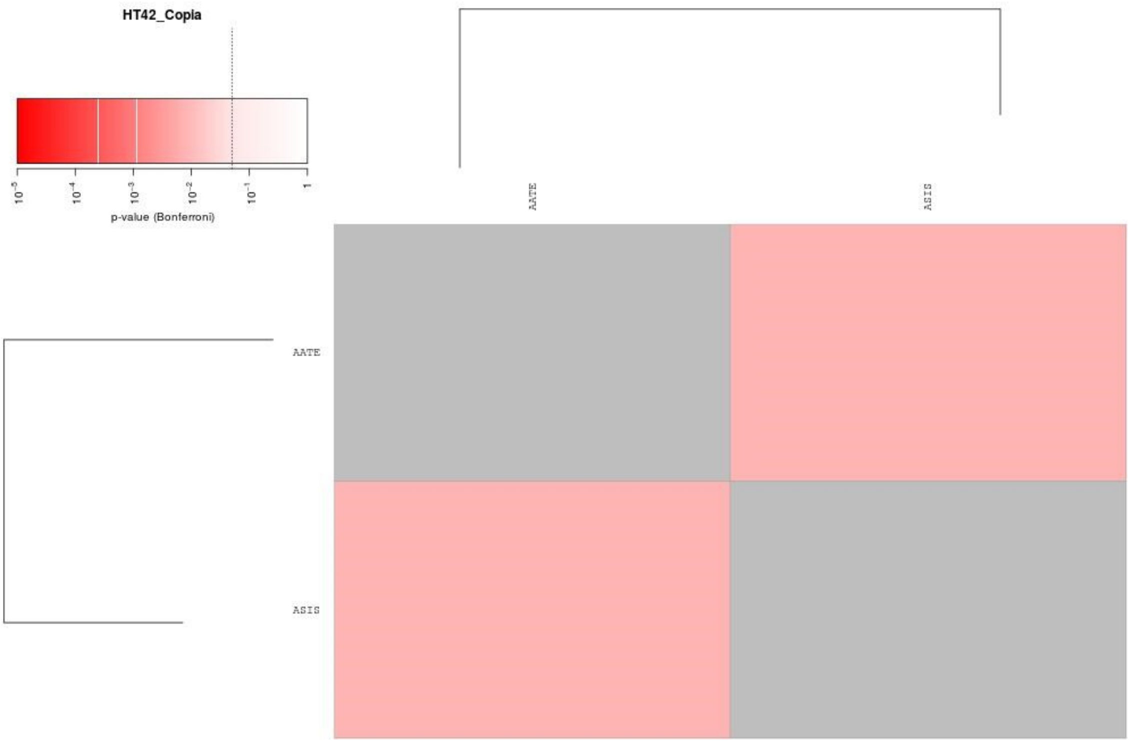
HT42_Copia
p-value (Bonferroni)
AATE
ASIS
AATE
ASIS

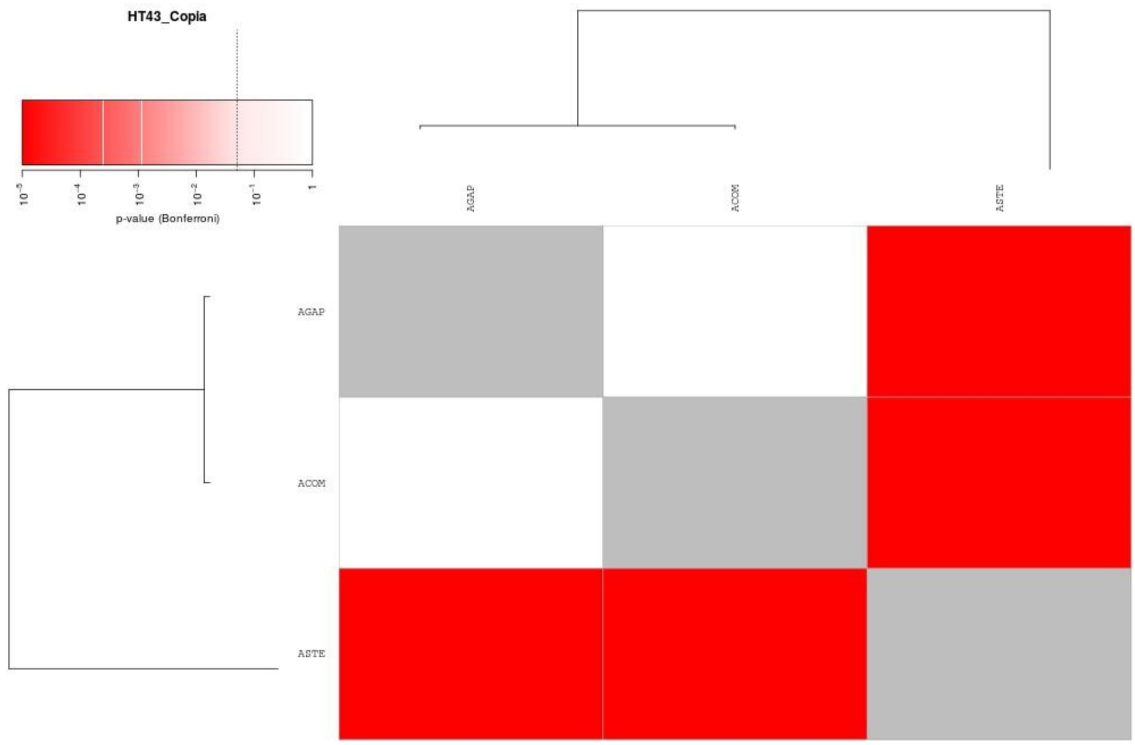
HT43_Copia
$10^{-5}$
$10^{-4}$
$10^{-3}$
$10^{-2}$
$10^{-1}$
1
p-value (Bonferroni)
AGAP
ACOM
ASTE
AGAP
ACOM
ASTE

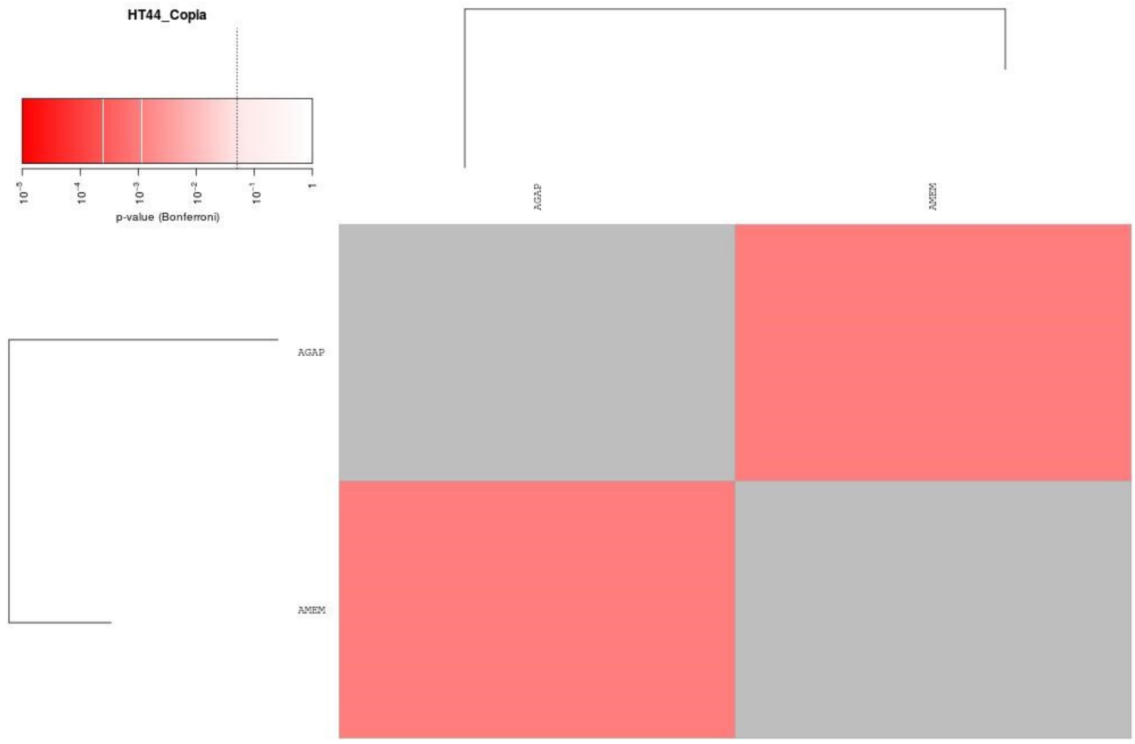
HT44_Copia
$10^{-5}$
$10^{-4}$
$10^{-3}$
$10^{-2}$
$10^{-1}$
1
p-value (Bonferroni)
AGAP
AMEM
AGAP
AMEM

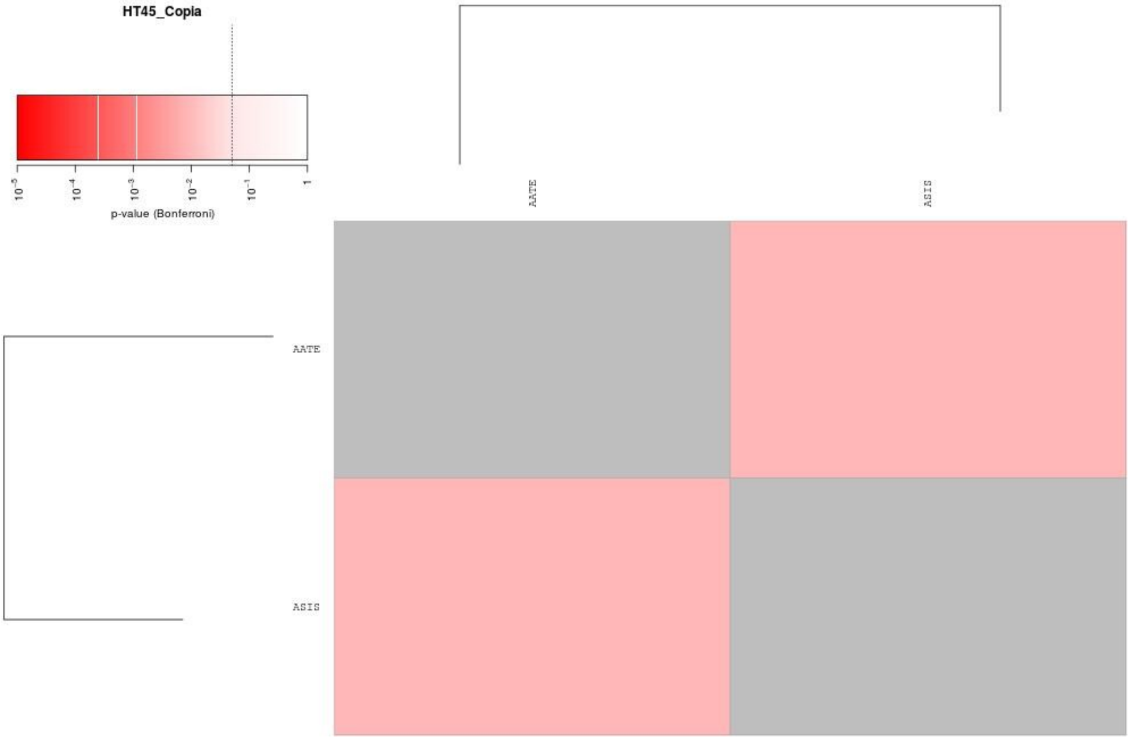
HT45_Copia
p-value (Bonferroni)
AATE
ASIS
AATE
ASIS

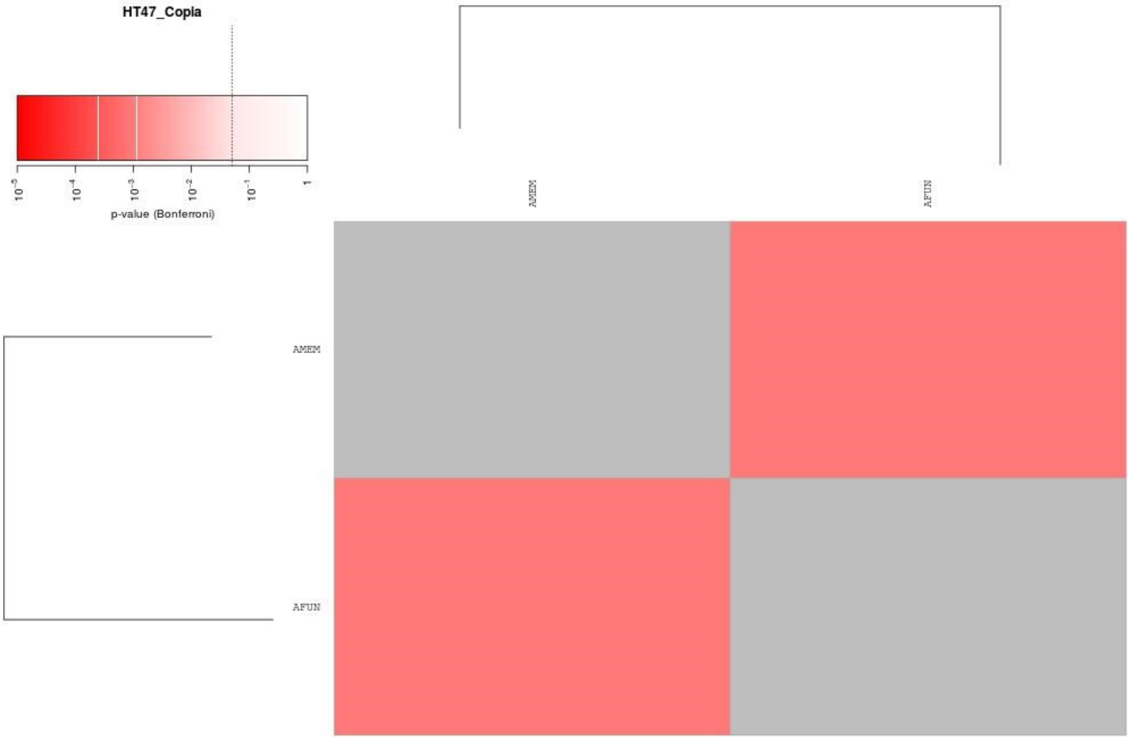
HT47_Copia
p-value (Bonferroni)
AMEM
AFUN
AMEM
AFUN

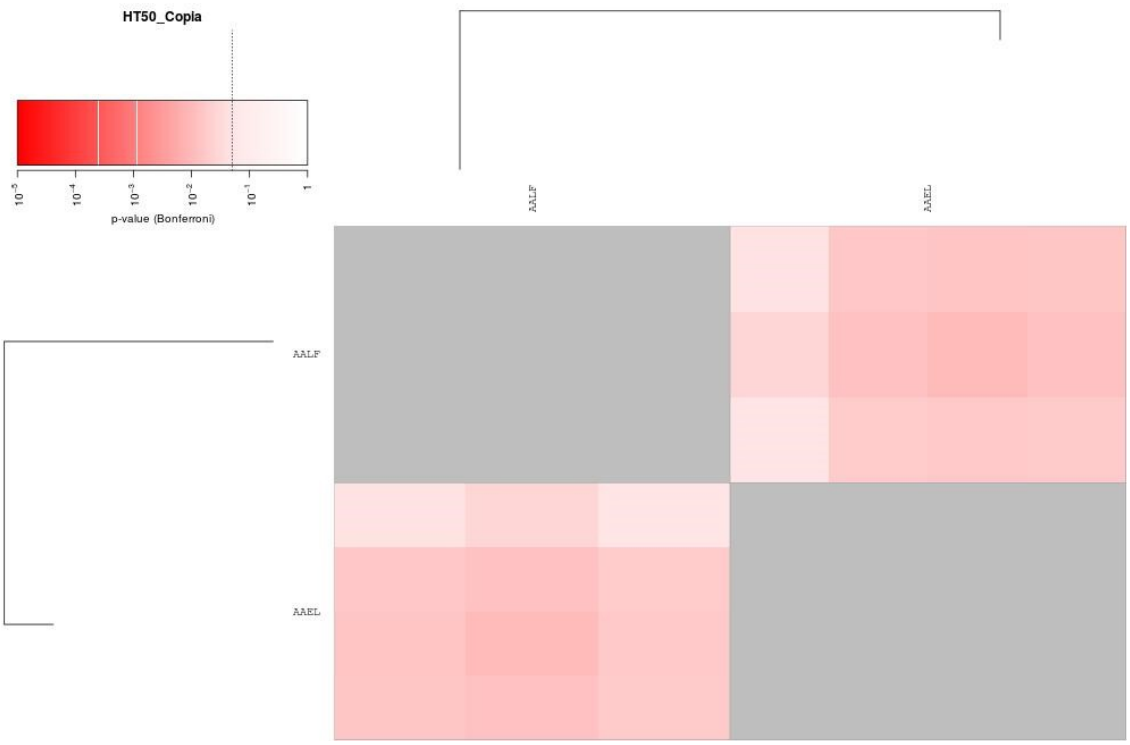
HT50_Copia
p-value (Bonferroni)
AALF
AAEL
AALF
AAEL

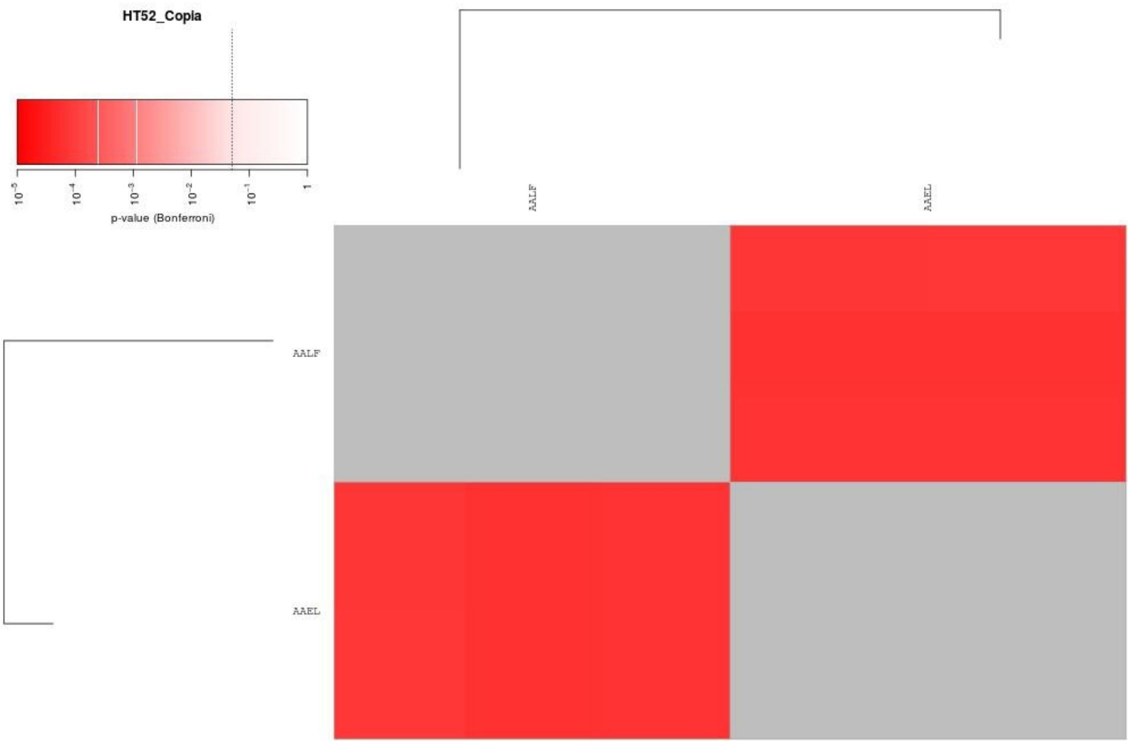
HT52_Copia
p-value (Bonferroni)
AALF
AAEL
AALF
AAEL

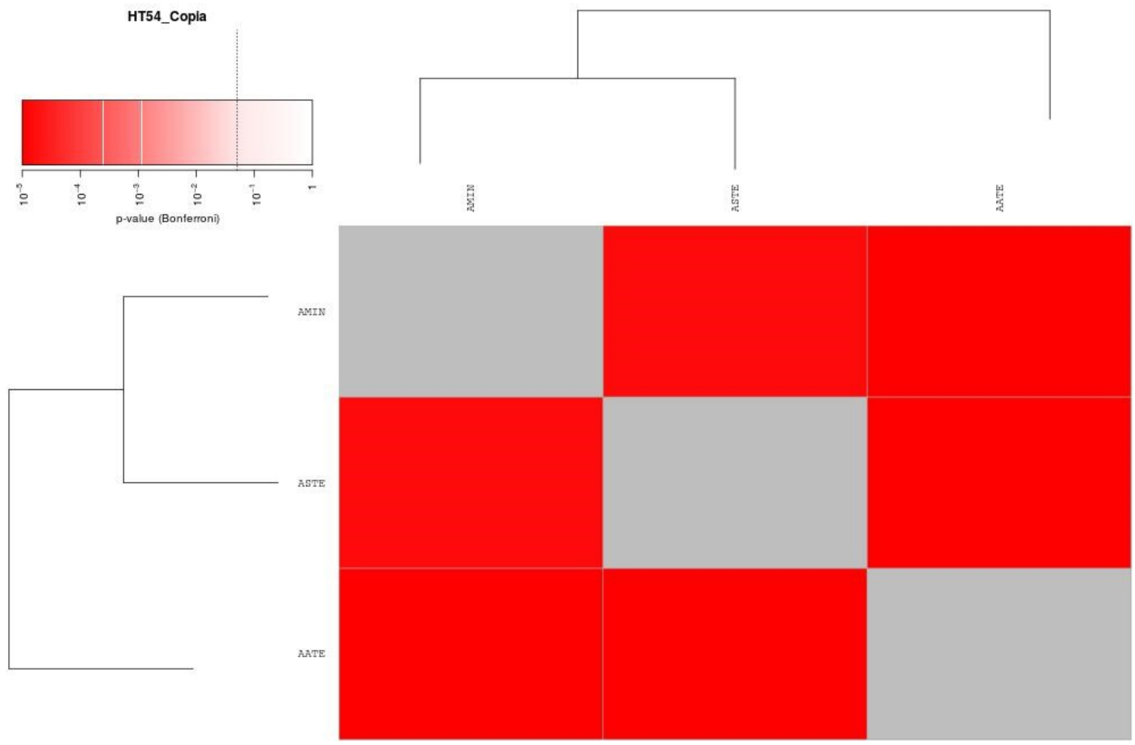
HT54_Copia
$10^{-5}$
$10^{-4}$
$10^{-3}$
$10^{-2}$
$10^{-1}$
1
p-value (Bonferroni)
AMIN
ASTE
AATE
AMIN
ASTE
AATE

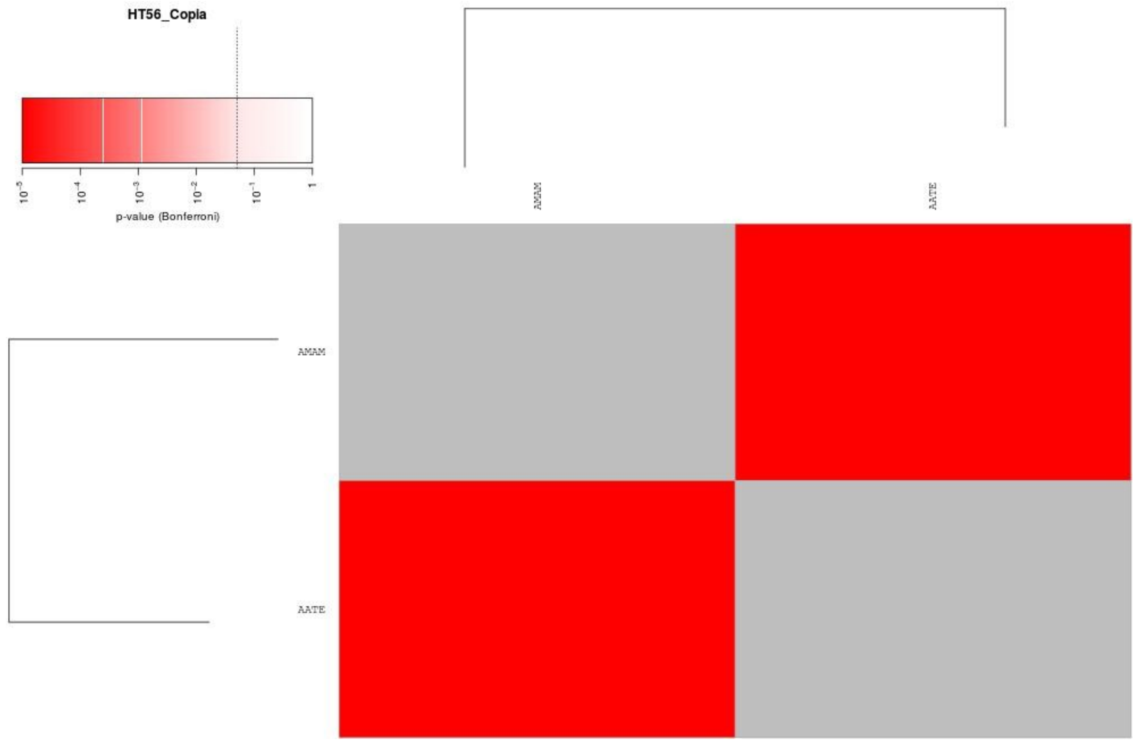
HT56_Copia
$10^{-5}$
$10^{-4}$
$10^{-3}$
$10^{-2}$
$10^{-1}$
1
p-value (Bonferroni)
AMAM
AATE
AMAM
AATE

## Gypsy

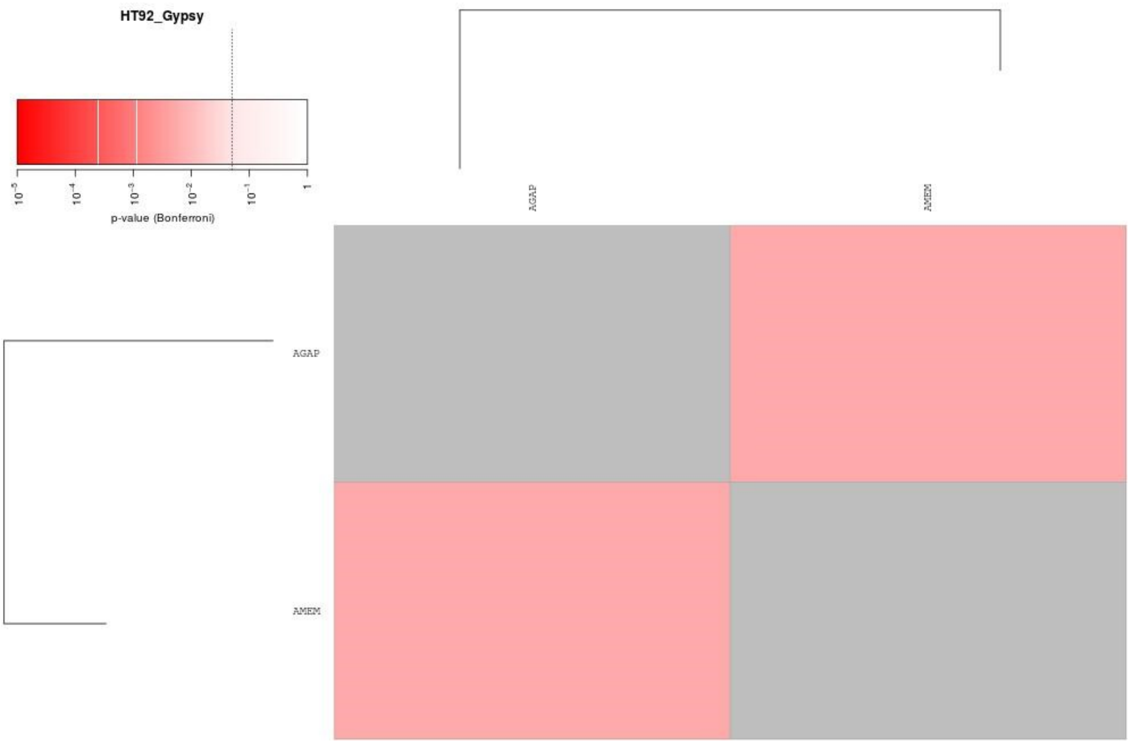
HT92_Gypsy
$10^{-5}$
$10^{-4}$
$10^{-3}$
$10^{-2}$
$10^{-1}$
1
p-value (Bonferroni)
AGAP
AMEM
AGAP
AMEM

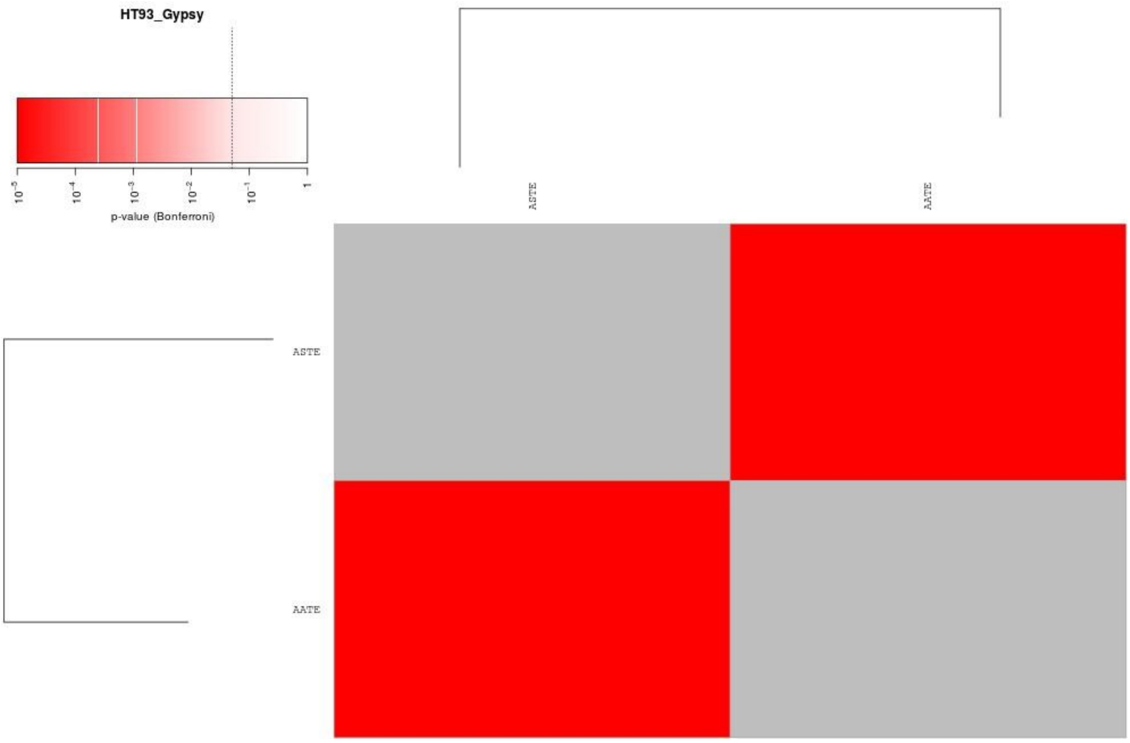
HT93_Gypsy
$10^{-5}$
$10^{-4}$
$10^{-3}$
$10^{-2}$
$10^{-1}$
1
p-value (Bonferroni)
ASTE
AATE
ASTE
AATE

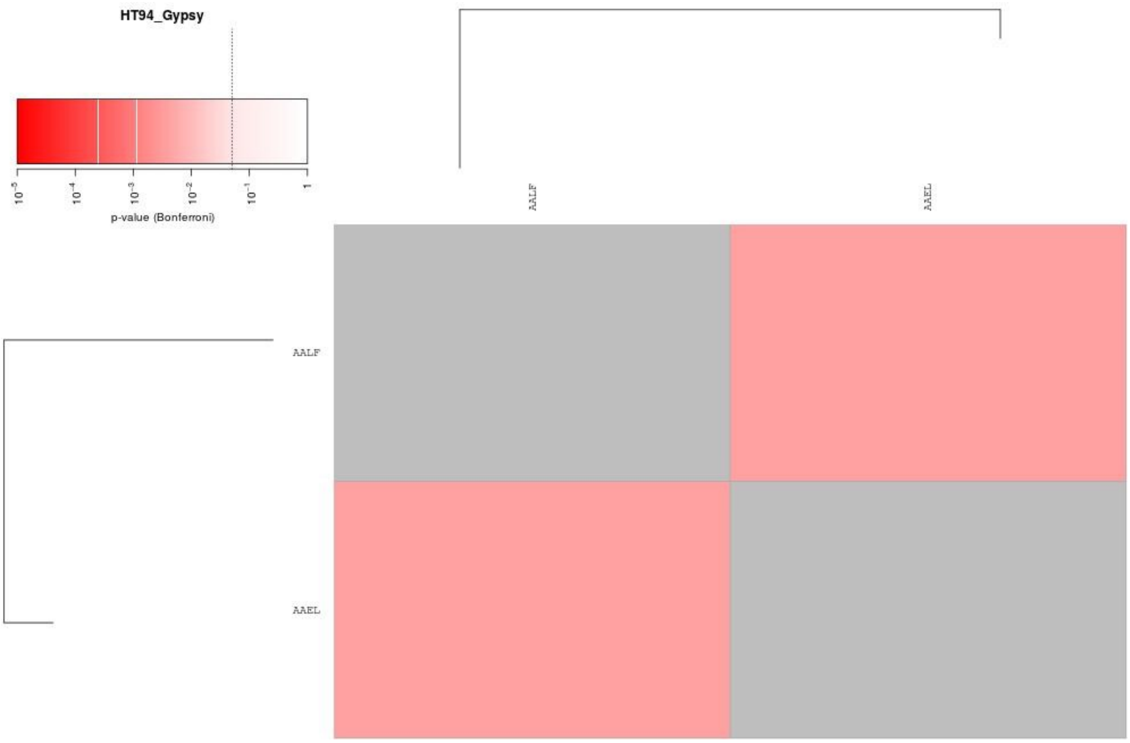
HT94_Gypsy
p-value (Bonferroni)
AALF
AAEL
AALF
AAEL

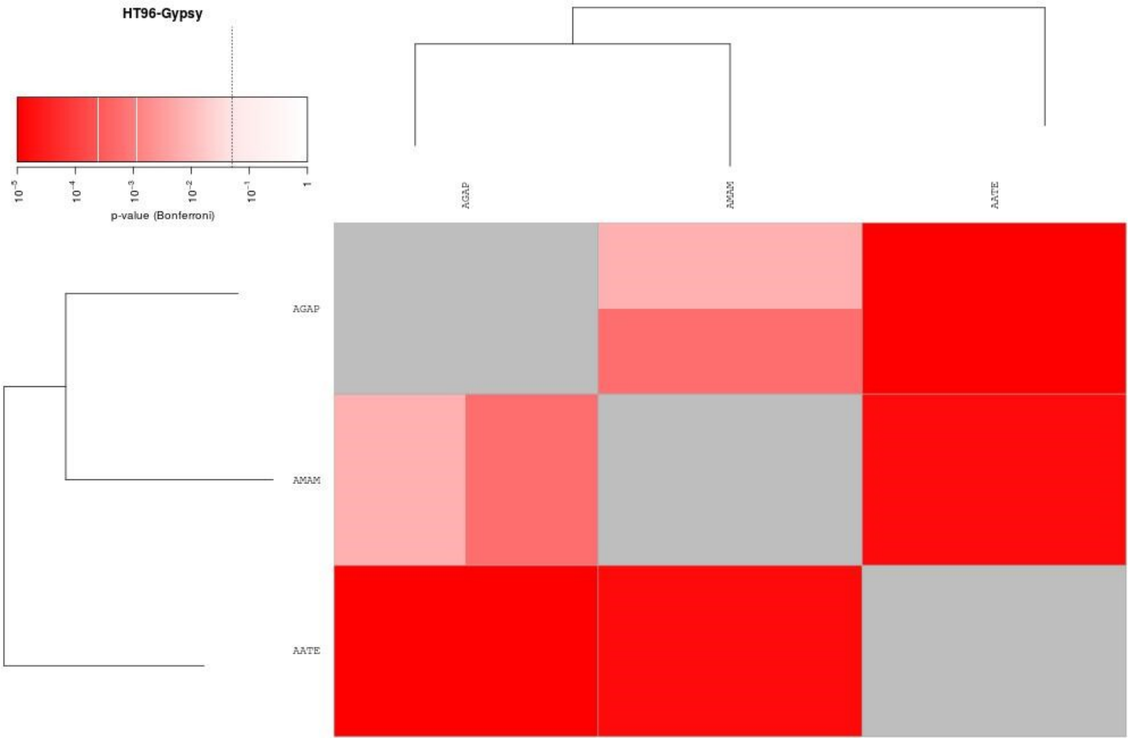
HT96-Gypsy
p-value (Bonferroni)
AGAP
AMAM
AATE
AGAP
AMAM
AATE

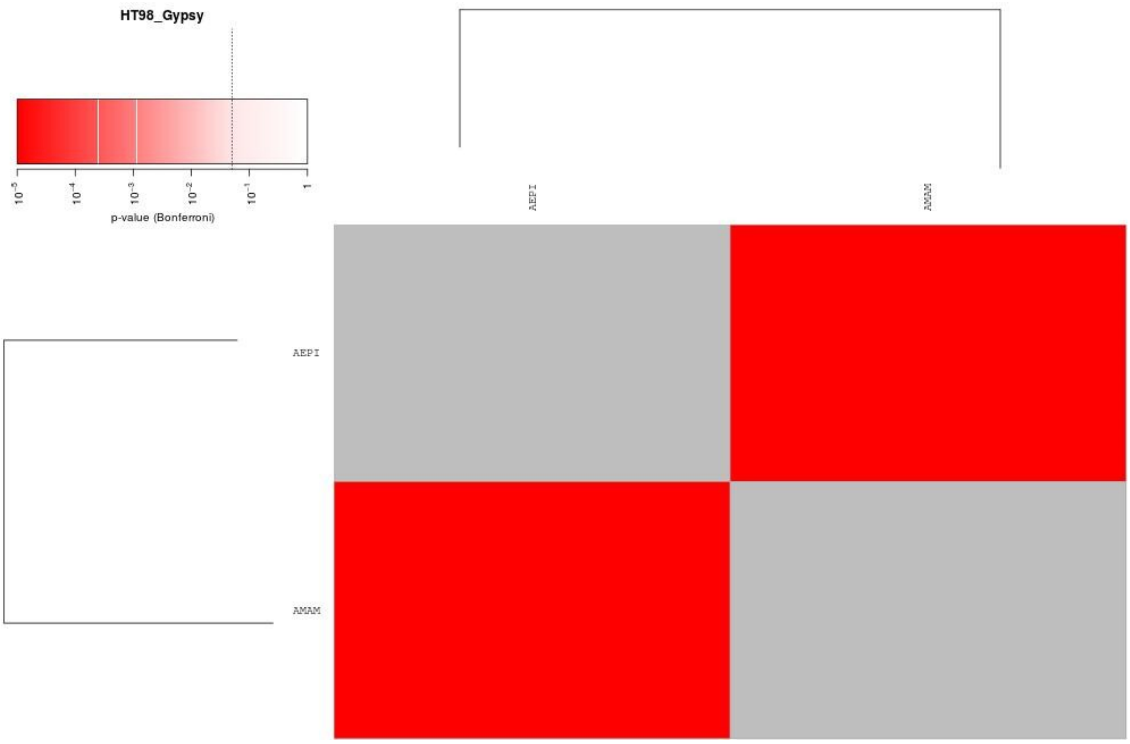
HT98_Gypsy
p-value (Bonferroni)
AEPI
AMAM
AEPI
AMAM

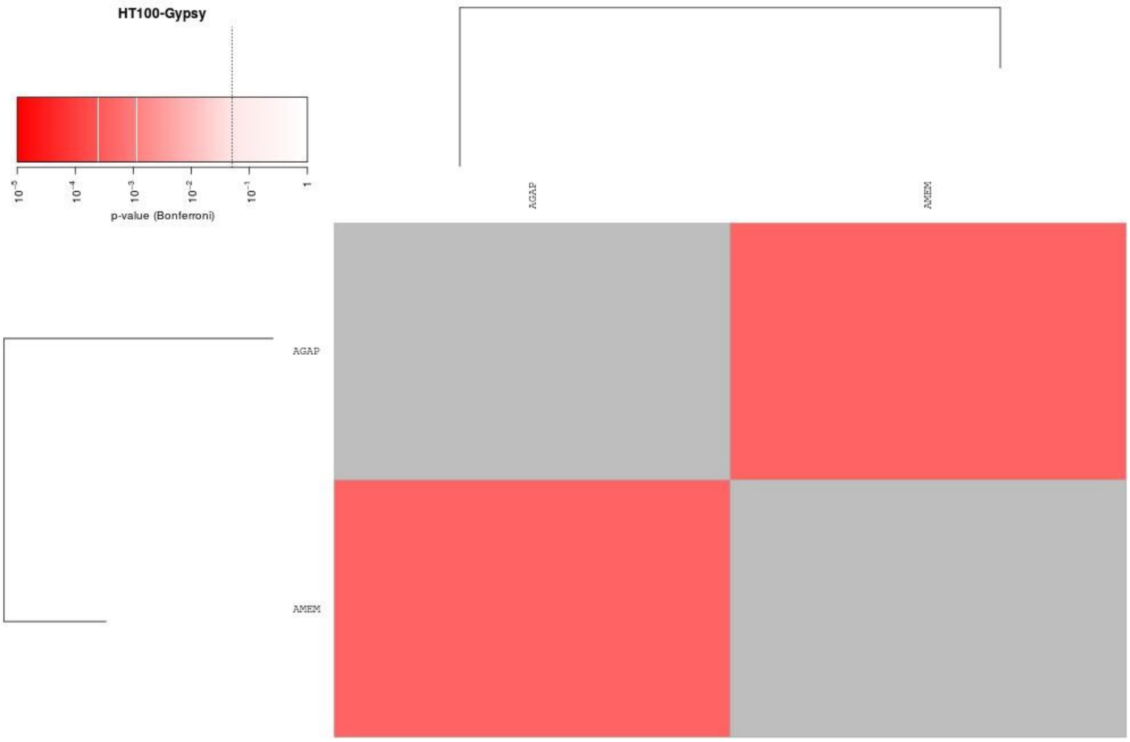
HT100-Gypsy
p-value (Bonferroni)
AGAP
AMEM
AGAP
AMEM

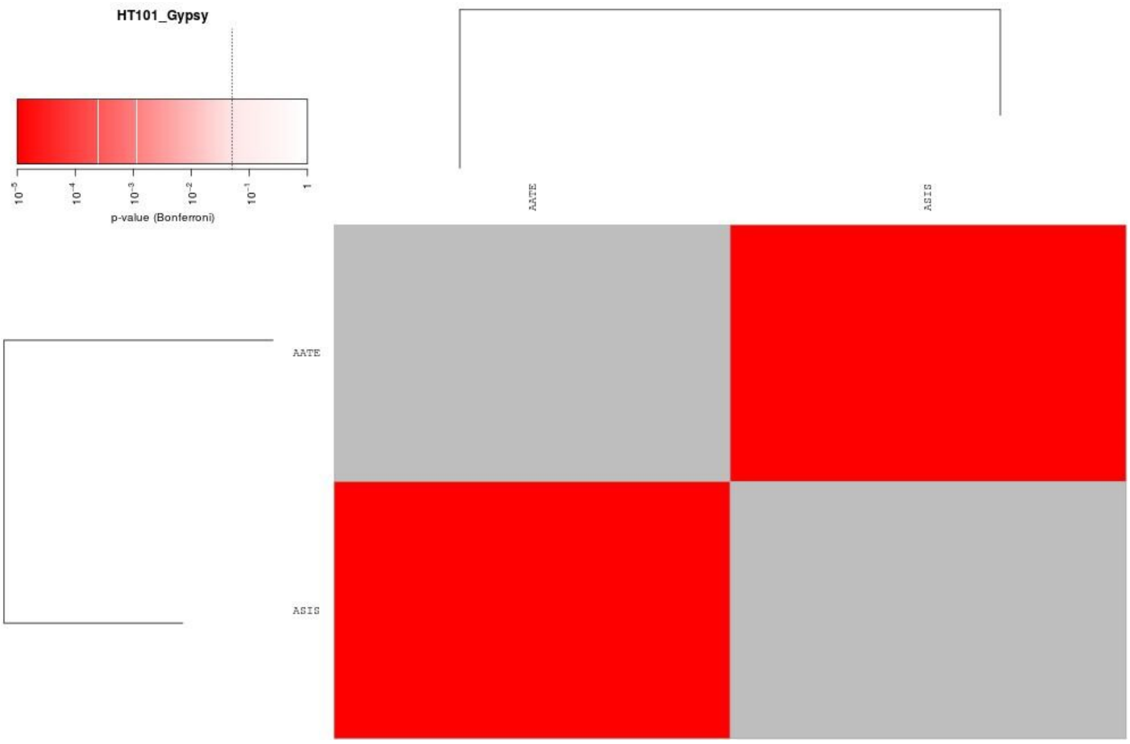
HT101_Gypsy
p-value (Bonferroni)
AATE
ASIS
AATE
ASIS

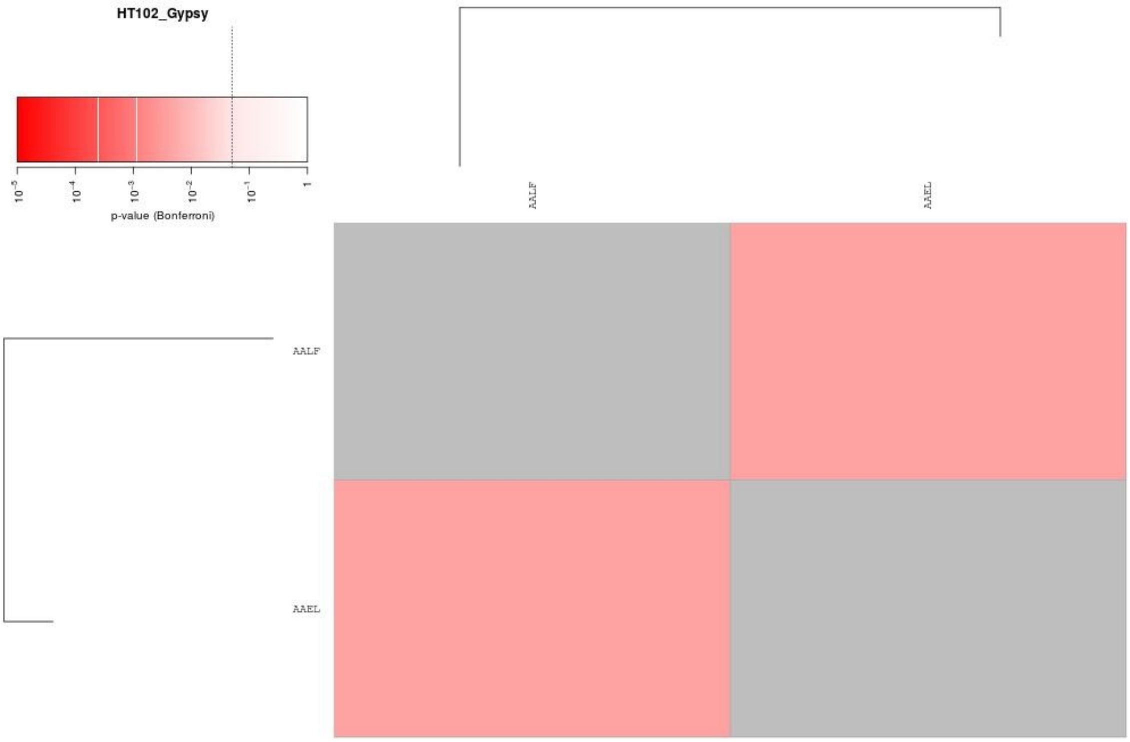
HT102_Gypsy
p-value (Bonferroni)
AALF
AAEL
AALF
AAEL

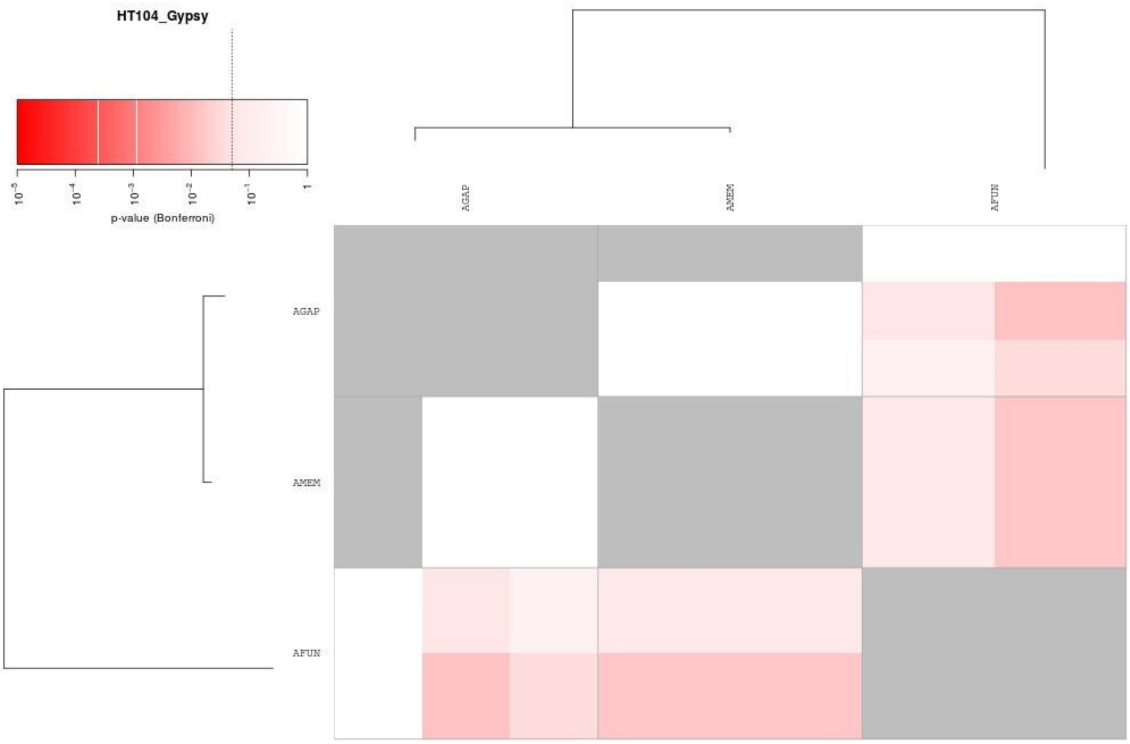
HT104_Gypsy
$10^{-5}$
$10^{-4}$
$10^{-3}$
$10^{-2}$
$10^{-1}$
1
p-value (Bonferroni)
AGAP
AMEM
AFUN
AGAP
AMEM
AFUN

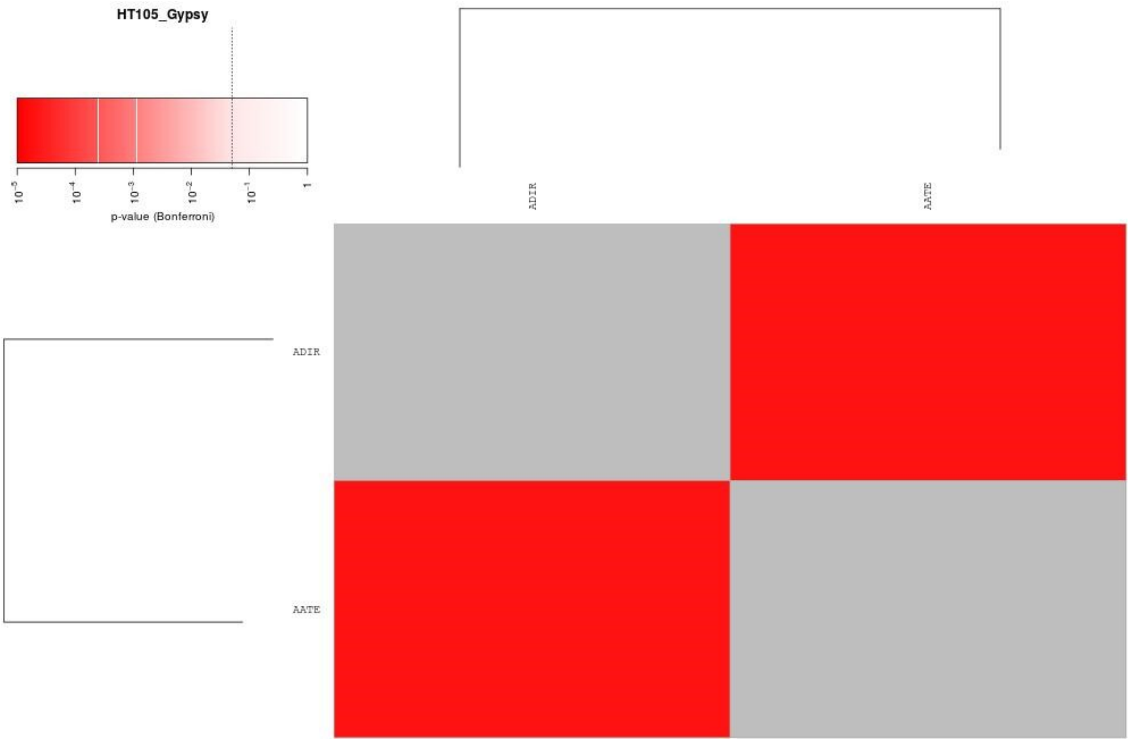
HT105_Gypsy
$10^{-5}$
$10^{-4}$
$10^{-3}$
$10^{-2}$
$10^{-1}$
1
p-value (Bonferroni)
ADIR
AATE
ADIR
AATE

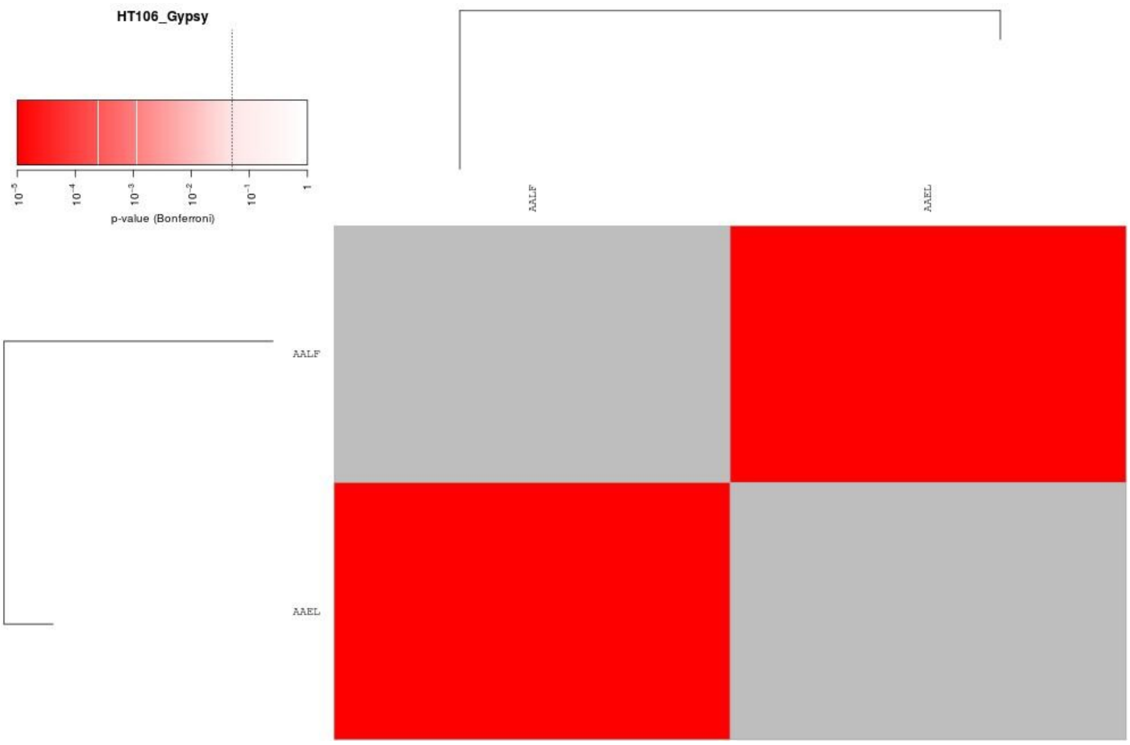
HT106_Gypsy
p-value (Bonferroni)
AALF
AAEL
AALF
AAEL

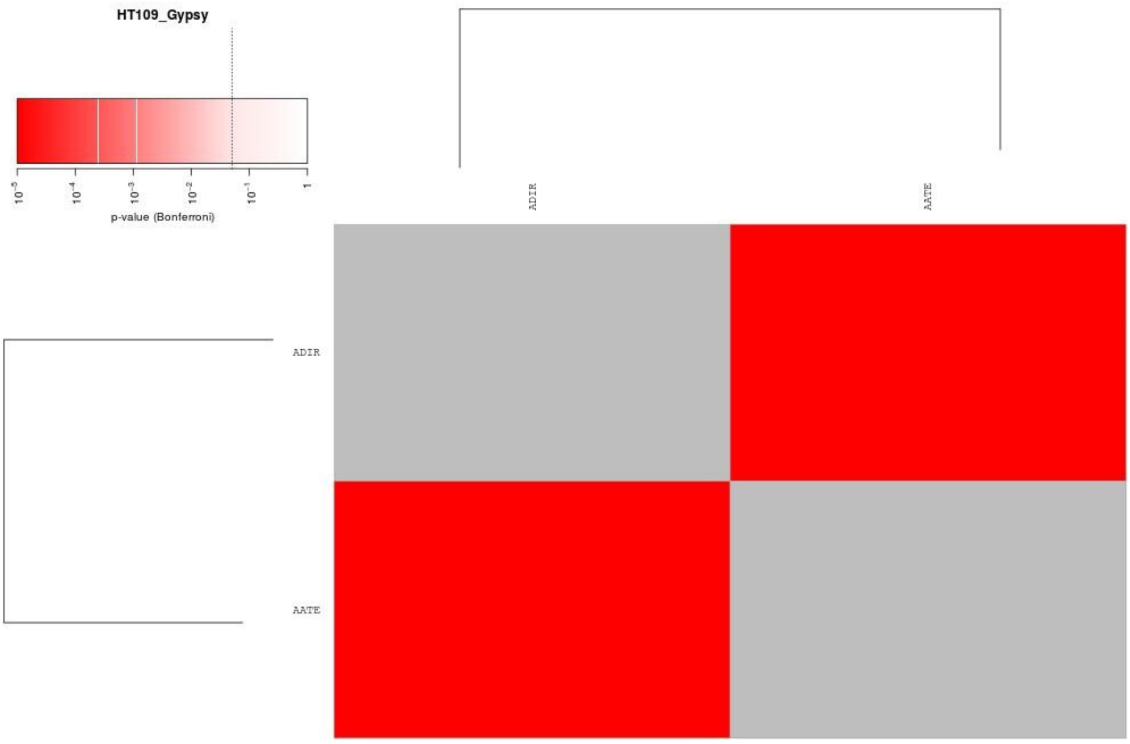
HT109_Gypsy
p-value (Bonferroni)
ADIR
AATE
ADIR
AATE

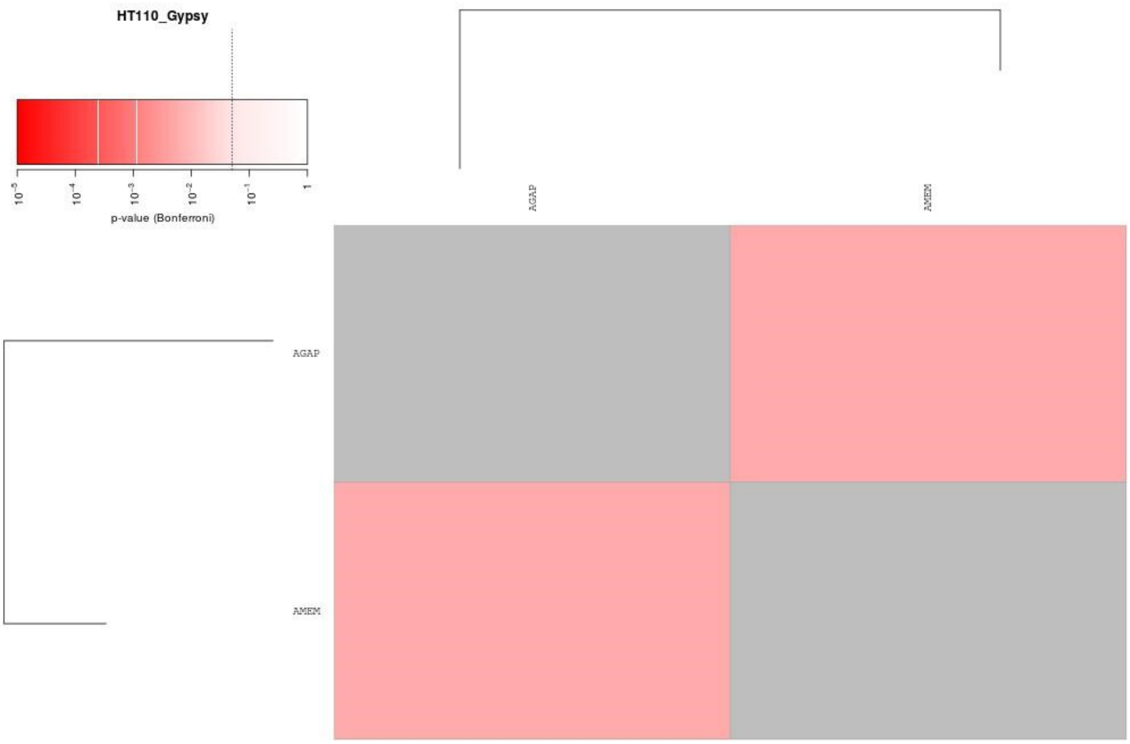
HT110_Gypsy
p-value (Bonferroni)
AGAP
AMEM
AGAP
AMEM

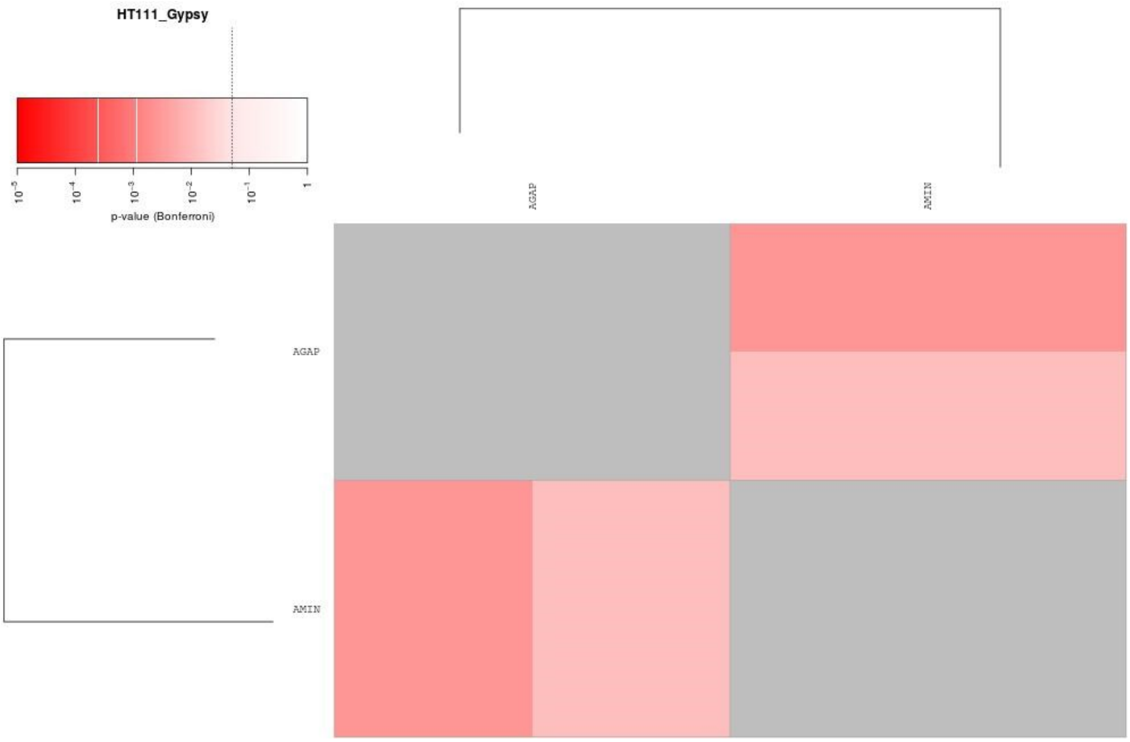
HT111_Gypsy
p-value (Bonferroni)
AGAP
AMIN
AGAP
AMIN

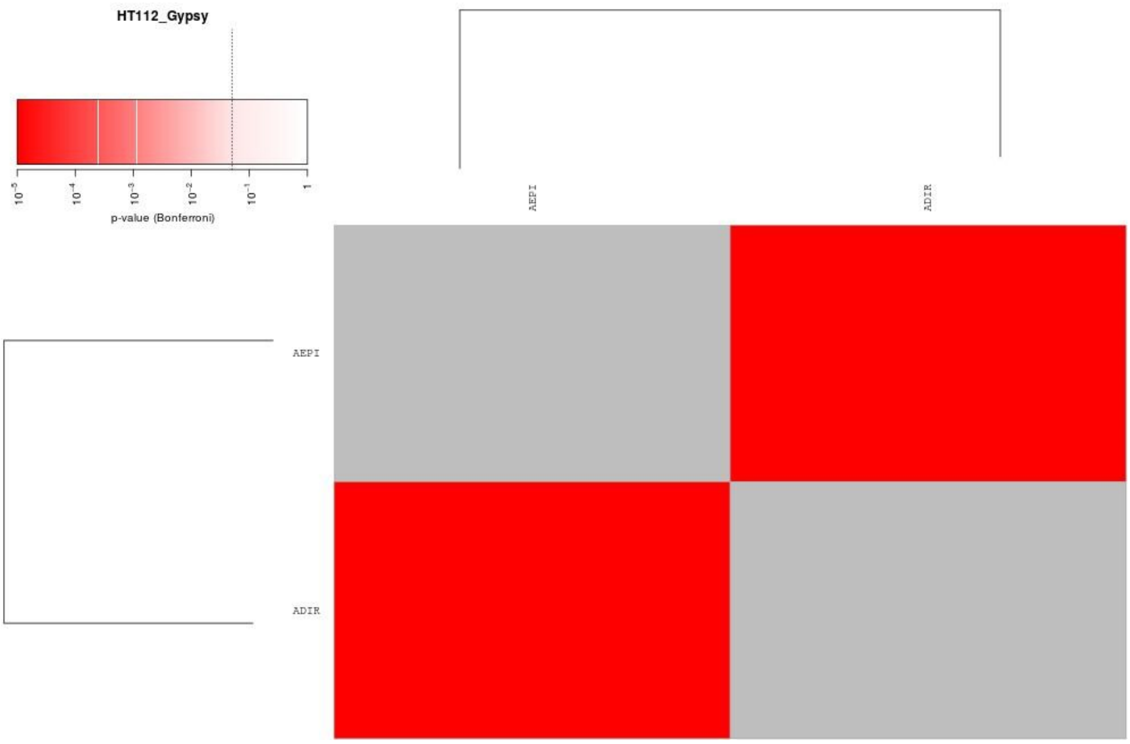
HT112_Gypsy
p-value (Bonferroni)
AEPI
ADIR
AEPI
ADIR

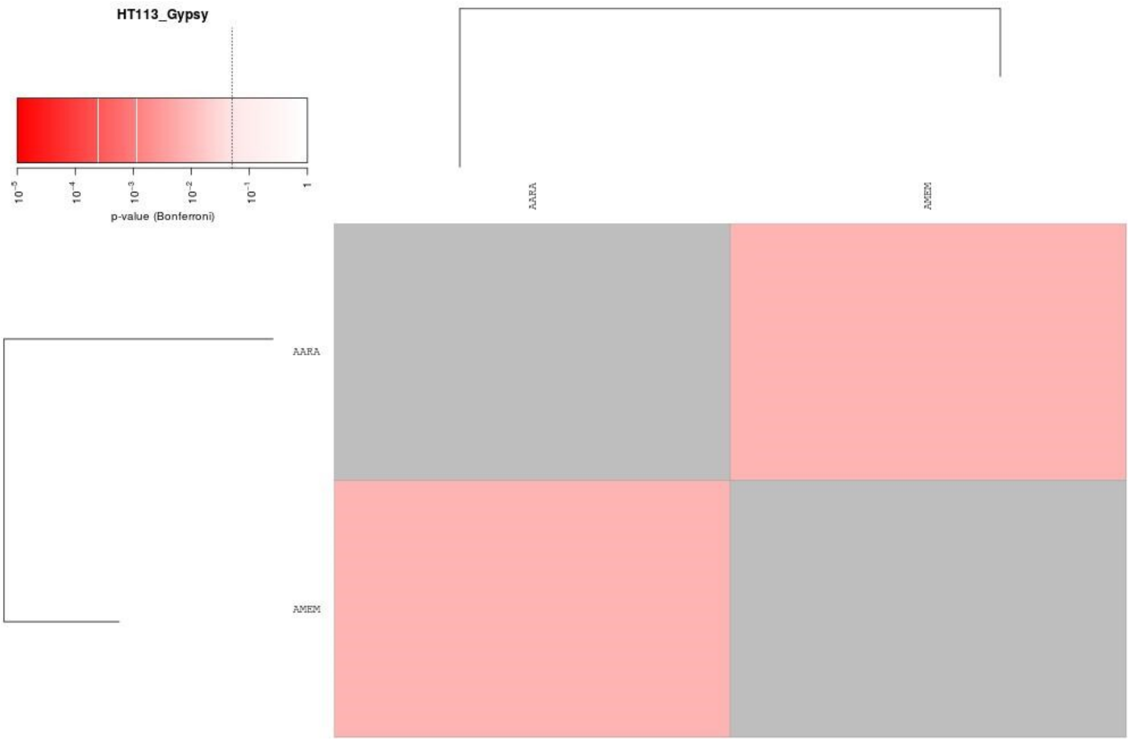
HT113_Gypsy
p-value (Bonferroni)
AARA
AMEM
AARA
AMEM

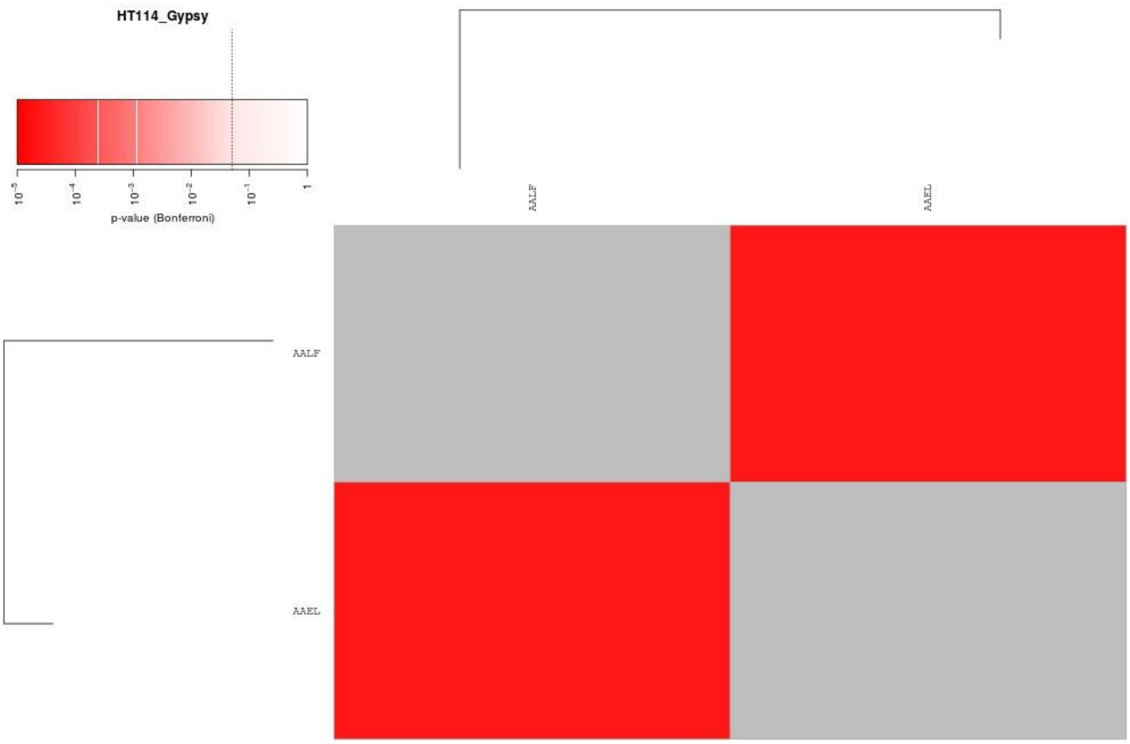
HT114_Gypsy
p-value (Bonferroni)
AALF
AAEL
AALF
AAEL

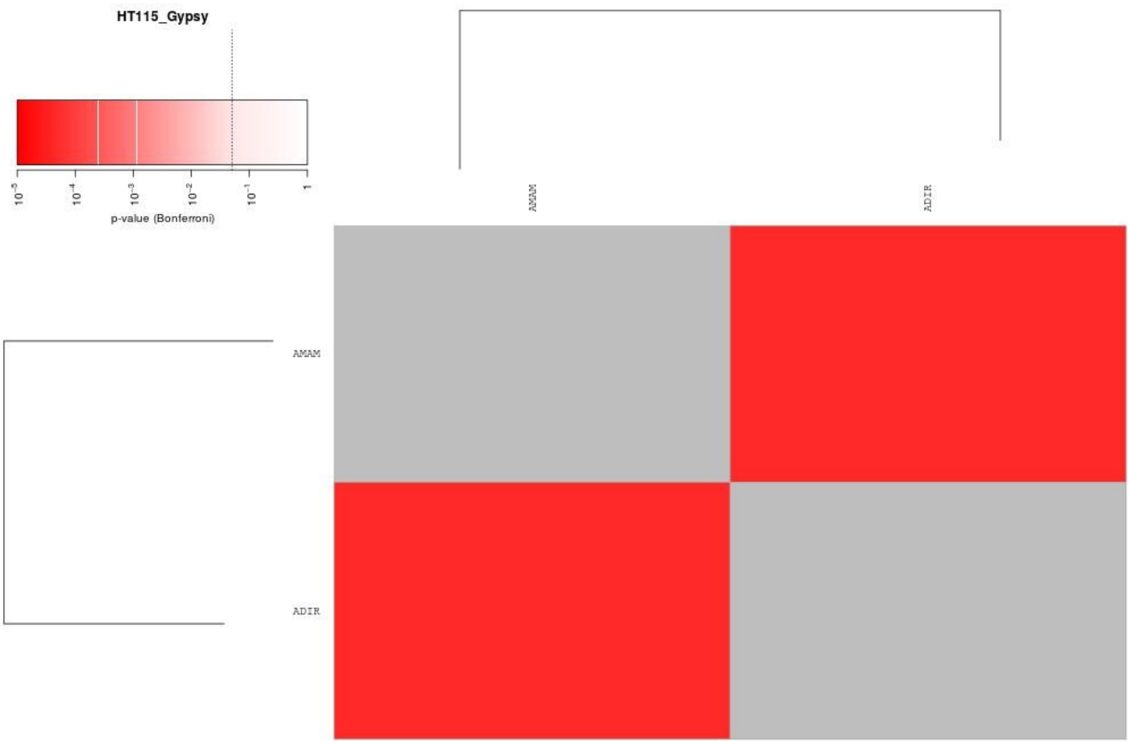
HT115_Gypsy
p-value (Bonferroni)
AMAM
ADIR
AMAM
ADIR

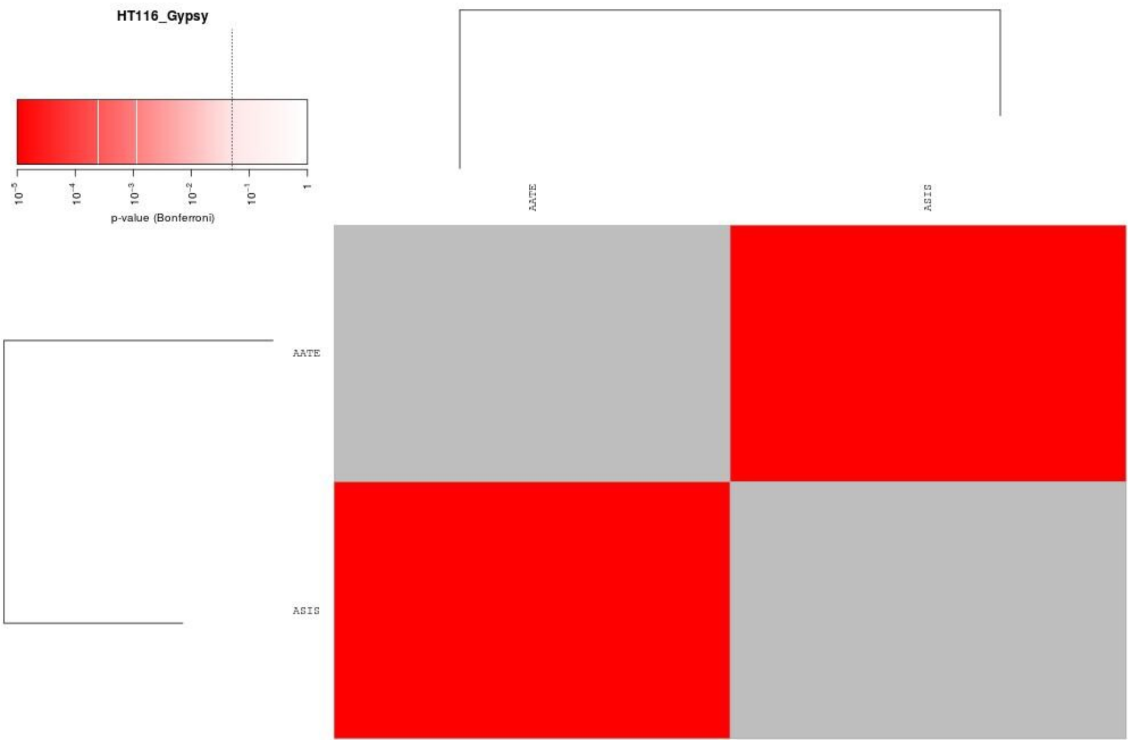
HT116_Gypsy
p-value (Bonferroni)
AATE
ASIS
AATE
ASIS

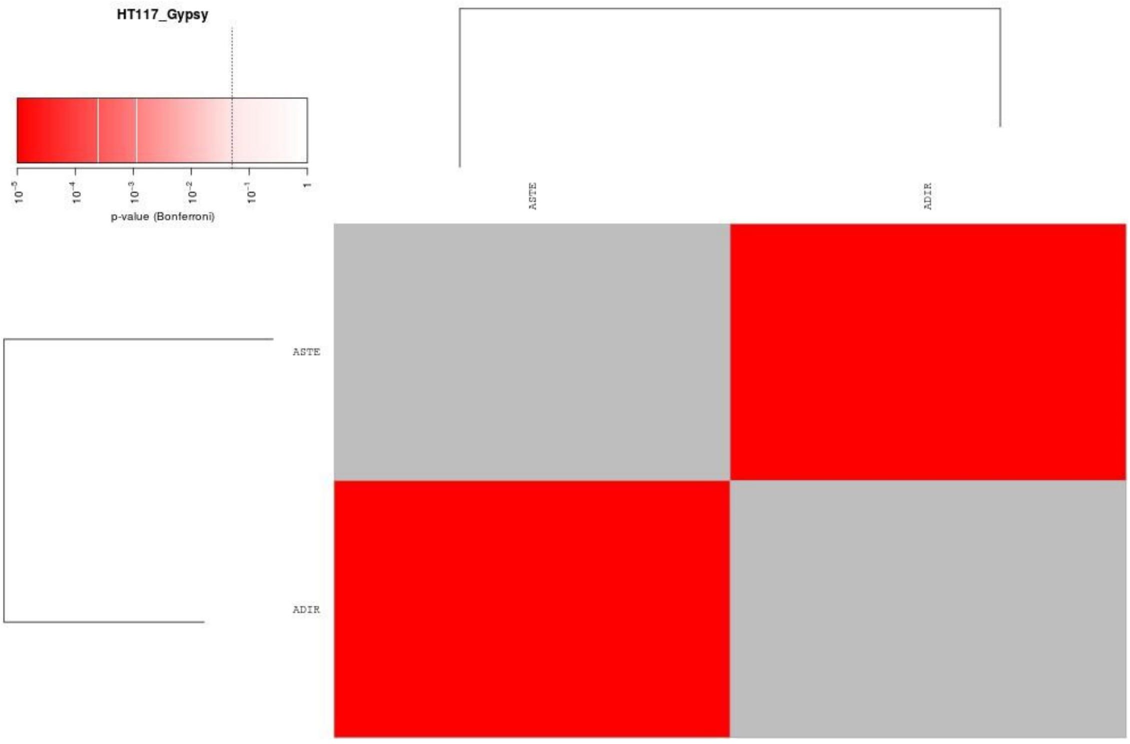
HT117_Gypsy
p-value (Bonferroni)
ASTE
ADIR
ASTE
ADIR

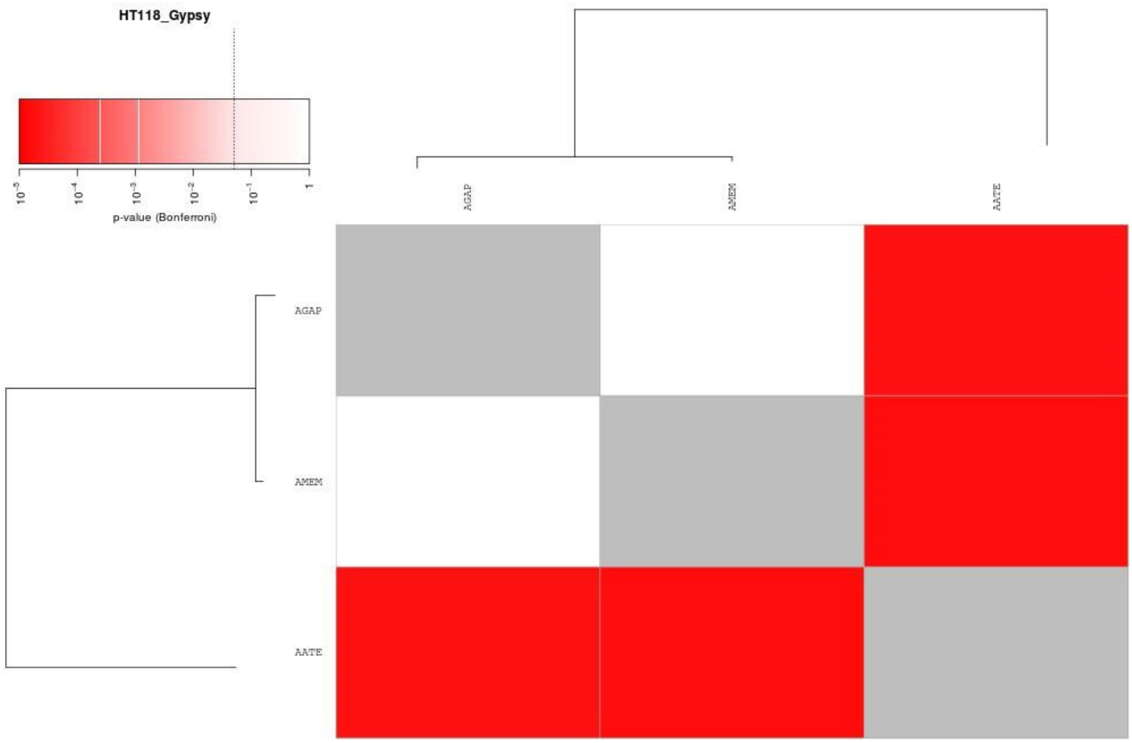
HT118_Gypsy
p-value (Bonferroni)
AGAP
AMEM
AATE

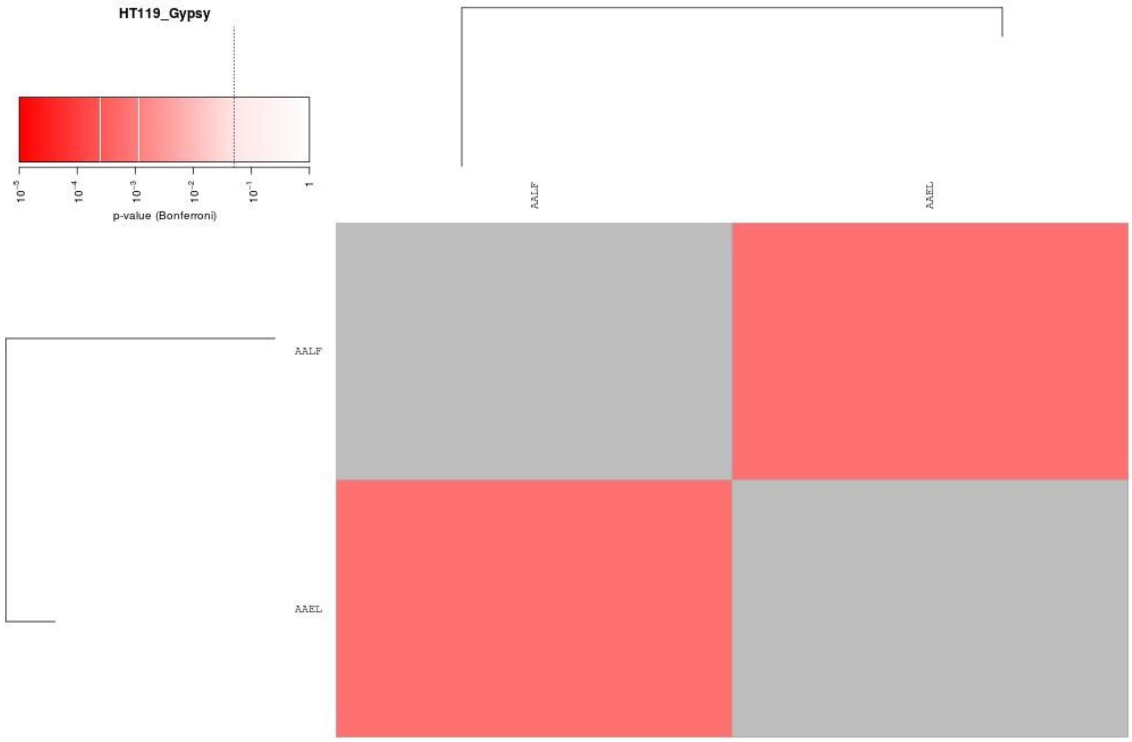
HT119_Gypsy
p-value (Bonferroni)
AALF
AAEL

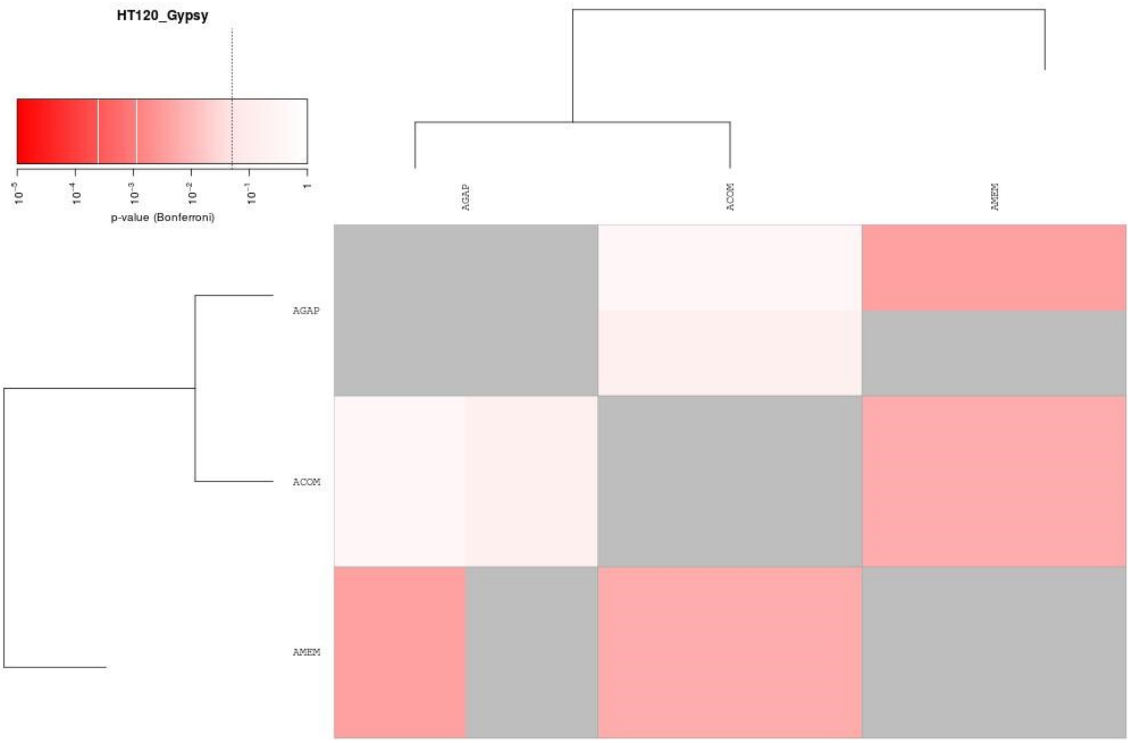
HT120_Gypsy
p-value (Bonferroni)
AGAP
ACOM
AMEM

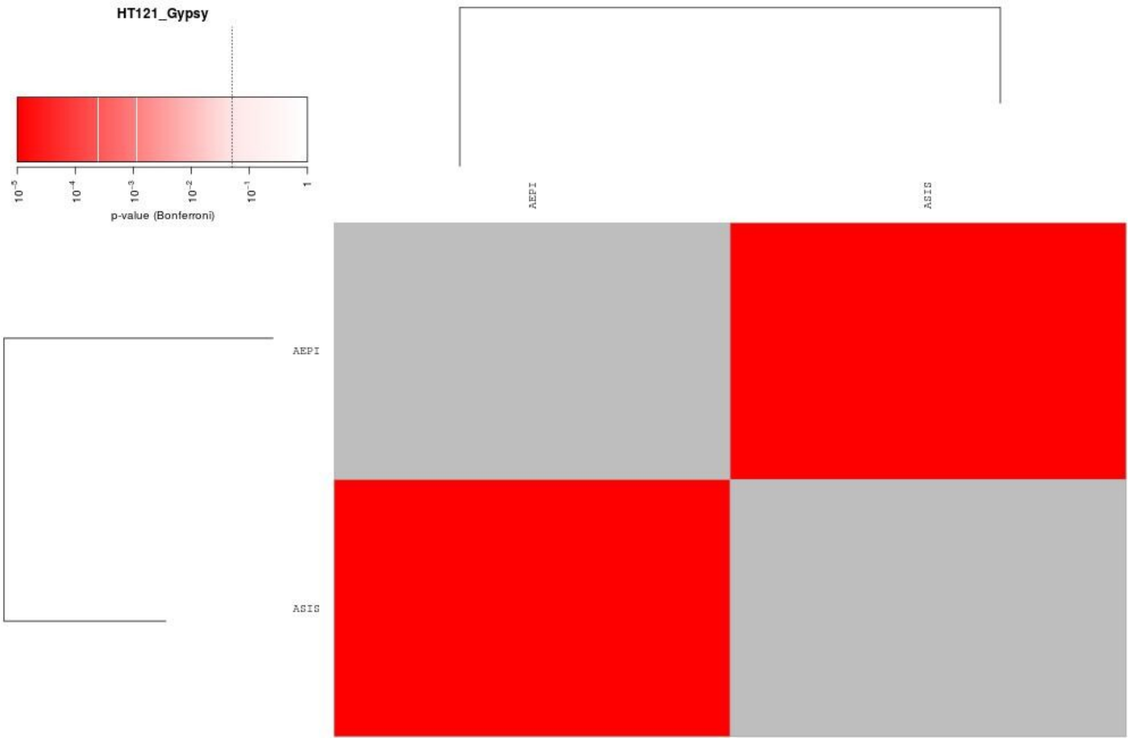
HT121_Gypsy
p-value (Bonferroni)
AEPI
ASIS

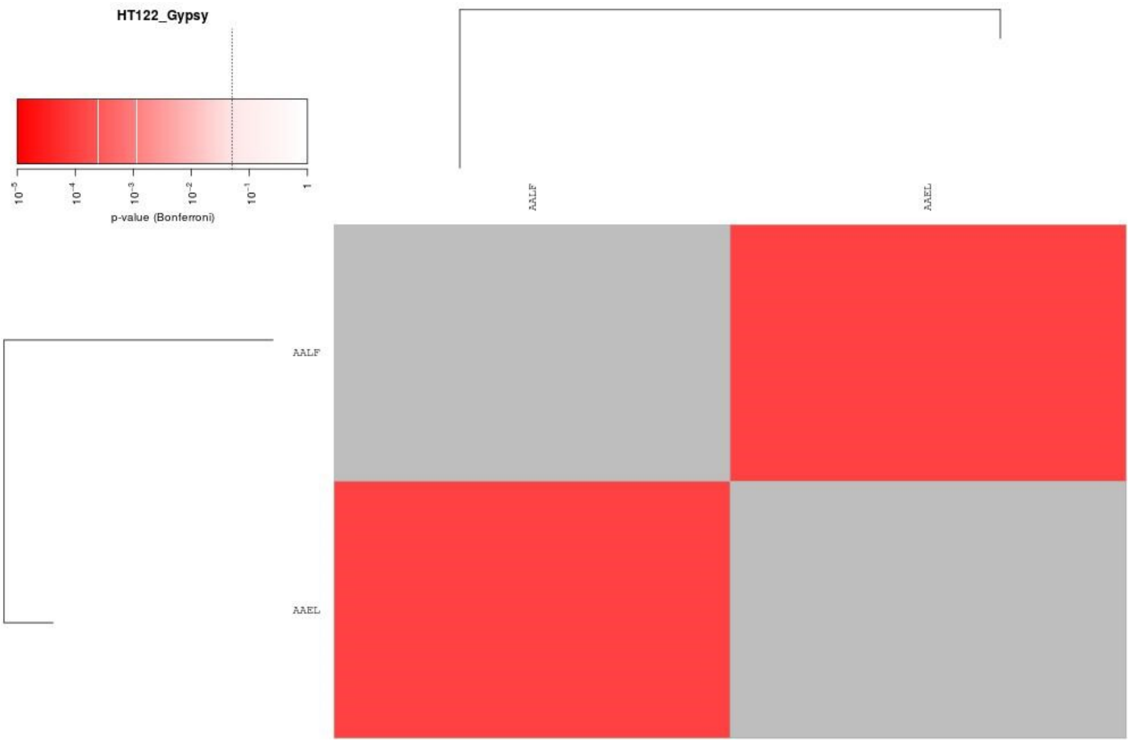
HT122_Gypsy
p-value (Bonferroni)
AALF
AAEL
AALF
AAEL

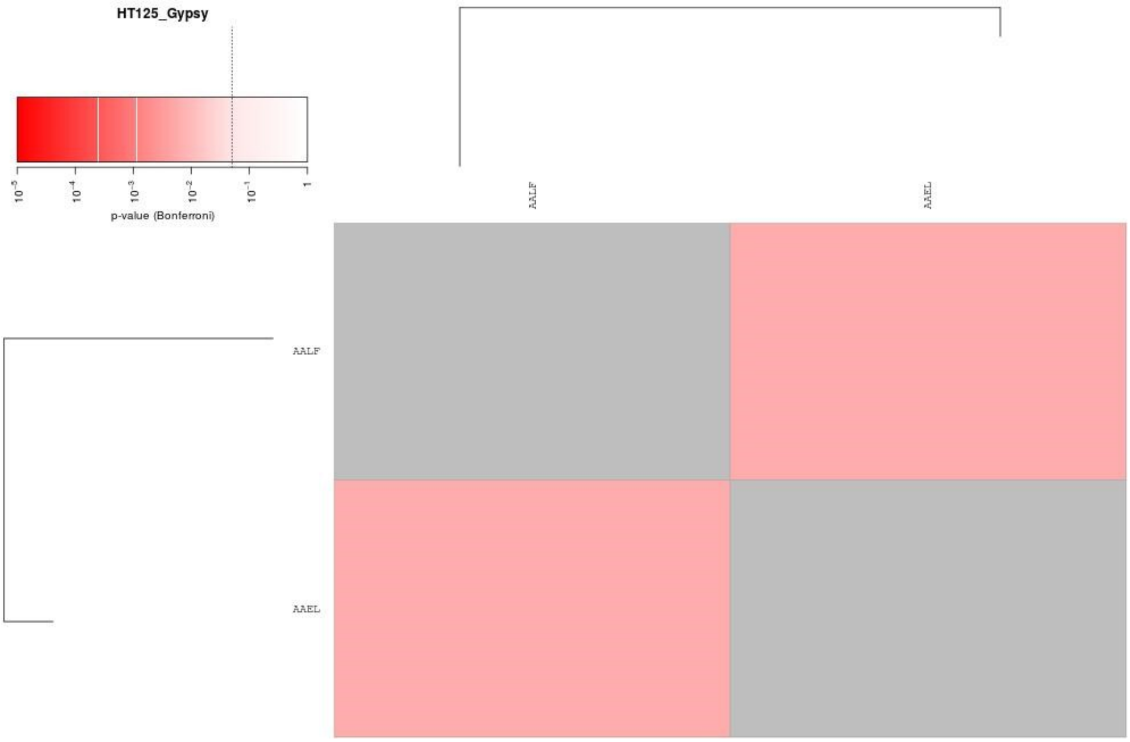
HT125_Gypsy
p-value (Bonferroni)
AALF
AAEL
AALF
AAEL

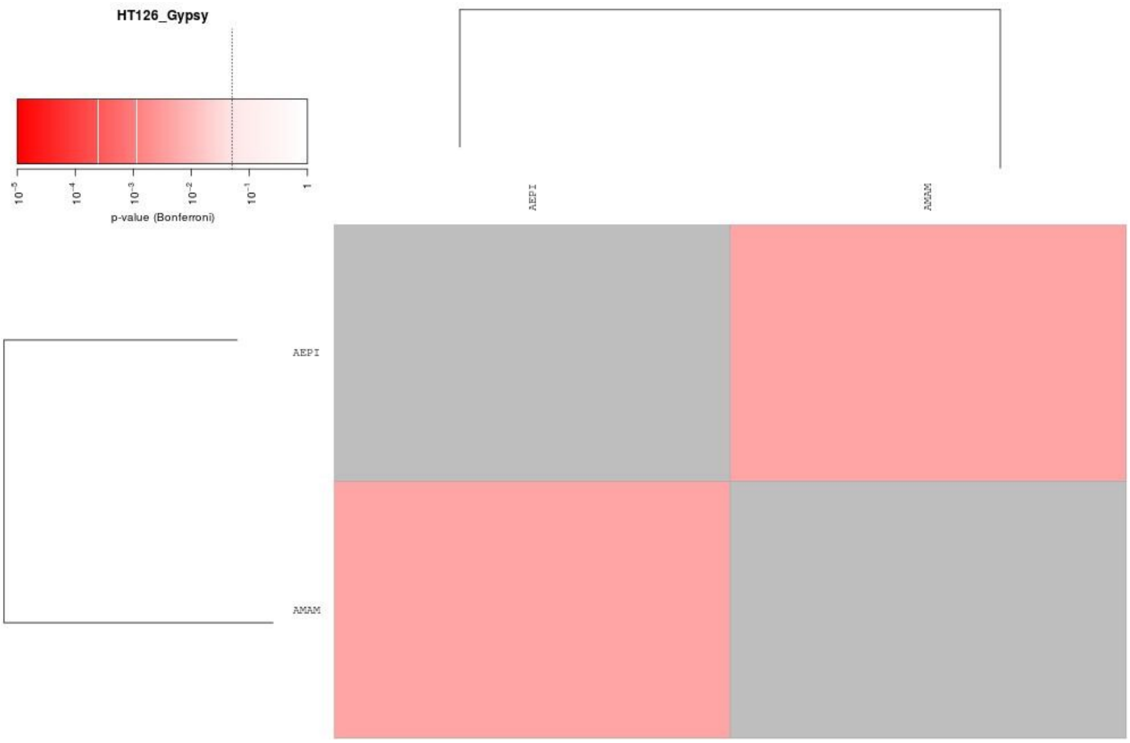
HT126_Gypsy
$10^{-5}$
$10^{-4}$
$10^{-3}$
$10^{-2}$
$10^{-1}$
1
p-value (Bonferroni)
AEPI
AMAM
AEPI
AMAM

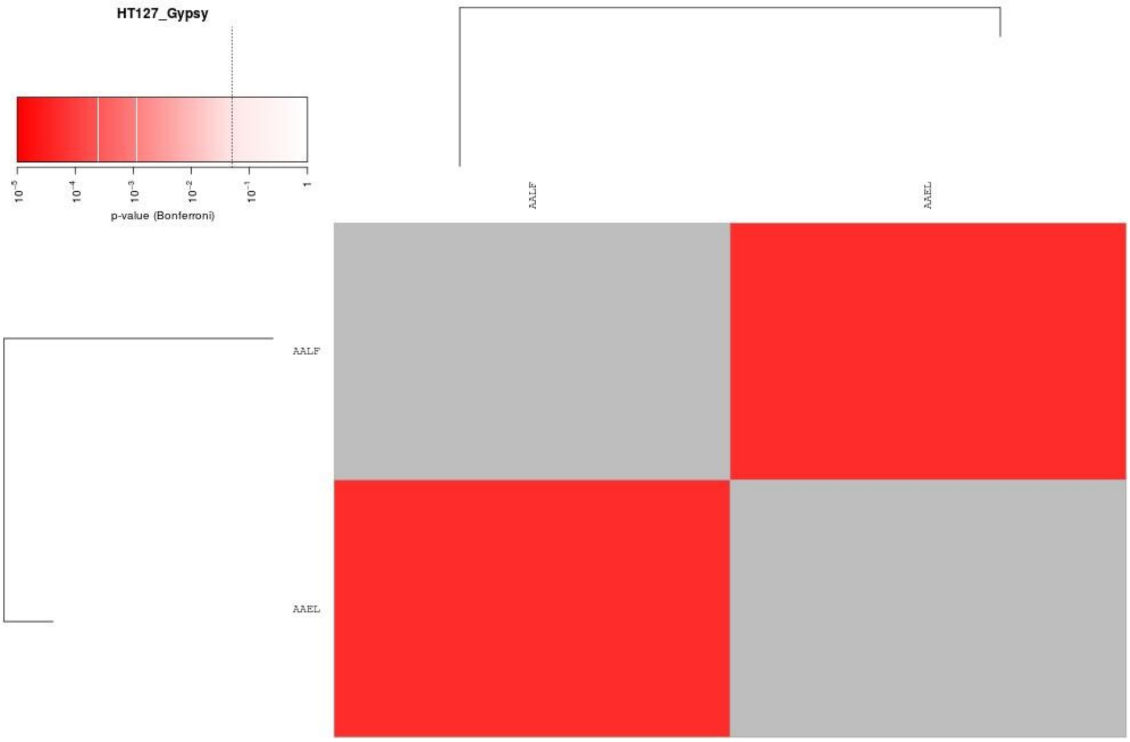
HT127_Gypsy
$10^{-5}$
$10^{-4}$
$10^{-3}$
$10^{-2}$
$10^{-1}$
1
p-value (Bonferroni)
AALF
AAEL
AALF
AAEL

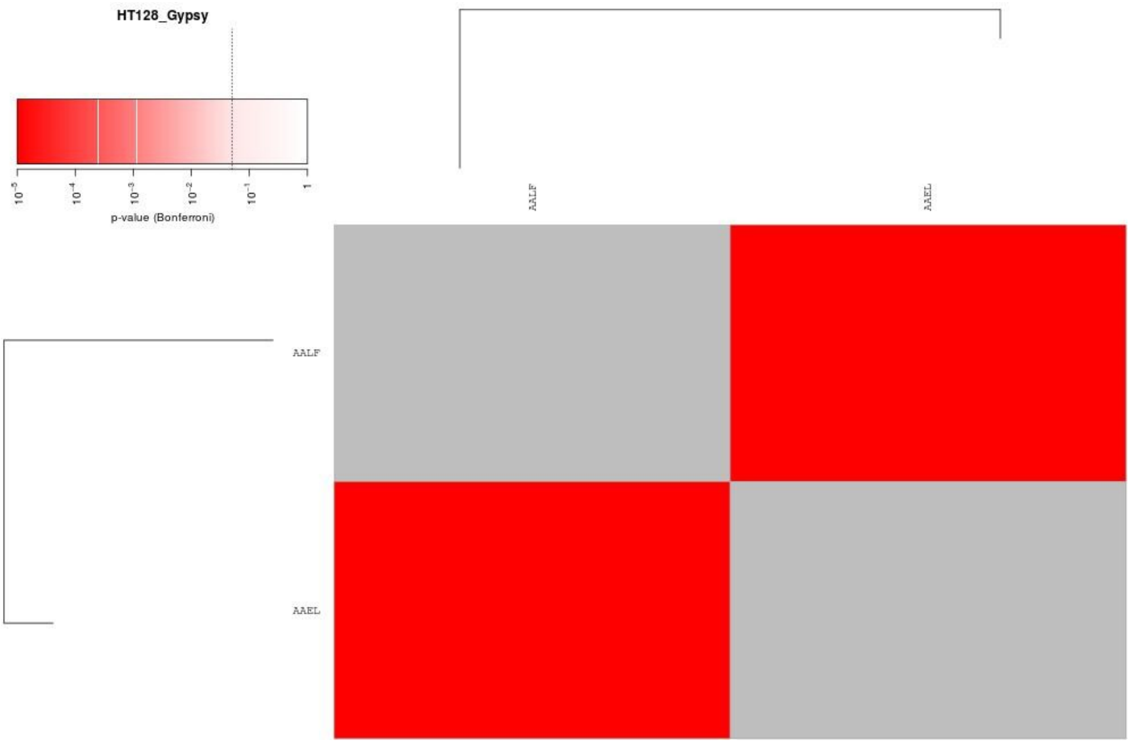
HT128_Gypsy
p-value (Bonferroni)
AALF
AAEL
AALF
AAEL

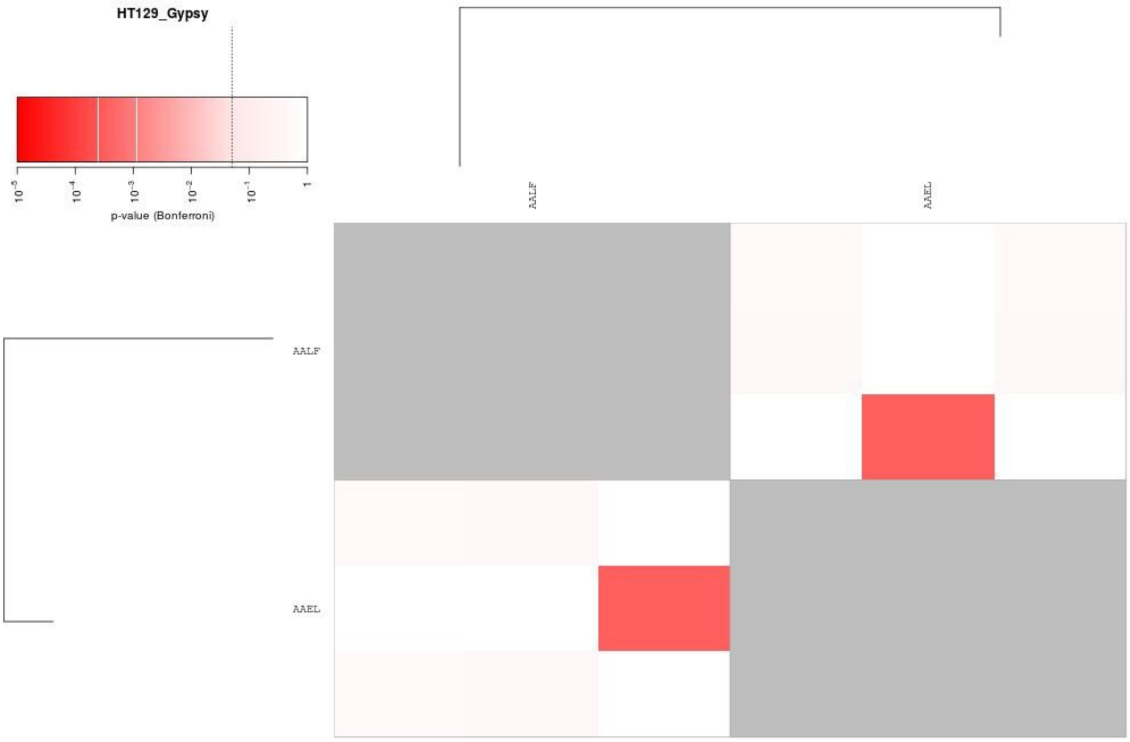
HT129_Gypsy
p-value (Bonferroni)
AALF
AAEL
AALF
AAEL

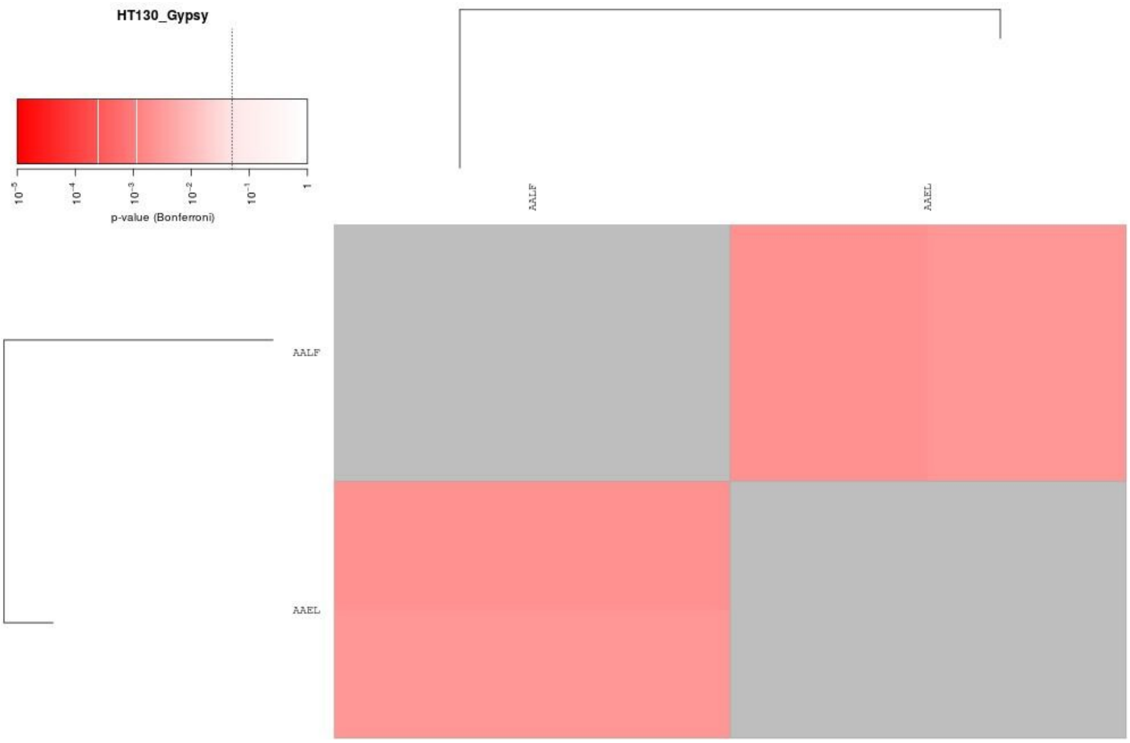
HT130_Gypsy
p-value (Bonferroni)
AALF
AAEL
AALF
AAEL

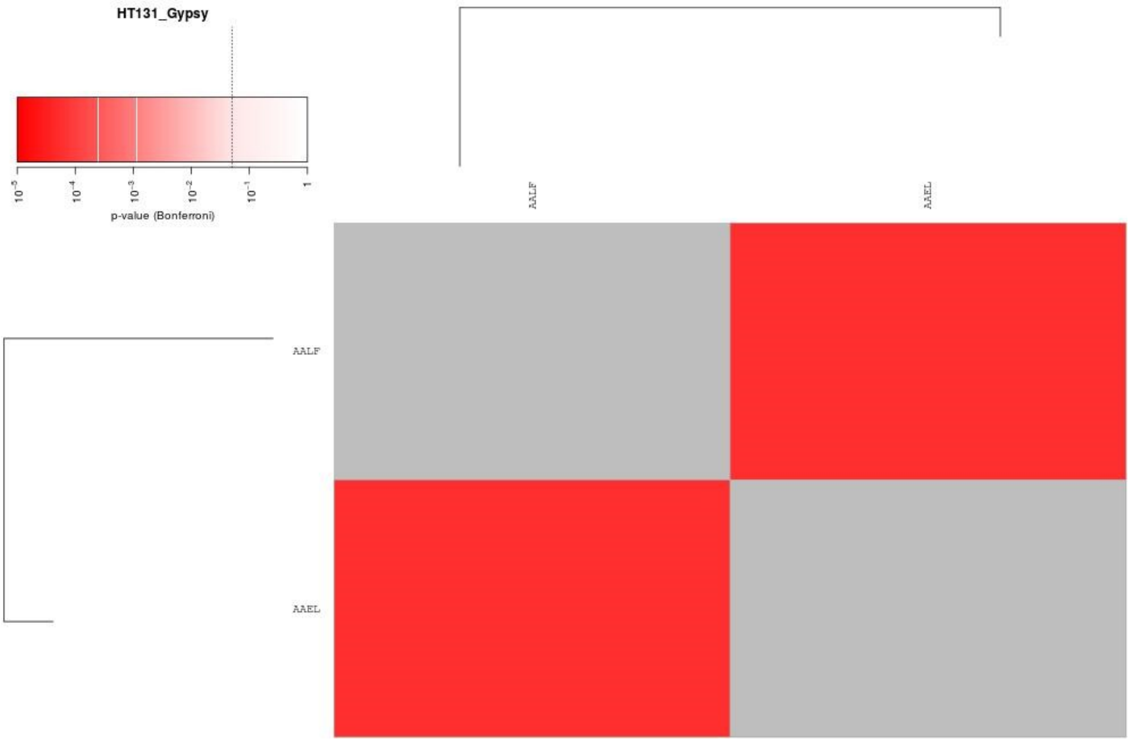
HT131_Gypsy
p-value (Bonferroni)
AALF
AAEL
AALF
AAEL

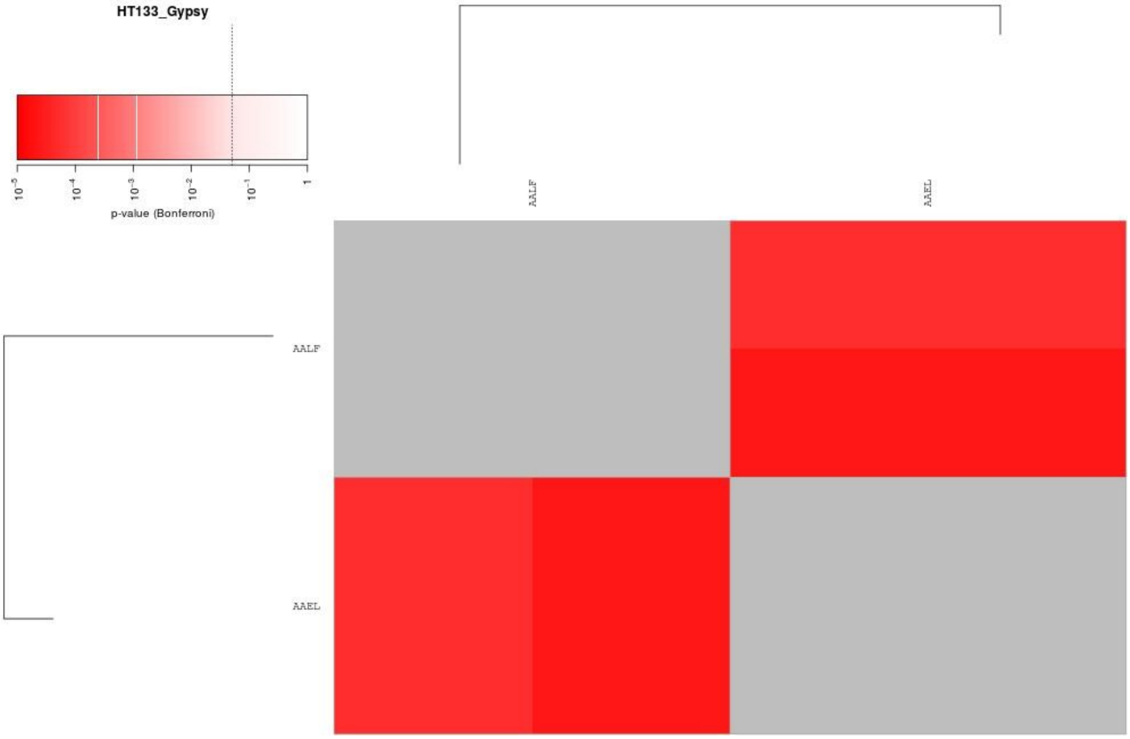
HT133_Gypsy
10^-5
10^-4
10^-3
10^-2
10^-1
1
p-value (Bonferroni)
AALF
AAEL
AALF
AAEL

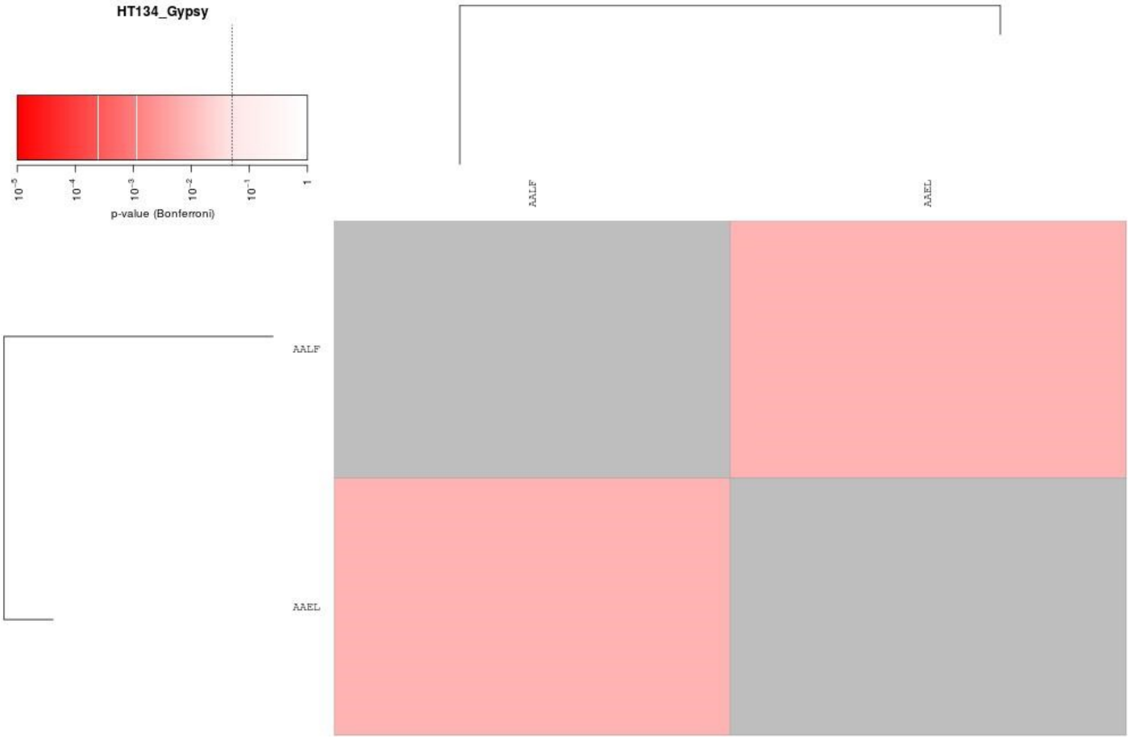
HT134_Gypsy
10^-5
10^-4
10^-3
10^-2
10^-1
1
p-value (Bonferroni)
AALF
AAEL
AALF
AAEL

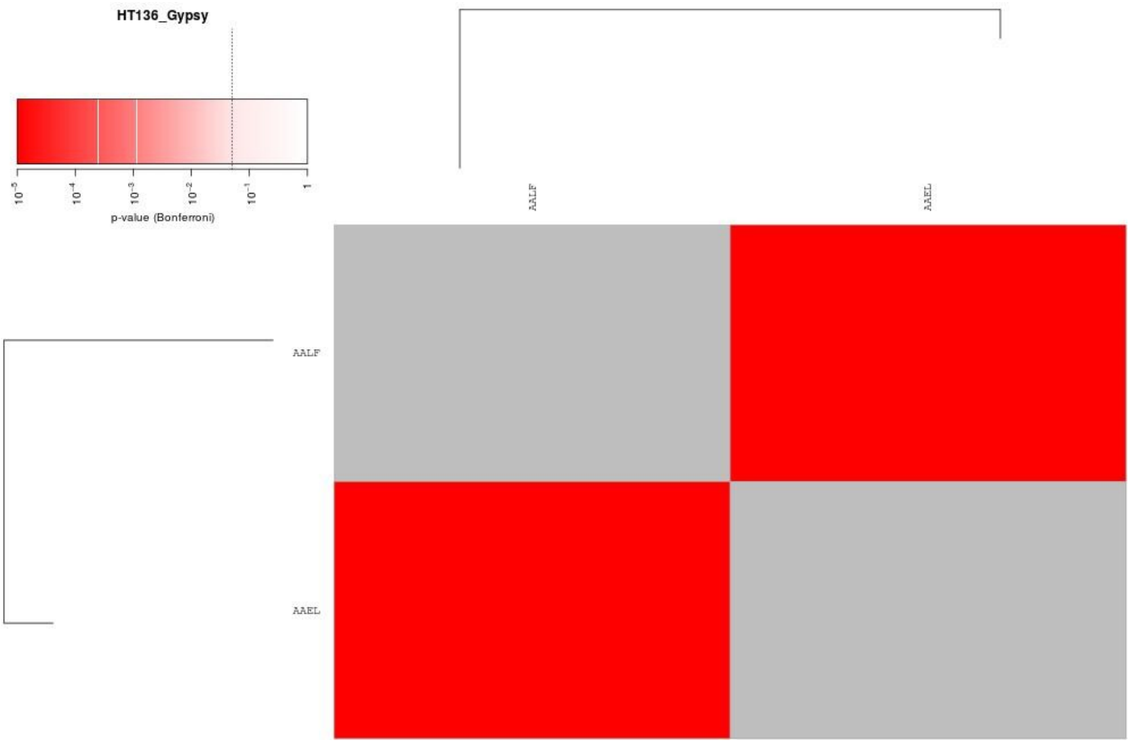
HT136_Gypsy
p-value (Bonferroni)
AALF
AAEL
AALF
AAEL

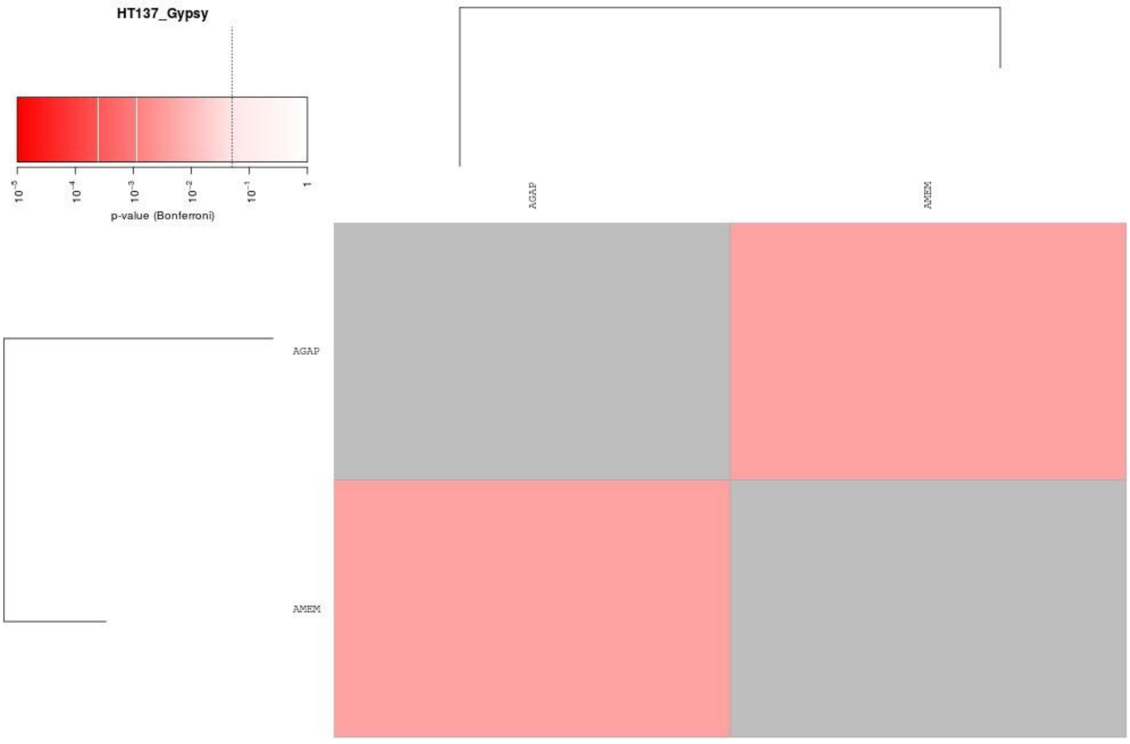
HT137_Gypsy
p-value (Bonferroni)
AGAP
AMEM
AGAP
AMEM

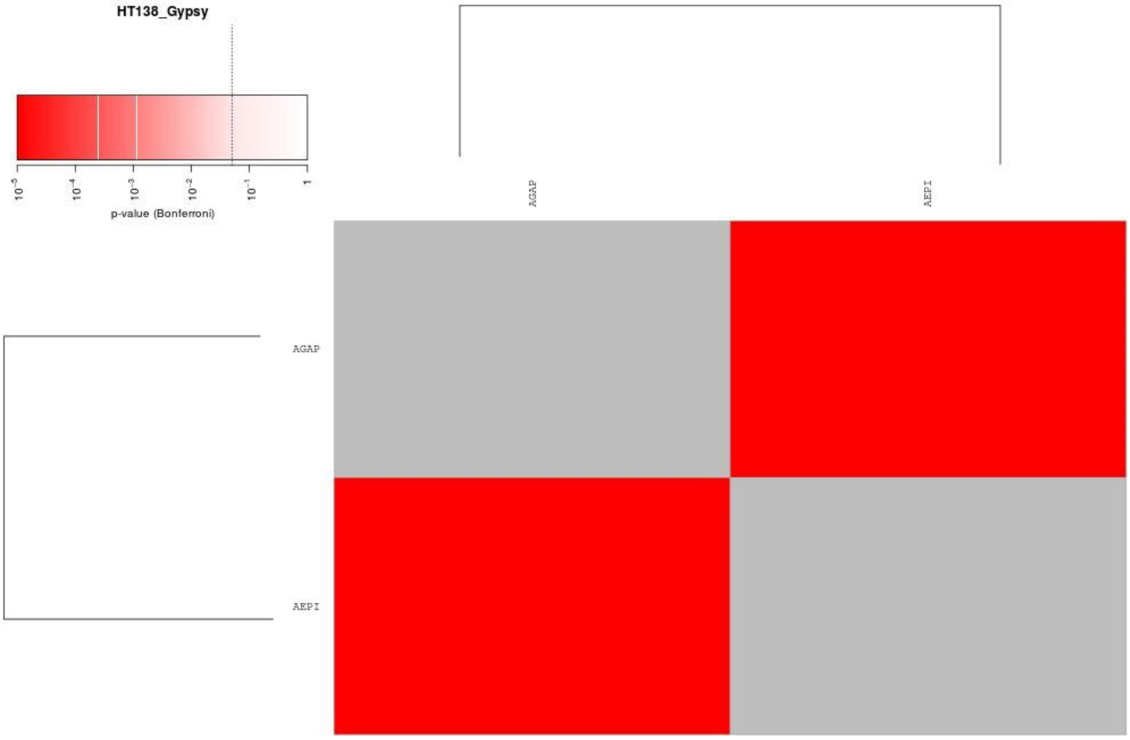
HT138_Gypsy
p-value (Bonferroni)
AGAP
AEPI
AGAP
AEPI

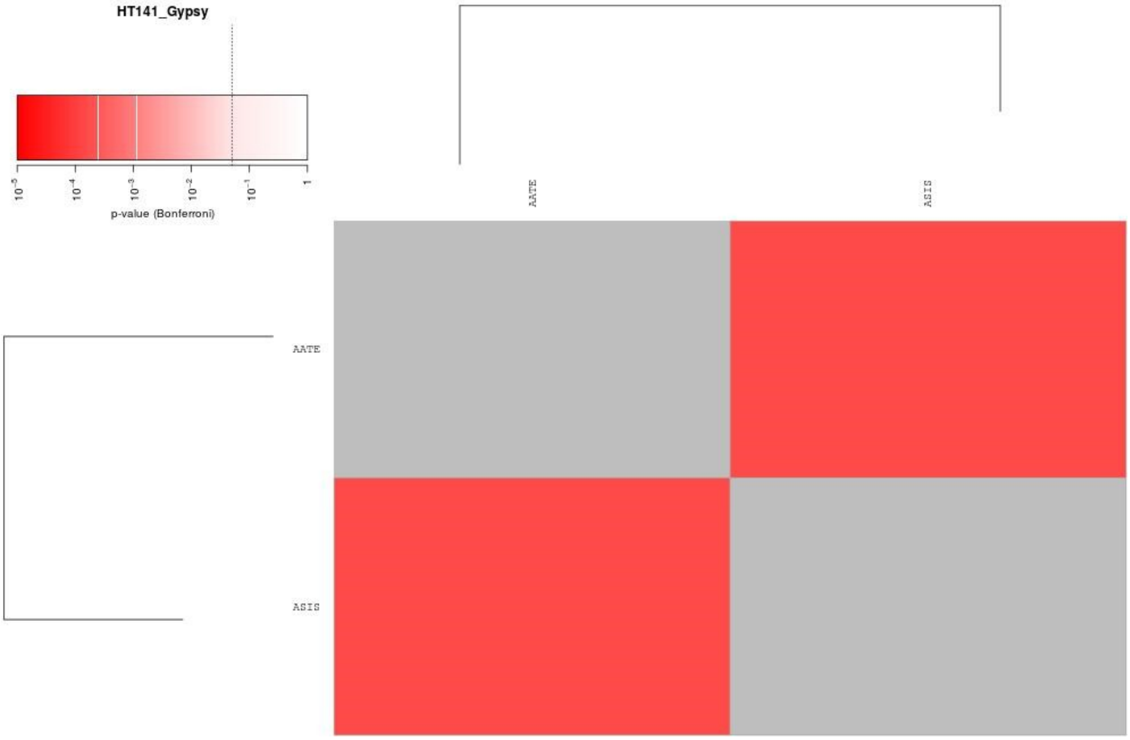
HT141_Gypsy
p-value (Bonferroni)
AATE
ASIS
AATE
ASIS

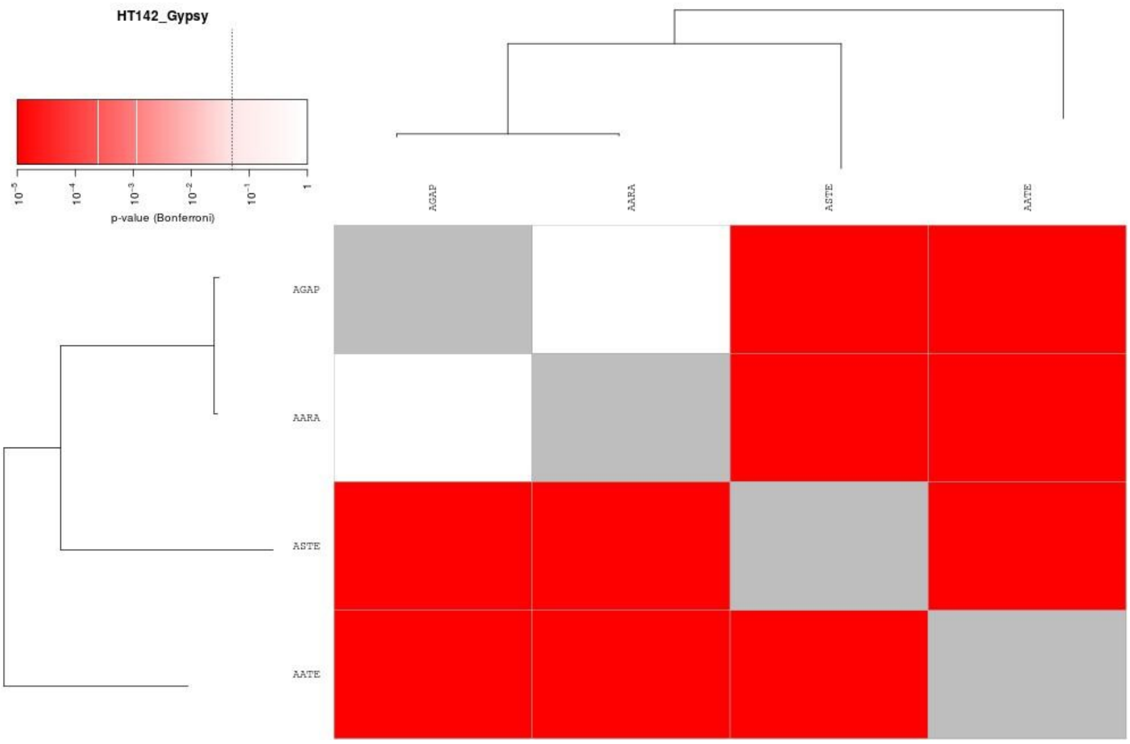
HT142_Gypsy
p-value (Bonferroni)
AGAP
AARA
ASTE
AATE

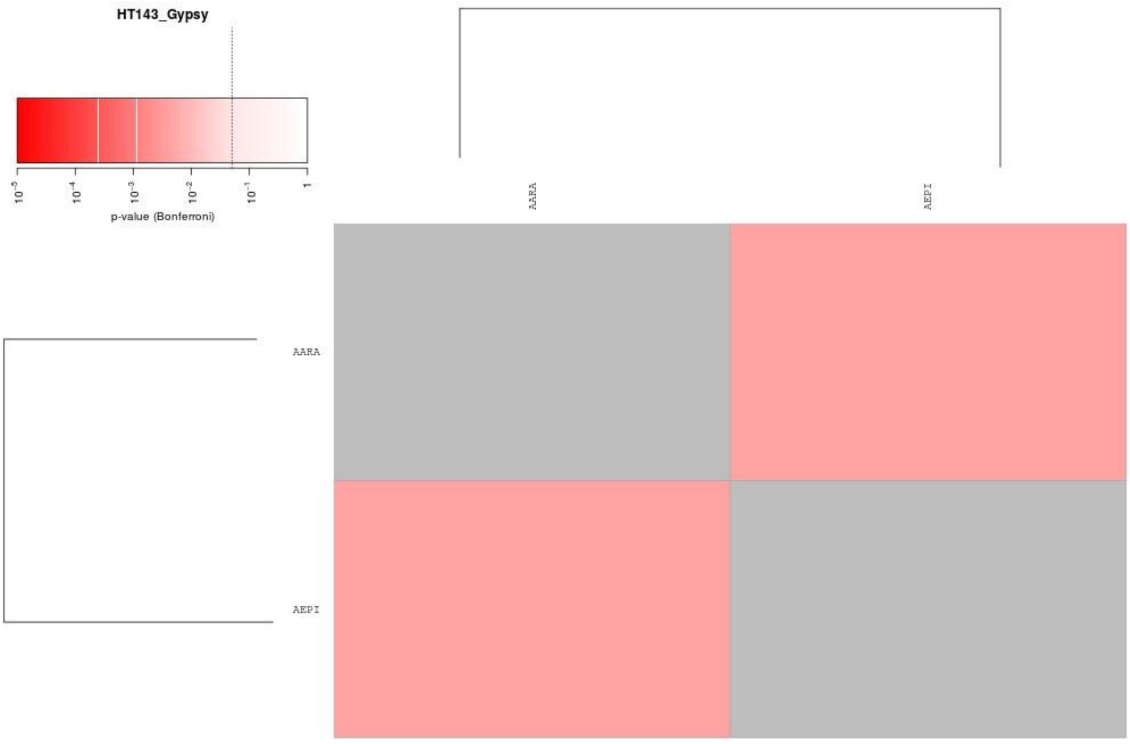
HT143_Gypsy
p-value (Bonferroni)
AARA
AEPI

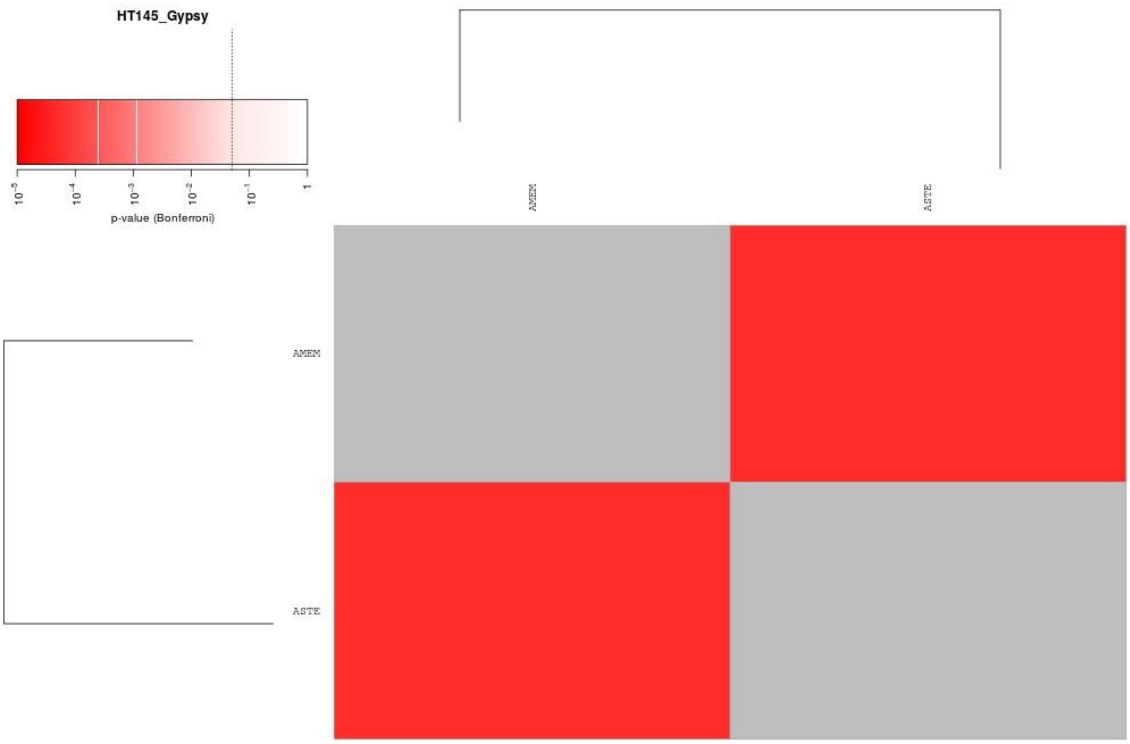
HT145_Gypsy
p-value (Bonferroni)
AMEM
ASTE
AMEM
ASTE

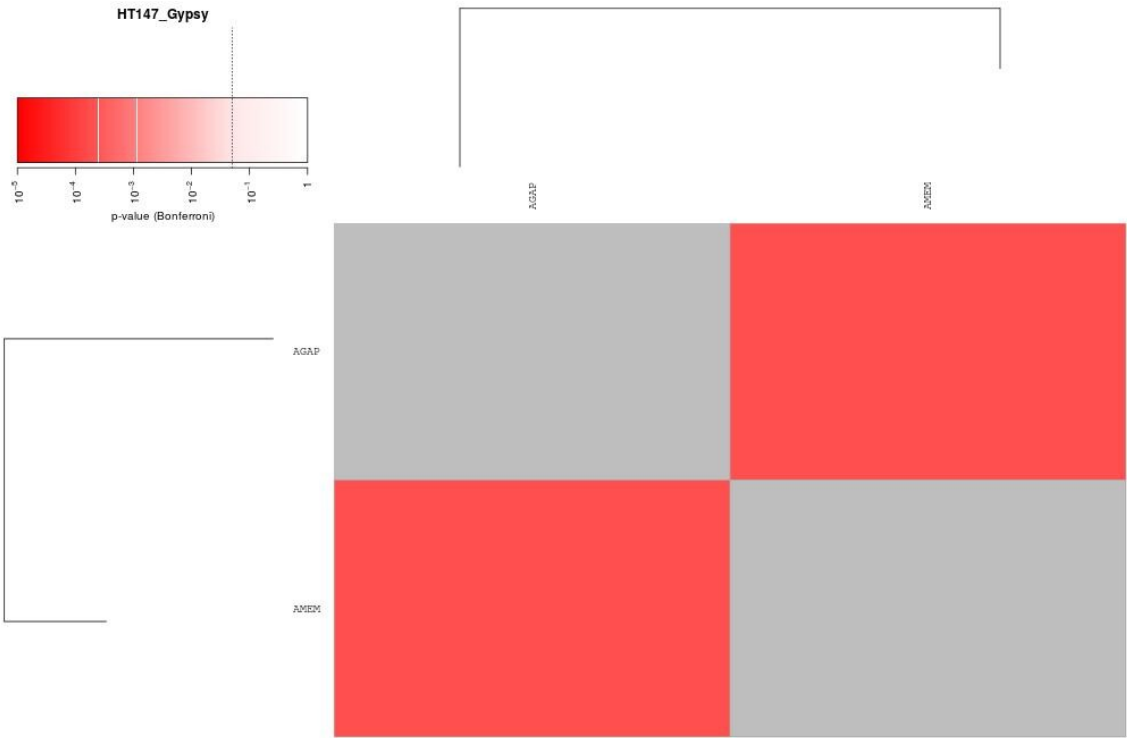
HT147_Gypsy
p-value (Bonferroni)
AGAP
AMEM
AGAP
AMEM

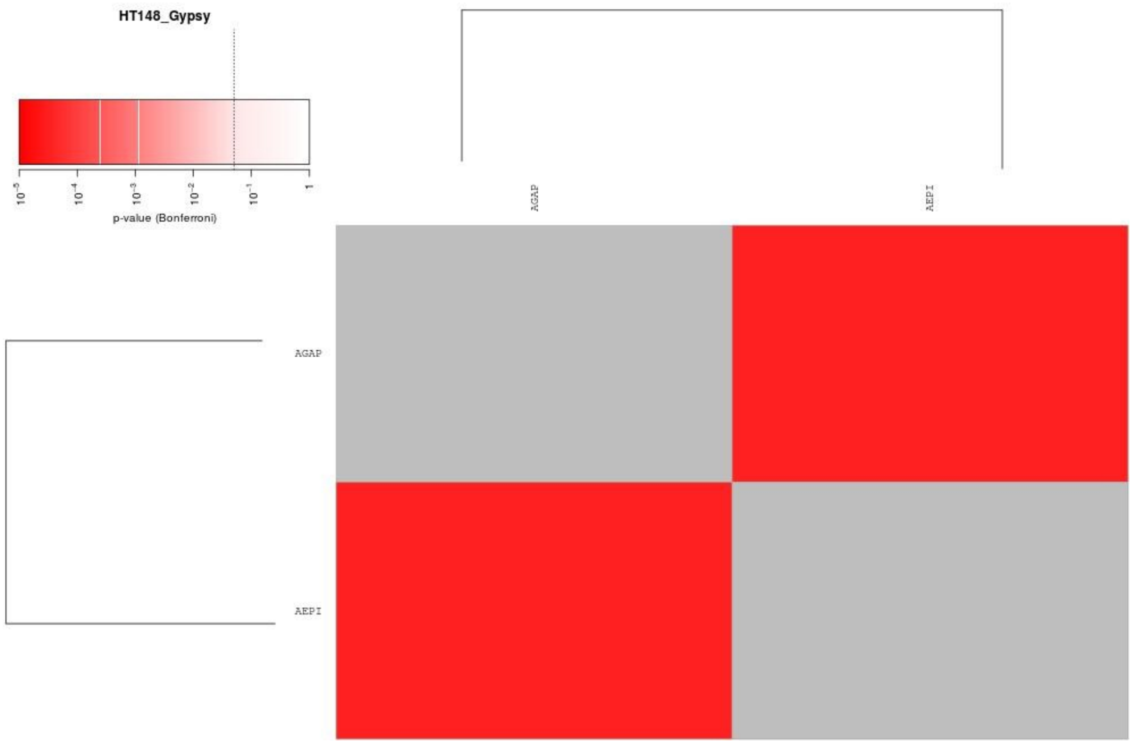
HT148_Gypsy
p-value (Bonferroni)
AGAP
AEPI
AGAP
AEPI

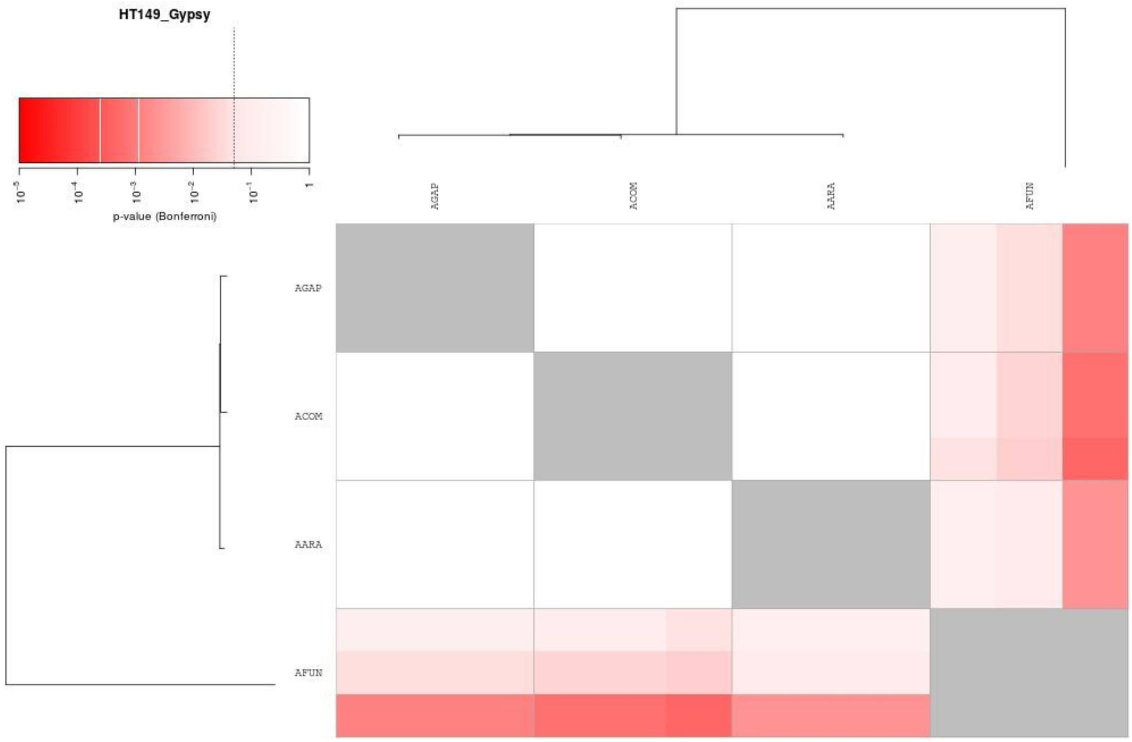
HT149_Gypsy
p-value (Bonferroni)
AGAP
ACOM
AARA
AFUN
AGAP
ACOM
AARA
AFUN

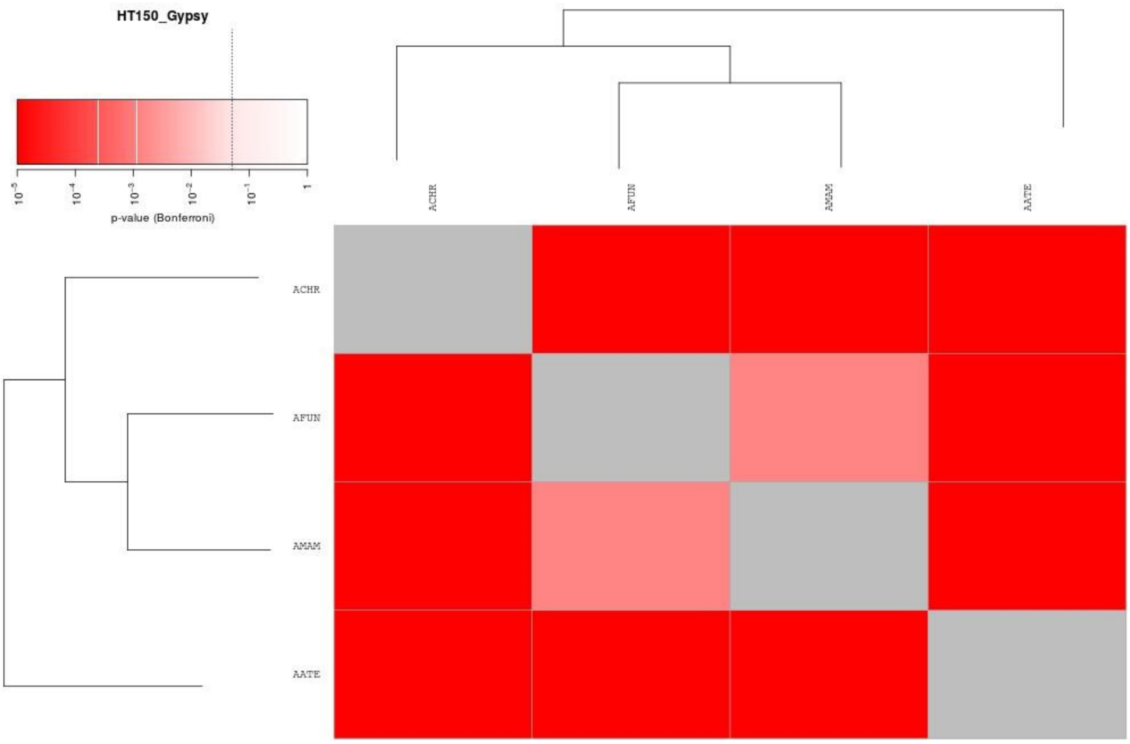
HT150_Gypsy
p-value (Bonferroni)
ACHR
AFUN
AMAM
AATE

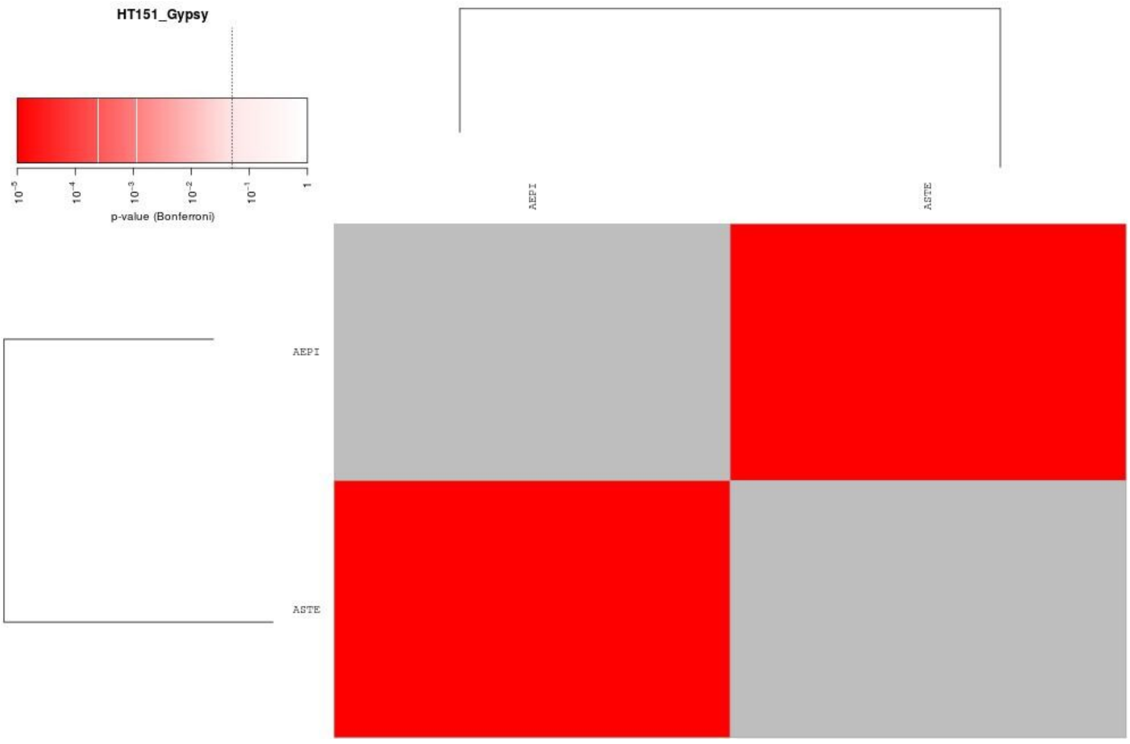
HT151_Gypsy
p-value (Bonferroni)
AEPI
ASTE

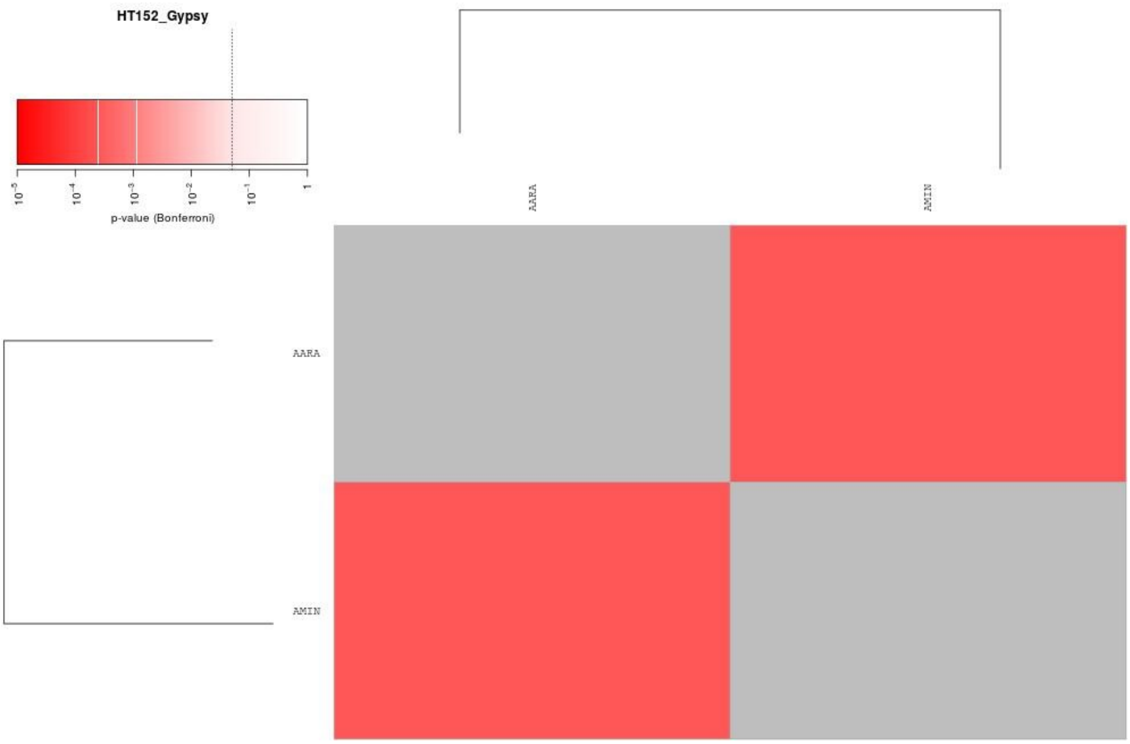
HT152_Gypsy
$10^{-5}$
$10^{-4}$
$10^{-3}$
$10^{-2}$
$10^{-1}$
1
p-value (Bonferroni)
AARA
AMIN
AARA
AMIN

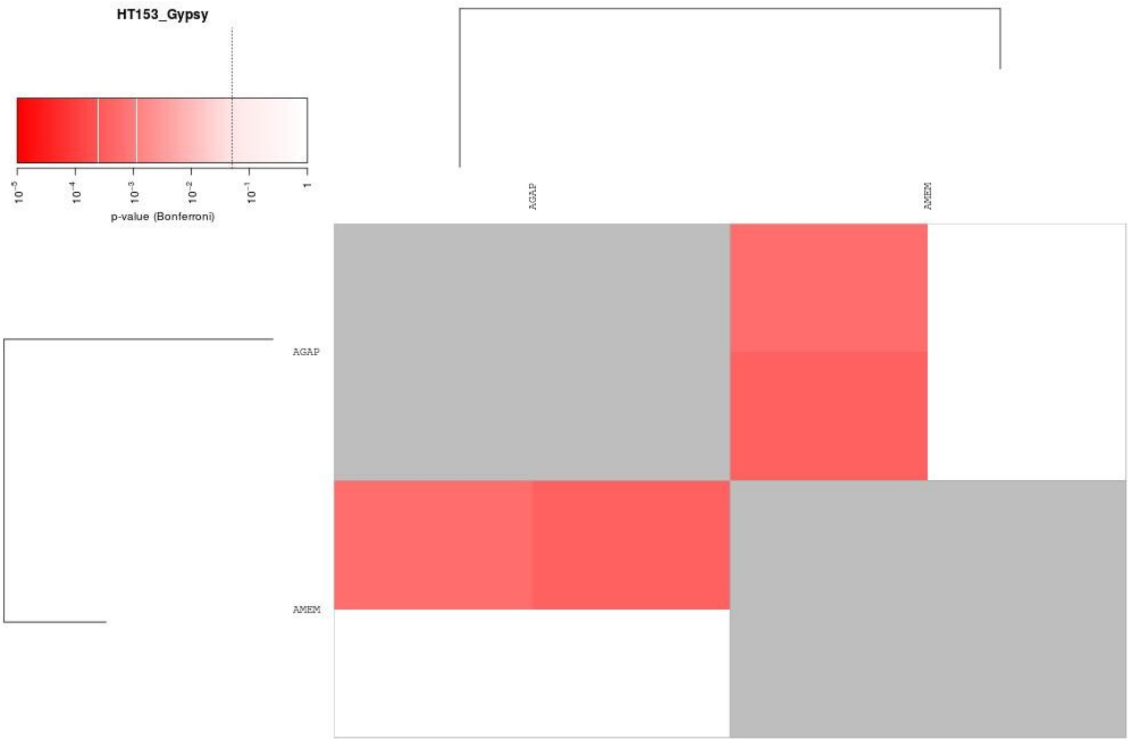
HT153_Gypsy
$10^{-5}$
$10^{-4}$
$10^{-3}$
$10^{-2}$
$10^{-1}$
1
p-value (Bonferroni)
AGAP
AMEM
AGAP
AMEM

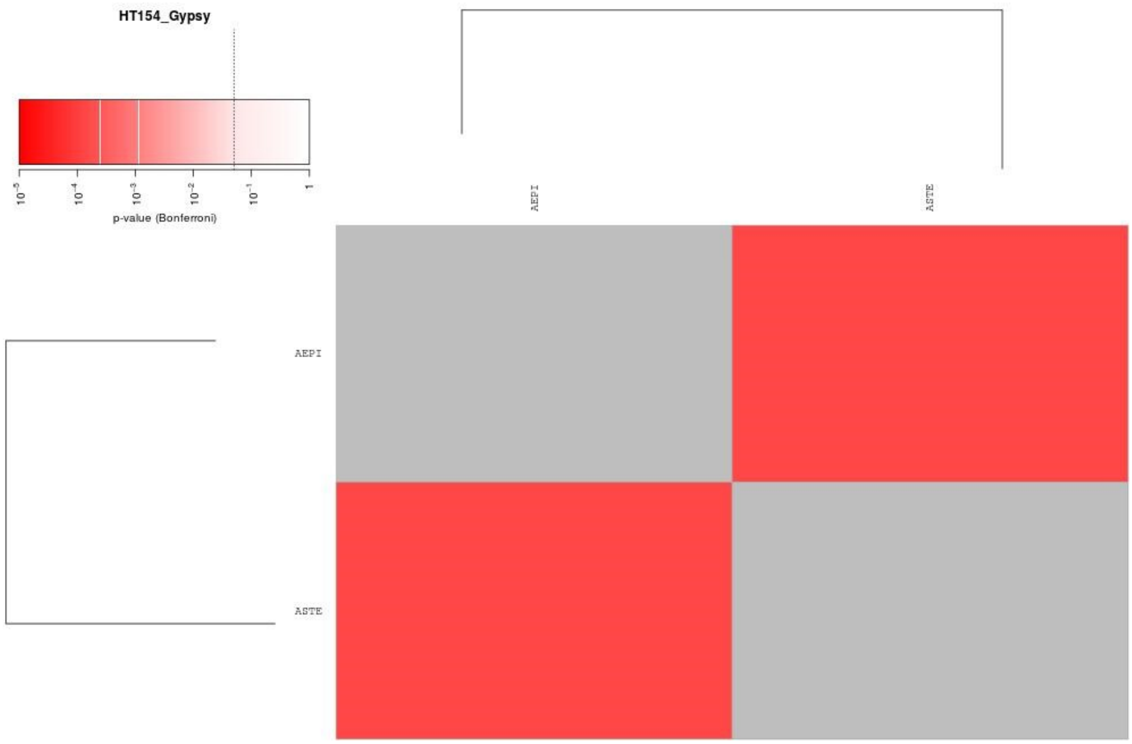
HT154_Gypsy
p-value (Bonferroni)
AEPI
ASTE
AEPI
ASTE

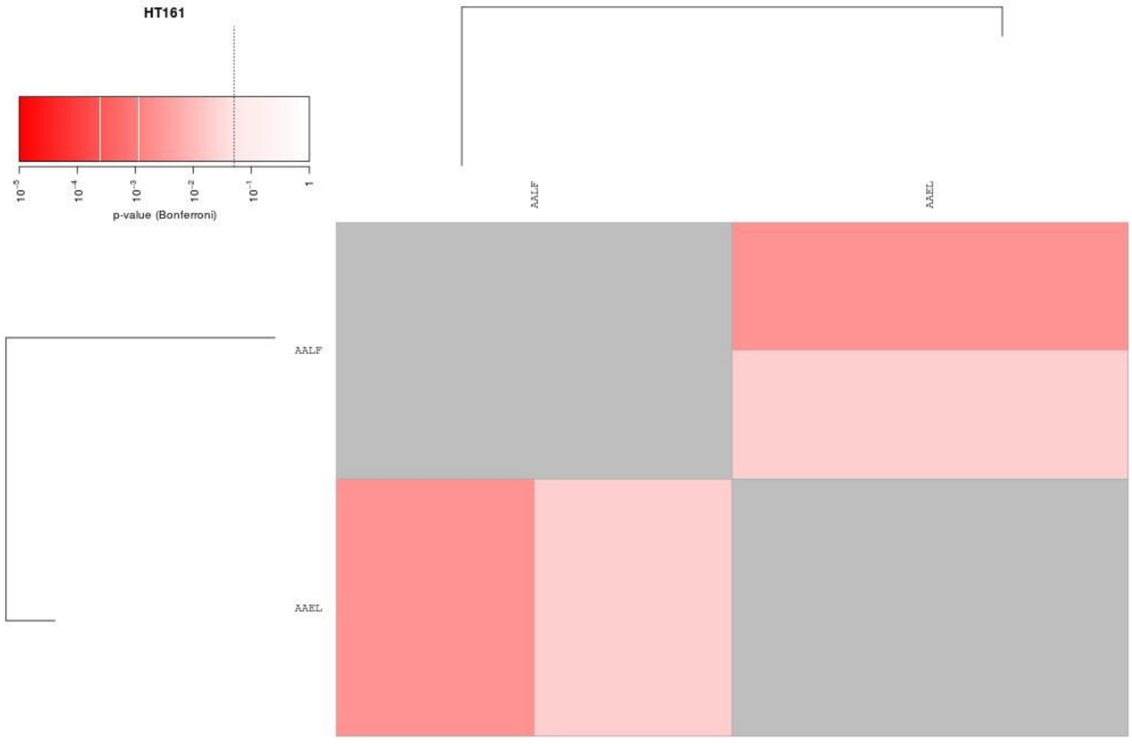
HT161
p-value (Bonferroni)
AALF
AAEL
AALF
AAEL

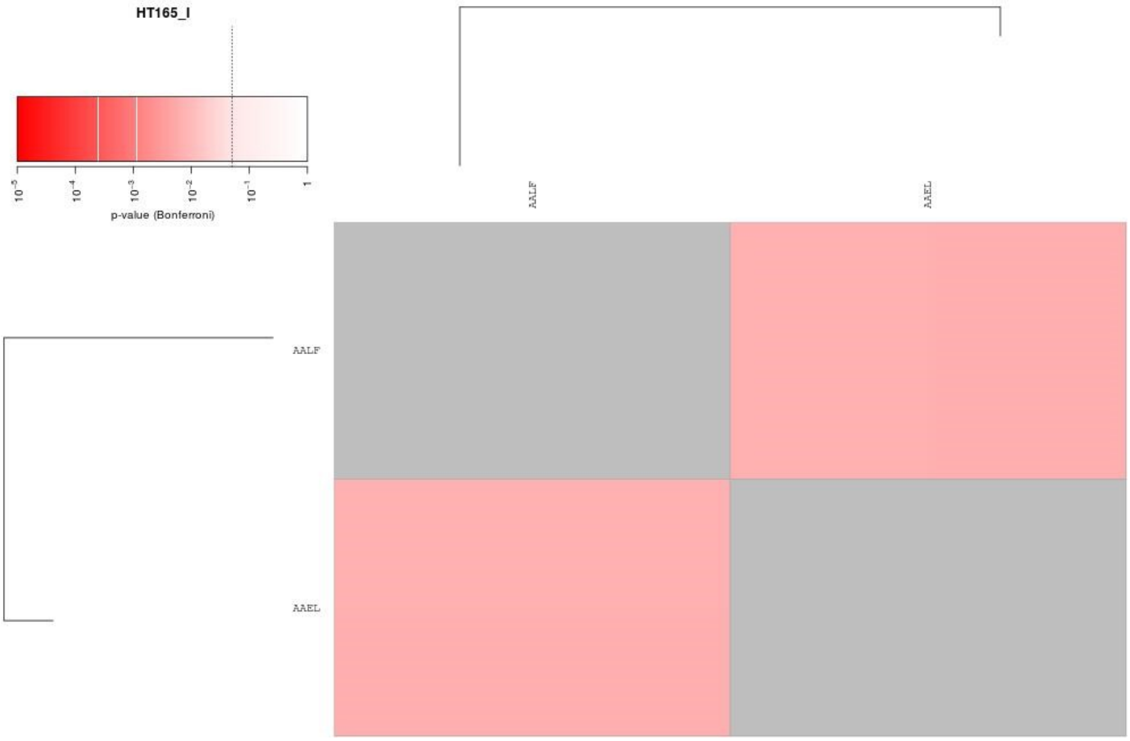
HT165_I
p-value (Bonferroni)
AALF
AAEL
AALF
AAEL

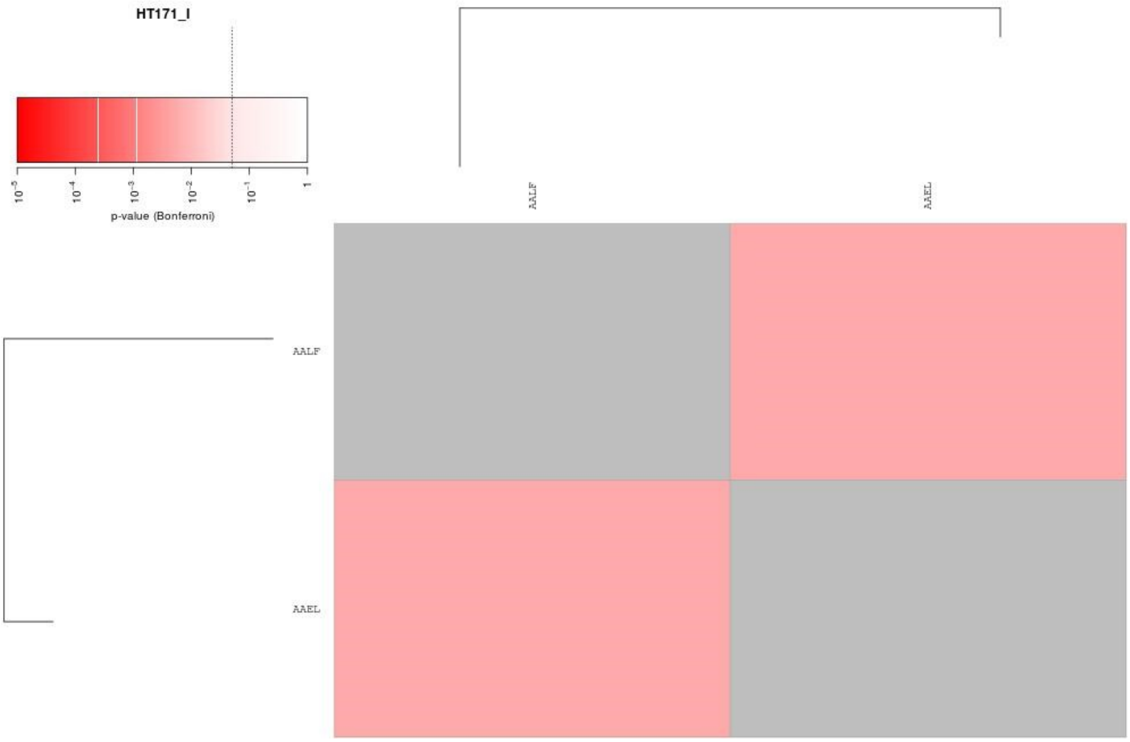
HT171_I
p-value (Bonferroni)
AALF
AAEL
AALF
AAEL

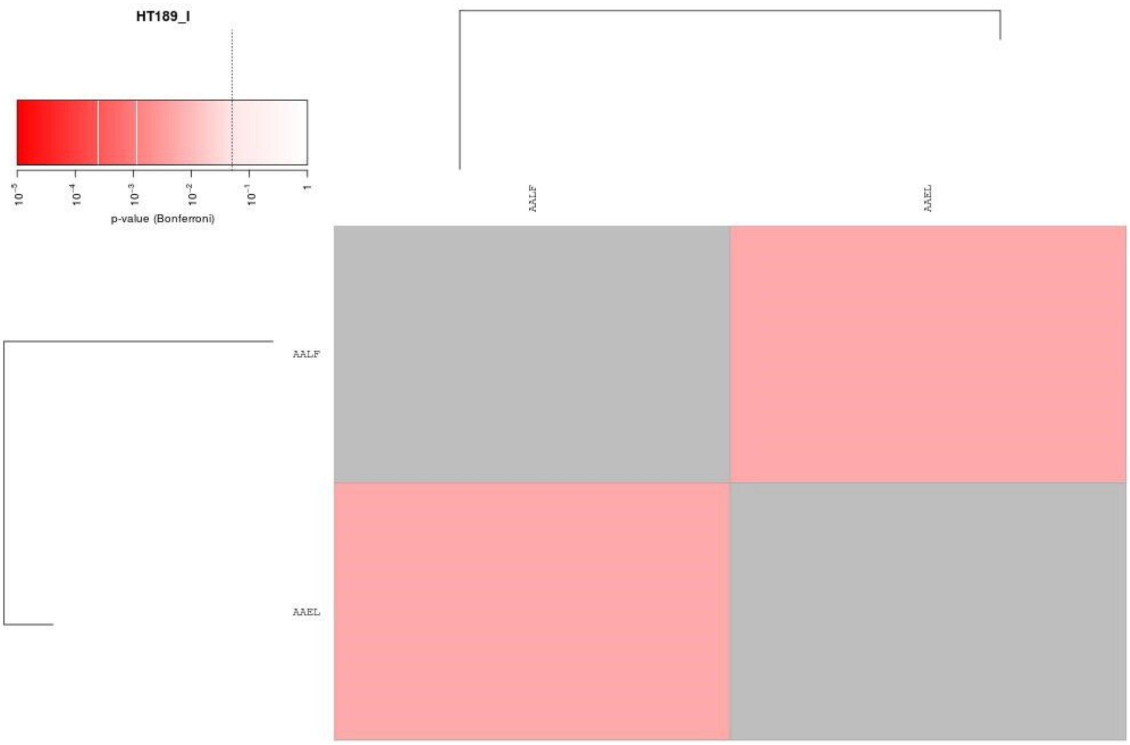
HT189_I
p-value (Bonferroni)
AALF
AAEL
AALF
AAEL

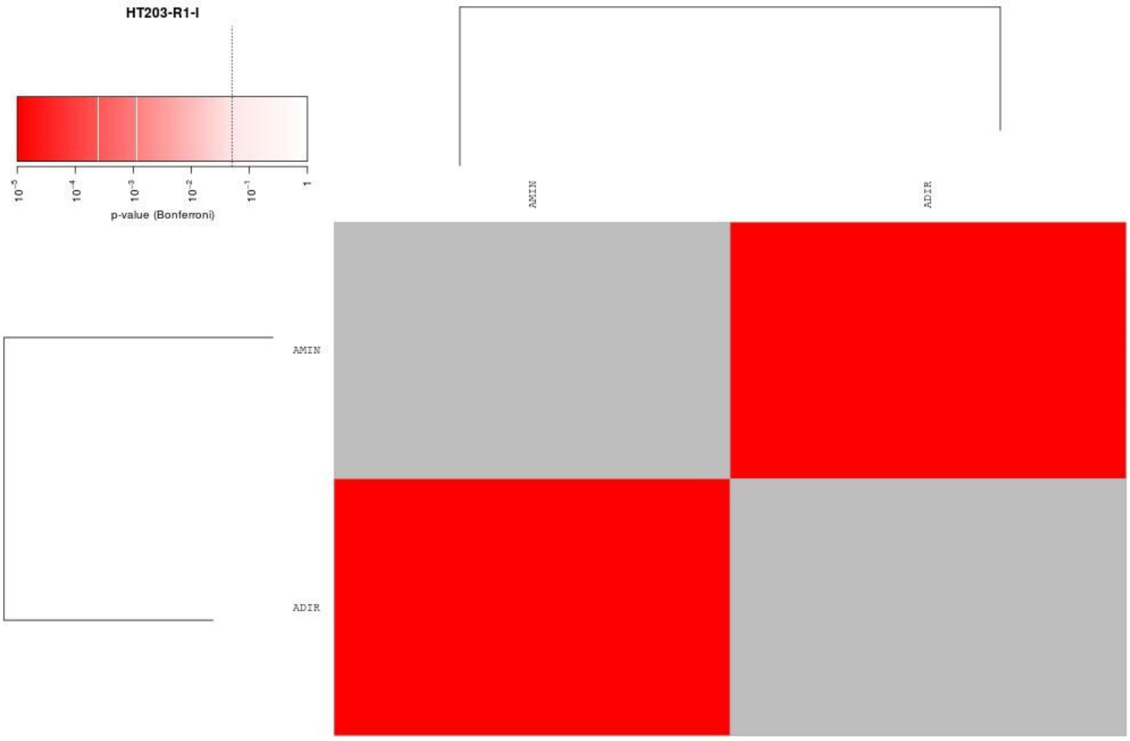
HT203-R1-I
p-value (Bonferroni)
AMIN
ADIR
AMIN
ADIR

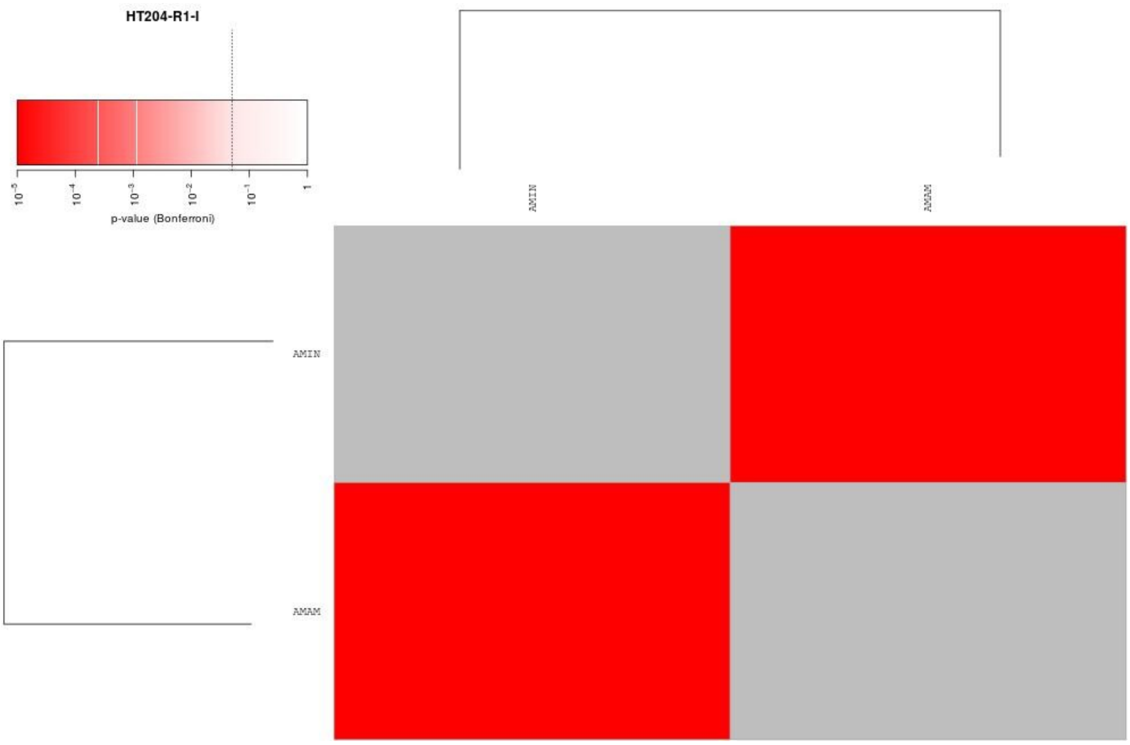
HT204-R1-I
p-value (Bonferroni)
AMIN
AMAM
AMIN
AMAM

## Jockey

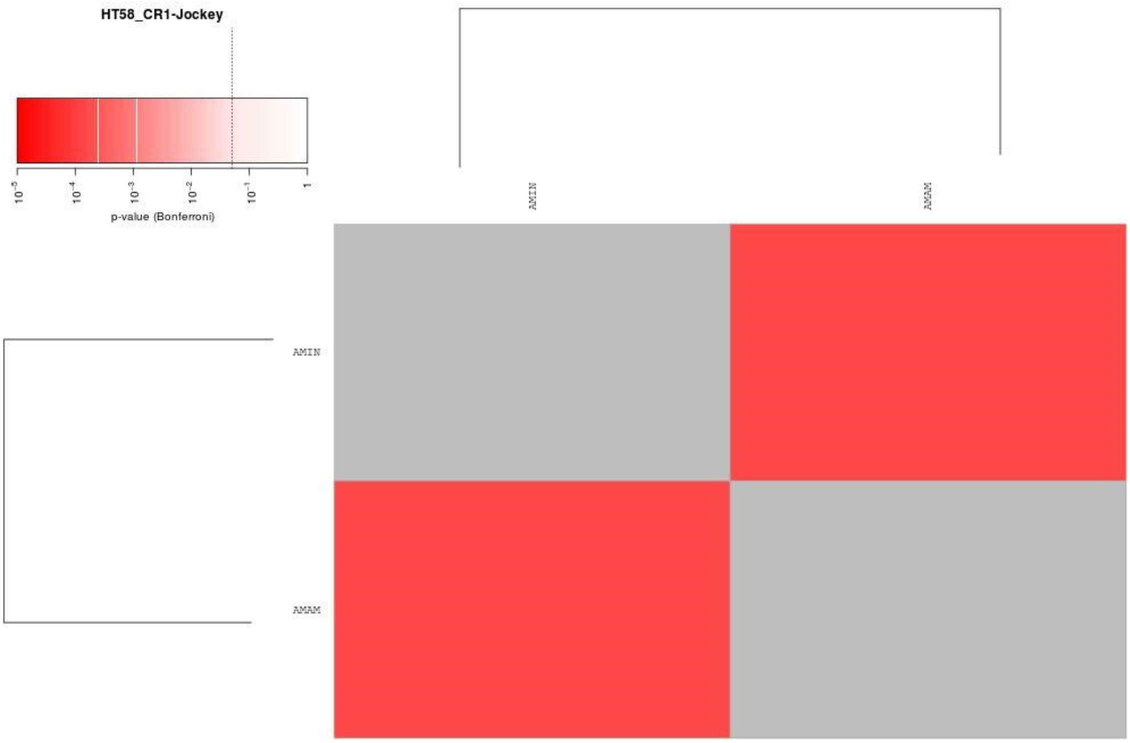
HT58_CR1-Jockey
p-value (Bonferroni)
AMIN
AMAM
AMIN
AMAM

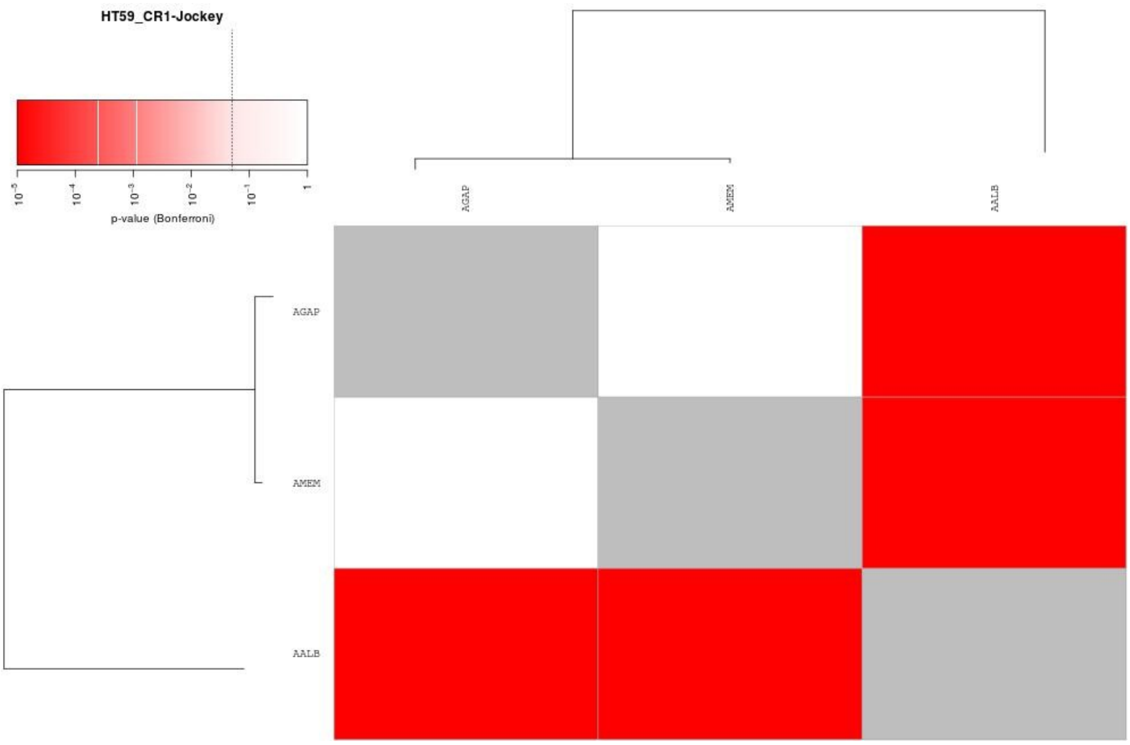
HT59_CR1-Jockey
p-value (Bonferroni)
AGAP
AMEM
AALB

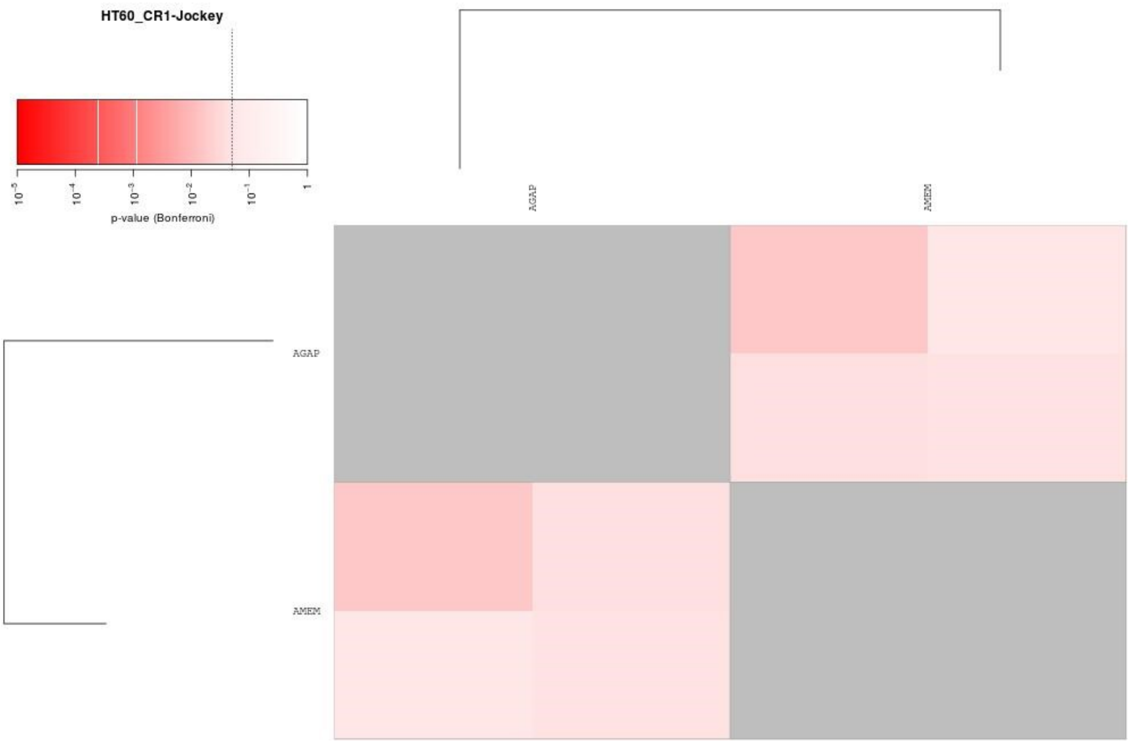
HT60_CR1-Jockey
p-value (Bonferroni)
AGAP
AMEM

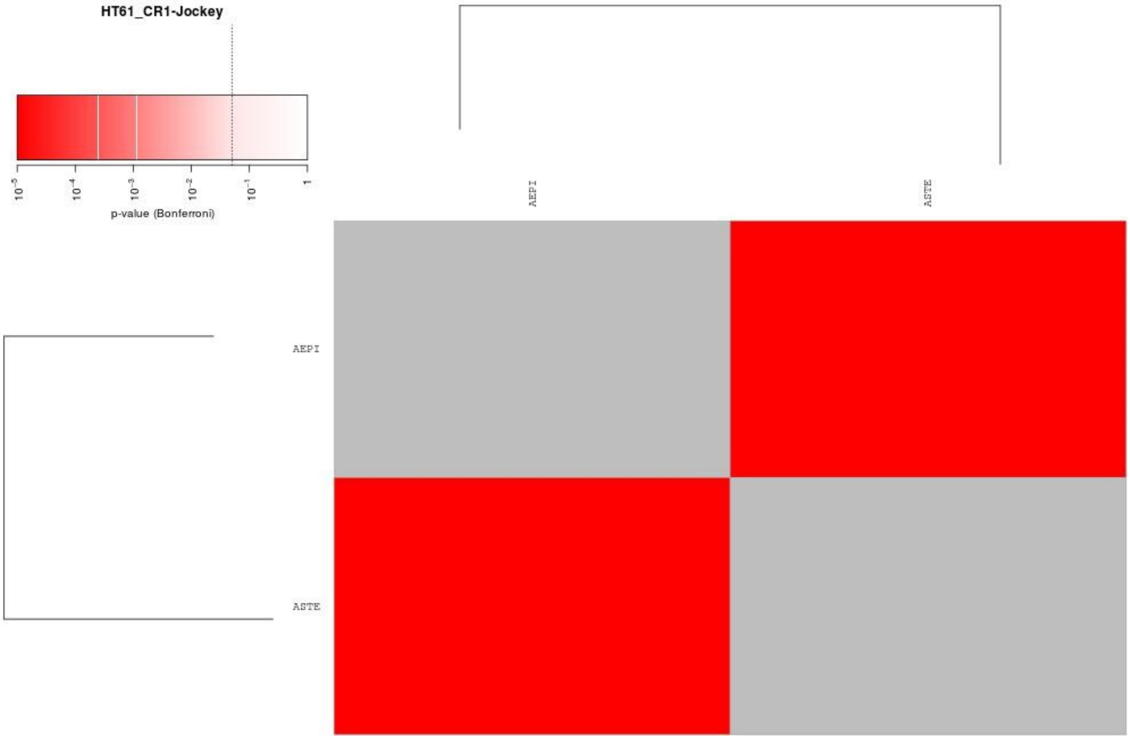
HT61_CR1-Jockey
p-value (Bonferroni)
AEPI
ASTE
AEPI
ASTE

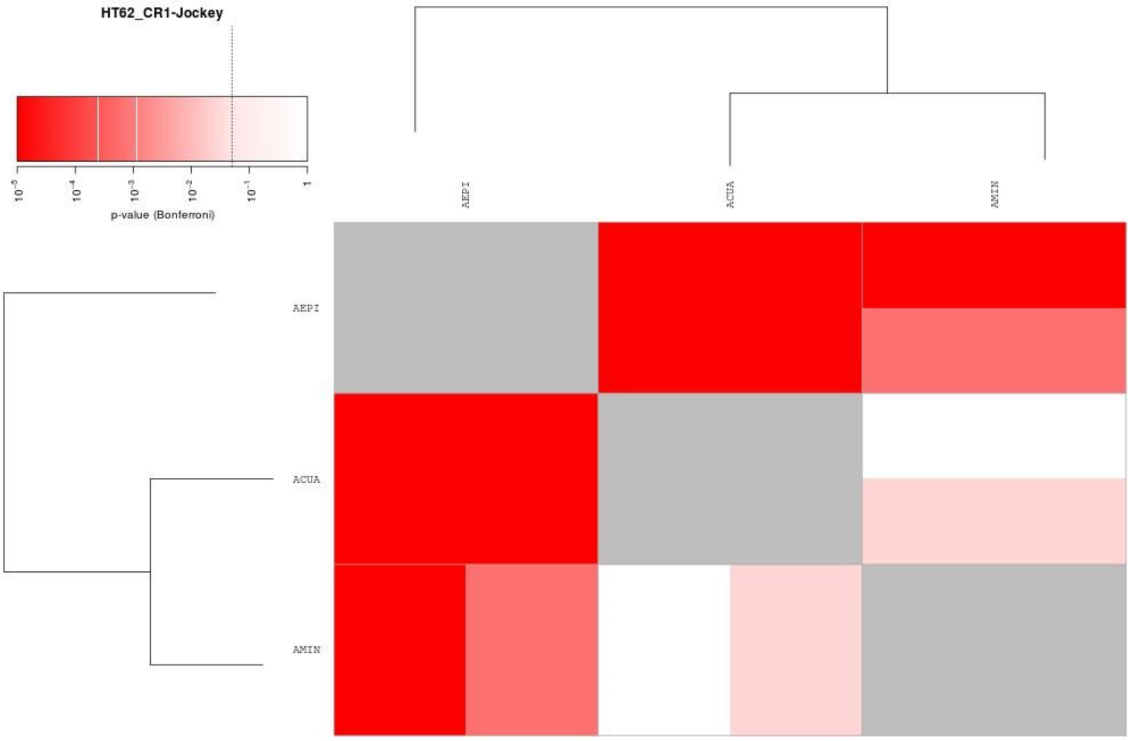
HT62_CR1-Jockey
p-value (Bonferroni)
AEPI
ACUA
AMIN
AEPI
ACUA
AMIN

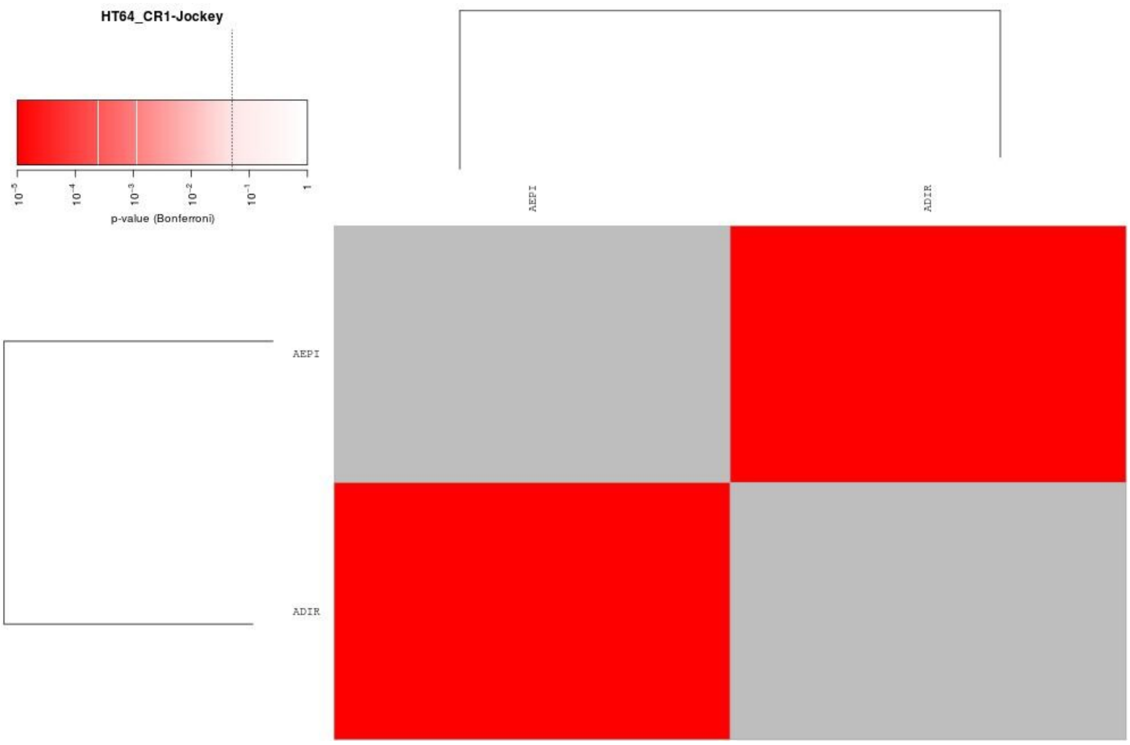
HT64_CR1-Jockey
$10^{-5}$ $10^{-4}$ $10^{-3}$ $10^{-2}$ $10^{-1}$ 1
p-value (Bonferroni)
AEPI
ADIR
AEPI
ADIR

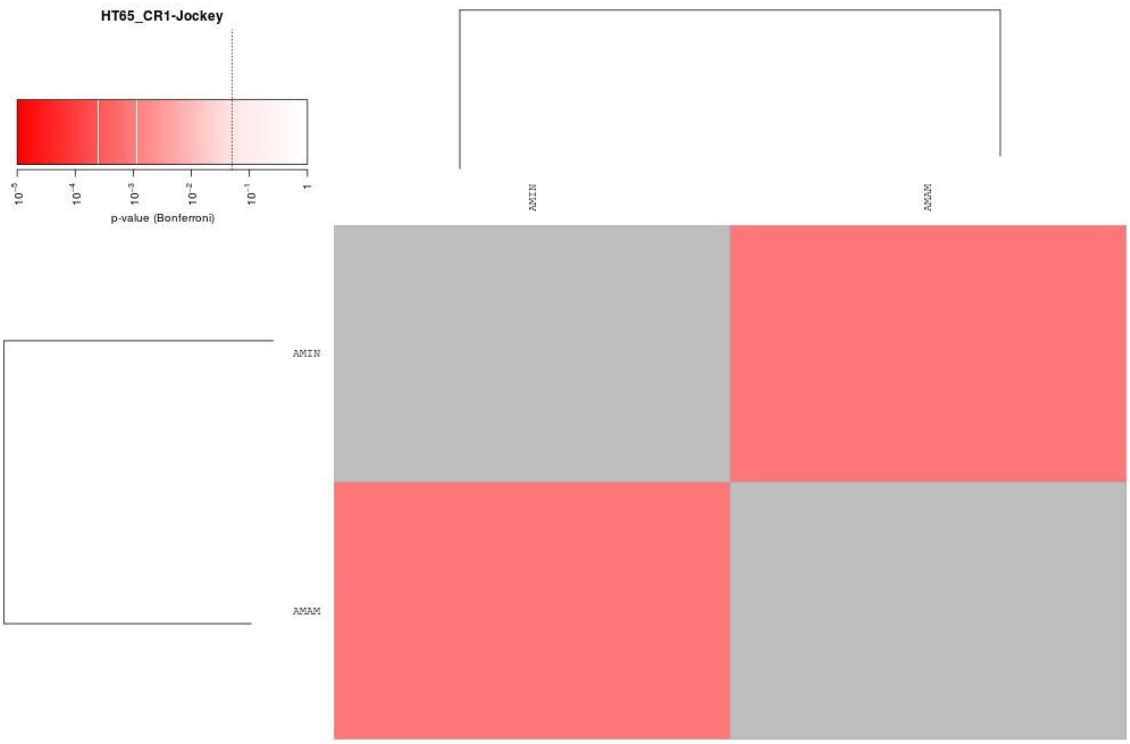
HT65_CR1-Jockey
$10^{-5}$ $10^{-4}$ $10^{-3}$ $10^{-2}$ $10^{-1}$ 1
p-value (Bonferroni)
AMIN
AMAM
AMIN
AMAM

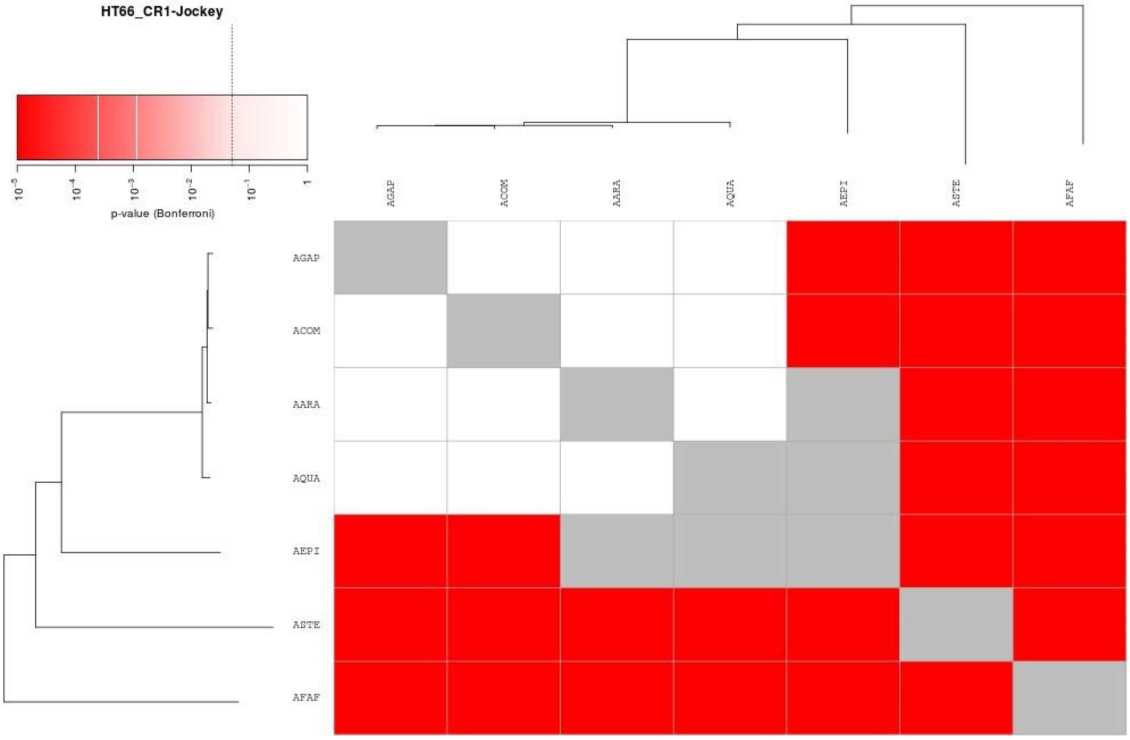
HT66_CR1-Jockey
p-value (Bonferroni)
AGAP
ACOM
AARA
AQUA
AEPI
ASTE
AFAF

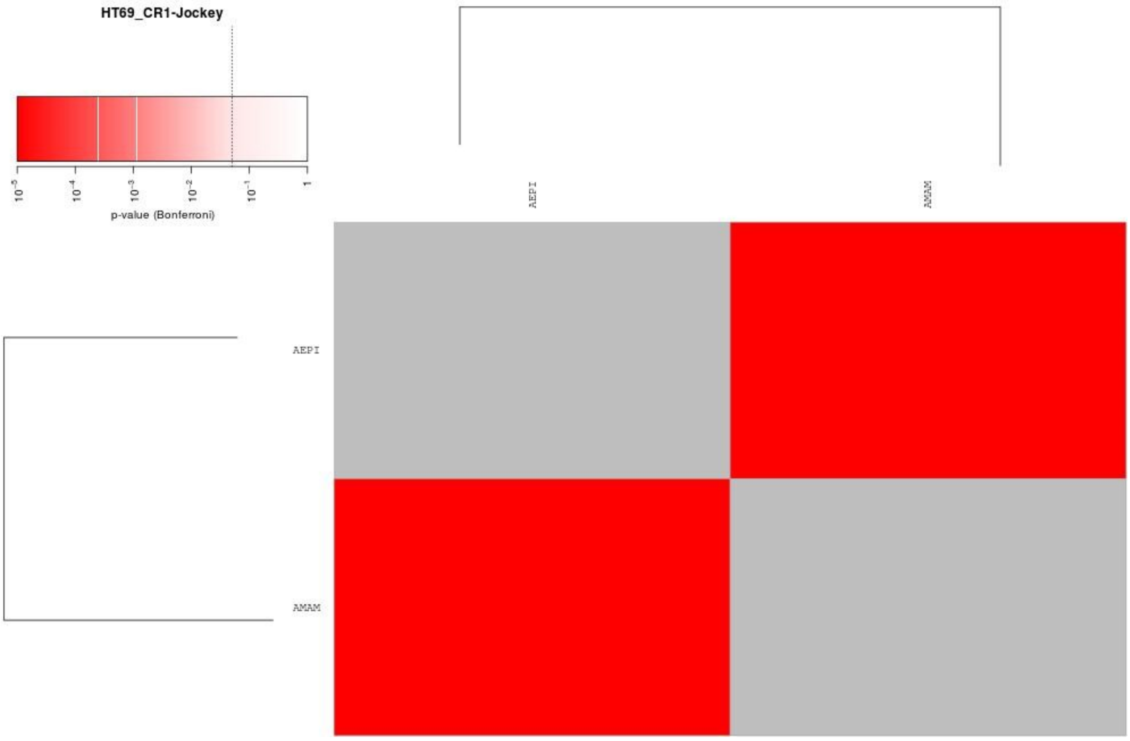
HT69_CR1-Jockey
p-value (Bonferroni)
AEPI
AMAM

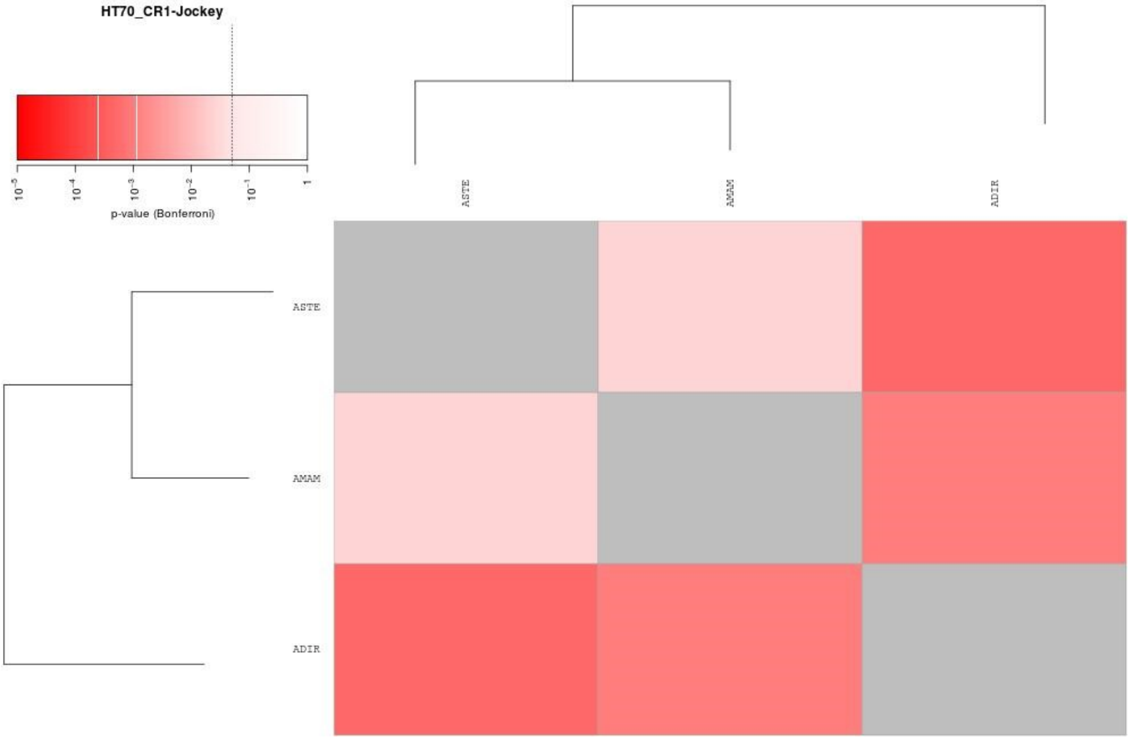
HT70_CR1-Jockey
p-value (Bonferroni)
ASTE
AMAM
ADIR
ASTE
AMAM
ADIR

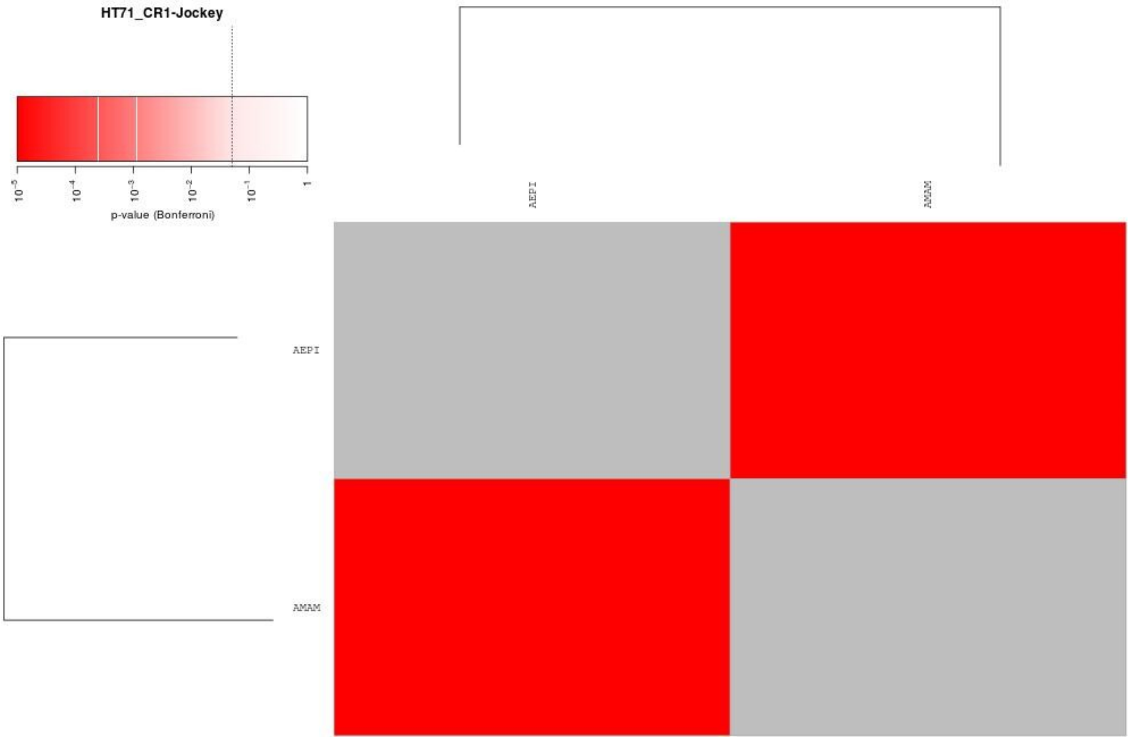
HT71_CR1-Jockey
p-value (Bonferroni)
AEPI
AMAM
AEPI
AMAM

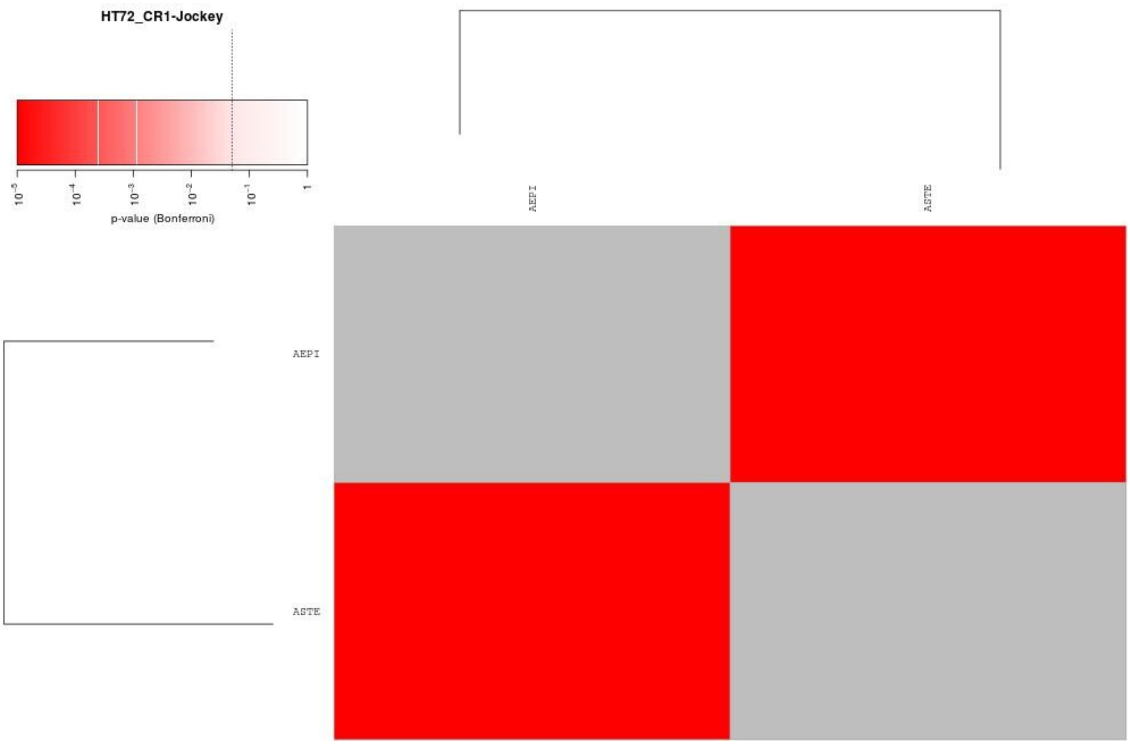
HT72_CR1-Jockey
p-value (Bonferroni)
AEPI
ASTE
AEPI
ASTE

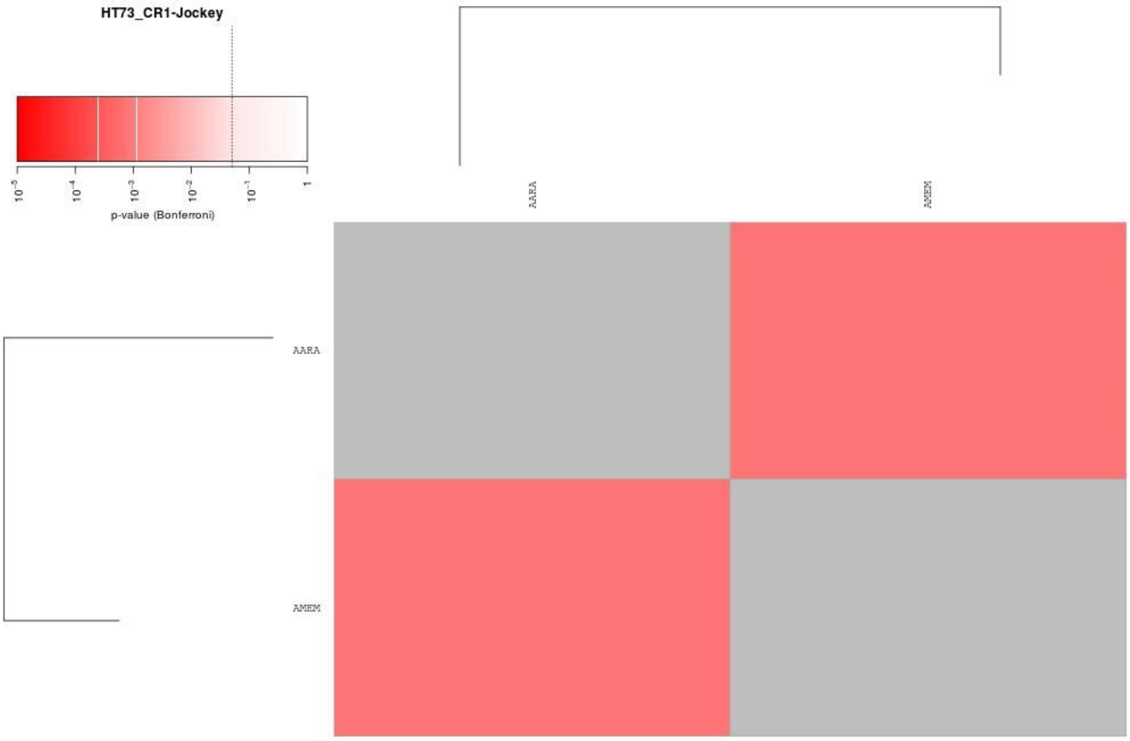
HT73_CR1-Jockey
p-value (Bonferroni)
AARA
AMEM
AARA
AMEM

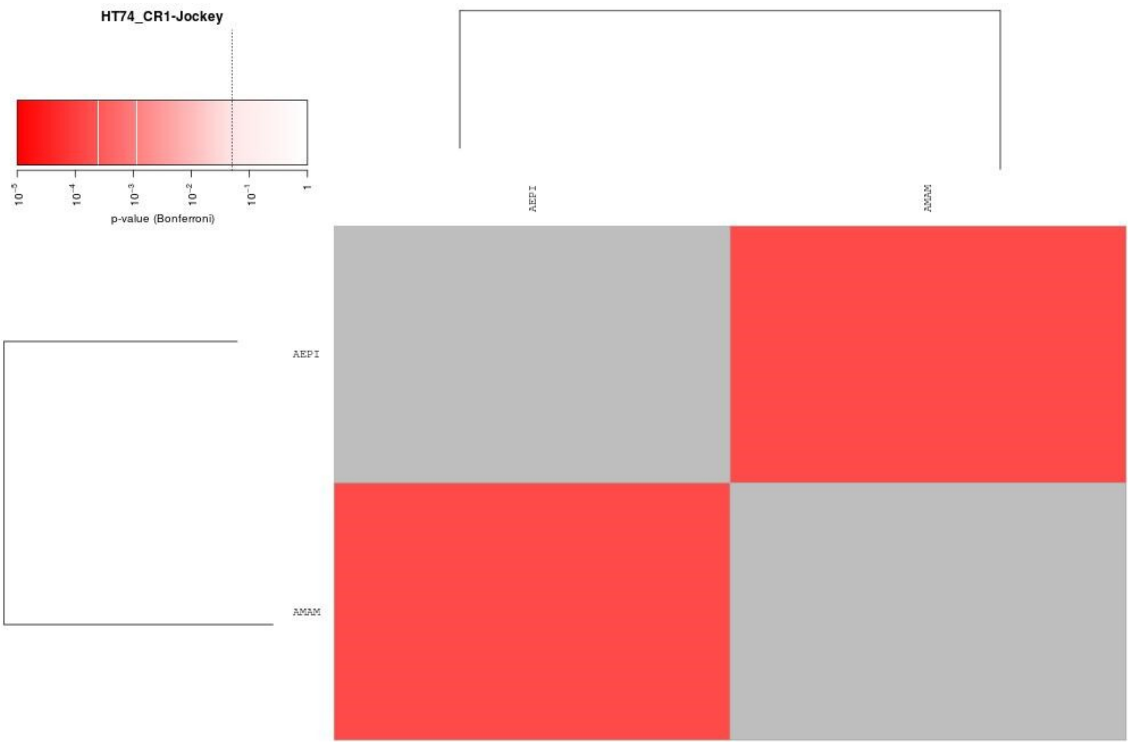
HT74_CR1-Jockey
p-value (Bonferroni)
AEPI
AMAM
AEPI
AMAM

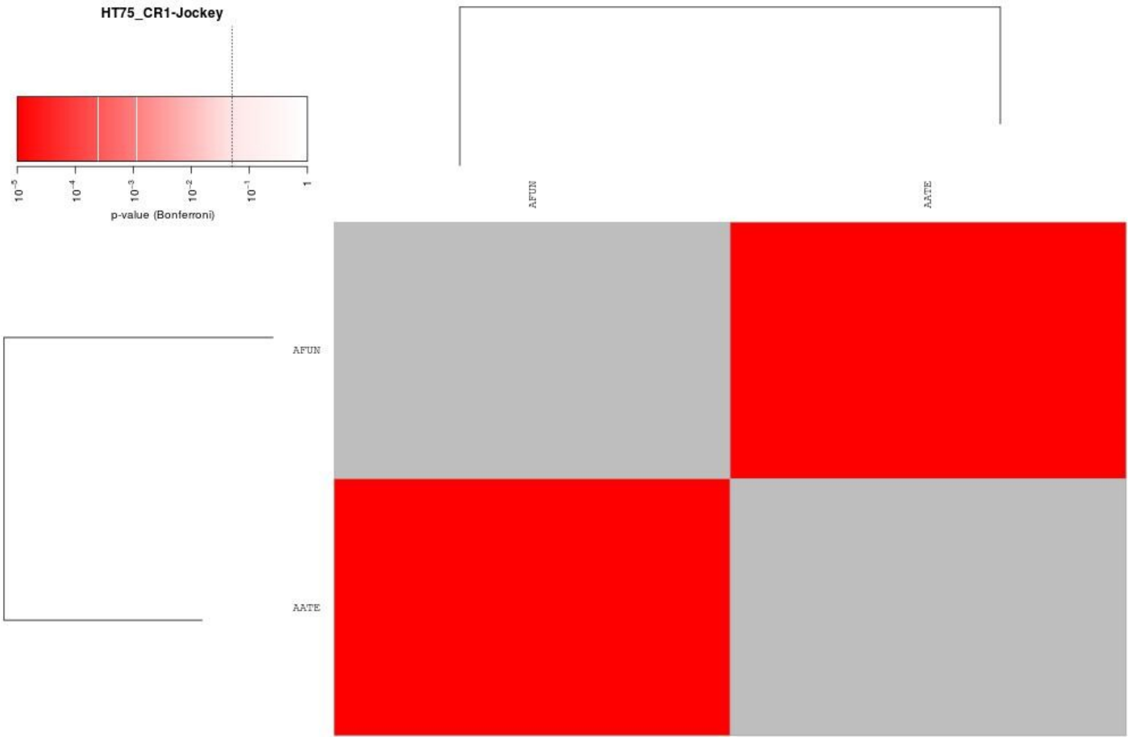
HT75_CR1-Jockey
p-value (Bonferroni)
AFUN
AATE
AFUN
AATE

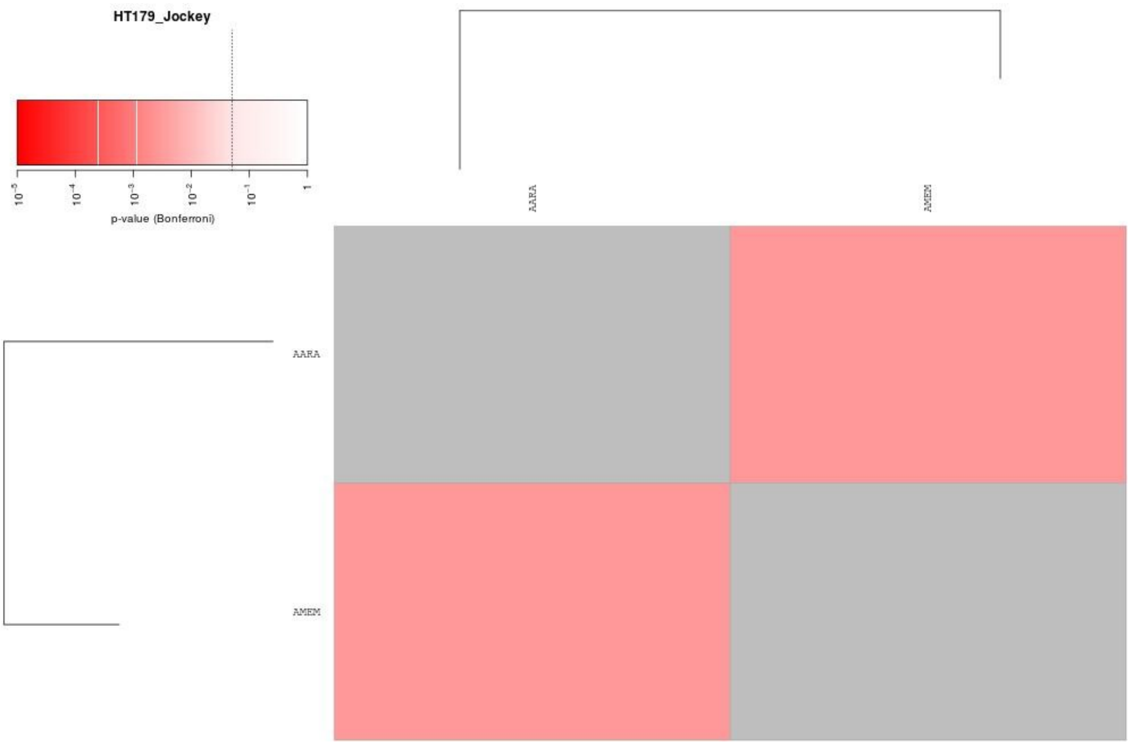
HT179_Jockey
$10^{-5}$
$10^{-4}$
$10^{-3}$
$10^{-2}$
$10^{-1}$
1
p-value (Bonferroni)
AARA
AMEM
AARA
AMEM

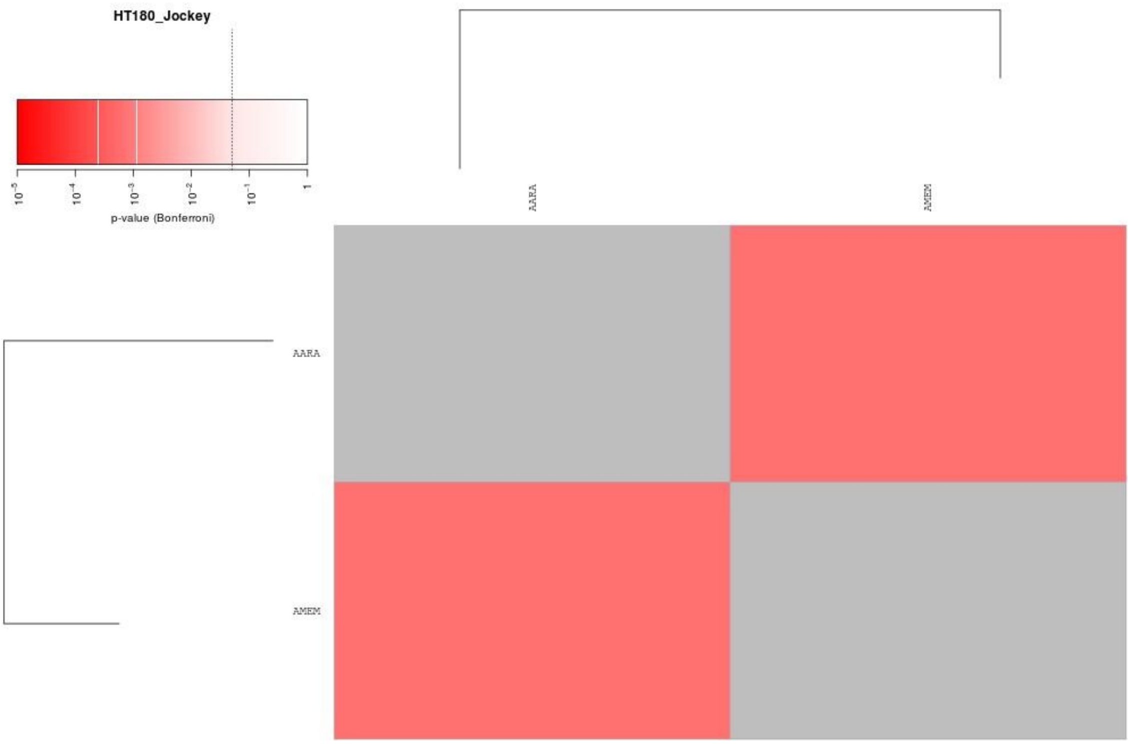
HT180_Jockey
$10^{-5}$
$10^{-4}$
$10^{-3}$
$10^{-2}$
$10^{-1}$
1
p-value (Bonferroni)
AARA
AMEM
AARA
AMEM

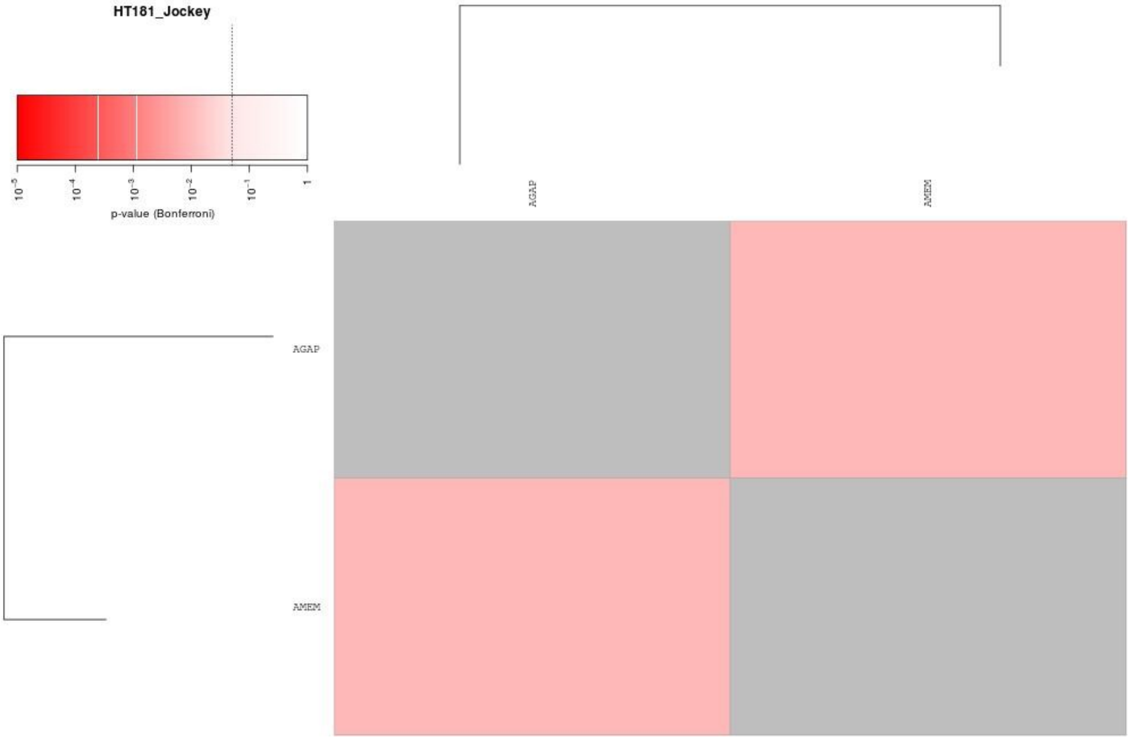
HT181_Jockey
p-value (Bonferroni)
AGAP
AMEM

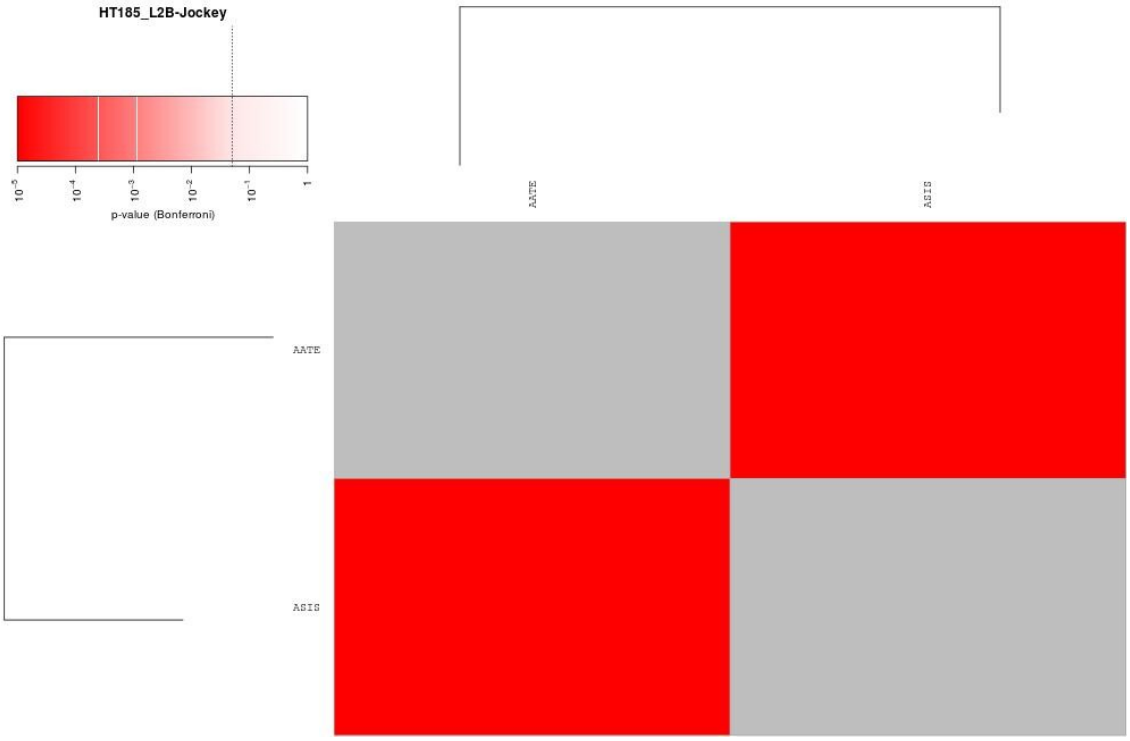
HT185_L2B-Jockey
p-value (Bonferroni)
AATE
ASIS

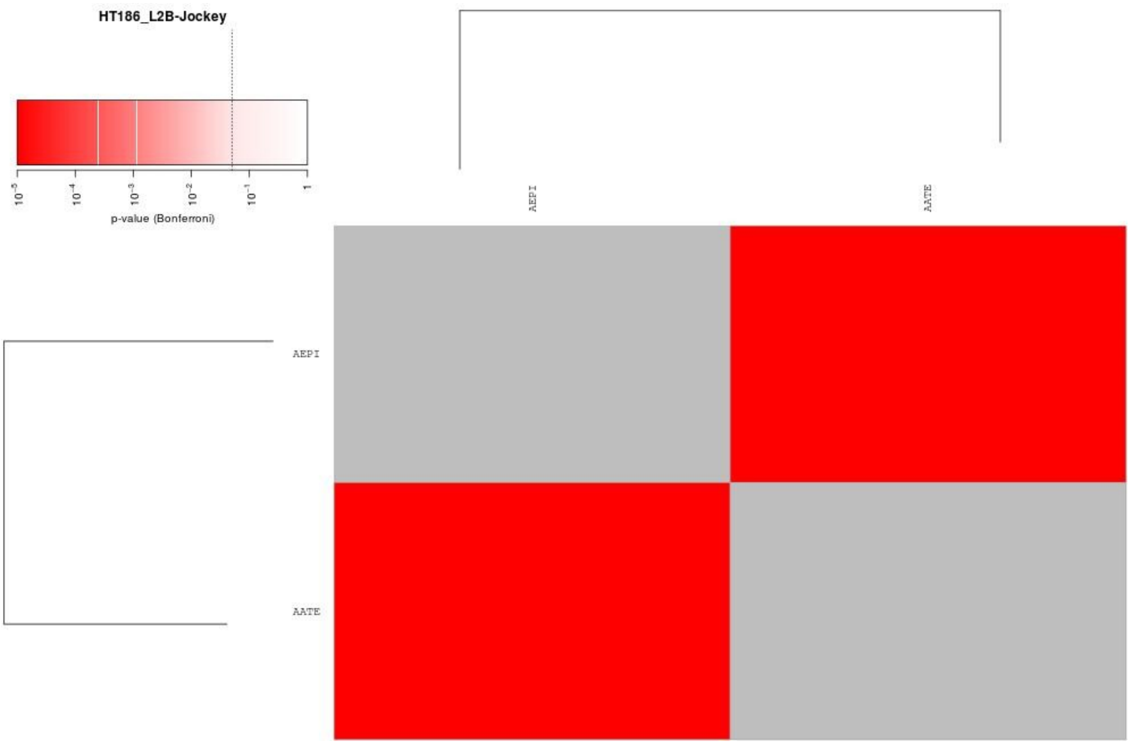
HT186_L2B-Jockey
p-value (Bonferroni)
AEPI
AATE
AEPI
AATE

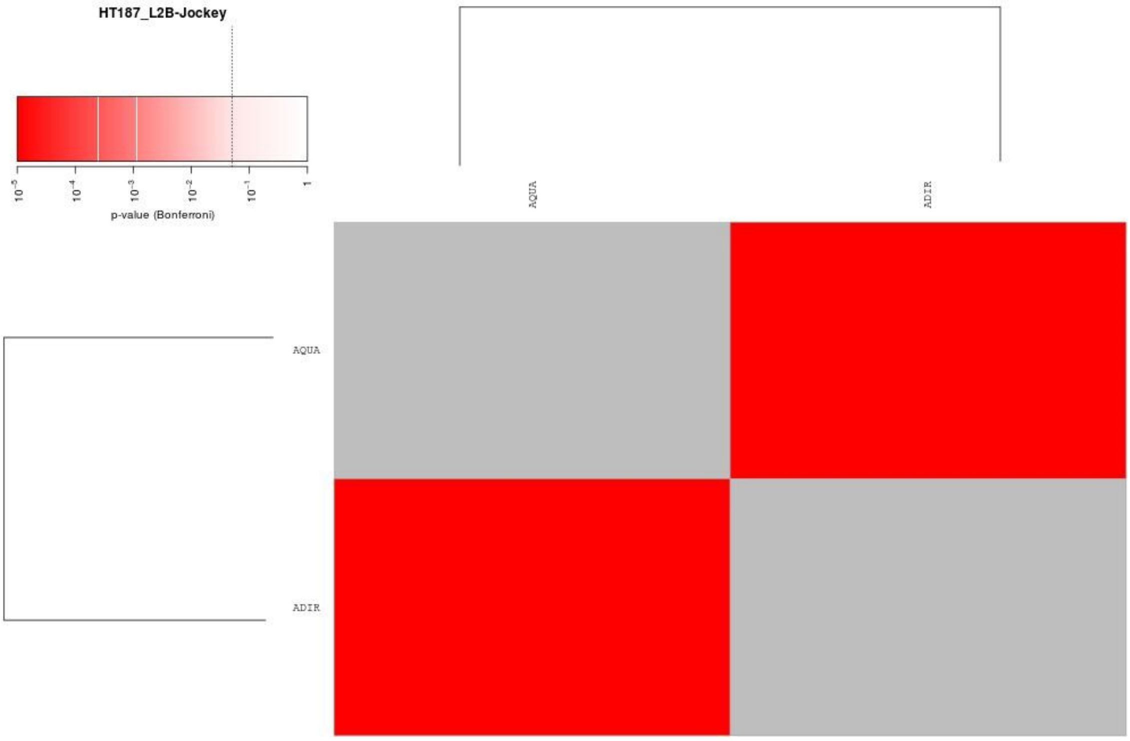
HT187_L2B-Jockey
p-value (Bonferroni)
AQUA
ADIR
AQUA
ADIR

## L1

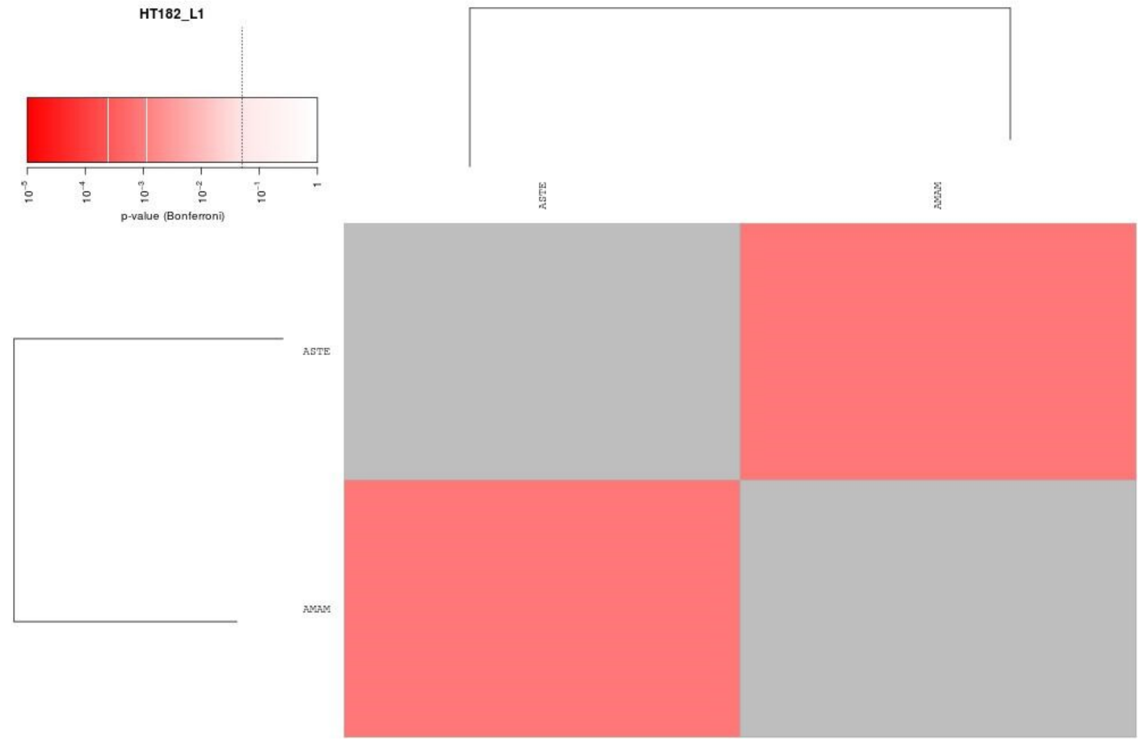
HT182_L1
p-value (Bonferroni)
ASTE
AMAM
ASTE
AMAM

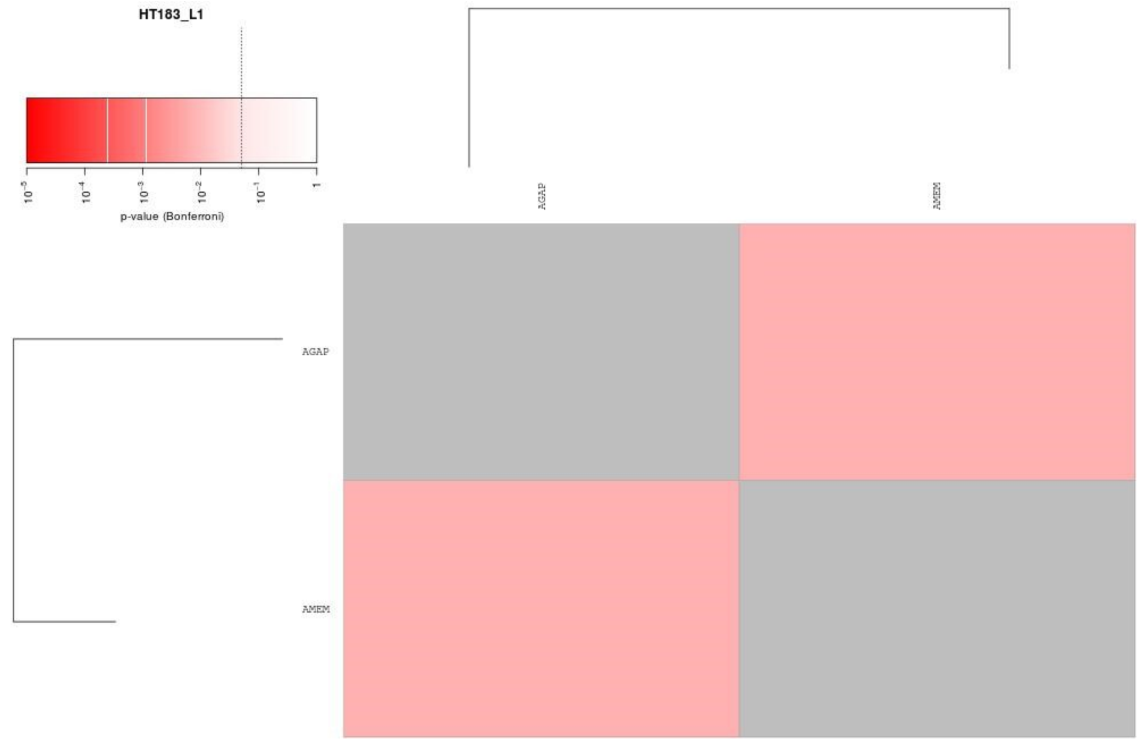
HT183_L1
p-value (Bonferroni)
AGAP
AMEM
AGAP
AMEM

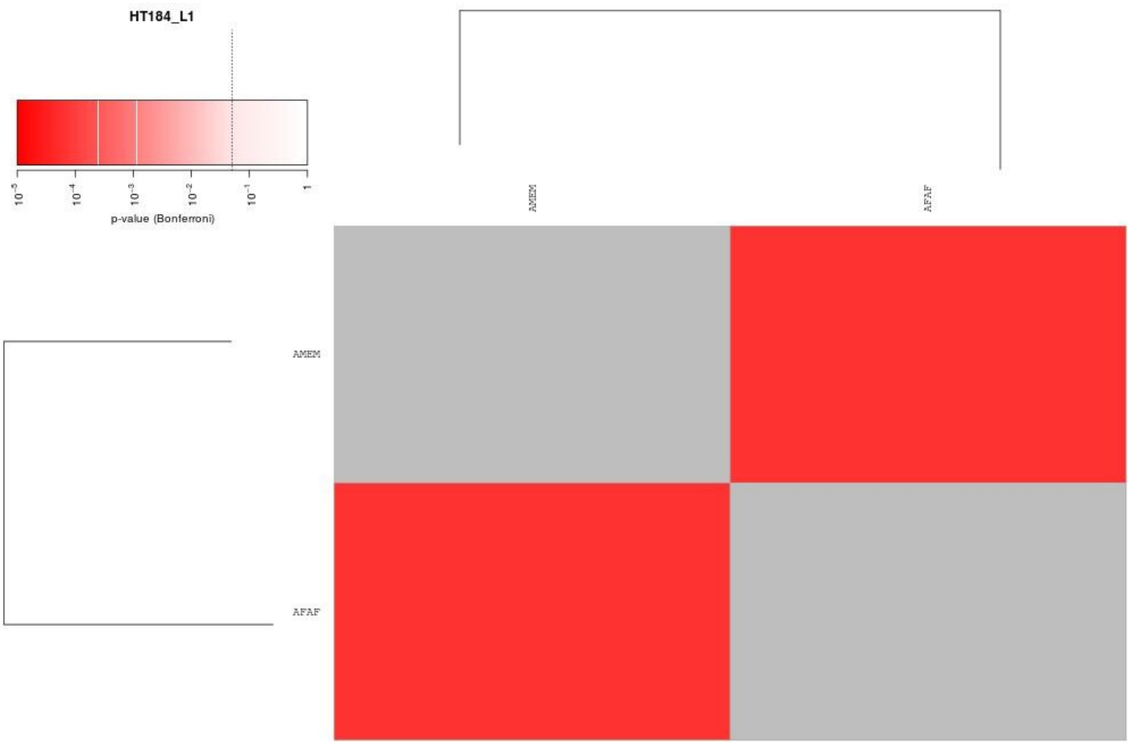
HT184_L1
p-value (Bonferroni)
AMEM
AFAF

# R2

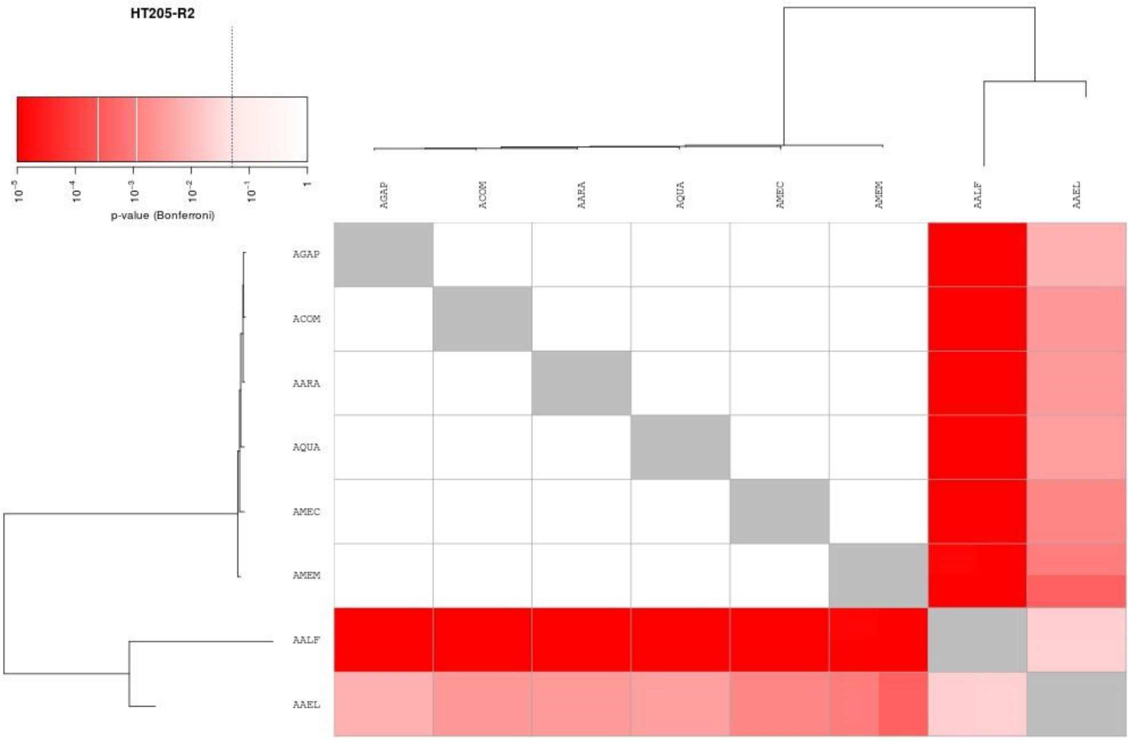
HT205-R2
p-value (Bonferroni)
AGAP
ACOM
AARA
AQUA
AMEC
AMEM
AALF
AAEL

# RTE

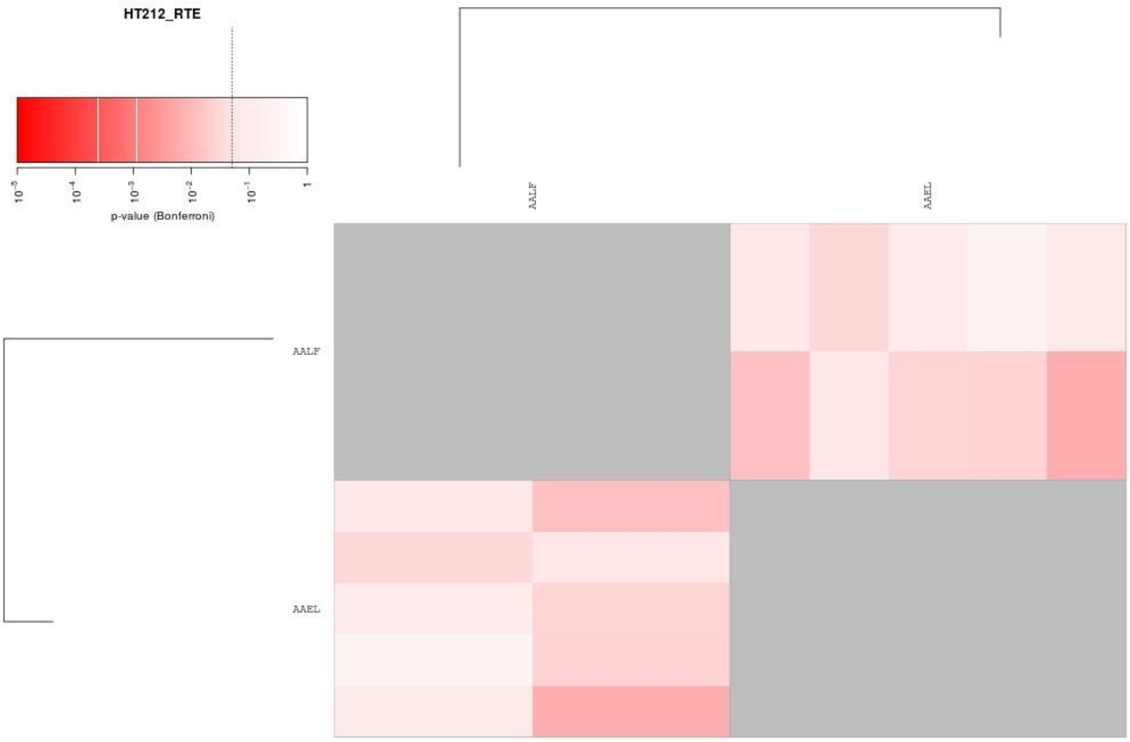
HT212_RTE
p-value (Bonferroni)
AALF
AAEL
AALF
AAEL

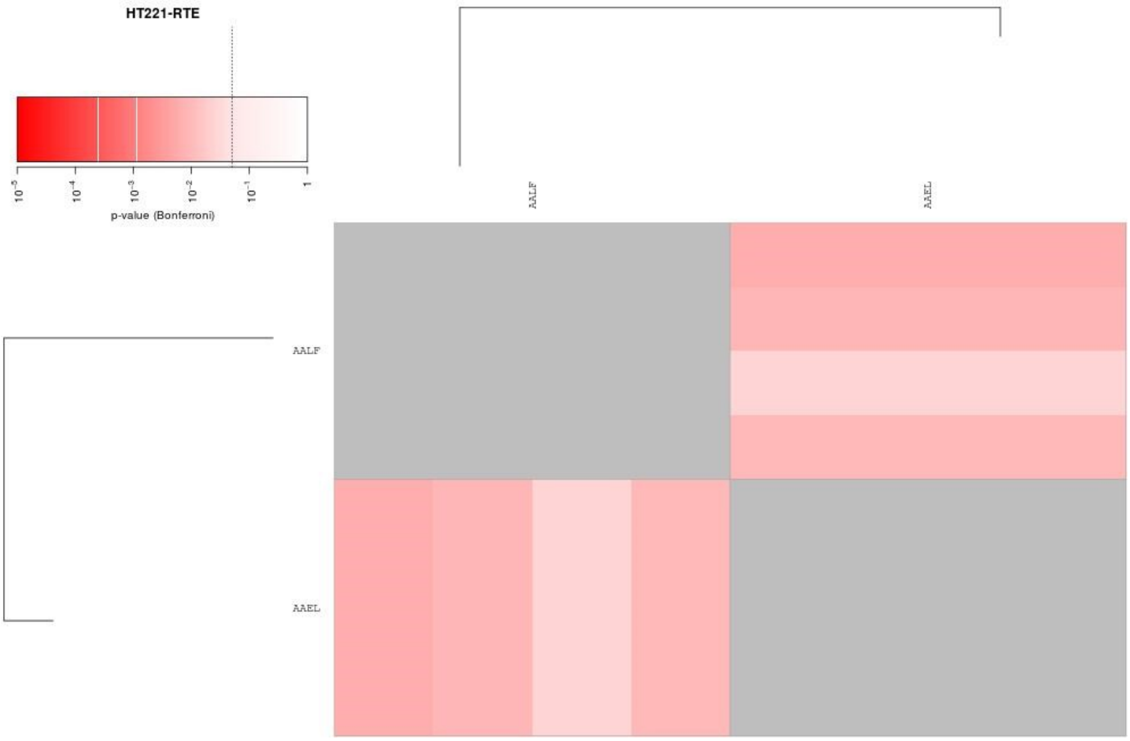
HT221-RTE
p-value (Bonferroni)
AALF
AAEL
AALF
AAEL

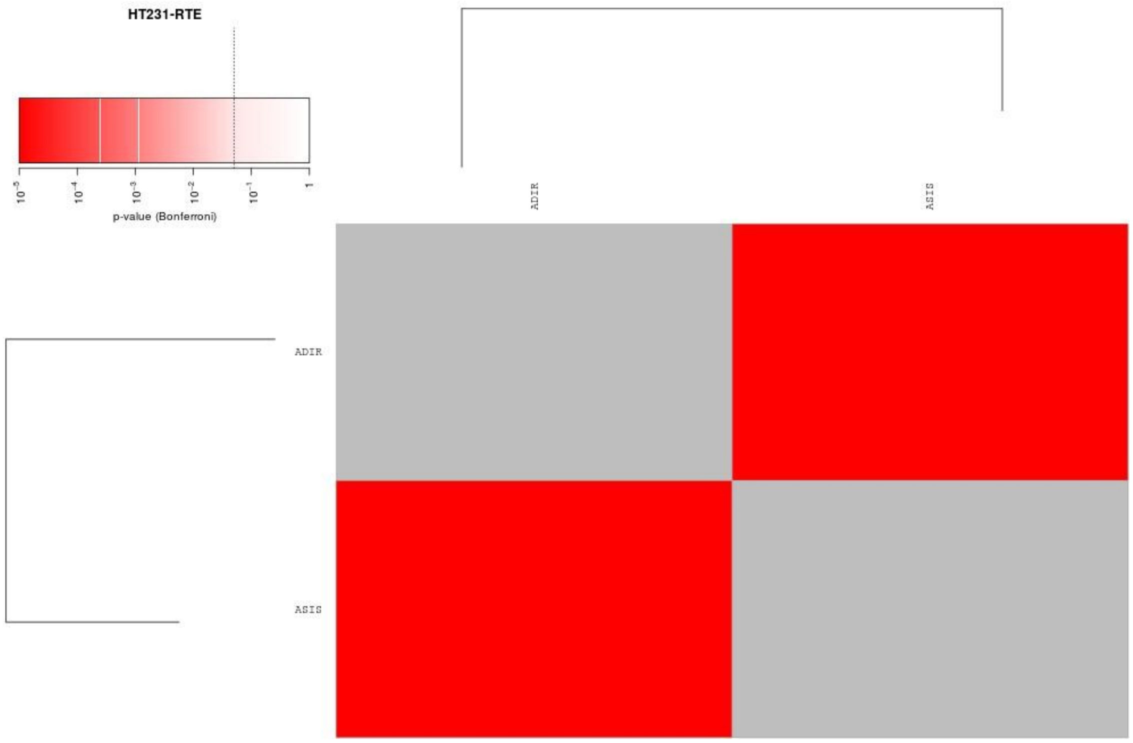
HT231-RTE
p-value (Bonferroni)
ADIR
ASIS
ADIR
ASIS

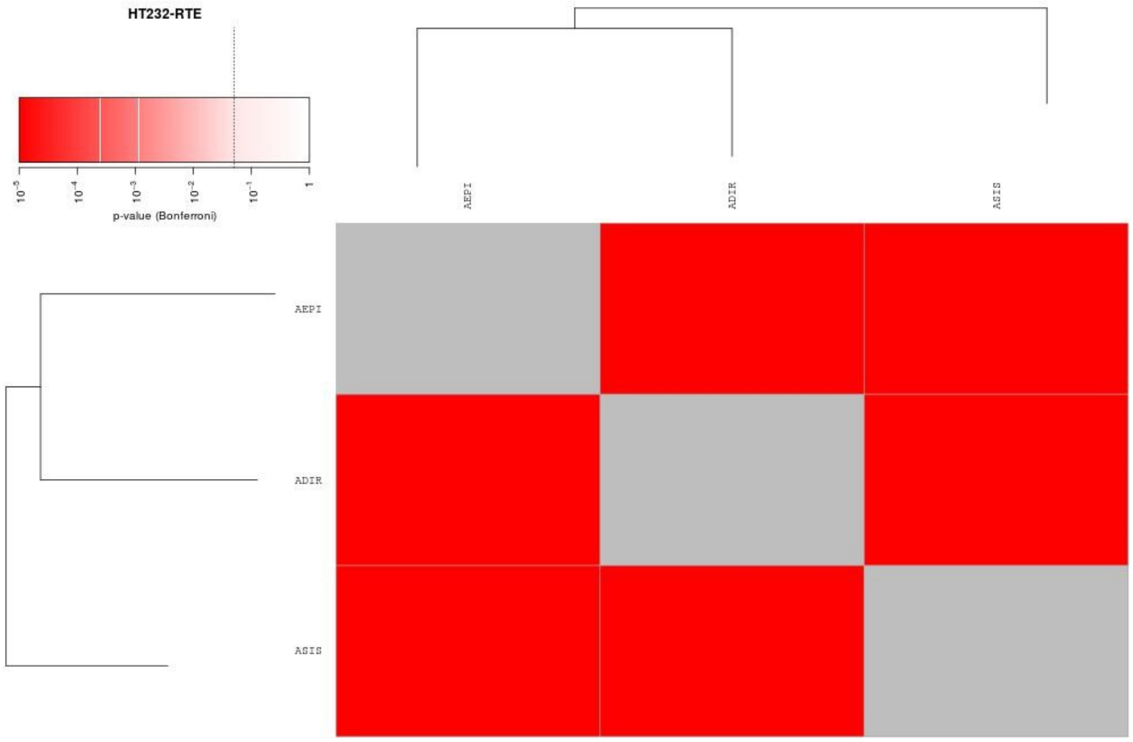
HT232-RTE
p-value (Bonferroni)
AEPI
ADIR
ASIS
AEPI
ADIR
ASIS

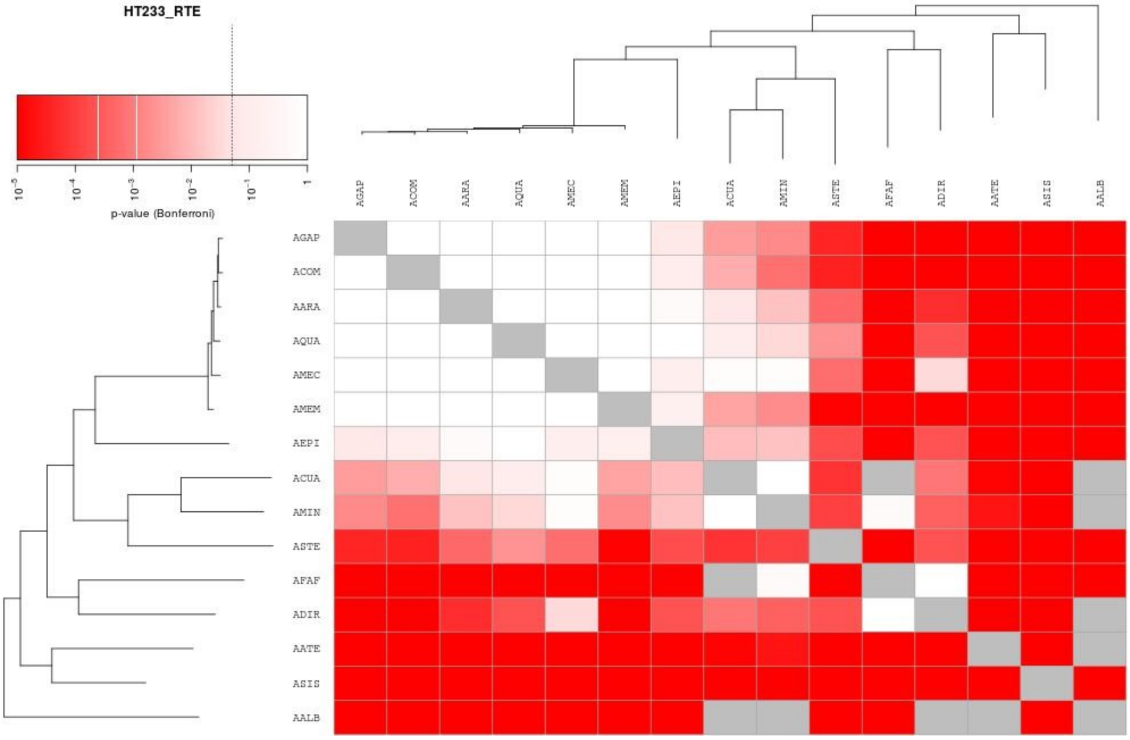
HT233_RTE
p-value (Bonferroni)
AGAP
ACOM
AARA
AQUA
AMEC
AMEM
AEPI
ACUA
AMIN
ASTE
AFAF
ADIR
AATE
ASIS
AALB

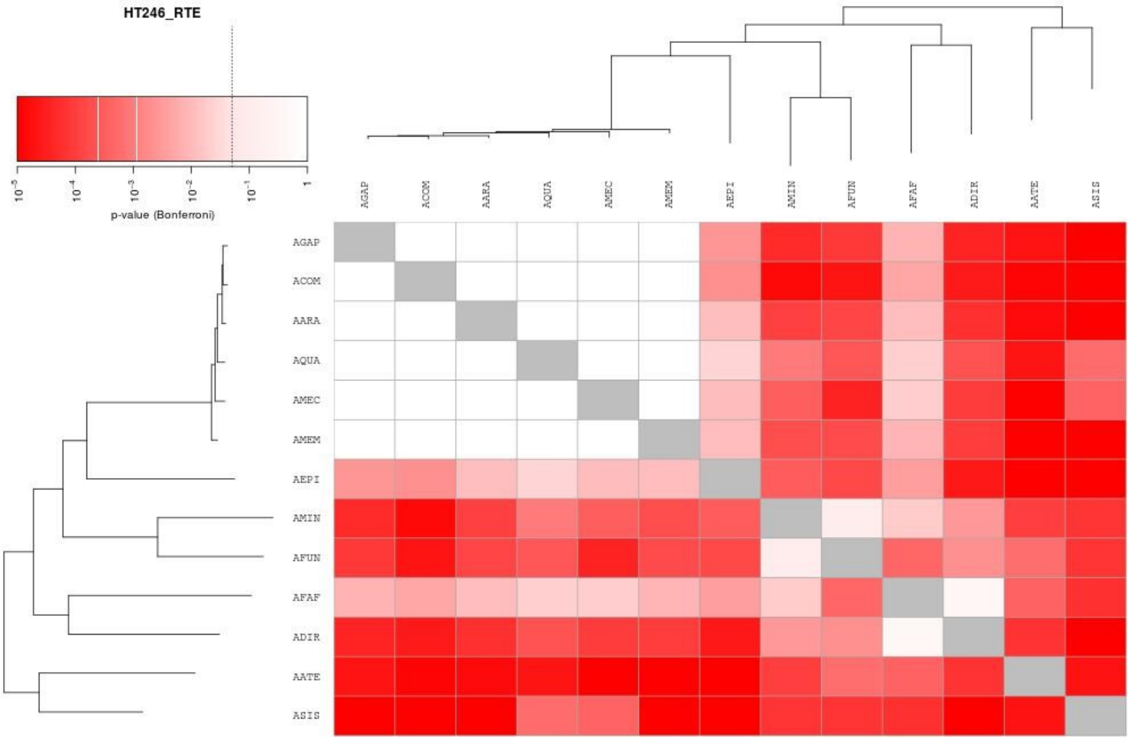
HT246_RTE
p-value (Bonferroni)
AGAP
ACOM
AARA
AQUA
AMEC
AMEM
AEPI
AMIN
AFUN
AFAF
ADIR
AATE
ASIS

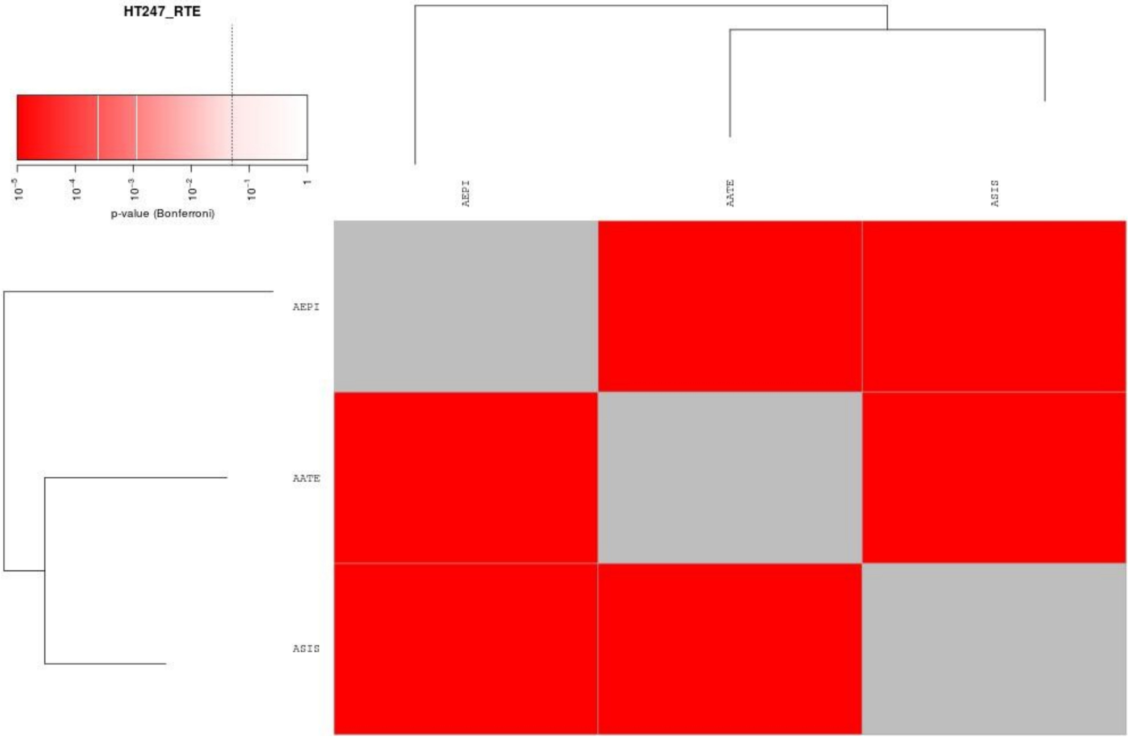
HT247_RTE
$10^{-5}$ $10^{-4}$ $10^{-3}$ $10^{-2}$ $10^{-1}$ 1
p-value (Bonferroni)
AEPI
AATE
ASIS
AEPI
AATE
ASIS

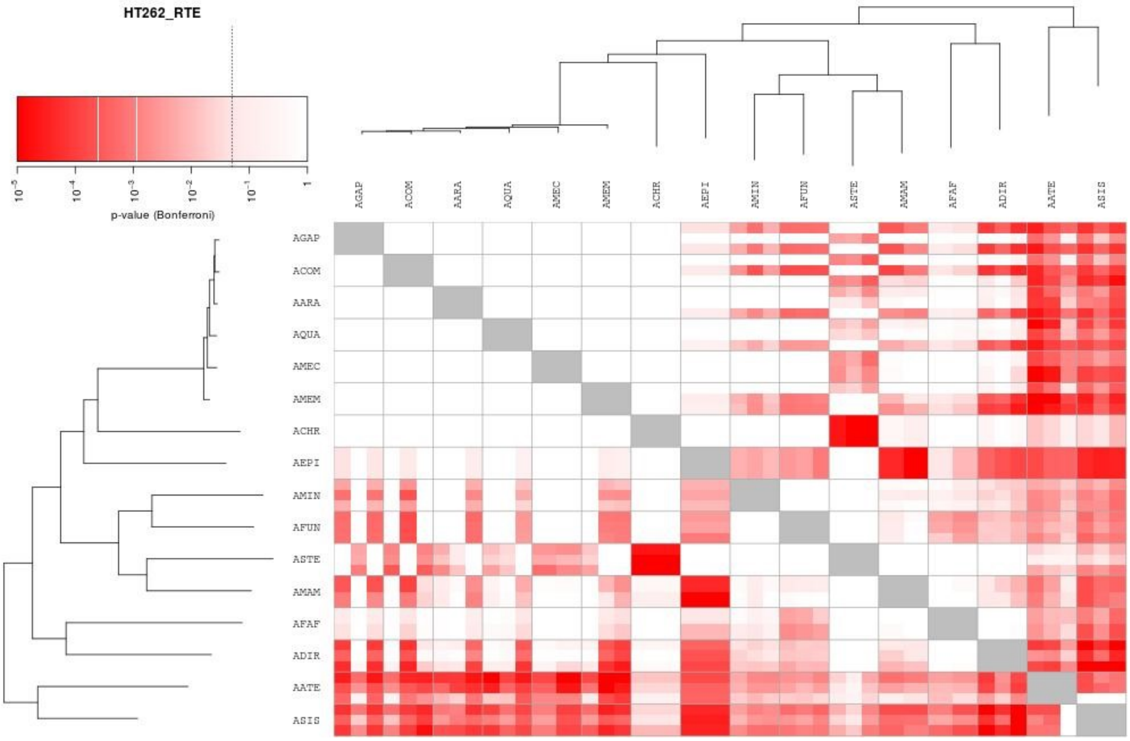
HT262_RTE
$10^{-5}$ $10^{-4}$ $10^{-3}$ $10^{-2}$ $10^{-1}$ 1
p-value (Bonferroni)
AGAP ACOM AARA AQUA AMEC AMEM ACHR AEPI AMIN AFUN ASTE AMAM AFAF ADIR AATE ASIS
AGAP
ACOM
AARA
AQUA
AMEC
AMEM
ACHR
AEPI
AMIN
AFUN
ASTE
AMAM
AFAF
ADIR
AATE
ASIS

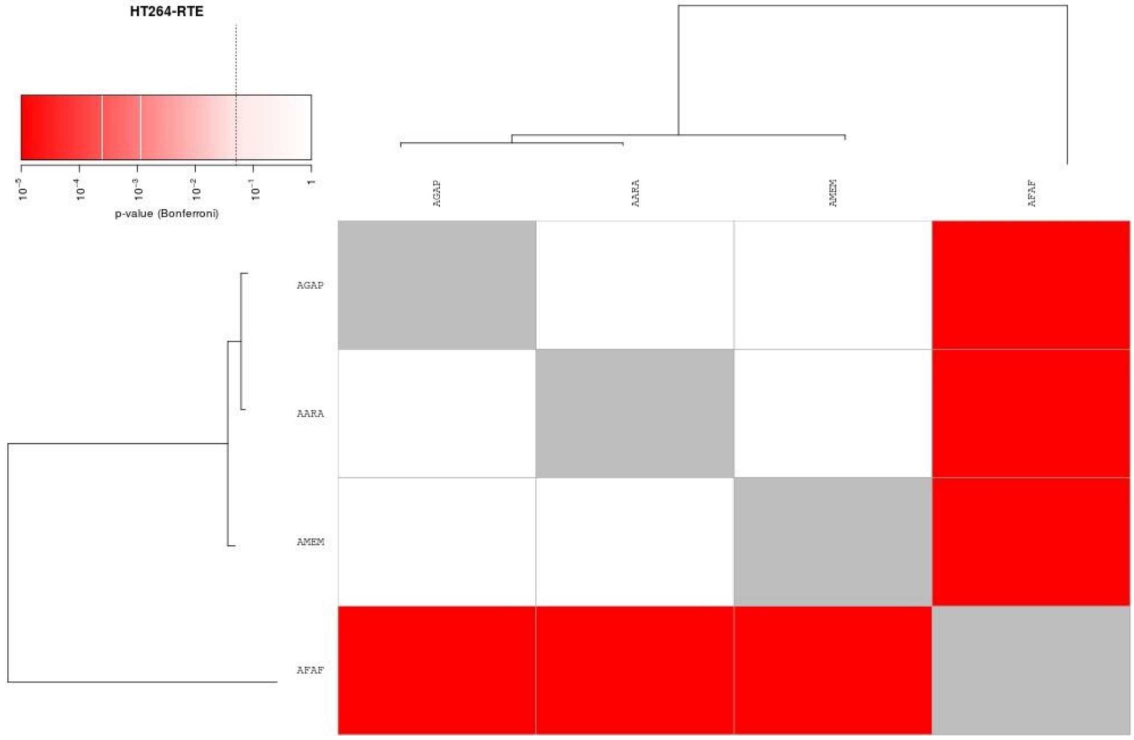
HT264-RTE
p-value (Bonferroni)
AGAP
AARA
AMEM
AFAF

## CACTA

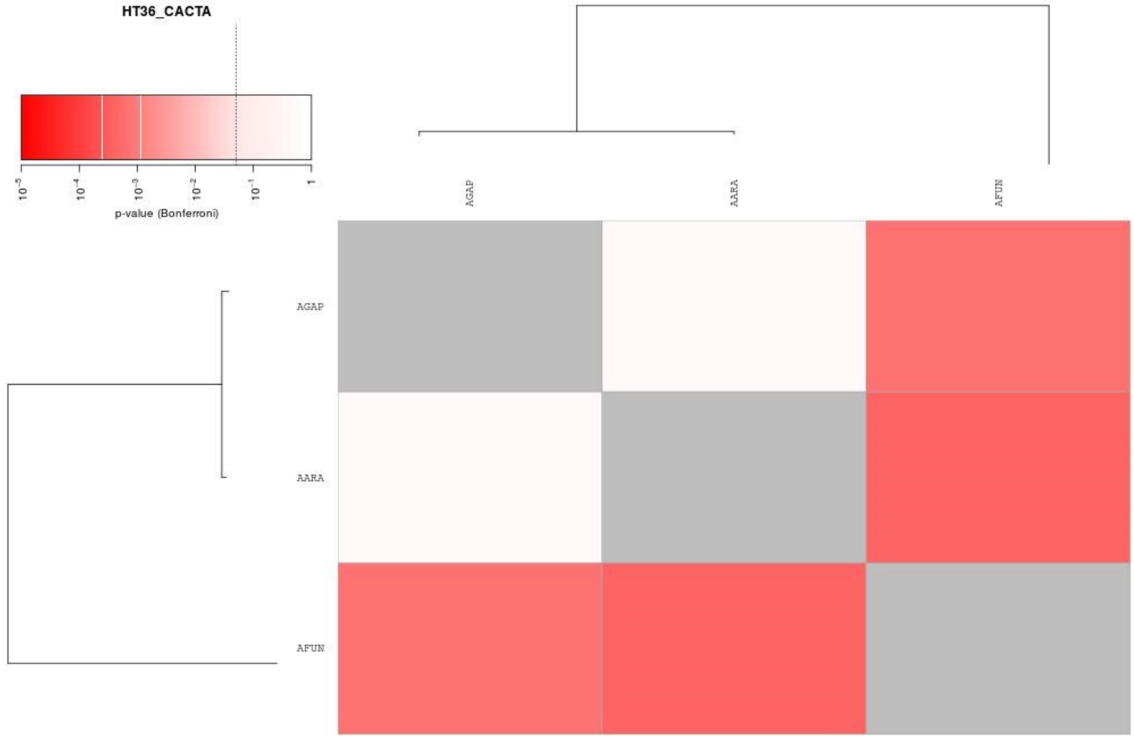
HT36_CACTA
p-value (Bonferroni)
AGAP
AARA
AFUN

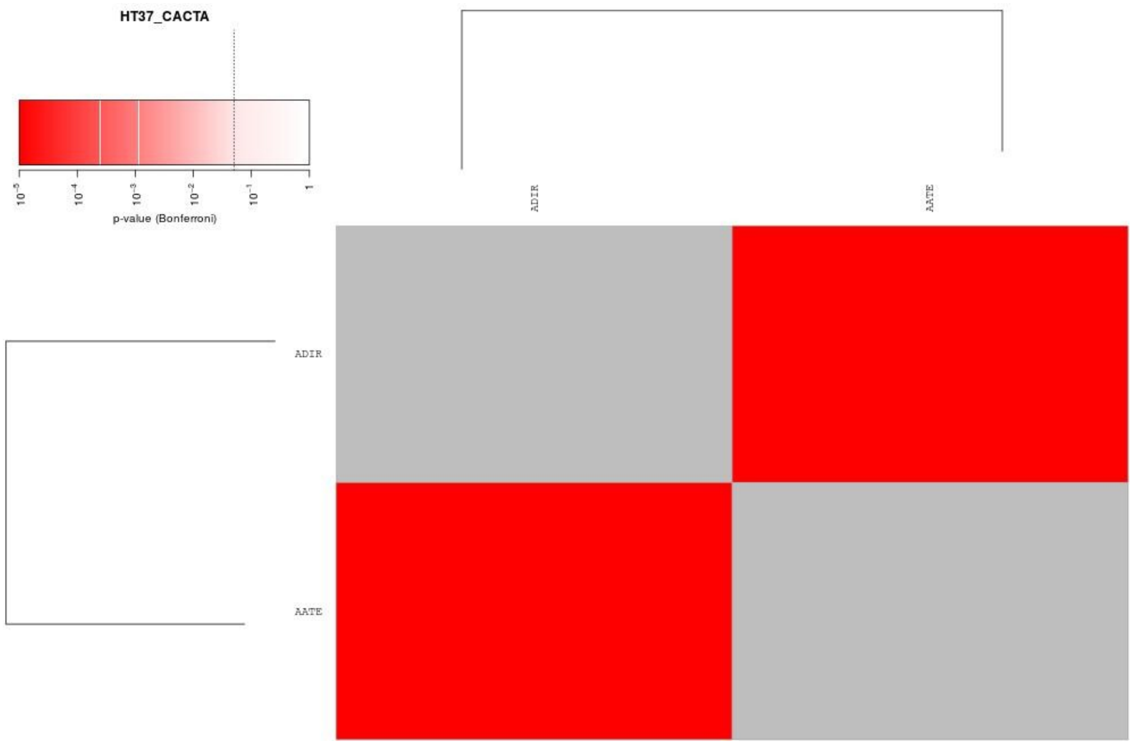
HT37_CACTA
p-value (Bonferroni)
ADIR
AATE

## PHIS

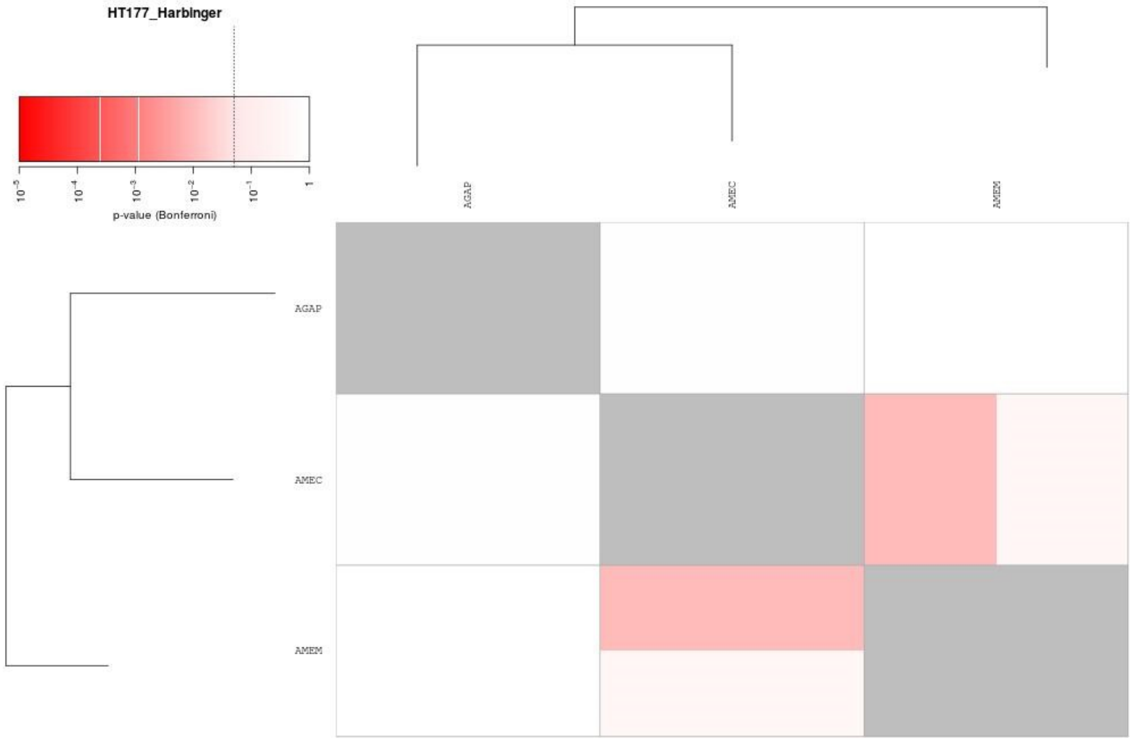
HT177_Harbinger
p-value (Bonferroni)
AGAP
AMEC
AMEM

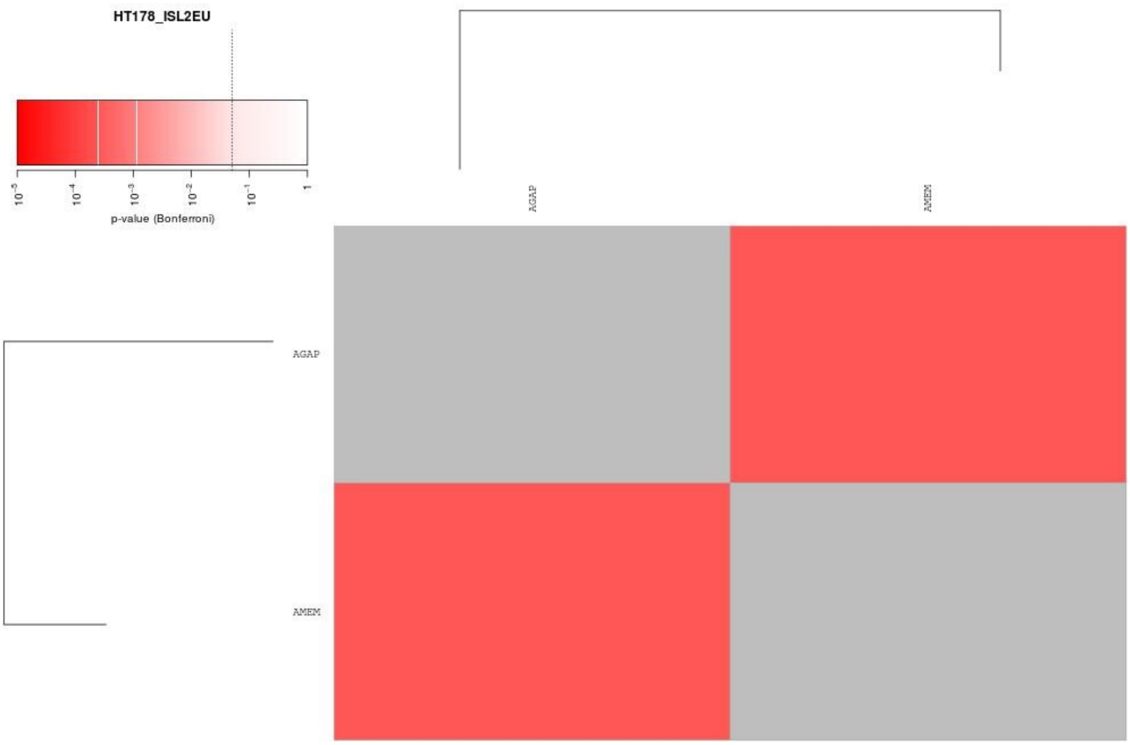

HT178_ISL2EU
p-value (Bonferroni)
AGAP
AMEM
AGAP
AMEM


## hAT

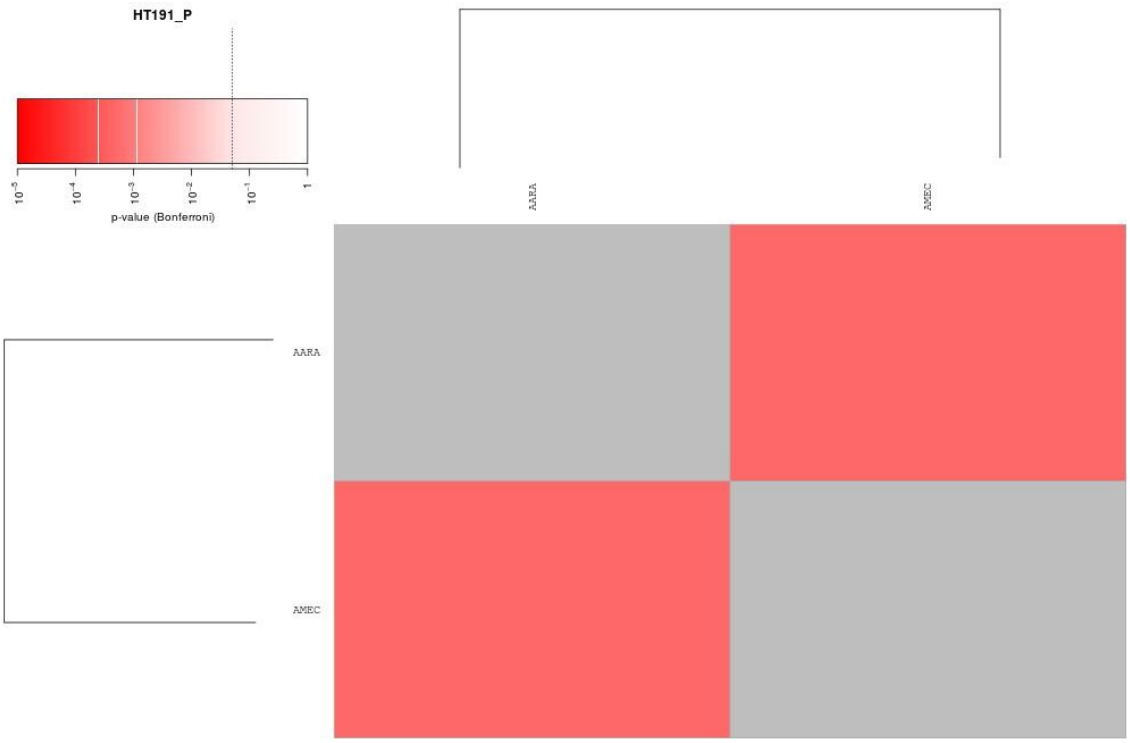

HT191_P
p-value (Bonferroni)
AARA
AMEC
AARA
AMEC

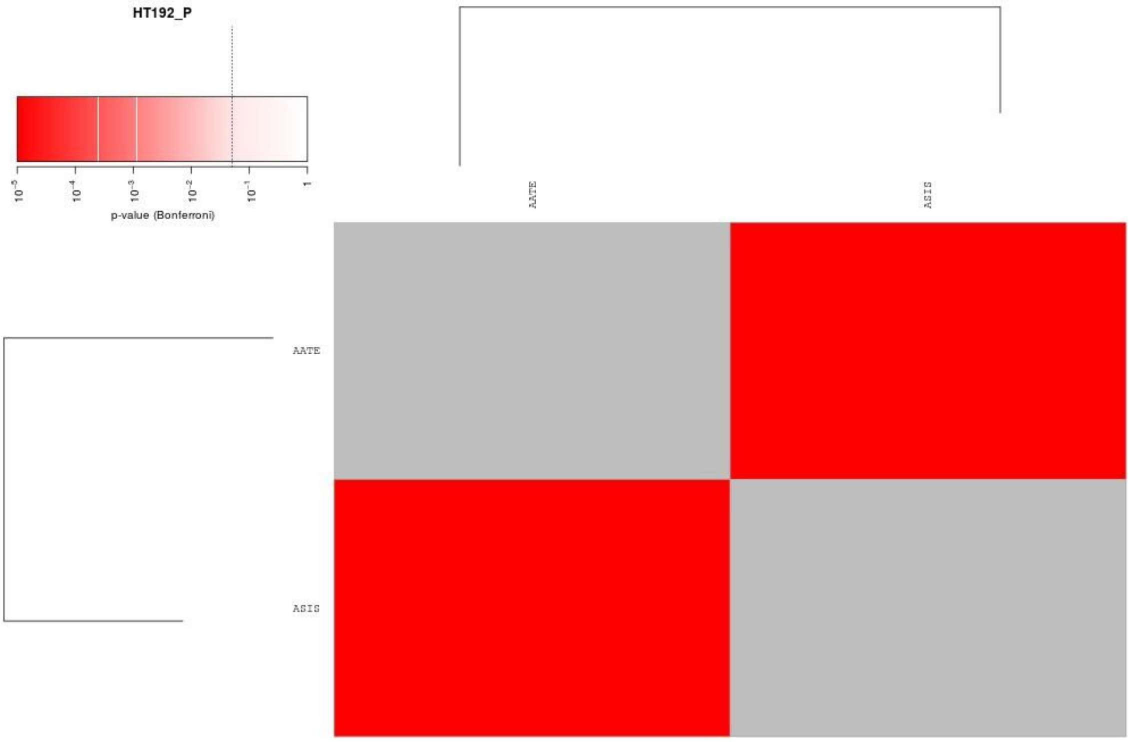
HT192_P
$10^{-5}$ $10^{-4}$ $10^{-3}$ $10^{-2}$ $10^{-1}$ 1
p-value (Bonferroni)
AATE
ASIS
AATE
ASIS

P

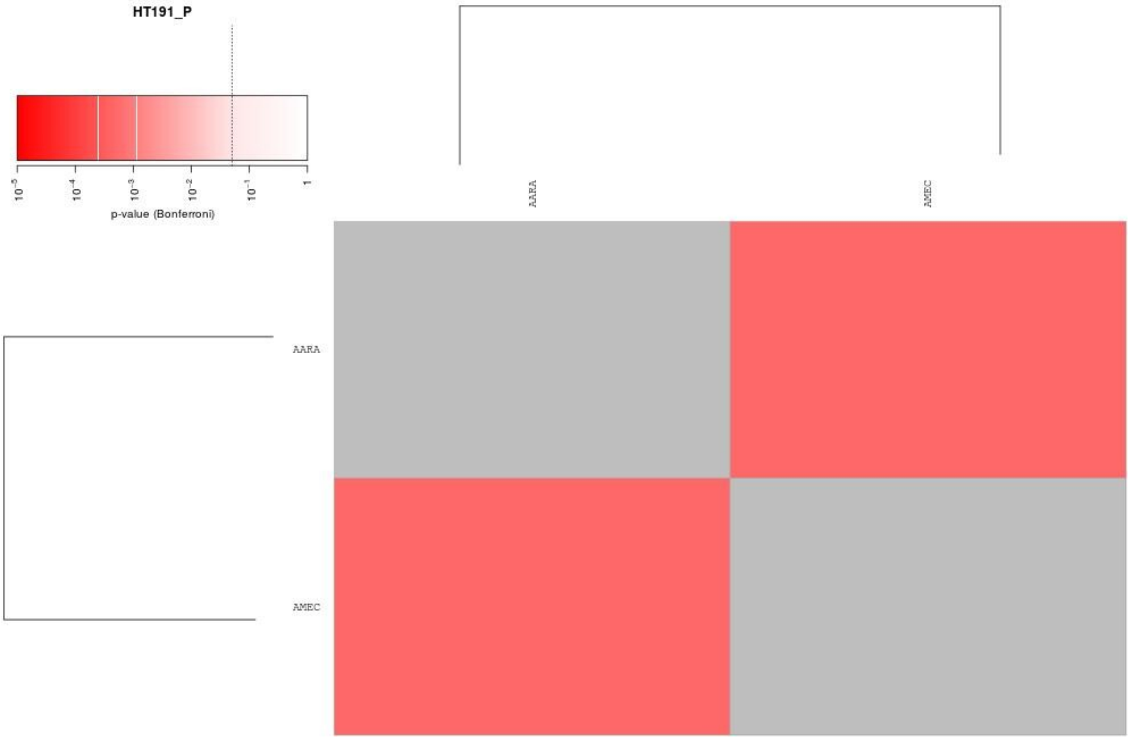
HT191_P
$10^{-5}$ $10^{-4}$ $10^{-3}$ $10^{-2}$ $10^{-1}$ 1
p-value (Bonferroni)
AARA
AMEC
AARA
AMEC

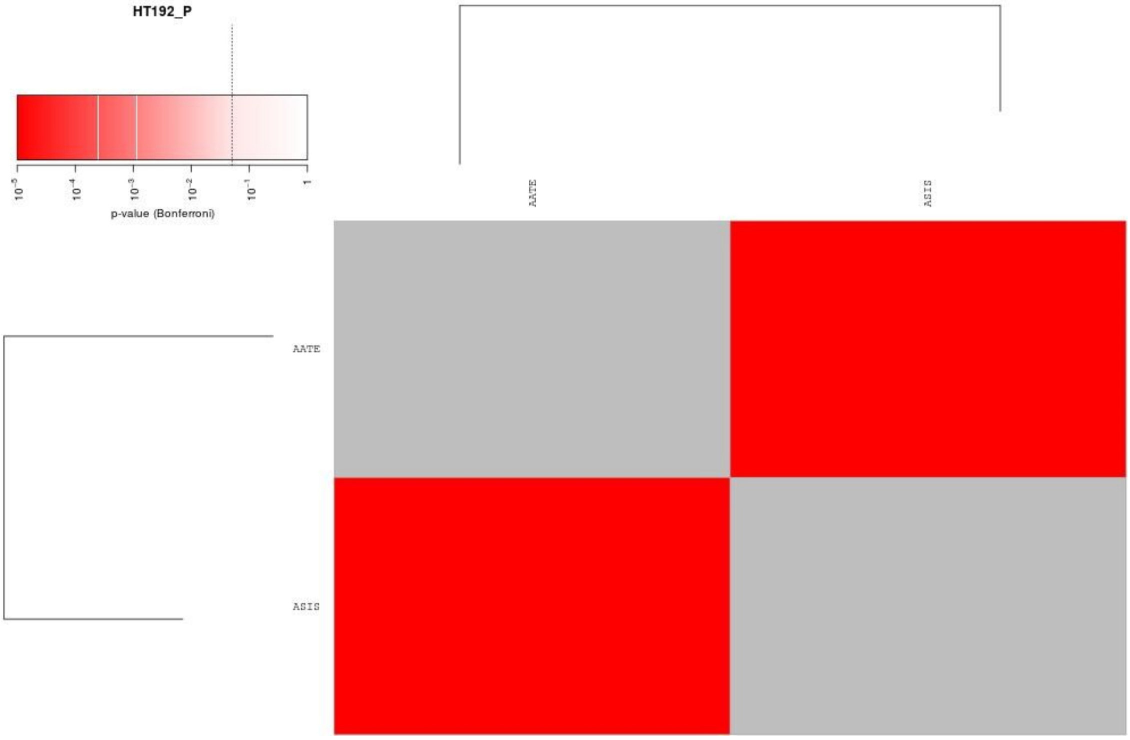
HT192_P
p-value (Bonferroni)
AATE
ASIS
AATE
ASIS

## PiggyBac

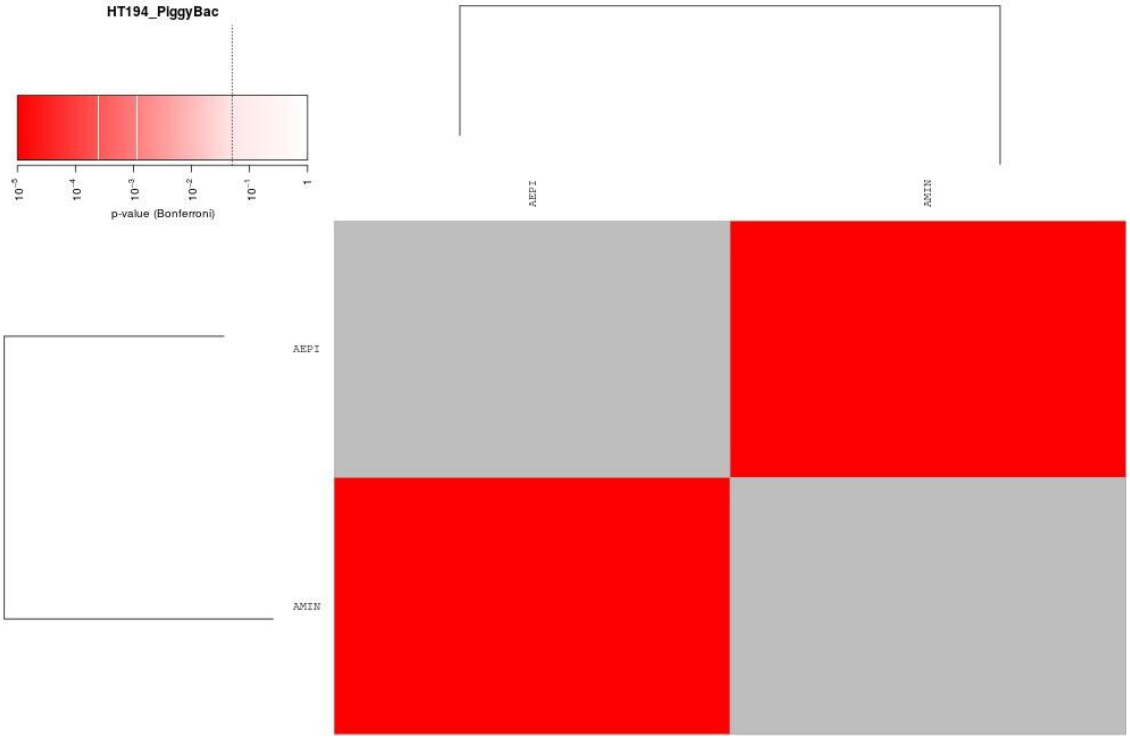
HT194_PiggyBac
p-value (Bonferroni)
AEPI
AMIN
AEPI
AMIN

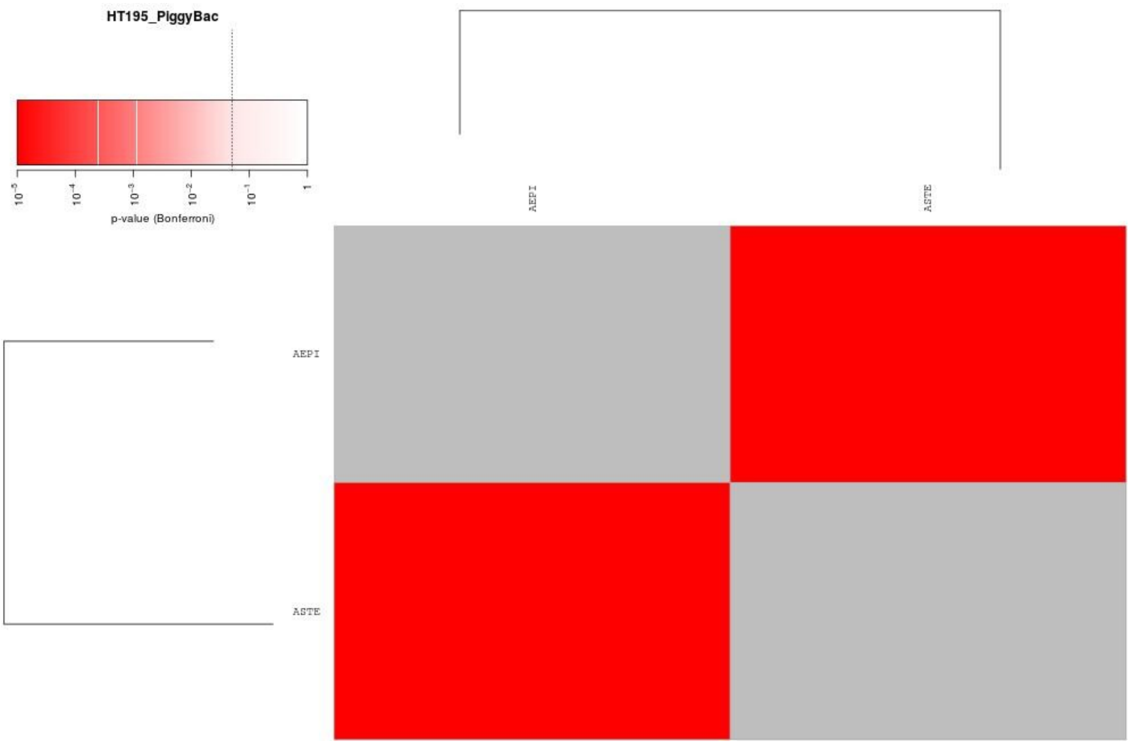
HT195_PiggyBac
p-value (Bonferroni)
AEPI
ASTE
AEPI
ASTE

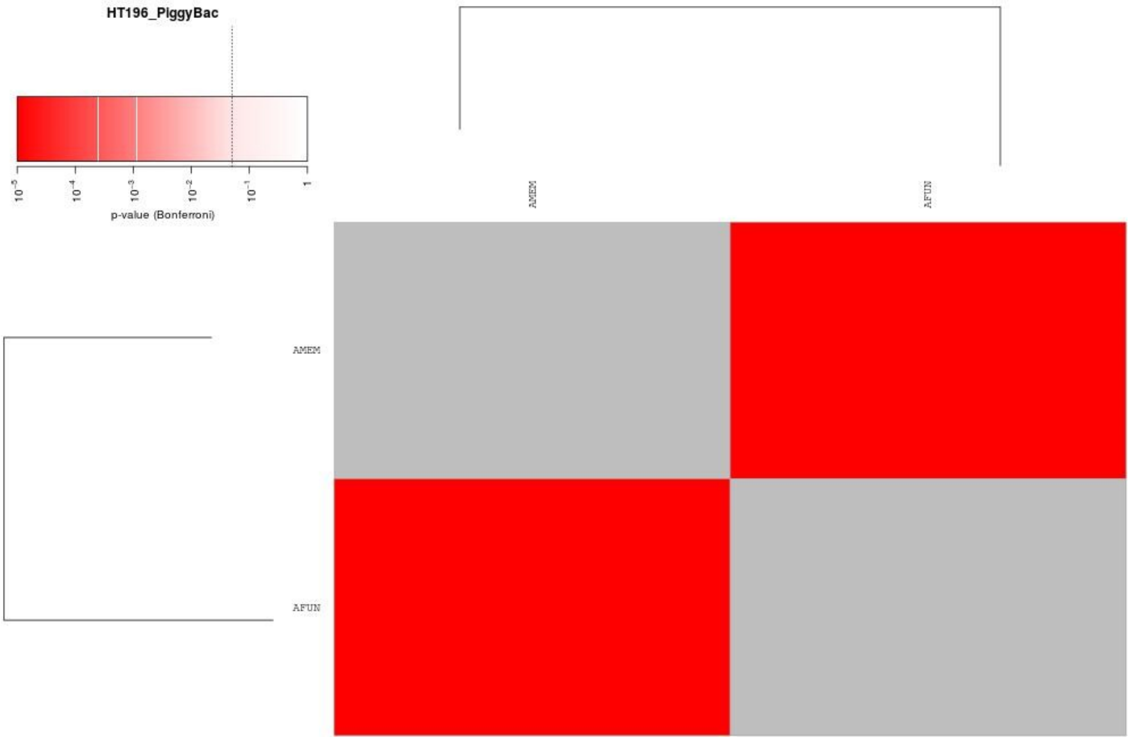
HT196_PiggyBac
p-value (Bonferroni)
AMEM
AFUN
AMEM
AFUN

## Sola

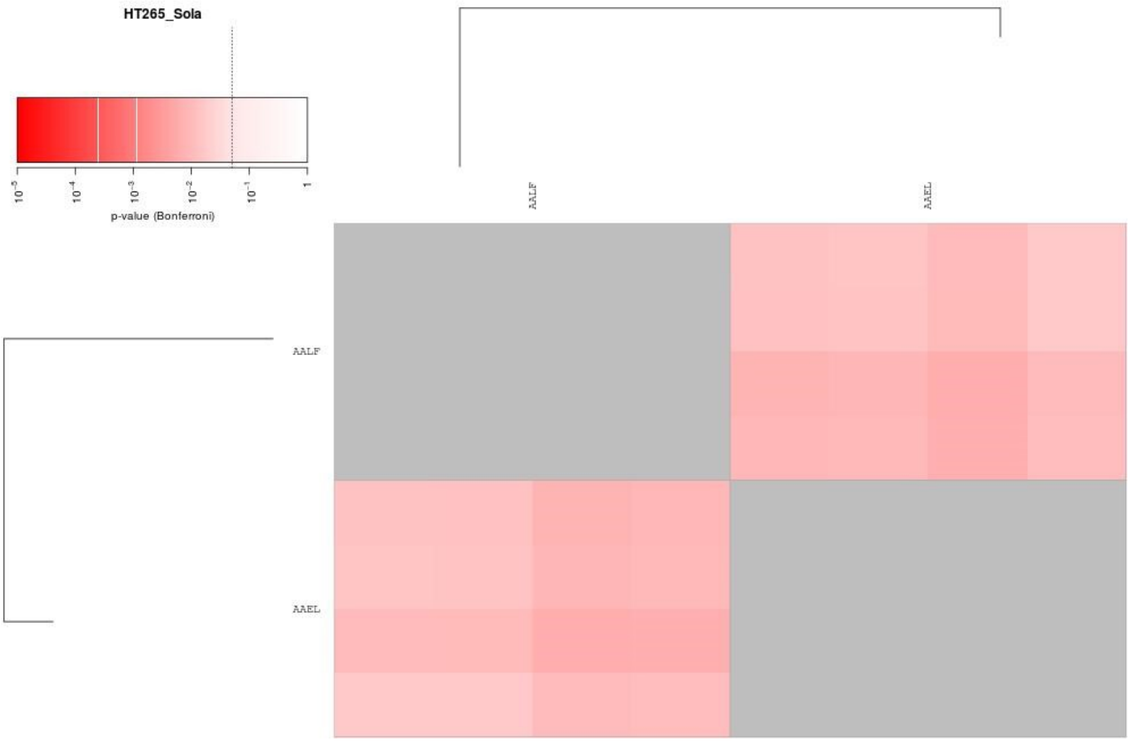


## Tc1-Mariner

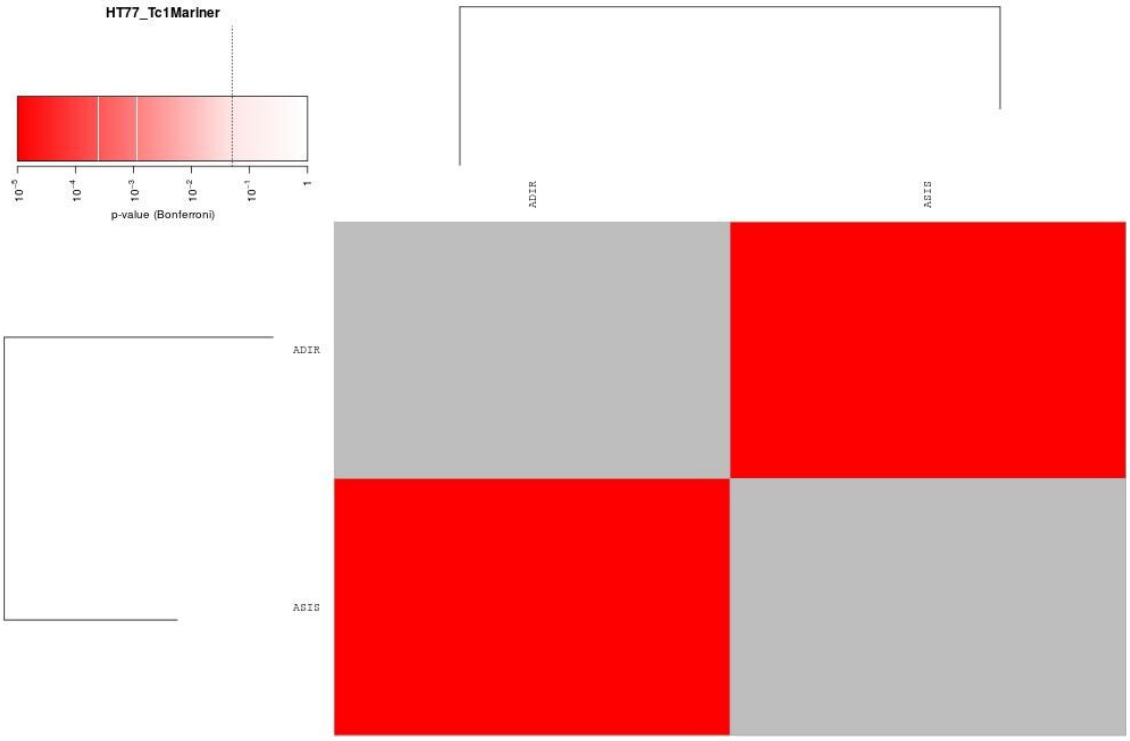

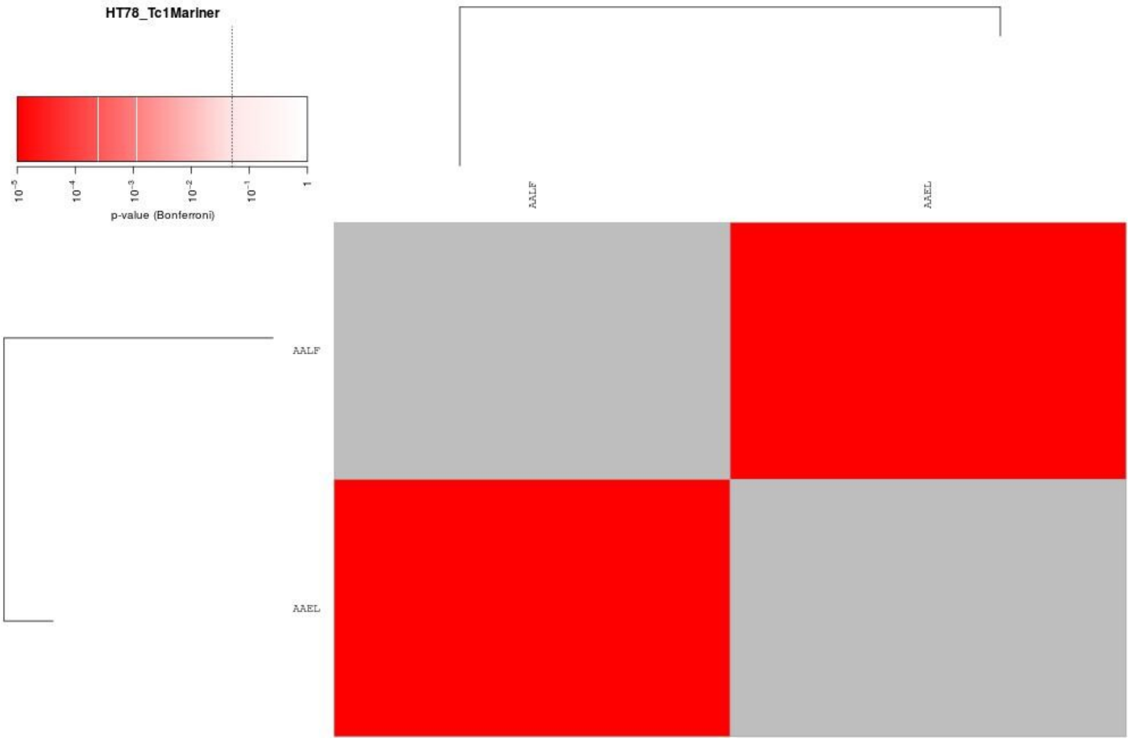
HT78_Tc1Mariner
p-value (Bonferroni)
AALF
AAEL
AALF
AAEL

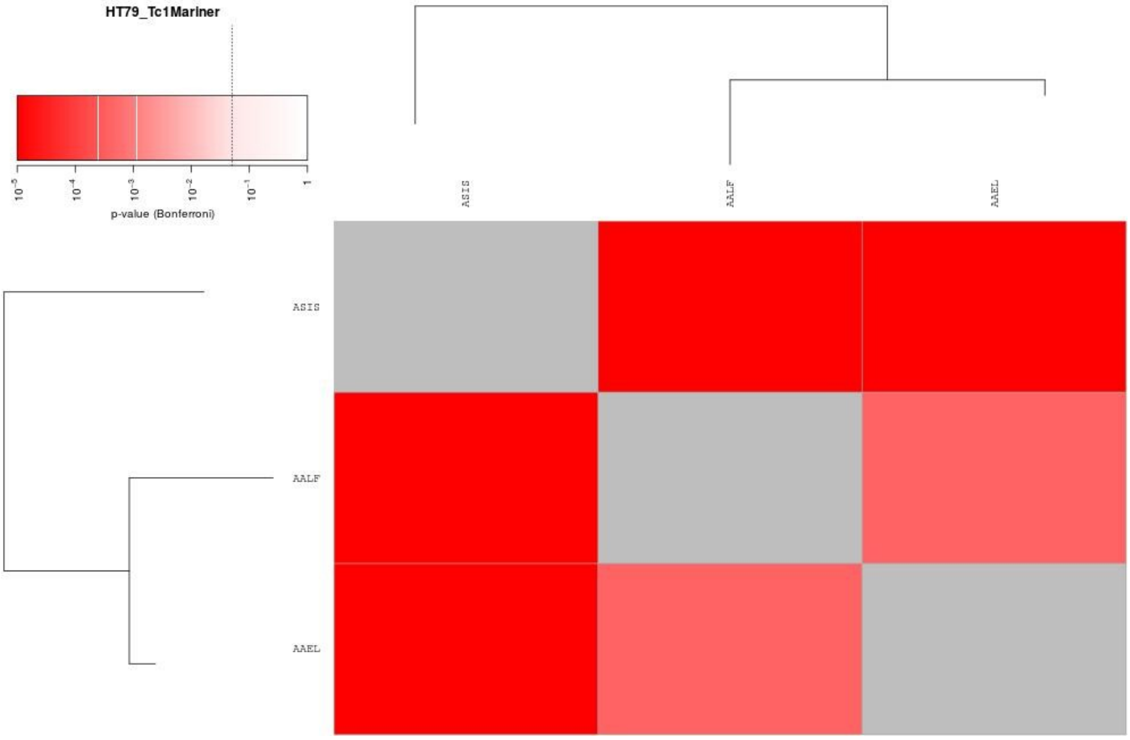
HT79_Tc1Mariner
p-value (Bonferroni)
ASIS
AALF
AAEL
ASIS
AALF
AAEL

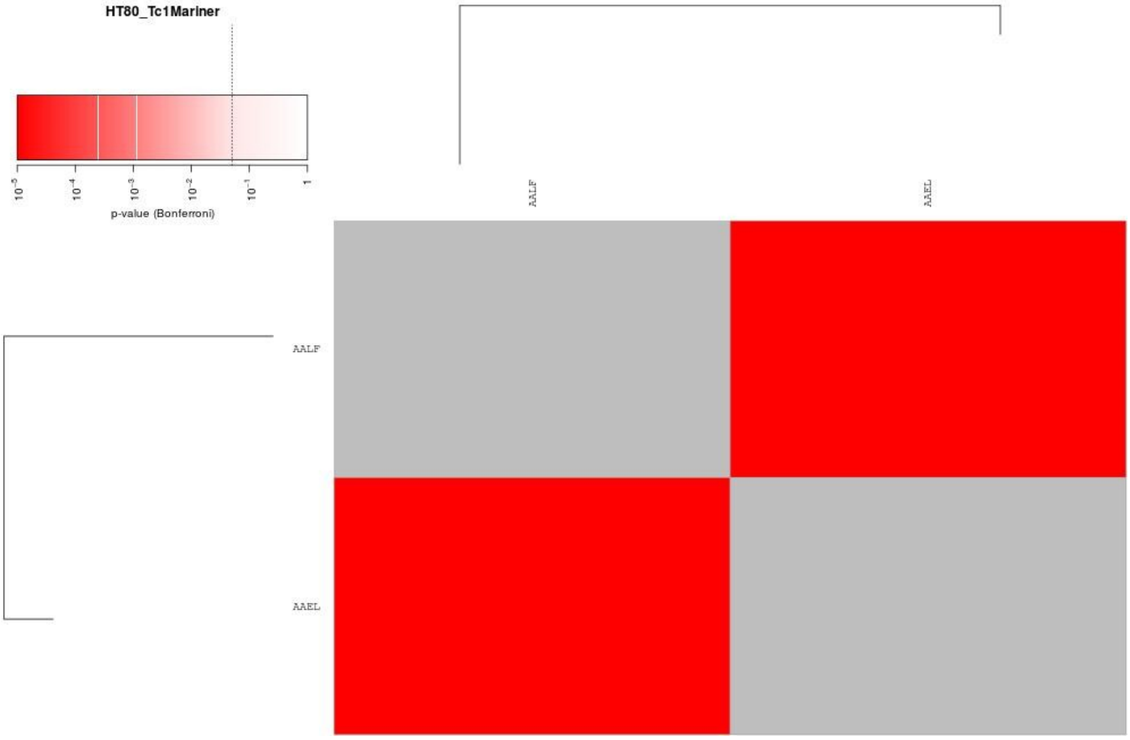
HT80_Tc1Mariner
p-value (Bonferroni)
AALF
AAEL
AALF
AAEL

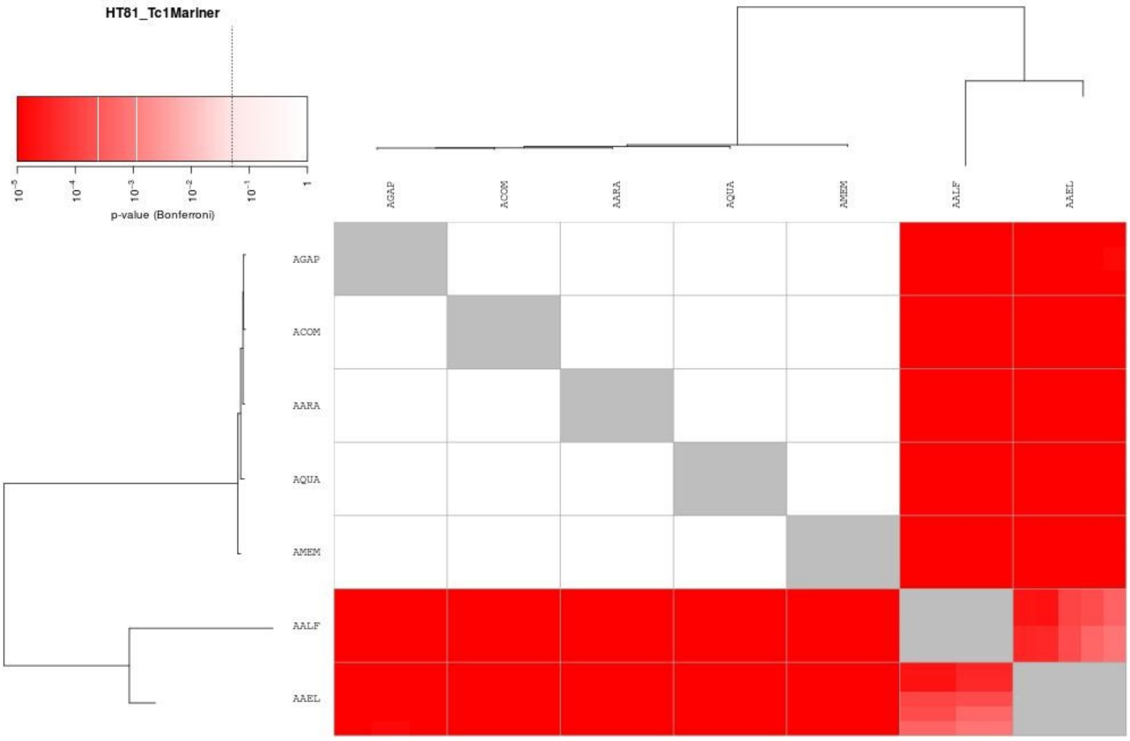
HT81_Tc1Mariner
p-value (Bonferroni)
AGAP
ACOM
AARA
AQUA
AMEM
AALF
AAEL
AGAP
ACOM
AARA
AQUA
AMEM
AALF
AAEL

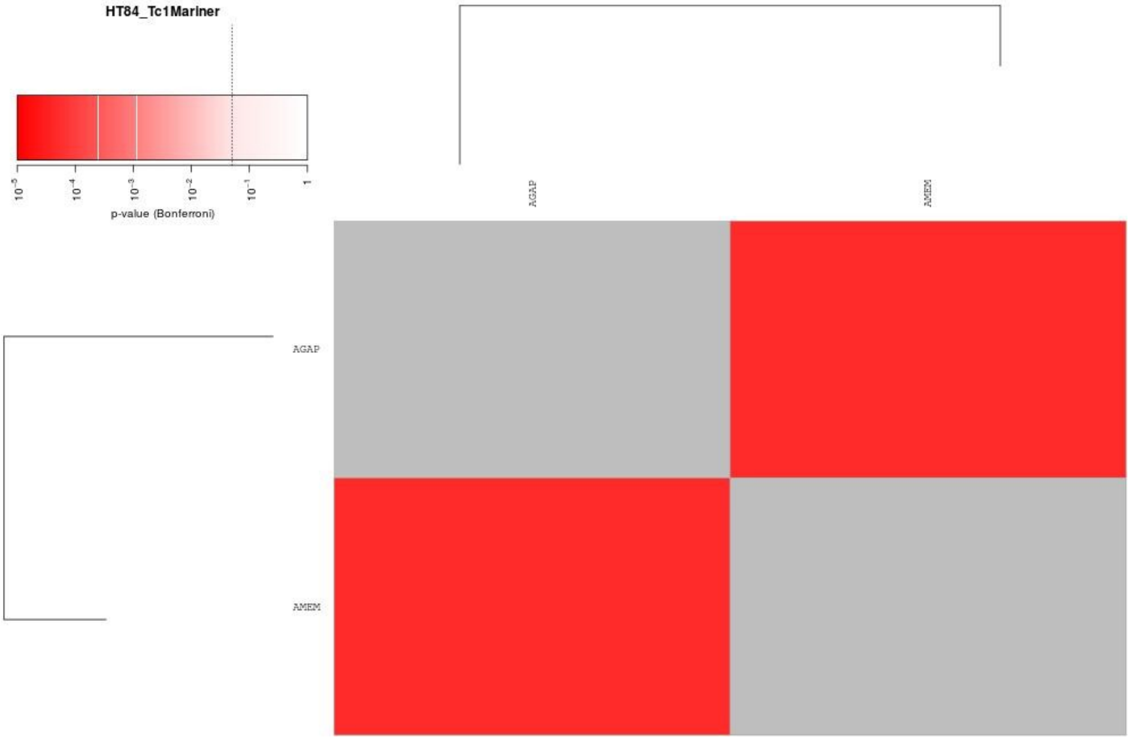
HT84_Tc1Mariner
p-value (Bonferroni)
AGAP
AMEM
AGAP
AMEM

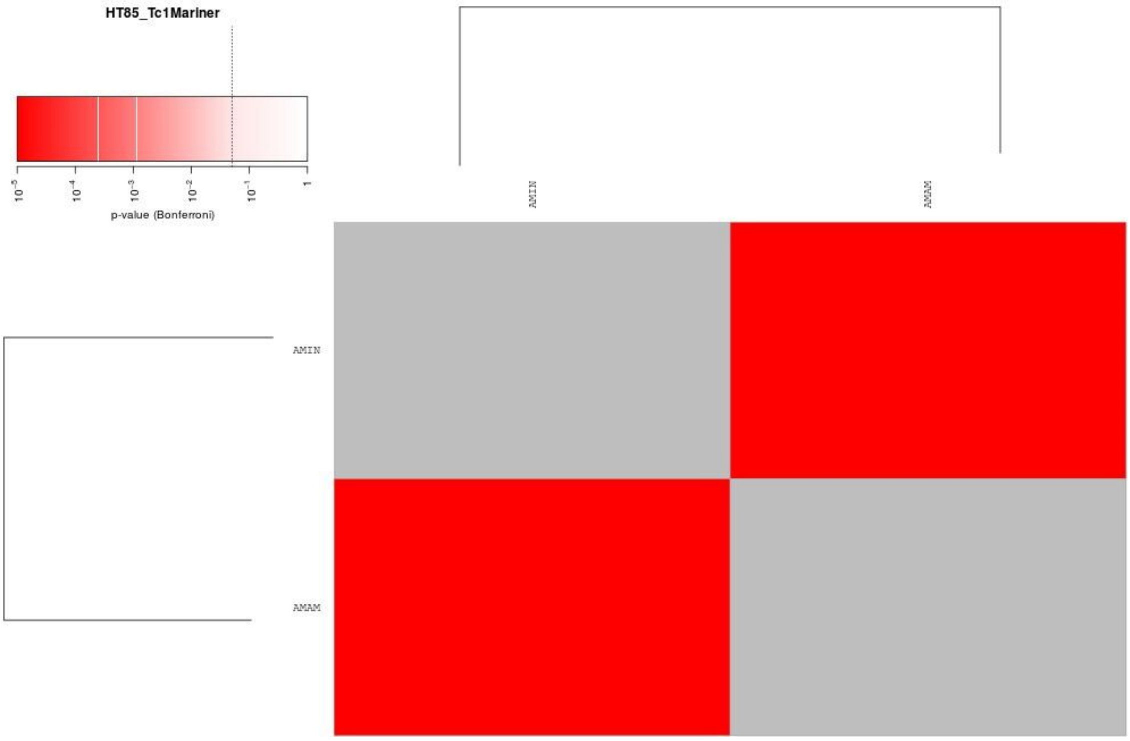
HT85_Tc1Mariner
p-value (Bonferroni)
AMIN
AMAM
AMIN
AMAM

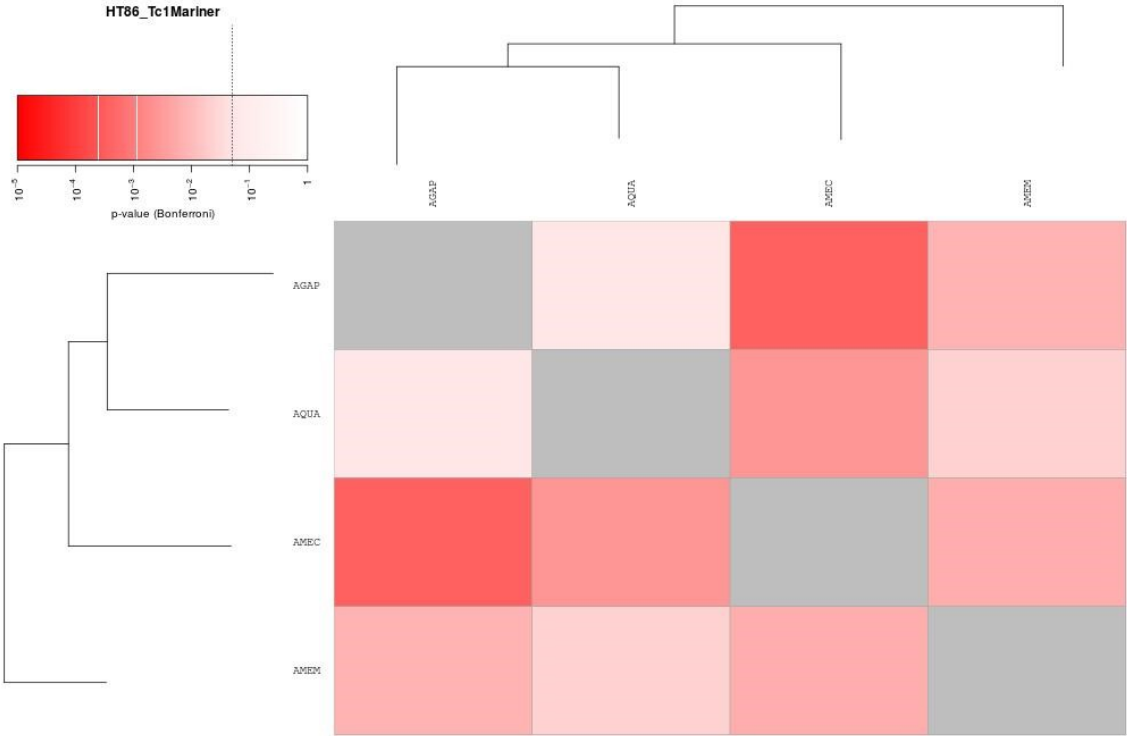
HT86_Tc1Mariner
p-value (Bonferroni)
AGAP
AQUA
AMEC
AMEM

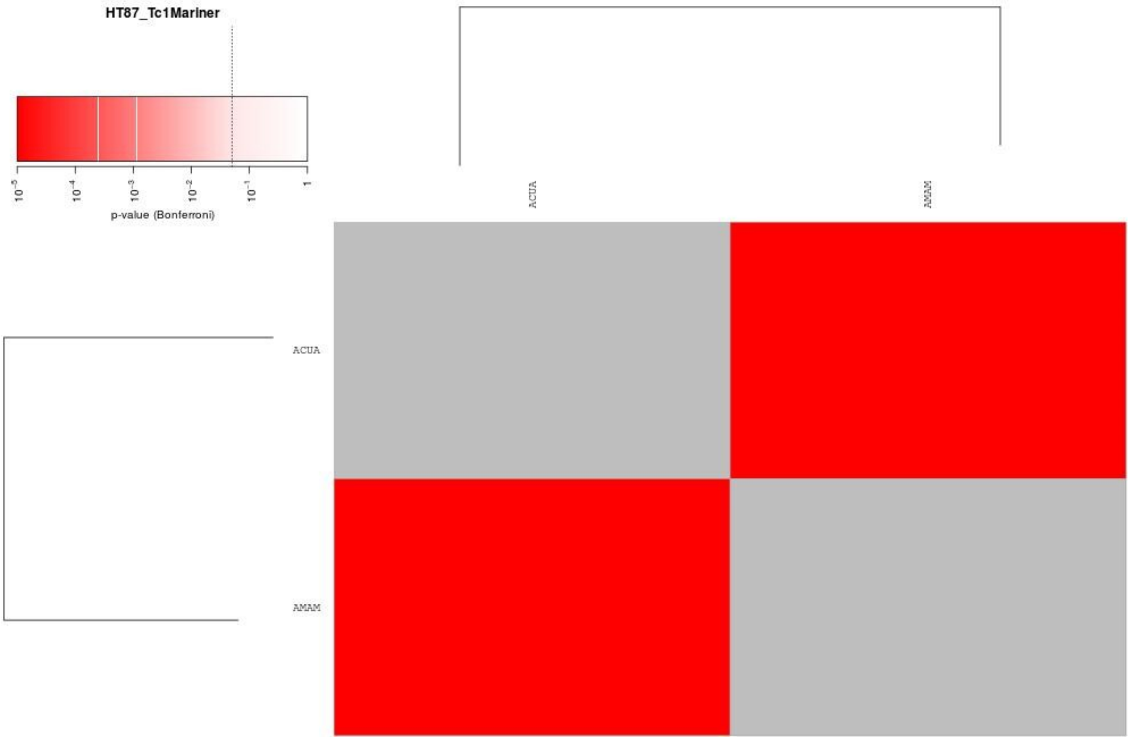
HT87_Tc1Mariner
p-value (Bonferroni)
ACUA
AMAM

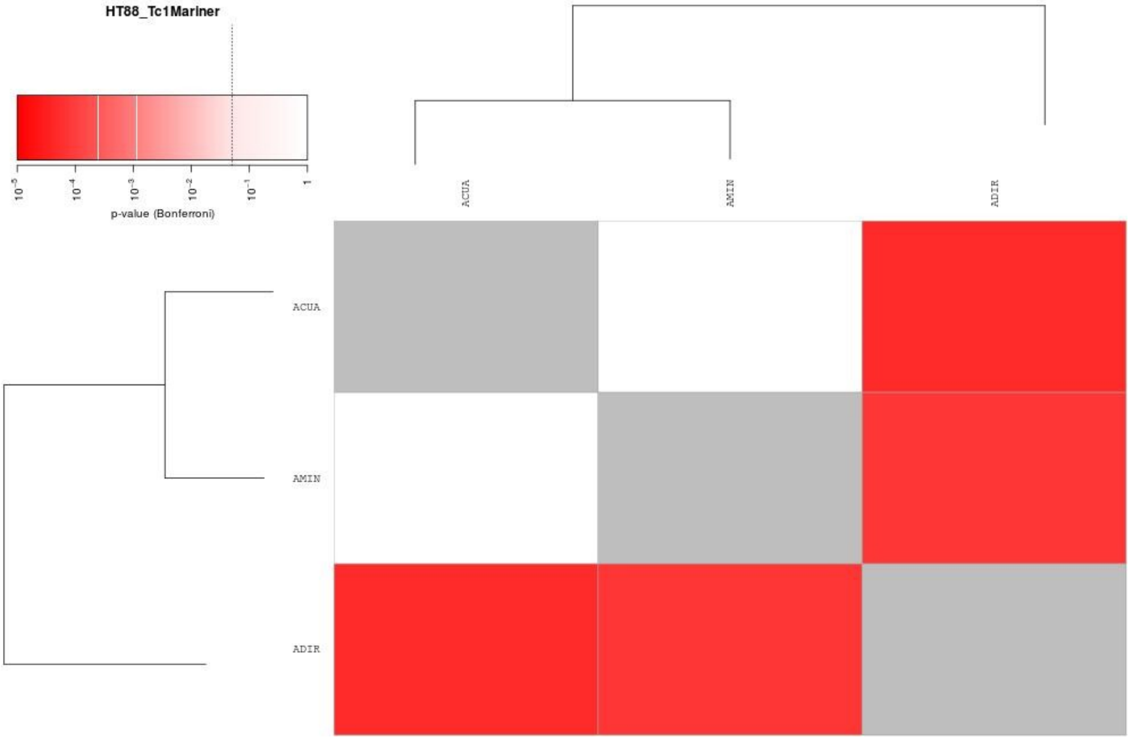
HT88_Tc1Mariner
p-value (Bonferroni)
ACUA
AMIN
ADIR

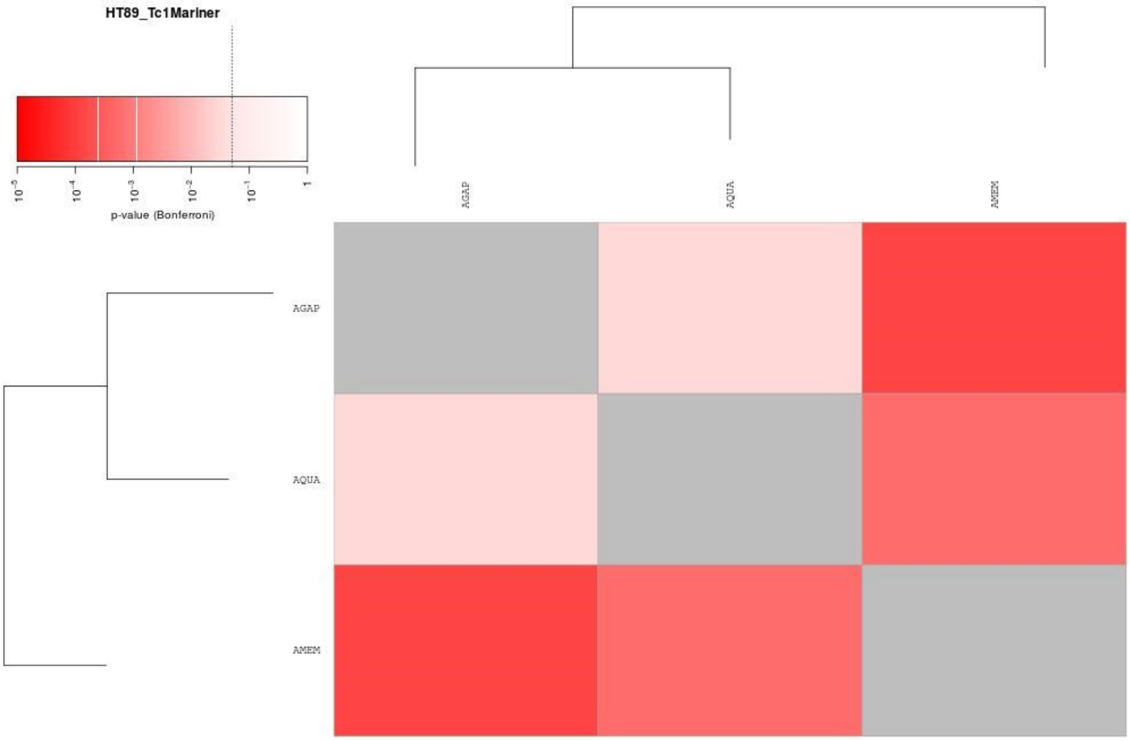
HT89_Tc1Mariner
p-value (Bonferroni)
AGAP
AQUA
AMEM

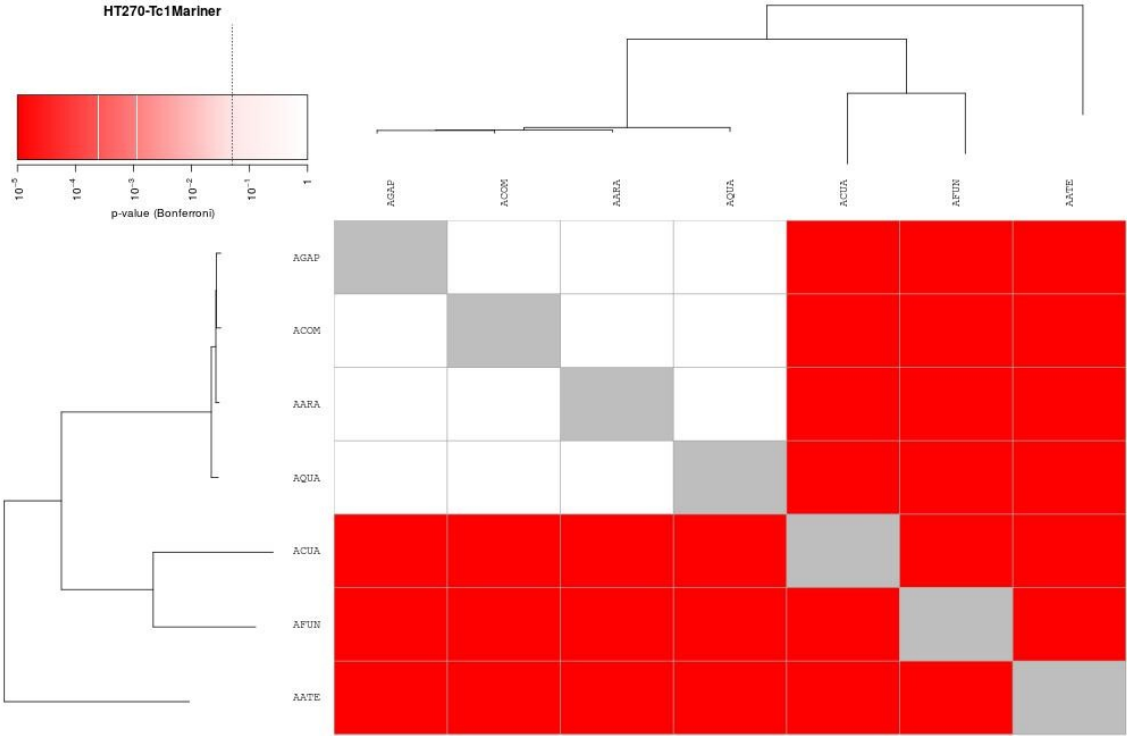
HT270-Tc1Mariner
p-value (Bonferroni)
AGAP
ACOM
AARA
AQUA
ACUA
AFUN
AATE

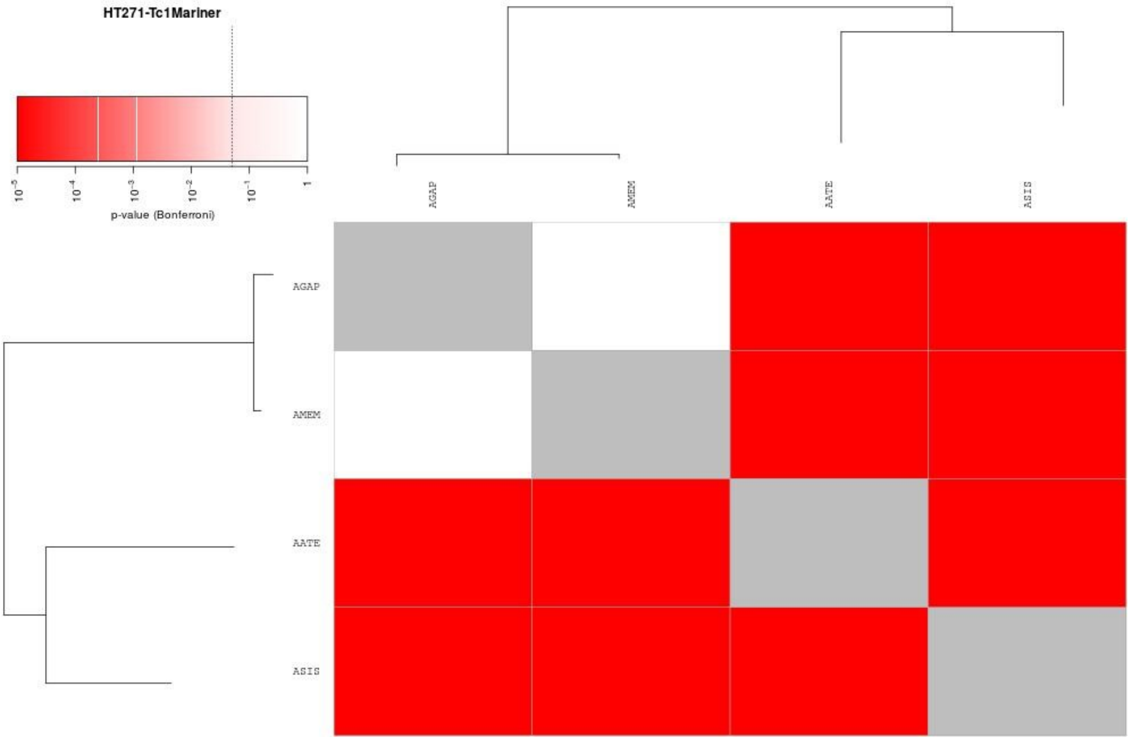
HT271-Tc1Mariner
p-value (Bonferroni)
AGAP
AMEM
AATE
ASIS

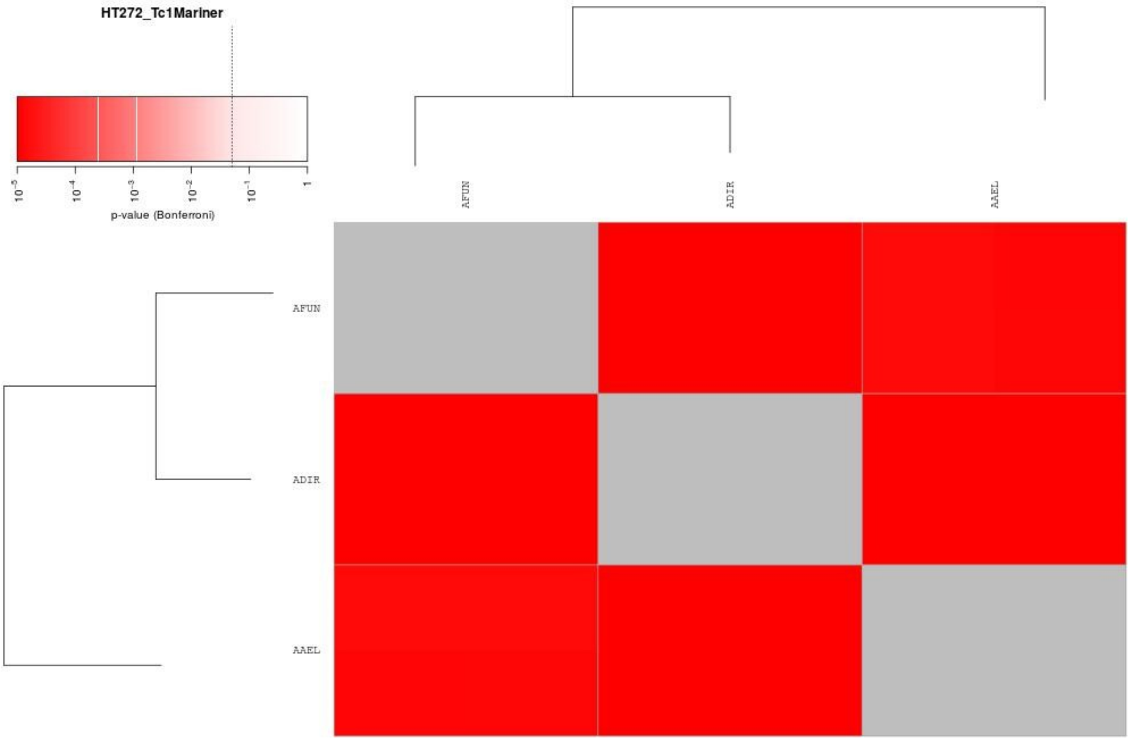
HT272_Tc1Mariner
p-value (Bonferroni)
AFUN
ADIR
AAEL
AFUN
ADIR
AAEL

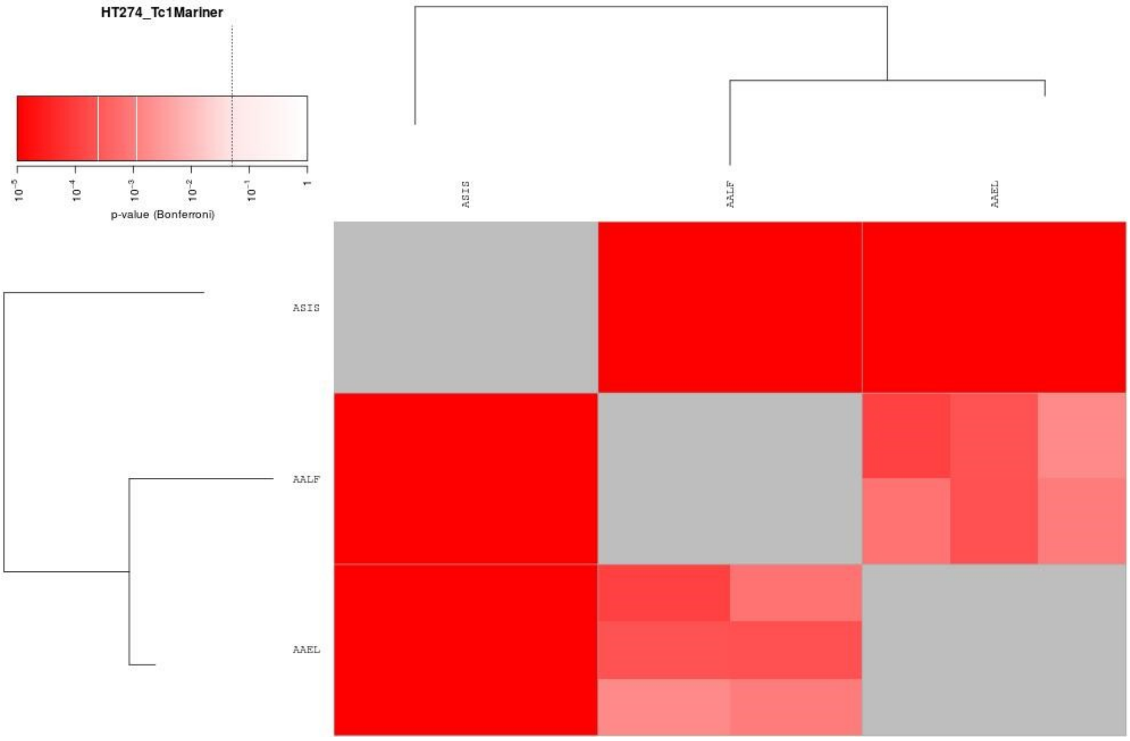
HT274_Tc1Mariner
p-value (Bonferroni)
ASIS
AALF
AAEL
ASIS
AALF
AAEL

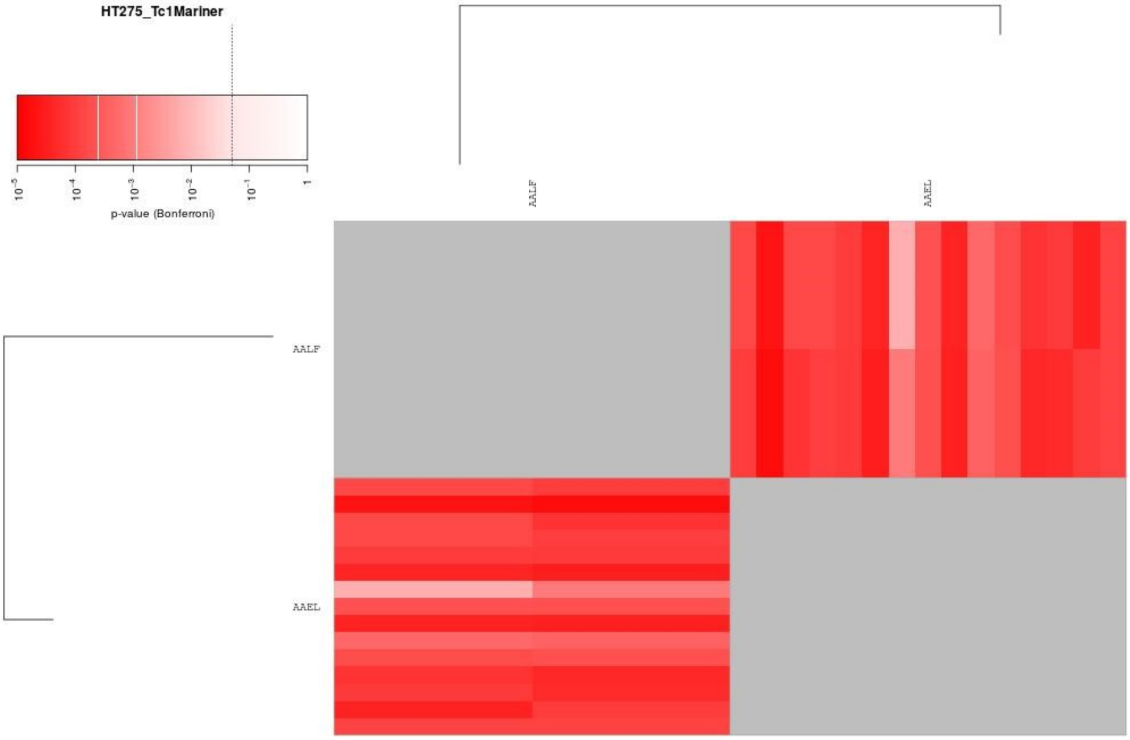
HT275_Tc1Mariner
p-value (Bonferroni)
AALF
AAEL
AALF
AAEL

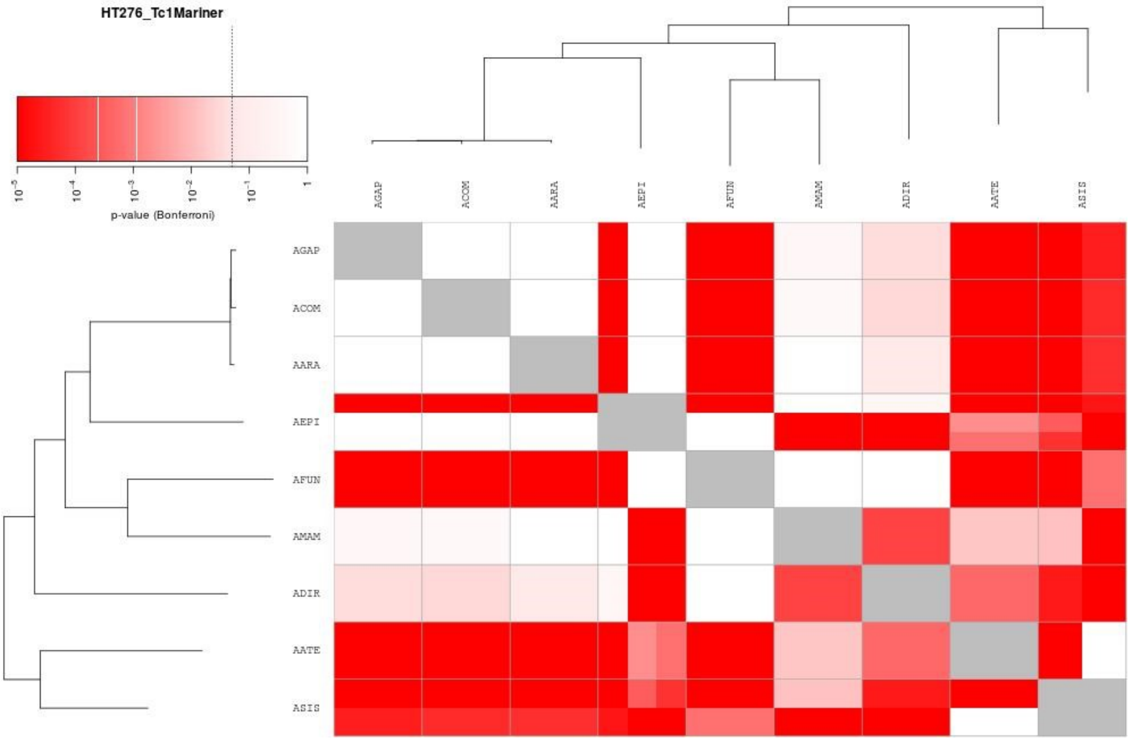
HT276_Tc1Mariner
p-value (Bonferroni)
AGAP
ACOM
AARA
AEPI
AFUN
AMAM
ADIR
AATE
ASIS
AGAP
ACOM
AARA
AEPI
AFUN
AMAM
ADIR
AATE
ASIS

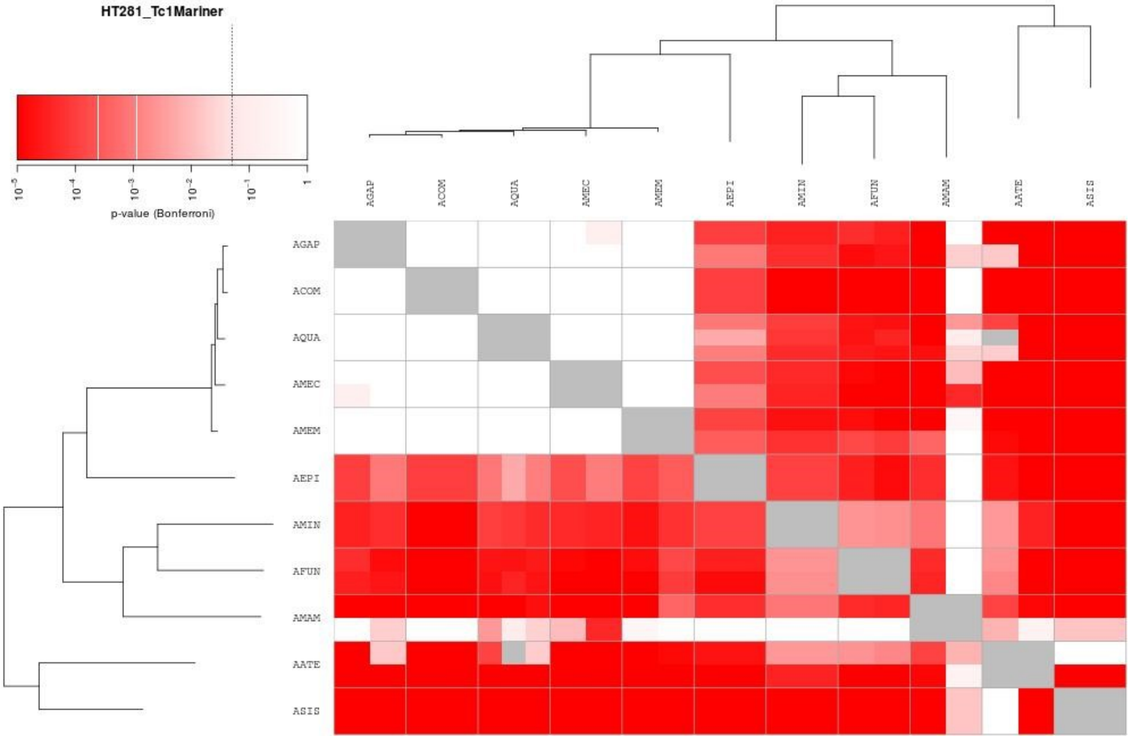
HT281_Tc1Mariner
p-value (Bonferroni)
AGAP
ACOM
AQUA
AMEC
AMEM
AEPI
AMIN
AFUN
AMAM
AATE
ASIS

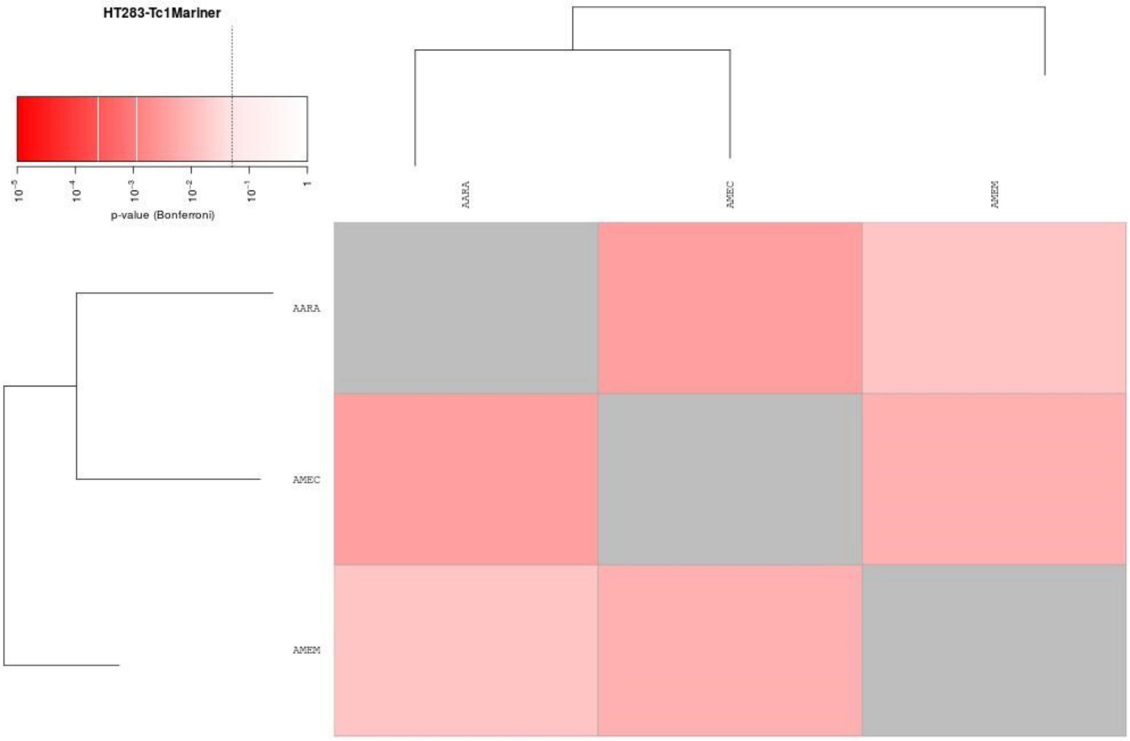
HT283-Tc1Mariner
p-value (Bonferroni)
AARA
AMEC
AMEM

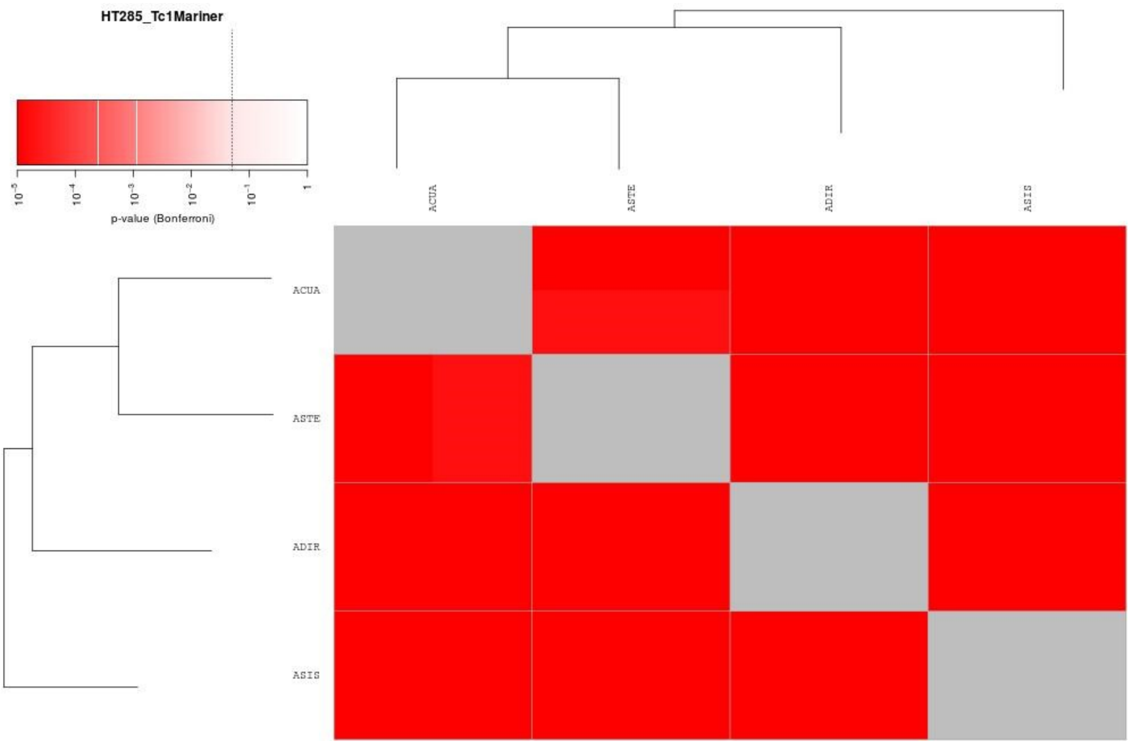
HT285_Tc1Mariner
p-value (Bonferroni)
ACUA
ASTE
ADIR
ASIS

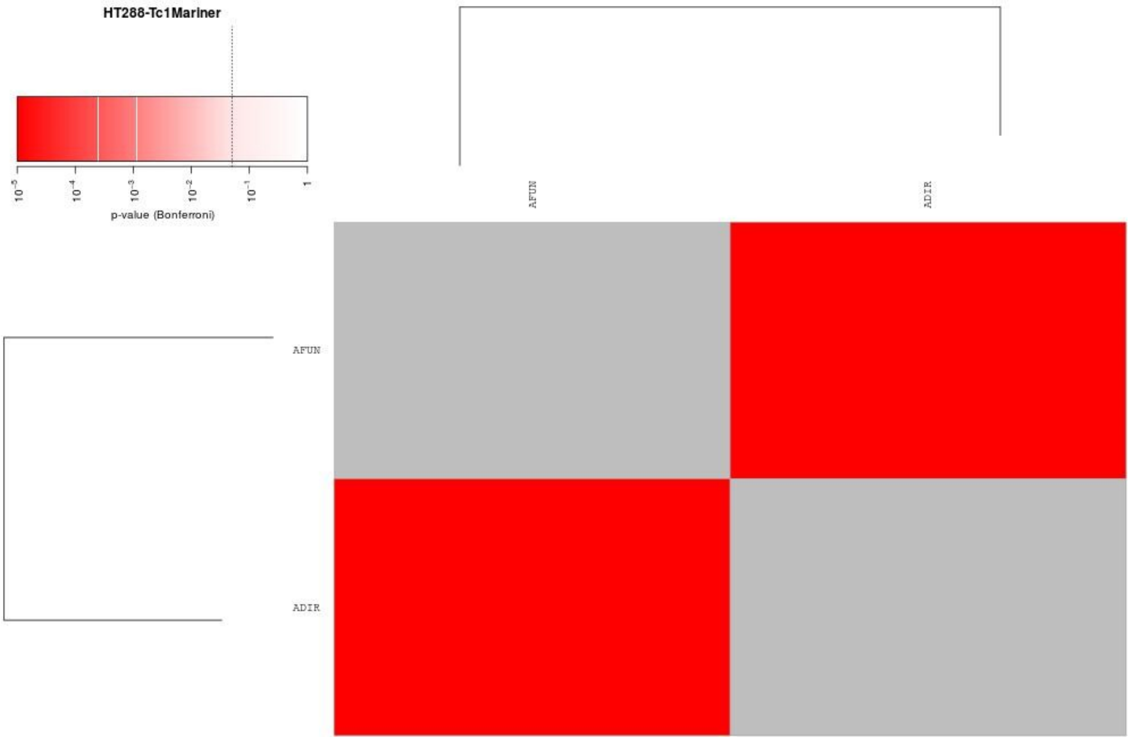
HT288-Tc1Mariner
p-value (Bonferroni)
AFUN
ADIR

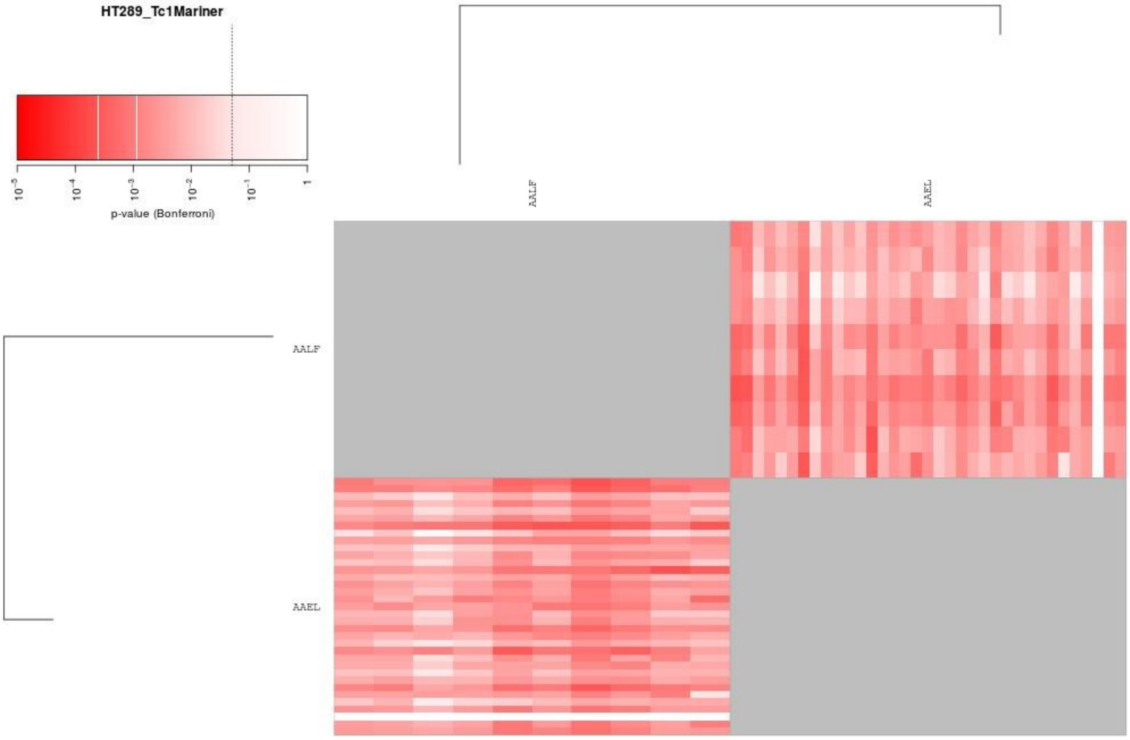
HT289_Tc1Mariner
p-value (Bonferroni)
AALF
AAEL
AALF
AAEL

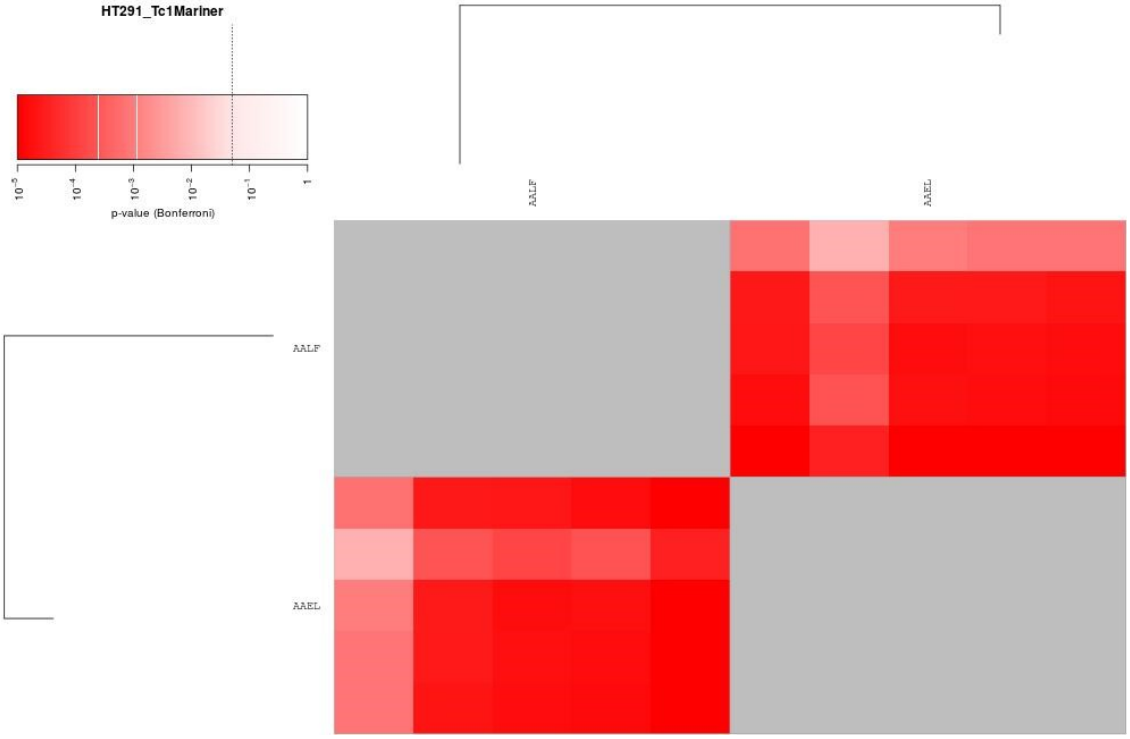
HT291_Tc1Mariner
p-value (Bonferroni)
AALF
AAEL
AALF
AAEL

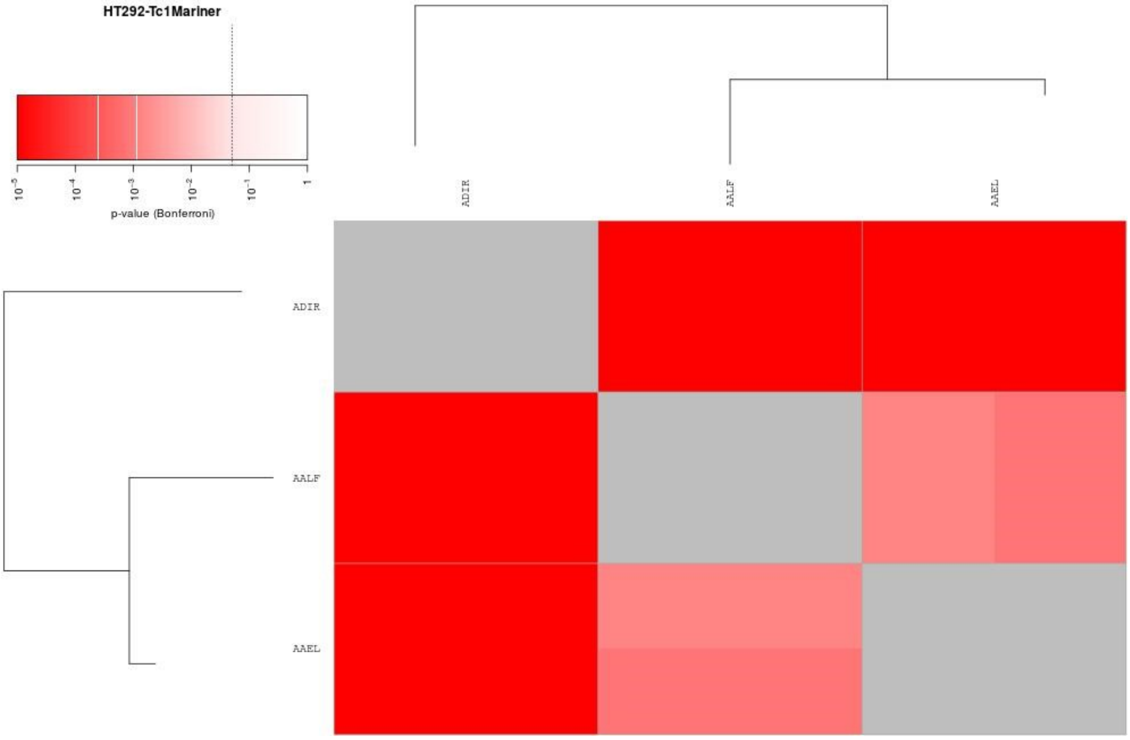
HT292-Tc1Mariner
p-value (Bonferroni)
ADIR
AALF
AAEL
ADIR
AALF
AAEL

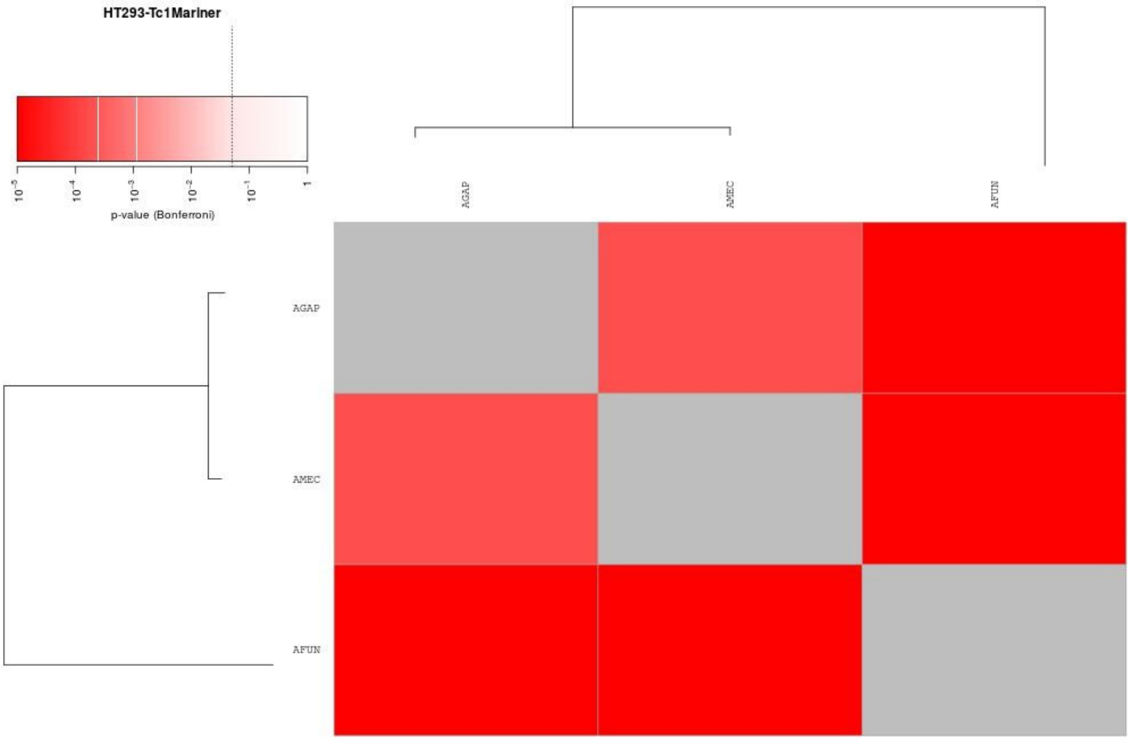
HT293-Tc1Mariner
p-value (Bonferroni)
AGAP
AMEC
AFUN
AGAP
AMEC
AFUN

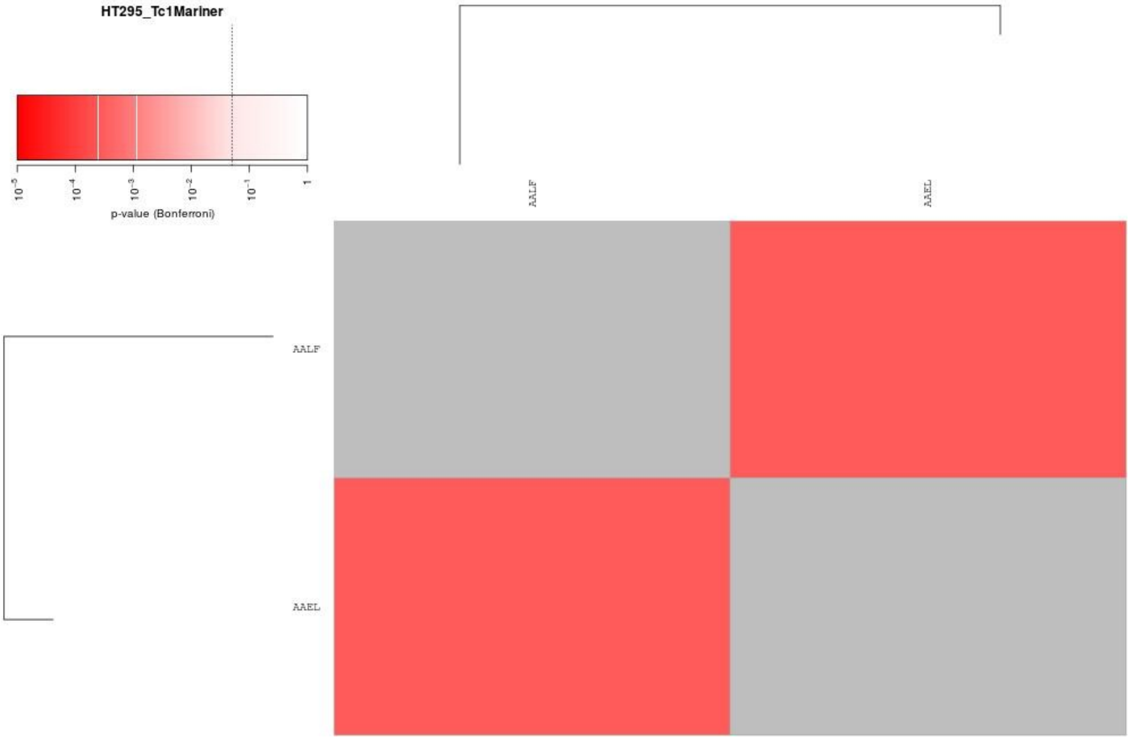
HT295_Tc1Mariner
p-value (Bonferroni)
AALF
AAEL
AALF
AAEL

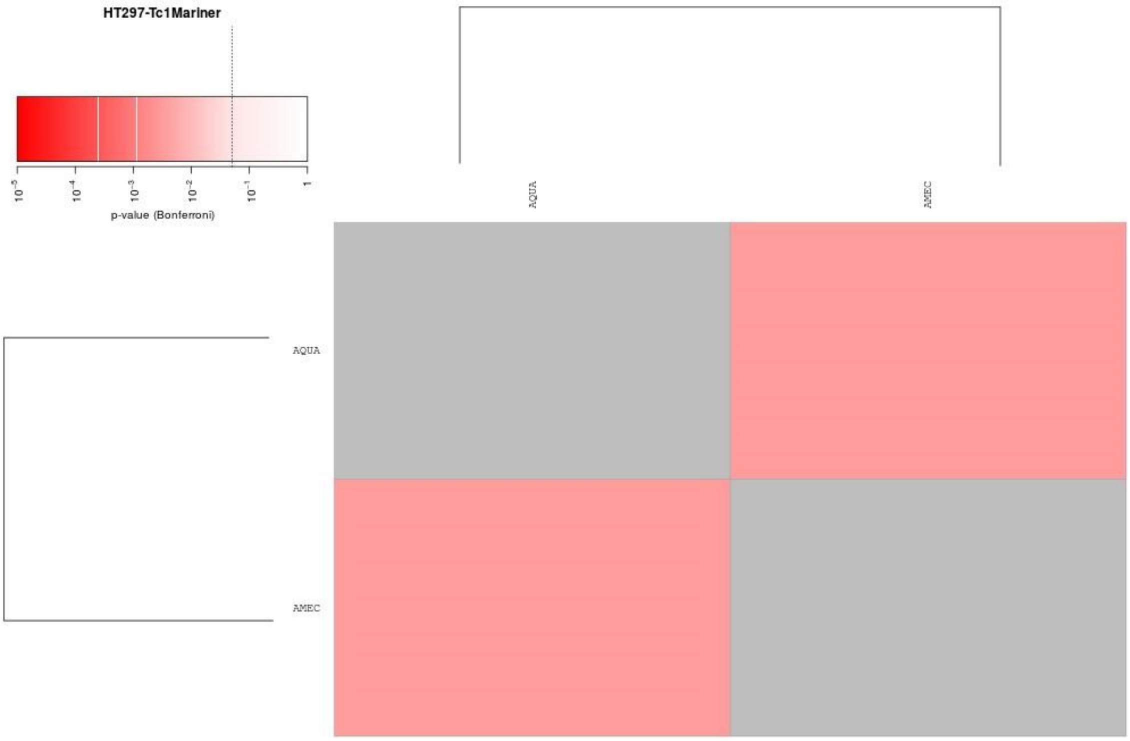
HT297-Tc1Mariner
p-value (Bonferroni)
AQUA
AMEC
AQUA
AMEC

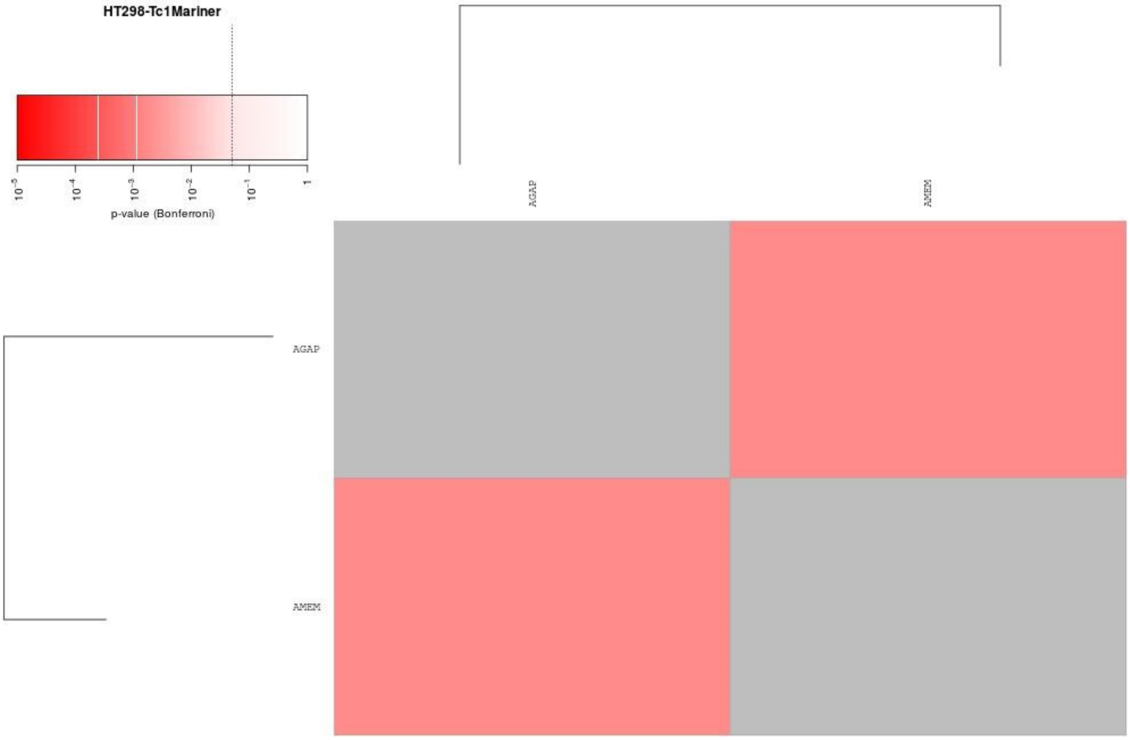
HT298-Tc1Mariner
p-value (Bonferroni)
AGAP
AMEM
AGAP
AMEM

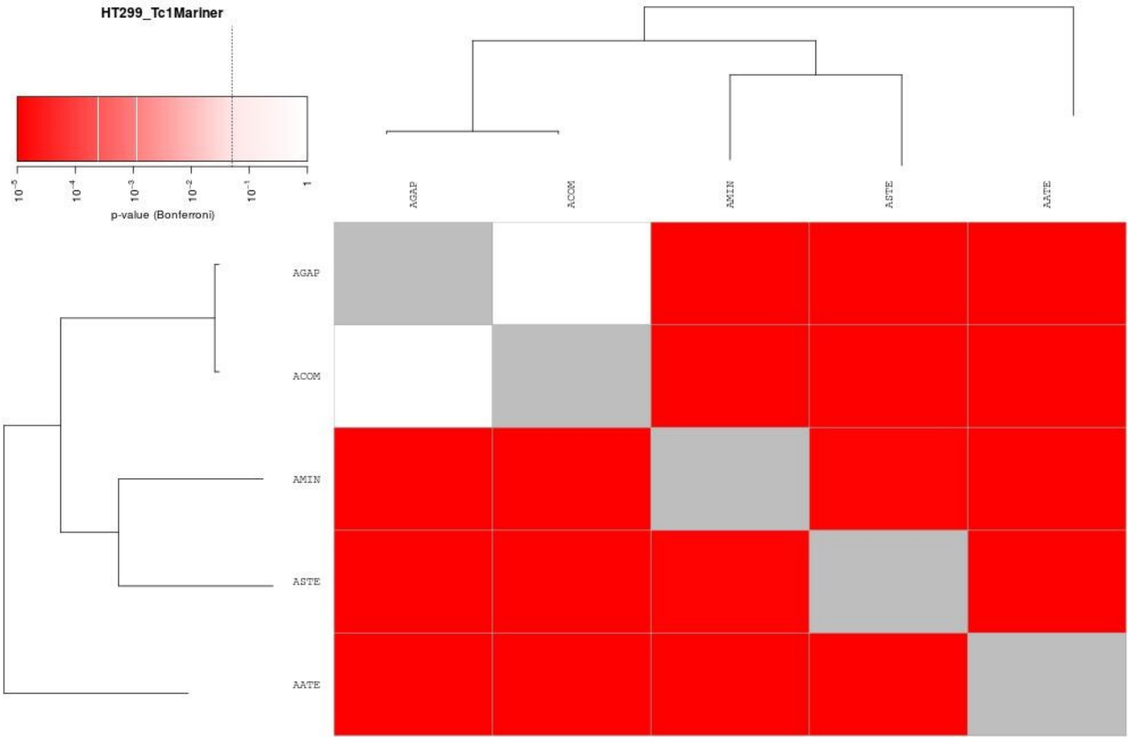
HT299_Tc1Mariner
p-value (Bonferroni)
AGAP
ACOM
AMIN
ASTE
AATE
AGAP
ACOM
AMIN
ASTE
AATE

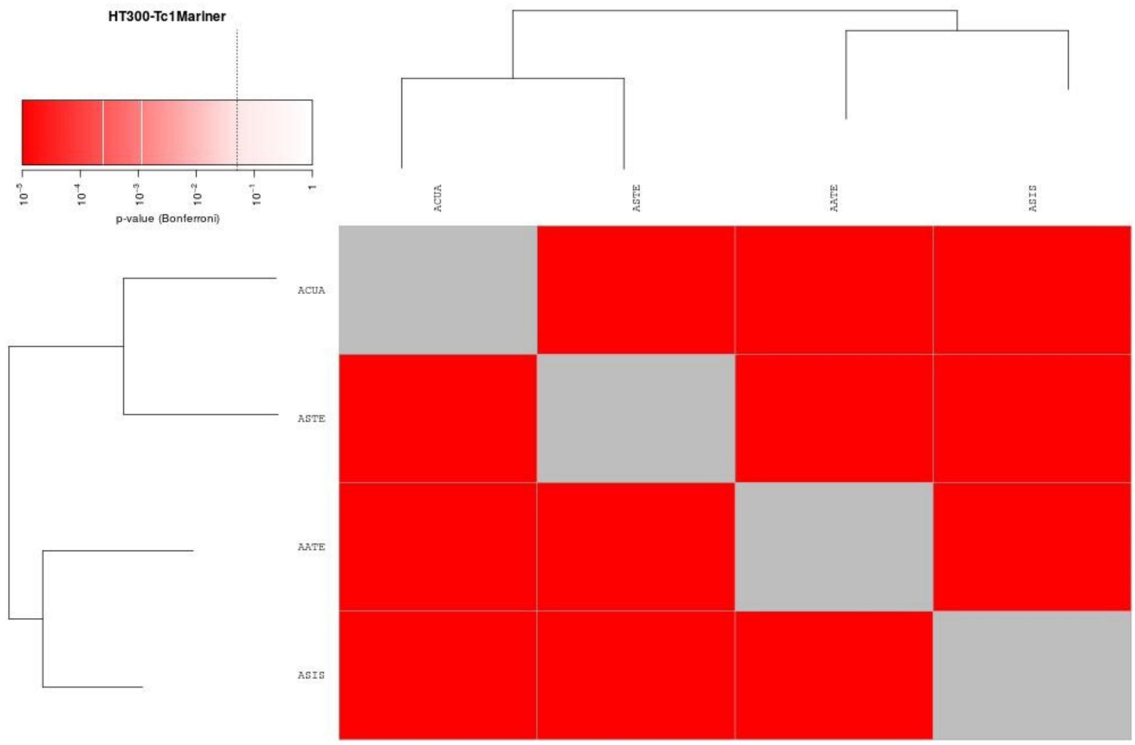
HT300-Tc1Mariner
p-value (Bonferroni)
ACUA
ASTE
AATE
ASIS

## Transib

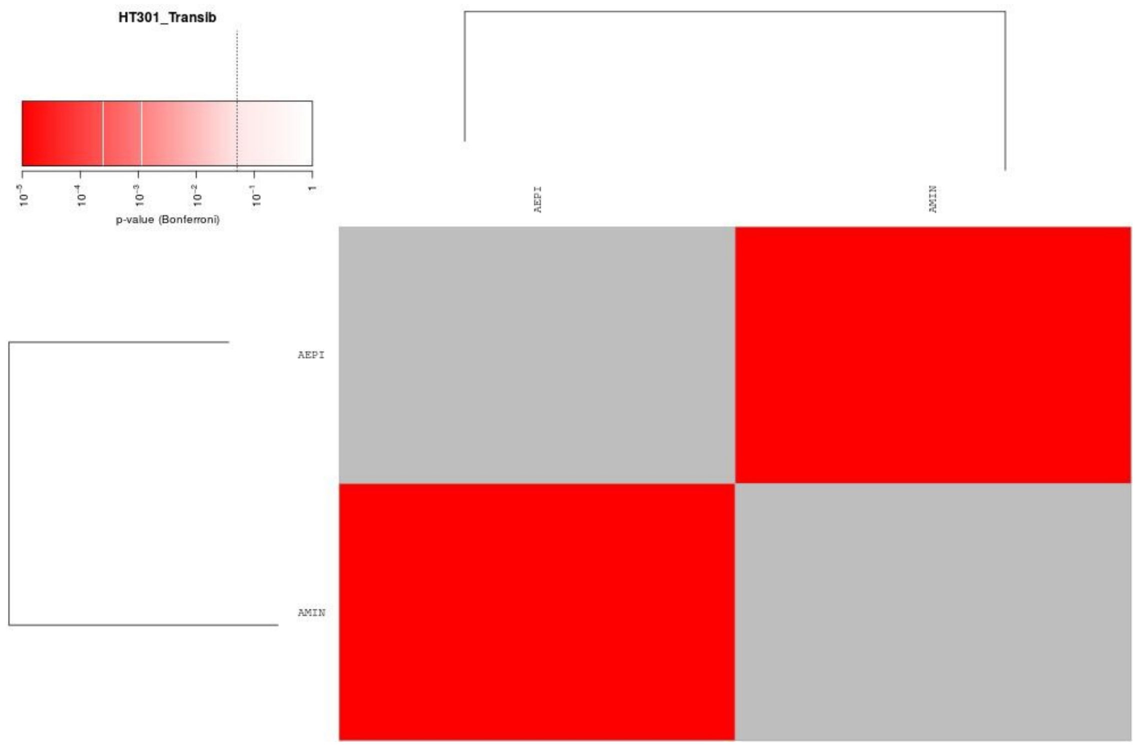
HT301_Transib
p-value (Bonferroni)
AEPI
AMIN
