# Supplementary material for: Mosquito genomes are frequently invaded by transposable elements through horizontal transfer
Source: PLoS Genet. 2020 Nov 30;16(11):e1008946. doi: 10.1371/journal.pgen.1008946 (PMC7728395; doi:10.1371/journal.pgen.1008946)
Supplement: S4 File — The genome IDs, taxa, and species/virus names of more than 32,000 genomes that were searched for horizontally transferred mosquito TEs. (PDF) [file pgen.1008946.s004.pdf]

Viridiplantae

taxid	Acession	Species
1590841	GCA_003024255.1	Actinidia chinensis var. chinensis (eudicots)
169297	GCA_001957025.1	Aegilops tauschii subsp. tauschii (monocots)
228871	GCA_000411095.1	Aethionema arabicum (eudicots)
3517	GCA_003254965.1	Alnus glutinosa (eudicots)
28502	GCA_000753965.1	Amaranthus hypochondriacus (grain amaranth)
277990	GCA_000180655.1	Amaranthus tuberculatus (eudicots)
13333	GCA_000471905.1	Amborella trichopoda (flowering plants)
4615	GCA_001540865.1	Ananas comosus (pineapple)
1088818	GCA_002786265.1	Apostasia shenzhenica (monocots)
1472648	GCA_000696445.1	Aquilaria agallochum (eudicots)
218851	GCA_002738505.1	Aquilegia coerulea (Rocky Mountain columbine)
63677	GCA_900078215.1	Arabidopsis halleri subsp. gemmifera (eudicots)
81972	GCA_000004255.1	Arabidopsis lyrata subsp. lyrata (lyrate rockcress)
50452	GCA_900128785.1	Arabis alpina (gray rockcress)
746018	GCA_001484125.1	Arabis montbretiana (eudicots)
571359	GCA_001484925.1	Arabis nordmanniana (eudicots)
130453	GCA_000817695.2	Arachis duranensis (eudicots)
3818	GCA_003086295.1	Arachis hypogaea (peanut)
130454	GCA_000816755.2	Arachis ipaensis (eudicots)
227691	GCA_003063285.2	Arachis monticola (eudicots)
85884	GCA_003260245.1	Argania spinosa (eudicots)
35608	GCA_003112345.1	Artemisia annua (sweet wormwood)
709039	GCA_002024485.1	Artocarpus camansi (eudicots)
48545	GCA_002018285.1	Asclepias syriaca (eudicots)
4686	GCA_001876935.1	Asparagus officinalis (garden asparagus)
76974	GCA_002013935.1	Atalantia buxifolia (eudicots)
3075	GCA_000733215.1	Auxenochlorella protothecoides (green algae)
3078	GCA_001430745.1	Auxenochlorella pyrenoidosa (green algae)
124943	GCA_000439995.3	Azadirachta indica (eudicots)
50459	GCA_001920985.1	Barbarea vulgaris (winter cress)
41875	GCA_002220235.1	Bathycoccus prasinos (green algae)
1735750	GCA_900128745.1	Bathycoccus sp. TOSAG39-1 (green algae)
200001	GCA_003255005.1	Begonia fuchsioides (eudicots)
121720	GCA_003290165.1	Berberis thunbergii (Japanese barberry)
3555	GCA_000511025.2	Beta vulgaris subsp. vulgaris (beet)
216990	GCA_000327005.1	Betula nana (alpine birch)
3505	GCA_900184695.1	Betula pendula (European white birch)
72658	GCA_002079875.1	Boechera stricta (eudicots)
83906	GCA_002937015.1	Boehmeria nivea (eudicots)
38881	GCA_002005505.1	Botryococcus braunii (green algae)
15368	GCA_000005505.4	Brachypodium distachyon (stiff brome)
69181	GCA_003260655.1	Brassica cretica (eudicots)
323352	GCA_001687265.1	Brassica juncea var. tumida (zha cai)
3708	GCA_000686985.2	Brassica napus (rape)

3710	GCA_001682895.1	Brassica nigra (black mustard)
109376	GCA_000695525.1	Brassica oleracea var. oleracea (wild cabbage)
3711	GCA_000309985.2	Brassica rapa (field mustard)
3821	GCA_000340665.1	Cajanus cajan (pigeon pea)
746888	GCA_900491605.1	Calamus simplicifolius (monocots)
90675	GCA_000633955.1	Camelina sativa (false flax)
3483	GCA_003417725.2	Cannabis sativa (hemp)
3719	GCA_001974645.1	Capsella bursa-pastoris (shepherd's purse)
81985	GCA_000375325.1	Capsella rubella (eudicots)
4072	GCA_000710875.1	Capsicum annuum (eudicots)
33114	GCA_002271885.2	Capsicum baccatum (eudicots)
80379	GCA_002271895.2	Capsicum chinense (eudicots)
3649	GCA_000150535.1	Carica papaya (papaya)
171969	GCA_002740515.1	Carnegiea gigantea (saguaro)
4222	GCA_001633085.1	Carthamus tinctorius (safflower)
60419	GCA_000763605.1	Castanea mollissima (Chinese chestnut)
228173	GCA_003795335.1	Casuarina equisetifolia subsp. incana (eudicots)
3522	GCA_003255045.1	Casuarina glauca (swamp oak)
4058	GCA_000949345.1	Catharanthus roseus (Madagascar periwinkle)
4543	GCA_002174835.2	Cenchrus americanus (monocots)
3775	GCA_001972305.1	Cephalotus follicularis (eudicots)
49801	GCA_003255065.1	Cercis canadensis (eudicots)
53854	GCA_003254925.1	Chamaecrista fasciculata (eudicots)
69332	GCA_003427395.1	Chara braunii (green plants)
882375	GCA_001687005.1	Chenopodium pallidicaule (eudicots)
63459	GCA_001683475.1	Chenopodium quinoa (quinoa)
434248	GCA_001687025.1	Chenopodium suecicum (eudicots)
35704	GCA_001662365.1	Chlamydomonas applanata (green algae)
51683	GCA_001662385.1	Chlamydomonas asymmetrica (green algae)
1157962	GCA_002335675.1	Chlamydomonas eustigma (green algae)
3055	GCA_000002595.3	Chlamydomonas reinhardtii (green algae)
28458	GCA_001662425.1	Chlamydomonas sphaeroides (green algae)
3076	GCA_003130725.1	Chlorella sorokiniana (green algae)
2041167	GCA_003063905.1	Chlorella sp. A99 (green algae)
1415603	GCA_002896455.3	Chlorella sp. ArM0029B (green algae)
554065	GCA_000147415.1	Chlorella variabilis (green algae)
3077	GCA_001021125.1	Chlorella vulgaris (green algae)
3827	GCA_000331145.1	Cicer arietinum (chickpea)
90897	GCA_002896215.1	Cicer echinospermum (eudicots)
90898	GCA_003689015.1	Cicer reticulatum (eudicots)
337451	GCA_003546025.1	Cinnamomum micranthum f. kanehirae (flowering plants)
165298	GCA_002878655.1	Cissus quadrangularis (eudicots)
3654	GCA_000238415.1	Citrullus lanatus (wild melon)
2709	GCA_002013975.2	Citrus cavaleriei (Ichang papeda)
85681	GCA_000493195.1	Citrus clementina (clementine)
37334	GCA_002006925.1	Citrus maxima (eudicots)

171251	GCA_002013955.2	Citrus medica (eudicots)
85571	GCA_003258625.1	Citrus reticulata (eudicots)
2711	GCA_000317415.1	Citrus sinensis (sweet orange)
55188	GCA_002897195.1	Citrus unshiu (eudicots)
309804	GCA_001929425.1	Citrus x paradisi x Citrus trifoliata (citrumelo)
1579116	GCA_000812005.1	Coccomyxa sp. LA000219 (green algae)
1667192	GCA_001244535.1	Coccomyxa sp. SUA001 (green algae)
574566	GCA_000258705.1	Coccomyxa subellipsoidea C-169 (green algae)
13894	GCA_003604295.1	Cocos nucifera (coconut palm)
1489057	GCA_001630525.1	Coelastrella sp. M60 (green algae)
2033643	GCA_002588565.1	Coelastrella sp. UTEX B 3026 (green algae)
13443	GCA_003713225.1	Coffea arabica (coffee)
49369	GCA_003713205.1	Coffea eugenioides (eudicots)
311912	GCA_900108845.1	Conringia planisiliqua (eudicots)
210143	GCA_001974805.1	Corchorus capsularis (jute)
93759	GCA_001974825.1	Corchorus olitorius (Nalta jute)
3656	GCA_000313045.1	Cucumis melo (muskmelon)
3659	GCA_000004075.2	Cucumis sativus (cucumber)
3661	GCA_002738345.1	Cucurbita maxima (winter squash)
3662	GCA_002738365.1	Cucurbita moschata (crookneck pumpkin)
3664	GCA_002806865.2	Cucurbita pepo subsp. pepo (vegetable marrow)
267555	GCA_003260385.1	Cuscuta australis (eudicots)
132261	GCA_900332095.1	Cuscuta campestris (field dodder)
36881	GCA_001247695.1	Cymbomonas tetramitiformis (green algae)
59895	GCA_001531365.1	Cynara cardunculus var. scolymus (eudicots)
4509	GCA_002892645.1	Dactylis glomerata (orchard grass)
34297	GCA_003255025.1	Datisca glomerata (eudicots)
79200	GCA_001625215.1	Daucus carota subsp. sativus (carrot)
906689	GCA_001605985.2	Dendrobium catenatum (monocots)
3570	GCA_000512335.1	Dianthus caryophyllus (clove pink)
888268	GCA_001633215.2	Dichanthelium oligosanthes (monocots)
55571	GCA_002904275.2	Dioscorea alata (monocots)
55577	GCA_002240015.2	Dioscorea rotundata (monocots)
55363	GCA_000774125.1	Diospyros lotus (eudicots)
472368	GCA_001598015.1	Dorcoceras hygrometricum (eudicots)
4366	GCA_001925005.1	Drosera capensis (eudicots)
48231	GCA_003254865.1	Dryas drummondii (eudicots)
3046	GCA_002284615.1	Dunaliella salina (green algae)
66656	GCA_002303985.1	Durio zibethinus (durian)
90397	GCA_900205405.1	Echinochloa crus-galli (monocots)
113446	GCA_003412495.1	Echium plantagineum (eudicots)
47281	GCA_001662405.1	Edaphochlamys debaryana (green algae)
44951	GCA_001647135.1	Eichhornia paniculata (monocots)
51953	GCA_000442705.1	Elaeis guineensis (African oil palm)
80265	GCA_000441515.1	Elaeis oleifera (American oil palm)
191504	GCA_002180455.1	Eleusine coracana subsp. coracana (finger millet)

459629	GCA_001753735.1	Embelia ribes (eudicots)
4639	GCA_000818735.2	Ensete ventricosum (monocots)
110835	GCA_000970635.1	Eragrostis tef (tef)
72917	GCA_000775935.1	Erigeron canadensis (horseweed)
4155	GCA_000504015.1	Erythranthe guttata (spotted monkey flower)
222997	GCA_002897215.1	Eschscholzia californica subsp. californica (California poppy)
34316	GCA_000260855.1	Eucalyptus camaldulensis (Murray red gum)
71139	GCA_000612305.1	Eucalyptus grandis (rose gum)
345537	GCA_900116095.1	Euclidium syriacum (eudicots)
593785	GCA_003117195.1	Eudorina sp. 2006-703-Eu-15 (green algae)
3993	GCA_002919075.1	Euphorbia esula (leafy spurge)
347889	GCA_002933915.1	Eutrema heterophyllum (eudicots)
72664	GCA_000478725.1	Eutrema salsugineum (saltwater cress)
347893	GCA_002933935.1	Eutrema yunnanense (eudicots)
3617	GCA_001661195.1	Fagopyrum esculentum (common buckwheat)
62330	GCA_002319775.1	Fagopyrum tataricum (Tartarian buckwheat)
28930	GCA_003347535.1	Fagus sylvatica (European beech)
3494	GCA_002002945.1	Ficus carica (common fig)
48038	GCA_003724115.1	Foeniculum vulgare (fennel)
64939	GCA_000511975.1	Fragaria iinumae (eudicots)
101012	GCA_000512025.1	Fragaria nipponica (eudicots)
60188	GCA_000511995.1	Fragaria nubicola (eudicots)
101013	GCA_000517285.1	Fragaria orientalis (eudicots)
101020	GCA_000184155.1	Fragaria vesca subsp. vesca (wild strawberry)
3747	GCA_000511835.1	Fragaria x ananassa (strawberry)
38873	GCA_900149125.1	Fraxinus excelsior (European ash)
2003741	GCA_002966915.1	Gastrodia elata f. glauca (monocots)
192259	GCA_000441915.1	Genlisea aurea (eudicots)
57919	GCA_900236755.1	Geum urbanum (eudicots)
3847	GCA_000004515.4	Glycine max (soybean)
3848	GCA_002907465.1	Glycine soja (wild soybean)
33097	GCA_001584585.1	Gonium pectorale (green algae)
47615	GCA_002818315.1	Gossypioides kirkii (eudicots)
29729	GCA_000612285.2	Gossypium arboreum (tree cotton)
3634	GCA_001856525.1	Gossypium barbadense (sea-island cotton)
3635	GCA_000987745.1	Gossypium hirsutum (cotton)
29730	GCA_000327365.1	Gossypium raimondii (eudicots)
429701	GCA_002762385.1	Handroanthus impetiginosus (eudicots)
4232	GCA_002127325.1	Helianthus annuus (common sunflower)
1291522	GCA_000690575.1	Helicosporidium sp. ATCC 50920 (green algae)
108875	GCA_002168275.2	Herrania umbratica (eudicots)
3981	GCA_001654055.1	Hevea brasiliensis (rubber tree)
106335	GCA_001696755.1	Hibiscus syriacus (Rose-of-Sharon)
4516	GCA_900070015.1	Hordeum bulbosum (monocots)
112521	GCA_000582825.1	Hordeum pubiflorum (monocots)
4513	GCA_900075435.2	Hordeum vulgare (barley)

1571165	GCA_000831365.1	Humulus lupulus var. lupulus (European hop)
4120	GCA_002525835.2	Ipomoea batatas (sweet potato)
35883	GCA_001879475.1	Ipomoea nil (Japanese morning glory)
35884	GCA_003576665.1	Ipomoea trifida (eudicots)
35885	GCA_003576645.1	Ipomoea triloba (trilobed morning glory)
180498	GCA_000696525.1	Jatropha curcas (eudicots)
91213	GCA_003122765.1	Juglans cathayensis (eudicots)
91216	GCA_003123825.1	Juglans hindsii (eudicots)
91218	GCA_002916435.1	Juglans mandshurica (eudicots)
91219	GCA_003123845.1	Juglans microcarpa (eudicots)
16719	GCA_003123865.1	Juglans nigra (black walnut)
51240	GCA_001411555.1	Juglans regia (English walnut)
224355	GCA_003123805.1	Juglans sigillata (eudicots)
63787	GCA_002312845.1	Kalanchoe fedtschenkoi (South American air plant)
105231	GCA_000708835.1	Klebsormidium nitens (green plants)
47617	GCA_002814295.1	Kokia drynarioides (eudicots)
4236	GCA_002870075.1	Lactuca sativa (eudicots)
3668	GCA_003268545.1	Lagenaria siceraria (white-flowered gourd)
310722	GCA_000411055.1	Leavenworthia alabamica (eudicots)
77586	GCA_000325765.3	Leersia perrieri (monocots)
3414	GCA_003013855.2	Liriodendron chinense (flowering plants)
4522	GCA_001735685.1	Lolium perenne (perennial ryegrass)
153875	GCA_002740545.1	Lophocereus schottii (eudicots)
34305	GCA_000181115.2	Lotus japonicus (eudicots)
3871	GCA_001865875.1	Lupinus angustifolius (narrow-leaved blue lupine)
60698	GCA_900087525.1	Macadamia integrifolia (macadamia nut)
56857	GCA_002174775.1	Macleaya cordata (flowering plants)
86777	GCA_003571905.1	Magnolia ashei (flowering plants)
3750	GCA_000148765.2	Malus domestica (apple)
3983	GCA_001659605.1	Manihot esculenta (cassava)
3197	GCA_003032435.1	Marchantia polymorpha (liverwort)
3880	GCA_000219495.2	Medicago truncatula (barrel medic)
38859	GCA_001642375.1	Mentha longifolia (horsemint)
101978	GCA_001662345.1	Metrosideros polymorpha var. glaberrima ('ohi'a lehua)
554055	GCA_002245815.2	Micractinium conductrix (green algae)
296587	GCA_000090985.2	Micromonas commoda (green algae)
564608	GCA_000151265.1	Micromonas pusilla CCMP1545 (green algae)
1621096	GCA_001430725.1	Micromonas sp. ASP10-01a (green algae)
76306	GCA_003254945.1	Mimosa pudica (sensitive-plant)
183675	GCA_002993905.1	Miscanthus sacchariflorus (Amur silver grass)
3673	GCA_001995035.1	Momordica charantia (bitter melon)
145388	GCA_000611645.1	Monoraphidium neglectum (green algae)
2020292	GCA_002814315.1	Monoraphidium sp. 549 (green algae)
176248	GCA_002855965.1	Monotropa hypopitys (eudicots)
981085	GCA_000414095.2	Morus notabilis (eudicots)
157652	GCA_003370565.1	Mucuna pruriens (eudicots)

214687	GCA_000313855.2	Musa acuminata subsp. malaccensis (wild Malaysian banana)
574487	GCA_001649415.1	Musa itinerans (monocots)
4432	GCA_000365185.2	Nelumbo nucifera (sacred lotus)
49451	GCA_001879085.1	Nicotiana attenuata (eudicots)
4090	GCA_002930595.1	Nicotiana glauca (tree tobacco)
200316	GCA_002018475.1	Nicotiana obtusifolia (eudicots)
4091	GCA_000715115.1	Nicotiana otophora (eudicots)
4096	GCA_000393655.1	Nicotiana sylvestris (wood tobacco)
4097	GCA_000715135.1	Nicotiana tabacum (common tobacco)
4098	GCA_000390325.2	Nicotiana tomentosiformis (eudicots)
105944	GCA_003254905.1	Nissolia schottii (eudicots)
159386	GCA_002091855.1	Nothapodytes nimmoniana (eudicots)
262936	GCA_003254975.1	Ochetophila trinervis (eudicots)
204149	GCA_001278415.1	Ocimum tenuiflorum (holy basil)
158386	GCA_002742605.1	Olea europaea var. sylvestris (wild olive)
1148796	GCA_001182835.1	Oropetium thomaeum (monocots)
65489	GCA_000182155.3	Oryza barthii (African wild rice)
4533	GCA_000231095.2	Oryza brachyantha (malo sina)
4538	GCA_000147395.2	Oryza glaberrima (African rice)
40148	GCA_000576495.1	Oryza glumipatula (monocots)
4528	GCA_001514335.2	Oryza longistaminata (monocots)
40149	GCA_000338895.2	Oryza meridionalis (Australian wild rice)
4537	GCA_000573905.1	Oryza punctata (monocots)
4529	GCA_000817225.1	Oryza rufipogon (monocots)
39947	GCA_001433935.1	Oryza sativa Japonica Group (Japanese rice)
436017	GCA_000092065.1	Ostreococcus lucimarinus CCE9901 (green algae)
70448	GCA_000214015.2	Ostreococcus tauri (green algae)
154437	GCA_002740445.1	Pachycereus pringlei (eudicots)
206008	GCA_002211085.2	Panicum hallii (monocots)
4540	GCA_002895445.2	Panicum miliaceum (monocots)
3469	GCA_003573695.1	Papaver somniferum (opium poppy)
3074	GCA_001598975.1	Parachlorella kessleri (green algae)
3476	GCA_002914805.1	Parasponia andersonii (eudicots)
78168	GCA_002156105.1	Passiflora edulis (passion fruit)
160364	GCA_003313485.1	Penstemon barbatus (eudicots)
69924	GCA_000737435.1	Penstemon centranthifolius (eudicots)
388155	GCA_000737425.1	Penstemon grinnellii (eudicots)
1931378	GCA_002740485.1	Pereskia humboldtii (eudicots)
3435	GCA_002908915.1	Persea americana (avocado)
212056	GCA_003013225.1	Phalaenopsis aphrodite (monocots)
78828	GCA_001263595.1	Phalaenopsis equestris (monocots)
200642	GCA_002079205.1	Phalaenopsis hybrid cultivar (monocots)
412098	GCA_003122825.1	Phaseolus coccineus subsp. coccineus (scarlet runner bean)
3885	GCA_000499845.1	Phaseolus vulgaris (eudicots)
42345	GCA_000413155.1	Phoenix dactylifera (date palm)

3218	GCA_000002425.2	Physcomitrella patens (mosses)
3329	GCA_900067695.1	Picea abies (Norway spruce)
3330	GCA_000411955.5	Picea glauca (white spruce)
1470871	GCA_000876415.1	Picochlorum sp. SENEW3 (green algae)
2053932	GCA_002818215.1	Picochlorum sp. 'soloecismus' (green algae)
2320818	GCA_003665715.1	Picocystis sp. ML (green algae)
3343	GCA_001447015.2	Pinus lambertiana (sugar pine)
3352	GCA_000404065.3	Pinus taeda (loblolly pine)
3888	GCA_003013575.1	Pisum sativum (pea)
28511	GCA_003675935.1	Pogostemon cablin (patchouli)
75702	GCA_000495115.1	Populus euphratica (Euphrates poplar)
3694	GCA_000002775.3	Populus trichocarpa (black cottonwood)
170927	GCA_000788445.1	Primula veris (cowslip)
175104	GCA_001077355.1	Primula vulgaris (eudicots)
2509265	GCA_003612995.1	Prototheca bovis (green algae)
575411	GCA_002897115.1	Prototheca cutis (green algae)
215448	GCA_002794665.1	Prototheca stagnorum (green algae)
3111	GCA_003255715.1	Prototheca wickerhamii (green algae)
42229	GCA_002207925.1	Prunus avium (sweet cherry)
102107	GCA_000346735.1	Prunus mume (Japanese apricot)
3760	GCA_000346465.2	Prunus persica (peach)
2094558	GCA_900382725.1	Prunus yedoensis var. nudiflora (eudicots)
3357	GCA_001517045.1	Pseudotsuga menziesii (Douglas-fir)
120290	GCA_002914565.1	Psidium guajava (guava)
91225	GCA_003123785.1	Pterocarya stenoptera (eudicots)
22663	GCA_002201585.1	Punica granatum (pomegranate)
32243	GCA_003254885.1	Purshia tridentata (bitterbrush)
225117	GCA_000315295.1	Pyrus x bretschneideri (Chinese white pear)
97700	GCA_001633185.1	Quercus lobata (valley oak)
38942	GCA_900291515.1	Quercus robur (eudicots)
58331	GCA_002906115.1	Quercus suber (cork oak)
32244	GCA_003338715.1	Quillaja saponaria (eudicots)
109997	GCA_000769845.1	Raphanus raphanistrum subsp. raphanistrum (eudicots)
3726	GCA_000801105.2	Raphanus sativus (radish)
307507	GCA_003203535.1	Raphidocelis subcapitata (green algae)
396313	GCA_001752375.1	Rhazya stricta (eudicots)
106626	GCA_900174605.1	Rhizophora apiculata (eudicots)
3988	GCA_000151685.2	Ricinus communis (castor bean)
74649	GCA_002994745.1	Rosa chinensis (China rose)
74647	GCA_002564525.1	Rosa multiflora (Japanese rose)
3765	GCA_001662545.1	Rosa x damascena (damask rose)
441023	GCA_001909325.1	Ruellia speciosa (eudicots)
128810	GCA_900465005.1	Saccharum hybrid cultivar (sugarcane)
62335	GCA_003544955.1	Saccharum spontaneum (monocots)
35974	GCA_002911635.1	Santalum album (white sandalwood)
3089	GCA_002317545.1	Scenedesmus quadricauda (green algae)

98039	GCA_000218505.1	Schrenkiella parvula (eudicots)
4550	GCA_900079665.1	Secale cereale (rye)
81964	GCA_001021135.1	Selaginella kraussiana (club-mosses)
88036	GCA_000143415.2	Selaginella moellendorffii (club-mosses)
137178	GCA_003024785.1	Selaginella tamariscina (club-mosses)
4182	GCA_000512975.1	Sesamum indicum (sesame)
4555	GCA_000263155.2	Setaria italica (foxtail millet)
37657	GCA_003260165.1	Silene latifolia (eudicots)
53588	GCA_900538075.1	Silphium perfoliatum (cup-plant)
92921	GCA_001541825.1	Silybum marianum (eudicots)
3730	GCA_000411075.1	Sisymbrium irio (eudicots)
109975	GCA_900188915.1	Solanum americanum (eudicots)
376710	GCA_000612985.1	Solanum arcanum (eudicots)
4109	GCA_001239805.1	Solanum commersonii (Commerson's wild potato)
62890	GCA_000577655.1	Solanum habrochaites (eudicots)
4081	GCA_000188115.3	Solanum lycopersicum (tomato)
4111	GCA_000787875.1	Solanum melongena (eggplant)
28526	GCA_001406875.2	Solanum pennellii (eudicots)
4084	GCA_003660305.1	Solanum pimpinellifolium (currant tomato)
4113	GCA_000226075.1	Solanum tuberosum (potato)
315347	GCA_900185145.1	Solanum verrucosum (eudicots)
4558	GCA_000003195.3	Sorghum bicolor (sorghum)
3562	GCA_002007265.1	Spinacia oleracea (spinach)
29656	GCA_001981405.1	Spirodela polyrhiza (great duckweed)
171973	GCA_002740465.1	Stenocereus thurberi (eudicots)
28532	GCA_000463585.1	Tarenaya hassleriana (spider flower)
47790	GCA_002891735.1	Tetrabaena socialis (green algae)
3088	GCA_900108755.1	Tetradesmus obliquus (green algae)
3641	GCA_000208745.2	Theobroma cacao (cacao)
13288	GCA_000956625.1	Thlaspi arvense (eudicots)
53269	GCA_000818905.1	Trebouxia gelatinosa (green algae)
1917417	GCA_002118135.1	Trebouxia sp. TZW2008 (green algae)
1713842	GCA_003568905.1	Trebouxiophyceae sp. KSI-1 (green algae)
63057	GCA_002914845.1	Trema orientale (eudicots)
97028	GCA_003490085.1	Trifolium medium (eudicots)
57577	GCA_900079335.1	Trifolium pratense (eudicots)
3900	GCA_001742945.1	Trifolium subterraneum (eudicots)
4565	GCA_900519105.1	Triticum aestivum (bread wheat)
85692	GCA_900184675.1	Triticum dicoccoides (wild emmer wheat)
4572	GCA_003073215.1	Triticum urartu (monocots)
498180	GCA_900538255.1	Ulva mutabilis (green algae)
240450	GCA_003016355.1	Urochloa ruziziensis (Congo grass)
13748	GCA_002189035.1	Utricularia gibba (humped bladderwort)
13750	GCA_000775335.2	Vaccinium macrocarpon (eudicots)
3906	GCA_001375635.1	Vicia faba (fava bean)
3914	GCA_001190045.1	Vigna angularis (adzuki bean)

3916	GCA_000741045.2	Vigna radiata var. radiata (mung bean)
3920	GCA_001687525.1	Vigna unguiculata subsp. unguiculata (cowpea)
97446	GCA_002752925.1	Viola pubescens var. scabriuscula (eudicots)
3605	GCA_001562795.1	Vitis aestivalis (eudicots)
266788	GCA_001282645.1	Vitis cinerea x Vitis riparia (eudicots)
29760	GCA_000003745.2	Vitis vinifera (wine grape)
3068	GCA_000143455.1	Volvox carteri f. nagariensis (green algae)
99658	GCA_003430845.1	Xanthoceras sorbifolium (yellow-horn)
90708	GCA_002076135.1	Xerophyta viscosa (monocots)
51707	GCA_003116995.1	Yamagishiella unicocca (green algae)
4577	GCA_000005005.6	Zea mays (maize)
58934	GCA_000418225.1	Zizania latifolia (monocots)
326968	GCA_000826755.1	Ziziphus jujuba (common jujube)
29655	GCA_001185155.1	Zostera marina (monocots)
309978	GCA_001602275.1	Zoysia japonica (monocots)
38722	GCA_001602295.1	Zoysia matrella (Manila grass)
1070856	GCA_001602315.1	Zoysia pacifica (monocots)

Sauropsida

taxid	Acession	Species
57068	GCA_000695815.1	Acanthisitta chloris (rifleman)
451385	GCA_002849675.1	Acridotheres javanicus (Javan myna)
60468	GCA_002631895.1	Agapornis roseicollis (peach-faced lovebird)
8496	GCA_000281125.4	Alligator mississippiensis (American alligator)
38654	GCA_000455745.1	Alligator sinensis (Chinese alligator)
12930	GCA_001420675.1	Amazona aestiva (blue-fronted amazon)
241587	GCA_003947215.1	Amazona collaria (yellow-billed parrot)
241585	GCA_000332375.1	Amazona vittata (Puerto Rican parrot)
8839	GCA_003850225.1	Anas platyrhynchos (mallard)
75864	GCA_002224875.1	Anas zonorhyncha (Eastern spot-billed duck)
28377	GCA_000090745.2	Anolis carolinensis (green anole)
132585	GCA_002592135.1	Anser brachyrhynchus (Pink-footed goose)
381198	GCA_000971095.1	Anser cygnoides domesticus (Swan goose)
279965	GCA_000700745.1	Antrostomus carolinensis (chuck-will's-widow)
57397	GCA_000703405.1	Apaloderma vittatum (bar-tailed trogon)
55534	GCA_000385615.1	Apalone spinifera (spiny softshell turtle)
9233	GCA_000699145.1	Aptenodytes forsteri (emperor penguin)
202946	GCA_001039765.2	Apteryx australis mantelli (brown kiwi)
8823	GCA_003342985.1	Apteryx haastii (Great spotted kiwi)
8824	GCA_003342965.1	Apteryx owenii (little spotted kiwi)
308060	GCA_003343035.1	Apteryx rowi (Okarito brown kiwi)
216574	GCA_000766835.1	Aquila chrysaetos canadensis (golden eagle)
176014	GCA_000400695.1	Ara macao (Scarlet macaw)
194338	GCA_003259725.1	Athene cunicularia (burrowing owl)
100784	GCA_000709895.1	Balearica regulorum gibbericeps (East African grey crowned-crane)
9083	GCA_002909625.1	Bambusicola thoracicus (Chinese bamboo-partridge)
175836	GCA_000710305.1	Buceros rhinoceros silvestris (Rhinoceros hornbill)
198806	GCA_001431845.1	Calidris pugnax (ruff)
425635	GCA_003697955.1	Calidris pygmaea (Spoon-billed sandpiper)
9009	GCA_002218305.1	Callipepla squamata (scaled quail)
9244	GCA_000699085.1	Calypte anna (Anna's hummingbird)
54380	GCA_000690535.1	Cariama cristata (Red-legged seriema)
8787	GCA_003342895.1	Casuarius casuarius (southern cassowary)
43455	GCA_000699945.1	Cathartes aura (turkey vulture)
8897	GCA_000747805.1	Chaetura pelagica (chimney swift)
50402	GCA_000708025.2	Charadrius vociferus (killdeer)
8469	GCA_000344595.1	Chelonia mydas (Green sea turtle)
106734	GCA_003597395.1	Chelonoidis abingdonii (Abingdon island giant tortoise)
187382	GCA_000695195.1	Chlamydotis macqueenii (Macqueen's bustard)
187383	GCA_003400225.1	Chlamydotis undulata undulata (Houbara bustard)
8478	GCA_000241765.2	Chrysemys picta bellii (western painted turtle)
9089	GCA_003413605.1	Chrysolophus pictus (golden pheasant)
36263	GCA_003713305.1	Cicinnurus regius (king bird of paradise)

52775	GCA_002002965.1	Ciconia boyciana (Oriental stork)
9014	GCA_000599465.2	Colinus virginianus (northern bobwhite)
57412	GCA_000690715.1	Colius striatus (speckled mousebird)
8932	GCA_000337935.2	Columba livia (rock pigeon)
415028	GCA_003945725.1	Corapipo altera (White-ruffed manakin)
85066	GCA_000691975.1	Corvus brachyrhynchos (American crow)
932674	GCA_000738735.2	Corvus cornix cornix (hooded crow)
134902	GCA_003402825.1	Corvus hawaiiensis (hawaiian crow)
93934	GCA_001577835.1	Coturnix japonica (Japanese quail)
8502	GCA_001723895.1	Crocodylus porosus (Australian saltwater crocodile)
35024	GCA_001625485.1	Crotalus horridus (timber rattlesnake)
384069	GCA_000737285.1	Crotalus pyrrhus (snakes)
8742	GCA_003400415.2	Crotalus viridis viridis (prairie rattlesnake)
889801	GCA_003342915.1	Crypturellus cinnamomeus (birds)
55661	GCA_000709325.1	Cuculus canorus (common cuckoo)
74909	GCA_004028625.1	Cuora amboinensis (Amboina box turtle)
241437	GCA_003846335.1	Cuora mccordi (McCord's box turtle)
156563	GCA_002901205.1	Cyanistes caeruleus (blue tit)
28716	GCA_003713285.1	Diphyllodes magnificus (birds)
8790	GCA_003342905.1	Dromaius novaehollandiae (emu)
188379	GCA_000687185.1	Egretta garzetta (little egret)
164674	GCA_003031625.1	Empidonax traillii (willow flycatcher)
44318	GCA_003426825.1	Eopsaltria australis (eastern yellow robin)
44316	GCA_003676055.1	Erythrura gouldiae (Gouldian finch)
8805	GCA_003342815.1	Eudromia elegans (elegant crested-tinamou)
54383	GCA_000690775.1	Eurypyga helias (sunbittern)
345164	GCA_000337975.1	Falco cherrug (Saker falcon)
8954	GCA_000337955.1	Falco peregrinus (peregrine falcon)
59894	GCA_000247815.2	Ficedula albicollis (collared flycatcher)
30455	GCA_000690835.1	Fulmarus glacialis (Northern fulmar)
547194	GCA_002003005.1	Gallirallus okinawae (Okinawa rail)
9031	GCA_000002315.5	Gallus gallus (chicken)
37040	GCA_000690875.1	Gavia stellata (red-throated loon)
94835	GCA_001723915.1	Gavialis gangeticus (Gharial)
146911	GCA_001447785.1	Gekko japonicus (lizards)
48883	GCA_000277835.1	Geospiza fortis (medium ground-finch)
38772	GCA_002896415.1	Gopherus agassizii (Agassiz's desert tortoise)
30415	GCA_002002985.1	Grus japonensis (Red-crowned crane)
8969	GCA_000691405.1	Haliaeetus albicilla (white-tailed eagle)
52644	GCA_000737465.1	Haliaeetus leucocephalus (bald eagle)
64799	GCA_003286495.1	Hemignathus virens (Hawaii amakihi)
507594	GCA_003993805.1	Himantopus himantopus leucocephalus (black-winged stilt)
333673	GCA_003692655.1	Hirundo rustica rustica (Barn swallow)
8686	GCA_004023725.1	Hydrophis cyanocinctus (Asian annulated sea snake)
8781	GCA_004023765.1	Hydrophis hardwickii (Hardwick's sea snake)
40217	GCA_003829775.1	Junco hyemalis (dark-eyed junco)

95620	GCA_900245895.1	Lacerta bilineata (lizards)
65476	GCA_900245905.1	Lacerta viridis (green lizard)
321398	GCA_001604755.1	Lepidothrix coronata (blue-crowned manakin)
188344	GCA_000691785.1	Leptosomus discolor (cuckoo roller)
1758121	GCA_002844005.1	Limosa lapponica baueri (bar-tailed godwit)
299123	GCA_002197715.1	Lonchura striata domestica (Bengalese finch)
162951	GCA_000586395.1	Lyrurus tetrix tetrix (black grouse)
391341	GCA_001728815.2	Malaclemys terrapin terrapin (diamondback terrapin)
328815	GCA_001715985.2	Manacus vitellinus (golden-collared manakin)
9103	GCA_000146605.3	Meleagris gallopavo (turkey)
13146	GCA_000238935.1	Melopsittacus undulatus (budgerigar)
57421	GCA_000691845.1	Merops nubicus (carmine bee-eater)
54374	GCA_000695765.1	Mesitornis unicolor (brown roatelo)
1948886	GCA_003546035.1	Mixornis gularis (Striped tit-babbler)
56069	GCA_002173455.1	Nannopterum auritus (double-crested cormorant)
37046	GCA_002174335.1	Nannopterum brasilianus (Neotropic cormorant)
473964	GCA_002173475.1	Nannopterum harrisi (Galapagos flightless cormorant)
114329	GCA_003984885.2	Neopelma chrysocephalum (saffron-crested tyrant-manakin)
176057	GCA_000696875.1	Nestor notabilis (Kea)
128390	GCA_000708225.1	Nipponia nippon (crested ibis)
8663	GCA_900518725.1	Notechis scutatus (mainland tiger snake)
30464	GCA_003342845.1	Nothoprocta perdicaria (birds)
8996	GCA_002078875.2	Numida meleagris (helmeted guineafowl)
8665	GCA_000516915.1	Ophiophagus hannah (king cobra)
30419	GCA_000692075.1	Opisthocomus hoazin (hoatzin)
94885	GCA_001185365.1	Pantherophis guttatus (snakes)
36268	GCA_003713265.1	Paradisaea raggiana (Raggiana bird of paradise)
39963	GCA_003713215.1	Paradisaea rubra (red bird of paradise)
143630	GCA_003118565.1	Paroedura picta (lizards)
39961	GCA_003713295.1	Parotia lawesii (Lawes's parotia)
9157	GCA_001522545.2	Parus major (Great Tit)
48849	GCA_001700915.1	Passer domesticus (House sparrow)
372326	GCA_002029285.1	Patagioenas fasciata monilis (band-tailed pigeon)
36300	GCA_000687375.1	Pelecanus crispus (Dalmatian pelican)
13735	GCA_000230535.1	Pelodiscus sinensis (Chinese soft-shelled turtle)
97097	GCA_000687285.1	Phaethon lepturus (White-tailed tropicbird)
9209	GCA_000708925.1	Phalacrocorax carbo (great cormorant)
9054	GCA_004143745.1	Phasianus colchicus (Ring-necked pheasant)
9218	GCA_000687265.1	Phoenicopterus ruber ruber (American flamingo)
56441	GCA_001655115.1	Phylloscopus plumbeitarsus (birds)
144762	GCA_001655095.1	Phylloscopus trochiloides viridanus (greenish warbler)
209779	GCA_002305835.1	Phylloscopus trochilus acredula (Willow warbler)
118200	GCA_000699005.1	Picoides pubescens (Downy woodpecker)
649802	GCA_003945595.1	Pipra filicauda (Wire-tailed manakin)

55544	GCA_003942145.1	Platysternon megacephalum (big-headed turtle)
345573	GCA_000699545.1	Podiceps cristatus (great crested grebe)
103695	GCA_900067755.1	Pogona vitticeps (central bearded dragon)
88087	GCA_003402635.1	Protobothrops flavoviridis (habu)
103944	GCA_001527695.3	Protobothrops mucrosquamatus (snakes)
8673	GCA_900518735.1	Pseudonaja textilis (eastern brown snake)
181119	GCA_000331425.1	Pseudopodoces humilis (Tibetan ground-tit)
9228	GCA_002870145.1	Psittacula krameri (Rose-ringed parakeet)
240206	GCA_000699245.1	Pterocles gutturalis (yellow-throated sandgrouse)
8795	GCA_003342835.1	Pterocnemia pennata (lesser rhea)
9238	GCA_000699105.1	Pygoscelis adeliae (Adelie penguin)
79643	GCA_003264595.1	Pygoscelis antarcticus (chinstrap penguin)
30457	GCA_003264615.1	Pygoscelis papua (Gentoo penguin)
176946	GCA_000186305.2	Python bivittatus (Burmese python)
171275	GCA_004023745.1	Recurvirostra avosetta (Pied Avocet)
8797	GCA_003343005.1	Rhea americana (greater rhea)
96440	GCA_003586115.2	Salvator merianae (Argentine black and white tegu)
1187130	GCA_900205225.1	Saxicola maurus maurus (Siberian stonechat)
9135	GCA_000534875.1	Serinus canaria (Common canary)
1037456	GCA_001746935.1	Setophaga coronata coronata (myrtle warbler)
9240	GCA_003264545.1	Spheniscus humboldti (Humboldt's penguin)
37081	GCA_003264715.1	Spheniscus magellanicus (Magellanic penguin)
156760	GCA_003264655.1	Spheniscus mendiculus (Galapagos penguin)
8508	GCA_003113815.1	Sphenodon punctatus (tuatara)
256693	GCA_002167245.1	Sporophila hypoxantha (tawny-bellied seedeater)
2489341	GCA_004011185.1	Strigops habroptila (Kakapo)
311401	GCA_002372975.1	Strix occidentalis caurina (northern spotted owl)
441894	GCA_000698965.1	Struthio camelus australis (African ostrich)
9172	GCA_001447265.1	Sturnus vulgaris (Common starling)
9065	GCA_003435085.1	Syrmaticus mikado (Mikado pheasant)
59729	GCA_000151805.2	Taeniopygia guttata (zebra finch)
121530	GCA_000709365.1	Tauraco erythrolophus (red-crested turaco)
2587831	GCA_002925995.2	Terrapene carolina triunguis (Three-toed box turtle)
35019	GCA_001077635.2	Thamnophis sirtalis (snakes)
527827	GCA_003457575.1	Thermophis baileyi (snakes)
94827	GCA_000705375.2	Tinamus guttatus (white-throated tinamou)
211916	GCA_001870855.1	Tympanuchus cupido pinnatus (greater prairie chicken)
56313	GCA_000687205.1	Tyto alba (Barn owl)
28711	GCA_002289315.1	Uria lomvia (thick-billed guillemot)
56070	GCA_002173435.1	Urile pelagicus (pelagic cormorant)
31156	GCA_000800605.1	Vipera berus berus (common viper)
44394	GCA_000385455.1	Zonotrichia albicollis (white-throated sparrow)
1220523	GCA_001281735.1	Zosterops lateralis melanops (silver-eye)

Mammalia

taxid	Acession	Species
32536	GCA_003709585.1	Acinonyx jubatus (cheetah)
10068	GCA_004027535.1	Acomys cahirinus (Egyptian spiny mouse)
9646	GCA_000004335.1	Ailuropoda melanoleuca (giant panda)
424585	GCA_002007465.1	Ailurus fulgens styani (lesser panda)
1041416	GCA_004027895.1	Allactaga bullata (Gobi jerboa)
30589	GCA_004027835.1	Alouatta palliata (mantled howler monkey)
9899	GCA_002201775.1	Ammotragus lervia (aoudad)
27642	GCA_004027475.1	Anoura caudifer (tailed tailless bat)
9891	GCA_004027515.1	Antilocapra americana (pronghorn)
37293	GCA_000952055.2	Aotus nancymaae (Ma's night monkey)
51342	GCA_004027875.1	Aplodontia rufa (mountain beaver)
105296	GCA_002335545.1	Apodemus speciosus (large Japanese field mouse)
10129	GCA_001305905.1	Apodemus sylvaticus (European woodmouse)
37190	GCA_900642305.1	Arctocephalus gazella (antarctic fur seal)
9417	GCA_004027435.1	Artibeus jamaicensis (Jamaican fruit-eating bat)
9509	GCA_004024785.1	Ateles geoffroyi (black-handed spider monkey)
57737	GCA_003798545.1	Axis porcinus (Hog deer)
310752	GCA_000493695.1	Balaenoptera acutorostrata scammoni (minke whale)
33556	GCA_000978805.1	Balaenoptera bonaerensis (Antarctic minke whale)
59527	GCA_004027495.1	Beatragus hunteri (even-toed ungulates)
43346	GCA_000754665.1	Bison bison bison (American bison)
9915	GCA_000247795.2	Bos indicus (zebu cattle)
30522	GCA_003369695.2	Bos indicus x Bos taurus (hybrid cattle)
72004	GCA_000298355.1	Bos mutus (wild yak)
9913	GCA_002263795.2	Bos taurus (cattle)
9355	GCA_004027775.1	Bradypus variegatus (Brown-throated sloth)
89462	GCA_003121395.1	Bubalus bubalis (water buffalo)
9483	GCA_000004665.1	Callithrix jacchus (white-tufted-ear marmoset)
34884	GCA_003265705.1	Callorhinus ursinus (northern fur seal)
9837	GCA_000767855.1	Camelus bactrianus (Bactrian camel)
9838	GCA_000767585.1	Camelus dromedarius (Arabian camel)
419612	GCA_000311805.2	Camelus ferus (Wild Bactrian camel)
286419	GCA_003254725.1	Canis lupus dingo (dingo)
9615	GCA_000002285.2	Canis lupus familiaris (dog)
9923	GCA_000978405.1	Capra aegagrus (wild goat)
9925	GCA_001704415.1	Capra hircus (goat)
72544	GCA_003182615.2	Capra sibirica (Siberian ibex)
9858	GCA_000751575.1	Capreolus capreolus (Western roe deer)
34842	GCA_004027915.1	Capromys pilorides (Desmarest's hutia)
1868482	GCA_000164805.2	Carlito syrichta (Philippine tarsier)
40233	GCA_004027735.1	Carollia perspicillata (Seba's short-tailed bat)
51338	GCA_001984765.1	Castor canadensis (American beaver)
51154	GCA_004024745.1	Catagonus wagneri (Chacoan peccary)
37548	GCA_000688575.1	Cavia aperea (Brazilian guinea pig)

10141	GCA_000151735.1	Cavia porcellus (domestic guinea pig)
143287	GCA_004027695.1	Cavia tschudii (Montane guinea pig)
9514	GCA_004027755.1	Cebus albifrons (white-fronted capuchin)
1737458	GCA_001604975.1	Cebus capucinus imitator (white-faced sapajou)
73337	GCA_000283155.1	Ceratotherium simum simum (southern white rhinoceros)
9531	GCA_000955945.1	Cercocebus atys (sooty mangabey)
36227	GCA_004027615.1	Cercopithecus neglectus (De Brazza's monkey)
46360	GCA_002197005.1	Cervus elaphus hippelaphus (red deer)
340076	GCA_004027955.1	Chaetophractus vellerosus (Screaming hairy armadillo)
9460	GCA_004024725.1	Cheirogaleus medius (Lesser dwarf lemur)
34839	GCA_000276665.1	Chinchilla lanigera (long-tailed chinchilla)
60711	GCA_000409795.2	Chlorocebus sabaeus (green monkey)
27675	GCA_004027855.1	Choloepus didactylus (southern two-toed sloth)
9358	GCA_000164785.2	Choloepus hoffmanni (Hoffmann's two-fingered sloth)
185453	GCA_000296735.1	Chrysochloris asiatica (Cape golden mole)
336983	GCA_000951035.1	Colobus angolensis palliatus (Angolan colobus)
143302	GCA_000260355.1	Condylura cristata (star-nosed mole)
208972	GCA_004027555.1	Craseonycteris thonglongyai (hog-nosed bat)
10085	GCA_004027575.1	Cricetomys gambianus (Gambian giant pouched rat)
10029	GCA_000223135.1	Cricetulus griseus (Chinese hamster)
876679	GCA_004027635.1	Crocidura indochinensis (insectivores)
94188	GCA_004023885.1	Cryptoprocta ferox (fossa)
10166	GCA_004027205.1	Ctenodactylus gundi (northern gundi)
43321	GCA_004027165.1	Ctenomys sociabilis (social tuco-tuco)
9361	GCA_000208655.2	Dasypus novemcinctus (nine-banded armadillo)
31869	GCA_004027145.1	Daubentonia madagascariensis (aye-aye)
9749	GCA_002288925.2	Delphinapterus leucas (beluga whale)
9430	GCA_002940915.2	Desmodus rotundus (common vampire bat)
310712	GCA_002844835.1	Dicerorhinus sumatrensis sumatrensis (Sumatran rhinoceros)
9805	GCA_004027315.1	Diceros bicornis (black rhinoceros)
108858	GCA_004027595.1	Dinomys branickii (pacarana)
10020	GCA_000151885.2	Dipodomys ordii (Ord's kangaroo rat)
323379	GCA_004024685.1	Dipodomys stephensi (Stephens's kangaroo rat)
29091	GCA_004027295.1	Dolichotis patagonum (Patagonian cavy)
9371	GCA_000313985.1	Echinops telfairi (small Madagascar hedgehog)
77214	GCA_000465285.1	Eidolon helvum (straw-colored fruit bat)
43332	GCA_002443075.1	Elaphurus davidianus (Pere David's deer)
28737	GCA_000299155.1	Elephantulus edwardii (Cape elephant shrew)
39086	GCA_001685075.1	Ellobius lutescens (Transcaucasian mole vole)
329620	GCA_001685095.1	Ellobius talpinus (Northern mole vole)
391180	GCA_002288905.2	Enhydra lutris kenyoni (sea otter)
58065	GCA_003508835.1	Eonycteris spelaea (lesser dawn bat)
29078	GCA_000308155.1	Eptesicus fuscus (big brown bat)
9793	GCA_001305755.1	Equus asinus (ass)
9796	GCA_002863925.1	Equus caballus (horse)
9798	GCA_000696695.1	Equus przewalskii (Przewalski's horse)

9365	GCA_000296755.1	Erinaceus europaeus (western European hedgehog)
9538	GCA_004027335.1	Erythrocebus patas (red guenon)
9764	GCA_002189225.1	Eschrichtius robustus (grey whale)
87288	GCA_001262665.1	Eulemur flavifrons (Sclater's lemur)
13515	GCA_004027275.1	Eulemur fulvus (brown lemur)
30602	GCA_001262655.1	Eulemur macaco (black lemur)
34886	GCA_004028035.1	Eumetopias jubatus (Steller sea lion)
9685	GCA_000181335.4	Felis catus (domestic cat)
61379	GCA_004023925.1	Felis nigripes (black-footed cat)
885580	GCA_000743615.1	Fukomys damarensis (Damara mole-rat)
482537	GCA_000696425.1	Galeopterus variegatus (Sunda flying lemur)
439328	GCA_001651235.1	Giraffa tippelskirchi (even-toed ungulates)
41261	GCA_004027185.1	Glis glis (Fat dormouse)
9595	GCA_000151905.3	Gorilla gorilla gorilla (western lowland gorilla)
51346	GCA_004027655.1	Graphiurus murinus (woodland dormouse)
48420	GCA_900006375.2	Gulo gulo (wolverine)
210647	GCA_004023845.1	Helogale parvula (dwarf mongoose)
330464	GCA_004026825.1	Hemitragus hylocrius (Nilgiri tahr)
10181	GCA_000247695.1	Heterocephalus glaber (naked mole-rat)
77598	GCA_004026845.1	Heterohyrax brucei (yellow-spotted hyrax)
9833	GCA_002995585.1	Hippopotamus amphibius (hippopotamus)
186990	GCA_001890085.1	Hipposideros armiger (great roundleaf bat)
58069	GCA_004027415.1	Hipposideros galeritus (Cantor's roundleaf bat)
9606	GCA_000001405.27	Homo sapiens (human)
95912	GCA_003009895.1	Hyaena hyaena (striped hyena)
10149	GCA_004027455.1	Hydrochoerus hydrochaeris (capybara)
10137	GCA_004026905.1	Hystrix cristata (crested porcupine)
43179	GCA_000236235.1	Ictidomys tridecemlineatus (thirteen-lined ground squirrel)
51337	GCA_000280705.1	Jaculus jaculus (lesser Egyptian jerboa)
90247	GCA_003676395.1	Lagenorhynchus obliquidens (Pacific white-sided dolphin)
258930	GCA_004026805.1	Lasiurus borealis (red bat)
9447	GCA_004024665.1	Lemur catta (Ring-tailed lemur)
9713	GCA_000349705.1	Leptonychotes weddellii (Weddell seal)
48086	GCA_004026855.1	Lepus americanus (snowshoe hare)
118797	GCA_000442215.1	Lipotes vexillifer (Yangtze River dolphin)
9785	GCA_000001905.1	Loxodonta africana (African savanna elephant)
9622	GCA_001887905.1	Lycaon pictus (African hunting dog)
9541	GCA_000364345.1	Macaca fascicularis (crab-eating macaque)
9543	GCA_003118495.1	Macaca fuscata fuscata (Japanese macaque)
9544	GCA_000772875.3	Macaca mulatta (Rhesus monkey)
9545	GCA_000956065.1	Macaca nemestrina (pig-tailed macaque)
326083	GCA_004027375.1	Macroglossus sobrinus (bats)
9315	GCA_000004035.1	Macropus eugenii (tammar wallaby)
9568	GCA_000951045.1	Mandrillus leucophaeus (drill)
9974	GCA_001685135.1	Manis javanica (Malayan pangolin)

143292	GCA_000738955.1	Manis pentadactyla (Chinese pangolin)
93162	GCA_003676075.1	Marmota flaviventris (yellow-bellied marmot)
9994	GCA_001458135.1	Marmota marmota marmota (Alpine marmot)
9413	GCA_004026885.1	Megaderma lyra (Indian false vampire)
9664	GCA_004024625.1	Mellivora capensis (ratel)
10047	GCA_002204375.1	Meriones unguiculatus (Mongolian gerbil)
10036	GCA_000349665.1	Mesocricetus auratus (golden hamster)
48745	GCA_004027085.1	Mesoplodon bidens (Sowerby's beaked whale)
30608	GCA_000165445.3	Microcebus murinus (gray mouse lemur)
176115	GCA_004026705.1	Microgale talazaci (Talazac's shrew tenrec)
148065	GCA_004026765.1	Micronycteris hirsuta (bats)
29092	GCA_001305995.1	Microtus agrestis (short-tailed field vole)
79684	GCA_000317375.1	Microtus ochrogaster (prairie vole)
291302	GCA_001595765.1	Miniopterus natalensis (bats)
9433	GCA_004026525.1	Miniopterus schreibersii (Schreibers' long-fingered bat)
9716	GCA_004023865.1	Mirounga angustirostris (Northern elephant seal)
47180	GCA_004024645.1	Mirza coquereli (Coquerel's mouse lemur)
13616	GCA_000002295.1	Monodelphis domestica (gray short-tailed opossum)
40151	GCA_004027045.1	Monodon monoceros (narwhal)
118852	GCA_004026545.1	Mormoops blainvillei (Antillean ghost-faced bat)
68415	GCA_004024705.1	Moschus moschiferus (Siberian musk deer)
210652	GCA_004023785.1	Mungos mungo (banded mongoose)
1453894	GCA_004026665.1	Murina aurata feae (Little tube-nosed bat)
10089	GCA_900094665.2	Mus caroli (Ryukyu mouse)
10090	GCA_000001635.8	Mus musculus (house mouse)
10093	GCA_900095145.2	Mus pahari (shrew mouse)
10103	GCA_003336285.1	Mus spicilegus (steppe mouse)
10096	GCA_001624865.1	Mus spretus (western wild mouse)
39082	GCA_004027005.1	Muscardinus avellanarius (hazel dormouse)
9669	GCA_000215625.1	Mustela putorius furo (domestic ferret)
10157	GCA_004027025.1	Myocastor coypus (nutria)
447135	GCA_001305785.1	Myodes glareolus (Bank vole)
109478	GCA_000412655.1	Myotis brandtii (Brandt's bat)
225400	GCA_000327345.1	Myotis davidii (bats)
59463	GCA_000147115.1	Myotis lucifugus (little brown bat)
51298	GCA_004026985.1	Myotis myotis (bats)
71006	GCA_004026745.1	Myrmecophaga tridactyla (giant anteater)
1026970	GCA_000622305.1	Nannospalax galili (Upper Galilee mountains blind mole rat)
43780	GCA_000772465.1	Nasalis larvatus (proboscis monkey)
29088	GCA_002201575.1	Neomonachus schauinslandi (Hawaiian monk seal)
1706337	GCA_003031525.1	Neophocaena asiaeorientalis asiaeorientalis (Yangtze finless porpoise)
56216	GCA_001675575.1	Neotoma lepida (desert woodrat)
452646	GCA_900108605.1	Neovison vison (American mink)
94963	GCA_004026585.1	Noctilio leporinus (greater bulldog bat)
61853	GCA_000146795.3	Nomascus leucogenys (northern white-cheeked gibbon)

9470	GCA_004027815.1	Nycticebus coucang (slow loris)
9978	GCA_000292845.1	Ochotona princeps (American pika)
10160	GCA_000260255.1	Octodon degus (degu)
135583	GCA_002564305.1	Octomys mimax (viscacha rat)
9708	GCA_000321225.1	Odobenus rosmarus divergens (Pacific walrus)
9877	GCA_004115125.1	Odocoileus hemionus hemionus (mule deer)
9880	GCA_002102435.1	Odocoileus virginianus texanus (white-tailed deer)
86973	GCA_001660835.1	Okapia johnstoni (okapi)
10060	GCA_004026605.1	Ondatra zibethicus (muskrat)
38674	GCA_004026725.1	Onychomys torridus (southern grasshopper mouse)
9733	GCA_000331955.2	Orcinus orca (killer whale)
9258	GCA_000002275.2	Ornithorhynchus anatinus (platypus)
1230840	GCA_000298275.1	Orycteropus afer afer (aardvark)
9986	GCA_000003625.1	Oryctolagus cuniculus (rabbit)
9958	GCA_003945745.1	Oryx gazella (gemsbok)
30611	GCA_000181295.3	Otolemur garnettii (small-eared galago)
30527	GCA_003121645.1	Ovis ammon (argali)
9940	GCA_002742125.1	Ovis aries (sheep)
37174	GCA_004026945.1	Ovis canadensis (bighorn sheep)
9597	GCA_000258655.2	Pan paniscus (pygmy chimpanzee)
9598	GCA_002880755.3	Pan troglodytes (chimpanzee)
9690	GCA_004023805.1	Panthera onca (jaguar)
9691	GCA_001857705.1	Panthera pardus (leopard)
74533	GCA_000464555.1	Panthera tigris altaica (Amur tiger)
59538	GCA_000400835.1	Pantholops hodgsonii (chiru)
9555	GCA_000264685.2	Papio anubis (olive baboon)
71117	GCA_004024585.1	Paradoxurus hermaphroditus (Asian palm civet)
230844	GCA_000500345.1	Peromyscus maniculatus bairdii (prairie deer mouse)
369710	GCA_003704135.1	Peromyscus polionotus subgriseus (oldfield mouse)
10183	GCA_004026965.1	Petromus typicus (dassie-rat)
38626	GCA_002099425.1	Phascolarctos cinereus (koala)
9742	GCA_003071005.1	Phocoena phocoena (harbor porpoise)
10044	GCA_001707965.1	Phodopus sungorus (rodents)
89673	GCA_004126475.1	Phyllostomus discolor (pale spear-nosed bat)
9755	GCA_002837175.1	Physeter catodon (sperm whale)
591936	GCA_002776525.2	Piliocolobus tephrosceles (Ugandan red Colobus)
59474	GCA_004026625.1	Pipistrellus pipistrellus (common pipistrelle)
43777	GCA_004026645.1	Pithecia pithecia (white-faced saki)
230833	GCA_004027715.1	Plecturocebus donacophilus (Bolivian titi)
9601	GCA_002880775.3	Pongo abelii (Sumatran orangutan)
9600	GCA_900086635.1	Pongo pygmaeus (Bornean orangutan)
9813	GCA_000152225.2	Procavia capensis (Cape rock hyrax)
1328070	GCA_003258685.1	Prolemur simus (greater bamboo lemur)
379532	GCA_000956105.1	Propithecus coquereli (Coquerel's sifaka)
48139	GCA_002215935.1	Psammomys obesus (fat sand rat)
59542	GCA_003182575.1	Pseudois nayaur (bharal)

59476	GCA_000465405.1	Pteronotus parnellii (Parnell's mustached bat)
9672	GCA_004024605.1	Pteronura brasiliensis (giant otter)
9402	GCA_000325575.1	Pteropus alecto (black flying fox)
132908	GCA_000151845.2	Pteropus vampyrus (large flying fox)
9696	GCA_003327715.1	Puma concolor (puma)
54133	GCA_004024825.1	Pygathrix nemaeus (Red shanked douc langur)
9870	GCA_004026565.1	Rangifer tarandus (reindeer)
10116	GCA_000001895.4	Rattus norvegicus (Norway rat)
59479	GCA_004115295.1	Rhinolophus ferrumequinum (greater horseshoe bat)
59479	GCA_004115265.2	Rhinolophus ferrumequinum (greater horseshoe bat)
59479	GCA_004115265.1	Rhinolophus ferrumequinum (greater horseshoe bat)
61621	GCA_001698545.1	Rhinopithecus bieti (black snub-nosed monkey)
61622	GCA_000769185.1	Rhinopithecus roxellana (golden snub-nosed monkey)
53275	GCA_004026225.1	Rhizomys pruinosus (hoary bamboo rat)
9407	GCA_001466805.2	Rousettus aegyptiacus (Egyptian rousette)
9491	GCA_004024885.1	Saguinus imperator (tamarin)
34875	GCA_004024985.1	Saiga tatarica (even-toed ungulates)
39432	GCA_000235385.1	Saimiri boliviensis boliviensis (Bolivian squirrel monkey)
9305	GCA_000189315.1	Sarcophilus harrisii (Tasmanian devil)
71119	GCA_004024925.1	Scalopus aquaticus (eastern mole)
88029	GCA_004025065.1	Semnopithecus entellus (Hanuman langur)
42415	GCA_004025045.1	Sigmodon hispidus (hispid cotton rat)
1906353	GCA_002901085.1	Solenodon paradoxus woodi (Hispaniolan solenodon)
42254	GCA_000181275.2	Sorex araneus (European shrew)
103600	GCA_003521335.2	Sousa chinensis (Indo-pacific humpbacked dolphin)
99837	GCA_002406435.1	Spermophilus dauricus (Daurian ground squirrel)
30551	GCA_004023965.1	Spilogale gracilis (western spotted skunk)
37032	GCA_004023905.1	Suricata suricatta (meerkat)
9823	GCA_000003025.6	Sus scrofa (pig)
9438	GCA_004025005.1	Tadarida brasiliensis (Brazilian free-tailed bat)
48850	GCA_004025105.1	Tamandua tetradactyla (southern tamandua)
9802	GCA_004024905.1	Tapirus indicus (Asiatic tapir)
9801	GCA_004025025.1	Tapirus terrestris (Brazilian tapir)
2282171	GCA_003697995.1	Taxidea taxus jeffersonii (American badger)
9565	GCA_003255815.1	Theropithecus gelada (gelada)
10169	GCA_004025085.1	Thryonomys swinderianus (Greater cane rat)
183749	GCA_004025125.1	Tolypeutes matacus (placentals)
171122	GCA_004024845.1	Tonatia saurophila (stripe-headed round-eared bat)
9849	GCA_004024965.1	Tragulus javanicus (Java mouse-deer)
127582	GCA_000243295.1	Trichechus manatus latirostris (Florida manatee)
37347	GCA_000181375.1	Tupaia belangeri (northern tree shrew)
246437	GCA_000334495.1	Tupaia chinensis (Chinese tree shrew)
79784	GCA_003227395.1	Tursiops aduncus (Indo-pacific bottlenose dolphin)
9739	GCA_001922835.1	Tursiops truncatus (common bottlenose dolphin)
61882	GCA_002564285.1	Tympanoctomys barrerae (plains viscacha rat)
9999	GCA_003426925.1	Urocitellus parryii (Arctic ground squirrel)

182669	GCA_004024945.1	Uropsilus gracilis (gracile shrew mole)
9643	GCA_003344425.1	Ursus americanus (American black bear)
116960	GCA_003584765.1	Ursus arctos horribilis (brown bear)
29073	GCA_000687225.1	Ursus maritimus (polar bear)
30538	GCA_000164845.3	Vicugna pacos (alpaca)
29139	GCA_900497805.2	Vombatus ursinus (common wombat)
494514	GCA_004023825.1	Vulpes lagopus (Arctic fox)
9627	GCA_003160815.1	Vulpes vulpes (red fox)
234690	GCA_004024805.1	Xerus inauris (South African ground squirrel)
9704	GCA_900631625.1	Zalophus californianus (California sea lion)
160400	GCA_004024765.1	Zapus hudsonius (meadow jumping mouse)

Phatyhelminthes

taxid	Acession	Species
79923	GCA_003604175.1	Clonorchis sinensis (oriental liver fluke)
57078	GCA_000950715.1	Dicrocoelium dendriticum (flatworms)
6161	GCA_001938525.1	Dugesia japonica (flatworms)
6210	GCA_000524195.1	Echinococcus granulosus (flatworms)
6211	GCA_000469725.3	Echinococcus multilocularis (flatworms)
46835	GCA_002867515.1	Fasciola gigantica (flatworms)
6192	GCA_900302435.1	Fasciola hepatica (liver fluke)
6162	GCA_001938485.1	Girardia tigrina (flatworms)
37629	GCA_000715275.1	Gyrodactylus salaris (gyrodactylosis fluke)
85433	GCA_000469805.2	Hymenolepis microstoma (flatworms)
282301	GCA_002269645.1	Macrostomum lignano (flatworms)
6198	GCA_000715545.1	Opisthorchis viverrini (Southeast Asian liver fluke)
6185	GCA_000699445.1	Schistosoma haematobium (flatworms)
6182	GCA_000151775.1	Schistosoma japonicum (flatworms)
6183	GCA_000237925.2	Schistosoma mansoni (flatworms)
79327	GCA_002600895.1	Schmidtea mediterranea (freshwater planarian)
99802	GCA_000951995.1	Spirometra erinaceieuropaei (flatworms)
60517	GCA_001693035.2	Taenia asiatica (Asian tapeworm)
94034	GCA_001923025.3	Taenia multiceps (flatworms)
6206	GCA_001693075.2	Taenia saginata (beef tapeworm)
6204	GCA_001870725.1	Taenia solium (pork tapeworm)

Protostomia

taxid	Acession	Species
575412	GCA_000661875.1	Acanthoscurria geniculata (spiders)
136180	GCA_900241095.1	Acartia tonsa (crustaceans)
229769	GCA_000988765.1	Achipteria coleoptrata (mites & ticks)
290746	GCA_900406225.1	Acrobeloides nanus (nematodes)
103372	GCA_000204515.1	Acromyrmex echinatior (Panamanian leafcutter ant)
7029	GCA_000142985.2	Acyrthosiphon pisum (pea aphid)
249248	GCA_900240375.1	Adineta ricciae (rotifers)
104782	GCA_000513175.1	Adineta vaga (rotifers)
7159	GCA_002204515.1	Aedes aegypti (yellow fever mosquito)
7160	GCA_001876365.2	Aedes albopictus (Asian tiger mosquito)
116153	GCA_001937115.1	Aethina tumida (small hive beetle)
224129	GCA_000699045.2	Agrilus planipennis (emerald ash borer)
135921	GCA_003054995.1	Aleochara bilineata (beetles)
143724	GCA_001676325.1	Amphinemura sulcicollis (stoneflies)
680683	GCA_001186105.1	Amyelois transitella (moths)
351238	GCA_900184025.1	Amynthas corticis (segmented worms)
29170	GCA_003336725.1	Ancylostoma caninum (dog hookworm)
53326	GCA_000688135.1	Ancylostoma ceylanicum (nematodes)
51022	GCA_000816745.1	Ancylostoma duodenale (nematodes)
75152	GCA_900474205.1	Andricus curvator (wasps, ants, and bees)
75169	GCA_900474195.1	Andricus grossulariae (wasps, ants, and bees)
75162	GCA_900474215.1	Andricus inflator (wasps, ants, and bees)
199937	GCA_900474265.1	Andricus quercusramuli (wasps, ants, and bees)
6313	GCA_001884285.1	Angiostrongylus cantonensis (rat lungworm)
7167	GCA_000349125.2	Anopheles albimanus (mosquitos)
42839	GCA_002846955.1	Anopheles aquasalis (mosquitos)
7173	GCA_000349185.1	Anopheles arabiensis (mosquitos)
41427	GCA_000473505.1	Anopheles atroparvus (mosquitos)
43041	GCA_000349165.1	Anopheles christyi (mosquitos)
1518534	GCA_000150765.1	Anopheles coluzzii (mosquitos)
123217	GCA_002091845.1	Anopheles cracens (mosquitos)
139723	GCA_000473375.1	Anopheles culicifacies (mosquitos)
43151	GCA_000211455.3	Anopheles darlingi (American malaria mosquito)
7168	GCA_000349145.1	Anopheles dirus (mosquitos)
199890	GCA_000349105.1	Anopheles epiroticus (mosquitos)
69004	GCA_000473445.2	Anopheles farauti (mosquitos)
59191	GCA_000956215.1	Anopheles farauti No. 4 (mosquitos)
62324	GCA_000349085.1	Anopheles funestus (African malaria mosquito)
180454	GCA_000005575.1	Anopheles gambiae str. PEST (African malaria mosquito)
30065	GCA_000956275.1	Anopheles koliensis (mosquitos)
74869	GCA_002091835.1	Anopheles maculatus (mosquitos)
34690	GCA_000473525.2	Anopheles melas (mosquitos)
30066	GCA_000473845.2	Anopheles merus (mosquitos)
112268	GCA_000349025.1	Anopheles minimus (mosquitos)

185578	GCA_000439205.1	Anopheles nili (mosquitos)
30068	GCA_000956255.1	Anopheles punctulatus (mosquitos)
34691	GCA_000349065.1	Anopheles quadriannulatus (mosquitos)
74873	GCA_000441895.2	Anopheles sinensis (mosquitos)
30069	GCA_003448975.1	Anopheles stephensi (Asian malaria mosquito)
217634	GCA_000390285.2	Anoplophora glabripennis (Asian longhorned beetle)
602585	GCA_003063805.1	Aphaenogaster ashmeadi (ants)
602633	GCA_003063835.1	Aphaenogaster floridana (ants)
602636	GCA_003063765.1	Aphaenogaster fulva (ants)
602682	GCA_003063725.1	Aphaenogaster miamiana (ants)
602698	GCA_003063865.1	Aphaenogaster picea (ants)
500656	GCA_003063815.1	Aphaenogaster rudis (ants)
7461	GCA_001442555.1	Apis cerana (Asiatic honeybee)
7462	GCA_000469605.1	Apis dorsata (giant honeybee)
7463	GCA_000184785.1	Apis florea (little honeybee)
7460	GCA_003254395.2	Apis mellifera (honey bee)
6500	GCA_000002075.2	Aplysia californica (California sea hare)
13347	GCA_001887335.1	Armadillidium vulgare (common pillbug)
6253	GCA_000187025.3	Ascaris suum (pig roundworm)
37344	GCA_000344095.2	Athalia rosae (coleseed sawfly)
12957	GCA_000143395.2	Atta cephalotes (ants)
520822	GCA_001594045.1	Atta colombica (ants)
27457	GCA_000789215.2	Bactrocera dorsalis (oriental fruit fly)
174628	GCA_001853355.1	Bactrocera latifrons (flies)
104688	GCA_001188975.2	Bactrocera oleae (olive fruit fly)
59916	GCA_000695345.1	Bactrocera tryoni (Queensland fruit fly)
189839	GCA_001676355.1	Baetis rhodani (mayflies)
693219	GCA_001922985.1	Bankia setacea (bivalves)
220390	GCA_002080005.1	Bathymodiolus platifrons (bivalves)
315563	GCA_000775305.1	Belgica antarctica (flies)
7038	GCA_001854935.1	Bemisia tabaci (sweet potato whitefly)
110368	GCA_900239965.1	Bicyclus anynana (squinting bush brown)
6526	GCA_000457365.1	Biomphalaria glabrata (bloodfluke planorb)
6973	GCA_003018175.1	Blattella germanica (German cockroach)
132113	GCA_000188095.3	Bombus impatiens (common eastern bumble bee)
30195	GCA_000214255.1	Bombus terrestris (buff-tailed bumblebee)
998830	GCA_002197625.1	Bombyx huttoni (moths)
7091	GCA_000151625.1	Bombyx mori (domestic silkworm)
104777	GCA_002922825.1	Brachionus calyciflorus (rotifers)
10195	GCA_003710015.1	Brachionus plicatilis (rotifers)
6279	GCA_000002995.4	Brugia malayi (agent of lymphatic filariasis)
6280	GCA_001280985.1	Brugia pahangi (nematodes)
6326	GCA_000231135.1	Bursaphelenchus xylophilus (pine wood nematode)
860376	GCA_000165025.1	Caenorhabditis angaria (nematodes)
135651	GCA_000143925.2	Caenorhabditis brenneri (nematodes)
6238	GCA_000004555.3	Caenorhabditis briggsae (nematodes)

1978547	GCA_003052745.1	Caenorhabditis inopinata (nematodes)
281687	GCA_000147155.1	Caenorhabditis japonica (nematodes)
1503980	GCA_002259235.1	Caenorhabditis latens (nematodes)
1611254	GCA_002742825.1	Caenorhabditis nigoni (nematodes)
2301259	GCA_900536275.1	Caenorhabditis panamensis (nematodes)
31234	GCA_000149515.1	Caenorhabditis remanei (nematodes)
1737332	GCA_900536235.1	Caenorhabditis sp. 21 LS-2015 (nematodes)
1737333	GCA_900536285.1	Caenorhabditis sp. 26 LS-2015 (nematodes)
2301260	GCA_900536315.1	Caenorhabditis sp. 29 LS-2018 (nematodes)
1737334	GCA_900536295.1	Caenorhabditis sp. 31 LS-2015 (nematodes)
1737335	GCA_900536325.1	Caenorhabditis sp. 32 LS-2015 (nematodes)
1729975	GCA_900536415.1	Caenorhabditis sp. 38 MB-2015 (nematodes)
1737336	GCA_900536345.1	Caenorhabditis sp. 39 LS-2015 (nematodes)
1737337	GCA_900536305.1	Caenorhabditis sp. 40 LS-2015 (nematodes)
1561998	GCA_000186765.1	Caenorhabditis tropicalis (nematodes)
6837	GCA_002740975.1	Calanus finmarchicus (crustaceans)
113644	GCA_002740985.1	Calanus glacialis (crustaceans)
2010996	GCA_002245505.1	Calephelis nemesis (butterflies)
2010997	GCA_002245475.1	Calephelis virginiensis (butterflies)
217165	GCA_001005385.1	Caligus rogercresseyi (crustaceans)
7373	GCA_001017275.1	Calliphora vicina (urban bluebottle blowfly)
52612	GCA_002093875.1	Calopteryx splendens (banded demoiselle)
691633	GCA_001625245.1	Calycopis cecrops (butterflies)
104421	GCA_003227725.1	Camponotus floridanus (Florida carpenter ant)
283909	GCA_000328365.1	Capitella teleta (segmented worms)
293153	GCA_002091895.1	Caridina multidentata (crustaceans)
438503	GCA_000934665.2	Catajapyx aquilonaris (insects)
797374	GCA_900474305.1	Cecidostiba fungosa (wasps, ants, and bees)
909773	GCA_900474235.1	Cecidostiba semifascia (wasps, ants, and bees)
2558925	GCA_002930495.1	Cecropterus lyciades (moths)
218467	GCA_000671375.2	Centruroides sculpturatus (bark scorpion)
211228	GCA_000341935.1	Cephus cinctus (wheat stem sawfly)
156304	GCA_001652005.1	Ceratina calcarata (bees)
7213	GCA_000347755.4	Ceratitis capitata (Mediterranean fruit fly)
326594	GCA_000503995.1	Ceratosolen solmsi marchali (wasps, ants, and bees)
204567	GCA_001014815.1	Chaoborus trivitattus (flies)
168631	GCA_000636095.1	Chilo suppressalis (striped riceborer)
315576	GCA_001014505.1	Chironomus riparius (flies)
7153	GCA_000786525.1	Chironomus tentans (flies)
79782	GCA_000648675.3	Cimex lectularius (bed bug)
1577616	GCA_001015075.1	Cirrula hians (flies)
325643	GCA_002778355.1	Clitarchus hookeri (smooth stick-insect)
85120	GCA_001014945.1	Clogmia albipunctata (mothmidge)
568069	GCA_900005825.1	Clunio marinus (flies)
170625	GCA_001014335.1	Coboldia fuscipes (flies)
41139	GCA_003568925.1	Coccinella septempunctata (seven-spotted ladybird)

1577614	GCA_001014875.1	Condylostylus patibulatus (flies)
101761	GCA_001262575.1	Conus tribblei (gastropods)
29053	GCA_000648655.2	Copidosoma floridanum (wasps, ants, and bees)
45949	GCA_001632725.1	Corbicula fluminea (asian clam)
51811	GCA_003123905.1	Cordylochernes scorpioides (false scorpions)
217443	GCA_000956155.1	Cotesia vestalis (diamondback moth parasitoid)
29159	GCA_000297895.1	Crassostrea gigas (Pacific oyster)
6565	GCA_002022765.4	Crassostrea virginica (eastern oyster)
105785	GCA_002891405.2	Cryptotermes secundus (termites)
7515	GCA_003426905.1	Ctenocephalides felis (cat flea)
7176	GCA_000209185.1	Culex quinquefasciatus (southern house mosquito)
179676	GCA_900258525.2	Culicoides sonorensis (flies)
82600	GCA_003425675.1	Cydia pomonella (codling moth)
456900	GCA_001594065.1	Cyphomyrmex costatus (ants)
765876	GCA_000833685.1	Dactylopius coccus (scale insects)
278856	GCA_000235995.2	Danaus plexippus plexippus (monarch butterfly)
35525	GCA_001632505.1	Daphnia magna (crustaceans)
6669	GCA_000187875.1	Daphnia pulex (common water flea)
77166	GCA_000355655.1	Dendroctonus ponderosae (mountain pine beetle)
6954	GCA_002085665.1	Dermatophagoides farinae (American house dust mite)
6956	GCA_001901225.2	Dermatophagoides pteronyssinus (European house dust mite)
50390	GCA_003013835.1	Diabrotica virgifera virgifera (western corn rootworm)
454923	GCA_001412515.1	Diachasma alloeum (wasps, ants, and bees)
121845	GCA_000475195.1	Diaphorina citri (Asian citrus psyllid)
29172	GCA_000816705.1	Dictyocaulus viviparus (bovine lungworm)
609295	GCA_001313825.1	Dinoponera quadriceps (ants)
1965070	GCA_003675995.1	Dinothrombium tinctorium (mites & ticks)
288516	GCA_002207785.1	Diploscapter coronatus (nematodes)
2018661	GCA_002287525.1	Diploscapter pachys (nematodes)
6287	GCA_001077395.1	Dirofilaria immitis (dog heartworm nematode)
166010	GCA_001579705.1	Ditylenchus destructor (nematodes)
143948	GCA_001186385.1	Diuraphis noxia (Russian wheat aphid)
7291	GCA_000298335.1	Drosophila albomicans (flies)
40366	GCA_001245395.1	Drosophila americana (flies)
7217	GCA_000005115.1	Drosophila ananassae (flies)
7263	GCA_001654025.1	Drosophila arizonae (flies)
7248	GCA_003185025.1	Drosophila athabasca (flies)
125945	GCA_000233415.2	Drosophila biarmipes (flies)
42026	GCA_000236285.2	Drosophila bipectinata (flies)
30019	GCA_001277935.1	Drosophila busckii (flies)
30023	GCA_000224195.2	Drosophila elegans (flies)
7220	GCA_003286155.2	Drosophila erecta (flies)
29029	GCA_000236325.2	Drosophila eugracilis (flies)
30025	GCA_000220665.2	Drosophila ficusphila (flies)
7222	GCA_000005155.1	Drosophila grimshawi (flies)
7224	GCA_002780465.1	Drosophila hydei (flies)

30033	GCA_000224215.2	Drosophila kikkawai (flies)
7229	GCA_000269505.2	Drosophila miranda (flies)
7230	GCA_000005175.1	Drosophila mojavensis (flies)
40370	GCA_003086615.1	Drosophila montana (flies)
42062	GCA_002222885.1	Drosophila nasuta (flies)
7232	GCA_001654015.1	Drosophila navojoa (flies)
47314	GCA_003285875.2	Drosophila novamexicana (flies)
7282	GCA_002217835.1	Drosophila obscura (flies)
7234	GCA_003286085.2	Drosophila persimilis (flies)
46245	GCA_000001765.3	Drosophila pseudoobscura pseudoobscura (flies)
1041015	GCA_000236305.2	Drosophila rhopaloa (flies)
7238	GCA_000005215.1	Drosophila sechellia (flies)
7274	GCA_002093755.1	Drosophila serrata (flies)
7240	GCA_000754195.3	Drosophila simulans (flies)
7241	GCA_002749795.1	Drosophila subobscura (flies)
28584	GCA_000472105.1	Drosophila suzukii (flies)
29030	GCA_000224235.2	Drosophila takahashii (flies)
7244	GCA_000005245.1	Drosophila virilis (flies)
7260	GCA_000005925.1	Drosophila willistoni (flies)
7245	GCA_000005975.1	Drosophila yakuba (flies)
614101	GCA_002236955.1	Dryococelus australis (walking sticks)
178035	GCA_001272555.1	Dufourea novaeangliae (bees)
6396	GCA_900000155.1	Eisenia fetida (common brandling worm)
1049336	GCA_000507165.2	Ephemera danica (mayflies)
1577615	GCA_001014675.1	Ephydra gracilis (flies)
95602	GCA_003336515.1	Eriocheir sinensis (Chinese mitten crab)
1352481	GCA_001015145.1	Eristalis dimidiata (flies)
516756	GCA_001483705.1	Eufriesea mexicana (bees)
1507135	GCA_002201625.1	Euglossa dilemma (dilemma orchid bee)
117534	GCA_002872375.1	Eulimnadia texana (crustaceans)
909778	GCA_900480025.1	Eupelmus annulatus (wasps, ants, and bees)
310279	GCA_900480035.1	Eupelmus urozonus (wasps, ants, and bees)
49087	GCA_003024985.1	Euperipatoides rowelli (velvet worms)
6958	GCA_002135145.1	Euroglyphus maynei (mites & ticks)
88015	GCA_000591075.2	Eurytemora affinis (crustaceans)
1172129	GCA_900480045.1	Eurytoma adleriae (wasps, ants, and bees)
261815	GCA_900475205.1	Eurytoma brunniventris (wasps, ants, and bees)
437493	GCA_003667255.1	Euschistus heros (neotropical brown stinkbug)
1577624	GCA_001015115.1	Eutreta diana (flies)
223228	GCA_900060175.1	Ferrisia virgata (striped mealybug)
158441	GCA_002217175.1	Folsomia candida (springtails)
64838	GCA_000806365.1	Fopius arisanus (wasps, ants, and bees)
72781	GCA_003651465.1	Formica exsecta (ants)
133901	GCA_000697945.3	Frankliniella occidentalis (western flower thrips)
34638	GCA_000255335.1	Galendromus occidentalis (western predatory mite)
7137	GCA_003640425.1	Galleria mellonella (greater wax moth)

56086	GCA_001010745.2	Gerris buenoi (bugs)
1517492	GCA_001723225.1	Globodera ellingtonae (nematodes)
36090	GCA_000724045.1	Globodera pallida (nematodes)
7395	GCA_000688735.1	Glossina austeni (tsetse fly)
37001	GCA_000671755.1	Glossina brevipalpis (tsetse fly)
201502	GCA_000671735.1	Glossina fuscipes fuscipes (tsetse fly)
37546	GCA_001077435.1	Glossina morsitans morsitans (tsetse fly)
7398	GCA_000688715.1	Glossina pallidipes (tsetse fly)
67801	GCA_000818775.1	Glossina palpalis gambiensis (tsetse fly)
1875375	GCA_003347265.1	Glossosoma conforme (caddisflies)
863708	GCA_003055095.1	Goniozus legneri (wasps, ants, and bees)
597456	GCA_001263275.1	Habropoda laboriosa (bees)
7368	GCA_003123925.1	Haematobia irritans (horn fly)
6289	GCA_000469685.1	Haemonchus contortus (barber pole worm)
36100	GCA_003918875.1	Haliotis rubra (blacklip abalone)
6454	GCA_003343065.1	Haliotis rufescens (red abalone)
286706	GCA_000696795.2	Halyomorpha halys (brown marmorated stink bug)
115357	GCA_003402655.1	Harmonia axyridis (beetles)
610380	GCA_003227715.1	Harpegnathos saltator (Jerdon's jumping ant)
249251	GCA_900068235.1	Heliconius cydno alithea (butterflies)
1756245	GCA_900068365.1	Heliconius elevatus bari (butterflies)
441047	GCA_001485985.1	Heliconius ethilla aerotome (butterflies)
1109109	GCA_001486065.1	Heliconius hecale felix (butterflies)
1756246	GCA_900068455.1	Heliconius hecuba flava (butterflies)
33423	GCA_900067975.1	Heliconius heurippa (butterflies)
171913	GCA_900068475.1	Heliconius hierax (butterflies)
33427	GCA_001485965.1	Heliconius ismenius (butterflies)
171917	GCA_000313835.2	Heliconius melpomene melpomene (postman butterfly)
310797	GCA_900068715.1	Heliconius numata bicoloratus (butterflies)
33428	GCA_900068735.1	Heliconius pachinus (butterflies)
884342	GCA_001486225.1	Heliconius pardalinus sergestus (butterflies)
573617	GCA_900068805.1	Heliconius timareta timareta (butterflies)
33429	GCA_900068815.1	Heliconius wallacei (butterflies)
33440	GCA_900068825.1	Heliconius xanthocles (butterflies)
29058	GCA_002156985.1	Helicoverpa armigera (cotton bollworm)
7113	GCA_002150865.1	Helicoverpa zea (corn earworm)
375939	GCA_900096555.1	Heligmosomoides polygyrus bakeri (nematodes)
7102	GCA_002382865.1	Heliothis virescens (tobacco budworm)
6412	GCA_000326865.1	Helobdella robusta (segmented worms)
343691	GCA_001014895.1	Hermetia illucens (flies)
51029	GCA_000150805.1	Heterodera glycines (soybean cyst nematode)
37862	GCA_000223415.1	Heterorhabditis bacteriophora (nematodes)
2041911	GCA_002916965.1	Hexapoda sp. (insects)
1983282	GCA_002738285.1	Holacanthella duospinosa (springtails)
1577617	GCA_001015215.1	Holcocephala fusca (flies)
197043	GCA_000696855.2	Homalodisca vitripennis (glassy-winged sharpshooter)

294128	GCA_000764305.2	Hyalella azteca (amphipods)
216498	GCA_001703475.1	Hydroides elegans (calcareous tube worm)
39466	GCA_003709505.1	Hyphantria cunea (fall webworm moth)
66581	GCA_000988845.1	Hypochthonius rufulus (mites & ticks)
1477025	GCA_003589595.1	Hyposmocoma kahamanoa (moths)
57062	GCA_001012855.1	Hypothenemus hampei (coffee berry borer)
232323	GCA_002082055.1	Hypsibius dujardini (tardigrades)
552050	GCA_001676475.1	Isoperla grammatica (stoneflies)
34613	GCA_000973045.2	Ixodes ricinus (castor bean tick)
6945	GCA_000208615.1	Ixodes scapularis (black-legged tick)
123851	GCA_000376725.2	Ladona fulva (scarce chaser)
195883	GCA_003335185.1	Laodelphax striatella (small brown planthopper)
1608916	GCA_900068335.1	Laparus doris doris (butterflies)
88501	GCA_000346575.1	Lasioglossum albipes (bees)
67767	GCA_001045655.1	Lasius niger (ants)
256737	GCA_000697925.2	Latrodectus hesperus (western black widow)
109027	GCA_002313205.1	Laupala kohalensis (crickets)
466727	GCA_003287335.1	Lednia tumana (stoneflies)
72036	GCA_001005205.1	Lepeophtheirus salmonis (salmon louse)
2004999	GCA_002806875.1	Lepidotrigona ventralis hoosana (bees)
217954	GCA_003723985.1	Lepidurus apus lubbocki (crustaceans)
77708	GCA_003724045.1	Lepidurus arcticus (crustaceans)
189913	GCA_900199415.1	Leptidea sinapis (butterflies)
7539	GCA_000500325.2	Leptinotarsa decemlineata (Colorado potato beetle)
63433	GCA_003121605.1	Leptopilina boulardi (wasps, ants, and bees)
63434	GCA_001855655.1	Leptopilina clavipes (wasps, ants, and bees)
299467	GCA_003675905.1	Leptotrombidium deliense (scrub typhus mite)
691651	GCA_001278395.1	Lerema accius (moths)
142080	GCA_002091915.1	Ligia exotica (isopods)
1218281	GCA_000648945.2	Limnephilus lunatus (caddisflies)
356393	GCA_003130415.1	Limnoperna fortunei (bivalves)
6850	GCA_000517525.1	Limulus polyphemus (Atlantic horseshoe crab)
83485	GCA_000217595.1	Linepithema humile (Argentine ant)
7574	GCA_001039355.2	Lingula anatina (brachiopods)
198433	GCA_001014935.1	Liriomyza trifolii (celery leafminer)
7209	GCA_000183805.3	Loa loa (eye worm)
7004	GCA_000516895.1	Locusta migratoria (migratory locust)
225164	GCA_000327385.1	Lottia gigantea (owl limpet)
6921	GCA_001188405.1	Loxosceles reclusa (brown recluse)
7375	GCA_000699065.2	Lucilia cuprina (Australian sheep blowfly)
13632	GCA_001014835.1	Lucilia sericata (common green bottle fly)
7200	GCA_000265325.1	Lutzomyia longipalpis (flies)
6523	GCA_900036025.1	Lymnaea stagnalis (great pond snail)
438506	GCA_003456935.1	Machilis hrabei (insects)
177089	GCA_003261595.1	Maconellicoccus hirsutus (hibiscus mealybug)
535359	GCA_002156465.1	Macrocentrus cingulum (wasps, ants, and bees)

174822	GCA_002192655.1	Mamestra configurata (bertha armyworm)
7130	GCA_000262585.1	Manduca sexta (tobacco hornworm)
39758	GCA_000149185.1	Mayetiola destructor (Hessian fly)
614211	GCA_003012365.1	Medauroidea extradentata (walking sticks)
143995	GCA_000220905.1	Megachile rotundata (alfalfa leafcutting bee)
88686	GCA_001015175.1	Megaselia abdita (flies)
36166	GCA_000341915.2	Megaselia scalaris (flies)
246452	GCA_900490025.1	Megastigmus dorsalis (wasps, ants, and bees)
311874	GCA_900490015.1	Megastigmus stigmatizans (wasps, ants, and bees)
1945771	GCA_003671415.1	Megathymus ursus violae (moths)
742174	GCA_002803265.2	Melanaphis sacchari (aphids)
166423	GCA_001276565.1	Melipona quadrifasciata (bees)
113334	GCA_000716385.1	Melitaea cinxia (Glanville fritillary)
6304	GCA_003133805.1	Meloidogyne arenaria (nematodes)
390850	GCA_003693675.1	Meloidogyne enterolobii (nematodes)
298350	GCA_003693605.1	Meloidogyne floridensis (nematodes)
189291	GCA_002778205.1	Meloidogyne graminicola (nematodes)
6305	GCA_000172435.1	Meloidogyne hapla (nematodes)
6306	GCA_900182535.1	Meloidogyne incognita (southern root-knot nematode)
6303	GCA_003693625.1	Meloidogyne javanica (root-knot nematode)
1155016	GCA_000281935.1	Mengenilla moldrzyki (twisted-wing parasites)
34649	GCA_000484575.1	Mesobuthus martensii (Chinese scorpion)
1250332	GCA_900490955.1	Micoletzkya japonica (nematodes)
69319	GCA_000572035.2	Microplitis demolitor (wasps, ants, and bees)
6573	GCA_002113885.2	Mizuhopecten yessoensis (Yesso scallop)
1577623	GCA_001014845.1	Mochlonyx cinctipes (flies)
310899	GCA_002080025.1	Modiolus philippinarum (bivalves)
307658	GCA_000980195.3	Monomorium pharaonis (pharaoh ant)
7370	GCA_000371365.1	Musca domestica (house fly)
29158	GCA_001676915.1	Mytilus galloprovincialis (Mediterranean mussel)
13164	GCA_001856785.1	Myzus persicae (green peach aphid)
7426	GCA_000004775.1	Nasonia giraulti (wasps, ants, and bees)
7427	GCA_000004795.1	Nasonia longicornis (wasps, ants, and bees)
7425	GCA_000002325.2	Nasonia vitripennis (jewel wasp)
51031	GCA_000507365.1	Necator americanus (nematodes)
441921	GCA_001263575.2	Neodiprion lecontei (redheaded pine sawfly)
33457	GCA_900068225.1	Neruda aoede (butterflies)
351381	GCA_900490065.1	Neuroterus quercusbaccarum (wasps, ants, and bees)
110193	GCA_001412225.1	Nicrophorus vespilloides (beetles)
108931	GCA_000757685.1	Nilaparvata lugens (brown planthopper)
27835	GCA_900200055.1	Nippostrongylus brasiliensis (nematodes)
2448451	GCA_003710045.1	Nomia melanderi (Alkali bee)
416868	GCA_002633025.1	Notospermus geniculatus (ribbon worms)
37653	GCA_001194135.1	Octopus bimaculoides (cephalopods)
61180	GCA_000797555.1	Oesophagostomum dentatum (nematodes)
136191	GCA_900157175.1	Oithona nana (crustaceans)

387005	GCA_002249935.1	Onchocerca flexuosa (nematodes)
42157	GCA_000950515.2	Onchocerca ochengi (nematodes)
6282	GCA_000499405.2	Onchocerca volvulus (nematodes)
7536	GCA_000696205.2	Oncopeltus fasciatus (milkweed bug)
166361	GCA_000648695.2	Onthophagus taurus (beetles)
2015173	GCA_003672135.1	Ooceraea biroi (clonal raider ant)
104452	GCA_001266575.1	Operophtera brumata (winter moth)
48709	GCA_001718145.1	Orchesella cincta (springtails)
591002	GCA_900474335.1	Ormyrus nitidulus (wasps, ants, and bees)
909788	GCA_900474385.1	Ormyrus pomaceus (wasps, ants, and bees)
222816	GCA_000612105.2	Orussus abietinus (hymenopterans)
1629725	GCA_001443705.1	Oryctes borbonicus (beetles)
1523172	GCA_000934875.1	Oscheius sp. MCB (nematodes)
1573569	GCA_001513535.1	Oscheius sp. TEL-2014 (nematodes)
141969	GCA_900184235.1	Oscheius tipulae (nematodes)
38123	GCA_000695645.2	Pachypsylla venusta (hackberry petiole gall psyllid)
6233	GCA_000341325.1	Panagrellus redivivus (nematodes)
45779	GCA_000931545.1	Papilio glaucus (eastern tiger swallowtail)
76193	GCA_001298355.1	Papilio machaon (common yellow swallowtail)
76198	GCA_003118335.1	Papilio memnon (butterflies)
76194	GCA_000836215.1	Papilio polytes (common Mormon)
66420	GCA_000836235.1	Papilio xuthus (Asian swallowtail)
252483	GCA_900065295.1	Paracoccus marginatus (scale insects)
1187980	GCA_900491355.1	Parapristionchus giblindavisi (nematodes)
116150	GCA_900499025.1	Pararge aegeria (specked wood butterfly)
6257	GCA_002259215.1	Parascaris univalens (nematodes)
114398	GCA_000365465.2	Parasteatoda tepidariorum (common house spider)
131310	GCA_000941615.1	Parastrongyloides trichosuri (nematodes)
317513	GCA_001587735.2	Parhyale hawaiensis (amphipods)
559696	GCA_003055125.1	Paykullia maculata (flies)
121224	GCA_000006295.1	Pediculus humanus corporis (human body louse)
27405	GCA_002291165.1	Penaeus japonicus (crustaceans)
6687	GCA_002291185.1	Penaeus monodon (black tiger shrimp)
6689	GCA_003789085.1	Penaeus vannamei (Pacific white shrimp)
6978	GCA_002939525.1	Periplaneta americana (American cockroach)
36667	GCA_002233535.1	Philaenus spumarius (meadow spittlebug)
29031	GCA_000262795.1	Phlebotomus papatasi (flies)
40077	GCA_001586405.1	Phoebis sennae (cloudless sulphur butterfly)
7380	GCA_001735545.1	Phormia regina (black blowfly)
115415	GCA_002633005.1	Phoronis australis (phoronid worms)
462259	GCA_001014415.1	Phortica variegata (flies)
64459	GCA_001856805.1	Pieris rapae (cabbage white)
437484	GCA_000786065.1	Piezodorus guildinii (redbanded stink bug)
66713	GCA_002216045.1	Pinctada imbricata (Akoya pearl oyster)
128015	GCA_000988905.1	Platynothrus peltifer (mites & ticks)
2011161	GCA_002796945.1	Plectus sambesii (nematodes)

58824	GCA_900182495.1	Plodia interpunctella (Indianmeal moth)
51655	GCA_000330985.1	Plutella xylostella (diamondback moth)
144034	GCA_000187915.1	Pogonomyrmex barbatus (red harvester ant)
235516	GCA_002278615.1	Pogonus chalceus (beetles)
91411	GCA_001313835.1	Polistes canadensis (wasps, ants, and bees)
743375	GCA_001465965.1	Polistes dominula (European paper wasp)
400727	GCA_003073045.1	Pomacea canaliculata (gastropods)
37621	GCA_000485595.2	Priapulus caudatus (priapulids)
1195655	GCA_900490705.1	Pristionchus arcanus (nematodes)
358040	GCA_900490825.1	Pristionchus entomophagus (nematodes)
1195656	GCA_900380275.1	Pristionchus exspectatus (nematodes)
1538716	GCA_900490895.1	Pristionchus fissidentatus (nematodes)
1195657	GCA_900490845.1	Pristionchus japonicus (nematodes)
1317128	GCA_900490775.1	Pristionchus maxplancki (nematodes)
1317129	GCA_900490875.1	Pristionchus mayeri (nematodes)
54126	GCA_000180635.3	Pristionchus pacificus (nematodes)
2065263	GCA_002838885.1	Procambarus virginalis (crustaceans)
1717472	GCA_001932985.1	Proctacanthus coquilletti (flies)
13233	GCA_900064475.1	Pseudococcus longispinus (long-tailed mealybug)
219809	GCA_002006095.1	Pseudomyrmex gracilis (ants)
83912	GCA_002943765.1	Psoroptes ovis (sheep scab mite)
52793	GCA_002072015.1	Radix auricularia (gastropods)
947166	GCA_001949185.1	Ramazzottius varieornatus (tardigrades)
114890	GCA_000944355.1	Rhabditophanes sp. KR3021 (nematodes)
28612	GCA_001687245.1	Rhagoletis zephyria (snowberry fruit fly)
6941	GCA_002176555.1	Rhipicephalus microplus (southern cattle tick)
13249	GCA_000181055.3	Rhodnius prolixus (bugs)
43146	GCA_003676215.1	Rhopalosiphum maidis (corn leaf aphid)
13658	GCA_001039655.1	Romanomermis culicivorax (nematodes)
392029	GCA_900239685.1	Rotaria macrura (rotifers)
392030	GCA_900239745.1	Rotaria magnacalcarata (rotifers)
239373	GCA_001026735.1	Rotylenchulus reniformis (nematodes)
157728	GCA_003671525.1	Saccostrea glomerata (Sydney rock oyster)
7385	GCA_001017455.1	Sarcophaga bullata (grey fleshfly)
1577618	GCA_001047195.1	Sarcophagidae sp. BV-2014 (flies)
52283	GCA_000828355.1	Sarcoptes scabiei (mites & ticks)
7225	GCA_003285725.1	Scaptodrosophila lebanonensis (flies)
13262	GCA_003264975.1	Schizaphis graminum (greenbug)
94630	GCA_003709985.1	Semibalanus balanoides (northern acorn barnacle)
1977532	GCA_003003475.1	Sericostoma sp. HW-2014 (caddisflies)
48799	GCA_003640385.1	Setaria digitata (nematodes)
221942	GCA_003012265.1	Setaria equina (nematodes)
143950	GCA_003268045.1	Sipha flava (yellow sugarcane aphid)
7048	GCA_002938485.1	Sitophilus oryzae (rice weevil)
625136	GCA_003595255.1	Solenopsis fugax (ants)
13686	GCA_000188075.2	Solenopsis invicta (red fire ant)

139677	GCA_001015235.1	Sphyracephala brevicornis (flies)
7108	GCA_002213285.1	Spodoptera frugiperda (fall armyworm)
69820	GCA_002706865.1	Spodoptera litura (moths)
52000	GCA_000988885.1	Steganacarus magnus (mites & ticks)
407821	GCA_000611955.2	Stegodyphus mimosarum (spiders)
34508	GCA_000757645.1	Steinernema carpocapsae (nematodes)
52066	GCA_000757705.1	Steinernema feltiae (nematodes)
37863	GCA_000757755.1	Steinernema glaseri (nematodes)
90984	GCA_000505645.1	Steinernema monticolum (nematodes)
90986	GCA_000757745.1	Steinernema scapterisci (mole cricket nematode)
35570	GCA_001015335.1	Stomoxys calcitrans (stable fly)
126957	GCA_000239455.1	Strigamia maritima (centipedes)
174720	GCA_000936265.1	Strongyloides papillosus (nematodes)
34506	GCA_001040885.1	Strongyloides ratti (nematodes)
6248	GCA_000947215.1	Strongyloides stercoralis (nematodes)
75913	GCA_001028725.1	Strongyloides venezuelensis (nematodes)
1120120	GCA_000981365.1	Subanguina moxae (nematodes)
452695	GCA_900474275.1	Synergus japonicus (wasps, ants, and bees)
331713	GCA_900474325.1	Synergus umbraculus (wasps, ants, and bees)
45464	GCA_002352805.1	Teladorsagia circumcincta (nematodes)
139649	GCA_002237135.1	Teleopsis dalmanni (flies)
300111	GCA_003070985.1	Temnothorax curvispinosus (ants)
594049	GCA_001017515.1	Tephritis californica (flies)
32264	GCA_000239435.1	Tetranychus urticae (two-spotted spider mite)
292402	GCA_001014575.1	Themira minor (flies)
61476	GCA_002928295.1	Timema cristinae (walking sticks)
312236	GCA_001017535.1	Tipula oleracea (flies)
714620	GCA_900474315.1	Torymus auratus (wasps, ants, and bees)
310282	GCA_900474355.1	Torymus geranii (wasps, ants, and bees)
6265	GCA_000803305.1	Toxocara canis (dog roundworm)
471704	GCA_001594075.1	Trachymyrmex cornetzi (ants)
34720	GCA_001594115.1	Trachymyrmex septentrionalis (ants)
64791	GCA_001594055.1	Trachymyrmex zeteki (ants)
7070	GCA_000002335.3	Tribolium castaneum (red flour beetle)
45882	GCA_001447585.1	Trichinella britovi (nematodes)
144512	GCA_002221485.1	Trichinella murrelli (nematodes)
6335	GCA_001447565.1	Trichinella nativa (nematodes)
6336	GCA_001447455.1	Trichinella nelsoni (nematodes)
268474	GCA_001447755.1	Trichinella papuae (nematodes)
990121	GCA_001447655.1	Trichinella patagoniensis (nematodes)
6337	GCA_001447645.1	Trichinella pseudospiralis (nematodes)
92179	GCA_001447435.1	Trichinella sp. T6 (nematodes)
92180	GCA_001447745.1	Trichinella sp. T8 (nematodes)
181606	GCA_001447505.1	Trichinella sp. T9 (nematodes)
6334	GCA_000181795.2	Trichinella spiralis (nematodes)
268475	GCA_001447665.1	Trichinella zimbabwensis (nematodes)

1577620	GCA_001014425.1	Trichoceridae sp. BV-2014 (flies)
7493	GCA_000599845.3	Trichogramma pretiosum (wasps, ants, and bees)
543379	GCA_002249905.1	Trichomalopsis sarcophagae (wasps, ants, and bees)
2585209	GCA_002102615.1	Trichonephila clavipes (spiders)
7111	GCA_003590095.1	Trichoplusia ni (cabbage looper)
70415	GCA_000612645.1	Trichuris muris (nematodes)
68888	GCA_000797535.1	Trichuris suis (pig whipworm)
36087	GCA_000613005.1	Trichuris trichiura (human whipworm)
1295211	GCA_900050545.1	Trionymus perrisii (scale insects)
194544	GCA_000981345.1	Triops cancriformis (crustaceans)
418985	GCA_002081605.1	Tropilaelaps mercedesae (mites & ticks)
1582032	GCA_001014665.1	Trupanea jonesi (flies)
334116	GCA_002938995.1	Vanessa tameamea (butterflies)
109461	GCA_002443255.1	Varroa destructor (honeybee mite)
62625	GCA_002532875.1	Varroa jacobsoni (mites & ticks)
301928	GCA_003401595.1	Venustaconcha ellipsiformis (ellipse)
411798	GCA_000949405.1	Vollenhovia emeryi (ants)
64793	GCA_000956235.1	Wasmannia auropunctata (little fire ant)
6293	GCA_000180755.1	Wuchereria bancrofti (agent of lymphatic filariasis)
6293	GCA_001555675.1	Wuchereria bancrofti (agent of lymphatic filariasis)
6293	GCA_005281725.1	Wuchereria bancrofti (agent of lymphatic filariasis)
6293	GCA_900622535.1	Wuchereria bancrofti (agent of lymphatic filariasis)
227619	GCA_900249655.1	Xenocatantops brachycerus (grasshoppers)
76712	GCA_001752445.1	Zaprionus indianus (flies)
28588	GCA_000806345.1	Zeugodacus cucurbitae (melon fly)
136037	GCA_000696155.1	Zootermopsis nevadensis (termites)

Other Metazoa

taxid	Acession	Species
133434	GCA_001949145.1	Acanthaster planci (crown-of-thorns starfish)
473344	GCA_900312575.1	Acanthochaenus luetkenii (pricklefish)
80966	GCA_002109545.1	Acanthochromis polyacanthus (spiny chromis)
7906	GCA_004119895.1	Acipenser ruthenus (sterlet)
2066578	GCA_003347165.1	Ageneiosus marmoratus (bony fishes)
8296	GCA_002915635.2	Ambystoma mexicanum (axolotl)
61819	GCA_000751415.1	Amphilophus citrinellus (Midas cichlid)
80972	GCA_002776465.1	Amphiprion ocellaris (clown anemonefish)
161767	GCA_003047355.2	Amphiprion percula (orange clownfish)
495550	GCA_003731715.1	Anabarilius grahami (bony fishes)
64144	GCA_900324465.2	Anabas testudineus (climbing perch)
64144	GCA_900650485.1	Anabas testudineus (climbing perch)
433405	GCA_004355925.1	Anarrhichthys ocellatus (wolf-eel)
7936	GCA_000695075.1	Anguilla anguilla (European eel)
7937	GCA_003597225.1	Anguilla japonica (Japanese eel)
7938	GCA_001606085.1	Anguilla rostrata (American eel)
88656	GCA_900683385.1	Anoplogaster cornuta (common fangtooth)
229290	GCA_000499045.1	Anoplopoma fimbria (sablefish)
241820	GCA_900303275.1	Antennarius striatus (striated frogfish)
52653	GCA_006937985.1	Aphyosemion australe (lyretail killifish)
307972	GCA_002754855.1	Apostichopus japonicus (Japanese sea cucumber)
1902835	GCA_000934455.1	Apostichopus parvimensis (sea cucumbers)
113544	GCA_900497675.1	Arapaima gigas (arapaima)
63155	GCA_007364235.1	Archocentrus centrarchus (flier cichlid)
63155	GCA_007364275.2	Archocentrus centrarchus (flier cichlid)
185735	GCA_900303235.1	Arctogadus glacialis (Arctic cod)
8154	GCA_900246225.3	Astatotilapia calliptera (eastern happy)
7994	GCA_000372685.2	Astyanax mexicanus (Mexican tetra)
223987	GCA_001663935.1	Asymmetron lucayanum (lancelets)
52670	GCA_001266775.1	Austrofundulus limnaeus (bony fishes)
175774	GCA_005784505.1	Bagarius yarrelli (goonch)
2005485	GCA_004367955.1	Bathochordaeus stygius (tunicates)
630650	GCA_900302375.1	Bathygadus melanobranchus (Vaillant's grenadier)
125796	GCA_900323375.1	Benthosema glaciale (glacier lanternfish)
88663	GCA_900312565.1	Beryx splendens (splendid alfonsino)
158456	GCA_900634795.2	Betta splendens (Siamese fighting fish)
150288	GCA_000788275.1	Boleophthalmus pectinirostris (great blue-spotted mudskipper)
44932	GCA_900302515.1	Boreogadus saida (bony fishes)
473354	GCA_900323325.1	Borostomias antarcticus (large-eye snaggletooth)
30301	GCA_000444245.1	Botryllus schlosseri (tunicates)
7741	GCA_001625305.1	Branchiostoma belcheri (Belcher's lancelet)
7739	GCA_000003815.1	Branchiostoma floridae (Florida lancelet)
7740	GCA_900088365.1	Branchiostoma lanceolatum (amphioxus)

630652	GCA_900302395.1	Bregmaceros cantori (striped codlet)
81638	GCA_900302425.1	Brosme brosme (tusk)
432164	GCA_900303265.1	Brotula barbata (bearded brotula)
60409	GCA_006937965.1	Callopanchax toddi (bony fishes)
7868	GCA_000165045.2	Callorhinchus milii (elephant shark)
1491482	GCA_900312935.1	Carapus acus (pearlfish)
7957	GCA_003368295.1	Carassius auratus (goldfish)
13397	GCA_902204185.1	Carcharodon carcharias (great white shark)
115865	GCA_900660305.1	Cetomimus sp. NI1144 (bony fishes)
36190	GCA_900302675.1	Chaenocephalus aceratus (blackfin icefish)
2518323	GCA_004786185.1	Channa argus var. Kimnra (platinum snakehead)
29144	GCA_902362185.1	Chanos chanos (milkfish)
990972	GCA_900302635.1	Chatrabus melanurus (pony toadfish)
36176	GCA_004010195.1	Chiloscyllium plagiosum (whitespotted bambooshark)
137246	GCA_003427335.1	Chiloscyllium punctatum (brownbanded bambooshark)
91607	GCA_900302755.1	Chromis chromis (damselfish)
7719	GCA_000224145.2	Ciona intestinalis (vase tunicate)
51511	GCA_000149265.1	Ciona savignyi (Pacific transparent sea squirt)
172907	GCA_004028445.1	Cirrhinus molitorella (mud carp)
59899	GCA_003987875.1	Clarias batrachus (walking catfish)
7950	GCA_000966335.1	Clupea harengus (Atlantic herring)
365059	GCA_007927625.1	Coilia nasus (Japanese grenadier anchovy)
240159	GCA_004119915.1	Collichthys lucidus (big head croaker)
861768	GCA_902175075.1	Coregonus sp. 'balchen' (bony fishes)
163118	GCA_002895965.1	Coryphaenoides rupestris (roundnose grenadier)
56716	GCA_900634435.1	Cottoperca gobio (bony fishes)
56716	GCA_900634415.1	Cottoperca gobio (bony fishes)
446433	GCA_001455555.1	Cottus rhenanus (bony fishes)
244447	GCA_000523025.1	Cynoglossus semilaevis (tongue sole)
69242	GCA_000776015.1	Cyprinodon nevadensis pectoralis (Amargosa pupfish)
28743	GCA_000732505.1	Cyprinodon variegatus (sheepshead minnow)
7962	GCA_000951615.2	Cyprinus carpio (common carp)
1176755	GCA_900302355.1	Cyttopsis rosea (rosy dory)
623740	GCA_900490495.1	Danionella dracula (bony fishes)
623744	GCA_007224835.1	Danionella translucida (bony fishes)
299321	GCA_900700375.1	Denticeps clupeoides (denticle herring)
299321	GCA_900700345.2	Denticeps clupeoides (denticle herring)
13489	GCA_000689215.1	Dicentrarchus labrax (European seabass)
1415272	GCA_900660315.1	Diretmoides pauciradiatus (bony fishes)
88682	GCA_900660295.1	Diretmus argenteus (silver spinyfin)
173247	GCA_900963505.1	Echeneis naucrates (live sharksucker)
173247	GCA_900963305.1	Echeneis naucrates (live sharksucker)
8005	GCA_003665695.2	Electrophorus electricus (electric eel)
310571	GCA_005281545.1	Epinephelus lanceolatus (giant grouper)
300413	GCA_006386435.1	Epinephelus moara (kelp grouper)
7764	GCA_900186335.2	Eptatretus burgeri (inshore hagfish)

27687	GCA_900747795.2	Erpetoichthys calabaricus (reedfish)
8010	GCA_004634155.1	Esox lucius (northern pike)
7632	GCA_001188425.1	Eucidaris tribuloides (sea urchins)
2507563	GCA_004368075.1	Fritillaria borealis (tunicates)
8078	GCA_000826765.1	Fundulus heteroclitus (mummichog)
185737	GCA_900302595.1	Gadiculus argenteus (silvery cod)
1042646	GCA_900302575.1	Gadus chalcogrammus (walleye pollock)
8049	GCA_902167405.1	Gadus morhua (Atlantic cod)
33528	GCA_003097735.1	Gambusia affinis (western mosquitofish)
69293	GCA_006229165.1	Gasterosteus aculeatus (three-spined stickleback)
334984	GCA_900660455.1	Gephyroberyx darwinii (Darwin's slimehead)
441366	GCA_900634775.1	Gouania willdenowi (blunt-snouted clingfish)
441366	GCA_900650505.1	Gouania willdenowi (blunt-snouted clingfish)
1263181	GCA_900312595.1	Guentherus altivela (highfin tadpole fish)
8153	GCA_000239415.1	Haplochromis burtoni (Burton's mouthbrooder)
243708	GCA_900302695.1	Helostoma temminkii (kissing gourami)
7650	GCA_003118195.1	Hemicentrotus pulcherrimus (sea urchins)
109280	GCA_001891065.1	Hippocampus comes (tiger tail seahorse)
722565	GCA_900302615.1	Holocentrus rufus (longspine squirrelfish)
96778	GCA_900660355.1	Hoplostethus atlanticus (orange roughy)
62062	GCA_003317085.1	Hucho hucho (huchen)
13095	GCA_004764525.1	Hypophthalmichthys molitrix (silver carp)
7965	GCA_004193235.1	Hypophthalmichthys nobilis (bighead carp)
146810	GCA_900610375.1	Hypoplectrus puella (barred hamlet)
7998	GCA_001660625.1	Ictalurus punctatus (channel catfish)
1747188	GCA_007896545.1	Kryptolebias hermaphroditus (bony fishes)
37003	GCA_001649575.1	Kryptolebias marmoratus (mangrove rivulus)
84645	GCA_004120215.1	Labeo rohita (rohu)
57307	GCA_000150875.1	Labeotropheus fuelleborni (blue mbuna)
56723	GCA_900080235.1	Labrus bergylta (ballan wrasse)
1784819	GCA_900303225.1	Laemonema laureysi (bony fishes)
81370	GCA_900302545.1	Lampris guttatus (North Atlantic opah)
1592065	GCA_900312555.1	Lamprogrammus exutus (bony fishes)
215358	GCA_000972845.2	Larimichthys crocea (large yellow croaker)
315492	GCA_004023545.1	Lateolabrax maculatus (spotted sea bass)
8187	GCA_001640805.1	Lates calcarifer (barramundi perch)
7897	GCA_000225785.1	Latimeria chalumnae (coelacanth)
7918	GCA_000242695.1	Lepisosteus oculatus (spotted gar)
1365564	GCA_900303255.1	Lesueurigobius sanzi (Sanzo's goby)
980415	GCA_000466285.1	Lethenteron camtschaticum (Arctic lamprey)
155063	GCA_900092035.1	Leuciscus waleckii (Amur ide)
7782	GCA_000238235.1	Leucoraja erinacea (little skate)
230148	GCA_006348945.1	Liparis tanakae (Tanaka's snailfish)
69944	GCA_900302385.1	Lota lota (burbot)
7654	GCA_000239495.2	Lytechinus variegatus (green sea urchin)
135761	GCA_002120245.1	Maccullochella peelii (Murray cod)

135765	GCA_005408345.1	Macquaria australasica (Macquarie perch)
473319	GCA_900302365.1	Macrourus berglax (onion-eye grenadier)
630739	GCA_900312585.1	Malacocephalus occidentalis (western softhead grenadier)
205130	GCA_900700395.1	Mastacembelus armatus (zig-zag eel)
205130	GCA_900324485.2	Mastacembelus armatus (zig-zag eel)
106582	GCA_000238955.5	Maylandia zebra (zebra mbuna)
35575	GCA_000150855.1	Mchenga conophoros (bony fishes)
27751	GCA_000150895.1	Melanochromis auratus (golden mbuna)
8056	GCA_900291075.1	Melanogrammus aeglefinus (haddock)
181410	GCA_900302345.1	Melanonus zugmayeri (arrowtail)
8058	GCA_900323355.1	Merlangius merlangus (whiting)
89947	GCA_900312945.1	Merluccius capensis (shallow-water Cape hake)
8063	GCA_900312545.1	Merluccius merluccius (European hake)
89951	GCA_900312625.1	Merluccius polli (Benguela hake)
2501294	GCA_004367975.1	Mesochordaeus erythrocephalus (tunicates)
225391	GCA_002592385.1	Micropterus floridanus (Florida bass)
240162	GCA_001593715.1	Miichthys miiuy (Mi-iuy croaker)
94237	GCA_001698575.1	Mola mola (ocean sunfish)
163112	GCA_900323295.1	Molva molva (ling)
181435	GCA_900323365.1	Monocentris japonicus (pineconefish)
43700	GCA_001952655.1	Monopterus albus (swamp eel)
248765	GCA_900303205.1	Mora moro (bony fishes)
46259	GCA_003610055.1	Morone chrysops (white bass)
34816	GCA_004916995.1	Morone saxatilis (striped sea-bass)
487677	GCA_900302325.1	Muraenolepis marmoratus (marbled moray cod)
8097	GCA_900312955.1	Myoxocephalus scorpius (shorthorn sculpin)
371672	GCA_900302555.1	Myripristis jacobus (blackbar soldierfish)
586833	GCA_902150065.1	Myripristis murdjan (pinecone soldierfish)
125878	GCA_000935625.1	Nanorana parkeri (frogs & toads)
47308	GCA_007210695.1	Neogobius melanostomus (round goby)
32507	GCA_000239395.1	Neolamprologus brichardi (lyretail cichlid)
47706	GCA_900302535.1	Neoniphon sammara (Sammara squirrelfish)
240163	GCA_900327885.1	Nibea albiflora (white flower croaker)
105023	GCA_001465895.2	Nothobranchius furzeri (turquoise killifish)
321403	GCA_006942095.1	Nothobranchius kuhntae (Beira killifish)
8208	GCA_000735185.1	Notothenia coriiceps (black rockcod)
2500139	GCA_004367875.1	Oikopleura albicans (tunicates)
34765	GCA_000209535.1	Oikopleura dioica (tunicates)
107032	GCA_004367895.1	Oikopleura longicauda (tunicates)
2056977	GCA_004367855.1	Oikopleura vanhoeffeni (tunicates)
8019	GCA_002021735.1	Oncorhynchus kisutch (coho salmon)
8022	GCA_002163495.1	Oncorhynchus mykiss (rainbow trout)
8023	GCA_006149115.1	Oncorhynchus nerka (sockeye salmon)
74940	GCA_002872995.1	Oncorhynchus tshawytscha (Chinook salmon)
225387	GCA_004358465.1	Ophiodon elongatus (bony fishes)
1401014	GCA_900067615.1	Ophionereis fasciata (brittle stars)

1266684	GCA_000969725.1	Ophiothrix spiculata (brittle stars)
163134	GCA_003416845.1	Oplegnathus fasciatus (barred knifejaw)
95145	GCA_900660325.1	Opsanus beta (Gulf toadfish)
47969	GCA_005870065.1	Oreochromis aureus (blue tilapia)
8128	GCA_001858045.3	Oreochromis niloticus (Nile tilapia)
123683	GCA_003999625.1	Oryzias javanicus (javanese ricefish)
8090	GCA_002234675.1	Oryzias latipes (Japanese medaka)
30732	GCA_002922805.1	Oryzias melastigma (Indian medaka)
29151	GCA_900302275.1	Osmerus eperlanus (European smelt)
361644	GCA_003573665.1	Oxygymnocypris stewartii (bony fishes)
52664	GCA_006937955.1	Pachypanchax playfairii (golden panchax)
143350	GCA_002897255.1	Pagrus major (red seabream)
206143	GCA_000697985.1	Pampus argenteus (silver pomfret)
310915	GCA_003671635.1	Pangasianodon hypophthalmus (striped catfish)
171872	GCA_900302745.1	Parablennius parvicornis (rock-pool blenny)
8255	GCA_001970005.2	Paralichthys olivaceus (Japanese flounder)
210632	GCA_900651595.1	Parambassis ranga (Indian glassy fish)
210632	GCA_900634625.1	Parambassis ranga (Indian glassy fish)
1676925	GCA_002872115.1	Paramormyrops kingsleyae (bony fishes)
1784818	GCA_900302295.1	Parasudis fraserbrunneri (bony fishes)
46514	GCA_000285935.1	Patiria miniata (bat star)
46519	GCA_900067625.1	Patiriella regularis (starfish)
8167	GCA_004354835.1	Perca flavescens (yellow perch)
8168	GCA_003412525.1	Perca fluviatilis (European perch)
143327	GCA_900302285.1	Percopsis transmontana (sand roller)
1365757	GCA_000787095.1	Periophthalmodon schlosseri (giant mudskipper)
409849	GCA_000787105.1	Periophthalmus magnuspinnatus (bony fishes)
7757	GCA_002833325.1	Petromyzon marinus (sea lamprey)
59560	GCA_003260075.1	Phallusia mammillata (warty sea squirt)
163115	GCA_900302315.1	Phycis blennoides (bony fishes)
349666	GCA_900302335.1	Phycis phycis (bony fishes)
90988	GCA_000700825.1	Pimephales promelas (fathead minnow)
370040	GCA_005024645.1	Planiliza haematocheilus (so-iny mullet)
48698	GCA_000485575.1	Poecilia formosa (Amazon molly)
48699	GCA_001443285.1	Poecilia latipinna (sailfin molly)
48701	GCA_001443325.1	Poecilia mexicana (shortfin molly)
8081	GCA_000633615.2	Poecilia reticulata (guppy)
8060	GCA_900312635.1	Pollachius virens (saithe)
81385	GCA_900302305.1	Polymixia japonica (silver eye)
357532	GCA_004124795.1	Poropuntius huangchuchieni (bony fishes)
280673	GCA_900323345.1	Pseudochromis fuscus (brown dottyback)
2499240	GCA_004335475.1	Pseudoliparis sp. Yap Trench (bony fishes)
245875	GCA_000787555.1	Pseudopleuronectes yokohamae (marbled flounder)
63121	GCA_001465055.1	Ptychodera flava (hemichordates)
303518	GCA_000239375.1	Pundamilia nyererei (bony fishes)
134920	GCA_003399555.1	Pungitius pungitius (ninespine stickleback)

42514	GCA_001682695.1	Pygocentrus nattereri (red-bellied piranha)
8400	GCA_002284835.2	Rana catesbeiana (American bullfrog)
81389	GCA_900302585.1	Regalecus glesne (king of herrings)
111784	GCA_006182925.2	Reinhardtius hippoglossoides (Greenland flounder)
163638	GCA_000150935.1	Rhamphochromis esox (bony fishes)
259920	GCA_001642345.2	Rhincodon typus (whale shark)
8386	GCA_900303285.1	Rhinella marina (marine toad)
88713	GCA_900302605.1	Rondeletia loricata (redmouth whalefish)
10224	GCA_000003605.1	Saccoglossus kowalevskii (hemichordates)
181472	GCA_902148845.1	Salarias fasciatus (jewelled blenny)
8030	GCA_000233375.4	Salmo salar (Atlantic salmon)
8032	GCA_901001165.1	Salmo trutta (river trout)
569448	GCA_001749815.1	Salpa thompsoni (tunicates)
8036	GCA_002910315.2	Salvelinus alpinus (Arctic char)
27697	GCA_900499035.1	Sardina pilchardus (sardine)
166764	GCA_000787155.1	Scartelaos histophorus (walking goby)
113540	GCA_900964775.1	Scleropages formosus (Asian bonytongue)
52904	GCA_003186165.1	Scophthalmus maximus (turbot)
75743	GCA_003427355.1	Scyliorhinus torazame (cloudy catshark)
214485	GCA_001910805.2	Sebastes aleutianus (rougheye rockfish)
290523	GCA_004335335.1	Sebastes koreanus (bony fishes)
214483	GCA_001910765.2	Sebastes minor (bony fishes)
72089	GCA_000475235.3	Sebastes nigrocinctus (tiger rockfish)
394699	GCA_900302655.1	Sebastes norvegicus (golden redfish)
1617787	GCA_004335365.1	Sebastes nudus (ogon-murasoi)
72099	GCA_000475215.1	Sebastes rubrivinctus (flag rockfish)
214486	GCA_004335315.1	Sebastes schlegelii (Schlegel's black rockfish)
201708	GCA_001910785.2	Sebastes steindachneri (bony fishes)
179366	GCA_900303245.1	Selene dorsalis (African moonfish)
41447	GCA_002260705.1	Seriola dumerili (greater amberjack)
1841481	GCA_002814215.1	Seriola lalandi dorsalis (yellowtail amberjack)
8161	GCA_002217815.1	Seriola quinqueradiata (Japanese amberjack)
173321	GCA_002994505.1	Seriola rivoliana (Almaco jack)
43689	GCA_900408965.1	Simochromis diagramma (bony fishes)
1608454	GCA_001515605.1	Sinocyclocheilus anshuiensis (bony fishes)
75366	GCA_001515645.1	Sinocyclocheilus grahami (bony fishes)
307959	GCA_001515625.1	Sinocyclocheilus rhinocerous (bony fishes)
8175	GCA_900880675.1	Sparus aurata (gilthead seabream)
375764	GCA_902148855.1	Sphaeramia orbicularis (orbiculate cardinalfish)
50595	GCA_900302685.1	Spondyliosoma cantharus (black seabream)
144197	GCA_000690725.1	Stegastes partitus (bicolor damselfish)
7668	GCA_000002235.3	Strongylocentrotus purpuratus (purple sea urchin)
409996	GCA_900312615.1	Stylephorus chordatus (tube-eye)
171736	GCA_002819105.1	Symphodus melops (corkwing wrasse)
161584	GCA_901709675.1	Syngnathus acus (greater pipefish)
1234273	GCA_003724035.1	Tachysurus fulvidraco (yellow catfish)

433685	GCA_004026145.1	Takifugu bimaculatus (bony fishes)
433684	GCA_003711565.2	Takifugu flavidus (sansaifugu)
31033	GCA_901000745.3	Takifugu rubripes (torafugu)
31033	GCA_901000725.2	Takifugu rubripes (torafugu)
373995	GCA_003651195.1	Tenualosa ilisha (Hilsa shad)
99883	GCA_000180735.1	Tetraodon nigroviridis (spotted green pufferfish)
8236	GCA_900302625.1	Thunnus albacares (yellowfin tuna)
8238	GCA_000418415.1	Thunnus orientalis (Pacific bluefin tuna)
8237	GCA_003231725.1	Thunnus thynnus (Atlantic bluefin tuna)
36185	GCA_004348285.1	Thymallus thymallus (grayling)
173339	GCA_900607315.1	Trachinotus ovatus (derbio)
241836	GCA_900323305.1	Trachyrincus murrayi (roughnose grenadier)
562814	GCA_900303215.1	Trachyrincus scabrus (roughsnout grenadier)
422203	GCA_006030095.1	Triplophysa siluroides (bony fishes)
80722	GCA_900302415.1	Trisopterus minutus (poor cod)
940470	GCA_900302405.1	Typhlichthys subterraneus (southern cavefish)
8355	GCA_001663975.1	Xenopus laevis (African clawed frog)
8364	GCA_000004195.3	Xenopus tropicalis (tropical clawed frog)
32473	GCA_001444195.3	Xiphophorus couchianus (Monterrey platyfish)
8084	GCA_003331165.1	Xiphophorus hellerii (green swordtail)
8083	GCA_002775205.2	Xiphophorus maculatus (southern platyfish)
64108	GCA_900323335.1	Zeus faber (John dory)

Protists

taxid	Acession	Species
749232	GCA_002812265.1	Abeoforma whisleri (eukaryotes)
65658	GCA_000826245.1	Acanthamoeba astronyxis (eukaryotes)
1257118	GCA_000313135.1	Acanthamoeba castellanii str. Neff (eukaryotes)
65659	GCA_002025285.1	Acanthamoeba comandoni (eukaryotes)
43142	GCA_000826265.1	Acanthamoeba culbertsoni (eukaryotes)
202919	GCA_000826405.1	Acanthamoeba divionensis (eukaryotes)
65661	GCA_000826305.1	Acanthamoeba healyi (eukaryotes)
29196	GCA_002179805.1	Acanthamoeba lenticulata (eukaryotes)
61605	GCA_000826425.1	Acanthamoeba lugdunensis (eukaryotes)
196912	GCA_000826465.1	Acanthamoeba mauritaniensis (eukaryotes)
28015	GCA_000826325.1	Acanthamoeba palestinensis (eukaryotes)
65662	GCA_000826505.1	Acanthamoeba pearcei (eukaryotes)
5757	GCA_001567625.1	Acanthamoeba polyphaga (eukaryotes)
211522	GCA_000826445.1	Acanthamoeba quina (eukaryotes)
32599	GCA_000826385.1	Acanthamoeba rhysodes (eukaryotes)
32600	GCA_000826365.1	Acanthamoeba royreba (eukaryotes)
1202772	GCA_002081595.1	Achlya hypogyna (oomycetes)
133408	GCA_900092245.1	Acytostelium leptosomum (cellular slime molds)
1410327	GCA_000787575.2	Acytostelium subglobosum LB1 (cellular slime molds)
65357	GCA_001306755.1	Albugo candida (oomycetes)
112090	GCA_000520075.1	Aphanomyces astaci (oomycetes)
100861	GCA_900312765.1	Aphanomyces euteiches (oomycetes)
157072	GCA_000520115.1	Aphanomyces invadans (oomycetes)
120398	GCA_900243725.1	Aphanomyces stellatus (oomycetes)
210441	GCA_002256025.1	Asterionella formosa (diatoms)
46078	GCA_000211355.2	Astrammina rara (forams)
2172886	GCA_004332575.1	Aurantiochytrium acetophilum (slime nets)
2010233	GCA_003116975.1	Aurantiochytrium sp. KH105 (slime nets)
1749249	GCA_001462505.1	Aurantiochytrium sp. T66 (slime nets)
44056	GCA_000186865.1	Aureococcus anophagefferens (pelagophytes)
5866	GCA_000981445.1	Babesia bigemina (apicomplexans)
5865	GCA_000165395.1	Babesia bovis (apicomplexans)
1133968	GCA_000691945.2	Babesia microti strain RI (apicomplexans)
66527	GCA_001185145.1	Balamuthia mandrillaris (eukaryotes)
753081	GCA_000320545.1	Bigelowiella natans CCMP2755 (cercozoans)
12968	GCA_000151665.1	Blastocystis hominis (eukaryotes)
478820	GCA_001651215.1	Blastocystis sp. ATCC 50177/Nand II (eukaryotes)
944160	GCA_000963365.1	Blastocystis sp. subtype 2 (eukaryotes)
944168	GCA_000963385.1	Blastocystis sp. subtype 3 (eukaryotes)
944170	GCA_000743755.1	Blastocystis sp. subtype 4 (eukaryotes)
944208	GCA_000963415.1	Blastocystis sp. subtype 6 (eukaryotes)
429571	GCA_000963455.1	Blastocystis sp. subtype 8 (eukaryotes)
1544353	GCA_000963465.1	Blastocystis sp. subtype 9 (eukaryotes)
4779	GCA_004359215.1	Bremia lactucae (lettuce downy mildew)

595528	GCA_000151315.2	Capsaspora owczarzaki ATCC 30864 (eukaryotes)
361123	GCA_900092275.1	Cavenderia deminutiva (cellular slime molds)
261658	GCA_000203815.1	Cavenderia fasciculata (cellular slime molds)
2769	GCA_000350225.2	Chondrus crispus (carragheen)
127563	GCA_002887195.1	Chrysochromulina parva (haptophytes)
1460289	GCA_001275005.1	Chrysochromulina tobinii (haptophytes)
309737	GCA_001742925.1	Cladosiphon okamuranus (brown algae)
98351	GCA_002811645.1	Corallochytrium limacisporum (eukaryotes)
142831	GCA_900092265.1	Coremiostelium polycephalum (cellular slime molds)
470921	GCA_002024145.1	Creolimax fragrantissima (eukaryotes)
441375	GCA_000006515.1	Cryptosporidium muris RN66 (apicomplexans)
353152	GCA_000165345.1	Cryptosporidium parvum Iowa II (apicomplexans)
280699	GCA_000091205.1	Cyanidioschyzon merolae strain 10D (red algae)
2762	GCA_004431415.1	Cyanophora paradoxa (eukaryotes)
88456	GCA_002999335.1	Cyclospora cayetanensis (apicomplexans)
361072	GCA_000286055.1	Dictyostelium citrinum (cellular slime molds)
352472	GCA_000004695.1	Dictyostelium discoideum AX4 (cellular slime molds)
79012	GCA_000277485.1	Dictyostelium firmibasis (cellular slime molds)
361076	GCA_000277465.1	Dictyostelium intermedium (cellular slime molds)
5786	GCA_000190715.1	Dictyostelium purpureum (cellular slime molds)
945030	GCA_004798425.1	Digenea simplex (red algae)
2880	GCA_000310025.1	Ectocarpus siliculosus (brown algae)
1768030	GCA_004764655.1	Ectocarpus sp. Ec32 (brown algae)
5801	GCA_000499425.1	Eimeria acervulina (apicomplexans)
5804	GCA_000499605.1	Eimeria maxima (apicomplexans)
44415	GCA_000499745.1	Eimeria mitis (apicomplexans)
51315	GCA_000499385.1	Eimeria necatrix (apicomplexans)
5802	GCA_000499545.1	Eimeria tenella (apicomplexans)
280463	GCA_000372725.1	Emiliania huxleyi CCMP1516 (haptophytes)
370354	GCA_000209125.2	Entamoeba dispar SAW760 (eukaryotes)
294381	GCA_000208925.2	Entamoeba histolytica HM-1:IMSS (eukaryotes)
370355	GCA_000330505.1	Entamoeba invadens IP1 (eukaryotes)
41668	GCA_002914575.1	Entamoeba moshkovskii (eukaryotes)
1076696	GCA_000257125.1	Entamoeba nuttalli P19 (eukaryotes)
1519565	GCA_002217885.1	Fistulifera solaris (diatoms)
691883	GCA_000388065.2	Fonticula alba (eukaryotes)
1660239	GCA_001724245.1	Fonticula-like sp. SCN 57-25 (eukaryotes)
2109649	GCA_900642245.1	Fragilaria radians (diatoms)
635003	GCA_001750085.1	Fragilariopsis cylindrus CCMP1102 (diatoms)
1389228	GCA_006232345.1	Galdieria phlegrea (red algae)
130081	GCA_000341285.1	Galdieria sulphuraria (red algae)
431595	GCA_000143045.1	Globisporangium ultimum DAOM BR144 (oomycetes)
2058621	GCA_003354225.1	Globobulimina sp. (forams)
1255295	GCA_003573635.1	Goniomonas avonlea (cryptomonads)
448386	GCA_003194525.1	Gracilariopsis chorda (red algae)
2782	GCA_003346895.1	Gracilariopsis lemaneiformis (red algae)

110365	GCA_000223845.4	Gregarina niphandrodes (apicomplexans)
905079	GCA_000315625.1	Guillardia theta CCMP2712 (cryptomonads)
2056406	GCA_004335955.1	Halamphora sp. AAB (diatoms)
2056407	GCA_004335815.1	Halamphora sp. MG8b (diatoms)
357350	GCA_001687465.1	Halocafeteria seosinensis (eukaryotes)
99158	GCA_000258005.2	Hammondia hammondi (apicomplexans)
1140117	GCA_000498555.1	Heterococcus sp. DN1 (yellow-green algae)
670386	GCA_000004825.1	Heterostelium album PN500 (cellular slime molds)
1037917	GCA_003667245.1	Heterostelium multicystogenum (cellular slime molds)
2315210	GCA_002897355.1	Hondaea fermentalgiana (slime nets)
559515	GCA_000173235.2	Hyaloperonospora arabidopsidis Emoy2 (oomycetes)
2996	GCA_900617105.1	Hydrurus foetidus (golden algae)
42384	GCA_900088475.1	Hyphochytrium catenoides (eukaryotes)
39843	GCA_002751075.1	Ichthyophonus hoferi (eukaryotes)
5932	GCA_000220395.1	Ichthyophthirius multifiliis (ciliates)
1932427	GCA_002811675.1	Ichthyosporea sp. XGB-2017a (eukaryotes)
38544	GCA_002205965.2	Kappaphycus alvarezii (red algae)
797122	GCA_003568945.1	Kipferlia bialata (eukaryotes)
4803	GCA_002286825.1	Lagenidium giganteum (oomycetes)
420245	GCA_000002845.2	Leishmania braziliensis MHOM/BR/75/M2904 (kinetoplastids)
5661	GCA_000227135.2	Leishmania donovani (kinetoplastids)
435258	GCA_000002875.2	Leishmania infantum JPCM5 (kinetoplastids)
347515	GCA_000002725.2	Leishmania major strain Friedlin (kinetoplastids)
929439	GCA_000234665.4	Leishmania mexicana MHOM/GT/2001/U1103 (kinetoplastids)
5679	GCA_000755165.1	Leishmania panamensis (kinetoplastids)
1653305	GCA_001655205.1	Lenisia limosa (eukaryotes)
157538	GCA_001293395.1	Leptomonas pyrrhocoris (kinetoplastids)
297518	GCA_900291995.1	Licmophora abbreviata (diatoms)
1313166	GCA_000765095.1	Mastigamoeba balamuthi ATTC 30984 (eukaryotes)
453998	GCA_001643675.1	Monocercomonoides sp. PA203 (eukaryotes)
431895	GCA_000002865.1	Monosiga brevicollis MX1 (choanoflagellates)
5763	GCA_000499105.1	Naegleria fowleri (brain-eating amoeba)
5762	GCA_000004985.1	Naegleria gruberi (eukaryotes)
51637	GCA_003324165.1	Naegleria lovaniensis (eukaryotes)
1093141	GCA_000240725.1	Nannochloropsis gaditana CCMP526 (eukaryotes)
1259849	GCA_004335405.1	Nannochloropsis granulata CCMP529 (eukaryotes)
120807	GCA_001614225.1	Nannochloropsis limnetica (eukaryotes)
145522	GCA_004519485.1	Nannochloropsis oceanica (eukaryotes)
1259847	GCA_004335455.1	Nannochloropsis oculata CCMP525 (eukaryotes)
1027362	GCA_004335465.1	Nannochloropsis salina CCMP537 (eukaryotes)
572307	GCA_000208865.2	Neospora caninum Liverpool (apicomplexans)
2483409	GCA_001712635.2	Nothophytophthora sp. Chile5 (oomycetes)
1440115	GCA_002980425.1	Paralagenidium karlingii (oomycetes)
5888	GCA_000165425.1	Paramecium tetraurelia (ciliates)
180228	GCA_002151225.1	Paramoeba pemaquidensis (eukaryotes)

423536	GCA_000006405.1	Perkinsus marinus ATCC 50983 (eukaryotes)
622444	GCA_002864105.1	Peronospora belbahrii (oomycetes)
542832	GCA_003843895.1	Peronospora effusa (oomycetes)
230439	GCA_002099245.1	Peronospora tabacina (oomycetes)
556484	GCA_000150955.2	Phaeodactylum tricornutum CCAP 1055/1 (diatoms)
5791	GCA_000413255.3	Physarum polycephalum (plasmodial slime molds)
1642459	GCA_001314435.1	Phytophthora agathidicida (oomycetes)
29920	GCA_003287315.1	Phytophthora cactorum (oomycetes)
53983	GCA_000443045.1	Phytophthora cambivora (oomycetes)
763924	GCA_000325885.1	Phytophthora capsici LT1534 (oomycetes)
4785	GCA_001314365.1	Phytophthora cinnamomi (oomycetes)
129350	GCA_002288995.1	Phytophthora colocasiae (oomycetes)
4786	GCA_000468175.2	Phytophthora cryptogea (oomycetes)
53985	GCA_000686205.4	Phytophthora fragariae (oomycetes)
403677	GCA_000142945.1	Phytophthora infestans T30-4 (potato late blight agent)
325452	GCA_000448265.2	Phytophthora kernoviae (oomycetes)
1254522	GCA_000318465.2	Phytophthora lateralis MPF4 (oomycetes)
100870	GCA_002812785.1	Phytophthora litchii (oomycetes)
4795	GCA_002215365.1	Phytophthora megakarya (oomycetes)
596104	GCA_001314345.1	Phytophthora multivora (oomycetes)
4790	GCA_003328465.1	Phytophthora nicotianae (buckeye rot agent)
611791	GCA_002911725.1	Phytophthora palmivora var. palmivora (oomycetes)
761204	GCA_000247585.2	Phytophthora parasitica INRA-310 (oomycetes)
538568	GCA_000500225.2	Phytophthora pinifolia (oomycetes)
1131323	GCA_000751395.2	Phytophthora pisi (oomycetes)
639000	GCA_002247145.1	Phytophthora plurivora (oomycetes)
1330343	GCA_001314425.1	Phytophthora pluvialis (oomycetes)
164328	GCA_002968915.1	Phytophthora ramorum (Sudden oak death agent)
129364	GCA_000687305.2	Phytophthora rubi (oomycetes)
67593	GCA_000149755.2	Phytophthora sojae (oomycetes)
1690101	GCA_001314375.1	Phytophthora taxon totara (oomycetes)
299392	GCA_000439335.1	Phytophthora x alni (oomycetes)
907947	GCA_003413675.1	Phytopythium vexans (oomycetes)
1635159	GCA_001600495.1	Pilasporangium apinafurcum (oomycetes)
749231	GCA_002812295.1	Pirum gemmata (eukaryotes)
1890364	GCA_003024175.1	Planoprotostelium fungivorum (eukaryotes)
37360	GCA_003833335.1	Plasmodiophora brassicae (eukaryotes)
5823	GCA_900002375.1	Plasmodium berghei ANKA (apicomplexans)
31271	GCA_900002335.1	Plasmodium chabaudi chabaudi (apicomplexans)
208452	GCA_001680005.1	Plasmodium coatneyi (apicomplexans)
1120755	GCA_000321355.1	Plasmodium cynomolgi strain B (apicomplexans)
36329	GCA_000002765.2	Plasmodium falciparum 3D7 (malaria parasite P. falciparum)
5857	GCA_000956335.1	Plasmodium fragile (apicomplexans)
647221	GCA_001602025.1	Plasmodium gaboni (apicomplexans)
1237626	GCA_000524495.1	Plasmodium inui San Antonio 1 (apicomplexans)
5851	GCA_000006355.1	Plasmodium knowlesi strain H (apicomplexans)

5854	GCA_001601855.1	Plasmodium reichenowi (apicomplexans)
54757	GCA_000709005.1	Plasmodium vinckei vinckei (apicomplexans)
5855	GCA_000002415.2	Plasmodium vivax (malaria parasite P. vivax)
5861	GCA_900002385.1	Plasmodium yoelii (apicomplexans)
4781	GCA_900000015.1	Plasmopara halstedii (oomycetes)
1196443	GCA_003676415.1	Plasmopara muralis (oomycetes)
162140	GCA_003640625.1	Plasmopara obducens (oomycetes)
143451	GCA_001695595.3	Plasmopara viticola (oomycetes)
41456	GCA_003693705.1	Polymyxa betae (eukaryotes)
133409	GCA_000277445.1	Polysphondylium violaceum (cellular slime molds)
2786	GCA_002049455.2	Porphyra umbilicalis (laver)
35688	GCA_000397085.1	Porphyridium purpureum (red algae)
42746	GCA_002245135.1	Proteromonas lacertae (eukaryotes)
472931	GCA_002081555.1	Protostelium mycophagum (eukaryotes)
183589	GCA_900660405.1	Pseudo-nitzschia multistriata (diatoms)
143453	GCA_000252605.1	Pseudoperonospora cubensis (oomycetes)
162120	GCA_003991265.1	Pseudoperonospora humuli (oomycetes)
1223555	GCA_000387445.2	Pythium aphanidermatum DAOM BR444 (oomycetes)
1223556	GCA_000387505.2	Pythium arrhenomanes ATCC 12531 (oomycetes)
429562	GCA_003730235.1	Pythium guiyangense (oomycetes)
114742	GCA_001029375.1	Pythium insidiosum (oomycetes)
1223557	GCA_000387425.2	Pythium irregulare DAOM BR486 (oomycetes)
1223558	GCA_000387465.2	Pythium iwayamai DAOM BR242034 (oomycetes)
41045	GCA_005966545.1	Pythium oligandrum (oomycetes)
82946	GCA_001922765.1	Pythium periplocum (oomycetes)
82926	GCA_006386115.1	Pythium splendens (oomycetes)
46433	GCA_000512085.1	Reticulomyxa filosa (forams)
1660234	GCA_001724265.1	Rhizaria sp. SCN 62-66 (eukaryotes)
361140	GCA_900092235.1	Rostrostelium ellipticum (cellular slime molds)
88149	GCA_000978595.1	Saccharina japonica (brown algae)
946362	GCA_000188695.1	Salpingoeca rosetta (choanoflagellates)
1156394	GCA_000281045.1	Saprolegnia diclina VS20 (oomycetes)
695850	GCA_000151545.2	Saprolegnia parasitica CBS 223.65 (oomycetes)
1573607	GCA_000818945.1	Schizochytrium sp. CCTCC M209059 (slime nets)
559965	GCA_004764695.1	Schizochytrium sp. TIO01 (slime nets)
162130	GCA_002933675.1	Sclerospora graminicola (oomycetes)
361096	GCA_003667305.1	Speleostelium caveatum (cellular slime molds)
667725	GCA_001186125.1	Sphaeroforma arctica JP610 (eukaryotes)
1618205	GCA_001586965.3	Sphaeroforma sirkka (eukaryotes)
348837	GCA_000497125.1	Spironucleus salmonicida (diplomonads)
70186	GCA_900404475.1	Spongospora subterranea (eukaryotes)
1735743	GCA_900128395.1	Stramenopiles sp. TOSAG23-2 (eukaryotes)
1735745	GCA_900128585.1	Stramenopiles sp. TOSAG23-3 (eukaryotes)
1735747	GCA_900128565.1	Stramenopiles sp. TOSAG23-6 (eukaryotes)
1735744	GCA_900128575.1	Stramenopiles sp. TOSAG41-1 (eukaryotes)
361085	GCA_900092255.1	Synstelium polycarpum (cellular slime molds)

312017	GCA_000189635.1	Tetrahymena thermophila SB210 (ciliates)
159749	GCA_000296195.2	Thalassiosira oceanica (diatoms)
296543	GCA_000149405.2	Thalassiosira pseudonana CCMP1335 (diatoms)
5874	GCA_000003225.1	Theileria annulata (apicomplexans)
1537102	GCA_000342415.1	Theileria equi strain WA (apicomplexans)
869250	GCA_000740895.1	Theileria orientalis strain Shintoku (apicomplexans)
5875	GCA_000165365.1	Theileria parva (apicomplexans)
672127	GCA_002154235.1	Thraustochytrium sp. ATCC 26185 (slime nets)
74557	GCA_002081575.1	Thraustotheca clavata (oomycetes)
361077	GCA_001606155.1	Tieghemostelium lacteum (cellular slime molds)
508771	GCA_000006565.2	Toxoplasma gondii ME49 (apicomplexans)
412133	GCA_000002825.1	Trichomonas vaginalis G3 (trichomonads)
1144522	GCA_001839685.1	Tritrichomonas foetus (eukaryotes)
679716	GCA_000210295.1	Trypanosoma brucei gambiense DAL972 (kinetoplastids)
5693	GCA_000209065.1	Trypanosoma cruzi (kinetoplastids)
71804	GCA_000691245.1	Trypanosoma grayi (kinetoplastids)

Fungi

taxid	Acession	Species
45518	GCA_002068075.1	[Candida] aaseri (budding yeasts)
983967	GCA_001661425.1	[Candida] arabinofermentans NRRL YB-2248 (budding yeasts)
391823	GCA_003708745.2	[Candida] ascalaphidarum (budding yeasts)
304220	GCA_003705555.1	[Candida] athensensis (budding yeasts)
498019	GCA_002775015.1	[Candida] auris (budding yeasts)
391824	GCA_003706955.2	[Candida] blattae (budding yeasts)
5477	GCA_001599335.1	[Candida] boidinii (budding yeasts)
1279114	GCA_001077315.1	[Candida] bracarensis CBS 10154 (budding yeasts)
374178	GCA_001599235.1	[Candida] carpophila (budding yeasts)
1279086	GCA_001046935.1	[Candida] castellii CBS 4332 (budding yeasts)
1231522	GCA_002926085.1	[Candida] duobushaemulonis (budding yeasts)
1213352	GCA_001649435.1	[Candida] ethanolica M2 (budding yeasts)
52254	GCA_003706895.1	[Candida] fragi (budding yeasts)
45553	GCA_003045245.1	[Candida] galacta (budding yeasts)
5478	GCA_000002545.2	[Candida] glabrata (budding yeasts)
1323752	GCA_003708755.1	[Candida] golubevii (budding yeasts)
271357	GCA_003708985.2	[Candida] gorgasii (budding yeasts)
87895	GCA_003708205.1	[Candida] gotoi (budding yeasts)
45357	GCA_002926055.1	[Candida] haemulonis (budding yeasts)
197672	GCA_002893645.1	[Candida] hawaiiana (budding yeasts)
487108	GCA_003708405.1	[Candida] heveicola (budding yeasts)
49327	GCA_003706695.2	[Candida] incommunis (budding yeasts)
52247	GCA_004931855.1	[Candida] inconspicua (budding yeasts)
45354	GCA_900106115.1	[Candida] intermedia (budding yeasts)
1807681	GCA_002370145.1	[Candida] ipomoeae (budding yeasts)
49329	GCA_003706615.2	[Candida] montana (budding yeasts)
130814	GCA_003708295.2	[Candida] mycetangii (budding yeasts)
1279115	GCA_001046915.1	[Candida] nivariensis CBS 9983 (budding yeasts)
115786	GCA_003708145.2	[Candida] orba (budding yeasts)
46584	GCA_003707785.2	[Candida] oregonensis (budding yeasts)
135456	GCA_003706755.2	[Candida] ponderosae (budding yeasts)
418784	GCA_003013735.1	[Candida] pseudohaemulonis (budding yeasts)
45577	GCA_900186205.1	[Candida] psychrophila (budding yeasts)
77849	GCA_003707425.2	[Candida] restingae (budding yeasts)
45581	GCA_003708185.2	[Candida] rhagii (budding yeasts)
45587	GCA_003707405.1	[Candida] schatavii (budding yeasts)
49334	GCA_001599115.1	[Candida] sorboxylosa (budding yeasts)
56415	GCA_003707265.1	[Candida] stellimalicola (budding yeasts)
51927	GCA_001599255.1	[Candida] succiphila (budding yeasts)
130808	GCA_003708705.1	[Candida] tammaniensis (budding yeasts)
45599	GCA_002893725.1	[Candida] torresii (budding yeasts)
51918	GCA_900465365.1	[Candida] vartiovaarae (budding yeasts)
560360	GCA_003708715.2	[Candida] wancherniae (budding yeasts)

1235602	GCA_003708765.1	[Kloeckera] hatyaiensis (budding yeasts)
660122	GCA_000151355.1	[Nectria] haematococca mpVI 77-13-4 (ascomycetes)
196907	GCA_000787455.1	[Talaromyces] leycettanus (ascomycetes)
4829	GCA_900079185.1	Absidia glauca (fungi)
90262	GCA_002105175.1	Absidia repens (fungi)
215250	GCA_003144295.1	Acaromyces ingoldii (basidiomycetes)
1172177	GCA_004114255.1	Acaulopage tetraceros (fungi)
43968	GCA_003707435.1	Aciculoconidium aculeatum (budding yeasts)
1036760	GCA_000222935.2	Aciculosporium take MAFF-241224 (ascomycetes)
245562	GCA_003545705.1	Acidomyces richmondensis (ascomycetes)
857340	GCA_000769265.1	Acremonium chrysogenum ATCC 11550 (ascomycetes)
45276	GCA_001599815.1	Acremonium furcatum (ascomycetes)
64647	GCA_001599635.1	Actinomucor elegans (fungi)
2518135	GCA_004216415.1	Aeminium ludgeri (ascomycetes)
2607230	GCA_002718315.1	Agaricales sp. HM26-F1 (basidiomycetes)
936046	GCA_000300575.2	Agaricus bisporus var. bisporus H97 (basidiomycetes)
64608	GCA_003316655.1	Agrocybe cylindracea (basidiomycetes)
80570	GCA_003314395.1	Albatrellus ellisii (basidiomycetes)
2175974	GCA_003314695.1	Albatrellus sp. MG142 (basidiomycetes)
1635095	GCA_001600555.1	Albophoma yamanashiensis (ascomycetes)
54196	GCA_001600815.1	Alloascoidea hylecoeti (budding yeasts)
578462	GCA_000151295.1	Allomyces macrogynus ATCC 38327 (blastocladiomycetes)
5599	GCA_001642055.1	Alternaria alternata (ascomycetes)
156630	GCA_004154835.1	Alternaria arborescens (ascomycetes)
29911	GCA_004936725.1	Alternaria brassicae (ascomycetes)
29001	GCA_002796735.1	Alternaria brassicicola (ascomycetes)
230004	GCA_001950455.1	Alternaria consortialis (ascomycetes)
167740	GCA_004156025.1	Alternaria gaisen (ascomycetes)
48100	GCA_002952155.1	Alternaria solani (ascomycetes)
1678846	GCA_003574525.1	Alternaria sp. MG1 (ascomycetes)
119927	GCA_004156035.1	Alternaria tenuissima (ascomycetes)
87325	GCA_001983365.1	Amanita bisporigera (destroying angel)
1457509	GCA_001691785.2	Amanita brunnescens Koide BX004 (basidiomycetes)
1457513	GCA_001691775.2	Amanita inopinata Kibby_2008 (basidiomycetes)
1417757	GCA_000497225.1	Amanita jacksonii TRTC168611 (basidiomycetes)
946122	GCA_000827485.1	Amanita muscaria Koide BX008 (fly agaric)
67723	GCA_001983385.1	Amanita phalloides (death cap)
1457511	GCA_001691755.2	Amanita polypyramis BW_CC (basidiomycetes)
67725	GCA_003316615.1	Amanita pseudoporphyria (basidiomycetes)
703135	GCA_002554575.1	Amanita thiersii Skay4041 (basidiomycetes)
113399	GCA_001430935.1	Amauroascus mutatus (ascomycetes)
89421	GCA_001430945.1	Amauroascus niger (ascomycetes)
44383	GCA_002778035.1	Ambrosiella xylebori (ascomycetes)
53487	GCA_003705185.1	Ambrosiozyma ambrosiae (budding yeasts)
489715	GCA_001599075.1	Ambrosiozyma kashinagicola (budding yeasts)
904902	GCA_003706635.1	Ambrosiozyma maleeae (budding yeasts)

43982	GCA_001599995.1	Ambrosiozyma monospora (budding yeasts)
1382206	GCA_003706315.2	Ambrosiozyma oregonensis (budding yeasts)
43984	GCA_003707675.2	Ambrosiozyma philentoma (budding yeasts)
489716	GCA_003707895.2	Ambrosiozyma pseudovanderkliftii (budding yeasts)
489717	GCA_003705225.1	Ambrosiozyma vanderkliftii (budding yeasts)
1934353	GCA_004802645.1	Amesia nigricolor (ascomycetes)
857342	GCA_003019875.1	Amorphotheca resinae ATCC 22711 (creosote fungus)
1866961	GCA_001875675.1	Amphiamblys sp. WSBS2006 (microsporidians)
743113	GCA_004123355.1	Amphirosellinia nigrospora (ascomycetes)
1754192	GCA_002104895.1	Anaeromyces robustus (chytrids)
1288291	GCA_000385875.2	Anncaliia algerae PRA339 (microsporidians)
326628	GCA_003314315.1	Annulohypoxylon stygium (ascomycetes)
1277687	GCA_000417875.1	Anthracocystis flocculosa PF-1 (smut fungi)
2447956	GCA_004802725.1	Antrodiella citrinella (basidiomycetes)
82513	GCA_001600295.1	Apiotrichum brassicae (basidiomycetes)
82514	GCA_001599015.1	Apiotrichum domesticum (basidiomycetes)
1105092	GCA_001600315.1	Apiotrichum gamsii (basidiomycetes)
82516	GCA_001600335.1	Apiotrichum gracile (basidiomycetes)
82520	GCA_001600735.1	Apiotrichum laibachii (basidiomycetes)
82521	GCA_001598995.1	Apiotrichum montevideense (basidiomycetes)
105984	GCA_003942205.1	Apiotrichum porosum (basidiomycetes)
105713	GCA_001600595.1	Apiotrichum veenhuisii (basidiomycetes)
1274791	GCA_000696995.1	Apophysomyces elegans B7760 (fungi)
1274790	GCA_000696975.1	Apophysomyces trapeziformis B9324 (fungi)
760013	GCA_002749535.1	Apophysomyces variabilis (fungi)
1634478	GCA_003415625.1	Aquanectria penicillioides (ascomycetes)
2259763	GCA_003988855.1	Arachnopeziza araneosa (ascomycetes)
47426	GCA_900157415.1	Armillaria cepistipes (basidiomycetes)
108572	GCA_001679825.1	Armillaria fuscipes (basidiomycetes)
47427	GCA_002307695.1	Armillaria gallica (basidiomycetes)
47428	GCA_900157425.1	Armillaria ostoyae (basidiomycetes)
1076256	GCA_002307675.1	Armillaria solidipes (basidiomycetes)
39680	GCA_002989075.1	Arthonia radiata (ascomycetes)
97331	GCA_004000055.1	Arthrobotrys flagrans (ascomycetes)
756982	GCA_000225545.1	Arthrobotrys oligospora ATCC 24927 (ascomycetes)
1758292	GCA_003614865.1	Arthrocladium fulminans (ascomycetes)
253309	GCA_003415645.1	Articulospora tetracladia (ascomycetes)
1160509	GCA_003788565.2	Ascobolus immersus RN42 (ascomycetes)
372025	GCA_004335285.1	Ascochyta fabae (ascomycetes)
205686	GCA_004011705.1	Ascochyta lentis (ascomycetes)
5454	GCA_004011695.1	Ascochyta rabiei (ascomycetes)
1677696	GCA_004335155.1	Ascochyta viciae (ascomycetes)
487627	GCA_004335205.1	Ascochyta viciae-villosae (ascomycetes)
1016881	GCA_000328965.1	Ascocoryne sarcoides NRRL 50072 (ascomycetes)
341454	GCA_004786065.1	Ascodesmis nigricans (ascomycetes)
1301101	GCA_001600695.1	Ascoidea asiatica (budding yeasts)

1344418	GCA_001661345.1	Ascoidea rubescens DSM 1968 (budding yeasts)
392613	GCA_001636715.1	Ascosphaera apis ARSEF 7405 (ascomycetes)
1448322	GCA_003184765.1	Aspergillus aculeatinus CBS 121060 (ascomycetes)
690307	GCA_001890905.1	Aspergillus aculeatus ATCC 16872 (ascomycetes)
2562445	GCA_001931935.1	Aspergillus aff. floccosus IMV 01167 (ascomycetes)
656916	GCA_002749805.1	Aspergillus arachidicola (ascomycetes)
105351	GCA_003850985.1	Aspergillus awamori (ascomycetes)
109264	GCA_001792695.1	Aspergillus bombycis (ascomycetes)
767769	GCA_001889945.1	Aspergillus brasiliensis CBS 101740 (ascomycetes)
1450534	GCA_003184695.1	Aspergillus brunneoviolaceus CBS 621.78 (ascomycetes)
454130	GCA_001511075.1	Aspergillus calidoustus (ascomycetes)
1392248	GCA_002847485.1	Aspergillus campestris IBT 28561 (ascomycetes)
41067	GCA_002847045.1	Aspergillus candidus (ascomycetes)
602072	GCA_001990825.1	Aspergillus carbonarius ITEM 5010 (ascomycetes)
1884262	GCA_004769165.1	Aspergillus cejpii (ascomycetes)
182096	GCA_001599875.1	Aspergillus chevalieri (ascomycetes)
344612	GCA_000002715.1	Aspergillus clavatus NRRL 1 (ascomycetes)
1448317	GCA_003184835.1	Aspergillus costaricaensis CBS 115574 (ascomycetes)
573508	GCA_001693355.1	Aspergillus cristatus (ascomycetes)
1448320	GCA_003184645.1	Aspergillus ellipticus CBS 707.79 (ascomycetes)
1448314	GCA_003184535.1	Aspergillus eucalypticola CBS 122712 (ascomycetes)
1448319	GCA_003184825.1	Aspergillus fijiensis CBS 313.89 (ascomycetes)
331117	GCA_000149645.2	Aspergillus fischeri NRRL 181 (ascomycetes)
332952	GCA_000006275.2	Aspergillus flavus NRRL3357 (ascomycetes)
330879	GCA_000002655.1	Aspergillus fumigatus Af293 (ascomycetes)
1160497	GCA_001890805.1	Aspergillus glaucus CBS 516.65 (ascomycetes)
1873369	GCA_001696595.1	Aspergillus hancockii (ascomycetes)
1448321	GCA_003184545.1	Aspergillus heteromorphus CBS 117.55 (ascomycetes)
1450537	GCA_003184865.1	Aspergillus homomorphus CBS 101889 (ascomycetes)
1448316	GCA_003184845.1	Aspergillus ibericus CBS 121593 (ascomycetes)
1450541	GCA_003184685.1	Aspergillus indologenus CBS 114.80 (ascomycetes)
1448312	GCA_003184785.1	Aspergillus japonicus CBS 114.51 (ascomycetes)
1033177	GCA_000239835.2	Aspergillus kawachii IFO 4308 (ascomycetes)
293939	GCA_001445615.1	Aspergillus lentulus (ascomycetes)
1137211	GCA_001890685.1	Aspergillus luchuensis CBS 106.47 (ascomycetes)
1810919	GCA_003369625.1	Aspergillus mulundensis (ascomycetes)
91482	GCA_003116565.1	Aspergillus neoellipticus (ascomycetes)
1448310	GCA_003184625.1	Aspergillus neoniger CBS 115656 (ascomycetes)
227321	GCA_000149205.2	Aspergillus nidulans FGSC A4 (ascomycetes)
5061	GCA_000002855.2	Aspergillus niger (ascomycetes)
1509407	GCA_001204775.1	Aspergillus nomius NRRL 13137 (ascomycetes)
1392255	GCA_002847465.1	Aspergillus novofumigatus IBT 16806 (ascomycetes)
1392256	GCA_002846915.2	Aspergillus ochraceoroseus IBT 24754 (ascomycetes)
40380	GCA_004849945.1	Aspergillus ochraceus (ascomycetes)
2184023	GCA_003719415.1	Aspergillus olivimuriae (ascomycetes)
510516	GCA_000184455.3	Aspergillus oryzae RIB40 (ascomycetes)

1403190	GCA_000956085.1	Aspergillus parasiticus SU-1 (ascomycetes)
306094	GCA_002215965.1	Aspergillus persii (ascomycetes)
1353007	GCA_003344505.1	Aspergillus phoenicis ATCC 13157 (ascomycetes)
1448313	GCA_003184755.1	Aspergillus piperis CBS 112811 (ascomycetes)
1565506	GCA_002927005.1	Aspergillus pseudoterreus (ascomycetes)
308745	GCA_000986645.1	Aspergillus rambellii (ascomycetes)
1388766	GCA_000600275.1	Aspergillus ruber CBS 135680 (ascomycetes)
1450539	GCA_003184585.1	Aspergillus saccharolyticus JOP 1030-1 (ascomycetes)
2070753	GCA_003589665.1	Aspergillus sclerotialis (ascomycetes)
1448318	GCA_003184635.1	Aspergillus sclerotiicarbonarius CBS 121057 (ascomycetes)
1450535	GCA_003184525.1	Aspergillus sclerotioniger CBS 115572 (ascomycetes)
138282	GCA_000530345.1	Aspergillus sclerotiorum (ascomycetes)
927772	GCA_000226655.1	Aspergillus sojae NBRC 4239 (ascomycetes)
2153246	GCA_003138035.1	Aspergillus sp. MA 6037 (ascomycetes)
2153248	GCA_003138005.1	Aspergillus sp. MA 6041 (ascomycetes)
2484740	GCA_003719405.1	Aspergillus sp. SS-2018a (ascomycetes)
1662659	GCA_001044295.1	Aspergillus sp. Z5 (ascomycetes)
1810908	GCA_003574815.1	Aspergillus spinulosporus (ascomycetes)
1392250	GCA_002849105.1	Aspergillus steynii IBT 23096 (ascomycetes)
1036612	GCA_001890705.1	Aspergillus sydowii CBS 593.65 (ascomycetes)
482145	GCA_002850765.1	Aspergillus taichungensis (ascomycetes)
1220188	GCA_003426965.1	Aspergillus tanneri (ascomycetes)
341663	GCA_000149615.1	Aspergillus terreus NIH2624 (ascomycetes)
41047	GCA_002237265.2	Aspergillus thermomutatus (ascomycetes)
767770	GCA_001890745.1	Aspergillus tubingensis CBS 134.48 (ascomycetes)
1245748	GCA_002234965.2	Aspergillus turcosus (ascomycetes)
91492	GCA_001078395.1	Aspergillus udagawae (ascomycetes)
40381	GCA_003324175.1	Aspergillus unguis (ascomycetes)
40382	GCA_000812125.1	Aspergillus ustus (ascomycetes)
1448315	GCA_003184745.1	Aspergillus uvarum CBS 121591 (ascomycetes)
1448311	GCA_003184925.1	Aspergillus vadensis CBS 113365 (ascomycetes)
1036611	GCA_001890125.1	Aspergillus versicolor CBS 583.65 (ascomycetes)
1450538	GCA_003184705.1	Aspergillus violaceofuscus CBS 115571 (ascomycetes)
75553	GCA_004368095.1	Aspergillus viridinutans (ascomycetes)
1341132	GCA_003344945.1	Aspergillus welwitschiae (ascomycetes)
1073089	GCA_001890725.1	Aspergillus wentii DTO 134E9 (ascomycetes)
357447	GCA_001307345.1	Aspergillus westerdijkiae (ascomycetes)
39291	GCA_002940785.1	Athelia rolfsii (basidiomycetes)
47741	GCA_000729835.1	Atkinsonella hypoxylon (ascomycetes)
51582	GCA_001008035.1	Atkinsonella texensis (ascomycetes)
46634	GCA_002156615.1	Aureobasidium melanogenum (ascomycetes)
1043004	GCA_000721765.1	Aureobasidium namibiae CBS 147.97 (ascomycetes)
5580	GCA_003336255.1	Aureobasidium pullulans (ascomycetes)
1928478	GCA_001914275.1	Aureobasidium sp. FSWF8-4 (ascomycetes)
1771980	GCA_003992365.1	Aureobasidium sp. P6 (ascomycetes)
1043005	GCA_000721755.1	Aureobasidium subglaciale EXF-2481 (ascomycetes)

29892	GCA_002092955.1	Auricularia auricula-judae (ear fungus)
1579977	GCA_002287115.1	Auricularia heimuer (basidiomycetes)
29893	GCA_003316125.1	Auricularia polytricha (Chinese wood ear)
181123	GCA_003724095.1	Austropuccinia psidii (rust fungi)
984486	GCA_001661335.1	Babjeviella inositovora NRRL Y-12698 (budding yeasts)
139080	GCA_001179705.1	Baeospora myosura (basidiomycetes)
1405085	GCA_000709145.1	Balansia obtecta B249 (ascomycetes)
36038	GCA_003706655.2	Barnettozyma californica (budding yeasts)
96499	GCA_003706665.1	Barnettozyma hawaiiensis (budding yeasts)
53645	GCA_003706705.2	Barnettozyma populi (budding yeasts)
36040	GCA_003707865.1	Barnettozyma pratensis (budding yeasts)
1337058	GCA_003707885.1	Barnettozyma salicaria (budding yeasts)
1230402	GCA_000826855.1	Basidioascus undulatus (basidiomycetes)
1357690	GCA_000697455.1	Basidiobolus heterosporus B8920 (fungi)
1314790	GCA_002104905.1	Basidiobolus meristosporus CBS 931.73 (fungi)
89487	GCA_001599675.1	Basipetospora chlamydospora (ascomycetes)
684364	GCA_000203795.1	Batrachochytrium dendrobatidis JAM81 (chytrids)
1357716	GCA_002006685.1	Batrachochytrium salamandrivorans (chytrids)
717646	GCA_000338955.1	Baudoinia panamericana UAMH 10762 (ascomycetes)
655819	GCA_000280675.1	Beauveria bassiana ARSEF 2860 (ascomycetes)
1081107	GCA_001636735.1	Beauveria brongniartii RCEF 3172 (ascomycetes)
931006	GCA_003267905.1	Beauveria pseudobassiana (ascomycetes)
1481906	GCA_000733645.1	Beauveria rudraprayagi (ascomycetes)
2562442	GCA_001931865.2	Beauveria sp. IMV 00265 (ascomycetes)
124036	GCA_003671435.1	Berkeleyomyces basicola (ascomycetes)
409207	GCA_001599595.1	Beverwykella pulmonaria (ascomycetes)
1938954	GCA_002261195.1	Bifiguratus adelaidae (fungi)
74410	GCA_002286855.1	Bipolaris cookei (ascomycetes)
665024	GCA_000354255.1	Bipolaris maydis ATCC 48331 (southern corn leaf blight pathogen)
930090	GCA_000523455.1	Bipolaris oryzae ATCC 44560 (ascomycetes)
665912	GCA_000338995.1	Bipolaris sorokiniana ND90Pr (ascomycetes)
930091	GCA_000527765.1	Bipolaris victoriae FI3 (ascomycetes)
930089	GCA_000523435.1	Bipolaris zeicola 26-R-13 (ascomycetes)
1850976	GCA_003705795.2	Blastobotrys americana (budding yeasts)
267478	GCA_003705765.2	Blastobotrys mokoenaii (budding yeasts)
378046	GCA_003705745.2	Blastobotrys muscicola (budding yeasts)
44073	GCA_003707525.2	Blastobotrys niveus (budding yeasts)
378051	GCA_003705735.2	Blastobotrys peoriensis (budding yeasts)
44067	GCA_003707485.2	Blastobotrys proliferans (budding yeasts)
1548389	GCA_003705705.1	Blastobotrys raffinosifermentans (budding yeasts)
456365	GCA_003705695.1	Blastobotrys serpentis (budding yeasts)
559297	GCA_000003525.2	Blastomyces dermatitidis ER-3 (ascomycetes)
559298	GCA_000003855.2	Blastomyces gilchristii SLH14081 (ascomycetes)
2060905	GCA_002572885.1	Blastomyces parvus (ascomycetes)
1658174	GCA_003206225.1	Blastomyces percursus (ascomycetes)
2060906	GCA_001014755.1	Blastomyces silverae (ascomycetes)

2164086	GCA_003206725.1	Blastomyces sp. MA-2018 (ascomycetes)
546991	GCA_900239735.1	Blumeria graminis f. sp. hordei DH14 (grass mildew)
388810	GCA_003614705.1	Blyttiomyces helicus (chytrids)
279506	GCA_003316205.1	Boletus bicolor (basidiomycetes)
36056	GCA_003316165.1	Boletus edulis (basidiomycetes)
2175973	GCA_003313885.1	Boletus sp. MG55 (basidiomycetes)
2175972	GCA_003313155.1	Boletus sp. MG95 (basidiomycetes)
374761	GCA_003316055.1	Boletus speciosus (basidiomycetes)
279509	GCA_003316035.1	Boletus subvelutipes (basidiomycetes)
1095465	GCA_004802705.1	Bondarzewia mesenterica (basidiomycetes)
930990	GCA_000697705.1	Botryobasidium botryosum FD-172 SS1 (basidiomycetes)
55169	GCA_004016265.1	Botryosphaeria dothidea (ascomycetes)
2021124	GCA_004016305.1	Botryosphaeria kuwatsukai (ascomycetes)
38488	GCA_004379285.1	Botryotinia calthae (ascomycetes)
54673	GCA_004786275.1	Botryotinia convoluta (ascomycetes)
278944	GCA_004786225.1	Botryotinia narcissicola (ascomycetes)
44070	GCA_003707445.1	Botryozyma nematodophila (budding yeasts)
332648	GCA_000143535.4	Botrytis cinerea B05.10 (ascomycetes)
278938	GCA_004786205.1	Botrytis elliptica (ascomycetes)
182092	GCA_004335055.1	Botrytis fabae (ascomycetes)
278940	GCA_004916875.1	Botrytis galanthina (ascomycetes)
278943	GCA_004786245.1	Botrytis hyacinthi (ascomycetes)
278948	GCA_004786145.1	Botrytis paeoniae (ascomycetes)
87229	GCA_004786265.1	Botrytis porri (ascomycetes)
87230	GCA_004786125.1	Botrytis tulipae (ascomycetes)
37662	GCA_001754015.1	Brettanomyces anomalus (budding yeasts)
5007	GCA_900496985.1	Brettanomyces bruxellensis (budding yeasts)
13368	GCA_003705215.2	Brettanomyces custersianus (budding yeasts)
13370	GCA_900660285.1	Brettanomyces naardenensis (budding yeasts)
1836592	GCA_002018255.1	Bretziella fagacearum (ascomycetes)
157611	GCA_001600095.1	Bullera alba (basidiomycetes)
1325616	GCA_003315995.1	Butyriboletus roseoflavus (basidiomycetes)
5093	GCA_003116535.1	Byssochlamys nivea (ascomycetes)
2066500	GCA_002914405.1	Byssochlamys sp. AF001 (ascomycetes)
2059436	GCA_002242795.1	Byssochlamys sp. BYSS01 (ascomycetes)
2562440	GCA_001931905.2	Byssochlamys sp. IMV 00045 (ascomycetes)
264951	GCA_004022145.1	Byssochlamys spectabilis (ascomycetes)
166112	GCA_001430925.1	Byssoonygena ceratinophila (ascomycetes)
1485229	GCA_003073865.1	Cadophora sp. DSE1049 (ascomycetes)
1802957	GCA_001625345.1	Cairneyella variabilis (ascomycetes)
141855	GCA_003316085.1	Caloboletus calopus (basidiomycetes)
1353952	GCA_001632435.1	Calocera cornea HHB12733 (basidiomycetes)
1330018	GCA_001630345.1	Calocera viscosa TUFC12733 (basidiomycetes)
1868870	GCA_004380935.1	Calonectria henricotiae (ascomycetes)
149392	GCA_002179835.1	Calonectria leucothoes (ascomycetes)
182846	GCA_003031705.1	Calonectria naviculata (ascomycetes)

196064	GCA_004380915.1	Calonectria pseudonaviculata (ascomycetes)
707572	GCA_001879505.1	Calonectria pseudoreteaudii (ascomycetes)
237561	GCA_000182965.3	Candida albicans SC5314 (budding yeasts)
391826	GCA_003706475.2	Candida corydali (budding yeasts)
573826	GCA_000026945.1	Candida dubliniensis CD36 (budding yeasts)
64417	GCA_003707735.1	Candida freyschussii (budding yeasts)
312227	GCA_900535975.1	Candida hispaniensis (budding yeasts)
1245528	GCA_000344705.1	Candida maltosa Xu316 (budding yeasts)
1136231	GCA_000315875.1	Candida orthopsilosis Co 90-125 (budding yeasts)
5480	GCA_000182765.2	Candida parapsilosis (budding yeasts)
39397	GCA_003243815.1	Candida sake (budding yeasts)
52253	GCA_001442715.1	Candida sojae (budding yeasts)
485397	GCA_001950555.1	Candida sp. JCM 15000 (budding yeasts)
1759314	GCA_001005365.1	Candida sp. LDI48194 (budding yeasts)
294747	GCA_000006335.3	Candida tropicalis MYA-3404 (budding yeasts)
5486	GCA_003327735.1	Candida viswanathii (budding yeasts)
409893	GCA_003314335.1	Cantharellus appalachiensis (basidiomycetes)
36066	GCA_003521295.1	Cantharellus cibarius (chanterelle)
57193	GCA_003314235.1	Cantharellus cinnabarinus (basidiomycetes)
104198	GCA_003314295.1	Cantharellus lutescens (basidiomycetes)
4885	GCA_001661515.1	Capniomyces stellatus (fungi)
1182541	GCA_000585585.1	Capronia coronata CBS 617.96 (ascomycetes)
1182542	GCA_000585565.1	Capronia epimyces CBS 606.96 (ascomycetes)
765915	GCA_002102555.1	Catenaria anguillulae PL171 (blastocladiomycetes)
1555241	GCA_003615045.1	Caulochytrium protostelioides (chytrids)
794803	GCA_001692895.1	Cenococcum geophilum 1.58 (ascomycetes)
43958	GCA_003706495.1	Cephaloascus albidus (budding yeasts)
27311	GCA_003707825.2	Cephaloascus fragrans (budding yeasts)
401625	GCA_900000165.1	Ceraceosorus bombacis (basidiomycetes)
1522189	GCA_003144195.1	Ceraceosorus guamensis (basidiomycetes)
1202554	GCA_000372705.1	Cerataphis brasiliensis yeast-like symbiont (ascomycetes)
553390	GCA_002778105.1	Ceratocystiopsis brevicomis (ascomycetes)
360147	GCA_001676865.1	Ceratocystiopsis minuta (ascomycetes)
72027	GCA_001640685.1	Ceratocystis adiposa (ascomycetes)
357446	GCA_002742255.2	Ceratocystis albifundus (ascomycetes)
386457	GCA_002776505.1	Ceratocystis cacaofunesta (ascomycetes)
633669	GCA_001513815.1	Ceratocystis eucalypticola (ascomycetes)
1035309	GCA_000389695.3	Ceratocystis fimbriata CBS 114723 (ascomycetes)
312341	GCA_002018265.1	Ceratocystis harringtonii (ascomycetes)
929235	GCA_006408425.1	Ceratocystis manginecans (ascomycetes)
88771	GCA_000978885.1	Ceratocystis platani (ascomycetes)
312343	GCA_003449175.1	Ceratocystis smalleyi (ascomycetes)
357750	GCA_002933655.1	Cercospora berteroae (ascomycetes)
122368	GCA_002742065.1	Cercospora beticola (ascomycetes)
1268270	GCA_000347735.1	Cercospora canescens BHU (ascomycetes)
1868926	GCA_005356885.1	Cercospora cf. flagellaris (ascomycetes)

1868927	GCA_005356805.1	Cercospora cf. sigesbeckiae (ascomycetes)
84275	GCA_005356855.1	Cercospora kikuchii (ascomycetes)
29003	GCA_002994015.1	Cercospora nicotianae (ascomycetes)
438356	GCA_004299825.1	Cercospora sojina (ascomycetes)
348901	GCA_002844615.1	Cercospora zeina (ascomycetes)
83788	GCA_003521265.1	Cetradonia linearis (ascomycetes)
904709	GCA_001752565.1	Chaetomium cochliodes (ascomycetes)
306901	GCA_000143365.1	Chaetomium globosum CBS 148.51 (ascomycetes)
759272	GCA_000221225.1	Chaetomium thermophilum var. thermophilum DSM 1495 (ascomycetes)
2249419	GCA_003709865.1	Chaetothyriales sp. CBS 132003 (ascomycetes)
2249418	GCA_003709845.1	Chaetothyriales sp. CBS 134916 (ascomycetes)
2249417	GCA_003693665.1	Chaetothyriales sp. CBS 134920 (ascomycetes)
2249420	GCA_003709825.1	Chaetothyriales sp. CBS 135597 (ascomycetes)
112131	GCA_001599435.1	Chalaropsis thielavioides (ascomycetes)
1914213	GCA_003316485.1	Chiua virens (basidiomycetes)
163795	GCA_003988815.1	Chlorencoelia torta (ascomycetes)
296797	GCA_002276475.2	Chlorociboria aeruginascens (green elfcup)
101091	GCA_001683725.1	Choanephora cucurbitarum (fungi)
1336337	GCA_003788595.2	Choiromyces venosus 120613-1 (ascomycetes)
58369	GCA_004354395.1	Chondrostereum purpureum (basidiomycetes)
85976	GCA_003314275.1	Chroogomphus rutilus (basidiomycetes)
354353	GCA_001051155.1	Chrysoporthe austroafricana (ascomycetes)
305400	GCA_004802525.1	Chrysoporthe cubensis (ascomycetes)
764597	GCA_001513825.1	Chrysoporthe deuterocubensis (ascomycetes)
264361	GCA_001430955.1	Chrysosporium queenslandicum (ascomycetes)
647257	GCA_001247705.1	Ciborinia camelliae (ascomycetes)
1046750	GCA_003706345.1	Citeromyces hawaiiensis (budding yeasts)
36909	GCA_003705165.1	Citeromyces matritensis (budding yeasts)
109331	GCA_003706365.1	Citeromyces siamensis (budding yeasts)
767780	GCA_004303015.1	Cladobotryum protrusum (ascomycetes)
1353983	GCA_000444155.1	Cladonia macilenta KoLRI003786 (ascomycetes)
1400770	GCA_000482085.2	Cladonia metacorallifera KoLRI002260 (ascomycetes)
111670	GCA_006146055.1	Cladonia rangiferina (ascomycetes)
174080	GCA_002927785.1	Cladonia uncialis (ascomycetes)
1442370	GCA_000835475.1	Cladophialophora bantiana CBS 173.52 (ascomycetes)
1279043	GCA_000365165.2	Cladophialophora carrionii CBS 160.54 (ascomycetes)
569365	GCA_000835495.1	Cladophialophora immunda (ascomycetes)
1182543	GCA_000585535.1	Cladophialophora psammophila CBS 110553 (ascomycetes)
1182544	GCA_000585515.1	Cladophialophora yegresii CBS 114405 (ascomycetes)
2565360	GCA_001931875.2	Cladosporiaceae sp. IMV 00236 (ascomycetes)
29917	GCA_002901145.1	Cladosporium cladosporioides (ascomycetes)
1116209	GCA_003614995.1	Cladosporium phlei (ascomycetes)
2048672	GCA_002921095.1	Cladosporium sp. SL-16 (ascomycetes)
1151026	GCA_000261425.2	Cladosporium sphaerospermum UM 843 (ascomycetes)
1436886	GCA_001465935.1	Clarireedia homoeocarpa (ascomycetes)

264083	GCA_001179745.1	Clavaria fumosa (basidiomycetes)
1967640	GCA_004016085.1	Claviceps aff. purpurea (ascomycetes)
1036761	GCA_000223055.1	Claviceps fusiformis PRL 1980 (ascomycetes)
1294629	GCA_004016475.1	Claviceps humidiphila (ascomycetes)
1294628	GCA_004016465.1	Claviceps microcephala (ascomycetes)
877506	GCA_000223175.2	Claviceps paspali RRC 1481 (paspalum staggers ergot)
1111077	GCA_000347355.1	Claviceps purpurea 20.1 (ergot fungus)
1294630	GCA_004016175.1	Claviceps spartinae (ascomycetes)
46582	GCA_003707795.1	Clavispora fructus (budding yeasts)
306902	GCA_000003835.1	Clavispora lusitaniae ATCC 42720 (budding yeasts)
1231657	GCA_002105025.1	Clohesyomyces aquaticus (ascomycetes)
29856	GCA_000963775.2	Clonostachys rosea (ascomycetes)
246410	GCA_000149335.2	Coccidioides immitis RS (ascomycetes)
222929	GCA_000151335.1	Coccidioides posadasii C735 delta SOWgp (ascomycetes)
1634537	GCA_003693555.1	Coccinonectria pachysandricola (ascomycetes)
2082957	GCA_004114315.1	Cochlonema odontosperma (fungi)
763665	GCA_002705745.1	Coemansia reversa NRRL 1564 (fungi)
1357676	GCA_000697235.1	Cokeromyces recurvatus B5483 (fungi)
565419	GCA_003369635.1	Coleophoma crateriformis (ascomycetes)
1849047	GCA_003369665.1	Coleophoma cylindrospora (ascomycetes)
27357	GCA_001593745.1	Colletotrichum acutatum (ascomycetes)
708187	GCA_001937105.1	Colletotrichum chlorophyti (ascomycetes)
27358	GCA_002249775.1	Colletotrichum coccodes (ascomycetes)
129314	GCA_001484525.1	Colletotrichum falcatum (ascomycetes)
710243	GCA_002930455.1	Colletotrichum fioriniae (ascomycetes)
690256	GCA_002887685.1	Colletotrichum fructicola (ascomycetes)
474922	GCA_003243855.1	Colletotrichum gloeosporioides (ascomycetes)
1209918	GCA_001663355.1	Colletotrichum godetiae (ascomycetes)
645133	GCA_000149035.1	Colletotrichum graminicola M1.001 (ascomycetes)
759273	GCA_001672515.1	Colletotrichum higginsianum IMI 349063 (ascomycetes)
1573173	GCA_001855235.1	Colletotrichum incanum (ascomycetes)
1585795	GCA_003386485.1	Colletotrichum lentis (ascomycetes)
290576	GCA_001693025.2	Colletotrichum lindemuthianum (ascomycetes)
5464	GCA_002814275.1	Colletotrichum musae (ascomycetes)
1460502	GCA_001563115.1	Colletotrichum nymphaeae SA-01 (ascomycetes)
1213857	GCA_000350065.2	Colletotrichum orbiculare MAFF 240422 (ascomycetes)
1209926	GCA_001831195.1	Colletotrichum orchidophilum (ascomycetes)
1209931	GCA_001563125.1	Colletotrichum salicis (ascomycetes)
328473	GCA_002749775.1	Colletotrichum sansevieriae (ascomycetes)
1347389	GCA_004367935.1	Colletotrichum sidae (ascomycetes)
703756	GCA_001563135.1	Colletotrichum simmondsii (ascomycetes)
1938904	GCA_003122705.1	Colletotrichum sp. JS-367 (ascomycetes)
1347390	GCA_004366825.1	Colletotrichum spinosum (ascomycetes)
1173701	GCA_001951195.1	Colletotrichum sublineola (ascomycetes)
1306861	GCA_005350895.1	Colletotrichum tanaceti (ascomycetes)
708197	GCA_001618715.1	Colletotrichum tofieldiae (ascomycetes)

5466	GCA_004367215.1	Colletotrichum trifolii (ascomycetes)
5467	GCA_002632455.2	Colletotrichum truncatum (ascomycetes)
2175971	GCA_003313185.1	Collybia sp. MG36 (basidiomycetes)
796925	GCA_001566745.1	Conidiobolus coronatus NRRL 28638 (fungi)
1357681	GCA_000697335.1	Conidiobolus incongruus B7586 (fungi)
2025994	GCA_003019895.1	Coniella lustricola (ascomycetes)
175648	GCA_002794785.1	Coniferiporia sulphurascens (basidiomycetes)
91930	GCA_002798055.1	Coniochaeta hoffmannii (ascomycetes)
1408157	GCA_001879275.1	Coniochaeta ligniaria NRRL 30616 (ascomycetes)
177199	GCA_003635345.1	Coniochaeta pulveracea (ascomycetes)
741705	GCA_000271625.1	Coniophora puteana RWD-64-598 SS2 (basidiomycetes)
1168221	GCA_000281105.1	Coniosporium apollinis CBS 100218 (ascomycetes)
1077358	GCA_004523945.1	Coniothyrium glycines (ascomycetes)
71717	GCA_004369175.1	Coprinellus micaceus (basidiomycetes)
240176	GCA_000182895.1	Coprinopsis cinerea okayama7#130 (basidiomycetes)
230819	GCA_004369085.1	Coprinopsis marcescibilis (basidiomycetes)
76940	GCA_900156845.1	Coprinopsis strossmayeri (basidiomycetes)
56187	GCA_003316025.1	Coprinus comatus (shaggy mane)
218633	GCA_002968875.1	Cordyceps cicadae (ascomycetes)
1081108	GCA_001636795.1	Cordyceps confragosa RCEF 1005 (ascomycetes)
89141	GCA_003025275.1	Cordyceps farinosa (ascomycetes)
1081104	GCA_001636725.1	Cordyceps fumosorosea ARSEF 2679 (ascomycetes)
983644	GCA_000225605.1	Cordyceps militaris CM01 (ascomycetes)
129524	GCA_003025255.1	Cordyceps pruinosa (ascomycetes)
2004951	GCA_002591385.1	Cordyceps sp. RAO-2017 (ascomycetes)
45847	GCA_003025305.1	Cordyceps tenuipes (ascomycetes)
930093	GCA_003385255.1	Corinectria fuckeliana (ascomycetes)
1448308	GCA_003016335.1	Corynespora cassiicola Philippines (ascomycetes)
1617101	GCA_001179765.1	Crepidotus sp. BD-2015 (basidiomycetes)
1301515	GCA_000464975.1	Cronartium comandrae C4 (rust fungi)
1301520	GCA_000500775.1	Cronartium quercuum f. sp. banksianae CqE3WM (rust fungi)
1301521	GCA_000500245.1	Cronartium ribicola 11-2 (rust fungi)
68775	GCA_004379715.1	Crucibulum laeve (basidiomycetes)
1323836	GCA_000504465.1	Cryomyces antarcticus CCFEE 534 (ascomycetes)
331657	GCA_005059845.1	Cryomyces minteri (ascomycetes)
179775	GCA_004802535.1	Cryphonectria macrospora (ascomycetes)
187221	GCA_004802565.1	Cryphonectria nitschkei (ascomycetes)
40262	GCA_003264845.1	Cryphonectria radicalis (ascomycetes)
1295533	GCA_001720205.1	Cryptococcus amylolentus CBS 6039 (basidiomycetes)
1295532	GCA_001720245.1	Cryptococcus depauperatus CBS 7855 (basidiomycetes)
1296111	GCA_000855695.1	Cryptococcus gattii CA1873 (basidiomycetes)
1334442	GCA_000835815.1	Cryptococcus gattii VGII 2001/935-1 (basidiomycetes)
1296105	GCA_000835755.1	Cryptococcus gattii VGIV IND107 (basidiomycetes)
367775	GCA_000185945.1	Cryptococcus gattii WM276 (basidiomycetes)
214684	GCA_000091045.1	Cryptococcus neoformans var. neoformans JEC21 (basidiomycetes)

1639000	GCA_001600855.1	Cryptococcus sp. JCM 24511 (basidiomycetes)
1295528	GCA_001720155.1	Cryptococcus wingfieldii CBS 7118 (basidiomycetes)
1279998	GCA_000697215.1	Cunninghamella bertholletiae 175 (fungi)
1274792	GCA_000697015.1	Cunninghamella elegans B9769 (fungi)
418126	GCA_002982235.1	Curvularia geniculata (ascomycetes)
1263492	GCA_000743335.1	Curvularia lunata CX-3 (ascomycetes)
1537989	GCA_000817285.1	Curvularia papendorfii (ascomycetes)
1526222	GCA_002161795.1	Curvularia sp. IFB-Z10 (ascomycetes)
387937	GCA_002335565.1	Cutaneotrichosporon arboriformis (basidiomycetes)
57679	GCA_001712445.1	Cutaneotrichosporon curvatum (basidiomycetes)
5554	GCA_001613755.1	Cutaneotrichosporon cutaneum (basidiomycetes)
1078942	GCA_002335625.1	Cutaneotrichosporon cyanovorans (basidiomycetes)
181174	GCA_002335585.1	Cutaneotrichosporon daszewskae (basidiomycetes)
129132	GCA_003116895.1	Cutaneotrichosporon dermatis (basidiomycetes)
82522	GCA_003116955.1	Cutaneotrichosporon mucoides (basidiomycetes)
879819	GCA_001027345.1	Cutaneotrichosporon oleaginosum (basidiomycetes)
1986089	GCA_900618795.1	Cyanodermella asteris (ascomycetes)
36016	GCA_003708795.2	Cyberlindnera americana (budding yeasts)
36022	GCA_001599195.1	Cyberlindnera fabianii (budding yeasts)
983966	GCA_001661405.1	Cyberlindnera jadinii NRRL Y-1542 (budding yeasts)
65811	GCA_003708355.2	Cyberlindnera maclurae (budding yeasts)
131113	GCA_003707745.1	Cyberlindnera misumaiensis (budding yeasts)
1004253	GCA_003706445.2	Cyberlindnera mrakii (budding yeasts)
36028	GCA_003706915.2	Cyberlindnera petersonii (budding yeasts)
907340	GCA_003709245.2	Cyberlindnera saturnus (budding yeasts)
907738	GCA_003708225.2	Cyberlindnera suaveolens (budding yeasts)
1667614	GCA_003708285.1	Cyberlindnera xylosilytica (budding yeasts)
1314674	GCA_000934385.1	Cylindrobasidium torrendii FP15055 ss-10 (basidiomycetes)
1220924	GCA_000365145.2	Cyphellophora europaea CBS 101466 (ascomycetes)
2250717	GCA_003351005.1	Cystobasidiaceae sp. HBUAS51001 (basidiomycetes)
5440	GCA_001599975.1	Cystobasidiopsis lactophilus (basidiomycetes)
106015	GCA_001599955.1	Cystobasidium pallidum (basidiomycetes)
1230097	GCA_003795295.1	Cytospora leucostoma (ascomycetes)
1858805	GCA_000292625.1	Dacryopinax primogenitus (basidiomycetes)
307937	GCA_000935225.1	Dactylonectria macrodidyma (ascomycetes)
1314783	GCA_001632345.1	Daedalea quercina L-15889 (basidiomycetes)
292717	GCA_000751375.2	Daldinia eschscholtzii (ascomycetes)
1001832	GCA_002120325.1	Daldinia sp. EC12 (ascomycetes)
130807	GCA_003706395.1	Danielozyma ontarioensis (budding yeasts)
1580835	GCA_004009845.1	Davidsoniella eucalypti (ascomycetes)
1580837	GCA_001513805.1	Davidsoniella virescens (ascomycetes)
1470396	GCA_003706415.2	Deakozyma indianensis (budding yeasts)
58627	GCA_001447935.1	Debaryomyces fabryi (budding yeasts)
284592	GCA_000006445.2	Debaryomyces hansenii CBS767 (budding yeasts)
27297	GCA_003708605.2	Debaryomyces maramus (budding yeasts)
27299	GCA_003708585.1	Debaryomyces nepalensis (budding yeasts)

148098	GCA_003708935.1	Debaryomyces prosopidis (budding yeasts)
522693	GCA_003708965.1	Debaryomyces subglobosus (budding yeasts)
1314807	GCA_004369135.1	Dendrothele bispora CBS 962.96 (basidiomycetes)
205917	GCA_004679275.1	Dentipellis fragilis (basidiomycetes)
1883078	GCA_002286715.1	Dentipellis sp. KUC8613 (basidiomycetes)
1214573	GCA_001630405.1	Diaporthe ampelina (ascomycetes)
626713	GCA_001447215.1	Diaporthe aspalathi (ascomycetes)
158607	GCA_001702395.2	Diaporthe helianthi (ascomycetes)
732165	GCA_000275845.1	Dichomitus squalens LYAD-421 SS1 (basidiomycetes)
1194607	GCA_003705635.1	Diddensiella caesifluorescens (budding yeasts)
749627	GCA_004335245.1	Didymella lethalis (ascomycetes)
749589	GCA_004151525.1	Didymella pinodes (pea foot rot fungus)
2021029	GCA_004522025.1	Didymella segeticola (ascomycetes)
1635097	GCA_001600575.1	Didymobotryum rigidum (ascomycetes)
215637	GCA_003614675.1	Dimargaris cristalligena (fungi)
4973	GCA_001600655.1	Dioszegia aurantiaca (basidiomycetes)
4974	GCA_001600615.1	Dioszegia crocea (basidiomycetes)
946125	GCA_002317995.1	Diplocarpon rosae (ascomycetes)
236234	GCA_001883845.1	Diplodia corticola (ascomycetes)
1400760	GCA_000671355.1	Diplodia sapinea CMW 190 (ascomycetes)
280322	GCA_001455585.1	Diplodia scrobiculata (ascomycetes)
420778	GCA_001975905.1	Diplodia seriata (ascomycetes)
27315	GCA_003707475.1	Dipodascus albidus (budding yeasts)
44078	GCA_003708655.1	Dipodascus geniculatus (budding yeasts)
45537	GCA_003285555.1	Diutina catenulata (budding yeasts)
1348612	GCA_003547095.1	Diversispora epigaea (glomeromycetes)
1367539	GCA_002116355.1	Dothistroma pini CBS 116487 (ascomycetes)
675120	GCA_000340195.1	Dothistroma septosporum NZE10 (ascomycetes)
98403	GCA_001625195.1	Drechmeria coniospora (ascomycetes)
1043628	GCA_000525045.1	Drechslerella stenobrocha 248 (ascomycetes)
1003232	GCA_000230595.3	Edhazardia aedis USNM 41457 (microsporidians)
519963	GCA_002240705.1	Elaphomyces granulatus (ascomycetes)
302913	GCA_005959805.1	Elsinoe ampelina (ascomycetes)
40998	GCA_003013795.1	Elsinoe australis (ascomycetes)
1972497	GCA_002110485.1	Emergomyces orientalis (ascomycetes)
1447872	GCA_001883825.1	Emergomyces pasteurianus Ep9510 (ascomycetes)
73230	GCA_002572855.1	Emmonsia crescens (ascomycetes)
1658172	GCA_001660665.1	Emmonsia sp. CAC-2015a (ascomycetes)
284813	GCA_000091225.2	Encephalitozoon cuniculi GB-M1 (microsporidians)
907965	GCA_000277815.3	Encephalitozoon hellem ATCC 50504 (microsporidians)
876142	GCA_000146465.1	Encephalitozoon intestinalis ATCC 50506 (microsporidians)
1178016	GCA_000280035.2	Encephalitozoon romaleae SJ-2008 (microsporidians)
1633209	GCA_001600455.1	Endocalyx cinctus (ascomycetes)
1263415	GCA_000464535.1	Endocarpon pusillum Z07020 (ascomycetes)
1580848	GCA_001640655.1	Endoconidiophora laricicola (ascomycetes)
1580850	GCA_001856765.1	Endoconidiophora polonica (ascomycetes)

1300066	GCA_000500795.1	Endocronartium harknessii PhW48OC (rust fungi)
2340872	GCA_003990785.1	Endogone sp. FLAS-F59071 (fungi)
646526	GCA_002081675.1	Enterocytozoon hepatopenaei (microsporidians)
1081671	GCA_002087915.1	Enterospora canceri (microsporidians)
34485	GCA_900018355.1	Entomophthora muscae (fungi)
1037526	GCA_000223075.2	Epichloe amarillans E57 (ascomycetes)
170559	GCA_000729855.1	Epichloe aotearoae (ascomycetes)
1406312	GCA_000729845.1	Epichloe baconii ATCC 200745 (ascomycetes)
1036762	GCA_000222915.1	Epichloe brachyelytri E4804 (ascomycetes)
79588	GCA_001008065.1	Epichloe bromicola (ascomycetes)
55200	GCA_002591845.1	Epichloe elymi (ascomycetes)
877507	GCA_003814445.1	Epichloe festucae Fl1 (ascomycetes)
1037527	GCA_000222895.2	Epichloe gansuensis E7080 (ascomycetes)
1227659	GCA_000309355.1	Epichloe gansuensis var. inebrians E818 (ascomycetes)
1035635	GCA_000225285.2	Epichloe glyceriae E277 (ascomycetes)
1344566	GCA_000729825.1	Epichloe sp. AL9924 (ascomycetes)
79593	GCA_001008265.1	Epichloe sylvatica (ascomycetes)
1227655	GCA_000308955.1	Epichloe typhina E8 (ascomycetes)
5050	GCA_001043855.1	Epichloe uncinata (ascomycetes)
105696	GCA_002116315.1	Epicoccum nigrum (ascomycetes)
749593	GCA_001879705.1	Epicoccum sorghinum (ascomycetes)
1429780	GCA_000710315.1	Eremothecium coryli CBS 5749 (budding yeasts)
931890	GCA_000235365.1	Eremothecium cymbalariae DBVPG#7215 (budding yeasts)
284811	GCA_000091025.4	Eremothecium gossypii ATCC 10895 (budding yeasts)
52586	GCA_000798715.1	Erysiphe necator (grape powdery mildew)
36044	GCA_000208805.1	Erysiphe pisi (ascomycetes)
225359	GCA_002918395.1	Erysiphe pulchra (ascomycetes)
5414	GCA_001972285.1	Erythrobasidium hasegawianum (basidiomycetes)
114840	GCA_001600175.1	Erythrobasidium yunnanense (basidiomycetes)
2027528	GCA_003055955.1	Escovopsis sp. AC (ascomycetes)
2027527	GCA_003055925.1	Escovopsis sp. Ae720 (ascomycetes)
2006383	GCA_003055165.1	Escovopsis sp. Ae724 (ascomycetes)
2027526	GCA_003055945.1	Escovopsis sp. Ae733 (ascomycetes)
2006384	GCA_003055185.1	Escovopsis sp. TC (ascomycetes)
150374	GCA_003055145.1	Escovopsis weberi (ascomycetes)
522482	GCA_002778215.1	Esteya vermicola (ascomycetes)
1709936	GCA_002917005.1	Eurotiomycetes sp. (ascomycetes)
2080615	GCA_003004525.2	Eurotiomycetes sp. MA 6038 (ascomycetes)
2080616	GCA_003004485.1	Eurotiomycetes sp. MA 6039 (ascomycetes)
87257	GCA_003184365.1	Evernia prunastri (ascomycetes)
1314781	GCA_001632375.1	Exidia glandulosa HHB12029 (basidiomycetes)
91920	GCA_001599775.1	Exophiala alcalophila (ascomycetes)
1182545	GCA_000709125.1	Exophiala aquamarina CBS 119918 (ascomycetes)
135514	GCA_001599795.1	Exophiala calicioides (ascomycetes)
858893	GCA_000230625.1	Exophiala dermatitidis NIH/UT8656 (ascomycetes)
91925	GCA_003955835.1	Exophiala lecanii-corni (ascomycetes)

212818	GCA_000836275.1	Exophiala mesophila (ascomycetes)
215243	GCA_000835515.1	Exophiala oligosperma (ascomycetes)
1016849	GCA_000835395.1	Exophiala sideris (ascomycetes)
1718875	GCA_004026505.1	Exophiala sp. (ascomycetes)
91928	GCA_000836115.1	Exophiala spinifera (ascomycetes)
348802	GCA_000835505.1	Exophiala xenobiotica (ascomycetes)
671987	GCA_000359705.1	Exserohilum turcica Et28A (northern corn leaf blight)
1367536	GCA_000504385.2	Exutisphaerella laricina CBS 326.52 (ascomycetes)
1609947	GCA_000733355.1	Falciphora oryzae (ascomycetes)
599839	GCA_000313525.1	Fibroporia radiculosa (basidiomycetes)
436010	GCA_001630335.1	Fibularhizoctonia sp. CBS 109695 (basidiomycetes)
104416	GCA_001600055.1	Filobasidium wieringae (basidiomycetes)
1128425	GCA_000934395.1	Fistulina hepatica ATCC 64428 (basidiomycetes)
1300067	GCA_000633125.1	Flammulina velutipes KACC42780 (basidiomycetes)
493452	GCA_004012055.1	Floccularia luteovirens (basidiomycetes)
694068	GCA_000271605.1	Fomitiporia mediterranea MF3/22 (basidiomycetes)
186125	GCA_001937815.1	Fomitopsis palustris (basidiomycetes)
743788	GCA_000344655.2	Fomitopsis pinicola FP-58527 SS1 (basidiomycetes)
34475	GCA_004679265.1	Fomitopsis rosea (basidiomycetes)
1367422	GCA_001651985.1	Fonsecaea erecta (ascomycetes)
254056	GCA_001642475.1	Fonsecaea monophora (ascomycetes)
1442371	GCA_000836435.1	Fonsecaea multimorphosa CBS 102226 (ascomycetes)
856822	GCA_001646965.1	Fonsecaea nubica (ascomycetes)
1442368	GCA_000835455.1	Fonsecaea pedrosoi CBS 271.37 (ascomycetes)
95322	GCA_002778095.1	Fragosphaeria purpurea (ascomycetes)
329885	GCA_005059855.1	Friedmanniomyces endolithicus (ascomycetes)
329884	GCA_005059865.1	Friedmanniomyces simplex (ascomycetes)
1427494	GCA_002224055.1	fungal sp. ARF18 (fungi)
1117665	GCA_000292665.1	fungal sp. EF0021 (fungi)
2067060	GCA_002939055.1	fungal sp. Mo6-1 (fungi)
1603295	GCA_000836255.1	fungal sp. No.11243 (fungi)
1813822	GCA_002003505.1	fungal sp. No.14919 (fungi)
61424	GCA_003086725.1	Furculomyces boomerangus (fungi)
1803897	GCA_001654545.1	Fusarium agapanthi (ascomycetes)
2050978	GCA_002982035.1	Fusarium algeriense (ascomycetes)
131363	GCA_003947045.1	Fusarium ambrosium (ascomycetes)
282267	GCA_001717845.1	Fusarium asiaticum (ascomycetes)
40199	GCA_000769295.1	Fusarium avenaceum (ascomycetes)
1174670	GCA_001680625.1	Fusarium azukicola (ascomycetes)
44412	GCA_002980475.1	Fusarium beomiforme (ascomycetes)
235270	GCA_001680685.1	Fusarium brasiliense (ascomycetes)
1041577	GCA_002980515.1	Fusarium burgessii (ascomycetes)
57143	GCA_004367475.1	Fusarium camptoceras (ascomycetes)
48490	GCA_000497325.3	Fusarium circinatum (ascomycetes)
2495903	GCA_004367485.1	Fusarium citri (ascomycetes)
231269	GCA_004367465.1	Fusarium coffeatum (ascomycetes)

249398	GCA_001599515.1	Fusarium commune (ascomycetes)
5516	GCA_900074845.1	Fusarium culmorum (ascomycetes)
235271	GCA_001680505.1	Fusarium cuneirostrum (ascomycetes)
61235	GCA_003313175.1	Fusarium equiseti (ascomycetes)
1147111	GCA_002168265.2	Fusarium euwallaceae (ascomycetes)
1604329	GCA_003353625.1	Fusarium fracticaudum (ascomycetes)
1279085	GCA_900079805.1	Fusarium fujikuroi IMI 58289 (ascomycetes)
229533	GCA_000240135.3	Fusarium graminearum PH-1 (ascomycetes)
152502	GCA_002234235.1	Fusarium hostae (ascomycetes)
2494464	GCA_004366955.1	Fusarium humuli (ascomycetes)
298378	GCA_003709435.1	Fusarium incarnatum (ascomycetes)
2494466	GCA_004367085.1	Fusarium irregulare (ascomycetes)
179993	GCA_001292635.1	Fusarium langsethiae (ascomycetes)
694270	GCA_003012285.1	Fusarium longipes (ascomycetes)
192010	GCA_900044065.1	Fusarium mangiferae (ascomycetes)
282269	GCA_001717855.1	Fusarium meridionale (ascomycetes)
2421284	GCA_001633045.1	Fusarium metavorans (Fusarium solani species complex 6)
2494468	GCA_004367095.1	Fusarium nanum (ascomycetes)
42673	GCA_001262555.1	Fusarium nygamai (ascomycetes)
426428	GCA_000149955.2	Fusarium oxysporum f. sp. lycopersici 4287 (ascomycetes)
660029	GCA_000271745.2	Fusarium oxysporum NRRL 32931 (ascomycetes)
120645	GCA_001680515.1	Fusarium phaseoli (ascomycetes)
1604332	GCA_002165215.1	Fusarium pininemorale (ascomycetes)
36050	GCA_001675295.1	Fusarium poae (ascomycetes)
1824868	GCA_002093855.1	Fusarium praegraminearum (ascomycetes)
1227346	GCA_900067095.1	Fusarium proliferatum ET1 (ascomycetes)
1028729	GCA_000303195.2	Fusarium pseudograminearum CS3096 (ascomycetes)
5128	GCA_001567575.1	Fusarium sambucinum (ascomycetes)
2211609	GCA_004367495.1	Fusarium scirpi (ascomycetes)
169388	GCA_002215905.1	Fusarium solani (ascomycetes)
2010991	GCA_003698175.1	Fusarium sp. AF-12 (ascomycetes)
1325733	GCA_003947005.1	Fusarium sp. AF-3 (ascomycetes)
1325735	GCA_003946995.1	Fusarium sp. AF-4 (ascomycetes)
1325737	GCA_003947015.1	Fusarium sp. AF-6 (ascomycetes)
1325734	GCA_003946985.1	Fusarium sp. AF-8 (ascomycetes)
1755127	GCA_004367175.1	Fusarium sp. FIESC_12 (ascomycetes)
2109338	GCA_004367075.1	Fusarium sp. FIESC_23 (ascomycetes)
1385656	GCA_004367155.1	Fusarium sp. FIESC_5 (ascomycetes)
1580212	GCA_000966855.1	Fusarium sp. JS1030 (ascomycetes)
1580211	GCA_000966865.1	Fusarium sp. JS626 (ascomycetes)
451674	GCA_002234255.1	Fusarium sp. Na10 (ascomycetes)
5514	GCA_003012315.1	Fusarium sporotrichioides (ascomycetes)
2283686	GCA_003670145.1	Fusarium subtropicale (ascomycetes)
767483	GCA_001513835.1	Fusarium temperatum (ascomycetes)
61284	GCA_900382705.2	Fusarium tricinctum (ascomycetes)

232081	GCA_001680535.1	Fusarium tucumaniae (ascomycetes)
42665	GCA_002194535.1	Fusarium udum (ascomycetes)
56646	GCA_900007375.1	Fusarium venenatum (ascomycetes)
334819	GCA_000149555.1	Fusarium verticillioides 7600 (ascomycetes)
994087	GCA_000585705.1	Fusarium virguliforme Mont-1 (ascomycetes)
221167	GCA_004329255.1	Fusarium xylarioides (ascomycetes)
1938905	GCA_002197995.1	Gaeumannomyces sp. JS-464 (ascomycetes)
644352	GCA_000145635.1	Gaeumannomyces tritici R3-111a-1 (ascomycetes)
685588	GCA_000697645.1	Galerina marginata CBS 339.88 (basidiomycetes)
2037899	GCA_002803225.1	Gamarada debralockiae (ascomycetes)
34457	GCA_003697905.1	Ganoderma australe (basidiomycetes)
34458	GCA_002900995.2	Ganoderma boninense (basidiomycetes)
1077286	GCA_000271565.1	Ganoderma lucidum G.260125-1 (basidiomycetes)
1077348	GCA_002760635.1	Ganoderma sinense ZZ0214-1 (basidiomycetes)
34467	GCA_003057275.1	Ganoderma tsugae (basidiomycetes)
914234	GCA_000320605.2	Gelatoporia subvermispora B (basidiomycetes)
240216	GCA_900188565.1	Geosmithia flava (ascomycetes)
1094350	GCA_900108815.1	Geosmithia morbida (ascomycetes)
68827	GCA_900188575.1	Geosmithia putterillii (ascomycetes)
44066	GCA_002233575.1	Geotrichum fermentans (budding yeasts)
44941	GCA_003550325.1	Gigaspora rosea (glomeromycetes)
1332765	GCA_002917775.1	Glaciozyma antarctica PI12 (basidiomycetes)
1116229	GCA_000409485.1	Glarea lozoyensis ATCC 20868 (ascomycetes)
909944	GCA_001599755.1	Gliomastix tumulicola (ascomycetes)
670483	GCA_000344685.1	Gloeophyllum trabeum ATCC 11539 (basidiomycetes)
98760	GCA_004338095.1	Gloeostereum incarnatum (basidiomycetes)
658196	GCA_003550305.1	Glomus cerebriforme (glomeromycetes)
574774	GCA_001692915.1	Glonium stellatum (ascomycetes)
62708	GCA_003611235.1	Golovinomyces cichoracearum (ascomycetes)
58918	GCA_001599655.1	Golubevia pallescens (basidiomycetes)
104221	GCA_003316585.1	Gomphus bonarii (basidiomycetes)
2175977	GCA_003314385.1	Gomphus sp. MG54 (basidiomycetes)
1344416	GCA_001574975.1	Gonapodya prolifera JEL478 (monoblepharidomycetes)
483437	GCA_001650995.1	Gongronella sp. w5 (fungi)
1226780	GCA_002150815.3	Grammothele lineata (basidiomycetes)
67622	GCA_001513895.1	Graphilbum fragrans (ascomycetes)
5627	GCA_001683735.1	Grifola frondosa (maitake)
49332	GCA_003708315.2	Groenewaldozyma salmanticensis (budding yeasts)
655863	GCA_000143105.2	Grosmannia clavigera kw1407 (ascomycetes)
255215	GCA_004028395.1	Grosmannia galeiformis (ascomycetes)
360150	GCA_002778075.1	Grosmannia penicillata (ascomycetes)
1353255	GCA_000442125.1	Gyalolechia flavorubescens KoLRI002931 (ascomycetes)
231916	GCA_002938385.1	Gymnopilus dilepis (basidiomycetes)
944289	GCA_000827265.1	Gymnopus luxurians FD-317 M1 (basidiomycetes)
148818	GCA_004325065.1	Hamiltosporidium magnivora (microsporidians)
1176355	GCA_004325075.1	Hamiltosporidium tvaerminnensis (microsporidians)

211099	GCA_003706385.1	Hanseniaspora clermontiae (budding yeasts)
2094518	GCA_003020785.1	Hanseniaspora gamundiae (budding yeasts)
56406	GCA_004919775.1	Hanseniaspora guilliermondii (budding yeasts)
1926583	GCA_004919785.1	Hanseniaspora jakobsenii (budding yeasts)
211098	GCA_004919765.1	Hanseniaspora lachancei (budding yeasts)
211097	GCA_004919825.1	Hanseniaspora meyeri (budding yeasts)
1443074	GCA_004919845.1	Hanseniaspora nectarophila (budding yeasts)
331638	GCA_004919885.1	Hanseniaspora occidentalis var. citrica (budding yeasts)
211096	GCA_001749795.1	Hanseniaspora opuntiae (budding yeasts)
56408	GCA_001747045.1	Hanseniaspora osmophila (budding yeasts)
332229	GCA_003708335.1	Hanseniaspora pseudoguilliermondii (budding yeasts)
572238	GCA_003708365.1	Hanseniaspora singularis (budding yeasts)
1888986	GCA_004919795.1	Hanseniaspora sp. CRUB 1602 (budding yeasts)
689838	GCA_004919915.1	Hanseniaspora thailandica (budding yeasts)
29833	GCA_001747055.1	Hanseniaspora uvarum (budding yeasts)
766949	GCA_001664025.1	Hanseniaspora valbyensis NRRL Y-1626 (budding yeasts)
56409	GCA_000585475.3	Hanseniaspora vineae (budding yeasts)
431200	GCA_002917075.1	Hawksworthiomyces lignivorus (ascomycetes)
686832	GCA_000827355.1	Hebeloma cylindrosporum h7 (basidiomycetes)
1447875	GCA_002573585.1	Helicocarpus griseus UAMH5409 (ascomycetes)
5364	GCA_004369045.1	Heliocybe sulcata (basidiomycetes)
1400345	GCA_000498615.1	Helminthosporium solani B-AC-16A (ascomycetes)
1967635	GCA_002554605.1	Helotiales sp. F229 (ascomycetes)
203904	GCA_004125335.1	Hemileia vastatrix (coffee rust fungus)
1081669	GCA_002087885.1	Hepatospora eriocheir (microsporidians)
135208	GCA_004681135.1	Hericium alpestre (basidiomycetes)
100756	GCA_003675405.1	Hericium coralloides (basidiomycetes)
1240657	GCA_000315175.1	Herpotrichiellaceae sp. UM238 (ascomycetes)
101127	GCA_002104935.1	Hesseltinella vesiculosa (fungi)
13563	GCA_001457955.1	Heterobasidion annosum (basidiomycetes)
747525	GCA_000320585.2	Heterobasidion irregulare TC 32-1 (basidiomycetes)
207832	GCA_002994785.1	Heterobasidion parviporum (basidiomycetes)
1043627	GCA_000956045.1	Hirsutella minnesotensis 3608 (ascomycetes)
111463	GCA_004142005.1	Hirsutella rhossiliensis (ascomycetes)
1303645	GCA_000472125.2	Hirsutella thompsonii MTCC6686 (ascomycetes)
339724	GCA_000149585.1	Histoplasma capsulatum NAm1 (ascomycetes)
71782	GCA_001599935.1	Holtermannia corniformis (basidiomycetes)
104675	GCA_001600035.1	Holtermanniella nyarrowii (basidiomycetes)
166479	GCA_000235945.1	Homolaphlyctis polyrhiza JEL 142 (chytrids)
706561	GCA_005059885.1	Hortaea thailandica (ascomycetes)
1157616	GCA_002127715.1	Hortaea werneckii EXF-2000 (ascomycetes)
1580854	GCA_002018275.1	Huntiella bhutanensis (ascomycetes)
1580858	GCA_003032515.1	Huntiella decipiens (ascomycetes)
1580861	GCA_000712465.1	Huntiella moniliformis (ascomycetes)
1580864	GCA_000833645.1	Huntiella omanensis (ascomycetes)
1580867	GCA_001483325.1	Huntiella savannae (ascomycetes)

1095630	GCA_002865645.1	Hyaloscypha bicolor E (ascomycetes)
1149755	GCA_002865655.1	Hyaloscypha variabilis F (ascomycetes)
994086	GCA_000827185.1	Hydnomerulius pinastri MD-312 (basidiomycetes)
80590	GCA_003314045.1	Hygrophorus pudorinus (basidiomycetes)
264141	GCA_003314125.1	Hygrophorus russula (basidiomycetes)
861053	GCA_003314085.1	Hymenopellis chiangmaiae (basidiomycetes)
937743	GCA_003314005.1	Hymenopellis radicata (basidiomycetes)
746836	GCA_900184765.1	Hymenoscyphus fraxineus (Chalara fraxinea)
51284	GCA_001414455.1	Hymenoscyphus fructigenus (ascomycetes)
253104	GCA_001414485.1	Hymenoscyphus herbarum (ascomycetes)
1735278	GCA_001414345.1	Hymenoscyphus infarciens (ascomycetes)
1735277	GCA_001414375.1	Hymenoscyphus laetus (ascomycetes)
1622260	GCA_900536425.1	Hymenoscyphus linearis (ascomycetes)
1640341	GCA_900536445.1	Hymenoscyphus occultus (ascomycetes)
1735280	GCA_001414415.1	Hymenoscyphus repandus (ascomycetes)
1735279	GCA_001414355.1	Hymenoscyphus salicellus (ascomycetes)
253101	GCA_001399465.1	Hymenoscyphus scutula (ascomycetes)
2259768	GCA_003988905.1	Hymenotorrendiella dingleyae (ascomycetes)
2305101	GCA_003988895.1	Hyphodiscus sp. D1413 (ascomycetes)
945553	GCA_000827495.1	Hypholoma sublateritium FD-334 SS-4 (basidiomycetes)
984485	GCA_001661395.1	Hyphopichia burtonii NRRL Y-1933 (budding yeasts)
507511	GCA_003706925.2	Hyphopichia heimii (budding yeasts)
45558	GCA_001599095.1	Hyphopichia homilentoma (budding yeasts)
717741	GCA_003856775.1	Hyphopichia pseudoburtonii (budding yeasts)
696354	GCA_000731825.1	Hypocrella siamensis (ascomycetes)
1243767	GCA_002775035.1	Hypoxylon pulicicidum (ascomycetes)
1001833	GCA_002120315.1	Hypoxylon sp. CI-4A (ascomycetes)
1001938	GCA_002120305.1	Hypoxylon sp. CO27-5 (ascomycetes)
1489628	GCA_000931505.1	Hypoxylon sp. E7406B (ascomycetes)
1001937	GCA_002120335.1	Hypoxylon sp. EC38 (ascomycetes)
1136866	GCA_000467715.1	Hysterium pulicare CBS 123377 (ascomycetes)
64609	GCA_001913115.1	Ilyonectria destructans (ascomycetes)
1079131	GCA_002991585.1	Ilyonectria mors-panacis (ascomycetes)
5319	GCA_001986395.2	Irpex lacteus (basidiomycetes)
933084	GCA_000697665.1	Jaapia argillacea MUCL 33604 (basidiomycetes)
1569628	GCA_003144245.1	Jaminaea rosea (basidiomycetes)
994334	GCA_003990745.1	Jimgerdemannia flammicorona (fungi)
324621	GCA_003951145.1	Jimgerdemannia lactiflua (fungi)
1940567	GCA_003012975.1	Juglanconis juglandina (ascomycetes)
1940568	GCA_003012965.1	Juglanconis oblonga (ascomycetes)
1955135	GCA_003013055.1	Juglanconis sp. DMW523 (ascomycetes)
1365824	GCA_000497045.1	Kalmanozyma brasiliensis GHG001 (smut fungi)
271218	GCA_003708495.1	Kazachstania aerobia (budding yeasts)
1071382	GCA_000304475.1	Kazachstania africana CBS 2517 (budding yeasts)
1006466	GCA_003708535.2	Kazachstania bromeliacearum (budding yeasts)
935788	GCA_003708845.2	Kazachstania intestinalis (budding yeasts)

488016	GCA_003708465.1	Kazachstania kunashirensis (budding yeasts)
74404	GCA_003708925.2	Kazachstania martiniae (budding yeasts)
1071383	GCA_000348985.1	Kazachstania naganishii CBS 8797 (budding yeasts)
56413	GCA_003708425.2	Kazachstania rosinii (budding yeasts)
1789683	GCA_900180425.1	Kazachstania saulgeensis (budding yeasts)
27293	GCA_002214935.1	Kazachstania servazzii (budding yeasts)
382647	GCA_003708905.2	Kazachstania siamensis (budding yeasts)
1089532	GCA_003708835.2	Kazachstania solicola (budding yeasts)
1089533	GCA_003708825.2	Kazachstania spencerorum (budding yeasts)
655095	GCA_003708865.1	Kazachstania taianensis (budding yeasts)
36916	GCA_003708445.2	Kazachstania transvaalensis (budding yeasts)
432100	GCA_003708545.1	Kazachstania turicensis (budding yeasts)
27294	GCA_003708525.2	Kazachstania unispora (budding yeasts)
71246	GCA_003708455.1	Kazachstania viticola (budding yeasts)
432099	GCA_003709265.1	Kazachstania yakushimaensis (budding yeasts)
33165	GCA_003707555.1	Kluyveromyces aestuarii (budding yeasts)
51656	GCA_003705805.2	Kluyveromyces dobzhanskii (budding yeasts)
28985	GCA_000002515.1	Kluyveromyces lactis (budding yeasts)
1003335	GCA_001417885.1	Kluyveromyces marxianus DMKU3-1042 (budding yeasts)
74104	GCA_003670155.1	Kluyveromyces nonfermentans (budding yeasts)
861556	GCA_000179415.1	Kluyveromyces wickerhamii UCD 54-210 (budding yeasts)
114771	GCA_001510575.1	Knoxdaviesia capensis (ascomycetes)
1215355	GCA_001510565.1	Knoxdaviesia proteae (ascomycetes)
206542	GCA_002319055.1	Knufia petricola (ascomycetes)
4999	GCA_002102565.1	Kockovaella imperatae (basidiomycetes)
271274	GCA_003705675.1	Kodamaea laetipori (budding yeasts)
34356	GCA_004919595.1	Kodamaea ohmeri (budding yeasts)
4922	GCA_001708105.1	Komagataella pastoris (budding yeasts)
644223	GCA_000027005.1	Komagataella phaffii GS115 (budding yeasts)
1099808	GCA_003705255.2	Komagataella populi (budding yeasts)
169507	GCA_003671595.2	Komagataella pseudopastoris (budding yeasts)
324739	GCA_003707205.2	Kregervanrija delftensis (budding yeasts)
324740	GCA_003707275.2	Kregervanrija fluxuum (budding yeasts)
110526	GCA_002081935.3	Kretzschmaria deusta (ascomycetes)
1382522	GCA_000576695.1	Kuraishia capsulata CBS 1993 (budding yeasts)
317048	GCA_003706305.2	Kuraishia molischiana (budding yeasts)
537810	GCA_003706285.2	Kuraishia ogatae (budding yeasts)
121466	GCA_003707135.1	Kurtzmaniella cleridarum (budding yeasts)
1296100	GCA_000512585.2	Kwoniella bestiolae CBS 10118 (basidiomycetes)
1296121	GCA_000512565.2	Kwoniella dejecticola CBS 10117 (basidiomycetes)
1296120	GCA_000507405.3	Kwoniella heveanensis BCC8398 (basidiomycetes)
1296122	GCA_000507465.3	Kwoniella mangroviensis CBS 8507 (basidiomycetes)
1296096	GCA_000512605.2	Kwoniella pini CBS 10737 (basidiomycetes)
1095629	GCA_000827195.1	Laccaria amethystina LaAM-08-1 (basidiomycetes)
486041	GCA_000143565.1	Laccaria bicolor S238N-H82 (basidiomycetes)
1266660	GCA_900074725.1	Lachancea dasiensis CBS 10888 (budding yeasts)

4955	GCA_900074765.1	Lachancea fermentati (budding yeasts)
226302	GCA_000149225.1	Lachancea kluyveri NRRL Y-12651 (budding yeasts)
1245769	GCA_000938715.1	Lachancea lanzarotensis (budding yeasts)
1266667	GCA_900074715.1	Lachancea meyersii CBS 8951 (budding yeasts)
1230905	GCA_900074745.1	Lachancea mirantina (budding yeasts)
1266666	GCA_900074755.1	Lachancea nothofagi CBS 11611 (budding yeasts)
1654605	GCA_002900925.1	Lachancea quebecensis (budding yeasts)
433476	GCA_900074735.1	Lachancea sp. CBS 6924 (budding yeasts)
559295	GCA_000142805.1	Lachancea thermotolerans CBS 6340 (budding yeasts)
262981	GCA_000167115.1	Lachancea waltii NCYC 2644 (budding yeasts)
2283846	GCA_003988875.1	Lachnum nothofagi (ascomycetes)
55514	GCA_003316005.1	Lactarius deliciosus (basidiomycetes)
2017516	GCA_003315975.1	Lactarius echinatus (basidiomycetes)
416442	GCA_003315955.1	Lactarius hatsudake (basidiomycetes)
222702	GCA_003313985.1	Lactarius indigo (basidiomycetes)
71966	GCA_003315875.1	Lactarius piperatus (basidiomycetes)
2175976	GCA_003315925.1	Lactarius sp. MG121 (basidiomycetes)
2175975	GCA_003314065.1	Lactarius sp. MG50 (basidiomycetes)
217427	GCA_003315845.1	Lactarius trivialis (basidiomycetes)
71967	GCA_003315835.1	Lactarius volemus (basidiomycetes)
1837245	GCA_003314055.1	Lactifluus hygrophoroides (basidiomycetes)
904239	GCA_003313945.1	Lactifluus pinguis (basidiomycetes)
1837266	GCA_003315895.1	Lactifluus rugatus (basidiomycetes)
1314785	GCA_001632365.1	Laetiporus sulphureus 93-53 (chicken-of-the-woods)
1424426	GCA_000812885.1	Lanzia echinophila (ascomycetes)
580046	GCA_003254425.1	Lasallia hispanica (ascomycetes)
45133	GCA_002111425.1	Lasiodiplodia theobromae (ascomycetes)
1294127	GCA_900169235.1	Lecanicillium fungicola 150-1 (ascomycetes)
73499	GCA_002796755.1	Lecanicillium psalliotae (ascomycetes)
2013060	GCA_002242745.2	Lecanicillium sp. LEC01 (ascomycetes)
1964301	GCA_003056605.1	Lecanicillium sp. MT-2017a (ascomycetes)
111012	GCA_002441625.1	Lecanosticta acicola (brown spot needle blight)
5353	GCA_001562095.1	Lentinula edodes (shiitake mushroom)
292559	GCA_000787475.1	Lentinus polychrous (basidiomycetes)
1328758	GCA_003813185.1	Lentinus tigrinus ALCF2SS1-7 (basidiomycetes)
1314670	GCA_001692735.1	Lepidopterella palustris CBS 459.81 (ascomycetes)
2136145	GCA_004296355.1	Lepiota venenata (basidiomycetes)
123925	GCA_900168675.1	Lepista sordida (basidiomycetes)
96373	GCA_001455505.1	Leptographium lundbergii (ascomycetes)
100367	GCA_000806385.1	Leptographium procerum (ascomycetes)
225338	GCA_900465125.1	Leptosphaeria biglobosa 'brassicae' group (ascomycetes)
985895	GCA_000230375.1	Leptosphaeria maculans JN3 (blackleg of rapeseed fungus)
5474	GCA_001660795.1	Leptoxyphium fumago (ascomycetes)
1258663	GCA_000382605.1	Leucoagaricus gongylophorus Ac12 (basidiomycetes)
1714833	GCA_001563735.1	Leucoagaricus sp. SymC.cos (basidiomycetes)
106004	GCA_001600635.1	Leucosporidium creatinivorum (basidiomycetes)

5278	GCA_003054985.1	Leucosporidium scottii (basidiomycetes)
1263082	GCA_000723665.1	Lichtheimia corymbifera JMRC:FSU:9682 (fungi)
688394	GCA_000945115.1	Lichtheimia ramosa (fungi)
1379160	GCA_000743315.1	Lignosus rhinocerotis TM02 (basidiomycetes)
228944	GCA_002233555.1	Limonomyces culmigenus (basidiomycetes)
61395	GCA_002104995.1	Linderina pennispora (fungi)
56418	GCA_003707585.2	Lipomyces arxii (budding yeasts)
383834	GCA_003705865.1	Lipomyces doorenjongii (budding yeasts)
56871	GCA_003705895.1	Lipomyces japonicus (budding yeasts)
34357	GCA_003705905.1	Lipomyces kononenkoae (budding yeasts)
36043	GCA_003705915.1	Lipomyces lipofer (budding yeasts)
56874	GCA_003705845.2	Lipomyces mesembrius (budding yeasts)
45792	GCA_003707545.2	Lipomyces oligophaga (budding yeasts)
675824	GCA_001661325.1	Lipomyces starkeyi NRRL Y-11557 (budding yeasts)
1337062	GCA_003705835.2	Lipomyces suomiensis (budding yeasts)
64571	GCA_002105155.1	Lobosporangium transversale (fungi)
379508	GCA_000149685.1	Lodderomyces elongisporus NRRL YB-4239 (budding yeasts)
41688	GCA_002276285.1	Lomentospora prolificans (ascomycetes)
64660	GCA_001950495.1	Lyophyllum decastes (basidiomycetes)
47721	GCA_001950515.1	Lyophyllum shimeji (basidiomycetes)
182055	GCA_001179725.1	Macrocystidia cucumis (basidiomycetes)
190371	GCA_003315915.1	Macrolepiota dolichaula (basidiomycetes)
1126212	GCA_000302655.1	Macrophomina phaseolina MS6 (charcoal rot)
100816	GCA_001275765.2	Madurella mycetomatis (ascomycetes)
2051560	GCA_003709005.1	Magnaporthales sp. P1609 (ascomycetes)
1758317	GCA_001936055.1	Magnaporthe sp. MG03 (ascomycetes)
1758316	GCA_001936065.1	Magnaporthe sp. MG05 (ascomycetes)
1758318	GCA_001936455.1	Magnaporthe sp. MG07 (ascomycetes)
1758543	GCA_001936555.1	Magnaporthe sp. MG08 (ascomycetes)
1758544	GCA_001936955.1	Magnaporthe sp. MG12 (ascomycetes)
36781	GCA_003049425.1	Magnaporthiopsis incrustans (ascomycetes)
644358	GCA_000193285.1	Magnaporthiopsis poae ATCC 64411 (ascomycetes)
165777	GCA_003049465.1	Magnaporthiopsis rhizophila (ascomycetes)
1449804	GCA_000817185.1	Magnusiomyces capitatus CNRMA 12.647 (budding yeasts)
1294685	GCA_900497725.1	Magnusiomyces capitatus NRRL Y-17686 (budding yeasts)
1294639	GCA_900497715.1	Magnusiomyces ingens NRRL Y-17630 (budding yeasts)
1294638	GCA_003708635.2	Magnusiomyces tetraspermus NRRL Y-7288 (budding yeasts)
1381934	GCA_001264625.1	Malassezia caprae (basidiomycetes)
948313	GCA_001264635.1	Malassezia cuniculi (basidiomycetes)
169489	GCA_001600775.1	Malassezia dermatis (basidiomycetes)
1381935	GCA_001264685.1	Malassezia equina (basidiomycetes)
55194	GCA_002551515.1	Malassezia furfur (Pityriasis (Tinea) versicolor infection agent)
425265	GCA_000181695.1	Malassezia globosa CBS 7966 (basidiomycetes)

223818	GCA_001600795.1	Malassezia japonica (basidiomycetes)
180528	GCA_001600835.1	Malassezia nana (basidiomycetes)
76774	GCA_001264985.1	Malassezia obtusa (basidiomycetes)
77020	GCA_001278385.1	Malassezia pachydermatis (basidiomycetes)
76775	GCA_003290485.1	Malassezia restricta (basidiomycetes)
76776	GCA_001264965.1	Malassezia slooffiae (basidiomycetes)
2011732	GCA_004026415.1	Malassezia sp. (basidiomycetes)
1230383	GCA_000349305.2	Malassezia sympodialis ATCC 42132 (basidiomycetes)
2020962	GCA_002818225.1	Malassezia vespertilionis (basidiomycetes)
253288	GCA_001264885.1	Malassezia yamatoensis (basidiomycetes)
5041	GCA_900128795.2	Malbranchea cinnamomea (ascomycetes)
1072389	GCA_000298775.1	Marssonina brunnea f. sp. 'multigermtubi' MB_m1 (ascomycetes)
503106	GCA_002204255.1	Marssonina coronariae (ascomycetes)
324742	GCA_003705535.2	Martiniozyma abiesophila (budding yeasts)
527531	GCA_003313965.1	Megacollybia marginata (basidiomycetes)
1280837	GCA_003144205.1	Meira miltonrushii (basidiomycetes)
286112	GCA_001600355.1	Meira nashicola (basidiomycetes)
526922	GCA_002157025.1	Melampsora abietis-canadensis (rust fungi)
526923	GCA_002157015.1	Melampsora aecidioides (rust fungi)
242475	GCA_002157005.1	Melampsora allii-populina (rust fungi)
747676	GCA_000204055.1	Melampsora larici-populina 98AG31 (rust fungi)
258770	GCA_002157035.1	Melampsora medusae f. sp. deltoidis (rust fungi)
82102	GCA_002157085.1	Melampsora occidentalis (rust fungi)
1298852	GCA_000464645.1	Melampsora pinitorqua Mpini7 (rust fungi)
80383	GCA_001599555.1	Memnoniella echinata (ascomycetes)
655827	GCA_000187405.1	Metarhizium acridum CQMa 102 (ascomycetes)
1081103	GCA_000804445.1	Metarhizium album ARSEF 1941 (ascomycetes)
1276135	GCA_000814975.1	Metarhizium anisopliae ARSEF 549 (ascomycetes)
1276141	GCA_000814965.1	Metarhizium brunneum ARSEF 3297 (ascomycetes)
1276136	GCA_000814955.1	Metarhizium guizhouense ARSEF 977 (ascomycetes)
1081105	GCA_001636745.1	Metarhizium rileyi RCEF 4871 (ascomycetes)
655844	GCA_000187425.2	Metarhizium robertsii ARSEF 23 (ascomycetes)
2248764	GCA_003600395.1	Metchnikovella incurvata (microsporidians)
353897	GCA_002370615.1	Metschnikowia aberdeeniae (budding yeasts)
2163413	GCA_004217705.1	Metschnikowia aff. pulcherrima (budding yeasts)
150206	GCA_002370875.1	Metschnikowia arizonensis (budding yeasts)
27321	GCA_002073855.1	Metschnikowia australis (budding yeasts)
869754	GCA_001664035.1	Metschnikowia bicuspidata var. bicuspidata NRRL YB-4993 (budding yeasts)
73518	GCA_002374385.1	Metschnikowia borealis (budding yeasts)
1699426	GCA_002370295.1	Metschnikowia bowlesiae (budding yeasts)
390697	GCA_002370635.1	Metschnikowia cerradonensis (budding yeasts)
473035	GCA_002370175.1	Metschnikowia colocasiae (budding yeasts)
73517	GCA_002370835.1	Metschnikowia continentalis (budding yeasts)
1323754	GCA_002374405.1	Metschnikowia cubensis (budding yeasts)
174643	GCA_002374455.1	Metschnikowia dekortorum (budding yeasts)

1506548	GCA_002370475.1	Metschnikowia drakensbergensis (budding yeasts)
135833	GCA_002893735.1	Metschnikowia drosophilae (budding yeasts)
1200864	GCA_000317355.2	Metschnikowia fructicola 277 (budding yeasts)
301365	GCA_002370815.1	Metschnikowia hamakuensis (budding yeasts)
27323	GCA_002370325.1	Metschnikowia hawaiiensis (budding yeasts)
73520	GCA_002374725.1	Metschnikowia hibisci (budding yeasts)
301367	GCA_002374535.1	Metschnikowia kamakouana (budding yeasts)
197673	GCA_002370135.1	Metschnikowia kipukae (budding yeasts)
135832	GCA_002370915.1	Metschnikowia lochheadii (budding yeasts)
1697387	GCA_002370695.1	Metschnikowia matae var. maris (budding yeasts)
301368	GCA_002374555.1	Metschnikowia mauinuiana (budding yeasts)
150224	GCA_002893665.1	Metschnikowia orientalis (budding yeasts)
1150643	GCA_002370515.1	Metschnikowia proteae (budding yeasts)
27327	GCA_003401635.1	Metschnikowia reukaufii (budding yeasts)
197674	GCA_002374485.1	Metschnikowia santaceciliae (budding yeasts)
353899	GCA_002374645.1	Metschnikowia shivogae (budding yeasts)
473037	GCA_002370765.1	Metschnikowia similis (budding yeasts)
1807683	GCA_002370645.1	Metschnikowia sp. 00-154.1 (budding yeasts)
1807684	GCA_002370185.1	Metschnikowia sp. 01-655c1 (budding yeasts)
1807685	GCA_002370795.1	Metschnikowia sp. 03-147.1 (budding yeasts)
1807686	GCA_002374635.1	Metschnikowia sp. 04-218.3 (budding yeasts)
1807687	GCA_002370245.1	Metschnikowia sp. 04-226.1 (budding yeasts)
1807688	GCA_002374735.1	Metschnikowia sp. 13-106.1 (budding yeasts)
1769792	GCA_002894445.1	Metschnikowia sp. AWRI3582 (budding yeasts)
2562755	GCA_005406065.1	Metschnikowia sp. JCM 33374 (budding yeasts)
1807689	GCA_002370575.1	Metschnikowia sp. M2Y3 (budding yeasts)
1091232	GCA_000755205.1	Meyerozyma caribbica MG20W (budding yeasts)
294746	GCA_000149425.1	Meyerozyma guilliermondii ATCC 6260 (budding yeasts)
2028340	GCA_003676015.1	Meyerozyma sp. JA9 (budding yeasts)
269621	GCA_900096595.1	Microbotryum intermedium (basidiomycetes)
683840	GCA_001244265.1	Microbotryum lychnidis-dioicae p1A1 Lamole (basidiomycetes)
289078	GCA_900102585.1	Microbotryum saponariae (basidiomycetes)
370302	GCA_003665825.1	Microbotryum silenes-acaulis (basidiomycetes)
796604	GCA_900120095.1	Microbotryum silenes-dioicae (basidiomycetes)
5272	GCA_900015485.1	Microbotryum violaceum (basidiomycetes)
766055	GCA_002786065.1	Microcyclospora pomicola (ascomycetes)
766056	GCA_003012245.1	Microcyclospora tardicrescens (ascomycetes)
766057	GCA_002785985.1	Microcyclosporella mali (ascomycetes)
196109	GCA_001566295.1	Microdochium bolleyi (ascomycetes)
554155	GCA_000151145.1	Microsporum canis CBS 113480 (ascomycetes)
45597	GCA_003708105.2	Middelhovenomyces tepae (budding yeasts)
28986	GCA_001600675.1	Millerozyma acaciae (budding yeasts)
4920	GCA_002196765.1	Millerozyma farinosa (budding yeasts)
1485682	GCA_000760515.1	Mitosporidium daphniae (microsporidians)
764103	GCA_000708205.1	Mixia osmundae IAM 14324 (basidiomycetes)
1081109	GCA_001636675.1	Moelleriella libera RCEF 2490 (ascomycetes)

84753	GCA_000747765.1	Moesziomyces antarcticus (smut fungi)
1391700	GCA_000517465.1	Moesziomyces aphidis DSM 70725 (smut fungi)
2039273	GCA_003004725.1	Moesziomyces sp. F16C1 (smut fungi)
2039272	GCA_003004715.1	Moesziomyces sp. F5C1 (smut fungi)
5098	GCA_003184285.1	Monascus purpureus (ascomycetes)
89489	GCA_002976275.1	Monascus ruber (ascomycetes)
2020907	GCA_003971905.1	Moniliella sp. 'wahieum' (basidiomycetes)
61194	GCA_002162555.1	Monilinia aucupariae (ascomycetes)
38448	GCA_002162545.1	Monilinia fructicola (ascomycetes)
38457	GCA_003671625.1	Monilinia fructigena (ascomycetes)
61186	GCA_002909725.1	Monilinia laxa (ascomycetes)
255361	GCA_002909645.1	Monilinia polystroma (ascomycetes)
221103	GCA_001466705.1	Moniliophthora roreri (basidiomycetes)
155416	GCA_004154925.1	Monosporascus cannonballus (ascomycetes)
155417	GCA_004154915.1	Monosporascus ibericus (ascomycetes)
2211642	GCA_004155035.1	Monosporascus sp. 5C6A (ascomycetes)
2211644	GCA_004155055.1	Monosporascus sp. CRB-8-3 (ascomycetes)
2211643	GCA_004155915.1	Monosporascus sp. CRB-9-2 (ascomycetes)
2211647	GCA_004155935.1	Monosporascus sp. GIB2 (ascomycetes)
2211646	GCA_004154975.1	Monosporascus sp. MC13-8B (ascomycetes)
2211645	GCA_004155925.1	Monosporascus sp. MG133 (ascomycetes)
1081914	GCA_004155945.1	Monosporascus sp. mg162 (ascomycetes)
1392247	GCA_003790465.1	Morchella conica CCBAS932 (ascomycetes)
1582338	GCA_003314645.1	Morchella eximia (ascomycetes)
1174673	GCA_003444635.1	Morchella importuna (ascomycetes)
1174676	GCA_003313775.1	Morchella septimelata (ascomycetes)
685557	GCA_000240685.2	Mortierella alpina ATCC 32222 (fungi)
1314771	GCA_001651415.1	Mortierella elongata AG-77 (fungi)
1069443	GCA_000739165.1	Mortierella verticillata NRRL 6337 (fungi)
696254	GCA_000950635.1	Mrakia blollopis (basidiomycetes)
29902	GCA_001600395.1	Mrakia frigida (basidiomycetes)
72568	GCA_001889225.1	Mrakia psychrophila (basidiomycetes)
91626	GCA_000950595.1	Mucor ambiguus (fungi)
747725	GCA_001638945.1	Mucor circinelloides f. lusitanicus CBS 277.49 (fungi)
1357679	GCA_000697295.1	Mucor indicus B7402 (fungi)
1290454	GCA_000587855.1	Mucor irregularis B50 (fungi)
1357677	GCA_000697255.1	Mucor racemosus B9645 (fungi)
1274784	GCA_000696895.1	Mucor velutinosus B5328 (fungi)
658473	GCA_001612595.1	Mycena chlorophos (basidiomycetes)
2018698	GCA_003987915.1	Mycena citricolor (basidiomycetes)
143450	GCA_001297265.1	Mycosphaerella arachidis (ascomycetes)
1367537	GCA_002153405.1	Mycosphaerella populi pn0402 (ascomycetes)
1367542	GCA_002116345.1	Mycosphaerella sp. PB-2012b Mex 2-1-2 (ascomycetes)
333113	GCA_000504405.2	Mycosphaerella sp. Ston1 (ascomycetes)
1873265	GCA_002785995.1	Mycosphaerelloides madeirae (ascomycetes)
87228	GCA_002162495.1	Myriosclerotinia curreyana (ascomycetes)

108355	GCA_002162615.1	Myriosclerotinia duriaeana (ascomycetes)
38443	GCA_002162505.1	Myriosclerotinia scirpicola (ascomycetes)
857566	GCA_001661315.1	Nadsonia fulvescens var. elongata DSM 6958 (budding yeasts)
43970	GCA_003123035.1	Nadsonia starkeyi-henricii (budding yeasts)
71784	GCA_002105065.1	Naematelia encephala (basidiomycetes)
100951	GCA_001599735.1	Naganishia albida (basidiomycetes)
1279116	GCA_001046975.1	Nakaseomyces bacillisporus CBS 7720 (budding yeasts)
1279113	GCA_001039675.1	Nakaseomyces delphensis CBS 2170 (budding yeasts)
165778	GCA_003049435.1	Nakataea oryzae (ascomycetes)
36025	GCA_003706265.1	Nakazawaea holstii (budding yeasts)
1538182	GCA_001599355.1	Nakazawaea peltata (budding yeasts)
1538186	GCA_003243035.1	Nakazawaea wickerhamii (budding yeasts)
535722	GCA_000150975.2	Nannizzia gypsea CBS 118893 (ascomycetes)
1064592	GCA_000237345.1	Naumovozyma castellii CBS 4309 (budding yeasts)
1071378	GCA_000227115.2	Naumovozyma dairenensis CBS 421 (budding yeasts)
2020480	GCA_002682825.1	Nectria sp. B-13 (ascomycetes)
1805483	GCA_001642395.1	Nematocida displodere (microsporidians)
881290	GCA_000250985.1	Nematocida parisii ERTm1 (microsporidians)
1138374	GCA_000738915.1	Nematocida sp. 1 ERTm6 (microsporidians)
1805481	GCA_001642415.1	Nematocida sp. ERTm5 (microsporidians)
1603057	GCA_003316195.1	Neoboletus brunneissimus (basidiomycetes)
374759	GCA_003316145.1	Neoboletus magnificus (basidiomycetes)
1461596	GCA_003988965.1	Neobulgaria alba (ascomycetes)
1754190	GCA_002104975.1	Neocallimastix californiae (chytrids)
1198029	GCA_001929475.1	Neolecta irregularis DAH-3 (ascomycetes)
1314782	GCA_001632425.1	Neolentinus lepideus HHB14362 ss-1 (train wrecker)
78410	GCA_001305505.1	Neonectria ditissima (ascomycetes)
78402	GCA_003385265.1	Neonectria hederae (ascomycetes)
979145	GCA_003385315.1	Neonectria punicea (ascomycetes)
1735992	GCA_003934905.1	Neonectria sp. DH2 (ascomycetes)
71958	GCA_003987895.1	Neonothopanus nambi (basidiomycetes)
108428	GCA_900092665.1	Neoscytalidium dimidiatum (ascomycetes)
1266766	GCA_000604205.2	Neurospora africana FGSC 1740 (ascomycetes)
367110	GCA_000182925.2	Neurospora crassa OR74A (ascomycetes)
1266714	GCA_000604225.2	Neurospora pannonica FGSC 7221 (ascomycetes)
1266767	GCA_000604185.2	Neurospora sublineolata FGSC 5508 (ascomycetes)
1266715	GCA_000604245.2	Neurospora terricola FGSC 1889 (ascomycetes)
510951	GCA_000213175.1	Neurospora tetrasperma FGSC 2508 (ascomycetes)
1489893	GCA_001007845.1	Nigrograna mackinnonii (ascomycetes)
36927	GCA_000758425.1	Nilaparvata lugens yeast-like symbiont (ascomycetes)
1037528	GCA_000447185.1	Nosema apis BRL 01 (microsporidians)
578461	GCA_000383075.1	Nosema bombycis CQ1 (microsporidians)
40302	GCA_000988165.1	Nosema ceranae (microsporidians)
1367540	GCA_002116385.1	Nothophaeocryptopus gaeumannii CBS 267.37 (ascomycetes)
1052685	GCA_001687445.1	Obba rivulosa (basidiomycetes)

1442076	GCA_000611715.1	Ochroconis constricta UM 578 (ascomycetes)
118614	GCA_003833135.1	Oehlia diaphana (glomeromycetes)
36023	GCA_003706155.1	Ogataea glucozyma (budding yeasts)
36024	GCA_003705115.1	Ogataea henricii (budding yeasts)
50263	GCA_003706165.2	Ogataea kodamae (budding yeasts)
1156966	GCA_001600755.1	Ogataea methanolica (budding yeasts)
53649	GCA_003706205.1	Ogataea methylivora (budding yeasts)
36026	GCA_003706195.1	Ogataea minuta (budding yeasts)
50265	GCA_003706235.1	Ogataea naganishii (budding yeasts)
490708	GCA_003705625.1	Ogataea nitratoaversa (budding yeasts)
53937	GCA_003706255.1	Ogataea nonfermentans (budding yeasts)
871575	GCA_000187245.3	Ogataea parapolymorpha DL-1 (budding yeasts)
1378263	GCA_003706115.2	Ogataea philodendri (budding yeasts)
157292	GCA_003707685.1	Ogataea pilisensis (budding yeasts)
4923	GCA_003707665.1	Ogataea pini (budding yeasts)
460523	GCA_001664045.1	Ogataea polymorpha (budding yeasts)
550978	GCA_003707375.2	Ogataea populi-albae (budding yeasts)
104402	GCA_003706105.2	Ogataea ramenticola (budding yeasts)
169509	GCA_003706095.1	Ogataea trehaloabstinens (budding yeasts)
50267	GCA_003707645.1	Ogataea trehalophila (budding yeasts)
169510	GCA_003709205.2	Ogataea zsoltii (budding yeasts)
913774	GCA_000827325.1	Oidiodendron maius Zn (ascomycetes)
299130	GCA_003957845.1	Oidium heveae (ascomycetes)
212602	GCA_003610855.1	Oidium neolycopersici (ascomycetes)
1128401	GCA_000296255.1	Omphalotus olearius VT 653.13 (basidiomycetes)
180788	GCA_000812245.1	Onygena corvina (ascomycetes)
1387563	GCA_002167195.1	Ophidiomyces ophiodiicola (ascomycetes)
367097	GCA_003049485.1	Ophioceras dolichostomum (ascomycetes)
1399860	GCA_002591415.1	Ophiocordyceps australis (ascomycetes)
2004952	GCA_002591395.1	Ophiocordyceps camponoti-rufipedis (ascomycetes)
1330021	GCA_001633055.2	Ophiocordyceps polyrhachis-furcata BCC 54312 (ascomycetes)
72228	GCA_001648815.1	Ophiocordyceps sinensis (ascomycetes)
2039875	GCA_003339455.1	Ophiocordyceps sp. 'camponoti-leonardi' (ascomycetes)
2039874	GCA_003339415.1	Ophiocordyceps sp. 'camponoti-saundersi' (ascomycetes)
268505	GCA_001272575.2	Ophiocordyceps unilateralis (ascomycetes)
218668	GCA_003671545.1	Ophiognomonia clavigignenti-juglandacearum (ascomycetes)
5163	GCA_002917055.1	Ophiostoma ips (ascomycetes)
1224258	GCA_000317715.1	Ophiostoma novo-ulmi subsp. novo-ulmi H327 (Dutch elm disease fungus)
1262450	GCA_000410735.1	Ophiostoma piceae UAMH 11346 (ascomycetes)
1354746	GCA_000803265.1	Ordospora colligata OC4 (microsporidians)
669874	GCA_001661245.1	Pachysolen tannophilus NRRL Y-2460 (budding yeasts)
797268	GCA_001455915.2	Paecilomyces hepiali (ascomycetes)
264952	GCA_005765155.1	Paecilomyces penicillatus (ascomycetes)
131340	GCA_003666085.1	Pallidocercospora crystallina (ascomycetes)

181874	GCA_002938355.1	Panaeolus cyanescens (basidiomycetes)
1279117	GCA_000442785.1	Papiliotrema flavescens NRRL Y-50378 (basidiomycetes)
1354741	GCA_000738825.1	Papiliotrema laurentii RY1 (basidiomycetes)
502780	GCA_000150735.2	Paracoccidioides brasiliensis Pb18 (ascomycetes)
502779	GCA_000150705.2	Paracoccidioides lutzii Pb01 (ascomycetes)
179392	GCA_900634815.1	Paradendryphiella salina (ascomycetes)
1246581	GCA_002794465.1	Paramicrosporidium saccamoebae (fungi)
1859971	GCA_003012165.1	Paramyrothecium roridum (ascomycetes)
1460663	GCA_001642045.1	Paraphaeosphaeria sporulosa (ascomycetes)
1804209	GCA_001748405.1	Paraphoma sp. B47-9 (ascomycetes)
35722	GCA_000938895.1	Parasitella parasitica (fungi)
54790	GCA_003503115.1	Parastagonospora avenaria f. sp. tritici (ascomycetes)
321614	GCA_000146915.2	Parastagonospora nodorum SN15 (ascomycetes)
105714	GCA_001600415.1	Pascua guehoae (basidiomycetes)
1156157	GCA_000301015.1	Passalora fulva CBS 131901 (ascomycetes)
664439	GCA_000827475.1	Paxillus involutus ATCC 200175 (basidiomycetes)
930991	GCA_000827395.1	Paxillus rubicundulus Ve08.2h10 (basidiomycetes)
1987568	GCA_000412615.1	Pecoramyces ruminatium (chytrids)
286661	GCA_001592805.1	Peltaster fructicola (ascomycetes)
1073090	GCA_001890105.1	Penicilliopsis zonata CBS 506.65 (ascomycetes)
416450	GCA_002072345.1	Penicillium antarcticum (ascomycetes)
1835702	GCA_001773325.1	Penicillium arizonense (ascomycetes)
1439349	GCA_000577785.1	Penicillium biforme FM169 (ascomycetes)
104259	GCA_001048715.1	Penicillium brasilianum (ascomycetes)
1429867	GCA_000513335.1	Penicillium camemberti FM 013 (ascomycetes)
69766	GCA_000943775.1	Penicillium capsulatum (ascomycetes)
1439351	GCA_000577495.1	Penicillium carneum LCP05634 (ascomycetes)
5076	GCA_000710275.1	Penicillium chrysogenum (ascomycetes)
5077	GCA_001950535.1	Penicillium citrinum (ascomycetes)
36646	GCA_002072405.1	Penicillium coprophilum (ascomycetes)
69771	GCA_002072245.1	Penicillium decumbens (ascomycetes)
1170230	GCA_000315645.2	Penicillium digitatum Pd1 (ascomycetes)
27334	GCA_000769745.1	Penicillium expansum (ascomycetes)
254877	GCA_002072365.1	Penicillium flavigenum (ascomycetes)
48697	GCA_001513925.1	Penicillium freii (ascomycetes)
1439350	GCA_000576735.1	Penicillium fuscoglaucum FM041 (ascomycetes)
5078	GCA_001561935.1	Penicillium griseofulvum (ascomycetes)
40296	GCA_002116305.1	Penicillium italicum (ascomycetes)
5079	GCA_002369805.1	Penicillium janthinellum (ascomycetes)
60175	GCA_002072425.1	Penicillium nalgiovense (ascomycetes)
229535	GCA_000733025.2	Penicillium nordicum (ascomycetes)
933388	GCA_000346795.1	Penicillium oxalicum 114-2 (ascomycetes)
1439348	GCA_000577715.1	Penicillium paneum FM227 (ascomycetes)
1292256	GCA_000347475.1	Penicillium paxilli ATCC 26601 (ascomycetes)
60169	GCA_003344595.1	Penicillium polonicum (ascomycetes)
1365484	GCA_000513255.1	Penicillium roqueforti FM164 (ascomycetes)

500485	GCA_000226395.1	Penicillium rubens Wisconsin 54-1255 (ascomycetes)
69788	GCA_001750025.1	Penicillium sclerotiorum (ascomycetes)
60172	GCA_000952775.2	Penicillium solitum (ascomycetes)
2066501	GCA_005250745.1	Penicillium sp. CF01 (ascomycetes)
2066127	GCA_002916455.1	Penicillium sp. CF05 (ascomycetes)
887910	GCA_002000375.1	Penicillium sp. HKF2 (ascomycetes)
2153245	GCA_003138045.1	Penicillium sp. MA 6036 (ascomycetes)
2153247	GCA_003138025.1	Penicillium sp. MA 6040 (ascomycetes)
2138087	GCA_003852855.1	Penicillium sp. MT2 MMC-2018 (ascomycetes)
290292	GCA_002382855.1	Penicillium sp. 'occitanis' (ascomycetes)
2488753	GCA_003800495.1	Penicillium sp. SPG-F1 (ascomycetes)
2488754	GCA_003800485.1	Penicillium sp. SPG-F15 (ascomycetes)
2138086	GCA_004959885.1	Penicillium sp. W3 MMC-2018 (ascomycetes)
303698	GCA_002072375.1	Penicillium steckii (ascomycetes)
1316194	GCA_001908125.1	Penicillium subrubescens (ascomycetes)
60171	GCA_000970515.2	Penicillium verrucosum (ascomycetes)
29845	GCA_002072255.1	Penicillium vulpinum (ascomycetes)
1314672	GCA_001632445.1	Peniophora sp. CONT (basidiomycetes)
97972	GCA_003073855.1	Periconia macrospinosa (ascomycetes)
1037531	GCA_000222875.2	Periglandula ipomoeae IasaF13 (ascomycetes)
1229662	GCA_000516985.1	Pestalotiopsis fici W106-1 (ascomycetes)
1633207	GCA_001599175.1	Pestalotiopsis sp. JCM 9685 (ascomycetes)
1196395	GCA_003706045.1	Peterozyma toletana (budding yeasts)
1337056	GCA_003706035.1	Peterozyma xylosa (budding yeasts)
1663500	GCA_003008705.1	Pezicula radicicola (ascomycetes)
1745343	GCA_002865625.1	Pezoloma ericae (ascomycetes)
1286976	GCA_000392275.1	Phaeoacremonium minimum UCRPA7 (ascomycetes)
158046	GCA_001006345.1	Phaeomoniella chlamydospora (ascomycetes)
2304317	GCA_003501895.1	Phaeosphaeria sp. A1 3.1a (ascomycetes)
2304319	GCA_003503105.1	Phaeosphaeria sp. H6.2b (ascomycetes)
334123	GCA_001599715.1	Phaeotremella fagi (basidiomycetes)
89928	GCA_001599695.1	Phaeotremella skinneri (basidiomycetes)
264483	GCA_001579715.1	Phaffia rhodozyma (basidiomycetes)
54045	GCA_003707195.1	Phaffomyces antillensis (budding yeasts)
54194	GCA_003707165.1	Phaffomyces opuntiae (budding yeasts)
54046	GCA_003707215.1	Phaffomyces thermotolerans (budding yeasts)
650164	GCA_000300595.1	Phanerochaete carnosa HHB-10118-sp (basidiomycetes)
5306	GCA_001910725.1	Phanerochaete chrysosporium (basidiomycetes)
167371	GCA_004802695.1	Phellinidium pouzarii (basidiomycetes)
1156444	GCA_002794735.1	Phellinus lamaoensis (basidiomycetes)
1093900	GCA_004353045.1	Phialemoniopsis curvata (ascomycetes)
149040	GCA_001500285.1	Phialocephala scopiformis (ascomycetes)
2316158	GCA_003988865.1	Phialocephala sp. D728 (ascomycetes)
576137	GCA_900073065.1	Phialocephala subalpina (ascomycetes)
5601	GCA_000835435.1	Phialophora americana (ascomycetes)
1664694	GCA_001299255.1	Phialophora attae (ascomycetes)

39412	GCA_002099365.1	Phialophora verrucosa (ascomycetes)
1960876	GCA_003698115.1	Phialosimplex sp. HF37 (ascomycetes)
98765	GCA_001913855.2	Phlebia centrifuga (basidiomycetes)
745531	GCA_000832265.1	Phlebiopsis gigantea 11061_1 CR5-6 (basidiomycetes)
80661	GCA_000766925.2	Phlebopus portentosus (basidiomycetes)
1538424	GCA_003314615.1	Pholiota microspora (basidiomycetes)
73001	GCA_001599375.1	Phoma herbarum (ascomycetes)
507361	GCA_004151165.1	Phoma koolunga (ascomycetes)
2490640	GCA_004335185.1	Phoma sp. RAV-16-625 (ascomycetes)
2081634	GCA_004835665.1	Phoma sp. XZ068 (ascomycetes)
2608753	GCA_000800745.1	Phomopsis longicolla (ascomycetes)
763407	GCA_001638985.2	Phycomyces blakesleeanus NRRL 1555(-) (fungi)
121624	GCA_001604925.1	Phyllosticta capitalensis (ascomycetes)
55181	GCA_001604955.1	Phyllosticta citricarpa (ascomycetes)
1171599	GCA_003707355.1	Pichia exigua (budding yeasts)
53655	GCA_003339355.1	Pichia fermentans (budding yeasts)
53659	GCA_003705525.2	Pichia heedii (budding yeasts)
36015	GCA_003327685.1	Pichia kluyveri (budding yeasts)
4909	GCA_003054445.1	Pichia kudriavzevii (budding yeasts)
121235	GCA_005406165.1	Pichia manshurica (budding yeasts)
763406	GCA_001661235.1	Pichia membranifaciens NRRL Y-2026 (budding yeasts)
53656	GCA_003705495.1	Pichia nakasei (budding yeasts)
4921	GCA_003705465.1	Pichia norvegensis (budding yeasts)
54552	GCA_003705455.2	Pichia occidentalis (budding yeasts)
54551	GCA_003706875.2	Pichia terricola (budding yeasts)
765440	GCA_000827315.1	Piloderma croceum F 1598 (basidiomycetes)
1907219	GCA_003614145.1	Piptocephalis cylindrospora (fungi)
1754191	GCA_002104945.1	Piromyces finnis (chytrids)
73868	GCA_002157105.1	Piromyces sp. E2 (chytrids)
1603990	GCA_003988945.1	Pirottaea palmicola (ascomycetes)
765257	GCA_000827275.1	Pisolithus microcarpus 441 (basidiomycetes)
870435	GCA_000827335.1	Pisolithus tinctorius Marx 270 (basidiomycetes)
1162309	GCA_000263175.2	Pleosporales sp. UM 1110 (ascomycetes)
98342	GCA_003314595.1	Pleurotus citrinopileatus (basidiomycetes)
5323	GCA_003243765.1	Pleurotus eryngii (basidiomycetes)
2048520	GCA_005298045.1	Pleurotus ostreatoroseus (basidiomycetes)
5322	GCA_003313235.2	Pleurotus ostreatus (oyster mushroom)
2015914	GCA_003313735.1	Pleurotus platypus (basidiomycetes)
64638	GCA_002583695.1	Pleurotus salmoneostramineus (basidiomycetes)
716892	GCA_003314355.1	Pleurotus tuber-regium (basidiomycetes)
879823	GCA_003243755.1	Pleurotus tuoliensis (basidiomycetes)
944288	GCA_000827205.1	Plicaturopsis crispa FD-325 SS-3 (basidiomycetes)
2316157	GCA_003988805.1	Ploettnerulaceae sp. D365 (ascomycetes)
181527	GCA_004369065.1	Pluteus cervinus (basidiomycetes)
1408658	GCA_001477545.1	Pneumocystis carinii B80 (ascomycetes)
1408657	GCA_001477535.1	Pneumocystis jirovecii RU7 (ascomycetes)

1069680	GCA_000349005.2	Pneumocystis murina B123 (ascomycetes)
1380566	GCA_001653235.2	Pochonia chlamydosporia 170 (ascomycetes)
515849	GCA_000226545.1	Podospora anserina S mat+ (ascomycetes)
48703	GCA_900290415.1	Podospora comata (ascomycetes)
1314778	GCA_004369055.1	Polyporus arcularius HHB13444 (basidiomycetes)
139420	GCA_001792895.1	Polyporus brumalis (basidiomycetes)
1447883	GCA_002573605.1	Polytolypa hystricis UAMH7299 (ascomycetes)
108901	GCA_002794775.1	Porodaedalea pini (basidiomycetes)
670580	GCA_002117355.1	Postia placenta MAD-698-R-SB12 (basidiomycetes)
1712568	GCA_001553865.1	Preussia sp. BSL10 (ascomycetes)
28549	GCA_003709225.2	Priceomyces carsonii (budding yeasts)
45501	GCA_003706855.2	Priceomyces castillae (budding yeasts)
45502	GCA_001599895.1	Priceomyces haplophilus (budding yeasts)
45504	GCA_003705335.1	Priceomyces medius (budding yeasts)
181175	GCA_002335605.1	Prillingera fragicola (basidiomycetes)
2283848	GCA_003988795.1	Proliferodiscus dingleyae (ascomycetes)
42469	GCA_003717255.1	Protomyces gravidus (ascomycetes)
27332	GCA_003717155.1	Protomyces inouyei (ascomycetes)
27333	GCA_003717165.1	Protomyces inundatus (ascomycetes)
56484	GCA_002105105.1	Protomyces lactucaedebilis (ascomycetes)
48147	GCA_003717175.1	Protomyces macrosporus (ascomycetes)
48148	GCA_003717275.1	Protomyces pachydermus (ascomycetes)
1882900	GCA_003568695.1	Protomyces sp. C29 (ascomycetes)
2316362	GCA_004126415.1	Psathyrella aberdarensis (basidiomycetes)
136282	GCA_003184345.1	Pseudevernia furfuracea (ascomycetes)
321146	GCA_001578235.1	Pseudocercospora eumusae (ascomycetes)
383855	GCA_000340215.1	Pseudocercospora fijiensis CIRAD86 (ascomycetes)
113226	GCA_001578225.1	Pseudocercospora musae (ascomycetes)
1367541	GCA_000504365.2	Pseudocercospora pini-densiflorae CBS 125139 (ascomycetes)
655981	GCA_001641265.1	Pseudogymnoascus destructans (ascomycetes)
79859	GCA_001630605.1	Pseudogymnoascus pannorum var. pannorum (ascomycetes)
1622148	GCA_001662645.1	Pseudogymnoascus sp. 03VT05 (ascomycetes)
1752145	GCA_001630575.1	Pseudogymnoascus sp. 04NY16 (ascomycetes)
1622149	GCA_001662605.1	Pseudogymnoascus sp. 05NY08 (ascomycetes)
1524831	GCA_001662575.1	Pseudogymnoascus sp. 23342-1-I1 (ascomycetes)
1622150	GCA_001662595.1	Pseudogymnoascus sp. 24MN13 (ascomycetes)
1436940	GCA_001630595.1	Pseudogymnoascus sp. BL308 (ascomycetes)
1436941	GCA_001630585.1	Pseudogymnoascus sp. BL549 (ascomycetes)
1420912	GCA_000750895.1	Pseudogymnoascus sp. VKM F-103 (ascomycetes)
1437433	GCA_000750665.1	Pseudogymnoascus sp. VKM F-3557 (ascomycetes)
1420901	GCA_000750715.1	Pseudogymnoascus sp. VKM F-3775 (ascomycetes)
1391699	GCA_000750675.1	Pseudogymnoascus sp. VKM F-3808 (ascomycetes)
1420902	GCA_000750735.1	Pseudogymnoascus sp. VKM F-4246 (ascomycetes)
1420906	GCA_000750745.1	Pseudogymnoascus sp. VKM F-4281 (FW-2241) (ascomycetes)

1420907	GCA_000750755.1	Pseudogymnoascus sp. VKM F-4513 (FW-928) (ascomycetes)
1420908	GCA_000750795.1	Pseudogymnoascus sp. VKM F-4514 (FW-929) (ascomycetes)
1420909	GCA_000750805.1	Pseudogymnoascus sp. VKM F-4515 (FW-2607) (ascomycetes)
1420910	GCA_000750815.1	Pseudogymnoascus sp. VKM F-4516 (FW-969) (ascomycetes)
1420911	GCA_000750875.1	Pseudogymnoascus sp. VKM F-4517 (FW-2822) (ascomycetes)
1420913	GCA_000750925.1	Pseudogymnoascus sp. VKM F-4518 (FW-2643) (ascomycetes)
1420914	GCA_000750935.1	Pseudogymnoascus sp. VKM F-4519 (FW-2642) (ascomycetes)
1420915	GCA_000750995.1	Pseudogymnoascus sp. VKM F-4520 (FW-2644) (ascomycetes)
1622147	GCA_001662585.1	Pseudogymnoascus sp. WSF 3629 (ascomycetes)
342668	GCA_001662655.1	Pseudogymnoascus verrucosus (ascomycetes)
42149	GCA_003049395.1	Pseudohalonectria lignicola (ascomycetes)
146866	GCA_001432165.1	Pseudoloma neurophilia (microsporidians)
1141098	GCA_002105095.1	Pseudomassariella vexata (ascomycetes)
1684307	GCA_003144135.1	Pseudomicrostroma glucosiphilum (basidiomycetes)
1053252	GCA_003693545.1	Pseudonectria buxi (ascomycetes)
1634538	GCA_003693505.1	Pseudonectria foliicola (ascomycetes)
1671306	GCA_003868215.1	Pseudophaeomoniella oleicola (ascomycetes)
285811	GCA_003313425.1	Pseudopyrenochaeta lycopersici (ascomycetes)
1305764	GCA_000403515.1	Pseudozyma hubeiensis SY62 (smut fungi)
2039271	GCA_003004685.1	Pseudozyma sp. F8B2 (smut fungi)
5483	GCA_001736125.1	Pseudozyma tsukubaensis (smut fungi)
93625	GCA_002938375.1	Psilocybe cyanescens (basidiomycetes)
1884261	GCA_004369125.1	Pterula gracilis (basidiomycetes)
333523	GCA_001013415.1	Puccinia arachidis (rust fungi)
200324	GCA_002873125.1	Puccinia coronata var. avenae f. sp. avenae (rust fungi)
418459	GCA_000149925.1	Puccinia graminis f. sp. tritici CRL 75-36-700-3 (rust fungi)
331382	GCA_001624995.1	Puccinia horiana (rust fungi)
1904429	GCA_004348175.1	Puccinia novopanici (rust fungi)
27349	GCA_001263375.1	Puccinia sorghi (rust fungi)
168172	GCA_002900275.1	Puccinia striiformis f. sp. tritici (wheat yellow rust)
630390	GCA_000151525.2	Puccinia triticina 1-1 BBBD Race 1 (wheat leaf rust)
80668	GCA_003316545.1	Pulveroboletus ravenelii (basidiomycetes)
741275	GCA_000264995.1	Punctularia strigosozonata HHB-11173 SS5 (basidiomycetes)
33203	GCA_001653265.1	Purpureocillium lilacinum (ascomycetes)
765867	GCA_001644535.1	Pyrenochaeta sp. DS3sAY3a (ascomycetes)
1295359	GCA_000359685.2	Pyrenochaeta sp. UM 256 (ascomycetes)
1302712	GCA_000465215.2	Pyrenophora seminiperda CCB06 (ascomycetes)
97479	GCA_900232045.1	Pyrenophora teres f. teres (barley net-spot blotch disease agent)
426418	GCA_000149985.1	Pyrenophora tritici-repentis Pt-1C-BFP (ascomycetes)

148305	GCA_004355905.1	Pyricularia grisea (ascomycetes)
242507	GCA_000002495.2	Pyricularia oryzae 70-15 (rice blast fungus)
1578925	GCA_004337985.1	Pyricularia pennisetigena (ascomycetes)
403063	GCA_004337975.1	Pyricularia sp. CBS 133598 (ascomycetes)
2282107	GCA_002287475.1	Pyrrhoderma noxium (basidiomycetes)
363177	GCA_004016185.1	Quambalaria eucalypti (basidiomycetes)
1507870	GCA_002077065.1	Rachicladosporium antarcticum (ascomycetes)
1974281	GCA_002077045.2	Rachicladosporium sp. CCFEE 5018 (ascomycetes)
45341	GCA_002778245.1	Raffaelea albimanens (ascomycetes)
45346	GCA_002778195.1	Raffaelea ambrosiae (ascomycetes)
45342	GCA_002778165.1	Raffaelea arxii (ascomycetes)
483707	GCA_002778145.1	Raffaelea lauricola (ascomycetes)
637633	GCA_002778125.1	Raffaelea quercivora (ascomycetes)
1423150	GCA_002215975.1	Raffaelea quercus-mongolicae (ascomycetes)
2036197	GCA_002777955.1	Raffaelea sp. RL272 (ascomycetes)
490887	GCA_002778055.1	Raffaelea sulphurea (ascomycetes)
86788	GCA_003073195.1	Ramalina intermedia (ascomycetes)
475428	GCA_001956345.1	Ramalina peruviana (ascomycetes)
2016387	GCA_003316465.1	Ramaria cf. rubripermanens (basidiomycetes)
2175970	GCA_003314545.1	Ramaria sp. MG151 (basidiomycetes)
508918	GCA_002368545.1	Ramichloridium luteum (ascomycetes)
475927	GCA_004155245.1	Ramularia coccinea (ascomycetes)
112498	GCA_900074925.1	Ramularia collo-cygni (ascomycetes)
1367538	GCA_002116395.1	Ramularia endophylla CBS 113265 (ascomycetes)
1408163	GCA_000968595.1	Rasamsonia emersonii CBS 393.64 (ascomycetes)
1282276	GCA_003316065.1	Retiboletus ornatipes (basidiomycetes)
1028407	GCA_001557505.1	Reticulascus tulasneorum (ascomycetes)
1442369	GCA_000835555.1	Rhinocladiella mackenziei CBS 650.93 (ascomycetes)
329046	GCA_002104985.1	Rhizoclosmatium globosum (chytrids)
1086054	GCA_000524645.1	Rhizoctonia solani AG-3 Rhs1AP (basidiomycetes)
1031333	GCA_000611695.1	Rhizomucor miehei CAU432 (fungi)
4840	GCA_900175165.2	Rhizomucor pusillus (fungi)
94130	GCA_003203555.1	Rhizophagus clarus (glomeromycetes)
747089	GCA_000439145.3	Rhizophagus irregularis DAOM 181602=DAOM 197198 (glomeromycetes)
1803374	GCA_003549995.1	Rhizophagus sp. MUCL 43196 (glomeromycetes)
64517	GCA_002214945.1	Rhizophlyctis rosea (chytrids)
87122	GCA_002995455.1	Rhizopogon fuscorubens (basidiomycetes)
98233	GCA_002994995.1	Rhizopogon hawkerae (basidiomycetes)
87126	GCA_002994865.1	Rhizopogon parksii (basidiomycetes)
181343	GCA_002995055.1	Rhizopogon rudus (basidiomycetes)
176626	GCA_002995475.1	Rhizopogon salebrosus (basidiomycetes)
2107715	GCA_002995095.1	Rhizopogon sp. AM-2018a (basidiomycetes)
180088	GCA_001882365.1	Rhizopogon vesiculosus (basidiomycetes)
80599	GCA_002995315.1	Rhizopogon villosulus (basidiomycetes)
1314800	GCA_001658105.1	Rhizopogon vinicolor AM-OR11-026 (basidiomycetes)
36057	GCA_002995295.1	Rhizopogon vulgaris (basidiomycetes)

86630	GCA_003325435.1	Rhizopus azygosporus (fungi)
246409	GCA_000149305.1	Rhizopus delemar RA 99-880 (fungi)
1340429	GCA_002708625.1	Rhizopus microsporus ATCC 52813 (fungi)
1279482	GCA_000697725.1	Rhizopus oryzae 99-892 (fungi)
1274793	GCA_000697035.1	Rhizopus stolonifer B9770 (fungi)
5288	GCA_006352295.1	Rhodotorula diobovata (basidiomycetes)
578459	GCA_001329695.1	Rhodotorula graminis WP1 (basidiomycetes)
86836	GCA_002917965.1	Rhodotorula kratochvilovae (basidiomycetes)
5537	GCA_002806785.1	Rhodotorula mucilaginosa (basidiomycetes)
86838	GCA_005281665.1	Rhodotorula paludigena (basidiomycetes)
1965284	GCA_005059875.1	Rhodotorula sp. CCFEE 5036 (basidiomycetes)
1928479	GCA_001914285.1	Rhodotorula sp. FNED7-22 (basidiomycetes)
1305733	GCA_001541205.1	Rhodotorula sp. JG-1b (basidiomycetes)
741276	GCA_002922495.1	Rhodotorula taiwanensis (basidiomycetes)
1130832	GCA_000320785.2	Rhodotorula toruloides NP11 (basidiomycetes)
914238	GCA_900074905.1	Rhynchosporium agropyri (ascomycetes)
914237	GCA_900074885.1	Rhynchosporium commune (ascomycetes)
38038	GCA_900074895.1	Rhynchosporium secalis (leaf blotch of barley)
1136865	GCA_000467735.1	Rhytidhysteron rufulum CBS 306.38 (ascomycetes)
50990	GCA_004355085.1	Rickenella mellea (basidiomycetes)
77044	GCA_001445595.3	Rosellinia necatrix (ascomycetes)
988480	GCA_000442015.1	Rozella allomycis CSF55 (fungi)
482377	GCA_003313715.1	Russula abietina (basidiomycetes)
2017508	GCA_003313875.1	Russula aff. compacta (basidiomycetes)
131541	GCA_003316565.1	Russula foetens (basidiomycetes)
466936	GCA_004801975.1	Russula griseocarnosa (basidiomycetes)
152963	GCA_003316425.1	Russula lepida (basidiomycetes)
2175969	GCA_003313725.1	Russula sp. MG48 (basidiomycetes)
71688	GCA_003316435.1	Russula virescens (basidiomycetes)
2070414	GCA_002946425.1	Rutstroemia sp. NJR-2017a BBW (ascomycetes)
2070413	GCA_002946415.1	Rutstroemia sp. NJR-2017a BVV2 (ascomycetes)
2070412	GCA_002946385.1	Rutstroemia sp. NJR-2017a WRK4 (ascomycetes)
54687	GCA_000812895.1	Rutstroemia sydowiana (ascomycetes)
226231	GCA_000167035.1	Saccharomyces bayanus 623-6C (budding yeasts)
1095631	GCA_000326105.1	Saccharomyces cerevisiae x Saccharomyces kudriavzevii VIN7 (budding yeasts)
1080349	GCA_001298625.1	Saccharomyces eubayanus (budding yeasts)
1987369	GCA_900290405.1	Saccharomyces jurei (budding yeasts)
114524	GCA_003327635.1	Saccharomyces kudriavzevii (budding yeasts)
226126	GCA_000166975.1	Saccharomyces mikatae IFO 1815 (budding yeasts)
27291	GCA_002079145.1	Saccharomyces paradoxus (budding yeasts)
520522	GCA_001515485.2	Saccharomyces pastorianus Weihenstephan 34/70 (lager yeast)
252598	GCA_001413975.1	Saccharomyces sp. 'boulardii' (budding yeasts)
1337648	GCA_002375215.1	Saccharomyces sp. M14 (budding yeasts)
230603	GCA_002242645.1	Saccharomyces uvarum (budding yeasts)
566037	GCA_000412225.2	Saccharomycetaceae sp. 'Ashbya aceri' (budding yeasts)

36035	GCA_900491785.1	Saccharomycodes ludwigii (budding yeasts)
36036	GCA_003705375.1	Saccharomycopsis capsularis (budding yeasts)
53486	GCA_002564245.1	Saccharomycopsis fermentans (budding yeasts)
4944	GCA_001936275.1	Saccharomycopsis fibuligera (budding yeasts)
1725355	GCA_001936135.1	Saccharomycopsis fibuligera x Saccharomycopsis cf. fibuligera (budding yeasts)
1305767	GCA_002564235.1	Saccharomycopsis fodiens (budding yeasts)
44092	GCA_004014935.1	Saccharomycopsis malanga (budding yeasts)
1504575	GCA_002895615.1	Saccharomycopsis sp. UWO(PS) 91-127.1 (budding yeasts)
698492	GCA_001661265.1	Saitoella complicata NRRL Y-17804 (ascomycetes)
1890683	GCA_003942215.1	Saitozyma podzolica (basidiomycetes)
1357693	GCA_000697495.1	Saksenaea oblongispora B3353 (fungi)
1274794	GCA_000697055.1	Saksenaea vasiformis B4078 (fungi)
392538	GCA_001990185.1	Salmacisia buchloeana (basidiomycetes)
108892	GCA_001481415.2	Sanghuangporus baumii (basidiomycetes)
1734004	GCA_900654225.1	Saprochaete fungicola (budding yeasts)
44074	GCA_900642975.1	Saprochaete suaveolens (budding yeasts)
155012	GCA_003313825.1	Sarcodon aspratus (basidiomycetes)
2175968	GCA_003313065.1	Sarcodon sp. MG97 (basidiomycetes)
284135	GCA_001972265.1	Sarocladium oryzae (ascomycetes)
5046	GCA_900290465.1	Sarocladium strictum (ascomycetes)
29835	GCA_003243065.1	Saturnispora dispora (budding yeasts)
297360	GCA_003707255.1	Saturnispora hagleri (budding yeasts)
324741	GCA_003705365.1	Saturnispora mendoncae (budding yeasts)
58634	GCA_003707305.1	Saturnispora saitoi (budding yeasts)
756769	GCA_003705415.1	Saturnispora serradocipensis (budding yeasts)
52249	GCA_003707315.2	Saturnispora silvae (budding yeasts)
58635	GCA_003705435.1	Saturnispora zaruensis (budding yeasts)
563466	GCA_000732125.1	Scedosporium apiospermum (ascomycetes)
315946	GCA_000812075.1	Scedosporium aurantiacum (ascomycetes)
5597	GCA_002221725.1	Scedosporium boydii (ascomycetes)
563467	GCA_002812735.1	Scedosporium dehoogii (ascomycetes)
2562444	GCA_001931805.1	Scedosporium sp. IMV 00882 (ascomycetes)
1111081	GCA_001599395.1	Scheffersomyces lignosus (budding yeasts)
45589	GCA_002118035.1	Scheffersomyces shehatae (budding yeasts)
1751990	GCA_002245345.1	Scheffersomyces stambukii (budding yeasts)
322104	GCA_000209165.1	Scheffersomyces stipitis CBS 6054 (budding yeasts)
578458	GCA_000143185.1	Schizophyllum commune H4-8 (basidiomycetes)
27342	GCA_001020605.1	Schizopora paradoxa (basidiomycetes)
653667	GCA_000004155.2	Schizosaccharomyces cryophilus OY26 (ascomycetes)
402676	GCA_000149845.2	Schizosaccharomyces japonicus yFS275 (ascomycetes)
483514	GCA_000150505.2	Schizosaccharomyces octosporus yFS286 (ascomycetes)
4896	GCA_000002945.2	Schizosaccharomyces pombe (fission yeast)
1036808	GCA_000827425.1	Scleroderma citrinum Foug A (basidiomycetes)
1432307	GCA_000503235.1	Sclerotinia borealis F-4128 (ascomycetes)
107426	GCA_002162575.1	Sclerotinia glacialis (ascomycetes)

665079	GCA_000146945.2	Sclerotinia sclerotiorum 1980 UF-70 (ascomycetes)
38492	GCA_002162485.1	Sclerotium cepivorum (ascomycetes)
5539	GCA_002812745.2	Scytalidium lignicola (ascomycetes)
2487125	GCA_000743665.3	Scytalidium sp. 3C (ascomycetes)
1109443	GCA_000313545.1	Serendipita indica DSM 11827 (basidiomycetes)
933852	GCA_000827415.1	Serendipita vermifera MAFF 305830 (basidiomycetes)
578457	GCA_000218685.1	Serpula lacrymans var. lacrymans S7.9 (dry rot fungus)
665115	GCA_000498155.1	Shiraia sp. slf14 (ascomycetes)
1314777	GCA_001630475.1	Sistotremastrum niveocremeum HHB9708 (basidiomycetes)
1314776	GCA_001632355.1	Sistotremastrum suecicum HHB10207 ss-3 (basidiomycetes)
133377	GCA_003097675.1	Smittium angustum (fungi)
133412	GCA_001970855.1	Smittium culicis (fungi)
133381	GCA_003086715.1	Smittium megazygosporum (fungi)
133383	GCA_001953115.1	Smittium mucronatum (fungi)
133385	GCA_003086735.1	Smittium simulii (fungi)
1314773	GCA_003711515.1	Sodiomyces alkalinus F11 (ascomycetes)
104411	GCA_001600015.1	Solicoccozyma phenolica (basidiomycetes)
104414	GCA_001600875.1	Solicoccozyma terricola (basidiomycetes)
771870	GCA_000182805.2	Sordaria macrospora k-hell (ascomycetes)
139825	GCA_003851025.1	Sparassis crispa (basidiomycetes)
1202976	GCA_002607745.1	Sparassis latifolia (basidiomycetes)
1343157	GCA_000497715.1	Spathaspora arborariae UFMG-19.1A (budding yeasts)
1701548	GCA_002094185.1	Spathaspora boniae (budding yeasts)
1774708	GCA_001657455.1	Spathaspora girioi (budding yeasts)
1852163	GCA_001655765.1	Spathaspora gorwiae (budding yeasts)
1774706	GCA_001655755.1	Spathaspora hagerdaliae (budding yeasts)
619300	GCA_000223485.1	Spathaspora passalidarum NRRL Y-27907 (budding yeasts)
2028339	GCA_003676035.1	Spathaspora sp. JA1 (budding yeasts)
1864610	GCA_002911495.1	Spathaspora sp. UFMG-CM-Y6060 (budding yeasts)
1699422	GCA_002105455.1	Spathaspora xylofermentans (budding yeasts)
933994	GCA_003705305.2	Spencermartinsiella europaea (budding yeasts)
2082308	GCA_002895985.1	Sphaceloma murrayae (ascomycetes)
990650	GCA_000827215.1	Sphaerobolus stellatus SS14 (basidiomycetes)
692275	GCA_000320565.2	Sphaerulina musiva SO2202 (ascomycetes)
1136490	GCA_000291705.1	Sphaerulina populicola P02.02b (ascomycetes)
645134	GCA_000182565.2	Spizellomyces punctatus DAOM BR117 (chytrids)
280036	GCA_005498985.1	Sporisorium graminicola (smut fungi)
882909	GCA_001748505.1	Sporisorium iseilematis-ciliati (smut fungi)
72559	GCA_900162835.1	Sporisorium reilianum f. sp. reilianum (smut fungi)
49012	GCA_001010845.1	Sporisorium scitamineum (smut fungi)
45794	GCA_003705295.1	Sporopachydermia lactativora (budding yeasts)
54094	GCA_001599295.1	Sporopachydermia quercuum (budding yeasts)
1398154	GCA_000820605.1	Sporothrix brasiliensis 5110 (ascomycetes)
545651	GCA_001630435.1	Sporothrix globosa (ascomycetes)

1081102	GCA_001636815.1	Sporothrix insectorum RCEF 264 (ascomycetes)
431197	GCA_000710705.2	Sporothrix pallida (ascomycetes)
1397361	GCA_000961545.1	Sporothrix schenckii 1099-18 (ascomycetes)
51541	GCA_001887945.1	Spraguea lophii (microsporidians)
74722	GCA_001021365.1	Stachybotrys chartarum (ascomycetes)
1283841	GCA_000732775.1	Stachybotrys chlorohalonata IBT 40285 (ascomycetes)
765868	GCA_001644525.1	Stagonospora sp. SRC1lsM3a (ascomycetes)
1200839	GCA_000812845.1	Stagonosporopsis tanaceti (ascomycetes)
53643	GCA_003708085.1	Starmera amethionina (budding yeasts)
53646	GCA_003705275.1	Starmera quercuum (budding yeasts)
29830	GCA_001005415.1	Starmerella apicola (budding yeasts)
1247836	GCA_002270425.1	Starmerella bacillaris (budding yeasts)
75736	GCA_001599315.1	Starmerella bombicola (budding yeasts)
2528786	GCA_004124985.1	Starmerella davenportii (budding yeasts)
46361	GCA_004125165.1	Starmerella geochares (budding yeasts)
45555	GCA_004125075.1	Starmerella gropengiesseri (budding yeasts)
1008382	GCA_004124915.1	Starmerella kuoi (budding yeasts)
5490	GCA_003033435.1	Starmerella magnoliae (budding yeasts)
225544	GCA_004124975.1	Starmerella ratchasimensis (budding yeasts)
308924	GCA_004124955.1	Starmerella riodocensis (budding yeasts)
140312	GCA_004125005.1	Starmerella sorbosivorans (budding yeasts)
121468	GCA_004125055.1	Starmerella sp. 'tilneyi' (budding yeasts)
45600	GCA_004125185.1	Starmerella vaccinii (budding yeasts)
92696	GCA_004332605.1	Steccherinum ochraceum (basidiomycetes)
183478	GCA_001191545.1	Stemphylium lycopersici (ascomycetes)
119933	GCA_004380135.1	Stemphylium vesicarium (ascomycetes)
238245	GCA_002270565.1	Stenocarpella maydis (ascomycetes)
721885	GCA_000264905.1	Stereum hirsutum FP-91666 SS1 (basidiomycetes)
68746	GCA_003314255.1	Stropharia rugosoannulata (basidiomycetes)
2059718	GCA_005111325.1	Stylopage hadra (fungi)
796027	GCA_001640025.2	Sugiyamaella lignohabitans (budding yeasts)
1234609	GCA_001939105.2	Sugiyamaella xylanicola (budding yeasts)
147334	GCA_003706525.1	Suhomyces canberraensis (budding yeasts)
246006	GCA_003707835.2	Suhomyces emberorum (budding yeasts)
147331	GCA_003706505.2	Suhomyces pyralidae (budding yeasts)
984487	GCA_001661415.1	Suhomyces tanzawaensis NRRL Y-17324 (budding yeasts)
1930190	GCA_003316505.1	Suillus alpinus (basidiomycetes)
930992	GCA_000827255.1	Suillus luteus UH-Slu-Lm8-n1 (basidiomycetes)
222705	GCA_003313085.1	Suillus pictus (basidiomycetes)
48579	GCA_003313645.1	Suillus placidus (basidiomycetes)
2175967	GCA_003313855.1	Suillus sp. MG131 (basidiomycetes)
40220	GCA_001599915.1	Symbiotaphrina buchneri (ascomycetes)
1357682	GCA_000697355.1	Syncephalastrum monosporum B8922 (fungi)
13706	GCA_002105135.1	Syncephalastrum racemosum (fungi)
1712513	GCA_003614755.1	Syncephalis pseudoplumigaleata (fungi)
286115	GCA_001399855.1	Synchytrium endobioticum (chytrids)

196114	GCA_000766995.1	Taiwanofungus camphoratus (basidiomycetes)
1390368	GCA_003116875.1	Takashimella koratensis (basidiomycetes)
211102	GCA_003116915.1	Takashimella tepidaria (basidiomycetes)
1974180	GCA_002775195.1	Talaromyces adpressus (ascomycetes)
1196081	GCA_001896365.1	Talaromyces amestolkiae (ascomycetes)
1441469	GCA_001907595.1	Talaromyces atroroseus (ascomycetes)
2077152	GCA_002916415.1	Talaromyces borbonicus (ascomycetes)
1472165	GCA_000829775.1	Talaromyces cellulolyticus (ascomycetes)
28572	GCA_004299765.1	Talaromyces funiculosus (ascomycetes)
28573	GCA_000985935.1	Talaromyces islandicus (ascomycetes)
441960	GCA_000001985.1	Talaromyces marneffei ATCC 18224 (ascomycetes)
153982	GCA_001657655.1	Talaromyces piceae (ascomycetes)
128442	GCA_001571465.2	Talaromyces pinophilus (ascomycetes)
1266744	GCA_001270325.1	Talaromyces purpureogenus (ascomycetes)
441959	GCA_000003125.1	Talaromyces stipitatus ATCC 10500 (ascomycetes)
198730	GCA_001305275.1	Talaromyces verruculosus (ascomycetes)
28567	GCA_001939245.1	Talaromyces wortmannii (ascomycetes)
71559	GCA_005281525.1	Taphrina communis (ascomycetes)
203082	GCA_005281535.1	Taphrina confusa (ascomycetes)
5011	GCA_005281575.1	Taphrina deformans (peach leaf curl fungus)
1450759	GCA_000836175.1	Taphrina flavorubra JCM 22207 (ascomycetes)
1450760	GCA_000836195.1	Taphrina populina JCM 22190 (ascomycetes)
48692	GCA_005281585.1	Taphrina pruni (ascomycetes)
2305253	GCA_004000165.1	Taphrina sp. SM11 (ascomycetes)
5012	GCA_005281515.1	Taphrina wiesneri (ascomycetes)
1149723	GCA_001969225.1	Taxomyces andreanae CBS 279.92 (ascomycetes)
418781	GCA_004009835.1	Teratosphaeria destructans (ascomycetes)
1051890	GCA_003788615.2	Terfezia boudieri ATCC MYA-4762 (ascomycetes)
171347	GCA_003316525.1	Termitomyces eurrhizus (basidiomycetes)
71929	GCA_003313675.1	Termitomyces heimii (basidiomycetes)
1306850	GCA_001263195.1	Termitomyces sp. J132 (basidiomycetes)
1635102	GCA_001972325.1	Termitomyces sp. JCM 13351 (basidiomycetes)
2175966	GCA_003313055.1	Termitomyces sp. MG145 (basidiomycetes)
2175965	GCA_003313785.1	Termitomyces sp. MG148 (basidiomycetes)
2175964	GCA_003313075.1	Termitomyces sp. MG16 (basidiomycetes)
1882483	GCA_003144125.1	Testicularia cyperi (smut fungi)
1071380	GCA_000315915.1	Tetrapisispora blattae CBS 6284 (budding yeasts)
300277	GCA_003707605.1	Tetrapisispora fleetii (budding yeasts)
113606	GCA_003705975.1	Tetrapisispora iriomotensis (budding yeasts)
279732	GCA_003705985.1	Tetrapisispora namnaoensis (budding yeasts)
1071381	GCA_000236905.1	Tetrapisispora phaffii CBS 4417 (budding yeasts)
268492	GCA_003706575.1	Teunomyces cretensis (budding yeasts)
392264	GCA_003706565.2	Teunomyces gatunensis (budding yeasts)
45561	GCA_003706535.1	Teunomyces kruisii (budding yeasts)
78915	GCA_003614735.1	Thamnocephalis sphaerospora (fungi)
469304	GCA_900291925.1	Thecaphora thlaspeos (basidiomycetes)

654496	GCA_003316405.1	Thelephora aurantiotincta (basidiomycetes)
2562446	GCA_003123655.1	Thermoascaceae sp. COH1141 (ascomycetes)
5088	GCA_001599835.1	Thermoascus crustaceus (ascomycetes)
101121	GCA_000787465.1	Thermomucor indicae-seudaticae (fungi)
1158138	GCA_000315935.1	Thermomyces lanuginosus SSBP (ascomycetes)
573729	GCA_000226095.1	Thermothelomyces thermophilus ATCC 42464 (ascomycetes)
578455	GCA_000226115.1	Thermothielavioides terrestris NRRL 8126 (ascomycetes)
1526549	GCA_001599055.1	Thielaviopsis ethacetica (ascomycetes)
1346820	GCA_001599615.1	Thielaviopsis euricoi (ascomycetes)
1580842	GCA_001513885.1	Thielaviopsis musarum (ascomycetes)
72032	GCA_000968615.1	Thielaviopsis punctulata (ascomycetes)
13290	GCA_001645005.1	Tilletia caries (wheat bunt fungus)
13291	GCA_001645045.1	Tilletia controversa (dwarf bunt fungus)
155126	GCA_001006505.1	Tilletia horrida (rice bunt)
43049	GCA_001689995.1	Tilletia indica (Karnal bunt)
117179	GCA_001645055.1	Tilletia walkeri (basidiomycetes)
1037660	GCA_000711695.1	Tilletiaria anomala UBC 951 (basidiomycetes)
58919	GCA_003144115.1	Tilletiopsis washingtonensis (basidiomycetes)
45235	GCA_002901185.1	Tolypocladium capitatum (ascomycetes)
29910	GCA_003945565.1	Tolypocladium inflatum (ascomycetes)
1163406	GCA_001189435.1	Tolypocladium ophioglossoides CBS 100239 (ascomycetes)
94208	GCA_002916505.1	Tolypocladium paradoxum (ascomycetes)
1355412	GCA_000750145.2	Tolypocladium sp. Salcha MEA-2 (ascomycetes)
1501327	GCA_000750105.3	Tolypocladium sp. Sup5 PDA-1 (ascomycetes)
1531966	GCA_000825705.1	Torrubiella hemipterigena (ascomycetes)
767744	GCA_001661475.1	Tortispora caseinolytica NRRL Y-17796 (budding yeasts)
1326887	GCA_003707115.1	Tortispora ganteri (budding yeasts)
1326888	GCA_003706775.2	Tortispora starmeri (budding yeasts)
4950	GCA_000243375.1	Torulaspora delbrueckii (budding yeasts)
53488	GCA_003705175.2	Torulaspora franciscae (budding yeasts)
200334	GCA_003708055.2	Torulaspora maleeae (budding yeasts)
1136883	GCA_900186055.1	Torulaspora microellipsoides (budding yeasts)
35629	GCA_003706005.1	Torulaspora pretoriensis (budding yeasts)
72359	GCA_000316135.1	Trachipleistophora hominis (microsporidians)
5643	GCA_000765035.1	Trametes cinnabarina (basidiomycetes)
1353009	GCA_002092935.1	Trametes coccinea BRFM310 (basidiomycetes)
5327	GCA_001302255.2	Trametes hirsuta (basidiomycetes)
239206	GCA_001939255.1	Trametes polyzona (basidiomycetes)
154538	GCA_001895945.1	Trametes pubescens (basidiomycetes)
306582	GCA_001304625.1	Trametes sp. AH28-2 (basidiomycetes)
717944	GCA_000271585.1	Trametes versicolor FP-101664 SS1 (basidiomycetes)
47662	GCA_002964805.1	Trametes villosa (basidiomycetes)
64657	GCA_000987905.1	Tremella fuciformis (basidiomycetes)
578456	GCA_000271645.1	Tremella mesenterica DSM 1558 (witches' butter)
490622	GCA_003012105.1	Trichoderma arundinaceum (ascomycetes)

1042311	GCA_003025105.1	Trichoderma asperellum CBS 433.97 (ascomycetes)
1491457	GCA_003439915.1	Trichoderma atrobrunneum (ascomycetes)
452589	GCA_000171015.2	Trichoderma atroviride IMI 206040 (ascomycetes)
247546	GCA_003012085.1	Trichoderma brevicompactum (ascomycetes)
58853	GCA_003025115.1	Trichoderma citrinoviride (ascomycetes)
398673	GCA_001481775.2	Trichoderma gamsii (ascomycetes)
1491466	GCA_002022785.1	Trichoderma guizhouense (ascomycetes)
1247866	GCA_000331835.2	Trichoderma hamatum GD12 (ascomycetes)
983964	GCA_003025095.1	Trichoderma harzianum CBS 226.95 (ascomycetes)
97093	GCA_001950475.1	Trichoderma koningii (ascomycetes)
337941	GCA_002246955.1	Trichoderma koningiopsis (ascomycetes)
983965	GCA_003025155.1	Trichoderma longibrachiatum ATCC 18648 (ascomycetes)
858221	GCA_001050175.1	Trichoderma parareesei (ascomycetes)
500994	GCA_001721665.1	Trichoderma pleuroti (ascomycetes)
431241	GCA_000167675.2	Trichoderma reesei QM6a (ascomycetes)
2562443	GCA_001931985.1	Trichoderma sp. IMV 00454 (ascomycetes)
413071	GCA_000170995.2	Trichoderma virens Gv29-8 (ascomycetes)
51221	GCA_003313665.1	Tricholoma bakamatsutake (basidiomycetes)
80606	GCA_003313805.1	Tricholoma flavovirens (basidiomycetes)
40145	GCA_002939025.2	Tricholoma matsutake (matsutake)
113602	GCA_003313625.1	Tricholoma saponaceum (basidiomycetes)
2175963	GCA_003314665.1	Tricholoma sp. MG77 (basidiomycetes)
2175962	GCA_003521275.1	Tricholoma sp. MG99 (basidiomycetes)
76328	GCA_003316345.1	Tricholoma terreum (basidiomycetes)
663331	GCA_000151125.2	Trichophyton benhamiae CBS 112371 (ascomycetes)
559882	GCA_000151175.1	Trichophyton equinum CBS 127.97 (ascomycetes)
1215338	GCA_000622975.1	Trichophyton interdigitale MR816 (ascomycetes)
523103	GCA_003118255.1	Trichophyton mentagrophytes (ascomycetes)
559305	GCA_000151425.1	Trichophyton rubrum CBS 118892 (ascomycetes)
1215331	GCA_000616865.1	Trichophyton soudanense CBS 452.61 (ascomycetes)
647933	GCA_000151455.1	Trichophyton tonsurans CBS 112818 (ascomycetes)
663202	GCA_000151505.1	Trichophyton verrucosum HKI 0517 (ascomycetes)
34388	GCA_001651435.1	Trichophyton violaceum (ascomycetes)
279327	GCA_002973495.1	Trichosporon akiyoshidainum (basidiomycetes)
1186058	GCA_000293215.1	Trichosporon asahii var. asahii CBS 2479 (basidiomycetes)
82509	GCA_001752605.1	Trichosporon coremiiforme (basidiomycetes)
82510	GCA_001752585.1	Trichosporon faecale (basidiomycetes)
82517	GCA_001752625.1	Trichosporon inkin (basidiomycetes)
82524	GCA_001752645.1	Trichosporon ovoides (basidiomycetes)
508457	GCA_003012195.1	Trichothecium ovalisporum (ascomycetes)
47278	GCA_003012185.1	Trichothecium roseum (ascomycetes)
51912	GCA_003012115.1	Trichothecium sympodiale (ascomycetes)
34364	GCA_003707065.2	Trigonopsis variabilis (budding yeasts)
45603	GCA_003706805.2	Trigonopsis vinaria (budding yeasts)
42251	GCA_003070745.1	Tuber borchii (whitish truffle)
1894963	GCA_003316355.1	Tuber calosporum (ascomycetes)

55307	GCA_006112555.1	Tuber indicum (ascomycetes)
42249	GCA_003182015.1	Tuber magnatum (white Piedmont truffle)
39416	GCA_000151645.1	Tuber melanosporum (Perigord truffle)
1455713	GCA_003521225.1	Tuber microsphaerosporum (ascomycetes)
691171	GCA_003313605.1	Tuber umbilicatum (ascomycetes)
291195	GCA_004000155.1	Tubulinosema ratisbonensis (microsporidians)
1051891	GCA_000827465.1	Tulasnella calospora MUT 4182 (basidiomycetes)
374766	GCA_003316375.1	Tylopilus plumbeoviolaceoides (basidiomycetes)
1357687	GCA_000697415.1	Umbelopsis isabellina B7317 (fungi)
87280	GCA_000611775.1	Umbilicaria muehlenbergii (ascomycetes)
136370	GCA_900169345.1	Umbilicaria pustulata (ascomycetes)
336963	GCA_000003515.2	Uncinocarpus reesii 1704 (ascomycetes)
462795	GCA_002994575.1	Uromyces transversalis (rust fungi)
55588	GCA_000785685.1	Uromyces viciae-fabae (rust fungi)
1159556	GCA_002939685.1	Ustilaginoidea virens (ascomycetes)
307758	GCA_900080155.1	Ustilago bromivora (smut fungi)
63396	GCA_001736195.1	Ustilago cynodontis (smut fungi)
185366	GCA_000819925.1	Ustilago esculenta (smut fungi)
120017	GCA_003012045.1	Ustilago hordei (smut fungi)
237631	GCA_000328475.2	Ustilago maydis 521 (smut fungi)
86804	GCA_001654535.1	Ustilago trichophora (smut fungi)
117174	GCA_002993085.1	Ustilago tritici (smut fungi)
249491	GCA_001735935.1	Ustilago vetiveriae (smut fungi)
249492	GCA_001736075.1	Ustilago xerochloae (smut fungi)
105487	GCA_000818155.1	Valsa mali (ascomycetes)
356882	GCA_003795315.1	Valsa malicola (ascomycetes)
252740	GCA_003795275.1	Valsa sordida (ascomycetes)
436907	GCA_000150035.1	Vanderwaltozyma polyspora DSM 70294 (budding yeasts)
5417	GCA_001600235.1	Vanrija humicola (basidiomycetes)
948595	GCA_000192795.1	Vavraia culicis subsp. floridensis (microsporidians)
86256	GCA_003689335.1	Venturia asperata (ascomycetes)
470485	GCA_003693225.1	Venturia aucupariae (ascomycetes)
86257	GCA_001990985.1	Venturia carpophila (ascomycetes)
50376	GCA_001901625.1	Venturia effusa (ascomycetes)
5025	GCA_003689225.1	Venturia inaequalis (ascomycetes)
86259	GCA_004522655.2	Venturia nashicola (ascomycetes)
1437871	GCA_000738655.1	Venturia pyrina ICMP 11032 (ascomycetes)
2656787	GCA_003357145.1	Venustampulla echinocandica (ascomycetes)
253628	GCA_000836295.1	Verruconis gallopava (ascomycetes)
2011826	GCA_004026245.1	Verruconis sp. (ascomycetes)
27335	GCA_002851705.1	Verticillium albo-atrum (ascomycetes)
526221	GCA_000150825.1	Verticillium alfalfae VaMs.102 (ascomycetes)
498257	GCA_000150675.2	Verticillium dahliae VdLs.17 (ascomycetes)
1051614	GCA_002851775.1	Verticillium isaacii (ascomycetes)
1051615	GCA_002851715.1	Verticillium klebahnii (ascomycetes)
100787	GCA_001268145.1	Verticillium longisporum (ascomycetes)

1051616	GCA_003724135.1	Verticillium nonalfalfae (ascomycetes)
132580	GCA_002851675.1	Verticillium nubilum (ascomycetes)
1425883	GCA_000732205.1	Verticillium tricorpus MUCL 9792 (ascomycetes)
1051617	GCA_002851755.1	Verticillium zaregamsianum (ascomycetes)
1673888	GCA_003144235.1	Violaceomyces palustris (basidiomycetes)
993615	GCA_000231115.1	Vittaforma corneae ATCC 50505 (microsporidians)
706473	GCA_000349905.1	Volvariella volvacea V23 (basidiomycetes)
1540922	GCA_004918325.1	Wallemia hederae (basidiomycetes)
1299270	GCA_000400465.1	Wallemia ichthyophaga EXF-994 (basidiomycetes)
671144	GCA_000263375.1	Wallemia mellicola CBS 633.66 (basidiomycetes)
45790	GCA_001599155.1	Wickerhamia fluorescens (budding yeasts)
75733	GCA_003705615.1	Wickerhamiella cacticola (budding yeasts)
45788	GCA_001599275.1	Wickerhamiella domercqiae (budding yeasts)
446224	GCA_004125105.1	Wickerhamiella hasegawae (budding yeasts)
1116880	GCA_004125145.1	Wickerhamiella infanticola (budding yeasts)
75734	GCA_004125095.1	Wickerhamiella occidentalis (budding yeasts)
49331	GCA_004125235.1	Wickerhamiella pararugosa (budding yeasts)
45607	GCA_002251995.2	Wickerhamiella sorbophila (budding yeasts)
27304	GCA_001600375.1	Wickerhamiella versatilis (budding yeasts)
1337057	GCA_003707985.2	Wickerhamomyces alni (budding yeasts)
683960	GCA_001661255.1	Wickerhamomyces anomalus NRRL Y-366-8 (budding yeasts)
53647	GCA_003707945.2	Wickerhamomyces bovis (budding yeasts)
1156965	GCA_003707015.2	Wickerhamomyces canadensis (budding yeasts)
1041607	GCA_000313485.1	Wickerhamomyces ciferrii (budding yeasts)
1041608	GCA_003706765.2	Wickerhamomyces hampshirensis (budding yeasts)
2508582	GCA_003706815.2	Wickerhamomyces sp. NRRL YB-2243 (budding yeasts)
742152	GCA_000344635.1	Wolfiporia cocos MD-104 SS10 (basidiomycetes)
5421	GCA_001007165.2	Xanthophyllomyces dendrorhous (basidiomycetes)
121045	GCA_003316335.1	Xerocomus impolitus (basidiomycetes)
89491	GCA_900006255.1	Xeromyces bisporus (ascomycetes)
363999	GCA_004353285.1	Xylaria grammica (ascomycetes)
37992	GCA_004768795.1	Xylaria hypoxylon (ascomycetes)
114818	GCA_003426265.1	Xylaria longipes (ascomycetes)
77046	GCA_003426235.1	Xylaria polymorpha (dead man's fingers)
167374	GCA_005188305.1	Xylaria sp. BCC 1067 (ascomycetes)
1580213	GCA_000966885.1	Xylaria sp. JS573 (ascomycetes)
2023133	GCA_002288965.1	Xylaria sp. MSU_SB201401 (ascomycetes)
743301	GCA_002749545.1	Xylaria striata (ascomycetes)
1328760	GCA_001619985.1	Xylona heveae TC161 (ascomycetes)
1944661	GCA_002109505.1	Yamadazyma laniorum (budding yeasts)
45506	GCA_003706715.1	Yamadazyma nakazawae (budding yeasts)
45510	GCA_003706995.2	Yamadazyma philogaea (budding yeasts)
45511	GCA_003707025.1	Yamadazyma scolyti (budding yeasts)
590646	GCA_000223465.1	Yamadazyma tenuis ATCC 10573 (budding yeasts)
479092	GCA_900518985.1	Yarrowia alimentaria (budding yeasts)
1402413	GCA_900519075.1	Yarrowia bubula (budding yeasts)

1608523	GCA_900519085.1	Yarrowia deformans (budding yeasts)
479095	GCA_900519045.1	Yarrowia divulgata (budding yeasts)
197054	GCA_900519055.1	Yarrowia galli (budding yeasts)
479094	GCA_900519065.1	Yarrowia hollandica (budding yeasts)
1635098	GCA_001600195.1	Yarrowia keelungensis (budding yeasts)
284591	GCA_000002525.1	Yarrowia lipolytica CLIB122 (budding yeasts)
388557	GCA_900519015.1	Yarrowia osloensis (budding yeasts)
444778	GCA_900519005.1	Yarrowia phangngaensis (budding yeasts)
1540335	GCA_900519025.1	Yarrowia porcina (budding yeasts)
1635099	GCA_001600515.1	Yarrowia sp. JCM 30694 (budding yeasts)
1635101	GCA_001602355.1	Yarrowia sp. JCM 30695 (budding yeasts)
1635100	GCA_001600535.1	Yarrowia sp. JCM 30696 (budding yeasts)
1527289	GCA_900518995.1	Yarrowia yakushimensis (budding yeasts)
58632	GCA_003707995.1	Yueomyces sinensis (budding yeasts)
1213189	GCA_001969505.1	Zancudomyces culisetae (fungi)
1194242	GCA_002786045.1	Zasmidium angulare (ascomycetes)
1629917	GCA_002786025.1	Zasmidium citri-griseum (ascomycetes)
2364153	GCA_004114245.1	Zoopage sp. CT-All (fungi)
78907	GCA_004114325.1	Zoophagus insidians (fungi)
374461	GCA_003707935.2	Zygoascus meyerae (budding yeasts)
45509	GCA_003707925.2	Zygoascus ofunaensis (budding yeasts)
1333698	GCA_000442885.1	Zygosaccharomyces bailii CLIB 213 (budding yeasts)
4957	GCA_003707595.1	Zygosaccharomyces bisporus (budding yeasts)
166050	GCA_003705955.1	Zygosaccharomyces kombuchaensis (budding yeasts)
42258	GCA_005406105.1	Zygosaccharomyces mellis (budding yeasts)
1365886	GCA_001984395.2	Zygosaccharomyces parabailii (budding yeasts)
1365887	GCA_900408955.1	Zygosaccharomyces pseudobailii (budding yeasts)
4956	GCA_000026365.1	Zygosaccharomyces rouxii (budding yeasts)
1461763	GCA_900465325.1	Zygosaccharomyces sapae (budding yeasts)
48255	GCA_003671575.2	Zygotorulaspora florentina (budding yeasts)
42260	GCA_003671565.1	Zygotorulaspora mrakii (budding yeasts)
985145	GCA_000223765.2	Zymoseptoria ardabiliae STIR04_1.1.2 (ascomycetes)
1047168	GCA_000983655.1	Zymoseptoria brevis (ascomycetes)
985148	GCA_000223825.2	Zymoseptoria passerinii SP63 (ascomycetes)
985143	GCA_000223685.2	Zymoseptoria pseudotritici STIR04_4.3.1 (ascomycetes)
336722	GCA_000219625.1	Zymoseptoria tritici IPO323 (ascomycetes)

Viruses

taxid	Acession	Virus name
120087	GCA_000861565.1	A-2 plaque virus
438782	GCA_000872625.1	Abaca bunchy top virus
438782	GCA_003985405.1	Abaca bunchy top virus
1241371	GCA_000900375.1	Abalone herpesvirus Victoria/AUS/2009
491893	GCA_000882555.1	Abalone shriveling syndrome-associated virus
11788	GCA_000848265.1	Abelson murine leukemia virus
2025595	GCA_002270725.1	Abisko virus
665102	GCA_000889055.1	Abutilon Brazil virus
1312723	GCA_002821485.1	Abutilon golden mosaic Yucatan virus
932071	GCA_000890575.1	Abutilon mosaic Bolivia virus
1046572	GCA_000895175.1	Abutilon mosaic Brazil virus
10815	GCA_000847225.1	Abutilon mosaic virus
554168	GCA_002966335.1	Acanthamoeba castellanii mamavirus
1899318	GCA_002966345.1	Acanthamoeba castellanii mimivirus
1899318	GCA_002966355.1	Acanthamoeba castellanii mimivirus
1077221	GCA_000320725.1	Acanthamoeba polyphaga lentillevirus
212035	GCA_000888735.1	Acanthamoeba polyphaga mimivirus
212035	GCA_002966365.1	Acanthamoeba polyphaga mimivirus
212035	GCA_002966375.1	Acanthamoeba polyphaga mimivirus
212035	GCA_002966385.1	Acanthamoeba polyphaga mimivirus
212035	GCA_002966395.1	Acanthamoeba polyphaga mimivirus
212035	GCA_003815115.1	Acanthamoeba polyphaga mimivirus
212035	GCA_003815155.1	Acanthamoeba polyphaga mimivirus
212035	GCA_003815135.1	Acanthamoeba polyphaga mimivirus
1269028	GCA_000904035.1	Acanthamoeba polyphaga moumouvirus
322019	GCA_000869685.1	Acanthocystis turfacea chlorella virus 1
1278269	GCA_003093375.1	Acanthocystis turfacea Chlorella virus Can0610SP
1168547	GCA_000905055.1	Acartia tonsa copepod circovirus
645116	GCA_003146765.1	Acaryochloris phage A-HIS1
645117	GCA_003146785.1	Acaryochloris phage A-HIS2
185639	GCA_000858405.1	Acheta domestica densovirus
185639	GCA_003033195.1	Acheta domestica densovirus
1404345	GCA_000912155.1	Acheta domestica mini ambidensovirus
1291515	GCA_000908295.1	Acheta domesticus volvovirus
1261100	GCA_000925575.1	Achimota virus 1
1261101	GCA_000924735.1	Achimota virus 2
46014	GCA_000837825.1	Acholeplasma virus L2
1977403	GCA_000847085.1	Acholeplasma virus MV-L51
1589747	GCA_001504295.1	Achromobacter phage 83-24
1416009	GCA_000914775.1	Achromobacter phage JWAlpha
1416008	GCA_002604185.1	Achromobacter phage JWDelta
1589748	GCA_001551405.1	Achromobacter phage JWF
1589746	GCA_001503495.1	Achromobacter phage JWX
1610509	GCA_001504575.1	Achromobacter phage phiAxp-1
1664246	GCA_001551125.1	Achromobacter phage phiAxp-2
1664247	GCA_002211055.1	Achromobacter phage phiAxp-3
437444	GCA_000873825.1	Acidianus bottle-shaped virus

1732173	GCA_001502255.1	Acidianus bottle-shaped virus 2
1732174	GCA_001501475.1	Acidianus bottle-shaped virus 3
235266	GCA_000842625.1	Acidianus filamentous virus 1
300186	GCA_000873645.1	Acidianus filamentous virus 2
346881	GCA_000878935.1	Acidianus filamentous virus 3
346882	GCA_000871385.1	Acidianus filamentous virus 6
346883	GCA_000872245.1	Acidianus filamentous virus 7
346884	GCA_000872405.1	Acidianus filamentous virus 8
512792	GCA_000879855.1	Acidianus filamentous virus 9
309181	GCA_000871285.1	Acidianus rod-shaped virus 1
1732175	GCA_001579495.1	Acidianus rod-shaped virus 2
693629	GCA_000886935.1	Acidianus spindle-shaped virus 1
1797140	GCA_001579395.1	Acidianus tailed spindle virus
315953	GCA_000865845.1	Acidianus two-tailed virus
1898549	GCA_002614085.1	Acidianus two-tailed virus 2
1898550	GCA_002614105.1	Acidianus two-tailed virus variant 1
1229761	GCA_002602905.1	Acidithiobacillus phage AcaML1
1229761	GCA_004150025.1	Acidithiobacillus phage AcaML1
1229761	GCA_004150005.1	Acidithiobacillus phage AcaML1
2010329	GCA_002625205.1	Acidovorax phage ACP17
2301642	GCA_003575605.1	Acidovorax phage ACPWH
889876	GCA_002630805.1	Acinetobacter phage AB1
1718840	GCA_002607525.1	Acinetobacter phage Ab105-1phi
1718841	GCA_002607545.1	Acinetobacter phage Ab105-2phi
1718842	GCA_003329245.1	Acinetobacter phage Ab105-3phi
1273713	GCA_000908675.1	Acinetobacter phage AB3
2500826	GCA_004015525.1	Acinetobacter phage AbKT21phiIII
1235824	GCA_000907515.1	Acinetobacter phage Abp1
2015804	GCA_002625365.1	Acinetobacter phage AbP2
2282400	GCA_003423345.1	Acinetobacter phage ABPH49
2507835	GCA_004138775.1	Acinetobacter phage AbTJ
2500827	GCA_004015585.1	Acinetobacter phage AbTZA1
762660	GCA_000890275.1	Acinetobacter phage Ac42
760732	GCA_000887755.1	Acinetobacter phage Acj61
760939	GCA_000888755.1	Acinetobacter phage Acj9
1913571	GCA_002615385.1	Acinetobacter phage AM24
154784	GCA_000850225.1	Acinetobacter phage AP205
1187128	GCA_000894675.1	Acinetobacter phage AP22
1221835	GCA_000902615.1	Acinetobacter phage Bphi-B1251
1647373	GCA_001501195.1	Acinetobacter phage Fri1
2500136	GCA_004138755.1	Acinetobacter phage Henu6
1458669	GCA_000915715.1	Acinetobacter phage IME_AB3
1735582	GCA_001500795.2	Acinetobacter phage IME200
1243183	GCA_002602985.1	Acinetobacter phage IME-AB2
2301662	GCA_003606175.1	Acinetobacter phage KARL-1
1792222	GCA_001745775.1	Acinetobacter phage LZ35
2053691	GCA_002957355.1	Acinetobacter phage PBAB08
2053692	GCA_002957365.1	Acinetobacter phage PBAB25
1406779	GCA_000916495.1	Acinetobacter phage Petty
691318	GCA_001470235.1	Acinetobacter phage phiAB1

1698439	GCA_001745115.1	Acinetobacter phage phiAB6
1229760	GCA_001503595.1	Acinetobacter phage phiAC-1
1406780	GCA_000917335.1	Acinetobacter phage Presley
2060946	GCA_002957785.1	Acinetobacter phage SH-Ab 15497
1916127	GCA_002615865.1	Acinetobacter phage SH-Ab 15519
2202135	GCA_003369245.1	Acinetobacter phage SH-Ab 15599
2053604	GCA_002957285.1	Acinetobacter phage SWH-Ab-1
2059857	GCA_002955315.1	Acinetobacter phage SWH-Ab-3
1481186	GCA_000925015.1	Acinetobacter phage vB_AbaM_Acibel004
2315466	GCA_003613355.1	Acinetobacter phage vB_AbaM_B09_Aci01-1
2315467	GCA_003613375.1	Acinetobacter phage vB_AbaM_B09_Aci02-2
2315458	GCA_003613915.1	Acinetobacter phage vB_AbaM_B09_Aci05
2163899	GCA_003094375.1	Acinetobacter phage vB_AbaM_B9
2419625	GCA_003718955.1	Acinetobacter phage vB_AbaM_IME284
2419626	GCA_003718935.1	Acinetobacter phage vB_AbaM_IME285
2419627	GCA_003718975.1	Acinetobacter phage vB_AbaM_IME512
1837876	GCA_002609765.1	Acinetobacter phage vB_AbaM_ME3
1605379	GCA_001755565.1	Acinetobacter phage vB_AbaM_phiAbaA1
2315468	GCA_003613395.1	Acinetobacter phage vB_AbaP_46-62_Aci07
1481187	GCA_000927515.1	Acinetobacter phage vB_AbaP_Acibel007
1932886	GCA_002617985.1	Acinetobacter phage vB_AbaP_AS11
1932885	GCA_002617965.1	Acinetobacter phage vB_AbaP_AS12
2315601	GCA_003613955.1	Acinetobacter phage vB_AbaP_B09_Aci08
2016049	GCA_002627085.1	Acinetobacter phage vB_AbaP_B1
2016050	GCA_002627105.1	Acinetobacter phage vB_AbaP_B3
2678937	GCA_002627125.1	Acinetobacter phage vB_AbaP_B5
2126803	GCA_003023935.1	Acinetobacter phage vB_AbaP_D2
1735582	GCA_001500795.1	Acinetobacter phage vB_AbaP_IME200
1701807	GCA_001470095.1	Acinetobacter phage vB_AbaP_PD-6A3
1701808	GCA_001470635.1	Acinetobacter phage vB_AbaP_PD-AB9
2510492	GCA_004325255.1	Acinetobacter phage vB_AbaS_D0
1970374	GCA_900010585.1	Acinetobacter phage vB_AbaS_Loki
1852629	GCA_001743895.1	Acinetobacter phage vB_AbaS_TRS1
2269366	GCA_003369145.1	Acinetobacter phage vB_ApiM_fHyAci03
2016052	GCA_002627145.1	Acinetobacter phage vB_ApiP_P1
2016053	GCA_002627165.1	Acinetobacter phage vB_ApiP_P2
1983409	GCA_002623465.1	Acinetobacter phage WCHABP1
1965454	GCA_002620125.1	Acinetobacter phage WCHABP12
1983525	GCA_002623585.1	Acinetobacter phage WCHABP5
1628721	GCA_002605545.1	Acinetobacter phage YMC11/11/R3177
1628722	GCA_002605565.1	Acinetobacter phage YMC11/12/R1215
1628720	GCA_001501295.1	Acinetobacter phage YMC11/12/R2315
1560342	GCA_001042155.1	Acinetobacter phage YMC13/03/R2096
1505225	GCA_000926095.1	Acinetobacter phage YMC-13-01-C62
1049283	GCA_000898295.2	Acinetobacter phage ZZ1
279006	GCA_000891695.1	Acinetobacter virus 133
101764	GCA_000863345.1	Aconitum latent virus
1776763	GCA_002867245.1	Actinidia chlorotic ringspot-associated virus
2081931	GCA_004131265.1	Actinidia seed-borne latent virus
2024724	GCA_002270905.1	Actinidia virus 1

1112770	GCA_000896495.1	Actinidia virus B
1331744	GCA_001465485.1	Actinidia virus X
2099653	GCA_002997685.1	Actinomyces phage xhp1
338473	GCA_000874085.1	Actinomyces virus Av1
279303	GCA_000842685.1	Actinoplanes phage phiAsp2
92444	GCA_000856345.1	Acute bee paralysis virus
340187	GCA_000886815.1	Acyrthosiphon pisum virus
1611877	GCA_001550965.1	Adana virus
31612	GCA_001433545.1	Adelaide River virus
1590650	GCA_000930155.1	Adelie penguin polyomavirus
1930920	GCA_001957335.1	Adelphocoris suturalis virus
272636	GCA_004129875.1	Adeno-associated virus
85106	GCA_000863065.1	Adeno-associated virus - 1
10804	GCA_000838645.1	Adeno-associated virus - 2
46350	GCA_000841585.1	Adeno-associated virus - 3
57579	GCA_000836885.1	Adeno-associated virus - 4
82300	GCA_000846385.1	Adeno-associated virus - 5
202812	GCA_000845645.1	Adeno-associated virus - 7
202813	GCA_000846525.1	Adeno-associated virus - 8
1883104	GCA_004130615.1	Adonis mosaic virus
1293540	GCA_000427135.1	Adoxophyes honmai entomopoxvirus 'L'
224399	GCA_000843745.1	Adoxophyes honmai nucleopolyhedrovirus
170617	GCA_000841485.1	Adoxophyes orana granulovirus
542343	GCA_000883235.1	Adoxophyes orana nucleopolyhedrovirus
335103	GCA_000864245.1	Adult diarrheal rotavirus strain J19
1513196	GCA_000882875.1	Aedes aegypti densovirus 2
2010265	GCA_002210815.1	Aedes alboannulatus toti-like virus 1
1513199	GCA_000847125.1	Aedes albopictus densovirus 2
2230910	GCA_003260715.1	Aedes anphevirus
2010268	GCA_002210595.1	Aedes camptorhynchus negev-like virus
2010269	GCA_002288815.1	Aedes camptorhynchus reo-like virus
2010266	GCA_002211015.1	Aedes camptorhynchus toti-like virus 1
390845	GCA_000885715.1	Aedes flavivirus
341721	GCA_000866085.1	Aedes pseudoscutellaris reovirus
386586	GCA_000869125.1	Aedes taeniorhynchus iridescent virus
1962503	GCA_002867145.1	Aeonium ringspot virus
1913112	GCA_002615145.1	Aeribacillus phage AP45
2495576	GCA_003985385.1	Aerococcus phage vB_AviM_AVP
2163962	GCA_003143195.1	Aeromonas phage 13AhydR10PP
2163987	GCA_003143355.1	Aeromonas phage 14AhydR10PP
2163976	GCA_003143375.2	Aeromonas phage 25AhydR2PP
2163976	GCA_003143375.1	Aeromonas phage 25AhydR2PP
1932897	GCA_002618165.1	Aeromonas phage 3
1932898	GCA_002618185.1	Aeromonas phage 31.2
1932899	GCA_002618205.1	Aeromonas phage 32
1932900	GCA_002618225.1	Aeromonas phage 44RR2.8t.2
2163978	GCA_003143275.1	Aeromonas phage 50AhydR13PP
1932901	GCA_002618245.1	Aeromonas phage 51
1932902	GCA_002618265.1	Aeromonas phage 56
1932903	GCA_002618285.1	Aeromonas phage 59.1

2163979	GCA_003143415.1	Aeromonas phage 60AhydR15PP
2163963	GCA_003143235.1	Aeromonas phage 62AhydR11PP
1932896	GCA_002618145.1	Aeromonas phage 65.2
2163964	GCA_003143295.1	Aeromonas phage 85AhydR10PP
1198014	GCA_000907075.1	Aeromonas phage Aes012
1198013	GCA_000901975.1	Aeromonas phage Aes508
2053701	GCA_002957415.1	Aeromonas phage Ah1
1747286	GCA_002745815.1	Aeromonas phage Ahp1
1873997	GCA_002954805.1	Aeromonas phage Ahp2
2138299	GCA_003044105.1	Aeromonas phage AhSzw-1
2419736	GCA_004146885.1	Aeromonas phage AsFcp_2
2419737	GCA_004146925.1	Aeromonas phage AsFcp_4
2026082	GCA_002629085.1	Aeromonas phage AS-gz
1932904	GCA_002618305.1	Aeromonas phage Asp37
2419738	GCA_004146905.1	Aeromonas phage Assk
2419739	GCA_004146865.1	Aeromonas phage Asswx_1
2026114	GCA_002743635.1	Aeromonas phage AS-szw
2419740	GCA_004146945.1	Aeromonas phage Aswh_1
2182324	GCA_003342435.1	Aeromonas phage AsXd-1
2024208	GCA_002627665.1	Aeromonas phage AS-zj
1204516	GCA_000903495.1	Aeromonas phage CC2
2507411	GCA_002745795.1	Aeromonas phage CF7
1932905	GCA_002618325.1	Aeromonas phage L9-6
1505227	GCA_000929475.1	Aeromonas phage pAh6-C
1978922	GCA_002622325.1	Aeromonas phage phiA8-29
1748773	GCA_002607905.1	Aeromonas phage phiARM81ld
1754209	GCA_002607975.1	Aeromonas phage phiARM81mr
879628	GCA_000890235.1	Aeromonas phage phiAS4
879630	GCA_000887715.1	Aeromonas phage phiAS5
1141132	GCA_000902435.1	Aeromonas phage phiAS7
754050	GCA_002710125.1	Aeromonas phage pIS4-A
926067	GCA_000920195.1	Aeromonas phage PX29
1932906	GCA_002618345.1	Aeromonas phage Riv-10
2201378	GCA_003991605.1	Aeromonas phage SD04
1932907	GCA_002618365.1	Aeromonas phage SW69-9
1127514	GCA_000901775.1	Aeromonas phage vB_AsaM-56
2420320	GCA_003991665.1	Aeromonas phage ZPAH7
2501745	GCA_004146845.1	Aeromonas phage ZPAH7B
233894	GCA_000868205.1	Aeromonas virus 25
321023	GCA_000862645.1	Aeromonas virus 31
115987	GCA_000842425.1	Aeromonas virus 44RR2
260149	GCA_000893255.1	Aeromonas virus 65
227470	GCA_000843245.1	Aeromonas virus Aeh1
393598	GCA_000873905.1	Aeromonas virus phiO18P
1157339	GCA_002987785.1	Aeropyrum coil-shaped virus
1932713	GCA_002890195.1	Aeropyrum globular virus 1
700542	GCA_002814335.1	Aeropyrum pernix bacilliform virus 1
1032474	GCA_001440875.1	Aeropyrum pernix ovoid virus 1
1032473	GCA_001441135.1	Aeropyrum pernix spindle-shaped virus 1
2003501	GCA_000931355.1	African bat icavirus PREDICT-06105

10817	GCA_000857205.1	African cassava mosaic virus
1963256	GCA_002029515.1	African eggplant yellowing virus
1399914	GCA_000911115.1	African elephant polyomavirus 1
12480	GCA_000860565.1	African green monkey polyomavirus
40050	GCA_000856125.1	African horse sickness virus
40050	GCA_003081775.1	African horse sickness virus
40050	GCA_003081895.1	African horse sickness virus
40050	GCA_003081915.1	African horse sickness virus
40050	GCA_003081935.1	African horse sickness virus
40050	GCA_003081795.1	African horse sickness virus
40050	GCA_003081835.1	African horse sickness virus
40050	GCA_003081495.1	African horse sickness virus
40050	GCA_003082195.1	African horse sickness virus
40050	GCA_003081855.1	African horse sickness virus
40050	GCA_003081875.1	African horse sickness virus
40050	GCA_003081555.1	African horse sickness virus
40050	GCA_003081575.1	African horse sickness virus
40050	GCA_003081595.1	African horse sickness virus
40050	GCA_003081655.1	African horse sickness virus
40050	GCA_003081615.1	African horse sickness virus
40050	GCA_003081975.1	African horse sickness virus
40050	GCA_003081635.1	African horse sickness virus
40050	GCA_003081995.1	African horse sickness virus
40050	GCA_003082015.1	African horse sickness virus
40050	GCA_003081675.1	African horse sickness virus
40050	GCA_003081535.1	African horse sickness virus
40050	GCA_003082035.1	African horse sickness virus
40050	GCA_003081695.1	African horse sickness virus
40050	GCA_003081715.1	African horse sickness virus
40050	GCA_003081815.1	African horse sickness virus
40050	GCA_003081755.1	African horse sickness virus
40050	GCA_003085655.1	African horse sickness virus
40050	GCA_003085715.1	African horse sickness virus
40050	GCA_003084015.1	African horse sickness virus
40050	GCA_003085675.1	African horse sickness virus
40050	GCA_003085695.1	African horse sickness virus
33714	GCA_003082115.1	African horse sickness virus 1
33714	GCA_003083195.1	African horse sickness virus 1
33714	GCA_003083155.1	African horse sickness virus 1
33714	GCA_003082235.1	African horse sickness virus 1
33714	GCA_003082475.1	African horse sickness virus 1
33714	GCA_003082135.1	African horse sickness virus 1
33714	GCA_003083215.1	African horse sickness virus 1
33714	GCA_003082155.1	African horse sickness virus 1
33714	GCA_003082455.1	African horse sickness virus 1
33714	GCA_003082535.1	African horse sickness virus 1
33714	GCA_003081515.1	African horse sickness virus 1
33714	GCA_003082255.1	African horse sickness virus 1
33714	GCA_003082915.1	African horse sickness virus 1
33714	GCA_003082275.1	African horse sickness virus 1

33714	GCA_003082295.1	African horse sickness virus 1
33714	GCA_003081955.1	African horse sickness virus 1
33714	GCA_003082315.1	African horse sickness virus 1
33714	GCA_003083095.1	African horse sickness virus 1
33714	GCA_003082495.1	African horse sickness virus 1
33714	GCA_003082515.1	African horse sickness virus 1
33714	GCA_003082215.1	African horse sickness virus 1
33714	GCA_003082555.1	African horse sickness virus 1
33714	GCA_003082575.1	African horse sickness virus 1
33714	GCA_003082595.1	African horse sickness virus 1
33714	GCA_003082635.1	African horse sickness virus 1
33714	GCA_003082355.1	African horse sickness virus 1
33714	GCA_003082375.1	African horse sickness virus 1
33714	GCA_003082655.1	African horse sickness virus 1
33714	GCA_003082675.1	African horse sickness virus 1
33714	GCA_003083175.1	African horse sickness virus 1
33714	GCA_003082695.1	African horse sickness virus 1
33714	GCA_003082715.1	African horse sickness virus 1
33714	GCA_003082755.1	African horse sickness virus 1
33714	GCA_003082735.1	African horse sickness virus 1
33714	GCA_003082775.1	African horse sickness virus 1
33714	GCA_003082055.1	African horse sickness virus 1
33714	GCA_003082795.1	African horse sickness virus 1
33714	GCA_003082815.1	African horse sickness virus 1
33714	GCA_003082855.1	African horse sickness virus 1
33714	GCA_003082415.1	African horse sickness virus 1
33714	GCA_003082895.1	African horse sickness virus 1
33714	GCA_003082075.1	African horse sickness virus 1
33714	GCA_003081735.1	African horse sickness virus 1
33714	GCA_003082935.1	African horse sickness virus 1
33714	GCA_003082955.1	African horse sickness virus 1
33714	GCA_003082975.1	African horse sickness virus 1
33714	GCA_003082995.1	African horse sickness virus 1
33714	GCA_003082435.1	African horse sickness virus 1
33714	GCA_003083015.1	African horse sickness virus 1
33714	GCA_003082095.1	African horse sickness virus 1
33714	GCA_003083035.1	African horse sickness virus 1
33714	GCA_003082335.1	African horse sickness virus 1
33714	GCA_003083075.1	African horse sickness virus 1
33714	GCA_003082875.1	African horse sickness virus 1
33714	GCA_003083115.1	African horse sickness virus 1
33714	GCA_003083135.1	African horse sickness virus 1
33714	GCA_003083715.1	African horse sickness virus 1
33714	GCA_003085295.1	African horse sickness virus 1
33714	GCA_003084635.1	African horse sickness virus 1
33714	GCA_003083995.1	African horse sickness virus 1
33714	GCA_003084195.1	African horse sickness virus 1
33714	GCA_003084415.1	African horse sickness virus 1
33714	GCA_003084855.1	African horse sickness virus 1
33714	GCA_003085075.1	African horse sickness virus 1

33714	GCA_003085515.1	African horse sickness virus 1
48295	GCA_003083235.1	African horse sickness virus 2
48295	GCA_003083255.1	African horse sickness virus 2
48295	GCA_003083275.1	African horse sickness virus 2
48295	GCA_003083055.1	African horse sickness virus 2
48295	GCA_003082175.1	African horse sickness virus 2
48295	GCA_003083295.1	African horse sickness virus 2
48295	GCA_003082615.1	African horse sickness virus 2
48295	GCA_003082395.1	African horse sickness virus 2
48295	GCA_003082835.1	African horse sickness virus 2
48295	GCA_003083495.1	African horse sickness virus 2
48295	GCA_003083735.1	African horse sickness virus 2
117204	GCA_003083315.1	African horse sickness virus 3
117204	GCA_003083335.1	African horse sickness virus 3
117204	GCA_003083355.1	African horse sickness virus 3
117204	GCA_003083955.1	African horse sickness virus 3
117204	GCA_003084055.1	African horse sickness virus 3
117204	GCA_003084095.1	African horse sickness virus 3
117204	GCA_003084175.1	African horse sickness virus 3
117204	GCA_003084035.1	African horse sickness virus 3
117204	GCA_003084075.1	African horse sickness virus 3
117204	GCA_003084115.1	African horse sickness virus 3
117204	GCA_003084135.1	African horse sickness virus 3
117204	GCA_003084155.1	African horse sickness virus 3
36421	GCA_003083375.1	African horse sickness virus 4
36421	GCA_003083395.1	African horse sickness virus 4
36421	GCA_003083415.1	African horse sickness virus 4
36421	GCA_003083435.1	African horse sickness virus 4
36421	GCA_003083455.1	African horse sickness virus 4
36421	GCA_003083475.1	African horse sickness virus 4
36421	GCA_003083515.1	African horse sickness virus 4
36421	GCA_003083535.1	African horse sickness virus 4
36421	GCA_003084295.1	African horse sickness virus 4
36421	GCA_003084215.1	African horse sickness virus 4
36421	GCA_003084235.1	African horse sickness virus 4
36421	GCA_003084255.1	African horse sickness virus 4
36421	GCA_003084275.1	African horse sickness virus 4
36421	GCA_003084315.1	African horse sickness virus 4
36421	GCA_003084335.1	African horse sickness virus 4
36421	GCA_003084355.1	African horse sickness virus 4
36421	GCA_003084375.1	African horse sickness virus 4
36421	GCA_003084395.1	African horse sickness virus 4
36421	GCA_003084435.1	African horse sickness virus 4
36421	GCA_003084455.1	African horse sickness virus 4
36421	GCA_003084475.1	African horse sickness virus 4
86059	GCA_003083555.1	African horse sickness virus 5
86059	GCA_003084575.1	African horse sickness virus 5
86059	GCA_003083575.1	African horse sickness virus 5
86059	GCA_003083595.1	African horse sickness virus 5
86059	GCA_003084595.1	African horse sickness virus 5

86059	GCA_003084655.1	African horse sickness virus 5
86059	GCA_003084675.1	African horse sickness virus 5
86059	GCA_003084515.1	African horse sickness virus 5
86059	GCA_003084535.1	African horse sickness virus 5
86059	GCA_003084555.1	African horse sickness virus 5
86059	GCA_003084695.1	African horse sickness virus 5
86059	GCA_003084615.1	African horse sickness virus 5
86059	GCA_003084495.1	African horse sickness virus 5
86060	GCA_003084915.1	African horse sickness virus 6
86060	GCA_003083615.1	African horse sickness virus 6
86060	GCA_003083635.1	African horse sickness virus 6
86060	GCA_003083655.1	African horse sickness virus 6
86060	GCA_003083675.1	African horse sickness virus 6
86060	GCA_003084955.1	African horse sickness virus 6
86060	GCA_003084975.1	African horse sickness virus 6
86060	GCA_003084715.1	African horse sickness virus 6
86060	GCA_003084735.1	African horse sickness virus 6
86060	GCA_003084755.1	African horse sickness virus 6
86060	GCA_003084775.1	African horse sickness virus 6
86060	GCA_003084795.1	African horse sickness virus 6
86060	GCA_003084875.1	African horse sickness virus 6
86060	GCA_003084895.1	African horse sickness virus 6
86060	GCA_003084935.1	African horse sickness virus 6
86060	GCA_003084815.1	African horse sickness virus 6
86060	GCA_003084835.1	African horse sickness virus 6
86061	GCA_003083695.1	African horse sickness virus 7
86061	GCA_003083755.1	African horse sickness virus 7
86061	GCA_003083775.1	African horse sickness virus 7
86061	GCA_003083795.1	African horse sickness virus 7
86061	GCA_003085015.1	African horse sickness virus 7
86061	GCA_003085035.1	African horse sickness virus 7
86061	GCA_003085115.1	African horse sickness virus 7
86061	GCA_003085135.1	African horse sickness virus 7
86061	GCA_003084995.1	African horse sickness virus 7
86061	GCA_003085055.1	African horse sickness virus 7
86061	GCA_003085095.1	African horse sickness virus 7
86062	GCA_003085195.1	African horse sickness virus 8
86062	GCA_003085235.1	African horse sickness virus 8
86062	GCA_003085315.1	African horse sickness virus 8
86062	GCA_003085335.1	African horse sickness virus 8
86062	GCA_003085215.1	African horse sickness virus 8
86062	GCA_003085375.1	African horse sickness virus 8
86062	GCA_003085255.1	African horse sickness virus 8
86062	GCA_003085275.1	African horse sickness virus 8
86062	GCA_003083815.1	African horse sickness virus 8
86062	GCA_003083835.1	African horse sickness virus 8
86062	GCA_003083855.1	African horse sickness virus 8
86062	GCA_003083875.1	African horse sickness virus 8
86062	GCA_003083895.1	African horse sickness virus 8
86062	GCA_003083915.1	African horse sickness virus 8

86062	GCA_003085355.1	African horse sickness virus 8
86062	GCA_003085155.1	African horse sickness virus 8
86062	GCA_003085175.1	African horse sickness virus 8
10897	GCA_003085395.1	African horse sickness virus 9
10897	GCA_003085415.1	African horse sickness virus 9
10897	GCA_003085435.1	African horse sickness virus 9
10897	GCA_003085535.1	African horse sickness virus 9
10897	GCA_003085555.1	African horse sickness virus 9
10897	GCA_003085575.1	African horse sickness virus 9
10897	GCA_003085595.1	African horse sickness virus 9
10897	GCA_003085615.1	African horse sickness virus 9
10897	GCA_003085635.1	African horse sickness virus 9
10897	GCA_003083935.1	African horse sickness virus 9
10897	GCA_003083975.1	African horse sickness virus 9
10897	GCA_003085455.1	African horse sickness virus 9
10897	GCA_003085475.1	African horse sickness virus 9
10897	GCA_003085495.1	African horse sickness virus 9
185218	GCA_000882155.1	African oil palm ringspot virus
1965064	GCA_000931135.1	African pouched rat arterivirus
10497	GCA_000858485.1	African swine fever virus
10497	GCA_003032865.1	African swine fever virus
10497	GCA_003032935.1	African swine fever virus
10497	GCA_003032905.1	African swine fever virus
10497	GCA_003032975.1	African swine fever virus
10497	GCA_003032955.1	African swine fever virus
10497	GCA_003032945.1	African swine fever virus
10497	GCA_003032915.1	African swine fever virus
10497	GCA_003032965.1	African swine fever virus
10497	GCA_003032885.1	African swine fever virus
10497	GCA_003032985.1	African swine fever virus
10497	GCA_003032875.1	African swine fever virus
10497	GCA_003032995.1	African swine fever virus
10497	GCA_003033005.1	African swine fever virus
10497	GCA_003032925.1	African swine fever virus
10497	GCA_003032895.1	African swine fever virus
10497	GCA_003815375.1	African swine fever virus
10497	GCA_003815395.1	African swine fever virus
10497	GCA_003815415.1	African swine fever virus
10497	GCA_003815435.1	African swine fever virus
10497	GCA_003815455.1	African swine fever virus
10497	GCA_003815195.1	African swine fever virus
10497	GCA_003815215.1	African swine fever virus
10497	GCA_003815235.1	African swine fever virus
10497	GCA_003815255.1	African swine fever virus
10497	GCA_003815275.1	African swine fever virus
10497	GCA_003815175.1	African swine fever virus
10497	GCA_003815295.1	African swine fever virus
10497	GCA_003815315.1	African swine fever virus
10497	GCA_003815335.1	African swine fever virus
10497	GCA_003815355.1	African swine fever virus

10497	GCA_004135325.1	African swine fever virus
10497	GCA_004338215.1	African swine fever virus
10497	GCA_004338235.1	African swine fever virus
10497	GCA_900380425.1	African swine fever virus
443876	GCA_003047675.1	African swine fever virus Benin 97/1
686262	GCA_003047715.1	African swine fever virus E75
874269	GCA_003047755.1	African swine fever virus Georgia 2007/1
443878	GCA_003047695.1	African swine fever virus OURT 88/3
387423	GCA_002184195.1	Agave tequilana leaf virus
944995	GCA_000921555.1	Ageratum conyzoides associated symptomless alphasatelli
1705092	GCA_001429995.1	Ageratum conyzoides symptomless alphasatellite
1260769	GCA_000902015.1	Ageratum enation alphasatellite
188333	GCA_000859025.1	Ageratum enation virus
188333	GCA_002986325.1	Ageratum enation virus
1322247	GCA_002821505.1	Ageratum enation virus-Papaver somniferum
1386090	GCA_000912595.1	Ageratum latent virus
1175505	GCA_000904615.1	Ageratum leaf curl betasatellite
912035	GCA_000888815.1	Ageratum leaf curl Buea betasatellite
743035	GCA_000887815.1	Ageratum leaf curl Cameroon alphasatellite
635076	GCA_000884735.1	Ageratum leaf curl Cameroon betasatellite
635076	GCA_002987805.1	Ageratum leaf curl Cameroon betasatellite
912033	GCA_000888715.1	Ageratum leaf curl Cameroon virus
1048735	GCA_002987005.1	Ageratum leaf curl Cameroon virus [CM:AGFG24:2009]
298650	GCA_000844985.1	Ageratum leaf curl virus - [G52]
169687	GCA_000846445.1	Ageratum yellow leaf curl betasatellite
187850	GCA_000842005.1	Ageratum yellow vein alphasatellite
187850	GCA_001974535.1	Ageratum yellow vein alphasatellite
185750	GCA_000844745.1	Ageratum yellow vein betasatellite
1454227	GCA_000915415.1	Ageratum yellow vein China alphasatellite
329288	GCA_000866525.1	Ageratum yellow vein China betasatellite
222078	GCA_000845825.1	Ageratum yellow vein China virus - [Hn2]
1329380	GCA_000907355.1	Ageratum Yellow vein China virus - OX1
437060	GCA_000880075.1	Ageratum yellow vein Hualian virus-[Taiwan:Hsinchu:tom:2
437061	GCA_003180935.1	Ageratum yellow vein Hualian virus-[Taiwan:Hualian4:2000
1407058	GCA_000903535.1	Ageratum yellow vein India alphasatellite
2010316	GCA_002987825.1	Ageratum yellow vein India betasatellite
1468596	GCA_001962155.1	Ageratum yellow vein Pakistan alphasatellite
2010317	GCA_002987855.1	Ageratum yellow vein Sri Lanka betasatellite
222079	GCA_000838085.1	Ageratum yellow vein Sri Lanka virus
44560	GCA_000838225.1	Ageratum yellow vein virus
44560	GCA_000840465.1	Ageratum yellow vein virus
44560	GCA_000857345.1	Ageratum yellow vein virus
44560	GCA_001008515.1	Ageratum yellow vein virus
44560	GCA_002821605.1	Ageratum yellow vein virus
44560	GCA_002821645.1	Ageratum yellow vein virus
44560	GCA_002821525.1	Ageratum yellow vein virus
44560	GCA_002821565.1	Ageratum yellow vein virus
44560	GCA_002821585.1	Ageratum yellow vein virus
263002	GCA_002821545.1	Ageratum yellow vein virus-[Tomato]
446318	GCA_002821625.1	Ageratum yellow vein virus-Ishigaki

683735	GCA_001743535.1	Aggregatibacter phage S1249
1161931	GCA_000901835.1	Agrobacterium phage 7-7-1
2024261	GCA_002628185.1	Agrobacterium phage Atu_ph02
2024262	GCA_002628205.1	Agrobacterium phage Atu_ph03
2024263	GCA_002628225.1	Agrobacterium phage Atu_ph04
2024264	GCA_002628245.1	Agrobacterium phage Atu_ph07
2024265	GCA_002628265.1	Agrobacterium phage Atu_ph08
41763	GCA_000856705.1	Agropyron mosaic virus
208013	GCA_000883155.1	Agrotis ipsilon multiple nucleopolyhedrovirus
10464	GCA_000844105.1	Agrotis segetum granulovirus
10464	GCA_002819185.1	Agrotis segetum granulovirus
1962501	GCA_000868985.1	Agrotis segetum nucleopolyhedrovirus A
1580580	GCA_000928115.1	Agrotis segetum nucleopolyhedrovirus B
1006583	GCA_000890995.1	Aguacate virus
1313215	GCA_000861885.1	Aichi virus 1
1313215	GCA_002817065.1	Aichi virus 1
194965	GCA_000853805.1	Aichivirus B
2016454	GCA_002219585.1	Ailuropoda melanoleuca papillomavirus 1
2016455	GCA_002219765.1	Ailuropoda melanoleuca papillomavirus 2
2016453	GCA_002219945.1	Ailuropoda melanoleuca papillomavirus 4
2016453	GCA_003180135.1	Ailuropoda melanoleuca papillomavirus 4
2016448	GCA_002219405.1	Aimelvirus 2
11582	GCA_000898555.1	Aino virus
1692242	GCA_001274085.1	Aiptasia sp. sea anemone associated circular virus
70566	GCA_000871205.1	Akabane virus
2163422	GCA_004133585.1	Akodon montensis polyomavirus
1552846	GCA_002831185.1	Alajuela virus
35252	GCA_000838825.1	Alcelaphine gammaherpesvirus 1 (wildebeest herpesvirus)
138184	GCA_000923715.1	Alcelaphine gammaherpesvirus 2
2219143	GCA_003848145.1	Alces alces faeces associated circular virus MP65
2219111	GCA_003847845.1	Alces alces faeces associated genomovirus MP111
2219112	GCA_003847825.1	Alces alces faeces associated genomovirus MP146
2219113	GCA_003847805.1	Alces alces faeces associated genomovirus MP157
2219114	GCA_003847905.1	Alces alces faeces associated genomovirus MP43
2219115	GCA_003847885.1	Alces alces faeces associated genomovirus MP68
2219116	GCA_003847865.1	Alces alces faeces associated genomovirus MP84
2219133	GCA_003848285.1	Alces alces faeces associated microvirus MP10 5560
2219134	GCA_003848265.1	Alces alces faeces associated microvirus MP11 5517
2219135	GCA_003848245.1	Alces alces faeces associated microvirus MP12 5423
2219136	GCA_003848225.1	Alces alces faeces associated microvirus MP15 5067
2219137	GCA_003848205.1	Alces alces faeces associated microvirus MP18 4940
2219138	GCA_003848185.1	Alces alces faeces associated microvirus MP21 4718
2219139	GCA_003848305.1	Alces alces faeces associated microvirus MP3 6497
2219142	GCA_003963675.1	Alces alces faeces associated smacovirus MP78
28314	GCA_000838725.1	Aleutian mink disease virus
998864	GCA_001432075.1	Alfalfa dwarf virus
998864	GCA_001432075.2	Alfalfa dwarf virus
1770265	GCA_001634515.1	Alfalfa enamovirus 1
165250	GCA_000954395.1	Alfalfa latent virus
1306546	GCA_001271015.1	Alfalfa leaf curl virus

12321	GCA_000847525.1	Alfalfa mosaic virus
2170538	GCA_004132825.1	Alfalfa virus F
1985968	GCA_002158655.1	Alfalfa virus S
515575	GCA_000879995.1	Algerian watermelon mosaic virus
172148	GCA_000863365.1	Alkhumra hemorrhagic fever virus
452758	GCA_000874545.1	Allamanda leaf curl virus
1317107	GCA_000919815.2	Allamanda leaf mottle distortion virus
1317107	GCA_000919815.1	Allamanda leaf mottle distortion virus
2058778	GCA_002890315.1	Allium cepa amalgavirus 1
2058779	GCA_002889815.1	Allium cepa amalgavirus 2
317027	GCA_000882735.1	Allium virus X
144752	GCA_000872565.1	Allpahuayo mammarenavirus
318843	GCA_000927215.1	Almpiwar virus
1784959	GCA_003179735.1	Alouatta guariba papillomavirus 1
10570	GCA_000836865.1	Alphapapillomavirus 12
333767	GCA_004289735.1	Alphapapillomavirus 3
279383	GCA_000864205.1	Alphaproteobacteria virus phiJl001
97469	GCA_002889055.1	Alphavirus M1
316983	GCA_000866665.1	Alstroemeria virus X
2212644	GCA_004117275.1	Alstroemeria yellow spot virus
85454	GCA_000866985.1	Alternanthera mosaic virus
447606	GCA_000871765.1	Alternanthera yellow vein betasatellite
337826	GCA_000866585.1	Alternanthera yellow vein virus
337826	GCA_002821665.1	Alternanthera yellow vein virus
337826	GCA_002821685.1	Alternanthera yellow vein virus
2066695	GCA_004117195.1	Alternaria alternata chrysovirus 1
1935440	GCA_003729215.1	Alternaria alternata fusarivirus 1
483537	GCA_000874585.1	Alternaria alternata virus 1
1826822	GCA_001706985.1	Alternaria arborescens mitovirus 1
1728964	GCA_004130595.1	Alternaria arborescens victorivirus 1
1580606	GCA_000928255.1	Alternaria brassicicola betaendornavirus 1
1766767	GCA_001550805.1	Alternaria brassicicola fusarivirus 1
1532031	GCA_000924055.1	Alternaria longipes dsRNA virus 1
2094572	GCA_002990045.1	Alteromonadaceae phage B23
2249314	GCA_003307415.1	Alteromonas phage JH01
2500137	GCA_004015395.1	Alteromonas phage P24
1913113	GCA_002615165.1	Alteromonas phage PB15
1300004	GCA_000907735.1	Alteromonas phage vB_AmaP_AD45-P1
1300005	GCA_002755495.1	Alteromonas phage vB_AmaP_AD45-P2
1300006	GCA_002755515.1	Alteromonas phage vB_AmaP_AD45-P3
1300007	GCA_002755535.1	Alteromonas phage vB_AmaP_AD45-P4
2492447	GCA_004006835.1	Alteromonas phage ZP6
2016032	GCA_002627065.1	Alteromonas virus vB_AspP-H4/4
284687	GCA_000874385.1	Amasya cherry disease associated chrysovirus
284687	GCA_002986285.1	Amasya cherry disease associated chrysovirus
284689	GCA_000854105.1	Amasya cherry disease-associated mycovirus
1195163	GCA_000898455.1	Amazon lily mild mottle virus
1926500	GCA_002008455.1	Ambe virus
217733	GCA_000841005.1	Ambystoma tigrinum stebbensi virus
1908805	GCA_002374935.1	Amdoparvovirus sp.

1344113	GCA_000912275.1	American bat vesiculovirus TFFN-2013
465447	GCA_000879275.1	American grass carp reovirus
1177630	GCA_000898055.1	American hop latent virus
134632	GCA_000850385.1	American plum line pattern virus
1941435	GCA_002004335.1	Amphibola crenata associated bacilladnavirus 1
1941436	GCA_002004015.1	Amphibola crenata associated bacilladnavirus 2
1661395	GCA_001029005.1	Ampivirus A1
28321	GCA_000837185.1	Amsacta moorei entomopoxvirus
1357732	GCA_000923615.1	Anabaena phage A-4L
312275	GCA_000880855.1	Anagyris vein yellowing virus
104388	GCA_000885795.1	Anatid alphaherpesvirus 1
1542745	GCA_000922615.1	Ancient caribou feces associated virus
73819	GCA_000906715.1	Andean potato latent virus
1296569	GCA_000905815.1	Andean potato mild mosaic virus
1980456	GCA_000850405.1	Andes orthohantavirus
2094259	GCA_004132185.1	Andrena haemorrhoa nege-like virus
1476485	GCA_000920515.2	Andrographis yellow vein leaf curl betasatellite
1738045	GCA_001441075.1	Andrographis yellow vein leaf curl virus
2055263	GCA_003147525.1	Anelloviridae sp.
2055263	GCA_003390055.1	Anelloviridae sp.
2055263	GCA_003390095.1	Anelloviridae sp.
2055263	GCA_003390075.1	Anelloviridae sp.
2055263	GCA_004286575.1	Anelloviridae sp.
2055263	GCA_004290675.1	Anelloviridae sp.
2055263	GCA_004286595.1	Anelloviridae sp.
2055263	GCA_004286615.1	Anelloviridae sp.
2055263	GCA_004286635.1	Anelloviridae sp.
2055263	GCA_004290735.1	Anelloviridae sp.
2055263	GCA_004286655.1	Anelloviridae sp.
2055263	GCA_004290755.1	Anelloviridae sp.
2055263	GCA_004286675.1	Anelloviridae sp.
2055263	GCA_004286695.1	Anelloviridae sp.
2055263	GCA_004286715.1	Anelloviridae sp.
2055263	GCA_004286735.1	Anelloviridae sp.
2055263	GCA_004286755.1	Anelloviridae sp.
2055263	GCA_004290855.1	Anelloviridae sp.
2055263	GCA_004287415.1	Anelloviridae sp.
2055263	GCA_004286775.1	Anelloviridae sp.
2055263	GCA_004290875.1	Anelloviridae sp.
2055263	GCA_004284895.1	Anelloviridae sp.
2055263	GCA_004286795.1	Anelloviridae sp.
2055263	GCA_004290895.1	Anelloviridae sp.
2055263	GCA_004284915.1	Anelloviridae sp.
2055263	GCA_004286815.1	Anelloviridae sp.
2055263	GCA_004284935.1	Anelloviridae sp.
2055263	GCA_004286835.1	Anelloviridae sp.
2055263	GCA_004284955.1	Anelloviridae sp.
2055263	GCA_004286855.1	Anelloviridae sp.
2055263	GCA_004284975.1	Anelloviridae sp.
2055263	GCA_004286875.1	Anelloviridae sp.

2055263	GCA_004290975.1	Anelloviridae sp.
2055263	GCA_004286895.1	Anelloviridae sp.
2055263	GCA_004290995.1	Anelloviridae sp.
2055263	GCA_004284995.1	Anelloviridae sp.
2055263	GCA_004286915.1	Anelloviridae sp.
2055263	GCA_004285015.1	Anelloviridae sp.
2055263	GCA_004285035.1	Anelloviridae sp.
2055263	GCA_004287315.1	Anelloviridae sp.
2055263	GCA_004285055.1	Anelloviridae sp.
2055263	GCA_004285075.1	Anelloviridae sp.
2055263	GCA_004285095.1	Anelloviridae sp.
2055263	GCA_004285115.1	Anelloviridae sp.
2055263	GCA_004285135.1	Anelloviridae sp.
2055263	GCA_004285155.1	Anelloviridae sp.
2055263	GCA_004285175.1	Anelloviridae sp.
2055263	GCA_004287095.1	Anelloviridae sp.
2055263	GCA_004285195.1	Anelloviridae sp.
2055263	GCA_004287115.1	Anelloviridae sp.
2055263	GCA_004285215.1	Anelloviridae sp.
2055263	GCA_004287135.1	Anelloviridae sp.
2055263	GCA_004285235.1	Anelloviridae sp.
2055263	GCA_004287155.1	Anelloviridae sp.
2055263	GCA_004285255.1	Anelloviridae sp.
2055263	GCA_004287175.1	Anelloviridae sp.
2055263	GCA_004285275.1	Anelloviridae sp.
2055263	GCA_004287195.1	Anelloviridae sp.
2055263	GCA_004285295.1	Anelloviridae sp.
2055263	GCA_004287215.1	Anelloviridae sp.
2055263	GCA_004287235.1	Anelloviridae sp.
2055263	GCA_004287255.1	Anelloviridae sp.
2055263	GCA_004285355.1	Anelloviridae sp.
2055263	GCA_004287275.1	Anelloviridae sp.
2055263	GCA_004285375.1	Anelloviridae sp.
2055263	GCA_004287295.1	Anelloviridae sp.
2055263	GCA_004285395.1	Anelloviridae sp.
2055263	GCA_004287335.1	Anelloviridae sp.
2055263	GCA_004285415.1	Anelloviridae sp.
2055263	GCA_004287355.1	Anelloviridae sp.
2055263	GCA_004285435.1	Anelloviridae sp.
2055263	GCA_004287375.1	Anelloviridae sp.
2055263	GCA_004285455.1	Anelloviridae sp.
2055263	GCA_004285475.1	Anelloviridae sp.
2055263	GCA_004285495.1	Anelloviridae sp.
2055263	GCA_004287395.1	Anelloviridae sp.
2055263	GCA_004285515.1	Anelloviridae sp.
2055263	GCA_004285535.1	Anelloviridae sp.
2055263	GCA_004285555.1	Anelloviridae sp.
2055263	GCA_004285575.1	Anelloviridae sp.
2055263	GCA_004285615.1	Anelloviridae sp.
2055263	GCA_004285635.1	Anelloviridae sp.

2055263	GCA_004285655.1	Anelloviridae sp.
2055263	GCA_004287435.1	Anelloviridae sp.
2055263	GCA_004285335.1	Anelloviridae sp.
2055263	GCA_004285675.1	Anelloviridae sp.
2055263	GCA_004285695.1	Anelloviridae sp.
2055263	GCA_004285715.1	Anelloviridae sp.
2055263	GCA_004289775.1	Anelloviridae sp.
2055263	GCA_004285735.1	Anelloviridae sp.
2055263	GCA_004285755.1	Anelloviridae sp.
2055263	GCA_004287455.1	Anelloviridae sp.
2055263	GCA_004285775.1	Anelloviridae sp.
2055263	GCA_004289855.1	Anelloviridae sp.
2055263	GCA_004289875.1	Anelloviridae sp.
2055263	GCA_004285795.1	Anelloviridae sp.
2055263	GCA_004289895.1	Anelloviridae sp.
2055263	GCA_004285815.1	Anelloviridae sp.
2055263	GCA_004285835.1	Anelloviridae sp.
2055263	GCA_004285855.1	Anelloviridae sp.
2055263	GCA_004285875.1	Anelloviridae sp.
2055263	GCA_004289975.1	Anelloviridae sp.
2055263	GCA_004285935.1	Anelloviridae sp.
2055263	GCA_004285955.1	Anelloviridae sp.
2055263	GCA_004285975.1	Anelloviridae sp.
2055263	GCA_004285995.1	Anelloviridae sp.
2055263	GCA_004286015.1	Anelloviridae sp.
2055263	GCA_004286035.1	Anelloviridae sp.
2055263	GCA_004286055.1	Anelloviridae sp.
2055263	GCA_004286075.1	Anelloviridae sp.
2055263	GCA_004290175.1	Anelloviridae sp.
2055263	GCA_004286095.1	Anelloviridae sp.
2055263	GCA_004286115.1	Anelloviridae sp.
2055263	GCA_004286135.1	Anelloviridae sp.
2055263	GCA_004290235.1	Anelloviridae sp.
2055263	GCA_004286155.1	Anelloviridae sp.
2055263	GCA_004290255.1	Anelloviridae sp.
2055263	GCA_004286175.1	Anelloviridae sp.
2055263	GCA_004286195.1	Anelloviridae sp.
2055263	GCA_004286215.1	Anelloviridae sp.
2055263	GCA_004290315.1	Anelloviridae sp.
2055263	GCA_004286235.1	Anelloviridae sp.
2055263	GCA_004290335.1	Anelloviridae sp.
2055263	GCA_004286255.1	Anelloviridae sp.
2055263	GCA_004286275.1	Anelloviridae sp.
2055263	GCA_004286295.1	Anelloviridae sp.
2055263	GCA_004290395.1	Anelloviridae sp.
2055263	GCA_004286315.1	Anelloviridae sp.
2055263	GCA_004290415.1	Anelloviridae sp.
2055263	GCA_004286335.1	Anelloviridae sp.
2055263	GCA_004290435.1	Anelloviridae sp.
2055263	GCA_004286355.1	Anelloviridae sp.

2055263	GCA_004290455.1	Anelloviridae sp.
2055263	GCA_004286375.1	Anelloviridae sp.
2055263	GCA_004290475.1	Anelloviridae sp.
2055263	GCA_004285595.1	Anelloviridae sp.
2055263	GCA_004286395.1	Anelloviridae sp.
2055263	GCA_004285315.1	Anelloviridae sp.
2055263	GCA_004290495.1	Anelloviridae sp.
2055263	GCA_004286415.1	Anelloviridae sp.
2055263	GCA_004286435.1	Anelloviridae sp.
2055263	GCA_004286455.1	Anelloviridae sp.
2055263	GCA_004290555.1	Anelloviridae sp.
2055263	GCA_004286475.1	Anelloviridae sp.
2055263	GCA_004286495.1	Anelloviridae sp.
2055263	GCA_004286515.1	Anelloviridae sp.
2055263	GCA_004290615.1	Anelloviridae sp.
2055263	GCA_004286535.1	Anelloviridae sp.
2055263	GCA_004286555.1	Anelloviridae sp.
352882	GCA_000865425.2	Angelonia flower break virus
352882	GCA_000865425.1	Angelonia flower break virus
1420600	GCA_000915975.1	Anguilla anguilla circovirus
1420600	GCA_004030235.1	Anguilla anguilla circovirus
1420600	GCA_004032695.1	Anguilla anguilla circovirus
1420600	GCA_004032715.1	Anguilla anguilla circovirus
150286	GCA_000886195.1	Anguillid herpesvirus 1
904722	GCA_002008495.1	Anhanga virus
1424613	GCA_002145785.1	Anjozorobe hantavirus
1605378	GCA_003004805.1	ANMV-1 virus
62099	GCA_000916855.1	Anomala cuprea entomopoxvirus
1769780	GCA_001646035.1	Anopheles C virus
1903340	GCA_001754965.1	Anopheles flavivirus variant 1
487311	GCA_000880635.1	Anopheles gambiae densovirus
1465751	GCA_000918955.1	Anopheles minimus irodovirus
1398939	GCA_000916595.1	Anopheline-associated C virus
2006072	GCA_003032685.1	Antarctic penguin virus A
2006073	GCA_003032695.1	Antarctic penguin virus B
2006074	GCA_003032705.1	Antarctic penguin virus C
1648481	GCA_001654285.1	Antarctic picorna-like virus 1
1648482	GCA_001654385.1	Antarctic picorna-like virus 2
1648483	GCA_001654185.1	Antarctic picorna-like virus 3
1648484	GCA_001654105.1	Antarctic picorna-like virus 4
1460071	GCA_000916935.1	Antheraea pernyi iflavirus
161494	GCA_000867205.1	Antheraea pernyi nucleopolyhedrovirus
2065797	GCA_004128595.1	Anthoxanthum odoratum amalgavirus 1
268591	GCA_000868545.2	Anticarsia gemmatalis multiple nucleopolyhedrovirus
268591	GCA_001876855.1	Anticarsia gemmatalis multiple nucleopolyhedrovirus
1872708	GCA_002219845.1	Antonospora locustae virus 1
50290	GCA_000896555.1	Aotine betaherpesvirus 1
2047766	GCA_002956195.1	Aphanizomenon phage vB_AphaS-CL131
209529	GCA_000853305.1	Aphid lethal paralysis virus
1897732	GCA_001444105.1	Aphis glycines virus 2

2005444	GCA_002194445.1	Aphis glycines virus 3
1983569	GCA_002210615.1	Apis dicistrovirus
1983570	GCA_002204045.1	Apis flavivirus
2494736	GCA_004141515.1	Apis mellifera associated microvirus 1
2494737	GCA_004141255.1	Apis mellifera associated microvirus 10
2494738	GCA_004141675.1	Apis mellifera associated microvirus 11
2494738	GCA_004141575.1	Apis mellifera associated microvirus 11
2494739	GCA_004141035.1	Apis mellifera associated microvirus 12
2494740	GCA_004140755.1	Apis mellifera associated microvirus 13
2494741	GCA_004140775.1	Apis mellifera associated microvirus 14
2494742	GCA_004141275.1	Apis mellifera associated microvirus 15
2494743	GCA_004140715.1	Apis mellifera associated microvirus 16
2494744	GCA_004141195.1	Apis mellifera associated microvirus 17
2494745	GCA_004141215.1	Apis mellifera associated microvirus 18
2494746	GCA_004141235.1	Apis mellifera associated microvirus 19
2494747	GCA_004141635.1	Apis mellifera associated microvirus 2
2494748	GCA_004141555.1	Apis mellifera associated microvirus 20
2494749	GCA_004140995.1	Apis mellifera associated microvirus 21
2494750	GCA_004141375.1	Apis mellifera associated microvirus 22
2494751	GCA_004140875.1	Apis mellifera associated microvirus 23
2494752	GCA_004140835.1	Apis mellifera associated microvirus 24
2494752	GCA_004141595.1	Apis mellifera associated microvirus 24
2494752	GCA_004140615.1	Apis mellifera associated microvirus 24
2494753	GCA_004141175.1	Apis mellifera associated microvirus 25
2494754	GCA_004140935.1	Apis mellifera associated microvirus 26
2494755	GCA_004140635.1	Apis mellifera associated microvirus 27
2494756	GCA_004140955.1	Apis mellifera associated microvirus 28
2494757	GCA_004141095.1	Apis mellifera associated microvirus 29
2494758	GCA_004141535.1	Apis mellifera associated microvirus 3
2494758	GCA_004140575.1	Apis mellifera associated microvirus 3
2494759	GCA_004140855.1	Apis mellifera associated microvirus 30
2494760	GCA_004140495.1	Apis mellifera associated microvirus 31
2494761	GCA_004140915.1	Apis mellifera associated microvirus 32
2494762	GCA_004140735.1	Apis mellifera associated microvirus 33
2494763	GCA_004141475.1	Apis mellifera associated microvirus 34
2494764	GCA_004141115.1	Apis mellifera associated microvirus 35
2494765	GCA_004141415.1	Apis mellifera associated microvirus 36
2494766	GCA_004141655.1	Apis mellifera associated microvirus 37
2494767	GCA_004140375.1	Apis mellifera associated microvirus 38
2494768	GCA_004141015.1	Apis mellifera associated microvirus 39
2494769	GCA_004141735.1	Apis mellifera associated microvirus 4
2494769	GCA_004140655.1	Apis mellifera associated microvirus 4
2494769	GCA_004140535.1	Apis mellifera associated microvirus 4
2494769	GCA_004141615.1	Apis mellifera associated microvirus 4
2494770	GCA_004141155.1	Apis mellifera associated microvirus 40
2494771	GCA_004141395.1	Apis mellifera associated microvirus 41
2494772	GCA_004141355.1	Apis mellifera associated microvirus 42
2494773	GCA_004141335.1	Apis mellifera associated microvirus 43
2494774	GCA_004140415.1	Apis mellifera associated microvirus 44
2494775	GCA_004141055.1	Apis mellifera associated microvirus 45

2494776	GCA_004141795.1	Apis mellifera associated microvirus 46
2494777	GCA_004141455.1	Apis mellifera associated microvirus 47
2494778	GCA_004140515.1	Apis mellifera associated microvirus 48
2494779	GCA_004140895.1	Apis mellifera associated microvirus 49
2494780	GCA_004140595.1	Apis mellifera associated microvirus 5
2494780	GCA_004141695.1	Apis mellifera associated microvirus 5
2494781	GCA_004141075.1	Apis mellifera associated microvirus 50
2494782	GCA_004141765.1	Apis mellifera associated microvirus 51
2494783	GCA_004141315.1	Apis mellifera associated microvirus 52
2494784	GCA_004141295.1	Apis mellifera associated microvirus 53
2494785	GCA_004141435.1	Apis mellifera associated microvirus 54
2494786	GCA_004140435.1	Apis mellifera associated microvirus 55
2494787	GCA_004140555.1	Apis mellifera associated microvirus 56
2494788	GCA_004140455.1	Apis mellifera associated microvirus 57
2494789	GCA_004140795.1	Apis mellifera associated microvirus 58
2494790	GCA_004140675.1	Apis mellifera associated microvirus 59
2494791	GCA_004140475.1	Apis mellifera associated microvirus 6
2494792	GCA_004141495.1	Apis mellifera associated microvirus 60
2494792	GCA_004140975.1	Apis mellifera associated microvirus 60
2494793	GCA_004141135.1	Apis mellifera associated microvirus 61
2494794	GCA_004140395.1	Apis mellifera associated microvirus 7
2494795	GCA_004140695.1	Apis mellifera associated microvirus 8
2494796	GCA_004140815.1	Apis mellifera associated microvirus 9
1100043	GCA_001308775.1	Apis mellifera filamentous virus
168135	GCA_000891455.1	Apium virus Y
2283236	GCA_004133285.1	Aplysia californica nido-like virus
307461	GCA_000900035.1	Apocheima cinerarium nucleopolyhedrovirus
1036963	GCA_000926195.1	Apodemus sylvaticus papillomavirus 1
64280	GCA_000863285.1	Apoi virus
2059312	GCA_004127975.1	Apore mammarenavirus
12175	GCA_000848285.1	Apple chlorotic leaf spot virus
73494	GCA_000854605.1	Apple dimple fruit viroid
1633752	GCA_000969055.1	Apple geminivirus PL-2015
1211388	GCA_000898715.1	Apple green crinkle associated virus
101688	GCA_000861545.1	Apple latent spherical virus
2170544	GCA_004130875.1	Apple luteovirus 1
12319	GCA_000849545.1	Apple mosaic virus
1779339	GCA_004117155.1	Apple necrotic mosaic virus
12895	GCA_000854585.1	Apple scar skin viroid
28347	GCA_000859505.1	Apple stem grooving virus
35350	GCA_000850465.1	Apple stem pitting virus
2054409	GCA_004131505.1	Apple-associated luteovirus
75387	GCA_000888875.1	Apricot latent virus
307677	GCA_000858925.1	Apricot pseudo-chlorotic leaf spot virus
1343920	GCA_000916795.1	Apricot vein clearing associated virus
1634476	GCA_004129215.1	Apteryx rowi circovirus-like virus
1927013	GCA_002617405.1	Aquamicrobium phage P14
1715293	GCA_002366045.1	Aquatic bird bornavirus 1
1884917	GCA_001589995.1	Aquatic bird bornavirus 2
1849335	GCA_001725875.1	Arabidopsis halleri partitivirus 1

12271	GCA_000855205.1	Arabis mosaic virus
190809	GCA_000850505.1	Arabis mosaic virus large satellite RNA
196399	GCA_000836845.1	Arabis mosaic virus small satellite RNA
857288	GCA_000899115.1	Arabis mosaic virus small satellite RNA barley/CHE/1991
1921009	GCA_001904945.1	Arachis pintoi virus
211977	GCA_000905955.1	Aravan lyssavirus
2293273	GCA_003847205.1	Arboreal ant associated circular virus 1
2293273	GCA_003847185.1	Arboreal ant associated circular virus 1
2293273	GCA_003847225.1	Arboreal ant associated circular virus 1
1972683	GCA_000927335.1	Arboretum almendravirus
416419	GCA_000870325.1	Archaeal BJ1 virus
2282642	GCA_004134125.1	Arctopus echinatus-associated virus
2306623	GCA_004134025.1	Areca palm necrotic spindle-spot virus
1654603	GCA_001019835.1	Areca palm velarivirus 1
1518603	GCA_002818845.1	Arenaviridae sp. 13ZR68
2169991	GCA_000856545.1	Argentinian mammarenavirus
1585924	GCA_002289195.1	Arhar cryptic virus-I
72201	GCA_000923155.1	Armadillidium vulgare iridescent virus
2018471	GCA_002889975.1	Armigeres iflavirus
556524	GCA_000888675.1	Armigeres subalbatus virus SaX06-AK20
1457322	GCA_001550565.1	Arrabida virus
1457322	GCA_001550565.2	Arrabida virus
405558	GCA_000897375.1	Arracacha mottle virus
2201042	GCA_004133325.1	Arracacha virus 1
257463	GCA_000907115.1	Arracacha virus B
1972716	GCA_002116215.1	Arracacha virus V
2170061	GCA_003032565.1	Artashat orthonairovirus
1692243	GCA_001274325.1	Artemia melana sponge associated circular genome
1133751	GCA_000896195.1	Artemisia virus A
2015853	GCA_002626285.1	Arthrobacter phage Abidatro
2419945	GCA_003691915.1	Arthrobacter phage Adaia
2027883	GCA_002629205.1	Arthrobacter phage Adat
2488951	GCA_003868355.1	Arthrobacter phage Aledel
1772291	GCA_002622925.1	Arthrobacter phage Amigo
1772292	GCA_002608465.1	Arthrobacter phage Anansi
2419946	GCA_003692515.1	Arthrobacter phage Andrew
2484217	GCA_003722535.1	Arthrobacter phage Anjali
2024274	GCA_002628285.1	Arthrobacter phage Arcadia
2419947	GCA_003691955.1	Arthrobacter phage Atraxa
2419948	GCA_003691755.1	Arthrobacter phage Auxilium
2283238	GCA_003365995.1	Arthrobacter phage Azathoth
1796994	GCA_001754425.1	Arthrobacter phage BarretLemon
2015815	GCA_002625565.1	Arthrobacter phage Beans
2488952	GCA_003868315.1	Arthrobacter phage Beethoven
1772293	GCA_002622705.1	Arthrobacter phage Bennie
2488953	GCA_003868295.1	Arthrobacter phage BigMack
2517928	GCA_004520455.1	Arthrobacter phage Blair
2488954	GCA_003868245.1	Arthrobacter phage Bodacious
2250408	GCA_003364455.1	Arthrobacter phage Brad
1701798	GCA_002622425.1	Arthrobacter phage Brent

2250409	GCA_003364475.1	Arthrobacter phage Breylor17
2419949	GCA_003692035.1	Arthrobacter phage Bridgette
2488955	GCA_003868155.1	Arthrobacter phage CallieOMalley
2015854	GCA_002626305.1	Arthrobacter phage Canowicakte
1772294	GCA_002622965.1	Arthrobacter phage CaptnMurica
2488956	GCA_003868095.1	Arthrobacter phage Carpal
2015855	GCA_002626325.1	Arthrobacter phage Caterpillar
2283239	GCA_003366035.1	Arthrobacter phage CGermain
2015816	GCA_002625585.1	Arthrobacter phage Cheesy
1933772	GCA_002618745.1	Arthrobacter phage Chestnut
2498999	GCA_004006975.1	Arthrobacter phage ChewChew
2517929	GCA_004520515.1	Arthrobacter phage Chipper1996
1897526	GCA_002613125.1	Arthrobacter phage Chocolat
2499000	GCA_004008755.1	Arthrobacter phage Cholula
2015856	GCA_002626345.1	Arthrobacter phage Christian
1897527	GCA_002613145.1	Arthrobacter phage Chubster
1772295	GCA_002622905.1	Arthrobacter phage Circum
2015834	GCA_002625945.1	Arthrobacter phage Colucci
1873902	GCA_002757875.1	Arthrobacter phage Conboy
2419950	GCA_003692075.1	Arthrobacter phage Constance
2283240	GCA_003366095.1	Arthrobacter phage Copper
2419951	GCA_003692535.1	Arthrobacter phage Coral
2419952	GCA_003692095.1	Arthrobacter phage Corgi
2024275	GCA_002628305.1	Arthrobacter phage Correa
2419953	GCA_003692115.1	Arthrobacter phage Cote
1874014	GCA_002598605.1	Arthrobacter phage Courtney3
2499001	GCA_004007165.1	Arthrobacter phage CristinaYang
2250410	GCA_003364515.1	Arthrobacter phage Daiboju
2419954	GCA_003692135.1	Arthrobacter phage Daob
1698361	GCA_001470355.1	Arthrobacter phage Decurro
2283241	GCA_003366115.1	Arthrobacter phage Dewayne
2015857	GCA_002626365.1	Arthrobacter phage Dino
2419955	GCA_003692155.1	Arthrobacter phage DrManhattan
1772296	GCA_002622725.1	Arthrobacter phage DrRobert
1913079	GCA_002614965.1	Arthrobacter phage EdgarPoe
2419956	GCA_003692175.1	Arthrobacter phage Eileen
2014348	GCA_002625265.1	Arthrobacter phage ElephantMan
2510522	GCA_004147205.1	Arthrobacter phage Elesar
2015858	GCA_002626385.1	Arthrobacter phage Elkhorn
2024276	GCA_002628325.1	Arthrobacter phage Elsa
2499002	GCA_004007075.1	Arthrobacter phage Eunoia
2419957	GCA_003692195.1	Arthrobacter phage Faja
2047865	GCA_002956435.1	Arthrobacter phage Fluke
2024007	GCA_002627625.1	Arthrobacter phage Franzy
1772326	GCA_002608765.1	Arthrobacter phage Galaxy
1772297	GCA_002622745.1	Arthrobacter phage Glenn
1772298	GCA_002622985.1	Arthrobacter phage Gordon
1772299	GCA_002608505.1	Arthrobacter phage Gorgeous
2499003	GCA_004007235.1	Arthrobacter phage GreenHearts
1897428	GCA_002612225.1	Arthrobacter phage Greenhouse

2315528	GCA_003614245.1	Arthrobacter phage Guntur
2027884	GCA_002629225.1	Arthrobacter phage GurgleFerb
2499004	GCA_004008455.1	Arthrobacter phage HeadNerd
2024277	GCA_002628345.1	Arthrobacter phage Heisenberger
2250411	GCA_003364535.1	Arthrobacter phage Herb
2419609	GCA_003723075.1	Arthrobacter phage Hestia
2499005	GCA_004007295.1	Arthrobacter phage Huckleberry
1913080	GCA_002614985.1	Arthrobacter phage HumptyDumpty
2283242	GCA_003366175.1	Arthrobacter phage Hunnie
1772300	GCA_002622765.1	Arthrobacter phage HunterDalle
2047760	GCA_002956165.1	Arthrobacter phage Huntingdon
1772301	GCA_002608535.1	Arthrobacter phage Immaculata
2283243	GCA_003366215.1	Arthrobacter phage Inspire2
2419610	GCA_003723095.1	Arthrobacter phage Isolde
1772302	GCA_002608575.1	Arthrobacter phage Jasmine
1772327	GCA_002622445.1	Arthrobacter phage Jawnski
2027885	GCA_002629245.1	Arthrobacter phage JayCookie
1698362	GCA_002598625.1	Arthrobacter phage Jessica
1772303	GCA_002622785.1	Arthrobacter phage Joann
2024281	GCA_002628425.1	Arthrobacter phage Jordan
2419958	GCA_003692235.1	Arthrobacter phage Judy
2015835	GCA_002625965.1	Arthrobacter phage Kabreeze
2315608	GCA_003613795.1	Arthrobacter phage KBurrousTX
2250412	GCA_003364575.1	Arthrobacter phage KeaneyLin
1796995	GCA_001755285.1	Arthrobacter phage KellEzio
2419959	GCA_003692555.1	Arthrobacter phage Kepler
2250413	GCA_003364595.1	Arthrobacter phage KingBob
1796996	GCA_001755705.1	Arthrobacter phage Kitkat
1772304	GCA_002622805.1	Arthrobacter phage Korra
2015859	GCA_002626405.1	Arthrobacter phage KylieMac
1772305	GCA_002622885.1	Arthrobacter phage Laroye
2499006	GCA_004007575.1	Arthrobacter phage Lasagna
2315701	GCA_003613515.1	Arthrobacter phage LeeroyJ
2499007	GCA_004007375.1	Arthrobacter phage Lennox
2488780	GCA_003867095.1	Arthrobacter phage Liebe
2015861	GCA_002626445.1	Arthrobacter phage Link
2015860	GCA_002626425.1	Arthrobacter phage LiSara
2499008	GCA_004007395.1	Arthrobacter phage Litotes
2015862	GCA_002626465.1	Arthrobacter phage Lore
1897761	GCA_002613965.1	Arthrobacter phage Lucy
2419960	GCA_003692295.1	Arthrobacter phage Lunar
1772306	GCA_002598685.1	Arthrobacter phage Maggie
2499009	GCA_004008425.1	Arthrobacter phage Maja
2250414	GCA_003364635.1	Arthrobacter phage MargaretKali
2015863	GCA_002626485.1	Arthrobacter phage Mariposa
1772307	GCA_002622465.1	Arthrobacter phage Martha
1897762	GCA_002613985.1	Arthrobacter phage Massimo
2419961	GCA_003692315.1	Arthrobacter phage Maureen
2250415	GCA_003364675.1	Arthrobacter phage MediumFry
2047866	GCA_002956445.1	Arthrobacter phage MeganNoll

2419962	GCA_003692335.1	Arthrobacter phage Melons
2484218	GCA_003722715.1	Arthrobacter phage Mendel
2315609	GCA_003613835.1	Arthrobacter phage Moki
2015839	GCA_002626045.1	Arthrobacter phage Molivia
1772308	GCA_002598705.1	Arthrobacter phage Moloch
1796997	GCA_001754585.2	Arthrobacter phage Mudcat
1796997	GCA_001754585.1	Arthrobacter phage Mudcat
1772309	GCA_002608605.1	Arthrobacter phage Muttlie
2499010	GCA_004007515.1	Arthrobacter phage Nancia
2419963	GCA_003692355.1	Arthrobacter phage Nandita
2024278	GCA_002628365.1	Arthrobacter phage Nason
2027886	GCA_002629265.1	Arthrobacter phage Nellie
2015864	GCA_002626505.1	Arthrobacter phage Nightmare
2014347	GCA_002625245.1	Arthrobacter phage Niktson
2419964	GCA_003691775.1	Arthrobacter phage Noely
2015865	GCA_002626525.1	Arthrobacter phage Nubia
2499011	GCA_004007605.1	Arthrobacter phage OMalley
2499012	GCA_004007635.1	Arthrobacter phage OurGirlNessie
1897429	GCA_002612245.1	Arthrobacter phage Oxynfrius
2419965	GCA_003692375.1	Arthrobacter phage Peas
2024282	GCA_002628445.1	Arthrobacter phage Piccoletto
2015866	GCA_002626545.1	Arthrobacter phage PitaDog
2419966	GCA_003692395.1	Arthrobacter phage Polka
2499013	GCA_004008255.1	Arthrobacter phage Potatoes
1772310	GCA_002622825.1	Arthrobacter phage Preamble
1772328	GCA_002757175.1	Arthrobacter phage PrincessTrina
1897502	GCA_002612885.1	Arthrobacter phage Prospero
2499014	GCA_004007795.1	Arthrobacter phage Pterodactyl
1772311	GCA_002622845.1	Arthrobacter phage Pumancara
1772312	GCA_002608625.1	Arthrobacter phage RAP15
1897763	GCA_002614005.1	Arthrobacter phage RcigaStruga
2419967	GCA_003692575.1	Arthrobacter phage Richie
1772313	GCA_002608645.1	Arthrobacter phage Rings
2499015	GCA_004007695.1	Arthrobacter phage Riovina
2499016	GCA_004007715.1	Arthrobacter phage Riverdale
2283244	GCA_003366295.1	Arthrobacter phage Ronnie
2015836	GCA_002625985.1	Arthrobacter phage RosiePosie
2499017	GCA_004007735.1	Arthrobacter phage Rozby
2419968	GCA_003692435.1	Arthrobacter phage Ryan
1772314	GCA_002608665.1	Arthrobacter phage Salgado
1698363	GCA_002598645.1	Arthrobacter phage Sandman
2499018	GCA_004007855.1	Arthrobacter phage Savage2526
2015837	GCA_002626005.1	Arthrobacter phage Scavito
2419611	GCA_003723175.1	Arthrobacter phage Seahorse
2250416	GCA_003364795.1	Arthrobacter phage Sergei
2015867	GCA_002626565.1	Arthrobacter phage Seume
2024283	GCA_002628465.1	Arthrobacter phage Shade
2015868	GCA_002626585.1	Arthrobacter phage Shrooms
2510495	GCA_004147225.1	Arthrobacter phage Sonali
1772315	GCA_002622485.1	Arthrobacter phage Sonny

1772316	GCA_002608685.1	Arthrobacter phage SorJuana
2419969	GCA_003692455.1	Arthrobacter phage Sputnik
2517930	GCA_004520595.1	Arthrobacter phage StewieGriff
1772317	GCA_002598725.1	Arthrobacter phage Stratus
2499019	GCA_004008045.1	Arthrobacter phage Supakev
1897553	GCA_002613565.1	Arthrobacter phage Suppi
2015869	GCA_002626605.1	Arthrobacter phage Swenson
2250389	GCA_003365135.1	Arthrobacter phage Synepsis
1772318	GCA_002608705.1	Arthrobacter phage TaeYoung
2015870	GCA_002626625.1	Arthrobacter phage Taj14
1772319	GCA_002623305.1	Arthrobacter phage Tank
2250368	GCA_003341795.1	Arthrobacter phage Tatanka
2499020	GCA_004007895.1	Arthrobacter phage TattModd
2015871	GCA_002626645.1	Arthrobacter phage Teacup
2027887	GCA_002629285.1	Arthrobacter phage Temper16
2315702	GCA_003614575.1	Arthrobacter phage Tenno
2024006	GCA_002627605.1	Arthrobacter phage Timinator
2015872	GCA_002626665.1	Arthrobacter phage TinoCrisci
2015838	GCA_002626025.1	Arthrobacter phage Tophat
1772320	GCA_002598745.1	Arthrobacter phage Toulouse
2024279	GCA_002628385.1	Arthrobacter phage Tribby
1735465	GCA_002598665.1	Arthrobacter phage TymAbreu
2047867	GCA_002956455.1	Arthrobacter phage Urla
1897554	GCA_002613585.1	Arthrobacter phage Vallejo
1414742	GCA_000911555.1	Arthrobacter phage vB_ArS-ArV2
1566993	GCA_000954695.1	Arthrobacter phage vB_ArtM-ArV1
1772321	GCA_002608725.1	Arthrobacter phage Vulture
2047828	GCA_002956235.1	Arthrobacter phage Waltz
2499021	GCA_004007995.1	Arthrobacter phage Wawa
1772322	GCA_002622865.1	Arthrobacter phage Wayne
2015873	GCA_002626685.1	Arthrobacter phage Wheelbite
1772323	GCA_002608745.1	Arthrobacter phage Wilde
2419970	GCA_003692475.1	Arthrobacter phage Yang
1772324	GCA_002598765.1	Arthrobacter phage Yank
2499022	GCA_004008175.1	Arthrobacter phage Zorro
1131485	GCA_000894295.1	Artibeus jamaicensis parvovirus 1
1606497	GCA_002827885.1	Artibeus planirostris polyomavirus 1
1606498	GCA_002827805.1	Artibeus planirostris polyomavirus 2
1606499	GCA_002827825.1	Artibeus planirostris polyomavirus 3
46075	GCA_002986035.1	Artichoke Italian latent virus
46076	GCA_000969175.1	Artichoke latent virus
12142	GCA_000864965.1	Artichoke mottled crinkle virus
362830	GCA_000884655.1	Artogeia rapae granulovirus
904698	GCA_000914815.1	Arumowot virus
1027880	GCA_000891115.1	Asclepias asymptomatic virus
2249930	GCA_004134085.1	Ashy storm petrel gyrovirus
351427	GCA_000924795.1	Asian prunus virus 1
351426	GCA_001502755.1	Asian prunus virus 2
351428	GCA_001502135.1	Asian prunus virus 3
1980458	GCA_002815935.1	Asikkala orthohantavirus

443746	GCA_000928895.1	Asparagus virus 1
39681	GCA_000881975.1	Asparagus virus 2
445435	GCA_000855185.1	Asparagus virus 3
445435	GCA_000879175.1	Asparagus virus 3
1087068	GCA_000905675.1	Aspergillus foetidus dsRNA mycovirus
1087070	GCA_002988045.1	Aspergillus foetidus slow virus 1
607716	GCA_002986295.1	Aspergillus fumigatus chrysovirus
2250452	GCA_004117595.1	Aspergillus fumigatus partitivirus 2
2250469	GCA_004127995.1	Aspergillus fumigatus polymycovirus 1
358008	GCA_002868595.1	Aspergillus ochraceous virus
1676988	GCA_004061695.1	Asterias forbesi associated circular virus
1522179	GCA_000923275.1	Asterionellopsis glacialis RNA virus
1671382	GCA_001045265.1	Astrovirus Er/SZAL6/HUN/2011
568715	GCA_000880715.1	Astrovirus MLB1
683172	GCA_000895575.1	Astrovirus MLB2
1247114	GCA_000899535.1	Astrovirus MLB3
645687	GCA_000885815.1	Astrovirus VA1
683174	GCA_000901475.1	Astrovirus VA3
1247113	GCA_000897955.1	Astrovirus VA4
1137931	GCA_000895955.1	Astrovirus wild boar/WBAstV-1/2011/HUN
1611435	GCA_000943705.1	Asystasia mosaic Madagascar virus
1236391	GCA_000902815.1	Ateles paniscus polyomavirus 1
35243	GCA_002118845.1	Ateline alphaherpesvirus 1
85618	GCA_000839425.1	Ateline gammaherpesvirus 3
2315724	GCA_004117495.1	Athtab bunya-like virus
37961	GCA_000837505.2	Atkinsonella hypoxylon virus
37961	GCA_000837505.1	Atkinsonella hypoxylon virus
450278	GCA_003972645.1	Atlantic cod nodavirus
1489836	GCA_000919195.1	Atlantic salmon calicivirus
348296	GCA_000864385.1	Atlantic salmon swim bladder sarcoma virus
1711685	GCA_001308615.1	Atractylodes mild mottle virus
1702121	GCA_003029105.1	Atractylodes mottle virus
1873455	GCA_001695485.1	Atypical porcine pestivirus 1
44158	GCA_000852325.1	Aura virus
1503929	GCA_001041735.1	Aurantimonas phage AmM-1
674971	GCA_000865185.1	Aurantiochytrium single-stranded RNA virus 01
1474867	GCA_000922335.1	Aureococcus anophagefferens virus
2026601	GCA_002354925.1	Australian Anopheles totivirus
90961	GCA_000850325.1	Australian bat lyssavirus
190810	GCA_000854625.1	Australian grapevine viroid
46015	GCA_000838485.1	Autographa californica nucleopolyhedrovirus
1810950	GCA_004128015.1	Avalon virus
1239437	GCA_000857825.1	Avastrovirus 1
1239439	GCA_000856205.1	Avastrovirus 3
1239439	GCA_000883675.1	Avastrovirus 3
219704	GCA_000844805.1	Avian adeno-associated virus ATCC VR-865
281492	GCA_000846565.1	Avian adeno-associated virus strain DA-1
11176	GCA_002834085.1	Avian avulavirus 1
1204252	GCA_000924755.1	Avian avulavirus 11
1305716	GCA_000927075.1	Avian avulavirus 12

35302	GCA_001433585.1	Avian avulavirus 2
35302	GCA_002989675.1	Avian avulavirus 2
2067666	GCA_004130815.1	Avian avulavirus 20
207246	GCA_000927055.1	Avian avulavirus 3
207246	GCA_002815155.1	Avian avulavirus 3
28274	GCA_000901955.1	Avian avulavirus 4
28274	GCA_002815175.1	Avian avulavirus 4
740710	GCA_000924655.1	Avian avulavirus 5
157619	GCA_000848585.1	Avian avulavirus 6
157619	GCA_002815195.1	Avian avulavirus 6
622416	GCA_000926535.1	Avian avulavirus 7
623144	GCA_000925495.1	Avian avulavirus 8
623144	GCA_002815215.1	Avian avulavirus 8
580247	GCA_000926655.1	Avian avulavirus 9
11870	GCA_000849005.1	Avian carcinoma Mill Hill virus 2
93465	GCA_000859965.1	Avian endogenous retrovirus EAV-HP
1002273	GCA_000891935.1	Avian gyrovirus 2
2364132	GCA_004134625.1	Avian HDV-like agent
172851	GCA_002826385.1	Avian hepatitis E virus
172851	GCA_002826425.1	Avian hepatitis E virus
172851	GCA_002826405.1	Avian hepatitis E virus
11946	GCA_000891575.1	Avian leukemia virus
363745	GCA_000849845.1	Avian leukosis virus - RSA
38525	GCA_000865325.1	Avian metapneumovirus
38525	GCA_002815335.1	Avian metapneumovirus
38525	GCA_002815355.1	Avian metapneumovirus
38525	GCA_002989735.1	Avian metapneumovirus
519376	GCA_002985985.1	Avian metapneumovirus type D
11868	GCA_000853485.1	Avian myelocytomatosis virus 29
336960	GCA_000852205.1	Avian nephritis virus 1
38170	GCA_000891595.1	Avian orthoreovirus
38170	GCA_003092995.1	Avian orthoreovirus
38170	GCA_003093035.1	Avian orthoreovirus
38170	GCA_003093095.1	Avian orthoreovirus
38170	GCA_003093115.1	Avian orthoreovirus
38170	GCA_003093135.1	Avian orthoreovirus
38170	GCA_003093155.1	Avian orthoreovirus
38170	GCA_003093175.1	Avian orthoreovirus
38170	GCA_003092515.1	Avian orthoreovirus
38170	GCA_003092535.1	Avian orthoreovirus
38170	GCA_003092555.1	Avian orthoreovirus
38170	GCA_003092575.1	Avian orthoreovirus
38170	GCA_003092595.1	Avian orthoreovirus
38170	GCA_003092635.1	Avian orthoreovirus
38170	GCA_003092655.1	Avian orthoreovirus
38170	GCA_003092695.1	Avian orthoreovirus
38170	GCA_003092715.1	Avian orthoreovirus
38170	GCA_003092735.1	Avian orthoreovirus
38170	GCA_003092755.1	Avian orthoreovirus
38170	GCA_003092775.1	Avian orthoreovirus

38170	GCA_003092795.1	Avian orthoreovirus
38170	GCA_003092815.1	Avian orthoreovirus
38170	GCA_003092835.1	Avian orthoreovirus
38170	GCA_003092895.1	Avian orthoreovirus
38170	GCA_003092915.1	Avian orthoreovirus
38170	GCA_003092935.1	Avian orthoreovirus
38170	GCA_003092955.1	Avian orthoreovirus
38170	GCA_003092975.1	Avian orthoreovirus
1928005	GCA_003032665.1	Avian paramyxovirus 14
1983777	GCA_002197575.1	Avian paramyxovirus 15
1401445	GCA_001654405.1	Avian paramyxovirus goose/Shimane/67/2000
862945	GCA_002080355.1	Avian paramyxovirus penguin/Falkland Islands/324/2007
2006690	GCA_003032675.1	Avian paramyxovirus UPO216
686983	GCA_000854225.1	Avian sapelovirus
11878	GCA_002987745.1	Avian sarcoma virus CT10
385048	GCA_000868085.1	Avian sarcoma virus PR2257/16
1844928	GCA_001651125.1	Avian-like circovirus
1766559	GCA_001502795.1	Avisivirus Pf-CHK1/AsV
12896	GCA_000853625.1	Avocado sunblotch viroid
1941437	GCA_002004635.1	Avon-Heathcote estuary associated bacilladnavirus
1618232	GCA_000954875.1	Avon-Heathcote Estuary associated circular virus 1
1618233	GCA_000954515.1	Avon-Heathcote Estuary associated circular virus 10
1618234	GCA_000955235.1	Avon-Heathcote Estuary associated circular virus 11
1618235	GCA_000955575.1	Avon-Heathcote Estuary associated circular virus 12
1618236	GCA_000954855.1	Avon-Heathcote Estuary associated circular virus 13
1618237	GCA_000954495.1	Avon-Heathcote Estuary associated circular virus 14
1618238	GCA_000955215.1	Avon-Heathcote Estuary associated circular virus 15
1618239	GCA_000955555.1	Avon-Heathcote Estuary associated circular virus 16
1618240	GCA_000954835.1	Avon-Heathcote Estuary associated circular virus 17
1618242	GCA_000955195.1	Avon-Heathcote Estuary associated circular virus 19
1618243	GCA_000955535.1	Avon-Heathcote Estuary associated circular virus 2
1618244	GCA_000954815.1	Avon-Heathcote Estuary associated circular virus 20
1618245	GCA_000954455.1	Avon-Heathcote Estuary associated circular virus 21
1618246	GCA_000955175.1	Avon-Heathcote Estuary associated circular virus 22
1618247	GCA_000955515.1	Avon-Heathcote Estuary associated circular virus 23
1618248	GCA_000954795.1	Avon-Heathcote Estuary associated circular virus 24
1618249	GCA_000954435.1	Avon-Heathcote Estuary associated circular virus 25
1618250	GCA_000955155.1	Avon-Heathcote Estuary associated circular virus 26
1618251	GCA_000955495.1	Avon-Heathcote Estuary associated circular virus 27
1618252	GCA_000954775.1	Avon-Heathcote Estuary associated circular virus 28
1618253	GCA_000954415.1	Avon-Heathcote Estuary associated circular virus 29
1618254	GCA_000954575.1	Avon-Heathcote Estuary associated circular virus 3
1618255	GCA_000955295.1	Avon-Heathcote Estuary associated circular virus 4
1618256	GCA_000955635.1	Avon-Heathcote Estuary associated circular virus 5
1618257	GCA_000954915.1	Avon-Heathcote Estuary associated circular virus 6
1618258	GCA_000954555.1	Avon-Heathcote Estuary associated circular virus 7
1618259	GCA_000955275.1	Avon-Heathcote Estuary associated circular virus 8
1618260	GCA_000955615.1	Avon-Heathcote Estuary associated circular virus 9
1476487	GCA_000920415.1	Axonopus compressus streak virus
1920526	GCA_002091415.1	Azobacteroides phage ProJPt-Bp1

1920526	GCA_002091435.1	Azobacteroides phage ProJPt-Bp1
1920526	GCA_002091455.1	Azobacteroides phage ProJPt-Bp1
1920526	GCA_002633435.1	Azobacteroides phage ProJPt-Bp1
467481	GCA_000872705.1	Azospirillum phage Cd
2060511	GCA_002890015.1	Babaco mosaic virus
48540	GCA_002889295.1	Babanki virus
11764	GCA_000912135.1	Baboon endogenous virus strain M7
75888	GCA_000891275.1	Baboon orthoreovirus
2202647	GCA_003652385.1	Bacilladnaviridae sp.
2202647	GCA_003657365.1	Bacilladnaviridae sp.
2267680	GCA_003656665.1	Bacilladnavirus sp.
1379694	GCA_000928715.1	Bacillariodnavirus LDMD-2013
458639	GCA_000871045.1	Bacillus phage 0305phi8-36
2024230	GCA_002627805.1	Bacillus phage AaronPhadgers
2108114	GCA_003014185.1	Bacillus phage Anath
2024253	GCA_002628145.1	Bacillus phage Anthony
2483609	GCA_003865635.1	Bacillus phage AP631
1815509	GCA_001743835.1	Bacillus phage AR9
1874000	GCA_001743675.1	Bacillus phage Aurora
1698451	GCA_001505635.1	Bacillus phage AvesoBmore
1141133	GCA_000897755.1	Bacillus phage B4
1126949	GCA_002602065.1	Bacillus phage B5S
1567487	GCA_001736435.1	Bacillus phage BalMu-1
1567487	GCA_002605265.1	Bacillus phage BalMu-1
236750	GCA_000841545.1	Bacillus phage Bam35c
1296654	GCA_002603345.1	Bacillus phage Basilisk
57477	GCA_000899475.1	Bacillus phage Bastille
2039884	GCA_003555485.1	Bacillus phage BC01
1136534	GCA_000900775.1	Bacillus phage BCD7
1118064	GCA_003047795.1	Bacillus phage BceA1
294382	GCA_000846925.1	Bacillus phage BCJA1c
2059878	GCA_003979575.1	Bacillus phage BCP01
584892	GCA_000918315.1	Bacillus phage Bcp1
1913122	GCA_003085815.1	Bacillus phage BCP12
1126950	GCA_000898735.1	Bacillus phage BCP78
1129192	GCA_001041555.1	Bacillus phage BCP8-2
1126951	GCA_002602025.1	Bacillus phage BCU4
1983461	GCA_002623545.1	Bacillus phage BeachBum
1852564	GCA_001743915.1	Bacillus phage Belinda
1406781	GCA_000912355.1	Bacillus phage BigBertha
1909402	GCA_002614365.1	Bacillus phage BJ4
1735561	GCA_002607565.1	Bacillus phage BMBtp1
1868824	GCA_002611045.1	Bacillus phage BMBtpLA3
1527469	GCA_000921655.1	Bacillus phage Bobb
1445810	GCA_001551765.1	Bacillus phage Bp8p-C
1445811	GCA_002604305.1	Bacillus phage Bp8p-T
1277886	GCA_000915475.1	Bacillus phage BPS10C
1136731	GCA_000900195.1	Bacillus phage BPS13
2069312	GCA_002957965.1	Bacillus phage BSP10
2283013	GCA_003367035.1	Bacillus phage BSP38

2041339	GCA_003601495.1	Bacillus phage BSP9
1194641	GCA_000898915.1	Bacillus phage BtCS33
2069313	GCA_002957975.1	Bacillus phage BtiUFT6.51-F
2024231	GCA_002627825.1	Bacillus phage Bubs
1486657	GCA_000920935.1	Bacillus phage CAM003
1406783	GCA_000911315.1	Bacillus phage CampHawk
1983578	GCA_002623805.1	Bacillus phage Carmel_SA
2072797	GCA_002958285.1	Bacillus phage Carmen17
347966	GCA_002758475.1	Bacillus phage Cherry
1874001	GCA_001744995.1	Bacillus phage Claudi
1391188	GCA_000926735.1	Bacillus phage CP-51
1808964	GCA_002609465.1	Bacillus phage Crookii
1792245	GCA_001745535.1	Bacillus phage Deep Blue
1873341	GCA_002611685.1	Bacillus phage Deep-Purple
1805948	GCA_001744435.1	Bacillus phage DIGNKC
1873999	GCA_001744355.1	Bacillus phage DirtyBetty
2500808	GCA_004135185.1	Bacillus phage DK1
2500809	GCA_004135225.1	Bacillus phage DK2
2500810	GCA_004135245.1	Bacillus phage DK3
1776293	GCA_001736835.1	Bacillus phage Eldridge
1486658	GCA_000922835.1	Bacillus phage Evoli
1690431	GCA_001501855.1	Bacillus phage Eyuki
345922	GCA_000867025.1	Bacillus phage Fah
1983465	GCA_002623565.1	Bacillus phage Flapjack
347962	GCA_000864165.1	Bacillus phage Gamma
347962	GCA_002786445.1	Bacillus phage Gamma
347962	GCA_003006245.1	Bacillus phage Gamma
359962	GCA_002758495.1	Bacillus phage Gamma isolate d'Herelle
1273743	GCA_002603065.1	Bacillus phage Gemini
307240	GCA_000859725.1	Bacillus phage GIL16c
1406785	GCA_000913015.1	Bacillus phage Grass
1486659	GCA_000922815.1	Bacillus phage Hakuna
1981931	GCA_002622645.1	Bacillus phage Harambe
2301683	GCA_003443095.1	Bacillus phage Hobo
2079258	GCA_002958355.1	Bacillus phage HonestAbe
1486660	GCA_000920895.1	Bacillus phage Hoody T
2024232	GCA_002627845.1	Bacillus phage Janet
1498212	GCA_001041155.1	Bacillus phage JBP901
1296655	GCA_001504395.1	Bacillus phage JL
2023949	GCA_002627305.1	Bacillus phage Juan
1805949	GCA_002604025.1	Bacillus phage Juglone
2301684	GCA_003443115.1	Bacillus phage Kamfam
1873998	GCA_001745675.1	Bacillus phage Kida
2301685	GCA_003443155.1	Bacillus phage Kioshi
2023950	GCA_002627325.1	Bacillus phage KonjoTrouble
1815973	GCA_002609545.1	Bacillus phage Leo2
1540090	GCA_002149325.1	Bacillus phage Mater
1486661	GCA_000920055.1	Bacillus phage Megatron
1309583	GCA_000910075.1	Bacillus phage MG-B1
1796993	GCA_002609385.1	Bacillus phage Mgbh1

1540091	GCA_002149605.1	Bacillus phage Moonbeam
1983579	GCA_002623825.1	Bacillus phage Negev_SA
1805950	GCA_001743755.1	Bacillus phage Nemo
10753	GCA_002755055.1	Bacillus phage Nf
1805951	GCA_001745075.1	Bacillus phage Nigalana
1805952	GCA_001745735.1	Bacillus phage NotTheCreek
2301686	GCA_003443135.1	Bacillus phage OmnioDeoPrimus
2024233	GCA_002627865.1	Bacillus phage OTooleKemple52
1406786	GCA_000912335.1	Bacillus phage Page
1597966	GCA_001503455.1	Bacillus phage Palmer
1540092	GCA_002149225.1	Bacillus phage Pascal
1675598	GCA_001501875.1	Bacillus phage Pavlov
1161901	GCA_000898195.1	Bacillus phage PBC1
1675029	GCA_002606985.1	Bacillus phage PBC2
1675028	GCA_002606965.1	Bacillus phage PBC4
1675030	GCA_002607005.1	Bacillus phage PBC5
1673872	GCA_002606845.1	Bacillus phage PBC6
2558359	GCA_004359175.1	Bacillus phage PBP180
1868598	GCA_002610955.1	Bacillus phage PfEFR-4
1868599	GCA_001746175.1	Bacillus phage PfEFR-5
1868600	GCA_002610985.1	Bacillus phage PfIS075
1868601	GCA_002611025.1	Bacillus phage PfNC7401
1075161	GCA_003047535.1	Bacillus phage pGIL01
1768917	GCA_002990995.1	Bacillus phage pGIL02
10717	GCA_000841105.1	Bacillus phage phi105
10717	GCA_002921615.1	Bacillus phage phi105
10736	GCA_002601445.1	Bacillus phage phi3T
1643324	GCA_002149765.1	Bacillus phage phi4B1
1643325	GCA_002602345.1	Bacillus phage phi4I1
1643326	GCA_002149545.1	Bacillus phage phi4J1
1204533	GCA_000905015.1	Bacillus phage phiAGATE
1357713	GCA_000916355.1	Bacillus phage phiCM3
207656	GCA_000908075.1	Bacillus phage phiNIT1
1124578	GCA_000902395.1	Bacillus phage phIS3501
1643327	GCA_002605725.1	Bacillus phage phiS58
1805953	GCA_001744415.1	Bacillus phage Phrodo
1873894	GCA_002611725.1	Bacillus phage PK16
547228	GCA_000907095.1	Bacillus phage PM1
1406789	GCA_000911355.1	Bacillus phage Pony
1540093	GCA_002149425.1	Bacillus phage Pookie
1406787	GCA_002604105.1	Bacillus phage poppyseed
2024234	GCA_002627885.1	Bacillus phage PPIsBest
1357712	GCA_002603985.1	Bacillus phage proCM3
2500559	GCA_004015565.1	Bacillus phage pW2
2500560	GCA_004015605.1	Bacillus phage pW4
10757	GCA_002755075.1	Bacillus phage PZA
1909400	GCA_002614325.1	Bacillus phage QCM11
1909403	GCA_002614385.1	Bacillus phage QCM8
2023951	GCA_002627345.1	Bacillus phage RadRaab
2315627	GCA_003613715.1	Bacillus phage Ray17

1486662	GCA_000926075.1	Bacillus phage Riley
2282402	GCA_003369285.1	Bacillus phage Saddex
1805954	GCA_001743735.1	Bacillus phage SageFayge
1837830	GCA_001744615.1	Bacillus phage SalinJah
1909401	GCA_002614345.1	Bacillus phage SBP8a
2045371	GCA_002990925.1	Bacillus phage SBSphiC
2045370	GCA_002990895.1	Bacillus phage SBSphiJ
1983413	GCA_002623485.1	Bacillus phage SerPounce
1296656	GCA_001505855.1	Bacillus phage Shanette
1796992	GCA_001736935.1	Bacillus phage Shbh1
1675599	GCA_002607045.1	Bacillus phage Silence
1406790	GCA_000913795.1	Bacillus phage Slash
1852566	GCA_002611885.1	Bacillus phage Smudge
941058	GCA_000903255.1	Bacillus phage SP-10
1792032	GCA_001754685.1	Bacillus phage SP-15
1497851	GCA_001736955.2	Bacillus phage SPG24
1497851	GCA_001736955.1	Bacillus phage SPG24
1406791	GCA_000913815.1	Bacillus phage Spock
10724	GCA_000847145.1	Bacillus phage SPP1
1610832	GCA_002149345.1	Bacillus phage Stahl
1406792	GCA_000913835.1	Bacillus phage Staley
2483852	GCA_003865615.1	Bacillus phage StevenHerd11
1610833	GCA_002149365.1	Bacillus phage Stills
1874002	GCA_001745655.1	Bacillus phage Stitch
2030094	GCA_002629685.1	Bacillus phage Taffo16
1983581	GCA_002623865.1	Bacillus phage Tavor_SA
565140	GCA_000880955.1	Bacillus phage TP21-L
1382932	GCA_000913375.1	Bacillus phage Troll
1690456	GCA_001504235.1	Bacillus phage TsarBomba
2094735	GCA_003570785.1	Bacillus phage v_B-Bak1
2094736	GCA_003570825.1	Bacillus phage v_B-Bak10
2094737	GCA_003570805.1	Bacillus phage v_B-Bak6
1308863	GCA_000915835.1	Bacillus phage vB_BanS-Tsamsa
2419618	GCA_003718835.1	Bacillus phage vB_BboS-125
1195072	GCA_000906215.1	Bacillus phage vB_BceM_Bc431v3
2170705	GCA_003288735.1	Bacillus phage vB_BceM-HSE3
2419619	GCA_003718815.1	Bacillus phage vB_BcoS-136
1775140	GCA_001736615.1	Bacillus phage vB_BhaS-171
2419620	GCA_003718915.1	Bacillus phage vB_BpsM-61
2419621	GCA_004011255.1	Bacillus phage vB_BpsS-140
2419622	GCA_003718895.1	Bacillus phage vB_BpsS-36
1739968	GCA_002607645.1	Bacillus phage vB_BpuM-BpSp
1933062	GCA_002618385.1	Bacillus phage vB_BsuM-Goe2
1933063	GCA_002618405.1	Bacillus phage vB_BsuM-Goe3
1807511	GCA_002601545.1	Bacillus phage vB_BsuP-Goe1
2491346	GCA_003865795.1	Bacillus phage vB_BthM-Goe5
2315470	GCA_003614635.1	Bacillus phage vB_BthP-Goe4
2498843	GCA_003640685.1	Bacillus phage vB_BthS_BMBphi
1868825	GCA_002611065.1	Bacillus phage vB_BtS_BMBtp13
1868826	GCA_002611085.1	Bacillus phage vB_BtS_BMBtp14

1868827	GCA_002611105.1	Bacillus phage vB_BtS_BMBtp15
1701848	GCA_002607425.1	Bacillus phage vB_BtS_BMBtp16
1445809	GCA_001501795.1	Bacillus phage vB_BtS_BMBtp3
2022474	GCA_002627245.1	Bacillus phage vB_BveP-Goe6
1805955	GCA_002604625.1	Bacillus phage Vinny
1734382	GCA_001505535.1	Bacillus phage VMY22
764595	GCA_000896595.1	Bacillus phage W.Ph.
1510440	GCA_000925695.1	Bacillus phage Waukesha92
2283012	GCA_003423425.1	Bacillus phage Wes44
663237	GCA_000911915.1	Bacillus phage Wip1
2024235	GCA_002627905.1	Bacillus phage Zainny
1805956	GCA_001745055.1	Bacillus phage Zuko
396034	GCA_000873445.1	Bacillus virus 1
701257	GCA_001500675.1	Bacillus virus 250
1273739	GCA_000904275.1	Bacillus virus Andromeda
513550	GCA_000881695.1	Bacillus virus AP50
10778	GCA_000840305.1	Bacillus virus B103
1406782	GCA_000912395.1	Bacillus virus Blastoid
1985178	GCA_002617825.1	Bacillus virus BM15
1236573	GCA_000904835.1	Bacillus virus BMBtp2
1273740	GCA_000905895.1	Bacillus virus Curly
1273741	GCA_000905295.1	Bacillus virus Eoghan
1273742	GCA_000906775.1	Bacillus virus Finn
1084719	GCA_000917535.1	Bacillus virus G
12345	GCA_000858305.1	Bacillus virus GA1
1406784	GCA_000912995.1	Bacillus virus Glittering
552525	GCA_000881435.1	Bacillus virus IEBH
10683	GCA_002601325.1	Bacillus virus PBS1
10756	GCA_000880275.1	Bacillus virus phi29
1406788	GCA_000912375.1	Bacillus virus Riggi
66797	GCA_000837685.1	Bacillus virus SPbeta
10685	GCA_000881675.1	Bacillus virus SPO1
1273744	GCA_002603085.1	Bacillus virus Taylor
359961	GCA_000864445.1	Bacillus virus Wbeta
1729935	GCA_900008085.1	Bacteriophage 15G
1729934	GCA_900016755.1	Bacteriophage 4A
340054	GCA_000882435.1	Bacteriophage APSE-2
1589751	GCA_001503475.1	Bacteriophage Lily
1589753	GCA_002605385.1	Bacteriophage Redbud
2024324	GCA_002955435.1	Bacteriophage T5-like chee130_1
2024327	GCA_002955465.1	Bacteriophage T5-like chee158
2024330	GCA_002955495.1	Bacteriophage T5-like chee24
2024328	GCA_002955475.1	Bacteriophage T5-like cott162
2024331	GCA_002955505.1	Bacteriophage T5-like pork27
2024332	GCA_002955515.1	Bacteriophage T5-like pork29
2024323	GCA_002955425.1	Bacteriophage T5-like poul124
2024326	GCA_002955455.1	Bacteriophage T5-like poul149
2024322	GCA_002955415.1	Bacteriophage T5-like saus111K
2024325	GCA_002955445.1	Bacteriophage T5-like saus132
2024329	GCA_002955485.1	Bacteriophage T5-like saus176N

2024333	GCA_002955525.1	Bacteriophage T5-like saus47N
1777122	GCA_002609105.1	Bacteriophage vB_NpeS-2AV2
1105171	GCA_000895315.1	Bacteroides phage B124-14
99179	GCA_000883035.1	Bacteroides phage B40-8
2301731	GCA_003442555.1	Bacteroides phage crAss001
1634484	GCA_000973355.1	Badger associated gemykibivirus 1
1980609	GCA_002814795.1	Badu phasivirus
64290	GCA_000883735.1	Bagaza virus
1622279	GCA_002118625.1	Bahig virus
1966007	GCA_002029575.1	Bakunsa virus
1986118	GCA_000924075.1	Ball python nidovirus 1
1972684	GCA_002366085.1	Balsa almendravirus
1972684	GCA_002815515.1	Balsa almendravirus
1805494	GCA_002004915.1	Bamaga virus
35286	GCA_000847825.1	Bamboo mosaic virus
190811	GCA_000850485.1	Bamboo mosaic virus satellite RNA
2305270	GCA_004040675.1	Bamboo rat circovirus
2305270	GCA_004040715.1	Bamboo rat circovirus
2305270	GCA_004040655.1	Bamboo rat circovirus
2305270	GCA_004040695.1	Bamboo rat circovirus
2305270	GCA_004040735.1	Bamboo rat circovirus
2305270	GCA_004040755.1	Bamboo rat circovirus
2305270	GCA_004040775.1	Bamboo rat circovirus
2305270	GCA_004040795.1	Bamboo rat circovirus
1229324	GCA_000917875.1	Baminivirus
45661	GCA_000871865.1	Banana bract mosaic virus
2169721	GCA_003029585.1	Banana bunchy top alphasatellite 1
2169722	GCA_003028905.1	Banana bunchy top alphasatellite 2
2169723	GCA_003028945.1	Banana bunchy top alphasatellite 3
12585	GCA_000847305.1	Banana bunchy top virus
12585	GCA_003124245.1	Banana bunchy top virus
12585	GCA_003124385.1	Banana bunchy top virus
12585	GCA_003124725.1	Banana bunchy top virus
12585	GCA_003124665.1	Banana bunchy top virus
12585	GCA_003124185.1	Banana bunchy top virus
12585	GCA_003124765.1	Banana bunchy top virus
12585	GCA_003124165.1	Banana bunchy top virus
12585	GCA_003124325.1	Banana bunchy top virus
12585	GCA_003124805.1	Banana bunchy top virus
12585	GCA_003124425.1	Banana bunchy top virus
12585	GCA_003124465.1	Banana bunchy top virus
12585	GCA_003124745.1	Banana bunchy top virus
12585	GCA_003124605.1	Banana bunchy top virus
12585	GCA_003124885.1	Banana bunchy top virus
12585	GCA_003124985.1	Banana bunchy top virus
12585	GCA_003124205.1	Banana bunchy top virus
12585	GCA_003124685.1	Banana bunchy top virus
12585	GCA_003124305.1	Banana bunchy top virus
12585	GCA_003124505.1	Banana bunchy top virus
12585	GCA_003125025.1	Banana bunchy top virus

12585	GCA_003124365.1	Banana bunchy top virus
12585	GCA_003125045.1	Banana bunchy top virus
12585	GCA_003124825.1	Banana bunchy top virus
12585	GCA_003124525.1	Banana bunchy top virus
12585	GCA_003124225.1	Banana bunchy top virus
12585	GCA_003124965.1	Banana bunchy top virus
12585	GCA_003124285.1	Banana bunchy top virus
12585	GCA_003124785.1	Banana bunchy top virus
12585	GCA_003124485.1	Banana bunchy top virus
12585	GCA_003124905.1	Banana bunchy top virus
12585	GCA_003124565.1	Banana bunchy top virus
12585	GCA_003124265.1	Banana bunchy top virus
12585	GCA_003124865.1	Banana bunchy top virus
12585	GCA_003125065.1	Banana bunchy top virus
12585	GCA_003125005.1	Banana bunchy top virus
12585	GCA_003124705.1	Banana bunchy top virus
12585	GCA_003124405.1	Banana bunchy top virus
12585	GCA_003124845.1	Banana bunchy top virus
12585	GCA_003124345.1	Banana bunchy top virus
12585	GCA_003124945.1	Banana bunchy top virus
12585	GCA_003124645.1	Banana bunchy top virus
12585	GCA_003124445.1	Banana bunchy top virus
12585	GCA_003124925.1	Banana bunchy top virus
12585	GCA_003124585.1	Banana bunchy top virus
12585	GCA_003124625.1	Banana bunchy top virus
12585	GCA_003124545.1	Banana bunchy top virus
12585	GCA_003125685.1	Banana bunchy top virus
12585	GCA_003126565.1	Banana bunchy top virus
12585	GCA_003125705.1	Banana bunchy top virus
12585	GCA_003125725.1	Banana bunchy top virus
12585	GCA_003125745.1	Banana bunchy top virus
12585	GCA_003125765.1	Banana bunchy top virus
12585	GCA_003125785.1	Banana bunchy top virus
12585	GCA_003125805.1	Banana bunchy top virus
12585	GCA_003125825.1	Banana bunchy top virus
12585	GCA_003125865.1	Banana bunchy top virus
12585	GCA_003125885.1	Banana bunchy top virus
12585	GCA_003125905.1	Banana bunchy top virus
12585	GCA_003125925.1	Banana bunchy top virus
12585	GCA_003125945.1	Banana bunchy top virus
12585	GCA_003125965.1	Banana bunchy top virus
12585	GCA_003125985.1	Banana bunchy top virus
12585	GCA_003126025.1	Banana bunchy top virus
12585	GCA_003126425.1	Banana bunchy top virus
12585	GCA_003126045.1	Banana bunchy top virus
12585	GCA_003125405.1	Banana bunchy top virus
12585	GCA_003126065.1	Banana bunchy top virus
12585	GCA_003126085.1	Banana bunchy top virus
12585	GCA_003126105.1	Banana bunchy top virus
12585	GCA_003126445.1	Banana bunchy top virus

12585	GCA_003126125.1	Banana bunchy top virus
12585	GCA_003125105.1	Banana bunchy top virus
12585	GCA_003126305.1	Banana bunchy top virus
12585	GCA_003126345.1	Banana bunchy top virus
12585	GCA_003126385.1	Banana bunchy top virus
12585	GCA_003126145.1	Banana bunchy top virus
12585	GCA_003126405.1	Banana bunchy top virus
12585	GCA_003126465.1	Banana bunchy top virus
12585	GCA_003126485.1	Banana bunchy top virus
12585	GCA_003126505.1	Banana bunchy top virus
12585	GCA_003126165.1	Banana bunchy top virus
12585	GCA_003126605.1	Banana bunchy top virus
12585	GCA_003126525.1	Banana bunchy top virus
12585	GCA_003126185.1	Banana bunchy top virus
12585	GCA_003125845.1	Banana bunchy top virus
12585	GCA_003125165.1	Banana bunchy top virus
12585	GCA_003126545.1	Banana bunchy top virus
12585	GCA_003126205.1	Banana bunchy top virus
12585	GCA_003125525.1	Banana bunchy top virus
12585	GCA_003126225.1	Banana bunchy top virus
12585	GCA_003126585.1	Banana bunchy top virus
12585	GCA_003126245.1	Banana bunchy top virus
12585	GCA_003126265.1	Banana bunchy top virus
12585	GCA_003125085.1	Banana bunchy top virus
12585	GCA_003125585.1	Banana bunchy top virus
12585	GCA_003125125.1	Banana bunchy top virus
12585	GCA_003125145.1	Banana bunchy top virus
12585	GCA_003126625.1	Banana bunchy top virus
12585	GCA_003125185.1	Banana bunchy top virus
12585	GCA_003126285.1	Banana bunchy top virus
12585	GCA_003125205.1	Banana bunchy top virus
12585	GCA_003125225.1	Banana bunchy top virus
12585	GCA_003125245.1	Banana bunchy top virus
12585	GCA_003125265.1	Banana bunchy top virus
12585	GCA_003125305.1	Banana bunchy top virus
12585	GCA_003125325.1	Banana bunchy top virus
12585	GCA_003125285.1	Banana bunchy top virus
12585	GCA_003125345.1	Banana bunchy top virus
12585	GCA_003125365.1	Banana bunchy top virus
12585	GCA_003125385.1	Banana bunchy top virus
12585	GCA_003125425.1	Banana bunchy top virus
12585	GCA_003126325.1	Banana bunchy top virus
12585	GCA_003125445.1	Banana bunchy top virus
12585	GCA_003125645.1	Banana bunchy top virus
12585	GCA_003125465.1	Banana bunchy top virus
12585	GCA_003125485.1	Banana bunchy top virus
12585	GCA_003125505.1	Banana bunchy top virus
12585	GCA_003125545.1	Banana bunchy top virus
12585	GCA_003126005.1	Banana bunchy top virus
12585	GCA_003125565.1	Banana bunchy top virus

12585	GCA_003125665.1	Banana bunchy top virus
12585	GCA_003125605.1	Banana bunchy top virus
12585	GCA_003125625.1	Banana bunchy top virus
12585	GCA_003126365.1	Banana bunchy top virus
148879	GCA_000848545.1	Banana mild mosaic virus
1016852	GCA_000891095.1	Banana streak CA virus
328670	GCA_000862625.1	Banana streak GF virus
1016853	GCA_000892735.1	Banana streak IM virus
1476909	GCA_000858465.1	Banana streak MY virus
328671	GCA_000857505.1	Banana streak OL virus
1016854	GCA_000891075.1	Banana streak UA virus
1016855	GCA_000892715.1	Banana streak UI virus
1016856	GCA_000892055.1	Banana streak UL virus
1016857	GCA_000893615.1	Banana streak UM virus
334778	GCA_000867185.1	Banana streak virus Acuminata Yunnan
1411991	GCA_000859245.1	Banana streak VN virus
479058	GCA_000872365.1	Bandicoot papillomatosis carcinomatosis virus type 1
500654	GCA_000880095.1	Bandicoot papillomatosis carcinomatosis virus type 2
2071566	GCA_002966195.1	Bandra megavirus
1737522	GCA_001430175.1	Bank vole polyomavirus
77763	GCA_000858185.1	Banna virus
38837	GCA_002820485.1	Banzi virus
2170132	GCA_001717275.1	Barbacena virus Y
759938	GCA_000892615.1	Barbel circovirus
759938	GCA_004085055.1	Barbel circovirus
43761	GCA_000884415.1	Barfin flounder nervous necrosis virus
546552	GCA_000880335.1	Barfin flounder virus BF93Hok
2293274	GCA_003847505.1	Bark beetle associated circular virus 1
2230896	GCA_003847685.1	Bark beetle-associated genomovirus 1
2230897	GCA_003847665.1	Bark beetle-associated genomovirus 2
2230898	GCA_003847645.1	Bark beetle-associated genomovirus 3
2230899	GCA_003847625.1	Bark beetle-associated genomovirus 4
2230900	GCA_003847605.1	Bark beetle-associated genomovirus 5
2230901	GCA_003847585.1	Bark beetle-associated genomovirus 6
12466	GCA_000862285.1	Barley mild mosaic virus
12327	GCA_000855725.1	Barley stripe mosaic virus
1825924	GCA_001630025.1	Barley virus G
31723	GCA_000850645.1	Barley yellow dwarf virus (ISOLATE MAV-PS1)
224578	GCA_000854205.1	Barley yellow dwarf virus GAV
2169987	GCA_000907695.1	Barley yellow dwarf virus kerII
2169985	GCA_000850165.1	Barley yellow dwarf virus PAS
2169986	GCA_000859045.1	Barley yellow dwarf virus PAV
12465	GCA_000860765.1	Barley yellow mosaic virus
1985699	GCA_001432155.1	Barley yellow striate mosaic cytorhabdovirus
11020	GCA_000847585.1	Barmah Forest virus
380438	GCA_002145665.1	Barur virus
1930507	GCA_002375015.1	Basavirus sp.
1930507	GCA_003727835.1	Basavirus sp.
1930507	GCA_003727815.1	Basavirus sp.
1930507	GCA_003727875.1	Basavirus sp.

1930507	GCA_003727895.1	Basavirus sp.
1930507	GCA_003727735.1	Basavirus sp.
1930507	GCA_003727755.1	Basavirus sp.
1930507	GCA_003728855.1	Basavirus sp.
1930507	GCA_003728875.1	Basavirus sp.
1930507	GCA_003728015.1	Basavirus sp.
1930507	GCA_003727995.1	Basavirus sp.
1930507	GCA_003727935.1	Basavirus sp.
1930507	GCA_003727955.1	Basavirus sp.
1930507	GCA_003727975.1	Basavirus sp.
1930507	GCA_003728035.1	Basavirus sp.
1930507	GCA_003727915.1	Basavirus sp.
1384490	GCA_002820405.1	Basella alba alphaendornavirus 1
373398	GCA_000874125.1	Basella rugose mosaic virus
1803394	GCA_002285025.1	Bastrovirus 7
1906172	GCA_002271045.1	Bastrovirus/VietNam/Bat/16715_78
1906173	GCA_001925475.1	Bastrovirus/VietNam/Porcine/17489_85
1906175	GCA_001926815.1	Bastrovirus/VietNam/Rat/16715_10
1906176	GCA_001925975.1	Bastrovirus/VietNam/Rat/16715_26
1906178	GCA_001925315.1	Bastrovirus/VietNam/Rat/16715_58
1906170	GCA_001925335.1	Bastrovirus-like_virus/VietNam/Bat/17819_21
727962	GCA_000888555.1	Bat adeno-associated virus YNM
696069	GCA_000893815.1	Bat adenovirus 2
727977	GCA_000894355.2	Bat adenovirus TJM
2486204	GCA_004036835.1	Bat associated circovirus
1868218	GCA_002819465.1	Bat associated circovirus 1
2560339	GCA_004049235.1	Bat associated circovirus 11
1868219	GCA_000908355.1	Bat associated circovirus 2
2003310	GCA_002819505.1	Bat associated circovirus 5
2003311	GCA_002819525.1	Bat associated circovirus 6
2003312	GCA_002819545.1	Bat associated circovirus 7
2003313	GCA_002819565.1	Bat associated circovirus 8
2169823	GCA_003033015.1	Bat associated circovirus 9
1987736	GCA_002819785.1	Bat associated cyclovirus 11
1987738	GCA_002819805.1	Bat associated cyclovirus 13
2050585	GCA_002819645.1	Bat associated cyclovirus 2
2050586	GCA_002819665.1	Bat associated cyclovirus 3
2050587	GCA_002819685.1	Bat associated cyclovirus 4
1987731	GCA_002819705.1	Bat associated cyclovirus 6
1987732	GCA_002819725.1	Bat associated cyclovirus 7
1987733	GCA_002819745.1	Bat associated cyclovirus 8
1987734	GCA_002819765.1	Bat associated cyclovirus 9
1958785	GCA_002008875.1	Bat badicivirus 1
1958786	GCA_002008675.1	Bat badicivirus 2
1329649	GCA_001564365.1	Bat bocavirus
2259810	GCA_003033255.1	Bat bocavirus WM40
2259811	GCA_003033265.1	Bat bocavirus XM30
2026815	GCA_004049255.1	Bat bug circovirus
1329650	GCA_002355085.1	Bat circovirus
1329650	GCA_004036235.1	Bat circovirus

1329650	GCA_004036255.1	Bat circovirus
1329650	GCA_004049275.1	Bat circovirus
1329650	GCA_004049295.1	Bat circovirus
1329650	GCA_004049315.1	Bat circovirus
1329650	GCA_004049335.1	Bat circovirus
1329650	GCA_004049395.1	Bat circovirus
1329650	GCA_004049415.1	Bat circovirus
1329650	GCA_004049355.1	Bat circovirus
1329650	GCA_004049375.1	Bat circovirus
1329650	GCA_004369505.1	Bat circovirus
1329650	GCA_004369345.1	Bat circovirus
1329650	GCA_004369565.1	Bat circovirus
1329650	GCA_004369365.1	Bat circovirus
1329650	GCA_004369385.1	Bat circovirus
1329650	GCA_004369685.1	Bat circovirus
1329650	GCA_004369605.1	Bat circovirus
1329650	GCA_004369325.1	Bat circovirus
1329650	GCA_004369425.1	Bat circovirus
1329650	GCA_004369465.1	Bat circovirus
1329650	GCA_004369405.1	Bat circovirus
1329650	GCA_004369645.1	Bat circovirus
1329650	GCA_004369445.1	Bat circovirus
1329650	GCA_004369585.1	Bat circovirus
1329650	GCA_004369485.1	Bat circovirus
1329650	GCA_004369525.1	Bat circovirus
1329650	GCA_004369545.1	Bat circovirus
1329650	GCA_004369625.1	Bat circovirus
1329650	GCA_004369665.1	Bat circovirus
1572236	GCA_004066075.1	Bat circovirus POA/2012/I
1572237	GCA_000930515.1	Bat circovirus POA/2012/II
1572238	GCA_004066055.1	Bat circovirus POA/2012/V
1572239	GCA_000929875.1	Bat circovirus POA/2012/VI
1072162	GCA_003985465.1	Bat circovirus ZS/China/2011
1072162	GCA_004369285.1	Bat circovirus ZS/China/2011
1072204	GCA_004369305.1	Bat circovirus ZS/Yunnan-China/2009
1508220	GCA_002118885.1	Bat coronavirus
864596	GCA_000887595.1	Bat coronavirus BM48-31/BGR/2008
1384461	GCA_000913415.1	Bat coronavirus CDPHE15/USA/2006
424359	GCA_000870505.1	Bat coronavirus HKU4-1
424363	GCA_000873025.1	Bat coronavirus HKU5-1
424367	GCA_000868045.1	Bat coronavirus HKU9-1
795381	GCA_002145985.1	Bat cyclovirus GF-4c
1958784	GCA_002008695.1	Bat dicibavirus
1325446	GCA_001882015.1	Bat hepacivirus
1745250	GCA_001443765.1	Bat hepatovirus
1987143	GCA_002817035.1	Bat hepatovirus BUO2BF86Colafr2010
1987139	GCA_002816915.1	Bat hepatovirus SMG18520Minmav2014
1541205	GCA_000926915.1	Bat Hp-betacoronavirus/Zhejiang2013
1958778	GCA_002008415.1	Bat iflavirus
2015376	GCA_001885425.1	Bat mastadenovirus G

1788432	GCA_001630065.1	Bat mastadenovirus WIV10
1788433	GCA_001630005.1	Bat mastadenovirus WIV11
1788434	GCA_001722965.1	Bat mastadenovirus WIV12
1788435	GCA_001722705.1	Bat mastadenovirus WIV13
1986505	GCA_002158575.1	Bat mastadenovirus WIV17
1986506	GCA_002366205.1	Bat mastadenovirus WIV18
1788436	GCA_001629825.1	Bat mastadenovirus WIV9
2301732	GCA_003260815.1	Bat Middle East Hepe-Astrovirus
1514709	GCA_003147425.1	Bat norovirus
1112631	GCA_000925295.1	Bat Paramyxovirus Eid_hel/GH-M74a/GHA/2009
1112597	GCA_002815275.1	Bat Paramyxovirus Epo_spe/AR1/DRC/2009
1074863	GCA_000893855.1	Bat picornavirus 1
1074864	GCA_000891335.1	Bat picornavirus 2
1074865	GCA_000892935.1	Bat picornavirus 3
2003500	GCA_002817245.1	Bat picornavirus BatPV/V1/13 Hun
2035815	GCA_000968995.1	Bat polyomanvirus 5b-1
1221637	GCA_004129735.1	Bat polyomavirus
1623687	GCA_000969095.1	Bat polyomavirus 5a
1623684	GCA_002827785.1	Bat polyomavirus 5b
1623685	GCA_000970605.1	Bat polyomavirus 6a
1623689	GCA_000969215.1	Bat polyomavirus 6b
1623688	GCA_000969035.1	Bat polyomavirus 6c
1623686	GCA_000969155.1	Bat polyomavirus 6d
1340801	GCA_004117615.1	Bat rotavirus
1958777	GCA_002008535.1	Bat sapelovirus
1959930	GCA_002005625.1	Bat sapovirus
1185359	GCA_000894635.1	Bat sapovirus TLC58/HK
1888311	GCA_001717375.1	Bat tymo-like virus
880159	GCA_000889515.1	Bathycoccus sp. RCC1105 virus BpV1
880160	GCA_003051645.1	Bathycoccus sp. RCC1105 virus BpV2
1980459	GCA_002816435.1	Bayou orthohantavirus
1035662	GCA_000892195.1	Bdellovibrio phage phi1402
1127515	GCA_000903295.1	Bdellovibrio phage phi1422
145579	GCA_000838865.1	Bdellovibrio phage phiMH2K
77856	GCA_000843965.1	Beak and feather disease virus
77856	GCA_004032875.1	Beak and feather disease virus
77856	GCA_004035815.1	Beak and feather disease virus
77856	GCA_004033535.1	Beak and feather disease virus
77856	GCA_004033615.1	Beak and feather disease virus
77856	GCA_004036015.1	Beak and feather disease virus
77856	GCA_004033635.1	Beak and feather disease virus
77856	GCA_004035795.1	Beak and feather disease virus
77856	GCA_004036095.1	Beak and feather disease virus
77856	GCA_004036155.1	Beak and feather disease virus
77856	GCA_004036195.1	Beak and feather disease virus
77856	GCA_004033435.1	Beak and feather disease virus
77856	GCA_004036175.1	Beak and feather disease virus
77856	GCA_004035895.1	Beak and feather disease virus
77856	GCA_004035835.1	Beak and feather disease virus
77856	GCA_004032895.1	Beak and feather disease virus

77856	GCA_004036035.1	Beak and feather disease virus
77856	GCA_004011295.1	Beak and feather disease virus
77856	GCA_004035855.1	Beak and feather disease virus
77856	GCA_004034015.1	Beak and feather disease virus
77856	GCA_004036115.1	Beak and feather disease virus
77856	GCA_004035875.1	Beak and feather disease virus
77856	GCA_004035975.1	Beak and feather disease virus
77856	GCA_004035915.1	Beak and feather disease virus
77856	GCA_004035995.1	Beak and feather disease virus
77856	GCA_004036055.1	Beak and feather disease virus
77856	GCA_004035955.1	Beak and feather disease virus
77856	GCA_004032855.1	Beak and feather disease virus
77856	GCA_004031375.1	Beak and feather disease virus
77856	GCA_004030695.1	Beak and feather disease virus
77856	GCA_004033595.1	Beak and feather disease virus
77856	GCA_004030755.1	Beak and feather disease virus
77856	GCA_004036135.1	Beak and feather disease virus
77856	GCA_004035935.1	Beak and feather disease virus
77856	GCA_004036075.1	Beak and feather disease virus
77856	GCA_004033335.1	Beak and feather disease virus
77856	GCA_004033315.1	Beak and feather disease virus
77856	GCA_004057155.1	Beak and feather disease virus
77856	GCA_004057115.1	Beak and feather disease virus
77856	GCA_004050435.1	Beak and feather disease virus
77856	GCA_004057295.1	Beak and feather disease virus
77856	GCA_004055795.1	Beak and feather disease virus
77856	GCA_004056955.1	Beak and feather disease virus
77856	GCA_004057435.1	Beak and feather disease virus
77856	GCA_004055935.1	Beak and feather disease virus
77856	GCA_004057095.1	Beak and feather disease virus
77856	GCA_004054915.1	Beak and feather disease virus
77856	GCA_004057575.1	Beak and feather disease virus
77856	GCA_004055835.1	Beak and feather disease virus
77856	GCA_004056895.1	Beak and feather disease virus
77856	GCA_004050195.1	Beak and feather disease virus
77856	GCA_004055875.1	Beak and feather disease virus
77856	GCA_004050235.1	Beak and feather disease virus
77856	GCA_004057535.1	Beak and feather disease virus
77856	GCA_004059695.1	Beak and feather disease virus
77856	GCA_004050275.1	Beak and feather disease virus
77856	GCA_004058455.1	Beak and feather disease virus
77856	GCA_004050315.1	Beak and feather disease virus
77856	GCA_004061635.1	Beak and feather disease virus
77856	GCA_004056015.1	Beak and feather disease virus
77856	GCA_004050335.1	Beak and feather disease virus
77856	GCA_004057175.1	Beak and feather disease virus
77856	GCA_004055755.1	Beak and feather disease virus
77856	GCA_004050535.1	Beak and feather disease virus
77856	GCA_004050415.1	Beak and feather disease virus
77856	GCA_004053495.1	Beak and feather disease virus

77856	GCA_004050455.1	Beak and feather disease virus
77856	GCA_004057315.1	Beak and feather disease virus
77856	GCA_004055815.1	Beak and feather disease virus
77856	GCA_004050475.1	Beak and feather disease virus
77856	GCA_004058475.1	Beak and feather disease virus
77856	GCA_004050495.1	Beak and feather disease virus
77856	GCA_004059635.1	Beak and feather disease virus
77856	GCA_004057235.1	Beak and feather disease virus
77856	GCA_004050515.1	Beak and feather disease virus
77856	GCA_004061995.1	Beak and feather disease virus
77856	GCA_004050215.1	Beak and feather disease virus
77856	GCA_004055715.1	Beak and feather disease virus
77856	GCA_004057455.1	Beak and feather disease virus
77856	GCA_004056995.1	Beak and feather disease virus
77856	GCA_004055975.1	Beak and feather disease virus
77856	GCA_004057595.1	Beak and feather disease virus
77856	GCA_004058415.1	Beak and feather disease virus
77856	GCA_004056915.1	Beak and feather disease virus
77856	GCA_004050255.1	Beak and feather disease virus
77856	GCA_004058395.1	Beak and feather disease virus
77856	GCA_004057395.1	Beak and feather disease virus
77856	GCA_004055895.1	Beak and feather disease virus
77856	GCA_004057055.1	Beak and feather disease virus
77856	GCA_004059715.1	Beak and feather disease virus
77856	GCA_004057515.1	Beak and feather disease virus
77856	GCA_004054875.1	Beak and feather disease virus
77856	GCA_004057015.1	Beak and feather disease virus
77856	GCA_004056035.1	Beak and feather disease virus
77856	GCA_004057615.1	Beak and feather disease virus
77856	GCA_004057195.1	Beak and feather disease virus
77856	GCA_004057375.1	Beak and feather disease virus
77856	GCA_004061595.1	Beak and feather disease virus
77856	GCA_004050295.1	Beak and feather disease virus
77856	GCA_004057655.1	Beak and feather disease virus
77856	GCA_004057635.1	Beak and feather disease virus
77856	GCA_004057335.1	Beak and feather disease virus
77856	GCA_004058495.1	Beak and feather disease virus
77856	GCA_004059655.1	Beak and feather disease virus
77856	GCA_004058515.1	Beak and feather disease virus
77856	GCA_004055995.1	Beak and feather disease virus
77856	GCA_004057475.1	Beak and feather disease virus
77856	GCA_004058635.1	Beak and feather disease virus
77856	GCA_004057135.1	Beak and feather disease virus
77856	GCA_004059675.1	Beak and feather disease virus
77856	GCA_004045155.1	Beak and feather disease virus
77856	GCA_004055775.1	Beak and feather disease virus
77856	GCA_004045175.1	Beak and feather disease virus
77856	GCA_004058435.1	Beak and feather disease virus
77856	GCA_004056935.1	Beak and feather disease virus
77856	GCA_004045195.1	Beak and feather disease virus

77856	GCA_004045215.1	Beak and feather disease virus
77856	GCA_004050355.1	Beak and feather disease virus
77856	GCA_004045235.1	Beak and feather disease virus
77856	GCA_004045255.1	Beak and feather disease virus
77856	GCA_004056975.1	Beak and feather disease virus
77856	GCA_004057415.1	Beak and feather disease virus
77856	GCA_004045275.1	Beak and feather disease virus
77856	GCA_004055915.1	Beak and feather disease virus
77856	GCA_004057075.1	Beak and feather disease virus
77856	GCA_004055735.1	Beak and feather disease virus
77856	GCA_004050375.1	Beak and feather disease virus
77856	GCA_004054895.1	Beak and feather disease virus
77856	GCA_004057555.1	Beak and feather disease virus
77856	GCA_004056055.1	Beak and feather disease virus
77856	GCA_004057215.1	Beak and feather disease virus
77856	GCA_004058375.1	Beak and feather disease virus
77856	GCA_004050395.1	Beak and feather disease virus
77856	GCA_004057275.1	Beak and feather disease virus
77856	GCA_004057355.1	Beak and feather disease virus
77856	GCA_004057495.1	Beak and feather disease virus
77856	GCA_004055855.1	Beak and feather disease virus
77856	GCA_004057035.1	Beak and feather disease virus
77856	GCA_004061615.1	Beak and feather disease virus
77856	GCA_004057255.1	Beak and feather disease virus
77856	GCA_004055955.1	Beak and feather disease virus
77856	GCA_004061655.1	Beak and feather disease virus
77856	GCA_004068175.1	Beak and feather disease virus
77856	GCA_004079455.1	Beak and feather disease virus
77856	GCA_004068195.1	Beak and feather disease virus
77856	GCA_004080615.1	Beak and feather disease virus
77856	GCA_004079275.1	Beak and feather disease virus
77856	GCA_004079115.1	Beak and feather disease virus
77856	GCA_004068215.1	Beak and feather disease virus
77856	GCA_004076715.1	Beak and feather disease virus
77856	GCA_004080915.1	Beak and feather disease virus
77856	GCA_004076375.1	Beak and feather disease virus
77856	GCA_004074375.1	Beak and feather disease virus
77856	GCA_004068235.1	Beak and feather disease virus
77856	GCA_004074335.1	Beak and feather disease virus
77856	GCA_004085915.1	Beak and feather disease virus
77856	GCA_004074435.1	Beak and feather disease virus
77856	GCA_004080755.1	Beak and feather disease virus
77856	GCA_004086075.1	Beak and feather disease virus
77856	GCA_004076735.1	Beak and feather disease virus
77856	GCA_004076395.1	Beak and feather disease virus
77856	GCA_004085935.1	Beak and feather disease virus
77856	GCA_004074515.1	Beak and feather disease virus
77856	GCA_004080275.1	Beak and feather disease virus
77856	GCA_004074355.1	Beak and feather disease virus
77856	GCA_004079135.1	Beak and feather disease virus

77856	GCA_004079375.1	Beak and feather disease virus
77856	GCA_004063435.1	Beak and feather disease virus
77856	GCA_004074555.1	Beak and feather disease virus
77856	GCA_004080895.1	Beak and feather disease virus
77856	GCA_004072995.1	Beak and feather disease virus
77856	GCA_004068435.1	Beak and feather disease virus
77856	GCA_004074595.1	Beak and feather disease virus
77856	GCA_004068455.1	Beak and feather disease virus
77856	GCA_004076755.1	Beak and feather disease virus
77856	GCA_004079055.1	Beak and feather disease virus
77856	GCA_004076415.1	Beak and feather disease virus
77856	GCA_004085875.1	Beak and feather disease virus
77856	GCA_004074615.1	Beak and feather disease virus
77856	GCA_004068475.1	Beak and feather disease virus
77856	GCA_004086195.1	Beak and feather disease virus
77856	GCA_004068495.1	Beak and feather disease virus
77856	GCA_004068515.1	Beak and feather disease virus
77856	GCA_004063455.1	Beak and feather disease virus
77856	GCA_004068535.1	Beak and feather disease virus
77856	GCA_004068555.1	Beak and feather disease virus
77856	GCA_004079195.1	Beak and feather disease virus
77856	GCA_004068575.1	Beak and feather disease virus
77856	GCA_004076775.1	Beak and feather disease virus
77856	GCA_004086015.1	Beak and feather disease virus
77856	GCA_004076435.1	Beak and feather disease virus
77856	GCA_004086035.1	Beak and feather disease virus
77856	GCA_004068595.1	Beak and feather disease virus
77856	GCA_004068615.1	Beak and feather disease virus
77856	GCA_004074395.1	Beak and feather disease virus
77856	GCA_004080795.1	Beak and feather disease virus
77856	GCA_004068635.1	Beak and feather disease virus
77856	GCA_004068655.1	Beak and feather disease virus
77856	GCA_004079335.1	Beak and feather disease virus
77856	GCA_004068675.1	Beak and feather disease virus
77856	GCA_004079295.1	Beak and feather disease virus
77856	GCA_004086155.1	Beak and feather disease virus
77856	GCA_004080495.1	Beak and feather disease virus
77856	GCA_004068695.1	Beak and feather disease virus
77856	GCA_004076795.1	Beak and feather disease virus
77856	GCA_004076455.1	Beak and feather disease virus
77856	GCA_004068715.1	Beak and feather disease virus
77856	GCA_004068735.1	Beak and feather disease virus
77856	GCA_004074415.1	Beak and feather disease virus
77856	GCA_004068755.1	Beak and feather disease virus
77856	GCA_004063495.1	Beak and feather disease virus
77856	GCA_004068775.1	Beak and feather disease virus
77856	GCA_004079475.1	Beak and feather disease virus
77856	GCA_004068795.1	Beak and feather disease virus
77856	GCA_004080635.1	Beak and feather disease virus
77856	GCA_004079555.1	Beak and feather disease virus

77856	GCA_004068815.1	Beak and feather disease virus
77856	GCA_004076815.1	Beak and feather disease virus
77856	GCA_004076475.1	Beak and feather disease virus
77856	GCA_004068835.1	Beak and feather disease virus
77856	GCA_004068855.1	Beak and feather disease virus
77856	GCA_004068875.1	Beak and feather disease virus
77856	GCA_004068895.1	Beak and feather disease virus
77856	GCA_004073075.1	Beak and feather disease virus
77856	GCA_004080775.1	Beak and feather disease virus
77856	GCA_004068915.1	Beak and feather disease virus
77856	GCA_004073015.1	Beak and feather disease virus
77856	GCA_004086095.1	Beak and feather disease virus
77856	GCA_004068935.1	Beak and feather disease virus
77856	GCA_004076835.1	Beak and feather disease virus
77856	GCA_004076495.1	Beak and feather disease virus
77856	GCA_004079515.1	Beak and feather disease virus
77856	GCA_004074455.1	Beak and feather disease virus
77856	GCA_004073095.1	Beak and feather disease virus
77856	GCA_004073115.1	Beak and feather disease virus
77856	GCA_004079415.1	Beak and feather disease virus
77856	GCA_004073135.1	Beak and feather disease virus
77856	GCA_004086235.1	Beak and feather disease virus
77856	GCA_004080675.1	Beak and feather disease virus
77856	GCA_004076855.1	Beak and feather disease virus
77856	GCA_004079075.1	Beak and feather disease virus
77856	GCA_004076515.1	Beak and feather disease virus
77856	GCA_004085895.1	Beak and feather disease virus
77856	GCA_004076175.1	Beak and feather disease virus
77856	GCA_004074475.1	Beak and feather disease virus
77856	GCA_004073215.1	Beak and feather disease virus
77856	GCA_004079215.1	Beak and feather disease virus
77856	GCA_004080935.1	Beak and feather disease virus
77856	GCA_004076875.1	Beak and feather disease virus
77856	GCA_004076535.1	Beak and feather disease virus
77856	GCA_004074495.1	Beak and feather disease virus
77856	GCA_004069255.1	Beak and feather disease virus
77856	GCA_004086055.1	Beak and feather disease virus
77856	GCA_004080855.1	Beak and feather disease virus
77856	GCA_004079355.1	Beak and feather disease virus
77856	GCA_004069275.1	Beak and feather disease virus
77856	GCA_004080515.1	Beak and feather disease virus
77856	GCA_004069295.1	Beak and feather disease virus
77856	GCA_004076895.1	Beak and feather disease virus
77856	GCA_004085955.1	Beak and feather disease virus
77856	GCA_004076555.1	Beak and feather disease virus
77856	GCA_004069315.1	Beak and feather disease virus
77856	GCA_004085835.1	Beak and feather disease virus
77856	GCA_004069335.1	Beak and feather disease virus
77856	GCA_004069355.1	Beak and feather disease virus
77856	GCA_004079395.1	Beak and feather disease virus

77856	GCA_004080815.1	Beak and feather disease virus
77856	GCA_004069375.1	Beak and feather disease virus
77856	GCA_004079495.1	Beak and feather disease virus
77856	GCA_004073155.1	Beak and feather disease virus
77856	GCA_004080575.1	Beak and feather disease virus
77856	GCA_004069395.1	Beak and feather disease virus
77856	GCA_004079155.1	Beak and feather disease virus
77856	GCA_004069415.1	Beak and feather disease virus
77856	GCA_004076575.1	Beak and feather disease virus
77856	GCA_004085975.1	Beak and feather disease virus
77856	GCA_004086215.1	Beak and feather disease virus
77856	GCA_004069435.1	Beak and feather disease virus
77856	GCA_004086175.1	Beak and feather disease virus
77856	GCA_004069455.1	Beak and feather disease virus
77856	GCA_004080555.1	Beak and feather disease virus
77856	GCA_004063315.1	Beak and feather disease virus
77856	GCA_004074535.1	Beak and feather disease virus
77856	GCA_004069475.1	Beak and feather disease virus
77856	GCA_004063335.1	Beak and feather disease virus
77856	GCA_004069495.1	Beak and feather disease virus
77856	GCA_004073175.1	Beak and feather disease virus
77856	GCA_004069515.1	Beak and feather disease virus
77856	GCA_004086115.1	Beak and feather disease virus
77856	GCA_004069535.1	Beak and feather disease virus
77856	GCA_004076595.1	Beak and feather disease virus
77856	GCA_004069555.1	Beak and feather disease virus
77856	GCA_004063415.1	Beak and feather disease virus
77856	GCA_004069575.1	Beak and feather disease virus
77856	GCA_004079255.1	Beak and feather disease virus
77856	GCA_004069595.1	Beak and feather disease virus
77856	GCA_004069615.1	Beak and feather disease virus
77856	GCA_004063475.1	Beak and feather disease virus
77856	GCA_004073195.1	Beak and feather disease virus
77856	GCA_004079435.1	Beak and feather disease virus
77856	GCA_004069635.1	Beak and feather disease virus
77856	GCA_004086255.1	Beak and feather disease virus
77856	GCA_004080595.1	Beak and feather disease virus
77856	GCA_004069655.1	Beak and feather disease virus
77856	GCA_004079095.1	Beak and feather disease virus
77856	GCA_004063525.1	Beak and feather disease virus
77856	GCA_004076615.1	Beak and feather disease virus
77856	GCA_004069675.1	Beak and feather disease virus
77856	GCA_004080255.1	Beak and feather disease virus
77856	GCA_004069695.1	Beak and feather disease virus
77856	GCA_004086135.1	Beak and feather disease virus
77856	GCA_004074575.1	Beak and feather disease virus
77856	GCA_004069715.1	Beak and feather disease virus
77856	GCA_004069735.1	Beak and feather disease virus
77856	GCA_004069755.1	Beak and feather disease virus
77856	GCA_004079235.1	Beak and feather disease virus

77856	GCA_004069775.1	Beak and feather disease virus
77856	GCA_004076635.1	Beak and feather disease virus
77856	GCA_004069795.1	Beak and feather disease virus
77856	GCA_004069815.1	Beak and feather disease virus
77856	GCA_004069835.1	Beak and feather disease virus
77856	GCA_004079035.1	Beak and feather disease virus
77856	GCA_004069855.1	Beak and feather disease virus
77856	GCA_004073235.1	Beak and feather disease virus
77856	GCA_004080875.1	Beak and feather disease virus
77856	GCA_004069875.1	Beak and feather disease virus
77856	GCA_004069895.1	Beak and feather disease virus
77856	GCA_004079535.1	Beak and feather disease virus
77856	GCA_004076655.1	Beak and feather disease virus
77856	GCA_004076055.1	Beak and feather disease virus
77856	GCA_004069915.1	Beak and feather disease virus
77856	GCA_004085855.1	Beak and feather disease virus
77856	GCA_004076075.1	Beak and feather disease virus
77856	GCA_004069935.1	Beak and feather disease virus
77856	GCA_004076095.1	Beak and feather disease virus
77856	GCA_004069955.1	Beak and feather disease virus
77856	GCA_004076115.1	Beak and feather disease virus
77856	GCA_004069975.1	Beak and feather disease virus
77856	GCA_004063355.1	Beak and feather disease virus
77856	GCA_004076135.1	Beak and feather disease virus
77856	GCA_004069995.1	Beak and feather disease virus
77856	GCA_004076155.1	Beak and feather disease virus
77856	GCA_004079175.1	Beak and feather disease virus
77856	GCA_004076675.1	Beak and feather disease virus
77856	GCA_004085995.1	Beak and feather disease virus
77856	GCA_004080835.1	Beak and feather disease virus
77856	GCA_004076195.1	Beak and feather disease virus
77856	GCA_004076215.1	Beak and feather disease virus
77856	GCA_004080535.1	Beak and feather disease virus
77856	GCA_004076235.1	Beak and feather disease virus
77856	GCA_004063375.1	Beak and feather disease virus
77856	GCA_004068055.1	Beak and feather disease virus
77856	GCA_004076255.1	Beak and feather disease virus
77856	GCA_004079315.1	Beak and feather disease virus
77856	GCA_004068075.1	Beak and feather disease virus
77856	GCA_004076275.1	Beak and feather disease virus
77856	GCA_004080475.1	Beak and feather disease virus
77856	GCA_004080655.1	Beak and feather disease virus
77856	GCA_004068095.1	Beak and feather disease virus
77856	GCA_004076695.1	Beak and feather disease virus
77856	GCA_004076295.1	Beak and feather disease virus
77856	GCA_004068115.1	Beak and feather disease virus
77856	GCA_004076315.1	Beak and feather disease virus
77856	GCA_004068135.1	Beak and feather disease virus
77856	GCA_004076335.1	Beak and feather disease virus
77856	GCA_004068155.1	Beak and feather disease virus

77856	GCA_004076355.1	Beak and feather disease virus
77856	GCA_004063395.1	Beak and feather disease virus
77856	GCA_004074315.1	Beak and feather disease virus
77856	GCA_004092755.1	Beak and feather disease virus
77856	GCA_004088695.1	Beak and feather disease virus
77856	GCA_004097235.1	Beak and feather disease virus
77856	GCA_004098955.1	Beak and feather disease virus
77856	GCA_004097255.1	Beak and feather disease virus
77856	GCA_004089155.1	Beak and feather disease virus
77856	GCA_004089195.1	Beak and feather disease virus
77856	GCA_004089215.1	Beak and feather disease virus
77856	GCA_004089235.1	Beak and feather disease virus
77856	GCA_004093535.1	Beak and feather disease virus
77856	GCA_004093555.1	Beak and feather disease virus
77856	GCA_004093575.1	Beak and feather disease virus
77856	GCA_004088115.1	Beak and feather disease virus
77856	GCA_004093655.1	Beak and feather disease virus
77856	GCA_004093675.1	Beak and feather disease virus
77856	GCA_004093695.1	Beak and feather disease virus
77856	GCA_004093715.1	Beak and feather disease virus
77856	GCA_004088135.1	Beak and feather disease virus
77856	GCA_004093735.1	Beak and feather disease virus
77856	GCA_004093755.1	Beak and feather disease virus
77856	GCA_004093775.1	Beak and feather disease virus
77856	GCA_004095835.1	Beak and feather disease virus
77856	GCA_004093795.1	Beak and feather disease virus
77856	GCA_004095855.1	Beak and feather disease virus
77856	GCA_004093815.1	Beak and feather disease virus
77856	GCA_004089175.1	Beak and feather disease virus
77856	GCA_004095875.1	Beak and feather disease virus
77856	GCA_004093835.1	Beak and feather disease virus
77856	GCA_004095895.1	Beak and feather disease virus
77856	GCA_004088175.1	Beak and feather disease virus
77856	GCA_004093855.1	Beak and feather disease virus
77856	GCA_004095915.1	Beak and feather disease virus
77856	GCA_004093875.1	Beak and feather disease virus
77856	GCA_004088555.1	Beak and feather disease virus
77856	GCA_004088215.1	Beak and feather disease virus
77856	GCA_004088195.1	Beak and feather disease virus
77856	GCA_004088595.1	Beak and feather disease virus
77856	GCA_004092575.1	Beak and feather disease virus
77856	GCA_004092595.1	Beak and feather disease virus
77856	GCA_004088515.1	Beak and feather disease virus
77856	GCA_004092615.1	Beak and feather disease virus
77856	GCA_004088535.1	Beak and feather disease virus
77856	GCA_004092635.1	Beak and feather disease virus
77856	GCA_004092655.1	Beak and feather disease virus
77856	GCA_004088575.1	Beak and feather disease virus
77856	GCA_004092675.1	Beak and feather disease virus
77856	GCA_004092695.1	Beak and feather disease virus

77856	GCA_004088615.1	Beak and feather disease virus
77856	GCA_004092715.1	Beak and feather disease virus
77856	GCA_004092735.1	Beak and feather disease virus
67082	GCA_001907825.1	BeAn 58058 virus
31602	GCA_000840005.1	Bean calico mosaic virus
1227354	GCA_000902035.1	Bean chlorosis virus
43240	GCA_000861385.1	Bean common mosaic necrosis virus
12196	GCA_000857245.1	Bean common mosaic virus
12196	GCA_002828265.1	Bean common mosaic virus
10838	GCA_000838545.1	Bean dwarf mosaic virus
10839	GCA_002821705.1	Bean golden mosaic virus
222444	GCA_000841845.1	Bean golden mosaic virus-[Brazil]
222445	GCA_002867405.1	Bean golden yellow mosaic virus-[Dominican Republic]
222449	GCA_000840225.1	Bean golden yellow mosaic virus-[Puerto Rico-Japan]
439420	GCA_002986445.1	Bean leaf curl Madagascar virus
12041	GCA_000850345.1	Bean leafroll virus
1033976	GCA_000897275.1	Bean necrotic mosaic virus
12260	GCA_000862925.1	Bean pod mottle virus
128790	GCA_001430295.1	Bean rugose mosaic virus
2169732	GCA_000910695.1	Bean white chlorosis mosaic virus
267970	GCA_000875065.1	Bean yellow disorder virus
57119	GCA_000849725.1	Bean yellow dwarf virus
714310	GCA_000893575.1	Bean yellow mosaic Mexico virus
12197	GCA_000863145.1	Bean yellow mosaic virus
192848	GCA_000874845.1	Bear Canyon mammarenavirus
1670662	GCA_001045385.1	Bearded dragon parvovirus
1819301	GCA_003032725.1	Beatrice Hill virus
1740646	GCA_002080235.1	Beauveria bassiana polymycovirus 1
1054649	GCA_001045305.1	Beauveria bassiana RNA virus 1
1054649	GCA_003956645.1	Beauveria bassiana RNA virus 1
1685109	GCA_002988055.1	Beauveria bassiana victorivirus 1
1485922	GCA_000922795.1	Beauveria bassiana victorivirus NZL/1980
59305	GCA_000894395.1	Bebaru virus
1682186	GCA_001190355.1	Bee Macula-like virus
2094260	GCA_004132205.1	Bee Macula-Like virus 2
196375	GCA_000855285.1	Beet black scorch virus
298965	GCA_000849805.1	Beet black scorch virus satellite RNA
131082	GCA_000847745.1	Beet chlorosis virus
509923	GCA_000883355.1	Beet cryptic virus 1
912029	GCA_002868515.1	Beet cryptic virus 2
391228	GCA_002821425.1	Beet curly top Iran virus
391228	GCA_002821445.1	Beet curly top Iran virus
391228	GCA_002821465.1	Beet curly top Iran virus
501371	GCA_000879795.1	Beet curly top Iran virus-[K]
10840	GCA_002824885.1	Beet curly top virus
10840	GCA_002824785.1	Beet curly top virus
10840	GCA_002824825.1	Beet curly top virus
10840	GCA_002824845.1	Beet curly top virus
10840	GCA_002824905.1	Beet curly top virus
268960	GCA_000843505.1	Beet curly top virus - California [Logan]

268956	GCA_002824865.1	Beet mild curly top virus - [Worland]
156690	GCA_000857745.1	Beet mild yellowing virus
114921	GCA_000854465.1	Beet mosaic virus
31721	GCA_000854885.1	Beet necrotic yellow vein virus
72750	GCA_000853445.1	Beet pseudoyellows virus
191547	GCA_000860405.1	Beet ringspot virus
268958	GCA_002824805.1	Beet severe curly top virus - Cfh
76343	GCA_000850425.1	Beet soil-borne mosaic virus
76343	GCA_002867265.1	Beet soil-borne mosaic virus
46436	GCA_000851945.1	Beet soil-borne virus
71972	GCA_000855145.1	Beet virus Q
198589	GCA_000851905.1	Beet western yellows ST9 associated virus
12042	GCA_000852565.1	Beet western yellows virus
1425367	GCA_003071245.1	Beet western yellows virus associated RNA
12161	GCA_000860605.1	Beet yellows virus
302069	GCA_000858105.1	Begomovirus-associated DNA-II
302070	GCA_000857145.1	Begomovirus-associated DNA-III
1922352	GCA_001925395.1	Beihai anemone virus 1
1922353	GCA_001925295.1	Beihai astro-like virus
1922354	GCA_001925435.1	Beihai barnacle virus 10
1922355	GCA_001926775.1	Beihai barnacle virus 11
1922356	GCA_001925375.1	Beihai barnacle virus 12
1922357	GCA_001921375.1	Beihai barnacle virus 13
1922359	GCA_001925275.1	Beihai barnacle virus 15
1922360	GCA_001925415.1	Beihai barnacle virus 2
1922361	GCA_001926755.1	Beihai barnacle virus 3
1922362	GCA_001925715.1	Beihai barnacle virus 4
1922365	GCA_001926475.1	Beihai barnacle virus 7
1922366	GCA_001926975.1	Beihai barnacle virus 8
1922367	GCA_001926135.1	Beihai barnacle virus 9
1746057	GCA_001443865.1	Beihai barnacle viurs 1
1922368	GCA_001925695.1	Beihai blue swimmer crab virus 1
1922370	GCA_001926455.1	Beihai blue swimmer crab virus 3
1922376	GCA_001926955.1	Beihai charybdis crab virus 1
1922379	GCA_001926435.1	Beihai echinoderm virus 1
1922381	GCA_001926935.1	Beihai hepe-like virus 10
1922387	GCA_001921475.1	Beihai hepe-like virus 4
1922385	GCA_001926095.1	Beihai hepe-like virus 8
1922386	GCA_001925655.1	Beihai hepe-like virus 9
1922388	GCA_001926415.1	Beihai hermit crab virus 1
1922390	GCA_001926915.1	Beihai hermit crab virus 3
1922392	GCA_001925635.1	Beihai horseshoe crab virus 1
1922393	GCA_001925555.1	Beihai hypo-like virus 1
1922428	GCA_001926895.1	Beihai mantis shrimp virus 1
1922429	GCA_001926055.1	Beihai mantis shrimp virus 2
1922430	GCA_001925615.1	Beihai mantis shrimp virus 3
1922431	GCA_001925535.1	Beihai mantis shrimp virus 4
1922432	GCA_001926875.1	Beihai mantis shrimp virus 5
1922433	GCA_001926035.1	Beihai mantis shrimp virus 6
1922434	GCA_001921455.1	Beihai mollusks virus 1

1922435	GCA_001925595.1	Beihai mollusks virus 2
1922436	GCA_001925515.1	Beihai narna-like virus 1
1922437	GCA_001926855.1	Beihai narna-like virus 10
1922438	GCA_001926015.1	Beihai narna-like virus 11
1922439	GCA_001925575.1	Beihai narna-like virus 12
1922440	GCA_001925495.1	Beihai narna-like virus 13
1922441	GCA_001926295.1	Beihai narna-like virus 14
1922442	GCA_001925855.1	Beihai narna-like virus 15
1922443	GCA_001926615.1	Beihai narna-like virus 16
1922444	GCA_001927115.1	Beihai narna-like virus 17
1922445	GCA_001926275.1	Beihai narna-like virus 18
1922446	GCA_001925835.1	Beihai narna-like virus 19
1922447	GCA_001926595.1	Beihai narna-like virus 2
1922448	GCA_001927095.1	Beihai narna-like virus 20
1922449	GCA_001926255.1	Beihai narna-like virus 21
1922450	GCA_001925355.1	Beihai narna-like virus 22
1922451	GCA_001925815.1	Beihai narna-like virus 23
1922452	GCA_001926575.1	Beihai narna-like virus 24
1922454	GCA_001927075.1	Beihai narna-like virus 26
1922455	GCA_001926235.1	Beihai narna-like virus 3
1922456	GCA_001925795.1	Beihai narna-like virus 4
1922458	GCA_001926555.1	Beihai narna-like virus 6
1922460	GCA_001927055.1	Beihai narna-like virus 8
1922461	GCA_001926215.1	Beihai narna-like virus 9
1922351	GCA_001926795.1	Beihai Nido-like virus 2
1922464	GCA_001925775.1	Beihai noda-like virus 11
1922471	GCA_001923575.1	Beihai noda-like virus 18
1922483	GCA_001924455.1	Beihai noda-like virus 29
1922487	GCA_001924915.1	Beihai noda-like virus 5
1922492	GCA_001926535.1	Beihai octopus virus 1
1922493	GCA_001927035.1	Beihai octopus virus 2
1922496	GCA_001926195.1	Beihai paphia shell virus 1
1922497	GCA_001925755.1	Beihai paphia shell virus 2
1922498	GCA_001926515.1	Beihai paphia shell virus 3
1922499	GCA_001927015.1	Beihai paphia shell virus 4
1922504	GCA_001926175.1	Beihai partiti-like virus 2
1922513	GCA_001921275.1	Beihai permutotetra-like virus 2
1922524	GCA_001925735.1	Beihai picobirna-like virus 7
1922525	GCA_001926495.1	Beihai picobirna-like virus 8
1922527	GCA_001935065.1	Beihai picorna-like virus 1
1922528	GCA_001935705.1	Beihai picorna-like virus 100
1922529	GCA_001935285.1	Beihai picorna-like virus 101
1922530	GCA_001935645.1	Beihai picorna-like virus 102
1922531	GCA_001923535.1	Beihai picorna-like virus 103
1922532	GCA_001934125.1	Beihai picorna-like virus 104
1922533	GCA_001934485.1	Beihai picorna-like virus 105
1922534	GCA_001934985.1	Beihai picorna-like virus 106
1922535	GCA_001935625.1	Beihai picorna-like virus 107
1922536	GCA_001934105.1	Beihai picorna-like virus 108
1922538	GCA_001934465.1	Beihai picorna-like virus 11

1922539	GCA_001934965.1	Beihai picorna-like virus 110
1922540	GCA_001934905.1	Beihai picorna-like virus 111
1922541	GCA_001934085.1	Beihai picorna-like virus 112
1922542	GCA_001934445.1	Beihai picorna-like virus 113
1922543	GCA_001934945.1	Beihai picorna-like virus 114
1922544	GCA_001934885.1	Beihai picorna-like virus 115
1922545	GCA_001934065.1	Beihai picorna-like virus 116
1922546	GCA_001934425.1	Beihai picorna-like virus 117
1922547	GCA_001934925.1	Beihai picorna-like virus 118
1922548	GCA_001934865.1	Beihai picorna-like virus 119
1922549	GCA_001934045.1	Beihai picorna-like virus 120
1922550	GCA_001934405.1	Beihai picorna-like virus 121
1922551	GCA_001933765.1	Beihai picorna-like virus 122
1922552	GCA_001934845.1	Beihai picorna-like virus 123
1922553	GCA_001934025.1	Beihai picorna-like virus 124
1922554	GCA_001934385.1	Beihai picorna-like virus 125
1922556	GCA_001933745.1	Beihai picorna-like virus 14
1922557	GCA_001923995.1	Beihai picorna-like virus 15
1922558	GCA_001934825.1	Beihai picorna-like virus 16
1922559	GCA_001934005.1	Beihai picorna-like virus 17
1922560	GCA_001934365.1	Beihai picorna-like virus 18
1922561	GCA_001933725.1	Beihai picorna-like virus 19
1922562	GCA_001934805.1	Beihai picorna-like virus 2
1922563	GCA_001933985.1	Beihai picorna-like virus 20
1922564	GCA_001935825.1	Beihai picorna-like virus 21
1922566	GCA_001935405.1	Beihai picorna-like virus 23
1922567	GCA_001935545.1	Beihai picorna-like virus 24
1922569	GCA_002149785.1	Beihai picorna-like virus 26
1922570	GCA_001935165.1	Beihai picorna-like virus 27
1922571	GCA_001935805.1	Beihai picorna-like virus 28
1922573	GCA_001935385.1	Beihai picorna-like virus 30
1922575	GCA_001935525.1	Beihai picorna-like virus 32
1922576	GCA_001935145.1	Beihai picorna-like virus 33
1922578	GCA_001924435.1	Beihai picorna-like virus 35
1922582	GCA_001935785.1	Beihai picorna-like virus 39
1922583	GCA_001935365.1	Beihai picorna-like virus 4
1922584	GCA_001935505.1	Beihai picorna-like virus 40
1922585	GCA_001935125.1	Beihai picorna-like virus 41
1922586	GCA_001935765.1	Beihai picorna-like virus 42
1922587	GCA_001924895.1	Beihai picorna-like virus 43
1922588	GCA_001935345.1	Beihai picorna-like virus 44
1922589	GCA_001935485.1	Beihai picorna-like virus 45
1922590	GCA_001935105.1	Beihai picorna-like virus 46
1922591	GCA_001935745.1	Beihai picorna-like virus 47
1922592	GCA_001935325.1	Beihai picorna-like virus 48
1922593	GCA_001934585.1	Beihai picorna-like virus 49
1922594	GCA_001923555.1	Beihai picorna-like virus 5
1922595	GCA_001934545.1	Beihai picorna-like virus 50
1922596	GCA_001935045.1	Beihai picorna-like virus 51
1922597	GCA_001935685.1	Beihai picorna-like virus 52

1922598	GCA_001923515.1	Beihai picorna-like virus 53
1922599	GCA_001935265.1	Beihai picorna-like virus 54
1922600	GCA_001934525.1	Beihai picorna-like virus 55
1922601	GCA_001935025.1	Beihai picorna-like virus 56
1922602	GCA_002008835.1	Beihai picorna-like virus 57
1922603	GCA_001935665.1	Beihai picorna-like virus 58
1922604	GCA_002008475.1	Beihai picorna-like virus 59
1922605	GCA_001934145.1	Beihai picorna-like virus 6
1922606	GCA_001934505.1	Beihai picorna-like virus 60
1922607	GCA_001935005.1	Beihai picorna-like virus 61
1922608	GCA_001923975.1	Beihai picorna-like virus 62
1922609	GCA_001934345.1	Beihai picorna-like virus 63
1922610	GCA_001933705.1	Beihai picorna-like virus 64
1922611	GCA_001934785.1	Beihai picorna-like virus 65
1922612	GCA_001933965.1	Beihai picorna-like virus 66
1922613	GCA_001934325.1	Beihai picorna-like virus 67
1922614	GCA_001933685.1	Beihai picorna-like virus 68
1922615	GCA_001934765.1	Beihai picorna-like virus 69
1922616	GCA_001933945.1	Beihai picorna-like virus 7
1922617	GCA_001924415.1	Beihai picorna-like virus 70
1922618	GCA_001934305.1	Beihai picorna-like virus 71
1922619	GCA_001933665.1	Beihai picorna-like virus 72
1922620	GCA_001924875.1	Beihai picorna-like virus 73
1922621	GCA_001934745.1	Beihai picorna-like virus 74
1922622	GCA_001933925.1	Beihai picorna-like virus 75
1922624	GCA_001934285.1	Beihai picorna-like virus 77
1922626	GCA_001933645.1	Beihai picorna-like virus 79
1922627	GCA_001934725.1	Beihai picorna-like virus 8
1922628	GCA_001933905.1	Beihai picorna-like virus 80
1922629	GCA_001934265.1	Beihai picorna-like virus 81
1922630	GCA_001933625.1	Beihai picorna-like virus 82
1922631	GCA_001934705.1	Beihai picorna-like virus 83
1922632	GCA_001933885.1	Beihai picorna-like virus 84
1922633	GCA_001934245.1	Beihai picorna-like virus 85
1922634	GCA_001933605.1	Beihai picorna-like virus 87
1922636	GCA_001934685.1	Beihai picorna-like virus 9
1922637	GCA_001933865.1	Beihai picorna-like virus 90
1922638	GCA_001934225.1	Beihai picorna-like virus 91
1922639	GCA_001933585.1	Beihai picorna-like virus 93
1922641	GCA_001935085.1	Beihai picorna-like virus 96
1922644	GCA_001935725.1	Beihai picorna-like virus 99
1922645	GCA_001935305.1	Beihai razor shell virus 1
1922646	GCA_001934565.1	Beihai razor shell virus 2
1922647	GCA_001934665.1	Beihai razor shell virus 3
1922648	GCA_001933845.1	Beihai razor shell virus 4
1922651	GCA_001934205.1	Beihai rhabdo-like virus 1
1922652	GCA_001933565.1	Beihai rhabdo-like virus 2
1922653	GCA_003673945.1	Beihai rhabdo-like virus 3
1922654	GCA_001934645.1	Beihai rhabdo-like virus 4
1922655	GCA_001933825.1	Beihai rhabdo-like virus 5

1922656	GCA_001934185.1	Beihai rhabdo-like virus 6
1922657	GCA_001933545.1	Beihai sea slater virus 1
1922658	GCA_001934625.1	Beihai sea slater virus 2
1922659	GCA_001933805.1	Beihai sea slater virus 3
1922660	GCA_001934165.1	Beihai sea slater virus 4
1922661	GCA_001933525.1	Beihai sesarmid crab virus 1
1922662	GCA_001934605.1	Beihai sesarmid crab virus 2
1922663	GCA_002288695.1	Beihai sesarmid crab virus 3
1922664	GCA_002288775.1	Beihai sesarmid crab virus 4
1922666	GCA_001933785.1	Beihai sesarmid crab virus 7
1922667	GCA_001935245.1	Beihai shrimp virus 1
1922668	GCA_001935885.1	Beihai shrimp virus 2
1922669	GCA_002004255.1	Beihai shrimp virus 3
1922670	GCA_001935465.1	Beihai shrimp virus 4
1922671	GCA_001935605.1	Beihai shrimp virus 5
1922672	GCA_001935225.1	Beihai shrimp virus 6
1922673	GCA_001935865.1	Beihai sipunculid worm virus 1
1922674	GCA_001935445.1	Beihai sipunculid worm virus 2
1922675	GCA_001935585.1	Beihai sipunculid worm virus 3
1922676	GCA_001935205.1	Beihai sipunculid worm virus 4
1922677	GCA_001935845.1	Beihai sipunculid worm virus 5
1922678	GCA_001935425.1	Beihai sipunculid worm virus 6
1922680	GCA_001935565.1	Beihai sobemo-like virus 1
1922681	GCA_001924395.1	Beihai sobemo-like virus 10
1922682	GCA_001935185.1	Beihai sobemo-like virus 11
1922683	GCA_001961555.1	Beihai sobemo-like virus 12
1922684	GCA_001962295.1	Beihai sobemo-like virus 13
1922685	GCA_001924855.1	Beihai sobemo-like virus 14
1922686	GCA_001963815.1	Beihai sobemo-like virus 15
1922687	GCA_001963215.1	Beihai sobemo-like virus 16
1922688	GCA_001923495.1	Beihai sobemo-like virus 17
1922689	GCA_001961535.1	Beihai sobemo-like virus 18
1922690	GCA_001962275.1	Beihai sobemo-like virus 19
1922691	GCA_001963795.1	Beihai sobemo-like virus 2
1922692	GCA_001963195.1	Beihai sobemo-like virus 20
1922693	GCA_001961515.1	Beihai sobemo-like virus 21
1922694	GCA_001962255.1	Beihai sobemo-like virus 22
1922695	GCA_001963775.1	Beihai sobemo-like virus 23
1922696	GCA_001923955.1	Beihai sobemo-like virus 24
1922697	GCA_001963175.1	Beihai sobemo-like virus 25
1922698	GCA_001924375.1	Beihai sobemo-like virus 26
1922699	GCA_001961495.1	Beihai sobemo-like virus 27
1922700	GCA_001962235.1	Beihai sobemo-like virus 3
1922701	GCA_001963755.1	Beihai sobemo-like virus 4
1922702	GCA_001963155.1	Beihai sobemo-like virus 5
1922703	GCA_001961475.1	Beihai sobemo-like virus 6
1922704	GCA_001962215.1	Beihai sobemo-like virus 7
1922705	GCA_001963735.1	Beihai sobemo-like virus 8
1922706	GCA_001963135.1	Beihai sobemo-like virus 9
1922707	GCA_001961455.1	Beihai sphaeromadae virus 1

1922708	GCA_001962195.1	Beihai sphaeromadae virus 2
1922709	GCA_001921295.1	Beihai sphaeromadae virus 3
1922710	GCA_001963715.1	Beihai sphaeromadae virus 4
1922711	GCA_001963115.1	Beihai tiger crab virus 1
1922712	GCA_001961435.1	Beihai tombus-like virus 1
1922713	GCA_001962175.1	Beihai tombus-like virus 10
1922714	GCA_001963695.1	Beihai tombus-like virus 11
1922715	GCA_001961715.1	Beihai tombus-like virus 12
1922716	GCA_001962455.1	Beihai tombus-like virus 13
1922717	GCA_001963975.1	Beihai tombus-like virus 14
1922718	GCA_001963375.1	Beihai tombus-like virus 15
1922719	GCA_001961695.1	Beihai tombus-like virus 16
1922720	GCA_001962435.1	Beihai tombus-like virus 17
1922721	GCA_001963955.1	Beihai tombus-like virus 18
1922722	GCA_001967275.1	Beihai tombus-like virus 19
1922723	GCA_001963355.1	Beihai tombus-like virus 2
1922724	GCA_001961675.1	Beihai tombus-like virus 3
1922725	GCA_001924835.1	Beihai tombus-like virus 4
1922726	GCA_001962415.1	Beihai tombus-like virus 5
1922728	GCA_001963935.1	Beihai tombus-like virus 7
1922729	GCA_001963335.1	Beihai tombus-like virus 8
1922730	GCA_001961655.1	Beihai tombus-like virus 9
1922734	GCA_001962395.1	Beihai toti-like virus 4
1922736	GCA_001963915.1	Beihai uca arcuata virus 1
1922737	GCA_001963315.1	Beihai victori-like virus 1
1922738	GCA_001961635.1	Beihai weivirus-like virus 1
1922740	GCA_001962375.1	Beihai weivirus-like virus 11
1922741	GCA_001963895.1	Beihai weivirus-like virus 12
1922742	GCA_001963295.1	Beihai weivirus-like virus 13
1922743	GCA_001961615.1	Beihai weivirus-like virus 15
1922744	GCA_001962355.1	Beihai weivirus-like virus 16
1922745	GCA_001963875.1	Beihai weivirus-like virus 17
1922746	GCA_001963275.1	Beihai weivirus-like virus 18
1922748	GCA_001961595.1	Beihai weivirus-like virus 2
1922749	GCA_001962335.1	Beihai weivirus-like virus 20
1922750	GCA_001963855.1	Beihai weivirus-like virus 21
1922751	GCA_001963255.1	Beihai weivirus-like virus 3
1922752	GCA_001961575.1	Beihai weivirus-like virus 4
1922753	GCA_001964135.1	Beihai weivirus-like virus 5
1922754	GCA_001963535.1	Beihai weivirus-like virus 7
1922755	GCA_001961855.1	Beihai weivirus-like virus 8
1922756	GCA_001962595.1	Beihai weivirus-like virus 9
1922757	GCA_001964115.1	Beihai zhaovirus-like virus 1
1922758	GCA_001963515.1	Beihai zhaovirus-like virus 2
1922759	GCA_001961835.1	Beihai zhaovirus-like virus 3
1922760	GCA_001962575.1	Beihai zhaovirus-like virus 4
1922761	GCA_001964095.1	Beihai zhaovirus-like virus 5
341053	GCA_000868925.1	Beilong virus
354328	GCA_000892875.1	Bell pepper alphaendornavirus
354328	GCA_002820425.1	Bell pepper alphaendornavirus

368735	GCA_000873425.1	Bell pepper mottle virus
1720595	GCA_001308735.1	Bellflower vein chlorosis virus
1982660	GCA_003029555.1	Bellflower veinal mottle virus
694015	GCA_000872845.1	Beluga whale coronavirus SW1
1986477	GCA_002210855.1	Bemisia-associated genomovirus AdDF
1986478	GCA_002211035.1	Bemisia-associated genomovirus AdO
1986479	GCA_002210835.1	Bemisia-associated genomovirus NfO
713090	GCA_003034035.1	Bendhi Yellow Vein Mosaic/Mesta Yellow Vein Mosaic alph
1930269	GCA_001926835.1	Bermuda grass latent virus
318834	GCA_000927035.1	Berrimah virus
1263720	GCA_002816195.1	Betacoronavirus England 1
1385427	GCA_000912235.1	Betacoronavirus Erinaceus/VMC/DEU/2012
1385427	GCA_002816175.1	Betacoronavirus Erinaceus/VMC/DEU/2012
1590370	GCA_000930095.1	Betacoronavirus HKU24
337051	GCA_004289675.1	Betapapillomavirus 1
337051	GCA_004289695.1	Betapapillomavirus 1
337051	GCA_004289715.1	Betapapillomavirus 1
333924	GCA_004289575.1	Betapapillomavirus 2
333924	GCA_004289595.1	Betapapillomavirus 2
333924	GCA_004289615.1	Betapapillomavirus 2
333924	GCA_004289635.1	Betapapillomavirus 2
333924	GCA_004289655.1	Betapapillomavirus 2
334208	GCA_004289535.1	Betapapillomavirus 4
334208	GCA_004289555.1	Betapapillomavirus 4
334209	GCA_004289515.1	Betapapillomavirus 5
759701	GCA_000887415.1	Bettongia penicillata papillomavirus 1
1213620	GCA_001019855.1	Bhanja virus
591160	GCA_000881075.1	Bhendi yellow vein Bhubhaneswar virus
587620	GCA_001046725.1	Bhendi yellow vein Delhi virus [2004:New Delhi]
908010	GCA_000891395.1	Bhendi yellow vein India betasatellite [India:Aurangabad:O
908028	GCA_000889575.1	Bhendi yellow vein India virus [India:Dharwad OYDWR2:20
759124	GCA_000989055.1	Bhendi yellow vein mosaic alphasatellite
908070	GCA_000843925.1	Bhendi yellow vein mosaic betasatellite
908072	GCA_000889595.1	Bhendi yellow vein mosaic betasatellite [India:Coimbator:(
120168	GCA_002821725.1	Bhendi yellow vein mosaic virus
120168	GCA_002821745.1	Bhendi yellow vein mosaic virus
120168	GCA_002986335.1	Bhendi yellow vein mosaic virus
611724	GCA_002821765.1	Bhendi yellow vein mosaic virus [2005:Thadagan:India]
221523	GCA_001046705.1	Bhendi yellow vein mosaic virus-[Madurai]
1225644	GCA_000898635.1	Bhendi yellow vein mosaic virus-associated alphasatellite
1841147	GCA_001579335.1	Bhindi yellow vein alphasatellite
320856	GCA_000915195.1	Bidens mosaic virus
433357	GCA_000889115.1	Bidens mottle virus
743964	GCA_002633625.1	Bifidobacterium phage Bbif-1
2576879	GCA_003368805.1	Bifidobacterium phage PMBT6
1955196	GCA_002024675.1	Big Cypress virus
1041883	GCA_002219505.1	Big Sioux River virus
1678224	GCA_002831265.1	Bimiti virus
1712387	GCA_001308375.1	Binucleate Rhizoctonia mitovirus K1
1931369	GCA_001967655.1	Biomphalaria virus 1

1931370	GCA_001974175.1	Biomphalaria virus 2
1931371	GCA_001968155.1	Biomphalaria virus 3
2138326	GCA_004117235.1	Bipolaris maydis botybirnavirus 1
1980631	GCA_002145905.1	Bipolaris maydis partitivirus 1
2483307	GCA_004117555.1	Bipolaris maydis partitivirus 2
1955197	GCA_002024795.1	Biratnagar virus
2057979	GCA_004132845.1	Birch leaf roll-associated virus
316106	GCA_000866905.1	Bitter gourd leaf curl betasatellite
1513489	GCA_000926175.1	Bitter gourd yellow vein virus
1926998	GCA_001907865.1	Bivalve hepelivirus G
1926967	GCA_001907765.1	Bivalve RNA virus G1
1926996	GCA_001907885.1	Bivalve RNA virus G2
1926969	GCA_001907905.1	Bivalve RNA virus G3
1926997	GCA_001907805.1	Bivalve RNA virus G4
1926968	GCA_001907785.1	Bivalve RNA virus G5
909207	GCA_002815895.1	Bivens Arm virus
12285	GCA_000855345.1	Black beetle virus
1937789	GCA_002288795.1	Black currant leaf chlorosis associated virus
1587517	GCA_000970585.1	Black grass cryptic virus 2
1587515	GCA_000970565.1	Black grass varicosavirus-like virus
2038729	GCA_000914275.1	Black medic leaf roll virus
1441796	GCA_000920555.1	Black medic leafroll alphasatellite 1
92395	GCA_000851425.1	Black queen cell virus
367301	GCA_000867325.1	Black raspberry necrosis virus
463392	GCA_000872065.1	Black raspberry virus F
1391037	GCA_000928815.1	Black robin associated gemykibivirus 1
1572341	GCA_000928835.1	Black sea bass polyomavirus 1
339420	GCA_000881795.1	Blackberry chlorotic ringspot virus
1381464	GCA_000913355.1	Blackberry vein banding-associated virus
2185086	GCA_004132425.1	Blackberry virus A
1043184	GCA_000893735.1	Blackberry virus E
1490026	GCA_001567075.1	Blackberry Virus F
670883	GCA_002817835.1	Blackberry virus S
323118	GCA_000868605.1	Blackberry virus Y
404196	GCA_000858905.1	Blackberry yellow vein-associated virus
2099352	GCA_004132805.1	Blackbird arilivirus
1985371	GCA_000927895.1	Blackbird associated gemycircularvirus 1
1391038	GCA_000927915.1	Blackbird associated gemykibivirus 1
2292426	GCA_004134225.1	Blackcurrant leafroll-associated virus 1
2292426	GCA_004134285.1	Blackcurrant leafroll-associated virus 1
2292426	GCA_004134245.1	Blackcurrant leafroll-associated virus 1
65743	GCA_000849565.1	Blackcurrant reversion virus
99930	GCA_000852285.1	Blackcurrant reversion virus satellite RNA
2338394	GCA_004133905.1	Blackcurrant-associated closterovirus 1
536081	GCA_000880175.1	Blainvillea yellow spot virus
220638	GCA_000843285.1	Blattella germanica densovirus 1
2364200	GCA_004133405.1	Blechmonas luni narnavirus 1
2364202	GCA_004133425.1	Blechomonas maslovi narnavirus 1
2364201	GCA_004133445.1	Blechomonas wendygibsoni narnavirus 1
1246650	GCA_000897995.1	Blechum interveinal chlorosis virus

1654577	GCA_003729655.1	Bloomfield virus
311176	GCA_000853685.1	Blotched snakehead virus
1072517	GCA_000902235.1	Blue squill virus A
1751715	GCA_001448395.1	Blueberry fruit drop associated virus
1071488	GCA_002867165.1	Blueberry latent spherical virus
430710	GCA_000887675.1	Blueberry latent virus
1520332	GCA_000921335.1	Blueberry mosaic associated virus
1520332	GCA_000921335.2	Blueberry mosaic associated virus
1094249	GCA_000893955.1	Blueberry necrotic ring blotch virus
172220	GCA_000837345.1	Blueberry red ringspot virus
31722	GCA_000861365.1	Blueberry scorch virus
747056	GCA_000912635.1	Blueberry shock virus
1714570	GCA_001586925.1	Blueberry shoestring virus
1206566	GCA_000897595.1	Blueberry virus A
2169918	GCA_001678835.1	Bluegill hepatitis B virus
1223302	GCA_000898575.1	Bluegill picornavirus
40051	GCA_000854445.1	Bluetongue virus
40051	GCA_000854445.2	Bluetongue virus
40051	GCA_003081075.1	Bluetongue virus
40051	GCA_003080175.1	Bluetongue virus
40051	GCA_003080075.1	Bluetongue virus
40051	GCA_003081215.1	Bluetongue virus
40051	GCA_003081475.1	Bluetongue virus
40051	GCA_003080715.1	Bluetongue virus
40051	GCA_003080135.1	Bluetongue virus
40051	GCA_003080955.1	Bluetongue virus
40051	GCA_003079755.1	Bluetongue virus
40051	GCA_003081095.1	Bluetongue virus
40051	GCA_003080275.1	Bluetongue virus
40051	GCA_003079415.1	Bluetongue virus
40051	GCA_003078835.1	Bluetongue virus
40051	GCA_003080355.1	Bluetongue virus
40051	GCA_003080055.1	Bluetongue virus
40051	GCA_003077535.1	Bluetongue virus
40051	GCA_003080155.1	Bluetongue virus
40051	GCA_003079815.1	Bluetongue virus
40051	GCA_003080195.1	Bluetongue virus
40051	GCA_003080115.1	Bluetongue virus
40051	GCA_003080295.1	Bluetongue virus
40051	GCA_003078915.1	Bluetongue virus
40051	GCA_003080335.1	Bluetongue virus
40051	GCA_003079975.1	Bluetongue virus
40051	GCA_003081195.1	Bluetongue virus
40051	GCA_003078995.1	Bluetongue virus
40051	GCA_003081355.1	Bluetongue virus
40051	GCA_003081375.1	Bluetongue virus
40051	GCA_003080315.1	Bluetongue virus
40051	GCA_003078715.1	Bluetongue virus
40051	GCA_003078895.1	Bluetongue virus
40051	GCA_003080255.1	Bluetongue virus

40051	GCA_003173835.1	Bluetongue virus
35327	GCA_003078435.1	Bluetongue virus 1
35327	GCA_003079295.1	Bluetongue virus 1
35327	GCA_003080675.1	Bluetongue virus 1
35327	GCA_003081155.1	Bluetongue virus 1
35327	GCA_003078475.1	Bluetongue virus 1
35327	GCA_003078155.1	Bluetongue virus 1
35327	GCA_003079435.1	Bluetongue virus 1
35327	GCA_003078555.1	Bluetongue virus 1
35327	GCA_003081175.1	Bluetongue virus 1
35327	GCA_003080215.1	Bluetongue virus 1
35327	GCA_003079855.1	Bluetongue virus 1
35327	GCA_003077975.1	Bluetongue virus 1
35327	GCA_003078035.1	Bluetongue virus 1
35327	GCA_003078055.1	Bluetongue virus 1
35327	GCA_003078115.1	Bluetongue virus 1
35327	GCA_003081115.1	Bluetongue virus 1
35327	GCA_003078215.1	Bluetongue virus 1
35327	GCA_003078235.1	Bluetongue virus 1
35327	GCA_003078275.1	Bluetongue virus 1
35327	GCA_003078295.1	Bluetongue virus 1
35327	GCA_003078355.1	Bluetongue virus 1
35327	GCA_003078415.1	Bluetongue virus 1
35327	GCA_003077615.1	Bluetongue virus 1
35327	GCA_003077955.1	Bluetongue virus 1
35327	GCA_003078075.1	Bluetongue virus 1
35327	GCA_003078495.1	Bluetongue virus 1
35327	GCA_003077635.1	Bluetongue virus 1
35327	GCA_003079355.1	Bluetongue virus 1
35327	GCA_003079335.1	Bluetongue virus 1
35327	GCA_003081135.1	Bluetongue virus 1
35327	GCA_003077995.1	Bluetongue virus 1
35327	GCA_003079315.1	Bluetongue virus 1
35327	GCA_003078375.1	Bluetongue virus 1
35327	GCA_003079375.1	Bluetongue virus 1
35327	GCA_003079015.1	Bluetongue virus 1
35327	GCA_003079055.1	Bluetongue virus 1
10906	GCA_003079155.1	Bluetongue virus 10
10906	GCA_003078775.1	Bluetongue virus 10
10906	GCA_003077755.1	Bluetongue virus 10
10906	GCA_003080555.1	Bluetongue virus 10
35329	GCA_003079255.1	Bluetongue virus 11
35329	GCA_003079575.1	Bluetongue virus 11
35329	GCA_003080835.1	Bluetongue virus 11
35329	GCA_003079475.1	Bluetongue virus 11
35329	GCA_003080615.1	Bluetongue virus 11
35329	GCA_003079595.1	Bluetongue virus 11
35329	GCA_003079495.1	Bluetongue virus 11
35329	GCA_003079535.1	Bluetongue virus 11
35329	GCA_003080415.1	Bluetongue virus 11

35329	GCA_003079515.1	Bluetongue virus 11
35329	GCA_003079555.1	Bluetongue virus 11
35329	GCA_003081055.1	Bluetongue virus 11
35329	GCA_003080595.1	Bluetongue virus 11
35329	GCA_003079615.1	Bluetongue virus 11
94966	GCA_003078875.1	Bluetongue virus 12
94966	GCA_003080755.1	Bluetongue virus 12
35330	GCA_003080495.1	Bluetongue virus 13
248912	GCA_003081035.1	Bluetongue virus 14
35331	GCA_003079935.1	Bluetongue virus 15
35331	GCA_003080515.1	Bluetongue virus 15
45029	GCA_003078815.1	Bluetongue virus 16
45029	GCA_003077775.1	Bluetongue virus 16
45029	GCA_003080015.1	Bluetongue virus 16
45029	GCA_003077805.1	Bluetongue virus 16
45029	GCA_003077835.1	Bluetongue virus 16
45029	GCA_003077895.1	Bluetongue virus 16
45029	GCA_003080095.1	Bluetongue virus 16
45029	GCA_003080775.1	Bluetongue virus 16
45029	GCA_003078675.1	Bluetongue virus 16
45029	GCA_003078735.1	Bluetongue virus 16
45029	GCA_003078955.1	Bluetongue virus 16
10903	GCA_003079275.1	Bluetongue virus 17
10903	GCA_003080935.1	Bluetongue virus 17
197781	GCA_003080995.1	Bluetongue virus 18
248913	GCA_003080535.1	Bluetongue virus 19
35328	GCA_003080435.1	Bluetongue virus 2
35328	GCA_003079655.1	Bluetongue virus 2
35328	GCA_003081255.1	Bluetongue virus 2
35328	GCA_003081295.1	Bluetongue virus 2
35328	GCA_003081235.1	Bluetongue virus 2
35328	GCA_003077875.1	Bluetongue virus 2
35328	GCA_003080695.1	Bluetongue virus 2
35328	GCA_003077575.1	Bluetongue virus 2
35328	GCA_003079895.1	Bluetongue virus 2
35328	GCA_003078175.1	Bluetongue virus 2
35328	GCA_003078515.1	Bluetongue virus 2
35328	GCA_003077655.1	Bluetongue virus 2
35328	GCA_003081275.1	Bluetongue virus 2
35328	GCA_003077675.1	Bluetongue virus 2
35332	GCA_003079875.1	Bluetongue virus 20
35332	GCA_003081015.1	Bluetongue virus 20
45030	GCA_003077935.1	Bluetongue virus 21
45030	GCA_003079955.1	Bluetongue virus 21
248914	GCA_003080855.1	Bluetongue virus 22
45031	GCA_003080735.1	Bluetongue virus 23
45031	GCA_003079995.1	Bluetongue virus 23
45031	GCA_003081315.1	Bluetongue virus 23
45031	GCA_003081335.1	Bluetongue virus 23
248915	GCA_003080975.1	Bluetongue virus 24

36423	GCA_003079115.1	Bluetongue virus 3
36423	GCA_003080235.1	Bluetongue virus 3
36423	GCA_003080035.1	Bluetongue virus 3
36423	GCA_003080815.1	Bluetongue virus 3
94967	GCA_003079075.1	Bluetongue virus 4
94967	GCA_003079175.1	Bluetongue virus 4
94967	GCA_003079835.1	Bluetongue virus 4
94967	GCA_003080795.1	Bluetongue virus 4
94967	GCA_003079635.1	Bluetongue virus 4
94967	GCA_003078855.1	Bluetongue virus 4
94967	GCA_003080375.1	Bluetongue virus 4
94967	GCA_003079455.1	Bluetongue virus 4
248909	GCA_003080635.1	Bluetongue virus 5
248910	GCA_003080655.1	Bluetongue virus 6
248911	GCA_003080395.1	Bluetongue virus 7
248911	GCA_003080475.1	Bluetongue virus 7
197780	GCA_003080875.1	Bluetongue virus 8
197780	GCA_003079675.1	Bluetongue virus 8
197780	GCA_003078615.1	Bluetongue virus 8
197780	GCA_003078575.1	Bluetongue virus 8
197780	GCA_003078655.1	Bluetongue virus 8
197780	GCA_003079695.1	Bluetongue virus 8
197780	GCA_003080455.1	Bluetongue virus 8
45032	GCA_003081435.1	Bluetongue virus 9
45032	GCA_003077695.1	Bluetongue virus 9
45032	GCA_003079715.1	Bluetongue virus 9
45032	GCA_003079235.1	Bluetongue virus 9
45032	GCA_003081455.1	Bluetongue virus 9
45032	GCA_003080915.1	Bluetongue virus 9
45032	GCA_003079735.1	Bluetongue virus 9
45032	GCA_003080895.1	Bluetongue virus 9
45032	GCA_003077715.1	Bluetongue virus 9
45032	GCA_003079915.1	Bluetongue virus 9
45032	GCA_003080575.1	Bluetongue virus 9
45032	GCA_003079775.1	Bluetongue virus 9
45032	GCA_003079795.1	Bluetongue virus 9
45032	GCA_003081395.1	Bluetongue virus 9
45032	GCA_003081415.1	Bluetongue virus 9
2294156	GCA_004134345.1	Boa constrictor papillomavirus 1
864686	GCA_000889135.1	Bocavirus gorilla/GBoV1/2009
912967	GCA_002827285.1	Bocavirus pig/SX/China/2010
2024608	GCA_002966405.1	Bodo saltans virus
361697	GCA_002821785.1	Boerhavia yellow spot virus
100220	GCA_002826565.1	Bohle iridovirus
1379788	GCA_000924715.1	Boiling Springs Lake RNA-DNA hybrid virus
1072176	GCA_000924435.1	Bokeloh bat lyssavirus
1072176	GCA_900176785.1	Bokeloh bat lyssavirus
1072176	GCA_900176795.1	Bokeloh bat lyssavirus
1072176	GCA_900176815.1	Bokeloh bat lyssavirus
1072176	GCA_900176945.1	Bokeloh bat lyssavirus

1903560	GCA_003673905.1	Bolahun virus variant 2
1608041	GCA_001746095.1	Bole Tick Virus 2
1608042	GCA_001441115.1	Bole Tick Virus 3
1746058	GCA_001444065.1	Bole tick virus 4
2010960	GCA_003505815.1	Bombali ebolavirus
2094261	GCA_004132225.1	Bombus cryptarum densovirus
640862	GCA_000884815.1	Bombyx mandarina nucleopolyhedrovirus
284503	GCA_000846085.1	Bombyx mori cypovirus 1 satellite RNA
46253	GCA_003033205.1	Bombyx mori densovirus 1
328668	GCA_000908215.1	Bombyx mori densovirus 3
125788	GCA_000861145.1	Bombyx mori densovirus 5
530042	GCA_002819305.1	Bombyx mori densovirus Zhenjiang
1690666	GCA_001271195.1	Bombyx mori iflavirus
2065033	GCA_002817955.1	Bombyx mori latent virus
288456	GCA_000892755.1	Bombyx mori Macula-like virus
271108	GCA_000837145.1	Bombyx mori nucleopolyhedrovirus
12286	GCA_000852425.1	Boolarra virus
2034827	GCA_002816635.1	Boone cardiovirus 1
2169775	GCA_000930935.1	Bopivirus A
56948	GCA_000862865.1	Border disease virus X818
263375	GCA_002758455.1	Bordetella phage BIP-1
263374	GCA_002758435.1	Bordetella phage BMP-1
1916123	GCA_002954935.1	Bordetella phage CN1
1916124	GCA_002954945.1	Bordetella phage CN2
1916125	GCA_002954955.1	Bordetella phage FP1
1916126	GCA_002954965.1	Bordetella phage MW2
2029657	GCA_002743695.1	Bordetella phage vB_BbrM_PHB04
194699	GCA_000841725.1	Bordetella virus BPP1
1926943	GCA_002954975.2	Bordetella virus LK3
1926943	GCA_002954975.1	Bordetella virus LK3
1714621	GCA_002366305.1	Borna disease virus 1
1714621	GCA_900323385.1	Borna disease virus 1
1714621	GCA_900323405.1	Borna disease virus 1
1714621	GCA_900323395.1	Borna disease virus 1
1714622	GCA_000847565.2	Borna disease virus 2
1604874	GCA_000885375.1	Bornean orang-utan polyomavirus
1229657	GCA_003179355.1	Bos grunniens papillomavirus type 1
1070324	GCA_001430515.1	Bos taurus papillomavirus 12
1887213	GCA_001714395.1	Bos taurus papillomavirus 13
1887214	GCA_001714535.1	Bos taurus papillomavirus 16
1887215	GCA_001714435.1	Bos taurus papillomavirus 17
1887216	GCA_001714475.1	Bos taurus papillomavirus 18
1887217	GCA_001714375.1	Bos taurus papillomavirus 19
1887218	GCA_001714515.1	Bos taurus papillomavirus 20
1887219	GCA_001714415.1	Bos taurus papillomavirus 21
1001533	GCA_000864305.1	Bos taurus papillomavirus 7
1891754	GCA_000836825.1	Bos taurus polyomavirus 1
1917013	GCA_002366225.1	Bosavirus MS-2016a
1405299	GCA_002116115.1	Botryosphaeria dothidea chrysovirus 1
1547580	GCA_000924415.1	Botryosphaeria dothidea victorivirus 1

1516075	GCA_001974015.1	Botryosphaeria dothidea virus 1
425010	GCA_000874945.1	Botryotinia fuckeliana partitivirus 1
425009	GCA_000873065.1	Botryotinia fuckeliana totivirus 1
2169747	GCA_001866855.1	Botrytis cinerea betaendornavirus 1
2219106	GCA_003260875.1	Botrytis cinerea fusarivirus 1
2219107	GCA_003260895.1	Botrytis cinerea fusarivirus 1-S1
2219108	GCA_003260915.1	Botrytis cinerea fusarivirus 1-S2
2219105	GCA_003260855.1	Botrytis cinerea hypovirus 1
2219109	GCA_003260935.1	Botrytis cinerea hypovirus 1 satellite-like RNA
2219110	GCA_003260955.1	Botrytis cinerea hypovirus 1 satellite-like RNA-S
444193	GCA_000883195.1	Botrytis cinerea mitovirus 1
1629665	GCA_001461625.1	Botrytis cinerea mitovirus 2
1629666	GCA_001461465.1	Botrytis cinerea mitovirus 3
1629667	GCA_001461185.1	Botrytis cinerea mitovirus 4
1629671	GCA_001461065.1	Botrytis cinerea negative-stranded RNA virus 1
1568973	GCA_000929135.1	Botrytis cinerea RNA virus 1
1872719	GCA_001461305.1	Botrytis ourmia-like virus
1918014	GCA_000899715.1	Botrytis porri botybirnavirus 1
129395	GCA_000848525.1	Botrytis virus F
174142	GCA_000854485.1	Botrytis virus X
1714377	GCA_900098775.1	Bottlenose dolphin adenovirus 1
64295	GCA_002004955.1	Bouboui virus
263892	GCA_000880895.1	Bougainvillea chlorotic vein banding virus
256548	GCA_000846165.1	Bovine adeno-associated virus
10546	GCA_002818015.1	Bovine adenovirus 1
114429	GCA_000844185.1	Bovine adenovirus 2
114429	GCA_000885895.1	Bovine adenovirus 2
10510	GCA_000844245.1	Bovine adenovirus 3
10510	GCA_000885255.1	Bovine adenovirus 3
70333	GCA_000845805.1	Bovine adenovirus 4
111167	GCA_000904975.1	Bovine adenovirus 6
129953	GCA_000847265.1	Bovine adenovirus A
35244	GCA_000842385.1	Bovine alphaherpesvirus 5
1435485	GCA_000922875.1	Bovine astrovirus
1027244	GCA_000918415.1	Bovine astrovirus B170/HK
1027245	GCA_000916515.1	Bovine astrovirus B18/HK
1027248	GCA_000917375.1	Bovine astrovirus B76/HK
1027247	GCA_000914835.1	Bovine astrovirus B76-2/HK
1522059	GCA_000922275.1	Bovine astrovirus CH13
1812179	GCA_001714355.1	Bovine calicivirus strain Kirklareli
85542	GCA_004058555.1	Bovine circovirus
11128	GCA_000862505.1	Bovine coronavirus
11303	GCA_000845545.1	Bovine ephemeral fever virus
1843763	GCA_001646435.1	Bovine faeces associated circular DNA molecule 1
1843764	GCA_001645855.1	Bovine faeces associated circular DNA virus 1
1843765	GCA_001646215.1	Bovine faeces associated circular DNA virus 2
1843749	GCA_001645915.1	Bovine faeces associated smacovirus 1
1843750	GCA_001646095.1	Bovine faeces associated smacovirus 2
1843751	GCA_001646295.1	Bovine faeces associated smacovirus 3
1843752	GCA_001646475.1	Bovine faeces associated smacovirus 4

1843753	GCA_001645895.1	Bovine faeces associated smacovirus 5
1843754	GCA_001646075.1	Bovine faeces associated smacovirus 6
207343	GCA_000848225.1	Bovine foamy virus
10385	GCA_000839745.1	Bovine gammaherpesvirus 4
1504288	GCA_000921015.1	Bovine gammaherpesvirus 6
1633878	GCA_000972615.1	Bovine hepacivirus
79889	GCA_000847945.1	Bovine herpesvirus type 1.1
494651	GCA_003033335.1	Bovine hokovirus 1
1431464	GCA_000897695.1	Bovine hungarovirus 1
11657	GCA_000848165.1	Bovine immunodeficiency virus
11901	GCA_000853665.1	Bovine leukemia virus
1631554	GCA_001021215.1	Bovine nidovirus TCH5
11246	GCA_000849305.1	Bovine orthopneumovirus
10571	GCA_002220025.1	Bovine papillomavirus
1932425	GCA_003179915.1	Bovine papillomavirus 04AC14
374077	GCA_003177995.1	Bovine papillomavirus 8
1969700	GCA_003179655.1	Bovine papillomavirus Aks-02
453461	GCA_003177435.1	Bovine papillomavirus type 10
714200	GCA_003177455.1	Bovine papillomavirus type 11
1232138	GCA_003179335.1	Bovine papillomavirus type 13
1639825	GCA_003179675.1	Bovine papillomavirus type 14
10560	GCA_003180015.1	Bovine papillomavirus type 2
10562	GCA_003180435.1	Bovine papillomavirus type 4
10563	GCA_003177695.1	Bovine papillomavirus type 6
453460	GCA_003177415.1	Bovine papillomavirus type 9
129727	GCA_000844045.1	Bovine papular stomatitis virus
10784	GCA_000837625.1	Bovine parvovirus
172296	GCA_000846945.1	Bovine parvovirus - 2
172296	GCA_004368215.1	Bovine parvovirus - 2
172297	GCA_002827445.1	Bovine parvovirus 3
172297	GCA_002937235.1	Bovine parvovirus 3
172297	GCA_004368195.1	Bovine parvovirus 3
365609	GCA_003033295.1	Bovine parvovirus-1
1578134	GCA_000927975.1	Bovine polyomavirus 2
1561705	GCA_000930535.1	Bovine polyomavirus 3
11247	GCA_002815455.1	Bovine respiratory syncytial virus ATCC51908
11215	GCA_000854745.1	Bovine respirovirus 3
1822365	GCA_001619055.1	Bovine retrovirus CH15
1155188	GCA_002816515.1	Bovine rhinitis A virus
693066	GCA_000879775.1	Bovine rhinitis B virus
1606765	GCA_002080315.1	Bovine rhinovirus 1
10927	GCA_002651955.1	Bovine rotavirus
10927	GCA_002652855.1	Bovine rotavirus
10927	GCA_002652175.1	Bovine rotavirus
10927	GCA_002651515.1	Bovine rotavirus
10927	GCA_002655955.1	Bovine rotavirus
10927	GCA_002655295.1	Bovine rotavirus
10927	GCA_002654395.1	Bovine rotavirus
10927	GCA_002652415.1	Bovine rotavirus
10927	GCA_002650415.1	Bovine rotavirus

10927	GCA_002653955.1	Bovine rotavirus
10927	GCA_002654635.1	Bovine rotavirus
10927	GCA_002653295.1	Bovine rotavirus
10927	GCA_002652635.1	Bovine rotavirus
10927	GCA_002650635.1	Bovine rotavirus
10927	GCA_002656395.1	Bovine rotavirus
10927	GCA_002651295.1	Bovine rotavirus
10927	GCA_002650855.1	Bovine rotavirus
10927	GCA_002655735.1	Bovine rotavirus
10927	GCA_002655075.1	Bovine rotavirus
10927	GCA_002651075.1	Bovine rotavirus
10927	GCA_002649075.1	Bovine rotavirus
10927	GCA_002656615.1	Bovine rotavirus
10927	GCA_002653075.1	Bovine rotavirus
10927	GCA_002649295.1	Bovine rotavirus
10927	GCA_002656175.1	Bovine rotavirus
10927	GCA_002653515.1	Bovine rotavirus
10927	GCA_002649515.1	Bovine rotavirus
10927	GCA_002655515.1	Bovine rotavirus
10927	GCA_002653735.1	Bovine rotavirus
10927	GCA_002654855.1	Bovine rotavirus
10927	GCA_002651735.1	Bovine rotavirus
10927	GCA_002654175.1	Bovine rotavirus
10927	GCA_002649735.1	Bovine rotavirus
35333	GCA_002636355.1	Bovine rotavirus A
35333	GCA_002634455.1	Bovine rotavirus A
35333	GCA_002643275.1	Bovine rotavirus A
35333	GCA_002661035.1	Bovine rotavirus A
35333	GCA_002661055.1	Bovine rotavirus A
35333	GCA_002666155.1	Bovine rotavirus A
35333	GCA_002665075.1	Bovine rotavirus A
35333	GCA_002667475.1	Bovine rotavirus A
31588	GCA_003087335.1	Bovine rotavirus C
31588	GCA_003087395.1	Bovine rotavirus C
31588	GCA_003087415.1	Bovine rotavirus C
31588	GCA_003087475.1	Bovine rotavirus C
31588	GCA_003087495.1	Bovine rotavirus C
31588	GCA_003087435.1	Bovine rotavirus C
31588	GCA_003087455.1	Bovine rotavirus C
195476	GCA_002667195.1	Bovine rotavirus G6
195476	GCA_002667215.1	Bovine rotavirus G6
195476	GCA_002667235.1	Bovine rotavirus G6
195476	GCA_002667255.1	Bovine rotavirus G6
2340909	GCA_004133865.1	Bovine serum-associated circular virus
2340909	GCA_004133885.1	Bovine serum-associated circular virus
11099	GCA_900173515.1	Bovine viral diarrhea virus 1
11099	GCA_900199735.1	Bovine viral diarrhea virus 1
11099	GCA_900199745.1	Bovine viral diarrhea virus 1
11099	GCA_900199475.1	Bovine viral diarrhea virus 1
11099	GCA_900167835.1	Bovine viral diarrhea virus 1

11100	GCA_000861245.1	Bovine viral diarrhea virus 1-NADL
54315	GCA_003029635.1	Bovine viral diarrhea virus 2
60869	GCA_000855985.1	Bovine viral diarrhea virus 2 C413
402584	GCA_000885615.1	Bovine viral diarrhea virus 3 Th/04_KhonKaen
1400425	GCA_002118085.1	Bowe virus
1911438	GCA_001866305.1	Bradson virus
1983459	GCA_002623525.1	Bradyrhizobium phage BDU-MI-1
2419797	GCA_004134645.1	Brassica napus RNA virus 1
1046403	GCA_000894875.2	Brassica yellows virus
1046403	GCA_000894875.1	Brassica yellows virus
2126980	GCA_900323535.1	Brazilian cedratvirus IHUMI
2169992	GCA_000855825.1	Brazilian mammarenavirus
1813599	GCA_001602085.1	Brazilian marseillevirus
1368616	GCA_000909835.1	Brazoran virus
46473	GCA_000865145.1	Breda virus
1552662	GCA_001707005.1	Brejeira virus
1296661	GCA_001505195.1	Brevibacillus phage Abouo
1296662	GCA_000914135.1	Brevibacillus phage Davies
1296660	GCA_002603365.1	Brevibacillus phage Emery
1691954	GCA_001504215.1	Brevibacillus phage Jenst
1296659	GCA_001551445.1	Brevibacillus phage Jimmer1
1296658	GCA_002624405.1	Brevibacillus phage Jimmer2
1691955	GCA_001505675.1	Brevibacillus phage Osiris
1691957	GCA_002607205.1	Brevibacillus phage Powder
1691956	GCA_002607185.1	Brevibacillus phage SecTim467
1691958	GCA_001500375.1	Brevibacillus phage Sundance
2338395	GCA_003722595.1	Brevibacterium phage Cantare
2027888	GCA_002629305.1	Brevibacterium phage LuckyBarnes
436447	GCA_000873865.1	Brevicoryne brassicae virus - UK
12301	GCA_000851565.1	Broad bean mottle virus
79918	GCA_000851065.1	Broad bean necrosis virus
649890	GCA_000910755.1	Broad bean true mosaic virus
50817	GCA_000853465.1	Broad bean wilt virus 1
76875	GCA_000861605.1	Broad bean wilt virus 2
1987403	GCA_002828025.1	Broad-leafed dock virus A
857312	GCA_000892375.1	Brochothrix phage A9
764562	GCA_000891715.1	Brochothrix phage BL3
764561	GCA_000890735.1	Brochothrix phage NF5
12302	GCA_000854965.1	Brome mosaic virus
42631	GCA_000860645.1	Brome streak mosaic virus
936005	GCA_000890415.1	Bromus catharticus striate mosaic virus
1590154	GCA_000930895.1	Bromus-associated circular DNA virus 1
1590155	GCA_000930255.1	Bromus-associated circular DNA virus 2
1590156	GCA_000931075.1	Bromus-associated circular DNA virus 3
1590172	GCA_000929195.1	Bromus-associated circular DNA virus 4
667093	GCA_000889955.1	Broome virus
2170139	GCA_003032745.1	Brown greater galago prosimian foamy virus
1667364	GCA_002595225.1	Brucella phage 02_141
1667365	GCA_002595205.1	Brucella phage 02_19
1667366	GCA_002595505.1	Brucella phage 1066_141

1667367	GCA_002595485.1	Brucella phage 1066_19
1667368	GCA_002595305.1	Brucella phage 110_141
1667369	GCA_002595285.1	Brucella phage 110_19
1667370	GCA_002595265.1	Brucella phage 11sa_141
1667371	GCA_002595245.1	Brucella phage 11sa_19
1667372	GCA_002595345.1	Brucella phage 141_141
1667373	GCA_002595325.1	Brucella phage 141_19
1667374	GCA_002595385.1	Brucella phage 177_141
1667375	GCA_002595365.1	Brucella phage 177_19
1667376	GCA_002595425.1	Brucella phage 281_141
1667377	GCA_002595405.1	Brucella phage 281_19
1667378	GCA_002595465.1	Brucella phage 544_141
1667379	GCA_002595445.1	Brucella phage 544_19
1718278	GCA_001754265.1	Brucella phage BiPBO1
1308869	GCA_002755555.1	Brucella phage Bk
1277896	GCA_002633075.1	Brucella phage Bk2
1401446	GCA_002595145.1	Brucella phage F1
1279070	GCA_002633125.1	Brucella phage Fi
1308870	GCA_002755575.1	Brucella phage Fz
1920338	GCA_002617205.1	Brucella phage Iz
1133293	GCA_000902275.1	Brucella phage Pr
1277895	GCA_002745615.1	Brucella phage R/C
1277895	GCA_003146825.1	Brucella phage R/C
1308871	GCA_002755595.1	Brucella phage S708
1133292	GCA_000900615.1	Brucella phage Tb
1133292	GCA_002595105.1	Brucella phage Tb
1133292	GCA_002595165.1	Brucella phage Tb
1667380	GCA_002595545.1	Brucella phage Tb_141
1667381	GCA_002595525.1	Brucella phage Tbilisi
1667382	GCA_002595585.1	Brucella phage V_141
1667383	GCA_002595565.1	Brucella phage V_19
1277893	GCA_002595125.1	Brucella phage Wb
1277893	GCA_002595185.1	Brucella phage Wb
1679445	GCA_002117675.1	Bruges virus
402399	GCA_000879495.1	Brugmansia mild mottle virus
1239452	GCA_000906515.1	Brugmansia mosaic virus
487155	GCA_000889295.1	Brugmansia suaveolens mottle virus
1503289	GCA_001503155.1	BtMr-AlphaCoV/SAX2011
1503291	GCA_001505415.1	BtNv-AlphaCoV/SC2013
1503292	GCA_001504755.1	BtRf-AlphaCoV/HuB2013
1503293	GCA_001501755.1	BtRf-AlphaCoV/YN2012
202910	GCA_002814715.1	Bubaline alphaherpesvirus 1
133789	GCA_000842245.1	Budgerigar fledgling disease virus - 1
1391667	GCA_000924255.1	Bufavirus-3
2282206	GCA_004130925.1	Bufonid herpesvirus 1
48541	GCA_002889335.1	Buggy Creek virus
572288	GCA_002816215.1	Bulbul coronavirus HKU11-934
565995	GCA_000889155.1	Bundibugyo ebolavirus
35304	GCA_000849925.1	Bunyamwera virus
1453404	GCA_003032555.1	Burana virus

1324959	GCA_000908875.1	Burdock mottle virus
1916647	GCA_001866205.1	Burke-Gilman virus
1916647	GCA_001870935.1	Burke-Gilman virus
1133022	GCA_000898995.1	Burkholderia phage AH2
348137	GCA_000865925.1	Burkholderia phage Bcep176
279530	GCA_000844905.1	Burkholderia phage BcepB1A
437329	GCA_000873805.1	Burkholderia phage BcepGomr
242861	GCA_000840725.1	Burkholderia phage BcepNazgul
1514988	GCA_002756315.1	Burkholderia phage BEK
1432428	GCA_002604225.1	Burkholderia phage Bp-AMP1
1437328	GCA_002604265.1	Burkholderia phage Bp-AMP2
1673729	GCA_002606825.1	Burkholderia phage Bp-AMP3
1437329	GCA_002604285.1	Burkholderia phage Bp-AMP4
1401297	GCA_000914995.1	Burkholderia phage JG068
1132026	GCA_000897395.1	Burkholderia phage KL1
910474	GCA_000893295.1	Burkholderia phage KL3
557289	GCA_000881475.1	Burkholderia phage KS10
910475	GCA_000891775.1	Burkholderia phage KS14
910473	GCA_000891735.1	Burkholderia phage KS5
335797	GCA_000885155.1	Burkholderia phage KS9
2321389	GCA_003668395.1	Burkholderia phage phiE058
2321388	GCA_003668375.1	Burkholderia phage phiE131
1235712	GCA_002755455.1	Burkholderia phage phiX216
1282994	GCA_000908615.1	Burkholderia phage ST79
1636201	GCA_002605605.1	Burkholderia phage vB_BceM_AP3
2115967	GCA_003094055.1	Burkholderia phage vB_BmuP_KL4
244310	GCA_000845445.1	Burkholderia virus Bcep1
242527	GCA_000840865.2	Burkholderia virus Bcep22
260373	GCA_000844585.1	Burkholderia virus Bcep43
209052	GCA_000845425.1	Burkholderia virus Bcep781
279280	GCA_000843605.1	Burkholderia virus BcepC6B
417280	GCA_000873005.1	Burkholderia virus BcepF1
644524	GCA_000882995.1	Burkholderia virus Bcepil02
1195073	GCA_000903775.1	Burkholderia virus Bcepmigl
264729	GCA_000841805.1	Burkholderia virus BcepMu
446807	GCA_000873385.1	Burkholderia virus BcepNY3
1136535	GCA_000899095.1	Burkholderia virus DC1
255131	GCA_000846305.1	Burkholderia virus phi1026b
332032	GCA_000861045.1	Burkholderia virus phi52237
431891	GCA_000893155.1	Burkholderia virus phi6442
431892	GCA_000871505.1	Burkholderia virus phiE122
180504	GCA_000840845.1	Burkholderia virus phiE125
431893	GCA_000870585.1	Burkholderia virus phiE202
431894	GCA_000873105.1	Burkholderia virus phiE255
64304	GCA_000872985.1	Bussuquara virus
1394033	GCA_000914155.1	Butcherbird polyomavirus
666859	GCA_000886075.1	Butterbur mosaic virus
74320	GCA_000914375.1	Buzura suppressaria nucleopolyhedrovirus
35310	GCA_002118905.1	Bwamba orthobunyavirus
60879	GCA_002829845.1	Cabassou virus

345184	GCA_002867425.1	Cabbage leaf curl Jamaica virus
51336	GCA_000838405.1	Cabbage leaf curl virus
2056878	GCA_004131865.1	Cacao Bacilliform SriLanka Virus
1940252	GCA_002004675.1	Cacao mild mosaic virus
1960255	GCA_002819325.1	Cacao swollen shoot CD virus
2056879	GCA_004131745.1	Cacao swollen shoot CE virus
2056880	GCA_004131765.1	Cacao swollen shoot Ghana J virus
2056881	GCA_004131785.1	Cacao swollen shoot Ghana K virus
2056882	GCA_004131805.1	Cacao swollen shoot Ghana L virus
2056884	GCA_004131825.1	Cacao swollen shoot Ghana N virus
2056886	GCA_004131845.1	Cacao swollen shoot Ghana R virus
1960254	GCA_002819345.1	Cacao swollen shoot Togo A virus
31559	GCA_000844305.1	Cacao swollen shoot virus
2169726	GCA_002005015.1	Cacao yellow vein banding virus
64305	GCA_000954535.1	Cacipacore virus
229030	GCA_000883515.1	Cactus mild mottle virus
112227	GCA_000856405.1	Cactus virus X
693272	GCA_000889395.1	Cafeteria roenbergensis virus BV-PW1
1932923	GCA_002954985.1	Cafeteriavirus-dependent mavirus
1198147	GCA_000897635.1	Caladenia virus A
1692244	GCA_001274205.1	Calanoida sp. copepod associated circular virus
2036768	GCA_002629785.1	Caldibacillus phage CBP1
2021957	GCA_003729475.1	Caledonia beadlet anemone Nora virus-like virus 1
2169993	GCA_000856465.1	Cali mammarenavirus
204928	GCA_000908175.1	Calibrachoa mottle virus
243550	GCA_000859525.1	Calicivirus isolate TCG
520973	GCA_000883855.1	Calicivirus pig/AB90/CAN
190239	GCA_000851625.1	Calicivirus strain NB
35305	GCA_003972565.1	California encephalitis virus
1073950	GCA_002827325.1	California sea lion adeno-associated virus 1
943083	GCA_000920015.1	California sea lion adenovirus 1
1073959	GCA_002827225.1	California sea lion bocavirus 1
1073961	GCA_002827245.1	California sea lion bocavirus 3
715223	GCA_000887115.1	California sea lion polyomavirus 1
309542	GCA_002890515.1	Calla lily chlorotic spot virus
1692245	GCA_001274445.1	Callinectes ornatus blue crab associated circular virus
1692246	GCA_001274065.1	Callinectes sapidus associated circular virus
1811230	GCA_001629885.2	Callinectes sapidus reovirus 1
1811230	GCA_001629885.1	Callinectes sapidus reovirus 1
1886606	GCA_001714495.1	Callistephus mottle virus
106331	GCA_000843305.1	Callitrichine gammaherpesvirus 3 (Marmoset lymphocrypt
1699095	GCA_001500975.1	Camel alphacoronavirus
2169876	GCA_003033415.1	Camel associated drosmacovirus 1
2169877	GCA_003033425.1	Camel associated drosmacovirus 2
2170105	GCA_003033505.1	Camel associated porprismacovirus 1
2170106	GCA_003033515.1	Camel associated porprismacovirus 2
2170107	GCA_003033525.1	Camel associated porprismacovirus 3
2170108	GCA_003033535.1	Camel associated porprismacovirus 4
2122733	GCA_004132585.1	Camellia chlorotic dwarf-associated virus
2069319	GCA_004128695.1	Camellia oleifera amalgavirus 1

203173	GCA_000839105.1	Camelpox virus M-96
996650	GCA_000890775.1	Camelus dromedarius papillomavirus 1
996651	GCA_000892415.1	Camelus dromedarius papillomavirus 2
10484	GCA_000867225.1	Campoletis sonorensis ichnovirus
1765754	GCA_001579375.1	Camponotus nipponicus virus
1608533	GCA_001028985.1	Camponotus yamaokai virus
2047877	GCA_002956545.1	Campylobacter phage A110a
2047878	GCA_002956555.1	Campylobacter phage A110b
2047880	GCA_002956575.1	Campylobacter phage A112b
2047881	GCA_002956585.1	Campylobacter phage A113
2047882	GCA_002956595.1	Campylobacter phage A115a
2047883	GCA_002956605.1	Campylobacter phage A116
2047884	GCA_002956615.1	Campylobacter phage A118
2047885	GCA_002956625.1	Campylobacter phage A119
2047886	GCA_002956635.1	Campylobacter phage A11a
2047887	GCA_002956645.1	Campylobacter phage A120
2047888	GCA_002956655.1	Campylobacter phage A121
2047889	GCA_002956665.1	Campylobacter phage A123
2047891	GCA_002956685.1	Campylobacter phage A127
2047892	GCA_002956695.1	Campylobacter phage A12a
2047893	GCA_002956705.1	Campylobacter phage A131
2047894	GCA_002956715.1	Campylobacter phage A132
2047895	GCA_002956725.1	Campylobacter phage A134
2047896	GCA_002956735.1	Campylobacter phage A135
2047897	GCA_002956745.1	Campylobacter phage A136
2047898	GCA_002956755.1	Campylobacter phage A138
2047899	GCA_002956765.1	Campylobacter phage A139
2047900	GCA_002956775.1	Campylobacter phage A13a
2047901	GCA_002956785.1	Campylobacter phage A13b
2047902	GCA_002956795.1	Campylobacter phage A140
2047903	GCA_002956805.1	Campylobacter phage A141
2047904	GCA_002956815.1	Campylobacter phage A142
2047905	GCA_002956825.1	Campylobacter phage A143
2047907	GCA_002956845.1	Campylobacter phage A145
2047908	GCA_002956855.1	Campylobacter phage A147
2047909	GCA_002956865.1	Campylobacter phage A148
2047910	GCA_002956875.1	Campylobacter phage A14a
2047911	GCA_002956885.1	Campylobacter phage A14b
2047912	GCA_002956895.1	Campylobacter phage A150
2496548	GCA_002956915.1	Campylobacter phage A15b
2047915	GCA_002956925.1	Campylobacter phage A16a
2047916	GCA_002956935.1	Campylobacter phage A18a
2047917	GCA_002956945.1	Campylobacter phage B14
2047918	GCA_002956955.1	Campylobacter phage B15
2047919	GCA_002956965.1	Campylobacter phage C10
2047920	GCA_002956975.1	Campylobacter phage C11
2047921	GCA_002956985.1	Campylobacter phage C12
2047923	GCA_002957005.1	Campylobacter phage C14
2047924	GCA_002957015.1	Campylobacter phage C15
2047925	GCA_002957025.1	Campylobacter phage C2

2047926	GCA_002957035.1	Campylobacter phage C3
2047927	GCA_002957045.1	Campylobacter phage C4
2047928	GCA_002957055.1	Campylobacter phage C5
2047929	GCA_002957065.1	Campylobacter phage C7
2047930	GCA_002957075.1	Campylobacter phage C8
2047931	GCA_002957085.1	Campylobacter phage C9
1470458	GCA_002755995.1	Campylobacter phage CJIE4-1
1470459	GCA_002756015.1	Campylobacter phage CJIE4-2
1470460	GCA_002756035.1	Campylobacter phage CJIE4-3
1470461	GCA_002756055.1	Campylobacter phage CJIE4-4
1470462	GCA_002756075.1	Campylobacter phage CJIE4-5
2506428	GCA_004139195.1	Campylobacter phage CP20
1229752	GCA_000899455.1	Campylobacter phage CP30A
1340809	GCA_002755815.1	Campylobacter phage CP8
2047932	GCA_002957095.1	Campylobacter phage D#
2047933	GCA_002957105.1	Campylobacter phage D1
2047934	GCA_002957115.1	Campylobacter phage E6
1541686	GCA_001884615.1	Campylobacter phage PC14
1541690	GCA_002604945.1	Campylobacter phage PC5
1904491	GCA_002614145.1	Campylobacter phage vB_CjeM_Los1
1190451	GCA_000902535.1	Campylobacter virus CP21
722417	GCA_001308835.1	Campylobacter virus CP220
990550	GCA_002986005.1	Campylobacter virus CP81
722418	GCA_001308555.1	Campylobacter virus CPt10
1110702	GCA_000895115.1	Campylobacter virus CPX
1006972	GCA_001308675.2	Campylobacter virus IBB35
1006972	GCA_001308675.1	Campylobacter virus IBB35
934027	GCA_000893555.1	Campylobacter virus NCTC12673
1715288	GCA_001305565.2	Canary bornavirus 1
1715290	GCA_002366325.1	Canary bornavirus 2
1715291	GCA_000921835.1	Canary bornavirus 3
142661	GCA_000846785.1	Canary circovirus
881945	GCA_000896095.1	Canary polyomavirus
44088	GCA_000841685.1	Canarypox virus
1388882	GCA_000929895.1	Cangyuan orthoreovirus
170325	GCA_001646155.1	Canid alphaherpesvirus 1
10512	GCA_000845925.1	Canine adenovirus 1
10512	GCA_000857845.1	Canine adenovirus type 1
10514	GCA_000856885.1	Canine adenovirus type 2
1157338	GCA_000973215.1	Canine astrovirus
1511885	GCA_000905315.1	Canine bocavirus 1
1194757	GCA_000906275.1	Canine circovirus
1194757	GCA_004028875.1	Canine circovirus
1194757	GCA_004029295.1	Canine circovirus
1194757	GCA_004028955.1	Canine circovirus
1194757	GCA_004029315.1	Canine circovirus
1194757	GCA_004028975.1	Canine circovirus
1194757	GCA_004029335.1	Canine circovirus
1194757	GCA_004028995.1	Canine circovirus
1194757	GCA_004037995.1	Canine circovirus

1194757	GCA_004037855.1	Canine circovirus
1194757	GCA_004029015.1	Canine circovirus
1194757	GCA_004033875.1	Canine circovirus
1194757	GCA_004029035.1	Canine circovirus
1194757	GCA_004033455.1	Canine circovirus
1194757	GCA_004037875.1	Canine circovirus
1194757	GCA_004037915.1	Canine circovirus
1194757	GCA_004029055.1	Canine circovirus
1194757	GCA_004029075.1	Canine circovirus
1194757	GCA_004037835.1	Canine circovirus
1194757	GCA_004029095.1	Canine circovirus
1194757	GCA_004037955.1	Canine circovirus
1194757	GCA_004029115.1	Canine circovirus
1194757	GCA_004029135.1	Canine circovirus
1194757	GCA_004037895.1	Canine circovirus
1194757	GCA_004029155.1	Canine circovirus
1194757	GCA_004037795.1	Canine circovirus
1194757	GCA_004029175.1	Canine circovirus
1194757	GCA_004029195.1	Canine circovirus
1194757	GCA_004037975.1	Canine circovirus
1194757	GCA_004037935.1	Canine circovirus
1194757	GCA_004029215.1	Canine circovirus
1194757	GCA_004029235.1	Canine circovirus
1194757	GCA_004028895.1	Canine circovirus
1194757	GCA_004029255.1	Canine circovirus
1194757	GCA_004028915.1	Canine circovirus
1194757	GCA_004037815.1	Canine circovirus
1194757	GCA_004029275.1	Canine circovirus
1194757	GCA_004028935.1	Canine circovirus
1194757	GCA_004059475.1	Canine circovirus
1194757	GCA_004038155.1	Canine circovirus
1194757	GCA_004059615.1	Canine circovirus
1194757	GCA_004059435.1	Canine circovirus
1194757	GCA_004038195.1	Canine circovirus
1194757	GCA_004045995.1	Canine circovirus
1194757	GCA_004059575.1	Canine circovirus
1194757	GCA_004059355.1	Canine circovirus
1194757	GCA_004038015.1	Canine circovirus
1194757	GCA_004038035.1	Canine circovirus
1194757	GCA_004038235.1	Canine circovirus
1194757	GCA_004038055.1	Canine circovirus
1194757	GCA_004042195.1	Canine circovirus
1194757	GCA_004059495.1	Canine circovirus
1194757	GCA_004038115.1	Canine circovirus
1194757	GCA_004038255.1	Canine circovirus
1194757	GCA_004038175.1	Canine circovirus
1194757	GCA_004038215.1	Canine circovirus
1194757	GCA_004059535.1	Canine circovirus
1194757	GCA_004059375.1	Canine circovirus
1194757	GCA_004059515.1	Canine circovirus

1194757	GCA_004061975.1	Canine circovirus
1194757	GCA_004059415.1	Canine circovirus
1194757	GCA_004059455.1	Canine circovirus
1194757	GCA_004059335.1	Canine circovirus
1194757	GCA_004061575.1	Canine circovirus
1194757	GCA_004038075.1	Canine circovirus
1194757	GCA_004059395.1	Canine circovirus
1194757	GCA_004059595.1	Canine circovirus
1194757	GCA_004043315.1	Canine circovirus
1194757	GCA_004059295.1	Canine circovirus
1194757	GCA_004038095.1	Canine circovirus
1194757	GCA_004059555.1	Canine circovirus
1194757	GCA_004059315.1	Canine circovirus
1194757	GCA_004038135.1	Canine circovirus
1194757	GCA_004077155.1	Canine circovirus
1194757	GCA_004077175.1	Canine circovirus
1194757	GCA_004069095.1	Canine circovirus
2017709	GCA_003849025.1	Canine feces-associated gemycircularvirus
1836608	GCA_002194365.1	Canine kobuvirus
329639	GCA_000863725.1	Canine minute virus
329639	GCA_003033225.1	Canine minute virus
11232	GCA_000854065.1	Canine morbillivirus
1899573	GCA_003179935.1	Canine Papillomavirus 19
2304619	GCA_004133925.1	Canine papillomavirus 21
2304620	GCA_004133945.1	Canine papillomavirus 22
658423	GCA_003178315.1	Canine papillomavirus 7
10788	GCA_000848925.1	Canine parvovirus
1150861	GCA_000908335.1	Canine picodicistrovirus
1196647	GCA_000895375.1	Canine picornavirus
557241	GCA_002635175.1	Canine rotavirus A79-10/G3P[3]
1176484	GCA_000851085.1	Canine vesivirus
1087109	GCA_000896375.1	Canis familiaris papillomavirus 10
1091166	GCA_003179175.1	Canis familiaris papillomavirus 11
1194330	GCA_003179315.1	Canis familiaris papillomavirus 12
1226723	GCA_000920395.1	Canis familiaris papillomavirus 13
1236767	GCA_000902855.1	Canis familiaris papillomavirus 14
1272519	GCA_003179475.1	Canis familiaris papillomavirus 15
1619253	GCA_000954895.1	Canis familiaris papillomavirus 16
1778550	GCA_003179755.1	Canis familiaris papillomavirus 17
1816242	GCA_003179775.1	Canis familiaris papillomavirus 18
292792	GCA_000844385.1	Canis familiaris papillomavirus 2
1843776	GCA_003179875.1	Canis familiaris papillomavirus 20
360397	GCA_000869325.1	Canis familiaris papillomavirus 3
464980	GCA_000878975.1	Canis familiaris papillomavirus 4
658422	GCA_003178335.1	Canis familiaris papillomavirus 5
1513269	GCA_000884335.1	Canis familiaris papillomavirus 6
1081055	GCA_000894855.1	Canis familiaris papillomavirus 8
1087108	GCA_000894895.1	Canis familiaris papillomavirus 9
1980633	GCA_002118645.1	Canis familiaris polyomavirus 1
419782	GCA_002819365.1	Canna yellow mottle virus

2044517	GCA_001684505.1	Canna yellow mottle-associated virus
433462	GCA_000885315.1	Canna yellow streak virus
1115692	GCA_001745435.1	Cannabis cryptic virus
1115692	GCA_002868155.1	Cannabis cryptic virus
1980463	GCA_002145545.1	Cano Delgadito orthohantavirus
1980464	GCA_002119025.1	Cao Bang orthohantavirus
2116599	GCA_004117415.1	Cape gooseberry ilarvirus 1
35312	GCA_002118425.1	Capim virus
338903	GCA_000868145.1	Capra hircus papillomavirus 1
1311277	GCA_000911775.1	Capraria yellow spot Yucatan virus
470214	GCA_000874665.1	Capreolus capreolus papillomavirus 1
11660	GCA_000857525.1	Caprine arthritis encephalitis virus
1452516	GCA_000915315.1	Caprine kobuvirus
1529392	GCA_001443845.1	Caprine parainfluenza virus 3
204932	GCA_002645735.1	Caprine rotavirus A
204932	GCA_002681275.1	Caprine rotavirus A
2069320	GCA_004128655.1	Capsicum annuum amalgavirus 1
163325	GCA_000868405.1	Capsicum chlorosis virus
239239	GCA_002815975.1	Carajas virus
192196	GCA_002118585.1	Caraparu virus
31711	GCA_000848365.1	Cardamine chlorotic fleck virus
2169724	GCA_000913955.1	Cardamom bushy dwarf alphasatellite
104637	GCA_003180955.1	Cardamom mosaic virus
1891718	GCA_000903895.1	Cardioderma cor polyomavirus 1
497865	GCA_000879055.1	Cardiospermum yellow leaf curl betasatellite
485241	GCA_000880755.1	Caretta caretta papillomavirus 1
1542743	GCA_000926215.1	Caribou feces-associated gemycircularvirus
1980625	GCA_002146105.1	Carnation cryptic virus 3
10640	GCA_000845845.1	Carnation etched ring virus
39443	GCA_000856765.1	Carnation Italian ringspot virus
11986	GCA_000854705.1	Carnation mottle virus
12268	GCA_000852765.1	Carnation ringspot virus
940280	GCA_000913155.1	Carnation yellow fleck virus
1606502	GCA_001430435.1	Carollia perspicillata polyomavirus 1
1439369	GCA_000915915.1	Carp picornavirus 1
696863	GCA_000850305.1	Carp sprivivirus
1425363	GCA_000925115.1	Carrot Ch virus 1
1425362	GCA_000925095.1	Carrot Ch virus 2
589918	GCA_002867815.1	Carrot cryptic virus
47736	GCA_000856805.1	Carrot mottle mimic virus
570949	GCA_001689795.1	Carrot mottle mimic virus satellite RNA
68033	GCA_000893875.1	Carrot mottle virus
570950	GCA_001689875.1	Carrot mottle virus satellite RNA
680112	GCA_002817415.1	Carrot necrotic dieback virus
67962	GCA_000851505.1	Carrot red leaf luteovirus associated RNA
66200	GCA_000854305.1	Carrot red leaf virus
114922	GCA_000924455.1	Carrot thin leaf virus
1425364	GCA_000927615.2	Carrot torradovirus 1
656190	GCA_000884075.1	Carrot yellow leaf virus
1223561	GCA_000898535.1	CAS virus

180586	GCA_000841125.1	Casphalia extranea densovirus
1985374	GCA_000919515.1	Cassava associated gemycircularvirus 1
1985374	GCA_003849045.1	Cassava associated gemycircularvirus 1
137758	GCA_000884835.1	Cassava brown streak virus
39046	GCA_000849345.1	Cassava common mosaic virus
1464778	GCA_000929575.1	Cassava Ivorian bacilliform virus
1231298	GCA_000898675.1	Cassava mosaic Madagascar alphasatellite
1125764	GCA_000895455.1	Cassava mosaic Madagascar virus
1958957	GCA_002008795.1	Cassava satellite virus
38062	GCA_000838625.1	Cassava vein mosaic virus
561576	GCA_000885215.1	Cassava virus C
1977392	GCA_002116295.1	Cassava virus X
300879	GCA_000861785.1	Cassia yellow blotch virus
1969351	GCA_002149845.1	Castlerea virus
1352235	GCA_000914255.1	Castor canadensis papillomavirus 1
1482319	GCA_000919795.1	Casuarina virus
1495866	GCA_000922635.1	Cat Que virus
369752	GCA_001029025.1	Catharanthus mosaic virus
1076345	GCA_000928135.1	Catharanthus yellow mosaic virus
1850906	GCA_001654205.1	Catopsilia pomona nucleopolyhedrovirus
2077298	GCA_004131905.1	Cattle blood-associated circovirus-like virus
2077298	GCA_004131885.1	Cattle blood-associated circovirus-like virus
2077298	GCA_004382845.1	Cattle blood-associated circovirus-like virus
2077296	GCA_003848565.1	Cattle blood-associated gemycircularvirus
2077296	GCA_003848585.1	Cattle blood-associated gemycircularvirus
1678225	GCA_002831305.1	Catu virus
1667230	GCA_002817735.1	Caucasus prunus virus
2202568	GCA_003652845.1	Caudovirales sp.
2202568	GCA_003657045.1	Caudovirales sp.
2202568	GCA_003657545.1	Caudovirales sp.
10641	GCA_000848745.1	Cauliflower mosaic virus
1959735	GCA_002619445.1	Caulobacter phage Ccr10
1959736	GCA_002619465.1	Caulobacter phage Ccr2
1959737	GCA_002619485.1	Caulobacter phage Ccr29
1959738	GCA_002619505.1	Caulobacter phage Ccr32
1959739	GCA_002619525.1	Caulobacter phage Ccr34
1959740	GCA_002619545.1	Caulobacter phage Ccr5
2283269	GCA_003443255.1	Caulobacter phage CcrBL10
2283270	GCA_003443295.1	Caulobacter phage CcrBL9
1211640	GCA_000899635.1	Caulobacter phage CcrColossus
2283271	GCA_003443275.1	Caulobacter phage CcrPW
2283272	GCA_003443315.1	Caulobacter phage CcrSC
1357714	GCA_000927355.1	Caulobacter phage Cr30
2340873	GCA_003613235.1	Caulobacter phage Kronos
2024607	GCA_002743595.1	Caulobacter phage Lullwater
1701809	GCA_002149385.1	Caulobacter phage Percy
767473	GCA_000903235.1	Caulobacter phage phiCb5
1675600	GCA_002607065.1	Caulobacter phage Sansa
1675601	GCA_002607085.1	Caulobacter phage Seuss
1211641	GCA_000901575.1	Caulobacter virus Karma

1211642	GCA_000903095.1	Caulobacter virus Magneto
1204537	GCA_000900535.1	Caulobacter virus phiCbK
1204537	GCA_002602425.1	Caulobacter virus phiCbK
1211643	GCA_000900555.1	Caulobacter virus Rogue
1211644	GCA_000899655.1	Caulobacter virus Swift
1041929	GCA_000891195.1	Cavally virus
33706	GCA_000904135.1	Caviid betaherpesvirus 2
1236392	GCA_000902195.1	Cebus albifrons polyomavirus 1
1221391	GCA_000924595.1	Cedar virus
1903266	GCA_001995575.1	Cedratvirus A11
2023205	GCA_900205285.1	Cedratvirus lausannensis
2126979	GCA_900323555.1	Cedratvirus Zaza IHUMI
1197951	GCA_000898435.1	Celeribacter phage P12053L
112436	GCA_000893475.1	Celery mosaic virus
31658	GCA_000862225.1	Cell fusing agent virus
1327981	GCA_000910535.1	Cellulophaga phage phi10:1
1327976	GCA_000909675.1	Cellulophaga phage phi12:1
1327969	GCA_000910435.1	Cellulophaga phage phi12:2
1327979	GCA_002603585.1	Cellulophaga phage phi12:3
1327987	GCA_000910495.1	Cellulophaga phage phi12a:1
1327992	GCA_002755755.1	Cellulophaga phage phi13:1
1328030	GCA_000907995.1	Cellulophaga phage phi13:2
1327990	GCA_000909615.1	Cellulophaga phage phi14:2
1327980	GCA_000909655.1	Cellulophaga phage phi17:1
1327972	GCA_000908055.1	Cellulophaga phage phi17:2
1747283	GCA_002603565.1	Cellulophaga phage phi17:2_18
1327982	GCA_000911595.1	Cellulophaga phage phi18:1
1327995	GCA_002603625.1	Cellulophaga phage phi18:2
1327983	GCA_000910515.1	Cellulophaga phage phi18:3
1327997	GCA_002755775.1	Cellulophaga phage phi18:4
1327970	GCA_000908935.1	Cellulophaga phage phi19:1
1327984	GCA_002755735.1	Cellulophaga phage phi19:2
1327971	GCA_000909575.1	Cellulophaga phage phi19:3
756279	GCA_002600505.1	Cellulophaga phage phi3:1
1327977	GCA_000909635.1	Cellulophaga phage phi38:1
1327999	GCA_002600485.1	Cellulophaga phage phi38:2
1327993	GCA_000908015.1	Cellulophaga phage phi39:1
1327973	GCA_002600445.1	Cellulophaga phage phi3ST:2
1328029	GCA_000910475.1	Cellulophaga phage phi4:1
1747284	GCA_002757055.1	Cellulophaga phage phi4:1_13
1747285	GCA_002757075.1	Cellulophaga phage phi4:1_18
1327975	GCA_002603605.1	Cellulophaga phage phi40:1
1327974	GCA_000908035.1	Cellulophaga phage phi46:1
1327985	GCA_000911615.1	Cellulophaga phage phi46:3
756281	GCA_002600525.1	Cellulophaga phage phi47:1
1328028	GCA_002755795.1	Cellulophaga phage phi48:1
1327968	GCA_000908955.1	Cellulophaga phage phi48:2
756280	GCA_000904495.1	Cellulophaga phage phiSM
756280	GCA_002600465.1	Cellulophaga phage phiSM
756282	GCA_000906115.1	Cellulophaga phage phiST

756282	GCA_002746075.1	Cellulophaga phage phiST
1916650	GCA_001866225.1	Centovirus AC
2170194	GCA_003032755.1	Central cimpanzee simian foamy virus
1129032	GCA_000896055.1	Centrosema yellow spot virus
1908806	GCA_001792745.1	Ceratobasidium endornavirus A
1908807	GCA_001792725.1	Ceratobasidium endornavirus B
1908813	GCA_002149885.1	Ceratobasidium endornavirus C
1908808	GCA_001777285.1	Ceratobasidium endornavirus D
1908811	GCA_001792765.1	Ceratobasidium endornavirus G
235434	GCA_000872885.1	Ceratocystis polonica partitivirus
235434	GCA_002868175.1	Ceratocystis polonica partitivirus
674982	GCA_000879375.1	Ceratocystis resinifera virus 1
10317	GCA_000846965.1	Cercopithecine alphaherpesvirus 2
35246	GCA_000848845.1	Cercopithecine alphaherpesvirus 9 (Simian varicella virus)
50292	GCA_000884915.1	Cercopithecine betaherpesvirus 5
1236395	GCA_000929995.1	Cercopithecus erythrotis polyomavirus 1
228582	GCA_000848445.1	Cereal yellow dwarf virus RPS
2170100	GCA_000853365.1	Cereal yellow dwarf virus RPV
191032	GCA_000840025.1	Cereal yellow dwarf virus-RPV satellite RNA
1747359	GCA_003033185.1	Cervus elaphus papillomavirus type 2
1434070	GCA_001646555.1	Cervus papillomavirus 2
175814	GCA_000845725.1	Cestrum yellow leaf curling virus
1158189	GCA_002145805.1	Chaco virus
1276181	GCA_000904955.1	Chaerephon polyomavirus 1
2170140	GCA_000863905.1	Chaetoceros protobacilladnavirus 1
2170142	GCA_000892315.1	Chaetoceros protobacilladnavirus 3
2170143	GCA_000887835.1	Chaetoceros protobacilladnavirus 4
1290581	GCA_003028895.1	Chaetoceros setoense DNA virus
2169725	GCA_000882095.1	Chaetoceros socialis forma radians RNA virus 1
1382995	GCA_000916915.1	Chaetoceros sp. DNA virus 7
1516127	GCA_000930655.1	Chaetoceros tenuissimus DNA virus type-II
497136	GCA_002817455.1	Chaetoceros tenuissimus RNA virus 01
1516128	GCA_000930635.1	Chaetoceros tenuissimus RNA virus type-II
267285	GCA_000858705.1	Chalara elegans RNA Virus 1
1843766	GCA_001646415.1	Chamois faeces associated circular DNA virus 1
11272	GCA_000906815.1	Chandipura virus
629725	GCA_000891915.1	Chandiru virus
1922764	GCA_001963495.1	Changjiang astro-like virus
1922765	GCA_001961815.1	Changjiang crawfish virus 1
1922766	GCA_001962555.1	Changjiang crawfish virus 2
1922767	GCA_001964075.1	Changjiang crawfish virus 3
1922768	GCA_001923475.1	Changjiang crawfish virus 4
1922769	GCA_001963475.1	Changjiang crawfish virus 5
1922770	GCA_001961795.1	Changjiang crawfish virus 6
1922771	GCA_001962535.1	Changjiang crawfish virus 7
1922772	GCA_001964055.1	Changjiang hepe-like virus 1
1922776	GCA_001963455.1	Changjiang narna-like virus 1
1922777	GCA_001961775.1	Changjiang narna-like virus 2
1922778	GCA_001962515.1	Changjiang narna-like virus 3
1922779	GCA_001964035.1	Changjiang narna-like virus 4

1922782	GCA_001924615.1	Changjiang picorna-like virus 1
1922783	GCA_001963435.1	Changjiang picorna-like virus 10
1922784	GCA_001961755.1	Changjiang picorna-like virus 11
1922785	GCA_001962495.1	Changjiang picorna-like virus 12
1922786	GCA_001964015.1	Changjiang picorna-like virus 13
1922787	GCA_001963415.1	Changjiang picorna-like virus 14
1922788	GCA_001961735.1	Changjiang picorna-like virus 15
1922789	GCA_001962475.1	Changjiang picorna-like virus 16
1922791	GCA_001963995.1	Changjiang picorna-like virus 2
1922792	GCA_001963395.1	Changjiang picorna-like virus 3
1922793	GCA_001964415.1	Changjiang picorna-like virus 4
1922794	GCA_001965115.1	Changjiang picorna-like virus 5
1922795	GCA_001964275.1	Changjiang picorna-like virus 6
1922796	GCA_001965935.1	Changjiang picorna-like virus 7
1922797	GCA_001964395.1	Changjiang picorna-like virus 8
1922798	GCA_001962735.1	Changjiang picorna-like virus 9
1922799	GCA_001964255.1	Changjiang polero-like virus 1
1922800	GCA_001965915.1	Changjiang sobemo-like virus 1
1922801	GCA_001964375.1	Changjiang sobemo-like virus 2
1922802	GCA_001962715.1	Changjiang tombus-like virus 1
1922803	GCA_001964235.1	Changjiang tombus-like virus 10
1922804	GCA_001965895.1	Changjiang tombus-like virus 11
1922805	GCA_001964355.1	Changjiang tombus-like virus 12
1922807	GCA_001962695.1	Changjiang tombus-like virus 14
1922808	GCA_001923255.1	Changjiang tombus-like virus 15
1922809	GCA_001964215.1	Changjiang tombus-like virus 16
1922810	GCA_001965875.1	Changjiang tombus-like virus 17
1922811	GCA_001961935.1	Changjiang tombus-like virus 18
1922812	GCA_001962675.1	Changjiang tombus-like virus 19
1922813	GCA_001964195.1	Changjiang tombus-like virus 2
1922814	GCA_001965855.1	Changjiang tombus-like virus 20
1922815	GCA_001961915.1	Changjiang tombus-like virus 21
1922816	GCA_001962655.1	Changjiang tombus-like virus 22
1922817	GCA_001964175.1	Changjiang tombus-like virus 3
1922818	GCA_001965835.1	Changjiang tombus-like virus 4
1922819	GCA_001961895.1	Changjiang tombus-like virus 5
1922820	GCA_001962635.1	Changjiang tombus-like virus 6
1922821	GCA_001964155.1	Changjiang tombus-like virus 7
1922822	GCA_001965815.1	Changjiang tombus-like virus 8
1922823	GCA_001961875.1	Changjiang tombus-like virus 9
1922824	GCA_001962615.1	Changjiang zhaovirus-like virus 1
1922826	GCA_001966675.1	Changping earthworm virus 1
1922827	GCA_002288755.1	Changping earthworm virus 2
1608044	GCA_001440855.1	Changping Tick Virus 2
1608045	GCA_001440935.1	Changping Tick Virus 3
40052	GCA_000911195.1	Changuinola virus
631267	GCA_000895475.1	Chaoyang virus
499556	GCA_000879235.1	Chapare mammarenavirus
71030	GCA_000862665.1	Chayote mosaic virus
1736759	GCA_001669805.1	Chayote yellow mosaic Benin betasatellite

1736759	GCA_002830105.1	Chayote yellow mosaic Benin betasatellite
222450	GCA_000859865.1	Chayote yellow mosaic virus
485242	GCA_003178295.1	Chelonia mydas papillomavirus 1
1032478	GCA_002821805.1	Chenopodium leaf curl virus
2185087	GCA_004131185.1	Chenopodium quinoa mitovirus 1
40065	GCA_001184925.1	Chenuda virus
2054920	GCA_002831445.1	Chequa iflavirus
1642018	GCA_000989115.1	Cherax quadricarinatus densovirus
2035708	GCA_004131025.1	Cherax quadricarinatus iridovirus
1912598	GCA_001879265.1	Cherry associated luteovirus
284688	GCA_002987375.1	Cherry chlorotic rusty spot associated partitivirus
65467	GCA_000848245.1	Cherry green ring mottle virus
12615	GCA_000893515.1	Cherry leaf roll virus
131226	GCA_000848465.1	Cherry mottle leaf virus
129143	GCA_000849465.1	Cherry necrotic rusty mottle virus
202566	GCA_000859565.1	Cherry rasp leaf virus
1312929	GCA_000907155.1	Cherry rusty mottle associated virus
1424279	GCA_000921255.1	Cherry twisted leaf associated virus
42882	GCA_000855945.1	Cherry virus A
2074132	GCA_004132605.1	Cherry virus Trakiya
12618	GCA_000864805.1	Chicken anemia virus
2109365	GCA_004133245.1	Chicken associated cyclovirus 2
2169932	GCA_003033445.1	Chicken associated huchismacovirus 1
2169933	GCA_003033465.1	Chicken associated huchismacovirus 2
1932006	GCA_001957695.1	Chicken associated smacovirus
1932006	GCA_003963595.1	Chicken associated smacovirus
1932006	GCA_003963615.1	Chicken associated smacovirus
1932006	GCA_003963635.1	Chicken associated smacovirus
1932006	GCA_003963655.1	Chicken associated smacovirus
1932006	GCA_003963755.1	Chicken associated smacovirus
1172196	GCA_001963095.1	Chicken calicivirus
1534550	GCA_000924095.1	Chicken gallivirus 1
1477515	GCA_000918935.1	Chicken megrivirus
1477515	GCA_003029035.1	Chicken megrivirus
717641	GCA_000922115.1	Chicken parvovirus ABU-P1
1930304	GCA_004117635.1	Chicken picobirnavirus
1534544	GCA_000923455.1	Chicken picornavirus 1
1534545	GCA_000924115.1	Chicken picornavirus 2
1534546	GCA_000923475.1	Chicken picornavirus 3
1534547	GCA_000922415.1	Chicken picornavirus 4
1534548	GCA_000921575.1	Chicken picornavirus 5
1746030	GCA_001443985.1	Chicken sicinivirus JSY
2109373	GCA_003729875.1	Chicken stool associated circular virus 1
2109374	GCA_003729855.1	Chicken stool associated circular virus 2
1930302	GCA_001968175.1	Chicken stool-associated circular virus
1930298	GCA_001967675.1	Chicken stool-associated gemycircularvirus
1930298	GCA_003847525.1	Chicken stool-associated gemycircularvirus
1302848	GCA_000911975.1	Chickpea chlorosis Australia virus
887825	GCA_000887795.1	Chickpea chlorosis virus-A
887826	GCA_002824965.1	Chickpea chlorosis virus-B

1162554	GCA_002824985.1	Chickpea chlorosis virus-C
1162556	GCA_002825005.1	Chickpea chlorosis virus-E
463360	GCA_000880315.1	Chickpea chlorotic dwarf virus
463360	GCA_002987215.1	Chickpea chlorotic dwarf virus
463360	GCA_002987235.1	Chickpea chlorotic dwarf virus
463360	GCA_002987255.1	Chickpea chlorotic dwarf virus
463360	GCA_002987205.1	Chickpea chlorotic dwarf virus
328430	GCA_000868345.1	Chickpea chlorotic stunt virus
887827	GCA_000890315.1	Chickpea redleaf virus
1568974	GCA_000927635.1	Chickpea yellow dwarf virus
1162557	GCA_002825025.1	Chickpea yellows virus
192021	GCA_000852985.1	Chicory yellow mottle virus large satellite RNA
192022	GCA_000854685.1	Chicory yellow mottle virus satellite RNA
37124	GCA_000854045.1	Chikungunya virus
2010286	GCA_000843905.1	Chili leaf curl betasatellite
1335766	GCA_000908515.1	Chili leaf curl Bhatinda betasatellite
2010315	GCA_002830025.1	Chili leaf curl Sri Lanka betasatellite
1678003	GCA_001316395.1	Chilli leaf curl Ahmedabad virus-India [India/Ahmedabad/2
1428342	GCA_000922955.1	Chilli leaf curl alphasatellite
1428342	GCA_003029015.1	Chilli leaf curl alphasatellite
1507725	GCA_001045325.1	Chilli leaf curl India alphasatellite
577124	GCA_002986345.1	Chilli leaf curl India virus
1000665	GCA_002821825.1	Chilli leaf curl Kanpur virus [India/Kanpur/2008]
661505	GCA_000885195.1	Chilli leaf curl Multan alphasatellite
1000664	GCA_002821845.1	Chilli leaf curl Vellanad virus [India/Vellanad/2008]
172278	GCA_002821865.1	Chilli leaf curl virus
172278	GCA_002821905.1	Chilli leaf curl virus
172278	GCA_002821885.1	Chilli leaf curl virus
222451	GCA_000841265.1	Chilli leaf curl virus-[Multan]
414528	GCA_000896355.1	Chilli ringspot virus
52280	GCA_000860025.1	Chilli veinal mottle virus
688701	GCA_000889035.1	Chiltepin yellow mosaic virus
2170062	GCA_003032545.1	Chim orthonairovirus
1608440	GCA_001021315.1	Chimeric virus 14
1123958	GCA_000897015.1	Chimpanzee adenovirus Y25
2170109	GCA_003033555.1	Chimpanzee associated porprismacovirus 1
2170110	GCA_003033565.1	Chimpanzee associated porprismacovirus 2
1676185	GCA_001684485.1	Chimpanzee faeces associated circular DNA molecule 1
1676184	GCA_001684725.1	Chimpanzee faeces associated circular DNA virus 1
1676181	GCA_001685325.1	Chimpanzee faeces associated microphage 1
1676182	GCA_001684845.1	Chimpanzee faeces associated microphage 2
1676183	GCA_001685285.1	Chimpanzee faeces associated microphage 3
332937	GCA_000918475.1	Chimpanzee herpesvirus strain 105640
2035845	GCA_000890335.1	Chimpanzee polyomavirus Bob
702718	GCA_003967815.1	Chimpanzee stool associated circular ssDNA virus
702718	GCA_003967835.1	Chimpanzee stool associated circular ssDNA virus
702718	GCA_003967855.1	Chimpanzee stool associated circular ssDNA virus
702718	GCA_003967875.1	Chimpanzee stool associated circular ssDNA virus
743290	GCA_002819585.1	Chimpanzee stool avian-like circovirus Chimp17
83544	GCA_000850145.1	Chinese wheat mosaic virus

128818	GCA_000897555.1	Chinese yam necrotic mosaic virus
858516	GCA_002821945.1	Chino del tomate Amazonas virus
47838	GCA_002821985.1	Chino del tomate virus
47838	GCA_002821965.1	Chino del tomate virus
222455	GCA_000838385.1	Chino del tomate virus-[IC]
1611837	GCA_000973255.1	Chinook salmon bafinivirus
56820	GCA_002827425.1	Chipmunk parvovirus
2250219	GCA_004117575.1	Chiqui virus
105154	GCA_000849665.1	Chlamydia phage 2
225067	GCA_002601265.1	Chlamydia phage 3
313629	GCA_000865125.1	Chlamydia phage 4
2003327	GCA_000839525.1	Chlamydia virus Chp1
1986029	GCA_001275455.1	Chlamydia virus CPAR39
10820	GCA_000839545.1	Chloris striate mosaic virus
1891719	GCA_000903695.1	Chlorocebus pygerythrus polyomavirus 1
1891758	GCA_000928095.1	Chlorocebus pygerythrus polyomavirus 2
1891720	GCA_000928075.1	Chlorocebus pygerythrus polyomavirus 3
1679172	GCA_001184885.1	Chobar Gorge virus
1120751	GCA_000895075.1	Chocolate lily virus A
529380	GCA_002987385.1	Chondrostereum purpureum cryptic virus 1
10288	GCA_000427115.1	Choristoneura biennis entomopoxvirus
74660	GCA_000857025.1	Choristoneura fumiferana DEF multiple nucleopolyhedrovi
56947	GCA_000869805.1	Choristoneura fumiferana granulovirus
56947	GCA_002819205.1	Choristoneura fumiferana granulovirus
208973	GCA_000857005.1	Choristoneura fumiferana multiple nucleopolyhedrovirus
1987479	GCA_000915935.1	Choristoneura murinana nucleopolyhedrovirus
1293539	GCA_000427175.1	Choristoneura rosaceana entomopoxvirus 'L'
58094	GCA_000910655.1	Choristoneura rosaceana nucleopolyhedrovirus
58094	GCA_002819085.1	Choristoneura rosaceana nucleopolyhedrovirus
180822	GCA_000875145.1	Chronic bee paralysis virus
68402	GCA_000839165.1	Chrysanthemum chlorotic mottle viroid
83871	GCA_001343765.1	Chrysanthemum stem necrosis virus
12897	GCA_000853905.1	Chrysanthemum stunt viroid
12165	GCA_000870605.1	Chrysanthemum virus B
2116736	GCA_004132565.1	Chrysanthemum virus R
455364	GCA_001399245.1	Chrysochromulina ericina virus
320432	GCA_000863745.1	Chrysodeixis chalcites nucleopolyhedrovirus
173082	GCA_000866805.1	Chum salmon reovirus CS
77204	GCA_000856065.1	Chuzan virus
1427476	GCA_001343745.1	Cimodo virus
1346815	GCA_000919735.1	Circo-like virus-Brazil hs1
1346816	GCA_003985525.1	Circo-like virus-Brazil hs2
1379692	GCA_000927715.1	Circoviridae 1 LDMD-2013
1379714	GCA_000928695.1	Circoviridae 10 LDMD-2013
1379715	GCA_000927795.1	Circoviridae 11 LDMD-2013
1379717	GCA_000930355.1	Circoviridae 13 LDMD-2013
1379718	GCA_000929715.1	Circoviridae 14 LDMD-2013
1379719	GCA_000928675.1	Circoviridae 15 LDMD-2013
1379720	GCA_000927775.1	Circoviridae 16 LDMD-2013
1379721	GCA_000930335.1	Circoviridae 17 LDMD-2013

1379722	GCA_000929775.1	Circoviridae 18 LDMD-2013
1379723	GCA_000929695.1	Circoviridae 19 LDMD-2013
1379695	GCA_000930395.1	Circoviridae 2 LDMD-2013
1379725	GCA_000928655.1	Circoviridae 21 LDMD-2013
1379707	GCA_000929755.1	Circoviridae 3 LDMD-2013
1379708	GCA_000927835.1	Circoviridae 4 LDMD-2013
1379709	GCA_000927755.1	Circoviridae 5 LDMD-2013
1379710	GCA_000928735.1	Circoviridae 6 LDMD-2013
1379711	GCA_000927815.1	Circoviridae 7 LDMD-2013
1379712	GCA_000930375.1	Circoviridae 8 LDMD-2013
1379713	GCA_000929735.1	Circoviridae 9 LDMD-2013
1127491	GCA_003033385.1	Circoviridae bovine stool/BK/KOR/2011
1548712	GCA_000926895.1	Circoviridae SFBeef
1954248	GCA_003659705.1	Circoviridae sp.
1954248	GCA_003652205.1	Circoviridae sp.
1954248	GCA_003659025.1	Circoviridae sp.
1954248	GCA_003654265.1	Circoviridae sp.
1954248	GCA_003652225.1	Circoviridae sp.
1954248	GCA_003658005.1	Circoviridae sp.
1954248	GCA_003652245.1	Circoviridae sp.
1954248	GCA_003657325.1	Circoviridae sp.
1954248	GCA_003654305.1	Circoviridae sp.
1954248	GCA_003656985.1	Circoviridae sp.
1954248	GCA_003652265.1	Circoviridae sp.
1954248	GCA_003656645.1	Circoviridae sp.
1954248	GCA_003656305.1	Circoviridae sp.
1954248	GCA_003652285.1	Circoviridae sp.
1954248	GCA_003655625.1	Circoviridae sp.
1954248	GCA_003654365.1	Circoviridae sp.
1954248	GCA_003659725.1	Circoviridae sp.
1954248	GCA_003652325.1	Circoviridae sp.
1954248	GCA_003659045.1	Circoviridae sp.
1954248	GCA_003652345.1	Circoviridae sp.
1954248	GCA_003658365.1	Circoviridae sp.
1954248	GCA_003654405.1	Circoviridae sp.
1954248	GCA_003657345.1	Circoviridae sp.
1954248	GCA_003654425.1	Circoviridae sp.
1954248	GCA_003656485.1	Circoviridae sp.
1954248	GCA_003654445.1	Circoviridae sp.
1954248	GCA_003656325.1	Circoviridae sp.
1954248	GCA_003652405.1	Circoviridae sp.
1954248	GCA_003655645.1	Circoviridae sp.
1954248	GCA_003654465.1	Circoviridae sp.
1954248	GCA_003655305.1	Circoviridae sp.
1954248	GCA_003654485.1	Circoviridae sp.
1954248	GCA_003658705.1	Circoviridae sp.
1954248	GCA_003652445.1	Circoviridae sp.
1954248	GCA_003659405.1	Circoviridae sp.
1954248	GCA_003654505.1	Circoviridae sp.
1954248	GCA_003658725.1	Circoviridae sp.

1954248	GCA_003654525.1	Circoviridae sp.
1954248	GCA_003658045.1	Circoviridae sp.
1954248	GCA_003652485.1	Circoviridae sp.
1954248	GCA_003656585.1	Circoviridae sp.
1954248	GCA_003656525.1	Circoviridae sp.
1954248	GCA_003654565.1	Circoviridae sp.
1954248	GCA_003658665.1	Circoviridae sp.
1954248	GCA_003655665.1	Circoviridae sp.
1954248	GCA_003654585.1	Circoviridae sp.
1954248	GCA_003652545.1	Circoviridae sp.
1954248	GCA_003654605.1	Circoviridae sp.
1954248	GCA_003659765.1	Circoviridae sp.
1954248	GCA_003656685.1	Circoviridae sp.
1954248	GCA_003654645.1	Circoviridae sp.
1954248	GCA_003652605.1	Circoviridae sp.
1954248	GCA_003657385.1	Circoviridae sp.
1954248	GCA_003654665.1	Circoviridae sp.
1954248	GCA_003652625.1	Circoviridae sp.
1954248	GCA_003654685.1	Circoviridae sp.
1954248	GCA_003656365.1	Circoviridae sp.
1954248	GCA_003652645.1	Circoviridae sp.
1954248	GCA_003655685.1	Circoviridae sp.
1954248	GCA_003654705.1	Circoviridae sp.
1954248	GCA_003655345.1	Circoviridae sp.
1954248	GCA_003652665.1	Circoviridae sp.
1954248	GCA_003654725.1	Circoviridae sp.
1954248	GCA_003659785.1	Circoviridae sp.
1954248	GCA_003652685.1	Circoviridae sp.
1954248	GCA_003659445.1	Circoviridae sp.
1954248	GCA_003659105.1	Circoviridae sp.
1954248	GCA_003658765.1	Circoviridae sp.
1954248	GCA_003652705.1	Circoviridae sp.
1954248	GCA_003652725.1	Circoviridae sp.
1954248	GCA_003654785.1	Circoviridae sp.
1954248	GCA_003658885.1	Circoviridae sp.
1954248	GCA_003657065.1	Circoviridae sp.
1954248	GCA_003654805.1	Circoviridae sp.
1954248	GCA_003658905.1	Circoviridae sp.
1954248	GCA_003656865.1	Circoviridae sp.
1954248	GCA_003654825.1	Circoviridae sp.
1954248	GCA_003655365.1	Circoviridae sp.
1954248	GCA_003652785.1	Circoviridae sp.
1954248	GCA_003654845.1	Circoviridae sp.
1954248	GCA_003659805.1	Circoviridae sp.
1954248	GCA_003652805.1	Circoviridae sp.
1954248	GCA_003659465.1	Circoviridae sp.
1954248	GCA_003654865.1	Circoviridae sp.
1954248	GCA_003658785.1	Circoviridae sp.
1954248	GCA_003652825.1	Circoviridae sp.
1954248	GCA_003656925.1	Circoviridae sp.

1954248	GCA_003658445.1	Circoviridae sp.
1954248	GCA_003656565.1	Circoviridae sp.
1954248	GCA_003657425.1	Circoviridae sp.
1954248	GCA_003654905.1	Circoviridae sp.
1954248	GCA_003657085.1	Circoviridae sp.
1954248	GCA_003652865.1	Circoviridae sp.
1954248	GCA_003654925.1	Circoviridae sp.
1954248	GCA_003656405.1	Circoviridae sp.
1954248	GCA_003652885.1	Circoviridae sp.
1954248	GCA_003655725.1	Circoviridae sp.
1954248	GCA_003654965.1	Circoviridae sp.
1954248	GCA_003659825.1	Circoviridae sp.
1954248	GCA_003652925.1	Circoviridae sp.
1954248	GCA_003659145.1	Circoviridae sp.
1954248	GCA_003654985.1	Circoviridae sp.
1954248	GCA_003658805.1	Circoviridae sp.
1954248	GCA_003658465.1	Circoviridae sp.
1954248	GCA_003655005.1	Circoviridae sp.
1954248	GCA_003656825.1	Circoviridae sp.
1954248	GCA_003652965.1	Circoviridae sp.
1954248	GCA_003657445.1	Circoviridae sp.
1954248	GCA_003655025.1	Circoviridae sp.
1954248	GCA_003652985.1	Circoviridae sp.
1954248	GCA_003656765.1	Circoviridae sp.
1954248	GCA_003655045.1	Circoviridae sp.
1954248	GCA_003656425.1	Circoviridae sp.
1954248	GCA_003653005.1	Circoviridae sp.
1954248	GCA_003657105.1	Circoviridae sp.
1954248	GCA_003655065.1	Circoviridae sp.
1954248	GCA_003653025.1	Circoviridae sp.
1954248	GCA_003659845.1	Circoviridae sp.
1954248	GCA_003655105.1	Circoviridae sp.
1954248	GCA_003658485.1	Circoviridae sp.
1954248	GCA_003657805.1	Circoviridae sp.
1954248	GCA_003657465.1	Circoviridae sp.
1954248	GCA_003655145.1	Circoviridae sp.
1954248	GCA_003653105.1	Circoviridae sp.
1954248	GCA_003653125.1	Circoviridae sp.
1954248	GCA_003656105.1	Circoviridae sp.
1954248	GCA_003655185.1	Circoviridae sp.
1954248	GCA_003655425.1	Circoviridae sp.
1954248	GCA_003653145.1	Circoviridae sp.
1954248	GCA_003659745.1	Circoviridae sp.
1954248	GCA_003655205.1	Circoviridae sp.
1954248	GCA_003653165.1	Circoviridae sp.
1954248	GCA_003659525.1	Circoviridae sp.
1954248	GCA_003657265.1	Circoviridae sp.
1954248	GCA_003659185.1	Circoviridae sp.
1954248	GCA_003655225.1	Circoviridae sp.
1954248	GCA_003658845.1	Circoviridae sp.

1954248	GCA_003653185.1	Circoviridae sp.
1954248	GCA_003657285.1	Circoviridae sp.
1954248	GCA_003655245.1	Circoviridae sp.
1954248	GCA_003655265.1	Circoviridae sp.
1954248	GCA_003653245.1	Circoviridae sp.
1954248	GCA_003655445.1	Circoviridae sp.
1954248	GCA_003653265.1	Circoviridae sp.
1954248	GCA_003659885.1	Circoviridae sp.
1954248	GCA_003659205.1	Circoviridae sp.
1954248	GCA_003658865.1	Circoviridae sp.
1954248	GCA_003653305.1	Circoviridae sp.
1954248	GCA_003653325.1	Circoviridae sp.
1954248	GCA_003657165.1	Circoviridae sp.
1954248	GCA_003653345.1	Circoviridae sp.
1954248	GCA_003653365.1	Circoviridae sp.
1954248	GCA_003655465.1	Circoviridae sp.
1954248	GCA_003659905.1	Circoviridae sp.
1954248	GCA_003653405.1	Circoviridae sp.
1954248	GCA_003659565.1	Circoviridae sp.
1954248	GCA_003659225.1	Circoviridae sp.
1954248	GCA_003653445.1	Circoviridae sp.
1954248	GCA_003657865.1	Circoviridae sp.
1954248	GCA_003657185.1	Circoviridae sp.
1954248	GCA_003653465.1	Circoviridae sp.
1954248	GCA_003655525.1	Circoviridae sp.
1954248	GCA_003653485.1	Circoviridae sp.
1954248	GCA_003656165.1	Circoviridae sp.
1954248	GCA_003655825.1	Circoviridae sp.
1954248	GCA_003655545.1	Circoviridae sp.
1954248	GCA_003653505.1	Circoviridae sp.
1954248	GCA_003655565.1	Circoviridae sp.
1954248	GCA_003659925.1	Circoviridae sp.
1954248	GCA_003653525.1	Circoviridae sp.
1954248	GCA_003659585.1	Circoviridae sp.
1954248	GCA_003657625.1	Circoviridae sp.
1954248	GCA_003659245.1	Circoviridae sp.
1954248	GCA_003655585.1	Circoviridae sp.
1954248	GCA_003653545.1	Circoviridae sp.
1954248	GCA_003655605.1	Circoviridae sp.
1954248	GCA_003653565.1	Circoviridae sp.
1954248	GCA_003652425.1	Circoviridae sp.
1954248	GCA_003657205.1	Circoviridae sp.
1954248	GCA_003653585.1	Circoviridae sp.
1954248	GCA_003653605.1	Circoviridae sp.
1954248	GCA_003656185.1	Circoviridae sp.
1954248	GCA_003657705.1	Circoviridae sp.
1954248	GCA_003655505.1	Circoviridae sp.
1954248	GCA_003653625.1	Circoviridae sp.
1954248	GCA_003659945.1	Circoviridae sp.
1954248	GCA_003653645.1	Circoviridae sp.

1954248	GCA_003659605.1	Circoviridae sp.
1954248	GCA_003659265.1	Circoviridae sp.
1954248	GCA_003655705.1	Circoviridae sp.
1954248	GCA_003653665.1	Circoviridae sp.
1954248	GCA_003653685.1	Circoviridae sp.
1954248	GCA_003651645.1	Circoviridae sp.
1954248	GCA_003655745.1	Circoviridae sp.
1954248	GCA_003653705.1	Circoviridae sp.
1954248	GCA_003656885.1	Circoviridae sp.
1954248	GCA_003653725.1	Circoviridae sp.
1954248	GCA_003655785.1	Circoviridae sp.
1954248	GCA_003653745.1	Circoviridae sp.
1954248	GCA_003659965.1	Circoviridae sp.
1954248	GCA_003653765.1	Circoviridae sp.
1954248	GCA_003651725.1	Circoviridae sp.
1954248	GCA_003653785.1	Circoviridae sp.
1954248	GCA_003657885.1	Circoviridae sp.
1954248	GCA_003658605.1	Circoviridae sp.
1954248	GCA_003655845.1	Circoviridae sp.
1954248	GCA_003653805.1	Circoviridae sp.
1954248	GCA_003657905.1	Circoviridae sp.
1954248	GCA_003653825.1	Circoviridae sp.
1954248	GCA_003657925.1	Circoviridae sp.
1954248	GCA_003653845.1	Circoviridae sp.
1954248	GCA_003655885.1	Circoviridae sp.
1954248	GCA_003653865.1	Circoviridae sp.
1954248	GCA_003651825.1	Circoviridae sp.
1954248	GCA_003659985.1	Circoviridae sp.
1954248	GCA_003653885.1	Circoviridae sp.
1954248	GCA_003659305.1	Circoviridae sp.
1954248	GCA_003655945.1	Circoviridae sp.
1954248	GCA_003658965.1	Circoviridae sp.
1954248	GCA_003653905.1	Circoviridae sp.
1954248	GCA_003658625.1	Circoviridae sp.
1954248	GCA_003653925.1	Circoviridae sp.
1954248	GCA_003656845.1	Circoviridae sp.
1954248	GCA_003657945.1	Circoviridae sp.
1954248	GCA_003657605.1	Circoviridae sp.
1954248	GCA_003656505.1	Circoviridae sp.
1954248	GCA_003656005.1	Circoviridae sp.
1954248	GCA_003653965.1	Circoviridae sp.
1954248	GCA_003658065.1	Circoviridae sp.
1954248	GCA_003655905.1	Circoviridae sp.
1954248	GCA_003656025.1	Circoviridae sp.
1954248	GCA_003658085.1	Circoviridae sp.
1954248	GCA_003651945.1	Circoviridae sp.
1954248	GCA_003660005.1	Circoviridae sp.
1954248	GCA_003654005.1	Circoviridae sp.
1954248	GCA_003659665.1	Circoviridae sp.
1954248	GCA_003656065.1	Circoviridae sp.

1954248	GCA_003658985.1	Circoviridae sp.
1954248	GCA_003658645.1	Circoviridae sp.
1954248	GCA_003651985.1	Circoviridae sp.
1954248	GCA_003654045.1	Circoviridae sp.
1954248	GCA_003657965.1	Circoviridae sp.
1954248	GCA_003656805.1	Circoviridae sp.
1954248	GCA_003654065.1	Circoviridae sp.
1954248	GCA_003658165.1	Circoviridae sp.
1954248	GCA_003656945.1	Circoviridae sp.
1954248	GCA_003656605.1	Circoviridae sp.
1954248	GCA_003654085.1	Circoviridae sp.
1954248	GCA_003658185.1	Circoviridae sp.
1954248	GCA_003656145.1	Circoviridae sp.
1954248	GCA_003660025.1	Circoviridae sp.
1954248	GCA_003654125.1	Circoviridae sp.
1954248	GCA_003659685.1	Circoviridae sp.
1954248	GCA_003659345.1	Circoviridae sp.
1954248	GCA_003659005.1	Circoviridae sp.
1954248	GCA_003654165.1	Circoviridae sp.
1954248	GCA_003658265.1	Circoviridae sp.
1954248	GCA_003656225.1	Circoviridae sp.
1954248	GCA_003658285.1	Circoviridae sp.
1954248	GCA_003652145.1	Circoviridae sp.
1954248	GCA_003656245.1	Circoviridae sp.
1954248	GCA_003656625.1	Circoviridae sp.
1954248	GCA_003656445.1	Circoviridae sp.
1954248	GCA_003654205.1	Circoviridae sp.
1954248	GCA_003658305.1	Circoviridae sp.
1954248	GCA_003652165.1	Circoviridae sp.
1954248	GCA_003656265.1	Circoviridae sp.
1954248	GCA_003654225.1	Circoviridae sp.
1954248	GCA_003658325.1	Circoviridae sp.
1954248	GCA_003656705.1	Circoviridae sp.
1954248	GCA_003656285.1	Circoviridae sp.
1954248	GCA_003660045.1	Circoviridae sp.
1954248	GCA_003950095.1	Circoviridae sp.
1954248	GCA_004099285.1	Circoviridae sp.
1954248	GCA_004099325.1	Circoviridae sp.
1954248	GCA_004099305.1	Circoviridae sp.
1954248	GCA_004099365.1	Circoviridae sp.
1954248	GCA_004099385.1	Circoviridae sp.
1954248	GCA_004099345.1	Circoviridae sp.
1954248	GCA_004099405.1	Circoviridae sp.
1954248	GCA_004099445.1	Circoviridae sp.
1954248	GCA_004099545.1	Circoviridae sp.
1954248	GCA_004099585.1	Circoviridae sp.
1954248	GCA_004099565.1	Circoviridae sp.
1954248	GCA_004099425.1	Circoviridae sp.
1954248	GCA_004099465.1	Circoviridae sp.
1954248	GCA_004099485.1	Circoviridae sp.

1954248	GCA_004099505.1	Circoviridae sp.
1954248	GCA_004099525.1	Circoviridae sp.
2094724	GCA_003389855.1	Circoviridae TaCV1
2094725	GCA_003389875.1	Circoviridae TaCV2
942030	GCA_004054935.1	Circovirus NGchicken38/NGA/2009
1964372	GCA_002375075.1	Circovirus sp.
1964372	GCA_004043955.1	Circovirus sp.
1964372	GCA_004043975.1	Circovirus sp.
1964372	GCA_004043995.1	Circovirus sp.
1964372	GCA_004044015.1	Circovirus sp.
1964372	GCA_004042775.1	Circovirus sp.
1964372	GCA_004042795.1	Circovirus sp.
1964372	GCA_004042815.1	Circovirus sp.
1964372	GCA_004042835.1	Circovirus sp.
1964372	GCA_004042855.1	Circovirus sp.
1964372	GCA_004042875.1	Circovirus sp.
1964372	GCA_004042895.1	Circovirus sp.
1964372	GCA_004041475.1	Circovirus sp.
642260	GCA_000885735.1	Circovirus-like genome BBC-A
642256	GCA_000885775.1	Circovirus-like genome CB-A
642257	GCA_000885135.1	Circovirus-like genome CB-B
1788437	GCA_001684945.1	Circovirus-like genome DCCV-1
1788438	GCA_001684825.1	Circovirus-like genome DCCV-10
1788439	GCA_001684585.1	Circovirus-like genome DCCV-11
1788440	GCA_001684705.1	Circovirus-like genome DCCV-12
1788441	GCA_001684925.1	Circovirus-like genome DCCV-13
1788442	GCA_001684805.1	Circovirus-like genome DCCV-2
1788443	GCA_001684565.1	Circovirus-like genome DCCV-3
1788444	GCA_001684685.1	Circovirus-like genome DCCV-4
1788445	GCA_001684905.1	Circovirus-like genome DCCV-5
1788446	GCA_001684785.1	Circovirus-like genome DCCV-6
1788447	GCA_001684545.1	Circovirus-like genome DCCV-7
1788448	GCA_001684665.1	Circovirus-like genome DCCV-8
1788449	GCA_001684885.1	Circovirus-like genome DCCV-9
1788450	GCA_001684765.1	Circovirus-like genome DHCV-1
1788451	GCA_001684525.1	Circovirus-like genome DHCV-2
1788452	GCA_001684645.1	Circovirus-like genome DHCV-3
1788454	GCA_001684865.1	Circovirus-like genome DHCV-5
1788455	GCA_001684745.1	Circovirus-like genome DHCV-6
642251	GCA_000884135.1	Circovirus-like genome RW-A
642252	GCA_000885755.1	Circovirus-like genome RW-B
642253	GCA_000885115.1	Circovirus-like genome RW-C
642254	GCA_000886635.1	Circovirus-like genome RW-D
642255	GCA_000884155.1	Circovirus-like genome RW-E
642258	GCA_000886655.1	Circovirus-like genome SAR-A
642259	GCA_000886595.1	Circovirus-like genome SAR-B
2202954	GCA_004196935.1	Circular genetic element sp.
2202954	GCA_004203655.1	Circular genetic element sp.
2202954	GCA_004201615.1	Circular genetic element sp.
2202954	GCA_004203315.1	Circular genetic element sp.

2202954	GCA_004198535.1	Circular genetic element sp.
2202954	GCA_004196955.1	Circular genetic element sp.
2202954	GCA_004207755.1	Circular genetic element sp.
2202954	GCA_004201635.1	Circular genetic element sp.
2202954	GCA_004198415.1	Circular genetic element sp.
2202954	GCA_004198555.1	Circular genetic element sp.
2202954	GCA_004207415.1	Circular genetic element sp.
2202954	GCA_004196975.1	Circular genetic element sp.
2202954	GCA_004207795.1	Circular genetic element sp.
2202954	GCA_004201655.1	Circular genetic element sp.
2202954	GCA_004206735.1	Circular genetic element sp.
2202954	GCA_004205755.1	Circular genetic element sp.
2202954	GCA_004206395.1	Circular genetic element sp.
2202954	GCA_004196995.1	Circular genetic element sp.
2202954	GCA_004206055.1	Circular genetic element sp.
2202954	GCA_004201675.1	Circular genetic element sp.
2202954	GCA_004198575.1	Circular genetic element sp.
2202954	GCA_004205715.1	Circular genetic element sp.
2202954	GCA_004197015.1	Circular genetic element sp.
2202954	GCA_004201695.1	Circular genetic element sp.
2202954	GCA_004205035.1	Circular genetic element sp.
2202954	GCA_004205795.1	Circular genetic element sp.
2202954	GCA_004197975.1	Circular genetic element sp.
2202954	GCA_004197035.1	Circular genetic element sp.
2202954	GCA_004207855.1	Circular genetic element sp.
2202954	GCA_004197635.1	Circular genetic element sp.
2202954	GCA_004201715.1	Circular genetic element sp.
2202954	GCA_004198715.1	Circular genetic element sp.
2202954	GCA_004198615.1	Circular genetic element sp.
2202954	GCA_004197055.1	Circular genetic element sp.
2202954	GCA_004203675.1	Circular genetic element sp.
2202954	GCA_004201735.1	Circular genetic element sp.
2202954	GCA_004205835.1	Circular genetic element sp.
2202954	GCA_004208115.1	Circular genetic element sp.
2202954	GCA_004197075.1	Circular genetic element sp.
2202954	GCA_004207775.1	Circular genetic element sp.
2202954	GCA_004201755.1	Circular genetic element sp.
2202954	GCA_004198635.1	Circular genetic element sp.
2202954	GCA_004197095.1	Circular genetic element sp.
2202954	GCA_004207095.1	Circular genetic element sp.
2202954	GCA_004207915.1	Circular genetic element sp.
2202954	GCA_004201775.1	Circular genetic element sp.
2202954	GCA_004198655.1	Circular genetic element sp.
2202954	GCA_004208375.1	Circular genetic element sp.
2202954	GCA_004206415.1	Circular genetic element sp.
2202954	GCA_004197115.1	Circular genetic element sp.
2202954	GCA_004201795.1	Circular genetic element sp.
2202954	GCA_004198675.1	Circular genetic element sp.
2202954	GCA_004205735.1	Circular genetic element sp.
2202954	GCA_004203855.1	Circular genetic element sp.

2202954	GCA_004201815.1	Circular genetic element sp.
2202954	GCA_004198335.1	Circular genetic element sp.
2202954	GCA_004205915.1	Circular genetic element sp.
2202954	GCA_004204715.1	Circular genetic element sp.
2202954	GCA_004207975.1	Circular genetic element sp.
2202954	GCA_004197655.1	Circular genetic element sp.
2202954	GCA_004201835.1	Circular genetic element sp.
2202954	GCA_004205935.1	Circular genetic element sp.
2202954	GCA_004204035.1	Circular genetic element sp.
2202954	GCA_004197175.1	Circular genetic element sp.
2202954	GCA_004203695.1	Circular genetic element sp.
2202954	GCA_004201855.1	Circular genetic element sp.
2202954	GCA_004196635.1	Circular genetic element sp.
2202954	GCA_004198695.1	Circular genetic element sp.
2202954	GCA_004208135.1	Circular genetic element sp.
2202954	GCA_004203915.1	Circular genetic element sp.
2202954	GCA_004201875.1	Circular genetic element sp.
2202954	GCA_004205975.1	Circular genetic element sp.
2202954	GCA_004207455.1	Circular genetic element sp.
2202954	GCA_004197215.1	Circular genetic element sp.
2202954	GCA_004207115.1	Circular genetic element sp.
2202954	GCA_004208035.1	Circular genetic element sp.
2202954	GCA_004201895.1	Circular genetic element sp.
2202954	GCA_004205995.1	Circular genetic element sp.
2202954	GCA_004203955.1	Circular genetic element sp.
2202954	GCA_004206095.1	Circular genetic element sp.
2202954	GCA_004201915.1	Circular genetic element sp.
2202954	GCA_004206015.1	Circular genetic element sp.
2202954	GCA_004200775.1	Circular genetic element sp.
2202954	GCA_004197255.1	Circular genetic element sp.
2202954	GCA_004201935.1	Circular genetic element sp.
2202954	GCA_004198355.1	Circular genetic element sp.
2202954	GCA_004206035.1	Circular genetic element sp.
2202954	GCA_004197275.1	Circular genetic element sp.
2202954	GCA_004206995.1	Circular genetic element sp.
2202954	GCA_004208095.1	Circular genetic element sp.
2202954	GCA_004196535.1	Circular genetic element sp.
2202954	GCA_004201955.1	Circular genetic element sp.
2202954	GCA_004198755.1	Circular genetic element sp.
2202954	GCA_004197295.1	Circular genetic element sp.
2202954	GCA_004203715.1	Circular genetic element sp.
2202954	GCA_004201975.1	Circular genetic element sp.
2202954	GCA_004196655.1	Circular genetic element sp.
2202954	GCA_004206075.1	Circular genetic element sp.
2202954	GCA_004197315.1	Circular genetic element sp.
2202954	GCA_004207815.1	Circular genetic element sp.
2202954	GCA_004201995.1	Circular genetic element sp.
2202954	GCA_004198795.1	Circular genetic element sp.
2202954	GCA_004207475.1	Circular genetic element sp.
2202954	GCA_004204055.1	Circular genetic element sp.

2202954	GCA_004208155.1	Circular genetic element sp.
2202954	GCA_004202015.1	Circular genetic element sp.
2202954	GCA_004206795.1	Circular genetic element sp.
2202954	GCA_004206115.1	Circular genetic element sp.
2202954	GCA_004199055.1	Circular genetic element sp.
2202954	GCA_004204075.1	Circular genetic element sp.
2202954	GCA_004202035.1	Circular genetic element sp.
2202954	GCA_004206135.1	Circular genetic element sp.
2202954	GCA_004205775.1	Circular genetic element sp.
2202954	GCA_004204095.1	Circular genetic element sp.
2202954	GCA_004208355.1	Circular genetic element sp.
2202954	GCA_004202055.1	Circular genetic element sp.
2202954	GCA_004205095.1	Circular genetic element sp.
2202954	GCA_004198815.1	Circular genetic element sp.
2202954	GCA_004198035.1	Circular genetic element sp.
2202954	GCA_004208215.1	Circular genetic element sp.
2202954	GCA_004202075.1	Circular genetic element sp.
2202954	GCA_004208295.1	Circular genetic element sp.
2202954	GCA_004198835.1	Circular genetic element sp.
2202954	GCA_004197355.1	Circular genetic element sp.
2202954	GCA_004204135.1	Circular genetic element sp.
2202954	GCA_004203735.1	Circular genetic element sp.
2202954	GCA_004202095.1	Circular genetic element sp.
2202954	GCA_004203395.1	Circular genetic element sp.
2202954	GCA_004198855.1	Circular genetic element sp.
2202954	GCA_004208175.1	Circular genetic element sp.
2202954	GCA_004204155.1	Circular genetic element sp.
2202954	GCA_004207835.1	Circular genetic element sp.
2202954	GCA_004202115.1	Circular genetic element sp.
2202954	GCA_004198875.1	Circular genetic element sp.
2202954	GCA_004207495.1	Circular genetic element sp.
2202954	GCA_004197455.1	Circular genetic element sp.
2202954	GCA_004208275.1	Circular genetic element sp.
2202954	GCA_004202135.1	Circular genetic element sp.
2202954	GCA_004206815.1	Circular genetic element sp.
2202954	GCA_004206235.1	Circular genetic element sp.
2202954	GCA_004204195.1	Circular genetic element sp.
2202954	GCA_004201075.1	Circular genetic element sp.
2202954	GCA_004202155.1	Circular genetic element sp.
2202954	GCA_004198915.1	Circular genetic element sp.
2202954	GCA_004198595.1	Circular genetic element sp.
2202954	GCA_004197495.1	Circular genetic element sp.
2202954	GCA_004202175.1	Circular genetic element sp.
2202954	GCA_004205115.1	Circular genetic element sp.
2202954	GCA_004206275.1	Circular genetic element sp.
2202954	GCA_004204775.1	Circular genetic element sp.
2202954	GCA_004197515.1	Circular genetic element sp.
2202954	GCA_004208335.1	Circular genetic element sp.
2202954	GCA_004197795.1	Circular genetic element sp.
2202954	GCA_004202195.1	Circular genetic element sp.

2202954	GCA_004206295.1	Circular genetic element sp.
2202954	GCA_004197375.1	Circular genetic element sp.
2202954	GCA_004204255.1	Circular genetic element sp.
2202954	GCA_004203755.1	Circular genetic element sp.
2202954	GCA_004199415.1	Circular genetic element sp.
2202954	GCA_004202215.1	Circular genetic element sp.
2202954	GCA_004203415.1	Circular genetic element sp.
2202954	GCA_004198955.1	Circular genetic element sp.
2202954	GCA_004208195.1	Circular genetic element sp.
2202954	GCA_004197555.1	Circular genetic element sp.
2202954	GCA_004199435.1	Circular genetic element sp.
2202954	GCA_004202235.1	Circular genetic element sp.
2202954	GCA_004198975.1	Circular genetic element sp.
2202954	GCA_004207515.1	Circular genetic element sp.
2202954	GCA_004204295.1	Circular genetic element sp.
2202954	GCA_004208395.1	Circular genetic element sp.
2202954	GCA_004202255.1	Circular genetic element sp.
2202954	GCA_004206355.1	Circular genetic element sp.
2202954	GCA_004204755.1	Circular genetic element sp.
2202954	GCA_004199075.1	Circular genetic element sp.
2202954	GCA_004197595.1	Circular genetic element sp.
2202954	GCA_004199455.1	Circular genetic element sp.
2202954	GCA_004206155.1	Circular genetic element sp.
2202954	GCA_004202275.1	Circular genetic element sp.
2202954	GCA_004206375.1	Circular genetic element sp.
2202954	GCA_004200815.1	Circular genetic element sp.
2202954	GCA_004200415.1	Circular genetic element sp.
2202954	GCA_004204335.1	Circular genetic element sp.
2202954	GCA_004204415.1	Circular genetic element sp.
2202954	GCA_004202295.1	Circular genetic element sp.
2202954	GCA_004199015.1	Circular genetic element sp.
2202954	GCA_004200435.1	Circular genetic element sp.
2202954	GCA_004204455.1	Circular genetic element sp.
2202954	GCA_004202315.1	Circular genetic element sp.
2202954	GCA_004199035.1	Circular genetic element sp.
2202954	GCA_004204115.1	Circular genetic element sp.
2202954	GCA_004200455.1	Circular genetic element sp.
2202954	GCA_004204375.1	Circular genetic element sp.
2202954	GCA_004203775.1	Circular genetic element sp.
2202954	GCA_004199475.1	Circular genetic element sp.
2202954	GCA_004202335.1	Circular genetic element sp.
2202954	GCA_004203435.1	Circular genetic element sp.
2202954	GCA_004206435.1	Circular genetic element sp.
2202954	GCA_004200475.1	Circular genetic element sp.
2202954	GCA_004204395.1	Circular genetic element sp.
2202954	GCA_004199495.1	Circular genetic element sp.
2202954	GCA_004207875.1	Circular genetic element sp.
2202954	GCA_004207895.1	Circular genetic element sp.
2202954	GCA_004202355.1	Circular genetic element sp.
2202954	GCA_004206455.1	Circular genetic element sp.

2202954	GCA_004207535.1	Circular genetic element sp.
2202954	GCA_004200495.1	Circular genetic element sp.
2202954	GCA_004197695.1	Circular genetic element sp.
2202954	GCA_004200855.1	Circular genetic element sp.
2202954	GCA_004202375.1	Circular genetic element sp.
2202954	GCA_004206475.1	Circular genetic element sp.
2202954	GCA_004200515.1	Circular genetic element sp.
2202954	GCA_004199095.1	Circular genetic element sp.
2202954	GCA_004204435.1	Circular genetic element sp.
2202954	GCA_004199515.1	Circular genetic element sp.
2202954	GCA_004206175.1	Circular genetic element sp.
2202954	GCA_004202395.1	Circular genetic element sp.
2202954	GCA_004206495.1	Circular genetic element sp.
2202954	GCA_004200535.1	Circular genetic element sp.
2202954	GCA_004197735.1	Circular genetic element sp.
2202954	GCA_004199535.1	Circular genetic element sp.
2202954	GCA_004202415.1	Circular genetic element sp.
2202954	GCA_004206515.1	Circular genetic element sp.
2202954	GCA_004200555.1	Circular genetic element sp.
2202954	GCA_004204815.1	Circular genetic element sp.
2202954	GCA_004204475.1	Circular genetic element sp.
2202954	GCA_004197755.1	Circular genetic element sp.
2202954	GCA_004202435.1	Circular genetic element sp.
2202954	GCA_004206535.1	Circular genetic element sp.
2202954	GCA_004197415.1	Circular genetic element sp.
2202954	GCA_004200575.1	Circular genetic element sp.
2202954	GCA_004204495.1	Circular genetic element sp.
2202954	GCA_004203795.1	Circular genetic element sp.
2202954	GCA_004202455.1	Circular genetic element sp.
2202954	GCA_004203455.1	Circular genetic element sp.
2202954	GCA_004204015.1	Circular genetic element sp.
2202954	GCA_004206555.1	Circular genetic element sp.
2202954	GCA_004200595.1	Circular genetic element sp.
2202954	GCA_004208235.1	Circular genetic element sp.
2202954	GCA_004197575.1	Circular genetic element sp.
2202954	GCA_004204515.1	Circular genetic element sp.
2202954	GCA_004197715.1	Circular genetic element sp.
2202954	GCA_004202475.1	Circular genetic element sp.
2202954	GCA_004206575.1	Circular genetic element sp.
2202954	GCA_004204535.1	Circular genetic element sp.
2202954	GCA_004207215.1	Circular genetic element sp.
2202954	GCA_004199575.1	Circular genetic element sp.
2202954	GCA_004197235.1	Circular genetic element sp.
2202954	GCA_004202495.1	Circular genetic element sp.
2202954	GCA_004206875.1	Circular genetic element sp.
2202954	GCA_004206595.1	Circular genetic element sp.
2202954	GCA_004206195.1	Circular genetic element sp.
2202954	GCA_004199115.1	Circular genetic element sp.
2202954	GCA_004204555.1	Circular genetic element sp.
2202954	GCA_004204695.1	Circular genetic element sp.

2202954	GCA_004199595.1	Circular genetic element sp.
2202954	GCA_004201135.1	Circular genetic element sp.
2202954	GCA_004202515.1	Circular genetic element sp.
2202954	GCA_004206615.1	Circular genetic element sp.
2202954	GCA_004205855.1	Circular genetic element sp.
2202954	GCA_004200615.1	Circular genetic element sp.
2202954	GCA_004204575.1	Circular genetic element sp.
2202954	GCA_004198435.1	Circular genetic element sp.
2202954	GCA_004199615.1	Circular genetic element sp.
2202954	GCA_004202535.1	Circular genetic element sp.
2202954	GCA_004204355.1	Circular genetic element sp.
2202954	GCA_004206635.1	Circular genetic element sp.
2202954	GCA_004204835.1	Circular genetic element sp.
2202954	GCA_004197875.1	Circular genetic element sp.
2202954	GCA_004199635.1	Circular genetic element sp.
2202954	GCA_004202555.1	Circular genetic element sp.
2202954	GCA_004206655.1	Circular genetic element sp.
2202954	GCA_004197435.1	Circular genetic element sp.
2202954	GCA_004200655.1	Circular genetic element sp.
2202954	GCA_004204615.1	Circular genetic element sp.
2202954	GCA_004203815.1	Circular genetic element sp.
2202954	GCA_004199655.1	Circular genetic element sp.
2202954	GCA_004202575.1	Circular genetic element sp.
2202954	GCA_004196755.1	Circular genetic element sp.
2202954	GCA_004206675.1	Circular genetic element sp.
2202954	GCA_004200675.1	Circular genetic element sp.
2202954	GCA_004208255.1	Circular genetic element sp.
2202954	GCA_004199675.1	Circular genetic element sp.
2202954	GCA_004202595.1	Circular genetic element sp.
2202954	GCA_004206695.1	Circular genetic element sp.
2202954	GCA_004207575.1	Circular genetic element sp.
2202954	GCA_004204635.1	Circular genetic element sp.
2202954	GCA_004200695.1	Circular genetic element sp.
2202954	GCA_004204655.1	Circular genetic element sp.
2202954	GCA_004207235.1	Circular genetic element sp.
2202954	GCA_004199695.1	Circular genetic element sp.
2202954	GCA_004202615.1	Circular genetic element sp.
2202954	GCA_004199315.1	Circular genetic element sp.
2202954	GCA_004206715.1	Circular genetic element sp.
2202954	GCA_004200715.1	Circular genetic element sp.
2202954	GCA_004204675.1	Circular genetic element sp.
2202954	GCA_004199715.1	Circular genetic element sp.
2202954	GCA_004206215.1	Circular genetic element sp.
2202954	GCA_004202635.1	Circular genetic element sp.
2202954	GCA_004199235.1	Circular genetic element sp.
2202954	GCA_004200875.1	Circular genetic element sp.
2202954	GCA_004200735.1	Circular genetic element sp.
2202954	GCA_004199735.1	Circular genetic element sp.
2202954	GCA_004202655.1	Circular genetic element sp.
2202954	GCA_004206755.1	Circular genetic element sp.

2202954	GCA_004204855.1	Circular genetic element sp.
2202954	GCA_004197995.1	Circular genetic element sp.
2202954	GCA_004199755.1	Circular genetic element sp.
2202954	GCA_004198775.1	Circular genetic element sp.
2202954	GCA_004202675.1	Circular genetic element sp.
2202954	GCA_004208315.1	Circular genetic element sp.
2202954	GCA_004206775.1	Circular genetic element sp.
2202954	GCA_004197855.1	Circular genetic element sp.
2202954	GCA_004204735.1	Circular genetic element sp.
2202954	GCA_004203835.1	Circular genetic element sp.
2202954	GCA_004202695.1	Circular genetic element sp.
2202954	GCA_004203495.1	Circular genetic element sp.
2202954	GCA_004199255.1	Circular genetic element sp.
2202954	GCA_004200795.1	Circular genetic element sp.
2202954	GCA_004199775.1	Circular genetic element sp.
2202954	GCA_004207935.1	Circular genetic element sp.
2202954	GCA_004202715.1	Circular genetic element sp.
2202954	GCA_004199275.1	Circular genetic element sp.
2202954	GCA_004207595.1	Circular genetic element sp.
2202954	GCA_004198055.1	Circular genetic element sp.
2202954	GCA_004199795.1	Circular genetic element sp.
2202954	GCA_004202735.1	Circular genetic element sp.
2202954	GCA_004206835.1	Circular genetic element sp.
2202954	GCA_004198075.1	Circular genetic element sp.
2202954	GCA_004199815.1	Circular genetic element sp.
2202954	GCA_004198895.1	Circular genetic element sp.
2202954	GCA_004202755.1	Circular genetic element sp.
2202954	GCA_004206855.1	Circular genetic element sp.
2202954	GCA_004205895.1	Circular genetic element sp.
2202954	GCA_004200835.1	Circular genetic element sp.
2202954	GCA_004198095.1	Circular genetic element sp.
2202954	GCA_004202775.1	Circular genetic element sp.
2202954	GCA_004199295.1	Circular genetic element sp.
2202954	GCA_004204875.1	Circular genetic element sp.
2202954	GCA_004198115.1	Circular genetic element sp.
2202954	GCA_004199835.1	Circular genetic element sp.
2202954	GCA_004197815.1	Circular genetic element sp.
2202954	GCA_004202795.1	Circular genetic element sp.
2202954	GCA_004206895.1	Circular genetic element sp.
2202954	GCA_004198135.1	Circular genetic element sp.
2202954	GCA_004197135.1	Circular genetic element sp.
2202954	GCA_004202815.1	Circular genetic element sp.
2202954	GCA_004196795.1	Circular genetic element sp.
2202954	GCA_004199335.1	Circular genetic element sp.
2202954	GCA_004200895.1	Circular genetic element sp.
2202954	GCA_004196455.1	Circular genetic element sp.
2202954	GCA_004198155.1	Circular genetic element sp.
2202954	GCA_004207955.1	Circular genetic element sp.
2202954	GCA_004202835.1	Circular genetic element sp.
2202954	GCA_004206935.1	Circular genetic element sp.

2202954	GCA_004207615.1	Circular genetic element sp.
2202954	GCA_004198175.1	Circular genetic element sp.
2202954	GCA_004207275.1	Circular genetic element sp.
2202954	GCA_004199855.1	Circular genetic element sp.
2202954	GCA_004202855.1	Circular genetic element sp.
2202954	GCA_004206955.1	Circular genetic element sp.
2202954	GCA_004199195.1	Circular genetic element sp.
2202954	GCA_004200755.1	Circular genetic element sp.
2202954	GCA_004200935.1	Circular genetic element sp.
2202954	GCA_004198195.1	Circular genetic element sp.
2202954	GCA_004199875.1	Circular genetic element sp.
2202954	GCA_004206255.1	Circular genetic element sp.
2202954	GCA_004202875.1	Circular genetic element sp.
2202954	GCA_004197895.1	Circular genetic element sp.
2202954	GCA_004200915.1	Circular genetic element sp.
2202954	GCA_004198215.1	Circular genetic element sp.
2202954	GCA_004205575.1	Circular genetic element sp.
2202954	GCA_004199895.1	Circular genetic element sp.
2202954	GCA_004202895.1	Circular genetic element sp.
2202954	GCA_004200955.1	Circular genetic element sp.
2202954	GCA_004204895.1	Circular genetic element sp.
2202954	GCA_004198235.1	Circular genetic element sp.
2202954	GCA_004199915.1	Circular genetic element sp.
2202954	GCA_004197835.1	Circular genetic element sp.
2202954	GCA_004202915.1	Circular genetic element sp.
2202954	GCA_004207015.1	Circular genetic element sp.
2202954	GCA_004204215.1	Circular genetic element sp.
2202954	GCA_004200975.1	Circular genetic element sp.
2202954	GCA_004204975.1	Circular genetic element sp.
2202954	GCA_004197155.1	Circular genetic element sp.
2202954	GCA_004199935.1	Circular genetic element sp.
2202954	GCA_004202935.1	Circular genetic element sp.
2202954	GCA_004196815.1	Circular genetic element sp.
2202954	GCA_004203195.1	Circular genetic element sp.
2202954	GCA_004198275.1	Circular genetic element sp.
2202954	GCA_004199955.1	Circular genetic element sp.
2202954	GCA_004202955.1	Circular genetic element sp.
2202954	GCA_004207055.1	Circular genetic element sp.
2202954	GCA_004207635.1	Circular genetic element sp.
2202954	GCA_004200995.1	Circular genetic element sp.
2202954	GCA_004271985.1	Circular genetic element sp.
2202954	GCA_004198295.1	Circular genetic element sp.
2202954	GCA_004207295.1	Circular genetic element sp.
2202954	GCA_004199975.1	Circular genetic element sp.
2202954	GCA_004202975.1	Circular genetic element sp.
2202954	GCA_004199355.1	Circular genetic element sp.
2202954	GCA_004207075.1	Circular genetic element sp.
2202954	GCA_004201015.1	Circular genetic element sp.
2202954	GCA_004199135.1	Circular genetic element sp.
2202954	GCA_004198315.1	Circular genetic element sp.

2202954	GCA_004199995.1	Circular genetic element sp.
2202954	GCA_004202995.1	Circular genetic element sp.
2202954	GCA_004201035.1	Circular genetic element sp.
2202954	GCA_004205055.1	Circular genetic element sp.
2202954	GCA_004198475.1	Circular genetic element sp.
2202954	GCA_004200015.1	Circular genetic element sp.
2202954	GCA_004203015.1	Circular genetic element sp.
2202954	GCA_004204915.1	Circular genetic element sp.
2202954	GCA_004205075.1	Circular genetic element sp.
2202954	GCA_004204235.1	Circular genetic element sp.
2202954	GCA_004203035.1	Circular genetic element sp.
2202954	GCA_004207135.1	Circular genetic element sp.
2202954	GCA_004198375.1	Circular genetic element sp.
2202954	GCA_004203055.1	Circular genetic element sp.
2202954	GCA_004203555.1	Circular genetic element sp.
2202954	GCA_004207155.1	Circular genetic element sp.
2202954	GCA_004203895.1	Circular genetic element sp.
2202954	GCA_004203215.1	Circular genetic element sp.
2202954	GCA_004198395.1	Circular genetic element sp.
2202954	GCA_004207995.1	Circular genetic element sp.
2202954	GCA_004203075.1	Circular genetic element sp.
2202954	GCA_004207175.1	Circular genetic element sp.
2202954	GCA_004207655.1	Circular genetic element sp.
2202954	GCA_004201095.1	Circular genetic element sp.
2202954	GCA_004205135.1	Circular genetic element sp.
2202954	GCA_004200055.1	Circular genetic element sp.
2202954	GCA_004203095.1	Circular genetic element sp.
2202954	GCA_004199375.1	Circular genetic element sp.
2202954	GCA_004207195.1	Circular genetic element sp.
2202954	GCA_004201115.1	Circular genetic element sp.
2202954	GCA_004199155.1	Circular genetic element sp.
2202954	GCA_004196395.1	Circular genetic element sp.
2202954	GCA_004205155.1	Circular genetic element sp.
2202954	GCA_004200075.1	Circular genetic element sp.
2202954	GCA_004198935.1	Circular genetic element sp.
2202954	GCA_004203115.1	Circular genetic element sp.
2202954	GCA_004205955.1	Circular genetic element sp.
2202954	GCA_004196415.1	Circular genetic element sp.
2202954	GCA_004205175.1	Circular genetic element sp.
2202954	GCA_004200635.1	Circular genetic element sp.
2202954	GCA_004205875.1	Circular genetic element sp.
2202954	GCA_004203135.1	Circular genetic element sp.
2202954	GCA_004201155.1	Circular genetic element sp.
2202954	GCA_004204935.1	Circular genetic element sp.
2202954	GCA_004205195.1	Circular genetic element sp.
2202954	GCA_004200095.1	Circular genetic element sp.
2202954	GCA_004204595.1	Circular genetic element sp.
2202954	GCA_004196435.1	Circular genetic element sp.
2202954	GCA_004207255.1	Circular genetic element sp.
2202954	GCA_004201175.1	Circular genetic element sp.

2202954	GCA_004205215.1	Circular genetic element sp.
2202954	GCA_004197195.1	Circular genetic element sp.
2202954	GCA_004200115.1	Circular genetic element sp.
2202954	GCA_004203175.1	Circular genetic element sp.
2202954	GCA_004199555.1	Circular genetic element sp.
2202954	GCA_004205235.1	Circular genetic element sp.
2202954	GCA_004200135.1	Circular genetic element sp.
2202954	GCA_004208015.1	Circular genetic element sp.
2202954	GCA_004198495.1	Circular genetic element sp.
2202954	GCA_004196475.1	Circular genetic element sp.
2202954	GCA_004201195.1	Circular genetic element sp.
2202954	GCA_004205255.1	Circular genetic element sp.
2202954	GCA_004207335.1	Circular genetic element sp.
2202954	GCA_004200155.1	Circular genetic element sp.
2202954	GCA_004196495.1	Circular genetic element sp.
2202954	GCA_004199395.1	Circular genetic element sp.
2202954	GCA_004207315.1	Circular genetic element sp.
2202954	GCA_004201215.1	Circular genetic element sp.
2202954	GCA_004199175.1	Circular genetic element sp.
2202954	GCA_004205275.1	Circular genetic element sp.
2202954	GCA_004200175.1	Circular genetic element sp.
2202954	GCA_004206315.1	Circular genetic element sp.
2202954	GCA_004203235.1	Circular genetic element sp.
2202954	GCA_004201235.1	Circular genetic element sp.
2202954	GCA_004205295.1	Circular genetic element sp.
2202954	GCA_004198515.1	Circular genetic element sp.
2202954	GCA_004200195.1	Circular genetic element sp.
2202954	GCA_004203255.1	Circular genetic element sp.
2202954	GCA_004201255.1	Circular genetic element sp.
2202954	GCA_004204955.1	Circular genetic element sp.
2202954	GCA_004205315.1	Circular genetic element sp.
2202954	GCA_004200215.1	Circular genetic element sp.
2202954	GCA_004196555.1	Circular genetic element sp.
2202954	GCA_004204175.1	Circular genetic element sp.
2202954	GCA_004207375.1	Circular genetic element sp.
2202954	GCA_004204275.1	Circular genetic element sp.
2202954	GCA_004201275.1	Circular genetic element sp.
2202954	GCA_004205335.1	Circular genetic element sp.
2202954	GCA_004203935.1	Circular genetic element sp.
2202954	GCA_004197775.1	Circular genetic element sp.
2202954	GCA_004200235.1	Circular genetic element sp.
2202954	GCA_004203295.1	Circular genetic element sp.
2202954	GCA_004196875.1	Circular genetic element sp.
2202954	GCA_004201295.1	Circular genetic element sp.
2202954	GCA_004205355.1	Circular genetic element sp.
2202954	GCA_004197535.1	Circular genetic element sp.
2202954	GCA_004196595.1	Circular genetic element sp.
2202954	GCA_004207695.1	Circular genetic element sp.
2202954	GCA_004201315.1	Circular genetic element sp.
2202954	GCA_004205375.1	Circular genetic element sp.

2202954	GCA_004207355.1	Circular genetic element sp.
2202954	GCA_004200255.1	Circular genetic element sp.
2202954	GCA_004196615.1	Circular genetic element sp.
2202954	GCA_004207435.1	Circular genetic element sp.
2202954	GCA_004201335.1	Circular genetic element sp.
2202954	GCA_004205395.1	Circular genetic element sp.
2202954	GCA_004200275.1	Circular genetic element sp.
2202954	GCA_004206335.1	Circular genetic element sp.
2202954	GCA_004203355.1	Circular genetic element sp.
2202954	GCA_004203155.1	Circular genetic element sp.
2202954	GCA_004205415.1	Circular genetic element sp.
2202954	GCA_004196855.1	Circular genetic element sp.
2202954	GCA_004200295.1	Circular genetic element sp.
2202954	GCA_004203375.1	Circular genetic element sp.
2202954	GCA_004205815.1	Circular genetic element sp.
2202954	GCA_004201355.1	Circular genetic element sp.
2202954	GCA_004198255.1	Circular genetic element sp.
2202954	GCA_004205435.1	Circular genetic element sp.
2202954	GCA_004198015.1	Circular genetic element sp.
2202954	GCA_004200315.1	Circular genetic element sp.
2202954	GCA_004197915.1	Circular genetic element sp.
2202954	GCA_004203875.1	Circular genetic element sp.
2202954	GCA_004196675.1	Circular genetic element sp.
2202954	GCA_004206975.1	Circular genetic element sp.
2202954	GCA_004196515.1	Circular genetic element sp.
2202954	GCA_004201375.1	Circular genetic element sp.
2202954	GCA_004205455.1	Circular genetic element sp.
2202954	GCA_004200335.1	Circular genetic element sp.
2202954	GCA_004196695.1	Circular genetic element sp.
2202954	GCA_004196895.1	Circular genetic element sp.
2202954	GCA_004197675.1	Circular genetic element sp.
2202954	GCA_004201395.1	Circular genetic element sp.
2202954	GCA_004203275.1	Circular genetic element sp.
2202954	GCA_004205475.1	Circular genetic element sp.
2202954	GCA_004208055.1	Circular genetic element sp.
2202954	GCA_004201055.1	Circular genetic element sp.
2202954	GCA_004196715.1	Circular genetic element sp.
2202954	GCA_004207715.1	Circular genetic element sp.
2202954	GCA_004205495.1	Circular genetic element sp.
2202954	GCA_004197335.1	Circular genetic element sp.
2202954	GCA_004200355.1	Circular genetic element sp.
2202954	GCA_004196735.1	Circular genetic element sp.
2202954	GCA_004207035.1	Circular genetic element sp.
2202954	GCA_004207555.1	Circular genetic element sp.
2202954	GCA_004201415.1	Circular genetic element sp.
2202954	GCA_004204795.1	Circular genetic element sp.
2202954	GCA_004205515.1	Circular genetic element sp.
2202954	GCA_004200375.1	Circular genetic element sp.
2202954	GCA_004198995.1	Circular genetic element sp.
2202954	GCA_004203475.1	Circular genetic element sp.

2202954	GCA_004201435.1	Circular genetic element sp.
2202954	GCA_004205535.1	Circular genetic element sp.
2202954	GCA_004200395.1	Circular genetic element sp.
2202954	GCA_004203335.1	Circular genetic element sp.
2202954	GCA_004196775.1	Circular genetic element sp.
2202954	GCA_004201455.1	Circular genetic element sp.
2202954	GCA_004204995.1	Circular genetic element sp.
2202954	GCA_004205555.1	Circular genetic element sp.
2202954	GCA_004197935.1	Circular genetic element sp.
2202954	GCA_004203515.1	Circular genetic element sp.
2202954	GCA_004198735.1	Circular genetic element sp.
2202954	GCA_004204315.1	Circular genetic element sp.
2202954	GCA_004201475.1	Circular genetic element sp.
2202954	GCA_004198455.1	Circular genetic element sp.
2202954	GCA_004203975.1	Circular genetic element sp.
2202954	GCA_004203535.1	Circular genetic element sp.
2202954	GCA_004196915.1	Circular genetic element sp.
2202954	GCA_004201495.1	Circular genetic element sp.
2202954	GCA_004196575.1	Circular genetic element sp.
2202954	GCA_004205595.1	Circular genetic element sp.
2202954	GCA_004208075.1	Circular genetic element sp.
2202954	GCA_004196835.1	Circular genetic element sp.
2202954	GCA_004197475.1	Circular genetic element sp.
2202954	GCA_004201515.1	Circular genetic element sp.
2202954	GCA_004205615.1	Circular genetic element sp.
2202954	GCA_004207395.1	Circular genetic element sp.
2202954	GCA_004203575.1	Circular genetic element sp.
2202954	GCA_004207675.1	Circular genetic element sp.
2202954	GCA_004201535.1	Circular genetic element sp.
2202954	GCA_004199215.1	Circular genetic element sp.
2202954	GCA_004205635.1	Circular genetic element sp.
2202954	GCA_004203595.1	Circular genetic element sp.
2202954	GCA_004201555.1	Circular genetic element sp.
2202954	GCA_004205655.1	Circular genetic element sp.
2202954	GCA_004200035.1	Circular genetic element sp.
2202954	GCA_004203615.1	Circular genetic element sp.
2202954	GCA_004201575.1	Circular genetic element sp.
2202954	GCA_004205015.1	Circular genetic element sp.
2202954	GCA_004205675.1	Circular genetic element sp.
2202954	GCA_004203635.1	Circular genetic element sp.
2202954	GCA_004197955.1	Circular genetic element sp.
2202954	GCA_004207735.1	Circular genetic element sp.
2202954	GCA_004197615.1	Circular genetic element sp.
2202954	GCA_004206915.1	Circular genetic element sp.
2202954	GCA_004201595.1	Circular genetic element sp.
2202954	GCA_004205695.1	Circular genetic element sp.
2202954	GCA_004203995.1	Circular genetic element sp.
862944	GCA_000890015.1	Circulifer tenellus virus 1
2267237	GCA_002621165.1	Citrobacter phage CF1 DK-2017
2267236	GCA_002922425.1	Citrobacter phage CF1 ERZ-2017

1455075	GCA_000915535.1	Citrobacter phage CR44b
1455076	GCA_000917035.1	Citrobacter phage CR8
1622234	GCA_001308795.2	Citrobacter phage CVT22
1673887	GCA_001502955.1	Citrobacter phage IME-CF2
2419704	GCA_003723435.1	Citrobacter phage Maleficent
1701810	GCA_002149305.1	Citrobacter phage Margaery
2315469	GCA_003668275.1	Citrobacter phage Maroon
1675602	GCA_002149285.1	Citrobacter phage Merlin
1675603	GCA_002149565.1	Citrobacter phage Michonne
1965456	GCA_002620165.1	Citrobacter phage Mijalis
1527524	GCA_000987735.1	Citrobacter phage Miller
1540094	GCA_002149585.1	Citrobacter phage Moogle
1540095	GCA_001041495.1	Citrobacter phage Moon
1701846	GCA_002624425.1	Citrobacter phage Mordin
1610508	GCA_001506035.1	Citrobacter phage phiCFP-1
2315629	GCA_003575745.1	Citrobacter phage Sazh
1805464	GCA_001744455.1	Citrobacter phage SH1
1805465	GCA_001743775.1	Citrobacter phage SH2
1805466	GCA_001745095.1	Citrobacter phage SH3
1805467	GCA_001745755.1	Citrobacter phage SH4
1805468	GCA_002609405.1	Citrobacter phage SH5
1871313	GCA_001744555.1	Citrobacter phage vB_CfrM_CfP1
2079276	GCA_002958385.2	Citrobacter phage vB_CroM_CrRp10
2079276	GCA_002958385.1	Citrobacter phage vB_CroM_CrRp10
2079275	GCA_002958375.2	Citrobacter phage vB_CroP_CrRp3
2079275	GCA_002958375.1	Citrobacter phage vB_CroP_CrRp3
1540096	GCA_001041115.1	Citrobacter virus Stevie
2053684	GCA_002957335.1	Citromicrobium phage vB_Cib_ssDNA_P1
12898	GCA_000852785.1	Citrus bark cracking viroid
12906	GCA_000855385.1	Citrus bent leaf viroid
1202142	GCA_000898955.1	Citrus chlorotic dwarf associated virus
2024604	GCA_002270765.2	Citrus concave gum-associated virus
2024604	GCA_002270765.1	Citrus concave gum-associated virus
551523	GCA_000856305.1	Citrus dwarfing viroid
1435008	GCA_000916755.1	Citrus endogenous pararetrovirus
12890	GCA_000849865.1	Citrus exocortis viroid
129141	GCA_000852305.1	Citrus leaf blotch virus
37126	GCA_000851985.1	Citrus leaf rugose virus
347219	GCA_000868185.1	Citrus leprosis virus C
2052685	GCA_002868615.1	Citrus leprosis virus C2
73561	GCA_000855005.1	Citrus psorosis virus
312008	GCA_000858145.1	Citrus sudden death-associated virus
12162	GCA_000862265.1	Citrus tristeza virus
37127	GCA_000870745.1	Citrus variegation virus
1301220	GCA_000910315.1	Citrus vein enation virus
459949	GCA_000878955.1	Citrus viroid V
697192	GCA_000884515.1	Citrus viroid VI
174178	GCA_000838245.1	Citrus yellow mosaic virus
1214459	GCA_000955035.1	Citrus yellow vein clearing virus
1297894	GCA_003726535.1	Citrus yellow vein-associated virus

1529605	GCA_000921535.1	Cladosporium cladosporioides virus 1
2052899	GCA_003972105.1	Cladosporium fulvum T-1 virus
1307957	GCA_000869305.1	Clanis bilineata nucleopolyhedrovirus
11096	GCA_900044245.1	Classical swine fever virus
11096	GCA_900089415.1	Classical swine fever virus
11096	GCA_900089195.1	Classical swine fever virus
11096	GCA_900044235.1	Classical swine fever virus
11096	GCA_900089315.1	Classical swine fever virus
11096	GCA_900089405.1	Classical swine fever virus
11096	GCA_900045915.1	Classical swine fever virus
11096	GCA_900044225.1	Classical swine fever virus
11096	GCA_900089165.1	Classical swine fever virus
11096	GCA_900039515.1	Classical swine fever virus
11096	GCA_900089135.1	Classical swine fever virus
11096	GCA_900046445.1	Classical swine fever virus
11096	GCA_900039505.1	Classical swine fever virus
11096	GCA_900089235.1	Classical swine fever virus
11096	GCA_900039495.1	Classical swine fever virus
11096	GCA_900089295.1	Classical swine fever virus
11096	GCA_900047525.1	Classical swine fever virus
11096	GCA_900089325.1	Classical swine fever virus
11096	GCA_900089355.1	Classical swine fever virus
11096	GCA_900089155.1	Classical swine fever virus
11096	GCA_900089285.1	Classical swine fever virus
11096	GCA_900045925.1	Classical swine fever virus
11096	GCA_900089375.1	Classical swine fever virus
11096	GCA_900039525.1	Classical swine fever virus
11096	GCA_900089385.1	Classical swine fever virus
11096	GCA_900089265.1	Classical swine fever virus
358769	GCA_003034095.1	Classical swine fever virus - Alfort/187
149596	GCA_000864685.1	Classical swine fever virus strain Eystrup
686439	GCA_000887075.1	Clavibacter phage CMP1
1406793	GCA_000914735.1	Clavibacter phage CN1A
1950126	GCA_002005705.1	Clematis chlorotic mottle virus
2069321	GCA_004128675.1	Cleome droserifolia amalgavirus 1
858517	GCA_000893495.1	Cleome golden mosaic virus
2169824	GCA_000890255.1	Cleome leaf crumple alphasatellite
666144	GCA_000894195.1	Cleome leaf crumple virus
390438	GCA_000875165.1	Clerodendron golden mosaic virus
326811	GCA_000873145.1	Clerodendron yellow mosaic virus
559878	GCA_000882255.1	Clerodendrum golden mosaic China virus
559878	GCA_002986355.1	Clerodendrum golden mosaic China virus
649509	GCA_002986365.1	Clerodendrum golden mosaic Jiangsu virus
1162083	GCA_000896075.1	Clitocybe odora virus
1128119	GCA_000896575.1	Clitoria yellow mottle virus
1810952	GCA_002145585.1	Clo Mor virus
283675	GCA_000890975.1	Clostera anachoreta granulovirus
1986290	GCA_002819225.1	Clostera anastomosis granulovirus B
1986291	GCA_000911215.1	Clostera anastomosis granulovirus Henan
2069615	GCA_003051305.1	Clostridioides phage LIBA2945

2069614	GCA_003051285.1	Clostridioides phage LIBA6276
1971232	GCA_002621305.1	Clostridioides phage phiSemix9P1
1868595	GCA_002610875.1	Clostridium phage CDKM15
1868594	GCA_002610845.1	Clostridium phage CDKM9
1411095	GCA_000922715.1	Clostridium phage CDMH1
1755693	GCA_002608065.1	Clostridium phage CDSH1
1927016	GCA_002617465.1	Clostridium phage Clo-PEP-1
1983541	GCA_002743535.1	Clostridium phage CPS1
2175605	GCA_003364375.1	Clostridium phage CPS2
12336	GCA_000865225.1	Clostridium phage c-st
1779843	GCA_002609125.1	Clostridium phage HM T
1874333	GCA_900093805.1	Clostridium phage HM2
1128071	GCA_000901795.1	Clostridium phage phi24R
190478	GCA_000839145.1	Clostridium phage phi3626
1147137	GCA_000904815.1	Clostridium phage phi8074-B1
1582150	GCA_001504535.1	Clostridium phage phiCD111
1582151	GCA_001503755.1	Clostridium phage phiCD146
1582152	GCA_002277885.1	Clostridium phage phiCD211
1582149	GCA_003147205.1	Clostridium phage phiCD24-1
1032362	GCA_000891135.1	Clostridium phage phiCD38-2
1582153	GCA_001505375.1	Clostridium phage phiCD481-1
1582154	GCA_001506015.1	Clostridium phage phiCD505
1582155	GCA_001504555.1	Clostridium phage phiCD506
864178	GCA_000893275.1	Clostridium phage phiCD6356
1445553	GCA_001504815.1	Clostridium phage phiCDHM11
1437363	GCA_001550945.1	Clostridium phage phiCDHM13
1522091	GCA_003146985.1	Clostridium phage phiCDHM14
1522092	GCA_001501695.1	Clostridium phage phiCDHM19
1042122	GCA_000901755.1	Clostridium phage phiCP13O
673376	GCA_000903475.1	Clostridium phage phiCP26F
1042123	GCA_000903275.1	Clostridium phage phiCP34O
541865	GCA_000882235.1	Clostridium phage phiCP39-O
1162304	GCA_000897195.1	Clostridium phage phiCP7R
1042124	GCA_002630625.1	Clostridium phage phiCP9O
1162305	GCA_000899755.1	Clostridium phage phiCPV4
1567009	GCA_002149505.1	Clostridium phage phiCT19406A
1567010	GCA_002149685.1	Clostridium phage phiCT19406B
1567011	GCA_001505355.1	Clostridium phage phiCT19406C
1567012	GCA_002149745.1	Clostridium phage phiCT453A
1567013	GCA_002149525.1	Clostridium phage phiCT453B
1567014	GCA_001503735.1	Clostridium phage phiCT9441A
1567015	GCA_002149705.1	Clostridium phage phiCTC2A
1567016	GCA_002149825.1	Clostridium phage phiCTC2B
871584	GCA_000890075.1	Clostridium phage phiCTP1
1582156	GCA_001503775.1	Clostridium phage phiMMP01
1204534	GCA_000901555.1	Clostridium phage phiMMP02
1582157	GCA_001505395.1	Clostridium phage phiMMP03
1204535	GCA_000900495.1	Clostridium phage phiMMP04
1187894	GCA_000896275.1	Clostridium phage PhiS63
396359	GCA_001275475.1	Clostridium phage phiSM101

1162306	GCA_000897315.1	Clostridium phage phiZP2
2316154	GCA_003575845.1	Clostridium phage susfortuna
1305708	GCA_000909215.1	Clostridium phage vB_CpeS-CP51
262071	GCA_000870565.1	Clostridium virus phiC2
320122	GCA_000864625.1	Clostridium virus phiCD119
559189	GCA_000881655.1	Clostridium virus phiCD27
12177	GCA_000849905.1	Clover yellow mosaic virus
12198	GCA_000861485.1	Clover yellow vein virus
2340808	GCA_004133085.1	Cluster bean endornavirus 1
1750712	GCA_001567015.1	Cnaphalocrocis medinalis granulovirus
1747500	GCA_003029265.1	Cnidoscolus mosaic leaf deformation virus
1747500	GCA_003029265.2	Cnidoscolus mosaic leaf deformation virus
764599	GCA_000924775.1	Coastal Plains virus
50713	GCA_001440915.1	Cocal virus
1532882	GCA_000922555.1	Coccinia mosaic Tamil Nadu virus
1890365	GCA_001717335.1	Coccinia mottle virus
2050038	GCA_000905135.1	Cockroach associated cyclovirus 1
479060	GCA_000875565.1	Cocksfoot mild mosaic virus
40979	GCA_000855085.1	Cocksfoot mottle virus
192452	GCA_000862565.1	Cocksfoot streak virus
36453	GCA_000848145.1	Coconut cadang-cadang viroid
2161876	GCA_004132025.1	Coconut foliar decay alphasatellite 3
2161876	GCA_004132045.1	Coconut foliar decay alphasatellite 3
2161879	GCA_004132065.1	Coconut foliar decay alphasatellite 6
2161880	GCA_004132085.1	Coconut foliar decay alphasatellite 7
12474	GCA_000837045.1	Coconut foliar decay virus
12889	GCA_000848305.1	Coconut tinangaja viroid
745716	GCA_002867045.1	Coffee ringspot dichorhavirus
12899	GCA_000850865.1	Coleus blumei viroid
192025	GCA_000852905.1	Coleus blumei viroid 1
53188	GCA_000851325.1	Coleus blumei viroid 2-RL
53190	GCA_000852105.1	Coleus blumei viroid 3-FR
598747	GCA_000883615.1	Coleus blumei viroid 5
651134	GCA_000883995.1	Coleus blumei viroid 6
404404	GCA_000871925.1	Coleus vein necrosis virus
2304034	GCA_004117455.1	Colletotrichum fructicola chrysovirus 1
1565088	GCA_001431895.1	Colletotrichum higginsianum non-segmented dsRNA virus [illegible]
889813	GCA_000892175.1	Colobus guereza papillomavirus 2
889812	GCA_003178755.1	Colobus guereza papillomavirus type 1
1775456	GCA_002146065.1	Colocasia bobone disease-associated virus
91613	GCA_000903975.1	Colombian datura virus
1758139	GCA_001502195.1	Colombian potato soil-borne virus
46839	GCA_000853265.1	Colorado tick fever virus
93386	GCA_002116095.1	Columbid alphaherpesvirus 1
126070	GCA_000843885.1	Columbid circovirus
126070	GCA_004031415.1	Columbid circovirus
126070	GCA_004031435.1	Columbid circovirus
126070	GCA_004032595.1	Columbid circovirus
126070	GCA_004032555.1	Columbid circovirus
126070	GCA_004035715.1	Columbid circovirus

126070	GCA_004032575.1	Columbid circovirus
126070	GCA_004035695.1	Columbid circovirus
126070	GCA_004032615.1	Columbid circovirus
126070	GCA_004039835.1	Columbid circovirus
126070	GCA_004070615.1	Columbid circovirus
126070	GCA_004070635.1	Columbid circovirus
126070	GCA_004070655.1	Columbid circovirus
126070	GCA_004070675.1	Columbid circovirus
126070	GCA_004070695.1	Columbid circovirus
126070	GCA_004070715.1	Columbid circovirus
126070	GCA_004077475.1	Columbid circovirus
126070	GCA_004070735.1	Columbid circovirus
126070	GCA_004070755.1	Columbid circovirus
126070	GCA_004070775.1	Columbid circovirus
126070	GCA_004070795.1	Columbid circovirus
126070	GCA_004070815.1	Columbid circovirus
126070	GCA_004070835.1	Columbid circovirus
126070	GCA_004077495.1	Columbid circovirus
126070	GCA_004070855.1	Columbid circovirus
126070	GCA_004070875.1	Columbid circovirus
126070	GCA_004070895.1	Columbid circovirus
126070	GCA_004070915.1	Columbid circovirus
126070	GCA_004070935.1	Columbid circovirus
126070	GCA_004070955.1	Columbid circovirus
126070	GCA_004070975.1	Columbid circovirus
126070	GCA_004070995.1	Columbid circovirus
126070	GCA_004071015.1	Columbid circovirus
126070	GCA_004071035.1	Columbid circovirus
126070	GCA_004071055.1	Columbid circovirus
126070	GCA_004071075.1	Columbid circovirus
126070	GCA_004071095.1	Columbid circovirus
126070	GCA_004071115.1	Columbid circovirus
126070	GCA_004071135.1	Columbid circovirus
126070	GCA_004071155.1	Columbid circovirus
126070	GCA_004071175.1	Columbid circovirus
126070	GCA_004071195.1	Columbid circovirus
126070	GCA_004063075.1	Columbid circovirus
126070	GCA_004063095.1	Columbid circovirus
126070	GCA_004084515.1	Columbid circovirus
126070	GCA_004070015.1	Columbid circovirus
126070	GCA_004070035.1	Columbid circovirus
126070	GCA_004070055.1	Columbid circovirus
126070	GCA_004070075.1	Columbid circovirus
126070	GCA_004070095.1	Columbid circovirus
126070	GCA_004070115.1	Columbid circovirus
126070	GCA_004094955.1	Columbid circovirus
126070	GCA_004098635.1	Columbid circovirus
126070	GCA_004098735.1	Columbid circovirus
126070	GCA_004098755.1	Columbid circovirus
126070	GCA_004098775.1	Columbid circovirus

126070	GCA_004098655.1	Columbid circovirus
126070	GCA_004098675.1	Columbid circovirus
126070	GCA_004098695.1	Columbid circovirus
126070	GCA_004098715.1	Columbid circovirus
12901	GCA_000850525.1	Columnea latent viroid
765765	GCA_000898315.1	Colwellia phage 9A
10653	GCA_000849585.1	Commelina yellow mottle virus
1915202	GCA_003029365.1	Common bean mottle virus
1915202	GCA_003029365.2	Common bean mottle virus
1915203	GCA_002366145.2	Common bean severe mosaic virus
1915203	GCA_002366145.1	Common bean severe mosaic virus
1915203	GCA_003029375.1	Common bean severe mosaic virus
1897538	GCA_001725855.1	Common bean-associated gemycircularvirus
1897538	GCA_003849065.1	Common bean-associated gemycircularvirus
1897538	GCA_003849085.1	Common bean-associated gemycircularvirus
2022783	GCA_002210635.1	Common bottlenose dolphin gammaherpesvirus 1 strain Sa
540070	GCA_003033105.1	Common midwife toad virus
1159902	GCA_000896895.1	Common moorhen coronavirus HKU21
1737523	GCA_001430575.1	Common vole polyomavirus
1592576	GCA_000931335.1	Condylorrhiza vestigialis MNPV
210827	GCA_000866765.1	Coniothyrium minitans RNA virus
1972685	GCA_002366165.1	Coot Bay almendravirus
390436	GCA_000873405.1	Corchorus golden mosaic virus
582838	GCA_002997365.1	Corchorus golden mosaic virus [India:Bahraich:2008]
403897	GCA_000867685.1	Corchorus yellow spot virus
1297646	GCA_000904215.1	Corchorus yellow vein mosaic betasatellite
1297645	GCA_000906695.1	Corchorus yellow vein mosaic virus
293284	GCA_000845265.1	Corchorus yellow vein virus - [Hoa Binh]
1955175	GCA_002024695.1	Cordoba virus
937809	GCA_002820345.1	Cordyline virus 1
1911439	GCA_001866325.1	Corey virus
1964806	GCA_002194405.1	Coronavirus AcCoV-JC34
40053	GCA_002829365.1	Corriparta virus
1891748	GCA_000868965.1	Corvus monedula polyomavirus 1
28358	GCA_000874185.2	Corynebacterium phage BFK20
2047868	GCA_002956465.1	Corynebacterium phage C3PO
2483718	GCA_003722415.1	Corynebacterium phage Cruella
2047869	GCA_002956475.1	Corynebacterium phage Darwin
1965535	GCA_002620985.1	Corynebacterium phage IME1320_01
2301600	GCA_003601415.1	Corynebacterium phage Juicebox
2483719	GCA_003723395.1	Corynebacterium phage Kimchi1738
1972433	GCA_002622125.1	Corynebacterium phage LGCM-V2
1972434	GCA_002622145.1	Corynebacterium phage LGCM-V3
1972435	GCA_002622165.1	Corynebacterium phage LGCM-V4
1972436	GCA_002622185.1	Corynebacterium phage LGCM-V5
1972437	GCA_002622205.1	Corynebacterium phage LGCM-V6
1972438	GCA_002622225.1	Corynebacterium phage LGCM-V7
1972439	GCA_002622245.1	Corynebacterium phage LGCM-V8
1972440	GCA_002622265.1	Corynebacterium phage LGCM-V9
1965283	GCA_002619825.1	Corynebacterium phage LGCM-VI

384848	GCA_000874205.1	Corynebacterium phage P1201
2483663	GCA_003722955.1	Corynebacterium phage PeteyPab
1927022	GCA_002617565.1	Corynebacterium phage phi16
2052821	GCA_003004815.1	Corynebacterium phage phi673
2052822	GCA_003004825.1	Corynebacterium phage phi674
2047870	GCA_002956485.1	Corynebacterium phage PotatoChip
2015851	GCA_002626265.1	Corynebacterium phage Poushou
2301601	GCA_003601335.1	Corynebacterium phage SamW
2201425	GCA_003308135.1	Corynebacterium phage TouchMeNot
2483720	GCA_003722495.1	Corynebacterium phage Troy
2047871	GCA_002956495.1	Corynebacterium phage Zion
1330491	GCA_000885595.1	Cosavirus A
2003652	GCA_002117635.1	Cosavirus F
1582094	GCA_000930055.1	Cosavirus JMY-2014
39640	GCA_000844405.1	Cotesia congregata bracovirus
47223	GCA_002836525.1	Cotesia rubecula bracovirus
930275	GCA_000894375.1	Cotia virus SPAn232
1396815	GCA_002867455.1	Cotton chlorotic spot virus
335400	GCA_000845165.1	Cotton leaf crumple virus - [Mexico : Sonora]
335398	GCA_002822005.1	Cotton leaf crumple virus - [TX]
222456	GCA_002822045.1	Cotton leaf curl Alabad virus
222456	GCA_002822065.1	Cotton leaf curl Alabad virus
222457	GCA_000842045.1	Cotton leaf curl Alabad virus-[802a]
908137	GCA_000890455.1	Cotton leaf curl Allahabad virus [India:Karnal:OY77:2005]
908136	GCA_002822025.1	Cotton leaf curl Allahabad virus [India:Karnal:OY81B:2005]
307111	GCA_000865005.1	Cotton leaf curl Bangalore betasatellite
307110	GCA_000865045.1	Cotton leaf curl Bangalore virus
134386	GCA_000845565.1	Cotton leaf curl betasatellite
713091	GCA_000884675.1	Cotton leaf curl Burewala alphasatellite
713092	GCA_000887135.1	Cotton leaf curl Burewala betasatellite
620894	GCA_002986455.1	Cotton leaf curl Burewala virus
418448	GCA_000882715.1	Cotton leaf curl Burewala virus - [India:Vehari:2006]
679536	GCA_000875105.1	Cotton leaf curl Gezira alphasatellite
679536	GCA_000886955.1	Cotton leaf curl Gezira alphasatellite
679536	GCA_003073075.1	Cotton leaf curl Gezira alphasatellite
304222	GCA_000858125.1	Cotton leaf curl Gezira betasatellite
304222	GCA_002830045.1	Cotton leaf curl Gezira betasatellite
222459	GCA_000838805.1	Cotton leaf curl Gezira virus
222459	GCA_002822105.1	Cotton leaf curl Gezira virus
222459	GCA_002822165.1	Cotton leaf curl Gezira virus
627501	GCA_002986415.1	Cotton leaf curl Gezira virus-[Cameroon]
222460	GCA_002822085.1	Cotton leaf curl Gezira virus-[Cotton]
679314	GCA_002986375.1	Cotton leaf curl Gezira virus-[okra:BFA]
502871	GCA_002822145.1	Cotton leaf curl Gezira virus-[okra:Niger]
222464	GCA_002822185.1	Cotton leaf curl Kokhran virus
222465	GCA_000840425.1	Cotton leaf curl Kokhran virus-[806b]
2169841	GCA_000890935.1	Cotton leaf curl Lucknow alphasatellite
1048668	GCA_000897295.1	Cotton leaf curl Multan alphasatellite
306025	GCA_000866685.1	Cotton leaf curl Multan betasatellite
306025	GCA_002987865.1	Cotton leaf curl Multan betasatellite

223252	GCA_000839845.1	Cotton leaf curl Multan virus
223252	GCA_002822225.1	Cotton leaf curl Multan virus
260479	GCA_000866125.1	Cotton leaf curl Multan virus satellite U36-1
223254	GCA_000841225.1	Cotton leaf curl Multan virus-[62]
223256	GCA_002986465.1	Cotton leaf curl Multan virus-[Faisalabad3]
1143709	GCA_002822205.1	Cotton leaf curl Multan virus-[Hibiscus rosa-sinensis]
674996	GCA_001593375.1	Cotton leaf curl Shahdadpur virus
53010	GCA_001876875.1	Cotton leaf curl virus
189515	GCA_000897035.1	Cotton leaf curl virus betasatellite
312295	GCA_000890175.1	Cotton leafroll dwarf virus
79236	GCA_001669825.1	Cotton yellow mosaic virus
79236	GCA_003029335.1	Cotton yellow mosaic virus
79236	GCA_003029335.2	Cotton yellow mosaic virus
2056780	GCA_004117295.1	Cow vetch latent virus
12199	GCA_000861665.1	Cowpea aphid-borne mosaic virus
12303	GCA_000851205.1	Cowpea chlorotic mottle virus
223260	GCA_002822245.1	Cowpea golden mosaic virus-[Nigeria]
67761	GCA_000888775.1	Cowpea mild mottle virus
12264	GCA_000860385.1	Cowpea mosaic virus
12627	GCA_000851965.1	Cowpea mottle virus
1913124	GCA_002080275.1	Cowpea polerovirus 1
1913125	GCA_002080295.1	Cowpea polerovirus 2
292627	GCA_000858085.1	Cowpea severe leaf curl-associated DNA beta
12261	GCA_000861225.1	Cowpea severe mosaic virus
10243	GCA_000839185.1	Cowpox virus
10243	GCA_004025355.1	Cowpox virus
10243	GCA_004025395.1	Cowpox virus
10243	GCA_003971385.1	Cowpox virus
10243	GCA_004025425.1	Cowpox virus
10243	GCA_004025455.1	Cowpox virus
10243	GCA_004025495.1	Cowpox virus
10243	GCA_004025545.1	Cowpox virus
10243	GCA_003971405.1	Cowpox virus
10243	GCA_004025605.1	Cowpox virus
10243	GCA_004025655.1	Cowpox virus
10243	GCA_004025885.1	Cowpox virus
10243	GCA_004025695.1	Cowpox virus
10243	GCA_004025765.1	Cowpox virus
10243	GCA_004025815.1	Cowpox virus
10243	GCA_004025855.1	Cowpox virus
10243	GCA_004025905.1	Cowpox virus
10243	GCA_900187685.1	Cowpox virus
10243	GCA_900187705.1	Cowpox virus
10243	GCA_900187755.1	Cowpox virus
10243	GCA_900187695.1	Cowpox virus
10243	GCA_900187745.1	Cowpox virus
10243	GCA_900187765.1	Cowpox virus
10243	GCA_900187715.1	Cowpox virus
10243	GCA_900187835.1	Cowpox virus
10243	GCA_900327605.1	Cowpox virus

10243	GCA_900187845.1	Cowpox virus
10243	GCA_900187735.1	Cowpox virus
10243	GCA_900187795.1	Cowpox virus
10243	GCA_900327585.1	Cowpox virus
10243	GCA_900187775.2	Cowpox virus
10243	GCA_900327555.1	Cowpox virus
10243	GCA_900327535.1	Cowpox virus
10243	GCA_900327495.1	Cowpox virus
10243	GCA_900187675.1	Cowpox virus
10243	GCA_900187725.1	Cowpox virus
10243	GCA_900187825.1	Cowpox virus
10243	GCA_900187665.1	Cowpox virus
31704	GCA_000861905.1	Coxsackievirus A16
33757	GCA_002816655.1	Coxsackievirus A2
42782	GCA_003266285.1	Coxsackievirus A20
42782	GCA_003266345.1	Coxsackievirus A20
42782	GCA_003266205.1	Coxsackievirus A20
42782	GCA_003266245.1	Coxsackievirus A20
42782	GCA_003266265.1	Coxsackievirus A20
42782	GCA_003266305.1	Coxsackievirus A20
42782	GCA_003266325.1	Coxsackievirus A20
42782	GCA_003266365.1	Coxsackievirus A20
42782	GCA_003266505.1	Coxsackievirus A20
42782	GCA_003266225.1	Coxsackievirus A20
42783	GCA_003266385.1	Coxsackievirus A22
42783	GCA_003266405.1	Coxsackievirus A22
42783	GCA_003266465.1	Coxsackievirus A22
42783	GCA_003266445.1	Coxsackievirus A22
42783	GCA_003266485.1	Coxsackievirus A22
42783	GCA_003266425.1	Coxsackievirus A22
12089	GCA_003108505.1	Coxsackievirus A24
12071	GCA_000861325.1	Coxsackievirus B1
12072	GCA_002816685.1	Coxsackievirus B3
2480172	GCA_004132265.1	Cragig virus 1
417288	GCA_000869725.1	Crassocephalum yellow vein virus - Jinghong
2212563	GCA_003456955.1	CrAssphage sp.
2212563	GCA_003456995.1	CrAssphage sp.
2482789	GCA_003718755.1	CrAssphage sp. O-152
2202563	GCA_003618855.1	CRESS virus sp.
2202563	GCA_003618775.1	CRESS virus sp.
2202563	GCA_003616755.1	CRESS virus sp.
2202563	GCA_003615435.1	CRESS virus sp.
2202563	GCA_003616815.1	CRESS virus sp.
2202563	GCA_003615375.1	CRESS virus sp.
2202563	GCA_003618795.1	CRESS virus sp.
2202563	GCA_003618455.1	CRESS virus sp.
2202563	GCA_003618115.1	CRESS virus sp.
2202563	GCA_003616875.1	CRESS virus sp.
2202563	GCA_003617435.1	CRESS virus sp.
2202563	GCA_003616895.1	CRESS virus sp.

2202563	GCA_003617095.1	CRESS virus sp.
2202563	GCA_003619015.1	CRESS virus sp.
2202563	GCA_003618815.1	CRESS virus sp.
2202563	GCA_003618475.1	CRESS virus sp.
2202563	GCA_003619035.1	CRESS virus sp.
2202563	GCA_003617795.1	CRESS virus sp.
2202563	GCA_003619055.1	CRESS virus sp.
2202563	GCA_003619075.1	CRESS virus sp.
2202563	GCA_003615395.1	CRESS virus sp.
2202563	GCA_003617035.1	CRESS virus sp.
2202563	GCA_003617415.1	CRESS virus sp.
2202563	GCA_003616095.1	CRESS virus sp.
2202563	GCA_003619095.1	CRESS virus sp.
2202563	GCA_003615755.1	CRESS virus sp.
2202563	GCA_003617075.1	CRESS virus sp.
2202563	GCA_003619115.1	CRESS virus sp.
2202563	GCA_003618095.1	CRESS virus sp.
2202563	GCA_003616375.1	CRESS virus sp.
2202563	GCA_003619135.1	CRESS virus sp.
2202563	GCA_003616735.1	CRESS virus sp.
2202563	GCA_003617975.1	CRESS virus sp.
2202563	GCA_003619155.1	CRESS virus sp.
2202563	GCA_003619175.1	CRESS virus sp.
2202563	GCA_003617615.1	CRESS virus sp.
2202563	GCA_003617135.1	CRESS virus sp.
2202563	GCA_003616795.1	CRESS virus sp.
2202563	GCA_003619195.1	CRESS virus sp.
2202563	GCA_003618715.1	CRESS virus sp.
2202563	GCA_003617175.1	CRESS virus sp.
2202563	GCA_003619235.1	CRESS virus sp.
2202563	GCA_003619275.1	CRESS virus sp.
2202563	GCA_003617835.1	CRESS virus sp.
2202563	GCA_003617495.1	CRESS virus sp.
2202563	GCA_003619315.1	CRESS virus sp.
2202563	GCA_003617275.1	CRESS virus sp.
2202563	GCA_003619335.1	CRESS virus sp.
2202563	GCA_003615795.1	CRESS virus sp.
2202563	GCA_003619355.1	CRESS virus sp.
2202563	GCA_003617315.1	CRESS virus sp.
2202563	GCA_003619555.1	CRESS virus sp.
2202563	GCA_003619375.1	CRESS virus sp.
2202563	GCA_003619215.1	CRESS virus sp.
2202563	GCA_003617335.1	CRESS virus sp.
2202563	GCA_003617855.1	CRESS virus sp.
2202563	GCA_003619415.1	CRESS virus sp.
2202563	GCA_003616835.1	CRESS virus sp.
2202563	GCA_003619435.1	CRESS virus sp.
2202563	GCA_003619455.1	CRESS virus sp.
2202563	GCA_003615475.1	CRESS virus sp.
2202563	GCA_003617155.1	CRESS virus sp.

2202563	GCA_003619475.1	CRESS virus sp.
2202563	GCA_003619815.1	CRESS virus sp.
2202563	GCA_003619495.1	CRESS virus sp.
2202563	GCA_003617455.1	CRESS virus sp.
2202563	GCA_003618895.1	CRESS virus sp.
2202563	GCA_003619515.1	CRESS virus sp.
2202563	GCA_003618215.1	CRESS virus sp.
2202563	GCA_003617475.1	CRESS virus sp.
2202563	GCA_003617875.1	CRESS virus sp.
2202563	GCA_003619535.1	CRESS virus sp.
2202563	GCA_003617195.1	CRESS virus sp.
2202563	GCA_003617515.1	CRESS virus sp.
2202563	GCA_003616175.1	CRESS virus sp.
2202563	GCA_003619575.1	CRESS virus sp.
2202563	GCA_003615835.1	CRESS virus sp.
2202563	GCA_003617535.1	CRESS virus sp.
2202563	GCA_003615495.1	CRESS virus sp.
2202563	GCA_003618875.1	CRESS virus sp.
2202563	GCA_003616955.1	CRESS virus sp.
2202563	GCA_003619595.1	CRESS virus sp.
2202563	GCA_003619615.1	CRESS virus sp.
2202563	GCA_003619255.1	CRESS virus sp.
2202563	GCA_003618915.1	CRESS virus sp.
2202563	GCA_003617555.1	CRESS virus sp.
2202563	GCA_003617215.1	CRESS virus sp.
2202563	GCA_003619675.1	CRESS virus sp.
2202563	GCA_003617635.1	CRESS virus sp.
2202563	GCA_003615575.1	CRESS virus sp.
2202563	GCA_003615515.1	CRESS virus sp.
2202563	GCA_003619755.1	CRESS virus sp.
2202563	GCA_003618935.1	CRESS virus sp.
2202563	GCA_003618595.1	CRESS virus sp.
2202563	GCA_003618255.1	CRESS virus sp.
2202563	GCA_003617915.1	CRESS virus sp.
2202563	GCA_003615675.1	CRESS virus sp.
2202563	GCA_003619775.1	CRESS virus sp.
2202563	GCA_003617575.1	CRESS virus sp.
2202563	GCA_003617735.1	CRESS virus sp.
2202563	GCA_003615695.1	CRESS virus sp.
2202563	GCA_003617755.1	CRESS virus sp.
2202563	GCA_003615715.1	CRESS virus sp.
2202563	GCA_003617235.1	CRESS virus sp.
2202563	GCA_003617775.1	CRESS virus sp.
2202563	GCA_003615535.1	CRESS virus sp.
2202563	GCA_003615735.1	CRESS virus sp.
2202563	GCA_003617395.1	CRESS virus sp.
2202563	GCA_003619635.1	CRESS virus sp.
2202563	GCA_003619295.1	CRESS virus sp.
2202563	GCA_003617815.1	CRESS virus sp.
2202563	GCA_003618955.1	CRESS virus sp.

2202563	GCA_003615775.1	CRESS virus sp.
2202563	GCA_003618615.1	CRESS virus sp.
2202563	GCA_003617935.1	CRESS virus sp.
2202563	GCA_003617595.1	CRESS virus sp.
2202563	GCA_003617255.1	CRESS virus sp.
2202563	GCA_003615815.1	CRESS virus sp.
2202563	GCA_003616235.1	CRESS virus sp.
2202563	GCA_003615555.1	CRESS virus sp.
2202563	GCA_003615855.1	CRESS virus sp.
2202563	GCA_003615875.1	CRESS virus sp.
2202563	GCA_003618975.1	CRESS virus sp.
2202563	GCA_003615895.1	CRESS virus sp.
2202563	GCA_003618295.1	CRESS virus sp.
2202563	GCA_003617955.1	CRESS virus sp.
2202563	GCA_003615915.1	CRESS virus sp.
2202563	GCA_003617895.1	CRESS virus sp.
2202563	GCA_003616935.1	CRESS virus sp.
2202563	GCA_003615975.1	CRESS virus sp.
2202563	GCA_003618035.1	CRESS virus sp.
2202563	GCA_003619735.1	CRESS virus sp.
2202563	GCA_003615455.1	CRESS virus sp.
2202563	GCA_003618995.1	CRESS virus sp.
2202563	GCA_003615335.1	CRESS virus sp.
2202563	GCA_003617295.1	CRESS virus sp.
2202563	GCA_003616055.1	CRESS virus sp.
2202563	GCA_003616075.1	CRESS virus sp.
2202563	GCA_003615935.1	CRESS virus sp.
2202563	GCA_003615595.1	CRESS virus sp.
2202563	GCA_003618155.1	CRESS virus sp.
2202563	GCA_003619695.1	CRESS virus sp.
2202563	GCA_003616115.1	CRESS virus sp.
2202563	GCA_003618175.1	CRESS virus sp.
2202563	GCA_003618135.1	CRESS virus sp.
2202563	GCA_003615635.1	CRESS virus sp.
2202563	GCA_003616135.1	CRESS virus sp.
2202563	GCA_003618195.1	CRESS virus sp.
2202563	GCA_003617995.1	CRESS virus sp.
2202563	GCA_003616155.1	CRESS virus sp.
2202563	GCA_003617655.1	CRESS virus sp.
2202563	GCA_003618415.1	CRESS virus sp.
2202563	GCA_003616975.1	CRESS virus sp.
2202563	GCA_003616635.1	CRESS virus sp.
2202563	GCA_003618235.1	CRESS virus sp.
2202563	GCA_003616295.1	CRESS virus sp.
2202563	GCA_003616195.1	CRESS virus sp.
2202563	GCA_003615955.1	CRESS virus sp.
2202563	GCA_003616215.1	CRESS virus sp.
2202563	GCA_003617115.1	CRESS virus sp.
2202563	GCA_003618275.1	CRESS virus sp.
2202563	GCA_003615615.1	CRESS virus sp.

2202563	GCA_003619715.1	CRESS virus sp.
2202563	GCA_003616775.1	CRESS virus sp.
2202563	GCA_003616255.1	CRESS virus sp.
2202563	GCA_003618315.1	CRESS virus sp.
2202563	GCA_003616275.1	CRESS virus sp.
2202563	GCA_003617675.1	CRESS virus sp.
2202563	GCA_003618335.1	CRESS virus sp.
2202563	GCA_003616995.1	CRESS virus sp.
2202563	GCA_003618355.1	CRESS virus sp.
2202563	GCA_003616315.1	CRESS virus sp.
2202563	GCA_003616335.1	CRESS virus sp.
2202563	GCA_003616355.1	CRESS virus sp.
2202563	GCA_003616915.1	CRESS virus sp.
2202563	GCA_003619395.1	CRESS virus sp.
2202563	GCA_003615415.1	CRESS virus sp.
2202563	GCA_003618375.1	CRESS virus sp.
2202563	GCA_003618435.1	CRESS virus sp.
2202563	GCA_003616395.1	CRESS virus sp.
2202563	GCA_003617695.1	CRESS virus sp.
2202563	GCA_003617355.1	CRESS virus sp.
2202563	GCA_003616415.1	CRESS virus sp.
2202563	GCA_003617015.1	CRESS virus sp.
2202563	GCA_003619795.1	CRESS virus sp.
2202563	GCA_003616435.1	CRESS virus sp.
2202563	GCA_003615995.1	CRESS virus sp.
2202563	GCA_003618495.1	CRESS virus sp.
2202563	GCA_003615655.1	CRESS virus sp.
2202563	GCA_003616455.1	CRESS virus sp.
2202563	GCA_003618515.1	CRESS virus sp.
2202563	GCA_003616475.1	CRESS virus sp.
2202563	GCA_003618535.1	CRESS virus sp.
2202563	GCA_003616495.1	CRESS virus sp.
2202563	GCA_003618735.1	CRESS virus sp.
2202563	GCA_003618395.1	CRESS virus sp.
2202563	GCA_003618555.1	CRESS virus sp.
2202563	GCA_003618055.1	CRESS virus sp.
2202563	GCA_003616515.1	CRESS virus sp.
2202563	GCA_003617715.1	CRESS virus sp.
2202563	GCA_003618575.1	CRESS virus sp.
2202563	GCA_003617375.1	CRESS virus sp.
2202563	GCA_003616535.1	CRESS virus sp.
2202563	GCA_003616555.1	CRESS virus sp.
2202563	GCA_003616015.1	CRESS virus sp.
2202563	GCA_003616575.1	CRESS virus sp.
2202563	GCA_003618635.1	CRESS virus sp.
2202563	GCA_003616595.1	CRESS virus sp.
2202563	GCA_003616855.1	CRESS virus sp.
2202563	GCA_003618655.1	CRESS virus sp.
2202563	GCA_003615355.1	CRESS virus sp.
2202563	GCA_003616615.1	CRESS virus sp.

2202563	GCA_003618015.1	CRESS virus sp.
2202563	GCA_003618675.1	CRESS virus sp.
2202563	GCA_003618075.1	CRESS virus sp.
2202563	GCA_003618695.1	CRESS virus sp.
2202563	GCA_003616655.1	CRESS virus sp.
2202563	GCA_003617055.1	CRESS virus sp.
2202563	GCA_003616715.1	CRESS virus sp.
2202563	GCA_003618835.1	CRESS virus sp.
2202563	GCA_003616675.1	CRESS virus sp.
2202563	GCA_003616035.1	CRESS virus sp.
2202563	GCA_003616695.1	CRESS virus sp.
2202563	GCA_003618755.1	CRESS virus sp.
2202563	GCA_003619655.1	CRESS virus sp.
2202563	GCA_003622875.1	CRESS virus sp.
2202563	GCA_003620835.1	CRESS virus sp.
2202563	GCA_003624935.1	CRESS virus sp.
2202563	GCA_003622895.1	CRESS virus sp.
2202563	GCA_003620855.1	CRESS virus sp.
2202563	GCA_003624955.1	CRESS virus sp.
2202563	GCA_003622915.1	CRESS virus sp.
2202563	GCA_003624975.1	CRESS virus sp.
2202563	GCA_003622935.1	CRESS virus sp.
2202563	GCA_003620895.1	CRESS virus sp.
2202563	GCA_003624995.1	CRESS virus sp.
2202563	GCA_003620915.1	CRESS virus sp.
2202563	GCA_003625015.1	CRESS virus sp.
2202563	GCA_003622975.1	CRESS virus sp.
2202563	GCA_003620935.1	CRESS virus sp.
2202563	GCA_003625035.1	CRESS virus sp.
2202563	GCA_003622995.1	CRESS virus sp.
2202563	GCA_003620955.1	CRESS virus sp.
2202563	GCA_003625055.1	CRESS virus sp.
2202563	GCA_003623015.1	CRESS virus sp.
2202563	GCA_003623235.1	CRESS virus sp.
2202563	GCA_003620975.1	CRESS virus sp.
2202563	GCA_003625075.1	CRESS virus sp.
2202563	GCA_003622555.1	CRESS virus sp.
2202563	GCA_003623035.1	CRESS virus sp.
2202563	GCA_003620995.1	CRESS virus sp.
2202563	GCA_003625095.1	CRESS virus sp.
2202563	GCA_003623055.1	CRESS virus sp.
2202563	GCA_003621535.1	CRESS virus sp.
2202563	GCA_003625115.1	CRESS virus sp.
2202563	GCA_003621435.1	CRESS virus sp.
2202563	GCA_003623075.1	CRESS virus sp.
2202563	GCA_003620515.1	CRESS virus sp.
2202563	GCA_003621035.1	CRESS virus sp.
2202563	GCA_003625135.1	CRESS virus sp.
2202563	GCA_003623095.1	CRESS virus sp.
2202563	GCA_003627175.1	CRESS virus sp.

2202563	GCA_003625155.1	CRESS virus sp.
2202563	GCA_003623115.1	CRESS virus sp.
2202563	GCA_003625175.1	CRESS virus sp.
2202563	GCA_003623135.1	CRESS virus sp.
2202563	GCA_003625195.1	CRESS virus sp.
2202563	GCA_003623155.1	CRESS virus sp.
2202563	GCA_003622235.1	CRESS virus sp.
2202563	GCA_003625215.1	CRESS virus sp.
2202563	GCA_003623175.1	CRESS virus sp.
2202563	GCA_003621135.1	CRESS virus sp.
2202563	GCA_003621215.1	CRESS virus sp.
2202563	GCA_003625235.1	CRESS virus sp.
2202563	GCA_003620875.1	CRESS virus sp.
2202563	GCA_003620535.1	CRESS virus sp.
2202563	GCA_003625255.1	CRESS virus sp.
2202563	GCA_003623215.1	CRESS virus sp.
2202563	GCA_003621175.1	CRESS virus sp.
2202563	GCA_003625275.1	CRESS virus sp.
2202563	GCA_003621195.1	CRESS virus sp.
2202563	GCA_003625295.1	CRESS virus sp.
2202563	GCA_003623255.1	CRESS virus sp.
2202563	GCA_003625315.1	CRESS virus sp.
2202563	GCA_003622595.1	CRESS virus sp.
2202563	GCA_003623275.1	CRESS virus sp.
2202563	GCA_003622255.1	CRESS virus sp.
2202563	GCA_003625335.1	CRESS virus sp.
2202563	GCA_003623295.1	CRESS virus sp.
2202563	GCA_003621575.1	CRESS virus sp.
2202563	GCA_003621235.1	CRESS virus sp.
2202563	GCA_003625355.1	CRESS virus sp.
2202563	GCA_003620555.1	CRESS virus sp.
2202563	GCA_003625375.1	CRESS virus sp.
2202563	GCA_003623335.1	CRESS virus sp.
2202563	GCA_003621295.1	CRESS virus sp.
2202563	GCA_003625395.1	CRESS virus sp.
2202563	GCA_003623355.1	CRESS virus sp.
2202563	GCA_003623375.1	CRESS virus sp.
2202563	GCA_003622955.1	CRESS virus sp.
2202563	GCA_003622615.1	CRESS virus sp.
2202563	GCA_003623395.1	CRESS virus sp.
2202563	GCA_003622275.1	CRESS virus sp.
2202563	GCA_003621375.1	CRESS virus sp.
2202563	GCA_003621255.1	CRESS virus sp.
2202563	GCA_003620575.1	CRESS virus sp.
2202563	GCA_003622675.1	CRESS virus sp.
2202563	GCA_003620235.1	CRESS virus sp.
2202563	GCA_003623455.1	CRESS virus sp.
2202563	GCA_003621415.1	CRESS virus sp.
2202563	GCA_003623495.1	CRESS virus sp.
2202563	GCA_003622635.1	CRESS virus sp.

2202563	GCA_003623515.1	CRESS virus sp.
2202563	GCA_003621475.1	CRESS virus sp.
2202563	GCA_003623535.1	CRESS virus sp.
2202563	GCA_003621275.1	CRESS virus sp.
2202563	GCA_003623555.1	CRESS virus sp.
2202563	GCA_003620595.1	CRESS virus sp.
2202563	GCA_003621515.1	CRESS virus sp.
2202563	GCA_003623575.1	CRESS virus sp.
2202563	GCA_003619915.1	CRESS virus sp.
2202563	GCA_003623595.1	CRESS virus sp.
2202563	GCA_003621555.1	CRESS virus sp.
2202563	GCA_003623615.1	CRESS virus sp.
2202563	GCA_003622655.1	CRESS virus sp.
2202563	GCA_003623635.1	CRESS virus sp.
2202563	GCA_003622315.1	CRESS virus sp.
2202563	GCA_003621595.1	CRESS virus sp.
2202563	GCA_003623655.1	CRESS virus sp.
2202563	GCA_003621635.1	CRESS virus sp.
2202563	GCA_003621615.1	CRESS virus sp.
2202563	GCA_003623675.1	CRESS virus sp.
2202563	GCA_003620615.1	CRESS virus sp.
2202563	GCA_003620275.1	CRESS virus sp.
2202563	GCA_003623695.1	CRESS virus sp.
2202563	GCA_003619935.1	CRESS virus sp.
2202563	GCA_003623715.1	CRESS virus sp.
2202563	GCA_003621675.1	CRESS virus sp.
2202563	GCA_003623735.1	CRESS virus sp.
2202563	GCA_003621695.1	CRESS virus sp.
2202563	GCA_003623755.1	CRESS virus sp.
2202563	GCA_003621715.1	CRESS virus sp.
2202563	GCA_003623775.1	CRESS virus sp.
2202563	GCA_003621655.1	CRESS virus sp.
2202563	GCA_003621735.1	CRESS virus sp.
2202563	GCA_003621315.1	CRESS virus sp.
2202563	GCA_003623795.1	CRESS virus sp.
2202563	GCA_003621755.1	CRESS virus sp.
2202563	GCA_003620295.1	CRESS virus sp.
2202563	GCA_003623815.1	CRESS virus sp.
2202563	GCA_003623835.1	CRESS virus sp.
2202563	GCA_003623855.1	CRESS virus sp.
2202563	GCA_003622075.1	CRESS virus sp.
2202563	GCA_003622695.1	CRESS virus sp.
2202563	GCA_003623875.1	CRESS virus sp.
2202563	GCA_003622355.1	CRESS virus sp.
2202563	GCA_003623895.1	CRESS virus sp.
2202563	GCA_003621855.1	CRESS virus sp.
2202563	GCA_003621335.1	CRESS virus sp.
2202563	GCA_003623915.1	CRESS virus sp.
2202563	GCA_003620655.1	CRESS virus sp.
2202563	GCA_003621875.1	CRESS virus sp.

2202563	GCA_003620315.1	CRESS virus sp.
2202563	GCA_003619835.1	CRESS virus sp.
2202563	GCA_003623935.1	CRESS virus sp.
2202563	GCA_003621895.1	CRESS virus sp.
2202563	GCA_003619855.1	CRESS virus sp.
2202563	GCA_003623955.1	CRESS virus sp.
2202563	GCA_003621915.1	CRESS virus sp.
2202563	GCA_003619875.1	CRESS virus sp.
2202563	GCA_003623975.1	CRESS virus sp.
2202563	GCA_003621935.1	CRESS virus sp.
2202563	GCA_003619895.1	CRESS virus sp.
2202563	GCA_003622715.1	CRESS virus sp.
2202563	GCA_003623995.1	CRESS virus sp.
2202563	GCA_003621955.1	CRESS virus sp.
2202563	GCA_003622035.1	CRESS virus sp.
2202563	GCA_003624015.1	CRESS virus sp.
2202563	GCA_003621975.1	CRESS virus sp.
2202563	GCA_003621355.1	CRESS virus sp.
2202563	GCA_003621015.1	CRESS virus sp.
2202563	GCA_003624035.1	CRESS virus sp.
2202563	GCA_003620675.1	CRESS virus sp.
2202563	GCA_003621995.1	CRESS virus sp.
2202563	GCA_003619955.1	CRESS virus sp.
2202563	GCA_003624055.1	CRESS virus sp.
2202563	GCA_003622015.1	CRESS virus sp.
2202563	GCA_003619975.1	CRESS virus sp.
2202563	GCA_003624075.1	CRESS virus sp.
2202563	GCA_003619995.1	CRESS virus sp.
2202563	GCA_003624095.1	CRESS virus sp.
2202563	GCA_003623415.1	CRESS virus sp.
2202563	GCA_003622055.1	CRESS virus sp.
2202563	GCA_003620015.1	CRESS virus sp.
2202563	GCA_003622735.1	CRESS virus sp.
2202563	GCA_003624115.1	CRESS virus sp.
2202563	GCA_003620035.1	CRESS virus sp.
2202563	GCA_003624135.1	CRESS virus sp.
2202563	GCA_003622095.1	CRESS virus sp.
2202563	GCA_003624155.1	CRESS virus sp.
2202563	GCA_003622115.1	CRESS virus sp.
2202563	GCA_003624175.1	CRESS virus sp.
2202563	GCA_003622135.1	CRESS virus sp.
2202563	GCA_003624195.1	CRESS virus sp.
2202563	GCA_003624215.1	CRESS virus sp.
2202563	GCA_003623435.1	CRESS virus sp.
2202563	GCA_003622755.1	CRESS virus sp.
2202563	GCA_003624235.1	CRESS virus sp.
2202563	GCA_003622415.1	CRESS virus sp.
2202563	GCA_003622195.1	CRESS virus sp.
2202563	GCA_003620155.1	CRESS virus sp.
2202563	GCA_003624255.1	CRESS virus sp.

2202563	GCA_003622215.1	CRESS virus sp.
2202563	GCA_003621395.1	CRESS virus sp.
2202563	GCA_003620175.1	CRESS virus sp.
2202563	GCA_003621055.1	CRESS virus sp.
2202563	GCA_003624275.1	CRESS virus sp.
2202563	GCA_003620375.1	CRESS virus sp.
2202563	GCA_003620195.1	CRESS virus sp.
2202563	GCA_003624295.1	CRESS virus sp.
2202563	GCA_003620215.1	CRESS virus sp.
2202563	GCA_003624315.1	CRESS virus sp.
2202563	GCA_003624335.1	CRESS virus sp.
2202563	GCA_003622295.1	CRESS virus sp.
2202563	GCA_003620255.1	CRESS virus sp.
2202563	GCA_003622775.1	CRESS virus sp.
2202563	GCA_003624355.1	CRESS virus sp.
2202563	GCA_003622435.1	CRESS virus sp.
2202563	GCA_003624375.1	CRESS virus sp.
2202563	GCA_003622335.1	CRESS virus sp.
2202563	GCA_003621075.1	CRESS virus sp.
2202563	GCA_003624395.1	CRESS virus sp.
2202563	GCA_003624415.1	CRESS virus sp.
2202563	GCA_003620055.1	CRESS virus sp.
2202563	GCA_003622375.1	CRESS virus sp.
2202563	GCA_003620335.1	CRESS virus sp.
2202563	GCA_003624435.1	CRESS virus sp.
2202563	GCA_003622395.1	CRESS virus sp.
2202563	GCA_003620355.1	CRESS virus sp.
2202563	GCA_003624455.1	CRESS virus sp.
2202563	GCA_003623475.1	CRESS virus sp.
2202563	GCA_003622795.1	CRESS virus sp.
2202563	GCA_003624475.1	CRESS virus sp.
2202563	GCA_003622455.1	CRESS virus sp.
2202563	GCA_003620395.1	CRESS virus sp.
2202563	GCA_003624495.1	CRESS virus sp.
2202563	GCA_003621775.1	CRESS virus sp.
2202563	GCA_003620415.1	CRESS virus sp.
2202563	GCA_003624515.1	CRESS virus sp.
2202563	GCA_003620755.1	CRESS virus sp.
2202563	GCA_003622475.1	CRESS virus sp.
2202563	GCA_003620435.1	CRESS virus sp.
2202563	GCA_003624535.1	CRESS virus sp.
2202563	GCA_003620075.1	CRESS virus sp.
2202563	GCA_003622495.1	CRESS virus sp.
2202563	GCA_003620455.1	CRESS virus sp.
2202563	GCA_003624555.1	CRESS virus sp.
2202563	GCA_003622515.1	CRESS virus sp.
2202563	GCA_003624575.1	CRESS virus sp.
2202563	GCA_003622535.1	CRESS virus sp.
2202563	GCA_003620495.1	CRESS virus sp.
2202563	GCA_003622815.1	CRESS virus sp.

2202563	GCA_003624595.1	CRESS virus sp.
2202563	GCA_003624615.1	CRESS virus sp.
2202563	GCA_003621795.1	CRESS virus sp.
2202563	GCA_003622575.1	CRESS virus sp.
2202563	GCA_003621455.1	CRESS virus sp.
2202563	GCA_003621115.1	CRESS virus sp.
2202563	GCA_003624635.1	CRESS virus sp.
2202563	GCA_003620775.1	CRESS virus sp.
2202563	GCA_003624655.1	CRESS virus sp.
2202563	GCA_003620095.1	CRESS virus sp.
2202563	GCA_003624875.1	CRESS virus sp.
2202563	GCA_003624675.1	CRESS virus sp.
2202563	GCA_003624695.1	CRESS virus sp.
2202563	GCA_003622835.1	CRESS virus sp.
2202563	GCA_003624715.1	CRESS virus sp.
2202563	GCA_003622155.1	CRESS virus sp.
2202563	GCA_003620635.1	CRESS virus sp.
2202563	GCA_003624735.1	CRESS virus sp.
2202563	GCA_003621815.1	CRESS virus sp.
2202563	GCA_003624755.1	CRESS virus sp.
2202563	GCA_003620795.1	CRESS virus sp.
2202563	GCA_003624775.1	CRESS virus sp.
2202563	GCA_003620115.1	CRESS virus sp.
2202563	GCA_003620695.1	CRESS virus sp.
2202563	GCA_003624795.1	CRESS virus sp.
2202563	GCA_003620715.1	CRESS virus sp.
2202563	GCA_003624815.1	CRESS virus sp.
2202563	GCA_003623195.1	CRESS virus sp.
2202563	GCA_003620735.1	CRESS virus sp.
2202563	GCA_003622855.1	CRESS virus sp.
2202563	GCA_003624835.1	CRESS virus sp.
2202563	GCA_003622175.1	CRESS virus sp.
2202563	GCA_003624855.1	CRESS virus sp.
2202563	GCA_003621835.1	CRESS virus sp.
2202563	GCA_003621495.1	CRESS virus sp.
2202563	GCA_003620815.1	CRESS virus sp.
2202563	GCA_003620475.1	CRESS virus sp.
2202563	GCA_003624895.1	CRESS virus sp.
2202563	GCA_003620135.1	CRESS virus sp.
2202563	GCA_003621155.1	CRESS virus sp.
2202563	GCA_003624915.1	CRESS virus sp.
2202563	GCA_004290695.1	CRESS virus sp.
2202563	GCA_004290715.1	CRESS virus sp.
2202563	GCA_004290795.1	CRESS virus sp.
2202563	GCA_004290815.1	CRESS virus sp.
2202563	GCA_004290835.1	CRESS virus sp.
2202563	GCA_004290915.1	CRESS virus sp.
2202563	GCA_004290935.1	CRESS virus sp.
2202563	GCA_004290955.1	CRESS virus sp.
2202563	GCA_004290375.1	CRESS virus sp.

2202563	GCA_004291015.1	CRESS virus sp.
2202563	GCA_004291035.1	CRESS virus sp.
2202563	GCA_004291055.1	CRESS virus sp.
2202563	GCA_004290775.1	CRESS virus sp.
2202563	GCA_004290115.1	CRESS virus sp.
2202563	GCA_004290135.1	CRESS virus sp.
2202563	GCA_004289755.1	CRESS virus sp.
2202563	GCA_004289795.1	CRESS virus sp.
2202563	GCA_004289815.1	CRESS virus sp.
2202563	GCA_004289835.1	CRESS virus sp.
2202563	GCA_004290195.1	CRESS virus sp.
2202563	GCA_004289915.1	CRESS virus sp.
2202563	GCA_004289935.1	CRESS virus sp.
2202563	GCA_004289955.1	CRESS virus sp.
2202563	GCA_004289995.1	CRESS virus sp.
2202563	GCA_004290015.1	CRESS virus sp.
2202563	GCA_004290035.1	CRESS virus sp.
2202563	GCA_004290055.1	CRESS virus sp.
2202563	GCA_004290075.1	CRESS virus sp.
2202563	GCA_004290095.1	CRESS virus sp.
2202563	GCA_004290155.1	CRESS virus sp.
2202563	GCA_004290215.1	CRESS virus sp.
2202563	GCA_004290275.1	CRESS virus sp.
2202563	GCA_004290295.1	CRESS virus sp.
2202563	GCA_004290355.1	CRESS virus sp.
2202563	GCA_004290515.1	CRESS virus sp.
2202563	GCA_004290535.1	CRESS virus sp.
2202563	GCA_004290575.1	CRESS virus sp.
2202563	GCA_004290595.1	CRESS virus sp.
2202563	GCA_004290635.1	CRESS virus sp.
2202563	GCA_004290655.1	CRESS virus sp.
1605972	GCA_000892215.1	Cricetid gammaherpesvirus 2
2293276	GCA_003846925.1	Cricket associated circular virus 1
12136	GCA_000853145.1	Cricket paralysis virus
1980519	GCA_000854165.1	Crimean-Congo hemorrhagic fever orthonairovirus
1323528	GCA_002868195.1	Crimson clover cryptic virus 2
1541211	GCA_000926275.1	Cripavirus NB-1/2011/HUN
1176422	GCA_000897435.1	Croceibacter phage P2559S
1327037	GCA_000915675.1	Croceibacter phage P2559Y
1104917	GCA_000900055.1	Crocuta crocuta papillomavirus 1
2169853	GCA_000925975.1	Crohivirus A
2169854	GCA_002008575.1	Crohivirus B
1816484	GCA_001678435.1	Cronartium ribicola mitovirus 1
1816485	GCA_001678275.1	Cronartium ribicola mitovirus 2
1816486	GCA_001678355.1	Cronartium ribicola mitovirus 3
1816487	GCA_001678315.1	Cronartium ribicola mitovirus 4
1816488	GCA_001678395.1	Cronartium ribicola mitovirus 5
1162295	GCA_000894715.1	Cronobacter phage CR3
1195085	GCA_000910235.1	Cronobacter phage CR5
1327934	GCA_000923635.1	Cronobacter phage CR8

1162290	GCA_000920235.1	Cronobacter phage CR9
2496544	GCA_003668515.1	Cronobacter phage CS01
1410331	GCA_000915495.1	Cronobacter phage Dev2
1712539	GCA_001550485.1	Cronobacter phage Dev-CD-23823
984175	GCA_000904915.1	Cronobacter phage ENT39118
984186	GCA_000903875.1	Cronobacter phage ENT47670
1073754	GCA_002601465.1	Cronobacter phage ES2
947842	GCA_002630925.1	Cronobacter phage ESSI-2
2200756	GCA_004319625.1	Cronobacter phage GW1
1684115	GCA_001470535.1	Cronobacter phage PBES 02
1892569	GCA_900096485.1	Cronobacter phage Pet-CM3-4
1168280	GCA_000898515.1	Cronobacter phage phiES15
1327935	GCA_001503575.1	Cronobacter phage S13
1141138	GCA_000901535.1	Cronobacter phage vB_CsaM_GAP161
1141135	GCA_000900455.1	Cronobacter phage vB_CsaM_GAP31
1141136	GCA_000898015.1	Cronobacter phage vB_CsaM_GAP32
1885242	GCA_002612105.1	Cronobacter phage vB_CsaM_leB
1873954	GCA_002611805.1	Cronobacter phage vB_CsaM_leE
1885245	GCA_002612125.1	Cronobacter phage vB_CsaM_leN
1141137	GCA_000899595.1	Cronobacter phage vB_CsaP_GAP52
1498011	GCA_002604665.1	Cronobacter phage vB_CsaP_Ss1
1264737	GCA_000903995.1	Cronobacter phage vB_CskP_GAP227
1073767	GCA_000901875.1	Cronobacter virus Esp2949-1
327839	GCA_002988015.1	Croton yellow vein betasatellite
713089	GCA_000888115.1	Croton yellow vein mosaic alphasatellite
411248	GCA_000869565.1	Croton yellow vein mosaic betasatellite
207697	GCA_000857305.1	Croton yellow vein mosaic virus
207697	GCA_002986775.1	Croton yellow vein mosaic virus
671095	GCA_000889255.1	Croton yellow vein virus
40281	GCA_000849145.1	Cryphonectria hypovirus 1
40268	GCA_000851185.1	Cryphonectria hypovirus 2-NB58
106962	GCA_000850125.1	Cryphonectria hypovirus 3
245101	GCA_000855565.1	Cryphonectria hypovirus 4
399394	GCA_002867325.1	Cryphonectria nitschkei chrysovirus 1
1329781	GCA_000906455.1	Cryphonectria parasitica bipartite mycovirus 1
186769	GCA_000852365.1	Cryphonectria parasitica mitovirus 1-NB631
230407	GCA_000879395.1	Cryphonectria parasitica mycoreovirus-1 (9B21)
35254	GCA_000841505.1	Cryptophlebia leucotreta granulovirus
675060	GCA_002868475.1	Cryptosporidium parvum virus 1
1778570	GCA_001551325.1	Ctenophore-associated circular genome 1
1778572	GCA_001550985.1	Ctenophore-associated circular genome 3
1778573	GCA_001550605.1	Ctenophore-associated circular genome 4
1778558	GCA_001551145.1	Ctenophore-associated circular virus 1
1778559	GCA_001551485.1	Ctenophore-associated circular virus 2
1778560	GCA_001551005.1	Ctenophore-associated circular virus 3
1778561	GCA_001550625.1	Ctenophore-associated circular virus 4
1188819	GCA_000909475.1	Cuban alphasatellite 1
220618	GCA_000852545.1	Cucumber Bulgarian virus
146499	GCA_000849365.1	Cucumber fruit mottle mosaic virus
12235	GCA_000849225.1	Cucumber green mottle mosaic virus

165432	GCA_000868905.1	Cucumber leaf spot virus
12305	GCA_000864745.1	Cucumber mosaic virus (cucumber mosaic cucumovirus)
12305	GCA_003110565.1	Cucumber mosaic virus (cucumber mosaic cucumovirus)
12305	GCA_003110585.1	Cucumber mosaic virus (cucumber mosaic cucumovirus)
12305	GCA_003110605.1	Cucumber mosaic virus (cucumber mosaic cucumovirus)
12305	GCA_003110645.1	Cucumber mosaic virus (cucumber mosaic cucumovirus)
12305	GCA_003110665.1	Cucumber mosaic virus (cucumber mosaic cucumovirus)
12305	GCA_003110685.1	Cucumber mosaic virus (cucumber mosaic cucumovirus)
12305	GCA_003110705.1	Cucumber mosaic virus (cucumber mosaic cucumovirus)
12305	GCA_003111265.1	Cucumber mosaic virus (cucumber mosaic cucumovirus)
12305	GCA_003110725.1	Cucumber mosaic virus (cucumber mosaic cucumovirus)
12305	GCA_003110745.1	Cucumber mosaic virus (cucumber mosaic cucumovirus)
12305	GCA_003110765.1	Cucumber mosaic virus (cucumber mosaic cucumovirus)
12305	GCA_003110785.1	Cucumber mosaic virus (cucumber mosaic cucumovirus)
12305	GCA_003110805.1	Cucumber mosaic virus (cucumber mosaic cucumovirus)
12305	GCA_003110625.1	Cucumber mosaic virus (cucumber mosaic cucumovirus)
12305	GCA_003110845.1	Cucumber mosaic virus (cucumber mosaic cucumovirus)
12305	GCA_003110265.1	Cucumber mosaic virus (cucumber mosaic cucumovirus)
12305	GCA_003110865.1	Cucumber mosaic virus (cucumber mosaic cucumovirus)
12305	GCA_003110885.1	Cucumber mosaic virus (cucumber mosaic cucumovirus)
12305	GCA_003110905.1	Cucumber mosaic virus (cucumber mosaic cucumovirus)
12305	GCA_003110925.1	Cucumber mosaic virus (cucumber mosaic cucumovirus)
12305	GCA_003110945.1	Cucumber mosaic virus (cucumber mosaic cucumovirus)
12305	GCA_003110965.1	Cucumber mosaic virus (cucumber mosaic cucumovirus)
12305	GCA_003110985.1	Cucumber mosaic virus (cucumber mosaic cucumovirus)
12305	GCA_003111025.1	Cucumber mosaic virus (cucumber mosaic cucumovirus)
12305	GCA_003111045.1	Cucumber mosaic virus (cucumber mosaic cucumovirus)
12305	GCA_003111065.1	Cucumber mosaic virus (cucumber mosaic cucumovirus)
12305	GCA_003111085.1	Cucumber mosaic virus (cucumber mosaic cucumovirus)
12305	GCA_003111105.1	Cucumber mosaic virus (cucumber mosaic cucumovirus)
12305	GCA_003111125.1	Cucumber mosaic virus (cucumber mosaic cucumovirus)
12305	GCA_003111145.1	Cucumber mosaic virus (cucumber mosaic cucumovirus)
12305	GCA_003111165.1	Cucumber mosaic virus (cucumber mosaic cucumovirus)
12305	GCA_003111185.1	Cucumber mosaic virus (cucumber mosaic cucumovirus)
12305	GCA_003111005.1	Cucumber mosaic virus (cucumber mosaic cucumovirus)
12305	GCA_003110325.1	Cucumber mosaic virus (cucumber mosaic cucumovirus)
12305	GCA_003111225.1	Cucumber mosaic virus (cucumber mosaic cucumovirus)
12305	GCA_003111245.1	Cucumber mosaic virus (cucumber mosaic cucumovirus)
12305	GCA_003111285.1	Cucumber mosaic virus (cucumber mosaic cucumovirus)
12305	GCA_003111305.1	Cucumber mosaic virus (cucumber mosaic cucumovirus)
12305	GCA_003111325.1	Cucumber mosaic virus (cucumber mosaic cucumovirus)
12305	GCA_003111345.1	Cucumber mosaic virus (cucumber mosaic cucumovirus)
12305	GCA_003111365.1	Cucumber mosaic virus (cucumber mosaic cucumovirus)
12305	GCA_003111385.1	Cucumber mosaic virus (cucumber mosaic cucumovirus)
12305	GCA_003111405.1	Cucumber mosaic virus (cucumber mosaic cucumovirus)
12305	GCA_003111425.1	Cucumber mosaic virus (cucumber mosaic cucumovirus)
12305	GCA_003111445.1	Cucumber mosaic virus (cucumber mosaic cucumovirus)
12305	GCA_003111465.1	Cucumber mosaic virus (cucumber mosaic cucumovirus)
12305	GCA_003111485.1	Cucumber mosaic virus (cucumber mosaic cucumovirus)
12305	GCA_003111505.1	Cucumber mosaic virus (cucumber mosaic cucumovirus)

12305	GCA_003109465.1	Cucumber mosaic virus (cucumber mosaic cucumovirus)
12305	GCA_003111525.1	Cucumber mosaic virus (cucumber mosaic cucumovirus)
12305	GCA_003109485.1	Cucumber mosaic virus (cucumber mosaic cucumovirus)
12305	GCA_003111545.1	Cucumber mosaic virus (cucumber mosaic cucumovirus)
12305	GCA_003109505.1	Cucumber mosaic virus (cucumber mosaic cucumovirus)
12305	GCA_003111565.1	Cucumber mosaic virus (cucumber mosaic cucumovirus)
12305	GCA_003109525.1	Cucumber mosaic virus (cucumber mosaic cucumovirus)
12305	GCA_003111585.1	Cucumber mosaic virus (cucumber mosaic cucumovirus)
12305	GCA_003109545.1	Cucumber mosaic virus (cucumber mosaic cucumovirus)
12305	GCA_003109565.1	Cucumber mosaic virus (cucumber mosaic cucumovirus)
12305	GCA_003109585.1	Cucumber mosaic virus (cucumber mosaic cucumovirus)
12305	GCA_003109605.1	Cucumber mosaic virus (cucumber mosaic cucumovirus)
12305	GCA_003109625.1	Cucumber mosaic virus (cucumber mosaic cucumovirus)
12305	GCA_003109645.1	Cucumber mosaic virus (cucumber mosaic cucumovirus)
12305	GCA_003109665.1	Cucumber mosaic virus (cucumber mosaic cucumovirus)
12305	GCA_003109725.1	Cucumber mosaic virus (cucumber mosaic cucumovirus)
12305	GCA_003109685.1	Cucumber mosaic virus (cucumber mosaic cucumovirus)
12305	GCA_003109705.1	Cucumber mosaic virus (cucumber mosaic cucumovirus)
12305	GCA_003109765.1	Cucumber mosaic virus (cucumber mosaic cucumovirus)
12305	GCA_003109785.1	Cucumber mosaic virus (cucumber mosaic cucumovirus)
12305	GCA_003109745.1	Cucumber mosaic virus (cucumber mosaic cucumovirus)
12305	GCA_003109805.1	Cucumber mosaic virus (cucumber mosaic cucumovirus)
12305	GCA_003109825.1	Cucumber mosaic virus (cucumber mosaic cucumovirus)
12305	GCA_003109845.1	Cucumber mosaic virus (cucumber mosaic cucumovirus)
12305	GCA_003109865.1	Cucumber mosaic virus (cucumber mosaic cucumovirus)
12305	GCA_003109885.1	Cucumber mosaic virus (cucumber mosaic cucumovirus)
12305	GCA_003109905.1	Cucumber mosaic virus (cucumber mosaic cucumovirus)
12305	GCA_003109925.1	Cucumber mosaic virus (cucumber mosaic cucumovirus)
12305	GCA_003109945.1	Cucumber mosaic virus (cucumber mosaic cucumovirus)
12305	GCA_003109965.1	Cucumber mosaic virus (cucumber mosaic cucumovirus)
12305	GCA_003109985.1	Cucumber mosaic virus (cucumber mosaic cucumovirus)
12305	GCA_003110005.1	Cucumber mosaic virus (cucumber mosaic cucumovirus)
12305	GCA_003110025.1	Cucumber mosaic virus (cucumber mosaic cucumovirus)
12305	GCA_003110825.1	Cucumber mosaic virus (cucumber mosaic cucumovirus)
12305	GCA_003110045.1	Cucumber mosaic virus (cucumber mosaic cucumovirus)
12305	GCA_003110065.1	Cucumber mosaic virus (cucumber mosaic cucumovirus)
12305	GCA_003110085.1	Cucumber mosaic virus (cucumber mosaic cucumovirus)
12305	GCA_003110105.1	Cucumber mosaic virus (cucumber mosaic cucumovirus)
12305	GCA_003110125.1	Cucumber mosaic virus (cucumber mosaic cucumovirus)
12305	GCA_003110145.1	Cucumber mosaic virus (cucumber mosaic cucumovirus)
12305	GCA_003110165.1	Cucumber mosaic virus (cucumber mosaic cucumovirus)
12305	GCA_003110205.1	Cucumber mosaic virus (cucumber mosaic cucumovirus)
12305	GCA_003110225.1	Cucumber mosaic virus (cucumber mosaic cucumovirus)
12305	GCA_003110245.1	Cucumber mosaic virus (cucumber mosaic cucumovirus)
12305	GCA_003110285.1	Cucumber mosaic virus (cucumber mosaic cucumovirus)
12305	GCA_003110305.1	Cucumber mosaic virus (cucumber mosaic cucumovirus)
12305	GCA_003110345.1	Cucumber mosaic virus (cucumber mosaic cucumovirus)
12305	GCA_003111205.1	Cucumber mosaic virus (cucumber mosaic cucumovirus)
12305	GCA_003110365.1	Cucumber mosaic virus (cucumber mosaic cucumovirus)
12305	GCA_003110185.1	Cucumber mosaic virus (cucumber mosaic cucumovirus)

12305	GCA_003110385.1	Cucumber mosaic virus (cucumber mosaic cucumovirus)
12305	GCA_003110405.1	Cucumber mosaic virus (cucumber mosaic cucumovirus)
12305	GCA_003110425.1	Cucumber mosaic virus (cucumber mosaic cucumovirus)
12305	GCA_003110465.1	Cucumber mosaic virus (cucumber mosaic cucumovirus)
12305	GCA_003110485.1	Cucumber mosaic virus (cucumber mosaic cucumovirus)
12305	GCA_003110525.1	Cucumber mosaic virus (cucumber mosaic cucumovirus)
12305	GCA_003127745.1	Cucumber mosaic virus (cucumber mosaic cucumovirus)
12305	GCA_003127785.1	Cucumber mosaic virus (cucumber mosaic cucumovirus)
12305	GCA_003128425.1	Cucumber mosaic virus (cucumber mosaic cucumovirus)
12305	GCA_003128085.1	Cucumber mosaic virus (cucumber mosaic cucumovirus)
12305	GCA_003127805.1	Cucumber mosaic virus (cucumber mosaic cucumovirus)
12305	GCA_003127825.1	Cucumber mosaic virus (cucumber mosaic cucumovirus)
12305	GCA_003127845.1	Cucumber mosaic virus (cucumber mosaic cucumovirus)
12305	GCA_003127865.1	Cucumber mosaic virus (cucumber mosaic cucumovirus)
12305	GCA_003127885.1	Cucumber mosaic virus (cucumber mosaic cucumovirus)
12305	GCA_003127905.1	Cucumber mosaic virus (cucumber mosaic cucumovirus)
12305	GCA_003128445.1	Cucumber mosaic virus (cucumber mosaic cucumovirus)
12305	GCA_003128105.1	Cucumber mosaic virus (cucumber mosaic cucumovirus)
12305	GCA_003127925.1	Cucumber mosaic virus (cucumber mosaic cucumovirus)
12305	GCA_003127765.1	Cucumber mosaic virus (cucumber mosaic cucumovirus)
12305	GCA_003127085.1	Cucumber mosaic virus (cucumber mosaic cucumovirus)
12305	GCA_003127945.1	Cucumber mosaic virus (cucumber mosaic cucumovirus)
12305	GCA_003127965.1	Cucumber mosaic virus (cucumber mosaic cucumovirus)
12305	GCA_003127985.1	Cucumber mosaic virus (cucumber mosaic cucumovirus)
12305	GCA_003128005.1	Cucumber mosaic virus (cucumber mosaic cucumovirus)
12305	GCA_003128025.1	Cucumber mosaic virus (cucumber mosaic cucumovirus)
12305	GCA_003128125.1	Cucumber mosaic virus (cucumber mosaic cucumovirus)
12305	GCA_003128485.1	Cucumber mosaic virus (cucumber mosaic cucumovirus)
12305	GCA_003128145.1	Cucumber mosaic virus (cucumber mosaic cucumovirus)
12305	GCA_003128165.1	Cucumber mosaic virus (cucumber mosaic cucumovirus)
12305	GCA_003128185.1	Cucumber mosaic virus (cucumber mosaic cucumovirus)
12305	GCA_003128205.1	Cucumber mosaic virus (cucumber mosaic cucumovirus)
12305	GCA_003128225.1	Cucumber mosaic virus (cucumber mosaic cucumovirus)
12305	GCA_003128245.1	Cucumber mosaic virus (cucumber mosaic cucumovirus)
12305	GCA_003128265.1	Cucumber mosaic virus (cucumber mosaic cucumovirus)
12305	GCA_003128505.1	Cucumber mosaic virus (cucumber mosaic cucumovirus)
12305	GCA_003127145.1	Cucumber mosaic virus (cucumber mosaic cucumovirus)
12305	GCA_003128305.1	Cucumber mosaic virus (cucumber mosaic cucumovirus)
12305	GCA_003128345.1	Cucumber mosaic virus (cucumber mosaic cucumovirus)
12305	GCA_003128365.1	Cucumber mosaic virus (cucumber mosaic cucumovirus)
12305	GCA_003128525.1	Cucumber mosaic virus (cucumber mosaic cucumovirus)
12305	GCA_003128405.1	Cucumber mosaic virus (cucumber mosaic cucumovirus)
12305	GCA_003128465.1	Cucumber mosaic virus (cucumber mosaic cucumovirus)
12305	GCA_003128545.1	Cucumber mosaic virus (cucumber mosaic cucumovirus)
12305	GCA_003128565.1	Cucumber mosaic virus (cucumber mosaic cucumovirus)
12305	GCA_003128685.1	Cucumber mosaic virus (cucumber mosaic cucumovirus)
12305	GCA_003126645.1	Cucumber mosaic virus (cucumber mosaic cucumovirus)
12305	GCA_003126665.1	Cucumber mosaic virus (cucumber mosaic cucumovirus)
12305	GCA_003126685.1	Cucumber mosaic virus (cucumber mosaic cucumovirus)
12305	GCA_003128585.1	Cucumber mosaic virus (cucumber mosaic cucumovirus)

12305	GCA_003126705.1	Cucumber mosaic virus (cucumber mosaic cucumovirus)
12305	GCA_003128765.1	Cucumber mosaic virus (cucumber mosaic cucumovirus)
12305	GCA_003126725.1	Cucumber mosaic virus (cucumber mosaic cucumovirus)
12305	GCA_003128785.1	Cucumber mosaic virus (cucumber mosaic cucumovirus)
12305	GCA_003126745.1	Cucumber mosaic virus (cucumber mosaic cucumovirus)
12305	GCA_003128805.1	Cucumber mosaic virus (cucumber mosaic cucumovirus)
12305	GCA_003126765.1	Cucumber mosaic virus (cucumber mosaic cucumovirus)
12305	GCA_003128825.1	Cucumber mosaic virus (cucumber mosaic cucumovirus)
12305	GCA_003126785.1	Cucumber mosaic virus (cucumber mosaic cucumovirus)
12305	GCA_003128845.1	Cucumber mosaic virus (cucumber mosaic cucumovirus)
12305	GCA_003126805.1	Cucumber mosaic virus (cucumber mosaic cucumovirus)
12305	GCA_003128865.1	Cucumber mosaic virus (cucumber mosaic cucumovirus)
12305	GCA_003128605.1	Cucumber mosaic virus (cucumber mosaic cucumovirus)
12305	GCA_003126825.1	Cucumber mosaic virus (cucumber mosaic cucumovirus)
12305	GCA_003128885.1	Cucumber mosaic virus (cucumber mosaic cucumovirus)
12305	GCA_003126845.1	Cucumber mosaic virus (cucumber mosaic cucumovirus)
12305	GCA_003128905.1	Cucumber mosaic virus (cucumber mosaic cucumovirus)
12305	GCA_003126905.1	Cucumber mosaic virus (cucumber mosaic cucumovirus)
12305	GCA_003126865.1	Cucumber mosaic virus (cucumber mosaic cucumovirus)
12305	GCA_003128925.1	Cucumber mosaic virus (cucumber mosaic cucumovirus)
12305	GCA_003126885.1	Cucumber mosaic virus (cucumber mosaic cucumovirus)
12305	GCA_003128945.1	Cucumber mosaic virus (cucumber mosaic cucumovirus)
12305	GCA_003128965.1	Cucumber mosaic virus (cucumber mosaic cucumovirus)
12305	GCA_003126925.1	Cucumber mosaic virus (cucumber mosaic cucumovirus)
12305	GCA_003128985.1	Cucumber mosaic virus (cucumber mosaic cucumovirus)
12305	GCA_003128625.1	Cucumber mosaic virus (cucumber mosaic cucumovirus)
12305	GCA_003126945.1	Cucumber mosaic virus (cucumber mosaic cucumovirus)
12305	GCA_003128285.1	Cucumber mosaic virus (cucumber mosaic cucumovirus)
12305	GCA_003129005.1	Cucumber mosaic virus (cucumber mosaic cucumovirus)
12305	GCA_003126965.1	Cucumber mosaic virus (cucumber mosaic cucumovirus)
12305	GCA_003129025.1	Cucumber mosaic virus (cucumber mosaic cucumovirus)
12305	GCA_003126985.1	Cucumber mosaic virus (cucumber mosaic cucumovirus)
12305	GCA_003129045.1	Cucumber mosaic virus (cucumber mosaic cucumovirus)
12305	GCA_003129065.1	Cucumber mosaic virus (cucumber mosaic cucumovirus)
12305	GCA_003129085.1	Cucumber mosaic virus (cucumber mosaic cucumovirus)
12305	GCA_003127045.1	Cucumber mosaic virus (cucumber mosaic cucumovirus)
12305	GCA_003129105.1	Cucumber mosaic virus (cucumber mosaic cucumovirus)
12305	GCA_003128645.1	Cucumber mosaic virus (cucumber mosaic cucumovirus)
12305	GCA_003127065.1	Cucumber mosaic virus (cucumber mosaic cucumovirus)
12305	GCA_003129125.1	Cucumber mosaic virus (cucumber mosaic cucumovirus)
12305	GCA_003129145.1	Cucumber mosaic virus (cucumber mosaic cucumovirus)
12305	GCA_003127105.1	Cucumber mosaic virus (cucumber mosaic cucumovirus)
12305	GCA_003129165.1	Cucumber mosaic virus (cucumber mosaic cucumovirus)
12305	GCA_003127125.1	Cucumber mosaic virus (cucumber mosaic cucumovirus)
12305	GCA_003129185.1	Cucumber mosaic virus (cucumber mosaic cucumovirus)
12305	GCA_003129205.1	Cucumber mosaic virus (cucumber mosaic cucumovirus)
12305	GCA_003127165.1	Cucumber mosaic virus (cucumber mosaic cucumovirus)
12305	GCA_003129225.1	Cucumber mosaic virus (cucumber mosaic cucumovirus)
12305	GCA_003128665.1	Cucumber mosaic virus (cucumber mosaic cucumovirus)
12305	GCA_003127185.1	Cucumber mosaic virus (cucumber mosaic cucumovirus)

12305	GCA_003128325.1	Cucumber mosaic virus (cucumber mosaic cucumovirus)
12305	GCA_003129245.1	Cucumber mosaic virus (cucumber mosaic cucumovirus)
12305	GCA_003127205.1	Cucumber mosaic virus (cucumber mosaic cucumovirus)
12305	GCA_003127025.1	Cucumber mosaic virus (cucumber mosaic cucumovirus)
12305	GCA_003129265.1	Cucumber mosaic virus (cucumber mosaic cucumovirus)
12305	GCA_003127225.1	Cucumber mosaic virus (cucumber mosaic cucumovirus)
12305	GCA_003129285.1	Cucumber mosaic virus (cucumber mosaic cucumovirus)
12305	GCA_003127245.1	Cucumber mosaic virus (cucumber mosaic cucumovirus)
12305	GCA_003129305.1	Cucumber mosaic virus (cucumber mosaic cucumovirus)
12305	GCA_003127265.1	Cucumber mosaic virus (cucumber mosaic cucumovirus)
12305	GCA_003129325.1	Cucumber mosaic virus (cucumber mosaic cucumovirus)
12305	GCA_003127285.1	Cucumber mosaic virus (cucumber mosaic cucumovirus)
12305	GCA_003129345.1	Cucumber mosaic virus (cucumber mosaic cucumovirus)
12305	GCA_003127305.1	Cucumber mosaic virus (cucumber mosaic cucumovirus)
12305	GCA_003129365.1	Cucumber mosaic virus (cucumber mosaic cucumovirus)
12305	GCA_003127325.1	Cucumber mosaic virus (cucumber mosaic cucumovirus)
12305	GCA_003129385.1	Cucumber mosaic virus (cucumber mosaic cucumovirus)
12305	GCA_003127345.1	Cucumber mosaic virus (cucumber mosaic cucumovirus)
12305	GCA_003129405.1	Cucumber mosaic virus (cucumber mosaic cucumovirus)
12305	GCA_003127365.1	Cucumber mosaic virus (cucumber mosaic cucumovirus)
12305	GCA_003129425.1	Cucumber mosaic virus (cucumber mosaic cucumovirus)
12305	GCA_003127385.1	Cucumber mosaic virus (cucumber mosaic cucumovirus)
12305	GCA_003129445.1	Cucumber mosaic virus (cucumber mosaic cucumovirus)
12305	GCA_003127405.1	Cucumber mosaic virus (cucumber mosaic cucumovirus)
12305	GCA_003128705.1	Cucumber mosaic virus (cucumber mosaic cucumovirus)
12305	GCA_003127425.1	Cucumber mosaic virus (cucumber mosaic cucumovirus)
12305	GCA_003127445.1	Cucumber mosaic virus (cucumber mosaic cucumovirus)
12305	GCA_003127685.1	Cucumber mosaic virus (cucumber mosaic cucumovirus)
12305	GCA_003127005.1	Cucumber mosaic virus (cucumber mosaic cucumovirus)
12305	GCA_003127465.1	Cucumber mosaic virus (cucumber mosaic cucumovirus)
12305	GCA_003127485.1	Cucumber mosaic virus (cucumber mosaic cucumovirus)
12305	GCA_003127505.1	Cucumber mosaic virus (cucumber mosaic cucumovirus)
12305	GCA_003127525.1	Cucumber mosaic virus (cucumber mosaic cucumovirus)
12305	GCA_003128725.1	Cucumber mosaic virus (cucumber mosaic cucumovirus)
12305	GCA_003127545.1	Cucumber mosaic virus (cucumber mosaic cucumovirus)
12305	GCA_003128385.1	Cucumber mosaic virus (cucumber mosaic cucumovirus)
12305	GCA_003128045.1	Cucumber mosaic virus (cucumber mosaic cucumovirus)
12305	GCA_003127565.1	Cucumber mosaic virus (cucumber mosaic cucumovirus)
12305	GCA_003127705.1	Cucumber mosaic virus (cucumber mosaic cucumovirus)
12305	GCA_003127585.1	Cucumber mosaic virus (cucumber mosaic cucumovirus)
12305	GCA_003127605.1	Cucumber mosaic virus (cucumber mosaic cucumovirus)
12305	GCA_003127625.1	Cucumber mosaic virus (cucumber mosaic cucumovirus)
12305	GCA_003127645.1	Cucumber mosaic virus (cucumber mosaic cucumovirus)
12305	GCA_003128745.1	Cucumber mosaic virus (cucumber mosaic cucumovirus)
12305	GCA_003127665.1	Cucumber mosaic virus (cucumber mosaic cucumovirus)
12305	GCA_003128065.1	Cucumber mosaic virus (cucumber mosaic cucumovirus)
12305	GCA_003127725.1	Cucumber mosaic virus (cucumber mosaic cucumovirus)
255721	GCA_003110445.1	Cucumber mosaic virus (strain Mf) (cucumber mosaic cucu
367797	GCA_003110505.1	Cucumber mosaic virus (strain Pl-1) (cucumber mosaic cuc
367798	GCA_003110545.1	Cucumber mosaic virus (strain Ri-8) (cucumber mosaic cuc

12436	GCA_000847065.1	Cucumber mosaic virus satellite RNA
388038	GCA_000869625.1	Cucumber mottle virus
12143	GCA_000848185.1	Cucumber necrosis virus
137475	GCA_000858065.1	Cucumber vein yellowing virus
1776177	GCA_001550465.1	Cucumis melo alphaendornavirus
1611875	GCA_000943725.1	Cucurbit aphid borne yellows virus associated RNA
91753	GCA_000855065.1	Cucurbit aphid-borne yellows virus
558690	GCA_000898355.1	Cucurbit chlorotic yellows virus
134681	GCA_000837305.1	Cucurbit leaf crumple virus
1131416	GCA_002867105.1	Cucurbit mild mosaic virus
2005044	GCA_002210975.1	Cucurbit vein banding virus
1819678	GCA_001629965.1	Cucurbit yellow mosaic alphasatellite
51330	GCA_000851805.1	Cucurbit yellow stunting disorder virus
220814	GCA_000846845.1	Cucurbita yellow vein virus-associated DNA beta
2304505	GCA_004133765.1	Culex Bastrovirus-like virus
2304514	GCA_004133805.1	Culex circovirus-like virus
2304514	GCA_004133825.1	Culex circovirus-like virus
2304510	GCA_004133645.1	Culex Flavi-like virus
390844	GCA_000869605.1	Culex flavivirus
2304477	GCA_004133665.1	Culex Iflavi-like virus 1
2304480	GCA_004133705.1	Culex Iflavi-like virus 4
2304480	GCA_004133685.1	Culex Iflavi-like virus 4
2304480	GCA_004133725.1	Culex Iflavi-like virus 4
2010272	GCA_002210955.1	Culex mononega-like virus 2
2010272	GCA_003729275.1	Culex mononega-like virus 2
2010272	GCA_003729335.1	Culex mononega-like virus 2
2010272	GCA_003729415.1	Culex mononega-like virus 2
2010272	GCA_003729455.1	Culex mononega-like virus 2
2010272	GCA_003729395.1	Culex mononega-like virus 2
2010273	GCA_002210795.1	Culex negev-like virus 1
2010273	GCA_003729295.1	Culex negev-like virus 1
2010273	GCA_003764605.1	Culex negev-like virus 1
2010274	GCA_002210995.1	Culex negev-like virus 2
2010275	GCA_002210575.1	Culex negev-like virus 3
130556	GCA_000838125.1	Culex nigripalpus nucleopolyhedrovirus
1236047	GCA_000898695.1	Culex originated Tymoviridae-like virus
2079148	GCA_004134385.1	Culex pipiens associated Tunisia virus
185638	GCA_000883835.1	Culex pipiens densovirus
2010277	GCA_002210935.1	Culex rhabdo-like virus
2010277	GCA_003729355.1	Culex rhabdo-like virus
2010277	GCA_003729435.1	Culex rhabdo-like virus
2010277	GCA_003729895.1	Culex rhabdo-like virus
2304512	GCA_004133785.1	Culex Tetra-like virus
1244563	GCA_004130945.1	Culex theileri flavivirus
936308	GCA_000924695.1	Culex tritaeniorhynchus rhabdovirus
1870981	GCA_004129775.1	Culex tritaeniorhynchus totivirus
2304516	GCA_004133745.1	Culex-associated Tombus-like virus
1821222	GCA_001661835.1	Culiseta flavivirus
1457166	GCA_002814615.1	Cumuto virus
208899	GCA_000872485.1	Cupixi mammarenavirus

490110	GCA_000924675.1	Curionopolis virus
490110	GCA_002815535.1	Curionopolis virus
1476584	GCA_001503195.2	Currant latent virus
1770618	GCA_001567035.1	Currant virus A
1983783	GCA_003571845.1	Curvibacter phage P26059A
1983784	GCA_003571865.1	Curvibacter phage P26059B
421976	GCA_000880195.1	Curvularia thermal tolerance virus
1867125	GCA_003033315.1	Cutavirus
1016879	GCA_000892075.1	Cutthroat trout virus
335924	GCA_004028855.1	Cyanobacteria phage AS-1
444875	GCA_000896715.1	Cyanophage 9515-10a
889951	GCA_000904515.1	Cyanophage KBS-P-1A
889953	GCA_000906135.1	Cyanophage KBS-S-2A
889954	GCA_000905535.1	Cyanophage MED4-117
445693	GCA_000894275.1	Cyanophage NATL1A-7
445692	GCA_000895875.1	Cyanophage NATL2A-133
434346	GCA_000913755.1	Cyanophage PP
536444	GCA_000908275.1	Cyanophage P-RSM1
536446	GCA_002710185.1	Cyanophage P-RSM3
929832	GCA_000906155.1	Cyanophage P-RSM6
658401	GCA_000885095.1	Cyanophage PSS2
658401	GCA_003329025.1	Cyanophage PSS2
444876	GCA_000895235.1	Cyanophage P-SSP2
1589733	GCA_001470935.1	Cyanophage P-TIM40
1278402	GCA_002596065.1	Cyanophage S-RIM12
1278402	GCA_002596085.1	Cyanophage S-RIM12
1278402	GCA_002596105.1	Cyanophage S-RIM12
1278402	GCA_002596125.1	Cyanophage S-RIM12
1278402	GCA_002596145.1	Cyanophage S-RIM12
1278402	GCA_002596165.1	Cyanophage S-RIM12
1278402	GCA_002596185.1	Cyanophage S-RIM12
1278402	GCA_002596205.1	Cyanophage S-RIM12
1278402	GCA_002596225.1	Cyanophage S-RIM12
1278402	GCA_002596245.1	Cyanophage S-RIM12
1278402	GCA_002596265.1	Cyanophage S-RIM12
1278402	GCA_002596285.1	Cyanophage S-RIM12
1278402	GCA_002596305.1	Cyanophage S-RIM12
1278402	GCA_002596325.1	Cyanophage S-RIM12
1278402	GCA_002596345.1	Cyanophage S-RIM12
1278402	GCA_002596365.1	Cyanophage S-RIM12
1278402	GCA_002596385.1	Cyanophage S-RIM12
1278402	GCA_002596405.1	Cyanophage S-RIM12
1278402	GCA_002596425.1	Cyanophage S-RIM12
1278402	GCA_002596445.1	Cyanophage S-RIM12
1278402	GCA_002596465.1	Cyanophage S-RIM12
1278423	GCA_002598985.1	Cyanophage S-RIM14
1278423	GCA_002599005.1	Cyanophage S-RIM14
1278423	GCA_002599025.1	Cyanophage S-RIM14
1278423	GCA_002599105.1	Cyanophage S-RIM14
1278423	GCA_002599125.1	Cyanophage S-RIM14

1278423	GCA_002599045.1	Cyanophage S-RIM14
1278423	GCA_002599065.1	Cyanophage S-RIM14
1278423	GCA_002599085.1	Cyanophage S-RIM14
1278423	GCA_002598965.1	Cyanophage S-RIM14
1278479	GCA_001754165.1	Cyanophage S-RIM32
1278485	GCA_002599145.1	Cyanophage S-RIM44
1278485	GCA_002599165.1	Cyanophage S-RIM44
1278485	GCA_002599185.1	Cyanophage S-RIM44
1278485	GCA_002599225.1	Cyanophage S-RIM44
1278485	GCA_002599265.1	Cyanophage S-RIM44
1278485	GCA_002599285.1	Cyanophage S-RIM44
1278485	GCA_002599205.1	Cyanophage S-RIM44
1278485	GCA_002599245.1	Cyanophage S-RIM44
687803	GCA_001754245.1	Cyanophage S-RIM50
616674	GCA_000907035.1	Cyanophage SS120-1
536464	GCA_002710275.1	Cyanophage S-SSM2
682650	GCA_002710385.1	Cyanophage S-SSM6a
682651	GCA_002710425.1	Cyanophage S-SSM6b
1048189	GCA_003344325.1	Cyanophage S-TIM4
1137745	GCA_000902515.1	Cyanophage S-TIM5
536472	GCA_002710245.1	Cyanophage Syn10
536473	GCA_002710325.1	Cyanophage Syn2
536474	GCA_000907215.1	Cyanophage Syn30
1282444	GCA_000918055.1	Cyanoramphus nest associated circular K DNA virus
1282443	GCA_000919075.1	Cyanoramphus nest associated circular X DNA virus
2293277	GCA_003847005.1	Cybaeus spider associated circular virus 1
2293278	GCA_003847305.1	Cybaeus spider associated circular virus 2
549205	GCA_000875545.1	Cycad leaf necrosis virus
173976	GCA_000860925.1	Cycas necrotic stunt virus
942035	GCA_000890515.1	Cyclovirus bat/USA/2009
742920	GCA_002819925.1	Cyclovirus Chimp11
1673638	GCA_002820045.1	Cyclovirus Equ1
742922	GCA_002820165.1	Cyclovirus NG12
742923	GCA_002820185.1	Cyclovirus NG14
942037	GCA_000887995.1	Cyclovirus NGchicken15/NGA/2009
942036	GCA_002819905.1	Cyclovirus NGchicken8/NGA/2009
742915	GCA_002820085.1	Cyclovirus PK5006
742916	GCA_002820145.1	Cyclovirus PK5034
742917	GCA_002820105.1	Cyclovirus PK5222
742918	GCA_002820065.1	Cyclovirus PK5510
942032	GCA_002819885.1	Cyclovirus PKbeef23/PAK/2009
942034	GCA_000891475.1	Cyclovirus PKgoat11/PAK/2009
942033	GCA_000889655.1	Cyclovirus PKgoat21/PAK/2009
1520935	GCA_002820225.1	Cyclovirus SL-108277
742924	GCA_002820125.1	Cyclovirus TN25
1592764	GCA_001184985.1	Cyclovirus TsCyV-1_JP-NUBS-2014
1348500	GCA_000923835.1	Cyclovirus VN
1348500	GCA_002820205.1	Cyclovirus VN
1506568	GCA_000925995.1	Cyclovirus ZM36a
28289	GCA_000839785.1	Cydia pomonella granulovirus

2293728	GCA_003403335.1	Cylindrospermopsis raciborskii virus RM-2018a
1602124	GCA_001020015.1	Cymbidium chlorotic mosaic virus
12178	GCA_000865585.1	Cymbidium mosaic virus
12144	GCA_000854345.1	Cymbidium ringspot virus
192023	GCA_000854005.1	Cymbidium ringspot virus satellite RNA
507488	GCA_002114045.1	Cynomolgus adenovirus 1
1919083	GCA_001963235.1	Cynomolgus cytomegalovirus
317858	GCA_000903355.1	Cyprinid herpesvirus 1
317878	GCA_000900815.1	Cyprinid herpesvirus 2
180230	GCA_000871465.1	Cyprinid herpesvirus 3
861561	GCA_000898155.1	Cyrtanthus elatus virus A
1758881	GCA_001461225.1	Daeseongdong virus 1
1758882	GCA_001461345.1	Daeseongdong virus 2
1278205	GCA_000906555.1	Dahlia latent viroid
213888	GCA_000900115.1	Dahlia mosaic virus
1238455	GCA_002816415.1	Dak Nong virus
1227356	GCA_000900175.1	Dalechampia chlorotic mosaic virus
1469956	GCA_000918875.1	Danaus plexippus plexippus iteravirus
578107	GCA_000869045.1	Daphne mosaic virus
216614	GCA_000867245.1	Daphne virus S
282712	GCA_003029315.1	Daphne virus Y
1934253	GCA_900473875.1	Daphnia iridovirus 1
1986950	GCA_004117655.1	Daphnis nerii cypovirus
29271	GCA_000860885.1	Dasheen mosaic virus
1227357	GCA_000897735.1	Datura leaf distortion virus
195059	GCA_001432095.1	Datura yellow vein nucleorhabdovirus
1965063	GCA_000943665.1	DeBrazza's monkey arterivirus
1513224	GCA_000844705.1	Decapod penstyldensovirus 1
749413	GCA_000900995.1	Deep-sea thermophilic phage D6E
1843767	GCA_001646595.1	Deer faeces associated circular DNA virus 1
2170000	GCA_002163405.1	Deer mastadenovirus B
305674	GCA_000861985.1	Deerpox virus W-848-83
198112	GCA_000852585.1	Deformed wing virus
1812308	GCA_001619035.1	Deinbollia mosaic virus
1647385	GCA_001470995.1	Delftia phage IME-DE1
665032	GCA_000888055.1	Delftia phage PhiW-14
1563661	GCA_002037815.1	Delftia phage RG-2014
1781241	GCA_001560985.1	Delisea pulchra RNA virus
706524	GCA_003178775.1	Delphinus delphis papillomavirus
1891756	GCA_000928955.1	Delphinus delphis polyomavirus 1
10565	GCA_000864665.1	Deltapapillomavirus 1
10564	GCA_000838705.1	Deltapapillomavirus 2
56144	GCA_000863705.1	Deltapapillomavirus 3
337052	GCA_000863985.1	Deltapapillomavirus 4
1175853	GCA_003179795.1	Deltapapillomavirus 5
1890427	GCA_002830505.1	Deltasatellite sat-603
1577776	GCA_000930615.1	Dendrolimus punctatus cypovirus 22
292208	GCA_000844365.1	Dendrolimus punctatus densovirus
272751	GCA_000857905.1	Dendrolimus punctatus virus
11053	GCA_000862125.1	Dengue virus 1

31634	GCA_000871845.1	Dengue virus 2 Thailand/16681/84
11069	GCA_000866625.1	Dengue virus 3
11070	GCA_000865065.1	Dengue virus 4
361731	GCA_000869465.1	Desmodium leaf distortion virus
1960710	GCA_003029545.2	Desmodium mottle virus
1960710	GCA_003029545.1	Desmodium mottle virus
1622024	GCA_001012905.1	Desmodus rotundus endogenous retrovirus
1926498	GCA_001904865.1	Desmodus rotundus parvovirus
1606503	GCA_001430015.1	Desmodus rotundus polyomavirus 1
1920991	GCA_003729055.1	Diabrotica virgifera virgifera virus 1
1920988	GCA_002116275.1	Diabrotica virgifera virgifera virus 2
1920989	GCA_002080155.1	Diabrotica virgifera virgifera virus 3
109981	GCA_003181295.1	Diachasmimorpha longicaudata entomopoxvirus
1585246	GCA_001684605.1	Diachasmimorpha longicaudata rhabdovirus
158683	GCA_000881595.1	Diadromus pulchellus ascovirus 4a
1972700	GCA_002117775.1	Diamondback moth iflavirus
2060479	GCA_002890255.1	Dianke virus
1776153	GCA_001661795.1	Diaphorina citri densovirus
1868621	GCA_001685345.1	Diaphorina citri flavi-like virus
111470	GCA_000856245.1	Diaporthe ambigua RNA virus 1
547467	GCA_000874725.1	Diascia yellow mottle virus
1678159	GCA_003957765.1	Diatom colony associated dsRNA virus 1
1678170	GCA_004128435.1	Diatom colony associated dsRNA virus 10
1678171	GCA_004128455.1	Diatom colony associated dsRNA virus 11
1678172	GCA_004128475.1	Diatom colony associated dsRNA virus 12
1678173	GCA_004128495.1	Diatom colony associated dsRNA virus 13
1678174	GCA_004106585.1	Diatom colony associated dsRNA virus 14
1678175	GCA_004128515.1	Diatom colony associated dsRNA virus 15
1678176	GCA_003726135.1	Diatom colony associated dsRNA virus 16
1678177	GCA_003956605.1	Diatom colony associated dsRNA virus 17 genome type A
1678178	GCA_003956585.1	Diatom colony associated dsRNA virus 17 genome type B
1678160	GCA_003956625.1	Diatom colony associated dsRNA virus 2
1678161	GCA_004128275.1	Diatom colony associated dsRNA virus 3
1678162	GCA_004128295.1	Diatom colony associated dsRNA virus 4 genome type A
1678163	GCA_004128315.1	Diatom colony associated dsRNA virus 4 genome type B
1678164	GCA_004128335.1	Diatom colony associated dsRNA virus 5
1678165	GCA_004128355.1	Diatom colony associated dsRNA virus 6
1678166	GCA_004128375.1	Diatom colony associated dsRNA virus 7
1678167	GCA_004128395.1	Diatom colony associated dsRNA virus 8
1678168	GCA_004128415.1	Diatom colony associated dsRNA virus 9 genome type A
1678179	GCA_004128535.1	Diatom colony associated ssRNA virus 1
1678180	GCA_004128555.1	Diatom colony associated ssRNA virus 2
72003	GCA_000839405.1	Diatraea saccharalis densovirus
1675862	GCA_001461605.1	Diatraea saccharalis granulovirus
2163629	GCA_003093915.1	Dickeya phage Amaethon
1698708	GCA_002607285.1	Dickeya phage BF25/12
2320188	GCA_003691715.1	Dickeya phage Coodle
2163630	GCA_003094255.1	Dickeya phage Dagda
2163630	GCA_003094255.2	Dickeya phage Dagda
2320189	GCA_003575345.1	Dickeya phage Dagda_B1

1983655	GCA_002623965.1	Dickeya phage JA15
2320190	GCA_003691735.1	Dickeya phage Kamild
2320191	GCA_003575385.1	Dickeya phage Katbat
2320192	GCA_003575425.1	Dickeya phage Luksen
2320193	GCA_003575465.1	Dickeya phage Mysterion
2163631	GCA_003094335.1	Dickeya phage Ninurta
1542131	GCA_002601885.1	Dickeya phage phiD3
1542132	GCA_003059875.1	Dickeya phage phiDP10.3
1542133	GCA_003059825.1	Dickeya phage phiDP23.1
2053675	GCA_002957315.1	Dickeya phage PP35
1477406	GCA_000924915.1	Dickeya phage RC-2014
2163632	GCA_003093895.1	Dickeya phage Sucellus
2283029	GCA_003568515.1	Dickeya phage vB_DsoM_AD1
2382310	GCA_003613635.1	Dickeya phage vB_DsoM_JA11
2283030	GCA_003568455.1	Dickeya phage vB_DsoM_JA13
2283031	GCA_003568475.1	Dickeya phage vB_DsoM_JA29
2283032	GCA_003568495.1	Dickeya phage vB_DsoM_JA33
2283033	GCA_003568435.1	Dickeya phage vB_DsoP_JA10
1983656	GCA_002623985.1	Dickeya phage XF4
1091052	GCA_000903075.1	Dickeya virus Limestone
94700	GCA_000840105.1	Dicliptera yellow mottle virus
94700	GCA_002986495.1	Dicliptera yellow mottle virus
1692247	GCA_001274305.1	Didemnum sp. Sea Squirt associated virus
1196237	GCA_000900075.1	Digitaria ciliaris striate mosaic virus
1196237	GCA_002825045.1	Digitaria ciliaris striate mosaic virus
889510	GCA_000889315.1	Digitaria didactyla striate mosaic virus
10837	GCA_000838685.1	Digitaria streak virus
1408895	GCA_000912815.1	Dill cryptic virus 1
1323529	GCA_000908315.1	Dill cryptic virus 2
1541119	GCA_000929955.1	Dinocampus coccinellae paralysis virus
1477404	GCA_000923015.1	Dinoroseobacter phage DFL12phi1
1965368	GCA_002619945.1	Dinoroseobacter phage vB_DshS-R5C
1498169	GCA_002604525.1	Dinoroseobacter phage vBDshPR2C
656520	GCA_002867365.1	Diodia vein chlorosis virus
2083300	GCA_002833945.1	Diolcogaster facetosa bracovirus
1619899	GCA_002819385.1	Dioscorea bacilliform AL virus
2169727	GCA_003029345.1	Dioscorea bacilliform RT virus 1
2011125	GCA_003029355.1	Dioscorea bacilliform RT virus 2
1755696	GCA_000870445.1	Dioscorea bacilliform SN virus
2169728	GCA_003029445.1	Dioscorea bacilliform TR virus
2170181	GCA_001876835.1	Dioscorea mosaic associated virus
2303485	GCA_004133365.1	Dioscorea nummularia-associated virus
459770	GCA_000888075.1	Diplodia scrobiculata RNA virus 1
1974422	GCA_003729015.1	Diporeia-associated CRESS-DNA virus LH481
1974423	GCA_003729035.1	Diporeia-associated CRESS-DNA virus LM122
148880	GCA_000837285.1	Discula destructiva virus 1
148880	GCA_000837285.2	Discula destructiva virus 1
160484	GCA_000851345.1	Discula destructiva virus 2
2079134	GCA_002937195.1	Dishui lake phycodnavirus 1
1739971	GCA_002607685.1	Dishui lake virophage 1

1247115	GCA_000899555.1	Diuris virus A
1247116	GCA_000901495.1	Diuris virus B
1980467	GCA_000863045.1	Dobrava-Belgrade orthohantavirus
333968	GCA_001343705.1	Dolichos yellow mosaic virus
333968	GCA_002822285.1	Dolichos yellow mosaic virus
37131	GCA_000857405.1	Dolphin morbillivirus
1511639	GCA_000924815.1	Dolphin rhabdovirus
2107574	GCA_004133985.1	Domestic cat hepadnavirus
985683	GCA_000894455.1	Donggang virus
1400526	GCA_000914975.1	Donkey orchid symptomless virus
1198144	GCA_000907335.1	Donkey orchid virus A
380669	GCA_000867285.1	Dracaena mottle virus
292633	GCA_003972305.1	Dragon grouper nervous necrosis virus
2169905	GCA_000900895.1	Dragonfly associated alphasatellite
2152567	GCA_004133105.1	Dragonfly associated cyclovirus
1234879	GCA_000920575.1	Dragonfly associated cyclovirus 1
1234879	GCA_002819945.1	Dragonfly associated cyclovirus 1
1234880	GCA_000919015.1	Dragonfly associated cyclovirus 2
1234881	GCA_000917995.1	Dragonfly associated cyclovirus 3
1234882	GCA_000919655.1	Dragonfly associated cyclovirus 4
1234882	GCA_002819965.1	Dragonfly associated cyclovirus 4
1234883	GCA_000920495.1	Dragonfly associated cyclovirus 5
1574360	GCA_002819985.1	Dragonfly associated cyclovirus 6
1574361	GCA_002820005.1	Dragonfly associated cyclovirus 7
1574362	GCA_002820025.1	Dragonfly associated cyclovirus 8
1234887	GCA_000917975.1	Dragonfly associated gemykibivirus 1
1234872	GCA_000917895.1	Dragonfly circularisvirus
1234878	GCA_000919555.1	Dragonfly cyclicusvirus
1454021	GCA_000916895.1	Dragonfly larvae associated circular virus-1
1454022	GCA_000914355.1	Dragonfly larvae associated circular virus-10
1454022	GCA_003986425.1	Dragonfly larvae associated circular virus-10
1454023	GCA_000916035.1	Dragonfly larvae associated circular virus-2
1454024	GCA_000914335.1	Dragonfly larvae associated circular virus-3
1454025	GCA_000915375.1	Dragonfly larvae associated circular virus-4
1454026	GCA_000916875.1	Dragonfly larvae associated circular virus-5
1454027	GCA_000915355.1	Dragonfly larvae associated circular virus-6
1454028	GCA_000916015.1	Dragonfly larvae associated circular virus-7
1454029	GCA_000914315.1	Dragonfly larvae associated circular virus-8
1454030	GCA_000915335.1	Dragonfly larvae associated circular virus-9
1234874	GCA_000918915.1	Dragonfly orbiculatusvirus
1234874	GCA_003985505.1	Dragonfly orbiculatusvirus
1234886	GCA_000919635.1	Dragonfly-associated circular virus 2
1234884	GCA_000918995.1	Dragonfly-associated circular virus 3
1249648	GCA_000903435.1	Dragonfly-associated mastrevirus
1249648	GCA_003028965.1	Dragonfly-associated mastrevirus
1234888	GCA_000901395.1	Dragonfly-associated microphage 1
1662272	GCA_001271295.1	Dromedary astrovirus
2014603	GCA_002219805.1	Dromedary camel bocaparvovirus 1
2014604	GCA_002237215.1	Dromedary camel bocaparvovirus 2
1637527	GCA_002816815.1	Dromedary camel enterovirus 19CC

1574422	GCA_000929015.1	Dromedary stool-associated circular ssDNA virus
595895	GCA_000884055.1	Drosophila A virus
1308859	GCA_002815695.1	Drosophila affinis sigmavirus
1002359	GCA_002815715.1	Drosophila ananassae sigmavirus
64279	GCA_000850085.1	Drosophila C virus
1500866	GCA_000921395.1	Drosophila immigrans Nora virus
1002360	GCA_002815735.1	Drosophila immigrans sigmavirus
2057187	GCA_004132165.1	Drosophila innubila nudivirus
663279	GCA_003972825.1	Drosophila melanogaster American nodavirus (ANV) SW-20
666363	GCA_000885235.1	Drosophila melanogaster sigmavirus AP30
666961	GCA_002815755.1	Drosophila melanogaster sigmavirus HAP23
663282	GCA_000884455.1	Drosophila melanogaster totivirus SW-2009a
666962	GCA_000911135.1	Drosophila obscura sigmavirus 10A
1500865	GCA_000922235.1	Drosophila subobscura Nora virus
1802951	GCA_003673805.1	Drosophila unispina virus 1
48981	GCA_000851665.1	Drosophila X virus
1482734	GCA_000919155.1	Duck aalivirus 1
130329	GCA_000845945.1	Duck adenovirus 1
130329	GCA_000886775.1	Duck adenovirus 1
1520006	GCA_000923915.1	Duck adenovirus 2
2006585	GCA_002194385.1	Duck associated cyclovirus 1
2006585	GCA_004320265.1	Duck associated cyclovirus 1
2006585	GCA_004320285.1	Duck associated cyclovirus 1
2006585	GCA_004320305.1	Duck associated cyclovirus 1
2006585	GCA_004320325.1	Duck associated cyclovirus 1
324685	GCA_000864045.1	Duck circovirus
324685	GCA_004033715.1	Duck circovirus
324685	GCA_004034095.1	Duck circovirus
324685	GCA_003985365.1	Duck circovirus
324685	GCA_004033555.1	Duck circovirus
324685	GCA_004034235.1	Duck circovirus
324685	GCA_004062275.1	Duck circovirus
324685	GCA_004044415.1	Duck circovirus
324685	GCA_004044435.1	Duck circovirus
324685	GCA_004044455.1	Duck circovirus
324685	GCA_004044475.1	Duck circovirus
324685	GCA_004072275.1	Duck circovirus
324685	GCA_004072295.1	Duck circovirus
324685	GCA_004072315.1	Duck circovirus
324685	GCA_004072335.1	Duck circovirus
324685	GCA_004072355.1	Duck circovirus
324685	GCA_004072375.1	Duck circovirus
324685	GCA_004086535.1	Duck circovirus
324685	GCA_004074715.1	Duck circovirus
324685	GCA_004074655.1	Duck circovirus
324685	GCA_004081035.1	Duck circovirus
324685	GCA_004074675.1	Duck circovirus
324685	GCA_004074695.1	Duck circovirus
324685	GCA_004065855.1	Duck circovirus
324685	GCA_004086455.1	Duck circovirus

324685	GCA_004074755.1	Duck circovirus
324685	GCA_004074735.1	Duck circovirus
324685	GCA_004079675.1	Duck circovirus
324685	GCA_004073715.1	Duck circovirus
324685	GCA_004086495.1	Duck circovirus
324685	GCA_004074855.1	Duck circovirus
324685	GCA_004065875.1	Duck circovirus
324685	GCA_004081055.1	Duck circovirus
324685	GCA_004065895.1	Duck circovirus
324685	GCA_004074775.1	Duck circovirus
324685	GCA_004073755.1	Duck circovirus
324685	GCA_004065915.1	Duck circovirus
324685	GCA_004074795.1	Duck circovirus
324685	GCA_004086575.1	Duck circovirus
324685	GCA_004074815.1	Duck circovirus
324685	GCA_004065955.1	Duck circovirus
324685	GCA_004074835.1	Duck circovirus
324685	GCA_004086515.1	Duck circovirus
324685	GCA_004065995.1	Duck circovirus
324685	GCA_004074875.1	Duck circovirus
324685	GCA_004086475.1	Duck circovirus
324685	GCA_004073675.1	Duck circovirus
324685	GCA_004073695.1	Duck circovirus
324685	GCA_004086595.1	Duck circovirus
324685	GCA_004073735.1	Duck circovirus
324685	GCA_004073775.1	Duck circovirus
324685	GCA_004073795.1	Duck circovirus
324685	GCA_004065695.1	Duck circovirus
324685	GCA_004065715.1	Duck circovirus
324685	GCA_004065735.1	Duck circovirus
324685	GCA_004086555.1	Duck circovirus
324685	GCA_004071995.1	Duck circovirus
324685	GCA_004072015.1	Duck circovirus
324685	GCA_004081015.1	Duck circovirus
324685	GCA_004072035.1	Duck circovirus
324685	GCA_004081075.1	Duck circovirus
324685	GCA_004072055.1	Duck circovirus
324685	GCA_004072075.1	Duck circovirus
324685	GCA_004065935.1	Duck circovirus
324685	GCA_004072095.1	Duck circovirus
324685	GCA_004072115.1	Duck circovirus
324685	GCA_004072135.1	Duck circovirus
324685	GCA_004072155.1	Duck circovirus
324685	GCA_004072175.1	Duck circovirus
324685	GCA_004072195.1	Duck circovirus
324685	GCA_004072215.1	Duck circovirus
324685	GCA_004072235.1	Duck circovirus
324685	GCA_004072255.1	Duck circovirus
324685	GCA_004086615.1	Duck circovirus
324685	GCA_004097875.1	Duck circovirus

324685	GCA_004089355.1	Duck circovirus
324685	GCA_004097895.1	Duck circovirus
324685	GCA_004097915.1	Duck circovirus
324685	GCA_004097935.1	Duck circovirus
324685	GCA_004097335.1	Duck circovirus
324685	GCA_004097355.1	Duck circovirus
324685	GCA_004089375.1	Duck circovirus
324685	GCA_004097835.1	Duck circovirus
324685	GCA_004097855.1	Duck circovirus
324685	GCA_004097375.1	Duck circovirus
324685	GCA_004093995.1	Duck circovirus
324685	GCA_004098355.1	Duck circovirus
324685	GCA_004098375.1	Duck circovirus
324685	GCA_004089315.1	Duck circovirus
324685	GCA_004089335.1	Duck circovirus
1843768	GCA_001646015.1	Duck faeces associated circular DNA virus 1
1843769	GCA_001646195.1	Duck faeces associated circular DNA virus 2
1843770	GCA_001646395.1	Duck faeces associated circular DNA virus 3
1006061	GCA_000869945.1	Duck hepatitis A virus 1
12639	GCA_000847905.1	Duck hepatitis B virus
1171667	GCA_003093055.1	Duck reovirus
1171667	GCA_003092855.1	Duck reovirus
1171667	GCA_003093195.1	Duck reovirus
1532849	GCA_003093015.1	Duck reovirus SD-12
70823	GCA_000864285.1	Dulcamara mottle virus
754069	GCA_000920355.1	Dunaliella viridis virus SI2
742163	GCA_003029245.1	Duranta leaf curl virus
38767	GCA_000905375.1	Duvenhage lyssavirus
1888317	GCA_001717435.1	Duwamo virus
551224	GCA_002145645.1	Dysaphis plantaginea densovirus
223262	GCA_000858965.1	East African cassava mosaic Cameroon virus
393599	GCA_000882315.1	East African cassava mosaic Kenya virus
223264	GCA_000912855.1	East African cassava mosaic Malawi virus
223265	GCA_002986505.1	East African cassava mosaic Malawi virus-Malawi[K]
62079	GCA_002822305.1	East African cassava mosaic virus
62079	GCA_002867495.1	East African cassava mosaic virus
374778	GCA_002986525.1	East African cassava mosaic virus-KE2
223272	GCA_000859785.1	East African cassava mosaic virus-Uganda2 Severe
223275	GCA_000845125.1	East African cassava mosaic Zanzibar virus
341167	GCA_000866965.1	East Asian Passiflora virus
2170195	GCA_003032765.1	Eastern chimpanzee simian foamy virus
11021	GCA_000862705.1	Eastern equine encephalitis virus
1570291	GCA_900094155.1	Ebola virus
128952	GCA_000848505.1	Ebola virus - Mayinga, Zaire, 1976
2480191	GCA_004132965.1	Ecklonia radiata-associated virus 1
2480193	GCA_004132985.1	Ecklonia radiata-associated virus 3
2480195	GCA_004133005.1	Ecklonia radiata-associated virus 5
2480197	GCA_004133025.1	Ecklonia radiata-associated virus 7
2480198	GCA_004133045.1	Ecklonia radiata-associated virus 8
875324	GCA_000895255.1	Eclipta yellow vein virus

37665	GCA_000839765.1	Ectocarpus siliculosus virus 1
12643	GCA_000841905.1	Ectromelia virus
59376	GCA_000868645.1	Ectropis obliqua nucleopolyhedrovirus
240555	GCA_000855305.1	Ectropis obliqua picorna-like virus
64296	GCA_001661755.1	Edge Hill virus
2419942	GCA_003718995.1	Edwardsiella phage Edno5
945083	GCA_002630905.1	Edwardsiella phage eiAU
1391659	GCA_000914675.1	Edwardsiella phage eiAU-183
1537091	GCA_000954295.1	Edwardsiella phage GF-2
1244857	GCA_002603005.1	Edwardsiella phage IW-1
1244856	GCA_000900595.1	Edwardsiella phage KF-1
1264700	GCA_000905655.1	Edwardsiella phage MSW-3
1608310	GCA_001470475.1	Edwardsiella phage PEi20
1325372	GCA_000915155.1	Edwardsiella phage PEi21
1608311	GCA_002633215.1	Edwardsiella phage PEi26
1398149	GCA_000910995.1	Eel picornavirus 1
1609634	GCA_000959675.1	Eel River basin pequenovirus
1609634	GCA_002922005.1	Eel River basin pequenovirus
1609634	GCA_002922065.1	Eel River basin pequenovirus
1609634	GCA_002922035.1	Eel River basin pequenovirus
1609634	GCA_002921875.1	Eel River basin pequenovirus
1609634	GCA_002921945.1	Eel River basin pequenovirus
1609634	GCA_002922015.1	Eel River basin pequenovirus
1609634	GCA_002921905.1	Eel River basin pequenovirus
1609634	GCA_002921885.1	Eel River basin pequenovirus
1609634	GCA_002921985.1	Eel River basin pequenovirus
1609634	GCA_002922025.1	Eel River basin pequenovirus
1609634	GCA_002921995.1	Eel River basin pequenovirus
1609634	GCA_002921975.1	Eel River basin pequenovirus
1609634	GCA_002921925.1	Eel River basin pequenovirus
1609634	GCA_002921955.1	Eel River basin pequenovirus
1609634	GCA_002921895.1	Eel River basin pequenovirus
1609634	GCA_002921965.1	Eel River basin pequenovirus
1609634	GCA_002921915.1	Eel River basin pequenovirus
1609634	GCA_002922045.1	Eel River basin pequenovirus
1609634	GCA_002922055.1	Eel River basin pequenovirus
1609634	GCA_002921935.1	Eel River basin pequenovirus
1609634	GCA_003846075.1	Eel River basin pequenovirus
685443	GCA_000912795.1	Eel virus European X
1888318	GCA_001717235.1	Egaro virus
2283015	GCA_003367055.1	Eggerthella phage PMBT5
219174	GCA_000853945.1	Eggplant latent viroid
219174	GCA_003047555.1	Eggplant latent viroid
981403	GCA_002827945.1	Eggplant mild leaf mottle virus
12151	GCA_000847385.1	Eggplant mosaic virus
270256	GCA_000916815.1	Eggplant mottled crinkle virus
488317	GCA_000927295.1	Eggplant mottled dwarf nucleorhabdovirus
1131483	GCA_000896735.1	Eidolon helvum (bat) parvovirus
1163701	GCA_002826965.1	Eidolon helvum papillomavirus 1
1335476	GCA_002004435.1	Eidolon helvum papillomavirus 2

1335477	GCA_002004275.1	Eidolon helvum papillomavirus 3
1891722	GCA_000903035.1	Eidolon helvum polyomavirus 1
1231903	GCA_000898655.1	Eilat virus
155414	GCA_000849445.1	Eimeria brunetti RNA virus 1
1898175	GCA_004129715.1	Eimeria stiedai RNA virus 1
1566868	GCA_000929115.1	Eimeria tenella RNA virus 1
1987021	GCA_002815875.1	Ekpoma virus 2
2304214	GCA_004133385.1	Elderberry aureusvirus 1
1569052	GCA_001551605.1	Elderberry carlavirus A
1569053	GCA_001550925.1	Elderberry carlavirus B
1569054	GCA_001550545.1	Elderberry carlavirus C
1569055	GCA_001551245.1	Elderberry carlavirus D
1569056	GCA_001551585.1	Elderberry carlavirus E
167018	GCA_000930275.1	Elderberry latent virus
548914	GCA_001443905.1	Elephant endotheliotropic herpesvirus 4
768738	GCA_000922355.1	Elephant endotheliotropic herpesvirus 5
146015	GCA_000905835.1	Elephantid betaherpesvirus 1
2480175	GCA_004132285.1	Elicom virus 1
56486	GCA_000850545.1	Elm mottle virus
2020881	GCA_002270945.1	Emilia sonchifolia yellow vein Thailand virus
498805	GCA_000879075.1	Emilia yellow vein virus-[Fz1]
640136	GCA_000881315.1	Emilia yellow vein virus-associated DNA beta
181216	GCA_003258235.1	Emiliania huxleyi virus 84
181082	GCA_000865825.1	Emiliania huxleyi virus 86
187397	GCA_003071505.1	Emiliania huxleyi virus 99B1
754068	GCA_003225875.1	Emiliania huxleyi virus PS401
12104	GCA_000862985.1	Encephalomyocarditis virus
73423	GCA_002116235.1	Endive necrotic mosaic virus
1472717	GCA_000919095.1	Enhydra lutris papillomavirus 1
64283	GCA_000870345.1	Entebbe bat virus
1864622	GCA_001744875.1	Enterobacter phage Arya
709484	GCA_000889435.1	Enterobacter phage CC31
1636313	GCA_001550525.1	Enterobacter phage E-2
1636314	GCA_001501615.1	Enterobacter phage E-3
1636315	GCA_002605665.1	Enterobacter phage E-4
2070180	GCA_003013875.1	Enterobacter phage Ec_L1
942016	GCA_000900655.1	Enterobacter phage EcP1
2483604	GCA_003991785.1	Enterobacter phage EcpYZU01
1150990	GCA_000901895.1	Enterobacter phage Enc34
1871708	GCA_002709805.1	Enterobacter phage KNP3
1871712	GCA_002991185.1	Enterobacter phage KNP7
2108137	GCA_003034585.1	Enterobacter phage myPSH1140
1455074	GCA_000916315.1	Enterobacter phage PG7
2340711	GCA_003613535.1	Enterobacter phage phi63_307
1587520	GCA_001503795.1	Enterobacter phage phiEap-1
1701257	GCA_001471055.1	Enterobacter phage phiEap-2
1682394	GCA_002624485.1	Enterobacter phage phiEap-3
2137745	GCA_003034835.1	Enterobacter phage phiEM4
1147139	GCA_002602105.1	Enterobacter phage phiKDA1
2340712	GCA_003613605.1	Enterobacter phage phiT5282H

1864623	GCA_001744195.1	Enterobacter phage Tyrion
532076	GCA_000880255.1	Enterobacteria phage 13a
254397	GCA_002594645.1	Enterobacteria phage 2851
669008	GCA_000892355.1	Enterobacteria phage 285P
10730	GCA_000837725.1	Enterobacteria phage 933W
1468411	GCA_000920815.1	Enterobacteria phage 9g
10849	GCA_000846145.1	Enterobacteria phage alpha3
10849	GCA_002601605.1	Enterobacteria phage alpha3
1651202	GCA_003605995.1	Enterobacteria phage Aplg8
1651196	GCA_003606015.1	Enterobacteria phage ATK47
1651197	GCA_003606035.1	Enterobacteria phage ATK48
532074	GCA_000874625.1	Enterobacteria phage BA14
196242	GCA_000843125.1	Enterobacteria phage BP-4795
1052121	GCA_000900735.1	Enterobacteria phage Bp7
1206313	GCA_000901295.1	Enterobacteria phage C-1 INW-2012
414970	GCA_000873845.1	Enterobacteria phage cdtI
539221	GCA_002630425.1	Enterobacteria phage CUS-3
482822	GCA_002758635.1	Enterobacteria phage DE3
1567007	GCA_002605085.1	Enterobacteria phage DT571/2
1784948	GCA_002609205.1	Enterobacteria phage ECGD1
532075	GCA_000875445.1	Enterobacteria phage EcoDS1
101570	GCA_000858165.1	Enterobacteria phage ES18
10863	GCA_000929915.1	Enterobacteria phage f1
10863	GCA_002921565.1	Enterobacteria phage f1
10863	GCA_002921535.1	Enterobacteria phage f1
10863	GCA_002921675.1	Enterobacteria phage f1
10863	GCA_002921545.1	Enterobacteria phage f1
10863	GCA_002921685.1	Enterobacteria phage f1
10863	GCA_002921555.1	Enterobacteria phage f1
10863	GCA_002921365.1	Enterobacteria phage f1
10863	GCA_002921695.1	Enterobacteria phage f1
10863	GCA_002921355.1	Enterobacteria phage f1
10863	GCA_002991195.1	Enterobacteria phage f1
10864	GCA_000930555.1	Enterobacteria phage fd
1357825	GCA_000912255.1	Enterobacteria phage fiAA91-ss
1340813	GCA_002755855.1	Enterobacteria phage FL68 Tallahassee/FL/2012
1340814	GCA_002755875.1	Enterobacteria phage FL76 Tallahassee/FL/2012
12017	GCA_002786405.1	Enterobacteria phage fr
10843	GCA_000840785.1	Enterobacteria phage G4
10843	GCA_002921665.1	Enterobacteria phage G4
10843	GCA_002921595.1	Enterobacteria phage G4
10843	GCA_002921605.1	Enterobacteria phage G4
10843	GCA_002921575.1	Enterobacteria phage G4
10843	GCA_002921645.1	Enterobacteria phage G4
10843	GCA_002921375.1	Enterobacteria phage G4
10843	GCA_002921585.1	Enterobacteria phage G4
10843	GCA_002921655.1	Enterobacteria phage G4
12018	GCA_000847365.1	Enterobacteria phage GA
1222338	GCA_000926715.1	Enterobacteria phage GEC-3S
1651199	GCA_003606055.1	Enterobacteria phage GiZh

1206300	GCA_000903835.1	Enterobacteria phage Hgal1
432198	GCA_000903655.1	Enterobacteria phage HK106
1147143	GCA_000901015.1	Enterobacteria phage HK140
1147144	GCA_000902675.1	Enterobacteria phage HK225
338111	GCA_002596545.1	Enterobacteria phage ID1
310953	GCA_002537585.1	Enterobacteria phage ID11
310953	GCA_002539615.1	Enterobacteria phage ID11
310953	GCA_002543665.1	Enterobacteria phage ID11
310953	GCA_002539635.1	Enterobacteria phage ID11
310953	GCA_002543695.1	Enterobacteria phage ID11
310953	GCA_002535665.1	Enterobacteria phage ID11
310953	GCA_002539655.1	Enterobacteria phage ID11
310953	GCA_002545385.1	Enterobacteria phage ID11
310953	GCA_002545785.1	Enterobacteria phage ID11
310953	GCA_002543735.1	Enterobacteria phage ID11
310953	GCA_002545815.1	Enterobacteria phage ID11
310953	GCA_002539675.1	Enterobacteria phage ID11
310953	GCA_002541645.1	Enterobacteria phage ID11
310953	GCA_002537635.1	Enterobacteria phage ID11
310953	GCA_002535685.1	Enterobacteria phage ID11
310953	GCA_002539695.1	Enterobacteria phage ID11
310953	GCA_002543755.1	Enterobacteria phage ID11
310953	GCA_002535705.1	Enterobacteria phage ID11
310953	GCA_002539715.1	Enterobacteria phage ID11
310953	GCA_002537665.1	Enterobacteria phage ID11
310953	GCA_002541745.1	Enterobacteria phage ID11
310953	GCA_002535745.1	Enterobacteria phage ID11
310953	GCA_002539735.1	Enterobacteria phage ID11
310953	GCA_002537715.1	Enterobacteria phage ID11
310953	GCA_002539755.1	Enterobacteria phage ID11
310953	GCA_002545905.1	Enterobacteria phage ID11
310953	GCA_002539775.1	Enterobacteria phage ID11
310953	GCA_002537755.1	Enterobacteria phage ID11
310953	GCA_002541835.1	Enterobacteria phage ID11
310953	GCA_002539805.1	Enterobacteria phage ID11
310953	GCA_002544045.1	Enterobacteria phage ID11
310953	GCA_002545945.1	Enterobacteria phage ID11
310953	GCA_002539825.1	Enterobacteria phage ID11
310953	GCA_002535835.1	Enterobacteria phage ID11
310953	GCA_002539845.1	Enterobacteria phage ID11
310953	GCA_002541865.1	Enterobacteria phage ID11
310953	GCA_002539865.1	Enterobacteria phage ID11
310953	GCA_002535855.1	Enterobacteria phage ID11
310953	GCA_002539885.1	Enterobacteria phage ID11
310953	GCA_002543935.1	Enterobacteria phage ID11
310953	GCA_002545745.1	Enterobacteria phage ID11
310953	GCA_002537825.1	Enterobacteria phage ID11
310953	GCA_002541905.1	Enterobacteria phage ID11
310953	GCA_002545405.1	Enterobacteria phage ID11
310953	GCA_002535875.1	Enterobacteria phage ID11

310953	GCA_002539915.1	Enterobacteria phage ID11
310953	GCA_002545065.1	Enterobacteria phage ID11
310953	GCA_002543965.1	Enterobacteria phage ID11
310953	GCA_002537865.1	Enterobacteria phage ID11
310953	GCA_002535925.1	Enterobacteria phage ID11
310953	GCA_002544385.1	Enterobacteria phage ID11
310953	GCA_002541945.1	Enterobacteria phage ID11
310953	GCA_002541685.1	Enterobacteria phage ID11
310953	GCA_002544005.1	Enterobacteria phage ID11
310953	GCA_002539945.1	Enterobacteria phage ID11
310953	GCA_002535975.1	Enterobacteria phage ID11
310953	GCA_002542675.1	Enterobacteria phage ID11
310953	GCA_002539965.1	Enterobacteria phage ID11
310953	GCA_002537915.1	Enterobacteria phage ID11
310953	GCA_002541975.1	Enterobacteria phage ID11
310953	GCA_002539985.1	Enterobacteria phage ID11
310953	GCA_002541995.1	Enterobacteria phage ID11
310953	GCA_002538615.1	Enterobacteria phage ID11
310953	GCA_002540005.1	Enterobacteria phage ID11
310953	GCA_002542035.1	Enterobacteria phage ID11
310953	GCA_002537615.1	Enterobacteria phage ID11
310953	GCA_002540025.1	Enterobacteria phage ID11
310953	GCA_002545095.1	Enterobacteria phage ID11
310953	GCA_002544755.1	Enterobacteria phage ID11
310953	GCA_002539315.1	Enterobacteria phage ID11
310953	GCA_002540045.1	Enterobacteria phage ID11
310953	GCA_002536705.1	Enterobacteria phage ID11
310953	GCA_002544075.1	Enterobacteria phage ID11
310953	GCA_002542075.1	Enterobacteria phage ID11
310953	GCA_002536035.1	Enterobacteria phage ID11
310953	GCA_002537975.1	Enterobacteria phage ID11
310953	GCA_002536055.1	Enterobacteria phage ID11
310953	GCA_002540065.1	Enterobacteria phage ID11
310953	GCA_002538005.1	Enterobacteria phage ID11
310953	GCA_002536995.1	Enterobacteria phage ID11
310953	GCA_002536115.1	Enterobacteria phage ID11
310953	GCA_002540085.1	Enterobacteria phage ID11
310953	GCA_002542125.1	Enterobacteria phage ID11
310953	GCA_002538035.1	Enterobacteria phage ID11
310953	GCA_002537955.1	Enterobacteria phage ID11
310953	GCA_002540105.1	Enterobacteria phage ID11
310953	GCA_002545115.1	Enterobacteria phage ID11
310953	GCA_002538085.1	Enterobacteria phage ID11
310953	GCA_002542165.1	Enterobacteria phage ID11
310953	GCA_002540125.1	Enterobacteria phage ID11
310953	GCA_002542195.1	Enterobacteria phage ID11
310953	GCA_002538125.1	Enterobacteria phage ID11
310953	GCA_002536015.1	Enterobacteria phage ID11
310953	GCA_002540155.1	Enterobacteria phage ID11
310953	GCA_002536175.1	Enterobacteria phage ID11

310953	GCA_002540175.1	Enterobacteria phage ID11
310953	GCA_002536195.1	Enterobacteria phage ID11
310953	GCA_002540195.1	Enterobacteria phage ID11
310953	GCA_002536215.1	Enterobacteria phage ID11
310953	GCA_002540225.1	Enterobacteria phage ID11
310953	GCA_002538185.1	Enterobacteria phage ID11
310953	GCA_002542265.1	Enterobacteria phage ID11
310953	GCA_002545475.1	Enterobacteria phage ID11
310953	GCA_002536245.1	Enterobacteria phage ID11
310953	GCA_002540245.1	Enterobacteria phage ID11
310953	GCA_002545145.1	Enterobacteria phage ID11
310953	GCA_002544675.1	Enterobacteria phage ID11
310953	GCA_002536275.1	Enterobacteria phage ID11
310953	GCA_002540265.1	Enterobacteria phage ID11
310953	GCA_002542305.1	Enterobacteria phage ID11
310953	GCA_002541715.1	Enterobacteria phage ID11
310953	GCA_002544115.1	Enterobacteria phage ID11
310953	GCA_002544355.1	Enterobacteria phage ID11
310953	GCA_002540295.1	Enterobacteria phage ID11
310953	GCA_002538225.1	Enterobacteria phage ID11
310953	GCA_002536295.1	Enterobacteria phage ID11
310953	GCA_002540315.1	Enterobacteria phage ID11
310953	GCA_002542345.1	Enterobacteria phage ID11
310953	GCA_002536345.1	Enterobacteria phage ID11
310953	GCA_002538265.1	Enterobacteria phage ID11
310953	GCA_002540345.1	Enterobacteria phage ID11
310953	GCA_002542375.1	Enterobacteria phage ID11
310953	GCA_002538295.1	Enterobacteria phage ID11
310953	GCA_002542425.1	Enterobacteria phage ID11
310953	GCA_002544445.1	Enterobacteria phage ID11
310953	GCA_002540365.1	Enterobacteria phage ID11
310953	GCA_002545175.1	Enterobacteria phage ID11
310953	GCA_002544815.1	Enterobacteria phage ID11
310953	GCA_002537055.1	Enterobacteria phage ID11
310953	GCA_002536385.1	Enterobacteria phage ID11
310953	GCA_002544475.1	Enterobacteria phage ID11
310953	GCA_002540385.1	Enterobacteria phage ID11
310953	GCA_002538325.1	Enterobacteria phage ID11
310953	GCA_002543775.1	Enterobacteria phage ID11
310953	GCA_002540415.1	Enterobacteria phage ID11
310953	GCA_002536415.1	Enterobacteria phage ID11
310953	GCA_002538355.1	Enterobacteria phage ID11
310953	GCA_002542465.1	Enterobacteria phage ID11
310953	GCA_002540435.1	Enterobacteria phage ID11
310953	GCA_002539245.1	Enterobacteria phage ID11
310953	GCA_002538385.1	Enterobacteria phage ID11
310953	GCA_002536445.1	Enterobacteria phage ID11
310953	GCA_002540455.1	Enterobacteria phage ID11
310953	GCA_002542495.1	Enterobacteria phage ID11
310953	GCA_002538675.1	Enterobacteria phage ID11

310953	GCA_002536465.1	Enterobacteria phage ID11
310953	GCA_002540475.1	Enterobacteria phage ID11
310953	GCA_002540495.1	Enterobacteria phage ID11
310953	GCA_002536525.1	Enterobacteria phage ID11
310953	GCA_002542515.1	Enterobacteria phage ID11
310953	GCA_002540515.1	Enterobacteria phage ID11
310953	GCA_002544155.1	Enterobacteria phage ID11
310953	GCA_002538465.1	Enterobacteria phage ID11
310953	GCA_002542575.1	Enterobacteria phage ID11
310953	GCA_002544595.1	Enterobacteria phage ID11
310953	GCA_002543815.1	Enterobacteria phage ID11
310953	GCA_002540535.1	Enterobacteria phage ID11
310953	GCA_002545885.1	Enterobacteria phage ID11
310953	GCA_002542635.1	Enterobacteria phage ID11
310953	GCA_002536565.1	Enterobacteria phage ID11
310953	GCA_002540565.1	Enterobacteria phage ID11
310953	GCA_002540585.1	Enterobacteria phage ID11
310953	GCA_002538495.1	Enterobacteria phage ID11
310953	GCA_002540605.1	Enterobacteria phage ID11
310953	GCA_002538525.1	Enterobacteria phage ID11
310953	GCA_002543135.1	Enterobacteria phage ID11
310953	GCA_002536595.1	Enterobacteria phage ID11
310953	GCA_002540625.1	Enterobacteria phage ID11
310953	GCA_002538575.1	Enterobacteria phage ID11
310953	GCA_002544715.1	Enterobacteria phage ID11
310953	GCA_002536615.1	Enterobacteria phage ID11
310953	GCA_002544495.1	Enterobacteria phage ID11
310953	GCA_002540645.1	Enterobacteria phage ID11
310953	GCA_002536755.1	Enterobacteria phage ID11
310953	GCA_002542655.1	Enterobacteria phage ID11
310953	GCA_002543835.1	Enterobacteria phage ID11
310953	GCA_002536655.1	Enterobacteria phage ID11
310953	GCA_002544415.1	Enterobacteria phage ID11
310953	GCA_002540665.1	Enterobacteria phage ID11
310953	GCA_002535795.1	Enterobacteria phage ID11
310953	GCA_002540695.1	Enterobacteria phage ID11
310953	GCA_002542695.1	Enterobacteria phage ID11
310953	GCA_002545855.1	Enterobacteria phage ID11
310953	GCA_002542715.1	Enterobacteria phage ID11
310953	GCA_002540715.1	Enterobacteria phage ID11
310953	GCA_002538645.1	Enterobacteria phage ID11
310953	GCA_002542735.1	Enterobacteria phage ID11
310953	GCA_002540735.1	Enterobacteria phage ID11
310953	GCA_002545235.1	Enterobacteria phage ID11
310953	GCA_002544885.1	Enterobacteria phage ID11
310953	GCA_002542755.1	Enterobacteria phage ID11
310953	GCA_002544525.1	Enterobacteria phage ID11
310953	GCA_002540765.1	Enterobacteria phage ID11
310953	GCA_002544175.1	Enterobacteria phage ID11
310953	GCA_002542775.1	Enterobacteria phage ID11

310953	GCA_002543875.1	Enterobacteria phage ID11
310953	GCA_002541145.1	Enterobacteria phage ID11
310953	GCA_002540785.1	Enterobacteria phage ID11
310953	GCA_002538695.1	Enterobacteria phage ID11
310953	GCA_002542795.1	Enterobacteria phage ID11
310953	GCA_002540805.1	Enterobacteria phage ID11
310953	GCA_002538725.1	Enterobacteria phage ID11
310953	GCA_002542815.1	Enterobacteria phage ID11
310953	GCA_002540825.1	Enterobacteria phage ID11
310953	GCA_002536775.1	Enterobacteria phage ID11
310953	GCA_002539105.1	Enterobacteria phage ID11
310953	GCA_002542875.1	Enterobacteria phage ID11
310953	GCA_002540845.1	Enterobacteria phage ID11
310953	GCA_002538785.1	Enterobacteria phage ID11
310953	GCA_002540875.1	Enterobacteria phage ID11
310953	GCA_002540895.1	Enterobacteria phage ID11
310953	GCA_002544205.1	Enterobacteria phage ID11
310953	GCA_002538835.1	Enterobacteria phage ID11
310953	GCA_002542935.1	Enterobacteria phage ID11
310953	GCA_002540925.1	Enterobacteria phage ID11
310953	GCA_002544915.1	Enterobacteria phage ID11
310953	GCA_002536815.1	Enterobacteria phage ID11
310953	GCA_002538855.1	Enterobacteria phage ID11
310953	GCA_002540945.1	Enterobacteria phage ID11
310953	GCA_002542955.1	Enterobacteria phage ID11
310953	GCA_002538425.1	Enterobacteria phage ID11
310953	GCA_002538895.1	Enterobacteria phage ID11
310953	GCA_002542975.1	Enterobacteria phage ID11
310953	GCA_002536915.1	Enterobacteria phage ID11
310953	GCA_002540965.1	Enterobacteria phage ID11
310953	GCA_002543005.1	Enterobacteria phage ID11
310953	GCA_002536935.1	Enterobacteria phage ID11
310953	GCA_002538925.1	Enterobacteria phage ID11
310953	GCA_002544565.1	Enterobacteria phage ID11
310953	GCA_002540985.1	Enterobacteria phage ID11
310953	GCA_002544235.1	Enterobacteria phage ID11
310953	GCA_002536965.1	Enterobacteria phage ID11
310953	GCA_002538965.1	Enterobacteria phage ID11
310953	GCA_002543215.1	Enterobacteria phage ID11
310953	GCA_002543045.1	Enterobacteria phage ID11
310953	GCA_002541005.1	Enterobacteria phage ID11
310953	GCA_002538995.1	Enterobacteria phage ID11
310953	GCA_002543095.1	Enterobacteria phage ID11
310953	GCA_002541035.1	Enterobacteria phage ID11
310953	GCA_002536835.1	Enterobacteria phage ID11
310953	GCA_002539035.1	Enterobacteria phage ID11
310953	GCA_002541065.1	Enterobacteria phage ID11
310953	GCA_002539055.1	Enterobacteria phage ID11
310953	GCA_002541095.1	Enterobacteria phage ID11
310953	GCA_002545285.1	Enterobacteria phage ID11

310953	GCA_002544945.1	Enterobacteria phage ID11
310953	GCA_002545205.1	Enterobacteria phage ID11
310953	GCA_002537085.1	Enterobacteria phage ID11
310953	GCA_002539075.1	Enterobacteria phage ID11
310953	GCA_002541515.1	Enterobacteria phage ID11
310953	GCA_002543915.1	Enterobacteria phage ID11
310953	GCA_002541175.1	Enterobacteria phage ID11
310953	GCA_002545965.1	Enterobacteria phage ID11
310953	GCA_002543165.1	Enterobacteria phage ID11
310953	GCA_002543185.1	Enterobacteria phage ID11
310953	GCA_002545265.1	Enterobacteria phage ID11
310953	GCA_002537115.1	Enterobacteria phage ID11
310953	GCA_002539125.1	Enterobacteria phage ID11
310953	GCA_002539145.1	Enterobacteria phage ID11
310953	GCA_002543245.1	Enterobacteria phage ID11
310953	GCA_002537135.1	Enterobacteria phage ID11
310953	GCA_002537795.1	Enterobacteria phage ID11
310953	GCA_002545325.1	Enterobacteria phage ID11
310953	GCA_002539165.1	Enterobacteria phage ID11
310953	GCA_002544975.1	Enterobacteria phage ID11
310953	GCA_002542235.1	Enterobacteria phage ID11
310953	GCA_002541195.1	Enterobacteria phage ID11
310953	GCA_002543275.1	Enterobacteria phage ID11
310953	GCA_002544285.1	Enterobacteria phage ID11
310953	GCA_002539195.1	Enterobacteria phage ID11
310953	GCA_002541245.1	Enterobacteria phage ID11
310953	GCA_002537225.1	Enterobacteria phage ID11
310953	GCA_002539215.1	Enterobacteria phage ID11
310953	GCA_002541275.1	Enterobacteria phage ID11
310953	GCA_002544855.1	Enterobacteria phage ID11
310953	GCA_002543305.1	Enterobacteria phage ID11
310953	GCA_002537175.1	Enterobacteria phage ID11
310953	GCA_002543345.1	Enterobacteria phage ID11
310953	GCA_002537265.1	Enterobacteria phage ID11
310953	GCA_002545995.1	Enterobacteria phage ID11
310953	GCA_002539275.1	Enterobacteria phage ID11
310953	GCA_002541315.1	Enterobacteria phage ID11
310953	GCA_002539295.1	Enterobacteria phage ID11
310953	GCA_002543365.1	Enterobacteria phage ID11
310953	GCA_002544615.1	Enterobacteria phage ID11
310953	GCA_002412105.1	Enterobacteria phage ID11
310953	GCA_002545445.1	Enterobacteria phage ID11
310953	GCA_002544775.1	Enterobacteria phage ID11
310953	GCA_002537295.1	Enterobacteria phage ID11
310953	GCA_002541355.1	Enterobacteria phage ID11
310953	GCA_002538155.1	Enterobacteria phage ID11
310953	GCA_002543395.1	Enterobacteria phage ID11
310953	GCA_002537335.1	Enterobacteria phage ID11
310953	GCA_002539335.1	Enterobacteria phage ID11
310953	GCA_002543415.1	Enterobacteria phage ID11

310953	GCA_002537365.1	Enterobacteria phage ID11
310953	GCA_002541375.1	Enterobacteria phage ID11
310953	GCA_002536885.1	Enterobacteria phage ID11
310953	GCA_002539355.1	Enterobacteria phage ID11
310953	GCA_002543445.1	Enterobacteria phage ID11
310953	GCA_002541425.1	Enterobacteria phage ID11
310953	GCA_002545525.1	Enterobacteria phage ID11
310953	GCA_002546035.1	Enterobacteria phage ID11
310953	GCA_002539375.1	Enterobacteria phage ID11
310953	GCA_002541465.1	Enterobacteria phage ID11
310953	GCA_002545345.1	Enterobacteria phage ID11
310953	GCA_002539395.1	Enterobacteria phage ID11
310953	GCA_002545015.1	Enterobacteria phage ID11
310953	GCA_002543475.1	Enterobacteria phage ID11
310953	GCA_002545555.1	Enterobacteria phage ID11
310953	GCA_002544645.1	Enterobacteria phage ID11
310953	GCA_002544335.1	Enterobacteria phage ID11
310953	GCA_002539415.1	Enterobacteria phage ID11
310953	GCA_002543515.1	Enterobacteria phage ID11
310953	GCA_002539445.1	Enterobacteria phage ID11
310953	GCA_002537435.1	Enterobacteria phage ID11
310953	GCA_002545585.1	Enterobacteria phage ID11
310953	GCA_002539465.1	Enterobacteria phage ID11
310953	GCA_002543565.1	Enterobacteria phage ID11
310953	GCA_002545615.1	Enterobacteria phage ID11
310953	GCA_002539485.1	Enterobacteria phage ID11
310953	GCA_002541785.1	Enterobacteria phage ID11
310953	GCA_002541585.1	Enterobacteria phage ID11
310953	GCA_002537455.1	Enterobacteria phage ID11
310953	GCA_002545645.1	Enterobacteria phage ID11
310953	GCA_002539515.1	Enterobacteria phage ID11
310953	GCA_002537495.1	Enterobacteria phage ID11
310953	GCA_002541535.1	Enterobacteria phage ID11
310953	GCA_002539535.1	Enterobacteria phage ID11
310953	GCA_002545045.1	Enterobacteria phage ID11
310953	GCA_002543605.1	Enterobacteria phage ID11
310953	GCA_002537525.1	Enterobacteria phage ID11
310953	GCA_002541565.1	Enterobacteria phage ID11
310953	GCA_002545695.1	Enterobacteria phage ID11
310953	GCA_002539555.1	Enterobacteria phage ID11
310953	GCA_002407555.1	Enterobacteria phage ID11
310953	GCA_002545725.1	Enterobacteria phage ID11
310953	GCA_002539575.1	Enterobacteria phage ID11
310953	GCA_002543635.1	Enterobacteria phage ID11
338136	GCA_002598205.1	Enterobacteria phage ID12
384642	GCA_000867085.1	Enterobacteria phage ID18
511969	GCA_000864545.1	Enterobacteria phage ID2 Moscow/ID/2001
1273714	GCA_002755475.1	Enterobacteria phage ID204 Moscow/ID
338112	GCA_002596565.1	Enterobacteria phage ID22
338113	GCA_002596585.1	Enterobacteria phage ID34

338134	GCA_002598165.1	Enterobacteria phage ID41
338114	GCA_002596605.1	Enterobacteria phage ID45
338129	GCA_002598065.1	Enterobacteria phage ID8
10868	GCA_000836925.1	Enterobacteria phage If1
10867	GCA_000848985.1	Enterobacteria phage Ike
1414766	GCA_002604125.1	Enterobacteria phage IME_EC2
697227	GCA_000889095.1	Enterobacteria phage IME08
1090133	GCA_000903395.1	Enterobacteria phage IME10
1090133	GCA_002601845.1	Enterobacteria phage IME10
1536597	GCA_000927415.1	Enterobacteria phage J8-65
1610836	GCA_001503835.1	Enterobacteria phage JenK1
1610837	GCA_001503035.1	Enterobacteria phage JenP1
1610838	GCA_001504635.1	Enterobacteria phage JenP2
576790	GCA_000882975.1	Enterobacteria phage JS10
344021	GCA_000866705.1	Enterobacteria phage K1F
344021	GCA_002629985.1	Enterobacteria phage K1F
1651198	GCA_003606075.1	Enterobacteria phage Kha5h
1651203	GCA_003606095.1	Enterobacteria phage KhF1
1651204	GCA_003606115.1	Enterobacteria phage KhF2
1651200	GCA_003606135.1	Enterobacteria phage KhF3
12021	GCA_002755235.1	Enterobacteria phage KU1
318593	GCA_002600105.1	Enterobacteria phage L17
1235640	GCA_000902155.1	Enterobacteria phage M
1508658	GCA_002596525.1	Enterobacteria phage MED1
1150757	GCA_003423325.1	Enterobacteria phage mEp021
1147149	GCA_000902075.1	Enterobacteria phage mEp043 c-1
1147150	GCA_000903595.1	Enterobacteria phage mEp235
1147151	GCA_000901055.1	Enterobacteria phage mEp237
1147152	GCA_000902715.1	Enterobacteria phage mEp460
12022	GCA_000847485.1	Enterobacteria phage MS2
12022	GCA_002755255.1	Enterobacteria phage MS2
12022	GCA_002921435.1	Enterobacteria phage MS2
12022	GCA_002921445.1	Enterobacteria phage MS2
12022	GCA_002921455.1	Enterobacteria phage MS2
12022	GCA_004008725.1	Enterobacteria phage MS2
75723	GCA_000866345.1	Enterobacteria phage MX1
338115	GCA_002596625.1	Enterobacteria phage NC1
338137	GCA_002598225.1	Enterobacteria phage NC10
338118	GCA_002596685.1	Enterobacteria phage NC11
338132	GCA_002598125.1	Enterobacteria phage NC13
338119	GCA_002596705.1	Enterobacteria phage NC16
338133	GCA_002598145.1	Enterobacteria phage NC19
338131	GCA_002598105.1	Enterobacteria phage NC2
338109	GCA_002630345.1	Enterobacteria phage NC3
338120	GCA_002596725.1	Enterobacteria phage NC37
338121	GCA_002596745.1	Enterobacteria phage NC41
338116	GCA_002596645.1	Enterobacteria phage NC5
338122	GCA_002596765.1	Enterobacteria phage NC51
338123	GCA_002596785.1	Enterobacteria phage NC56
338138	GCA_002598005.1	Enterobacteria phage NC6

338117	GCA_002596665.1	Enterobacteria phage NC7
75725	GCA_002629905.1	Enterobacteria phage NL95
2282412	GCA_003423365.1	Enterobacteria phage O276
10680	GCA_000846325.1	Enterobacteria phage P4
10682	GCA_002755035.1	Enterobacteria phage P7
1567486	GCA_000930115.1	Enterobacteria phage P88
448384	GCA_000874225.1	Enterobacteria phage Phi1
10713	GCA_001015325.1	Enterobacteria phage phi80
948870	GCA_000914915.1	Enterobacteria phage phi92
451705	GCA_000871345.1	Enterobacteria phage phiEcoM-GJ1
1273706	GCA_002603025.1	Enterobacteria phage phiJLA23
103807	GCA_000847205.1	Enterobacteria phage phiP27
318594	GCA_002600125.1	Enterobacteria phage PR3
318595	GCA_002600145.1	Enterobacteria phage PR4
318596	GCA_002600165.1	Enterobacteria phage PR5
261665	GCA_002600085.1	Enterobacteria phage PR772
261665	GCA_002600185.1	Enterobacteria phage PR772
10658	GCA_000837025.1	Enterobacteria phage PRD1
69608	GCA_002758795.1	Enterobacteria phage RB10
10692	GCA_003369385.1	Enterobacteria phage RB18
69609	GCA_002149245.1	Enterobacteria phage RB27
134822	GCA_002630305.1	Enterobacteria phage RB33
50948	GCA_000840705.1	Enterobacteria phage RB49
1112578	GCA_002755135.1	Enterobacteria phage RB5
10693	GCA_000881275.1	Enterobacteria phage RB51
697289	GCA_002599685.1	Enterobacteria phage RB55
697290	GCA_002599705.1	Enterobacteria phage RB59
69610	GCA_002758815.1	Enterobacteria phage RB6
36339	GCA_002149445.1	Enterobacteria phage RB68
12353	GCA_000858005.1	Enterobacteria phage RB69
697291	GCA_002758855.1	Enterobacteria phage RB7
69612	GCA_002758835.1	Enterobacteria phage RB9
10844	GCA_002596485.1	Enterobacteria phage S13
10844	GCA_002596505.1	Enterobacteria phage S13
1805456	GCA_002601865.1	Enterobacteria phage SEGD1
1524881	GCA_001041875.1	Enterobacteria phage Sf101
1225789	GCA_001042255.1	Enterobacteria phage SfI
55884	GCA_000839125.1	Enterobacteria phage SfV
12027	GCA_000862845.1	Enterobacteria phage SP
10845	GCA_000884975.1	Enterobacteria phage St-1
221029	GCA_000846745.1	Enterobacteria phage ST104
10759	GCA_000841665.1	Enterobacteria phage T3
10759	GCA_002745435.1	Enterobacteria phage T3
857277	GCA_002599605.1	Enterobacteria phage T4T
10666	GCA_003423385.1	Enterobacteria phage T6
10760	GCA_000844825.1	Enterobacteria phage T7
10760	GCA_002600025.1	Enterobacteria phage T7
10760	GCA_002599965.1	Enterobacteria phage T7
10760	GCA_002599985.1	Enterobacteria phage T7
10760	GCA_002600005.1	Enterobacteria phage T7

10760	GCA_002600045.1	Enterobacteria phage T7
10760	GCA_002600065.1	Enterobacteria phage T7
10760	GCA_003059695.1	Enterobacteria phage T7
1075774	GCA_002755095.1	Enterobacteria phage T7M
876449	GCA_001746135.1	Enterobacteria phage UAB_Phi20
979726	GCA_000905795.1	Enterobacteria phage UAB_Phi78
2163887	GCA_003094115.1	Enterobacteria phage vB_EcoM_IME281
2163888	GCA_003094135.1	Enterobacteria phage vB_EcoM_IME338
2163889	GCA_003094155.1	Enterobacteria phage vB_EcoM_IME339
2163890	GCA_003094175.1	Enterobacteria phage vB_EcoM_IME340
2163891	GCA_003094195.1	Enterobacteria phage vB_EcoM_IME341
1567026	GCA_001505315.1	Enterobacteria phage vB_EcoM_VR5
1141139	GCA_000900475.1	Enterobacteria phage vB_EcoP_ACG-C91
2315471	GCA_003613975.1	Enterobacteria phage vB_EcoP_IME390
2163885	GCA_003094095.1	Enterobacteria phage vB_EcoS_IME167
2163886	GCA_003094075.1	Enterobacteria phage vB_EcoS_IME18
2496546	GCA_003094215.1	Enterobacteria phage vB_EcoS_IME347
2507711	GCA_004138795.1	Enterobacteria phage vB_EcoS_IME542
1852563	GCA_001744595.1	Enterobacteria phage vB_EcoS_NBD2
1147155	GCA_000901075.1	Enterobacteria phage vB_EcoS_Rogue1
1147094	GCA_000903335.1	Enterobacteria phage vB_KleM-RaK2
462299	GCA_004319605.1	Enterobacteria phage VT1-Sakai
936054	GCA_001470595.1	Enterobacteria phage VT2phi_272
97081	GCA_000844925.1	Enterobacteria phage VT2-Sakai
338125	GCA_002596825.1	Enterobacteria phage WA10
338126	GCA_002596845.1	Enterobacteria phage WA11
384643	GCA_000866265.1	Enterobacteria phage WA13
338140	GCA_002630365.1	Enterobacteria phage WA14
338127	GCA_002598025.1	Enterobacteria phage WA2
338128	GCA_002598045.1	Enterobacteria phage WA3
338124	GCA_002596805.1	Enterobacteria phage WA4
338130	GCA_002598085.1	Enterobacteria phage WA5
338135	GCA_002598185.1	Enterobacteria phage WA6
1651201	GCA_003606155.1	Enterobacteria phage XTG1
564886	GCA_000882275.1	Enterobacteria phage YYZ-2008
1147156	GCA_000902735.1	Enterobacterial phage mEp213
1147158	GCA_000903635.1	Enterobacterial phage mEp390
1197935	GCA_001041175.1	Enterobacteriaphage UAB_Phi87
1173135	GCA_000898895.1	Enterococcus phage BC611
1498168	GCA_001041015.1	Enterococcus phage ECP3
1647400	GCA_001754705.1	Enterococcus phage Ec-ZZ2
977801	GCA_001275495.1	Enterococcus phage EF62phi
1204540	GCA_000927555.1	Enterococcus phage EfaCPT1
627087	GCA_000882115.1	Enterococcus phage EFAP-1
1486428	GCA_000927435.1	Enterococcus phage EFC-1
1597976	GCA_001504255.1	Enterococcus phage EFDG1
1640885	GCA_001502015.1	Enterococcus phage EFLK1
1926594	GCA_002617385.1	Enterococcus phage EFP01
1958912	GCA_002619285.1	Enterococcus phage EF-P10
1932891	GCA_002618045.1	Enterococcus phage EF-P29

767806	GCA_000893315.1	Enterococcus phage EFRM31
2419745	GCA_004147165.1	Enterococcus phage EfsSzw-1
2419746	GCA_004147185.1	Enterococcus phage EfsWh-1
2315766	GCA_003668315.1	Enterococcus phage EfV12-phi1
2017580	GCA_900092395.1	Enterococcus phage Idefix
1416012	GCA_000917055.1	Enterococcus phage IME_EF3
1351735	GCA_002623265.1	Enterococcus phage IMEEF1
1432658	GCA_000916395.1	Enterococcus phage IME-EF4
1445858	GCA_000921115.1	Enterococcus phage IME-EFm1
1718158	GCA_001505575.1	Enterococcus phage IME-EFm5
2172042	GCA_003094235.1	Enterococcus phage LY0322
2233540	GCA_003307495.1	Enterococcus phage LY0323
2488858	GCA_004006795.1	Enterococcus phage Nonaheksakonda
663241	GCA_000886215.1	Enterococcus phage phiEf11
2218497	GCA_003231575.1	Enterococcus phage phiEF17H
442493	GCA_000872105.1	Enterococcus phage phiEF24C
947379	GCA_002630265.1	Enterococcus phage phiEF24C-P2
673832	GCA_000886175.1	Enterococcus phage phiFL1A
673833	GCA_002758755.1	Enterococcus phage phiFL1B
673834	GCA_002758775.1	Enterococcus phage phiFL1C
673835	GCA_000884575.1	Enterococcus phage phiFL2A
673836	GCA_002630645.1	Enterococcus phage phiFL2B
673837	GCA_000884555.1	Enterococcus phage phiFL3A
673838	GCA_002630665.1	Enterococcus phage phiFL3B
673839	GCA_000887035.1	Enterococcus phage phiFL4A
2218498	GCA_003231595.1	Enterococcus phage phiM1EF22
2050964	GCA_002957215.1	Enterococcus phage phiNASRA1
2030922	GCA_002629705.1	Enterococcus phage phiSHEF2
2030923	GCA_002629725.1	Enterococcus phage phiSHEF4
2030924	GCA_002629745.1	Enterococcus phage phiSHEF5
2070197	GCA_002958215.1	Enterococcus phage PMBT2
1871692	GCA_001744175.1	Enterococcus phage SANTOR1
1073766	GCA_002623285.1	Enterococcus phage SAP6
1161939	GCA_000929535.1	Enterococcus phage vB_Efae230P-4
1747288	GCA_001470835.1	Enterococcus phage vB_EfaP_IME195
1747288	GCA_001470835.2	Enterococcus phage vB_EfaP_IME195
1747351	GCA_002607875.1	Enterococcus phage vB_EfaP_IME199
2501743	GCA_004147145.1	Enterococcus phage vB_EfaP_Zip
2175688	GCA_003143335.1	Enterococcus phage vB_EfaS_AL2
2175687	GCA_003143315.1	Enterococcus phage vB_EfaS_AL3
1747289	GCA_001502215.1	Enterococcus phage vB_EfaS_IME196
1747326	GCA_001470055.1	Enterococcus phage vB_EfaS_IME197
1747326	GCA_001470055.2	Enterococcus phage vB_EfaS_IME197
1747287	GCA_001502835.1	Enterococcus phage vB_EfaS_IME198
2234084	GCA_003342455.1	Enterococcus phage vB_EfaS_LM99
2501744	GCA_004147125.1	Enterococcus phage vB_EfaS_Max
1458851	GCA_000920875.1	Enterococcus phage VD13
1458851	GCA_002604365.1	Enterococcus phage VD13
1813888	GCA_900078345.1	Enterococcus phage VFW
1813887	GCA_900078355.1	Enterococcus phage VPE25

1929150	GCA_002005035.1	Enterovirus AN12
42789	GCA_002816725.1	Enterovirus D68
12064	GCA_000863205.1	Enterovirus E
1330520	GCA_000907315.1	Enterovirus F
1849328	GCA_002088385.1	Enterovirus goat/JL14
1330521	GCA_000875085.1	Enterovirus J
1330521	GCA_000884595.1	Enterovirus J
1826059	GCA_001629865.1	Enterovirus SEV-gx
47681	GCA_000919855.1	Enterovirus sp.
432371	GCA_003330105.1	environmental halophage 1 AAJ-2005
1168811	GCA_003285305.1	environmental Halophage eHP-1
1168816	GCA_003286035.1	environmental Halophage eHP-14
1168820	GCA_003286135.1	environmental Halophage eHP-18
1168823	GCA_003286215.1	environmental Halophage eHP-20
1168829	GCA_003286275.1	environmental Halophage eHP-28
1168830	GCA_003285635.1	environmental Halophage eHP-29
1168832	GCA_003286295.1	environmental Halophage eHP-30
1168837	GCA_003285745.1	environmental Halophage eHP-35
1168839	GCA_003285805.1	environmental Halophage eHP-37
1168843	GCA_003286335.1	environmental Halophage eHP-40
1168844	GCA_003286355.1	environmental Halophage eHP-41
1168845	GCA_003286375.1	environmental Halophage eHP-42
1168852	GCA_003285915.1	environmental Halophage eHP-E5
239365	GCA_000853405.1	Enzootic nasal tumour virus of goats
1788456	GCA_002118945.1	Eothenomys eleusis hantavirus FUGV
943342	GCA_002827125.1	Eothenomys miletus hantavirus LX309
382962	GCA_002988065.1	Epichloe festucae virus 1
143921	GCA_000851645.1	Epinephelus tauvina nervous necrosis virus
166056	GCA_000901435.1	Epinotia aporema granulovirus
70600	GCA_000838965.1	Epiphyas postvittana nucleopolyhedrovirus
544686	GCA_000875525.1	Epirus cherry virus
100217	GCA_001448375.1	Epizootic haematopoietic necrosis virus
449133	GCA_000885335.1	Epizootic hemorrhagic disease virus (serotype 1 / strain Ne
40537	GCA_000841945.1	Epsilonpapillomavirus 1
2035399	GCA_004131205.1	Eptesicus fuscus gammaherpesvirus
1464071	GCA_002826905.1	Eptesicus serotinus papillomavirus 1
1464072	GCA_002826865.1	Eptesicus serotinus papillomavirus 2
1464073	GCA_003179535.1	Eptesicus serotinus papillomavirus 3
1329402	GCA_002354985.1	Eptesipox virus
10326	GCA_000844025.1	Equid alphaherpesvirus 1 (Equine herpesvirus 1)
80341	GCA_000921595.1	Equid alphaherpesvirus 3
10331	GCA_000846345.1	Equid alphaherpesvirus 4 (Equine herpesvirus 4)
39637	GCA_000894575.1	Equid alphaherpesvirus 8
55744	GCA_000883455.1	Equid alphaherpesvirus 9
12657	GCA_000843985.2	Equid gammaherpesvirus 2 (Equine herpesvirus 2)
10371	GCA_000929435.1	Equid gammaherpesvirus 5
46916	GCA_001714455.1	Equine adenovirus 1
67603	GCA_001271175.1	Equine adenovirus 2
11047	GCA_000860865.1	Equine arteritis virus
201490	GCA_002829385.1	Equine encephalosis virus

109270	GCA_000850365.1	Equine foamy virus
1416349	GCA_000922575.1	Equine hepacivirus JPN3/JAPAN/2013
2079554	GCA_004134265.1	Equine parvovirus H
1307803	GCA_000904655.1	Equine pegivirus 1
47000	GCA_000859925.1	Equine rhinitis A virus
47000	GCA_002816535.1	Equine rhinitis A virus
47001	GCA_000858325.1	Equine rhinitis B virus 1
31590	GCA_002660415.1	Equine rotavirus A
31590	GCA_002644835.1	Equine rotavirus A
31590	GCA_002647075.1	Equine rotavirus A
31590	GCA_002645055.1	Equine rotavirus A
31590	GCA_002667895.1	Equine rotavirus A
31590	GCA_002667915.1	Equine rotavirus A
31590	GCA_002667955.1	Equine rotavirus A
31590	GCA_002667975.1	Equine rotavirus A
31590	GCA_002667995.1	Equine rotavirus A
31590	GCA_002668035.1	Equine rotavirus A
31590	GCA_002668075.1	Equine rotavirus A
31590	GCA_002667935.1	Equine rotavirus A
329862	GCA_002833565.2	Equine torovirus
1163703	GCA_000920535.1	Equus asinus papillomavirus 1
333920	GCA_000866385.1	Equus caballus papillomavirus 1
526413	GCA_000882695.1	Equus caballus papillomavirus 2
940834	GCA_000897055.1	Equus caballus papillomavirus 3
1235428	GCA_000906495.1	Equus caballus papillomavirus 4
1235429	GCA_000904015.1	Equus caballus papillomavirus 5
1235427	GCA_000906795.1	Equus caballus papillomavirus 6
1235430	GCA_000904315.1	Equus caballus papillomavirus 7
1912759	GCA_001866975.1	Equus caballus papillomavirus 8
1891761	GCA_000898215.1	Equus caballus polyomavirus 1
638358	GCA_000884795.1	Eragrostis curvula streak virus
638358	GCA_002824945.1	Eragrostis curvula streak virus
1030595	GCA_000893655.1	Eragrostis minor streak virus
496807	GCA_000872685.1	Eragrostis streak virus
390443	GCA_000873285.1	Erectites yellow mosaic virus
447603	GCA_000874005.1	Erectites yellow mosaic virus satellite DNA beta
291590	GCA_000857185.1	Erethizon dorsatum papillomavirus 1
2268125	GCA_004134045.1	Erethizon dorsatum papillomavirus 2
2069322	GCA_004128715.1	Erigeron breviscapus amalgavirus 1
2069323	GCA_004128735.1	Erigeron breviscapus amalgavirus 2
445217	GCA_000880995.1	Erinaceus europaeus papillomavirus 1
307444	GCA_000926335.1	Erinnyis ello granulovirus
273810	GCA_002829345.1	Eriocheir sinensis reovirus
248062	GCA_001629985.1	Erve virus
418443	GCA_000867985.1	Erwinia amylovora phage Era103
2163633	GCA_003093935.2	Erwinia phage Cronus
2163633	GCA_003093935.1	Erwinia phage Cronus
2530027	GCA_004521655.1	Erwinia phage Derbicus
1429768	GCA_000914635.1	Erwinia phage Ea35-70
1429767	GCA_000917295.1	Erwinia phage Ea9-2

947843	GCA_000901315.1	Erwinia phage ENT90
2014586	GCA_002625345.1	Erwinia phage EtG
2014586	GCA_002625345.2	Erwinia phage EtG
2182346	GCA_003183745.1	Erwinia phage Faunus
1407608	GCA_000912295.1	Erwinia phage FE44
2282175	GCA_003340815.1	Erwinia phage LS-2018a
2267655	GCA_003345005.1	Erwinia phage Pavtok
1131315	GCA_000894335.1	Erwinia phage PEp14
925983	GCA_000901255.1	Erwinia phage phiEa100
925986	GCA_000890875.1	Erwinia phage phiEa104
925985	GCA_003047735.1	Erwinia phage phiEa1H
557393	GCA_000881995.1	Erwinia phage phiEa21-4
1564096	GCA_001041355.1	Erwinia phage phiEa2809
1401669	GCA_000914555.1	Erwinia phage PhiEaH1
1029988	GCA_000902915.1	Erwinia phage phiEaH2
2178928	GCA_003719115.1	Erwinia phage phiEaP-8
925984	GCA_000893415.1	Erwinia phage phiEt88
2530026	GCA_004521635.1	Erwinia phage Rebecca
2267654	GCA_003423405.1	Erwinia phage SunLIRen
2201424	GCA_003308555.1	Erwinia phage vB_EamM_Alexandra
1883370	GCA_001744335.2	Erwinia phage vB_EamM_Asesino
1883370	GCA_001744335.1	Erwinia phage vB_EamM_Asesino
2060126	GCA_002957735.1	Erwinia phage vB_EamM_Bosolaphorus
1883379	GCA_001744955.1	Erwinia phage vB_EamM_Caitlin
1883371	GCA_001743655.1	Erwinia phage vB_EamM_ChrisDB
1815986	GCA_002624325.1	Erwinia phage vB_EamM_Deimos-Minion
2060127	GCA_002957745.1	Erwinia phage vB_EamM_Desertfox
1883372	GCA_001744975.1	Erwinia phage vB_EamM_EarlPhillipIV
1883373	GCA_001745635.1	Erwinia phage vB_EamM_Huxley
2026081	GCA_002629065.1	Erwinia phage vB_EamM_Joad
1883374	GCA_001744315.1	Erwinia phage vB_EamM_Kwan
1883375	GCA_002758035.1	Erwinia phage vB_EamM_Machina
2060128	GCA_002957755.1	Erwinia phage vB_EamM_MadMel
2060129	GCA_002957765.1	Erwinia phage vB_EamM_Mortimer
1883376	GCA_002758055.1	Erwinia phage vB_EamM_Parshik
1883377	GCA_001743635.1	Erwinia phage vB_EamM_Phobos
1815987	GCA_002624345.1	Erwinia phage vB_EamM_RAY
2026080	GCA_002629045.1	Erwinia phage vB_EamM_RisingSun
1815988	GCA_002624365.1	Erwinia phage vB_EamM_Simmy50
1815989	GCA_002624385.1	Erwinia phage vB_EamM_Special G
1883378	GCA_002600675.1	Erwinia phage vB_EamM_Stratton
1983553	GCA_002955045.1	Erwinia phage vB_EamM_Y3
1958956	GCA_002619345.1	Erwinia phage vB_EamM_Yoloswag
2099338	GCA_003014175.1	Erwinia phage vB_EamM-Bue1
1051674	GCA_002624445.1	Erwinia phage vB_Eam-MM7
1051676	GCA_000903375.1	Erwinia phage vB_EamM-Y2
1852641	GCA_002211075.1	Erwinia phage vB_EamP_Frozen
1852643	GCA_002757715.1	Erwinia phage vB_EamP_Gutmeister
1852642	GCA_002757695.1	Erwinia phage vB_EamP_Rexella
1051673	GCA_000902475.1	Erwinia phage vB_EamP-L1

2070198	GCA_002958225.1	Erwinia phage vB_EamP-S2
1051675	GCA_000901855.1	Erwinia phage vB_EamP-S6
2267653	GCA_003344985.1	Erwinia phage Wellington
12152	GCA_000847445.1	Erysimum latent virus
2006938	GCA_002955325.1	Erysipelothrix phage phi1605
1675317	GCA_001551205.1	Erysipelothrix phage SE-1
1777015	GCA_001551165.1	Erysiphe cichoracearum alphaendornavirus
2052561	GCA_002937205.1	Erysiphe necator mitovirus 1
2052562	GCA_002937215.1	Erysiphe necator mitovirus 2
2052563	GCA_002937225.1	Erysiphe necator mitovirus 3
1913119	GCA_002615245.1	Erythrobacter phage vB_EliS_R6L
2169903	GCA_003033365.1	Erythrura gouldiae polyomavirus 1
1508671	GCA_002624465.1	Escherichia coli O157 typing phage 1
1508672	GCA_002604765.1	Escherichia coli O157 typing phage 10
1508673	GCA_002604785.1	Escherichia coli O157 typing phage 11
1508674	GCA_002604805.1	Escherichia coli O157 typing phage 12
1508678	GCA_002604825.1	Escherichia coli O157 typing phage 3
1508680	GCA_002604845.1	Escherichia coli O157 typing phage 5
1508681	GCA_002604865.1	Escherichia coli O157 typing phage 6
1508683	GCA_003329085.1	Escherichia coli O157 typing phage 8
1555202	GCA_000926855.1	Escherichia phage 121Q
1115653	GCA_002601985.1	Escherichia phage 1720a-02
1598146	GCA_001505715.1	Escherichia phage 172-1
1391428	GCA_000915095.1	Escherichia phage 4MG
1837842	GCA_001744055.1	Escherichia phage 64795_ec1
1216926	GCA_000901095.1	Escherichia phage ADB-2
2499909	GCA_004138735.1	Escherichia phage AnYang
2045361	GCA_002745275.1	Escherichia phage APC_JM3.2
1655305	GCA_001551685.1	Escherichia phage APCEc01
1655314	GCA_002606705.1	Escherichia phage APCEc02
1655307	GCA_002606685.1	Escherichia phage APCEc03
66711	GCA_001310115.1	Escherichia phage AR1
1983463	GCA_003051265.1	Escherichia phage ArgO145
1527519	GCA_000928035.1	Escherichia phage Av-05
2040288	GCA_002629805.1	Escherichia phage Ayreon
2060112	GCA_003364355.1	Escherichia phage B2
1458848	GCA_000922735.2	Escherichia phage Bp4
2488948	GCA_003865755.1	Escherichia phage BRET
1416027	GCA_002604205.1	Escherichia phage bV_EcoS_AHP24
2340716	GCA_003723295.1	Escherichia phage C1
1735565	GCA_002745315.1	Escherichia phage C119
2234093	GCA_003600665.1	Escherichia phage C130_2
2340717	GCA_003613675.1	Escherichia phage C5
1610828	GCA_001501655.1	Escherichia phage CAjan
1542094	GCA_003060545.1	Escherichia phage CEV1
1965365	GCA_002619885.1	Escherichia phage CF2
1527506	GCA_001041375.1	Escherichia phage CICC 80001
2267248	GCA_003341815.1	Escherichia phage CMSTMSU
665033	GCA_000884495.1	Escherichia phage D108
2502298	GCA_004521095.1	Escherichia phage D5505

2014434	GCA_002625325.1	Escherichia phage D6
2048061	GCA_002957145.1	Escherichia phage DTL
1495286	GCA_000918355.1	Escherichia phage e4/1c
1048207	GCA_000914935.1	Escherichia phage EB49
2099356	GCA_002997865.1	Escherichia phage Ebrios
2025815	GCA_003328705.1	Escherichia phage EC121
1258572	GCA_002617245.1	Escherichia phage EC1-UPM
1229757	GCA_001041415.1	Escherichia phage EC6
1852630	GCA_002610185.1	Escherichia phage ECA2
1604356	GCA_000900235.1	Escherichia phage ECBP1
1604355	GCA_000897795.1	Escherichia phage ECBP2
1498172	GCA_001040975.1	Escherichia phage ECBP5
1981499	GCA_002622545.1	Escherichia phage ECD7
1204521	GCA_000925795.1	Escherichia phage ECML-117
1204522	GCA_000925055.1	Escherichia phage ECML-134
1204523	GCA_000924955.1	Escherichia phage ECML-4
2175169	GCA_003308575.1	Escherichia phage Eco_BIFF
2015807	GCA_002625425.1	Escherichia phage ECP1
2083276	GCA_003004875.1	Escherichia phage EcS1
2419742	GCA_004146605.1	Escherichia phage EcSzw_1
2419741	GCA_004146545.1	Escherichia phage EcSzw-2
2419743	GCA_004146565.1	Escherichia phage EcWhh-1
2419744	GCA_004146585.1	Escherichia phage Ecwhy_1
2502307	GCA_004521115.1	Escherichia phage EdH4
2053563	GCA_002957255.1	Escherichia phage EG1
1527514	GCA_000922495.1	Escherichia phage EK99P-1
2060119	GCA_002957675.1	Escherichia phage EMCL318
1883200	GCA_001745495.1	Escherichia phage Envy
2070199	GCA_003288715.1	Escherichia phage EP335
2070200	GCA_003288695.1	Escherichia phage EP75
1881104	GCA_002611945.1	Escherichia phage ESCO13
1897495	GCA_002612725.1	Escherichia phage ESCO5
2053671	GCA_002957295.1	Escherichia phage FEC14
2315486	GCA_003613335.1	Escherichia phage FEC19
1446490	GCA_000919935.1	Escherichia phage FFH2
1131317	GCA_000903315.1	Escherichia phage FV3
1933113	GCA_002618605.1	Escherichia phage G AB-2017
2070178	GCA_002958035.1	Escherichia phage GER2
1883202	GCA_001746155.1	Escherichia phage Gluttony
2182345	GCA_003183765.1	Escherichia phage Gostya9
1883203	GCA_003329265.1	Escherichia phage Greed
2234092	GCA_003341015.1	Escherichia phage Halfdan
1147145	GCA_000902055.1	Escherichia phage HK446
432200	GCA_000901115.1	Escherichia phage HK542
432201	GCA_000902775.1	Escherichia phage HK544
1147142	GCA_000903555.1	Escherichia phage HK578
1147148	GCA_000902695.1	Escherichia phage HK629
1147146	GCA_000903575.1	Escherichia phage HK630
1147147	GCA_000901035.1	Escherichia phage HK633
906669	GCA_000893995.1	Escherichia phage HK639

906668	GCA_000894975.1	Escherichia phage HK75
1965367	GCA_002619925.1	Escherichia phage HP3
1237364	GCA_000901375.1	Escherichia phage HX01
1434323	GCA_001041535.1	Escherichia phage HY01
1527531	GCA_001504355.1	Escherichia phage HY02
1654926	GCA_001745795.1	Escherichia phage HY03
2079317	GCA_002958515.1	Escherichia phage HZ2R8
2530019	GCA_004338395.1	Escherichia phage HZP2
338101	GCA_002618885.1	Escherichia phage ID21
338102	GCA_002618945.1	Escherichia phage ID32
338108	GCA_002614425.1	Escherichia phage ID52
338107	GCA_002618965.1	Escherichia phage ID62
1054834	GCA_000902495.1	Escherichia phage ime09
1239384	GCA_000903115.1	Escherichia phage IME11
2041760	GCA_003601515.1	Escherichia phage IMM-002
1327956	GCA_000913575.1	Escherichia phage JES2013
1340750	GCA_001504375.1	Escherichia phage JH2
1147722	GCA_002633045.1	Escherichia phage JLK-2012
1772219	GCA_002608295.1	Escherichia phage JMPW1
1772218	GCA_002608255.1	Escherichia phage JMPW2
293178	GCA_000872205.1	Escherichia phage JS98
576789	GCA_000883895.1	Escherichia phage JSE
1897443	GCA_002612525.1	Escherichia phage JSS1
698487	GCA_001308535.1	Escherichia phage K1-dep(1)
698486	GCA_001308815.1	Escherichia phage K1-dep(4)
698489	GCA_002614465.1	Escherichia phage K1-ind(2)
698490	GCA_002630685.1	Escherichia phage K1-ind(3)
698488	GCA_002614445.1	Escherichia phage K1ind1
1041524	GCA_000891215.1	Escherichia phage K30
1436889	GCA_000917255.1	Escherichia phage KBNP1711
2218499	GCA_003764585.1	Escherichia phage KIT03
1871707	GCA_002709745.1	Escherichia phage KNP1
1933114	GCA_002618625.1	Escherichia phage L AB-2017
2065191	GCA_002957905.1	Escherichia phage LAMP
2315628	GCA_003575725.1	Escherichia phage LL11
2233993	GCA_003342355.1	Escherichia phage LL12
2301650	GCA_003575705.1	Escherichia phage LL2
2233992	GCA_003307595.1	Escherichia phage LL5
1788294	GCA_001881735.1	Escherichia phage LM33_P1
1883204	GCA_002757995.1	Escherichia phage Lust
1307804	GCA_000907595.1	Escherichia phage Lw1
1147157	GCA_000902115.1	Escherichia phage mEp234
1147153	GCA_000902095.1	Escherichia phage mEpX1
1147154	GCA_000903615.1	Escherichia phage mEpX2
489779	GCA_000872765.1	Escherichia phage Min27
2547246	GCA_004521615.1	Escherichia phage Minorna
2448909	GCA_003723015.1	Escherichia phage MLF4
1675606	GCA_002607105.1	Escherichia phage Murica
1837930	GCA_001884695.1	Escherichia phage MX01
2108117	GCA_003031055.1	Escherichia phage myPSH1131

2108113	GCA_003024145.1	Escherichia phage myPSH2311
2340718	GCA_003613695.1	Escherichia phage N13
2340719	GCA_003613655.1	Escherichia phage N30
2340720	GCA_003723275.1	Escherichia phage N7
338106	GCA_002618905.1	Escherichia phage NC28
338110	GCA_002618925.1	Escherichia phage NC29
338103	GCA_002618865.1	Escherichia phage NC35
1237159	GCA_000899435.1	Escherichia phage NJ01
2448910	GCA_003723055.1	Escherichia phage OLB145
2448911	GCA_003723035.1	Escherichia phage OLB35
2020879	GCA_002627205.1	Escherichia phage OSYSP
1933115	GCA_002618645.1	Escherichia phage P AB-2017
2479933	GCA_003691835.1	Escherichia phage p000v
2479934	GCA_003691855.1	Escherichia phage p000y
1451116	GCA_003146945.1	Escherichia phage P13353
1451077	GCA_003146965.1	Escherichia phage P13357
1451074	GCA_002604325.1	Escherichia phage P13363
1451075	GCA_003146925.1	Escherichia phage P13368
1150869	GCA_000900315.1	Escherichia phage P13374
1429232	GCA_003146885.1	Escherichia phage P13771
1429230	GCA_003146845.1	Escherichia phage P13803
1429233	GCA_003146905.1	Escherichia phage P14437
1572753	GCA_001503655.1	Escherichia phage P483
1572754	GCA_001504455.1	Escherichia phage P694
1429231	GCA_003146865.1	Escherichia phage P8983
1660361	GCA_002594725.1	Escherichia phage PA11
1660362	GCA_002595025.1	Escherichia phage PA12
1660363	GCA_002594745.1	Escherichia phage PA16
1660364	GCA_002594765.1	Escherichia phage PA18
1660365	GCA_001447065.1	Escherichia phage PA2
1660366	GCA_002594785.1	Escherichia phage PA21
1660367	GCA_002594805.1	Escherichia phage PA27
1660368	GCA_002622525.1	Escherichia phage PA28
1660369	GCA_002594825.1	Escherichia phage PA29
1660370	GCA_002594845.1	Escherichia phage PA30
1660371	GCA_002594865.1	Escherichia phage PA32
1660372	GCA_002594885.1	Escherichia phage PA33
1660373	GCA_002594905.1	Escherichia phage PA36
1660374	GCA_002594685.1	Escherichia phage PA4
1660375	GCA_002594925.1	Escherichia phage PA42
1660376	GCA_002594945.1	Escherichia phage PA44
1660377	GCA_002594965.1	Escherichia phage PA45
1660378	GCA_002594705.1	Escherichia phage PA5
1660379	GCA_002594985.1	Escherichia phage PA50
1660380	GCA_002595045.1	Escherichia phage PA51
1660381	GCA_002595005.1	Escherichia phage PA52
1660382	GCA_002606765.1	Escherichia phage PA8
1273738	GCA_001041035.1	Escherichia phage PBECO 4
2316016	GCA_003575305.1	Escherichia phage PD38
2099658	GCA_002997845.1	Escherichia phage PDX

1498170	GCA_000923095.1	Escherichia phage PE3-1
1837875	GCA_002609745.1	Escherichia phage PE37
1647412	GCA_002604245.1	Escherichia phage PEC04
2315696	GCA_003575565.1	Escherichia phage PGN829.1
2047782	GCA_002956205.1	Escherichia phage PGT2
1229753	GCA_000906475.1	Escherichia phage phAPEC8
926589	GCA_000902355.1	Escherichia phage PhaxI
2530035	GCA_004340085.1	Escherichia phage PHB10
2530036	GCA_004340065.1	Escherichia phage PHB11
2530037	GCA_004521495.1	Escherichia phage PHB12
2234086	GCA_003307555.1	Escherichia phage phi G17
1542095	GCA_003060565.1	Escherichia phage Phi05_1387 B
1542096	GCA_003060585.1	Escherichia phage Phi05_1999 B
1542097	GCA_003060605.1	Escherichia phage Phi05_2388 B
1542098	GCA_003060625.1	Escherichia phage Phi06_2974 B
1542099	GCA_003060645.1	Escherichia phage Phi06_2987 S
1458706	GCA_001470555.1	Escherichia phage phi191
1774508	GCA_003147405.1	Escherichia phage phi467
1970776	GCA_002621185.1	Escherichia phage phiC120
1965366	GCA_002619905.1	Escherichia phage phiEC1
2507234	GCA_001504835.1	Escherichia phage phiK
1141519	GCA_000901815.1	Escherichia phage phiKT
1965465	GCA_002620345.1	Escherichia phage phiLLS
1777071	GCA_002609085.1	Escherichia phage phiON-2011
343516	GCA_000866205.1	Escherichia phage phiV10
2079259	GCA_002958365.1	Escherichia phage PMBT57
1540097	GCA_001042195.1	Escherichia phage Pollock
2060720	GCA_003094415.1	Escherichia phage PP01
1883205	GCA_002758015.1	Escherichia phage Pride
1649239	GCA_001501155.1	Escherichia phage pro147
1649240	GCA_001500495.1	Escherichia phage pro483
1673871	GCA_001501135.1	Escherichia phage QL01
1759531	GCA_002608155.1	Escherichia phage Rac-SA53
31533	GCA_002149625.1	Escherichia phage RB3
1590550	GCA_003146805.1	Escherichia phage RCS47
2045372	GCA_002990885.1	Escherichia phage SECphi17
2045373	GCA_002990915.1	Escherichia phage SECphi18
2496550	GCA_002990945.1	Escherichia phage SECphi27
1540098	GCA_002149205.1	Escherichia phage Seurat
2234080	GCA_003308515.1	Escherichia phage SF
2108115	GCA_003085755.1	Escherichia phage SH2026Stx1
2488654	GCA_003865675.1	Escherichia phage Skarpretter
1883206	GCA_003329505.1	Escherichia phage Sloth
1720493	GCA_001502275.1	Escherichia phage slur01
1720494	GCA_001501495.1	Escherichia phage slur02
1720496	GCA_003147245.1	Escherichia phage slur03
1720497	GCA_003147265.1	Escherichia phage slur04
1720498	GCA_001500835.1	Escherichia phage slur05
1720499	GCA_003147285.1	Escherichia phage slur06
1720500	GCA_001502875.1	Escherichia phage slur07

1720501	GCA_003147305.1	Escherichia phage slur08
1728958	GCA_001504595.1	Escherichia phage slur09
1720502	GCA_003147325.1	Escherichia phage slur11
1720503	GCA_003147345.1	Escherichia phage slur12
1720505	GCA_003147365.1	Escherichia phage slur13
1720504	GCA_001447045.1	Escherichia phage slur14
1720495	GCA_001433645.1	Escherichia phage slur16
1720506	GCA_003147385.1	Escherichia phage slur17
2071659	GCA_003958845.1	Escherichia phage sp.
2071659	GCA_003958705.1	Escherichia phage sp.
2071659	GCA_003958725.1	Escherichia phage sp.
2071659	GCA_003958745.1	Escherichia phage sp.
2071659	GCA_003958765.1	Escherichia phage sp.
2071659	GCA_003958905.1	Escherichia phage sp.
2071659	GCA_003967255.1	Escherichia phage sp.
2268589	GCA_003341035.1	Escherichia phage SRT7
2268589	GCA_003341035.2	Escherichia phage SRT7
2496545	GCA_002744055.1	Escherichia phage SRT8
2005047	GCA_002624805.1	Escherichia phage ST0
2047765	GCA_002956185.1	Escherichia phage St11Ph5
2569975	GCA_002625065.1	Escherichia phage ST20
1983789	GCA_002624065.1	Escherichia phage ST31
2005048	GCA_002624825.1	Escherichia phage ST32
2005048	GCA_002624825.2	Escherichia phage ST32
194949	GCA_002221805.1	Escherichia phage Stx2 II
1718606	GCA_001500855.1	Escherichia phage SUSP1
1718606	GCA_001500855.2	Escherichia phage SUSP1
1718669	GCA_001502895.2	Escherichia phage SUSP2
1718669	GCA_001502895.1	Escherichia phage SUSP2
2492962	GCA_004008695.1	Escherichia phage T1
2060721	GCA_003094435.1	Escherichia phage T2
2060721	GCA_003613935.1	Escherichia phage T2
1124654	GCA_000903215.1	Escherichia phage TL-2011b
1124655	GCA_000900675.1	Escherichia phage TL-2011c
2268588	GCA_003341455.1	Escherichia phage UB
1837867	GCA_001743935.1	Escherichia phage UFV-AREG1
1916098	GCA_002615565.1	Escherichia phage Utah
1981500	GCA_002622565.1	Escherichia phage V18
399183	GCA_000875465.1	Escherichia phage V5
1141140	GCA_000900515.1	Escherichia phage vB_Eco_ACG-M12
2029169	GCA_002990855.1	Escherichia phage vB_Eco_SLUR25
2029173	GCA_900303305.1	Escherichia phage vB_Eco_SLUR26
2029170	GCA_900303335.1	Escherichia phage vB_Eco_SLUR63
2029171	GCA_900303325.1	Escherichia phage vB_Eco_SLUR75
2029172	GCA_900303315.1	Escherichia phage vB_Eco_SLUR76
1874688	GCA_900094095.1	Escherichia phage vB_Eco_slurp01
2496549	GCA_900178515.1	Escherichia phage vB_Eco_swan01
2268611	GCA_003861755.1	Escherichia phage vB_EcoM Sa157lw
2500761	GCA_004015845.1	Escherichia phage vB_EcoM_005
1495285	GCA_000918255.1	Escherichia phage vB_EcoM_112

1141141	GCA_000899615.1	Escherichia phage vB_EcoM_ACG-C40
1873990	GCA_001745015.1	Escherichia phage vB_EcoM_Alf5
1636202	GCA_001502055.1	Escherichia phage vB_EcoM_AYO145A
2420240	GCA_003719095.1	Escherichia phage vB_EcoM_DalCa
669875	GCA_001310155.1	Escherichia phage vB_EcoM_ECO1230-10
1970797	GCA_002621265.1	Escherichia phage vB_EcoM_ECOO78
2502300	GCA_004521255.1	Escherichia phage vB_EcoM_G10400
2502408	GCA_004521135.1	Escherichia phage vB_EcoM_G17
2502297	GCA_004521075.1	Escherichia phage vB_EcoM_G2133
2502410	GCA_004521155.1	Escherichia phage vB_EcoM_G2248
2502414	GCA_004521175.1	Escherichia phage vB_EcoM_G2285
2502415	GCA_004521195.1	Escherichia phage vB_EcoM_G2469
2502411	GCA_004521215.1	Escherichia phage vB_EcoM_G2494
2502299	GCA_004521235.1	Escherichia phage vB_EcoM_G2540
2502304	GCA_004521395.1	Escherichia phage vB_EcoM_G2540-3
2502302	GCA_004521315.1	Escherichia phage vB_EcoM_G29
2502412	GCA_004521335.1	Escherichia phage vB_EcoM_G37-3
2502301	GCA_004521295.1	Escherichia phage vB_EcoM_G4498
2502305	GCA_004521415.1	Escherichia phage vB_EcoM_G4500
2502306	GCA_004521435.1	Escherichia phage vB_EcoM_G4507
2502303	GCA_004521355.1	Escherichia phage vB_EcoM_G50
2502413	GCA_004521455.1	Escherichia phage vB_EcoM_G5211
2502416	GCA_004521375.1	Escherichia phage vB_EcoM_G53
2502409	GCA_004521275.1	Escherichia phage vB_EcoM_Goslar
2301649	GCA_003575825.1	Escherichia phage vB_EcoM_IME392
2234085	GCA_003340995.1	Escherichia phage vB_EcoM_JB75
1430444	GCA_000919915.2	Escherichia phage vB_EcoM_JS09
2184698	GCA_003177195.1	Escherichia phage vB_EcoM_NBG1
2184699	GCA_003177215.1	Escherichia phage vB_EcoM_NBG2
1391224	GCA_000926115.1	Escherichia phage vB_EcoM_PhAPEC2
2041347	GCA_002743975.1	Escherichia phage vB_EcoM_PHB05
2483417	GCA_003723315.1	Escherichia phage vB_EcoM_Sa157lw
1567027	GCA_001503695.1	Escherichia phage vB_EcoM_VR20
1567028	GCA_001504495.1	Escherichia phage vB_EcoM_VR25
1567029	GCA_001505955.1	Escherichia phage vB_EcoM_VR26
700939	GCA_000890395.1	Escherichia phage vB_EcoM_VR7
1541883	GCA_000925835.1	Escherichia phage vB_EcoM-ep3
2079315	GCA_002958495.1	Escherichia phage vB_EcoM-fFiEco06
2079316	GCA_002958505.1	Escherichia phage vB_EcoM-fHoEco02
2081604	GCA_004139175.1	Escherichia phage vB_EcoM-G28
2306966	GCA_003575285.1	Escherichia phage vB_EcoM-Pr121LW
1815590	GCA_001744235.1	Escherichia phage vB_EcoM-UFV13
1555238	GCA_001041835.1	Escherichia phage vB_EcoM-VpaE1
866553	GCA_001308415.1	Escherichia phage vB_EcoP_24B
1933107	GCA_002618485.1	Escherichia phage vB_EcoP_B
1933108	GCA_002618505.1	Escherichia phage vB_EcoP_C
1933109	GCA_002618525.1	Escherichia phage vB_EcoP_D
1933110	GCA_002618545.1	Escherichia phage vB_EcoP_F
1054461	GCA_000891295.1	Escherichia phage vB_EcoP_G7C
1755695	GCA_001882295.1	Escherichia phage vB_EcoP_GA2A

1933111	GCA_002618565.1	Escherichia phage vB_EcoP_K
1395983	GCA_000922515.1	Escherichia phage vB_EcoP_PhAPEC5
1391223	GCA_000924215.1	Escherichia phage vB_EcoP_PhAPEC7
1933112	GCA_002618585.1	Escherichia phage vB_EcoP_R
2233775	GCA_003307575.1	Escherichia phage vB_EcoP_S523
1519788	GCA_001042235.1	Escherichia phage vB_EcoP_SU10
2498616	GCA_003994835.1	Escherichia phage vB_EcoP-Ro45lw
2126819	GCA_003014935.1	Escherichia phage vB_EcoS Sa179lw
1416028	GCA_000921675.1	Escherichia phage vB_EcoS_AHP42
1416030	GCA_000921635.1	Escherichia phage vB_EcoS_AHS24
1416031	GCA_000924195.1	Escherichia phage vB_EcoS_AKS96
1933774	GCA_002618785.1	Escherichia phage vB_Ecos_CEB_EC3a
2496547	GCA_002619785.1	Escherichia phage vB_EcoS_ESCO41
1446489	GCA_000920795.1	Escherichia phage vB_EcoS_FFH1
2488596	GCA_003865875.1	Escherichia phage vB_EcoS_PNS1
1983554	GCA_002623665.1	Escherichia phage vB_EcoS_SH2
2026130	GCA_003991625.1	Escherichia phage vB_EcoS-95
2047801	GCA_002956225.1	Escherichia phage VB_EcoS-Golestan
1933412	GCA_002618665.1	Escherichia phage vB_EcoS-IME253
2079541	GCA_004146625.1	Escherichia phage vB_EcoS-Ro145clw
2315527	GCA_003613295.1	Escherichia phage vB_vPM_PD06
2315580	GCA_003613315.1	Escherichia phage vB_vPM_PD112
2316019	GCA_003575325.1	Escherichia phage vB_vPM_PD114
338105	GCA_002618845.1	Escherichia phage WA45
1837931	GCA_001882255.1	Escherichia phage WG01
1054480	GCA_000900835.1	Escherichia phage wV7
576791	GCA_000886395.1	Escherichia phage wV8
1567004	GCA_001041055.1	Escherichia phage YD-2008.s
2056767	GCA_002959395.1	Escherichia phage YDC107_1
2056768	GCA_002959415.1	Escherichia phage YDC107_2
1932889	GCA_002618005.1	Escherichia phage YUEEL01
2079534	GCA_002958915.1	Escherichia phage YZ1
2530021	GCA_004340425.1	Escherichia phage ZCEC5
1897641	GCA_002613645.2	Escherichia phage ZG49
1897641	GCA_002613645.1	Escherichia phage ZG49
194948	GCA_002221785.1	Escherichia Stx1 converting phage
1506453	GCA_002594665.1	Escherichia Stx1-converting recombinant phage HUN/2013
29252	GCA_001500715.1	Escherichia virus 186
1873778	GCA_002611705.1	Escherichia virus AAPEc6
1112008	GCA_000894655.1	Escherichia virus AKFV33
329853	GCA_002921465.1	Escherichia virus BZ13
329853	GCA_002921475.1	Escherichia virus BZ13
329853	GCA_002921485.1	Escherichia virus BZ13
1987159	GCA_000895155.1	Escherichia virus CBA120
1567006	GCA_001042135.1	Escherichia virus DT57C
491003	GCA_000872825.1	Escherichia virus EPS7
39804	GCA_001502735.1	Escherichia virus FI
39804	GCA_002921515.1	Escherichia virus FI
39804	GCA_002921505.1	Escherichia virus FI
39804	GCA_002921525.1	Escherichia virus FI

39804	GCA_002921495.1	Escherichia virus FI
352889	GCA_002814475.1	Escherichia virus H8
10742	GCA_000836965.1	Escherichia virus HK022
37554	GCA_000848825.1	Escherichia virus HK97
10869	GCA_000847885.1	Escherichia virus I22
1920998	GCA_000865785.1	Escherichia virus Jk06
1245890	GCA_000900575.1	Escherichia virus JL1
187764	GCA_000869785.1	Escherichia virus K1-5
344022	GCA_000866045.1	Escherichia virus K1E
344022	GCA_002922415.1	Escherichia virus K1E
2250214	GCA_003345025.1	Escherichia virus KFS-EC
1920999	GCA_002743515.1	Escherichia virus KP26
10710	GCA_000840245.1	Escherichia virus Lambda
10710	GCA_002745415.1	Escherichia virus Lambda
1977402	GCA_000845205.1	Escherichia virus M13
1977402	GCA_002745915.1	Escherichia virus M13
329852	GCA_002921425.1	Escherichia virus MS2
329852	GCA_900197495.1	Escherichia virus MS2
10677	GCA_000837225.1	Escherichia virus Mu
2047761	GCA_002956175.1	Escherichia virus mutPK1A2
40631	GCA_000839625.1	Escherichia virus N15
10752	GCA_000867865.1	Escherichia virus N4
10678	GCA_000844165.1	Escherichia virus P1
10678	GCA_002745375.1	Escherichia virus P1
10678	GCA_003628075.1	Escherichia virus P1
10678	GCA_003628095.1	Escherichia virus P1
10679	GCA_000836905.1	Escherichia virus P2
10679	GCA_002601305.1	Escherichia virus P2
490103	GCA_000879095.1	Escherichia virus phiEco32
10847	GCA_000819615.1	Escherichia virus phiX174
10847	GCA_002586235.1	Escherichia virus phiX174
10847	GCA_002585775.1	Escherichia virus phiX174
10847	GCA_002585855.1	Escherichia virus phiX174
10847	GCA_002585885.1	Escherichia virus phiX174
10847	GCA_002586565.1	Escherichia virus phiX174
10847	GCA_002585325.1	Escherichia virus phiX174
10847	GCA_002587175.1	Escherichia virus phiX174
10847	GCA_002585965.1	Escherichia virus phiX174
10847	GCA_002585995.1	Escherichia virus phiX174
10847	GCA_002586025.1	Escherichia virus phiX174
10847	GCA_002585355.1	Escherichia virus phiX174
10847	GCA_002586055.1	Escherichia virus phiX174
10847	GCA_002586095.1	Escherichia virus phiX174
10847	GCA_002586295.1	Escherichia virus phiX174
10847	GCA_002586125.1	Escherichia virus phiX174
10847	GCA_002585705.1	Escherichia virus phiX174
10847	GCA_002585655.1	Escherichia virus phiX174
10847	GCA_002586155.1	Escherichia virus phiX174
10847	GCA_002585445.1	Escherichia virus phiX174
10847	GCA_002586185.1	Escherichia virus phiX174

10847	GCA_002586205.1	Escherichia virus phiX174
10847	GCA_002585405.1	Escherichia virus phiX174
10847	GCA_002586925.1	Escherichia virus phiX174
10847	GCA_002586365.1	Escherichia virus phiX174
10847	GCA_002586385.1	Escherichia virus phiX174
10847	GCA_002585745.1	Escherichia virus phiX174
10847	GCA_002586405.1	Escherichia virus phiX174
10847	GCA_002586425.1	Escherichia virus phiX174
10847	GCA_002586455.1	Escherichia virus phiX174
10847	GCA_002586505.1	Escherichia virus phiX174
10847	GCA_002586525.1	Escherichia virus phiX174
10847	GCA_002586545.1	Escherichia virus phiX174
10847	GCA_002585625.1	Escherichia virus phiX174
10847	GCA_002586585.1	Escherichia virus phiX174
10847	GCA_002586605.1	Escherichia virus phiX174
10847	GCA_002585215.1	Escherichia virus phiX174
10847	GCA_002586625.1	Escherichia virus phiX174
10847	GCA_002586985.1	Escherichia virus phiX174
10847	GCA_002586645.1	Escherichia virus phiX174
10847	GCA_002586665.1	Escherichia virus phiX174
10847	GCA_002586685.1	Escherichia virus phiX174
10847	GCA_002586705.1	Escherichia virus phiX174
10847	GCA_002586725.1	Escherichia virus phiX174
10847	GCA_002585505.1	Escherichia virus phiX174
10847	GCA_002586765.1	Escherichia virus phiX174
10847	GCA_002586785.1	Escherichia virus phiX174
10847	GCA_002586805.1	Escherichia virus phiX174
10847	GCA_002586825.1	Escherichia virus phiX174
10847	GCA_002587025.1	Escherichia virus phiX174
10847	GCA_002586845.1	Escherichia virus phiX174
10847	GCA_002586865.1	Escherichia virus phiX174
10847	GCA_002586885.1	Escherichia virus phiX174
10847	GCA_002586325.1	Escherichia virus phiX174
10847	GCA_002586905.1	Escherichia virus phiX174
10847	GCA_002587345.1	Escherichia virus phiX174
10847	GCA_002586485.1	Escherichia virus phiX174
10847	GCA_002587045.1	Escherichia virus phiX174
10847	GCA_002585555.1	Escherichia virus phiX174
10847	GCA_002587115.1	Escherichia virus phiX174
10847	GCA_002585285.1	Escherichia virus phiX174
10847	GCA_002587145.1	Escherichia virus phiX174
10847	GCA_002586965.1	Escherichia virus phiX174
10847	GCA_002587085.1	Escherichia virus phiX174
10847	GCA_002587205.1	Escherichia virus phiX174
10847	GCA_002587245.1	Escherichia virus phiX174
10847	GCA_002587265.1	Escherichia virus phiX174
10847	GCA_002585585.1	Escherichia virus phiX174
10847	GCA_002587285.1	Escherichia virus phiX174
10847	GCA_002585255.1	Escherichia virus phiX174
10847	GCA_002585465.1	Escherichia virus phiX174

10847	GCA_002587305.1	Escherichia virus phiX174
10847	GCA_002587325.1	Escherichia virus phiX174
10847	GCA_002585915.1	Escherichia virus phiX174
10847	GCA_002585185.1	Escherichia virus phiX174
10847	GCA_002587365.1	Escherichia virus phiX174
10847	GCA_002587385.1	Escherichia virus phiX174
10847	GCA_002587405.1	Escherichia virus phiX174
10847	GCA_002587425.1	Escherichia virus phiX174
10847	GCA_002587465.1	Escherichia virus phiX174
10847	GCA_002587485.1	Escherichia virus phiX174
10847	GCA_002587505.1	Escherichia virus phiX174
10847	GCA_002587565.1	Escherichia virus phiX174
10847	GCA_002587445.1	Escherichia virus phiX174
10847	GCA_002587585.1	Escherichia virus phiX174
10847	GCA_002587625.1	Escherichia virus phiX174
10847	GCA_002587665.1	Escherichia virus phiX174
10847	GCA_002587725.1	Escherichia virus phiX174
10847	GCA_002587745.1	Escherichia virus phiX174
10847	GCA_002587765.1	Escherichia virus phiX174
10847	GCA_002587785.1	Escherichia virus phiX174
10847	GCA_002587805.1	Escherichia virus phiX174
10847	GCA_002587895.1	Escherichia virus phiX174
10847	GCA_002587925.1	Escherichia virus phiX174
10847	GCA_002587945.1	Escherichia virus phiX174
10847	GCA_002587965.1	Escherichia virus phiX174
10847	GCA_002587985.1	Escherichia virus phiX174
10847	GCA_002587825.1	Escherichia virus phiX174
10847	GCA_002588005.1	Escherichia virus phiX174
10847	GCA_002588025.1	Escherichia virus phiX174
10847	GCA_002588045.1	Escherichia virus phiX174
10847	GCA_002588065.1	Escherichia virus phiX174
10847	GCA_002588085.1	Escherichia virus phiX174
10847	GCA_002587845.1	Escherichia virus phiX174
10847	GCA_002588105.1	Escherichia virus phiX174
10847	GCA_002587525.1	Escherichia virus phiX174
10847	GCA_002588165.1	Escherichia virus phiX174
10847	GCA_002588185.1	Escherichia virus phiX174
10847	GCA_002587545.1	Escherichia virus phiX174
10847	GCA_002588245.1	Escherichia virus phiX174
10847	GCA_002588305.1	Escherichia virus phiX174
10847	GCA_002587865.1	Escherichia virus phiX174
10847	GCA_002588325.1	Escherichia virus phiX174
10847	GCA_002588395.1	Escherichia virus phiX174
10847	GCA_002588445.1	Escherichia virus phiX174
10847	GCA_002588465.1	Escherichia virus phiX174
10847	GCA_002588495.1	Escherichia virus phiX174
10847	GCA_002588525.1	Escherichia virus phiX174
10847	GCA_002588605.1	Escherichia virus phiX174
10847	GCA_002588645.1	Escherichia virus phiX174
10847	GCA_002588675.1	Escherichia virus phiX174

10847	GCA_002587605.1	Escherichia virus phiX174
10847	GCA_002588715.1	Escherichia virus phiX174
10847	GCA_002588755.1	Escherichia virus phiX174
10847	GCA_002588795.1	Escherichia virus phiX174
10847	GCA_002588815.1	Escherichia virus phiX174
10847	GCA_002588855.1	Escherichia virus phiX174
10847	GCA_002587645.1	Escherichia virus phiX174
10847	GCA_002587685.1	Escherichia virus phiX174
10847	GCA_002587705.1	Escherichia virus phiX174
10847	GCA_003342615.1	Escherichia virus phiX174
10847	GCA_003342635.1	Escherichia virus phiX174
10847	GCA_003342595.1	Escherichia virus phiX174
39803	GCA_002630205.1	Escherichia virus Qbeta
39803	GCA_002630185.1	Escherichia virus Qbeta
39803	GCA_002630105.1	Escherichia virus Qbeta
39803	GCA_002630125.1	Escherichia virus Qbeta
39803	GCA_002630145.1	Escherichia virus Qbeta
39803	GCA_002630165.1	Escherichia virus Qbeta
1914209	GCA_000884775.1	Escherichia virus RB14
329381	GCA_000889235.1	Escherichia virus RB16
45406	GCA_000870165.1	Escherichia virus RB32
115991	GCA_000863505.1	Escherichia virus RB43
115991	GCA_003047815.1	Escherichia virus RB43
115991	GCA_003047855.1	Escherichia virus RB43
355246	GCA_000865245.1	Escherichia virus Rtp
624134	GCA_000881155.1	Escherichia virus SSL2009a
1921008	GCA_000845005.1	Escherichia virus T1
10665	GCA_000836945.1	Escherichia virus T4
10665	GCA_002599665.1	Escherichia virus T4
10665	GCA_002599625.1	Escherichia virus T4
10665	GCA_002599645.1	Escherichia virus T4
10726	GCA_000858785.1	Escherichia virus T5
10726	GCA_002921405.1	Escherichia virus T5
10726	GCA_002921415.1	Escherichia virus T5
245685	GCA_000871665.1	Escherichia virus TLS
2282173	GCA_003368725.1	Escherichia virus Vec13
2053697	GCA_002957385.1	Escherichia virus VEc3
103216	GCA_001504035.1	Escherichia virus Wphi
2072209	GCA_004130435.1	Esparto virus
1127767	GCA_000895095.1	Espirito Santo virus
197771	GCA_000841045.1	Etapapillomavirus 1
1862978	GCA_001678455.1	Etheostoma fonticola aquareovirus
1538549	GCA_000921715.1	Ethiopian tobacco bushy top virus
1538550	GCA_000924235.1	Ethiopian tobacco bushy top virus satellite RNA
40056	GCA_002829405.1	Eubenangee virus
2170148	GCA_000973435.1	Eulipotyphla protoparvovirus 1
2013968	GCA_002219625.1	Euonymus yellow vein virus
515444	GCA_000872905.1	Eupatorium vein clearing virus
221054	GCA_000846485.1	Eupatorium yellow vein betasatellite
2010319	GCA_002830065.1	Eupatorium yellow vein mosaic betasatellite

2080107	GCA_000838325.1	Eupatorium yellow vein virus
269095	GCA_002822325.1	Eupatorium yellow vein virus - [Yamaguchi]
519350	GCA_002822365.1	Eupatorium yellow vein virus-[Japan:Kagawa:Tomato:1997
223276	GCA_002986575.1	Eupatorium yellow vein virus-[MNS2]
335404	GCA_002986545.1	Eupatorium yellow vein virus-[SOJ3]
436246	GCA_002822345.1	Eupatorium yellow vein virus-[Suya]
1853865	GCA_001654365.1	Euphorbia caput-medusae latent virus
1853865	GCA_002987195.1	Euphorbia caput-medusae latent virus
2291614	GCA_003847545.1	Euphorbia heterophylla associated gemycircularvirus
412688	GCA_002986585.1	Euphorbia leaf curl Guangxi virus
269060	GCA_000843385.1	Euphorbia leaf curl virus - [G35]
2169733	GCA_003034025.1	Euphorbia mosaic Peru virus
429564	GCA_000869345.1	Euphorbia mosaic virus - A [Mexico:Yucatan:2004]
291286	GCA_001766625.1	Euphorbia ringspot virus
1578825	GCA_003029205.1	Euphorbia yellow leaf curl virus
598494	GCA_000882835.1	Euphorbia yellow mosaic virus
1016875	GCA_002822385.1	Euphorbia yellow mosaic virus - Goias [Brazil:GO:Luziania 8
666147	GCA_000889375.1	Euphorbia yellow mosaic virus associated DNA 1
307467	GCA_000883795.1	Euproctis pseudoconspersa nucleopolyhedrovirus
186165	GCA_000849505.1	Euprosterna elaeasa virus
57482	GCA_000870765.1	European bat 1 lyssavirus
57482	GCA_900176735.1	European bat 1 lyssavirus
57482	GCA_900176725.1	European bat 1 lyssavirus
57482	GCA_900176695.1	European bat 1 lyssavirus
57482	GCA_900176765.1	European bat 1 lyssavirus
57482	GCA_900176705.1	European bat 1 lyssavirus
57482	GCA_900176775.1	European bat 1 lyssavirus
57482	GCA_900176745.1	European bat 1 lyssavirus
57482	GCA_900176755.1	European bat 1 lyssavirus
57483	GCA_000871625.2	European bat 2 lyssavirus
33756	GCA_000857785.1	European brown hare syndrome virus
84739	GCA_000897115.1	European catfish virus
84739	GCA_002826585.1	European catfish virus
1980426	GCA_000884235.1	European mountain ash ringspot-associated emaravirus
1920698	GCA_001904925.1	Euscelidius variegatus virus 1
2083198	GCA_002829865.1	Everglades virus
2419623	GCA_003718875.1	Exiguobacterium phage vB_EalM-132
2419624	GCA_003718855.1	Exiguobacterium phage vB_EalM-137
2546569	GCA_004355685.1	Exiguobacterium phage vB_EauM-23
1775141	GCA_002608805.1	Exiguobacterium phage vB_EauS-123
2093275	GCA_002937325.1	Exomis microphylla associated virus
230604	GCA_003972145.1	Extra small virus
230604	GCA_003972245.1	Extra small virus
230604	GCA_003972265.1	Extra small virus
230604	GCA_003972165.1	Extra small virus
230604	GCA_003972205.1	Extra small virus
230604	GCA_003972225.1	Extra small virus
62352	GCA_000852925.1	Eyach virus
1441773	GCA_000919695.1	Faba bean necrotic stunt alphasatellite
1441799	GCA_000919055.1	Faba bean necrotic stunt alphasatellite 1

283824	GCA_000884215.1	Faba bean necrotic stunt virus
1453080	GCA_000926155.1	Faba bean necrotic yellows C1 alphasatellite
1453081	GCA_000922135.1	Faba bean necrotic yellows C11 alphasatellite
1453082	GCA_000921295.1	Faba bean necrotic yellows C7 alphasatellite
1453083	GCA_000923815.1	Faba bean necrotic yellows C9 alphasatellite
1453083	GCA_003033965.1	Faba bean necrotic yellows C9 alphasatellite
59817	GCA_000844665.1	Faba bean necrotic yellows virus
1391029	GCA_000930455.1	Faecal-associated gemycircularvirus 1b
1391030	GCA_000929815.1	Faecal-associated gemycircularvirus 1c
2070181	GCA_002958055.1	Faecalibacterium phage FP_Brigit
2070182	GCA_002958065.1	Faecalibacterium phage FP_Epona
2070183	GCA_002958075.1	Faecalibacterium phage FP_Lagaffe
2070184	GCA_002958085.1	Faecalibacterium phage FP_Lugh
2070185	GCA_002958095.1	Faecalibacterium phage FP_Mushu
2070188	GCA_002958125.1	Faecalibacterium phage FP_oengus
2070186	GCA_002958105.1	Faecalibacterium phage FP_Taranis
2070187	GCA_002958115.1	Faecalibacterium phage FP_Toutatis
1391025	GCA_000930475.1	Faeces associated gemycircularvirus 10
1391026	GCA_000929835.1	Faeces associated gemycircularvirus 11
1391027	GCA_000928795.1	Faeces associated gemycircularvirus 12
1843734	GCA_001646535.1	Faeces associated gemycircularvirus 14
1843734	GCA_003849245.1	Faeces associated gemycircularvirus 14
1843735	GCA_001645955.1	Faeces associated gemycircularvirus 15
1843736	GCA_001646135.1	Faeces associated gemycircularvirus 16
1843736	GCA_003849145.1	Faeces associated gemycircularvirus 16
1843737	GCA_001646335.1	Faeces associated gemycircularvirus 17
1843738	GCA_001646515.1	Faeces associated gemycircularvirus 18
1843739	GCA_001645935.1	Faeces associated gemycircularvirus 19
1391031	GCA_000928775.1	Faeces associated gemycircularvirus 2
1843740	GCA_001646115.1	Faeces associated gemycircularvirus 20
1843740	GCA_003849205.1	Faeces associated gemycircularvirus 20
1843740	GCA_003849185.1	Faeces associated gemycircularvirus 20
1843741	GCA_001646315.1	Faeces associated gemycircularvirus 21
1843741	GCA_003849165.1	Faeces associated gemycircularvirus 21
1843742	GCA_001646495.1	Faeces associated gemycircularvirus 22
1391032	GCA_000927875.1	Faeces associated gemycircularvirus 3
1391033	GCA_000930435.1	Faeces associated gemycircularvirus 4
1391033	GCA_003849225.1	Faeces associated gemycircularvirus 4
1391034	GCA_000929795.1	Faeces associated gemycircularvirus 5
1391035	GCA_000928755.1	Faeces associated gemycircularvirus 6
1391036	GCA_000927855.1	Faeces associated gemycircularvirus 7
1567404	GCA_000925135.1	Fako virus
1964370	GCA_002355005.1	Falcon picornavirus
1510155	GCA_000922095.1	Falconid herpesvirus 1
1641896	GCA_000981755.1	Falcovirus A1
1963254	GCA_002288715.1	Fall chinook aquareovirus
2293279	GCA_003846625.1	False black widow spider associated circular virus 1
248053	GCA_002118485.1	Farallon virus
1380894	GCA_003986365.1	Farfantepenaeus duorarum circovirus
1692248	GCA_001274185.1	Farfantepenaeus duorarum pink shrimp associated circular

1027468	GCA_000926315.1	Farmington virus
1949201	GCA_002285005.1	Fathead minnow calicivirus
889873	GCA_002816255.1	Fathead minnow nidovirus
1401263	GCA_000916055.1	Fathead minnow picornavirus
1401263	GCA_002817215.1	Fathead minnow picornavirus
1477405	GCA_003051125.1	Faustovirus
1477405	GCA_003051205.1	Faustovirus
1477405	GCA_003051145.1	Faustovirus
1477405	GCA_003051165.1	Faustovirus
1477405	GCA_003051705.1	Faustovirus
1477405	GCA_003051225.1	Faustovirus
1477405	GCA_003051185.1	Faustovirus
1973452	GCA_900176665.1	Faustovirus ST1
39420	GCA_000874805.1	Feldmannia species virus
10334	GCA_000885455.1	Felid alphaherpesvirus 1
1389204	GCA_000910975.1	Feline astrovirus 2
1538452	GCA_000921515.1	Feline astrovirus D1
1417756	GCA_000911415.1	Feline bocaparvovirus 2
2259807	GCA_003033235.1	Feline bocaparvovirus 3
1174530	GCA_000896155.1	Feline bocavirus
11978	GCA_000853345.1	Feline calicivirus
1530454	GCA_000923395.1	Feline cyclovirus
53182	GCA_000857425.1	Feline foamy virus
53182	GCA_003047975.1	Feline foamy virus
11673	GCA_000854865.1	Feline immunodeficiency virus
11135	GCA_000856025.1	Feline infectious peritonitis virus
11768	GCA_000850105.1	Feline leukemia virus
1170234	GCA_000925315.1	Feline morbillivirus
1170234	GCA_002815235.1	Feline morbillivirus
568209	GCA_002826945.1	Feline papillomavirus type 2
1108810	GCA_000894955.1	Feline picornavirus
1416021	GCA_000913895.1	Feline sakobuvirus A
2249941	GCA_004041515.1	Feline stool-associated circular virus KU14
1452540	GCA_001430095.1	Felis catus gammaherpesvirus 1
1336600	GCA_000910155.1	Felis catus papillomavirus 3
1398507	GCA_000912755.1	Felis catus papillomavirus 4
2025339	GCA_002271065.1	Felis catus papillomavirus type 5
188524	GCA_000845485.1	Felis domesticus papillomavirus type 1
1692107	GCA_001274225.2	Fengkai orbivirus
797418	GCA_000887475.1	Fenneropenaeus chinensis hepandensovirus
1664810	GCA_002814355.1	Ferak virus
1264898	GCA_001661775.1	Ferret coronavirus
1264898	GCA_003971885.1	Ferret coronavirus
1213422	GCA_002826485.1	Ferret hepatitis E virus
1213422	GCA_002826505.1	Ferret hepatitis E virus
1394142	GCA_002118445.1	Ferret parechovirus
2069324	GCA_004128755.1	Festuca pratensis amalgavirus 1
2069325	GCA_004128775.1	Festuca pratensis amalgavirus 2
1692249	GCA_001274425.1	Fiddler Crab associated circular virus
1034096	GCA_000895535.1	Fig badnavirus 1

882768	GCA_000893595.1	Fig cryptic virus
548908	GCA_000893235.1	Fig fleck-associated virus
1980427	GCA_001580335.1	Fig mosaic emaravirus
10649	GCA_000845905.1	Figwort mosaic virus
77698	GCA_000863765.1	Fiji disease virus
1408144	GCA_000927015.1	Fikirini rhabdovirus
400122	GCA_000869525.1	Finch circovirus
353765	GCA_002889515.1	Finkel-Biskis-Jinkins murine sarcoma virus
2293280	GCA_003846985.1	Fire ant associated circular virus 1
1574287	GCA_000928055.1	Fisavirus 1
2059380	GCA_002889855.1	Flamingopox virus FGPVKD09
568090	GCA_002867835.1	Flammulina velutipes browning virus
1972612	GCA_001432015.1	Flanders hapavirus
1972612	GCA_002815595.1	Flanders hapavirus
294631	GCA_000846125.1	Flavobacterium phage 11b
1792272	GCA_001884555.1	Flavobacterium phage 1H
1814279	GCA_002603385.1	Flavobacterium phage 23T
1792273	GCA_001881715.1	Flavobacterium phage 2A
1325731	GCA_000909695.1	Flavobacterium phage 6H
908819	GCA_001019815.1	Flavobacterium phage FCL-2
1033977	GCA_002600755.1	Flavobacterium phage FCV-1
1984747	GCA_002624085.1	Flavobacterium phage FCV-10
1983585	GCA_002623885.1	Flavobacterium phage FCV-11
1984748	GCA_002624105.1	Flavobacterium phage FCV-16
1984758	GCA_002624305.1	Flavobacterium phage FCV-20
1983586	GCA_002623905.1	Flavobacterium phage FCV-3
908820	GCA_002630825.1	Flavobacterium phage FL-1
2023716	GCA_002627285.1	Flavobacterium phage FLiP
1792274	GCA_001882275.1	Flavobacterium phage Fpv1
1814280	GCA_001885365.1	Flavobacterium phage Fpv10
1814281	GCA_001884635.1	Flavobacterium phage Fpv11
1814282	GCA_001884595.1	Flavobacterium phage Fpv2
1814283	GCA_001882215.1	Flavobacterium phage Fpv20
1740107	GCA_002607705.1	Flavobacterium phage FpV21
1814284	GCA_001881895.1	Flavobacterium phage Fpv3
1740108	GCA_002607785.1	Flavobacterium phage FpV4
1814285	GCA_001882235.1	Flavobacterium phage Fpv5
1814286	GCA_001881855.1	Flavobacterium phage Fpv6
1814287	GCA_001881775.1	Flavobacterium phage Fpv7
1814288	GCA_001881815.1	Flavobacterium phage Fpv8
1740109	GCA_002607815.1	Flavobacterium phage FpV9
1984749	GCA_002624125.1	Flavobacterium phage V156
1984750	GCA_002624145.1	Flavobacterium phage V157
1984751	GCA_002624165.1	Flavobacterium phage V165
1983543	GCA_002623625.1	Flavobacterium phage V175
1983544	GCA_002623645.1	Flavobacterium phage V181
1984752	GCA_002624185.1	Flavobacterium phage V182
1984753	GCA_002624205.1	Flavobacterium phage VK20
1984754	GCA_002624225.1	Flavobacterium phage VK42
1984755	GCA_002624245.1	Flavobacterium phage VK48

1984756	GCA_002624265.1	Flavobacterium phage VK52
1984757	GCA_002624285.1	Flavobacterium phage VK58
1458858	GCA_002604425.1	Flavobacterium sp. phage 1/32
45220	GCA_000872925.1	Flexal mammarenavirus
12287	GCA_000854385.1	Flock House virus
12287	GCA_003971805.1	Flock House virus
12287	GCA_003971825.1	Flock House virus
2293281	GCA_003847165.1	Fly associated circular virus 1
2293282	GCA_003847485.1	Fly associated circular virus 2
2293283	GCA_003846965.1	Fly associated circular virus 3
2293284	GCA_003846945.1	Fly associated circular virus 4
2293285	GCA_003847105.1	Fly associated circular virus 5
2293286	GCA_003846845.1	Fly associated circular virus 6
2293287	GCA_003846825.1	Fly associated circular virus 7
12118	GCA_000863325.1	Foot-and-mouth disease virus - type O
12118	GCA_002816555.1	Foot-and-mouth disease virus - type O
1432856	GCA_000915215.1	Formica exsecta virus 1
1432857	GCA_000916735.1	Formica exsecta virus 2
1955198	GCA_002024755.1	Fort Crockett virus
48544	GCA_000885435.1	Fort Morgan virus
10553	GCA_000884315.1	Fowl adenovirus 1
172864	GCA_000886795.1	Fowl adenovirus 9
10553	GCA_000845105.1	Fowl aviadenovirus 1
172861	GCA_000907375.1	Fowl aviadenovirus 5
172862	GCA_002817995.1	Fowl aviadenovirus 6
172864	GCA_000844285.1	Fowl aviadenovirus 9
190063	GCA_000890915.1	Fowl aviadenovirus C
190065	GCA_000890555.1	Fowl aviadenovirus E
10261	GCA_000838605.1	Fowlpox virus
1678251	GCA_004050155.1	Fox circovirus
1678251	GCA_004062915.1	Fox circovirus
1678251	GCA_004062875.1	Fox circovirus
1678251	GCA_004062895.1	Fox circovirus
1504569	GCA_001503895.1	Fox fecal rhabdovirus
12179	GCA_000849045.1	Foxtail mosaic virus
335204	GCA_000873185.1	Fragaria chiloensis cryptic virus
255238	GCA_000858685.1	Fragaria chiloensis latent virus
485362	GCA_000884255.1	Francolinus leucoscepus papillomavirus 1
99585	GCA_000887655.1	Frangipani mosaic virus
1737586	GCA_001634555.1	Free State vervet virus
1737586	GCA_003972005.1	Free State vervet virus
421012	GCA_000889895.1	Freesia mosaic virus
1200971	GCA_000897335.1	French bean leaf curl betasatellite-Kanpur
2050584	GCA_000899775.1	French bean leaf curl virus
1218727	GCA_000899975.1	French bean severe leaf curl virus
1838151	GCA_002609825.1	Freshwater phage uvFW-CGR-AMD-COM-C203
1838152	GCA_002609845.1	Freshwater phage uvFW-CGR-AMD-COM-C403
1838153	GCA_002609865.1	Freshwater phage uvFW-CGR-AMD-COM-C429
1838154	GCA_002609885.1	Freshwater phage uvFW-CGR-AMD-COM-C440
1838155	GCA_002609905.1	Freshwater phage uvFW-CGR-AMD-COM-C449

1838156	GCA_002609925.1	Freshwater phage uvFW-CGR-AMD-COM-C455
1838157	GCA_002609945.1	Freshwater phage uvFW-CGR-AMD-COM-C493
1838158	GCA_002609965.1	Freshwater phage uvFW-CGR-AMDFOS-S50-C341
11795	GCA_000859065.1	Friend murine leukemia virus
11820	GCA_000849885.1	Friend spleen focus-forming virus
332471	GCA_000880155.1	Fritillary virus Y
114102	GCA_000844965.1	Frog adenovirus 1
10493	GCA_000844425.1	Frog virus 3
1343901	GCA_000921855.1	Fruit bat alphaherpesvirus 1
1437064	GCA_003090935.1	FTLS virus
1437064	GCA_003090955.1	FTLS virus
1437064	GCA_003090995.1	FTLS virus
1437064	GCA_003088995.1	FTLS virus
1437064	GCA_003091195.1	FTLS virus
1437064	GCA_003089215.1	FTLS virus
1437064	GCA_003091415.1	FTLS virus
1437064	GCA_003090335.1	FTLS virus
1437064	GCA_003089435.1	FTLS virus
1437064	GCA_003091635.1	FTLS virus
1437064	GCA_003089655.1	FTLS virus
1437064	GCA_003088335.1	FTLS virus
1437064	GCA_003091855.1	FTLS virus
1437064	GCA_003087675.1	FTLS virus
1437064	GCA_003089895.1	FTLS virus
1437064	GCA_003087895.1	FTLS virus
1437064	GCA_003091995.1	FTLS virus
1437064	GCA_003092015.1	FTLS virus
1437064	GCA_003092035.1	FTLS virus
1437064	GCA_003092055.1	FTLS virus
1437064	GCA_003092075.1	FTLS virus
1437064	GCA_003092095.1	FTLS virus
1437064	GCA_003092115.1	FTLS virus
1437064	GCA_003092135.1	FTLS virus
1437064	GCA_003092155.1	FTLS virus
1437064	GCA_003090115.1	FTLS virus
1437064	GCA_003092175.1	FTLS virus
1437064	GCA_003092195.1	FTLS virus
1437064	GCA_003088115.1	FTLS virus
1437064	GCA_003092215.1	FTLS virus
1437064	GCA_003092235.1	FTLS virus
1437064	GCA_003092295.1	FTLS virus
1437064	GCA_003090555.1	FTLS virus
1437064	GCA_003088555.1	FTLS virus
1437064	GCA_003090755.1	FTLS virus
1437064	GCA_003088775.1	FTLS virus
1437064	GCA_003090875.1	FTLS virus
1437064	GCA_003090895.1	FTLS virus
1437064	GCA_003090915.1	FTLS virus
11885	GCA_000847725.1	Fujinami sarcoma virus
318849	GCA_002118965.1	Fukuoka virus

1463817	GCA_000922895.1	Fulmarus glacialis papillomavirus 1
1353241	GCA_000919715.1	Fur seal faeces associated circular DNA virus
2013564	GCA_002237235.1	Fur seal picorna-like virus
676234	GCA_000905235.1	Furcraea necrotic streak virus
2268743	GCA_004134365.1	Fusarium asiaticum victorivirus 1
1562380	GCA_000955595.1	Fusarium coeruleum mitovirus 1
1562379	GCA_000955475.1	Fusarium globosum mitovirus 1
2060778	GCA_002890645.1	Fusarium graminearum alternavirus 1
1872710	GCA_001695505.1	Fusarium graminearum deltaflexivirus 1
194397	GCA_000857105.1	Fusarium graminearum dsRNA mycovirus-1
687917	GCA_000885415.1	Fusarium graminearum dsRNA mycovirus-3
687918	GCA_000886915.1	Fusarium graminearum dsRNA mycovirus-4
1284208	GCA_000917655.1	Fusarium graminearum hypovirus 1
1640379	GCA_000973455.1	Fusarium graminearum hypovirus 2
1809243	GCA_004129275.1	Fusarium graminearum mycotymovirus 1
1926644	GCA_001923275.1	Fusarium langsethiae hypovirus 1
434898	GCA_002833645.1	Fusarium oxysporum chrysovirus 1
1679238	GCA_001184825.1	Fusarium oxysporum f. sp. dianthi mycovirus 1
1849539	GCA_001723025.1	Fusarium poae alternavirus 1
1848042	GCA_001651085.1	Fusarium poae dsRNA virus 2
1848169	GCA_001651205.1	Fusarium poae dsRNA virus 3
1849537	GCA_001722745.1	Fusarium poae fusarivirus 1
1848150	GCA_001722985.1	Fusarium poae mitovirus 1
1848151	GCA_001722785.1	Fusarium poae mitovirus 2
1848152	GCA_001722865.1	Fusarium poae mitovirus 3
1848153	GCA_001722685.1	Fusarium poae mitovirus 4
1849542	GCA_001722825.1	Fusarium poae mycovirus 1
1849543	GCA_001722905.1	Fusarium poae mycovirus 2
1849531	GCA_001722945.1	Fusarium poae narnavirus 1
1849532	GCA_001722765.1	Fusarium poae narnavirus 2
1849544	GCA_001722725.1	Fusarium poae negative-stranded virus 1
1849545	GCA_001723005.1	Fusarium poae negative-stranded virus 2
1849533	GCA_001723045.1	Fusarium poae partitivirus 2
1849535	GCA_001722925.1	Fusarium poae victorivirus 1
75747	GCA_000853125.1	Fusarium poae virus 1
1849534	GCA_001722845.1	Fusarium poae virus 1-240374
1511847	GCA_000851545.1	Fusarium solani virus 1
2530024	GCA_004340475.1	Fusobacterium phage Fnu1
1640977	GCA_002605705.1	Fusobacterium phage Funu1
1640978	GCA_003328525.1	Fusobacterium phage Funu2
64307	GCA_002003975.1	Gadgets Gully virus
1468172	GCA_000919115.1	Gaillardia latent virus
1535802	GCA_000930915.1	Gairo mammarenavirus
60714	GCA_000859945.1	Galinsoga mosaic virus
37138	GCA_000840325.1	Galleria mellonella densovirus
10386	GCA_000847005.2	Gallid alphaherpesvirus 1
10390	GCA_000846265.1	Gallid alphaherpesvirus 2 (Marek disease virus type 1)
35250	GCA_000838845.1	Gallid alphaherpesvirus 3
1766557	GCA_001500755.1	Gallivirus Pf-CHK1/GV
1746059	GCA_001443745.1	Gamboa mosquito virus

35313	GCA_002831325.1	Gamboa virus
1175852	GCA_004288735.1	Gammapapillomavirus 10
1513256	GCA_004288715.1	Gammapapillomavirus 11
1513256	GCA_004288695.1	Gammapapillomavirus 11
1513257	GCA_004288635.1	Gammapapillomavirus 12
1513257	GCA_004288655.1	Gammapapillomavirus 12
1513257	GCA_004288675.1	Gammapapillomavirus 12
1513257	GCA_004288595.1	Gammapapillomavirus 12
1513257	GCA_004288615.1	Gammapapillomavirus 12
1513258	GCA_004288555.1	Gammapapillomavirus 13
1513258	GCA_004288575.1	Gammapapillomavirus 13
1513259	GCA_004288535.1	Gammapapillomavirus 14
1513260	GCA_004288455.1	Gammapapillomavirus 15
1513260	GCA_004288475.1	Gammapapillomavirus 15
1513260	GCA_004288495.1	Gammapapillomavirus 15
1513260	GCA_004288515.1	Gammapapillomavirus 15
1513261	GCA_004288435.1	Gammapapillomavirus 16
1513263	GCA_004288395.1	Gammapapillomavirus 18
1513263	GCA_004287835.1	Gammapapillomavirus 18
1513263	GCA_004288415.1	Gammapapillomavirus 18
1513263	GCA_004288375.1	Gammapapillomavirus 18
1513264	GCA_004287715.1	Gammapapillomavirus 19
1513264	GCA_004287735.1	Gammapapillomavirus 19
1513264	GCA_004287755.1	Gammapapillomavirus 19
1513264	GCA_004287775.1	Gammapapillomavirus 19
1513264	GCA_004287795.1	Gammapapillomavirus 19
1961679	GCA_004287695.1	Gammapapillomavirus 22
1961679	GCA_004288135.1	Gammapapillomavirus 22
1961679	GCA_004288155.1	Gammapapillomavirus 22
1961679	GCA_004288175.1	Gammapapillomavirus 22
1961679	GCA_004288195.1	Gammapapillomavirus 22
1961679	GCA_004288215.1	Gammapapillomavirus 22
1961679	GCA_004288235.1	Gammapapillomavirus 22
1961679	GCA_004288255.1	Gammapapillomavirus 22
1961679	GCA_004288275.1	Gammapapillomavirus 22
1961679	GCA_004288295.1	Gammapapillomavirus 22
1961679	GCA_004288315.1	Gammapapillomavirus 22
1961680	GCA_004288095.1	Gammapapillomavirus 23
1961680	GCA_004288115.1	Gammapapillomavirus 23
1961681	GCA_004287595.1	Gammapapillomavirus 24
1961681	GCA_004287975.1	Gammapapillomavirus 24
1961681	GCA_004287995.1	Gammapapillomavirus 24
1961681	GCA_004288015.1	Gammapapillomavirus 24
1961681	GCA_004288035.1	Gammapapillomavirus 24
1961681	GCA_004288055.1	Gammapapillomavirus 24
1961681	GCA_004288075.1	Gammapapillomavirus 24
333931	GCA_004289455.1	Gammapapillomavirus 5
1175848	GCA_004289415.1	Gammapapillomavirus 6
1175848	GCA_004289435.1	Gammapapillomavirus 6
1175849	GCA_004289295.1	Gammapapillomavirus 7

1175849	GCA_004289315.1	Gammapapillomavirus 7
1175849	GCA_004289335.1	Gammapapillomavirus 7
1175849	GCA_004289355.1	Gammapapillomavirus 7
1175849	GCA_004289375.1	Gammapapillomavirus 7
1175849	GCA_004289395.1	Gammapapillomavirus 7
1175850	GCA_004289195.1	Gammapapillomavirus 8
1175850	GCA_004289215.1	Gammapapillomavirus 8
1175850	GCA_004289235.1	Gammapapillomavirus 8
1175850	GCA_004289255.1	Gammapapillomavirus 8
1175850	GCA_004289275.1	Gammapapillomavirus 8
1175851	GCA_004288755.1	Gammapapillomavirus 9
1175851	GCA_004288775.1	Gammapapillomavirus 9
1175851	GCA_004288795.1	Gammapapillomavirus 9
1175851	GCA_004288815.1	Gammapapillomavirus 9
1175851	GCA_004287875.1	Gammapapillomavirus 9
2049444	GCA_004131605.1	Gammapapillomavirus sp.
2049444	GCA_004131685.1	Gammapapillomavirus sp.
2049444	GCA_004131525.1	Gammapapillomavirus sp.
2049444	GCA_004131625.1	Gammapapillomavirus sp.
2049444	GCA_004131565.1	Gammapapillomavirus sp.
2049444	GCA_004131665.1	Gammapapillomavirus sp.
2049444	GCA_004131645.1	Gammapapillomavirus sp.
2049444	GCA_004131545.1	Gammapapillomavirus sp.
2049444	GCA_004131585.1	Gammapapillomavirus sp.
2049444	GCA_004289495.1	Gammapapillomavirus sp.
2049444	GCA_004289475.1	Gammapapillomavirus sp.
2049444	GCA_004287475.1	Gammapapillomavirus sp.
2049444	GCA_004287935.1	Gammapapillomavirus sp.
2049444	GCA_004287495.1	Gammapapillomavirus sp.
1692250	GCA_001274045.1	Gammarus sp. amphipod associated circular virus
1846259	GCA_001887845.1	Gannoruwa bat lyssavirus
47900	GCA_000896535.1	Garlic common latent virus
12458	GCA_000861065.1	Garlic latent virus
12433	GCA_000848665.1	Garlic virus A
12432	GCA_000928855.1	Garlic virus B
12431	GCA_000847865.1	Garlic virus C
12430	GCA_000915015.1	Garlic virus D
150285	GCA_000852345.1	Garlic virus E
150284	GCA_000856365.1	Garlic virus X
2006642	GCA_002219445.1	Garrulus glandarius associated circular virus 1
2006642	GCA_004320345.1	Garrulus glandarius associated circular virus 1
1296573	GCA_000905215.1	Gastropod associated circular ssDNA virus
1911435	GCA_001866265.1	Gata virus
578305	GCA_000881115.1	Gayfeather mild mottle virus
54290	GCA_000862005.1	GB virus C
54290	GCA_002821165.1	GB virus C
54290	GCA_002821185.1	GB virus C
54290	GCA_002821205.1	GB virus C
54290	GCA_002821285.1	GB virus C
54290	GCA_002821145.1	GB virus C

54290	GCA_002821225.1	GB virus C
54290	GCA_002821245.1	GB virus C
54290	GCA_002821265.1	GB virus C
93986	GCA_002821305.1	GB virus C variant troglodytes
2202566	GCA_003659065.1	Geminiviridae sp.
2202566	GCA_003652465.1	Geminiviridae sp.
2202566	GCA_003659425.1	Geminiviridae sp.
2202566	GCA_003655485.1	Geminiviridae sp.
2202566	GCA_003659625.1	Geminiviridae sp.
2202566	GCA_003653985.1	Geminiviridae sp.
2202566	GCA_003652045.1	Geminiviridae sp.
1588761	GCA_003849445.1	Gemycircularvirus BZ1
1588762	GCA_003849425.1	Gemycircularvirus BZ2
1673681	GCA_002826005.1	Gemycircularvirus C1c
1708653	GCA_001292975.1	Gemycircularvirus gemy-ch-rat1
1862824	GCA_001679855.1	Gemycircularvirus HV-GcV1
1862825	GCA_001679875.1	Gemycircularvirus HV-GcV2
1588763	GCA_003849405.1	Gemycircularvirus NP
1588764	GCA_000973195.1	Gemycircularvirus SL1
1588765	GCA_003849485.1	Gemycircularvirus SL2
1588766	GCA_003849465.1	Gemycircularvirus SL3
1983771	GCA_003848465.1	Gemycircularvirus sp.
1983771	GCA_003848605.1	Gemycircularvirus sp.
1983771	GCA_003848445.1	Gemycircularvirus sp.
1983771	GCA_003848625.1	Gemycircularvirus sp.
2202565	GCA_003656345.1	Genomoviridae sp.
2202565	GCA_003846465.1	Genomoviridae sp.
2202565	GCA_003846485.1	Genomoviridae sp.
1150353	GCA_000906635.1	Gentian Kobu-sho-associated virus
1150353	GCA_003956685.1	Gentian Kobu-sho-associated virus
182452	GCA_002867125.1	Gentian mosaic virus
1920772	GCA_000921435.1	Gentian ovary ringspot virus
1458842	GCA_000914595.1	Geobacillus phage GBK2
365048	GCA_000867645.1	Geobacillus phage GBSV1
1965361	GCA_002619845.2	Geobacillus phage TP-84
1965361	GCA_002619845.1	Geobacillus phage TP-84
447909	GCA_000870845.1	Geobacillus virus E2
1572712	GCA_001550705.1	Geobacillus virus E3
59300	GCA_000855805.1	Getah virus
2293288	GCA_003847265.1	Giant house spider associated circular virus 1
2293289	GCA_003846725.1	Giant house spider associated circular virus 2
2293290	GCA_003846705.1	Giant house spider associated circular virus 3
2293291	GCA_003846645.1	Giant house spider associated circular virus 4
2016460	GCA_002219365.1	Giant panda anellovirus
2016461	GCA_002219905.1	Giant panda associated gemycircularvirus
2016461	GCA_003848705.1	Giant panda associated gemycircularvirus
2016461	GCA_003848725.1	Giant panda associated gemycircularvirus
2016461	GCA_003848745.1	Giant panda associated gemycircularvirus
2016461	GCA_003848765.1	Giant panda associated gemycircularvirus
2016461	GCA_003848785.1	Giant panda associated gemycircularvirus

2016461	GCA_003848805.1	Giant panda associated gemycircularvirus
2016461	GCA_003848825.1	Giant panda associated gemycircularvirus
2016461	GCA_003848845.1	Giant panda associated gemycircularvirus
2016461	GCA_003848865.1	Giant panda associated gemycircularvirus
2016461	GCA_003848885.1	Giant panda associated gemycircularvirus
2016461	GCA_003848905.1	Giant panda associated gemycircularvirus
2016461	GCA_003848925.1	Giant panda associated gemycircularvirus
2016461	GCA_003848945.1	Giant panda associated gemycircularvirus
2016461	GCA_003848965.1	Giant panda associated gemycircularvirus
2016462	GCA_003729495.1	Giant panda associated partiti-like virus
2016456	GCA_002219385.1	Giant panda circovirus 1
2016457	GCA_002219925.1	Giant panda circovirus 2
2016458	GCA_002219565.1	Giant panda circovirus 3
2016459	GCA_002219745.1	Giant panda circovirus 4
2016463	GCA_002219545.1	Giant panda polyomavirus
697298	GCA_002641055.1	Giant panda rotavirus A
2282853	GCA_900500615.1	Giant squirrel respirovirus
29255	GCA_000854825.1	Giardia lamblia virus
11840	GCA_000849965.1	Gibbon ape leukemia virus
2082658	GCA_004132545.1	Gigaspora margarita giardia-like virus 1
2082665	GCA_004132465.1	Gigaspora margarita mitovirus 1
2082666	GCA_004132485.1	Gigaspora margarita mitovirus 2
2082667	GCA_004132505.1	Gigaspora margarita mitovirus 3
2082668	GCA_004132525.1	Gigaspora margarita mitovirus 4
2249931	GCA_003848385.1	Gila monster-associated gemycircularvirus
96491	GCA_000872605.1	Gill-associated virus
1922325	GCA_003033125.1	Giraffa camelopardalis papillomavirus 1
2170404	GCA_004132885.1	Glis glis polyomavirus 1
12201	GCA_002828505.1	Gloriosa stripe mosaic virus
379529	GCA_000879155.1	Glossina pallidipes salivary gland hypertrophy virus
379529	GCA_003687705.1	Glossina pallidipes salivary gland hypertrophy virus
2047788	GCA_002956215.1	Gluconobacter phage GC1
419435	GCA_000872945.1	Glypta fumiferanae ichnovirus
1837217	GCA_002194465.1	Goat torovirus
376852	GCA_000840165.1	Goatpox virus Pellor
1655646	GCA_001190615.1	Gokushovirinae Bog1183_53
1655649	GCA_001190475.1	Gokushovirinae Bog5712_52
1655650	GCA_001190595.1	Gokushovirinae Bog8989_22
1655656	GCA_001190535.1	Gokushovirinae Fen672_31
1655659	GCA_001190375.1	Gokushovirinae Fen7875_21
1701404	GCA_001502915.1	Gokushovirinae GAIR4
1701405	GCA_001502295.1	Gokushovirinae GNX3R
2073143	GCA_002990155.1	Gokushovirus MK-2017
1758150	GCA_002589315.1	Gokushovirus WZ-2015a
1758150	GCA_002589345.1	Gokushovirus WZ-2015a
1758150	GCA_002589385.1	Gokushovirus WZ-2015a
1758150	GCA_002589425.1	Gokushovirus WZ-2015a
1758150	GCA_002589025.1	Gokushovirus WZ-2015a
1758150	GCA_002589465.1	Gokushovirus WZ-2015a
1758150	GCA_002589495.1	Gokushovirus WZ-2015a

1758150	GCA_002589575.1	Gokushovirus WZ-2015a
1758150	GCA_002589615.1	Gokushovirus WZ-2015a
1758150	GCA_002589655.1	Gokushovirus WZ-2015a
1758150	GCA_002589675.1	Gokushovirus WZ-2015a
1758150	GCA_002589765.1	Gokushovirus WZ-2015a
1758150	GCA_002589835.1	Gokushovirus WZ-2015a
1758150	GCA_002589725.1	Gokushovirus WZ-2015a
1758150	GCA_002589865.1	Gokushovirus WZ-2015a
1758150	GCA_002589885.1	Gokushovirus WZ-2015a
1758150	GCA_002589905.1	Gokushovirus WZ-2015a
1758150	GCA_002589925.1	Gokushovirus WZ-2015a
1758150	GCA_002589945.1	Gokushovirus WZ-2015a
1758150	GCA_002589965.1	Gokushovirus WZ-2015a
1758150	GCA_002589985.1	Gokushovirus WZ-2015a
1758150	GCA_002590005.1	Gokushovirus WZ-2015a
1758150	GCA_002590025.1	Gokushovirus WZ-2015a
1758150	GCA_002590045.1	Gokushovirus WZ-2015a
1758150	GCA_002590065.1	Gokushovirus WZ-2015a
1758150	GCA_002590095.1	Gokushovirus WZ-2015a
1758150	GCA_002590115.1	Gokushovirus WZ-2015a
1758150	GCA_002590135.1	Gokushovirus WZ-2015a
1758150	GCA_002590155.1	Gokushovirus WZ-2015a
1758150	GCA_002590175.1	Gokushovirus WZ-2015a
1758150	GCA_002590195.1	Gokushovirus WZ-2015a
1758150	GCA_002590215.1	Gokushovirus WZ-2015a
1758150	GCA_002590235.1	Gokushovirus WZ-2015a
1758150	GCA_002590255.1	Gokushovirus WZ-2015a
1758150	GCA_002590275.1	Gokushovirus WZ-2015a
1758150	GCA_002590295.1	Gokushovirus WZ-2015a
1758150	GCA_002590315.1	Gokushovirus WZ-2015a
1758150	GCA_002590335.1	Gokushovirus WZ-2015a
1758150	GCA_002590355.1	Gokushovirus WZ-2015a
1758150	GCA_002589805.1	Gokushovirus WZ-2015a
1758150	GCA_002590375.1	Gokushovirus WZ-2015a
1758150	GCA_002590395.1	Gokushovirus WZ-2015a
1758150	GCA_002590415.1	Gokushovirus WZ-2015a
1758150	GCA_002590435.1	Gokushovirus WZ-2015a
1758150	GCA_002590455.1	Gokushovirus WZ-2015a
1758150	GCA_002590475.1	Gokushovirus WZ-2015a
1758150	GCA_002590495.1	Gokushovirus WZ-2015a
1758150	GCA_002590515.1	Gokushovirus WZ-2015a
1758150	GCA_002590535.1	Gokushovirus WZ-2015a
1758150	GCA_002590555.1	Gokushovirus WZ-2015a
1758150	GCA_002590575.1	Gokushovirus WZ-2015a
1758150	GCA_002590595.1	Gokushovirus WZ-2015a
1758150	GCA_002590615.1	Gokushovirus WZ-2015a
1758150	GCA_002590635.1	Gokushovirus WZ-2015a
1758150	GCA_002590655.1	Gokushovirus WZ-2015a
1758150	GCA_002590675.1	Gokushovirus WZ-2015a
1758150	GCA_002590695.1	Gokushovirus WZ-2015a

1758150	GCA_002589545.1	Gokushovirus WZ-2015a
1758150	GCA_002590715.1	Gokushovirus WZ-2015a
1758150	GCA_002588925.1	Gokushovirus WZ-2015a
1758150	GCA_002590735.1	Gokushovirus WZ-2015a
1758150	GCA_002590755.1	Gokushovirus WZ-2015a
1758150	GCA_002588945.1	Gokushovirus WZ-2015a
1758150	GCA_002590775.1	Gokushovirus WZ-2015a
1758150	GCA_002590795.1	Gokushovirus WZ-2015a
1758150	GCA_002590815.1	Gokushovirus WZ-2015a
1758150	GCA_002588985.1	Gokushovirus WZ-2015a
1758150	GCA_002590835.1	Gokushovirus WZ-2015a
1758150	GCA_002590855.1	Gokushovirus WZ-2015a
1758150	GCA_002590875.1	Gokushovirus WZ-2015a
1758150	GCA_002589055.1	Gokushovirus WZ-2015a
1758150	GCA_002590895.1	Gokushovirus WZ-2015a
1758150	GCA_002589085.1	Gokushovirus WZ-2015a
1758150	GCA_002590915.1	Gokushovirus WZ-2015a
1758150	GCA_002589115.1	Gokushovirus WZ-2015a
1758150	GCA_002590935.1	Gokushovirus WZ-2015a
1758150	GCA_002590955.1	Gokushovirus WZ-2015a
1758150	GCA_002590975.1	Gokushovirus WZ-2015a
1758150	GCA_002589145.1	Gokushovirus WZ-2015a
1758150	GCA_002590995.1	Gokushovirus WZ-2015a
1758150	GCA_002591015.1	Gokushovirus WZ-2015a
1758150	GCA_002589175.1	Gokushovirus WZ-2015a
1758150	GCA_002589205.1	Gokushovirus WZ-2015a
1758150	GCA_002591035.1	Gokushovirus WZ-2015a
1758150	GCA_002589225.1	Gokushovirus WZ-2015a
1758150	GCA_002591055.1	Gokushovirus WZ-2015a
1758150	GCA_002589265.1	Gokushovirus WZ-2015a
1223562	GCA_000899995.1	Golden Gate virus
1720526	GCA_001806195.1	Golden Marseillevirus
185783	GCA_000853585.1	Golden shiner reovirus
1856030	GCA_001661715.1	Golden shiner totivirus
2293292	GCA_003847045.1	Golden silk orbweaver associated circular virus 1
1884832	GCA_001706965.1	Gompholobium virus A
1193422	GCA_000897155.1	Goose adenovirus 4
1349999	GCA_002146085.1	Goose astrovirus
1493858	GCA_000920715.1	Goose calicivirus
146032	GCA_000837325.1	Goose circovirus
146032	GCA_004032935.1	Goose circovirus
146032	GCA_004035435.1	Goose circovirus
146032	GCA_004032835.1	Goose circovirus
146032	GCA_004035515.1	Goose circovirus
146032	GCA_004035535.1	Goose circovirus
146032	GCA_004035475.1	Goose circovirus
146032	GCA_004035555.1	Goose circovirus
146032	GCA_004030255.1	Goose circovirus
146032	GCA_004034615.1	Goose circovirus
146032	GCA_004032955.1	Goose circovirus

146032	GCA_004034635.1	Goose circovirus
146032	GCA_004035455.1	Goose circovirus
146032	GCA_004035495.1	Goose circovirus
146032	GCA_004034915.1	Goose circovirus
146032	GCA_004049435.1	Goose circovirus
146032	GCA_004060915.1	Goose circovirus
146032	GCA_004049475.1	Goose circovirus
146032	GCA_004061675.1	Goose circovirus
146032	GCA_004046955.1	Goose circovirus
146032	GCA_004046975.1	Goose circovirus
146032	GCA_004046995.1	Goose circovirus
146032	GCA_004047015.1	Goose circovirus
146032	GCA_004047035.1	Goose circovirus
146032	GCA_004047055.1	Goose circovirus
146032	GCA_004047075.1	Goose circovirus
146032	GCA_004047095.1	Goose circovirus
146032	GCA_004047115.1	Goose circovirus
146032	GCA_004047135.1	Goose circovirus
146032	GCA_004047155.1	Goose circovirus
146032	GCA_004047175.1	Goose circovirus
146032	GCA_004047195.1	Goose circovirus
146032	GCA_004047215.1	Goose circovirus
146032	GCA_004047235.1	Goose circovirus
146032	GCA_004047255.1	Goose circovirus
146032	GCA_004047275.1	Goose circovirus
146032	GCA_004047295.1	Goose circovirus
146032	GCA_004047315.1	Goose circovirus
146032	GCA_004047335.1	Goose circovirus
146032	GCA_004047355.1	Goose circovirus
146032	GCA_004049455.1	Goose circovirus
146032	GCA_004049495.1	Goose circovirus
146032	GCA_004064095.1	Goose circovirus
146032	GCA_004064115.1	Goose circovirus
146032	GCA_004064155.1	Goose circovirus
146032	GCA_004064175.1	Goose circovirus
146032	GCA_004065815.1	Goose circovirus
146032	GCA_004082055.1	Goose circovirus
146032	GCA_004065835.1	Goose circovirus
146032	GCA_004082115.1	Goose circovirus
146032	GCA_004064135.1	Goose circovirus
146032	GCA_004082135.1	Goose circovirus
146032	GCA_004082195.1	Goose circovirus
146032	GCA_004082075.1	Goose circovirus
146032	GCA_004065975.1	Goose circovirus
146032	GCA_004082155.1	Goose circovirus
146032	GCA_004082235.1	Goose circovirus
146032	GCA_004082215.1	Goose circovirus
146032	GCA_004082095.1	Goose circovirus
146032	GCA_004082035.1	Goose circovirus
146032	GCA_004065795.1	Goose circovirus

146032	GCA_004082175.1	Goose circovirus
146032	GCA_004065755.1	Goose circovirus
146032	GCA_004064055.1	Goose circovirus
146032	GCA_004065775.1	Goose circovirus
146032	GCA_004064075.1	Goose circovirus
146032	GCA_004320365.1	Goose circovirus
146032	GCA_004320385.1	Goose circovirus
146032	GCA_004320405.1	Goose circovirus
1776109	GCA_001549565.1	Goose dicistrovirus
208491	GCA_000841425.1	Goose hemorrhagic polyomavirus
1960206	GCA_002008395.1	Goose megrivirus
204987	GCA_000852605.1	Goose paramyxovirus SF02
38251	GCA_000839685.1	Goose parvovirus
2079465	GCA_004131145.1	Goose picornavirus 1
157270	GCA_000899795.1	Gooseberry vein banding associated virus
2041419	GCA_003846525.1	Gopherus associated circular DNA virus 1
2041417	GCA_003846545.1	Gopherus associated genomovirus 1
2041417	GCA_003846565.1	Gopherus associated genomovirus 1
2041417	GCA_003846605.1	Gopherus associated genomovirus 1
2041417	GCA_003846585.1	Gopherus associated genomovirus 1
2079413	GCA_002958695.1	Gordonia phage Adgers
2488781	GCA_003866935.1	Gordonia phage Adora
2483664	GCA_003723335.1	Gordonia phage Affeca
2483665	GCA_003723475.1	Gordonia phage Ailee
2301561	GCA_003442195.1	Gordonia phage Ali17
2047829	GCA_002956245.1	Gordonia phage Anamika
2301695	GCA_003442575.1	Gordonia phage Angelicage
2301691	GCA_003442595.1	Gordonia phage Angelique
2507860	GCA_004139075.1	Gordonia phage Aphelion
2250319	GCA_003365615.1	Gordonia phage Apricot
2507862	GCA_004139115.1	Gordonia phage Asapag
2301692	GCA_003442615.1	Gordonia phage Ashertheman
1821548	GCA_002609625.1	Gordonia phage Attis
1838061	GCA_001736795.1	Gordonia phage Bachita
1887641	GCA_001745615.1	Gordonia phage Bantam
2517926	GCA_004325235.1	Gordonia phage Barco
1887642	GCA_002609605.1	Gordonia phage BatStarr
1821549	GCA_001754145.1	Gordonia phage BaxterFox
2079397	GCA_002958545.1	Gordonia phage Beenie
1821550	GCA_002609785.1	Gordonia phage Benczkowski14
2047830	GCA_002956255.1	Gordonia phage BENtherdunthat
1821551	GCA_001754365.1	Gordonia phage BetterKatz
2283245	GCA_003366515.1	Gordonia phage Beyoncage
2484205	GCA_003722575.1	Gordonia phage Bialota
2483666	GCA_003723355.1	Gordonia phage Bibwit
2507861	GCA_004139095.1	Gordonia phage BiPauneto
2047831	GCA_002956265.1	Gordonia phage BirksAndSocks
2483667	GCA_003722815.1	Gordonia phage Bizzy
2047832	GCA_002956275.1	Gordonia phage Bjanes7
1838062	GCA_001737015.1	Gordonia phage Blueberry

2079414	GCA_002958705.1	Gordonia phage Boneham
1838063	GCA_001736335.1	Gordonia phage Bowser
2510501	GCA_004149925.1	Gordonia phage Bradissa
2079564	GCA_002958945.1	Gordonia phage Brandonk123
1838064	GCA_001736555.1	Gordonia phage BritBrat
2530116	GCA_004521015.1	Gordonia phage BrutonGaster
2507859	GCA_004139055.1	Gordonia phage Brylie
2315529	GCA_003614015.1	Gordonia phage Buggaboo
2510502	GCA_004149885.1	Gordonia phage Butterball
1887643	GCA_001744295.1	Gordonia phage CaptainKirk2
1887644	GCA_001743615.1	Gordonia phage CarolAnn
2301538	GCA_003441955.1	Gordonia phage Catfish
1838065	GCA_001736775.1	Gordonia phage ClubL
2081621	GCA_002990335.1	Gordonia phage Confidence
1838066	GCA_001736995.1	Gordonia phage Cozz
1887645	GCA_001744935.1	Gordonia phage Cucurbita
2250390	GCA_003364875.1	Gordonia phage Danyall
2283286	GCA_003366935.1	Gordonia phage Daredevil
2250288	GCA_003365255.1	Gordonia phage DelRio
1838067	GCA_001736315.1	Gordonia phage Demosthenes
1958908	GCA_002619205.1	Gordonia phage DinoDaryn
2126784	GCA_003024135.1	Gordonia phage Djokovic
2530117	GCA_004338875.1	Gordonia phage Dogfish
2499023	GCA_004006995.1	Gordonia phage Dorito
2507858	GCA_004139035.1	Gordonia phage Duffington
2182395	GCA_003183225.1	Gordonia phage Easley
2201426	GCA_003307695.1	Gordonia phage Ebert
1821552	GCA_001755225.1	Gordonia phage Emalyn
2315530	GCA_003614055.1	Gordonia phage Emianna
2201427	GCA_003307715.1	Gordonia phage Emperor
2510503	GCA_004149945.1	Gordonia phage EMsquaredA
2510504	GCA_004149865.1	Gordonia phage Exiguo
2488782	GCA_003867035.1	Gordonia phage Eyes
1887646	GCA_001745595.1	Gordonia phage Eyre
2510505	GCA_004149825.1	Gordonia phage FelixAlejandro
2126808	GCA_003024025.1	Gordonia phage Fenry
2079280	GCA_002958395.1	Gordonia phage Flakey
2079415	GCA_002958715.1	Gordonia phage Flapper
2483668	GCA_003722835.1	Gordonia phage Fosterous
2301602	GCA_003601095.1	Gordonia phage Foxboro
2250320	GCA_003365735.1	Gordonia phage Frokostdame
2250392	GCA_003423245.1	Gordonia phage Fryberger
2250321	GCA_003365755.1	Gordonia phage Fury
2483669	GCA_003722855.1	Gordonia phage Gaea
1647469	GCA_001884535.1	Gordonia phage GAL1
2250393	GCA_003364935.1	Gordonia phage GEazy
2483670	GCA_003722875.1	Gordonia phage Geodirt
2315531	GCA_003614115.1	Gordonia phage Getalong
1887647	GCA_001744275.1	Gordonia phage Ghobes
1647470	GCA_001737095.1	Gordonia phage GMA1

1647283	GCA_002605765.1	Gordonia phage GMA2
1647284	GCA_001470695.1	Gordonia phage GMA3
1647471	GCA_001736415.1	Gordonia phage GMA4
1647472	GCA_001736635.1	Gordonia phage GMA5
1647285	GCA_001736875.1	Gordonia phage GMA6
1647286	GCA_001470395.1	Gordonia phage GMA7
1622190	GCA_001502075.1	Gordonia phage Gmala1
1622191	GCA_001551065.1	Gordonia phage GordDuk1
1622192	GCA_001550685.1	Gordonia phage GordTnk2
2094133	GCA_003014305.1	Gordonia phage Gravy
2499024	GCA_004007195.1	Gordonia phage Gray
1109710	GCA_000896515.1	Gordonia phage GRU1
1647473	GCA_001736855.1	Gordonia phage GRU3
1622193	GCA_001736655.1	Gordonia phage Gsput1
981323	GCA_000892855.1	Gordonia phage GTE2
319522	GCA_000894075.1	Gordonia phage GTE5
1647474	GCA_001470375.1	Gordonia phage GTE6
1100814	GCA_000894035.1	Gordonia phage GTE7
1647475	GCA_001470895.1	Gordonia phage GTE8
1821553	GCA_001755645.1	Gordonia phage Guacamole
2510570	GCA_004149725.1	Gordonia phage Guillaume
2047872	GCA_002956505.1	Gordonia phage Gustav
2126785	GCA_003024125.1	Gordonia phage Hail2Pitt
2510575	GCA_004149745.1	Gordonia phage Harambe
1887648	GCA_001743595.1	Gordonia phage Hedwig
2510573	GCA_004149765.1	Gordonia phage Hello
2301696	GCA_003442775.1	Gordonia phage Horus
1821554	GCA_001754785.1	Gordonia phage Hotorobo
1777061	GCA_002609045.1	Gordonia phage Howe
1958909	GCA_002619225.1	Gordonia phage Huffy
2510506	GCA_004149905.1	Gordonia phage IDyn
1838068	GCA_002609805.1	Gordonia phage Jeanie
2315532	GCA_003614295.1	Gordonia phage Jifall16
2517931	GCA_004520495.1	Gordonia phage Jormungandr
1887649	GCA_001744915.1	Gordonia phage JSwag
1887650	GCA_001745575.1	Gordonia phage Jumbo
2041511	GCA_002744155.1	Gordonia phage Kabluna
1838069	GCA_002757635.1	Gordonia phage Kampe
1838070	GCA_001698415.1	Gordonia phage KatherineG
1821555	GCA_002622945.1	Gordonia phage Katyusha
2499025	GCA_004008895.1	Gordonia phage Kenna
2094134	GCA_003014315.1	Gordonia phage Kerry
2301603	GCA_003601175.1	Gordonia phage KidneyBean
2500571	GCA_004015745.1	Gordonia phage Kiko
2250394	GCA_003364975.1	Gordonia phage KimmyK
1821556	GCA_001754345.1	Gordonia phage Kita
2483671	GCA_003722895.1	Gordonia phage Kroos
2315533	GCA_003614335.1	Gordonia phage Kurt
1838071	GCA_001736535.1	Gordonia phage Kvothe
2517932	GCA_004520555.1	Gordonia phage Lahirium

2250295	GCA_003365375.1	Gordonia phage LastResort
2041512	GCA_002744185.1	Gordonia phage Lennon
2500572	GCA_004015725.1	Gordonia phage Lilas
2041513	GCA_002744215.1	Gordonia phage Lozinak
1821557	GCA_001755205.1	Gordonia phage Lucky10
2041514	GCA_002744255.1	Gordonia phage Lysidious
2047873	GCA_002956515.1	Gordonia phage Mahdia
2201428	GCA_003307835.1	Gordonia phage Margaret
2488957	GCA_003868375.1	Gordonia phage Maridalia
2301558	GCA_003442335.1	Gordonia phage Marietta
1838072	GCA_001736755.1	Gordonia phage McGonagall
1838073	GCA_001736975.1	Gordonia phage Monty
2201429	GCA_003307895.1	Gordonia phage Morrissey
2510507	GCA_004149805.1	Gordonia phage Msay19
2500788	GCA_004149625.1	Gordonia phage Mulch
2500789	GCA_004149645.1	Gordonia phage Mutzi
2250369	GCA_003341655.1	Gordonia phage Nadeem
2250322	GCA_003365855.1	Gordonia phage NatB6
2234025	GCA_003308315.1	Gordonia phage Nedarya
2510574	GCA_004149785.1	Gordonia phage Neoevie
2301693	GCA_003442895.1	Gordonia phage Neville
2517933	GCA_004520795.1	Gordonia phage Nimi13
2499026	GCA_004008225.1	Gordonia phage Nina
2483672	GCA_003722915.1	Gordonia phage Nordenberg
2507863	GCA_004139135.1	Gordonia phage NosilaM
1887651	GCA_001744255.1	Gordonia phage Nyceirae
1821558	GCA_001745275.1	Gordonia phage Nymphadora
1821559	GCA_001755625.1	Gordonia phage Obliviate
2483673	GCA_003722935.1	Gordonia phage Octobien14
1838074	GCA_001736295.1	Gordonia phage OneUp
1838075	GCA_001736515.1	Gordonia phage Orchid
2502437	GCA_004149705.1	Gordonia phage Parada
2041515	GCA_002956095.1	Gordonia phage Patio
1838076	GCA_002757655.1	Gordonia phage PatrickStar
2182347	GCA_003183065.1	Gordonia phage Petra
1821560	GCA_001745935.1	Gordonia phage Phinally
2301694	GCA_003442955.1	Gordonia phage Phistory
2283246	GCA_003366275.1	Gordonia phage Pleakley
2250296	GCA_003365475.1	Gordonia phage Pollux
1887652	GCA_001743575.1	Gordonia phage Remus
2250417	GCA_003364755.1	Gordonia phage Ribeye
2507854	GCA_004138975.1	Gordonia phage Rickmore
2250395	GCA_003365095.1	Gordonia phage RobinSparkles
2250396	GCA_003365115.1	Gordonia phage Rofo
2250397	GCA_003423265.1	Gordonia phage Ronaldo
1838077	GCA_001698375.1	Gordonia phage Rosalind
2250323	GCA_003365895.1	Gordonia phage Ruthy
2079570	GCA_002997425.1	Gordonia phage SallySpecial
2499027	GCA_004008575.1	Gordonia phage Savage
2301697	GCA_003443035.1	Gordonia phage Schmidt

1821561	GCA_001754765.2	Gordonia phage Schnabeltier
1821561	GCA_001754765.1	Gordonia phage Schwabeltier
2027889	GCA_002629325.1	Gordonia phage ShayRa
2182348	GCA_003258215.1	Gordonia phage Sitar
2250418	GCA_003364815.1	Gordonia phage SketchMex
2301559	GCA_003442455.1	Gordonia phage Skysand
1838078	GCA_001698335.1	Gordonia phage Smoothie
1821562	GCA_001754325.1	Gordonia phage SoilAssassin
2502420	GCA_004149665.1	Gordonia phage Sombrero
1838079	GCA_001698275.1	Gordonia phage Soups
2182349	GCA_003183085.1	Gordonia phage Sour
1838080	GCA_001736735.1	Gordonia phage Splinter
2499028	GCA_004008535.1	Gordonia phage Sproutie
2079281	GCA_002958405.1	Gordonia phage SteveFrench
1887653	GCA_002612145.1	Gordonia phage Strosahl
2483674	GCA_003722995.1	Gordonia phage Stultus
2079416	GCA_002958725.1	Gordonia phage SuperSulley
2201430	GCA_003308055.1	Gordonia phage Suzy
2483675	GCA_003723495.1	Gordonia phage Tangent
2283247	GCA_003366595.1	Gordonia phage Teatealatte
1887654	GCA_001744815.1	Gordonia phage Terapin
2510508	GCA_004149845.1	Gordonia phage Tiamoceli
2301560	GCA_003442495.1	Gordonia phage TillyBobJoe
2041516	GCA_002956105.1	Gordonia phage Toniann
2517934	GCA_004520355.1	Gordonia phage Tredge
2201431	GCA_003308155.1	Gordonia phage Trine
2079282	GCA_002958415.1	Gordonia phage Troje
2315610	GCA_003613895.1	Gordonia phage Turuncu
1887655	GCA_001744135.1	Gordonia phage Twister6
1821563	GCA_001755185.1	Gordonia phage UmaThurman
1838081	GCA_001737135.1	Gordonia phage Utz
2502431	GCA_004149685.1	Gordonia phage Vasanti
1838082	GCA_001736455.1	Gordonia phage Vendetta
1821564	GCA_001755605.1	Gordonia phage Vivi2
2108120	GCA_003013975.1	Gordonia phage Waits
2517927	GCA_004325215.1	Gordonia phage Walrus
2499029	GCA_004008015.1	Gordonia phage WheatThin
2507855	GCA_004138995.1	Gordonia phage WhoseManz
2507857	GCA_004139215.1	Gordonia phage WilliamBoone
1838083	GCA_001736675.1	Gordonia phage Wizard
1838084	GCA_001736895.1	Gordonia phage Woes
1821565	GCA_001754745.1	Gordonia phage Yeezy
1821566	GCA_001754305.1	Gordonia phage Yvonnetastic
2283248	GCA_003366455.1	Gordonia phage Zarbodnamra
1887656	GCA_001745455.1	Gordonia phage Zirinka
1743411	GCA_001689855.1	Gorilla anellovirus
2170111	GCA_000929415.1	Gorilla associated porprismacovirus 1
928214	GCA_000924615.1	Gorilla gorilla gorilla polyomavirus 1
1603068	GCA_003963855.1	Gorilla smacovirus
656025	GCA_000886575.1	Gossypium darwinii symptomless alphasatellite

656025	GCA_003028915.1	Gossypium darwinii symptomless alphasatellite
509531	GCA_000881015.1	Gossypium darwinii symptomless virus
656028	GCA_000885695.1	Gossypium davidsonii symptomless alphasatellite
2169907	GCA_003028925.1	Gossypium mustelinum symptomless alphasatellite
509532	GCA_000882615.1	Gossypium punctatum mild leaf curl virus
603003	GCA_002814655.1	Gouleako virus
1560351	GCA_000926435.1	Goutanap virus
1629132	GCA_000960945.1	Graminella nigrifrons virus 1
208084	GCA_000883275.1	Grapevine Algerian latent virus
223769	GCA_000897495.1	Grapevine Anatolian ringspot virus
908834	GCA_001461205.1	Grapevine associated narnavirus-1
2338396	GCA_004134105.1	Grapevine associated tymo-like virus
2169998	GCA_001856635.1	Grapevine asteroid mosaic associated virus
81877	GCA_000893215.1	Grapevine berry inner necrosis virus
748667	GCA_000892035.1	Grapevine Bulgarian latent virus
1640277	GCA_002375125.2	Grapevine Cabernet Sauvignon reovirus
12273	GCA_000862025.1	Grapevine chrome mosaic virus
233784	GCA_000898115.1	Grapevine deformation virus
2560515	GCA_002184215.1	Grapevine enamovirus 1
1249676	GCA_000902555.1	Grapevine endophyte alphaendornavirus
1849838	GCA_003033815.1	Grapevine fabavirus
12274	GCA_000860305.1	Grapevine fanleaf virus
141860	GCA_000855465.1	Grapevine fanleaf virus satellite RNA
103722	GCA_000859005.1	Grapevine fleck virus
1906317	GCA_001766605.1	Grapevine geminivirus A
47985	GCA_000895715.1	Grapevine leafroll-associated virus 1
367121	GCA_000883475.1	Grapevine leafroll-associated virus 10
1815581	GCA_001605795.1	Grapevine leafroll-associated virus 13
64003	GCA_000864185.1	Grapevine leafroll-associated virus 2
55951	GCA_000851885.1	Grapevine leafroll-associated virus 3
70177	GCA_000894055.1	Grapevine leafroll-associated virus 4
71032	GCA_000894915.1	Grapevine leafroll-associated virus 5
203168	GCA_000895655.1	Grapevine leafroll-associated virus 6
217615	GCA_000895675.1	Grapevine leafroll-associated virus 7
1051792	GCA_000894735.2	Grapevine Pinot gris virus
1051792	GCA_000894735.1	Grapevine Pinot gris virus
1381007	GCA_000909815.1	Grapevine red blotch virus
1381007	GCA_000898075.1	Grapevine red blotch virus
103723	GCA_001698255.1	Grapevine Red Globe virus
1471299	GCA_001019755.1	Grapevine Roditis leaf discoloration-associated virus
167634	GCA_000851765.1	Grapevine rootstock stem lesion associated virus
81832	GCA_000860125.1	Grapevine rupestris stem pitting-associated virus 1
204933	GCA_002029535.1	Grapevine rupestris vein feathering virus
1343493	GCA_000910175.1	Grapevine satellite virus
630199	GCA_000882775.1	Grapevine Syrah virus 1
1050407	GCA_000891255.1	Grapevine vein clearing virus
35288	GCA_000855125.1	Grapevine virus A
35289	GCA_000857765.1	Grapevine virus B
516956	GCA_000881395.1	Grapevine virus E
1221437	GCA_000899135.1	Grapevine virus F

2022475	GCA_004131225.1	Grapevine virus G
2022475	GCA_004132005.1	Grapevine virus G
2045345	GCA_004131305.1	Grapevine virus H
2052157	GCA_002937185.1	Grapevine virus I
2093496	GCA_004132725.1	Grapevine virus J
2016034	GCA_002219425.1	Grapevine virus K
2016035	GCA_002219965.1	Grapevine virus T
12904	GCA_000854565.1	Grapevine yellow speckle viroid 1
46342	GCA_000850565.1	Grapevine yellow speckle viroid 2
1288359	GCA_000924575.1	Grass carp virus
2293293	GCA_003847455.1	Grasshopper associated circular virus 1
1093101	GCA_002827185.1	Gray fox amdovirus
1272942	GCA_002145965.1	Gray Lodge virus
204269	GCA_000887635.1	Great Island virus
1810946	GCA_004128035.1	Great Saltee virus
279538	GCA_000860045.1	Gremmeniella abietina mitochondrial RNA virus S2
152217	GCA_000851525.1	Gremmeniella abietina RNA virus L1
279539	GCA_000858445.1	Gremmeniella abietina RNA virus L2
191436	GCA_000853165.1	Gremmeniella abietina RNA virus MS1
279540	GCA_000859225.1	Gremmeniella abietina RNA virus MS2
374005	GCA_000867145.1	Gremmeniella abietina type B RNA virus XL1
2170196	GCA_000874485.1	Grivet simian foamy virus
10406	GCA_000837845.1	Ground squirrel hepatitis virus
1027232	GCA_000892015.1	Groundnut ringspot and Tomato chlorotic spot virus reasso
12675	GCA_003972785.1	Groundnut ringspot virus
47740	GCA_000851225.1	Groundnut rosette virus
127441	GCA_000845525.1	Groundnut rosette virus satellite RNA
432587	GCA_000870665.1	Gryllus bimaculatus nudivirus
1933272	GCA_002831345.1	Guajara orthobunyavirus
1678234	GCA_002831365.1	Guama virus
45219	GCA_000853765.1	Guanarito mammarenavirus
2306813	GCA_004117355.1	Guangxi orbivirus
748170	GCA_000905935.1	Guar leaf curl alphasatellite
80941	GCA_002118805.1	Guaroa virus
80941	GCA_003333065.1	Guaroa virus
80941	GCA_003333025.1	Guaroa virus
80941	GCA_003332945.1	Guaroa virus
80941	GCA_003332965.1	Guaroa virus
80941	GCA_003333105.1	Guaroa virus
80941	GCA_003333085.1	Guaroa virus
80941	GCA_003332905.1	Guaroa virus
80941	GCA_003332985.1	Guaroa virus
80941	GCA_003333045.1	Guaroa virus
80941	GCA_003333005.1	Guaroa virus
80941	GCA_003332925.1	Guaroa virus
2170197	GCA_003032775.1	Guenon simian foamy virus
1354498	GCA_001885465.1	Guereza hepacivirus
1354498	GCA_002820965.1	Guereza hepacivirus
90963	GCA_000836805.1	Guinea pig Chlamydia phage
400121	GCA_000870205.1	Gull circovirus

400121	GCA_004059775.1	Gull circovirus
400121	GCA_004059795.1	Gull circovirus
400121	GCA_004059815.1	Gull circovirus
400121	GCA_004080995.1	Gull circovirus
2218660	GCA_003260835.1	Gyrovirus 10
1214955	GCA_000899075.1	Gyrovirus 4
1163715	GCA_000896975.1	Gyrovirus GyV3
1548711	GCA_000924395.1	Gyrovirus GyV7-SF
1670973	GCA_001045285.1	Gyrovirus GyV8
1415627	GCA_000913875.1	Gyrovirus Tu243
1415628	GCA_000912415.1	Gyrovirus Tu789
10799	GCA_000848765.1	H-1 parvovirus
1672380	GCA_003032615.1	Haartman Institute snake virus
1324128	GCA_000908975.1	Habenaria mosaic virus
230158	GCA_000859205.1	Haemophilus phage Aaphi23
483266	GCA_000903175.1	Haemophilus phage SuMu
10690	GCA_000837885.1	Haemophilus virus HP1
157239	GCA_000842865.1	Haemophilus virus HP2
1088890	GCA_000895035.1	Halastavi arva RNA virus
2480177	GCA_004132305.1	Halhan virus 2
2480178	GCA_004132325.1	Halhan virus 3
1735722	GCA_001717355.1	Haloarcula californiae icosahedral virus 1
1154689	GCA_000896015.1	Haloarcula hispanica icosahedral virus 2
710112	GCA_000884635.1	Haloarcula hispanica pleomorphic virus 1
1442594	GCA_000914515.1	Haloarcula hispanica pleomorphic virus 2
1879051	GCA_002989875.1	Haloarcula hispanica pleomorphic virus 3
1980140	GCA_003012505.1	Haloarcula hispanica pleomorphic virus 4
1282967	GCA_000908235.1	Haloarcula hispanica virus PH1
326574	GCA_000864025.1	Haloarcula hispanica virus SH1
169684	GCA_003687545.1	Halobacterium phage phiH
2496992	GCA_004338415.1	Halobacterium virus ChaoS9
1173297	GCA_000897215.1	Halocynthia phage JM-2012
2507575	GCA_004208775.1	Haloferax tailed virus 1
1156722	GCA_000895495.1	Halogeometricum pleomorphic virus 1
1913108	GCA_002615065.1	Halomonas phage QHHSV-1
499235	GCA_000879135.1	Halomonas virus HAP1
754066	GCA_000909335.1	Halorubrum phage CGphi46
33771	GCA_000839925.1	Halorubrum phage HF2
634168	GCA_000883755.1	Halorubrum pleomorphic virus 1
2507576	GCA_004208755.1	Halorubrum pleomorphic virus 10
2507577	GCA_004208795.1	Halorubrum pleomorphic virus 11
2507578	GCA_004208735.1	Halorubrum pleomorphic virus 12
1156719	GCA_000896955.1	Halorubrum pleomorphic virus 2
1156720	GCA_000894515.1	Halorubrum pleomorphic virus 3
1156721	GCA_000896115.1	Halorubrum pleomorphic virus 6
2126525	GCA_004291235.1	Halorubrum pleomorphic virus 9
1273746	GCA_000909275.1	Halovirus HCTV-1
1273747	GCA_000907655.1	Halovirus HCTV-2
1273748	GCA_000910135.1	Halovirus HCTV-5
222645	GCA_000841465.1	Halovirus HF1

1273749	GCA_000908655.1	Halovirus HGTV-1
1273750	GCA_000909255.1	Halovirus HHTV-1
1273751	GCA_000907635.1	Halovirus HHTV-2
1273752	GCA_000910115.1	Halovirus HRTV-4
1273753	GCA_000908635.1	Halovirus HRTV-5
1273754	GCA_000909235.1	Halovirus HRTV-7
1273755	GCA_000907615.1	Halovirus HRTV-8
1262530	GCA_000907675.1	Halovirus HSTV-1
1262527	GCA_000905695.1	Halovirus HSTV-2
1262528	GCA_000905075.1	Halovirus HVTV-1
1500510	GCA_000924375.1	Halovirus VNH-1
1410467	GCA_000911155.1	Halyomorpha halys virus
67571	GCA_000837745.1	Hamiltonella virus APSE1
1286140	GCA_000906255.1	Hana virus
1125677	GCA_001678195.1	Hanko virus
1980471	GCA_000856085.1	Hantaan orthohantavirus
93830	GCA_000855785.1	Hantavirus Z10
409486	GCA_000890955.1	Hardenbergia mosaic virus
1003891	GCA_000892595.1	Hardenbergia virus A
1987722	GCA_002184175.1	Harrier picornavirus 1
200401	GCA_002118985.1	Hart Park virus
70564	GCA_001502715.1	Hawaiian green turtle herpesvirus
11596	GCA_002831085.1	Hazara virus
489460	GCA_003114295.1	HBV genotype B
489483	GCA_900157455.1	HBV genotype D
489483	GCA_900157435.1	HBV genotype D
489483	GCA_900157445.1	HBV genotype D
489491	GCA_900232405.1	HBV genotype F
489491	GCA_900232365.1	HBV genotype F
489491	GCA_900232385.1	HBV genotype F
489491	GCA_900232395.1	HBV genotype F
489491	GCA_900232375.1	HBV genotype F
489491	GCA_900232415.1	HBV genotype F
489491	GCA_900232425.1	HBV genotype F
489491	GCA_900232435.1	HBV genotype F
489491	GCA_900232445.1	HBV genotype F
489491	GCA_900232455.1	HBV genotype F
489491	GCA_900232465.1	HBV genotype F
489491	GCA_900232475.1	HBV genotype F
1516079	GCA_000921475.1	HCBI8.215 virus
1516080	GCA_000923995.1	HCBI9.212 virus
1216928	GCA_000922255.1	Heartland virus
2041149	GCA_004131005.1	Hedgehog dicipivirus
1987144	GCA_001444085.1	Hedgehog hepatovirus Igel8Erieur2014
1428190	GCA_002822405.1	Hedyotis uncinella yellow mosaic virus
1428189	GCA_000915855.1	Hedyotis yellow mosaic betasatellite
2184469	GCA_004131165.1	Helianthus annuus alphaendornavirus
1154995	GCA_000903415.1	Helicobacter phage 1961P
1852661	GCA_002610205.1	Helicobacter phage DeM53M
1852662	GCA_002610225.1	Helicobacter phage FrANT170U

1852663	GCA_002610245.1	Helicobacter phage FrB41M
1852664	GCA_002610265.1	Helicobacter phage FrB58M
1852665	GCA_002610285.1	Helicobacter phage FrG12G
1852666	GCA_002610305.1	Helicobacter phage FrGC43A
1852667	GCA_002610325.1	Helicobacter phage FrMEG235U
1208236	GCA_000902955.1	Helicobacter phage KHP30
1204178	GCA_000902935.1	Helicobacter phage KHP40
1069378	GCA_000894175.1	Helicobacter phage phiHP33
1852669	GCA_002610345.1	Helicobacter phage Pt1293U
1852670	GCA_002610365.1	Helicobacter phage Pt1846U
1852671	GCA_002610385.1	Helicobacter phage Pt1918U
1852672	GCA_002610405.1	Helicobacter phage Pt21299RU
1852673	GCA_002610425.1	Helicobacter phage Pt22899G
1852675	GCA_002610445.1	Helicobacter phage Pt4472G
1852676	GCA_002610465.1	Helicobacter phage Pt4481G
1852677	GCA_002610485.1	Helicobacter phage Pt4497U
1852679	GCA_002610505.1	Helicobacter phage Pt5322G
1852680	GCA_002610525.1	Helicobacter phage Pt5771G
1852681	GCA_002610545.1	Helicobacter phage PtB89G
1852682	GCA_002610565.1	Helicobacter phage PtB92G
1852683	GCA_002610585.1	Helicobacter phage Sw577G
1852684	GCA_002610605.1	Helicobacter phage SwA626G
1852687	GCA_002610625.1	Helicobacter phage UKEN31U
1852688	GCA_002610645.1	Helicobacter phage UKEN32U
675833	GCA_000886015.1	Helicobasidium mompa alphaendornavirus 1
196690	GCA_000853525.1	Helicobasidium mompa totivirus 1-17
196690	GCA_000853525.3	Helicobasidium mompa totivirus 1-17
1487185	GCA_000918155.1	Heliconius erato iflavirus
1045778	GCA_000893755.1	Helicoverpa armigera densovirus
489830	GCA_000872285.1	Helicoverpa armigera granulovirus
1936269	GCA_002004415.1	Helicoverpa armigera iflavirus
566972	GCA_000883175.1	Helicoverpa armigera NPV NNg1
51313	GCA_000849765.1	Helicoverpa armigera nucleopolyhedrovirus
148363	GCA_000838025.1	Helicoverpa armigera nucleopolyhedrovirus G4
37206	GCA_000849105.1	Helicoverpa armigera stunt virus
1128424	GCA_000841925.1	Helicoverpa zea nudivirus 2
332585	GCA_000875125.1	Heliothis armigera cypovirus 5
260797	GCA_000871485.1	Heliothis virescens ascovirus 3e
328614	GCA_002818865.1	Heliothis virescens ascovirus 3f
1246651	GCA_002818885.1	Heliothis virescens ascovirus 3g
29250	GCA_002826785.1	Heliothis zea nudivirus
592206	GCA_000883575.1	Helleborus net necrosis virus
164750	GCA_000855265.1	Helminthosporium victoriae 145S virus
45237	GCA_000852005.1	Helminthosporium victoriae virus 190S
1383052	GCA_000911895.1	Hemidesmus yellow mosaic virus
1367203	GCA_000909795.1	Hemileuca sp. nucleopolyhedrovirus
63330	GCA_000852685.1	Hendra henipavirus
2008762	GCA_002820785.1	Hepacivirus B
11103	GCA_002820865.1	Hepacivirus C
11103	GCA_002820845.1	Hepacivirus C

11103	GCA_002820885.1	Hepacivirus C
11103	GCA_003106465.1	Hepacivirus C
11103	GCA_003106485.1	Hepacivirus C
11103	GCA_003105785.1	Hepacivirus C
11103	GCA_003106585.1	Hepacivirus C
11103	GCA_003106605.1	Hepacivirus C
11103	GCA_003106625.1	Hepacivirus C
11103	GCA_003106645.1	Hepacivirus C
11103	GCA_003106685.1	Hepacivirus C
11103	GCA_003106705.1	Hepacivirus C
11103	GCA_003106725.1	Hepacivirus C
11103	GCA_003106765.1	Hepacivirus C
11103	GCA_003106785.1	Hepacivirus C
11103	GCA_003107205.1	Hepacivirus C
11103	GCA_003106845.1	Hepacivirus C
11103	GCA_003106865.1	Hepacivirus C
11103	GCA_003105845.1	Hepacivirus C
11103	GCA_003106885.1	Hepacivirus C
11103	GCA_003106925.1	Hepacivirus C
11103	GCA_003106965.1	Hepacivirus C
11103	GCA_003106985.1	Hepacivirus C
11103	GCA_003107005.1	Hepacivirus C
11103	GCA_003107045.1	Hepacivirus C
11103	GCA_003107065.1	Hepacivirus C
11103	GCA_003107085.1	Hepacivirus C
11103	GCA_003107125.1	Hepacivirus C
11103	GCA_003107165.1	Hepacivirus C
11103	GCA_003107245.1	Hepacivirus C
11103	GCA_003107265.1	Hepacivirus C
11103	GCA_003107285.1	Hepacivirus C
11103	GCA_003107325.1	Hepacivirus C
11103	GCA_003107345.1	Hepacivirus C
11103	GCA_003107365.1	Hepacivirus C
11103	GCA_003105345.1	Hepacivirus C
11103	GCA_003107425.1	Hepacivirus C
11103	GCA_003105385.1	Hepacivirus C
11103	GCA_003107445.1	Hepacivirus C
11103	GCA_003105405.1	Hepacivirus C
11103	GCA_003105425.1	Hepacivirus C
11103	GCA_003105445.1	Hepacivirus C
11103	GCA_003107565.1	Hepacivirus C
11103	GCA_003107625.1	Hepacivirus C
11103	GCA_003105585.1	Hepacivirus C
11103	GCA_003107645.1	Hepacivirus C
11103	GCA_003105605.1	Hepacivirus C
11103	GCA_003107665.1	Hepacivirus C
11103	GCA_003105625.1	Hepacivirus C
11103	GCA_003105645.1	Hepacivirus C
11103	GCA_003107705.1	Hepacivirus C
11103	GCA_003105665.1	Hepacivirus C

11103	GCA_003105685.1	Hepacivirus C
11103	GCA_003117855.1	Hepacivirus C
11103	GCA_003105725.1	Hepacivirus C
11103	GCA_003105745.1	Hepacivirus C
11103	GCA_003105765.1	Hepacivirus C
11103	GCA_003106345.1	Hepacivirus C
11103	GCA_003105805.1	Hepacivirus C
11103	GCA_003105825.1	Hepacivirus C
11103	GCA_003107885.1	Hepacivirus C
11103	GCA_003108405.1	Hepacivirus C
11103	GCA_003105865.1	Hepacivirus C
11103	GCA_003105885.1	Hepacivirus C
11103	GCA_003105905.1	Hepacivirus C
11103	GCA_003107965.1	Hepacivirus C
11103	GCA_003105925.1	Hepacivirus C
11103	GCA_003105945.1	Hepacivirus C
11103	GCA_003108005.1	Hepacivirus C
11103	GCA_003105965.1	Hepacivirus C
11103	GCA_003117715.1	Hepacivirus C
11103	GCA_003105985.1	Hepacivirus C
11103	GCA_003108065.1	Hepacivirus C
11103	GCA_003117895.1	Hepacivirus C
11103	GCA_003105705.1	Hepacivirus C
11103	GCA_003108085.1	Hepacivirus C
11103	GCA_003105365.1	Hepacivirus C
11103	GCA_003108105.1	Hepacivirus C
11103	GCA_003108125.1	Hepacivirus C
11103	GCA_003108145.1	Hepacivirus C
11103	GCA_003108165.1	Hepacivirus C
11103	GCA_003108205.1	Hepacivirus C
11103	GCA_003106165.1	Hepacivirus C
11103	GCA_003108225.1	Hepacivirus C
11103	GCA_003106185.1	Hepacivirus C
11103	GCA_003108245.1	Hepacivirus C
11103	GCA_003108465.1	Hepacivirus C
11103	GCA_003108265.1	Hepacivirus C
11103	GCA_003107785.1	Hepacivirus C
11103	GCA_003108285.1	Hepacivirus C
11103	GCA_003106425.1	Hepacivirus C
11103	GCA_003108305.1	Hepacivirus C
11103	GCA_003106265.1	Hepacivirus C
11103	GCA_003108325.1	Hepacivirus C
11103	GCA_003106285.1	Hepacivirus C
11103	GCA_003108345.1	Hepacivirus C
11103	GCA_003108365.1	Hepacivirus C
11103	GCA_003108385.1	Hepacivirus C
11103	GCA_003106365.1	Hepacivirus C
11103	GCA_003108425.1	Hepacivirus C
11103	GCA_003106385.1	Hepacivirus C
11103	GCA_003108445.1	Hepacivirus C

11103	GCA_003106445.1	Hepacivirus C
2008764	GCA_000904775.1	Hepacivirus E
2008765	GCA_002820985.1	Hepacivirus F
2008766	GCA_000929615.1	Hepacivirus G
2008767	GCA_000925175.1	Hepacivirus H
2008768	GCA_002821005.1	Hepacivirus I
2008769	GCA_002821025.1	Hepacivirus J
2008770	GCA_002821045.1	Hepacivirus K
2008771	GCA_002375095.1	Hepacivirus L
2008772	GCA_002821065.1	Hepacivirus M
2008773	GCA_002821085.1	Hepacivirus N
2202225	GCA_004132385.1	Hepacivirus P
10407	GCA_000861825.2	Hepatitis B virus
10407	GCA_003047375.1	Hepatitis B virus
10407	GCA_003047955.1	Hepatitis B virus
10407	GCA_003052205.1	Hepatitis B virus
10407	GCA_003047935.1	Hepatitis B virus
10407	GCA_003047475.1	Hepatitis B virus
10407	GCA_003047395.1	Hepatitis B virus
10407	GCA_003047895.1	Hepatitis B virus
10407	GCA_003047415.1	Hepatitis B virus
10407	GCA_003047435.1	Hepatitis B virus
10407	GCA_003047455.1	Hepatitis B virus
10407	GCA_003033045.1	Hepatitis B virus
10407	GCA_003033055.1	Hepatitis B virus
10407	GCA_003033065.1	Hepatitis B virus
10407	GCA_003033085.1	Hepatitis B virus
10407	GCA_003114275.1	Hepatitis B virus
10407	GCA_003114175.1	Hepatitis B virus
10407	GCA_003114375.1	Hepatitis B virus
10407	GCA_003114205.1	Hepatitis B virus
10407	GCA_003114495.1	Hepatitis B virus
10407	GCA_003114395.1	Hepatitis B virus
10407	GCA_003114525.1	Hepatitis B virus
10407	GCA_003114315.1	Hepatitis B virus
10407	GCA_003114555.1	Hepatitis B virus
10407	GCA_003114355.1	Hepatitis B virus
10407	GCA_003114575.1	Hepatitis B virus
10407	GCA_003114475.1	Hepatitis B virus
10407	GCA_003114255.1	Hepatitis B virus
10407	GCA_003114235.1	Hepatitis B virus
10407	GCA_003114415.1	Hepatitis B virus
10407	GCA_900324095.1	Hepatitis B virus
10407	GCA_900324185.1	Hepatitis B virus
10407	GCA_900324175.1	Hepatitis B virus
10407	GCA_900324085.1	Hepatitis B virus
10407	GCA_900324125.1	Hepatitis B virus
10407	GCA_900324115.1	Hepatitis B virus
10407	GCA_900324155.1	Hepatitis B virus
10407	GCA_900324075.1	Hepatitis B virus

10407	GCA_900324145.1	Hepatitis B virus
10407	GCA_900324195.1	Hepatitis B virus
10407	GCA_900324105.1	Hepatitis B virus
106821	GCA_003033035.1	Hepatitis B virus subtype adw
106821	GCA_003114435.1	Hepatitis B virus subtype adw
356391	GCA_003107525.1	Hepatitis C virus (isolate 6a33)
356413	GCA_003106125.1	Hepatitis C virus (isolate BEBE1)
356388	GCA_003106245.1	Hepatitis C virus (isolate D54)
356419	GCA_000873605.1	Hepatitis C virus (isolate EUH1480)
356420	GCA_002986315.1	Hepatitis C virus (isolate EUHK2)
63746	GCA_002820805.1	Hepatitis C virus (isolate H77)
356410	GCA_003105545.1	Hepatitis C virus (isolate HC-G9)
356416	GCA_003106505.1	Hepatitis C virus (isolate HCV-K3a/650)
356386	GCA_003105565.1	Hepatitis C virus (isolate India)
356423	GCA_002820945.1	Hepatitis C virus (isolate JK046)
356417	GCA_002820905.1	Hepatitis C virus (isolate JK049)
356412	GCA_003106065.1	Hepatitis C virus (isolate JPUT971017)
356415	GCA_000874285.1	Hepatitis C virus (isolate NZL1)
356421	GCA_000872025.1	Hepatitis C virus (isolate Th580)
357355	GCA_003106565.1	Hepatitis C virus (isolate Tr Kj)
356414	GCA_003106305.1	Hepatitis C virus (isolate VAT96)
356424	GCA_003107745.1	Hepatitis C virus (isolate VN004)
356422	GCA_003107605.1	Hepatitis C virus (isolate VN235)
356425	GCA_003107845.1	Hepatitis C virus (isolate VN405)
356418	GCA_000874265.1	Hepatitis C virus ED43
356418	GCA_002820925.1	Hepatitis C virus ED43
33745	GCA_003106805.1	Hepatitis C virus genotype 4
356411	GCA_003106005.1	Hepatitis C virus JFH-1
1544902	GCA_001712785.1	Hepatitis C virus QC69
356390	GCA_003107465.1	Hepatitis C virus SA13
31646	GCA_000861845.1	Hepatitis C virus subtype 1a
31646	GCA_002820825.1	Hepatitis C virus subtype 1a
31647	GCA_003105465.1	Hepatitis C virus subtype 1b
31647	GCA_003105485.1	Hepatitis C virus subtype 1b
31647	GCA_003105505.1	Hepatitis C virus subtype 1b
31647	GCA_003105525.1	Hepatitis C virus subtype 1b
31648	GCA_003108625.1	Hepatitis C virus subtype 1c
484894	GCA_003117875.1	Hepatitis C virus subtype 1g
31649	GCA_000871165.1	Hepatitis C virus subtype 2a
31649	GCA_003106025.1	Hepatitis C virus subtype 2a
31649	GCA_003106045.1	Hepatitis C virus subtype 2a
31650	GCA_003106085.1	Hepatitis C virus subtype 2b
31650	GCA_003106105.1	Hepatitis C virus subtype 2b
44021	GCA_003106205.1	Hepatitis C virus subtype 2f
44021	GCA_003106225.1	Hepatitis C virus subtype 2f
356466	GCA_003106325.1	Hepatitis C virus subtype 2k
1094901	GCA_003106405.1	Hepatitis C virus subtype 2m
356426	GCA_003106525.1	Hepatitis C virus subtype 3a
356426	GCA_003106545.1	Hepatitis C virus subtype 3a
42792	GCA_003106665.1	Hepatitis C virus subtype 3g

1094895	GCA_003106745.1	Hepatitis C virus subtype 3i
31653	GCA_003106825.1	Hepatitis C virus subtype 4a
128819	GCA_003106905.1	Hepatitis C virus subtype 4d
44023	GCA_003106945.1	Hepatitis C virus subtype 4f
1094899	GCA_003107025.1	Hepatitis C virus subtype 4g
1094898	GCA_003107105.1	Hepatitis C virus subtype 4L
1094900	GCA_003107145.1	Hepatitis C virus subtype 4m
693426	GCA_003107185.1	Hepatitis C virus subtype 4n
745709	GCA_003107225.1	Hepatitis C virus subtype 4o
693427	GCA_003107305.1	Hepatitis C virus subtype 4r
1208062	GCA_003107385.1	Hepatitis C virus subtype 4v
1208062	GCA_003107405.1	Hepatitis C virus subtype 4v
31655	GCA_003107545.1	Hepatitis C virus subtype 6a
438880	GCA_003107585.1	Hepatitis C virus subtype 6c
467354	GCA_003107685.1	Hepatitis C virus subtype 6f
356427	GCA_003107725.1	Hepatitis C virus subtype 6g
467337	GCA_003107765.1	Hepatitis C virus subtype 6i
467338	GCA_003107825.1	Hepatitis C virus subtype 6j
467338	GCA_003107805.1	Hepatitis C virus subtype 6j
356469	GCA_003107485.1	Hepatitis C virus subtype 6k
356469	GCA_003107505.1	Hepatitis C virus subtype 6k
413256	GCA_003107865.1	Hepatitis C virus subtype 6l
378507	GCA_003107905.1	Hepatitis C virus subtype 6m
378507	GCA_003107925.1	Hepatitis C virus subtype 6m
378506	GCA_003107945.1	Hepatitis C virus subtype 6n
413257	GCA_003107985.1	Hepatitis C virus subtype 6o
438881	GCA_003108025.1	Hepatitis C virus subtype 6p
467339	GCA_003108045.1	Hepatitis C virus subtype 6q
569610	GCA_003108185.1	Hepatitis C virus subtype 6v
12475	GCA_000856565.1	Hepatitis delta virus
12475	GCA_003047655.1	Hepatitis delta virus
12475	GCA_003047595.1	Hepatitis delta virus
12475	GCA_003047915.1	Hepatitis delta virus
12475	GCA_003047615.1	Hepatitis delta virus
12475	GCA_003047635.1	Hepatitis delta virus
12475	GCA_003033635.1	Hepatitis delta virus
12475	GCA_003033645.1	Hepatitis delta virus
12475	GCA_003971865.1	Hepatitis delta virus
12461	GCA_000861105.1	Hepatitis E virus
12461	GCA_002826365.1	Hepatitis E virus
12461	GCA_002826285.1	Hepatitis E virus
12461	GCA_002826305.1	Hepatitis E virus
12461	GCA_002826325.1	Hepatitis E virus
12461	GCA_002826225.1	Hepatitis E virus
12461	GCA_002826345.1	Hepatitis E virus
879096	GCA_002826445.1	Hepatitis E virus rat/R63/DEU/2009
39113	GCA_000862405.1	Hepatitis GB virus B
12092	GCA_000860505.1	Hepatovirus A
1230253	GCA_002831165.1	Herbert virus strain F23/CI/2004
1692251	GCA_001274105.1	Hermit crab associated circular genome

1692252	GCA_001274285.1	Hermit crab associated circular virus
28300	GCA_000861965.1	Heron hepatitis B virus
942041	GCA_002867875.1	Heterobasidion partitivirus 1
1469905	GCA_002867895.1	Heterobasidion partitivirus 12
1469906	GCA_002867935.1	Heterobasidion partitivirus 13
1469906	GCA_002867915.1	Heterobasidion partitivirus 13
1469908	GCA_002867995.1	Heterobasidion partitivirus 15
872291	GCA_002868215.1	Heterobasidion partitivirus 2
631431	GCA_002868015.1	Heterobasidion partitivirus 3
1387301	GCA_002378515.1	Heterobasidion partitivirus 7
1249677	GCA_002868435.1	Heterobasidion partitivirus 8
2030964	GCA_000865985.1	Heterocapsa circularisquama RNA virus 01
239720	GCA_000863545.1	Heterosigma akashiwo RNA virus SOG263
97195	GCA_002827745.1	Heterosigma akashiwo virus 01
1459800	GCA_000916095.1	Hibiscus bacilliform virus GD1
53181	GCA_000859105.1	Hibiscus chlorotic ringspot virus
1513278	GCA_000894935.1	Hibiscus green spot virus 2
233051	GCA_000925375.1	Hibiscus latent Fort Pierce virus
185955	GCA_000867565.1	Hibiscus latent Singapore virus
1468597	GCA_001963675.1	Hibiscus leaf curl alphasatellite
1980428	GCA_002375145.1	High Plains wheat mosaic emaravirus
11024	GCA_000881255.1	Highlands J virus
81583	GCA_000850745.1	Himetobi P virus
335963	GCA_000880795.1	Hippeastrum latent virus
421010	GCA_000894695.1	Hippeastrum mosaic virus
1980915	GCA_000857965.1	Hirame novirhabdovirus
1420594	GCA_002966415.1	Hirudovirus strain Sangsue
128708	GCA_000868945.1	His 1 virus
128710	GCA_000864585.1	His2 virus
540995	GCA_003098435.1	HIV-1 CRF03_AB
540992	GCA_003097935.1	HIV-1 CRF04_cpx
576900	GCA_003109185.1	HIV-1 M_02CD.KS069
576901	GCA_003109205.1	HIV-1 M_02CD.LBTB084
576902	GCA_003109225.1	HIV-1 M_02CD.MBTB047
576897	GCA_003109165.1	HIV-1 M_97CD.KTB119
2282643	GCA_004134205.1	Holcus lanatus-associated virus
269096	GCA_002986405.1	Hollyhock leaf crumple virus - [Cairo2]
169696	GCA_000840265.1	Hollyhock leaf crumple virus satellite DNA
223279	GCA_000842745.1	Hollyhock leaf crumple virus-[Cairo]
163655	GCA_002986605.1	Hollyhock leaf curl virus
1504732	GCA_001019895.1	Hollyhock yellow vein mosaic Islamabad virus
1504732	GCA_003073095.1	Hollyhock yellow vein mosaic Islamabad virus
944994	GCA_000896675.1	Hollyhock yellow vein mosaic virus
944996	GCA_003034075.1	Hollyhock yellow vein virus associated symptomless alphas
1960046	GCA_002118765.1	Hom-1 vesivirus
2170238	GCA_000869745.1	Homalodisca coagulata virus 1
411854	GCA_000881235.1	Homalodisca vitripennis reovirus
943272	GCA_000891515.1	Honeysuckle ringspot virus
435486	GCA_000870705.1	Honeysuckle yellow vein beta-[Japan:Fukui:2001]
221037	GCA_000848105.1	Honeysuckle yellow vein betasatellite

1233675	GCA_000870405.1	Honeysuckle yellow vein Kagoshima virus
1198498	GCA_002830085.1	Honeysuckle yellow vein mosaic betasatellite
417193	GCA_000873365.1	Honeysuckle yellow vein mosaic disease associated satellit
180587	GCA_000837545.1	Honeysuckle yellow vein mosaic virus
240865	GCA_000843705.1	Honeysuckle yellow vein virus
240865	GCA_002822445.1	Honeysuckle yellow vein virus
240865	GCA_002822465.1	Honeysuckle yellow vein virus
240865	GCA_002822485.1	Honeysuckle yellow vein virus
240865	GCA_002822505.1	Honeysuckle yellow vein virus
240865	GCA_002822525.1	Honeysuckle yellow vein virus
240865	GCA_002822545.1	Honeysuckle yellow vein virus
240865	GCA_002822565.1	Honeysuckle yellow vein virus
240865	GCA_002822585.1	Honeysuckle yellow vein virus
240865	GCA_002822625.1	Honeysuckle yellow vein virus
240865	GCA_002822645.1	Honeysuckle yellow vein virus
240865	GCA_002822665.1	Honeysuckle yellow vein virus
1313353	GCA_002822425.1	Honeysuckle yellow vein virus-[Australia:Ayr:1983]
435489	GCA_002822605.1	Honeysuckle yellow vein virus-[Japan:Masuda:2003]
240866	GCA_000859185.1	Honeysuckle yellow vein virus-[UK1]
12907	GCA_000856285.1	Hop latent viroid
104263	GCA_000863305.1	Hop latent virus
142843	GCA_000875045.1	Hop mosaic virus
12893	GCA_000847785.1	Hop stunt viroid
1323523	GCA_000906375.1	Hop trefoil cryptic virus 2
41764	GCA_000855025.1	Hordeum mosaic virus
1774276	GCA_001502155.1	Hordeum vulgare alphaendornavirus
263793	GCA_000840965.1	Horsegram yellow mosaic virus
46448	GCA_000837985.1	Horseradish curly top virus
264076	GCA_000897895.1	Horseradish latent virus
1508711	GCA_000923115.1	Horseshoe bat hepatitis B virus
214439	GCA_000880815.1	Hosta virus X
1711684	GCA_001308595.1	Hot pepper alphaendornavirus
2170112	GCA_000931115.1	Howler monkey associated porprismacovirus 1
1979541	GCA_002145505.1	Hoya chlorotic spot virus
1001303	GCA_003090975.1	Huaiyangshan virus
1001303	GCA_003089875.1	Huaiyangshan virus
1608047	GCA_001744775.1	Huangpi Tick Virus 1
1608048	GCA_001744095.1	Huangpi Tick Virus 2
1608049	GCA_001745415.1	Huangpi Tick Virus 3
1922835	GCA_001966655.1	Hubei arthropod virus 1
1922836	GCA_001966075.1	Hubei arthropod virus 3
1922837	GCA_001923715.1	Hubei astro-like virus
1922857	GCA_001964535.1	Hubei chuvirus-like virus 1
1922858	GCA_001965235.1	Hubei chuvirus-like virus 3
1922860	GCA_001966635.1	Hubei coleoptera virus 1
1922861	GCA_001966055.1	Hubei coleoptera virus 2
1922862	GCA_001964515.1	Hubei coleoptera virus 3
1922866	GCA_001965215.1	Hubei dimarhabdovirus virus 1
1922867	GCA_001966615.1	Hubei dimarhabdovirus virus 2
1922868	GCA_001966035.1	Hubei dimarhabdovirus virus 3

1922870	GCA_001964495.1	Hubei diptera virus 1
1922871	GCA_001965195.1	Hubei diptera virus 10
1922872	GCA_001966595.1	Hubei diptera virus 11
1922873	GCA_001966015.1	Hubei diptera virus 12
1922874	GCA_001964475.1	Hubei diptera virus 13
1922875	GCA_001965175.1	Hubei diptera virus 14
1922876	GCA_001964335.1	Hubei diptera virus 15
1922877	GCA_001965995.1	Hubei diptera virus 16
1922878	GCA_001964455.1	Hubei diptera virus 17
1922879	GCA_001921555.1	Hubei diptera virus 18
1922883	GCA_001965155.1	Hubei diptera virus 22
1922884	GCA_001921655.1	Hubei diptera virus 3
1922885	GCA_001924135.1	Hubei diptera virus 4
1922886	GCA_001924595.1	Hubei diptera virus 5
1922889	GCA_001964315.1	Hubei diptera virus 9
1922892	GCA_001923235.1	Hubei earwig virus 3
1922893	GCA_001965975.1	Hubei endorna-like virus 1
1922894	GCA_001964435.1	Hubei hepe-like virus 1
1922895	GCA_001965135.1	Hubei hepe-like virus 2
1922896	GCA_001964295.1	Hubei hepe-like virus 3
1922898	GCA_001921355.1	Hubei insect virus 2
1922899	GCA_001965955.1	Hubei leech virus 1
1922900	GCA_001923695.1	Hubei leech virus 2
1922901	GCA_001965415.1	Hubei leech virus 3
1922902	GCA_001966815.1	Hubei leech virus 4
1922903	GCA_001924115.1	Hubei lepidoptera virus 1
1922904	GCA_001966215.1	Hubei lepidoptera virus 2
1922905	GCA_001924575.1	Hubei lepidoptera virus 3
1922922	GCA_001964695.1	Hubei macula-like virus 1
1922923	GCA_001965395.1	Hubei macula-like virus 2
1922925	GCA_001966795.1	Hubei mosquito virus 1
1922926	GCA_001966195.1	Hubei mosquito virus 2
1922927	GCA_001964675.1	Hubei mosquito virus 3
1922928	GCA_001923215.1	Hubei mosquito virus 4
1922930	GCA_001923675.1	Hubei myriapoda virus 1
1922931	GCA_001924095.1	Hubei myriapoda virus 2
1922932	GCA_001924555.1	Hubei myriapoda virus 3
1922933	GCA_001923195.1	Hubei myriapoda virus 4
1922934	GCA_002003935.1	Hubei myriapoda virus 5
1922936	GCA_001923655.1	Hubei myriapoda virus 7
1922937	GCA_001924075.1	Hubei myriapoda virus 8
1922938	GCA_002004555.1	Hubei myriapoda virus 9
1922941	GCA_001965375.1	Hubei narna-like virus 11
1922942	GCA_001966775.1	Hubei narna-like virus 12
1922943	GCA_001966175.1	Hubei narna-like virus 13
1922945	GCA_001964655.1	Hubei narna-like virus 15
1922947	GCA_001965355.1	Hubei narna-like virus 17
1922948	GCA_001924535.1	Hubei narna-like virus 18
1922949	GCA_001966755.1	Hubei narna-like virus 19
1922950	GCA_001966155.1	Hubei narna-like virus 2

1922952	GCA_001964635.1	Hubei narna-like virus 21
1922953	GCA_001965335.1	Hubei narna-like virus 22
1922954	GCA_001966735.1	Hubei narna-like virus 23
1922955	GCA_001910755.1	Hubei narna-like virus 24
1922956	GCA_001968355.1	Hubei narna-like virus 25
1922957	GCA_001966135.1	Hubei narna-like virus 3
1922958	GCA_001964615.1	Hubei narna-like virus 4
1922959	GCA_001965315.1	Hubei narna-like virus 5
1922960	GCA_001966715.1	Hubei narna-like virus 7
1922962	GCA_001966115.1	Hubei narna-like virus 9
1922972	GCA_001923175.1	Hubei noda-like virus 17
1922988	GCA_001964595.1	Hubei noda-like virus 8
1922989	GCA_001965295.1	Hubei noda-like virus 9
1922990	GCA_001966695.1	Hubei odonate virus 1
1922991	GCA_001966095.1	Hubei odonate virus 10
1922992	GCA_001964575.1	Hubei odonate virus 11
1922993	GCA_001965275.1	Hubei odonate virus 12
1922997	GCA_001966375.1	Hubei odonate virus 2
1922998	GCA_001964855.1	Hubei odonate virus 3
1922999	GCA_001965555.1	Hubei odonate virus 4
1923000	GCA_001966955.1	Hubei odonate virus 5
1923001	GCA_001966355.1	Hubei odonate virus 6
1923002	GCA_001964835.1	Hubei odonate virus 7
1923003	GCA_001921435.1	Hubei odonate virus 8
1923004	GCA_001921535.1	Hubei odonate virus 9
1923009	GCA_001965535.1	Hubei orthoptera virus 1
1923011	GCA_001966935.1	Hubei orthoptera virus 3
1923012	GCA_001966335.1	Hubei orthoptera virus 4
1923013	GCA_001964815.1	Hubei orthoptera virus 5
1923017	GCA_001921635.1	Hubei partiti-like virus 11
1923073	GCA_001965515.1	Hubei permutotetra-like virus 1
1923074	GCA_001966915.1	Hubei permutotetra-like virus 10
1923075	GCA_001966315.1	Hubei permutotetra-like virus 11
1923076	GCA_001964795.1	Hubei permutotetra-like virus 2
1923077	GCA_001965495.1	Hubei permutotetra-like virus 3
1923078	GCA_001966895.1	Hubei permutotetra-like virus 4
1923079	GCA_001966295.1	Hubei permutotetra-like virus 5
1923080	GCA_001964775.1	Hubei permutotetra-like virus 6
1923081	GCA_001965475.1	Hubei permutotetra-like virus 7
1923082	GCA_001966875.1	Hubei permutotetra-like virus 8
1923083	GCA_001966275.1	Hubei permutotetra-like virus 9
1923088	GCA_001964755.1	Hubei picorna-like virus 1
1923089	GCA_001965455.1	Hubei picorna-like virus 10
1923090	GCA_001966855.1	Hubei picorna-like virus 11
1923091	GCA_001966255.1	Hubei picorna-like virus 12
1923092	GCA_001964735.1	Hubei picorna-like virus 13
1923093	GCA_001965435.1	Hubei picorna-like virus 14
1923094	GCA_001966835.1	Hubei picorna-like virus 15
1923095	GCA_001966235.1	Hubei picorna-like virus 16
1923096	GCA_001964715.1	Hubei picorna-like virus 17

1923097	GCA_001967355.1	Hubei picorna-like virus 18
1923099	GCA_001967855.1	Hubei picorna-like virus 2
1923100	GCA_001957395.1	Hubei picorna-like virus 20
1923101	GCA_001968415.1	Hubei picorna-like virus 21
1923102	GCA_001965695.1	Hubei picorna-like virus 22
1923104	GCA_001923635.1	Hubei picorna-like virus 24
1923105	GCA_001967095.1	Hubei picorna-like virus 25
1923106	GCA_001966495.1	Hubei picorna-like virus 26
1923107	GCA_001964975.1	Hubei picorna-like virus 27
1923108	GCA_001965675.1	Hubei picorna-like virus 28
1923109	GCA_001967075.1	Hubei picorna-like virus 29
1923110	GCA_001966475.1	Hubei picorna-like virus 30
1923111	GCA_001924055.1	Hubei picorna-like virus 31
1923112	GCA_001964955.1	Hubei picorna-like virus 32
1923113	GCA_001965655.1	Hubei picorna-like virus 33
1923114	GCA_001967055.1	Hubei picorna-like virus 34
1923115	GCA_001966455.1	Hubei picorna-like virus 35
1923116	GCA_001964935.1	Hubei picorna-like virus 36
1923117	GCA_001965635.1	Hubei picorna-like virus 37
1923118	GCA_001967035.1	Hubei picorna-like virus 38
1923119	GCA_001966435.1	Hubei picorna-like virus 39
1923120	GCA_001964915.1	Hubei picorna-like virus 4
1923121	GCA_001965615.1	Hubei picorna-like virus 40
1923122	GCA_001967015.1	Hubei picorna-like virus 41
1923123	GCA_001966415.1	Hubei picorna-like virus 42
1923124	GCA_001964895.1	Hubei picorna-like virus 43
1923125	GCA_001965595.1	Hubei picorna-like virus 44
1923126	GCA_001966995.1	Hubei picorna-like virus 45
1923127	GCA_001924515.1	Hubei picorna-like virus 46
1923128	GCA_001966395.1	Hubei picorna-like virus 47
1923129	GCA_001964875.1	Hubei picorna-like virus 48
1923130	GCA_001965575.1	Hubei picorna-like virus 49
1923131	GCA_001966975.1	Hubei picorna-like virus 5
1923132	GCA_001968575.1	Hubei picorna-like virus 50
1923133	GCA_001967495.1	Hubei picorna-like virus 51
1923134	GCA_001967995.1	Hubei picorna-like virus 52
1923135	GCA_001957535.1	Hubei picorna-like virus 53
1923136	GCA_001968555.1	Hubei picorna-like virus 54
1923137	GCA_001967475.1	Hubei picorna-like virus 55
1923138	GCA_001967975.1	Hubei picorna-like virus 56
1923140	GCA_001957515.1	Hubei picorna-like virus 58
1923141	GCA_001968535.1	Hubei picorna-like virus 59
1923142	GCA_001967455.1	Hubei picorna-like virus 6
1923143	GCA_001967955.1	Hubei picorna-like virus 60
1923144	GCA_001957495.1	Hubei picorna-like virus 61
1923145	GCA_001968515.1	Hubei picorna-like virus 62
1923146	GCA_001967435.1	Hubei picorna-like virus 63
1923147	GCA_001967935.1	Hubei picorna-like virus 64
1923148	GCA_001957475.1	Hubei picorna-like virus 65
1923149	GCA_002366185.1	Hubei picorna-like virus 66

1923150	GCA_001968495.1	Hubei picorna-like virus 67
1923151	GCA_001967415.1	Hubei picorna-like virus 68
1923152	GCA_001967915.1	Hubei picorna-like virus 69
1923153	GCA_001957455.1	Hubei picorna-like virus 7
1923154	GCA_001968475.1	Hubei picorna-like virus 70
1923155	GCA_001967395.1	Hubei picorna-like virus 71
1923156	GCA_001967895.1	Hubei picorna-like virus 72
1923157	GCA_001957435.1	Hubei picorna-like virus 73
1923158	GCA_001968455.1	Hubei picorna-like virus 74
1923159	GCA_001967375.1	Hubei picorna-like virus 75
1923160	GCA_001967875.1	Hubei picorna-like virus 76
1923161	GCA_001957415.1	Hubei picorna-like virus 77
1923162	GCA_001968435.1	Hubei picorna-like virus 78
1923163	GCA_001966515.1	Hubei picorna-like virus 79
1923164	GCA_001964995.1	Hubei picorna-like virus 8
1923165	GCA_001968715.1	Hubei picorna-like virus 80
1923166	GCA_001967635.1	Hubei picorna-like virus 81
1923167	GCA_001923155.1	Hubei picorna-like virus 82
1923168	GCA_001968135.1	Hubei picorna-like virus 9
1923169	GCA_001923615.1	Hubei polero-like virus 1
1923170	GCA_001957675.1	Hubei polero-like virus 2
1922832	GCA_001964555.1	Hubei Poty-like virus 1
1938657	GCA_002368945.1	Hubei qinvirus-like virus 1
1923185	GCA_001968695.1	Hubei rhabdo-like virus 1
1923186	GCA_001967615.1	Hubei rhabdo-like virus 2
1923187	GCA_001968115.1	Hubei rhabdo-like virus 3
1923188	GCA_001957655.1	Hubei rhabdo-like virus 4
1923189	GCA_003674005.1	Hubei rhabdo-like virus 5
1923190	GCA_003673985.1	Hubei rhabdo-like virus 6
1923191	GCA_001968675.1	Hubei rhabdo-like virus 7
1923192	GCA_003673965.1	Hubei rhabdo-like virus 8
1923193	GCA_001967595.1	Hubei rhabdo-like virus 9
1923194	GCA_001924035.1	Hubei sobemo-like virus 1
1923195	GCA_001924495.1	Hubei sobemo-like virus 10
1923196	GCA_001923135.1	Hubei sobemo-like virus 11
1923197	GCA_001923595.1	Hubei sobemo-like virus 12
1923198	GCA_001924015.1	Hubei sobemo-like virus 13
1923199	GCA_001924475.1	Hubei sobemo-like virus 14
1923200	GCA_001923115.1	Hubei sobemo-like virus 15
1923201	GCA_001923935.1	Hubei sobemo-like virus 16
1923203	GCA_001924355.1	Hubei sobemo-like virus 18
1923204	GCA_001924815.1	Hubei sobemo-like virus 19
1923205	GCA_001923455.1	Hubei sobemo-like virus 2
1923206	GCA_001923915.1	Hubei sobemo-like virus 20
1923208	GCA_001924335.1	Hubei sobemo-like virus 22
1923209	GCA_001924795.1	Hubei sobemo-like virus 23
1923210	GCA_001923435.1	Hubei sobemo-like virus 24
1923211	GCA_001923895.1	Hubei sobemo-like virus 25
1923212	GCA_001924315.1	Hubei sobemo-like virus 26
1923213	GCA_001924775.1	Hubei sobemo-like virus 27

1923214	GCA_001923415.1	Hubei sobemo-like virus 28
1923215	GCA_001923875.1	Hubei sobemo-like virus 29
1923216	GCA_001924295.1	Hubei sobemo-like virus 3
1923217	GCA_001924755.1	Hubei sobemo-like virus 30
1923218	GCA_001923395.1	Hubei sobemo-like virus 31
1923219	GCA_001923855.1	Hubei sobemo-like virus 32
1923220	GCA_001924275.1	Hubei sobemo-like virus 33
1923221	GCA_001924735.1	Hubei sobemo-like virus 34
1923222	GCA_001923375.1	Hubei sobemo-like virus 35
1923223	GCA_001923835.1	Hubei sobemo-like virus 36
1923224	GCA_001924255.1	Hubei sobemo-like virus 37
1923225	GCA_001924715.1	Hubei sobemo-like virus 38
1923228	GCA_001923355.1	Hubei sobemo-like virus 40
1923229	GCA_001923815.1	Hubei sobemo-like virus 41
1923230	GCA_001924235.1	Hubei sobemo-like virus 42
1923231	GCA_001924695.1	Hubei sobemo-like virus 43
1923232	GCA_001923335.1	Hubei sobemo-like virus 44
1923233	GCA_001923795.1	Hubei sobemo-like virus 45
1923234	GCA_001924215.1	Hubei sobemo-like virus 46
1923235	GCA_001924675.1	Hubei sobemo-like virus 47
1923236	GCA_001923315.1	Hubei sobemo-like virus 48
1923237	GCA_001923775.1	Hubei sobemo-like virus 49
1923238	GCA_001924195.1	Hubei sobemo-like virus 5
1923239	GCA_001924655.1	Hubei sobemo-like virus 6
1923240	GCA_001923295.1	Hubei sobemo-like virus 7
1923241	GCA_001923755.1	Hubei sobemo-like virus 8
1923242	GCA_001924175.1	Hubei sobemo-like virus 9
1923243	GCA_001968095.1	Hubei tetragnatha maxillosa virus 1
1923244	GCA_001957635.1	Hubei tetragnatha maxillosa virus 2
1923245	GCA_001968655.1	Hubei tetragnatha maxillosa virus 3
1923246	GCA_001967575.1	Hubei tetragnatha maxillosa virus 4
1923247	GCA_001968075.1	Hubei tetragnatha maxillosa virus 5
1923248	GCA_001924635.1	Hubei tetragnatha maxillosa virus 6
1923249	GCA_001957615.1	Hubei tetragnatha maxillosa virus 7
1923250	GCA_001968635.1	Hubei tetragnatha maxillosa virus 8
1923252	GCA_001967555.1	Hubei tick virus 1
1923253	GCA_001968055.1	Hubei tick virus 2
1923254	GCA_001957595.1	Hubei tick virus 3
1923255	GCA_001968615.1	Hubei tombus-like virus 1
1923256	GCA_001967535.1	Hubei tombus-like virus 10
1923257	GCA_001968035.1	Hubei tombus-like virus 11
1923258	GCA_001957575.1	Hubei tombus-like virus 12
1923259	GCA_001968595.1	Hubei tombus-like virus 13
1923260	GCA_001967515.1	Hubei tombus-like virus 14
1923261	GCA_001968015.1	Hubei tombus-like virus 15
1923262	GCA_001926995.1	Hubei tombus-like virus 16
1923263	GCA_001957555.1	Hubei tombus-like virus 17
1923264	GCA_001967335.1	Hubei tombus-like virus 18
1923265	GCA_001967835.1	Hubei tombus-like virus 19
1923266	GCA_001957375.1	Hubei tombus-like virus 2

1923267	GCA_001968395.1	Hubei tombus-like virus 20
1923268	GCA_001967315.1	Hubei tombus-like virus 21
1923269	GCA_002366245.1	Hubei tombus-like virus 22
1923270	GCA_001967815.1	Hubei tombus-like virus 23
1923271	GCA_001957355.1	Hubei tombus-like virus 24
1923272	GCA_001968375.1	Hubei tombus-like virus 25
1923273	GCA_001967295.1	Hubei tombus-like virus 26
1923274	GCA_001967795.1	Hubei tombus-like virus 27
1923275	GCA_001965095.1	Hubei tombus-like virus 28
1923276	GCA_001965795.1	Hubei tombus-like virus 29
1923277	GCA_001967195.1	Hubei tombus-like virus 3
1923278	GCA_001926155.1	Hubei tombus-like virus 30
1923279	GCA_001967235.1	Hubei tombus-like virus 31
1923280	GCA_001926735.1	Hubei tombus-like virus 32
1923281	GCA_001965075.1	Hubei tombus-like virus 33
1923282	GCA_001927235.1	Hubei tombus-like virus 34
1923284	GCA_001965775.1	Hubei tombus-like virus 36
1923285	GCA_001926395.1	Hubei tombus-like virus 37
1923286	GCA_001967175.1	Hubei tombus-like virus 38
1923287	GCA_001966575.1	Hubei tombus-like virus 39
1923288	GCA_001965055.1	Hubei tombus-like virus 4
1923289	GCA_001965755.1	Hubei tombus-like virus 40
1923290	GCA_001967155.1	Hubei tombus-like virus 42
1923291	GCA_001966555.1	Hubei tombus-like virus 43
1923292	GCA_001965035.1	Hubei tombus-like virus 5
1923293	GCA_001965735.1	Hubei tombus-like virus 6
1923294	GCA_001967135.1	Hubei tombus-like virus 7
1923295	GCA_001966535.1	Hubei tombus-like virus 8
1923296	GCA_001965015.1	Hubei tombus-like virus 9
1923298	GCA_001965715.1	Hubei toti-like virus 10
1923300	GCA_001967115.1	Hubei toti-like virus 12
1923301	GCA_001961395.1	Hubei toti-like virus 13
1923303	GCA_001962135.1	Hubei toti-like virus 15
1923304	GCA_001963655.1	Hubei toti-like virus 16
1923305	GCA_001963055.1	Hubei toti-like virus 17
1923306	GCA_001961375.1	Hubei toti-like virus 18
1923307	GCA_001925955.1	Hubei toti-like virus 19
1923308	GCA_001962115.1	Hubei toti-like virus 2
1923309	GCA_001963635.1	Hubei toti-like virus 20
1923310	GCA_001963035.1	Hubei toti-like virus 21
1923313	GCA_001961355.1	Hubei toti-like virus 24
1923316	GCA_001962095.1	Hubei toti-like virus 5
1923320	GCA_001963615.1	Hubei toti-like virus 9
1923321	GCA_001963015.1	Hubei unio douglasiae virus 1
1923322	GCA_001961335.1	Hubei unio douglasiae virus 2
1923323	GCA_001962075.1	Hubei unio douglasiae virus 3
1923324	GCA_001963595.1	Hubei virga-like virus 1
1923326	GCA_001962995.1	Hubei virga-like virus 11
1923327	GCA_001961315.1	Hubei virga-like virus 12
1923330	GCA_001962055.1	Hubei virga-like virus 15

1923331	GCA_001963575.1	Hubei virga-like virus 16
1923332	GCA_001962975.1	Hubei virga-like virus 17
1923333	GCA_001926715.1	Hubei virga-like virus 18
1923335	GCA_001961295.1	Hubei virga-like virus 2
1923336	GCA_001962035.1	Hubei virga-like virus 21
1923338	GCA_001963555.1	Hubei virga-like virus 23
1923340	GCA_001962955.1	Hubei virga-like virus 7
1923342	GCA_001961275.1	Hubei virga-like virus 9
1922833	GCA_001965255.1	Hubei Wuhan insect virus 9
1923343	GCA_001962015.1	Hubei yanvirus-like virus 1
1923344	GCA_001961135.1	Hubei zhaovirus-like virus 1
1923345	GCA_001962935.1	Hubei zhaovirus-like virus 2
2021738	GCA_002375055.1	Hudisavirus sp.
2021738	GCA_003729515.1	Hudisavirus sp.
2021738	GCA_003729635.1	Hudisavirus sp.
2021738	GCA_003729595.1	Hudisavirus sp.
2021738	GCA_003729615.1	Hudisavirus sp.
2021738	GCA_003729715.1	Hudisavirus sp.
2021738	GCA_003729755.1	Hudisavirus sp.
2021738	GCA_003729535.1	Hudisavirus sp.
2021738	GCA_003729735.1	Hudisavirus sp.
2021738	GCA_003729575.1	Hudisavirus sp.
2021738	GCA_003729775.1	Hudisavirus sp.
2021738	GCA_003729795.1	Hudisavirus sp.
2021738	GCA_003729815.1	Hudisavirus sp.
2021738	GCA_003729555.1	Hudisavirus sp.
10541	GCA_000857085.1	Human adenovirus 11
28282	GCA_000846805.1	Human adenovirus 12
10515	GCA_000845085.1	Human adenovirus 2
651580	GCA_000885675.1	Human adenovirus 54
45659	GCA_000880515.1	Human adenovirus B3
10527	GCA_000845985.1	Human adenovirus D9
10533	GCA_000858645.1	Human adenovirus type 1
28282	GCA_000884295.1	Human adenovirus type 12
46922	GCA_000858625.1	Human adenovirus type 17
10515	GCA_000859465.1	Human adenovirus type 2
10522	GCA_000857885.1	Human adenovirus type 35
28285	GCA_000857865.1	Human adenovirus type 5
10519	GCA_000859485.1	Human adenovirus type 7
10298	GCA_000859985.2	Human alphaherpesvirus 1 (Herpes simplex virus type 1)
10298	GCA_003052245.1	Human alphaherpesvirus 1 (Herpes simplex virus type 1)
10298	GCA_900089815.1	Human alphaherpesvirus 1 (Herpes simplex virus type 1)
10298	GCA_900090015.1	Human alphaherpesvirus 1 (Herpes simplex virus type 1)
10298	GCA_900089865.1	Human alphaherpesvirus 1 (Herpes simplex virus type 1)
10298	GCA_900089925.1	Human alphaherpesvirus 1 (Herpes simplex virus type 1)
10298	GCA_900089915.1	Human alphaherpesvirus 1 (Herpes simplex virus type 1)
10298	GCA_900089895.1	Human alphaherpesvirus 1 (Herpes simplex virus type 1)
10298	GCA_900089935.1	Human alphaherpesvirus 1 (Herpes simplex virus type 1)
10298	GCA_900089875.1	Human alphaherpesvirus 1 (Herpes simplex virus type 1)
10298	GCA_900089905.1	Human alphaherpesvirus 1 (Herpes simplex virus type 1)

10298	GCA_900089885.1	Human alphaherpesvirus 1 (Herpes simplex virus type 1)
10299	GCA_002847095.1	Human alphaherpesvirus 1 strain 17 (Herpes simplex virus
10299	GCA_002847105.1	Human alphaherpesvirus 1 strain 17 (Herpes simplex virus
10299	GCA_002847115.1	Human alphaherpesvirus 1 strain 17 (Herpes simplex virus
10299	GCA_002847125.1	Human alphaherpesvirus 1 strain 17 (Herpes simplex virus
10299	GCA_002847135.1	Human alphaherpesvirus 1 strain 17 (Herpes simplex virus
10299	GCA_900088405.1	Human alphaherpesvirus 1 strain 17 (Herpes simplex virus
10310	GCA_000858385.2	Human alphaherpesvirus 2
10310	GCA_003052145.1	Human alphaherpesvirus 2
10310	GCA_900167795.1	Human alphaherpesvirus 2
10310	GCA_900167655.1	Human alphaherpesvirus 2
10310	GCA_900167675.1	Human alphaherpesvirus 2
10310	GCA_900167695.1	Human alphaherpesvirus 2
10310	GCA_900167735.1	Human alphaherpesvirus 2
10310	GCA_900168245.1	Human alphaherpesvirus 2
10310	GCA_900167775.1	Human alphaherpesvirus 2
10310	GCA_900167685.1	Human alphaherpesvirus 2
10310	GCA_900167665.1	Human alphaherpesvirus 2
10310	GCA_900167755.1	Human alphaherpesvirus 2
10310	GCA_900167805.1	Human alphaherpesvirus 2
10310	GCA_900167715.1	Human alphaherpesvirus 2
10310	GCA_900167785.1	Human alphaherpesvirus 2
10310	GCA_900167645.1	Human alphaherpesvirus 2
10310	GCA_900379695.1	Human alphaherpesvirus 2
10310	GCA_900167745.1	Human alphaherpesvirus 2
10310	GCA_900167705.1	Human alphaherpesvirus 2
2038728	GCA_000918035.1	Human associated cyclovirus 10
2004487	GCA_003849345.1	Human associated gemykibivirus 1
2004957	GCA_003846505.1	Human associated gemykibivirus 2
1985415	GCA_001448435.1	Human associated gemyvongvirus 1
2169934	GCA_003033475.1	Human associated huchismacovirus 1
2169935	GCA_003033485.1	Human associated huchismacovirus 2
2169936	GCA_003033495.1	Human associated huchismacovirus 3
2170114	GCA_000931315.1	Human associated porprismacovirus 2
12456	GCA_000861865.1	Human astrovirus 1
1518575	GCA_000923215.1	Human astrovirus BF34
1235996	GCA_000901155.1	Human betacoronavirus 2c EMC/2012
10359	GCA_000845245.1	Human betaherpesvirus 5 (Human cytomegalovirus)
10359	GCA_900230315.1	Human betaherpesvirus 5 (Human cytomegalovirus)
32603	GCA_000845685.1	Human betaherpesvirus 6A
32603	GCA_000845685.2	Human betaherpesvirus 6A
32604	GCA_000846365.1	Human betaherpesvirus 6B
10372	GCA_000848125.1	Human betaherpesvirus 7
1511882	GCA_000882675.1	Human bocavirus 2c PK
638313	GCA_000882855.1	Human bocavirus 3
1511883	GCA_000886375.1	Human bocavirus 4 NI
1525173	GCA_000924015.1	Human circovirus VS6600022
749224	GCA_003143455.1	Human circular dsDNA virus associated with alcoholic cirrh
11137	GCA_000853505.1	Human coronavirus 229E
290028	GCA_000858765.1	Human coronavirus HKU1

277944	GCA_000853865.1	Human coronavirus NL63
31631	GCA_003972325.1	Human coronavirus OC43
1233383	GCA_000920655.1	Human cosavirus
586459	GCA_000884955.1	Human cosavirus B1
586468	GCA_000886475.1	Human cosavirus D1
589919	GCA_000886455.1	Human cosavirus E1
12070	GCA_003108485.1	Human coxsackievirus A21 Coe
1345637	GCA_000908835.1	Human cyclovirus VS5700009
1904876	GCA_003727435.1	Human DNA virus
166122	GCA_000913595.1	Human endogenous retrovirus K113
1193974	GCA_001684625.1	Human enterovirus
12090	GCA_000861205.1	Human enterovirus 70
72197	GCA_000841965.1	Human erythrovirus V9
2017081	GCA_002219885.1	Human fecal virus Jorvi2
2017082	GCA_002219525.1	Human fecal virus Jorvi3
2017083	GCA_002219705.1	Human fecal virus Jorvi4
1820160	GCA_003726975.1	Human feces pecovirus
1820160	GCA_003726995.1	Human feces pecovirus
1820160	GCA_003727015.1	Human feces pecovirus
1820158	GCA_003033575.1	Human feces smacovirus 2
1820159	GCA_002271185.1	Human feces smacovirus 3
1820159	GCA_003963815.1	Human feces smacovirus 3
1820159	GCA_003963575.1	Human feces smacovirus 3
1914415	GCA_003963775.1	Human feces-associated smacovirus
1914415	GCA_003963795.1	Human feces-associated smacovirus
10376	GCA_000872045.1	Human gammaherpesvirus 4 (Epstein-Barr virus)
10376	GCA_002402265.1	Human gammaherpesvirus 4 (Epstein-Barr virus)
10376	GCA_900003475.1	Human gammaherpesvirus 4 (Epstein-Barr virus)
10376	GCA_900003915.1	Human gammaherpesvirus 4 (Epstein-Barr virus)
10376	GCA_900003815.1	Human gammaherpesvirus 4 (Epstein-Barr virus)
10376	GCA_900003895.1	Human gammaherpesvirus 4 (Epstein-Barr virus)
10376	GCA_900003745.1	Human gammaherpesvirus 4 (Epstein-Barr virus)
10376	GCA_900003825.1	Human gammaherpesvirus 4 (Epstein-Barr virus)
10376	GCA_900007025.1	Human gammaherpesvirus 4 (Epstein-Barr virus)
10376	GCA_900004115.1	Human gammaherpesvirus 4 (Epstein-Barr virus)
10376	GCA_900003615.1	Human gammaherpesvirus 4 (Epstein-Barr virus)
10376	GCA_900004315.1	Human gammaherpesvirus 4 (Epstein-Barr virus)
10376	GCA_900474055.1	Human gammaherpesvirus 4 (Epstein-Barr virus)
10376	GCA_900474035.1	Human gammaherpesvirus 4 (Epstein-Barr virus)
10376	GCA_900003435.1	Human gammaherpesvirus 4 (Epstein-Barr virus)
10376	GCA_900003835.1	Human gammaherpesvirus 4 (Epstein-Barr virus)
10376	GCA_900004125.1	Human gammaherpesvirus 4 (Epstein-Barr virus)
10376	GCA_900003445.1	Human gammaherpesvirus 4 (Epstein-Barr virus)
10376	GCA_900003845.1	Human gammaherpesvirus 4 (Epstein-Barr virus)
10376	GCA_900003495.1	Human gammaherpesvirus 4 (Epstein-Barr virus)
10376	GCA_900003925.1	Human gammaherpesvirus 4 (Epstein-Barr virus)
10376	GCA_900003935.1	Human gammaherpesvirus 4 (Epstein-Barr virus)
10376	GCA_900004135.1	Human gammaherpesvirus 4 (Epstein-Barr virus)
10376	GCA_900004635.1	Human gammaherpesvirus 4 (Epstein-Barr virus)
10376	GCA_900003855.1	Human gammaherpesvirus 4 (Epstein-Barr virus)

10376	GCA_900003865.1	Human gammaherpesvirus 4 (Epstein-Barr virus)
10376	GCA_900004255.1	Human gammaherpesvirus 4 (Epstein-Barr virus)
10376	GCA_900003655.1	Human gammaherpesvirus 4 (Epstein-Barr virus)
10376	GCA_900004145.1	Human gammaherpesvirus 4 (Epstein-Barr virus)
10376	GCA_900006265.1	Human gammaherpesvirus 4 (Epstein-Barr virus)
10376	GCA_900004665.1	Human gammaherpesvirus 4 (Epstein-Barr virus)
10376	GCA_900003585.1	Human gammaherpesvirus 4 (Epstein-Barr virus)
10376	GCA_900474145.1	Human gammaherpesvirus 4 (Epstein-Barr virus)
10376	GCA_900003995.1	Human gammaherpesvirus 4 (Epstein-Barr virus)
10376	GCA_900004005.1	Human gammaherpesvirus 4 (Epstein-Barr virus)
10376	GCA_900004625.1	Human gammaherpesvirus 4 (Epstein-Barr virus)
10376	GCA_900003555.1	Human gammaherpesvirus 4 (Epstein-Barr virus)
10376	GCA_900013555.1	Human gammaherpesvirus 4 (Epstein-Barr virus)
10376	GCA_900004215.1	Human gammaherpesvirus 4 (Epstein-Barr virus)
10376	GCA_900003545.1	Human gammaherpesvirus 4 (Epstein-Barr virus)
10376	GCA_900003955.1	Human gammaherpesvirus 4 (Epstein-Barr virus)
10376	GCA_900473995.1	Human gammaherpesvirus 4 (Epstein-Barr virus)
10376	GCA_900411615.1	Human gammaherpesvirus 4 (Epstein-Barr virus)
10376	GCA_900067285.1	Human gammaherpesvirus 4 (Epstein-Barr virus)
10376	GCA_900003665.1	Human gammaherpesvirus 4 (Epstein-Barr virus)
10376	GCA_900411515.1	Human gammaherpesvirus 4 (Epstein-Barr virus)
10376	GCA_900003885.1	Human gammaherpesvirus 4 (Epstein-Barr virus)
10376	GCA_900411405.1	Human gammaherpesvirus 4 (Epstein-Barr virus)
10376	GCA_900005835.1	Human gammaherpesvirus 4 (Epstein-Barr virus)
10376	GCA_900411415.1	Human gammaherpesvirus 4 (Epstein-Barr virus)
10376	GCA_900003595.1	Human gammaherpesvirus 4 (Epstein-Barr virus)
10376	GCA_900411445.1	Human gammaherpesvirus 4 (Epstein-Barr virus)
10376	GCA_900003625.1	Human gammaherpesvirus 4 (Epstein-Barr virus)
10376	GCA_900411425.1	Human gammaherpesvirus 4 (Epstein-Barr virus)
10376	GCA_900006285.1	Human gammaherpesvirus 4 (Epstein-Barr virus)
10376	GCA_900411435.1	Human gammaherpesvirus 4 (Epstein-Barr virus)
10376	GCA_900003675.1	Human gammaherpesvirus 4 (Epstein-Barr virus)
10376	GCA_900411475.1	Human gammaherpesvirus 4 (Epstein-Barr virus)
10376	GCA_900012645.1	Human gammaherpesvirus 4 (Epstein-Barr virus)
10376	GCA_900411455.1	Human gammaherpesvirus 4 (Epstein-Barr virus)
10376	GCA_900003645.1	Human gammaherpesvirus 4 (Epstein-Barr virus)
10376	GCA_900411525.1	Human gammaherpesvirus 4 (Epstein-Barr virus)
10376	GCA_900003405.1	Human gammaherpesvirus 4 (Epstein-Barr virus)
10376	GCA_900411485.1	Human gammaherpesvirus 4 (Epstein-Barr virus)
10376	GCA_900003735.1	Human gammaherpesvirus 4 (Epstein-Barr virus)
10376	GCA_900411505.1	Human gammaherpesvirus 4 (Epstein-Barr virus)
10376	GCA_900003685.1	Human gammaherpesvirus 4 (Epstein-Barr virus)
10376	GCA_900411495.1	Human gammaherpesvirus 4 (Epstein-Barr virus)
10376	GCA_900411535.1	Human gammaherpesvirus 4 (Epstein-Barr virus)
10376	GCA_900003695.1	Human gammaherpesvirus 4 (Epstein-Barr virus)
10376	GCA_900003635.1	Human gammaherpesvirus 4 (Epstein-Barr virus)
10376	GCA_900004075.1	Human gammaherpesvirus 4 (Epstein-Barr virus)
10376	GCA_900411545.1	Human gammaherpesvirus 4 (Epstein-Barr virus)
10376	GCA_900003605.1	Human gammaherpesvirus 4 (Epstein-Barr virus)
10376	GCA_900411565.1	Human gammaherpesvirus 4 (Epstein-Barr virus)

10376	GCA_900006575.1	Human gammaherpesvirus 4 (Epstein-Barr virus)
10376	GCA_900411555.1	Human gammaherpesvirus 4 (Epstein-Barr virus)
10376	GCA_900006275.1	Human gammaherpesvirus 4 (Epstein-Barr virus)
10376	GCA_900019245.1	Human gammaherpesvirus 4 (Epstein-Barr virus)
10376	GCA_900411575.1	Human gammaherpesvirus 4 (Epstein-Barr virus)
10376	GCA_900474065.1	Human gammaherpesvirus 4 (Epstein-Barr virus)
10376	GCA_900003775.1	Human gammaherpesvirus 4 (Epstein-Barr virus)
10376	GCA_900411595.1	Human gammaherpesvirus 4 (Epstein-Barr virus)
10376	GCA_900003785.1	Human gammaherpesvirus 4 (Epstein-Barr virus)
10376	GCA_900411585.1	Human gammaherpesvirus 4 (Epstein-Barr virus)
10376	GCA_900005075.1	Human gammaherpesvirus 4 (Epstein-Barr virus)
10376	GCA_900004285.1	Human gammaherpesvirus 4 (Epstein-Barr virus)
10376	GCA_900004235.1	Human gammaherpesvirus 4 (Epstein-Barr virus)
10376	GCA_900004295.1	Human gammaherpesvirus 4 (Epstein-Barr virus)
10376	GCA_900003905.1	Human gammaherpesvirus 4 (Epstein-Barr virus)
10376	GCA_900006305.1	Human gammaherpesvirus 4 (Epstein-Barr virus)
10376	GCA_900007265.1	Human gammaherpesvirus 4 (Epstein-Barr virus)
10376	GCA_900474155.1	Human gammaherpesvirus 4 (Epstein-Barr virus)
10376	GCA_900003795.1	Human gammaherpesvirus 4 (Epstein-Barr virus)
10376	GCA_900474115.1	Human gammaherpesvirus 4 (Epstein-Barr virus)
10376	GCA_900003415.1	Human gammaherpesvirus 4 (Epstein-Barr virus)
10376	GCA_900474255.1	Human gammaherpesvirus 4 (Epstein-Barr virus)
10376	GCA_900004095.1	Human gammaherpesvirus 4 (Epstein-Barr virus)
10376	GCA_900474595.1	Human gammaherpesvirus 4 (Epstein-Barr virus)
10376	GCA_900004245.1	Human gammaherpesvirus 4 (Epstein-Barr virus)
10376	GCA_900003465.1	Human gammaherpesvirus 4 (Epstein-Barr virus)
10376	GCA_900474125.1	Human gammaherpesvirus 4 (Epstein-Barr virus)
10376	GCA_900004105.1	Human gammaherpesvirus 4 (Epstein-Barr virus)
10376	GCA_900003425.1	Human gammaherpesvirus 4 (Epstein-Barr virus)
10376	GCA_900013545.1	Human gammaherpesvirus 4 (Epstein-Barr virus)
10376	GCA_900004305.1	Human gammaherpesvirus 4 (Epstein-Barr virus)
10376	GCA_900003805.1	Human gammaherpesvirus 4 (Epstein-Barr virus)
37296	GCA_000838265.1	Human gammaherpesvirus 8
1792832	GCA_002826025.1	Human gemycircularvirus GeTz1
1488574	GCA_000973295.1	Human genital-associated circular DNA virus-1
1986030	GCA_002624565.1	Human gokushovirus
1986031	GCA_002922405.1	Human gut gokushovirus
1986031	GCA_002922305.1	Human gut gokushovirus
1986031	GCA_002922385.1	Human gut gokushovirus
1986031	GCA_002922315.1	Human gut gokushovirus
1986031	GCA_002922325.1	Human gut gokushovirus
1986031	GCA_002922375.1	Human gut gokushovirus
1986031	GCA_002922345.1	Human gut gokushovirus
1986031	GCA_002922335.1	Human gut gokushovirus
1986031	GCA_002922355.1	Human gut gokushovirus
1986031	GCA_002922365.1	Human gut gokushovirus
1986031	GCA_002922395.1	Human gut gokushovirus
1986032	GCA_002624585.1	Human gut microviridae SH-CHD12
1986033	GCA_002624605.1	Human gut microviridae SH-CHD8
1704090	GCA_002821385.1	Human hepegivirus

10338	GCA_000858285.1	Human herpesvirus 3 strain Dumas (Varicella-zoster virus)
12721	GCA_003102595.1	Human immunodeficiency virus
12721	GCA_003109145.1	Human immunodeficiency virus
12721	GCA_003102915.1	Human immunodeficiency virus
12721	GCA_003190765.1	Human immunodeficiency virus
12721	GCA_003856135.1	Human immunodeficiency virus
11676	GCA_000864765.1	Human immunodeficiency virus 1
11676	GCA_003100455.1	Human immunodeficiency virus 1
11676	GCA_003104055.1	Human immunodeficiency virus 1
11676	GCA_003104795.1	Human immunodeficiency virus 1
11676	GCA_003100475.1	Human immunodeficiency virus 1
11676	GCA_003098415.1	Human immunodeficiency virus 1
11676	GCA_003104175.1	Human immunodeficiency virus 1
11676	GCA_003103715.1	Human immunodeficiency virus 1
11676	GCA_003100495.1	Human immunodeficiency virus 1
11676	GCA_003098395.1	Human immunodeficiency virus 1
11676	GCA_003098055.1	Human immunodeficiency virus 1
11676	GCA_003103395.1	Human immunodeficiency virus 1
11676	GCA_003098455.1	Human immunodeficiency virus 1
11676	GCA_003100515.1	Human immunodeficiency virus 1
11676	GCA_003102155.1	Human immunodeficiency virus 1
11676	GCA_003104515.1	Human immunodeficiency virus 1
11676	GCA_003098475.1	Human immunodeficiency virus 1
11676	GCA_003101815.1	Human immunodeficiency virus 1
11676	GCA_003103055.1	Human immunodeficiency virus 1
11676	GCA_003101475.1	Human immunodeficiency virus 1
11676	GCA_003101135.1	Human immunodeficiency virus 1
11676	GCA_003098495.1	Human immunodeficiency virus 1
11676	GCA_003102715.1	Human immunodeficiency virus 1
11676	GCA_003104815.1	Human immunodeficiency virus 1
11676	GCA_003108645.1	Human immunodeficiency virus 1
11676	GCA_003103855.1	Human immunodeficiency virus 1
11676	GCA_003104195.1	Human immunodeficiency virus 1
11676	GCA_003108665.1	Human immunodeficiency virus 1
11676	GCA_003098555.1	Human immunodeficiency virus 1
11676	GCA_003103515.1	Human immunodeficiency virus 1
11676	GCA_003098075.1	Human immunodeficiency virus 1
11676	GCA_003108685.1	Human immunodeficiency virus 1
11676	GCA_003108705.1	Human immunodeficiency virus 1
11676	GCA_003102175.1	Human immunodeficiency virus 1
11676	GCA_003101835.1	Human immunodeficiency virus 1
11676	GCA_003101495.1	Human immunodeficiency virus 1
11676	GCA_003108725.1	Human immunodeficiency virus 1
11676	GCA_003108745.1	Human immunodeficiency virus 1
11676	GCA_003102575.1	Human immunodeficiency virus 1
11676	GCA_003103995.1	Human immunodeficiency virus 1
11676	GCA_003104835.1	Human immunodeficiency virus 1
11676	GCA_003108765.1	Human immunodeficiency virus 1
11676	GCA_003102515.1	Human immunodeficiency virus 1
11676	GCA_003104535.1	Human immunodeficiency virus 1

11676	GCA_003098655.1	Human immunodeficiency virus 1
11676	GCA_003100715.1	Human immunodeficiency virus 1
11676	GCA_003104215.1	Human immunodeficiency virus 1
11676	GCA_003098775.1	Human immunodeficiency virus 1
11676	GCA_003103655.1	Human immunodeficiency virus 1
11676	GCA_003098675.1	Human immunodeficiency virus 1
11676	GCA_003098095.1	Human immunodeficiency virus 1
11676	GCA_003108805.1	Human immunodeficiency virus 1
11676	GCA_003100735.1	Human immunodeficiency virus 1
11676	GCA_003098695.1	Human immunodeficiency virus 1
11676	GCA_003108825.1	Human immunodeficiency virus 1
11676	GCA_003100755.1	Human immunodeficiency virus 1
11676	GCA_003102195.1	Human immunodeficiency virus 1
11676	GCA_003098715.1	Human immunodeficiency virus 1
11676	GCA_003108845.1	Human immunodeficiency virus 1
11676	GCA_003102995.1	Human immunodeficiency virus 1
11676	GCA_003101175.1	Human immunodeficiency virus 1
11676	GCA_003100835.1	Human immunodeficiency virus 1
11676	GCA_003108865.1	Human immunodeficiency virus 1
11676	GCA_003100795.1	Human immunodeficiency virus 1
11676	GCA_003104135.1	Human immunodeficiency virus 1
11676	GCA_003102655.1	Human immunodeficiency virus 1
11676	GCA_003104855.1	Human immunodeficiency virus 1
11676	GCA_003108885.1	Human immunodeficiency virus 1
11676	GCA_003100815.1	Human immunodeficiency virus 1
11676	GCA_003103795.1	Human immunodeficiency virus 1
11676	GCA_003104235.1	Human immunodeficiency virus 1
11676	GCA_003108905.1	Human immunodeficiency virus 1
11676	GCA_003098795.1	Human immunodeficiency virus 1
11676	GCA_003108925.1	Human immunodeficiency virus 1
11676	GCA_003103475.1	Human immunodeficiency virus 1
11676	GCA_003108945.1	Human immunodeficiency virus 1
11676	GCA_003100875.1	Human immunodeficiency virus 1
11676	GCA_003102215.1	Human immunodeficiency virus 1
11676	GCA_003101875.1	Human immunodeficiency virus 1
11676	GCA_003103135.1	Human immunodeficiency virus 1
11676	GCA_003101535.1	Human immunodeficiency virus 1
11676	GCA_003108965.1	Human immunodeficiency virus 1
11676	GCA_003100855.1	Human immunodeficiency virus 1
11676	GCA_003103215.1	Human immunodeficiency virus 1
11676	GCA_003108985.1	Human immunodeficiency virus 1
11676	GCA_003102795.1	Human immunodeficiency virus 1
11676	GCA_003100915.1	Human immunodeficiency virus 1
11676	GCA_003098875.1	Human immunodeficiency virus 1
11676	GCA_003104875.1	Human immunodeficiency virus 1
11676	GCA_003109005.1	Human immunodeficiency virus 1
11676	GCA_003098895.1	Human immunodeficiency virus 1
11676	GCA_003102455.1	Human immunodeficiency virus 1
11676	GCA_003104255.1	Human immunodeficiency virus 1
11676	GCA_003109025.1	Human immunodeficiency virus 1

11676	GCA_003100955.1	Human immunodeficiency virus 1
11676	GCA_003109045.1	Human immunodeficiency virus 1
11676	GCA_003100975.1	Human immunodeficiency virus 1
11676	GCA_003098935.1	Human immunodeficiency virus 1
11676	GCA_003103275.1	Human immunodeficiency virus 1
11676	GCA_003102235.1	Human immunodeficiency virus 1
11676	GCA_003101555.1	Human immunodeficiency virus 1
11676	GCA_003109085.1	Human immunodeficiency virus 1
11676	GCA_003101055.1	Human immunodeficiency virus 1
11676	GCA_003100535.1	Human immunodeficiency virus 1
11676	GCA_003101075.1	Human immunodeficiency virus 1
11676	GCA_003099015.1	Human immunodeficiency virus 1
11676	GCA_003103735.1	Human immunodeficiency virus 1
11676	GCA_003098835.1	Human immunodeficiency virus 1
11676	GCA_003103555.1	Human immunodeficiency virus 1
11676	GCA_003099035.1	Human immunodeficiency virus 1
11676	GCA_003101095.1	Human immunodeficiency virus 1
11676	GCA_003103415.1	Human immunodeficiency virus 1
11676	GCA_003101115.1	Human immunodeficiency virus 1
11676	GCA_003102255.1	Human immunodeficiency virus 1
11676	GCA_003099075.1	Human immunodeficiency virus 1
11676	GCA_003101915.1	Human immunodeficiency virus 1
11676	GCA_003101575.1	Human immunodeficiency virus 1
11676	GCA_003099095.1	Human immunodeficiency virus 1
11676	GCA_003103095.1	Human immunodeficiency virus 1
11676	GCA_003100895.1	Human immunodeficiency virus 1
11676	GCA_003101155.1	Human immunodeficiency virus 1
11676	GCA_003102735.1	Human immunodeficiency virus 1
11676	GCA_003099115.1	Human immunodeficiency virus 1
11676	GCA_003102855.1	Human immunodeficiency virus 1
11676	GCA_003109245.1	Human immunodeficiency virus 1
11676	GCA_003104595.1	Human immunodeficiency virus 1
11676	GCA_003103875.1	Human immunodeficiency virus 1
11676	GCA_003099135.1	Human immunodeficiency virus 1
11676	GCA_003104275.1	Human immunodeficiency virus 1
11676	GCA_003109265.1	Human immunodeficiency virus 1
11676	GCA_003101195.1	Human immunodeficiency virus 1
11676	GCA_003098855.1	Human immunodeficiency virus 1
11676	GCA_003099155.1	Human immunodeficiency virus 1
11676	GCA_003103535.1	Human immunodeficiency virus 1
11676	GCA_003109285.1	Human immunodeficiency virus 1
11676	GCA_003103315.1	Human immunodeficiency virus 1
11676	GCA_003101215.1	Human immunodeficiency virus 1
11676	GCA_003099175.1	Human immunodeficiency virus 1
11676	GCA_003109305.1	Human immunodeficiency virus 1
11676	GCA_003101235.1	Human immunodeficiency virus 1
11676	GCA_003102275.1	Human immunodeficiency virus 1
11676	GCA_003099195.1	Human immunodeficiency virus 1
11676	GCA_003101595.1	Human immunodeficiency virus 1
11676	GCA_003101255.1	Human immunodeficiency virus 1

11676	GCA_003099215.1	Human immunodeficiency virus 1
11676	GCA_003102875.1	Human immunodeficiency virus 1
11676	GCA_003101275.1	Human immunodeficiency virus 1
11676	GCA_003100585.1	Human immunodeficiency virus 1
11676	GCA_003099235.1	Human immunodeficiency virus 1
11676	GCA_003104015.1	Human immunodeficiency virus 1
11676	GCA_003102535.1	Human immunodeficiency virus 1
11676	GCA_003104615.1	Human immunodeficiency virus 1
11676	GCA_003099255.1	Human immunodeficiency virus 1
11676	GCA_003101315.1	Human immunodeficiency virus 1
11676	GCA_003103675.1	Human immunodeficiency virus 1
11676	GCA_003099275.1	Human immunodeficiency virus 1
11676	GCA_003098535.1	Human immunodeficiency virus 1
11676	GCA_003098195.1	Human immunodeficiency virus 1
11676	GCA_003101335.1	Human immunodeficiency virus 1
11676	GCA_003099295.1	Human immunodeficiency virus 1
11676	GCA_003101355.1	Human immunodeficiency virus 1
11676	GCA_003102295.1	Human immunodeficiency virus 1
11676	GCA_003101855.1	Human immunodeficiency virus 1
11676	GCA_003099315.1	Human immunodeficiency virus 1
11676	GCA_003102675.1	Human immunodeficiency virus 1
11676	GCA_003101615.1	Human immunodeficiency virus 1
11676	GCA_003103015.1	Human immunodeficiency virus 1
11676	GCA_003099335.1	Human immunodeficiency virus 1
11676	GCA_003101395.1	Human immunodeficiency virus 1
11676	GCA_003100605.1	Human immunodeficiency virus 1
11676	GCA_003099355.1	Human immunodeficiency virus 1
11676	GCA_003101415.1	Human immunodeficiency virus 1
11676	GCA_003099375.1	Human immunodeficiency virus 1
11676	GCA_003098175.1	Human immunodeficiency virus 1
11676	GCA_003101435.1	Human immunodeficiency virus 1
11676	GCA_003099395.1	Human immunodeficiency virus 1
11676	GCA_003101455.1	Human immunodeficiency virus 1
11676	GCA_003103495.1	Human immunodeficiency virus 1
11676	GCA_003099415.1	Human immunodeficiency virus 1
11676	GCA_003102315.1	Human immunodeficiency virus 1
11676	GCA_003099435.1	Human immunodeficiency virus 1
11676	GCA_003103155.1	Human immunodeficiency virus 1
11676	GCA_003101635.1	Human immunodeficiency virus 1
11676	GCA_003102935.1	Human immunodeficiency virus 1
11676	GCA_003101295.1	Human immunodeficiency virus 1
11676	GCA_003099455.1	Human immunodeficiency virus 1
11676	GCA_003100635.1	Human immunodeficiency virus 1
11676	GCA_003099475.1	Human immunodeficiency virus 1
11676	GCA_003099495.1	Human immunodeficiency virus 1
11676	GCA_003102475.1	Human immunodeficiency virus 1
11676	GCA_003098915.1	Human immunodeficiency virus 1
11676	GCA_003099515.1	Human immunodeficiency virus 1
11676	GCA_003098575.1	Human immunodeficiency virus 1
11676	GCA_003103615.1	Human immunodeficiency virus 1

11676	GCA_003097895.1	Human immunodeficiency virus 1
11676	GCA_003099535.1	Human immunodeficiency virus 1
11676	GCA_003103295.1	Human immunodeficiency virus 1
11676	GCA_003099555.1	Human immunodeficiency virus 1
11676	GCA_003101655.1	Human immunodeficiency virus 1
11676	GCA_003099575.1	Human immunodeficiency virus 1
11676	GCA_003103195.1	Human immunodeficiency virus 1
11676	GCA_003100655.1	Human immunodeficiency virus 1
11676	GCA_003099595.1	Human immunodeficiency virus 1
11676	GCA_003104095.1	Human immunodeficiency virus 1
11676	GCA_003102615.1	Human immunodeficiency virus 1
11676	GCA_003099615.1	Human immunodeficiency virus 1
11676	GCA_003104355.1	Human immunodeficiency virus 1
11676	GCA_003103755.1	Human immunodeficiency virus 1
11676	GCA_003104035.1	Human immunodeficiency virus 1
11676	GCA_003099635.1	Human immunodeficiency virus 1
11676	GCA_003098595.1	Human immunodeficiency virus 1
11676	GCA_003098255.1	Human immunodeficiency virus 1
11676	GCA_003101695.1	Human immunodeficiency virus 1
11676	GCA_003103435.1	Human immunodeficiency virus 1
11676	GCA_003099655.1	Human immunodeficiency virus 1
11676	GCA_003103935.1	Human immunodeficiency virus 1
11676	GCA_003103815.1	Human immunodeficiency virus 1
11676	GCA_003099675.1	Human immunodeficiency virus 1
11676	GCA_003102015.1	Human immunodeficiency virus 1
11676	GCA_003103695.1	Human immunodeficiency virus 1
11676	GCA_003101675.1	Human immunodeficiency virus 1
11676	GCA_003109065.1	Human immunodeficiency virus 1
11676	GCA_003103575.1	Human immunodeficiency virus 1
11676	GCA_003099695.1	Human immunodeficiency virus 1
11676	GCA_003100995.1	Human immunodeficiency virus 1
11676	GCA_003103455.1	Human immunodeficiency virus 1
11676	GCA_003100675.1	Human immunodeficiency virus 1
11676	GCA_003102755.1	Human immunodeficiency virus 1
11676	GCA_003099715.1	Human immunodeficiency virus 1
11676	GCA_003101775.1	Human immunodeficiency virus 1
11676	GCA_003103895.1	Human immunodeficiency virus 1
11676	GCA_003102555.1	Human immunodeficiency virus 1
11676	GCA_003099735.1	Human immunodeficiency virus 1
11676	GCA_003103355.1	Human immunodeficiency virus 1
11676	GCA_003104375.1	Human immunodeficiency virus 1
11676	GCA_003102415.1	Human immunodeficiency virus 1
11676	GCA_003101795.1	Human immunodeficiency virus 1
11676	GCA_003098955.1	Human immunodeficiency virus 1
11676	GCA_003099755.1	Human immunodeficiency virus 1
11676	GCA_003098615.1	Human immunodeficiency virus 1
11676	GCA_003103115.1	Human immunodeficiency virus 1
11676	GCA_003099775.1	Human immunodeficiency virus 1
11676	GCA_003103235.1	Human immunodeficiency virus 1
11676	GCA_003099795.1	Human immunodeficiency virus 1

11676	GCA_003099815.1	Human immunodeficiency virus 1
11676	GCA_003101035.1	Human immunodeficiency virus 1
11676	GCA_003102895.1	Human immunodeficiency virus 1
11676	GCA_003097775.1	Human immunodeficiency virus 1
11676	GCA_003100695.1	Human immunodeficiency virus 1
11676	GCA_003099835.1	Human immunodeficiency virus 1
11676	GCA_003101895.1	Human immunodeficiency virus 1
11676	GCA_003104715.1	Human immunodeficiency virus 1
11676	GCA_003099855.1	Human immunodeficiency virus 1
11676	GCA_003102815.1	Human immunodeficiency virus 1
11676	GCA_003097815.1	Human immunodeficiency virus 1
11676	GCA_003098975.1	Human immunodeficiency virus 1
11676	GCA_003103955.1	Human immunodeficiency virus 1
11676	GCA_003099875.1	Human immunodeficiency virus 1
11676	GCA_003098635.1	Human immunodeficiency virus 1
11676	GCA_003097835.1	Human immunodeficiency virus 1
11676	GCA_003098295.1	Human immunodeficiency virus 1
11676	GCA_003097955.1	Human immunodeficiency virus 1
11676	GCA_003099895.1	Human immunodeficiency virus 1
11676	GCA_003097855.1	Human immunodeficiency virus 1
11676	GCA_003099915.1	Human immunodeficiency virus 1
11676	GCA_003102055.1	Human immunodeficiency virus 1
11676	GCA_003097875.1	Human immunodeficiency virus 1
11676	GCA_003101715.1	Human immunodeficiency virus 1
11676	GCA_003103035.1	Human immunodeficiency virus 1
11676	GCA_003099935.1	Human immunodeficiency virus 1
11676	GCA_003099955.1	Human immunodeficiency virus 1
11676	GCA_003097915.1	Human immunodeficiency virus 1
11676	GCA_003104735.1	Human immunodeficiency virus 1
11676	GCA_003099995.1	Human immunodeficiency virus 1
11676	GCA_003104415.1	Human immunodeficiency virus 1
11676	GCA_003102035.1	Human immunodeficiency virus 1
11676	GCA_003098995.1	Human immunodeficiency virus 1
11676	GCA_003103075.1	Human immunodeficiency virus 1
11676	GCA_003100015.1	Human immunodeficiency virus 1
11676	GCA_003097975.1	Human immunodeficiency virus 1
11676	GCA_003100035.1	Human immunodeficiency virus 1
11676	GCA_003100055.1	Human immunodeficiency virus 1
11676	GCA_003102075.1	Human immunodeficiency virus 1
11676	GCA_003103175.1	Human immunodeficiency virus 1
11676	GCA_003101735.1	Human immunodeficiency virus 1
11676	GCA_003098015.1	Human immunodeficiency virus 1
11676	GCA_003100075.1	Human immunodeficiency virus 1
11676	GCA_003102835.1	Human immunodeficiency virus 1
11676	GCA_003098035.1	Human immunodeficiency virus 1
11676	GCA_003103975.1	Human immunodeficiency virus 1
11676	GCA_003100095.1	Human immunodeficiency virus 1
11676	GCA_003102495.1	Human immunodeficiency virus 1
11676	GCA_003100115.1	Human immunodeficiency virus 1
11676	GCA_003103635.1	Human immunodeficiency virus 1

11676	GCA_003103335.1	Human immunodeficiency virus 1
11676	GCA_003097995.1	Human immunodeficiency virus 1
11676	GCA_003100135.1	Human immunodeficiency virus 1
11676	GCA_003102435.1	Human immunodeficiency virus 1
11676	GCA_003100155.1	Human immunodeficiency virus 1
11676	GCA_003102095.1	Human immunodeficiency virus 1
11676	GCA_003098115.1	Human immunodeficiency virus 1
11676	GCA_003101755.1	Human immunodeficiency virus 1
11676	GCA_003098815.1	Human immunodeficiency virus 1
11676	GCA_003100175.1	Human immunodeficiency virus 1
11676	GCA_003102975.1	Human immunodeficiency virus 1
11676	GCA_003100195.1	Human immunodeficiency virus 1
11676	GCA_003104115.1	Human immunodeficiency virus 1
11676	GCA_003098155.1	Human immunodeficiency virus 1
11676	GCA_003102635.1	Human immunodeficiency virus 1
11676	GCA_003104755.1	Human immunodeficiency virus 1
11676	GCA_003100215.1	Human immunodeficiency virus 1
11676	GCA_003103775.1	Human immunodeficiency virus 1
11676	GCA_003100235.1	Human immunodeficiency virus 1
11676	GCA_003100255.1	Human immunodeficiency virus 1
11676	GCA_003097795.1	Human immunodeficiency virus 1
11676	GCA_003102695.1	Human immunodeficiency virus 1
11676	GCA_003100275.1	Human immunodeficiency virus 1
11676	GCA_003102115.1	Human immunodeficiency virus 1
11676	GCA_003098235.1	Human immunodeficiency virus 1
11676	GCA_003100295.1	Human immunodeficiency virus 1
11676	GCA_003104075.1	Human immunodeficiency virus 1
11676	GCA_003102775.1	Human immunodeficiency virus 1
11676	GCA_003100315.1	Human immunodeficiency virus 1
11676	GCA_003104775.1	Human immunodeficiency virus 1
11676	GCA_003103835.1	Human immunodeficiency virus 1
11676	GCA_003103915.1	Human immunodeficiency virus 1
11676	GCA_003100335.1	Human immunodeficiency virus 1
11676	GCA_003104475.1	Human immunodeficiency virus 1
11676	GCA_003104155.1	Human immunodeficiency virus 1
11676	GCA_003100355.1	Human immunodeficiency virus 1
11676	GCA_003098315.1	Human immunodeficiency virus 1
11676	GCA_003100375.1	Human immunodeficiency virus 1
11676	GCA_003103595.1	Human immunodeficiency virus 1
11676	GCA_003103255.1	Human immunodeficiency virus 1
11676	GCA_003100405.1	Human immunodeficiency virus 1
11676	GCA_003102135.1	Human immunodeficiency virus 1
11676	GCA_003103375.1	Human immunodeficiency virus 1
11676	GCA_003102955.1	Human immunodeficiency virus 1
11676	GCA_003098355.1	Human immunodeficiency virus 1
11676	GCA_003100435.1	Human immunodeficiency virus 1
11676	GCA_003098375.1	Human immunodeficiency virus 1
11676	GCA_003100775.1	Human immunodeficiency virus 1
11676	GCA_003189285.1	Human immunodeficiency virus 1
11676	GCA_003189645.1	Human immunodeficiency virus 1

11676	GCA_003187595.1	Human immunodeficiency virus 1
11676	GCA_003189705.1	Human immunodeficiency virus 1
11676	GCA_003187665.1	Human immunodeficiency virus 1
11676	GCA_003187685.1	Human immunodeficiency virus 1
11676	GCA_003189745.1	Human immunodeficiency virus 1
11676	GCA_003187705.1	Human immunodeficiency virus 1
11676	GCA_003189765.1	Human immunodeficiency virus 1
11676	GCA_003187725.1	Human immunodeficiency virus 1
11676	GCA_003189825.1	Human immunodeficiency virus 1
11676	GCA_003187745.1	Human immunodeficiency virus 1
11676	GCA_003190305.1	Human immunodeficiency virus 1
11676	GCA_003187765.1	Human immunodeficiency virus 1
11676	GCA_003189665.1	Human immunodeficiency virus 1
11676	GCA_003189875.1	Human immunodeficiency virus 1
11676	GCA_003187785.1	Human immunodeficiency virus 1
11676	GCA_003187825.1	Human immunodeficiency virus 1
11676	GCA_003187625.1	Human immunodeficiency virus 1
11676	GCA_003187855.1	Human immunodeficiency virus 1
11676	GCA_003189925.1	Human immunodeficiency virus 1
11676	GCA_003187885.1	Human immunodeficiency virus 1
11676	GCA_003190185.1	Human immunodeficiency virus 1
11676	GCA_003187905.1	Human immunodeficiency virus 1
11676	GCA_003187925.1	Human immunodeficiency virus 1
11676	GCA_003189985.1	Human immunodeficiency virus 1
11676	GCA_003187945.1	Human immunodeficiency virus 1
11676	GCA_003187965.1	Human immunodeficiency virus 1
11676	GCA_003190065.1	Human immunodeficiency virus 1
11676	GCA_003187995.1	Human immunodeficiency virus 1
11676	GCA_003190705.1	Human immunodeficiency virus 1
11676	GCA_003188045.1	Human immunodeficiency virus 1
11676	GCA_003190125.1	Human immunodeficiency virus 1
11676	GCA_003188085.1	Human immunodeficiency virus 1
11676	GCA_003188105.1	Human immunodeficiency virus 1
11676	GCA_003190165.1	Human immunodeficiency virus 1
11676	GCA_003188125.1	Human immunodeficiency virus 1
11676	GCA_003190145.1	Human immunodeficiency virus 1
11676	GCA_003188155.1	Human immunodeficiency virus 1
11676	GCA_003189725.1	Human immunodeficiency virus 1
11676	GCA_003192245.1	Human immunodeficiency virus 1
11676	GCA_003190215.1	Human immunodeficiency virus 1
11676	GCA_003192265.1	Human immunodeficiency virus 1
11676	GCA_003188205.1	Human immunodeficiency virus 1
11676	GCA_003190245.1	Human immunodeficiency virus 1
11676	GCA_003192285.1	Human immunodeficiency virus 1
11676	GCA_003188245.1	Human immunodeficiency virus 1
11676	GCA_003192305.1	Human immunodeficiency virus 1
11676	GCA_003190265.1	Human immunodeficiency virus 1
11676	GCA_003192325.1	Human immunodeficiency virus 1
11676	GCA_003188275.1	Human immunodeficiency virus 1
11676	GCA_003190285.1	Human immunodeficiency virus 1

11676	GCA_003202495.1	Human immunodeficiency virus 1
11676	GCA_003190685.1	Human immunodeficiency virus 1
11676	GCA_003188305.1	Human immunodeficiency virus 1
11676	GCA_003189385.1	Human immunodeficiency virus 1
11676	GCA_003188325.1	Human immunodeficiency virus 1
11676	GCA_003190325.1	Human immunodeficiency virus 1
11676	GCA_003190355.1	Human immunodeficiency virus 1
11676	GCA_003188345.1	Human immunodeficiency virus 1
11676	GCA_003190415.1	Human immunodeficiency virus 1
11676	GCA_003188365.1	Human immunodeficiency virus 1
11676	GCA_003190455.1	Human immunodeficiency virus 1
11676	GCA_003190085.1	Human immunodeficiency virus 1
11676	GCA_003190485.1	Human immunodeficiency virus 1
11676	GCA_003188405.1	Human immunodeficiency virus 1
11676	GCA_003190505.1	Human immunodeficiency virus 1
11676	GCA_003188455.1	Human immunodeficiency virus 1
11676	GCA_003188485.1	Human immunodeficiency virus 1
11676	GCA_003190565.1	Human immunodeficiency virus 1
11676	GCA_003190545.1	Human immunodeficiency virus 1
11676	GCA_003188525.1	Human immunodeficiency virus 1
11676	GCA_003188545.1	Human immunodeficiency virus 1
11676	GCA_003190585.1	Human immunodeficiency virus 1
11676	GCA_003188565.1	Human immunodeficiency virus 1
11676	GCA_003190785.1	Human immunodeficiency virus 1
11676	GCA_003190605.1	Human immunodeficiency virus 1
11676	GCA_003188585.1	Human immunodeficiency virus 1
11676	GCA_003188605.1	Human immunodeficiency virus 1
11676	GCA_003190025.1	Human immunodeficiency virus 1
11676	GCA_003188625.1	Human immunodeficiency virus 1
11676	GCA_003188665.1	Human immunodeficiency virus 1
11676	GCA_003188705.1	Human immunodeficiency virus 1
11676	GCA_003188725.1	Human immunodeficiency virus 1
11676	GCA_003190525.1	Human immunodeficiency virus 1
11676	GCA_003190805.1	Human immunodeficiency virus 1
11676	GCA_003188765.1	Human immunodeficiency virus 1
11676	GCA_003188785.1	Human immunodeficiency virus 1
11676	GCA_003190885.1	Human immunodeficiency virus 1
11676	GCA_003188805.1	Human immunodeficiency virus 1
11676	GCA_003188865.1	Human immunodeficiency virus 1
11676	GCA_003188885.1	Human immunodeficiency virus 1
11676	GCA_003189845.1	Human immunodeficiency virus 1
11676	GCA_003189165.1	Human immunodeficiency virus 1
11676	GCA_003188925.1	Human immunodeficiency virus 1
11676	GCA_003188955.1	Human immunodeficiency virus 1
11676	GCA_003188985.1	Human immunodeficiency virus 1
11676	GCA_003189025.1	Human immunodeficiency virus 1
11676	GCA_003192225.1	Human immunodeficiency virus 1
11676	GCA_003189045.1	Human immunodeficiency virus 1
11676	GCA_003189085.1	Human immunodeficiency virus 1
11676	GCA_003189105.1	Human immunodeficiency virus 1

11676	GCA_003189135.1	Human immunodeficiency virus 1
11676	GCA_003190845.1	Human immunodeficiency virus 1
11676	GCA_003189185.1	Human immunodeficiency virus 1
11676	GCA_003189225.1	Human immunodeficiency virus 1
11676	GCA_003189255.1	Human immunodeficiency virus 1
11676	GCA_003189905.1	Human immunodeficiency virus 1
11676	GCA_003189305.1	Human immunodeficiency virus 1
11676	GCA_003189335.1	Human immunodeficiency virus 1
11676	GCA_003189365.1	Human immunodeficiency virus 1
11676	GCA_003189425.1	Human immunodeficiency virus 1
11676	GCA_003190645.1	Human immunodeficiency virus 1
11676	GCA_003189445.1	Human immunodeficiency virus 1
11676	GCA_003189485.1	Human immunodeficiency virus 1
11676	GCA_003189505.1	Human immunodeficiency virus 1
11676	GCA_003189555.1	Human immunodeficiency virus 1
11676	GCA_003189575.1	Human immunodeficiency virus 1
11676	GCA_003193605.1	Human immunodeficiency virus 1
11676	GCA_003189595.1	Human immunodeficiency virus 1
11676	GCA_003189965.1	Human immunodeficiency virus 1
11676	GCA_003855675.1	Human immunodeficiency virus 1
11676	GCA_003855695.1	Human immunodeficiency virus 1
11676	GCA_003855715.1	Human immunodeficiency virus 1
11676	GCA_003855735.1	Human immunodeficiency virus 1
11676	GCA_003855755.1	Human immunodeficiency virus 1
11676	GCA_003855775.1	Human immunodeficiency virus 1
11676	GCA_003855795.1	Human immunodeficiency virus 1
11676	GCA_003855815.1	Human immunodeficiency virus 1
11676	GCA_003855835.1	Human immunodeficiency virus 1
11676	GCA_003856155.1	Human immunodeficiency virus 1
11676	GCA_003855855.1	Human immunodeficiency virus 1
11676	GCA_003855875.1	Human immunodeficiency virus 1
11676	GCA_003855895.1	Human immunodeficiency virus 1
11676	GCA_003855915.1	Human immunodeficiency virus 1
11676	GCA_003855935.1	Human immunodeficiency virus 1
11676	GCA_003855955.1	Human immunodeficiency virus 1
11676	GCA_003855975.1	Human immunodeficiency virus 1
11676	GCA_003855995.1	Human immunodeficiency virus 1
11676	GCA_003856015.1	Human immunodeficiency virus 1
11676	GCA_003856035.1	Human immunodeficiency virus 1
11676	GCA_003856055.1	Human immunodeficiency virus 1
11676	GCA_003856075.1	Human immunodeficiency virus 1
11676	GCA_003856095.1	Human immunodeficiency virus 1
11676	GCA_003856115.1	Human immunodeficiency virus 1
11709	GCA_000856385.1	Human immunodeficiency virus 2
11709	GCA_003104495.1	Human immunodeficiency virus 2
11709	GCA_003102375.1	Human immunodeficiency virus 2
11709	GCA_003104555.1	Human immunodeficiency virus 2
11709	GCA_003098135.1	Human immunodeficiency virus 2
11709	GCA_003104575.1	Human immunodeficiency virus 2
11709	GCA_003100555.1	Human immunodeficiency virus 2

11709	GCA_003102395.1	Human immunodeficiency virus 2
11709	GCA_003098515.1	Human immunodeficiency virus 2
11709	GCA_003109345.1	Human immunodeficiency virus 2
11709	GCA_003109445.1	Human immunodeficiency virus 2
11709	GCA_003104635.1	Human immunodeficiency virus 2
11709	GCA_003104315.1	Human immunodeficiency virus 2
11709	GCA_003104655.1	Human immunodeficiency virus 2
11709	GCA_003104675.1	Human immunodeficiency virus 2
11709	GCA_003102355.1	Human immunodeficiency virus 2
11709	GCA_003191385.1	Human immunodeficiency virus 2
11709	GCA_003191445.1	Human immunodeficiency virus 2
11709	GCA_003191145.1	Human immunodeficiency virus 2
11709	GCA_003192645.1	Human immunodeficiency virus 2
11709	GCA_003191525.1	Human immunodeficiency virus 2
11709	GCA_003192665.1	Human immunodeficiency virus 2
11709	GCA_003191205.1	Human immunodeficiency virus 2
11709	GCA_003190925.1	Human immunodeficiency virus 2
11709	GCA_003191005.1	Human immunodeficiency virus 2
11709	GCA_003191025.1	Human immunodeficiency virus 2
11709	GCA_003191045.1	Human immunodeficiency virus 2
11709	GCA_003191095.1	Human immunodeficiency virus 2
11709	GCA_003191225.1	Human immunodeficiency virus 2
11709	GCA_003191125.1	Human immunodeficiency virus 2
11709	GCA_003191175.1	Human immunodeficiency virus 2
11709	GCA_003191245.1	Human immunodeficiency virus 2
11709	GCA_003191295.1	Human immunodeficiency virus 2
11709	GCA_003191345.1	Human immunodeficiency virus 2
11709	GCA_003191405.1	Human immunodeficiency virus 2
11709	GCA_003191485.1	Human immunodeficiency virus 2
11709	GCA_003190945.1	Human immunodeficiency virus 2
11709	GCA_003191545.1	Human immunodeficiency virus 2
11709	GCA_003191325.1	Human immunodeficiency virus 2
467352	GCA_003109105.1	Human immunodeficiency virus type 1 02CD.LBR024
467353	GCA_003109125.1	Human immunodeficiency virus type 1 04CD.FR.KZS
130308	GCA_000859665.1	Human mastadenovirus E
130309	GCA_000846685.1	Human mastadenovirus F
162145	GCA_000865625.1	Human metapneumovirus
162145	GCA_002815415.1	Human metapneumovirus
162145	GCA_002815435.1	Human metapneumovirus
162145	GCA_002815375.1	Human metapneumovirus
162145	GCA_002815395.1	Human metapneumovirus
11250	GCA_000855545.1	Human orthopneumovirus
11250	GCA_002815475.1	Human orthopneumovirus
10566	GCA_001274345.1	Human papillomavirus
151757	GCA_003177495.1	Human papillomavirus - cand85
587349	GCA_003178415.1	Human papillomavirus 100
518628	GCA_003178435.1	Human papillomavirus 104
587350	GCA_003178455.1	Human papillomavirus 105
427343	GCA_003178055.1	Human papillomavirus 107
915426	GCA_000883695.1	Human papillomavirus 109

518629	GCA_003178215.1	Human papillomavirus 110
518630	GCA_003178235.1	Human papillomavirus 111
587351	GCA_003178475.1	Human papillomavirus 113
915428	GCA_000884175.1	Human papillomavirus 116
720708	GCA_003178615.1	Human papillomavirus 117
765052	GCA_003178675.1	Human papillomavirus 120
915429	GCA_000889075.1	Human papillomavirus 121
765054	GCA_003178695.1	Human papillomavirus 122
765055	GCA_003178715.1	Human papillomavirus 123
673323	GCA_003178495.1	Human papillomavirus 125
1055684	GCA_000896435.1	Human papillomavirus 126
746832	GCA_000890115.1	Human papillomavirus 127
909329	GCA_003178815.1	Human papillomavirus 130
909331	GCA_000888015.1	Human papillomavirus 132
909332	GCA_003178835.1	Human papillomavirus 133
909333	GCA_000889695.1	Human papillomavirus 134
1070408	GCA_000898235.1	Human papillomavirus 135
1070409	GCA_000899695.1	Human papillomavirus 136
1070411	GCA_003178915.1	Human papillomavirus 138
1070412	GCA_003178935.1	Human papillomavirus 139
1070413	GCA_000898855.1	Human papillomavirus 140
1070414	GCA_003178955.1	Human papillomavirus 141
1070415	GCA_003178975.1	Human papillomavirus 142
1070416	GCA_003178995.1	Human papillomavirus 143
1070418	GCA_003179015.1	Human papillomavirus 145
1070419	GCA_003179035.1	Human papillomavirus 146
942038	GCA_003178855.1	Human papillomavirus 148
909328	GCA_003178795.1	Human papillomavirus 149
10606	GCA_003180595.1	Human papillomavirus 15
743812	GCA_003178535.1	Human papillomavirus 151
990302	GCA_003179155.1	Human papillomavirus 152
1195796	GCA_000908695.1	Human papillomavirus 154
1165934	GCA_003179195.1	Human papillomavirus 155
2259331	GCA_003179815.1	Human papillomavirus 157
2259343	GCA_003179855.1	Human papillomavirus 158
1209820	GCA_003178875.1	Human papillomavirus 159
2259332	GCA_003177475.1	Human papillomavirus 160
1315266	GCA_003179455.1	Human papillomavirus 165
1420545	GCA_000913075.1	Human papillomavirus 167
1315260	GCA_003179375.1	Human papillomavirus 169
10607	GCA_003180615.1	Human papillomavirus 17
1315265	GCA_003179435.1	Human papillomavirus 170
1434986	GCA_003179575.1	Human papillomavirus 171
1434987	GCA_002827005.1	Human papillomavirus 172
1434988	GCA_003179595.1	Human papillomavirus 173
1347832	GCA_003178895.1	Human papillomavirus 174
1434782	GCA_002827025.1	Human papillomavirus 175
1478160	GCA_000918095.1	Human papillomavirus 178
1472342	GCA_000912535.1	Human papillomavirus 179
1449827	GCA_003179495.1	Human papillomavirus 180

1472343	GCA_002987365.1	Human papillomavirus 184
1851130	GCA_003055605.1	Human papillomavirus 187
10608	GCA_003180635.1	Human papillomavirus 19
1542134	GCA_003179635.1	Human papillomavirus 197
1545700	GCA_003179615.1	Human papillomavirus 199
1682340	GCA_001184845.1	Human papillomavirus 201
1682341	GCA_003179715.1	Human papillomavirus 202
1650736	GCA_002827045.1	Human papillomavirus 204
2259334	GCA_003179835.1	Human papillomavirus 205
37954	GCA_003180215.1	Human papillomavirus 22
37955	GCA_003180235.1	Human papillomavirus 23
37111	GCA_003180275.1	Human papillomavirus 28
37112	GCA_003180295.1	Human papillomavirus 29
10614	GCA_003180535.1	Human papillomavirus 3
37958	GCA_003180335.1	Human papillomavirus 37
37959	GCA_003180355.1	Human papillomavirus 38
10617	GCA_000864845.1	Human papillomavirus 4
333923	GCA_000866505.1	Human papillomavirus 5
28312	GCA_003180515.1	Human papillomavirus 65
69986	GCA_003180895.1	Human papillomavirus 77
69987	GCA_003180915.1	Human papillomavirus 80
10621	GCA_000863685.1	Human papillomavirus 9
247268	GCA_003177775.1	Human papillomavirus 93
260717	GCA_003177755.1	Human papillomavirus 94
260716	GCA_003177735.1	Human papillomavirus 95
587347	GCA_003178375.1	Human papillomavirus 98
587348	GCA_003178395.1	Human papillomavirus 99
1647924	GCA_000989155.1	Human papillomavirus KC5
192971	GCA_004287915.1	Human papillomavirus sp.
333759	GCA_000864905.1	Human papillomavirus type 10
915425	GCA_000869845.1	Human papillomavirus type 101
338327	GCA_003177975.1	Human papillomavirus type 102
338323	GCA_000868225.1	Human papillomavirus type 103
338326	GCA_003177955.1	Human papillomavirus type 106
565537	GCA_000883655.1	Human papillomavirus type 108
10580	GCA_003179995.1	Human papillomavirus type 11
915427	GCA_000882135.1	Human papillomavirus type 112
735496	GCA_003178595.1	Human papillomavirus type 114
696746	GCA_003178355.1	Human papillomavirus type 115
927771	GCA_003178635.1	Human papillomavirus type 118
765051	GCA_003178655.1	Human papillomavirus type 119
10604	GCA_003180555.1	Human papillomavirus type 12
765056	GCA_003178735.1	Human papillomavirus type 124
931209	GCA_000889675.1	Human papillomavirus type 128
931210	GCA_000891495.1	Human papillomavirus type 129
10573	GCA_003180475.1	Human papillomavirus type 13
909330	GCA_000890535.1	Human papillomavirus type 131
1070410	GCA_000897255.1	Human papillomavirus type 137
1070417	GCA_000898255.1	Human papillomavirus type 144
1070420	GCA_003179055.1	Human papillomavirus type 147

31546	GCA_003180575.1	Human papillomavirus type 14D
743811	GCA_003178515.1	Human papillomavirus type 150
1110710	GCA_003179215.1	Human papillomavirus type 153
1248396	GCA_002006915.1	Human papillomavirus type 156
333760	GCA_000863945.2	Human papillomavirus type 16
1315264	GCA_002826985.1	Human papillomavirus type 161
1315263	GCA_003179415.1	Human papillomavirus type 162
1315262	GCA_001430115.1	Human papillomavirus type 163
1315261	GCA_003179395.1	Human papillomavirus type 164
1315259	GCA_000899515.1	Human papillomavirus type 166
1420544	GCA_003179555.1	Human papillomavirus type 168
333761	GCA_000865665.1	Human papillomavirus type 18
1851215	GCA_003147485.1	Human papillomavirus type 190
1851219	GCA_003147505.1	Human papillomavirus type 194
10583	GCA_000866405.1	Human papillomavirus type 1a
31547	GCA_003180175.1	Human papillomavirus type 20
1682339	GCA_003179695.1	Human papillomavirus type 200
2093789	GCA_004133345.1	Human papillomavirus type 203
31548	GCA_003180195.1	Human papillomavirus type 21
37956	GCA_003180255.1	Human papillomavirus type 24
10609	GCA_003180655.1	Human papillomavirus type 25
333762	GCA_000866485.1	Human papillomavirus type 26
333752	GCA_003180675.1	Human papillomavirus type 27
10584	GCA_000864945.1	Human papillomavirus type 2a
10611	GCA_002987345.1	Human papillomavirus type 30
10585	GCA_003179095.1	Human papillomavirus type 31
333763	GCA_000864925.1	Human papillomavirus type 32
10586	GCA_003179955.1	Human papillomavirus type 33
333764	GCA_000863965.1	Human papillomavirus type 34
31551	GCA_003180695.1	Human papillomavirus type 35H
37957	GCA_003180315.1	Human papillomavirus type 36
10588	GCA_003180055.1	Human papillomavirus type 39
10615	GCA_003180715.1	Human papillomavirus type 40
10589	GCA_000863845.1	Human papillomavirus type 41
10590	GCA_003180095.1	Human papillomavirus type 42
10591	GCA_003177675.1	Human papillomavirus type 43
10592	GCA_003180375.1	Human papillomavirus type 44
10593	GCA_003180735.1	Human papillomavirus type 45
10594	GCA_003180035.1	Human papillomavirus type 47
40538	GCA_000839365.1	Human papillomavirus type 48
10616	GCA_000862825.1	Human papillomavirus type 49
40539	GCA_000841625.1	Human papillomavirus type 50
10595	GCA_003180075.1	Human papillomavirus type 51
10618	GCA_003180755.1	Human papillomavirus type 52
333765	GCA_000865685.1	Human papillomavirus type 53
1671798	GCA_000864725.1	Human papillomavirus type 54
10596	GCA_003180775.1	Human papillomavirus type 56
333753	GCA_003180455.1	Human papillomavirus type 57
10598	GCA_003177895.1	Human papillomavirus type 58
37115	GCA_003180795.1	Human papillomavirus type 59

40540	GCA_000838505.1	Human papillomavirus type 60
37116	GCA_000862805.1	Human papillomavirus type 61
334210	GCA_003177795.1	Human papillomavirus type 62
28311	GCA_000865565.1	Human papillomavirus type 63
37119	GCA_003180395.1	Human papillomavirus type 66
37120	GCA_003177875.1	Human papillomavirus type 67
338322	GCA_003177915.1	Human papillomavirus type 68a
37121	GCA_003177375.1	Human papillomavirus type 69
10600	GCA_000861945.1	Human papillomavirus type 6b
10620	GCA_000861925.1	Human papillomavirus type 7
39457	GCA_003180155.1	Human papillomavirus type 70
120686	GCA_002826825.1	Human papillomavirus type 71
333770	GCA_003180815.1	Human papillomavirus type 72
51033	GCA_003180835.1	Human papillomavirus type 73
44028	GCA_003177615.1	Human papillomavirus type 74
69984	GCA_003180855.1	Human papillomavirus type 75
69985	GCA_003180875.1	Human papillomavirus type 76
10579	GCA_003179975.1	Human papillomavirus type 8
333771	GCA_003177715.1	Human papillomavirus type 81
129724	GCA_003177395.1	Human papillomavirus type 82
333772	GCA_003177515.1	Human papillomavirus type 83
150546	GCA_003177535.1	Human papillomavirus type 84
652810	GCA_002153865.1	Human papillomavirus type 85
171370	GCA_003177555.1	Human papillomavirus type 86
120381	GCA_003177655.1	Human papillomavirus type 87
337054	GCA_000874925.1	Human papillomavirus type 88
202250	GCA_003177595.1	Human papillomavirus type 89
333769	GCA_000862685.1	Human papillomavirus type 90
202252	GCA_003177575.1	Human papillomavirus type 91
211787	GCA_000846285.1	Human papillomavirus type 92
247269	GCA_000864825.1	Human papillomavirus type 96
338324	GCA_003177935.1	Human papillomavirus type 97
1288116	GCA_003179515.1	Human papillomavirus type XS2
188538	GCA_000848705.1	Human parainfluenza virus 1 strain Washington/1964
11224	GCA_000910675.1	Human parainfluenza virus 4a
12063	GCA_002817305.1	Human parechovirus 1
39085	GCA_000861505.1	Human parechovirus 2
1511919	GCA_000861005.1	Human parvovirus 4 G1
10798	GCA_000839645.1	Human parvovirus B19
10798	GCA_002827345.1	Human parvovirus B19
1729141	GCA_001310135.2	Human pegivirus 2
1729141	GCA_003052225.1	Human pegivirus 2
145856	GCA_000859285.1	Human picobirnavirus
12080	GCA_003108525.1	Human poliovirus 1
12080	GCA_003108545.1	Human poliovirus 1
12080	GCA_003108565.1	Human poliovirus 1
12080	GCA_003108585.1	Human poliovirus 1
12081	GCA_000861165.1	Human poliovirus 1 Mahoney
12083	GCA_003108605.1	Human poliovirus 2
12086	GCA_003112265.1	Human poliovirus 3

1891762	GCA_000837865.1	Human polyomavirus 1
1891762	GCA_900231585.1	Human polyomavirus 1
1891762	GCA_900232185.1	Human polyomavirus 1
1303334	GCA_000906235.1	Human polyomavirus 12
746830	GCA_000888495.1	Human polyomavirus 6
746831	GCA_000889175.1	Human polyomavirus 7
943908	GCA_000891615.1	Human polyomavirus 9
208895	GCA_003266585.1	Human respiratory syncytial virus B
208895	GCA_003267745.1	Human respiratory syncytial virus B
208895	GCA_003267525.1	Human respiratory syncytial virus B
208895	GCA_003267605.1	Human respiratory syncytial virus B
208895	GCA_003267265.1	Human respiratory syncytial virus B
208895	GCA_003267065.1	Human respiratory syncytial virus B
208895	GCA_003266605.1	Human respiratory syncytial virus B
208895	GCA_003267285.1	Human respiratory syncytial virus B
208895	GCA_003267885.1	Human respiratory syncytial virus B
208895	GCA_003266945.1	Human respiratory syncytial virus B
208895	GCA_003267305.1	Human respiratory syncytial virus B
208895	GCA_003266625.1	Human respiratory syncytial virus B
208895	GCA_003267345.1	Human respiratory syncytial virus B
208895	GCA_003267645.1	Human respiratory syncytial virus B
208895	GCA_003267005.1	Human respiratory syncytial virus B
208895	GCA_003267845.1	Human respiratory syncytial virus B
208895	GCA_003267805.1	Human respiratory syncytial virus B
208895	GCA_003267485.1	Human respiratory syncytial virus B
208895	GCA_003267325.1	Human respiratory syncytial virus B
208895	GCA_003266985.1	Human respiratory syncytial virus B
208895	GCA_003266645.1	Human respiratory syncytial virus B
208895	GCA_003267625.1	Human respiratory syncytial virus B
208895	GCA_003266665.1	Human respiratory syncytial virus B
208895	GCA_003267705.1	Human respiratory syncytial virus B
208895	GCA_003267025.1	Human respiratory syncytial virus B
208895	GCA_003267445.1	Human respiratory syncytial virus B
208895	GCA_003267565.1	Human respiratory syncytial virus B
208895	GCA_003267725.1	Human respiratory syncytial virus B
208895	GCA_003267045.1	Human respiratory syncytial virus B
208895	GCA_003266705.1	Human respiratory syncytial virus B
208895	GCA_003266525.1	Human respiratory syncytial virus B
208895	GCA_003267365.1	Human respiratory syncytial virus B
208895	GCA_003267405.1	Human respiratory syncytial virus B
208895	GCA_003266565.1	Human respiratory syncytial virus B
208895	GCA_003267505.1	Human respiratory syncytial virus B
208895	GCA_003267665.1	Human respiratory syncytial virus B
208895	GCA_003267085.1	Human respiratory syncytial virus B
208895	GCA_003266825.1	Human respiratory syncytial virus B
208895	GCA_003266685.1	Human respiratory syncytial virus B
208895	GCA_003266725.1	Human respiratory syncytial virus B
208895	GCA_003267765.1	Human respiratory syncytial virus B
208895	GCA_003266745.1	Human respiratory syncytial virus B
208895	GCA_003267105.1	Human respiratory syncytial virus B

208895	GCA_003266765.1	Human respiratory syncytial virus B
208895	GCA_003266785.1	Human respiratory syncytial virus B
208895	GCA_003266805.1	Human respiratory syncytial virus B
208895	GCA_003266845.1	Human respiratory syncytial virus B
208895	GCA_003266865.1	Human respiratory syncytial virus B
208895	GCA_003267465.1	Human respiratory syncytial virus B
208895	GCA_003266905.1	Human respiratory syncytial virus B
208895	GCA_003267545.1	Human respiratory syncytial virus B
208895	GCA_003266925.1	Human respiratory syncytial virus B
208895	GCA_003267585.1	Human respiratory syncytial virus B
208895	GCA_003266965.1	Human respiratory syncytial virus B
208895	GCA_003267245.1	Human respiratory syncytial virus B
208895	GCA_003267145.1	Human respiratory syncytial virus B
208895	GCA_003267825.1	Human respiratory syncytial virus B
208895	GCA_003267425.1	Human respiratory syncytial virus B
208895	GCA_003267385.1	Human respiratory syncytial virus B
208895	GCA_003267165.1	Human respiratory syncytial virus B
208895	GCA_003267185.1	Human respiratory syncytial virus B
208895	GCA_003267865.1	Human respiratory syncytial virus B
208895	GCA_003267205.1	Human respiratory syncytial virus B
208895	GCA_003266545.1	Human respiratory syncytial virus B
208895	GCA_003267785.1	Human respiratory syncytial virus B
208895	GCA_003267125.1	Human respiratory syncytial virus B
208895	GCA_003267225.1	Human respiratory syncytial virus B
208895	GCA_003266885.1	Human respiratory syncytial virus B
208895	GCA_003267685.1	Human respiratory syncytial virus B
11216	GCA_000850205.1	Human respirovirus 3
573824	GCA_002816835.1	Human rhinovirus A1
12132	GCA_000862245.1	Human rhinovirus A89
44130	GCA_002816855.1	Human rhinovirus B3
992230	GCA_002816885.1	Human rhinovirus NAT001
10941	GCA_002635415.1	Human rotavirus A
10941	GCA_002637475.1	Human rotavirus A
10941	GCA_002640875.1	Human rotavirus A
10941	GCA_002637495.1	Human rotavirus A
10941	GCA_002640115.1	Human rotavirus A
10941	GCA_002637515.1	Human rotavirus A
10941	GCA_002639515.1	Human rotavirus A
10941	GCA_002637535.1	Human rotavirus A
10941	GCA_002637555.1	Human rotavirus A
10941	GCA_002637575.1	Human rotavirus A
10941	GCA_002640155.1	Human rotavirus A
10941	GCA_002637595.1	Human rotavirus A
10941	GCA_002640895.1	Human rotavirus A
10941	GCA_002637615.1	Human rotavirus A
10941	GCA_002637635.1	Human rotavirus A
10941	GCA_002637655.1	Human rotavirus A
10941	GCA_002637675.1	Human rotavirus A
10941	GCA_002637695.1	Human rotavirus A
10941	GCA_002637715.1	Human rotavirus A

10941	GCA_002640915.1	Human rotavirus A
10941	GCA_002637735.1	Human rotavirus A
10941	GCA_002637755.1	Human rotavirus A
10941	GCA_002637775.1	Human rotavirus A
10941	GCA_002638535.1	Human rotavirus A
10941	GCA_002637795.1	Human rotavirus A
10941	GCA_002637815.1	Human rotavirus A
10941	GCA_002637835.1	Human rotavirus A
10941	GCA_002640935.1	Human rotavirus A
10941	GCA_002637855.1	Human rotavirus A
10941	GCA_002637875.1	Human rotavirus A
10941	GCA_002637895.1	Human rotavirus A
10941	GCA_002637915.1	Human rotavirus A
10941	GCA_002637935.1	Human rotavirus A
10941	GCA_002637955.1	Human rotavirus A
10941	GCA_002640955.1	Human rotavirus A
10941	GCA_002637975.1	Human rotavirus A
10941	GCA_002637995.1	Human rotavirus A
10941	GCA_002638015.1	Human rotavirus A
10941	GCA_002638035.1	Human rotavirus A
10941	GCA_002638055.1	Human rotavirus A
10941	GCA_002638075.1	Human rotavirus A
10941	GCA_002640975.1	Human rotavirus A
10941	GCA_002638095.1	Human rotavirus A
10941	GCA_002639955.1	Human rotavirus A
10941	GCA_002638135.1	Human rotavirus A
10941	GCA_002638155.1	Human rotavirus A
10941	GCA_002638175.1	Human rotavirus A
10941	GCA_002638195.1	Human rotavirus A
10941	GCA_002640995.1	Human rotavirus A
10941	GCA_002638215.1	Human rotavirus A
10941	GCA_002636175.1	Human rotavirus A
10941	GCA_002638235.1	Human rotavirus A
10941	GCA_002639295.1	Human rotavirus A
10941	GCA_002638255.1	Human rotavirus A
10941	GCA_002638275.1	Human rotavirus A
10941	GCA_002638295.1	Human rotavirus A
10941	GCA_002638315.1	Human rotavirus A
10941	GCA_002638335.1	Human rotavirus A
10941	GCA_002638355.1	Human rotavirus A
10941	GCA_002638375.1	Human rotavirus A
10941	GCA_002638395.1	Human rotavirus A
10941	GCA_002638415.1	Human rotavirus A
10941	GCA_002638435.1	Human rotavirus A
10941	GCA_002638455.1	Human rotavirus A
10941	GCA_002638475.1	Human rotavirus A
10941	GCA_002638495.1	Human rotavirus A
10941	GCA_002638515.1	Human rotavirus A
10941	GCA_002637295.1	Human rotavirus A
10941	GCA_002638555.1	Human rotavirus A

10941	GCA_002638575.1	Human rotavirus A
10941	GCA_002640375.1	Human rotavirus A
10941	GCA_002636535.1	Human rotavirus A
10941	GCA_002638595.1	Human rotavirus A
10941	GCA_002636555.1	Human rotavirus A
10941	GCA_002638615.1	Human rotavirus A
10941	GCA_002636575.1	Human rotavirus A
10941	GCA_002638635.1	Human rotavirus A
10941	GCA_002636595.1	Human rotavirus A
10941	GCA_002638655.1	Human rotavirus A
10941	GCA_002636615.1	Human rotavirus A
10941	GCA_002638675.1	Human rotavirus A
10941	GCA_002636635.1	Human rotavirus A
10941	GCA_002638695.1	Human rotavirus A
10941	GCA_002640395.1	Human rotavirus A
10941	GCA_002636655.1	Human rotavirus A
10941	GCA_002636675.1	Human rotavirus A
10941	GCA_002638735.1	Human rotavirus A
10941	GCA_002638115.1	Human rotavirus A
10941	GCA_002640135.1	Human rotavirus A
10941	GCA_002636695.1	Human rotavirus A
10941	GCA_002638755.1	Human rotavirus A
10941	GCA_002636715.1	Human rotavirus A
10941	GCA_002638775.1	Human rotavirus A
10941	GCA_002636735.1	Human rotavirus A
10941	GCA_002634695.1	Human rotavirus A
10941	GCA_002638795.1	Human rotavirus A
10941	GCA_002638815.1	Human rotavirus A
10941	GCA_002636775.1	Human rotavirus A
10941	GCA_002638835.1	Human rotavirus A
10941	GCA_002639735.1	Human rotavirus A
10941	GCA_002636795.1	Human rotavirus A
10941	GCA_002638855.1	Human rotavirus A
10941	GCA_002638715.1	Human rotavirus A
10941	GCA_002636815.1	Human rotavirus A
10941	GCA_002638895.1	Human rotavirus A
10941	GCA_002640535.1	Human rotavirus A
10941	GCA_002636855.1	Human rotavirus A
10941	GCA_002638915.1	Human rotavirus A
10941	GCA_002636875.1	Human rotavirus A
10941	GCA_002638935.1	Human rotavirus A
10941	GCA_002636895.1	Human rotavirus A
10941	GCA_002638955.1	Human rotavirus A
10941	GCA_002636915.1	Human rotavirus A
10941	GCA_002638975.1	Human rotavirus A
10941	GCA_002636935.1	Human rotavirus A
10941	GCA_002638995.1	Human rotavirus A
10941	GCA_002636955.1	Human rotavirus A
10941	GCA_002639015.1	Human rotavirus A
10941	GCA_002636975.1	Human rotavirus A

10941	GCA_002639035.1	Human rotavirus A
10941	GCA_002636995.1	Human rotavirus A
10941	GCA_002640795.1	Human rotavirus A
10941	GCA_002640455.1	Human rotavirus A
10941	GCA_002637015.1	Human rotavirus A
10941	GCA_002639075.1	Human rotavirus A
10941	GCA_002637035.1	Human rotavirus A
10941	GCA_002639095.1	Human rotavirus A
10941	GCA_002637055.1	Human rotavirus A
10941	GCA_002639115.1	Human rotavirus A
10941	GCA_002639135.1	Human rotavirus A
10941	GCA_002637095.1	Human rotavirus A
10941	GCA_002637115.1	Human rotavirus A
10941	GCA_002640815.1	Human rotavirus A
10941	GCA_002637135.1	Human rotavirus A
10941	GCA_002637155.1	Human rotavirus A
10941	GCA_002637175.1	Human rotavirus A
10941	GCA_002637195.1	Human rotavirus A
10941	GCA_002635145.1	Human rotavirus A
10941	GCA_002637215.1	Human rotavirus A
10941	GCA_002637235.1	Human rotavirus A
10941	GCA_002635195.1	Human rotavirus A
10941	GCA_002637255.1	Human rotavirus A
10941	GCA_002640195.1	Human rotavirus A
10941	GCA_002635215.1	Human rotavirus A
10941	GCA_002637275.1	Human rotavirus A
10941	GCA_002635235.1	Human rotavirus A
10941	GCA_002640615.1	Human rotavirus A
10941	GCA_002635255.1	Human rotavirus A
10941	GCA_002640175.1	Human rotavirus A
10941	GCA_002637315.1	Human rotavirus A
10941	GCA_002635275.1	Human rotavirus A
10941	GCA_002637335.1	Human rotavirus A
10941	GCA_002636755.1	Human rotavirus A
10941	GCA_002637355.1	Human rotavirus A
10941	GCA_002640855.1	Human rotavirus A
10941	GCA_002637375.1	Human rotavirus A
10941	GCA_002637395.1	Human rotavirus A
10941	GCA_002637415.1	Human rotavirus A
10941	GCA_002637435.1	Human rotavirus A
10941	GCA_002637455.1	Human rotavirus A
10941	GCA_002661375.1	Human rotavirus A
10941	GCA_002655215.1	Human rotavirus A
10941	GCA_002660935.1	Human rotavirus A
10941	GCA_002664015.1	Human rotavirus A
10941	GCA_002641615.1	Human rotavirus A
10941	GCA_002662095.1	Human rotavirus A
10941	GCA_002641635.1	Human rotavirus A
10941	GCA_002641655.1	Human rotavirus A
10941	GCA_002664415.1	Human rotavirus A

10941	GCA_002642255.1	Human rotavirus A
10941	GCA_002641675.1	Human rotavirus A
10941	GCA_002658415.1	Human rotavirus A
10941	GCA_002655235.1	Human rotavirus A
10941	GCA_002661635.1	Human rotavirus A
10941	GCA_002662235.1	Human rotavirus A
10941	GCA_002641755.1	Human rotavirus A
10941	GCA_002641775.1	Human rotavirus A
10941	GCA_002642275.1	Human rotavirus A
10941	GCA_002661555.1	Human rotavirus A
10941	GCA_002643855.1	Human rotavirus A
10941	GCA_002641255.1	Human rotavirus A
10941	GCA_002662395.1	Human rotavirus A
10941	GCA_002661215.1	Human rotavirus A
10941	GCA_002643875.1	Human rotavirus A
10941	GCA_002641835.1	Human rotavirus A
10941	GCA_002662155.1	Human rotavirus A
10941	GCA_002662375.1	Human rotavirus A
10941	GCA_002655255.1	Human rotavirus A
10941	GCA_002643895.1	Human rotavirus A
10941	GCA_002641855.1	Human rotavirus A
10941	GCA_002661895.1	Human rotavirus A
10941	GCA_002660995.1	Human rotavirus A
10941	GCA_002643935.1	Human rotavirus A
10941	GCA_002661915.1	Human rotavirus A
10941	GCA_002641895.1	Human rotavirus A
10941	GCA_002661795.1	Human rotavirus A
10941	GCA_002650095.1	Human rotavirus A
10941	GCA_002661695.1	Human rotavirus A
10941	GCA_002661675.1	Human rotavirus A
10941	GCA_002662855.1	Human rotavirus A
10941	GCA_002661355.1	Human rotavirus A
10941	GCA_002641955.1	Human rotavirus A
10941	GCA_002655275.1	Human rotavirus A
10941	GCA_002654935.1	Human rotavirus A
10941	GCA_002641995.1	Human rotavirus A
10941	GCA_002650195.1	Human rotavirus A
10941	GCA_002661835.1	Human rotavirus A
10941	GCA_002661015.1	Human rotavirus A
10941	GCA_002642315.1	Human rotavirus A
10941	GCA_002641975.1	Human rotavirus A
10941	GCA_002661495.1	Human rotavirus A
10941	GCA_002641295.1	Human rotavirus A
10941	GCA_002659755.1	Human rotavirus A
10941	GCA_002646175.1	Human rotavirus A
10941	GCA_002658855.1	Human rotavirus A
10941	GCA_002654955.1	Human rotavirus A
10941	GCA_002664635.1	Human rotavirus A
10941	GCA_002642335.1	Human rotavirus A
10941	GCA_002642155.1	Human rotavirus A

10941	GCA_002658635.1	Human rotavirus A
10941	GCA_002641315.1	Human rotavirus A
10941	GCA_002659795.1	Human rotavirus A
10941	GCA_002642195.1	Human rotavirus A
10941	GCA_002662455.1	Human rotavirus A
10941	GCA_002661415.1	Human rotavirus A
10941	GCA_002654975.1	Human rotavirus A
10941	GCA_002659455.1	Human rotavirus A
10941	GCA_002646315.1	Human rotavirus A
10941	GCA_002662115.1	Human rotavirus A
10941	GCA_002661775.1	Human rotavirus A
10941	GCA_002642355.1	Human rotavirus A
10941	GCA_002642015.1	Human rotavirus A
10941	GCA_002646375.1	Human rotavirus A
10941	GCA_002661435.1	Human rotavirus A
10941	GCA_002641335.1	Human rotavirus A
10941	GCA_002642295.1	Human rotavirus A
10941	GCA_002646395.1	Human rotavirus A
10941	GCA_002654995.1	Human rotavirus A
10941	GCA_002662255.1	Human rotavirus A
10941	GCA_002657535.1	Human rotavirus A
10941	GCA_002657755.1	Human rotavirus A
10941	GCA_002646475.1	Human rotavirus A
10941	GCA_002642375.1	Human rotavirus A
10941	GCA_002662335.1	Human rotavirus A
10941	GCA_002661575.1	Human rotavirus A
10941	GCA_002642035.1	Human rotavirus A
10941	GCA_002662035.1	Human rotavirus A
10941	GCA_002641695.1	Human rotavirus A
10941	GCA_002641355.1	Human rotavirus A
10941	GCA_002642415.1	Human rotavirus A
10941	GCA_002661235.1	Human rotavirus A
10941	GCA_002646515.1	Human rotavirus A
10941	GCA_002641015.1	Human rotavirus A
10941	GCA_002661135.1	Human rotavirus A
10941	GCA_002661155.1	Human rotavirus A
10941	GCA_002642435.1	Human rotavirus A
10941	GCA_002646535.1	Human rotavirus A
10941	GCA_002655355.1	Human rotavirus A
10941	GCA_002655015.1	Human rotavirus A
10941	GCA_002661935.1	Human rotavirus A
10941	GCA_002642475.1	Human rotavirus A
10941	GCA_002642495.1	Human rotavirus A
10941	GCA_002661715.1	Human rotavirus A
10941	GCA_002642395.1	Human rotavirus A
10941	GCA_002642055.1	Human rotavirus A
10941	GCA_002662295.1	Human rotavirus A
10941	GCA_002641715.1	Human rotavirus A
10941	GCA_002641375.1	Human rotavirus A
10941	GCA_002642535.1	Human rotavirus A

10941	GCA_002646635.1	Human rotavirus A
10941	GCA_002656875.1	Human rotavirus A
10941	GCA_002659535.1	Human rotavirus A
10941	GCA_002655035.1	Human rotavirus A
10941	GCA_002646695.1	Human rotavirus A
10941	GCA_002661855.1	Human rotavirus A
10941	GCA_002646715.1	Human rotavirus A
10941	GCA_002642075.1	Human rotavirus A
10941	GCA_002646735.1	Human rotavirus A
10941	GCA_002661515.1	Human rotavirus A
10941	GCA_002641735.1	Human rotavirus A
10941	GCA_002661395.1	Human rotavirus A
10941	GCA_002646755.1	Human rotavirus A
10941	GCA_002661275.1	Human rotavirus A
10941	GCA_002661175.1	Human rotavirus A
10941	GCA_002646775.1	Human rotavirus A
10941	GCA_002655055.1	Human rotavirus A
10941	GCA_002650895.1	Human rotavirus A
10941	GCA_002646815.1	Human rotavirus A
10941	GCA_002661995.1	Human rotavirus A
10941	GCA_002646835.1	Human rotavirus A
10941	GCA_002642095.1	Human rotavirus A
10941	GCA_002646855.1	Human rotavirus A
10941	GCA_002641415.1	Human rotavirus A
10941	GCA_002662815.1	Human rotavirus A
10941	GCA_002646895.1	Human rotavirus A
10941	GCA_002660195.1	Human rotavirus A
10941	GCA_002651055.1	Human rotavirus A
10941	GCA_002642455.1	Human rotavirus A
10941	GCA_002642115.1	Human rotavirus A
10941	GCA_002661455.1	Human rotavirus A
10941	GCA_002663075.1	Human rotavirus A
10941	GCA_002655095.1	Human rotavirus A
10941	GCA_002662275.1	Human rotavirus A
10941	GCA_002662175.1	Human rotavirus A
10941	GCA_002661875.1	Human rotavirus A
10941	GCA_002661595.1	Human rotavirus A
10941	GCA_002642135.1	Human rotavirus A
10941	GCA_002641795.1	Human rotavirus A
10941	GCA_002663635.1	Human rotavirus A
10941	GCA_002641115.1	Human rotavirus A
10941	GCA_002657095.1	Human rotavirus A
10941	GCA_002661255.1	Human rotavirus A
10941	GCA_002655115.1	Human rotavirus A
10941	GCA_002657975.1	Human rotavirus A
10941	GCA_002662435.1	Human rotavirus A
10941	GCA_002662075.1	Human rotavirus A
10941	GCA_002641035.1	Human rotavirus A
10941	GCA_002661735.1	Human rotavirus A
10941	GCA_002641075.1	Human rotavirus A

10941	GCA_002641815.1	Human rotavirus A
10941	GCA_002641135.1	Human rotavirus A
10941	GCA_002641095.1	Human rotavirus A
10941	GCA_002664055.1	Human rotavirus A
10941	GCA_002655135.1	Human rotavirus A
10941	GCA_002662215.1	Human rotavirus A
10941	GCA_002645275.1	Human rotavirus A
10941	GCA_002662135.1	Human rotavirus A
10941	GCA_002642515.1	Human rotavirus A
10941	GCA_002642175.1	Human rotavirus A
10941	GCA_002657315.1	Human rotavirus A
10941	GCA_002641155.1	Human rotavirus A
10941	GCA_002662355.1	Human rotavirus A
10941	GCA_002655155.1	Human rotavirus A
10941	GCA_002661655.1	Human rotavirus A
10941	GCA_002659095.1	Human rotavirus A
10941	GCA_002662015.1	Human rotavirus A
10941	GCA_002658195.1	Human rotavirus A
10941	GCA_002662055.1	Human rotavirus A
10941	GCA_002661335.1	Human rotavirus A
10941	GCA_002641175.1	Human rotavirus A
10941	GCA_002662415.1	Human rotavirus A
10941	GCA_002662495.1	Human rotavirus A
10941	GCA_002655175.1	Human rotavirus A
10941	GCA_002663655.1	Human rotavirus A
10941	GCA_002643915.1	Human rotavirus A
10941	GCA_002641395.1	Human rotavirus A
10941	GCA_002645495.1	Human rotavirus A
10941	GCA_002642555.1	Human rotavirus A
10941	GCA_002642215.1	Human rotavirus A
10941	GCA_002662315.1	Human rotavirus A
10941	GCA_002641875.1	Human rotavirus A
10941	GCA_002661475.1	Human rotavirus A
10941	GCA_002659975.1	Human rotavirus A
10941	GCA_002662635.1	Human rotavirus A
10941	GCA_002661115.1	Human rotavirus A
10941	GCA_002655195.1	Human rotavirus A
10941	GCA_002641515.1	Human rotavirus A
10941	GCA_002661955.1	Human rotavirus A
10941	GCA_002659315.1	Human rotavirus A
10941	GCA_002661615.1	Human rotavirus A
10941	GCA_002642235.1	Human rotavirus A
10941	GCA_002677515.1	Human rotavirus A
10941	GCA_002666135.1	Human rotavirus A
10941	GCA_002681095.1	Human rotavirus A
10941	GCA_002670235.1	Human rotavirus A
10941	GCA_002677175.1	Human rotavirus A
10941	GCA_002668195.1	Human rotavirus A
10941	GCA_002670255.1	Human rotavirus A
10941	GCA_002668215.1	Human rotavirus A

10941	GCA_002670275.1	Human rotavirus A
10941	GCA_002682175.1	Human rotavirus A
10941	GCA_002678255.1	Human rotavirus A
10941	GCA_002681515.1	Human rotavirus A
10941	GCA_002679915.1	Human rotavirus A
10941	GCA_002670295.1	Human rotavirus A
10941	GCA_002678275.1	Human rotavirus A
10941	GCA_002679235.1	Human rotavirus A
10941	GCA_002670315.1	Human rotavirus A
10941	GCA_002681175.1	Human rotavirus A
10941	GCA_002668275.1	Human rotavirus A
10941	GCA_002682255.1	Human rotavirus A
10941	GCA_002678215.1	Human rotavirus A
10941	GCA_002670335.1	Human rotavirus A
10941	GCA_002682335.1	Human rotavirus A
10941	GCA_002677875.1	Human rotavirus A
10941	GCA_002668295.1	Human rotavirus A
10941	GCA_002682415.1	Human rotavirus A
10941	GCA_002680835.1	Human rotavirus A
10941	GCA_002677535.1	Human rotavirus A
10941	GCA_002670355.1	Human rotavirus A
10941	GCA_002668315.1	Human rotavirus A
10941	GCA_002681995.1	Human rotavirus A
10941	GCA_002670375.1	Human rotavirus A
10941	GCA_002680495.1	Human rotavirus A
10941	GCA_002668335.1	Human rotavirus A
10941	GCA_002665815.1	Human rotavirus A
10941	GCA_002670395.1	Human rotavirus A
10941	GCA_002679595.1	Human rotavirus A
10941	GCA_002668375.1	Human rotavirus A
10941	GCA_002681315.1	Human rotavirus A
10941	GCA_002679255.1	Human rotavirus A
10941	GCA_002670435.1	Human rotavirus A
10941	GCA_002668395.1	Human rotavirus A
10941	GCA_002682475.1	Human rotavirus A
10941	GCA_002678235.1	Human rotavirus A
10941	GCA_002670455.1	Human rotavirus A
10941	GCA_002680975.1	Human rotavirus A
10941	GCA_002677895.1	Human rotavirus A
10941	GCA_002668415.1	Human rotavirus A
10941	GCA_002677555.1	Human rotavirus A
10941	GCA_002670475.1	Human rotavirus A
10941	GCA_002668435.1	Human rotavirus A
10941	GCA_002670495.1	Human rotavirus A
10941	GCA_002665835.1	Human rotavirus A
10941	GCA_002670515.1	Human rotavirus A
10941	GCA_002680295.1	Human rotavirus A
10941	GCA_002668475.1	Human rotavirus A
10941	GCA_002679955.1	Human rotavirus A
10941	GCA_002670535.1	Human rotavirus A

10941	GCA_002681455.1	Human rotavirus A
10941	GCA_002679615.1	Human rotavirus A
10941	GCA_002668495.1	Human rotavirus A
10941	GCA_002670555.1	Human rotavirus A
10941	GCA_002682615.1	Human rotavirus A
10941	GCA_002668515.1	Human rotavirus A
10941	GCA_002668575.1	Human rotavirus A
10941	GCA_002670575.1	Human rotavirus A
10941	GCA_002668535.1	Human rotavirus A
10941	GCA_002682275.1	Human rotavirus A
10941	GCA_002670595.1	Human rotavirus A
10941	GCA_002668555.1	Human rotavirus A
10941	GCA_002670615.1	Human rotavirus A
10941	GCA_002680435.1	Human rotavirus A
10941	GCA_002670635.1	Human rotavirus A
10941	GCA_002681595.1	Human rotavirus A
10941	GCA_002679975.1	Human rotavirus A
10941	GCA_002670655.1	Human rotavirus A
10941	GCA_002679635.1	Human rotavirus A
10941	GCA_002668615.1	Human rotavirus A
10941	GCA_002679295.1	Human rotavirus A
10941	GCA_002681255.1	Human rotavirus A
10941	GCA_002670675.1	Human rotavirus A
10941	GCA_002668595.1	Human rotavirus A
10941	GCA_002682155.1	Human rotavirus A
10941	GCA_002676615.1	Human rotavirus A
10941	GCA_002668255.1	Human rotavirus A
10941	GCA_002670695.1	Human rotavirus A
10941	GCA_002668655.1	Human rotavirus A
10941	GCA_002670715.1	Human rotavirus A
10941	GCA_002682075.1	Human rotavirus A
10941	GCA_002680575.1	Human rotavirus A
10941	GCA_002670735.1	Human rotavirus A
10941	GCA_002682435.1	Human rotavirus A
10941	GCA_002668695.1	Human rotavirus A
10941	GCA_002665875.1	Human rotavirus A
10941	GCA_002670755.1	Human rotavirus A
10941	GCA_002665535.1	Human rotavirus A
10941	GCA_002680235.1	Human rotavirus A
10941	GCA_002668715.1	Human rotavirus A
10941	GCA_002665195.1	Human rotavirus A
10941	GCA_002679995.1	Human rotavirus A
10941	GCA_002670775.1	Human rotavirus A
10941	GCA_002679655.1	Human rotavirus A
10941	GCA_002668735.1	Human rotavirus A
10941	GCA_002679315.1	Human rotavirus A
10941	GCA_002680635.1	Human rotavirus A
10941	GCA_002670795.1	Human rotavirus A
10941	GCA_002668755.1	Human rotavirus A
10941	GCA_002681055.1	Human rotavirus A

10941	GCA_002678295.1	Human rotavirus A
10941	GCA_002670815.1	Human rotavirus A
10941	GCA_002679555.1	Human rotavirus A
10941	GCA_002677955.1	Human rotavirus A
10941	GCA_002668775.1	Human rotavirus A
10941	GCA_002682215.1	Human rotavirus A
10941	GCA_002680715.1	Human rotavirus A
10941	GCA_002668795.1	Human rotavirus A
10941	GCA_002668815.1	Human rotavirus A
10941	GCA_002665895.1	Human rotavirus A
10941	GCA_002680695.1	Human rotavirus A
10941	GCA_002681635.1	Human rotavirus A
10941	GCA_002668835.1	Human rotavirus A
10941	GCA_002680015.1	Human rotavirus A
10941	GCA_002670895.1	Human rotavirus A
10941	GCA_002679675.1	Human rotavirus A
10941	GCA_002668855.1	Human rotavirus A
10941	GCA_002679335.1	Human rotavirus A
10941	GCA_002681195.1	Human rotavirus A
10941	GCA_002680895.1	Human rotavirus A
10941	GCA_002668875.1	Human rotavirus A
10941	GCA_002668635.1	Human rotavirus A
10941	GCA_002676855.1	Human rotavirus A
10941	GCA_002678315.1	Human rotavirus A
10941	GCA_002682195.1	Human rotavirus A
10941	GCA_002676875.1	Human rotavirus A
10941	GCA_002670955.1	Human rotavirus A
10941	GCA_002668915.1	Human rotavirus A
10941	GCA_002682015.1	Human rotavirus A
10941	GCA_002676895.1	Human rotavirus A
10941	GCA_002670975.1	Human rotavirus A
10941	GCA_002668935.1	Human rotavirus A
10941	GCA_002665915.1	Human rotavirus A
10941	GCA_002676915.1	Human rotavirus A
10941	GCA_002670995.1	Human rotavirus A
10941	GCA_002665575.1	Human rotavirus A
10941	GCA_002681395.1	Human rotavirus A
10941	GCA_002668955.1	Human rotavirus A
10941	GCA_002680175.1	Human rotavirus A
10941	GCA_002676935.1	Human rotavirus A
10941	GCA_002682355.1	Human rotavirus A
10941	GCA_002680035.1	Human rotavirus A
10941	GCA_002671015.1	Human rotavirus A
10941	GCA_002679695.1	Human rotavirus A
10941	GCA_002668975.1	Human rotavirus A
10941	GCA_002681335.1	Human rotavirus A
10941	GCA_002679355.1	Human rotavirus A
10941	GCA_002676955.1	Human rotavirus A
10941	GCA_002671035.1	Human rotavirus A
10941	GCA_002668995.1	Human rotavirus A

10941	GCA_002681155.1	Human rotavirus A
10941	GCA_002678335.1	Human rotavirus A
10941	GCA_002671055.1	Human rotavirus A
10941	GCA_002681535.1	Human rotavirus A
10941	GCA_002680995.1	Human rotavirus A
10941	GCA_002680855.1	Human rotavirus A
10941	GCA_002669015.1	Human rotavirus A
10941	GCA_002681415.1	Human rotavirus A
10941	GCA_002677655.1	Human rotavirus A
10941	GCA_002676995.1	Human rotavirus A
10941	GCA_002671075.1	Human rotavirus A
10941	GCA_002681295.1	Human rotavirus A
10941	GCA_002676975.1	Human rotavirus A
10941	GCA_002677015.1	Human rotavirus A
10941	GCA_002671095.1	Human rotavirus A
10941	GCA_002677035.1	Human rotavirus A
10941	GCA_002671115.1	Human rotavirus A
10941	GCA_002680315.1	Human rotavirus A
10941	GCA_002665595.1	Human rotavirus A
10941	GCA_002680055.1	Human rotavirus A
10941	GCA_002681475.1	Human rotavirus A
10941	GCA_002680275.1	Human rotavirus A
10941	GCA_002679375.1	Human rotavirus A
10941	GCA_002677075.1	Human rotavirus A
10941	GCA_002682315.1	Human rotavirus A
10941	GCA_002682635.1	Human rotavirus A
10941	GCA_002677095.1	Human rotavirus A
10941	GCA_002669135.1	Human rotavirus A
10941	GCA_002681115.1	Human rotavirus A
10941	GCA_002677115.1	Human rotavirus A
10941	GCA_002680795.1	Human rotavirus A
10941	GCA_002669155.1	Human rotavirus A
10941	GCA_002677135.1	Human rotavirus A
10941	GCA_002669175.1	Human rotavirus A
10941	GCA_002680455.1	Human rotavirus A
10941	GCA_002665955.1	Human rotavirus A
10941	GCA_002677155.1	Human rotavirus A
10941	GCA_002665615.1	Human rotavirus A
10941	GCA_002669195.1	Human rotavirus A
10941	GCA_002681615.1	Human rotavirus A
10941	GCA_002665275.1	Human rotavirus A
10941	GCA_002680075.1	Human rotavirus A
10941	GCA_002679735.1	Human rotavirus A
10941	GCA_002669215.1	Human rotavirus A
10941	GCA_002679395.1	Human rotavirus A
10941	GCA_002682575.1	Human rotavirus A
10941	GCA_002681035.1	Human rotavirus A
10941	GCA_002669235.1	Human rotavirus A
10941	GCA_002668355.1	Human rotavirus A
10941	GCA_002669255.1	Human rotavirus A

10941	GCA_002680935.1	Human rotavirus A
10941	GCA_002673335.1	Human rotavirus A
10941	GCA_002669275.1	Human rotavirus A
10941	GCA_002682095.1	Human rotavirus A
10941	GCA_002671335.1	Human rotavirus A
10941	GCA_002669295.1	Human rotavirus A
10941	GCA_002682235.1	Human rotavirus A
10941	GCA_002680775.1	Human rotavirus A
10941	GCA_002665215.1	Human rotavirus A
10941	GCA_002669315.1	Human rotavirus A
10941	GCA_002679575.1	Human rotavirus A
10941	GCA_002680095.1	Human rotavirus A
10941	GCA_002665235.1	Human rotavirus A
10941	GCA_002679755.1	Human rotavirus A
10941	GCA_002679415.1	Human rotavirus A
10941	GCA_002667295.1	Human rotavirus A
10941	GCA_002665255.1	Human rotavirus A
10941	GCA_002669355.1	Human rotavirus A
10941	GCA_002681075.1	Human rotavirus A
10941	GCA_002669375.1	Human rotavirus A
10941	GCA_002682495.1	Human rotavirus A
10941	GCA_002665295.1	Human rotavirus A
10941	GCA_002680735.1	Human rotavirus A
10941	GCA_002669395.1	Human rotavirus A
10941	GCA_002677375.1	Human rotavirus A
10941	GCA_002665315.1	Human rotavirus A
10941	GCA_002669415.1	Human rotavirus A
10941	GCA_002665335.1	Human rotavirus A
10941	GCA_002669435.1	Human rotavirus A
10941	GCA_002665355.1	Human rotavirus A
10941	GCA_002679775.1	Human rotavirus A
10941	GCA_002679435.1	Human rotavirus A
10941	GCA_002682555.1	Human rotavirus A
10941	GCA_002665375.1	Human rotavirus A
10941	GCA_002681215.1	Human rotavirus A
10941	GCA_002678075.1	Human rotavirus A
10941	GCA_002669495.1	Human rotavirus A
10941	GCA_002671555.1	Human rotavirus A
10941	GCA_002677395.1	Human rotavirus A
10941	GCA_002669515.1	Human rotavirus A
10941	GCA_002682035.1	Human rotavirus A
10941	GCA_002680535.1	Human rotavirus A
10941	GCA_002673555.1	Human rotavirus A
10941	GCA_002666015.1	Human rotavirus A
10941	GCA_002665455.1	Human rotavirus A
10941	GCA_002680195.1	Human rotavirus A
10941	GCA_002680135.1	Human rotavirus A
10941	GCA_002682395.1	Human rotavirus A
10941	GCA_002680395.1	Human rotavirus A
10941	GCA_002665475.1	Human rotavirus A

10941	GCA_002679795.1	Human rotavirus A
10941	GCA_002669575.1	Human rotavirus A
10941	GCA_002681355.1	Human rotavirus A
10941	GCA_002679455.1	Human rotavirus A
10941	GCA_002665495.1	Human rotavirus A
10941	GCA_002682515.1	Human rotavirus A
10941	GCA_002677575.1	Human rotavirus A
10941	GCA_002665515.1	Human rotavirus A
10941	GCA_002669615.1	Human rotavirus A
10941	GCA_002677755.1	Human rotavirus A
10941	GCA_002677595.1	Human rotavirus A
10941	GCA_002677415.1	Human rotavirus A
10941	GCA_002669635.1	Human rotavirus A
10941	GCA_002680675.1	Human rotavirus A
10941	GCA_002677615.1	Human rotavirus A
10941	GCA_002665555.1	Human rotavirus A
10941	GCA_002669655.1	Human rotavirus A
10941	GCA_002677635.1	Human rotavirus A
10941	GCA_002680335.1	Human rotavirus A
10941	GCA_002681555.1	Human rotavirus A
10941	GCA_002669675.1	Human rotavirus A
10941	GCA_002679815.1	Human rotavirus A
10941	GCA_002669695.1	Human rotavirus A
10941	GCA_002680655.1	Human rotavirus A
10941	GCA_002679475.1	Human rotavirus A
10941	GCA_002677675.1	Human rotavirus A
10941	GCA_002680355.1	Human rotavirus A
10941	GCA_002679135.1	Human rotavirus A
10941	GCA_002669715.1	Human rotavirus A
10941	GCA_002677695.1	Human rotavirus A
10941	GCA_002681375.1	Human rotavirus A
10941	GCA_002665635.1	Human rotavirus A
10941	GCA_002669735.1	Human rotavirus A
10941	GCA_002671795.1	Human rotavirus A
10941	GCA_002680815.1	Human rotavirus A
10941	GCA_002677435.1	Human rotavirus A
10941	GCA_002665655.1	Human rotavirus A
10941	GCA_002669755.1	Human rotavirus A
10941	GCA_002677735.1	Human rotavirus A
10941	GCA_002665675.1	Human rotavirus A
10941	GCA_002669775.1	Human rotavirus A
10941	GCA_002680475.1	Human rotavirus A
10941	GCA_002666055.1	Human rotavirus A
10941	GCA_002665715.1	Human rotavirus A
10941	GCA_002665695.1	Human rotavirus A
10941	GCA_002669795.1	Human rotavirus A
10941	GCA_002680515.1	Human rotavirus A
10941	GCA_002677775.1	Human rotavirus A
10941	GCA_002679835.1	Human rotavirus A
10941	GCA_002669815.1	Human rotavirus A

10941	GCA_002677795.1	Human rotavirus A
10941	GCA_002680915.1	Human rotavirus A
10941	GCA_002679155.1	Human rotavirus A
10941	GCA_002669835.1	Human rotavirus A
10941	GCA_002680615.1	Human rotavirus A
10941	GCA_002677815.1	Human rotavirus A
10941	GCA_002682455.1	Human rotavirus A
10941	GCA_002665755.1	Human rotavirus A
10941	GCA_002669855.1	Human rotavirus A
10941	GCA_002680955.1	Human rotavirus A
10941	GCA_002682135.1	Human rotavirus A
10941	GCA_002677455.1	Human rotavirus A
10941	GCA_002665775.1	Human rotavirus A
10941	GCA_002680255.1	Human rotavirus A
10941	GCA_002669875.1	Human rotavirus A
10941	GCA_002682115.1	Human rotavirus A
10941	GCA_002680115.1	Human rotavirus A
10941	GCA_002665795.1	Human rotavirus A
10941	GCA_002669895.1	Human rotavirus A
10941	GCA_002666075.1	Human rotavirus A
10941	GCA_002669915.1	Human rotavirus A
10941	GCA_002665395.1	Human rotavirus A
10941	GCA_002679855.1	Human rotavirus A
10941	GCA_002669935.1	Human rotavirus A
10941	GCA_002681435.1	Human rotavirus A
10941	GCA_002679515.1	Human rotavirus A
10941	GCA_002677915.1	Human rotavirus A
10941	GCA_002679175.1	Human rotavirus A
10941	GCA_002665855.1	Human rotavirus A
10941	GCA_002669955.1	Human rotavirus A
10941	GCA_002680875.1	Human rotavirus A
10941	GCA_002672015.1	Human rotavirus A
10941	GCA_002681935.1	Human rotavirus A
10941	GCA_002669975.1	Human rotavirus A
10941	GCA_002682055.1	Human rotavirus A
10941	GCA_002677475.1	Human rotavirus A
10941	GCA_002680755.1	Human rotavirus A
10941	GCA_002669995.1	Human rotavirus A
10941	GCA_002677975.1	Human rotavirus A
10941	GCA_002681015.1	Human rotavirus A
10941	GCA_002670015.1	Human rotavirus A
10941	GCA_002666095.1	Human rotavirus A
10941	GCA_002677995.1	Human rotavirus A
10941	GCA_002680415.1	Human rotavirus A
10941	GCA_002665935.1	Human rotavirus A
10941	GCA_002670035.1	Human rotavirus A
10941	GCA_002678015.1	Human rotavirus A
10941	GCA_002681575.1	Human rotavirus A
10941	GCA_002679875.1	Human rotavirus A
10941	GCA_002670055.1	Human rotavirus A

10941	GCA_002679535.1	Human rotavirus A
10941	GCA_002678035.1	Human rotavirus A
10941	GCA_002679195.1	Human rotavirus A
10941	GCA_002665975.1	Human rotavirus A
10941	GCA_002681235.1	Human rotavirus A
10941	GCA_002670075.1	Human rotavirus A
10941	GCA_002678055.1	Human rotavirus A
10941	GCA_002665995.1	Human rotavirus A
10941	GCA_002681135.1	Human rotavirus A
10941	GCA_002670095.1	Human rotavirus A
10941	GCA_002677835.1	Human rotavirus A
10941	GCA_002670115.1	Human rotavirus A
10941	GCA_002678095.1	Human rotavirus A
10941	GCA_002666035.1	Human rotavirus A
10941	GCA_002680555.1	Human rotavirus A
10941	GCA_002670135.1	Human rotavirus A
10941	GCA_002666115.1	Human rotavirus A
10941	GCA_002678115.1	Human rotavirus A
10941	GCA_002670155.1	Human rotavirus A
10941	GCA_002665435.1	Human rotavirus A
10941	GCA_002680215.1	Human rotavirus A
10941	GCA_002678135.1	Human rotavirus A
10941	GCA_002679895.1	Human rotavirus A
10941	GCA_002670175.1	Human rotavirus A
10941	GCA_002672235.1	Human rotavirus A
10941	GCA_002679215.1	Human rotavirus A
10941	GCA_002670195.1	Human rotavirus A
10941	GCA_002682295.1	Human rotavirus A
10941	GCA_002682535.1	Human rotavirus A
10941	GCA_002678175.1	Human rotavirus A
10941	GCA_002682655.1	Human rotavirus A
10941	GCA_002668175.1	Human rotavirus A
10941	GCA_002670215.1	Human rotavirus A
10941	GCA_002677855.1	Human rotavirus A
10941	GCA_002678195.1	Human rotavirus A
10941	GCA_003004985.1	Human rotavirus A
10941	GCA_003005665.1	Human rotavirus A
10941	GCA_003005005.1	Human rotavirus A
10941	GCA_003071085.1	Human rotavirus A
10941	GCA_003004995.1	Human rotavirus A
10941	GCA_003071185.1	Human rotavirus A
10941	GCA_003259025.1	Human rotavirus A
10942	GCA_000907835.1	Human rotavirus B
10943	GCA_000864225.1	Human rotavirus C
10943	GCA_003086935.1	Human rotavirus C
10943	GCA_003087195.1	Human rotavirus C
10943	GCA_003086955.1	Human rotavirus C
10943	GCA_003086995.1	Human rotavirus C
10943	GCA_003087015.1	Human rotavirus C
10943	GCA_003087035.1	Human rotavirus C

10943	GCA_003087555.1	Human rotavirus C
10943	GCA_003087215.1	Human rotavirus C
10943	GCA_003087095.1	Human rotavirus C
10943	GCA_003087115.1	Human rotavirus C
10943	GCA_003087175.1	Human rotavirus C
10943	GCA_003087235.1	Human rotavirus C
10943	GCA_003087255.1	Human rotavirus C
10943	GCA_003087595.1	Human rotavirus C
10943	GCA_003087615.1	Human rotavirus C
10943	GCA_003087535.1	Human rotavirus C
10943	GCA_003087635.1	Human rotavirus C
10943	GCA_003087575.1	Human rotavirus C
10943	GCA_003087375.1	Human rotavirus C
10943	GCA_003087135.1	Human rotavirus C
10943	GCA_003087155.1	Human rotavirus C
10943	GCA_003087515.1	Human rotavirus C
10943	GCA_003156105.1	Human rotavirus C
408599	GCA_002666175.1	Human rotavirus G9P[8]
408599	GCA_002666195.1	Human rotavirus G9P[8]
408599	GCA_002666215.1	Human rotavirus G9P[8]
408599	GCA_002666255.1	Human rotavirus G9P[8]
557247	GCA_002647515.1	Human rotavirus HCR3A
557245	GCA_002660815.1	Human rotavirus Ro1845
1979160	GCA_000863525.1	Human rubulavirus 2
1595998	GCA_000929235.1	Human smacovirus 1
1595998	GCA_003963915.1	Human smacovirus 1
1595998	GCA_003963935.1	Human smacovirus 1
1595998	GCA_003963955.1	Human smacovirus 1
1595998	GCA_003963975.1	Human smacovirus 1
1595998	GCA_003963995.1	Human smacovirus 1
1595998	GCA_003964015.1	Human smacovirus 1
1595998	GCA_003964035.1	Human smacovirus 1
1595998	GCA_003964055.1	Human smacovirus 1
1595998	GCA_003964075.1	Human smacovirus 1
1595998	GCA_003964095.1	Human smacovirus 1
1595998	GCA_003964115.1	Human smacovirus 1
1595998	GCA_003964135.1	Human smacovirus 1
1595998	GCA_003964155.1	Human smacovirus 1
1595998	GCA_003964175.1	Human smacovirus 1
1595998	GCA_003964195.1	Human smacovirus 1
1595998	GCA_003963835.1	Human smacovirus 1
1595998	GCA_003963875.1	Human smacovirus 1
1595998	GCA_003963895.1	Human smacovirus 1
743300	GCA_002819605.1	Human stool-associated circular virus NG13
11908	GCA_000863585.1	Human T-cell leukemia virus type I
11908	GCA_003104295.1	Human T-cell leukemia virus type I
11908	GCA_003102335.1	Human T-cell leukemia virus type I
36368	GCA_003109365.1	Human T-cell lymphotropic virus type 2b
11909	GCA_000847505.1	Human T-lymphotropic virus 2
11909	GCA_003098735.1	Human T-lymphotropic virus 2

11909	GCA_003098755.1	Human T-lymphotropic virus 2
11909	GCA_003109385.1	Human T-lymphotropic virus 2
11909	GCA_003104335.1	Human T-lymphotropic virus 2
11909	GCA_003104395.1	Human T-lymphotropic virus 2
11909	GCA_003098335.1	Human T-lymphotropic virus 2
318279	GCA_000882595.1	Human T-lymphotropic virus 4
511755	GCA_000875265.1	Human TMEV-like cardiovirus
269213	GCA_000856225.1	Humulus japonicus latent virus
1457386	GCA_001271075.1	Hunter Island virus
1930509	GCA_002374975.1	Husavirus sp.
421013	GCA_002937175.1	Hyacinth mosaic virus
375546	GCA_000886495.1	Hydrangea chlorotic mottle virus
112228	GCA_000858825.1	Hydrangea ringspot virus
1732176	GCA_001470575.1	Hydrogenobaculum phage 1
1367218	GCA_002825665.1	Hypericum japonicum associated circular DNA virus
762905	GCA_000889975.1	Hyperthermophilic Archaeal Virus 1
762906	GCA_000888415.1	Hyperthermophilic Archaeal Virus 2
28288	GCA_000864485.1	Hyphantria cunea nucleopolyhedrovirus
265522	GCA_000867965.1	Hyposoter fugitivus ichnovirus
1450749	GCA_003443495.1	IAS virus
10401	GCA_000839325.1	Ictalurid herpesvirus 1 (Channel catfish virus)
508441	GCA_002922465.1	Ictalurid herpesvirus 2
1536592	GCA_000927495.1	Idiomarinaceae phage 1N2-2
1527515	GCA_000929595.1	Idiomarinaceae phage Phi1M2-2
2058759	GCA_003729695.1	Idotea virus IWaV278
79899	GCA_002888815.1	Igbo Ora virus
1167696	GCA_000899275.1	Ikoma lyssavirus
59563	GCA_000870465.1	Ilheus virus
1445557	GCA_000922535.1	Ilomantsi virus
1758883	GCA_001461645.1	Imjin River virus 1
467989	GCA_002146225.1	Imjin virus
2170135	GCA_001654345.1	Impatiens flower break virus
11612	GCA_000852025.1	Impatiens necrotic spot virus
524023	GCA_000880775.1	Imperata yellow mottle virus
1382295	GCA_000915435.1	Inachis io cypovirus 2
31600	GCA_000846665.1	Indian cassava mosaic virus
341699	GCA_002986625.1	Indian cassava mosaic virus-[Ker2]
104664	GCA_000847765.1	Indian citrus ringspot virus
1755290	GCA_001939215.1	Indian encephalitis associated cyclovirus
1755290	GCA_001939215.2	Indian encephalitis associated cyclovirus
32629	GCA_000851105.1	Indian peanut clump virus
11120	GCA_000862965.1	Infectious bronchitis virus
10995	GCA_000855485.1	Infectious bursal disease virus (Gumboro virus)
10995	GCA_003971645.1	Infectious bursal disease virus (Gumboro virus)
12742	GCA_000852185.1	Infectious flacherie virus
11290	GCA_000850065.1	Infectious hematopoietic necrosis virus
11002	GCA_000856525.1	Infectious pancreatic necrosis virus
11002	GCA_003971665.1	Infectious pancreatic necrosis virus
11002	GCA_003971725.1	Infectious pancreatic necrosis virus
11002	GCA_003971685.1	Infectious pancreatic necrosis virus

11002	GCA_003971705.1	Infectious pancreatic necrosis virus
11002	GCA_003971745.1	Infectious pancreatic necrosis virus
180170	GCA_000848865.1	Infectious spleen and kidney necrosis virus
641809	GCA_001343785.1	Influenza A virus (A/California/07/2009(H1N1))
1645815	GCA_001014065.1	Influenza A virus (A/England/00380709/2010(H1N1))
1645794	GCA_001013685.1	Influenza A virus (A/England/00380710/2010(H1N1))
1645883	GCA_000986595.1	Influenza A virus (A/England/00380718/2010(H1N1))
1645871	GCA_000986575.1	Influenza A virus (A/England/00380719/2010(H1N1))
1645818	GCA_001014005.1	Influenza A virus (A/England/01180007/2010(H1N1))
1645893	GCA_900004765.1	Influenza A virus (A/England/01180011/2010(H1N1))
1645857	GCA_900004875.1	Influenza A virus (A/England/01180015/2010(H1N1))
1645866	GCA_000986155.1	Influenza A virus (A/England/01180016/2010(H1N1))
1645863	GCA_900007785.1	Influenza A virus (A/England/01180017/2010(H1N1))
1645816	GCA_001014225.1	Influenza A virus (A/England/01180018/2010(H1N1))
1645875	GCA_900004385.1	Influenza A virus (A/England/01220639/2009(H1N1))
1645864	GCA_000986235.1	Influenza A virus (A/England/01220736/2010(H1N1))
1645872	GCA_900004595.1	Influenza A virus (A/England/01220740/2010(H1N1))
1645852	GCA_900004375.1	Influenza A virus (A/England/01220741/2010(H1N1))
1645834	GCA_900004775.1	Influenza A virus (A/England/01580003/2010(H1N1))
1645847	GCA_900004675.1	Influenza A virus (A/England/01580005/2010(H1N1))
1645862	GCA_900004815.1	Influenza A virus (A/England/04860560/2010(H1N1))
1645817	GCA_001014105.1	Influenza A virus (A/England/04900053/2010(H1N1))
1645891	GCA_900004485.1	Influenza A virus (A/England/04980045/2010(H1N1))
1645824	GCA_001014085.1	Influenza A virus (A/England/04980053/2010(H1N1))
1645870	GCA_900004475.1	Influenza A virus (A/England/04980055/2010(H1N1))
1645894	GCA_900004935.1	Influenza A virus (A/England/04980653/2010(H1N1))
1230200	GCA_900004605.1	Influenza A virus (A/England/05000088/2010(H1N1))
1645795	GCA_001013645.1	Influenza A virus (A/England/05020662/2010(H1N1))
1645798	GCA_000986475.1	Influenza A virus (A/England/05020665/2010(H1N1))
1645814	GCA_001014205.1	Influenza A virus (A/England/05020673/2010(H1N1))
1645854	GCA_000986195.1	Influenza A virus (A/England/05040138/2010(H1N1))
1645833	GCA_900004435.1	Influenza A virus (A/England/05080637/2010(H1N1))
1645859	GCA_900004745.1	Influenza A virus (A/England/05080831/2010(H1N1))
1645793	GCA_001013825.1	Influenza A virus (A/England/05100297/2010(H1N1))
1645845	GCA_000986215.1	Influenza A virus (A/England/05100298/2010(H1N1))
1645886	GCA_900004515.1	Influenza A virus (A/England/05100301/2010(H1N1))
1645890	GCA_900004445.1	Influenza A virus (A/England/05100660/2010(H1N1))
1645851	GCA_000986115.1	Influenza A virus (A/England/05120003/2010(H1N1))
1645874	GCA_900019215.1	Influenza A virus (A/England/05120013/2010(H1N1))
1645839	GCA_900005905.1	Influenza A virus (A/England/05140176/2010(H1N1))
1645873	GCA_900004585.1	Influenza A virus (A/England/05140177/2010(H1N1))
1645876	GCA_900019225.1	Influenza A virus (A/England/05140216/2010(H1N1))
1645843	GCA_900004425.1	Influenza A virus (A/England/05140281/2010(H1N1))
1645835	GCA_900005915.1	Influenza A virus (A/England/05140283/2010(H1N1))
1645880	GCA_900004535.1	Influenza A virus (A/England/05140847/2010(H1N1))
1645882	GCA_900004455.1	Influenza A virus (A/England/05140856/2010(H1N1))
1645825	GCA_001006905.1	Influenza A virus (A/England/05160420/2010(H1N1))
1645830	GCA_001006725.1	Influenza A virus (A/England/10/2010(H1N1))
1645827	GCA_001014185.1	Influenza A virus (A/England/10740561/2010(H1N1))
1645776	GCA_001013665.1	Influenza A virus (A/England/10740571/2010(H1N1))

1645776	GCA_000986455.1	Influenza A virus (A/England/10740571/2010(H1N1))
1645801	GCA_000986535.1	Influenza A virus (A/England/10740579/2010(H1N1))
1645868	GCA_001291385.1	Influenza A virus (A/England/10740581/2010(H1N1))
1645858	GCA_900004575.1	Influenza A virus (A/England/10740623/2011(H1N1))
1645861	GCA_900004725.1	Influenza A virus (A/England/10740658/2011(H1N1))
1645780	GCA_001013765.1	Influenza A virus (A/England/10740664/2011(H1N1))
1645829	GCA_001039825.1	Influenza A virus (A/England/10740681/2010(H1N1))
1645841	GCA_001006745.1	Influenza A virus (A/England/10740685/2010(H1N1))
1645791	GCA_001013865.1	Influenza A virus (A/England/10740688/2010(H1N1))
1645889	GCA_900004495.1	Influenza A virus (A/England/10740690/2011(H1N1))
1645879	GCA_900007775.1	Influenza A virus (A/England/10740691/2011(H1N1))
1645867	GCA_900004945.1	Influenza A virus (A/England/10740693/2011(H1N1))
1645819	GCA_001006865.1	Influenza A virus (A/England/10740704/2011(H1N1))
1645799	GCA_000986175.1	Influenza A virus (A/England/10740707/2011(H1N1))
1645779	GCA_001013885.1	Influenza A virus (A/England/10820733/2010(H1N1))
1645820	GCA_001014145.1	Influenza A virus (A/England/10820734/2010(H1N1))
1645869	GCA_000986435.1	Influenza A virus (A/England/10820742/2010(H1N1))
1645805	GCA_001013725.1	Influenza A virus (A/England/11/2010(H1N1))
1645821	GCA_001014125.1	Influenza A virus (A/England/11520759/2011(H1N1))
1645777	GCA_000986075.1	Influenza A virus (A/England/12/2010(H1N1))
1645888	GCA_900016655.1	Influenza A virus (A/England/12240607/2010(H1N1))
1645828	GCA_001039805.1	Influenza A virus (A/England/12240612/2010(H1N1))
1645860	GCA_900004365.1	Influenza A virus (A/England/12240614/2011(H1N1))
1645811	GCA_001014045.1	Influenza A virus (A/England/132/2011(H1N1))
1645838	GCA_900004525.1	Influenza A virus (A/England/213/2011(H1N1))
1645806	GCA_001013785.1	Influenza A virus (A/England/21680633/2010(H1N1))
1645865	GCA_000986295.1	Influenza A virus (A/England/245/2010(H1N1))
1645788	GCA_000986095.1	Influenza A virus (A/England/251/2010(H1N1))
1645837	GCA_900006035.1	Influenza A virus (A/England/283/2010(H1N1))
1645837	GCA_900016665.1	Influenza A virus (A/England/283/2010(H1N1))
1645784	GCA_001013605.1	Influenza A virus (A/England/29/2010(H1N1))
1645796	GCA_000986555.1	Influenza A virus (A/England/32/2010(H1N1))
1645782	GCA_000986375.1	Influenza A virus (A/England/331/2010(H1N1))
1645846	GCA_001006825.1	Influenza A virus (A/England/343/2010(H1N1))
1645840	GCA_001291425.1	Influenza A virus (A/England/374/2010(H1N1))
1645813	GCA_001014265.1	Influenza A virus (A/England/377/2010(H1N1))
1645822	GCA_001014245.1	Influenza A virus (A/England/393/2010(H1N1))
1645850	GCA_900004505.1	Influenza A virus (A/England/414/2010(H1N1))
1645790	GCA_000986275.1	Influenza A virus (A/England/446/2010(H1N1))
1645786	GCA_001013625.1	Influenza A virus (A/England/468/2010(H1N1))
1645856	GCA_900004835.1	Influenza A virus (A/England/665/2010(H1N1))
1645800	GCA_000986495.1	Influenza A virus (A/England/671/2010(H1N1))
1645887	GCA_900004345.1	Influenza A virus (A/England/672/2010(H1N1))
1645810	GCA_000986615.1	Influenza A virus (A/England/673/2010(H1N1))
1645844	GCA_000986335.1	Influenza A virus (A/England/675/2010(H1N1))
1645802	GCA_000986255.1	Influenza A virus (A/England/676/2010(H1N1))
1645823	GCA_001006845.1	Influenza A virus (A/England/677/2010(H1N1))
1645878	GCA_900006335.1	Influenza A virus (A/England/686/2010(H1N1))
1645789	GCA_001013845.1	Influenza A virus (A/England/687/2010(H1N1))
1645778	GCA_001013965.1	Influenza A virus (A/England/688/2010(H1N1))

1645778	GCA_001013805.1	Influenza A virus (A/England/688/2010(H1N1))
1645884	GCA_900004555.1	Influenza A virus (A/England/689/2010(H1N1))
1645849	GCA_900005085.1	Influenza A virus (A/England/690/2010(H1N1))
1645812	GCA_001014025.1	Influenza A virus (A/England/692/2010(H1N1))
1645785	GCA_001013945.1	Influenza A virus (A/England/694/2010(H1N1))
1645807	GCA_000986135.1	Influenza A virus (A/England/697/2010(H1N1))
1645832	GCA_001039865.1	Influenza A virus (A/England/700/2010(H1N1))
1645803	GCA_000986355.1	Influenza A virus (A/England/701/2010(H1N1))
1645797	GCA_001013925.1	Influenza A virus (A/England/717/2010(H1N1))
1645881	GCA_900004335.1	Influenza A virus (A/England/718/2010(H1N1))
1645826	GCA_001006685.1	Influenza A virus (A/England/719/2010(H1N1))
1645787	GCA_000986055.1	Influenza A virus (A/England/8/2009(H1N1))
1645783	GCA_000986515.1	Influenza A virus (A/England/9/2009(H1N1))
1645842	GCA_900005115.1	Influenza A virus (A/England/WTSI1751/2010(H1N1))
1645781	GCA_001013745.1	Influenza A virus (A/England/WTSI1769/2010(H1N1))
1645848	GCA_001291405.1	Influenza A virus (A/England/WTSI1774/2010(H1N1))
1645792	GCA_000986395.1	Influenza A virus (A/England/WTSI1778/2009(H1N1))
1645853	GCA_001006885.1	Influenza A virus (A/England/WTSI1781/2011(H1N1))
1645855	GCA_900004465.1	Influenza A virus (A/England/WTSI1783/2010(H1N1))
1645804	GCA_001013705.1	Influenza A virus (A/England/WTSI1807/2010(H1N1))
1645808	GCA_000986415.1	Influenza A virus (A/England/WTSI1822/2011(H1N1))
1645809	GCA_001014165.1	Influenza A virus (A/England/WTSI1826/2010(H1N1))
1645877	GCA_000986315.1	Influenza A virus (A/England/WTSI1832/2010(H1N1))
1645885	GCA_001006925.1	Influenza A virus (A/England/WTSI2051/2011(H1N1))
1645831	GCA_001039845.1	Influenza A virus (A/England/WTSI2060/2010(H1N1))
1645836	GCA_900004805.1	Influenza A virus (A/England/WTSI2067/2010(H1N1))
1645892	GCA_900009845.1	Influenza A virus (A/England/WTSI2076/2010(H1N1))
93838	GCA_000864105.1	Influenza A virus (A/goose/Guangdong/1/1996(H5N1))
130760	GCA_000851145.1	Influenza A virus (A/Hong Kong/1073/99(H9N2))
488241	GCA_000866645.1	Influenza A virus (A/Korea/426/1968(H2N2))
335341	GCA_000865085.1	Influenza A virus (A/New York/392/2004(H3N2))
211044	GCA_000865725.1	Influenza A virus (A/Puerto Rico/8/1934(H1N1))
1332244	GCA_000928555.1	Influenza A virus (A/Shanghai/02/2013(H7N9))
518987	GCA_000820495.2	Influenza B virus (B/Lee/1940)
11553	GCA_000856665.2	Influenza C virus (C/Ann Arbor/1/50)
1542973	GCA_000989135.1	Influenza D virus (D/bovine/France/2986/2012)
1173138	GCA_002867775.1	Influenza D virus (D/swine/Oklahoma/1334/2011)
1620892	GCA_001431955.1	Inhangapi virus
2219103	GCA_003654245.1	Inoviridae sp.
2219103	GCA_003659365.1	Inoviridae sp.
2219103	GCA_003654325.1	Inoviridae sp.
2219103	GCA_003655965.1	Inoviridae sp.
2219103	GCA_003654345.1	Inoviridae sp.
2219103	GCA_003652365.1	Inoviridae sp.
2219103	GCA_003654545.1	Inoviridae sp.
2219103	GCA_003657025.1	Inoviridae sp.
2219103	GCA_003652505.1	Inoviridae sp.
2219103	GCA_003652525.1	Inoviridae sp.
2219103	GCA_003654625.1	Inoviridae sp.
2219103	GCA_003652585.1	Inoviridae sp.

2219103	GCA_003658405.1	Inoviridae sp.
2219103	GCA_003656725.1	Inoviridae sp.
2219103	GCA_003654745.1	Inoviridae sp.
2219103	GCA_003654765.1	Inoviridae sp.
2219103	GCA_003657405.1	Inoviridae sp.
2219103	GCA_003652745.1	Inoviridae sp.
2219103	GCA_003656465.1	Inoviridae sp.
2219103	GCA_003656385.1	Inoviridae sp.
2219103	GCA_003652765.1	Inoviridae sp.
2219103	GCA_003654885.1	Inoviridae sp.
2219103	GCA_003656965.1	Inoviridae sp.
2219103	GCA_003654945.1	Inoviridae sp.
2219103	GCA_003652905.1	Inoviridae sp.
2219103	GCA_003652945.1	Inoviridae sp.
2219103	GCA_003656745.1	Inoviridae sp.
2219103	GCA_003657125.1	Inoviridae sp.
2219103	GCA_003655085.1	Inoviridae sp.
2219103	GCA_003653045.1	Inoviridae sp.
2219103	GCA_003659505.1	Inoviridae sp.
2219103	GCA_003653065.1	Inoviridae sp.
2219103	GCA_003655125.1	Inoviridae sp.
2219103	GCA_003655165.1	Inoviridae sp.
2219103	GCA_003653205.1	Inoviridae sp.
2219103	GCA_003657305.1	Inoviridae sp.
2219103	GCA_003657485.1	Inoviridae sp.
2219103	GCA_003657145.1	Inoviridae sp.
2219103	GCA_003655285.1	Inoviridae sp.
2219103	GCA_003653285.1	Inoviridae sp.
2219103	GCA_003655385.1	Inoviridae sp.
2219103	GCA_003655405.1	Inoviridae sp.
2219103	GCA_003653425.1	Inoviridae sp.
2219103	GCA_003658545.1	Inoviridae sp.
2219103	GCA_003657525.1	Inoviridae sp.
2219103	GCA_003651485.1	Inoviridae sp.
2219103	GCA_003655765.1	Inoviridae sp.
2219103	GCA_003656205.1	Inoviridae sp.
2219103	GCA_003651705.1	Inoviridae sp.
2219103	GCA_003651805.1	Inoviridae sp.
2219103	GCA_003651865.1	Inoviridae sp.
2219103	GCA_003653945.1	Inoviridae sp.
2219103	GCA_003651925.1	Inoviridae sp.
2219103	GCA_003656045.1	Inoviridae sp.
2219103	GCA_003654025.1	Inoviridae sp.
2219103	GCA_003652005.1	Inoviridae sp.
2219103	GCA_003652025.1	Inoviridae sp.
2219103	GCA_003656125.1	Inoviridae sp.
2219103	GCA_003655925.1	Inoviridae sp.
2219103	GCA_003654105.1	Inoviridae sp.
2219103	GCA_003654145.1	Inoviridae sp.
2219103	GCA_003656785.1	Inoviridae sp.

2219103	GCA_003652125.1	Inoviridae sp.
2219103	GCA_003654185.1	Inoviridae sp.
2219103	GCA_003652185.1	Inoviridae sp.
1370065	GCA_003726615.1	Insectomime virus
345198	GCA_000916235.1	Invertebrate iridescent virus 22
345585	GCA_000915575.1	Invertebrate iridescent virus 30
176652	GCA_000838105.1	Invertebrate iridescent virus 6
1301279	GCA_000909775.1	Invertebrate iridovirus 22
1301280	GCA_000914535.1	Invertebrate iridovirus 25
551895	GCA_000874785.1	Iodobacteriophage phiPLPE
10567	GCA_000839345.1	Iotapapillomavirus 1
1740360	GCA_002830465.1	Ipomea begomovirus satellite 1
653358	GCA_000886615.1	Ipomoea yellow vein virus
55096	GCA_000866285.1	Ippy mammarenavirus
204478	GCA_000897875.1	Iranian johnsongrass mosaic virus
53193	GCA_000852825.1	Iresine viroid 1
1620893	GCA_002145625.1	Iriri virus
32620	GCA_001551305.1	Iris severe mosaic virus
60456	GCA_001611645.1	Iris yellow spot virus
60456	GCA_001611645.2	Iris yellow spot virus
249583	GCA_000905355.1	Irkut lyssavirus
1930960	GCA_001968735.1	Isaria javanica chrysovirus 1
290008	GCA_000906835.1	Isfahan virus
294365	GCA_000870485.1	Israeli acute paralysis virus
490111	GCA_002146205.1	Itacaiunas virus
1809767	GCA_001602065.1	Ixeridium yellow mottle virus 1
1817526	GCA_002116155.1	Ixeridium yellow mottle virus 2
1526525	GCA_003726895.1	Ixodes scapularis associated virus 1
1526526	GCA_003726915.1	Ixodes scapularis associated virus 2
11746	GCA_000850005.1	Jaagsiekte sheep retrovirus
1198450	GCA_002867555.1	Jacquemontia mosaic Yucatan virus
1490227	GCA_003029025.2	Jacquemontia yellow mosaic virus
1490227	GCA_003029025.1	Jacquemontia yellow mosaic virus
712037	GCA_000890615.1	Japanese eel endothelial cells-infecting virus
712037	GCA_003143475.1	Japanese eel endothelial cells-infecting virus
712037	GCA_003143495.1	Japanese eel endothelial cells-infecting virus
11072	GCA_000862145.1	Japanese encephalitis virus
659660	GCA_000885875.1	Japanese holly fern mottle virus
77344	GCA_000856845.1	Japanese iris necrotic ring virus
2170198	GCA_003032785.1	Japanese macaque simian foamy virus
2030954	GCA_002868635.1	Japanese soil-borne wheat mosaic virus
79917	GCA_000863265.1	Japanese yam mosaic virus
1853762	GCA_001736475.1	Jasmine virus C
1775963	GCA_001549545.1	Jasmine virus T
749390	GCA_002867535.1	Jatropha curcas mosaic virus-[Dha]
1027399	GCA_002822685.1	Jatropha curcas mosaic virus-[Jalgaon]
1547577	GCA_000930415.1	Jatropha leaf crumple virus
2169734	GCA_003029055.1	Jatropha leaf curl Gujarat virus
543876	GCA_000880535.1	Jatropha leaf curl virus
543876	GCA_002822705.1	Jatropha leaf curl virus

1530093	GCA_003028955.1	Jatropha leaf yellow mosaic Katarniaghat virus
569949	GCA_002822725.1	Jatropha mosaic India virus-[Lucknow]
1213406	GCA_000900435.1	Jatropha mosaic Nigeria virus
75574	GCA_000919175.1	Jatropha mosaic virus
1485012	GCA_000881575.1	Jatropha yellow mosaic virus
10632	GCA_000863805.1	JC polyomavirus
10632	GCA_900096525.1	JC polyomavirus
10632	GCA_900096515.1	JC polyomavirus
10632	GCA_900096655.1	JC polyomavirus
10632	GCA_900096665.1	JC polyomavirus
10632	GCA_900096535.1	JC polyomavirus
990280	GCA_002117615.1	Jeju virus
36370	GCA_003196735.1	Jembrana disease virus
1923350	GCA_001961255.1	Jingmen picorna-like virus
1491393	GCA_000919875.1	Jingmen tick virus
1923351	GCA_002008755.1	Jingmen tombus-like virus 1
1938656	GCA_002004055.1	Jingmen tombus-like virus 2
229149	GCA_000853605.1	Johnsongrass chlorotic stripe mosaic virus
31742	GCA_000861465.1	Johnsongrass mosaic virus
1272943	GCA_002145765.1	Joinjakaka virus
1664809	GCA_002831065.1	Jonchet virus
64309	GCA_002004595.1	Jugra virus
2020956	GCA_002270645.1	Jujube mosaic-associated virus
2093273	GCA_002937335.1	Juncus maritimus associated virus
2016401	GCA_003032645.1	Jungle carpet python virus
12524	GCA_000861805.1	Junonia coenia densovirus
1972568	GCA_000926635.1	Jurona vesiculovirus
1972568	GCA_002816015.1	Jurona vesiculovirus
64299	GCA_000955135.1	Jutiapa virus
322067	GCA_000865905.1	J-virus
1851087	GCA_002890375.1	Kabuto mountain virus
64310	GCA_002004935.1	Kadam virus
104580	GCA_000851685.1	Kadipiro virus
1795438	GCA_003972025.1	Kadiweu virus
1795438	GCA_003972025.2	Kadiweu virus
307164	GCA_002118865.1	Kaeng Khoi virus
1823757	GCA_001550425.1	Kafue kinda chacma baboon virus
80939	GCA_002831385.1	Kairi virus
1564120	GCA_004117475.1	Kaisodi virus
132477	GCA_000886555.1	Kalanchoe latent virus
218387	GCA_000842025.1	Kalanchoe top-spotting virus
1654582	GCA_002008635.1	Kallithea virus
1456752	GCA_000915395.1	Kama virus
200402	GCA_002118705.1	Kamese virus
218849	GCA_000851165.1	Kamiti River virus
2045186	GCA_004130295.1	Kampung Karu virus
2014931	GCA_003032715.1	Kanyawara virus
122291	GCA_000837785.1	Kappapapillomavirus 1
10623	GCA_000837085.1	Kappapapillomavirus 2
1332248	GCA_002816295.1	Karang Sari virus

64287	GCA_000858045.1	Karshi virus
1969397	GCA_002210735.1	Karumba virus
68876	GCA_000853385.1	Kashmir bee virus
1712570	GCA_002288735.2	Kasokero virus
1712570	GCA_002288735.1	Kasokero virus
1859492	GCA_002116175.1	Kaumoebavirus
64311	GCA_000884715.1	Kedougou virus
340921	GCA_000865265.1	Kelp fly virus
1273128	GCA_001020035.1	Kenaf leaf curl betasatellite
508750	GCA_000879195.1	Kenaf leaf curl virus-[India:Bahraich:2007]
765147	GCA_002146025.1	Kenkeme virus
12158	GCA_000849065.1	Kennedya yellow mosaic virus
380433	GCA_002118685.1	Kern Canyon virus
1712571	GCA_002117595.1	Keterrah virus
1080799	GCA_000895595.1	Keunjorong mosaic virus
380440	GCA_002146245.1	Keuraliba virus
1980475	GCA_002146125.1	Khabarovsk orthohantavirus
237716	GCA_000926575.1	Khujand lyssavirus
423446	GCA_000873085.1	KI polyomavirus Stockholm 60
1406344	GCA_002119005.1	Kibale virus
1980539	GCA_002118825.1	Kigluaik phantom orthophasmavirus
1654355	GCA_001019875.1	Kilifi Virus
318835	GCA_000927315.1	Kimberley virus
1911104	GCA_001866955.1	King virus
1673637	GCA_003726955.1	Kirkovirus Equ1
909206	GCA_002146165.1	Klamath virus
2555680	GCA_004521475.1	Klebsiella phage 020009
1960654	GCA_002619565.1	Klebsiella phage 1 LV-2017
1610829	GCA_001503435.1	Klebsiella phage 1513
2047786	GCA_003991645.1	Klebsiella phage 1611E-K2-1
1960655	GCA_002619585.1	Klebsiella phage 2 LV-2017
2024247	GCA_002628025.1	Klebsiella phage 2044-307w
1960656	GCA_002619605.1	Klebsiella phage 2b LV-2017
1960657	GCA_002619625.1	Klebsiella phage 3 LV-2017
1960658	GCA_002619645.1	Klebsiella phage 4 LV-2017
1955242	GCA_002619065.1	Klebsiella phage 48ST307
1960659	GCA_002619665.1	Klebsiella phage 5 LV-2017
1960660	GCA_002619685.1	Klebsiella phage 6 LV-2017
2026943	GCA_002629145.1	Klebsiella phage AltoGao
1416011	GCA_000916415.1	Klebsiella phage F19
2419608	GCA_004322875.1	Klebsiella phage GH-K3
2492437	GCA_004006775.1	Klebsiella phage Henu1
1236000	GCA_000905755.1	Klebsiella phage JD001
1698360	GCA_001470655.1	Klebsiella phage JD18
2099585	GCA_002997875.1	Klebsiella phage JY917
532077	GCA_000890635.1	Klebsiella phage K11
1647374	GCA_001500935.1	Klebsiella phage K5
1932361	GCA_002617845.1	Klebsiella phage K5-2
1932362	GCA_002617865.1	Klebsiella phage K5-4
1439894	GCA_001041755.1	Klebsiella phage K64-1

1647408	GCA_001500515.1	Klebsiella phage KLPN1
2282629	GCA_003958785.1	Klebsiella phage KN1-1
2282630	GCA_003958825.1	Klebsiella phage KN3-1
2282631	GCA_003958805.1	Klebsiella phage KN4-1
1871716	GCA_002709905.1	Klebsiella phage KNP2
1970798	GCA_002621285.1	Klebsiella phage KOX1
2070202	GCA_002958245.1	Klebsiella phage KP1
707757	GCA_000887175.1	Klebsiella phage KP15
2315700	GCA_003613135.1	Klebsiella phage KP179
1701805	GCA_001470195.1	Klebsiella phage Kp2
1129147	GCA_000904995.1	Klebsiella phage KP27
674082	GCA_000884535.1	Klebsiella phage KP32
674081	GCA_000886155.1	Klebsiella phage KP34
1129191	GCA_001550825.1	Klebsiella phage KP36
2099850	GCA_002997455.1	Klebsiella phage KP8
2500828	GCA_004015545.1	Klebsiella phage KpKT21phi1
2024238	GCA_002627945.1	Klebsiella phage KPN N137
2024239	GCA_002627965.1	Klebsiella phage KPN N141
2024240	GCA_002627985.1	Klebsiella phage KPN N54
2079340	GCA_002958525.1	Klebsiella phage KPN N98
2024241	GCA_002628005.1	Klebsiella phage KPN U2874
1481110	GCA_002604565.1	Klebsiella phage Kpn112
2034166	GCA_002629765.1	Klebsiella phage KPP5665-2
1904925	GCA_002614165.1	Klebsiella phage KP-Rio/2015
2488949	GCA_003865715.1	Klebsiella phage kpssk3
1913572	GCA_002615405.1	Klebsiella phage KPV15
1747282	GCA_001470515.1	Klebsiella phage KpV41
1852657	GCA_001745875.1	Klebsiella phage KpV475
1796998	GCA_001754845.1	Klebsiella phage KpV71
1913574	GCA_002615425.1	Klebsiella phage KPV811
1675607	GCA_002149185.1	Klebsiella phage Matisse
2054272	GCA_002957475.1	Klebsiella phage May
2054273	GCA_002957485.1	Klebsiella phage Menlow
2026950	GCA_002629185.1	Klebsiella phage MezzoGao
2234047	GCA_003308395.1	Klebsiella phage Mineola
1675608	GCA_002624505.1	Klebsiella phage Miro
2099390	GCA_003023915.1	Klebsiella phage myPSH1235
2301687	GCA_003443235.1	Klebsiella phage NJR15
2268610	GCA_003368825.1	Klebsiella phage NJS1
2301688	GCA_003443195.1	Klebsiella phage NJS2
2301689	GCA_003443215.1	Klebsiella phage NJS3
1194091	GCA_000925715.1	Klebsiella phage NTUH-K2044-K1-1
1555207	GCA_002605045.1	Klebsiella phage phiBO1E
255431	GCA_000846725.1	Klebsiella phage phiKO2
1897515	GCA_002709945.2	Klebsiella phage phiKpS2
1897515	GCA_002709945.1	Klebsiella phage phiKpS2
1654928	GCA_001743795.1	Klebsiella phage PKO111
1654927	GCA_001744475.1	Klebsiella phage PKP126
1880822	GCA_900095325.1	Klebsiella phage PMBT1
2419682	GCA_003719015.1	Klebsiella phage Pylas

2315475	GCA_003668255.1	Klebsiella phage Seifer
1958964	GCA_003203715.1	Klebsiella phage SH-Kp 152234
2066504	GCA_002957955.1	Klebsiella phage SH-Kp 152410
1965362	GCA_003203735.1	Klebsiella phage SH-Kp 160016
2026944	GCA_002629165.1	Klebsiella phage SopranoGao
2555909	GCA_004520055.1	Klebsiella phage ST101-KPC2phi6.1
2510474	GCA_004521755.1	Klebsiella phage ST101-KPC2phi6.2
2510476	GCA_004521835.1	Klebsiella phage ST11-OXA245phi3.1
2510453	GCA_004519915.1	Klebsiella phage ST11-OXA48phi15.1
2510454	GCA_004519935.1	Klebsiella phage ST11-OXA48phi15.3
2555911	GCA_004520095.1	Klebsiella phage ST11-VIM1phi8.2
2510478	GCA_004521735.1	Klebsiella phage ST11-VIM1phi8.3
2510479	GCA_004521715.1	Klebsiella phage ST11-VIM1phi8.4
2510464	GCA_004519835.1	Klebsiella phage ST13-OXA48phi12.1
2510465	GCA_004519815.1	Klebsiella phage ST13-OXA48phi12.2
2510466	GCA_004519795.1	Klebsiella phage ST13-OXA48phi12.3
2555912	GCA_004520075.1	Klebsiella phage ST147-VIM1phi7.2
2555913	GCA_004520015.1	Klebsiella phage ST15-VIM1phi2.1
2555914	GCA_004520035.1	Klebsiella phage ST16-OXA48phi5.2
2510482	GCA_004521795.1	Klebsiella phage ST16-OXA48phi5.3
2510483	GCA_004521775.1	Klebsiella phage ST16-OXA48phi5.4
2510456	GCA_004519955.1	Klebsiella phage ST258-KPC3phi16.1
2510457	GCA_004519975.1	Klebsiella phage ST258-KPC3phi16.2
2510468	GCA_004519875.1	Klebsiella phage ST340-VIM1phi10.1
2510469	GCA_004519855.1	Klebsiella phage ST340-VIM1phi10.2
2516434	GCA_004194195.1	Klebsiella phage ST405-OXA48phi1.1
2510484	GCA_004521875.1	Klebsiella phage ST405-OXA48phi1.2
2510485	GCA_004521855.1	Klebsiella phage ST405-OXA48phi1.3
2510486	GCA_004521815.1	Klebsiella phage ST437-OXA245phi4.1
2510470	GCA_004519775.1	Klebsiella phage ST512-KPC3phi13.2
2510458	GCA_004519895.1	Klebsiella phage ST512-KPC3phi13.6
2510488	GCA_004521695.1	Klebsiella phage ST846-OXA48phi9.1
2510489	GCA_004521675.1	Klebsiella phage ST846-OXA48phi9.2
2555917	GCA_004520115.1	Klebsiella phage ST899-OXA48phi17.1
2510459	GCA_004519995.1	Klebsiella phage ST899-OXA48phi17.2
2555918	GCA_004520135.1	Klebsiella phage ST974-OXA48phi18.2
2053603	GCA_002957275.1	Klebsiella phage Sugarland
1675609	GCA_001501095.1	Klebsiella phage Sushi
2301690	GCA_003443175.1	Klebsiella phage TAH8
2301532	GCA_003934415.1	Klebsiella phage TSK1
1701804	GCA_001470975.1	Klebsiella phage vB_Kp1
1701806	GCA_002607405.1	Klebsiella phage vB_Kp3
2070028	GCA_002958005.1	Klebsiella phage vB_Kpn_F48
1912318	GCA_002614485.1	Klebsiella phage vB_Kpn_IME260
1907784	GCA_002614285.1	Klebsiella phage vB_KpnM_BIS47
1719140	GCA_001470815.1	Klebsiella phage vB_KpnM_KB57
1852625	GCA_001745235.1	Klebsiella phage vB_KpnM_KpV477
1912321	GCA_002614545.1	Klebsiella phage vB_KpnM_KpV52
2041212	GCA_002743875.1	Klebsiella phage vB_KpnM_KpV79
1907782	GCA_002614245.1	Klebsiella phage vB_KpnP_BIS33

1907783	GCA_002614265.1	Klebsiella phage vB_KpnP_IL33
1770232	GCA_002608235.1	Klebsiella phage vB_KpnP_IME205
2268396	GCA_003342575.1	Klebsiella phage vB_KpnP_IME321
1671396	GCA_001500915.1	Klebsiella phage vB_KpnP_KpV289
1912319	GCA_002614505.1	Klebsiella phage vB_KpnP_KpV48
1933773	GCA_002618765.1	Klebsiella phage vB_KpnP_KpV74
1882400	GCA_002612025.1	Klebsiella phage vB_KpnP_KpV763
1897431	GCA_002612285.1	Klebsiella phage vB_KpnP_KpV766
1897430	GCA_002612265.1	Klebsiella phage vB_KpnP_KpV767
1907781	GCA_002614225.1	Klebsiella phage vB_KpnP_PRA33
1610834	GCA_001501315.1	Klebsiella phage vB_KpnP_SU503
1610835	GCA_001500655.1	Klebsiella phage vB_KpnP_SU552A
2041211	GCA_002743855.1	Klebsiella phage vB_KpnS_IME279
1912320	GCA_002614525.1	Klebsiella phage vB_KpnS_KpV522
2026101	GCA_002629105.1	Klebsiella phage YMC15/11/N53_KPN_BP
2026102	GCA_002629125.1	Klebsiella phage YMC16/01/N133_KPN_BP
2500166	GCA_004146645.1	Klebsiella phage YX3973
2201417	GCA_003308535.1	Klebsiella phage ZCKP1
2169687	GCA_000912655.1	Klebsiella virus 0507KN21
2060945	GCA_002957775.1	Klebsiella virus GML-KpCol1
1985726	GCA_003181215.1	Klebsiella virus KP32
1985726	GCA_003181235.1	Klebsiella virus KP32
1985726	GCA_003181255.1	Klebsiella virus KP32
1985726	GCA_003181275.1	Klebsiella virus KP32
2079262	GCA_002990235.1	Klebsiella virus vB_KpnM_KpS110
47049	GCA_000881715.1	Kluyvera phage Kvp1
394239	GCA_000908855.1	Koala retrovirus
394239	GCA_002888855.1	Koala retrovirus
1659220	GCA_001308755.1	Kobuvirus cattle/Kagoshima-1-22-KoV/2014/JPN
1659219	GCA_001308635.1	Kobuvirus cattle/Kagoshima-2-24-KoV/2015/JPN
44024	GCA_002365985.1	Kokobera virus
1428456	GCA_000925335.1	Kolente virus
322053	GCA_000867105.1	Konjac mosaic virus
1550518	GCA_001432135.1	Koolpinyah virus
35314	GCA_002831405.1	Koongol virus
318836	GCA_000895515.1	Kotonkan virus
1435294	GCA_003159115.1	Koyama Hill virus
390437	GCA_000870965.1	Kudzu mosaic virus
1911440	GCA_003727475.1	Kuiper virus
1537975	GCA_001431915.1	Kumasi rhabdovirus
1755590	GCA_002817165.1	Kunsagivirus A
2169965	GCA_002008715.1	Kunsagivirus B
33743	GCA_002820625.1	Kyasanur Forest disease virus
111970	GCA_000859605.1	Kyuri green mottle mosaic virus
11577	GCA_000850965.1	La Crosse virus
11577	GCA_002831285.1	La Crosse virus
1654357	GCA_001019775.1	La Jolla virus
1272946	GCA_002145565.1	La Joya virus
1173709	GCA_000906535.1	Labidocera aestiva circovirus
11048	GCA_000850185.1	Lactate dehydrogenase-elevating virus

51369	GCA_000848025.1	Lactobacillus phage A2
1225795	GCA_002602605.1	Lactobacillus phage ATCC 8014-B2
2079429	GCA_002958875.1	Lactobacillus phage Bacchae
2079429	GCA_002958875.2	Lactobacillus phage Bacchae
2079430	GCA_002958885.2	Lactobacillus phage Bassarid
2079430	GCA_002958885.1	Lactobacillus phage Bassarid
1932007	GCA_003723375.1	Lactobacillus phage BH1
2315485	GCA_003665805.1	Lactobacillus phage Bromius
1739607	GCA_001504875.1	Lactobacillus phage CL1
1739608	GCA_001503255.1	Lactobacillus phage CL2
2315484	GCA_003665785.1	Lactobacillus phage Dionysus
980881	GCA_900009555.1	Lactobacillus phage EV3
1739609	GCA_001504055.1	Lactobacillus phage iA2
2315483	GCA_003665765.1	Lactobacillus phage Iacchus
1739611	GCA_001504855.1	Lactobacillus phage iLp1308
1739610	GCA_001505515.1	Lactobacillus phage iLp84
1414736	GCA_000911275.1	Lactobacillus phage J-1
37105	GCA_000902335.1	Lactobacillus phage JCL1032
363555	GCA_000868065.1	Lactobacillus phage KC5a
2048517	GCA_003288535.1	Lactobacillus phage Lb
755164	GCA_001308395.1	Lactobacillus phage LBR48
146269	GCA_000866745.1	Lactobacillus phage Lc-Nu
1500733	GCA_000925735.1	Lactobacillus phage Ld17
1500734	GCA_000924895.1	Lactobacillus phage Ld25A
1500735	GCA_000927375.1	Lactobacillus phage Ld3
1552735	GCA_000955415.1	Lactobacillus phage Ldl1
2053682	GCA_002957325.1	Lactobacillus phage Lenus
947980	GCA_000900715.1	Lactobacillus phage LF1
1567484	GCA_001551085.1	Lactobacillus phage LfeInf
1567453	GCA_001551425.1	Lactobacillus phage LfeSau
2041454	GCA_002744095.1	Lactobacillus phage LJ
12348	GCA_000873305.1	Lactobacillus phage LL-H
2059850	GCA_003991685.1	Lactobacillus phage Lpa804
2041210	GCA_002743835.1	Lactobacillus phage LpeD
2419581	GCA_003691875.1	Lactobacillus phage LR1
2419582	GCA_003691895.1	Lactobacillus phage LR2
496874	GCA_000875585.1	Lactobacillus phage Lrm1
578234	GCA_000882635.1	Lactobacillus phage Lv-1
2079431	GCA_002990195.1	Lactobacillus phage Maenad
2079432	GCA_002958895.1	Lactobacillus phage Nyseid
1846168	GCA_002610005.1	Lactobacillus phage P1
2099651	GCA_002997445.1	Lactobacillus phage P1174
1928330	GCA_003290305.1	Lactobacillus phage P2
1416334	GCA_001507495.1	Lactobacillus phage phi jlb1
12417	GCA_000848805.1	Lactobacillus phage phiadh
1206110	GCA_000905715.1	Lactobacillus phage phiAQ113
279281	GCA_000846625.1	Lactobacillus phage phiAT3
52979	GCA_000841565.1	Lactobacillus phage phig1e
1399941	GCA_000913855.1	Lactobacillus phage phiJB
1399942	GCA_000912975.1	Lactobacillus phage phiLdb

438780	GCA_001308435.1	Lactobacillus phage phiPYB5
39103	GCA_000912315.1	Lactobacillus phage PL-1
1815511	GCA_001745555.1	Lactobacillus phage PLE2
1815510	GCA_001744895.1	Lactobacillus phage PLE3
2079298	GCA_003011835.1	Lactobacillus phage PM411
2315482	GCA_003665745.1	Lactobacillus phage Sabazios
1755697	GCA_002608095.1	Lactobacillus phage SA-C12
2070201	GCA_002958235.1	Lactobacillus phage Satyr
2079433	GCA_002958905.1	Lactobacillus phage Semele
2079433	GCA_002958905.2	Lactobacillus phage Semele
947981	GCA_000902375.1	Lactobacillus phage Sha1
2079434	GCA_002990225.2	Lactobacillus phage Silenus
2079434	GCA_002990225.1	Lactobacillus phage Silenus
2036055	GCA_003014395.1	Lactobacillus phage T25
139871	GCA_000871405.1	Lactobacillus prophage Lj771
139872	GCA_000843425.1	Lactobacillus prophage Lj928
139870	GCA_000842565.1	Lactobacillus prophage Lj965
1225794	GCA_000901235.1	Lactobacillus virus ATCC8014
508419	GCA_000901715.1	Lactobacillus virus c5
632112	GCA_000883715.1	Lactobacillus virus Lb338-1
297254	GCA_000913995.1	Lactobacillus virus LLKu
298338	GCA_000846045.1	Lactobacillus virus LP65
235201	GCA_000859685.1	Lactobacillus virus phiJL1
270182	GCA_002592885.1	Lactococcus lactis phage 645
213773	GCA_002592945.1	Lactococcus lactis phage P113G
213777	GCA_002592965.1	Lactococcus lactis phage p272
213774	GCA_002592985.1	Lactococcus lactis phage P475
2029658	GCA_003389515.1	Lactococcus phage 05601
2024334	GCA_003314955.1	Lactococcus phage 05802
1874585	GCA_002954815.1	Lactococcus phage 10W18
1874586	GCA_002954825.1	Lactococcus phage 10W22S
1874570	GCA_003861935.1	Lactococcus phage 10W24
1874571	GCA_003861955.1	Lactococcus phage 11W16L
741942	GCA_001015305.1	Lactococcus phage 1358
1874587	GCA_002954835.1	Lactococcus phage 13W11L
2029659	GCA_003389535.1	Lactococcus phage 16802
1874588	GCA_002954845.1	Lactococcus phage 16W12L
1874572	GCA_003862015.1	Lactococcus phage 16W23
475178	GCA_000879895.1	Lactococcus phage 1706
1874573	GCA_003861975.1	Lactococcus phage 17W11
1874574	GCA_003861995.1	Lactococcus phage 17W12M
1874594	GCA_002954905.1	Lactococcus phage 19W07F
1871678	GCA_001743875.1	Lactococcus phage 28201
1874575	GCA_003861875.1	Lactococcus phage 2R06A
1874576	GCA_003861795.1	Lactococcus phage 2R14S
1874577	GCA_003861815.1	Lactococcus phage 2R15M
1874578	GCA_003861835.1	Lactococcus phage 2R15S
1874579	GCA_003861855.1	Lactococcus phage 2R15S2
2029660	GCA_003389555.1	Lactococcus phage 30804
1262533	GCA_000910635.1	Lactococcus phage 340

2029661	GCA_003389575.1	Lactococcus phage 37201
2024335	GCA_003314875.1	Lactococcus phage 37203
1868846	GCA_002611245.1	Lactococcus phage 38502
2029662	GCA_003389595.1	Lactococcus phage 38503
2029663	GCA_003389615.1	Lactococcus phage 38507
1874580	GCA_003861775.1	Lactococcus phage 3R07S
1874589	GCA_002954855.1	Lactococcus phage 3R16S
201847	GCA_000843105.1	Lactococcus phage 4268
1868847	GCA_002611265.1	Lactococcus phage 49801
1874590	GCA_002954865.1	Lactococcus phage 4R15L
1874591	GCA_002954875.1	Lactococcus phage 4R16L2
1871681	GCA_001745195.1	Lactococcus phage 50101
2024336	GCA_003314935.1	Lactococcus phage 50102
2024337	GCA_003314915.1	Lactococcus phage 50504
1871683	GCA_002757795.1	Lactococcus phage 50901
1868848	GCA_002611285.1	Lactococcus phage 50902
2029664	GCA_003389475.1	Lactococcus phage 51701
1868849	GCA_002611305.1	Lactococcus phage 53801
1868850	GCA_002611325.1	Lactococcus phage 53802
2029665	GCA_003389635.1	Lactococcus phage 56003
2029666	GCA_003389655.1	Lactococcus phage 56301
1871685	GCA_002611665.1	Lactococcus phage 56701
2029667	GCA_003389675.1	Lactococcus phage 57001
1868851	GCA_002611345.1	Lactococcus phage 58502
2024338	GCA_003314835.1	Lactococcus phage 62402
2024339	GCA_003314855.1	Lactococcus phage 62403
1871686	GCA_002757815.1	Lactococcus phage 62501
1868852	GCA_002611365.1	Lactococcus phage 62502
1868853	GCA_002611385.1	Lactococcus phage 62503
2029668	GCA_003389695.1	Lactococcus phage 62601
2029669	GCA_003389715.1	Lactococcus phage 62605
2024340	GCA_003314815.1	Lactococcus phage 62606
1871687	GCA_001745855.1	Lactococcus phage 63301
2029670	GCA_003389735.1	Lactococcus phage 63302
2029671	GCA_003389755.1	Lactococcus phage 66901
1874581	GCA_003861915.1	Lactococcus phage 6W06
1874582	GCA_003862075.1	Lactococcus phage 6W18L
2024341	GCA_003314895.1	Lactococcus phage 74001
2029672	GCA_003389775.1	Lactococcus phage 79201
1871689	GCA_002611625.1	Lactococcus phage 86501
2029673	GCA_003389795.1	Lactococcus phage 88605
1874583	GCA_003861895.1	Lactococcus phage 8R06S
39838	GCA_002592905.1	Lactococcus phage 936
1636554	GCA_002593725.1	Lactococcus phage 936 group phage Phi.16
1636555	GCA_002593765.1	Lactococcus phage 936 group phage Phi10.5
1636556	GCA_002593445.1	Lactococcus phage 936 group phage Phi109
1636557	GCA_002593345.1	Lactococcus phage 936 group phage Phi114
1636558	GCA_002593285.1	Lactococcus phage 936 group phage Phi129
1636559	GCA_002593365.1	Lactococcus phage 936 group phage Phi13.16
1636560	GCA_002593625.1	Lactococcus phage 936 group phage Phi155

1636561	GCA_002593325.1	Lactococcus phage 936 group phage Phi17
1636562	GCA_002593125.1	Lactococcus phage 936 group phage Phi19
1636563	GCA_002593745.1	Lactococcus phage 936 group phage Phi19.2
1636564	GCA_002593165.1	Lactococcus phage 936 group phage Phi19.3
1636565	GCA_002593085.1	Lactococcus phage 936 group phage Phi4
1636566	GCA_002593485.1	Lactococcus phage 936 group phage Phi4.2
1636567	GCA_002593565.1	Lactococcus phage 936 group phage Phi40
1636568	GCA_002593265.1	Lactococcus phage 936 group phage Phi43
1636569	GCA_002593505.1	Lactococcus phage 936 group phage Phi44
1636570	GCA_002593225.1	Lactococcus phage 936 group phage Phi5.12
1636571	GCA_002593525.1	Lactococcus phage 936 group phage Phi91127
1636724	GCA_002593105.1	Lactococcus phage 936 group phage PhiA.16
1636572	GCA_002593185.1	Lactococcus phage 936 group phage PhiA1127
1636573	GCA_002593145.1	Lactococcus phage 936 group phage PhiB1127
1636574	GCA_002593245.1	Lactococcus phage 936 group phage PhiC0139
1636575	GCA_002593205.1	Lactococcus phage 936 group phage PhiD.18
1636576	GCA_002593645.1	Lactococcus phage 936 group phage PhiE1127
1636577	GCA_002593305.1	Lactococcus phage 936 group phage PhiF.17
1636578	GCA_002593405.1	Lactococcus phage 936 group phage PhiF0139
1636579	GCA_002593385.1	Lactococcus phage 936 group phage PhiG
1636580	GCA_002593605.1	Lactococcus phage 936 group phage PhiJF1
1636581	GCA_002593425.1	Lactococcus phage 936 group phage PhiL.18
1636582	GCA_002593465.1	Lactococcus phage 936 group phage PhiL.6
1636583	GCA_002593685.1	Lactococcus phage 936 group phage PhiLj
1636584	GCA_002593585.1	Lactococcus phage 936 group phage PhiM.16
1636585	GCA_002593545.1	Lactococcus phage 936 group phage PhiM.5
1636586	GCA_002593665.1	Lactococcus phage 936 group phage PhiM1127
1636587	GCA_002593705.1	Lactococcus phage 936 group phage PhiS0139
881953	GCA_000890755.1	Lactococcus phage 949
2029674	GCA_003389815.1	Lactococcus phage 96401
2029675	GCA_003389835.1	Lactococcus phage 96403
2029676	GCA_003389495.1	Lactococcus phage 96603
1868854	GCA_002611405.1	Lactococcus phage 98101
1868855	GCA_002611425.1	Lactococcus phage 98102
1868856	GCA_002611445.1	Lactococcus phage 98103
1868857	GCA_002611465.1	Lactococcus phage 98104
1871690	GCA_001744535.1	Lactococcus phage 98201
1868858	GCA_002611485.1	Lactococcus phage 98202
1868859	GCA_002611505.1	Lactococcus phage 98203
1868860	GCA_002611525.1	Lactococcus phage 98204
1965467	GCA_002620365.1	Lactococcus phage AM1
1965468	GCA_002620385.1	Lactococcus phage AM11
1965469	GCA_002620405.1	Lactococcus phage AM12
1965470	GCA_002620425.1	Lactococcus phage AM2
1965471	GCA_002620445.1	Lactococcus phage AM3
1965472	GCA_002620465.1	Lactococcus phage AM4
1965473	GCA_002620485.1	Lactococcus phage AM5
1965474	GCA_002620505.1	Lactococcus phage AM6
1965475	GCA_002620525.1	Lactococcus phage AM7
1965476	GCA_002620545.1	Lactococcus phage AM8

1965477	GCA_002620565.1	Lactococcus phage AM9
1165137	GCA_002592445.1	Lactococcus phage ASCC284
1165138	GCA_002592465.1	Lactococcus phage ASCC287
1165139	GCA_002592485.1	Lactococcus phage ASCC310
1165140	GCA_002592505.1	Lactococcus phage ASCC324
1165141	GCA_002592525.1	Lactococcus phage ASCC337
1165142	GCA_002592545.1	Lactococcus phage ASCC356
1165143	GCA_002592565.1	Lactococcus phage ASCC358
1165144	GCA_002592585.1	Lactococcus phage ASCC365
1165145	GCA_002592605.1	Lactococcus phage ASCC368
1165146	GCA_002592625.1	Lactococcus phage ASCC395
1165147	GCA_002592645.1	Lactococcus phage ASCC397
1165148	GCA_002592665.1	Lactococcus phage ASCC406
1165149	GCA_002592685.1	Lactococcus phage ASCC454
1165150	GCA_002592705.1	Lactococcus phage ASCC460
1165152	GCA_002592405.1	Lactococcus phage ASCC473
1165153	GCA_002592725.1	Lactococcus phage ASCC476
1165154	GCA_002592425.1	Lactococcus phage ASCC489
1165155	GCA_002592745.1	Lactococcus phage ASCC497
1165156	GCA_002592765.1	Lactococcus phage ASCC502
1165157	GCA_002592785.1	Lactococcus phage ASCC506
1165158	GCA_002592805.1	Lactococcus phage ASCC527
1165159	GCA_002592825.1	Lactococcus phage ASCC531
1165161	GCA_002592845.1	Lactococcus phage ASCC544
503388	GCA_000872725.1	Lactococcus phage asccphi28
151535	GCA_000838885.1	Lactococcus phage bIL285
151536	GCA_000845385.1	Lactococcus phage bIL286
151537	GCA_000842505.1	Lactococcus phage bIL309
151538	GCA_000838045.1	Lactococcus phage bIL310
151534	GCA_000839025.1	Lactococcus phage bIL311
151539	GCA_000837265.1	Lactococcus phage bIL312
31754	GCA_000859265.1	Lactococcus phage BK5-T
31754	GCA_002629885.1	Lactococcus phage BK5-T
1229751	GCA_000910615.1	Lactococcus phage BM13
1868861	GCA_002611545.1	Lactococcus phage C41431
1289467	GCA_002592865.1	Lactococcus phage CaseusJM1
2050980	GCA_002957225.1	Lactococcus phage CB14a
2050981	GCA_002957235.1	Lactococcus phage CB14b
1862958	GCA_001744035.1	Lactococcus phage D4410
1862959	GCA_001745355.1	Lactococcus phage D4412
1868862	GCA_002611565.1	Lactococcus phage Dub35A
213782	GCA_002592925.1	Lactococcus phage fd13
1698369	GCA_001550865.1	Lactococcus phage GE1
1874593	GCA_002954895.1	Lactococcus phage i0139
1262535	GCA_000909735.1	Lactococcus phage jm2
1262536	GCA_000911695.1	Lactococcus phage jm3
2027261	GCA_002955735.1	Lactococcus phage LP0004a
2027262	GCA_002955745.1	Lactococcus phage LP0004b
2027263	GCA_002955755.1	Lactococcus phage LP0004c
2027264	GCA_002955765.1	Lactococcus phage LP0004d

2027265	GCA_002955775.1	Lactococcus phage LP0109
2027266	GCA_002955785.1	Lactococcus phage LP0202
2027267	GCA_002955795.1	Lactococcus phage LP0209
2027268	GCA_002955805.1	Lactococcus phage LP0212
2027269	GCA_002955815.1	Lactococcus phage LP0304
2027270	GCA_002955825.1	Lactococcus phage LP0509
2027271	GCA_002955835.1	Lactococcus phage LP0604
2027272	GCA_002955845.1	Lactococcus phage LP0903
2027273	GCA_002955855.1	Lactococcus phage LP1005
2027274	GCA_002955865.1	Lactococcus phage LP1011
2027275	GCA_002955875.1	Lactococcus phage LP1110
2027276	GCA_002955885.1	Lactococcus phage LP1407
2027277	GCA_002955895.1	Lactococcus phage LP1502a
2027278	GCA_002955905.1	Lactococcus phage LP1502b
2027279	GCA_002955915.1	Lactococcus phage LP1502c
2026814	GCA_002955555.1	Lactococcus phage LP8511
2027244	GCA_002955565.1	Lactococcus phage LP9104
2027245	GCA_002955575.1	Lactococcus phage LP9205a
2027246	GCA_002955585.1	Lactococcus phage LP9205b
2027247	GCA_002955595.1	Lactococcus phage LP9206a
2027248	GCA_002955605.1	Lactococcus phage LP9206b
2027249	GCA_002955615.1	Lactococcus phage LP9206c
2027250	GCA_002955625.1	Lactococcus phage LP9207
2027251	GCA_002955635.1	Lactococcus phage LP9210
2027252	GCA_002955645.1	Lactococcus phage LP9404
2027253	GCA_002955655.1	Lactococcus phage LP9405a
2027254	GCA_002955665.1	Lactococcus phage LP9405b
2027255	GCA_002955675.1	Lactococcus phage LP9406
2027256	GCA_002955685.1	Lactococcus phage LP9609
2027257	GCA_002955695.1	Lactococcus phage LP9701
2027258	GCA_002955705.1	Lactococcus phage LP9801
2027259	GCA_002955715.1	Lactococcus phage LP9903
2027260	GCA_002955725.1	Lactococcus phage LP9908
1965478	GCA_002620585.1	Lactococcus phage LW31
1965479	GCA_002620605.1	Lactococcus phage LW32
1965480	GCA_002620625.1	Lactococcus phage LW33
1965481	GCA_002620645.1	Lactococcus phage LW4
1965482	GCA_002620665.1	Lactococcus phage LW81
1862960	GCA_001746015.1	Lactococcus phage M5938
1862961	GCA_001744695.1	Lactococcus phage M6162
1862962	GCA_001744015.1	Lactococcus phage M6165
1862963	GCA_002757735.1	Lactococcus phage M6202
1862964	GCA_002757755.1	Lactococcus phage M6653
1862965	GCA_002757775.1	Lactococcus phage M6654
2079278	GCA_003862055.1	Lactococcus phage MP1
1874592	GCA_002954885.1	Lactococcus phage MW18L
1874584	GCA_003862035.1	Lactococcus phage MW18S
1476886	GCA_000920955.1	Lactococcus phage P078
641487	GCA_000882895.1	Lactococcus phage P087
1476887	GCA_000920915.1	Lactococcus phage P092

1476888	GCA_000922855.1	Lactococcus phage P118
1476889	GCA_000923495.1	Lactococcus phage P162
201846	GCA_002758375.1	Lactococcus phage P335
1262537	GCA_000911715.1	Lactococcus phage P680
1527692	GCA_002593065.1	Lactococcus phage phi145
1527693	GCA_002593025.1	Lactococcus phage phi15
1262538	GCA_000908115.1	Lactococcus phage phi7
1527694	GCA_002593045.1	Lactococcus phage phi93
1412875	GCA_000916455.1	Lactococcus phage phiL47
12390	GCA_000843485.1	Lactococcus phage phiLC3
2488571	GCA_004355665.1	Lactococcus phage phiQ1
444474	GCA_002758615.1	Lactococcus phage phismq86
1815955	GCA_001744635.1	Lactococcus phage PLgT-1
1913145	GCA_002615265.1	Lactococcus phage PLg-TB25
1983536	GCA_003307335.1	Lactococcus phage PLgW-1
1983589	GCA_003307355.1	Lactococcus phage PLgY-16
1983590	GCA_003307375.1	Lactococcus phage PLgY-30
254253	GCA_002758415.1	Lactococcus phage Q33
382685	GCA_000870085.1	Lactococcus phage Q54
43685	GCA_000840345.1	Lactococcus phage r1t
1965483	GCA_002620685.1	Lactococcus phage R3.4
1965484	GCA_002620705.1	Lactococcus phage R31
1414741	GCA_002593005.1	Lactococcus phage SK1833
314021	GCA_002633565.1	Lactococcus phage TP712
35345	GCA_000838065.1	Lactococcus phage TP901-1
35241	GCA_000838985.1	Lactococcus phage Tuc2009
114416	GCA_000841645.1	Lactococcus phage ul36
374525	GCA_002758515.1	Lactococcus phage ul36.k1
374526	GCA_002758535.1	Lactococcus phage ul36.k1t1
374527	GCA_002758555.1	Lactococcus phage ul36.t1
374529	GCA_002758575.1	Lactococcus phage ul36.t1k1
2053703	GCA_003288575.1	Lactococcus phage vB_Llc_bIBB14s
2053704	GCA_003288595.1	Lactococcus phage vB_Llc_bIBB24tp1
2053705	GCA_003288555.1	Lactococcus phage vB_Llc_bIBB5g1
2053706	GCA_003288615.1	Lactococcus phage vB_Llc_bIBB77s
2053707	GCA_003288635.1	Lactococcus phage vB_Llc_bIBBEg1
2053708	GCA_003288655.1	Lactococcus phage vB_Llc_bIBBF12
2053709	GCA_003288675.1	Lactococcus phage vB_Llc_bIBBF13
2024011	GCA_003288495.1	Lactococcus phage vB_Llc_bIBBF14
1486423	GCA_000919955.1	Lactococcus phage WP-2
1560313	GCA_001041075.1	Lactococcus phage WRP3
213769	GCA_000868485.1	Lactococcus virus 712
1165134	GCA_001505235.1	Lactococcus virus ASCC191
1165135	GCA_002602185.1	Lactococcus virus ASCC273
1165136	GCA_002602205.1	Lactococcus virus ASCC281
1165151	GCA_002602225.1	Lactococcus virus ASCC465
1165160	GCA_002602245.1	Lactococcus virus ASCC532
494269	GCA_000879615.1	Lactococcus virus Bibb29
63118	GCA_000838765.1	Lactococcus virus bIL170
36343	GCA_000882295.1	Lactococcus virus bIL67

31537	GCA_000837665.1	Lactococcus virus c2
665883	GCA_001502115.1	Lactococcus virus CB13
665884	GCA_001501335.1	Lactococcus virus CB14
665886	GCA_002630585.1	Lactococcus virus CB19
665887	GCA_002630605.1	Lactococcus virus CB20
213775	GCA_000870105.1	Lactococcus virus jj50
388452	GCA_000873545.1	Lactococcus virus KSY1
83129	GCA_000868465.1	Lactococcus virus P008
254252	GCA_002629865.1	Lactococcus virus P2
31532	GCA_000837965.1	Lactococcus virus sk1
287412	GCA_001501375.1	Lactococcus virus Sl4
1468454	GCA_000918455.1	Lagenaria siceraria endornavirus-California
1965306	GCA_002116255.1	Lagenaria siceraria endornavirus-Hubei
2169774	GCA_001501395.1	Lagomorph bocaparvovirus 1
38766	GCA_000905975.1	Lagos bat lyssavirus
1633187	GCA_002826765.1	Laibin virus
2047876	GCA_002956535.1	Lake Baikal phage Baikal-20-5m-C28
1685724	GCA_001590055.1	Lake Sarah-associated circular molecule 1
1685725	GCA_001589735.1	Lake Sarah-associated circular molecule 10
1685726	GCA_001589415.1	Lake Sarah-associated circular molecule 11
1809112	GCA_001590395.1	Lake Sarah-associated circular molecule 12
1685727	GCA_001590335.1	Lake Sarah-associated circular molecule 2
1685728	GCA_001590035.1	Lake Sarah-associated circular molecule 3
1685729	GCA_001589715.1	Lake Sarah-associated circular molecule 4
1685730	GCA_001589395.1	Lake Sarah-associated circular molecule 5
1685731	GCA_001590315.1	Lake Sarah-associated circular molecule 6
1685732	GCA_001590015.1	Lake Sarah-associated circular molecule 7
1685733	GCA_001589695.1	Lake Sarah-associated circular molecule 8
1685734	GCA_001589375.1	Lake Sarah-associated circular molecule 9
1685735	GCA_001590495.1	Lake Sarah-associated circular virus-1
1685736	GCA_001590195.1	Lake Sarah-associated circular virus-10
1685737	GCA_001589875.1	Lake Sarah-associated circular virus-11
1685738	GCA_001589555.1	Lake Sarah-associated circular virus-12
1685739	GCA_001590475.1	Lake Sarah-associated circular virus-13
1685740	GCA_001590175.1	Lake Sarah-associated circular virus-14
1685741	GCA_001589855.1	Lake Sarah-associated circular virus-15
1685742	GCA_001589535.1	Lake Sarah-associated circular virus-16
1685743	GCA_001590455.1	Lake Sarah-associated circular virus-17
1685744	GCA_001590155.1	Lake Sarah-associated circular virus-18
1685745	GCA_001589835.1	Lake Sarah-associated circular virus-19
1685746	GCA_001589515.1	Lake Sarah-associated circular virus-2
1685747	GCA_001590435.1	Lake Sarah-associated circular virus-20
1685748	GCA_001590135.1	Lake Sarah-associated circular virus-21
1685749	GCA_001589815.1	Lake Sarah-associated circular virus-22
1685750	GCA_001590095.1	Lake Sarah-associated circular virus-23
1685751	GCA_001589775.1	Lake Sarah-associated circular virus-24
1685752	GCA_001589455.1	Lake Sarah-associated circular virus-25
1685753	GCA_001590375.1	Lake Sarah-associated circular virus-26
1685754	GCA_001590075.1	Lake Sarah-associated circular virus-27
1685755	GCA_001589755.1	Lake Sarah-associated circular virus-28

1685756	GCA_001589435.1	Lake Sarah-associated circular virus-29
1685757	GCA_001590355.1	Lake Sarah-associated circular virus-3
1685758	GCA_001586885.1	Lake Sarah-associated circular virus-30
1685759	GCA_001586905.1	Lake Sarah-associated circular virus-31
1685760	GCA_001589675.1	Lake Sarah-associated circular virus-32
1685761	GCA_001590595.1	Lake Sarah-associated circular virus-33
1685762	GCA_001590295.1	Lake Sarah-associated circular virus-34
1685763	GCA_001589975.1	Lake Sarah-associated circular virus-35
1685764	GCA_001589655.1	Lake Sarah-associated circular virus-36
1685765	GCA_001590575.1	Lake Sarah-associated circular virus-37
1685766	GCA_001590275.1	Lake Sarah-associated circular virus-38
1685767	GCA_001589955.1	Lake Sarah-associated circular virus-39
1685768	GCA_001589635.1	Lake Sarah-associated circular virus-4
1685769	GCA_001590555.1	Lake Sarah-associated circular virus-40
1685770	GCA_001590255.1	Lake Sarah-associated circular virus-41
1685771	GCA_001589935.1	Lake Sarah-associated circular virus-42
1685772	GCA_001589615.1	Lake Sarah-associated circular virus-43
1685773	GCA_001590535.1	Lake Sarah-associated circular virus-44
1685774	GCA_001590235.1	Lake Sarah-associated circular virus-45
1685775	GCA_001589915.1	Lake Sarah-associated circular virus-46
1685776	GCA_001589595.1	Lake Sarah-associated circular virus-47
1685777	GCA_001590515.1	Lake Sarah-associated circular virus-48
1685778	GCA_001590215.1	Lake Sarah-associated circular virus-49
1685779	GCA_001589895.1	Lake Sarah-associated circular virus-5
1685780	GCA_001589575.1	Lake Sarah-associated circular virus-50
1685781	GCA_001589495.1	Lake Sarah-associated circular virus-51
1685783	GCA_001590415.1	Lake Sarah-associated circular virus-6
1685784	GCA_001590115.1	Lake Sarah-associated circular virus-7
1685785	GCA_001589795.1	Lake Sarah-associated circular virus-8
1685786	GCA_001589475.1	Lake Sarah-associated circular virus-9
1547219	GCA_001925995.1	Lake Sinai virus
1041806	GCA_002270865.1	Lake Sinai virus 1
1041806	GCA_002830825.1	Lake Sinai virus 1
1041831	GCA_002271025.1	Lake Sinai virus 2
1041831	GCA_002830845.1	Lake Sinai virus 2
1983561	GCA_002210515.1	Lake Sinai Virus NE
1983562	GCA_002237195.1	Lake Sinai Virus SA1
1983563	GCA_002210875.1	Lake Sinai Virus SA2
1983564	GCA_002210715.1	Lake Sinai Virus TO
28326	GCA_002640835.1	Lamb rotavirus
28326	GCA_002661975.1	Lamb rotavirus
28326	GCA_002662195.1	Lamb rotavirus
35258	GCA_000840825.1	Lambdapapillomavirus 2
1642929	GCA_000982285.1	Lambdina fiscellaria nucleopolyhedrovirus
515320	GCA_000879355.1	Lamium leaf distortion virus
1432135	GCA_000916715.1	Lamium mild mosaic virus
649187	GCA_000926135.1	Lammi virus
1272947	GCA_002146145.1	Landjia virus
11085	GCA_000860805.1	Langat virus
1464786	GCA_000914575.1	Laodelphax striatella honeydew virus 1

1566869	GCA_000927935.1	Laodelphax striatellus picorna-like virus 2
49892	GCA_002664855.1	Lapine rotavirus
2018501	GCA_002355045.1	Lasius neglectus virus 1
11620	GCA_000851705.1	Lassa mammarenavirus
11620	GCA_900094045.1	Lassa mammarenavirus
45221	GCA_000875205.1	Latino mammarenavirus
999883	GCA_000893455.1	Lausannevirus
1241918	GCA_001429915.1	Le Blanc nodavirus
318848	GCA_002118505.1	Le Dantec virus
40057	GCA_002829425.1	Lebombo virus
2024248	GCA_002628045.1	Leclercia phage 10164-302
2024249	GCA_002628065.1	Leclercia phage 10164RH
45224	GCA_000855105.1	Leek white stripe virus
42004	GCA_000858985.1	Leek yellow stripe virus
1497019	GCA_000918215.1	Leishmania aethiopica RNA virus
58103	GCA_000848325.1	Leishmania RNA virus 1 - 1
12530	GCA_000852805.1	Leishmania RNA virus 1 - 4
39116	GCA_000847665.1	Leishmania RNA virus 2 - 1
1542498	GCA_000927475.1	Lelliottia phage phD2B
11049	GCA_003971765.1	Lelystad virus
2170115	GCA_000928535.1	Lemur associated porprismacovirus 1
2301528	GCA_003843605.1	Lentibacter virus vB_LenP_ICBM1
2301529	GCA_003843585.1	Lentibacter virus vB_LenP_ICBM2
2301530	GCA_003843565.1	Lentibacter virus vB_LenP_ICBM3
58177	GCA_002822745.1	Leonurus mosaic virus
1381104	GCA_000927995.1	Leopards Hill virus
481315	GCA_001579315.1	Leporid alphaherpesvirus 4
1856767	GCA_004128055.1	Leptomonas seymouri Narna-like virus 1
2077302	GCA_002937275.1	Leptonychotes weddellii papillomavirus 1
2077303	GCA_002937285.1	Leptonychotes weddellii papillomavirus 2
2077304	GCA_002937295.1	Leptonychotes weddellii papillomavirus 3
2077305	GCA_002937305.1	Leptonychotes weddellii papillomavirus 4
2077306	GCA_002937315.1	Leptonychotes weddellii papillomavirus 5
2077307	GCA_004132685.1	Leptonychotes weddellii papillomavirus 6
1925019	GCA_001907925.1	Leptonychotes weddellii polyomavirus 1
552509	GCA_002005725.1	Leptopilina boulardi filamentous virus
1353795	GCA_000926255.2	Leptopilina boulardi Toti-like virus
2163916	GCA_004132785.1	Leptosphaeria biglobosa mitovirus 1
2041382	GCA_003423065.1	Leptospira phage LE3
2041383	GCA_003423085.1	Leptospira phage LE4
1905713	GCA_002614185.1	Leptospira phage Lin_34
1334242	GCA_002603645.1	Leptospira phage vB_LalZ_80412-LE1
1334245	GCA_002603705.1	Leptospira phage vB_LbrZ_5399-LE1
1334246	GCA_002603725.1	Leptospira phage vB_LinZ_10-LE1
1334244	GCA_002603685.1	Leptospira phage vb_LkmZ_Bejolso9-LE1
1334243	GCA_002603665.1	Leptospira phage vB_LnoZ_CZ214-LE1
2219117	GCA_003847705.1	Lepus americanus faeces associated genomovirus SHP11
2219118	GCA_003847785.1	Lepus americanus faeces associated genomovirus SHP111
2219119	GCA_003847765.1	Lepus americanus faeces associated genomovirus SHP216
2219120	GCA_003847745.1	Lepus americanus faeces associated genomovirus SHP7

2219121	GCA_003847725.1	Lepus americanus faeces associated genomovirus SHP9
2219217	GCA_003848165.1	Lepus americanus faeces associated microvirus SHP1 6472
1603963	GCA_000931095.1	Lesavirus 1
1603964	GCA_000931295.1	Lesavirus 2
2010278	GCA_002210775.1	Leshenault partiti-like virus
2012640	GCA_004129915.1	Lesser panda anellovirus
1985698	GCA_000881815.1	Lettuce big-vein associated varicosavirus
642478	GCA_000885635.1	Lettuce chlorosis virus
2200955	GCA_004132245.1	Lettuce chordovirus 1
31713	GCA_000850585.1	Lettuce infectious yellows virus
1688637	GCA_001271135.1	Lettuce Italian necrotic virus
12202	GCA_000862305.1	Lettuce mosaic virus
1358807	GCA_002219685.1	Lettuce necrotic leaf curl virus
167947	GCA_002830605.1	Lettuce necrotic stunt virus
32612	GCA_000865305.1	Lettuce necrotic yellows virus
274495	GCA_000854545.1	Lettuce ring necrosis virus
447171	GCA_000879535.1	Lettuce virus X
471285	GCA_000882355.1	Lettuce yellow mottle virus
1307956	GCA_000870065.1	Leucania separata nucleopolyhedrovirus
670251	GCA_000885355.1	Leucas zeylanica yellow vein virus satellite DNA beta
745088	GCA_001310175.1	Leuconostoc phage 1-A4
1897738	GCA_002613705.1	Leuconostoc phage CHA
1897737	GCA_002613685.1	Leuconostoc phage CHB
440576	GCA_002630245.1	Leuconostoc phage L5
1897539	GCA_002613305.1	Leuconostoc phage LDG
1161930	GCA_000897415.1	Leuconostoc phage Lmd1
1897736	GCA_002613665.1	Leuconostoc phage Ln-7
1536604	GCA_001042215.1	Leuconostoc phage Ln-8
1536605	GCA_001041855.1	Leuconostoc phage Ln-9
1262522	GCA_000905595.1	Leuconostoc phage P793
1262515	GCA_000923735.1	Leuconostoc phage phiLN03
1262516	GCA_000906195.1	Leuconostoc phage phiLN04
1262517	GCA_000921195.1	Leuconostoc phage phiLN12
1262518	GCA_000922055.1	Leuconostoc phage phiLN25
1262519	GCA_000921175.1	Leuconostoc phage phiLN34
1262520	GCA_000923055.1	Leuconostoc phage phiLN6B
1262523	GCA_000922035.1	Leuconostoc phage phiLNTR2
1262521	GCA_000922015.1	Leuconostoc phage phiLNTR3
1927956	GCA_900149655.1	LeviOr01 phage
1965344	GCA_002080215.1	LI polyomavirus
246280	GCA_000866185.1	Liao ning virus
1072683	GCA_002630885.1	Liberibacter phage FP2
1903185	GCA_002614125.1	Liberibacter phage HHCA1-2
1965455	GCA_002620145.1	Liberibacter phage P-JXGC-3
941969	GCA_000901995.1	Liberibacter phage SC1
941970	GCA_000903515.1	Liberibacter phage SC2
1903184	GCA_002945115.1	Liberibacter phage SGCA5-1
478550	GCA_000874885.1	Ligustrum necrotic ringspot virus
1899566	GCA_001744835.1	Ligustrum virus A
722755	GCA_000925955.1	Lilac leaf chlorosis virus

32624	GCA_000866425.1	Lily mottle virus
12173	GCA_000854125.1	Lily symptomless virus
12194	GCA_000865605.1	Lily virus X
2136283	GCA_004131465.1	Lily yellow mosaic virus
2093276	GCA_002937345.1	Limeum africanum associated virus
2014960	GCA_002223795.1	Linda virus
300411	GCA_000873925.1	Lindernia anagallis yellow vein virus
447605	GCA_000870905.1	Lindernia anagallis yellow vein virus satellite DNA beta
2259792	GCA_004133845.1	Linepithema humile entomopoxvirus 1
1739631	GCA_003729675.1	Linepithema humile virus 1
1608057	GCA_003673665.1	Lishi spider virus 1
1608058	GCA_001755725.1	Lishi Spider Virus 2
1740165	GCA_001806215.1	Lisianthus enation leaf curl virus
334425	GCA_002830545.1	Lisianthus necrosis virus
334425	GCA_002988025.1	Lisianthus necrosis virus
330399	GCA_000871145.1	Listeria phage A006
40521	GCA_000849645.1	Listeria phage A118
40522	GCA_000873565.1	Listeria phage A500
330396	GCA_000871985.1	Listeria phage B025
330397	GCA_000874245.1	Listeria phage B054
1486422	GCA_000923575.1	Listeria phage List-36
1486421	GCA_000923595.1	Listeria phage LMSP-25
1486413	GCA_000924175.1	Listeria phage LMTA-148
1486397	GCA_002756135.1	Listeria phage LMTA-34
1486414	GCA_002756155.1	Listeria phage LMTA-57
1486419	GCA_002756175.1	Listeria phage LMTA-94
1173745	GCA_000921915.1	Listeria phage LP-026
1173743	GCA_000909375.1	Listeria phage LP-030-2
1458852	GCA_000921995.1	Listeria phage LP-030-3
1173746	GCA_003051905.1	Listeria phage LP-032
1173747	GCA_000910455.1	Listeria phage LP-037
1173764	GCA_000921135.1	Listeria phage LP-048
1458853	GCA_002604385.1	Listeria phage LP-064
1458854	GCA_003051925.1	Listeria phage LP-083-1
1458855	GCA_000923695.1	Listeria phage LP-083-2
1458856	GCA_000923075.1	Listeria phage LP-101
1173748	GCA_000907955.1	Listeria phage LP-110
1458857	GCA_000921155.1	Listeria phage LP-114
1173765	GCA_002604405.1	Listeria phage LP-124
1173766	GCA_000908915.1	Listeria phage LP-125
1633782	GCA_002605585.1	Listeria phage LWP01
330398	GCA_000873585.1	Listeria phage P35
560178	GCA_000882215.1	Listeria phage P40
1225800	GCA_000898775.1	Listeria phage P70
171618	GCA_000839905.1	Listeria phage PSA
2202246	GCA_003183645.1	Listeria phage PSU-VKH-LP019
2202247	GCA_003183665.1	Listeria phage PSU-VKH-LP040
2202248	GCA_003183685.1	Listeria phage PSU-VKH-LP041
1168744	GCA_000905575.1	Listeria phage vB_LmoM_AG20
1591072	GCA_001505735.1	Listeria phage vB_LmoS_188

1591073	GCA_001505075.1	Listeria phage vB_LmoS_293
1541821	GCA_000926795.1	Listeria phage WIL-1
40523	GCA_000871125.1	Listeria virus A511
330395	GCA_002629965.1	Listeria virus P100
310539	GCA_000859765.1	Listonella phage phiHSIC
217686	GCA_000862325.1	Little cherry virus 1
154339	GCA_000855865.1	Little cherry virus 2
1692253	GCA_001274165.1	Littorina sp. associated circular virus
1926511	GCA_001921595.1	Livupivirus A
874272	GCA_000923975.1	Lizard adenovirus 2
172315	GCA_000860945.1	Ljungan virus 87-012
1843771	GCA_001646575.1	Llama faeces associated circular DNA virus-1
1213198	GCA_001885485.1	Lleida bat lyssavirus
1513237	GCA_000896415.1	Lloviu cuevavirus
1911102	GCA_001866895.1	Lodeiro virus
1437126	GCA_002818825.1	Loei River mammarenavirus
754044	GCA_000906875.1	Loktanella phage pCB2051-A
365150	GCA_000874985.1	Lolium latent virus
2282644	GCA_004134185.1	Lolium perenne-associated virus
2169904	GCA_003033375.1	Lonchura maja polyomavirus 1
1219465	GCA_000908395.1	Lone Star virus
1844927	GCA_001651165.1	Lonestar tick chuvirus 1
1459044	GCA_000926995.1	Long Island tick rhabdovirus
1993907	GCA_002163385.1	Longan witches broom-associated virus
2050037	GCA_000905615.1	Long-fingered bat hepatitis B virus
2293294	GCA_003847025.1	Longjawed orbweaver circular virus 1
2293295	GCA_003846865.1	Longjawed orbweaver circular virus 2
1170422	GCA_002024775.1	Loreto virus
11086	GCA_000863165.1	Louping ill virus
1881951	GCA_002374355.1	Loveridges garter snake virus 1
12470	GCA_000861405.1	Lucerne transient streak virus
12470	GCA_002830865.1	Lucerne transient streak virus
193118	GCA_000844205.1	Lucerne transient streak virus satellite RNA
1508224	GCA_001962315.1	Lucheng Rn rat coronavirus
570832	GCA_000872785.1	Ludwigia leaf distortion betasatellite [India:Amadalavalasa
390444	GCA_002822765.1	Ludwigia yellow vein Vietnam virus
325111	GCA_000865765.1	Ludwigia yellow vein virus
325113	GCA_000865025.1	Ludwigia yellow vein virus-associated DNA beta
1462682	GCA_001271315.1	Luffa aphid-borne yellows virus
1705090	GCA_001430615.1	Luffa begomovirus betasatellite
1506157	GCA_000921035.1	Luffa puckering and leaf distortion-associated betasatellite
207240	GCA_000841445.1	Luffa yellow mosaic virus
649188	GCA_000885555.1	Lujo mammarenavirus
1678227	GCA_002831205.1	Lukuni virus
2050971	GCA_004132105.1	Lumpfish flavivirus
376849	GCA_000839805.1	Lumpy skin disease virus NI-2490
883876	GCA_000893975.1	Luna mammarenavirus
1134579	GCA_001343805.1	Lunk virus NKS-1
2017714	GCA_004130235.1	Lupine bocavirus
2017711	GCA_003848985.1	Lupine feces-associated gemycircularvirus 1

2017710	GCA_003849005.1	Lupine feces-associated gemycircularvirus 2
573615	GCA_000887935.1	Lupinus mosaic virus
1670669	GCA_001185005.1	Lutzomyia reovirus 1
2022430	GCA_002354945.1	Lychee viroid-like RNA
1676256	GCA_003159095.1	Lychnis mottle virus
1779714	GCA_003029225.1	Lycianthes yellow mosaic virus
1779714	GCA_003029225.2	Lycianthes yellow mosaic virus
1072333	GCA_002816465.1	Lygus lineolaris virus 1
165803	GCA_000850245.1	Lymantria dispar cypovirus 1
165429	GCA_000858525.1	Lymantria dispar cypovirus 14
1521188	GCA_000923955.1	Lymantria dispar iflavirus 1
10449	GCA_000846205.1	Lymantria dispar multiple nucleopolyhedrovirus
166921	GCA_000884695.1	Lymantria xylina nucleopolyhedrovirus
166921	GCA_002819125.1	Lymantria xylina nucleopolyhedrovirus
256729	GCA_000844885.1	Lymphocystis disease virus - isolate China
36363	GCA_000839605.1	Lymphocystis disease virus 1
1898060	GCA_001974475.1	Lymphocystis disease virus Sa
11623	GCA_000851025.1	Lymphocytic choriomeningitis mammarenavirus
2219140	GCA_003848325.1	Lynx canadensis associated microvirus CLP 9366
2219141	GCA_003848345.1	Lynx canadensis associated microvirus CLP 9413
2219122	GCA_003848025.1	Lynx canadensis faeces associated genomovirus CL1 128
2219123	GCA_003848005.1	Lynx canadensis faeces associated genomovirus CL1 148
2219124	GCA_003848125.1	Lynx canadensis faeces associated genomovirus CL1 46
2219125	GCA_003848105.1	Lynx canadensis faeces associated genomovirus CL1 48
2219126	GCA_003848085.1	Lynx canadensis faeces associated genomovirus CL1 58
2219127	GCA_003848065.1	Lynx canadensis faeces associated genomovirus CL1 71
2219128	GCA_003848045.1	Lynx canadensis faeces associated genomovirus CL1 74
2219129	GCA_003847985.1	Lynx canadensis faeces associated genomovirus CL3 128
2219130	GCA_003847945.1	Lynx canadensis faeces associated genomovirus CL4 128
2219131	GCA_003847965.1	Lynx canadensis faeces associated genomovirus CL4 71
2219132	GCA_003847925.1	Lynx canadensis faeces associated genomovirus CL5 48
323364	GCA_003177815.1	Lynx rufus papillomavirus type 1
2041389	GCA_002956025.1	Lysinibacillus phage vB_LspM-01
642022	GCA_000925615.1	Lyssavirus Ozernoe
1692254	GCA_001274505.1	Lytechinus variegatus variable sea urchin associated circula
1969400	GCA_002219825.1	Mac Peak virus
524653	GCA_003178255.1	Macaca fascicularis papillomavirus 10
656889	GCA_003178575.1	Macaca fascicularis papillomavirus 11
915424	GCA_000892835.1	Macaca fascicularis papillomavirus 2
471181	GCA_003178095.1	Macaca fascicularis papillomavirus 3
471182	GCA_003178135.1	Macaca fascicularis papillomavirus 4
471183	GCA_003178175.1	Macaca fascicularis papillomavirus 5
471184	GCA_003178115.1	Macaca fascicularis papillomavirus 6
471185	GCA_003178075.1	Macaca fascicularis papillomavirus 7
471186	GCA_003178155.1	Macaca fascicularis papillomavirus 8
524652	GCA_003178275.1	Macaca fascicularis papillomavirus 9
419544	GCA_003178035.1	Macaca fascicularis papillomavirus type 1
1236398	GCA_000903715.1	Macaca fascicularis polyomavirus 1
1816787	GCA_003179895.1	Macaca fuscata papillomavirus 1
2499223	GCA_003673205.1	Macaca mulatta feces associated virus 1

2499232	GCA_003673225.1	Macaca mulatta feces associated virus 10
2499224	GCA_003673285.1	Macaca mulatta feces associated virus 2
2499225	GCA_003673505.1	Macaca mulatta feces associated virus 3
2499226	GCA_003673445.1	Macaca mulatta feces associated virus 4
2499226	GCA_003673185.1	Macaca mulatta feces associated virus 4
2499227	GCA_003673365.1	Macaca mulatta feces associated virus 5
2499228	GCA_003673145.1	Macaca mulatta feces associated virus 6
2499229	GCA_003673385.1	Macaca mulatta feces associated virus 7
2499231	GCA_003673425.1	Macaca mulatta feces associated virus 9
2294150	GCA_004133185.1	Macaca mulatta papillomavirus 2
2294151	GCA_004133205.1	Macaca mulatta papillomavirus 3
2294152	GCA_004133225.1	Macaca mulatta papillomavirus 4
2364645	GCA_004134565.1	Macaca mulatta papillomavirus 5
2364646	GCA_004134585.1	Macaca mulatta papillomavirus 6
2364644	GCA_004134605.1	Macaca mulatta papillomavirus 7
1891767	GCA_000837645.1	Macaca mulatta polyomavirus 1
83534	GCA_000844645.1	Macaca mulatta rhadinovirus 17577 (Rhesus rhadinovirus)
1846169	GCA_001651185.1	Macaca nemestrina herpesvirus 7
10325	GCA_000844145.1	Macacine alphaherpesvirus 1 (monkey B virus)
45455	GCA_000846585.1	Macacine gammaherpesvirus 4 (Rhesus lymphocryptovirus
338478	GCA_000880115.1	Macaque simian foamy virus
2499233	GCA_003623315.1	Macaque stool associated virus 11
2499234	GCA_003621095.1	Macaque stool associated virus 12
11628	GCA_000853725.1	Machupo mammarenavirus
222557	GCA_000856985.1	Macrobrachium rosenbergii nodavirus
1027947	GCA_000898595.2	Macrobrachium rosenbergii Taihu virus
1527523	GCA_000929655.1	Macrophomina phaseolina tobamo-like virus
137443	GCA_001561005.1	Macropodid alphaherpesvirus 1
1904880	GCA_001777185.1	Macroptilium bright mosaic virus
1904881	GCA_001777205.1	Macroptilium common mosaic virus
475627	GCA_000879515.1	Macroptilium golden mosaic virus-[Jamaica:Wissadula:Aug
2021666	GCA_002378485.1	Macroptilium golden yellow mosaic virus
223282	GCA_000846245.1	Macroptilium mosaic Puerto Rico virus
223284	GCA_000844525.1	Macroptilium yellow mosaic Florida virus
169865	GCA_000848005.1	Macroptilium yellow mosaic virus
223285	GCA_002986635.1	Macroptilium yellow mosaic virus-[Cuba]
1129033	GCA_000894475.1	Macroptilium yellow net virus
1129034	GCA_000895435.1	Macroptilium yellow spot virus
1129035	GCA_000896915.1	Macroptilium yellow vein virus
1685953	GCA_001430495.1	Macrosiphum euphorbiae virus 1
1440170	GCA_000917835.1	Madariaga virus
1440170	GCA_002888955.1	Madariaga virus
1440170	GCA_002889075.1	Madariaga virus
348013	GCA_002118405.1	Madrid virus
764348	GCA_000887575.1	Magnaporthe oryzae chrysovirus 1
764348	GCA_000887575.2	Magnaporthe oryzae chrysovirus 1
764348	GCA_000915895.1	Magnaporthe oryzae chrysovirus 1
1580607	GCA_000930815.1	Magnaporthe oryzae RNA virus
271257	GCA_000856605.1	Magnaporthe oryzae virus 1
441999	GCA_000874825.1	Magnaporthe oryzae virus 2

1661396	GCA_001028965.1	Magnaporthe oryzae virus 3
1159903	GCA_000894435.1	Magpie-robin coronavirus HKU18
2170064	GCA_001630105.1	Mahlapitsi orthoreovirus
80938	GCA_002994695.1	Main Drain virus
1756285	GCA_001678215.1	Maize associated totivirus
51354	GCA_000861445.1	Maize chlorotic dwarf virus
12138	GCA_000856925.1	Maize chlorotic mottle virus
12203	GCA_000863225.1	Maize dwarf mosaic virus
209854	GCA_000859145.1	Maize fine streak nucleorhabdovirus
348823	GCA_000881755.1	Maize Iranian mosaic nucleorhabdovirus
348823	GCA_002831425.1	Maize Iranian mosaic nucleorhabdovirus
279896	GCA_000852725.1	Maize mosaic nucleorhabdovirus
137556	GCA_000865385.1	Maize necrotic streak virus
59749	GCA_000862485.1	Maize rayado fino virus
10989	GCA_001461385.2	Maize rough dwarf virus
10989	GCA_001461385.1	Maize rough dwarf virus
10989	GCA_003032535.1	Maize rough dwarf virus
1182518	GCA_000894615.1	Maize streak Reunion virus
10821	GCA_002825125.1	Maize streak virus
10821	GCA_002825145.1	Maize streak virus
10821	GCA_002825165.1	Maize streak virus
10821	GCA_002825065.1	Maize streak virus
10821	GCA_002825185.1	Maize streak virus
10821	GCA_002825205.1	Maize streak virus
10821	GCA_002825225.1	Maize streak virus
10821	GCA_002825245.1	Maize streak virus
268343	GCA_002825105.1	Maize streak virus - [Raw]
268344	GCA_002825085.1	Maize streak virus - [Set]
268316	GCA_000847105.2	Maize streak virus - A[Ama]
268328	GCA_003048235.1	Maize streak virus - A[South Africa]
2025388	GCA_004130985.1	Maize striate mosaic virus
445227	GCA_000873205.1	Maize white line mosaic virus
2170101	GCA_000909295.1	Maize yellow dwarf virus RMV
1833824	GCA_001634415.1	Maize yellow dwarf virus-RMV2
1856642	GCA_003029295.1	Maize yellow mosaic virus
1729670	GCA_001310215.1	Maize-associated totivirus 1
1874267	GCA_001678215.2	Maize-associated totivirus 2
2057198	GCA_002890095.1	Maize-associated totivirus 3
185954	GCA_000869705.1	Mal de Rio Cuarto virus
2202146	GCA_003029595.1	Malachra yellow mosaic virus
1705091	GCA_001430415.1	Malachra yellow vein mosaic betasatellite
488255	GCA_000879715.1	Malachra yellow vein mosaic virus-associated satellite DNA
38012	GCA_004130555.1	Malacosoma neustria nucleopolyhedrovirus
1229186	GCA_000926615.1	Malakal virus
1972570	GCA_000927135.1	Malpais Spring vesiculovirus
392174	GCA_000869265.1	Malva mosaic virus
1906668	GCA_001777305.1	Malvastrum bright yellow mosaic virus
339965	GCA_000864405.1	Malvastrum leaf curl betasatellite
1993643	GCA_000910735.1	Malvastrum leaf curl deltasatellite
1461792	GCA_000918115.1	Malvastrum leaf curl Guangdong betasatellite

377610	GCA_000869365.1	Malvastrum leaf curl Guangdong virus
1333313	GCA_000909895.1	Malvastrum leaf curl Philippines virus
329291	GCA_000866145.1	Malvastrum leaf curl virus - [G87]
329289	GCA_000866945.1	Malvastrum leaf curl virus-associated defective DNA beta
377461	GCA_000869545.1	Malvastrum yellow mosaic alphasatellite
377461	GCA_003034005.1	Malvastrum yellow mosaic alphasatellite
741733	GCA_000887975.1	Malvastrum yellow mosaic Cameroon alphasatellite
643133	GCA_002822785.1	Malvastrum yellow mosaic Helshire virus
376612	GCA_002867575.1	Malvastrum yellow mosaic Jamaica virus
377462	GCA_000867765.1	Malvastrum yellow mosaic virus
377463	GCA_000868625.1	Malvastrum yellow mosaic virus satellite DNA beta
642493	GCA_000883815.1	Malvastrum yellow vein Baoshan virus
642493	GCA_002986645.1	Malvastrum yellow vein Baoshan virus
221036	GCA_000861305.1	Malvastrum yellow vein betasatellite
1633139	GCA_000969115.1	Malvastrum yellow vein Cambodia virus
911574	GCA_000887735.1	Malvastrum yellow vein Changa Manga virus
1246849	GCA_001505475.1	Malvastrum yellow vein Chitwan betasatellite
676044	GCA_001706885.1	Malvastrum yellow vein Honghe virus
222476	GCA_000842105.1	Malvastrum yellow vein virus-[Y47]
290030	GCA_000857485.1	Malvastrum yellow vein Yunnan virus
377611	GCA_000845285.1	Malvastrum yellow vein Yunnan virus satellite DNA beta
1239565	GCA_001736695.1	Mamastrovirus 1
1239574	GCA_000853425.1	Mamastrovirus 10
1239577	GCA_000855165.1	Mamastrovirus 13
1239577	GCA_900155515.1	Mamastrovirus 13
1239577	GCA_900155505.1	Mamastrovirus 13
1239566	GCA_002194425.1	Mamastrovirus 2
1239567	GCA_000927095.1	Mamastrovirus 3
1239567	GCA_900197215.1	Mamastrovirus 3
1239567	GCA_900197255.1	Mamastrovirus 3
1239572	GCA_000884395.1	Mamastrovirus 8
78219	GCA_000917455.1	Mamestra brassicae multiple nucleopolyhedrovirus
207830	GCA_000837525.1	Mamestra configurata nucleopolyhedrovirus A
207830	GCA_002888915.1	Mamestra configurata nucleopolyhedrovirus A
204440	GCA_000857945.1	Mamestra configurata nucleopolyhedrovirus B
538123	GCA_000924315.1	Mammalian orthoreovirus 3
1979162	GCA_000854805.1	Mammalian rubulavirus 5
1272949	GCA_002145945.1	Manitoba virus
376821	GCA_002758595.1	Mannheimia phage phiMhaA1-BAA410
1572746	GCA_002605285.1	Mannheimia phage vB_MhM_1127AP1
1182515	GCA_002755195.1	Mannheimia phage vB_MhM_1152AP
1572748	GCA_002756515.1	Mannheimia phage vB_MhM_2256AP1
1572750	GCA_001505275.1	Mannheimia phage vB_MhM_3927AP2
1572740	GCA_002756495.1	Mannheimia phage vB_MhM_535AP1
1572744	GCA_001504475.1	Mannheimia phage vB_MhM_587AP1
1572747	GCA_001505935.1	Mannheimia phage vB_MhS_1152AP2
1572749	GCA_002605305.1	Mannheimia phage vB_MhS_3927AP1
1572743	GCA_001503675.1	Mannheimia phage vB_MhS_535AP2
1572745	GCA_001482975.1	Mannheimia phage vB_MhS_587AP2
376820	GCA_000867345.1	Mannheimia virus PHL101

238817	GCA_002145825.3	Maporal virus
238817	GCA_002145825.1	Maporal virus
238817	GCA_002145825.2	Maporal virus
1590836	GCA_000929375.1	Maprik virus
1979163	GCA_000873165.1	Mapuera rubulavirus
1046251	GCA_000925275.1	Maraba virus
368736	GCA_000868765.1	Maracuja mosaic virus
1662286	GCA_001645975.1	Marbled eel polyomavirus
33727	GCA_000857325.2	Marburg virus - Musoke, Kenya, 1980
1158190	GCA_002145745.1	Marco virus
1642436	GCA_001020075.1	Mariental mammarenavirus
1385658	GCA_000911395.1	Marine gokushovirus
1385658	GCA_002921725.1	Marine gokushovirus
1385658	GCA_002921735.1	Marine gokushovirus
1385658	GCA_002921705.1	Marine gokushovirus
1385658	GCA_002921715.1	Marine gokushovirus
439015	GCA_000874145.1	Marine RNA virus JP-A
439016	GCA_000873485.1	Marine RNA virus JP-B
1804157	GCA_001579415.1	Marine RNA virus PAL_E4
1804153	GCA_001576855.1	Marine RNA virus PAL128
1804154	GCA_001579455.1	Marine RNA virus PAL156
1804155	GCA_001579355.1	Marine RNA virus PAL438
1804156	GCA_001579475.1	Marine RNA virus PAL473
439014	GCA_000871885.1	Marine RNA virus SOG
1692255	GCA_001274145.1	Marine snail associated circular virus
1629954	GCA_002989835.1	Marinitoga camini virus 1
1629953	GCA_002989855.1	Marinitoga camini virus 2
2484222	GCA_003718795.1	Marinobacter phage AS1
2041341	GCA_002743915.1	Marinobacter phage PS3
2041342	GCA_002743935.1	Marinobacter phage PS6
2022859	GCA_002627265.1	Marinomonas phage CB5A
1965369	GCA_002619965.1	Marinomonas phage CPG1g
1965370	GCA_002619985.1	Marinomonas phage CPP1m
1176423	GCA_000899035.1	Marinomonas phage P12026
2163588	GCA_003094355.1	Marinomonas phage YY
292278	GCA_002118745.1	Marituba virus
2161807	GCA_004130475.1	Marmot norovirus
2161803	GCA_004130455.1	Marmot sapelovirus 1
694581	GCA_000887095.1	Marseillevirus marseillevirus
1307954	GCA_000870385.1	Maruca vitrata nucleopolyhedrovirus
11855	GCA_000848405.1	Mason-Pfizer monkey virus
2099663	GCA_003423445.1	Mastigocladus phage CHP58
392505	GCA_000869505.1	Mastomys coucha papillomavirus 2
1891768	GCA_000928995.1	Mastomys natalensis polyomavirus 1
1932931	GCA_000890715.1	Maverick-related virus strain Spezl
59301	GCA_000863385.1	Mayaro virus
1521385	GCA_000921375.1	McMurdo Ice Shelf pond-associated circular DNA virus-1
1521386	GCA_000923895.1	McMurdo Ice Shelf pond-associated circular DNA virus-2
1521387	GCA_000923255.1	McMurdo Ice Shelf pond-associated circular DNA virus-3
1521388	GCA_000922195.1	McMurdo Ice Shelf pond-associated circular DNA virus-4

1521389	GCA_000921355.1	McMurdo Ice Shelf pond-associated circular DNA virus-5
1521390	GCA_000923875.1	McMurdo Ice Shelf pond-associated circular DNA virus-6
1521391	GCA_000923235.1	McMurdo Ice Shelf pond-associated circular DNA virus-7
1521392	GCA_000922175.1	McMurdo Ice Shelf pond-associated circular DNA virus-8
35279	GCA_002004975.1	Meaban virus
11234	GCA_000854845.1	Measles morbillivirus
2043550	GCA_004117755.1	Medicago sativa alphapartitivirus 1
2069326	GCA_004128635.1	Medicago sativa amalgavirus 1
1756191	GCA_001611665.1	Megabat bufavirus 1
1094892	GCA_000893915.1	Megavirus chiliensis
1128140	GCA_002924555.1	Megavirus courdo11
1128135	GCA_002966315.1	Megavirus courdo7
1235314	GCA_000906615.1	Megavirus lba
1128143	GCA_000914855.1	Megavirus terra1
2109586	GCA_004133165.1	Megavirus vitis
2109586	GCA_004156275.1	Megavirus vitis
2170004	GCA_000920755.1	Megrivirus A
2483662	GCA_004150045.1	Meiothermus phage MMP17
2483850	GCA_003865575.1	Meiothermus phage MMP7
419697	GCA_000904195.1	Melaka orthoreovirus
545259	GCA_000884355.1	Melandrium yellow fleck virus
2083191	GCA_000838565.1	Melanoplus sanguinipes entomopoxvirus 'O'
1560514	GCA_000924835.1	Melbournevirus
37108	GCA_000839725.1	Meleagrid alphaherpesvirus 1 (Turkey herpesvirus)
1330070	GCA_000917935.1	Melegrivirus A
1128073	GCA_003985485.1	Meles meles circovirus-like virus
1128422	GCA_003850265.1	Meles meles fecal virus
1608323	GCA_000931155.1	Meles meles polyomavirus 1
1742594	GCA_001430075.1	Melochia mosaic virus
1742595	GCA_001430275.1	Melochia yellow mosaic virus
471717	GCA_000879435.1	Melon aphid-borne yellows virus
165826	GCA_000839245.1	Melon chlorotic leaf curl virus
223286	GCA_001430695.1	Melon chlorotic leaf curl virus-[Guatemala]
2169825	GCA_000890035.1	Melon chlorotic mosaic alphasatellite
180399	GCA_000887515.1	Melon chlorotic mosaic virus
2479459	GCA_004117735.1	Melon chlorotic spot virus
669377	GCA_002867185.1	Melon mild mottle virus
11987	GCA_000865645.1	Melon necrotic spot virus
2305257	GCA_004117375.1	Melon partitivirus
485724	GCA_002008855.1	Melon severe mosaic tospovirus
89471	GCA_000867545.1	Melon yellow spot virus
255255	GCA_002817615.1	Melon yellowing-associated virus
152219	GCA_000866025.1	Menangle virus
152219	GCA_002815295.1	Menangle virus
1963247	GCA_002029595.1	Menghai flavivirus
1919071	GCA_004129935.1	Menghai rhabdovirus
1286141	GCA_000908195.1	Meno virus
1708574	GCA_001293015.1	Mercadeo virus
1803034	GCA_004129635.1	Merida virus
1969830	GCA_004130215.1	Merida-like virus KE-2017a

743685	GCA_000920335.1	Merino Walk mammarenavirus
493803	GCA_000874865.1	Merkel cell polyomavirus
714309	GCA_000892695.1	Merremia mosaic Puerto Rico virus
77813	GCA_000841785.1	Merremia mosaic virus
77813	GCA_000867165.1	Merremia mosaic virus
1408129	GCA_000913695.1	Mesocricetus auratus papillomavirus 1
1891729	GCA_000844005.1	Mesocricetus auratus polyomavirus 1
1527771	GCA_000924995.1	Mesorhizobium phage vB_MloP_Lo5R7ANS
1705093	GCA_000899235.1	Mesta yellow vein mosaic alphasatellite
508749	GCA_000879475.1	Mesta yellow vein mosaic Bahraich virus-[India:Bahraich:2(
360579	GCA_000873045.1	Mesta yellow vein mosaic virus
562946	GCA_002822825.1	Mesta yellow vein mosaic virus [India:Amadalavalasa:2008
360580	GCA_000874365.1	Mesta yellow vein mosaic virus-associated DNA beta
2023155	GCA_002271085.1	Metallosphaera turreted icosahedral virus
2023155	GCA_002990085.1	Metallosphaera turreted icosahedral virus
77048	GCA_000837485.1	Methanobacterium phage psiM2
1430441	GCA_003571885.1	Methanobacterium virus Drs3
2035535	GCA_002990055.1	Methanosarcina spherical virus
173824	GCA_000840645.2	Methanothermobacter phage psiM100
1913040	GCA_002954925.1	Methylophilaceae phage P19250A
79450	GCA_000852865.1	Mexican papita viroid
2094142	GCA_003014225.1	Microbacterium phage AlexAdler
2507864	GCA_004139155.1	Microbacterium phage Alleb
2283287	GCA_003366995.1	Microbacterium phage Andromedas
2201432	GCA_003307635.1	Microbacterium phage AnnaSerena
2126922	GCA_003034745.1	Microbacterium phage Antoinette
2182350	GCA_003182885.1	Microbacterium phage Appa
2500786	GCA_004016045.1	Microbacterium phage ArMaWen
2419971	GCA_003691935.1	Microbacterium phage Armstrong
2079577	GCA_002959015.1	Microbacterium phage Aubergine
2079578	GCA_002959025.1	Microbacterium phage AxiPup
2079579	GCA_002959035.1	Microbacterium phage Baines
2079580	GCA_002959045.1	Microbacterium phage Balsa
2126923	GCA_003034635.1	Microbacterium phage Bandik
2126924	GCA_003034645.1	Microbacterium phage BeeBee8
2419972	GCA_003691975.1	Microbacterium phage Bernstein
2126925	GCA_003034655.1	Microbacterium phage BonaeVitae
2099644	GCA_002997485.1	Microbacterium phage Bonino
2419973	GCA_003692495.1	Microbacterium phage Brahms
2315703	GCA_003613435.1	Microbacterium phage Burro
2126926	GCA_003034665.1	Microbacterium phage BurtonThePup
2182351	GCA_003182905.1	Microbacterium phage Camille
2099442	GCA_002997805.1	Microbacterium phage Casey
2283288	GCA_003366955.1	Microbacterium phage ColaCorta
2419974	GCA_003692055.1	Microbacterium phage Coltrane
2182352	GCA_003182925.1	Microbacterium phage Count
2126927	GCA_003034675.1	Microbacterium phage Dave
2126928	GCA_003034755.1	Microbacterium phage Didgeridoo
2065199	GCA_002957915.1	Microbacterium phage Dismas
2315611	GCA_003668355.1	Microbacterium phage Dongwon

2250289	GCA_003365275.1	Microbacterium phage Eden
2315704	GCA_003613455.1	Microbacterium phage Efeko
2079581	GCA_002959055.1	Microbacterium phage Eleri
2126929	GCA_003034765.1	Microbacterium phage Elva
2079582	GCA_002959065.1	Microbacterium phage Espinosa
2126930	GCA_003034685.1	Microbacterium phage Etna
2530118	GCA_004339065.1	Microbacterium phage Fireman
2201433	GCA_003307755.1	Microbacterium phage Floof
2250349	GCA_003341395.1	Microbacterium phage Fork
2182353	GCA_003182945.1	Microbacterium phage Gargoyle
2094132	GCA_003014295.1	Microbacterium phage Gelo
2099624	GCA_002997555.1	Microbacterium phage Golden
2484206	GCA_003722615.1	Microbacterium phage Goodman
2079583	GCA_002959075.1	Microbacterium phage Hamlet
2182341	GCA_003183625.1	Microbacterium phage Hendrix
2201434	GCA_003307795.1	Microbacterium phage Hortus1
2182354	GCA_003182965.1	Microbacterium phage Hyperion
2079584	GCA_002959085.1	Microbacterium phage Ilzat
2250370	GCA_003341615.1	Microbacterium phage Jacko
2484207	GCA_003722655.1	Microbacterium phage Johann
2283289	GCA_003366875.1	Microbacterium phage KaiHaiDragon
2079585	GCA_002959095.1	Microbacterium phage Kale
2250350	GCA_003341215.1	Microbacterium phage KayPaulus
2126931	GCA_003034815.1	Microbacterium phage Kieran
2079586	GCA_002959105.1	Microbacterium phage Knox
2099625	GCA_002997575.1	Microbacterium phage Koji
2201435	GCA_003307815.1	Microbacterium phage Krampus
2099626	GCA_002997595.1	Microbacterium phage Lucky3
2079587	GCA_002959115.1	Microbacterium phage Ludgate
2250351	GCA_003341375.1	Microbacterium phage Lyell
2182355	GCA_003183005.1	Microbacterium phage Martin
2201436	GCA_003307855.1	Microbacterium phage MementoMori
2201437	GCA_003307875.1	Microbacterium phage Metamorphoo
2315534	GCA_003614355.1	Microbacterium phage Miaurora
446529	GCA_000874045.1	Microbacterium phage Min1
2301698	GCA_003442875.1	Microbacterium phage Minima
2250324	GCA_003365835.1	Microbacterium phage Musetta
2126932	GCA_003034695.1	Microbacterium phage Nagem
2099627	GCA_002997645.1	Microbacterium phage Nattles
2301539	GCA_003442115.1	Microbacterium phage Neferthena
2250371	GCA_003341695.1	Microbacterium phage Noelani
2182356	GCA_003183025.1	Microbacterium phage Oats
2201438	GCA_003307935.1	Microbacterium phage OlinDD
2301604	GCA_003601255.1	Microbacterium phage OneinaGillian
2099443	GCA_002997695.1	Microbacterium phage Pajaza
2126933	GCA_003034705.1	Microbacterium phage PaoPu
2250297	GCA_003365515.1	Microbacterium phage Papafritta
2182400	GCA_003183345.1	Microbacterium phage Paschalis
2079588	GCA_002959125.1	Microbacterium phage Peep
2079589	GCA_002959135.1	Microbacterium phage Peppino

2201439	GCA_003307955.1	Microbacterium phage Percival
2099444	GCA_002997715.1	Microbacterium phage Pikmin
2201440	GCA_003307995.1	Microbacterium phage Pioneer3
2099445	GCA_002997725.1	Microbacterium phage PuppyEggo
2250352	GCA_003341335.1	Microbacterium phage Quaker
2201441	GCA_003308215.1	Microbacterium phage Quhwah
2079590	GCA_002959145.1	Microbacterium phage Raccoon
2126934	GCA_003034715.1	Microbacterium phage Raptor
2250399	GCA_003365075.1	Microbacterium phage Redfield
2126935	GCA_003034725.1	Microbacterium phage Robinson
2201442	GCA_003308035.1	Microbacterium phage RobsFeet
2419975	GCA_003692415.1	Microbacterium phage Rollins
2250298	GCA_003365535.1	Microbacterium phage Sansa
2283249	GCA_003366335.1	Microbacterium phage Scamander
2283290	GCA_003366695.1	Microbacterium phage Schnapsidee
2500787	GCA_004015965.1	Microbacterium phage Schubert
2182357	GCA_003183145.1	Microbacterium phage Squash
2099446	GCA_002997775.1	Microbacterium phage StingRay
2079591	GCA_002959155.1	Microbacterium phage Superfresh
2201443	GCA_003308115.1	Microbacterium phage Tandem
2182358	GCA_003183105.1	Microbacterium phage Teagan
2126936	GCA_003034735.1	Microbacterium phage TeddyBear
2079592	GCA_002959175.1	Microbacterium phage Tenda
2530119	GCA_004338815.1	Microbacterium phage TimoTea
2126721	GCA_003034785.1	Microbacterium phage Triscuit
2315705	GCA_003614595.1	Microbacterium phage ValentiniPuff
1458670	GCA_000917955.1	Microbacterium phage vB_MoxS-ISF9
2250353	GCA_003341095.1	Microbacterium phage VitulaEligans
2201444	GCA_003308195.1	Microbacterium phage Zeta1847
1969741	GCA_004320245.1	Microcystis phage MACPNOA1
1357715	GCA_001505175.1	Microcystis phage MaMV-DC
2483660	GCA_003865555.1	Microcystis phage Me-ZS1
2483660	GCA_003865555.2	Microcystis phage Me-ZS1
340435	GCA_000870225.1	Microcystis virus Ma-LMM01
338781	GCA_000869105.1	Micromonas pusilla reovirus
755272	GCA_000906035.1	Micromonas pusilla virus 12T
880161	GCA_000890375.1	Micromonas sp. RCC1109 virus MpV1
10568	GCA_000867785.1	Micromys minutus papillomavirus 1
53988	GCA_000860105.1	Microplitis demolitor bracovirus
1655647	GCA_001190195.1	Microviridae Bog1249_12
1655648	GCA_001190335.1	Microviridae Bog5275_51
1655651	GCA_001190555.1	Microviridae Bog9017_22
1655652	GCA_001190675.1	Microviridae Fen2266_11
1655653	GCA_001190255.1	Microviridae Fen418_41
1655654	GCA_001190395.1	Microviridae Fen4707_41
1655655	GCA_002606725.1	Microviridae Fen51_42
1655657	GCA_001190655.1	Microviridae Fen685_11
1655658	GCA_001190235.1	Microviridae Fen7786_21
1655660	GCA_001190515.1	Microviridae Fen7895_21
1655661	GCA_001190635.1	Microviridae Fen7918_21

1655662	GCA_001190215.1	Microviridae Fen7940_21
1544364	GCA_000928195.1	Microviridae IME-16
913970	GCA_000892895.1	Microviridae phi-CA82
2202644	GCA_003372645.2	Microviridae sp.
2202644	GCA_003371705.1	Microviridae sp.
2202644	GCA_003381645.2	Microviridae sp.
2202644	GCA_003375805.1	Microviridae sp.
2202644	GCA_003375925.2	Microviridae sp.
2202644	GCA_003379905.1	Microviridae sp.
2202644	GCA_003374985.2	Microviridae sp.
2202644	GCA_003373765.1	Microviridae sp.
2202644	GCA_003379365.2	Microviridae sp.
2202644	GCA_003377865.1	Microviridae sp.
2202644	GCA_003372625.2	Microviridae sp.
2202644	GCA_003381605.1	Microviridae sp.
2202644	GCA_003371725.1	Microviridae sp.
2202644	GCA_003381625.2	Microviridae sp.
2202644	GCA_003373945.2	Microviridae sp.
2202644	GCA_003375825.1	Microviridae sp.
2202644	GCA_003375885.2	Microviridae sp.
2202644	GCA_003381265.1	Microviridae sp.
2202644	GCA_003379925.1	Microviridae sp.
2202644	GCA_003374965.2	Microviridae sp.
2202644	GCA_003373785.1	Microviridae sp.
2202644	GCA_003379325.2	Microviridae sp.
2202644	GCA_003380925.1	Microviridae sp.
2202644	GCA_003377885.1	Microviridae sp.
2202644	GCA_003375485.2	Microviridae sp.
2202644	GCA_003371745.1	Microviridae sp.
2202644	GCA_003381605.2	Microviridae sp.
2202644	GCA_003375845.1	Microviridae sp.
2202644	GCA_003375845.2	Microviridae sp.
2202644	GCA_003379945.1	Microviridae sp.
2202644	GCA_003374945.2	Microviridae sp.
2202644	GCA_003373805.1	Microviridae sp.
2202644	GCA_003379305.2	Microviridae sp.
2202644	GCA_003377905.1	Microviridae sp.
2202644	GCA_003371765.1	Microviridae sp.
2202644	GCA_003381585.2	Microviridae sp.
2202644	GCA_003375865.1	Microviridae sp.
2202644	GCA_003375825.2	Microviridae sp.
2202644	GCA_003379965.1	Microviridae sp.
2202644	GCA_003374925.2	Microviridae sp.
2202644	GCA_003373825.1	Microviridae sp.
2202644	GCA_003379245.2	Microviridae sp.
2202644	GCA_003377925.1	Microviridae sp.
2202644	GCA_003371785.1	Microviridae sp.
2202644	GCA_003381565.2	Microviridae sp.
2202644	GCA_003375885.1	Microviridae sp.
2202644	GCA_003375785.2	Microviridae sp.

2202644	GCA_003379985.1	Microviridae sp.
2202644	GCA_003374885.2	Microviridae sp.
2202644	GCA_003373845.1	Microviridae sp.
2202644	GCA_003379225.2	Microviridae sp.
2202644	GCA_003371445.2	Microviridae sp.
2202644	GCA_003377945.1	Microviridae sp.
2202644	GCA_003372545.2	Microviridae sp.
2202644	GCA_003371805.1	Microviridae sp.
2202644	GCA_003381545.2	Microviridae sp.
2202644	GCA_003371825.2	Microviridae sp.
2202644	GCA_003375905.1	Microviridae sp.
2202644	GCA_003375765.2	Microviridae sp.
2202644	GCA_003372745.1	Microviridae sp.
2202644	GCA_003380005.1	Microviridae sp.
2202644	GCA_003374865.2	Microviridae sp.
2202644	GCA_003373865.1	Microviridae sp.
2202644	GCA_003379185.2	Microviridae sp.
2202644	GCA_003372405.1	Microviridae sp.
2202644	GCA_003377965.1	Microviridae sp.
2202644	GCA_003371825.1	Microviridae sp.
2202644	GCA_003381525.2	Microviridae sp.
2202644	GCA_003375925.1	Microviridae sp.
2202644	GCA_003375745.2	Microviridae sp.
2202644	GCA_003380025.1	Microviridae sp.
2202644	GCA_003374845.2	Microviridae sp.
2202644	GCA_003373885.1	Microviridae sp.
2202644	GCA_003379165.2	Microviridae sp.
2202644	GCA_003377985.1	Microviridae sp.
2202644	GCA_003381625.1	Microviridae sp.
2202644	GCA_003371845.1	Microviridae sp.
2202644	GCA_003381505.2	Microviridae sp.
2202644	GCA_003373925.2	Microviridae sp.
2202644	GCA_003375945.1	Microviridae sp.
2202644	GCA_003375725.2	Microviridae sp.
2202644	GCA_003381285.1	Microviridae sp.
2202644	GCA_003380045.1	Microviridae sp.
2202644	GCA_003374825.2	Microviridae sp.
2202644	GCA_003373905.1	Microviridae sp.
2202644	GCA_003379145.2	Microviridae sp.
2202644	GCA_003380945.1	Microviridae sp.
2202644	GCA_003378005.1	Microviridae sp.
2202644	GCA_003375465.2	Microviridae sp.
2202644	GCA_003371865.1	Microviridae sp.
2202644	GCA_003381485.2	Microviridae sp.
2202644	GCA_003375965.1	Microviridae sp.
2202644	GCA_003375705.2	Microviridae sp.
2202644	GCA_003380065.1	Microviridae sp.
2202644	GCA_003374805.2	Microviridae sp.
2202644	GCA_003373925.1	Microviridae sp.
2202644	GCA_003379125.2	Microviridae sp.

2202644	GCA_003378025.1	Microviridae sp.
2202644	GCA_003371885.1	Microviridae sp.
2202644	GCA_003381465.2	Microviridae sp.
2202644	GCA_003375985.1	Microviridae sp.
2202644	GCA_003375685.2	Microviridae sp.
2202644	GCA_003380085.1	Microviridae sp.
2202644	GCA_003374785.2	Microviridae sp.
2202644	GCA_003373945.1	Microviridae sp.
2202644	GCA_003379105.2	Microviridae sp.
2202644	GCA_003378045.1	Microviridae sp.
2202644	GCA_003371905.1	Microviridae sp.
2202644	GCA_003381445.2	Microviridae sp.
2202644	GCA_003376005.1	Microviridae sp.
2202644	GCA_003375645.2	Microviridae sp.
2202644	GCA_003380105.1	Microviridae sp.
2202644	GCA_003374765.2	Microviridae sp.
2202644	GCA_003373965.1	Microviridae sp.
2202644	GCA_003379085.2	Microviridae sp.
2202644	GCA_003371425.2	Microviridae sp.
2202644	GCA_003378065.1	Microviridae sp.
2202644	GCA_003371925.1	Microviridae sp.
2202644	GCA_003381425.2	Microviridae sp.
2202644	GCA_003376025.1	Microviridae sp.
2202644	GCA_003375625.2	Microviridae sp.
2202644	GCA_003372765.1	Microviridae sp.
2202644	GCA_003380125.1	Microviridae sp.
2202644	GCA_003374745.2	Microviridae sp.
2202644	GCA_003373985.1	Microviridae sp.
2202644	GCA_003379065.2	Microviridae sp.
2202644	GCA_003378085.1	Microviridae sp.
2202644	GCA_003372385.2	Microviridae sp.
2202644	GCA_003372605.2	Microviridae sp.
2202644	GCA_003371945.1	Microviridae sp.
2202644	GCA_003381405.2	Microviridae sp.
2202644	GCA_003376045.1	Microviridae sp.
2202644	GCA_003375605.2	Microviridae sp.
2202644	GCA_003380145.1	Microviridae sp.
2202644	GCA_003374705.2	Microviridae sp.
2202644	GCA_003374005.1	Microviridae sp.
2202644	GCA_003379045.2	Microviridae sp.
2202644	GCA_003378105.1	Microviridae sp.
2202644	GCA_003381645.1	Microviridae sp.
2202644	GCA_003371965.1	Microviridae sp.
2202644	GCA_003381385.2	Microviridae sp.
2202644	GCA_003373905.2	Microviridae sp.
2202644	GCA_003376065.1	Microviridae sp.
2202644	GCA_003375585.2	Microviridae sp.
2202644	GCA_003381305.1	Microviridae sp.
2202644	GCA_003380165.1	Microviridae sp.
2202644	GCA_003374685.2	Microviridae sp.

2202644	GCA_003374025.1	Microviridae sp.
2202644	GCA_003379025.2	Microviridae sp.
2202644	GCA_003380965.1	Microviridae sp.
2202644	GCA_003378125.1	Microviridae sp.
2202644	GCA_003375445.2	Microviridae sp.
2202644	GCA_003371985.1	Microviridae sp.
2202644	GCA_003381365.2	Microviridae sp.
2202644	GCA_003376085.1	Microviridae sp.
2202644	GCA_003375565.2	Microviridae sp.
2202644	GCA_003380185.1	Microviridae sp.
2202644	GCA_003374665.2	Microviridae sp.
2202644	GCA_003374045.1	Microviridae sp.
2202644	GCA_003379005.2	Microviridae sp.
2202644	GCA_003378145.1	Microviridae sp.
2202644	GCA_003372325.2	Microviridae sp.
2202644	GCA_003372005.1	Microviridae sp.
2202644	GCA_003381345.2	Microviridae sp.
2202644	GCA_003376105.1	Microviridae sp.
2202644	GCA_003375545.2	Microviridae sp.
2202644	GCA_003380205.1	Microviridae sp.
2202644	GCA_003374645.2	Microviridae sp.
2202644	GCA_003374065.1	Microviridae sp.
2202644	GCA_003378985.2	Microviridae sp.
2202644	GCA_003378165.1	Microviridae sp.
2202644	GCA_003372305.2	Microviridae sp.
2202644	GCA_003372025.1	Microviridae sp.
2202644	GCA_003381325.2	Microviridae sp.
2202644	GCA_003376125.1	Microviridae sp.
2202644	GCA_003380225.1	Microviridae sp.
2202644	GCA_003374625.2	Microviridae sp.
2202644	GCA_003374085.1	Microviridae sp.
2202644	GCA_003378225.2	Microviridae sp.
2202644	GCA_003378185.1	Microviridae sp.
2202644	GCA_003372285.2	Microviridae sp.
2202644	GCA_003372045.1	Microviridae sp.
2202644	GCA_003381305.2	Microviridae sp.
2202644	GCA_003376145.1	Microviridae sp.
2202644	GCA_003372785.1	Microviridae sp.
2202644	GCA_003380245.1	Microviridae sp.
2202644	GCA_003378925.2	Microviridae sp.
2202644	GCA_003372225.2	Microviridae sp.
2202644	GCA_003374105.1	Microviridae sp.
2202644	GCA_003378205.2	Microviridae sp.
2202644	GCA_003378205.1	Microviridae sp.
2202644	GCA_003372265.2	Microviridae sp.
2202644	GCA_003372585.2	Microviridae sp.
2202644	GCA_003372065.1	Microviridae sp.
2202644	GCA_003381285.2	Microviridae sp.
2202644	GCA_003376165.1	Microviridae sp.
2202644	GCA_003380265.1	Microviridae sp.

2202644	GCA_003378905.2	Microviridae sp.
2202644	GCA_003374125.1	Microviridae sp.
2202644	GCA_003378185.2	Microviridae sp.
2202644	GCA_003378225.1	Microviridae sp.
2202644	GCA_003372245.2	Microviridae sp.
2202644	GCA_003381665.1	Microviridae sp.
2202644	GCA_003372085.1	Microviridae sp.
2202644	GCA_003381265.2	Microviridae sp.
2202644	GCA_003373885.2	Microviridae sp.
2202644	GCA_003376185.1	Microviridae sp.
2202644	GCA_003381325.1	Microviridae sp.
2202644	GCA_003380285.1	Microviridae sp.
2202644	GCA_003378885.2	Microviridae sp.
2202644	GCA_003374145.1	Microviridae sp.
2202644	GCA_003378165.2	Microviridae sp.
2202644	GCA_003380985.1	Microviridae sp.
2202644	GCA_003378245.1	Microviridae sp.
2202644	GCA_003375425.2	Microviridae sp.
2202644	GCA_003372105.1	Microviridae sp.
2202644	GCA_003381245.2	Microviridae sp.
2202644	GCA_003376205.1	Microviridae sp.
2202644	GCA_003380305.1	Microviridae sp.
2202644	GCA_003378865.2	Microviridae sp.
2202644	GCA_003374165.1	Microviridae sp.
2202644	GCA_003378105.2	Microviridae sp.
2202644	GCA_003378265.1	Microviridae sp.
2202644	GCA_003372125.1	Microviridae sp.
2202644	GCA_003381225.2	Microviridae sp.
2202644	GCA_003376225.1	Microviridae sp.
2202644	GCA_003380325.1	Microviridae sp.
2202644	GCA_003378845.2	Microviridae sp.
2202644	GCA_003374185.1	Microviridae sp.
2202644	GCA_003378085.2	Microviridae sp.
2202644	GCA_003378285.1	Microviridae sp.
2202644	GCA_003372165.2	Microviridae sp.
2202644	GCA_003372145.1	Microviridae sp.
2202644	GCA_003381205.2	Microviridae sp.
2202644	GCA_003376245.1	Microviridae sp.
2202644	GCA_003380345.1	Microviridae sp.
2202644	GCA_003378825.2	Microviridae sp.
2202644	GCA_003374205.1	Microviridae sp.
2202644	GCA_003378025.2	Microviridae sp.
2202644	GCA_003378305.1	Microviridae sp.
2202644	GCA_003372105.2	Microviridae sp.
2202644	GCA_003372165.1	Microviridae sp.
2202644	GCA_003381185.2	Microviridae sp.
2202644	GCA_003376265.1	Microviridae sp.
2202644	GCA_003372805.1	Microviridae sp.
2202644	GCA_003380365.1	Microviridae sp.
2202644	GCA_003378805.2	Microviridae sp.

2202644	GCA_003372205.2	Microviridae sp.
2202644	GCA_003374225.1	Microviridae sp.
2202644	GCA_003378005.2	Microviridae sp.
2202644	GCA_003372465.1	Microviridae sp.
2202644	GCA_003378325.1	Microviridae sp.
2202644	GCA_003372085.2	Microviridae sp.
2202644	GCA_003372565.2	Microviridae sp.
2202644	GCA_003372185.1	Microviridae sp.
2202644	GCA_003381165.2	Microviridae sp.
2202644	GCA_003376285.1	Microviridae sp.
2202644	GCA_003374545.2	Microviridae sp.
2202644	GCA_003380385.1	Microviridae sp.
2202644	GCA_003378785.2	Microviridae sp.
2202644	GCA_003374245.1	Microviridae sp.
2202644	GCA_003377985.2	Microviridae sp.
2202644	GCA_003373425.2	Microviridae sp.
2202644	GCA_003378345.1	Microviridae sp.
2202644	GCA_003372065.2	Microviridae sp.
2202644	GCA_003381685.1	Microviridae sp.
2202644	GCA_003372205.1	Microviridae sp.
2202644	GCA_003381145.2	Microviridae sp.
2202644	GCA_003373845.2	Microviridae sp.
2202644	GCA_003376305.1	Microviridae sp.
2202644	GCA_003381345.1	Microviridae sp.
2202644	GCA_003380405.1	Microviridae sp.
2202644	GCA_003378765.2	Microviridae sp.
2202644	GCA_003374265.1	Microviridae sp.
2202644	GCA_003377965.2	Microviridae sp.
2202644	GCA_003381005.1	Microviridae sp.
2202644	GCA_003378365.1	Microviridae sp.
2202644	GCA_003372045.2	Microviridae sp.
2202644	GCA_003375385.2	Microviridae sp.
2202644	GCA_003372225.1	Microviridae sp.
2202644	GCA_003381125.2	Microviridae sp.
2202644	GCA_003376325.1	Microviridae sp.
2202644	GCA_003380425.1	Microviridae sp.
2202644	GCA_003378745.2	Microviridae sp.
2202644	GCA_003374285.1	Microviridae sp.
2202644	GCA_003377945.2	Microviridae sp.
2202644	GCA_003378385.1	Microviridae sp.
2202644	GCA_003372025.2	Microviridae sp.
2202644	GCA_003372245.1	Microviridae sp.
2202644	GCA_003381085.2	Microviridae sp.
2202644	GCA_003376345.1	Microviridae sp.
2202644	GCA_003374485.2	Microviridae sp.
2202644	GCA_003380445.1	Microviridae sp.
2202644	GCA_003378725.2	Microviridae sp.
2202644	GCA_003374305.1	Microviridae sp.
2202644	GCA_003377925.2	Microviridae sp.
2202644	GCA_003378405.1	Microviridae sp.

2202644	GCA_003371985.2	Microviridae sp.
2202644	GCA_003372265.1	Microviridae sp.
2202644	GCA_003381065.2	Microviridae sp.
2202644	GCA_003376365.1	Microviridae sp.
2202644	GCA_003380465.1	Microviridae sp.
2202644	GCA_003378705.2	Microviridae sp.
2202644	GCA_003374325.1	Microviridae sp.
2202644	GCA_003377905.2	Microviridae sp.
2202644	GCA_003371325.2	Microviridae sp.
2202644	GCA_003378425.1	Microviridae sp.
2202644	GCA_003371965.2	Microviridae sp.
2202644	GCA_003373165.1	Microviridae sp.
2202644	GCA_003372285.1	Microviridae sp.
2202644	GCA_003381005.2	Microviridae sp.
2202644	GCA_003371745.2	Microviridae sp.
2202644	GCA_003376385.1	Microviridae sp.
2202644	GCA_003374425.2	Microviridae sp.
2202644	GCA_003372825.1	Microviridae sp.
2202644	GCA_003380485.1	Microviridae sp.
2202644	GCA_003378685.2	Microviridae sp.
2202644	GCA_003374345.1	Microviridae sp.
2202644	GCA_003377885.2	Microviridae sp.
2202644	GCA_003372485.1	Microviridae sp.
2202644	GCA_003378445.1	Microviridae sp.
2202644	GCA_003372305.1	Microviridae sp.
2202644	GCA_003380985.2	Microviridae sp.
2202644	GCA_003376405.1	Microviridae sp.
2202644	GCA_003374405.2	Microviridae sp.
2202644	GCA_003380505.1	Microviridae sp.
2202644	GCA_003378645.2	Microviridae sp.
2202644	GCA_003374365.1	Microviridae sp.
2202644	GCA_003377865.2	Microviridae sp.
2202644	GCA_003373385.2	Microviridae sp.
2202644	GCA_003378465.1	Microviridae sp.
2202644	GCA_003381705.1	Microviridae sp.
2202644	GCA_003372325.1	Microviridae sp.
2202644	GCA_003380965.2	Microviridae sp.
2202644	GCA_003373825.2	Microviridae sp.
2202644	GCA_003376425.1	Microviridae sp.
2202644	GCA_003381365.1	Microviridae sp.
2202644	GCA_003380525.1	Microviridae sp.
2202644	GCA_003378625.2	Microviridae sp.
2202644	GCA_003374385.1	Microviridae sp.
2202644	GCA_003377825.2	Microviridae sp.
2202644	GCA_003381025.1	Microviridae sp.
2202644	GCA_003378485.1	Microviridae sp.
2202644	GCA_003371905.2	Microviridae sp.
2202644	GCA_003374905.2	Microviridae sp.
2202644	GCA_003372345.1	Microviridae sp.
2202644	GCA_003380945.2	Microviridae sp.

2202644	GCA_003376445.1	Microviridae sp.
2202644	GCA_003380545.1	Microviridae sp.
2202644	GCA_003378605.2	Microviridae sp.
2202644	GCA_003374405.1	Microviridae sp.
2202644	GCA_003377805.2	Microviridae sp.
2202644	GCA_003378505.1	Microviridae sp.
2202644	GCA_003372365.1	Microviridae sp.
2202644	GCA_003380925.2	Microviridae sp.
2202644	GCA_003376465.1	Microviridae sp.
2202644	GCA_003380565.1	Microviridae sp.
2202644	GCA_003378585.2	Microviridae sp.
2202644	GCA_003374425.1	Microviridae sp.
2202644	GCA_003377785.2	Microviridae sp.
2202644	GCA_003378525.1	Microviridae sp.
2202644	GCA_003372385.1	Microviridae sp.
2202644	GCA_003380905.2	Microviridae sp.
2202644	GCA_003376485.1	Microviridae sp.
2202644	GCA_003374325.2	Microviridae sp.
2202644	GCA_003380585.1	Microviridae sp.
2202644	GCA_003378565.2	Microviridae sp.
2202644	GCA_003374445.1	Microviridae sp.
2202644	GCA_003377765.2	Microviridae sp.
2202644	GCA_003378545.1	Microviridae sp.
2202644	GCA_003373185.1	Microviridae sp.
2202644	GCA_003380865.2	Microviridae sp.
2202644	GCA_003371725.2	Microviridae sp.
2202644	GCA_003376505.1	Microviridae sp.
2202644	GCA_003374285.2	Microviridae sp.
2202644	GCA_003372845.1	Microviridae sp.
2202644	GCA_003380605.1	Microviridae sp.
2202644	GCA_003378545.2	Microviridae sp.
2202644	GCA_003374465.1	Microviridae sp.
2202644	GCA_003377725.2	Microviridae sp.
2202644	GCA_003372505.1	Microviridae sp.
2202644	GCA_003378565.1	Microviridae sp.
2202644	GCA_003371805.2	Microviridae sp.
2202644	GCA_003372525.2	Microviridae sp.
2202644	GCA_003372425.1	Microviridae sp.
2202644	GCA_003380845.2	Microviridae sp.
2202644	GCA_003376525.1	Microviridae sp.
2202644	GCA_003374265.2	Microviridae sp.
2202644	GCA_003380625.1	Microviridae sp.
2202644	GCA_003378525.2	Microviridae sp.
2202644	GCA_003374485.1	Microviridae sp.
2202644	GCA_003377705.2	Microviridae sp.
2202644	GCA_003373365.2	Microviridae sp.
2202644	GCA_003378585.1	Microviridae sp.
2202644	GCA_003371785.2	Microviridae sp.
2202644	GCA_003381725.1	Microviridae sp.
2202644	GCA_003372445.1	Microviridae sp.

2202644	GCA_003380825.2	Microviridae sp.
2202644	GCA_003373745.2	Microviridae sp.
2202644	GCA_003376545.1	Microviridae sp.
2202644	GCA_003374245.2	Microviridae sp.
2202644	GCA_003381385.1	Microviridae sp.
2202644	GCA_003380645.1	Microviridae sp.
2202644	GCA_003378505.2	Microviridae sp.
2202644	GCA_003374505.1	Microviridae sp.
2202644	GCA_003377665.2	Microviridae sp.
2202644	GCA_003381045.1	Microviridae sp.
2202644	GCA_003378605.1	Microviridae sp.
2202644	GCA_003371765.2	Microviridae sp.
2202644	GCA_003374565.2	Microviridae sp.
2202644	GCA_003380805.2	Microviridae sp.
2202644	GCA_003376565.1	Microviridae sp.
2202644	GCA_003374225.2	Microviridae sp.
2202644	GCA_003380665.1	Microviridae sp.
2202644	GCA_003378465.2	Microviridae sp.
2202644	GCA_003374525.1	Microviridae sp.
2202644	GCA_003377645.2	Microviridae sp.
2202644	GCA_003378625.1	Microviridae sp.
2202644	GCA_003380785.2	Microviridae sp.
2202644	GCA_003376585.1	Microviridae sp.
2202644	GCA_003374205.2	Microviridae sp.
2202644	GCA_003380685.1	Microviridae sp.
2202644	GCA_003378445.2	Microviridae sp.
2202644	GCA_003374545.1	Microviridae sp.
2202644	GCA_003377625.2	Microviridae sp.
2202644	GCA_003378645.1	Microviridae sp.
2202644	GCA_003380745.2	Microviridae sp.
2202644	GCA_003376605.1	Microviridae sp.
2202644	GCA_003374185.2	Microviridae sp.
2202644	GCA_003380705.1	Microviridae sp.
2202644	GCA_003378425.2	Microviridae sp.
2202644	GCA_003374565.1	Microviridae sp.
2202644	GCA_003377605.2	Microviridae sp.
2202644	GCA_003378665.1	Microviridae sp.
2202644	GCA_003373205.1	Microviridae sp.
2202644	GCA_003372525.1	Microviridae sp.
2202644	GCA_003380725.2	Microviridae sp.
2202644	GCA_003371705.2	Microviridae sp.
2202644	GCA_003376625.1	Microviridae sp.
2202644	GCA_003372865.1	Microviridae sp.
2202644	GCA_003380725.1	Microviridae sp.
2202644	GCA_003378405.2	Microviridae sp.
2202644	GCA_003374585.1	Microviridae sp.
2202644	GCA_003377585.2	Microviridae sp.
2202644	GCA_003378685.1	Microviridae sp.
2202644	GCA_003371685.2	Microviridae sp.
2202644	GCA_003372485.2	Microviridae sp.

2202644	GCA_003380705.2	Microviridae sp.
2202644	GCA_003376645.1	Microviridae sp.
2202644	GCA_003372865.2	Microviridae sp.
2202644	GCA_003380745.1	Microviridae sp.
2202644	GCA_003378365.2	Microviridae sp.
2202644	GCA_003374605.1	Microviridae sp.
2202644	GCA_003377505.2	Microviridae sp.
2202644	GCA_003373345.2	Microviridae sp.
2202644	GCA_003378705.1	Microviridae sp.
2202644	GCA_003371665.2	Microviridae sp.
2202644	GCA_003381745.1	Microviridae sp.
2202644	GCA_003372565.1	Microviridae sp.
2202644	GCA_003380685.2	Microviridae sp.
2202644	GCA_003373725.2	Microviridae sp.
2202644	GCA_003376665.1	Microviridae sp.
2202644	GCA_003374105.2	Microviridae sp.
2202644	GCA_003381405.1	Microviridae sp.
2202644	GCA_003380765.1	Microviridae sp.
2202644	GCA_003378345.2	Microviridae sp.
2202644	GCA_003374165.2	Microviridae sp.
2202644	GCA_003374625.1	Microviridae sp.
2202644	GCA_003377485.2	Microviridae sp.
2202644	GCA_003381065.1	Microviridae sp.
2202644	GCA_003378725.1	Microviridae sp.
2202644	GCA_003371645.2	Microviridae sp.
2202644	GCA_003372585.1	Microviridae sp.
2202644	GCA_003380665.2	Microviridae sp.
2202644	GCA_003376685.1	Microviridae sp.
2202644	GCA_003374085.2	Microviridae sp.
2202644	GCA_003380785.1	Microviridae sp.
2202644	GCA_003378325.2	Microviridae sp.
2202644	GCA_003374645.1	Microviridae sp.
2202644	GCA_003377465.2	Microviridae sp.
2202644	GCA_003378745.1	Microviridae sp.
2202644	GCA_003372605.1	Microviridae sp.
2202644	GCA_003380645.2	Microviridae sp.
2202644	GCA_003376705.1	Microviridae sp.
2202644	GCA_003374065.2	Microviridae sp.
2202644	GCA_003380805.1	Microviridae sp.
2202644	GCA_003378305.2	Microviridae sp.
2202644	GCA_003374665.1	Microviridae sp.
2202644	GCA_003377445.2	Microviridae sp.
2202644	GCA_003378765.1	Microviridae sp.
2202644	GCA_003372625.1	Microviridae sp.
2202644	GCA_003380625.2	Microviridae sp.
2202644	GCA_003376725.1	Microviridae sp.
2202644	GCA_003374045.2	Microviridae sp.
2202644	GCA_003380825.1	Microviridae sp.
2202644	GCA_003378285.2	Microviridae sp.
2202644	GCA_003374685.1	Microviridae sp.

2202644	GCA_003377405.2	Microviridae sp.
2202644	GCA_003378785.1	Microviridae sp.
2202644	GCA_003372645.1	Microviridae sp.
2202644	GCA_003380605.2	Microviridae sp.
2202644	GCA_003376745.1	Microviridae sp.
2202644	GCA_003374025.2	Microviridae sp.
2202644	GCA_003372885.1	Microviridae sp.
2202644	GCA_003370605.1	Microviridae sp.
2202644	GCA_003378265.2	Microviridae sp.
2202644	GCA_003374705.1	Microviridae sp.
2202644	GCA_003377385.2	Microviridae sp.
2202644	GCA_003372545.1	Microviridae sp.
2202644	GCA_003378805.1	Microviridae sp.
2202644	GCA_003371545.2	Microviridae sp.
2202644	GCA_003372465.2	Microviridae sp.
2202644	GCA_003372665.1	Microviridae sp.
2202644	GCA_003380585.2	Microviridae sp.
2202644	GCA_003376765.1	Microviridae sp.
2202644	GCA_003374005.2	Microviridae sp.
2202644	GCA_003372845.2	Microviridae sp.
2202644	GCA_003370625.1	Microviridae sp.
2202644	GCA_003378245.2	Microviridae sp.
2202644	GCA_003374725.1	Microviridae sp.
2202644	GCA_003377365.2	Microviridae sp.
2202644	GCA_003373325.2	Microviridae sp.
2202644	GCA_003378825.1	Microviridae sp.
2202644	GCA_003381765.1	Microviridae sp.
2202644	GCA_003372685.1	Microviridae sp.
2202644	GCA_003380565.2	Microviridae sp.
2202644	GCA_003373705.2	Microviridae sp.
2202644	GCA_003376785.1	Microviridae sp.
2202644	GCA_003373985.2	Microviridae sp.
2202644	GCA_003381425.1	Microviridae sp.
2202644	GCA_003370645.1	Microviridae sp.
2202644	GCA_003374125.2	Microviridae sp.
2202644	GCA_003374745.1	Microviridae sp.
2202644	GCA_003377305.2	Microviridae sp.
2202644	GCA_003381085.1	Microviridae sp.
2202644	GCA_003378845.1	Microviridae sp.
2202644	GCA_003374525.2	Microviridae sp.
2202644	GCA_003372705.1	Microviridae sp.
2202644	GCA_003380545.2	Microviridae sp.
2202644	GCA_003376805.1	Microviridae sp.
2202644	GCA_003373965.2	Microviridae sp.
2202644	GCA_003370665.1	Microviridae sp.
2202644	GCA_003374765.1	Microviridae sp.
2202644	GCA_003377285.2	Microviridae sp.
2202644	GCA_003378865.1	Microviridae sp.
2202644	GCA_003371465.2	Microviridae sp.
2202644	GCA_003372725.1	Microviridae sp.

2202644	GCA_003380525.2	Microviridae sp.
2202644	GCA_003376825.1	Microviridae sp.
2202644	GCA_003370685.1	Microviridae sp.
2202644	GCA_003374785.1	Microviridae sp.
2202644	GCA_003377265.2	Microviridae sp.
2202644	GCA_003378885.1	Microviridae sp.
2202644	GCA_003380505.2	Microviridae sp.
2202644	GCA_003376845.1	Microviridae sp.
2202644	GCA_003370705.1	Microviridae sp.
2202644	GCA_003374805.1	Microviridae sp.
2202644	GCA_003377245.2	Microviridae sp.
2202644	GCA_003378905.1	Microviridae sp.
2202644	GCA_003380485.2	Microviridae sp.
2202644	GCA_003376865.1	Microviridae sp.
2202644	GCA_003370725.1	Microviridae sp.
2202644	GCA_003374825.1	Microviridae sp.
2202644	GCA_003377225.2	Microviridae sp.
2202644	GCA_003378925.1	Microviridae sp.
2202644	GCA_003371365.2	Microviridae sp.
2202644	GCA_003372445.2	Microviridae sp.
2202644	GCA_003380465.2	Microviridae sp.
2202644	GCA_003376885.1	Microviridae sp.
2202644	GCA_003370745.1	Microviridae sp.
2202644	GCA_003374845.1	Microviridae sp.
2202644	GCA_003377205.2	Microviridae sp.
2202644	GCA_003378945.1	Microviridae sp.
2202644	GCA_003371345.2	Microviridae sp.
2202644	GCA_003381785.1	Microviridae sp.
2202644	GCA_003380445.2	Microviridae sp.
2202644	GCA_003376905.1	Microviridae sp.
2202644	GCA_003381445.1	Microviridae sp.
2202644	GCA_003370765.1	Microviridae sp.
2202644	GCA_003374865.1	Microviridae sp.
2202644	GCA_003377105.2	Microviridae sp.
2202644	GCA_003381105.1	Microviridae sp.
2202644	GCA_003378965.1	Microviridae sp.
2202644	GCA_003374505.2	Microviridae sp.
2202644	GCA_003380425.2	Microviridae sp.
2202644	GCA_003376925.1	Microviridae sp.
2202644	GCA_003370785.1	Microviridae sp.
2202644	GCA_003374885.1	Microviridae sp.
2202644	GCA_003377085.2	Microviridae sp.
2202644	GCA_003378985.1	Microviridae sp.
2202644	GCA_003371305.2	Microviridae sp.
2202644	GCA_003380405.2	Microviridae sp.
2202644	GCA_003376945.1	Microviridae sp.
2202644	GCA_003370805.1	Microviridae sp.
2202644	GCA_003374905.1	Microviridae sp.
2202644	GCA_003377065.2	Microviridae sp.
2202644	GCA_003379005.1	Microviridae sp.

2202644	GCA_003371265.2	Microviridae sp.
2202644	GCA_003380385.2	Microviridae sp.
2202644	GCA_003376965.1	Microviridae sp.
2202644	GCA_003370825.1	Microviridae sp.
2202644	GCA_003374925.1	Microviridae sp.
2202644	GCA_003377045.2	Microviridae sp.
2202644	GCA_003379025.1	Microviridae sp.
2202644	GCA_003371245.2	Microviridae sp.
2202644	GCA_003380325.2	Microviridae sp.
2202644	GCA_003376985.1	Microviridae sp.
2202644	GCA_003370845.1	Microviridae sp.
2202644	GCA_003374945.1	Microviridae sp.
2202644	GCA_003377005.2	Microviridae sp.
2202644	GCA_003379045.1	Microviridae sp.
2202644	GCA_003371225.2	Microviridae sp.
2202644	GCA_003372425.2	Microviridae sp.
2202644	GCA_003372905.1	Microviridae sp.
2202644	GCA_003380305.2	Microviridae sp.
2202644	GCA_003377005.1	Microviridae sp.
2202644	GCA_003373665.2	Microviridae sp.
2202644	GCA_003370865.1	Microviridae sp.
2202644	GCA_003374965.1	Microviridae sp.
2202644	GCA_003376985.2	Microviridae sp.
2202644	GCA_003379065.1	Microviridae sp.
2202644	GCA_003371205.2	Microviridae sp.
2202644	GCA_003381805.1	Microviridae sp.
2202644	GCA_003372925.1	Microviridae sp.
2202644	GCA_003380285.2	Microviridae sp.
2202644	GCA_003377025.1	Microviridae sp.
2202644	GCA_003373645.2	Microviridae sp.
2202644	GCA_003381465.1	Microviridae sp.
2202644	GCA_003370885.1	Microviridae sp.
2202644	GCA_003374985.1	Microviridae sp.
2202644	GCA_003376965.2	Microviridae sp.
2202644	GCA_003381125.1	Microviridae sp.
2202644	GCA_003379085.1	Microviridae sp.
2202644	GCA_003371185.2	Microviridae sp.
2202644	GCA_003380265.2	Microviridae sp.
2202644	GCA_003377045.1	Microviridae sp.
2202644	GCA_003370905.1	Microviridae sp.
2202644	GCA_003375005.1	Microviridae sp.
2202644	GCA_003376945.2	Microviridae sp.
2202644	GCA_003379105.1	Microviridae sp.
2202644	GCA_003371165.2	Microviridae sp.
2202644	GCA_003380245.2	Microviridae sp.
2202644	GCA_003377065.1	Microviridae sp.
2202644	GCA_003370925.1	Microviridae sp.
2202644	GCA_003375025.1	Microviridae sp.
2202644	GCA_003376925.2	Microviridae sp.
2202644	GCA_003379125.1	Microviridae sp.

2202644	GCA_003371145.2	Microviridae sp.
2202644	GCA_003380225.2	Microviridae sp.
2202644	GCA_003377085.1	Microviridae sp.
2202644	GCA_003373585.2	Microviridae sp.
2202644	GCA_003370945.1	Microviridae sp.
2202644	GCA_003375045.1	Microviridae sp.
2202644	GCA_003376905.2	Microviridae sp.
2202644	GCA_003379145.1	Microviridae sp.
2202644	GCA_003371125.2	Microviridae sp.
2202644	GCA_003380205.2	Microviridae sp.
2202644	GCA_003371605.2	Microviridae sp.
2202644	GCA_003377105.1	Microviridae sp.
2202644	GCA_003372945.1	Microviridae sp.
2202644	GCA_003370965.1	Microviridae sp.
2202644	GCA_003375065.1	Microviridae sp.
2202644	GCA_003376885.2	Microviridae sp.
2202644	GCA_003379165.1	Microviridae sp.
2202644	GCA_003371105.2	Microviridae sp.
2202644	GCA_003372405.2	Microviridae sp.
2202644	GCA_003380185.2	Microviridae sp.
2202644	GCA_003377125.1	Microviridae sp.
2202644	GCA_003370985.1	Microviridae sp.
2202644	GCA_003375085.1	Microviridae sp.
2202644	GCA_003376865.2	Microviridae sp.
2202644	GCA_003379185.1	Microviridae sp.
2202644	GCA_003371085.2	Microviridae sp.
2202644	GCA_003381825.1	Microviridae sp.
2202644	GCA_003380165.2	Microviridae sp.
2202644	GCA_003373625.2	Microviridae sp.
2202644	GCA_003377145.1	Microviridae sp.
2202644	GCA_003381485.1	Microviridae sp.
2202644	GCA_003371005.1	Microviridae sp.
2202644	GCA_003375105.1	Microviridae sp.
2202644	GCA_003376845.2	Microviridae sp.
2202644	GCA_003381145.1	Microviridae sp.
2202644	GCA_003379205.1	Microviridae sp.
2202644	GCA_003371065.2	Microviridae sp.
2202644	GCA_003374465.2	Microviridae sp.
2202644	GCA_003380145.2	Microviridae sp.
2202644	GCA_003377165.1	Microviridae sp.
2202644	GCA_003373505.2	Microviridae sp.
2202644	GCA_003371025.1	Microviridae sp.
2202644	GCA_003375125.1	Microviridae sp.
2202644	GCA_003376785.2	Microviridae sp.
2202644	GCA_003379225.1	Microviridae sp.
2202644	GCA_003371045.2	Microviridae sp.
2202644	GCA_003373085.1	Microviridae sp.
2202644	GCA_003380125.2	Microviridae sp.
2202644	GCA_003377185.1	Microviridae sp.
2202644	GCA_003373485.2	Microviridae sp.

2202644	GCA_003371045.1	Microviridae sp.
2202644	GCA_003375145.1	Microviridae sp.
2202644	GCA_003376765.2	Microviridae sp.
2202644	GCA_003379245.1	Microviridae sp.
2202644	GCA_003371025.2	Microviridae sp.
2202644	GCA_003373105.1	Microviridae sp.
2202644	GCA_003380105.2	Microviridae sp.
2202644	GCA_003377205.1	Microviridae sp.
2202644	GCA_003373465.2	Microviridae sp.
2202644	GCA_003371065.1	Microviridae sp.
2202644	GCA_003375165.1	Microviridae sp.
2202644	GCA_003376725.2	Microviridae sp.
2202644	GCA_003379265.1	Microviridae sp.
2202644	GCA_003371005.2	Microviridae sp.
2202644	GCA_003373125.1	Microviridae sp.
2202644	GCA_003380085.2	Microviridae sp.
2202644	GCA_003371585.2	Microviridae sp.
2202644	GCA_003377225.1	Microviridae sp.
2202644	GCA_003373445.2	Microviridae sp.
2202644	GCA_003372965.1	Microviridae sp.
2202644	GCA_003371085.1	Microviridae sp.
2202644	GCA_003375185.1	Microviridae sp.
2202644	GCA_003376705.2	Microviridae sp.
2202644	GCA_003379285.1	Microviridae sp.
2202644	GCA_003370985.2	Microviridae sp.
2202644	GCA_003373145.1	Microviridae sp.
2202644	GCA_003380045.2	Microviridae sp.
2202644	GCA_003377245.1	Microviridae sp.
2202644	GCA_003371105.1	Microviridae sp.
2202644	GCA_003375205.1	Microviridae sp.
2202644	GCA_003376685.2	Microviridae sp.
2202644	GCA_003379305.1	Microviridae sp.
2202644	GCA_003370965.2	Microviridae sp.
2202644	GCA_003381845.1	Microviridae sp.
2202644	GCA_003380025.2	Microviridae sp.
2202644	GCA_003373605.2	Microviridae sp.
2202644	GCA_003377265.1	Microviridae sp.
2202644	GCA_003381505.1	Microviridae sp.
2202644	GCA_003371125.1	Microviridae sp.
2202644	GCA_003375225.1	Microviridae sp.
2202644	GCA_003376665.2	Microviridae sp.
2202644	GCA_003381165.1	Microviridae sp.
2202644	GCA_003379325.1	Microviridae sp.
2202644	GCA_003370945.2	Microviridae sp.
2202644	GCA_003380005.2	Microviridae sp.
2202644	GCA_003377285.1	Microviridae sp.
2202644	GCA_003371145.1	Microviridae sp.
2202644	GCA_003375245.1	Microviridae sp.
2202644	GCA_003376645.2	Microviridae sp.
2202644	GCA_003379345.1	Microviridae sp.

2202644	GCA_003370925.2	Microviridae sp.
2202644	GCA_003379985.2	Microviridae sp.
2202644	GCA_003377305.1	Microviridae sp.
2202644	GCA_003371165.1	Microviridae sp.
2202644	GCA_003375265.1	Microviridae sp.
2202644	GCA_003376625.2	Microviridae sp.
2202644	GCA_003379365.1	Microviridae sp.
2202644	GCA_003370905.2	Microviridae sp.
2202644	GCA_003373225.1	Microviridae sp.
2202644	GCA_003379965.2	Microviridae sp.
2202644	GCA_003377325.1	Microviridae sp.
2202644	GCA_003371185.1	Microviridae sp.
2202644	GCA_003375285.1	Microviridae sp.
2202644	GCA_003376585.2	Microviridae sp.
2202644	GCA_003379385.1	Microviridae sp.
2202644	GCA_003370885.2	Microviridae sp.
2202644	GCA_003373245.1	Microviridae sp.
2202644	GCA_003379945.2	Microviridae sp.
2202644	GCA_003371565.2	Microviridae sp.
2202644	GCA_003377345.1	Microviridae sp.
2202644	GCA_003373305.2	Microviridae sp.
2202644	GCA_003372985.1	Microviridae sp.
2202644	GCA_003371205.1	Microviridae sp.
2202644	GCA_003371945.2	Microviridae sp.
2202644	GCA_003375305.1	Microviridae sp.
2202644	GCA_003376565.2	Microviridae sp.
2202644	GCA_003379405.1	Microviridae sp.
2202644	GCA_003370865.2	Microviridae sp.
2202644	GCA_003372365.2	Microviridae sp.
2202644	GCA_003373265.1	Microviridae sp.
2202644	GCA_003379925.2	Microviridae sp.
2202644	GCA_003377365.1	Microviridae sp.
2202644	GCA_003373285.2	Microviridae sp.
2202644	GCA_003371225.1	Microviridae sp.
2202644	GCA_003375325.1	Microviridae sp.
2202644	GCA_003376545.2	Microviridae sp.
2202644	GCA_003379425.1	Microviridae sp.
2202644	GCA_003370845.2	Microviridae sp.
2202644	GCA_003381865.1	Microviridae sp.
2202644	GCA_003373285.1	Microviridae sp.
2202644	GCA_003379905.2	Microviridae sp.
2202644	GCA_003377385.1	Microviridae sp.
2202644	GCA_003373265.2	Microviridae sp.
2202644	GCA_003381525.1	Microviridae sp.
2202644	GCA_003371245.1	Microviridae sp.
2202644	GCA_003375345.1	Microviridae sp.
2202644	GCA_003376505.2	Microviridae sp.
2202644	GCA_003381185.1	Microviridae sp.
2202644	GCA_003379445.1	Microviridae sp.
2202644	GCA_003370825.2	Microviridae sp.

2202644	GCA_003373305.1	Microviridae sp.
2202644	GCA_003379885.2	Microviridae sp.
2202644	GCA_003380845.1	Microviridae sp.
2202644	GCA_003377405.1	Microviridae sp.
2202644	GCA_003373225.2	Microviridae sp.
2202644	GCA_003371265.1	Microviridae sp.
2202644	GCA_003375365.1	Microviridae sp.
2202644	GCA_003376465.2	Microviridae sp.
2202644	GCA_003379465.1	Microviridae sp.
2202644	GCA_003370765.2	Microviridae sp.
2202644	GCA_003373325.1	Microviridae sp.
2202644	GCA_003379865.2	Microviridae sp.
2202644	GCA_003377425.1	Microviridae sp.
2202644	GCA_003373205.2	Microviridae sp.
2202644	GCA_003371285.1	Microviridae sp.
2202644	GCA_003375385.1	Microviridae sp.
2202644	GCA_003376445.2	Microviridae sp.
2202644	GCA_003379485.1	Microviridae sp.
2202644	GCA_003370745.2	Microviridae sp.
2202644	GCA_003373345.1	Microviridae sp.
2202644	GCA_003379845.2	Microviridae sp.
2202644	GCA_003377445.1	Microviridae sp.
2202644	GCA_003371305.1	Microviridae sp.
2202644	GCA_003375405.1	Microviridae sp.
2202644	GCA_003376425.2	Microviridae sp.
2202644	GCA_003379505.1	Microviridae sp.
2202644	GCA_003370725.2	Microviridae sp.
2202644	GCA_003373365.1	Microviridae sp.
2202644	GCA_003379785.2	Microviridae sp.
2202644	GCA_003377465.1	Microviridae sp.
2202644	GCA_003373005.1	Microviridae sp.
2202644	GCA_003371325.1	Microviridae sp.
2202644	GCA_003371925.2	Microviridae sp.
2202644	GCA_003375425.1	Microviridae sp.
2202644	GCA_003376405.2	Microviridae sp.
2202644	GCA_003379525.1	Microviridae sp.
2202644	GCA_003370705.2	Microviridae sp.
2202644	GCA_003372345.2	Microviridae sp.
2202644	GCA_003373385.1	Microviridae sp.
2202644	GCA_003379765.2	Microviridae sp.
2202644	GCA_003377485.1	Microviridae sp.
2202644	GCA_003371345.1	Microviridae sp.
2202644	GCA_003375445.1	Microviridae sp.
2202644	GCA_003376365.2	Microviridae sp.
2202644	GCA_003373185.2	Microviridae sp.
2202644	GCA_003379545.1	Microviridae sp.
2202644	GCA_003370645.2	Microviridae sp.
2202644	GCA_003381885.1	Microviridae sp.
2202644	GCA_003373405.1	Microviridae sp.
2202644	GCA_003379745.2	Microviridae sp.

2202644	GCA_003373565.2	Microviridae sp.
2202644	GCA_003377505.1	Microviridae sp.
2202644	GCA_003373125.2	Microviridae sp.
2202644	GCA_003381545.1	Microviridae sp.
2202644	GCA_003371365.1	Microviridae sp.
2202644	GCA_003375465.1	Microviridae sp.
2202644	GCA_003376345.2	Microviridae sp.
2202644	GCA_003381205.1	Microviridae sp.
2202644	GCA_003379565.1	Microviridae sp.
2202644	GCA_003370785.2	Microviridae sp.
2202644	GCA_003374385.2	Microviridae sp.
2202644	GCA_003373425.1	Microviridae sp.
2202644	GCA_003379705.2	Microviridae sp.
2202644	GCA_003380865.1	Microviridae sp.
2202644	GCA_003377525.1	Microviridae sp.
2202644	GCA_003373105.2	Microviridae sp.
2202644	GCA_003371385.1	Microviridae sp.
2202644	GCA_003375485.1	Microviridae sp.
2202644	GCA_003376305.2	Microviridae sp.
2202644	GCA_003379585.1	Microviridae sp.
2202644	GCA_003380365.2	Microviridae sp.
2202644	GCA_003373445.1	Microviridae sp.
2202644	GCA_003379685.2	Microviridae sp.
2202644	GCA_003377545.1	Microviridae sp.
2202644	GCA_003373045.2	Microviridae sp.
2202644	GCA_003371405.1	Microviridae sp.
2202644	GCA_003375505.1	Microviridae sp.
2202644	GCA_003376285.2	Microviridae sp.
2202644	GCA_003379605.1	Microviridae sp.
2202644	GCA_003380345.2	Microviridae sp.
2202644	GCA_003373465.1	Microviridae sp.
2202644	GCA_003379665.2	Microviridae sp.
2202644	GCA_003377565.1	Microviridae sp.
2202644	GCA_003373005.2	Microviridae sp.
2202644	GCA_003371425.1	Microviridae sp.
2202644	GCA_003375525.1	Microviridae sp.
2202644	GCA_003376265.2	Microviridae sp.
2202644	GCA_003379625.1	Microviridae sp.
2202644	GCA_003375345.2	Microviridae sp.
2202644	GCA_003373485.1	Microviridae sp.
2202644	GCA_003379645.2	Microviridae sp.
2202644	GCA_003371505.2	Microviridae sp.
2202644	GCA_003377585.1	Microviridae sp.
2202644	GCA_003372985.2	Microviridae sp.
2202644	GCA_003373025.1	Microviridae sp.
2202644	GCA_003371445.1	Microviridae sp.
2202644	GCA_003375545.1	Microviridae sp.
2202644	GCA_003376245.2	Microviridae sp.
2202644	GCA_003379645.1	Microviridae sp.
2202644	GCA_003375325.2	Microviridae sp.

2202644	GCA_003373505.1	Microviridae sp.
2202644	GCA_003379625.2	Microviridae sp.
2202644	GCA_003377605.1	Microviridae sp.
2202644	GCA_003372965.2	Microviridae sp.
2202644	GCA_003371465.1	Microviridae sp.
2202644	GCA_003381925.2	Microviridae sp.
2202644	GCA_003375565.1	Microviridae sp.
2202644	GCA_003376225.2	Microviridae sp.
2202644	GCA_003373165.2	Microviridae sp.
2202644	GCA_003379665.1	Microviridae sp.
2202644	GCA_003375285.2	Microviridae sp.
2202644	GCA_003381905.1	Microviridae sp.
2202644	GCA_003373525.1	Microviridae sp.
2202644	GCA_003379605.2	Microviridae sp.
2202644	GCA_003373545.2	Microviridae sp.
2202644	GCA_003377625.1	Microviridae sp.
2202644	GCA_003372905.2	Microviridae sp.
2202644	GCA_003381565.1	Microviridae sp.
2202644	GCA_003371485.1	Microviridae sp.
2202644	GCA_003381905.2	Microviridae sp.
2202644	GCA_003375585.1	Microviridae sp.
2202644	GCA_003376205.2	Microviridae sp.
2202644	GCA_003381225.1	Microviridae sp.
2202644	GCA_003379685.1	Microviridae sp.
2202644	GCA_003375265.2	Microviridae sp.
2202644	GCA_003374365.2	Microviridae sp.
2202644	GCA_003373545.1	Microviridae sp.
2202644	GCA_003379585.2	Microviridae sp.
2202644	GCA_003380885.1	Microviridae sp.
2202644	GCA_003377645.1	Microviridae sp.
2202644	GCA_003371505.1	Microviridae sp.
2202644	GCA_003381865.2	Microviridae sp.
2202644	GCA_003375605.1	Microviridae sp.
2202644	GCA_003376185.2	Microviridae sp.
2202644	GCA_003379705.1	Microviridae sp.
2202644	GCA_003375245.2	Microviridae sp.
2202644	GCA_003373565.1	Microviridae sp.
2202644	GCA_003379565.2	Microviridae sp.
2202644	GCA_003377665.1	Microviridae sp.
2202644	GCA_003371525.1	Microviridae sp.
2202644	GCA_003381845.2	Microviridae sp.
2202644	GCA_003375625.1	Microviridae sp.
2202644	GCA_003376165.2	Microviridae sp.
2202644	GCA_003379725.1	Microviridae sp.
2202644	GCA_003375225.2	Microviridae sp.
2202644	GCA_003373585.1	Microviridae sp.
2202644	GCA_003379545.2	Microviridae sp.
2202644	GCA_003377685.1	Microviridae sp.
2202644	GCA_003372825.2	Microviridae sp.
2202644	GCA_003371545.1	Microviridae sp.

2202644	GCA_003381825.2	Microviridae sp.
2202644	GCA_003375645.1	Microviridae sp.
2202644	GCA_003376145.2	Microviridae sp.
2202644	GCA_003379745.1	Microviridae sp.
2202644	GCA_003375205.2	Microviridae sp.
2202644	GCA_003373605.1	Microviridae sp.
2202644	GCA_003379525.2	Microviridae sp.
2202644	GCA_003371485.2	Microviridae sp.
2202644	GCA_003377705.1	Microviridae sp.
2202644	GCA_003372805.2	Microviridae sp.
2202644	GCA_003373045.1	Microviridae sp.
2202644	GCA_003371565.1	Microviridae sp.
2202644	GCA_003381805.2	Microviridae sp.
2202644	GCA_003371885.2	Microviridae sp.
2202644	GCA_003375665.1	Microviridae sp.
2202644	GCA_003376125.2	Microviridae sp.
2202644	GCA_003379765.1	Microviridae sp.
2202644	GCA_003375185.2	Microviridae sp.
2202644	GCA_003373625.1	Microviridae sp.
2202644	GCA_003379505.2	Microviridae sp.
2202644	GCA_003377725.1	Microviridae sp.
2202644	GCA_003372785.2	Microviridae sp.
2202644	GCA_003371585.1	Microviridae sp.
2202644	GCA_003381785.2	Microviridae sp.
2202644	GCA_003375685.1	Microviridae sp.
2202644	GCA_003376085.2	Microviridae sp.
2202644	GCA_003373145.2	Microviridae sp.
2202644	GCA_003379785.1	Microviridae sp.
2202644	GCA_003375125.2	Microviridae sp.
2202644	GCA_003381925.1	Microviridae sp.
2202644	GCA_003373645.1	Microviridae sp.
2202644	GCA_003379485.2	Microviridae sp.
2202644	GCA_003373525.2	Microviridae sp.
2202644	GCA_003377745.1	Microviridae sp.
2202644	GCA_003372745.2	Microviridae sp.
2202644	GCA_003381585.1	Microviridae sp.
2202644	GCA_003371605.1	Microviridae sp.
2202644	GCA_003381745.2	Microviridae sp.
2202644	GCA_003375705.1	Microviridae sp.
2202644	GCA_003376065.2	Microviridae sp.
2202644	GCA_003381245.1	Microviridae sp.
2202644	GCA_003379805.1	Microviridae sp.
2202644	GCA_003375105.2	Microviridae sp.
2202644	GCA_003374345.2	Microviridae sp.
2202644	GCA_003373665.1	Microviridae sp.
2202644	GCA_003379465.2	Microviridae sp.
2202644	GCA_003380905.1	Microviridae sp.
2202644	GCA_003377765.1	Microviridae sp.
2202644	GCA_003372725.2	Microviridae sp.
2202644	GCA_003375505.2	Microviridae sp.

2202644	GCA_003371625.1	Microviridae sp.
2202644	GCA_003381725.2	Microviridae sp.
2202644	GCA_003375725.1	Microviridae sp.
2202644	GCA_003376045.2	Microviridae sp.
2202644	GCA_003379825.1	Microviridae sp.
2202644	GCA_003375085.2	Microviridae sp.
2202644	GCA_003373685.1	Microviridae sp.
2202644	GCA_003379445.2	Microviridae sp.
2202644	GCA_003377785.1	Microviridae sp.
2202644	GCA_003372705.2	Microviridae sp.
2202644	GCA_003371645.1	Microviridae sp.
2202644	GCA_003381705.2	Microviridae sp.
2202644	GCA_003375745.1	Microviridae sp.
2202644	GCA_003376025.2	Microviridae sp.
2202644	GCA_003379845.1	Microviridae sp.
2202644	GCA_003375065.2	Microviridae sp.
2202644	GCA_003373705.1	Microviridae sp.
2202644	GCA_003379425.2	Microviridae sp.
2202644	GCA_003377805.1	Microviridae sp.
2202644	GCA_003372685.2	Microviridae sp.
2202644	GCA_003371665.1	Microviridae sp.
2202644	GCA_003381685.2	Microviridae sp.
2202644	GCA_003375765.1	Microviridae sp.
2202644	GCA_003375985.2	Microviridae sp.
2202644	GCA_003379865.1	Microviridae sp.
2202644	GCA_003375045.2	Microviridae sp.
2202644	GCA_003373725.1	Microviridae sp.
2202644	GCA_003379405.2	Microviridae sp.
2202644	GCA_003377825.1	Microviridae sp.
2202644	GCA_003372665.2	Microviridae sp.
2202644	GCA_003373065.1	Microviridae sp.
2202644	GCA_003371685.1	Microviridae sp.
2202644	GCA_003381665.2	Microviridae sp.
2202644	GCA_003371865.2	Microviridae sp.
2202644	GCA_003375785.1	Microviridae sp.
2202644	GCA_003375965.2	Microviridae sp.
2202644	GCA_003379885.1	Microviridae sp.
2202644	GCA_003375025.2	Microviridae sp.
2202644	GCA_003373745.1	Microviridae sp.
2202644	GCA_003379385.2	Microviridae sp.
2202644	GCA_003377845.1	Microviridae sp.
2202644	GCA_003381945.1	Microviridae sp.
2202644	GCA_003406455.1	Microviridae sp.
2202644	GCA_003404415.1	Microviridae sp.
2202644	GCA_003381965.1	Microviridae sp.
2202644	GCA_003406475.1	Microviridae sp.
2202644	GCA_003404435.1	Microviridae sp.
2202644	GCA_003406795.1	Microviridae sp.
2202644	GCA_003381985.1	Microviridae sp.
2202644	GCA_003406495.1	Microviridae sp.

2202644	GCA_003404455.1	Microviridae sp.
2202644	GCA_003406115.1	Microviridae sp.
2202644	GCA_003382005.1	Microviridae sp.
2202644	GCA_003404475.1	Microviridae sp.
2202644	GCA_003382025.1	Microviridae sp.
2202644	GCA_003406535.1	Microviridae sp.
2202644	GCA_003404495.1	Microviridae sp.
2202644	GCA_003382045.1	Microviridae sp.
2202644	GCA_003404075.1	Microviridae sp.
2202644	GCA_003406555.1	Microviridae sp.
2202644	GCA_003404515.1	Microviridae sp.
2202644	GCA_003382065.1	Microviridae sp.
2202644	GCA_003406575.1	Microviridae sp.
2202644	GCA_003404535.1	Microviridae sp.
2202644	GCA_003382085.1	Microviridae sp.
2202644	GCA_003406595.1	Microviridae sp.
2202644	GCA_003404555.1	Microviridae sp.
2202644	GCA_003406815.1	Microviridae sp.
2202644	GCA_003382105.1	Microviridae sp.
2202644	GCA_003406615.1	Microviridae sp.
2202644	GCA_003404575.1	Microviridae sp.
2202644	GCA_003406135.1	Microviridae sp.
2202644	GCA_003382125.1	Microviridae sp.
2202644	GCA_003404595.1	Microviridae sp.
2202644	GCA_003382145.1	Microviridae sp.
2202644	GCA_003406655.1	Microviridae sp.
2202644	GCA_003382165.1	Microviridae sp.
2202644	GCA_003404095.1	Microviridae sp.
2202644	GCA_003406675.1	Microviridae sp.
2202644	GCA_003404635.1	Microviridae sp.
2202644	GCA_003406695.1	Microviridae sp.
2202644	GCA_003404655.1	Microviridae sp.
2202644	GCA_003406715.1	Microviridae sp.
2202644	GCA_003381985.2	Microviridae sp.
2202644	GCA_003404675.1	Microviridae sp.
2202644	GCA_003404695.1	Microviridae sp.
2202644	GCA_003406755.1	Microviridae sp.
2202644	GCA_003404715.1	Microviridae sp.
2202644	GCA_003406775.1	Microviridae sp.
2202644	GCA_003404735.1	Microviridae sp.
2202644	GCA_003404115.1	Microviridae sp.
2202644	GCA_003403775.1	Microviridae sp.
2202644	GCA_003404755.1	Microviridae sp.
2202644	GCA_003404775.1	Microviridae sp.
2202644	GCA_003406835.1	Microviridae sp.
2202644	GCA_003404795.1	Microviridae sp.
2202644	GCA_003406855.1	Microviridae sp.
2202644	GCA_003404815.1	Microviridae sp.
2202644	GCA_003406875.1	Microviridae sp.
2202644	GCA_003404835.1	Microviridae sp.

2202644	GCA_003406895.1	Microviridae sp.
2202644	GCA_003407455.1	Microviridae sp.
2202644	GCA_003404855.1	Microviridae sp.
2202644	GCA_003406915.1	Microviridae sp.
2202644	GCA_003404875.1	Microviridae sp.
2202644	GCA_003406935.1	Microviridae sp.
2202644	GCA_003404895.1	Microviridae sp.
2202644	GCA_003406955.1	Microviridae sp.
2202644	GCA_003404915.1	Microviridae sp.
2202644	GCA_003404935.1	Microviridae sp.
2202644	GCA_003404955.1	Microviridae sp.
2202644	GCA_003407015.1	Microviridae sp.
2202644	GCA_003404975.1	Microviridae sp.
2202644	GCA_003407035.1	Microviridae sp.
2202644	GCA_003407255.1	Microviridae sp.
2202644	GCA_003404995.1	Microviridae sp.
2202644	GCA_003407055.1	Microviridae sp.
2202644	GCA_003405015.1	Microviridae sp.
2202644	GCA_003405035.1	Microviridae sp.
2202644	GCA_003407095.1	Microviridae sp.
2202644	GCA_003405055.1	Microviridae sp.
2202644	GCA_003407115.1	Microviridae sp.
2202644	GCA_003405075.1	Microviridae sp.
2202644	GCA_003407135.1	Microviridae sp.
2202644	GCA_003405095.1	Microviridae sp.
2202644	GCA_003407155.1	Microviridae sp.
2202644	GCA_003405115.1	Microviridae sp.
2202644	GCA_003407175.1	Microviridae sp.
2202644	GCA_003405135.1	Microviridae sp.
2202644	GCA_003407195.1	Microviridae sp.
2202644	GCA_003405155.1	Microviridae sp.
2202644	GCA_003407215.1	Microviridae sp.
2202644	GCA_003405175.1	Microviridae sp.
2202644	GCA_003406235.1	Microviridae sp.
2202644	GCA_003407235.1	Microviridae sp.
2202644	GCA_003405195.1	Microviridae sp.
2202644	GCA_003405215.1	Microviridae sp.
2202644	GCA_003407275.1	Microviridae sp.
2202644	GCA_003405235.1	Microviridae sp.
2202644	GCA_003407295.1	Microviridae sp.
2202644	GCA_003405255.1	Microviridae sp.
2202644	GCA_003405275.1	Microviridae sp.
2202644	GCA_003407335.1	Microviridae sp.
2202644	GCA_003406515.1	Microviridae sp.
2202644	GCA_003406255.1	Microviridae sp.
2202644	GCA_003405315.1	Microviridae sp.
2202644	GCA_003407375.1	Microviridae sp.
2202644	GCA_003405335.1	Microviridae sp.
2202644	GCA_003404215.1	Microviridae sp.
2202644	GCA_003403875.1	Microviridae sp.

2202644	GCA_003405355.1	Microviridae sp.
2202644	GCA_003406995.1	Microviridae sp.
2202644	GCA_003405375.1	Microviridae sp.
2202644	GCA_003407435.1	Microviridae sp.
2202644	GCA_003405395.1	Microviridae sp.
2202644	GCA_003403355.1	Microviridae sp.
2202644	GCA_003405415.1	Microviridae sp.
2202644	GCA_003406275.1	Microviridae sp.
2202644	GCA_003403375.1	Microviridae sp.
2202644	GCA_003407475.1	Microviridae sp.
2202644	GCA_003405435.1	Microviridae sp.
2202644	GCA_003403395.1	Microviridae sp.
2202644	GCA_003407495.1	Microviridae sp.
2202644	GCA_003405455.1	Microviridae sp.
2202644	GCA_003403415.1	Microviridae sp.
2202644	GCA_003404235.1	Microviridae sp.
2202644	GCA_003407515.1	Microviridae sp.
2202644	GCA_003405475.1	Microviridae sp.
2202644	GCA_003403435.1	Microviridae sp.
2202644	GCA_003407535.1	Microviridae sp.
2202644	GCA_003405495.1	Microviridae sp.
2202644	GCA_003403455.1	Microviridae sp.
2202644	GCA_003407555.1	Microviridae sp.
2202644	GCA_003407315.1	Microviridae sp.
2202644	GCA_003405515.1	Microviridae sp.
2202644	GCA_003406975.1	Microviridae sp.
2202644	GCA_003403475.1	Microviridae sp.
2202644	GCA_003407575.1	Microviridae sp.
2202644	GCA_003406635.1	Microviridae sp.
2202644	GCA_003405535.1	Microviridae sp.
2202644	GCA_003403495.1	Microviridae sp.
2202644	GCA_003407595.1	Microviridae sp.
2202644	GCA_003405555.1	Microviridae sp.
2202644	GCA_003403515.1	Microviridae sp.
2202644	GCA_003407615.1	Microviridae sp.
2202644	GCA_003405575.1	Microviridae sp.
2202644	GCA_003403535.1	Microviridae sp.
2202644	GCA_003404255.1	Microviridae sp.
2202644	GCA_003407635.1	Microviridae sp.
2202644	GCA_003405595.1	Microviridae sp.
2202644	GCA_003403575.1	Microviridae sp.
2202644	GCA_003403555.1	Microviridae sp.
2202644	GCA_003407655.1	Microviridae sp.
2202644	GCA_003405615.1	Microviridae sp.
2202644	GCA_003407675.1	Microviridae sp.
2202644	GCA_003405635.1	Microviridae sp.
2202644	GCA_003403595.1	Microviridae sp.
2202644	GCA_003407695.1	Microviridae sp.
2202644	GCA_003405655.1	Microviridae sp.
2202644	GCA_003406315.1	Microviridae sp.

2202644	GCA_003403615.1	Microviridae sp.
2202644	GCA_003407715.1	Microviridae sp.
2202644	GCA_003405675.1	Microviridae sp.
2202644	GCA_003405295.1	Microviridae sp.
2202644	GCA_003403635.1	Microviridae sp.
2202644	GCA_003407735.1	Microviridae sp.
2202644	GCA_003405695.1	Microviridae sp.
2202644	GCA_003404615.1	Microviridae sp.
2202644	GCA_003403655.1	Microviridae sp.
2202644	GCA_003404275.1	Microviridae sp.
2202644	GCA_003407755.1	Microviridae sp.
2202644	GCA_003405715.1	Microviridae sp.
2202644	GCA_003403675.1	Microviridae sp.
2202644	GCA_003407775.1	Microviridae sp.
2202644	GCA_003405735.1	Microviridae sp.
2202644	GCA_003407795.1	Microviridae sp.
2202644	GCA_003407355.1	Microviridae sp.
2202644	GCA_003405755.1	Microviridae sp.
2202644	GCA_003403715.1	Microviridae sp.
2202644	GCA_003407815.1	Microviridae sp.
2202644	GCA_003405775.1	Microviridae sp.
2202644	GCA_003403735.1	Microviridae sp.
2202644	GCA_003407835.1	Microviridae sp.
2202644	GCA_003405795.1	Microviridae sp.
2202644	GCA_003403755.1	Microviridae sp.
2202644	GCA_003407855.1	Microviridae sp.
2202644	GCA_003405815.1	Microviridae sp.
2202644	GCA_003407875.1	Microviridae sp.
2202644	GCA_003405835.1	Microviridae sp.
2202644	GCA_003403795.1	Microviridae sp.
2202644	GCA_003407895.1	Microviridae sp.
2202644	GCA_003405855.1	Microviridae sp.
2202644	GCA_003403815.1	Microviridae sp.
2202644	GCA_003407915.1	Microviridae sp.
2202644	GCA_003405875.1	Microviridae sp.
2202644	GCA_003403835.1	Microviridae sp.
2202644	GCA_003407935.1	Microviridae sp.
2202644	GCA_003405895.1	Microviridae sp.
2202644	GCA_003403855.1	Microviridae sp.
2202644	GCA_003407955.1	Microviridae sp.
2202644	GCA_003405915.1	Microviridae sp.
2202644	GCA_003407975.1	Microviridae sp.
2202644	GCA_003405935.1	Microviridae sp.
2202644	GCA_003403895.1	Microviridae sp.
2202644	GCA_003407995.1	Microviridae sp.
2202644	GCA_003403975.1	Microviridae sp.
2202644	GCA_003405955.1	Microviridae sp.
2202644	GCA_003403915.1	Microviridae sp.
2202644	GCA_003408015.1	Microviridae sp.
2202644	GCA_003382165.2	Microviridae sp.

2202644	GCA_003405975.1	Microviridae sp.
2202644	GCA_003403935.1	Microviridae sp.
2202644	GCA_003408035.1	Microviridae sp.
2202644	GCA_003382145.2	Microviridae sp.
2202644	GCA_003407395.1	Microviridae sp.
2202644	GCA_003405995.1	Microviridae sp.
2202644	GCA_003403955.1	Microviridae sp.
2202644	GCA_003408055.1	Microviridae sp.
2202644	GCA_003382125.2	Microviridae sp.
2202644	GCA_003406015.1	Microviridae sp.
2202644	GCA_003406375.1	Microviridae sp.
2202644	GCA_003406035.1	Microviridae sp.
2202644	GCA_003408075.1	Microviridae sp.
2202644	GCA_003382105.2	Microviridae sp.
2202644	GCA_003408095.1	Microviridae sp.
2202644	GCA_003382085.2	Microviridae sp.
2202644	GCA_003404015.1	Microviridae sp.
2202644	GCA_003408115.1	Microviridae sp.
2202644	GCA_003382065.2	Microviridae sp.
2202644	GCA_003403995.1	Microviridae sp.
2202644	GCA_003406075.1	Microviridae sp.
2202644	GCA_003404035.1	Microviridae sp.
2202644	GCA_003408135.1	Microviridae sp.
2202644	GCA_003410075.1	Microviridae sp.
2202644	GCA_003382045.2	Microviridae sp.
2202644	GCA_003408155.1	Microviridae sp.
2202644	GCA_003382025.2	Microviridae sp.
2202644	GCA_003407415.1	Microviridae sp.
2202644	GCA_003407075.1	Microviridae sp.
2202644	GCA_003408175.1	Microviridae sp.
2202644	GCA_003406735.1	Microviridae sp.
2202644	GCA_003382005.2	Microviridae sp.
2202644	GCA_003406395.1	Microviridae sp.
2202644	GCA_003406055.1	Microviridae sp.
2202644	GCA_003408195.1	Microviridae sp.
2202644	GCA_003406155.1	Microviridae sp.
2202644	GCA_003408215.1	Microviridae sp.
2202644	GCA_003381965.2	Microviridae sp.
2202644	GCA_003406175.1	Microviridae sp.
2202644	GCA_003404135.1	Microviridae sp.
2202644	GCA_003408235.1	Microviridae sp.
2202644	GCA_003381945.2	Microviridae sp.
2202644	GCA_003406195.1	Microviridae sp.
2202644	GCA_003404155.1	Microviridae sp.
2202644	GCA_003408255.1	Microviridae sp.
2202644	GCA_003406215.1	Microviridae sp.
2202644	GCA_003404175.1	Microviridae sp.
2202644	GCA_003408275.1	Microviridae sp.
2202644	GCA_003404195.1	Microviridae sp.
2202644	GCA_003408295.1	Microviridae sp.

2202644	GCA_003406415.1	Microviridae sp.
2202644	GCA_003408315.1	Microviridae sp.
2202644	GCA_003408335.1	Microviridae sp.
2202644	GCA_003406295.1	Microviridae sp.
2202644	GCA_003404375.1	Microviridae sp.
2202644	GCA_003408355.1	Microviridae sp.
2202644	GCA_003403695.1	Microviridae sp.
2202644	GCA_003408375.1	Microviridae sp.
2202644	GCA_003406335.1	Microviridae sp.
2202644	GCA_003404295.1	Microviridae sp.
2202644	GCA_003408395.1	Microviridae sp.
2202644	GCA_003404315.1	Microviridae sp.
2202644	GCA_003408415.1	Microviridae sp.
2202644	GCA_003406435.1	Microviridae sp.
2202644	GCA_003404335.1	Microviridae sp.
2202644	GCA_003406095.1	Microviridae sp.
2202644	GCA_003408435.1	Microviridae sp.
2202644	GCA_003404355.1	Microviridae sp.
2202644	GCA_003408455.1	Microviridae sp.
2202644	GCA_003404395.1	Microviridae sp.
2202644	GCA_003408475.1	Microviridae sp.
2202644	GCA_003404055.1	Microviridae sp.
2202644	GCA_003723855.1	Microviridae sp.
2202644	GCA_003658685.1	Microviridae sp.
2202644	GCA_003658345.1	Microviridae sp.
2202644	GCA_003659085.2	Microviridae sp.
2202644	GCA_003660065.1	Microviridae sp.
2202644	GCA_003659385.1	Microviridae sp.
2202644	GCA_003723735.1	Microviridae sp.
2202644	GCA_003658345.2	Microviridae sp.
2202644	GCA_003658245.2	Microviridae sp.
2202644	GCA_003658205.2	Microviridae sp.
2202644	GCA_003658385.1	Microviridae sp.
2202644	GCA_003723795.1	Microviridae sp.
2202644	GCA_003658025.2	Microviridae sp.
2202644	GCA_003723535.1	Microviridae sp.
2202644	GCA_003659085.1	Microviridae sp.
2202644	GCA_003657765.2	Microviridae sp.
2202644	GCA_003659165.2	Microviridae sp.
2202644	GCA_003723675.1	Microviridae sp.
2202644	GCA_003723875.1	Microviridae sp.
2202644	GCA_003658505.2	Microviridae sp.
2202644	GCA_003658945.1	Microviridae sp.
2202644	GCA_003657765.1	Microviridae sp.
2202644	GCA_003659485.1	Microviridae sp.
2202644	GCA_003723605.1	Microviridae sp.
2202644	GCA_003659165.1	Microviridae sp.
2202644	GCA_003658825.1	Microviridae sp.
2202644	GCA_003658145.1	Microviridae sp.
2202644	GCA_003659545.1	Microviridae sp.

2202644	GCA_003658125.2	Microviridae sp.
2202644	GCA_003658825.2	Microviridae sp.
2202644	GCA_003657825.2	Microviridae sp.
2202644	GCA_003723555.1	Microviridae sp.
2202644	GCA_003657985.2	Microviridae sp.
2202644	GCA_003658505.1	Microviridae sp.
2202644	GCA_003657745.2	Microviridae sp.
2202644	GCA_003723815.1	Microviridae sp.
2202644	GCA_003723695.1	Microviridae sp.
2202644	GCA_003658945.2	Microviridae sp.
2202644	GCA_003658685.2	Microviridae sp.
2202644	GCA_003657845.2	Microviridae sp.
2202644	GCA_003659485.2	Microviridae sp.
2202644	GCA_003658525.1	Microviridae sp.
2202644	GCA_003657845.1	Microviridae sp.
2202644	GCA_003723835.1	Microviridae sp.
2202644	GCA_003658525.2	Microviridae sp.
2202644	GCA_003659545.2	Microviridae sp.
2202644	GCA_003658385.2	Microviridae sp.
2202644	GCA_003658565.1	Microviridae sp.
2202644	GCA_003723775.1	Microviridae sp.
2202644	GCA_003657745.1	Microviridae sp.
2202644	GCA_003658925.1	Microviridae sp.
2202644	GCA_003658585.1	Microviridae sp.
2202644	GCA_003657785.1	Microviridae sp.
2202644	GCA_003658105.2	Microviridae sp.
2202644	GCA_003723915.1	Microviridae sp.
2202644	GCA_003723715.1	Microviridae sp.
2202644	GCA_003657825.1	Microviridae sp.
2202644	GCA_003659285.2	Microviridae sp.
2202644	GCA_003659285.1	Microviridae sp.
2202644	GCA_003658925.2	Microviridae sp.
2202644	GCA_003723515.1	Microviridae sp.
2202644	GCA_003658025.1	Microviridae sp.
2202644	GCA_003723755.1	Microviridae sp.
2202644	GCA_003723625.1	Microviridae sp.
2202644	GCA_003658105.1	Microviridae sp.
2202644	GCA_003658125.1	Microviridae sp.
2202644	GCA_003657785.2	Microviridae sp.
2202644	GCA_003659385.2	Microviridae sp.
2202644	GCA_003723655.1	Microviridae sp.
2202644	GCA_003658205.1	Microviridae sp.
2202644	GCA_003723895.1	Microviridae sp.
2202644	GCA_003723575.1	Microviridae sp.
2202644	GCA_003660065.2	Microviridae sp.
2202644	GCA_003658245.1	Microviridae sp.
2202644	GCA_003657985.1	Microviridae sp.
2202644	GCA_003658585.2	Microviridae sp.
2202644	GCA_003826095.1	Microviridae sp.
2202644	GCA_003824055.1	Microviridae sp.

2202644	GCA_003844075.1	Microviridae sp.
2202644	GCA_003844055.1	Microviridae sp.
2202644	GCA_003828155.1	Microviridae sp.
2202644	GCA_003829495.1	Microviridae sp.
2202644	GCA_003826115.1	Microviridae sp.
2202644	GCA_003825075.1	Microviridae sp.
2202644	GCA_003824075.1	Microviridae sp.
2202644	GCA_003845515.1	Microviridae sp.
2202644	GCA_003832715.1	Microviridae sp.
2202644	GCA_003828175.1	Microviridae sp.
2202644	GCA_003824735.1	Microviridae sp.
2202644	GCA_003829515.1	Microviridae sp.
2202644	GCA_003826135.1	Microviridae sp.
2202644	GCA_003824095.1	Microviridae sp.
2202644	GCA_003844435.1	Microviridae sp.
2202644	GCA_003828195.1	Microviridae sp.
2202644	GCA_003844735.1	Microviridae sp.
2202644	GCA_003829535.1	Microviridae sp.
2202644	GCA_003826155.1	Microviridae sp.
2202644	GCA_003844395.1	Microviridae sp.
2202644	GCA_003824115.1	Microviridae sp.
2202644	GCA_003844455.1	Microviridae sp.
2202644	GCA_003828215.1	Microviridae sp.
2202644	GCA_003829555.1	Microviridae sp.
2202644	GCA_003845935.1	Microviridae sp.
2202644	GCA_003826175.1	Microviridae sp.
2202644	GCA_003822115.1	Microviridae sp.
2202644	GCA_003824135.1	Microviridae sp.
2202644	GCA_003817975.1	Microviridae sp.
2202644	GCA_003832735.1	Microviridae sp.
2202644	GCA_003828235.1	Microviridae sp.
2202644	GCA_003829575.1	Microviridae sp.
2202644	GCA_003827135.1	Microviridae sp.
2202644	GCA_003826195.1	Microviridae sp.
2202644	GCA_003826795.1	Microviridae sp.
2202644	GCA_003824155.1	Microviridae sp.
2202644	GCA_003832755.1	Microviridae sp.
2202644	GCA_003828255.1	Microviridae sp.
2202644	GCA_003826215.1	Microviridae sp.
2202644	GCA_003824175.1	Microviridae sp.
2202644	GCA_003844095.1	Microviridae sp.
2202644	GCA_003832775.1	Microviridae sp.
2202644	GCA_003828275.1	Microviridae sp.
2202644	GCA_003829595.1	Microviridae sp.
2202644	GCA_003826235.1	Microviridae sp.
2202644	GCA_003825095.1	Microviridae sp.
2202644	GCA_003824195.1	Microviridae sp.
2202644	GCA_003832795.1	Microviridae sp.
2202644	GCA_003828295.1	Microviridae sp.
2202644	GCA_003816415.1	Microviridae sp.

2202644	GCA_003829615.1	Microviridae sp.
2202644	GCA_003822155.1	Microviridae sp.
2202644	GCA_003826255.1	Microviridae sp.
2202644	GCA_003844895.1	Microviridae sp.
2202644	GCA_003824215.1	Microviridae sp.
2202644	GCA_003832815.1	Microviridae sp.
2202644	GCA_003828315.1	Microviridae sp.
2202644	GCA_003844755.1	Microviridae sp.
2202644	GCA_003829635.1	Microviridae sp.
2202644	GCA_003826275.1	Microviridae sp.
2202644	GCA_003844415.1	Microviridae sp.
2202644	GCA_003824235.1	Microviridae sp.
2202644	GCA_003844575.1	Microviridae sp.
2202644	GCA_003828335.1	Microviridae sp.
2202644	GCA_003829655.1	Microviridae sp.
2202644	GCA_003826295.1	Microviridae sp.
2202644	GCA_003845815.1	Microviridae sp.
2202644	GCA_003824255.1	Microviridae sp.
2202644	GCA_003822375.1	Microviridae sp.
2202644	GCA_003832835.1	Microviridae sp.
2202644	GCA_003828355.1	Microviridae sp.
2202644	GCA_003822035.1	Microviridae sp.
2202644	GCA_003826315.1	Microviridae sp.
2202644	GCA_003826815.1	Microviridae sp.
2202644	GCA_003824275.1	Microviridae sp.
2202644	GCA_003844615.1	Microviridae sp.
2202644	GCA_003828375.1	Microviridae sp.
2202644	GCA_003822235.1	Microviridae sp.
2202644	GCA_003826335.1	Microviridae sp.
2202644	GCA_003824295.1	Microviridae sp.
2202644	GCA_003844115.1	Microviridae sp.
2202644	GCA_003844635.1	Microviridae sp.
2202644	GCA_003828395.1	Microviridae sp.
2202644	GCA_003829675.1	Microviridae sp.
2202644	GCA_003822255.1	Microviridae sp.
2202644	GCA_003826355.1	Microviridae sp.
2202644	GCA_003825115.1	Microviridae sp.
2202644	GCA_003824315.1	Microviridae sp.
2202644	GCA_003832855.1	Microviridae sp.
2202644	GCA_003828415.1	Microviridae sp.
2202644	GCA_003816435.1	Microviridae sp.
2202644	GCA_003829695.1	Microviridae sp.
2202644	GCA_003826375.1	Microviridae sp.
2202644	GCA_003824435.1	Microviridae sp.
2202644	GCA_003824335.1	Microviridae sp.
2202644	GCA_003832875.1	Microviridae sp.
2202644	GCA_003828435.1	Microviridae sp.
2202644	GCA_003844775.1	Microviridae sp.
2202644	GCA_003829715.1	Microviridae sp.
2202644	GCA_003822295.1	Microviridae sp.

2202644	GCA_003844155.1	Microviridae sp.
2202644	GCA_003826395.1	Microviridae sp.
2202644	GCA_003817515.1	Microviridae sp.
2202644	GCA_003824355.1	Microviridae sp.
2202644	GCA_003832895.1	Microviridae sp.
2202644	GCA_003828455.1	Microviridae sp.
2202644	GCA_003829735.1	Microviridae sp.
2202644	GCA_003822315.1	Microviridae sp.
2202644	GCA_003826415.1	Microviridae sp.
2202644	GCA_003824375.1	Microviridae sp.
2202644	GCA_003844715.1	Microviridae sp.
2202644	GCA_003828475.1	Microviridae sp.
2202644	GCA_003822335.1	Microviridae sp.
2202644	GCA_003822055.1	Microviridae sp.
2202644	GCA_003826435.1	Microviridae sp.
2202644	GCA_003824395.1	Microviridae sp.
2202644	GCA_003832915.1	Microviridae sp.
2202644	GCA_003828495.1	Microviridae sp.
2202644	GCA_003826455.1	Microviridae sp.
2202644	GCA_003844135.1	Microviridae sp.
2202644	GCA_003816075.1	Microviridae sp.
2202644	GCA_003832935.1	Microviridae sp.
2202644	GCA_003828515.1	Microviridae sp.
2202644	GCA_003826475.1	Microviridae sp.
2202644	GCA_003825135.1	Microviridae sp.
2202644	GCA_003816095.1	Microviridae sp.
2202644	GCA_003832955.1	Microviridae sp.
2202644	GCA_003828535.1	Microviridae sp.
2202644	GCA_003822395.1	Microviridae sp.
2202644	GCA_003826495.1	Microviridae sp.
2202644	GCA_003824455.1	Microviridae sp.
2202644	GCA_003816115.1	Microviridae sp.
2202644	GCA_003832975.1	Microviridae sp.
2202644	GCA_003828555.1	Microviridae sp.
2202644	GCA_003826515.1	Microviridae sp.
2202644	GCA_003824475.1	Microviridae sp.
2202644	GCA_003828575.1	Microviridae sp.
2202644	GCA_003822435.1	Microviridae sp.
2202644	GCA_003826535.1	Microviridae sp.
2202644	GCA_003845855.1	Microviridae sp.
2202644	GCA_003824495.1	Microviridae sp.
2202644	GCA_003822415.1	Microviridae sp.
2202644	GCA_003828595.1	Microviridae sp.
2202644	GCA_003822075.1	Microviridae sp.
2202644	GCA_003826555.1	Microviridae sp.
2202644	GCA_003817355.1	Microviridae sp.
2202644	GCA_003824515.1	Microviridae sp.
2202644	GCA_003828615.1	Microviridae sp.
2202644	GCA_003826575.1	Microviridae sp.
2202644	GCA_003816195.1	Microviridae sp.

2202644	GCA_003827215.1	Microviridae sp.
2202644	GCA_003828635.1	Microviridae sp.
2202644	GCA_003822495.1	Microviridae sp.
2202644	GCA_003826595.1	Microviridae sp.
2202644	GCA_003825155.1	Microviridae sp.
2202644	GCA_003816215.1	Microviridae sp.
2202644	GCA_003828655.1	Microviridae sp.
2202644	GCA_003816475.1	Microviridae sp.
2202644	GCA_003817115.1	Microviridae sp.
2202644	GCA_003816135.1	Microviridae sp.
2202644	GCA_003824575.1	Microviridae sp.
2202644	GCA_003828675.1	Microviridae sp.
2202644	GCA_003822535.1	Microviridae sp.
2202644	GCA_003817135.1	Microviridae sp.
2202644	GCA_003844475.1	Microviridae sp.
2202644	GCA_003816255.1	Microviridae sp.
2202644	GCA_003828695.1	Microviridae sp.
2202644	GCA_003822555.1	Microviridae sp.
2202644	GCA_003826655.1	Microviridae sp.
2202644	GCA_003845875.1	Microviridae sp.
2202644	GCA_003824615.1	Microviridae sp.
2202644	GCA_003827555.1	Microviridae sp.
2202644	GCA_003828715.1	Microviridae sp.
2202644	GCA_003822575.1	Microviridae sp.
2202644	GCA_003822095.1	Microviridae sp.
2202644	GCA_003817175.1	Microviridae sp.
2202644	GCA_003817375.1	Microviridae sp.
2202644	GCA_003816295.1	Microviridae sp.
2202644	GCA_003828735.1	Microviridae sp.
2202644	GCA_003822595.1	Microviridae sp.
2202644	GCA_003824655.1	Microviridae sp.
2202644	GCA_003844795.1	Microviridae sp.
2202644	GCA_003822615.1	Microviridae sp.
2202644	GCA_003825515.1	Microviridae sp.
2202644	GCA_003826715.1	Microviridae sp.
2202644	GCA_003816835.1	Microviridae sp.
2202644	GCA_003831075.1	Microviridae sp.
2202644	GCA_003816335.1	Microviridae sp.
2202644	GCA_003844815.1	Microviridae sp.
2202644	GCA_003816495.1	Microviridae sp.
2202644	GCA_003822635.1	Microviridae sp.
2202644	GCA_003817235.1	Microviridae sp.
2202644	GCA_003816155.1	Microviridae sp.
2202644	GCA_003831095.1	Microviridae sp.
2202644	GCA_003824695.1	Microviridae sp.
2202644	GCA_003844835.1	Microviridae sp.
2202644	GCA_003817255.1	Microviridae sp.
2202644	GCA_003817595.1	Microviridae sp.
2202644	GCA_003823475.1	Microviridae sp.
2202644	GCA_003824715.1	Microviridae sp.

2202644	GCA_003844855.1	Microviridae sp.
2202644	GCA_003822675.1	Microviridae sp.
2202644	GCA_003826775.1	Microviridae sp.
2202644	GCA_003845895.1	Microviridae sp.
2202644	GCA_003831115.1	Microviridae sp.
2202644	GCA_003816395.1	Microviridae sp.
2202644	GCA_003827575.1	Microviridae sp.
2202644	GCA_003844875.1	Microviridae sp.
2202644	GCA_003822695.1	Microviridae sp.
2202644	GCA_003817735.1	Microviridae sp.
2202644	GCA_003817395.1	Microviridae sp.
2202644	GCA_003831135.1	Microviridae sp.
2202644	GCA_003824755.1	Microviridae sp.
2202644	GCA_003821935.1	Microviridae sp.
2202644	GCA_003822715.1	Microviridae sp.
2202644	GCA_003817315.1	Microviridae sp.
2202644	GCA_003831155.1	Microviridae sp.
2202644	GCA_003824775.1	Microviridae sp.
2202644	GCA_003844915.1	Microviridae sp.
2202644	GCA_003827155.1	Microviridae sp.
2202644	GCA_003822735.1	Microviridae sp.
2202644	GCA_003826835.1	Microviridae sp.
2202644	GCA_003820695.1	Microviridae sp.
2202644	GCA_003825195.1	Microviridae sp.
2202644	GCA_003831175.1	Microviridae sp.
2202644	GCA_003824795.1	Microviridae sp.
2202644	GCA_003844935.1	Microviridae sp.
2202644	GCA_003816515.1	Microviridae sp.
2202644	GCA_003822755.1	Microviridae sp.
2202644	GCA_003820715.1	Microviridae sp.
2202644	GCA_003822215.1	Microviridae sp.
2202644	GCA_003831195.1	Microviridae sp.
2202644	GCA_003824815.1	Microviridae sp.
2202644	GCA_003844955.1	Microviridae sp.
2202644	GCA_003826675.1	Microviridae sp.
2202644	GCA_003822775.1	Microviridae sp.
2202644	GCA_003826875.1	Microviridae sp.
2202644	GCA_003844515.1	Microviridae sp.
2202644	GCA_003820735.1	Microviridae sp.
2202644	GCA_003831215.1	Microviridae sp.
2202644	GCA_003824835.1	Microviridae sp.
2202644	GCA_003844975.1	Microviridae sp.
2202644	GCA_003822795.1	Microviridae sp.
2202644	GCA_003826895.1	Microviridae sp.
2202644	GCA_003820755.1	Microviridae sp.
2202644	GCA_003831235.1	Microviridae sp.
2202644	GCA_003824855.1	Microviridae sp.
2202644	GCA_003822475.1	Microviridae sp.
2202644	GCA_003844995.1	Microviridae sp.
2202644	GCA_003818715.1	Microviridae sp.

2202644	GCA_003822815.1	Microviridae sp.
2202644	GCA_003822135.1	Microviridae sp.
2202644	GCA_003826915.1	Microviridae sp.
2202644	GCA_003820775.1	Microviridae sp.
2202644	GCA_003817415.1	Microviridae sp.
2202644	GCA_003831255.1	Microviridae sp.
2202644	GCA_003816535.1	Microviridae sp.
2202644	GCA_003845015.1	Microviridae sp.
2202644	GCA_003818735.1	Microviridae sp.
2202644	GCA_003826935.1	Microviridae sp.
2202644	GCA_003827295.1	Microviridae sp.
2202644	GCA_003820795.1	Microviridae sp.
2202644	GCA_003831275.1	Microviridae sp.
2202644	GCA_003824895.1	Microviridae sp.
2202644	GCA_003843995.1	Microviridae sp.
2202644	GCA_003818755.1	Microviridae sp.
2202644	GCA_003822855.1	Microviridae sp.
2202644	GCA_003844015.1	Microviridae sp.
2202644	GCA_003826955.1	Microviridae sp.
2202644	GCA_003825215.1	Microviridae sp.
2202644	GCA_003816375.1	Microviridae sp.
2202644	GCA_003824915.1	Microviridae sp.
2202644	GCA_003845055.1	Microviridae sp.
2202644	GCA_003818775.1	Microviridae sp.
2202644	GCA_003824875.1	Microviridae sp.
2202644	GCA_003822875.1	Microviridae sp.
2202644	GCA_003817875.1	Microviridae sp.
2202644	GCA_003819415.1	Microviridae sp.
2202644	GCA_003820835.1	Microviridae sp.
2202644	GCA_003831295.1	Microviridae sp.
2202644	GCA_003824935.1	Microviridae sp.
2202644	GCA_003845075.1	Microviridae sp.
2202644	GCA_003827235.1	Microviridae sp.
2202644	GCA_003818795.1	Microviridae sp.
2202644	GCA_003822895.1	Microviridae sp.
2202644	GCA_003826995.1	Microviridae sp.
2202644	GCA_003844535.1	Microviridae sp.
2202644	GCA_003820855.1	Microviridae sp.
2202644	GCA_003823515.1	Microviridae sp.
2202644	GCA_003831315.1	Microviridae sp.
2202644	GCA_003824955.1	Microviridae sp.
2202644	GCA_003845095.1	Microviridae sp.
2202644	GCA_003818815.1	Microviridae sp.
2202644	GCA_003822915.1	Microviridae sp.
2202644	GCA_003827015.1	Microviridae sp.
2202644	GCA_003822835.1	Microviridae sp.
2202644	GCA_003820875.1	Microviridae sp.
2202644	GCA_003831335.1	Microviridae sp.
2202644	GCA_003816635.1	Microviridae sp.
2202644	GCA_003845115.1	Microviridae sp.

2202644	GCA_003818835.1	Microviridae sp.
2202644	GCA_003822935.1	Microviridae sp.
2202644	GCA_003827035.1	Microviridae sp.
2202644	GCA_003820895.1	Microviridae sp.
2202644	GCA_003817435.1	Microviridae sp.
2202644	GCA_003831355.1	Microviridae sp.
2202644	GCA_003824995.1	Microviridae sp.
2202644	GCA_003845135.1	Microviridae sp.
2202644	GCA_003827435.1	Microviridae sp.
2202644	GCA_003818855.1	Microviridae sp.
2202644	GCA_003822955.1	Microviridae sp.
2202644	GCA_003817555.1	Microviridae sp.
2202644	GCA_003820915.1	Microviridae sp.
2202644	GCA_003831375.1	Microviridae sp.
2202644	GCA_003825015.1	Microviridae sp.
2202644	GCA_003844215.1	Microviridae sp.
2202644	GCA_003845155.1	Microviridae sp.
2202644	GCA_003818875.1	Microviridae sp.
2202644	GCA_003844375.1	Microviridae sp.
2202644	GCA_003822975.1	Microviridae sp.
2202644	GCA_003827075.1	Microviridae sp.
2202644	GCA_003820935.1	Microviridae sp.
2202644	GCA_003825235.1	Microviridae sp.
2202644	GCA_003831395.1	Microviridae sp.
2202644	GCA_003825035.1	Microviridae sp.
2202644	GCA_003845175.1	Microviridae sp.
2202644	GCA_003818895.1	Microviridae sp.
2202644	GCA_003816555.1	Microviridae sp.
2202644	GCA_003822995.1	Microviridae sp.
2202644	GCA_003816315.1	Microviridae sp.
2202644	GCA_003827095.1	Microviridae sp.
2202644	GCA_003824555.1	Microviridae sp.
2202644	GCA_003816175.1	Microviridae sp.
2202644	GCA_003845035.1	Microviridae sp.
2202644	GCA_003831415.1	Microviridae sp.
2202644	GCA_003825055.1	Microviridae sp.
2202644	GCA_003845195.1	Microviridae sp.
2202644	GCA_003818915.1	Microviridae sp.
2202644	GCA_003817675.1	Microviridae sp.
2202644	GCA_003823015.1	Microviridae sp.
2202644	GCA_003824415.1	Microviridae sp.
2202644	GCA_003827495.1	Microviridae sp.
2202644	GCA_003827115.1	Microviridae sp.
2202644	GCA_003844555.1	Microviridae sp.
2202644	GCA_003820975.1	Microviridae sp.
2202644	GCA_003831435.1	Microviridae sp.
2202644	GCA_003816735.1	Microviridae sp.
2202644	GCA_003845215.1	Microviridae sp.
2202644	GCA_003818935.1	Microviridae sp.
2202644	GCA_003823035.1	Microviridae sp.

2202644	GCA_003817635.1	Microviridae sp.
2202644	GCA_003845955.1	Microviridae sp.
2202644	GCA_003820995.1	Microviridae sp.
2202644	GCA_003817335.1	Microviridae sp.
2202644	GCA_003831455.1	Microviridae sp.
2202644	GCA_003816755.1	Microviridae sp.
2202644	GCA_003822515.1	Microviridae sp.
2202644	GCA_003818955.1	Microviridae sp.
2202644	GCA_003823055.1	Microviridae sp.
2202644	GCA_003822175.1	Microviridae sp.
2202644	GCA_003817655.1	Microviridae sp.
2202644	GCA_003821015.1	Microviridae sp.
2202644	GCA_003817455.1	Microviridae sp.
2202644	GCA_003831475.1	Microviridae sp.
2202644	GCA_003821915.1	Microviridae sp.
2202644	GCA_003816775.1	Microviridae sp.
2202644	GCA_003818975.1	Microviridae sp.
2202644	GCA_003826615.1	Microviridae sp.
2202644	GCA_003823075.1	Microviridae sp.
2202644	GCA_003827175.1	Microviridae sp.
2202644	GCA_003821035.1	Microviridae sp.
2202644	GCA_003816795.1	Microviridae sp.
2202644	GCA_003820815.1	Microviridae sp.
2202644	GCA_003845235.1	Microviridae sp.
2202644	GCA_003818995.1	Microviridae sp.
2202644	GCA_003831575.1	Microviridae sp.
2202644	GCA_003823095.1	Microviridae sp.
2202644	GCA_003843915.1	Microviridae sp.
2202644	GCA_003827195.1	Microviridae sp.
2202644	GCA_003821055.1	Microviridae sp.
2202644	GCA_003816915.1	Microviridae sp.
2202644	GCA_003831495.1	Microviridae sp.
2202644	GCA_003816815.1	Microviridae sp.
2202644	GCA_003845255.1	Microviridae sp.
2202644	GCA_003819015.1	Microviridae sp.
2202644	GCA_003816575.1	Microviridae sp.
2202644	GCA_003823115.1	Microviridae sp.
2202644	GCA_003817715.1	Microviridae sp.
2202644	GCA_003816235.1	Microviridae sp.
2202644	GCA_003821075.1	Microviridae sp.
2202644	GCA_003831515.1	Microviridae sp.
2202644	GCA_003825175.1	Microviridae sp.
2202644	GCA_003819115.1	Microviridae sp.
2202644	GCA_003845275.1	Microviridae sp.
2202644	GCA_003819035.1	Microviridae sp.
2202644	GCA_003823135.1	Microviridae sp.
2202644	GCA_003821095.1	Microviridae sp.
2202644	GCA_003816855.1	Microviridae sp.
2202644	GCA_003845295.1	Microviridae sp.
2202644	GCA_003819055.1	Microviridae sp.

2202644	GCA_003817475.1	Microviridae sp.
2202644	GCA_003823155.1	Microviridae sp.
2202644	GCA_003827255.1	Microviridae sp.
2202644	GCA_003845975.1	Microviridae sp.
2202644	GCA_003821115.1	Microviridae sp.
2202644	GCA_003831535.1	Microviridae sp.
2202644	GCA_003816875.1	Microviridae sp.
2202644	GCA_003845315.1	Microviridae sp.
2202644	GCA_003819075.1	Microviridae sp.
2202644	GCA_003823175.1	Microviridae sp.
2202644	GCA_003827315.1	Microviridae sp.
2202644	GCA_003827275.1	Microviridae sp.
2202644	GCA_003821135.1	Microviridae sp.
2202644	GCA_003826975.1	Microviridae sp.
2202644	GCA_003831555.1	Microviridae sp.
2202644	GCA_003816895.1	Microviridae sp.
2202644	GCA_003845335.1	Microviridae sp.
2202644	GCA_003819095.1	Microviridae sp.
2202644	GCA_003826635.1	Microviridae sp.
2202644	GCA_003823195.1	Microviridae sp.
2202644	GCA_003817795.1	Microviridae sp.
2202644	GCA_003828755.1	Microviridae sp.
2202644	GCA_003821155.1	Microviridae sp.
2202644	GCA_003825255.1	Microviridae sp.
2202644	GCA_003844255.1	Microviridae sp.
2202644	GCA_003845355.1	Microviridae sp.
2202644	GCA_003823215.1	Microviridae sp.
2202644	GCA_003843935.1	Microviridae sp.
2202644	GCA_003817815.1	Microviridae sp.
2202644	GCA_003816455.1	Microviridae sp.
2202644	GCA_003828775.1	Microviridae sp.
2202644	GCA_003821175.1	Microviridae sp.
2202644	GCA_003825275.1	Microviridae sp.
2202644	GCA_003816935.1	Microviridae sp.
2202644	GCA_003845375.1	Microviridae sp.
2202644	GCA_003819135.1	Microviridae sp.
2202644	GCA_003816595.1	Microviridae sp.
2202644	GCA_003823235.1	Microviridae sp.
2202644	GCA_003817835.1	Microviridae sp.
2202644	GCA_003824595.1	Microviridae sp.
2202644	GCA_003844595.1	Microviridae sp.
2202644	GCA_003821195.1	Microviridae sp.
2202644	GCA_003816955.1	Microviridae sp.
2202644	GCA_003845395.1	Microviridae sp.
2202644	GCA_003819155.1	Microviridae sp.
2202644	GCA_003823255.1	Microviridae sp.
2202644	GCA_003817855.1	Microviridae sp.
2202644	GCA_003817615.1	Microviridae sp.
2202644	GCA_003828795.1	Microviridae sp.
2202644	GCA_003821215.1	Microviridae sp.

2202644	GCA_003831595.1	Microviridae sp.
2202644	GCA_003816975.1	Microviridae sp.
2202644	GCA_003845415.1	Microviridae sp.
2202644	GCA_003819175.1	Microviridae sp.
2202644	GCA_003823275.1	Microviridae sp.
2202644	GCA_003832075.1	Microviridae sp.
2202644	GCA_003827375.1	Microviridae sp.
2202644	GCA_003845995.1	Microviridae sp.
2202644	GCA_003828815.1	Microviridae sp.
2202644	GCA_003821235.1	Microviridae sp.
2202644	GCA_003831615.1	Microviridae sp.
2202644	GCA_003816995.1	Microviridae sp.
2202644	GCA_003845435.1	Microviridae sp.
2202644	GCA_003823295.1	Microviridae sp.
2202644	GCA_003827335.1	Microviridae sp.
2202644	GCA_003832095.1	Microviridae sp.
2202644	GCA_003817895.1	Microviridae sp.
2202644	GCA_003828835.1	Microviridae sp.
2202644	GCA_003821255.1	Microviridae sp.
2202644	GCA_003827515.1	Microviridae sp.
2202644	GCA_003817015.1	Microviridae sp.
2202644	GCA_003845455.1	Microviridae sp.
2202644	GCA_003817155.1	Microviridae sp.
2202644	GCA_003823315.1	Microviridae sp.
2202644	GCA_003832115.1	Microviridae sp.
2202644	GCA_003817915.1	Microviridae sp.
2202644	GCA_003821275.1	Microviridae sp.
2202644	GCA_003831655.1	Microviridae sp.
2202644	GCA_003817035.1	Microviridae sp.
2202644	GCA_003845475.1	Microviridae sp.
2202644	GCA_003823335.1	Microviridae sp.
2202644	GCA_003843955.1	Microviridae sp.
2202644	GCA_003832135.1	Microviridae sp.
2202644	GCA_003817935.1	Microviridae sp.
2202644	GCA_003828855.1	Microviridae sp.
2202644	GCA_003821295.1	Microviridae sp.
2202644	GCA_003825295.1	Microviridae sp.
2202644	GCA_003831675.1	Microviridae sp.
2202644	GCA_003817055.1	Microviridae sp.
2202644	GCA_003845495.1	Microviridae sp.
2202644	GCA_003819255.1	Microviridae sp.
2202644	GCA_003816615.1	Microviridae sp.
2202644	GCA_003823355.1	Microviridae sp.
2202644	GCA_003827455.1	Microviridae sp.
2202644	GCA_003816275.1	Microviridae sp.
2202644	GCA_003828875.1	Microviridae sp.
2202644	GCA_003821315.1	Microviridae sp.
2202644	GCA_003831695.1	Microviridae sp.
2202644	GCA_003825415.1	Microviridae sp.
2202644	GCA_003817755.1	Microviridae sp.

2202644	GCA_003819275.1	Microviridae sp.
2202644	GCA_003821955.1	Microviridae sp.
2202644	GCA_003823375.1	Microviridae sp.
2202644	GCA_003832155.1	Microviridae sp.
2202644	GCA_003827475.1	Microviridae sp.
2202644	GCA_003828895.1	Microviridae sp.
2202644	GCA_003821335.1	Microviridae sp.
2202644	GCA_003825435.1	Microviridae sp.
2202644	GCA_003844275.1	Microviridae sp.
2202644	GCA_003845535.1	Microviridae sp.
2202644	GCA_003819295.1	Microviridae sp.
2202644	GCA_003823395.1	Microviridae sp.
2202644	GCA_003832175.1	Microviridae sp.
2202644	GCA_003817995.1	Microviridae sp.
2202644	GCA_003846015.1	Microviridae sp.
2202644	GCA_003828915.1	Microviridae sp.
2202644	GCA_003821355.1	Microviridae sp.
2202644	GCA_003831715.1	Microviridae sp.
2202644	GCA_003825455.1	Microviridae sp.
2202644	GCA_003845555.1	Microviridae sp.
2202644	GCA_003819315.1	Microviridae sp.
2202644	GCA_003823415.1	Microviridae sp.
2202644	GCA_003827355.1	Microviridae sp.
2202644	GCA_003832195.1	Microviridae sp.
2202644	GCA_003818015.1	Microviridae sp.
2202644	GCA_003828935.1	Microviridae sp.
2202644	GCA_003821375.1	Microviridae sp.
2202644	GCA_003821895.1	Microviridae sp.
2202644	GCA_003825475.1	Microviridae sp.
2202644	GCA_003845575.1	Microviridae sp.
2202644	GCA_003819335.1	Microviridae sp.
2202644	GCA_003823435.1	Microviridae sp.
2202644	GCA_003832215.1	Microviridae sp.
2202644	GCA_003818035.1	Microviridae sp.
2202644	GCA_003828955.1	Microviridae sp.
2202644	GCA_003821395.1	Microviridae sp.
2202644	GCA_003831735.1	Microviridae sp.
2202644	GCA_003825495.1	Microviridae sp.
2202644	GCA_003845595.1	Microviridae sp.
2202644	GCA_003823455.1	Microviridae sp.
2202644	GCA_003825655.1	Microviridae sp.
2202644	GCA_003832235.1	Microviridae sp.
2202644	GCA_003818055.1	Microviridae sp.
2202644	GCA_003828975.1	Microviridae sp.
2202644	GCA_003821415.1	Microviridae sp.
2202644	GCA_003825315.1	Microviridae sp.
2202644	GCA_003831755.1	Microviridae sp.
2202644	GCA_003817695.1	Microviridae sp.
2202644	GCA_003845615.1	Microviridae sp.
2202644	GCA_003819375.1	Microviridae sp.

2202644	GCA_003824975.1	Microviridae sp.
2202644	GCA_003818075.1	Microviridae sp.
2202644	GCA_003824635.1	Microviridae sp.
2202644	GCA_003828995.1	Microviridae sp.
2202644	GCA_003821435.1	Microviridae sp.
2202644	GCA_003825535.1	Microviridae sp.
2202644	GCA_003845635.1	Microviridae sp.
2202644	GCA_003819395.1	Microviridae sp.
2202644	GCA_003823495.1	Microviridae sp.
2202644	GCA_003832255.1	Microviridae sp.
2202644	GCA_003818095.1	Microviridae sp.
2202644	GCA_003829015.1	Microviridae sp.
2202644	GCA_003821455.1	Microviridae sp.
2202644	GCA_003831775.1	Microviridae sp.
2202644	GCA_003825555.1	Microviridae sp.
2202644	GCA_003844295.1	Microviridae sp.
2202644	GCA_003845655.1	Microviridae sp.
2202644	GCA_003832275.1	Microviridae sp.
2202644	GCA_003827615.1	Microviridae sp.
2202644	GCA_003846035.1	Microviridae sp.
2202644	GCA_003821475.1	Microviridae sp.
2202644	GCA_003831795.1	Microviridae sp.
2202644	GCA_003825575.1	Microviridae sp.
2202644	GCA_003845675.1	Microviridae sp.
2202644	GCA_003819435.1	Microviridae sp.
2202644	GCA_003823535.1	Microviridae sp.
2202644	GCA_003832295.1	Microviridae sp.
2202644	GCA_003827635.1	Microviridae sp.
2202644	GCA_003821495.1	Microviridae sp.
2202644	GCA_003817535.1	Microviridae sp.
2202644	GCA_003831815.1	Microviridae sp.
2202644	GCA_003825595.1	Microviridae sp.
2202644	GCA_003845695.1	Microviridae sp.
2202644	GCA_003819455.1	Microviridae sp.
2202644	GCA_003817195.1	Microviridae sp.
2202644	GCA_003823555.1	Microviridae sp.
2202644	GCA_003832315.1	Microviridae sp.
2202644	GCA_003827655.1	Microviridae sp.
2202644	GCA_003829035.1	Microviridae sp.
2202644	GCA_003821515.1	Microviridae sp.
2202644	GCA_003831835.1	Microviridae sp.
2202644	GCA_003825615.1	Microviridae sp.
2202644	GCA_003845715.1	Microviridae sp.
2202644	GCA_003819475.1	Microviridae sp.
2202644	GCA_003823575.1	Microviridae sp.
2202644	GCA_003827675.1	Microviridae sp.
2202644	GCA_003829055.1	Microviridae sp.
2202644	GCA_003821535.1	Microviridae sp.
2202644	GCA_003825335.1	Microviridae sp.
2202644	GCA_003831855.1	Microviridae sp.

2202644	GCA_003825635.1	Microviridae sp.
2202644	GCA_003845735.1	Microviridae sp.
2202644	GCA_003819495.1	Microviridae sp.
2202644	GCA_003816655.1	Microviridae sp.
2202644	GCA_003823595.1	Microviridae sp.
2202644	GCA_003832335.1	Microviridae sp.
2202644	GCA_003827695.1	Microviridae sp.
2202644	GCA_003829075.1	Microviridae sp.
2202644	GCA_003821555.1	Microviridae sp.
2202644	GCA_003831875.1	Microviridae sp.
2202644	GCA_003819195.1	Microviridae sp.
2202644	GCA_003845755.1	Microviridae sp.
2202644	GCA_003819515.1	Microviridae sp.
2202644	GCA_003823615.1	Microviridae sp.
2202644	GCA_003832355.1	Microviridae sp.
2202644	GCA_003827715.1	Microviridae sp.
2202644	GCA_003844655.1	Microviridae sp.
2202644	GCA_003829095.1	Microviridae sp.
2202644	GCA_003821575.1	Microviridae sp.
2202644	GCA_003831895.1	Microviridae sp.
2202644	GCA_003825675.1	Microviridae sp.
2202644	GCA_003844315.1	Microviridae sp.
2202644	GCA_003845775.1	Microviridae sp.
2202644	GCA_003819535.1	Microviridae sp.
2202644	GCA_003823635.1	Microviridae sp.
2202644	GCA_003832375.1	Microviridae sp.
2202644	GCA_003827755.1	Microviridae sp.
2202644	GCA_003846055.1	Microviridae sp.
2202644	GCA_003829115.1	Microviridae sp.
2202644	GCA_003821595.1	Microviridae sp.
2202644	GCA_003831915.1	Microviridae sp.
2202644	GCA_003825695.1	Microviridae sp.
2202644	GCA_003829435.1	Microviridae sp.
2202644	GCA_003822275.1	Microviridae sp.
2202644	GCA_003832395.1	Microviridae sp.
2202644	GCA_003827595.1	Microviridae sp.
2202644	GCA_003829135.1	Microviridae sp.
2202644	GCA_003821615.1	Microviridae sp.
2202644	GCA_003827055.1	Microviridae sp.
2202644	GCA_003831935.1	Microviridae sp.
2202644	GCA_003825715.1	Microviridae sp.
2202644	GCA_003827775.1	Microviridae sp.
2202644	GCA_003819575.1	Microviridae sp.
2202644	GCA_003817215.1	Microviridae sp.
2202644	GCA_003832415.1	Microviridae sp.
2202644	GCA_003829155.1	Microviridae sp.
2202644	GCA_003821635.1	Microviridae sp.
2202644	GCA_003831955.1	Microviridae sp.
2202644	GCA_003831635.1	Microviridae sp.
2202644	GCA_003832435.1	Microviridae sp.

2202644	GCA_003844695.1	Microviridae sp.
2202644	GCA_003827795.1	Microviridae sp.
2202644	GCA_003829175.1	Microviridae sp.
2202644	GCA_003821655.1	Microviridae sp.
2202644	GCA_003825355.1	Microviridae sp.
2202644	GCA_003825755.1	Microviridae sp.
2202644	GCA_003819615.1	Microviridae sp.
2202644	GCA_003816675.1	Microviridae sp.
2202644	GCA_003823715.1	Microviridae sp.
2202644	GCA_003832455.1	Microviridae sp.
2202644	GCA_003827815.1	Microviridae sp.
2202644	GCA_003824675.1	Microviridae sp.
2202644	GCA_003829195.1	Microviridae sp.
2202644	GCA_003821675.1	Microviridae sp.
2202644	GCA_003826695.1	Microviridae sp.
2202644	GCA_003825775.1	Microviridae sp.
2202644	GCA_003819215.1	Microviridae sp.
2202644	GCA_003819635.1	Microviridae sp.
2202644	GCA_003823735.1	Microviridae sp.
2202644	GCA_003827855.1	Microviridae sp.
2202644	GCA_003844675.1	Microviridae sp.
2202644	GCA_003829215.1	Microviridae sp.
2202644	GCA_003821695.1	Microviridae sp.
2202644	GCA_003823655.1	Microviridae sp.
2202644	GCA_003831975.1	Microviridae sp.
2202644	GCA_003825795.1	Microviridae sp.
2202644	GCA_003844335.1	Microviridae sp.
2202644	GCA_003819655.1	Microviridae sp.
2202644	GCA_003817495.1	Microviridae sp.
2202644	GCA_003823755.1	Microviridae sp.
2202644	GCA_003845835.1	Microviridae sp.
2202644	GCA_003829235.1	Microviridae sp.
2202644	GCA_003821715.1	Microviridae sp.
2202644	GCA_003831995.1	Microviridae sp.
2202644	GCA_003825815.1	Microviridae sp.
2202644	GCA_003819675.1	Microviridae sp.
2202644	GCA_003823775.1	Microviridae sp.
2202644	GCA_003827415.1	Microviridae sp.
2202644	GCA_003832475.1	Microviridae sp.
2202644	GCA_003827875.1	Microviridae sp.
2202644	GCA_003829255.1	Microviridae sp.
2202644	GCA_003821735.1	Microviridae sp.
2202644	GCA_003817575.1	Microviridae sp.
2202644	GCA_003832015.1	Microviridae sp.
2202644	GCA_003825835.1	Microviridae sp.
2202644	GCA_003826735.1	Microviridae sp.
2202644	GCA_003823795.1	Microviridae sp.
2202644	GCA_003827395.1	Microviridae sp.
2202644	GCA_003827895.1	Microviridae sp.
2202644	GCA_003821755.1	Microviridae sp.

2202644	GCA_003832035.1	Microviridae sp.
2202644	GCA_003825855.1	Microviridae sp.
2202644	GCA_003823815.1	Microviridae sp.
2202644	GCA_003844035.1	Microviridae sp.
2202644	GCA_003832495.1	Microviridae sp.
2202644	GCA_003827935.1	Microviridae sp.
2202644	GCA_003829275.1	Microviridae sp.
2202644	GCA_003821775.1	Microviridae sp.
2202644	GCA_003825375.1	Microviridae sp.
2202644	GCA_003832055.1	Microviridae sp.
2202644	GCA_003825875.1	Microviridae sp.
2202644	GCA_003816695.1	Microviridae sp.
2202644	GCA_003823835.1	Microviridae sp.
2202644	GCA_003844495.1	Microviridae sp.
2202644	GCA_003832515.1	Microviridae sp.
2202644	GCA_003845915.1	Microviridae sp.
2202644	GCA_003816355.1	Microviridae sp.
2202644	GCA_003829295.1	Microviridae sp.
2202644	GCA_003821795.1	Microviridae sp.
2202644	GCA_003825895.1	Microviridae sp.
2202644	GCA_003819235.1	Microviridae sp.
2202644	GCA_003823855.1	Microviridae sp.
2202644	GCA_003832535.1	Microviridae sp.
2202644	GCA_003827955.1	Microviridae sp.
2202644	GCA_003822195.1	Microviridae sp.
2202644	GCA_003829315.1	Microviridae sp.
2202644	GCA_003821815.1	Microviridae sp.
2202644	GCA_003823675.1	Microviridae sp.
2202644	GCA_003825915.1	Microviridae sp.
2202644	GCA_003844355.1	Microviridae sp.
2202644	GCA_003819555.1	Microviridae sp.
2202644	GCA_003823875.1	Microviridae sp.
2202644	GCA_003832555.1	Microviridae sp.
2202644	GCA_003827975.1	Microviridae sp.
2202644	GCA_003829335.1	Microviridae sp.
2202644	GCA_003821835.1	Microviridae sp.
2202644	GCA_003825935.1	Microviridae sp.
2202644	GCA_003822655.1	Microviridae sp.
2202644	GCA_003817295.1	Microviridae sp.
2202644	GCA_003829455.1	Microviridae sp.
2202644	GCA_003823895.1	Microviridae sp.
2202644	GCA_003832575.1	Microviridae sp.
2202644	GCA_003827995.1	Microviridae sp.
2202644	GCA_003829355.1	Microviridae sp.
2202644	GCA_003821855.1	Microviridae sp.
2202644	GCA_003821975.1	Microviridae sp.
2202644	GCA_003827535.1	Microviridae sp.
2202644	GCA_003825955.1	Microviridae sp.
2202644	GCA_003826755.1	Microviridae sp.
2202644	GCA_003823915.1	Microviridae sp.

2202644	GCA_003828015.1	Microviridae sp.
2202644	GCA_003844235.1	Microviridae sp.
2202644	GCA_003829375.1	Microviridae sp.
2202644	GCA_003821875.1	Microviridae sp.
2202644	GCA_003824535.1	Microviridae sp.
2202644	GCA_003825975.1	Microviridae sp.
2202644	GCA_003820955.1	Microviridae sp.
2202644	GCA_003823935.1	Microviridae sp.
2202644	GCA_003825735.1	Microviridae sp.
2202644	GCA_003832595.1	Microviridae sp.
2202644	GCA_003828035.1	Microviridae sp.
2202644	GCA_003829395.1	Microviridae sp.
2202644	GCA_003825395.1	Microviridae sp.
2202644	GCA_003843975.1	Microviridae sp.
2202644	GCA_003825995.1	Microviridae sp.
2202644	GCA_003816715.1	Microviridae sp.
2202644	GCA_003823955.1	Microviridae sp.
2202644	GCA_003832615.1	Microviridae sp.
2202644	GCA_003828055.1	Microviridae sp.
2202644	GCA_003819595.1	Microviridae sp.
2202644	GCA_003829415.1	Microviridae sp.
2202644	GCA_003826855.1	Microviridae sp.
2202644	GCA_003826015.1	Microviridae sp.
2202644	GCA_003817775.1	Microviridae sp.
2202644	GCA_003823975.1	Microviridae sp.
2202644	GCA_003832635.1	Microviridae sp.
2202644	GCA_003828075.1	Microviridae sp.
2202644	GCA_003819695.1	Microviridae sp.
2202644	GCA_003823695.1	Microviridae sp.
2202644	GCA_003826035.1	Microviridae sp.
2202644	GCA_003822455.1	Microviridae sp.
2202644	GCA_003822355.1	Microviridae sp.
2202644	GCA_003823995.1	Microviridae sp.
2202644	GCA_003832655.1	Microviridae sp.
2202644	GCA_003828095.1	Microviridae sp.
2202644	GCA_003826055.1	Microviridae sp.
2202644	GCA_003819355.1	Microviridae sp.
2202644	GCA_003824015.1	Microviridae sp.
2202644	GCA_003817955.1	Microviridae sp.
2202644	GCA_003832675.1	Microviridae sp.
2202644	GCA_003828115.1	Microviridae sp.
2202644	GCA_003822015.1	Microviridae sp.
2202644	GCA_003826075.1	Microviridae sp.
2202644	GCA_003817275.1	Microviridae sp.
2202644	GCA_003824035.1	Microviridae sp.
2202644	GCA_003832695.1	Microviridae sp.
2202644	GCA_003828135.1	Microviridae sp.
2202644	GCA_003829475.1	Microviridae sp.
2202644	GCA_003821995.1	Microviridae sp.
2202559	GCA_003651525.2	Microvirus sp.

2202559	GCA_003653085.2	Microvirus sp.
2202559	GCA_003651545.2	Microvirus sp.
2202559	GCA_003651565.2	Microvirus sp.
2202559	GCA_003653085.1	Microvirus sp.
2202559	GCA_003651525.1	Microvirus sp.
2202559	GCA_003651545.1	Microvirus sp.
2202559	GCA_003651565.1	Microvirus sp.
11023	GCA_000921735.1	Middelburg virus
11023	GCA_002889115.1	Middelburg virus
644609	GCA_000883875.1	Midway nyavirus
487492	GCA_000880435.1	Mikania micrantha mosaic virus
1546177	GCA_000925195.1	Mikumi yellow baboon virus 1
1455651	GCA_000915455.1	Milk vetch dwarf C1 alphasatellite
1455652	GCA_000916635.1	Milk vetch dwarf C10 alphasatellite
1455653	GCA_000916115.1	Milk vetch dwarf C2 alphasatellite
1455654	GCA_000914415.1	Milk vetch dwarf C3 alphasatellite
67585	GCA_000840045.1	Milk vetch dwarf virus
2293296	GCA_003846805.1	Millipede associated circular virus 1
2005322	GCA_003729255.1	Milolii virus
1988039	GCA_002966295.1	Mimivirus AB-566-O17
1835008	GCA_002966425.1	Mimivirus Bombay
2496520	GCA_004151645.1	Mimivirus sp. SH
1128151	GCA_000918435.1	Mimivirus terra2
390440	GCA_000870825.1	Mimosa yellow leaf curl virus
447600	GCA_000873325.1	Mimosa yellow leaf curl virus satellite DNA beta
447598	GCA_000871725.1	Mimosa yellow leaf curl virus-associated DNA 1
2137787	GCA_004030215.1	Mini PCV-like virus
1891769	GCA_000901355.1	Miniopterus africanus polyomavirus 1
1985387	GCA_002825685.1	Miniopterus associated gemycircularvirus 1
694000	GCA_000879255.1	Miniopterus bat coronavirus 1
694001	GCA_000879875.1	Miniopterus bat coronavirus HKU8
1911554	GCA_003033275.1	Miniopterus schreibersii bat bocavirus
1195364	GCA_002826925.1	Miniopterus schreibersii papillomavirus 1
1195365	GCA_002113985.1	Miniopterus schreibersii picornavirus 1
1904408	GCA_002037795.1	Miniopterus schreibersii polyomavirus 1
1904409	GCA_002037755.1	Miniopterus schreibersii polyomavirus 2
2259808	GCA_001722885.1	Mink bocavirus 1
154122	GCA_000901135.1	Mink calicivirus
1475143	GCA_000918015.1	Mink circovirus
1475143	GCA_004046075.1	Mink circovirus
1475143	GCA_004046095.1	Mink circovirus
1475143	GCA_004046115.1	Mink circovirus
1475143	GCA_004046135.1	Mink circovirus
1475143	GCA_004046155.1	Mink circovirus
1475143	GCA_004046175.1	Mink circovirus
1475143	GCA_004051695.1	Mink circovirus
766791	GCA_000919475.1	Mink coronavirus strain WD1127
300740	GCA_000858345.1	Mint virus 1
301865	GCA_000859705.1	Mint virus X
10794	GCA_000838465.1	Minute virus of mice

1093773	GCA_000893055.1	Mirabilis jalapa mottle virus
2049883	GCA_002987875.1	Mirabilis leaf curl betasatellite
1507403	GCA_000923295.1	Mirabilis leaf curl India virus associated betasatellite
1431395	GCA_000923935.1	Mirabilis leaf curl virus
194445	GCA_000845365.1	Mirabilis mosaic virus
200255	GCA_000851125.1	Mirafiori lettuce big-vein virus
268776	GCA_000839945.1	Miscanthus streak virus - [91]
2480179	GCA_004132345.1	Mizyes virus 1
55097	GCA_000866245.1	Mobala mammarenavirus
1408137	GCA_000912215.1	Mobuck virus
2072024	GCA_001634575.1	Mocis latipes granulovirus
64300	GCA_000860905.1	Modoc virus
1172985	GCA_002037735.1	Mogiana tick virus
1474807	GCA_000926555.1	Mojiang virus
12538	GCA_000856485.1	Mokola lyssavirus
1906245	GCA_001766585.1	Moku virus
1678078	GCA_001292995.1	Mollivirus sibericum
10280	GCA_000843325.1	Molluscum contagiosum virus subtype 1
11801	GCA_000854185.1	Moloney murine leukemia virus
11809	GCA_000849425.1	Moloney murine sarcoma virus
1959842	GCA_004045975.1	Molossus molossus circovirus 1
1959843	GCA_004045955.1	Molossus molossus circovirus 2
1959844	GCA_004045935.1	Molossus molossus circovirus 3
1959845	GCA_004045915.1	Molossus molossus circovirus 4
1959848	GCA_004130195.1	Molossus molossus papillomavirus 1
1606504	GCA_001430215.1	Molossus molossus polyomavirus 1
2291613	GCA_003847565.1	Momordica charantia associated gemycircularvirus
1634485	GCA_000973335.1	Mongoose feces-associated gemycircularvirus a
1634486	GCA_000973415.1	Mongoose feces-associated gemycircularvirus b
1634488	GCA_000973235.1	Mongoose feces-associated gemycircularvirus c
1634487	GCA_000973475.1	Mongoose feces-associated gemycircularvirus d
619591	GCA_000857045.1	Monkeypox virus Zaire-96-I-16
64312	GCA_000861705.1	Montana myotis leukoencephalitis virus
1980481	GCA_002117715.1	Montano orthohantavirus
300180	GCA_000855745.1	Mopeia Lassa virus reassortant 29
300175	GCA_000856585.1	Mopeia virus AN20410
1647524	GCA_002605945.1	Moraxella phage Mcat1
1647525	GCA_002605965.1	Moraxella phage Mcat10
1647526	GCA_002605985.1	Moraxella phage Mcat11
1647527	GCA_002606005.1	Moraxella phage Mcat12
1647528	GCA_002606025.1	Moraxella phage Mcat13
1647529	GCA_002606045.1	Moraxella phage Mcat14
1647530	GCA_002606065.1	Moraxella phage Mcat15
1647532	GCA_002606105.1	Moraxella phage Mcat17
1647533	GCA_002606125.1	Moraxella phage Mcat18
1647534	GCA_002606145.1	Moraxella phage Mcat19
1647535	GCA_002606165.1	Moraxella phage Mcat2
1647536	GCA_002606185.1	Moraxella phage Mcat20
1647537	GCA_002606205.1	Moraxella phage Mcat21
1647538	GCA_002606225.1	Moraxella phage Mcat22

1647539	GCA_002606245.1	Moraxella phage Mcat23
1647540	GCA_002606265.1	Moraxella phage Mcat24
1647541	GCA_002606285.1	Moraxella phage Mcat25
1647542	GCA_002606305.1	Moraxella phage Mcat26
1647543	GCA_002606325.1	Moraxella phage Mcat27
1647544	GCA_002606345.1	Moraxella phage Mcat28
1647545	GCA_002606365.1	Moraxella phage Mcat29
1647546	GCA_002606385.1	Moraxella phage Mcat3
1647547	GCA_002606405.1	Moraxella phage Mcat30
1647548	GCA_002606425.1	Moraxella phage Mcat31
1647549	GCA_002606445.1	Moraxella phage Mcat32
1647550	GCA_002606465.1	Moraxella phage Mcat4
1647551	GCA_002606485.1	Moraxella phage Mcat5
1647552	GCA_002606505.1	Moraxella phage Mcat6
1647553	GCA_002606525.1	Moraxella phage Mcat7
1647554	GCA_002606545.1	Moraxella phage Mcat8
1647555	GCA_002606565.1	Moraxella phage Mcat9
1081054	GCA_000892995.1	Morelia spilota papillomavirus 1
2016400	GCA_002270705.1	Morelia viridis nidovirus
1965532	GCA_002620925.1	Morganella phage IME1369_01
1965488	GCA_002620785.1	Morganella phage IME1369_02
1965534	GCA_002620965.1	Morganella phage IME1369_03
526118	GCA_000881355.2	Morganella phage MmP1
1852628	GCA_001744575.1	Morganella phage vB_MmoM_MP1
1852627	GCA_001745895.1	Morganella phage vB_MmoP_MP2
208086	GCA_000903935.1	Moroccan pepper virus
167129	GCA_000871305.1	Moroccan watermelon mosaic virus
573900	GCA_000886675.1	Morogoro mammarenavirus
1972565	GCA_002145705.1	Morreton vesiculovirus
1481453	GCA_000918135.1	Mosavirus A2
200403	GCA_002118525.1	Mosqueiro virus
1611039	GCA_000943745.1	Mosquito circovirus
945962	GCA_000892235.1	Mosquito densovirus BR/07
1911442	GCA_001866285.1	Mosquito dicistrovirus
673515	GCA_000906355.1	Mosquito flavivirus
1034805	GCA_002825705.1	Mosquito VEM virus SDBVL G
1034806	GCA_003726155.1	Mosquito VEM virus SDRBAJ
241630	GCA_000852705.1	Mossman virus
2083199	GCA_002888775.1	Mosso das Pedras virus
200404	GCA_002815615.1	Mossuril virus
1561160	GCA_002220005.1	Motherwort yellow mottle virus
1892236	GCA_003673725.1	Mothra virus
2109587	GCA_004156295.1	Moumouvirus australiensis
1247379	GCA_002966435.1	Moumouvirus goulette
380434	GCA_002146045.1	Mount Elgon bat virus
2169856	GCA_001866815.1	Mouse associated cyclovirus 1
1074210	GCA_000894795.1	Mouse astrovirus M-52/USA/2008
2316143	GCA_004134425.1	Mouse kidney parvovirus
1074213	GCA_000893835.1	Mouse kobuvirus M-5/USA/2010
11757	GCA_000846425.1	Mouse mammary tumor virus

1074428	GCA_002817275.1	Mouse Mosavirus
698672	GCA_000925515.1	Moussa virus
1516081	GCA_000923355.1	MSSI2.225 virus
60875	GCA_002829885.1	Mucambo virus
932662	GCA_000887875.1	Mud crab virus
1569922	GCA_004114875.1	Mukawa virus
232802	GCA_000849785.1	Mulard duck circovirus
232802	GCA_004086415.1	Mulard duck circovirus
1227557	GCA_000930135.1	Mulberry badnavirus 1
1631303	GCA_000969015.1	Mulberry mosaic dwarf associated virus
1527441	GCA_002867205.1	Mulberry mosaic leaf roll associated virus
1266451	GCA_000954755.2	Mulberry vein banding virus
2137858	GCA_004133125.1	Mume virus A
1979165	GCA_000856685.1	Mumps rubulavirus
223287	GCA_000858565.1	Mungbean yellow mosaic India virus
1238162	GCA_000900355.1	Mungbean yellow mosaic India virus associated betasatelli
33726	GCA_000845225.1	Mungbean yellow mosaic virus
1048854	GCA_002008555.1	Munguba virus
572289	GCA_000880835.1	Munia coronavirus HKU13-3514
10366	GCA_000859805.1	Murid betaherpesvirus 1 (Murine cytomegalovirus)
1261657	GCA_000902655.1	Murid betaherpesvirus 8
33708	GCA_000845665.1	Murid gammaherpesvirus 4 (Murine herpesvirus 68)
10530	GCA_000844265.1	Murine adenovirus 1
10530	GCA_000885275.1	Murine adenovirus 1
931972	GCA_000889615.1	Murine adenovirus 2
573199	GCA_000884755.1	Murine adenovirus 3
1141625	GCA_000900135.1	Murine astrovirus
2171383	GCA_004042915.1	Murine circovirus
2171384	GCA_003848505.1	Murine feces-associated gemycircularvirus 1
2171385	GCA_003848485.1	Murine feces-associated gemycircularvirus 2
11138	GCA_000862345.1	Murine hepatitis virus
11138	GCA_003971785.1	Murine hepatitis virus
223997	GCA_000868425.1	Murine norovirus 1
11263	GCA_002815495.1	Murine orthopneumovirus
11830	GCA_000847465.1	Murine osteosarcoma virus
179241	GCA_000863865.1	Murine polyomavirus strain BG
11191	GCA_000855625.1	Murine respirovirus
1940555	GCA_002003795.1	Murine roseolovirus
28327	GCA_003259085.1	Murine rotavirus
44561	GCA_000859085.1	Murine type C retrovirus
2025359	GCA_002270885.1	Murmansk poxvirus
11079	GCA_000863565.1	Murray Valley encephalitis virus
1406134	GCA_000913635.1	Murrumbidgee virus
590745	GCA_001619015.1	Mus musculus mobilized endogenous polytropic provirus
763552	GCA_000888435.1	Mus musculus papillomavirus type 1
1891770	GCA_000837065.1	Mus musculus polyomavirus 2
1891770	GCA_002827905.1	Mus musculus polyomavirus 2
523909	GCA_000879935.1	Musca domestica salivary gland hypertrophy virus
257468	GCA_000846025.1	Muscovy duck circovirus
257468	GCA_004034215.1	Muscovy duck circovirus

257468	GCA_004034175.1	Muscovy duck circovirus
257468	GCA_004033675.1	Muscovy duck circovirus
257468	GCA_004034255.1	Muscovy duck circovirus
257468	GCA_004034075.1	Muscovy duck circovirus
257468	GCA_004034115.1	Muscovy duck circovirus
257468	GCA_004034155.1	Muscovy duck circovirus
257468	GCA_004035635.1	Muscovy duck circovirus
257468	GCA_004034135.1	Muscovy duck circovirus
257468	GCA_004034195.1	Muscovy duck circovirus
257468	GCA_004061535.1	Muscovy duck circovirus
257468	GCA_004061515.1	Muscovy duck circovirus
257468	GCA_004061555.1	Muscovy duck circovirus
257468	GCA_004062695.1	Muscovy duck circovirus
257468	GCA_004086435.1	Muscovy duck circovirus
257468	GCA_004088915.1	Muscovy duck circovirus
257468	GCA_004087515.1	Muscovy duck circovirus
257468	GCA_004096295.1	Muscovy duck circovirus
257468	GCA_004092415.1	Muscovy duck circovirus
257468	GCA_004092435.1	Muscovy duck circovirus
257468	GCA_004092455.1	Muscovy duck circovirus
37325	GCA_000845505.1	Muscovy duck parvovirus
77153	GCA_003093075.1	Muscovy duck reovirus
77153	GCA_003092615.1	Muscovy duck reovirus
77153	GCA_003092675.1	Muscovy duck reovirus
77153	GCA_003092875.1	Muscovy duck reovirus
32625	GCA_000849205.1	Mushroom bacilliform virus
2259540	GCA_000912015.1	Mustela putorius papillomavirus 1
1203539	GCA_000898335.1	MW polyomavirus
1195069	GCA_002602385.1	Mycobacteriophage ElTiger69
1458726	GCA_000916335.1	Mycobacterium phage 20ES
1445729	GCA_000915655.1	Mycobacterium phage 32HC
1463809	GCA_000915615.1	Mycobacterium phage 39HC
1458717	GCA_000917135.1	Mycobacterium phage 40AC
1463810	GCA_002604465.1	Mycobacterium phage 40BC
2072003	GCA_003004845.1	Mycobacterium phage A6
2163589	GCA_003143015.1	Mycobacterium phage AbbyPaige
1358124	GCA_002755895.1	Mycobacterium phage ABCat
1916112	GCA_002615705.1	Mycobacterium phage Abdiel
1551710	GCA_001502435.1	Mycobacterium phage Abrogate
1034126	GCA_002991115.1	Mycobacterium phage ABU
2500573	GCA_004015705.1	Mycobacterium phage ACFishhook
1916113	GCA_002615725.1	Mycobacterium phage Achebe
2079563	GCA_002958935.1	Mycobacterium phage Acme
2250290	GCA_003365155.1	Mycobacterium phage Acolyte
2499030	GCA_004008375.1	Mycobacterium phage Acquire49
2126944	GCA_003051325.1	Mycobacterium phage ActinUp
2301546	GCA_003441875.1	Mycobacterium phage Adahisdi
1354507	GCA_000911075.1	Mycobacterium phage Adawi
1034127	GCA_002632565.1	Mycobacterium phage Adephagia
2502471	GCA_004148585.1	Mycobacterium phage Ading

528321	GCA_002758675.1	Mycobacterium phage Adjutor
1327959	GCA_000918395.1	Mycobacterium phage Adler
1453337	GCA_002603545.1	Mycobacterium phage ADLER F1725
2510582	GCA_004147945.1	Mycobacterium phage Adlitam
2250354	GCA_003341315.1	Mycobacterium phage Adnama
2108121	GCA_003013965.1	Mycobacterium phage Adonis
1383059	GCA_000910775.1	Mycobacterium phage Adzzy
1168595	GCA_000919355.1	Mycobacterium phage Aeneas
2502418	GCA_004147525.1	Mycobacterium phage AFIS
2047833	GCA_002956285.1	Mycobacterium phage AgentM
2301547	GCA_003441895.1	Mycobacterium phage Aggie
1873697	GCA_002599905.1	Mycobacterium phage Aglet
1041808	GCA_002601225.1	Mycobacterium phage Airmid
1089110	GCA_000920295.1	Mycobacterium phage Akoma
1647307	GCA_002605785.1	Mycobacterium phage AlanGrant
1913041	GCA_002614805.1	Mycobacterium phage Albee
1235690	GCA_002600425.1	Mycobacterium phage Alex
2094143	GCA_003014235.1	Mycobacterium phage Alexphander
1034128	GCA_001505915.1	Mycobacterium phage Alice
2163590	GCA_003143035.1	Mycobacterium phage AlishaPH
2015840	GCA_002626065.1	Mycobacterium phage AlleyCat
1458724	GCA_000919595.1	Mycobacterium phage Alsfro
2094128	GCA_003014245.1	Mycobacterium phage AltPhacts
2488961	GCA_003868335.1	Mycobacterium phage Altwerkus
1567466	GCA_000954335.1	Mycobacterium phage Alvin
2502465	GCA_004148405.1	Mycobacterium phage Amataga
1913035	GCA_002614685.1	Mycobacterium phage Amelie
2015817	GCA_002625605.1	Mycobacterium phage Amgine
2250291	GCA_003365175.1	Mycobacterium phage Aminay
2301540	GCA_003441915.1	Mycobacterium phage Amochick
2015874	GCA_002626705.1	Mycobacterium phage Amohnition
2047834	GCA_002956295.1	Mycobacterium phage Andies
649688	GCA_000885575.1	Mycobacterium phage Angel
1076630	GCA_000911935.1	Mycobacterium phage AnnaL29
1701801	GCA_002607385.1	Mycobacterium phage Annihilator
2126945	GCA_003051345.1	Mycobacterium phage Annyong
2041517	GCA_002744295.1	Mycobacterium phage Anselm
2499031	GCA_004008515.1	Mycobacterium phage Antonia
1354511	GCA_001505835.2	Mycobacterium phage Anubis
1354511	GCA_001505835.1	Mycobacterium phage Anubis
1673886	GCA_001470115.1	Mycobacterium phage Apizium
2027890	GCA_002629345.1	Mycobacterium phage Apocalypse
2015841	GCA_002626085.1	Mycobacterium phage Appletree2
2047835	GCA_002956305.1	Mycobacterium phage Aragog
1079896	GCA_002601585.1	Mycobacterium phage Arbiter
2315612	GCA_003613735.1	Mycobacterium phage Arcanine
1815972	GCA_001754385.1	Mycobacterium phage ArcherNM
1327938	GCA_000907555.1	Mycobacterium phage ArcherS7
2047836	GCA_002956315.1	Mycobacterium phage Archetta
1718599	GCA_001504895.1	Mycobacterium phage Archie

2315613	GCA_003614655.1	Mycobacterium phage ArcusAngelus
1089112	GCA_002623405.1	Mycobacterium phage Ares
2517937	GCA_004520655.1	Mycobacterium phage Argie
2041518	GCA_002744315.1	Mycobacterium phage Arib1
1541824	GCA_001501835.1	Mycobacterium phage Ariel
1541824	GCA_001501835.2	Mycobacterium phage Ariel
2530173	GCA_004340285.1	Mycobacterium phage Arissanae
2283250	GCA_003366495.1	Mycobacterium phage Arlo
2027891	GCA_002629365.1	Mycobacterium phage Aroostook
1391429	GCA_000915115.1	Mycobacterium phage Artemis2UCLA
1933689	GCA_002758295.1	Mycobacterium phage Ashraf
2081618	GCA_002990275.1	Mycobacterium phage Asriel
1327762	GCA_000908575.1	Mycobacterium phage Astraea
1089113	GCA_002623425.1	Mycobacterium phage Athena
2502480	GCA_004148865.1	Mycobacterium phage Atlantean
1458709	GCA_002755935.1	Mycobacterium phage Audrey
2041519	GCA_002744335.1	Mycobacterium phage Audrick
2250325	GCA_003365635.1	Mycobacterium phage AugsMagnumOpus
1176426	GCA_002598505.1	Mycobacterium phage Ava3
2517938	GCA_004520935.1	Mycobacterium phage AvatarAhPeg
2250292	GCA_003365195.1	Mycobacterium phage Avle17
2024302	GCA_002628785.1	Mycobacterium phage Avocado
1089114	GCA_002601705.1	Mycobacterium phage Avrafan
2077133	GCA_002958315.1	Mycobacterium phage B1
2510523	GCA_004148965.1	Mycobacterium phage BabeRuth
2484208	GCA_003722555.1	Mycobacterium phage BaboJay
2502441	GCA_004147805.1	Mycobacterium phage Baby16
1897486	GCA_002612645.1	Mycobacterium phage BabyRay
1784956	GCA_001755125.1	Mycobacterium phage Bactobuster
2502475	GCA_004148685.1	Mycobacterium phage BadAgartude
1458710	GCA_001470175.1	Mycobacterium phage Badfish
1881140	GCA_002611985.1	Mycobacterium phage Badger
1647306	GCA_001482955.1	Mycobacterium phage Baee
1897448	GCA_002612565.1	Mycobacterium phage Baehexic
2099645	GCA_002997465.1	Mycobacterium phage Baloo
1354508	GCA_000912715.2	Mycobacterium phage Bane1
1354509	GCA_002603825.1	Mycobacterium phage Bane2
2315706	GCA_003613415.1	Mycobacterium phage Bangla1971
2178918	GCA_003182765.1	Mycobacterium phage Banjo
2081619	GCA_002990285.1	Mycobacterium phage Barbarian
205880	GCA_000840585.1	Mycobacterium phage Barnyard
1084722	GCA_000918555.1	Mycobacterium phage BarrelRoll
1675548	GCA_001500895.1	Mycobacterium phage Barriga
2015875	GCA_002626725.1	Mycobacterium phage Bartholomew
2502484	GCA_004148945.1	Mycobacterium phage Basquiat
2126812	GCA_003023965.1	Mycobacterium phage Batiatus
2094129	GCA_003014255.1	Mycobacterium phage BatteryCK
2041520	GCA_002744355.1	Mycobacterium phage BeanWater
2500791	GCA_004147325.1	Mycobacterium phage Beatrix
2182392	GCA_003183165.1	Mycobacterium phage Beauxregard13

2488783	GCA_003866955.1	Mycobacterium phage Beelzebub
2081620	GCA_002990295.1	Mycobacterium phage BeesKnees
2250293	GCA_003365215.1	Mycobacterium phage BEEST
2250355	GCA_003342475.1	Mycobacterium phage Beezoo
2024005	GCA_002627585.1	Mycobacterium phage Bella96
2301541	GCA_003441935.1	Mycobacterium phage Belladonna
1429791	GCA_000915695.1	Mycobacterium phage BellusTerra
1486424	GCA_000919995.1	Mycobacterium phage Bernal13
1429903	GCA_000911535.1	Mycobacterium phage Bernardo
2419976	GCA_003691995.1	Mycobacterium phage BigCheese
1897425	GCA_002612205.1	Mycobacterium phage Bigfoot
2250372	GCA_003341495.1	Mycobacterium phage Biglebops
2126786	GCA_003024115.1	Mycobacterium phage BigMama
2250419	GCA_003364435.1	Mycobacterium phage BigMau
1074309	GCA_000919215.1	Mycobacterium phage BigNuz
2488962	GCA_003868265.1	Mycobacterium phage BigPaolini
2126946	GCA_003051365.1	Mycobacterium phage BigPhil
2041521	GCA_002744375.1	Mycobacterium phage Bigswole
1551711	GCA_001501815.1	Mycobacterium phage Bipolar
2478683	GCA_004147965.1	Mycobacterium phage Bipolarisk
1805457	GCA_001754405.1	Mycobacterium phage Bipper
1873903	GCA_002611745.1	Mycobacterium phage Bircsak
2499032	GCA_004008195.1	Mycobacterium phage Bishoperium
2072006	GCA_003004835.1	Mycobacterium phage BK1
2047837	GCA_002956325.1	Mycobacterium phage Blackmoor
1983574	GCA_002623725.1	Mycobacterium phage BlackStallion
1927019	GCA_002617505.1	Mycobacterium phage Blue
1089117	GCA_000919395.1	Mycobacterium phage Blue7
1327949	GCA_002755695.1	Mycobacterium phage Bo4
2099646	GCA_002997475.1	Mycobacterium phage Bob3
2182393	GCA_003183185.1	Mycobacterium phage BobaPhett
2530120	GCA_004339365.1	Mycobacterium phage Bobby
1340708	GCA_000912515.1	Mycobacterium phage Bobi
2015842	GCA_002626105.1	Mycobacterium phage BobSwaget
2014350	GCA_002625305.1	Mycobacterium phage Bogie
2488963	GCA_003868225.1	Mycobacterium phage Bones
1088864	GCA_002624025.3	Mycobacterium phage Bongo
1088864	GCA_002624025.2	Mycobacterium phage Bongo
1088864	GCA_002624025.1	Mycobacterium phage Bongo
2488964	GCA_003868195.1	Mycobacterium phage Bonray
2419977	GCA_003692015.1	Mycobacterium phage BoostSeason
2530121	GCA_004339135.1	Mycobacterium phage Bowtie
2126947	GCA_003051385.1	Mycobacterium phage Boyle
515982	GCA_002758655.1	Mycobacterium phage BPs
2500790	GCA_004147245.1	Mycobacterium phage BQuat
2315535	GCA_003613995.1	Mycobacterium phage Bread
1340712	GCA_001516995.1	Mycobacterium phage Breeniome
1327763	GCA_002603465.1	Mycobacterium phage Breezona
2283251	GCA_003366015.1	Mycobacterium phage BreSam8
1718601	GCA_002603405.1	Mycobacterium phage Bricole

1815608	GCA_001755685.1	Mycobacterium phage Brocalys
2283252	GCA_003366535.1	Mycobacterium phage Bromden
1913036	GCA_002614705.1	Mycobacterium phage Broseidon
1698252	GCA_001505015.1	Mycobacterium phage BrownCNA
1872689	GCA_002757835.1	Mycobacterium phage BruceB
1391179	GCA_000911575.1	Mycobacterium phage Bruin
1916114	GCA_002615745.1	Mycobacterium phage Bruiser
1698358	GCA_001502415.1	Mycobacterium phage Brusacoram
1262532	GCA_000907755.1	Mycobacterium phage BTCU-1
1932895	GCA_002618125.1	Mycobacterium phage Bubbles123
1927023	GCA_002617585.1	Mycobacterium phage BubbleTrouble
2250420	GCA_003364495.1	Mycobacterium phage Buckeye
2530122	GCA_004339285.1	Mycobacterium phage Bud
2517940	GCA_004520675.1	Mycobacterium phage Bumblebee11
1821721	GCA_002597805.1	Mycobacterium phage Burger
2488965	GCA_003868175.1	Mycobacterium phage Burrough
2301702	GCA_003442635.1	Mycobacterium phage Burwell21
1296646	GCA_000904695.1	Mycobacterium phage Butters
561997	GCA_002758695.1	Mycobacterium phage Butterscotch
1873892	GCA_002757855.1	Mycobacterium phage BuzzBuzz
1536598	GCA_000954355.1	Mycobacterium phage BuzzLyseyear
205877	GCA_000842365.1	Mycobacterium phage Bxz1
2250373	GCA_003341515.1	Mycobacterium phage ByChance
2182394	GCA_003183205.1	Mycobacterium phage Byougenkin
2072005	GCA_003005755.1	Mycobacterium phage C3
1739967	GCA_001470315.1	Mycobacterium phage Cabrinians
1897449	GCA_002612585.1	Mycobacterium phage CactusRose
1718177	GCA_002597985.1	Mycobacterium phage Caelakin
2015818	GCA_002625625.1	Mycobacterium phage Cain
546803	GCA_000881515.1	Mycobacterium phage Cali
1647305	GCA_001501215.1	Mycobacterium phage Cambiare
2499033	GCA_004006945.1	Mycobacterium phage CamL
1927024	GCA_002617605.1	Mycobacterium phage Camperdownii
2301548	GCA_003435005.1	Mycobacterium phage Cane17
2488966	GCA_003868135.1	Mycobacterium phage Cannibal
2488958	GCA_003868115.1	Mycobacterium phage Capricorn
1556289	GCA_001505335.1	Mycobacterium phage CaptainTrips
1555233	GCA_001505995.1	Mycobacterium phage Carcharodon
2530123	GCA_004339205.1	Mycobacterium phage Carthage
1327035	GCA_000909195.1	Mycobacterium phage CASbig
1792253	GCA_001754865.1	Mycobacterium phage Catalina
1340819	GCA_000913335.1	Mycobacterium phage Catdawg
373404	GCA_000869865.1	Mycobacterium phage Catera
2250294	GCA_003365235.1	Mycobacterium phage Cborch11
1698364	GCA_002756835.1	Mycobacterium phage Cedasite
2488784	GCA_003866975.1	Mycobacterium phage Centaur
2517941	GCA_004520995.1	Mycobacterium phage Cepens
1527463	GCA_000954595.1	Mycobacterium phage Cerasum
2041522	GCA_002744395.1	Mycobacterium phage Cerulean
2099629	GCA_002997495.1	Mycobacterium phage ChaChing

1698366	GCA_001505655.1	Mycobacterium phage Chadwick
563124	GCA_002758715.1	Mycobacterium phage Chah
2301703	GCA_003442655.1	Mycobacterium phage ChampagnePapi
2041523	GCA_002744415.1	Mycobacterium phage Chance64
2015876	GCA_002626745.1	Mycobacterium phage Chancellor
1567465	GCA_002605145.1	Mycobacterium phage Chandler
1567465	GCA_002605145.2	Mycobacterium phage Chandler
2015877	GCA_002626765.1	Mycobacterium phage Changeling
2502463	GCA_004148365.1	Mycobacterium phage Chargie21
1056830	GCA_000920155.1	Mycobacterium phage Charlie
2301609	GCA_003719055.1	Mycobacterium phage CharlieB
1897764	GCA_002758215.1	Mycobacterium phage CharlieGBrown
2517942	GCA_004520535.1	Mycobacterium phage Charm
205876	GCA_000840885.1	Mycobacterium phage Che9d
2178919	GCA_003182785.1	Mycobacterium phage CheetO
1506716	GCA_001505155.1	Mycobacterium phage Cheetobro
2283294	GCA_003366975.1	Mycobacterium phage Cherrybomb426
2341077	GCA_003722395.1	Mycobacterium phage Chewbacca
2502482	GCA_004148905.1	Mycobacterium phage ChickenPhender
2250356	GCA_003341295.1	Mycobacterium phage Childish
2530124	GCA_004338755.1	Mycobacterium phage Chill
1821722	GCA_002609645.1	Mycobacterium phage ChipMunk
1965451	GCA_002758355.1	Mycobacterium phage Chorkpop
1034130	GCA_002633495.1	Mycobacterium phage ChrisnMich
2099630	GCA_002997505.1	Mycobacterium phage Chunky
1327947	GCA_000910035.1	Mycobacterium phage Chy4
1327948	GCA_000908555.1	Mycobacterium phage Chy5
2500799	GCA_004147405.1	Mycobacterium phage CicholasNage
2499034	GCA_004008395.1	Mycobacterium phage Cici
2041524	GCA_002744435.1	Mycobacterium phage Cindaradix
2499035	GCA_004008275.1	Mycobacterium phage Citius
1927025	GCA_002617625.1	Mycobacterium phage Clarenza
2015878	GCA_002626785.1	Mycobacterium phage Clautastrophe
2108122	GCA_003013995.1	Mycobacterium phage CLED96
2250357	GCA_003341275.1	Mycobacterium phage Clifton
1391430	GCA_000915075.1	Mycobacterium phage CloudWang3
2126805	GCA_003034615.1	Mycobacterium phage Cobra
1897499	GCA_002612825.1	Mycobacterium phage Cocoaberry
663553	GCA_001471015.1	Mycobacterium phage Colbert
2301704	GCA_003442675.1	Mycobacterium phage Collard
2502478	GCA_004148805.1	Mycobacterium phage Colt
2250400	GCA_003364855.1	Mycobacterium phage Commander
2178920	GCA_003182805.1	Mycobacterium phage ConceptII
2517943	GCA_004520415.1	Mycobacterium phage Connomayer
2081622	GCA_002990355.1	Mycobacterium phage Conquerage
1391431	GCA_000913215.1	Mycobacterium phage Conspiracy
2250374	GCA_003345045.1	Mycobacterium phage Constella
1340833	GCA_000912455.1	Mycobacterium phage Contagion
2126948	GCA_003051405.1	Mycobacterium phage Coog
2502438	GCA_004147745.1	Mycobacterium phage Cookies

373406	GCA_000867405.1	Mycobacterium phage Cooper
2517944	GCA_004520735.1	Mycobacterium phage Cornucopia
1647312	GCA_002605825.1	Mycobacterium phage Corofin
2099631	GCA_002997515.1	Mycobacterium phage Corvo
1567467	GCA_002605165.1	Mycobacterium phage Cosmo
2499036	GCA_004007135.1	Mycobacterium phage Cosmolli16
2250375	GCA_003341535.1	Mycobacterium phage Craff
1458841	GCA_000914655.1	Mycobacterium phage CRB1
2483623	GCA_004338005.1	Mycobacterium phage CRB2
2500800	GCA_004147365.1	Mycobacterium phage Crespo
1932363	GCA_002617885.1	Mycobacterium phage CREW
2301549	GCA_003441975.1	Mycobacterium phage Crispicous1
1340836	GCA_000910795.1	Mycobacterium phage Crossroads
2315536	GCA_003614035.1	Mycobacterium phage Crownjwl
2234072	GCA_003340835.1	Mycobacterium phage Crucio
1932894	GCA_002618105.1	Mycobacterium phage CrystalP
2502434	GCA_004147705.1	Mycobacterium phage Cueylyss
2079417	GCA_002958745.1	Mycobacterium phage Cuke
2502427	GCA_004147585.1	Mycobacterium phage Czyszczon1
2070496	GCA_002958255.1	Mycobacterium phage D12
1340822	GCA_000910835.1	Mycobacterium phage Daenerys
2041526	GCA_002744475.1	Mycobacterium phage Daffodil
1897480	GCA_002758115.1	Mycobacterium phage Daffy
1698254	GCA_002756795.1	Mycobacterium phage DaHudson
2510509	GCA_004149045.1	Mycobacterium phage Daishi
1034131	GCA_002632665.1	Mycobacterium phage Daisy
2041527	GCA_002744495.1	Mycobacterium phage Dalmatian
2301605	GCA_003601455.1	Mycobacterium phage Dalmuri
1486469	GCA_000921955.1	Mycobacterium phage Damien
1074305	GCA_000917595.1	Mycobacterium phage Dandelion
1698357	GCA_001504195.1	Mycobacterium phage Dante
2530125	GCA_004338595.1	Mycobacterium phage Darionha
2502479	GCA_004148845.1	Mycobacterium phage Darko
2015879	GCA_002626805.1	Mycobacterium phage DarthP
1912975	GCA_002614565.1	Mycobacterium phage DarthPhader
2502440	GCA_004147785.1	Mycobacterium phage Datway
1034102	GCA_002600835.1	Mycobacterium phage DaVinci
2041525	GCA_002744455.1	Mycobacterium phage DaWorst
2517945	GCA_004520155.1	Mycobacterium phage DBQu4n
540064	GCA_000875405.1	Mycobacterium phage DD5
2094130	GCA_003014265.1	Mycobacterium phage Deby
2502461	GCA_004148265.1	Mycobacterium phage Delilah
2530186	GCA_004340545.1	Mycobacterium phage Delton
2041528	GCA_002744525.1	Mycobacterium phage Demsculpinboyz
2234026	GCA_003308875.1	Mycobacterium phage Derek
1897765	GCA_002758235.1	Mycobacterium phage Derpp
1327769	GCA_002597945.1	Mycobacterium phage Dhanush
2500801	GCA_004148165.1	Mycobacterium phage Dietrick
2163591	GCA_003143055.1	Mycobacterium phage DillTech15
2041529	GCA_002744565.1	Mycobacterium phage Dingo

2488967	GCA_003868075.1	Mycobacterium phage Dione
2517947	GCA_004520295.1	Mycobacterium phage DirtyDunning
2015880	GCA_002626825.1	Mycobacterium phage DismalFunk
2301610	GCA_003601075.1	Mycobacterium phage DismalStressor
2301699	GCA_003442715.1	Mycobacterium phage Dixon
2250358	GCA_003341255.1	Mycobacterium phage DMoney
2041530	GCA_002744615.1	Mycobacterium phage DmpstrDiver
1327950	GCA_002755715.1	Mycobacterium phage DNAIII
2250376	GCA_003341555.1	Mycobacterium phage DoctorDiddles
2250359	GCA_003341415.1	Mycobacterium phage Doddsville
2094131	GCA_003014275.1	Mycobacterium phage DoesntMatter
2530126	GCA_004338535.1	Mycobacterium phage Donny
1429793	GCA_000917195.1	Mycobacterium phage Donovan
2283253	GCA_003366475.1	Mycobacterium phage DonSanchon
1089121	GCA_000920255.1	Mycobacterium phage Dori
2027892	GCA_002629385.1	Mycobacterium phage Drake55
1034132	GCA_002598445.1	Mycobacterium phage Drazdys
1357669	GCA_000910715.1	Mycobacterium phage DrDrey
1086749	GCA_000917555.1	Mycobacterium phage Dreamboat
2250326	GCA_003365695.1	Mycobacterium phage DrFeelGood
1912976	GCA_002614585.1	Mycobacterium phage DrHayes
2499037	GCA_004008635.1	Mycobacterium phage DrLupo
2510581	GCA_004148005.1	Mycobacterium phage DrPhinkDaddy
2250327	GCA_003365715.1	Mycobacterium phage Druantia
45764	GCA_000917695.1	Mycobacterium phage DS6A
1701800	GCA_002603505.1	Mycobacterium phage DTDevon
2234073	GCA_003340855.1	Mycobacterium phage Dublin
2126949	GCA_003051425.1	Mycobacterium phage DuchessDung
1897766	GCA_002614025.1	Mycobacterium phage DudeLittle
2499038	GCA_004007015.1	Mycobacterium phage Duke13
2517948	GCA_004520695.1	Mycobacterium phage Dulcie
1327764	GCA_000909955.1	Mycobacterium phage Dumbo
1327764	GCA_000909955.2	Mycobacterium phage Dumbo
2499039	GCA_004007055.1	Mycobacterium phage Durga
1679524	GCA_001550645.1	Mycobacterium phage Dusk
1340831	GCA_000913555.1	Mycobacterium phage Dylan
1792149	GCA_002609245.1	Mycobacterium phage Dynamix
861046	GCA_002758915.1	Mycobacterium phage Eagle
1429759	GCA_000914755.1	Mycobacterium phage EagleEye
2301611	GCA_003601395.1	Mycobacterium phage Eaglehorse
2250401	GCA_003364895.1	Mycobacterium phage Eapen
2301705	GCA_003442735.1	Mycobacterium phage Easy2Say
2234074	GCA_003340875.1	Mycobacterium phage Ebony
1437839	GCA_000914695.1	Mycobacterium phage Echild
1567468	GCA_001551745.1	Mycobacterium phage Edtherson
2015843	GCA_002626125.1	Mycobacterium phage Edugator
1821221	GCA_002601685.1	Mycobacterium phage Eidsmoe
2301550	GCA_003441995.1	Mycobacterium phage Ejimix
2301563	GCA_003442215.1	Mycobacterium phage EleanorGeorge
2530187	GCA_004340565.1	Mycobacterium phage Elephantoon

1070989	GCA_002601385.1	Mycobacterium phage Elph10
1486470	GCA_002604585.1	Mycobacterium phage Emerson
2500802	GCA_004148085.1	Mycobacterium phage Emiris
2027893	GCA_002629405.1	Mycobacterium phage Emma
2502457	GCA_004148325.1	Mycobacterium phage EmmaElysia
2301564	GCA_003442235.1	Mycobacterium phage Emmina
1913118	GCA_002615225.1	Mycobacterium phage Empress
1486471	GCA_002756195.1	Mycobacterium phage EmpTee
2502456	GCA_004148305.1	Mycobacterium phage EmToTheThree
1933770	GCA_002955005.1	Mycobacterium phage EniyanLRS
1698709	GCA_001504975.1	Mycobacterium phage Enkosi
2099628	GCA_002997525.1	Mycobacterium phage EpicPhail
1555201	GCA_001501035.1	Mycobacterium phage Equemioh13
1897432	GCA_002612305.1	Mycobacterium phage Erdmann
1527494	GCA_001470415.1	Mycobacterium phage Eremos
2301542	GCA_003442015.1	Mycobacterium phage EricMillard
1916115	GCA_002615765.1	Mycobacterium phage Eris
2234027	GCA_003308235.1	Mycobacterium phage Erk16
1701802	GCA_002630465.1	Mycobacterium phage ErnieJ
2502443	GCA_004147845.1	Mycobacterium phage Eros
2502477	GCA_004148785.1	Mycobacterium phage Essence
1536603	GCA_000955075.1	Mycobacterium phage Estave1
663554	GCA_000885515.1	Mycobacterium phage ET08
2015881	GCA_002626845.3	Mycobacterium phage Et2Brutus
2015881	GCA_002626845.2	Mycobacterium phage Et2Brutus
2015881	GCA_002626845.1	Mycobacterium phage Et2Brutus
1718174	GCA_002757015.1	Mycobacterium phage Evanesce
1821723	GCA_001755165.1	Mycobacterium phage EvilGenius
2502432	GCA_004147665.1	Mycobacterium phage Ewok
2530127	GCA_004338575.1	Mycobacterium phage Expelliarmus
2488785	GCA_003866995.1	Mycobacterium phage ExplosioNervosa
2081623	GCA_002990365.1	Mycobacterium phage Fajezeel
2126943	GCA_003051445.1	Mycobacterium phage Fameo
2047838	GCA_002956335.1	Mycobacterium phage Familton
2530128	GCA_004338835.1	Mycobacterium phage Fancypants
701454	GCA_002758875.1	Mycobacterium phage Fang
1527517	GCA_002604905.1	Mycobacterium phage Farber
2108118	GCA_003014205.1	Mycobacterium phage Fascinus
2488786	GCA_003867055.1	Mycobacterium phage Faze9
2500803	GCA_004148125.1	Mycobacterium phage Fenn
1131256	GCA_002602045.1	Mycobacterium phage Fezzik
2517949	GCA_004520955.1	Mycobacterium phage Fibonacci
2484209	GCA_003723415.1	Mycobacterium phage Filuzino
2015882	GCA_002626865.1	Mycobacterium phage Findley
2015844	GCA_002743555.1	Mycobacterium phage Finemlucis
2041531	GCA_002744635.1	Mycobacterium phage FireRed
1245814	GCA_000904575.1	Mycobacterium phage First
1327765	GCA_000908475.1	Mycobacterium phage Fishburne
2502464	GCA_004148385.1	Mycobacterium phage Flabslab
1647304	GCA_001501995.1	Mycobacterium phage FlagStaff

2315707	GCA_003613475.1	Mycobacterium phage Flare16
1897483	GCA_002612605.1	Mycobacterium phage Florean
1675549	GCA_001502935.1	Mycobacterium phage Florinda
2502481	GCA_004148885.1	Mycobacterium phage Fludd
1567469	GCA_002756455.1	Mycobacterium phage FluffyNinja
1168593	GCA_002597905.1	Mycobacterium phage Flux
2047839	GCA_002956345.1	Mycobacterium phage ForGetIt
2099632	GCA_002997545.1	Mycobacterium phage Forsytheast
1882439	GCA_002612045.1	Mycobacterium phage Fortunato
2419978	GCA_003692215.1	Mycobacterium phage Fowlmouth
2502473	GCA_004148625.1	Mycobacterium phage FoxtrotP1
2530129	GCA_004338735.1	Mycobacterium phage Francis47
2079565	GCA_002958955.1	Mycobacterium phage Frankie
2502435	GCA_004147725.1	Mycobacterium phage FrayBell
2015809	GCA_002625465.1	Mycobacterium phage Fred313
1354510	GCA_000913775.1	Mycobacterium phage Fredward
2301543	GCA_003442035.1	Mycobacterium phage FrenchFry
1897767	GCA_002758255.1	Mycobacterium phage FriarPreacher
2499040	GCA_004007115.1	Mycobacterium phage Fringe
2488968	GCA_003868055.1	Mycobacterium phage Froghopper
1698365	GCA_002756855.1	Mycobacterium phage Frosty24
2315537	GCA_003614075.1	Mycobacterium phage FudgeTart
2250402	GCA_003364915.1	Mycobacterium phage FugateOSU
1927026	GCA_002617645.1	Mycobacterium phage Funston
2502430	GCA_004147645.1	Mycobacterium phage Fushigi
1897435	GCA_002612365.1	Mycobacterium phage Gabriel
1089122	GCA_000917755.1	Mycobacterium phage Gadjet
1567470	GCA_002605185.1	Mycobacterium phage Gadost
2081624	GCA_002990395.1	Mycobacterium phage Gage
2126813	GCA_003023975.1	Mycobacterium phage GageAP
1486472	GCA_000955315.1	Mycobacterium phage Gaia
2301612	GCA_003601115.1	Mycobacterium phage Galactic
2301613	GCA_003601135.1	Mycobacterium phage Gancho
1873696	GCA_001745175.1	Mycobacterium phage Gardann
1970789	GCA_002621205.1	Mycobacterium phage GardenSalsa
2250377	GCA_003342495.1	Mycobacterium phage Gareth
1852567	GCA_002604505.1	Mycobacterium phage Gattaca
2315538	GCA_003614095.1	Mycobacterium phage Gemini
2500804	GCA_004148105.1	Mycobacterium phage Gemma
2250403	GCA_003364955.1	Mycobacterium phage GeneCoco
2024010	GCA_002627645.1	Mycobacterium phage GenevaB15
1891963	GCA_001744795.1	Mycobacterium phage Gengar
2027894	GCA_002629425.1	Mycobacterium phage Geralt
2301551	GCA_003442055.1	Mycobacterium phage Gex
1034134	GCA_002598425.1	Mycobacterium phage Ghost
2027895	GCA_002629445.1	Mycobacterium phage Gideon
480808	GCA_000872185.1	Mycobacterium phage Giles
2502447	GCA_004148465.1	Mycobacterium phage GingkoMaracino
1675550	GCA_002607025.1	Mycobacterium phage Girafales
2301565	GCA_003442255.1	Mycobacterium phage Girr

1327936	GCA_000909175.1	Mycobacterium phage Gizmo
1784939	GCA_002609165.1	Mycobacterium phage Glass
2250328	GCA_003365775.1	Mycobacterium phage Glexan
1675551	GCA_002756695.1	Mycobacterium phage Godines
2517950	GCA_004520975.1	Mycobacterium phage GodPhather
1383057	GCA_000913395.1	Mycobacterium phage Goku
1906944	GCA_002614205.1	Mycobacterium phage Goldilocks
1606762	GCA_002605465.1	Mycobacterium phage Gomashi
1873895	GCA_001745975.1	Mycobacterium phage Gompeii16
2301706	GCA_003442755.1	Mycobacterium phage Gophee
2283295	GCA_003366915.1	Mycobacterium phage Gorge
1391432	GCA_000914115.1	Mycobacterium phage Graduation
2315614	GCA_003613755.1	Mycobacterium phage Grand2040
2517951	GCA_004520815.1	Mycobacterium phage GreaseLightnin
2126950	GCA_003051465.1	Mycobacterium phage GreedyLawyer
2079566	GCA_002958965.1	Mycobacterium phage Greg
2315539	GCA_003614215.1	Mycobacterium phage Grizzly
2250378	GCA_003341575.1	Mycobacterium phage Grum1
1897770	GCA_002614065.1	Mycobacterium phage Gruunaga
2072004	GCA_003004855.1	Mycobacterium phage GS4E
2015845	GCA_002626145.1	Mycobacterium phage Guillsminger
561998	GCA_000881535.1	Mycobacterium phage Gumball
1498190	GCA_002756215.1	Mycobacterium phage Guo1
2015819	GCA_002625645.1	Mycobacterium phage GuuelaD
1235691	GCA_002600405.1	Mycobacterium phage Gyarad
1527511	GCA_000954735.1	Mycobacterium phage Hades
2079283	GCA_002958425.1	Mycobacterium phage Haimas
2499041	GCA_004007255.1	Mycobacterium phage Haleema
2517952	GCA_004520395.1	Mycobacterium phage Halena
2163592	GCA_003143075.1	Mycobacterium phage Halley
2315708	GCA_003613495.1	Mycobacterium phage Hamish
1920298	GCA_002617045.1	Mycobacterium phage Hammy
1567483	GCA_002605245.1	Mycobacterium phage HamSlice
1340824	GCA_000913295.1	Mycobacterium phage Hamulus
2250299	GCA_003365295.1	Mycobacterium phage Hangman
1429904	GCA_000914015.1	Mycobacterium phage HanShotFirst
2282909	GCA_003423205.1	Mycobacterium phage Harley
1034104	GCA_002754955.1	Mycobacterium phage Harvey
1458711	GCA_000920975.1	Mycobacterium phage Hawkeye
2077135	GCA_002958335.1	Mycobacterium phage HC
1458712	GCA_002755955.1	Mycobacterium phage Heathcliff
1089123	GCA_002633745.1	Mycobacterium phage Hedgerow
1897516	GCA_002613005.1	Mycobacterium phage HedwigODU
1983575	GCA_002623745.1	Mycobacterium phage Heffalump
1897479	GCA_002758095.1	Mycobacterium phage Held
2517953	GCA_004520915.1	Mycobacterium phage Heliosoles
1034105	GCA_002601125.1	Mycobacterium phage Henry
2517935	GCA_004521035.1	Mycobacterium phage HenryJackson
2492961	GCA_004138855.1	Mycobacterium phage Henu3
2571241	GCA_004340185.1	Mycobacterium phage Henu3 PeY-2017

1821724	GCA_002599885.1	Mycobacterium phage Hercules11
1034106	GCA_002754975.1	Mycobacterium phage Hertubise
1700833	GCA_002756915.1	Mycobacterium phage Hetaeria
2502454	GCA_004148825.1	Mycobacterium phage Hexamo
1471543	GCA_002756095.1	Mycobacterium phage HH92
2079418	GCA_002958755.1	Mycobacterium phage HighStump
1327770	GCA_000907455.1	Mycobacterium phage HINdeR
2079567	GCA_002958975.1	Mycobacterium phage Holeinone
1821725	GCA_002597785.1	Mycobacterium phage Holli
2282908	GCA_003423165.1	Mycobacterium phage Homines
2250329	GCA_003365795.1	Mycobacterium phage Homura
2234028	GCA_003308255.1	Mycobacterium phage Hookmount
663555	GCA_002758735.1	Mycobacterium phage Hope
2250300	GCA_003365315.1	Mycobacterium phage Hope4ever
2283255	GCA_003366135.1	Mycobacterium phage Hopey
2041532	GCA_002744655.1	Mycobacterium phage Horchata
1897517	GCA_002613025.1	Mycobacterium phage HortumSL17
1463811	GCA_000922775.1	Mycobacterium phage Hosp
2315540	GCA_003614275.1	Mycobacterium phage Hotshotbaby7
2250379	GCA_003345065.1	Mycobacterium phage Houdini22
2283254	GCA_003366155.1	Mycobacterium phage HSavage
1430411	GCA_000911515.1	Mycobacterium phage HufflyPuff
2530130	GCA_004338855.1	Mycobacterium phage Hughesyang
2500792	GCA_004147285.1	Mycobacterium phage HuhtaEnerson15
2015810	GCA_002625485.1	Mycobacterium phage Hurricane
1698710	GCA_001503355.1	Mycobacterium phage HyRo
2517954	GCA_004520195.1	Mycobacterium phage Ibrahim
2250380	GCA_003341595.1	Mycobacterium phage Icee
2099633	GCA_002997565.1	Mycobacterium phage Ichabod
1176425	GCA_002602305.1	Mycobacterium phage ICleared
1958895	GCA_002619165.1	Mycobacterium phage Idleandcovert
2250301	GCA_003365335.1	Mycobacterium phage IHOP
2041533	GCA_002744675.1	Mycobacterium phage ILeeKay
1933691	GCA_002758335.1	Mycobacterium phage ImtiyazSitla
2283256	GCA_003366195.1	Mycobacterium phage Inca
2301553	GCA_003575765.1	Mycobacterium phage InigoMontoya
2126958	GCA_003051605.1	Mycobacterium phage InterFolia
1527580	GCA_000954715.1	Mycobacterium phage Inventum
2502459	GCA_004148185.1	Mycobacterium phage Iota
2301552	GCA_003442075.1	Mycobacterium phage IPhane7
1755681	GCA_001471035.1	Mycobacterium phage Iracema64
1897552	GCA_002758195.1	Mycobacterium phage Iridoclysis
2484210	GCA_003722635.1	Mycobacterium phage IrishSherpFalk
2499042	GCA_004007315.1	Mycobacterium phage IronMan
1089124	GCA_002601765.1	Mycobacterium phage IsaacEli
1897544	GCA_002613405.1	Mycobacterium phage Isiphiwo
861048	GCA_002630485.1	Mycobacterium phage Island3
2079568	GCA_002958985.1	Mycobacterium phage ItsyBitsy1
1897439	GCA_002612445.1	Mycobacterium phage Jaan
1340820	GCA_000911835.1	Mycobacterium phage Jabbawokkie

2126951	GCA_003051485.1	Mycobacterium phage Jabiru
1959484	GCA_002619425.1	Mycobacterium phage Jabith
1089136	GCA_000919375.1	Mycobacterium phage JacAttac
2499043	GCA_004006855.1	Mycobacterium phage JacoRen57
2499044	GCA_004007345.1	Mycobacterium phage JakeO
1429905	GCA_000916435.1	Mycobacterium phage JAMaL
2530131	GCA_004339385.1	Mycobacterium phage James
1882465	GCA_002612065.1	Mycobacterium phage Jane
2081625	GCA_002990415.1	Mycobacterium phage JangDynasty
2079419	GCA_002958765.1	Mycobacterium phage JangoPhett
2301554	GCA_003442095.1	Mycobacterium phage Jaykayelowell
1076631	GCA_002624005.1	Mycobacterium phage Jebeks
2015883	GCA_002626885.1	Mycobacterium phage Jeckyll
1777058	GCA_002608985.1	Mycobacterium phage JenCasNa
2108123	GCA_003013935.1	Mycobacterium phage Jeon
1897503	GCA_002612905.2	Mycobacterium phage Jerm
1897503	GCA_002612905.1	Mycobacterium phage Jerm
2488969	GCA_003868035.1	Mycobacterium phage Jessibeth14
2041534	GCA_002744695.1	Mycobacterium phage JetBlade
2502450	GCA_004148665.1	Mycobacterium phage JewelBug
2488970	GCA_003868015.1	Mycobacterium phage Jillium
1327771	GCA_000910215.1	Mycobacterium phage Job42
1327772	GCA_000909355.1	Mycobacterium phage Jobu08
2301566	GCA_003442295.1	Mycobacterium phage JoeyJr
1463812	GCA_000916275.1	Mycobacterium phage Jolie1
1458831	GCA_000917155.1	Mycobacterium phage Jolie2
1927027	GCA_002617665.1	Mycobacterium phage JoongJeon
2517955	GCA_004520215.1	Mycobacterium phage Jordennis
2234075	GCA_003340895.1	Mycobacterium phage Joselito
2024294	GCA_002628685.1	Mycobacterium phage JoshKayV
1983408	GCA_002623445.1	Mycobacterium phage Journey13
1429912	GCA_000915055.1	Mycobacterium phage Jovo
2250330	GCA_003365815.1	Mycobacterium phage Joy99
2126814	GCA_003023985.1	Mycobacterium phage Jsquared
2502460	GCA_004148235.1	Mycobacterium phage JustHall
2015846	GCA_002626165.1	Mycobacterium phage KADY
2250360	GCA_003341235.1	Mycobacterium phage Kahve
2041535	GCA_002744725.1	Mycobacterium phage Kailash
2301614	GCA_003601155.1	Mycobacterium phage Kalah2
2488959	GCA_003867995.1	Mycobacterium Phage Kalb97
2250421	GCA_003364555.1	Mycobacterium phage Kalnoky
1897545	GCA_002613425.1	Mycobacterium phage Kalpine
1089126	GCA_002601745.1	Mycobacterium phage Kamiyu
1486425	GCA_000922755.1	Mycobacterium phage Kampy
2502458	GCA_004148285.1	Mycobacterium phage Kamryn
2419979	GCA_003692255.1	Mycobacterium phage KandZ
2510510	GCA_004149085.1	Mycobacterium phage Kareem
2315541	GCA_003614315.1	Mycobacterium phage Kasen3
2502428	GCA_004147605.1	Mycobacterium phage Katalie136
1340830	GCA_000911815.1	Mycobacterium phage KayaCho

1821726	GCA_002757615.1	Mycobacterium phage Kazan
2488971	GCA_003867975.1	Mycobacterium phage Keitherie
1897440	GCA_002612465.1	Mycobacterium phage Kerberos
1897501	GCA_002612865.1	Mycobacterium phage Kersh
1567471	GCA_000955055.1	Mycobacterium phage Keshu
2530132	GCA_004338795.1	Mycobacterium phage Kevin1
1927028	GCA_002617685.1	Mycobacterium phage KFPoly
2488972	GCA_003867955.1	Mycobacterium phage Khaleesi
2108124	GCA_003013955.1	Mycobacterium phage Kheth
1089127	GCA_001505895.1	Mycobacterium phage Kikipoo
1662284	GCA_001502535.1	Mycobacterium phage Kimberlium
2079420	GCA_002958775.1	Mycobacterium phage Kimchi
2024295	GCA_002628705.1	Mycobacterium phage Kimona
1679527	GCA_002756715.1	Mycobacterium phage Kinbote
2502419	GCA_004147545.1	Mycobacterium phage Kingmustik0402
1970794	GCA_002621225.1	Mycobacterium phage Kingsley
2015884	GCA_002626905.1	Mycobacterium phage Kingsolomon
2250422	GCA_003364615.1	Mycobacterium phage KingTut
1471544	GCA_002756115.1	Mycobacterium phage KingVeVeVe
2507856	GCA_004139015.1	Mycobacterium phage KiSi
2108125	GCA_003014025.1	Mycobacterium phage KittenMittens
2041536	GCA_002744775.1	Mycobacterium phage Klein
2301707	GCA_003442795.1	Mycobacterium phage KlimbOn
1034138	GCA_002600325.1	Mycobacterium phage KLucky39
2234029	GCA_003308275.1	Mycobacterium phage Koella
2041537	GCA_002744815.1	Mycobacterium phage Koguma
2047840	GCA_002956355.1	Mycobacterium phage Koko
563121	GCA_000882195.1	Mycobacterium phage Konstantine
2283296	GCA_003366835.1	Mycobacterium phage Krakatau
1927029	GCA_002617705.1	Mycobacterium phage Kratark
1606763	GCA_001505055.1	Mycobacterium phage Kratio
2301700	GCA_003442815.1	Mycobacterium phage KristaRAM
2517956	GCA_004520835.1	Mycobacterium phage Kristoff
2015820	GCA_002625665.1	Mycobacterium phage Krueger
2015885	GCA_002626925.1	Mycobacterium phage Krypton555
2015833	GCA_002625925.1	Mycobacterium phage Ksquared
2041344	GCA_002743955.1	Mycobacterium phage Kumao
2499045	GCA_004007545.1	Mycobacterium phage Kwadwo
2250302	GCA_003365355.1	Mycobacterium phage Kwksand96
2517957	GCA_004520855.1	Mycobacterium phage Kyee
2094135	GCA_003014325.1	Mycobacterium phage Kykar
2250404	GCA_003364995.1	Mycobacterium phage Labeouficaum
1718166	GCA_001482935.1	Mycobacterium phage LadyBird
2079569	GCA_002958995.1	Mycobacterium phage Lambert1
1468169	GCA_000918295.1	Mycobacterium phage Lamina13
1560285	GCA_001503715.1	Mycobacterium phage Larenn
1541886	GCA_002600285.1	Mycobacterium phage Lasso
2015886	GCA_002626945.1	Mycobacterium phage LastHope
2094136	GCA_003014335.1	Mycobacterium phage LaterM
1874015	GCA_002757915.1	Mycobacterium phage Laurie

2099634	GCA_002997585.1	Mycobacterium phage LeeLot
1897528	GCA_002758175.1	Mycobacterium phage Lego3393
2499046	GCA_004008775.1	Mycobacterium phage Legolas
2419612	GCA_003723115.1	Mycobacterium phage LeMond
2023958	GCA_002627385.1	Mycobacterium phage LemonSlice
2500805	GCA_004147345.1	Mycobacterium phage Lemur
1327952	GCA_000910295.1	Mycobacterium phage Leo
1916120	GCA_002615825.1	Mycobacterium phage LeoAvram
2126952	GCA_003051505.1	Mycobacterium phage Leston
2081630	GCA_002990435.1	Mycobacterium phage LifeSavor
1070990	GCA_000919275.1	Mycobacterium phage Lilac
2315542	GCA_003613175.1	Mycobacterium phage LilDestine
2041538	GCA_002744835.1	Mycobacterium phage LilHazelnut
2126815	GCA_003023995.1	Mycobacterium phage Lilith
2041539	GCA_002744855.1	Mycobacterium phage LilPharaoh
2315709	GCA_003614415.1	Mycobacterium phage LilTurb
1897546	GCA_002613445.1	Mycobacterium phage LindNT
1074307	GCA_000920095.1	Mycobacterium phage LinStu
1913042	GCA_002614825.1	Mycobacterium phage LittleB
1340818	GCA_000912575.1	Mycobacterium phage LittleCherry
1881139	GCA_002611965.1	Mycobacterium phage LittleGuy
2301615	GCA_003601195.1	Mycobacterium phage LittleLaf
1873888	GCA_002630065.1	Mycobacterium phage Littleton
1527533	GCA_002756335.1	Mycobacterium phage LizLemon
2419980	GCA_003692275.1	Mycobacterium phage Lizziana
1541823	GCA_001502455.1	Mycobacterium phage Llama
1837841	GCA_002709625.1	Mycobacterium phage Loadrie
2502483	GCA_004148925.1	Mycobacterium phage Loanshark
2015821	GCA_002625685.1	Mycobacterium phage Lokk
1698711	GCA_001504155.1	Mycobacterium phage Lolly9
2041540	GCA_002744875.1	Mycobacterium phage Longacauda
2126809	GCA_003024055.1	Mycobacterium phage Lopton
2517958	GCA_004520335.1	Mycobacterium phage Lorenzo
1815969	GCA_001754825.1	Mycobacterium phage Loser
1897523	GCA_002613065.1	Mycobacterium phage Louie6
2047841	GCA_002956365.1	Mycobacterium phage LouisV14
663556	GCA_002630445.1	Mycobacterium phage LRRHood
1647300	GCA_001501235.1	Mycobacterium phage Luchador
2014349	GCA_002625285.1	Mycobacterium phage Lucky2013
2488973	GCA_003867935.1	Mycobacterium phage LuckyMarjie
2079421	GCA_002958785.1	Mycobacterium phage LugYA
1913044	GCA_002614865.1	Mycobacterium phage Lukilu
2027896	GCA_002629465.1	Mycobacterium phage Lulumae
1701852	GCA_002607305.1	Mycobacterium phage Lumos
2027897	GCA_002629485.1	Mycobacterium phage MadamMonkfish
2301708	GCA_003442835.1	Mycobacterium phage MadMarie
1675552	GCA_002601505.1	Mycobacterium phage Madruga
2499048	GCA_004007435.1	Mycobacterium phage Mag7
2250361	GCA_003341355.1	Mycobacterium phage Magnar
1881141	GCA_002612005.1	Mycobacterium phage Magnito

2517959	GCA_004520315.1	Mycobacterium phage Mainiac
2024296	GCA_002628725.1	Mycobacterium phage Majeke
1873889	GCA_001743995.1	Mycobacterium phage Makemake
2500574	GCA_004015765.1	Mycobacterium phage Malec
1821720	GCA_002599865.1	Mycobacterium phage Malinsilva
1567472	GCA_000955395.1	Mycobacterium phage Malithi
1897482	GCA_002758135.1	Mycobacterium phage Mana
1486403	GCA_000922975.1	Mycobacterium phage Manad
2484211	GCA_003722675.1	Mycobacterium phage Manda
2484212	GCA_003722695.1	Mycobacterium phage Mangethe
2283297	GCA_003366795.1	Mycobacterium phage Mantra
1897445	GCA_002612545.1	Mycobacterium phage Marcoliusprime
1897496	GCA_002612745.1	Mycobacterium phage Margo
1792228	GCA_002599845.1	Mycobacterium phage Marie
2250423	GCA_003364655.1	Mycobacterium phage Marius
2488787	GCA_003867115.1	Mycobacterium phage Marley1013
2301709	GCA_003442855.1	Mycobacterium phage Maroc7
1527516	GCA_001505795.1	Mycobacterium phage MarQuardt
1034139	GCA_002633455.1	Mycobacterium phage Marvin
1933690	GCA_002758315.1	Mycobacterium phage Maskar
2182398	GCA_003183305.1	Mycobacterium phage Mattes
1701799	GCA_002607365.1	Mycobacterium phage Maverick
1955423	GCA_002619085.1	Mycobacterium phage Maxxinista
2517960	GCA_004520175.1	Mycobacterium phage Mazhar510
1805460	GCA_002757335.1	Mycobacterium phage McFly
2094137	GCA_003014345.1	Mycobacterium phage McGuire
2488974	GCA_003867915.1	Mycobacterium phage McWolfish
2499049	GCA_004007455.1	Mycobacterium phage Mecca
1340834	GCA_002597965.1	Mycobacterium phage Medusa
1056832	GCA_002597865.1	Mycobacterium phage MeeZee
2108126	GCA_003013895.1	Mycobacterium phage Megabear
2301562	GCA_003442355.1	Mycobacterium phage Megamind
2099636	GCA_002997605.1	Mycobacterium phage Megatron
2163593	GCA_003143095.1	Mycobacterium phage Melissauren88
2510578	GCA_004147905.1	Mycobacterium phage Melpomini
1429792	GCA_002755915.1	Mycobacterium phage Melvin
2099637	GCA_002997615.1	Mycobacterium phage Mendokysei
2315710	GCA_003614455.1	Mycobacterium phage Mesh1
2500806	GCA_004148145.1	Mycobacterium phage MetalQZJ
1327779	GCA_002755635.1	Mycobacterium phage Methuselah
1567005	GCA_001505975.1	Mycobacterium phage MiaZeal
2283257	GCA_003366235.1	Mycobacterium phage MichaelPhcott
1445726	GCA_000917275.1	Mycobacterium phage MichelleMyBell
2234076	GCA_003340915.1	Mycobacterium phage Michley
2126953	GCA_003051525.1	Mycobacterium phage Midas2
2126807	GCA_003034825.1	Mycobacterium phage MikeLiesIn
2041542	GCA_002744895.1	Mycobacterium phage Mikota
2015847	GCA_002626185.1	Mycobacterium phage Miley16
2315711	GCA_003614495.1	Mycobacterium phage MilleniumForce
1916116	GCA_002615785.1	Mycobacterium phage Millski

1567473	GCA_000954675.1	Mycobacterium phage Milly
1647311	GCA_001551045.1	Mycobacterium phage Mindy
1527513	GCA_000955095.1	Mycobacterium phage Minerva
2517961	GCA_004520715.1	Mycobacterium phage Miramae
2126810	GCA_003024035.1	Mycobacterium phage Misha28
2250303	GCA_003365395.1	Mycobacterium phage Misomonster
2530133	GCA_004338435.1	Mycobacterium phage MissDaisy
2024297	GCA_002628745.1	Mycobacterium phage MissWhite
2041541	GCA_002956115.1	Mycobacterium phage MISSy
2502433	GCA_004147685.1	Mycobacterium phage MisterCuddles
1897491	GCA_002758155.1	Mycobacterium phage MitKao
1917488	GCA_002617025.1	Mycobacterium phage Mitti
1777060	GCA_002609025.1	Mycobacterium phage MkaliMitinis3
2530188	GCA_004340585.1	Mycobacterium phage Moldemort
1647303	GCA_002598585.1	Mycobacterium phage Momo
1088865	GCA_000918815.1	Mycobacterium phage MoMoMixon
2108127	GCA_003013925.1	Mycobacterium phage MooMoo
1647309	GCA_001502595.1	Mycobacterium phage MOOREtheMARYer
2250405	GCA_003365015.1	Mycobacterium phage Moose
1084721	GCA_002600245.1	Mycobacterium phage Morgushi
2488975	GCA_003867895.1	Mycobacterium phage Morizzled23
2027898	GCA_002629505.1	Mycobacterium phage Morpher26
2094138	GCA_003014355.1	Mycobacterium phage Morrow
2234030	GCA_003308295.1	Mycobacterium phage Mortcellus
2250362	GCA_003341075.1	Mycobacterium phage Morty
2250304	GCA_003365415.1	Mycobacterium phage Morty007
2099638	GCA_002997625.1	Mycobacterium phage Mosaic
1429913	GCA_002603805.1	Mycobacterium phage Mosby
1471542	GCA_000919975.1	Mycobacterium phage MosMoris
2250406	GCA_003365035.1	Mycobacterium phage Mowgli
2126787	GCA_003024085.1	Mycobacterium phage MPhalcon
2099635	GCA_002997635.1	Mycobacterium phage MPlant7149
2499047	GCA_004007495.1	Mycobacterium phage MRabcd
1927020	GCA_002617525.1	Mycobacterium phage MrMagoo
2234077	GCA_003340935.1	Mycobacterium phage Mryolo
2301606	GCA_003601215.1	Mycobacterium phage MuchMore
1340829	GCA_000913315.1	Mycobacterium phage Muddy
1718600	GCA_001503275.1	Mycobacterium phage Mufasa
2301607	GCA_003601235.1	Mycobacterium phage Mulan
1805459	GCA_001755265.1	Mycobacterium phage Mulciber
2530134	GCA_004339225.1	Mycobacterium phage Munch
1897540	GCA_002613325.1	Mycobacterium phage Mundrea
1084720	GCA_002630565.1	Mycobacterium phage Murdoc
2041543	GCA_002744915.1	Mycobacterium phage Murica
1327939	GCA_000909035.1	Mycobacterium phage Murphy
1560286	GCA_001504515.1	Mycobacterium phage Murucutumbu
2250381	GCA_003341635.1	Mycobacterium phage Mutante
2250305	GCA_003365435.1	Mycobacterium phage Mynx
2024303	GCA_002628805.1	Mycobacterium phage MyraDee
546805	GCA_000880475.1	Mycobacterium phage Myrna

1805458	GCA_002609285.1	Mycobacterium phage Myxus
2126816	GCA_003024005.1	Mycobacterium phage Naca
1235692	GCA_002600385.1	Mycobacterium phage Nacho
2502466	GCA_004148425.1	Mycobacterium phage Naija
2530135	GCA_004339905.1	Mycobacterium phage Naira
2108128	GCA_003013945.1	Mycobacterium phage Nairb
1391180	GCA_000914075.1	Mycobacterium phage Nala
2502468	GCA_004148525.1	Mycobacterium phage Napoleon13
1088866	GCA_000917815.1	Mycobacterium phage Nappy
1777062	GCA_002609065.1	Mycobacterium phage NaSiaTalie
1897547	GCA_002613465.1	Mycobacterium phage Nazo
2315712	GCA_003614515.1	Mycobacterium phage NearlyHeadless
2484213	GCA_003722735.1	Mycobacterium phage Nebkiss
2502452	GCA_004148745.1	Mycobacterium phage Nebs
2283298	GCA_003366775.1	Mycobacterium phage NEHalo
1679530	GCA_001500435.1	Mycobacterium phage NelitzaMV
2163594	GCA_003143115.1	Mycobacterium phage Nemo27
2530136	GCA_004339845.1	Mycobacterium phage Nenae
1647308	GCA_001500555.1	Mycobacterium phage Nerujay
1327766	GCA_000909075.1	Mycobacterium phage Newman
1675553	GCA_001502315.1	Mycobacterium phage Nhonho
2510585	GCA_004148025.1	Mycobacterium phage Nibb
2015887	GCA_002626965.1	Mycobacterium phage Nicholas
2015888	GCA_002626985.1	Mycobacterium phage Nicholasp3
2315713	GCA_003614535.1	Mycobacterium phage NicoleTera
2502474	GCA_004148645.1	Mycobacterium phage Nidhogg
543152	GCA_000879635.1	Mycobacterium phage Nigel
2517936	GCA_004520755.1	Mycobacterium Phage Niklas
2108129	GCA_003013905.1	Mycobacterium phage Nilo
2301567	GCA_003442375.1	Mycobacterium phage Nimbo
2510511	GCA_004149025.1	Mycobacterium phage Nimrod
2301710	GCA_003442915.1	Mycobacterium phage Nivrat
2250382	GCA_003341675.1	Mycobacterium phage Niza
2517962	GCA_004520895.1	Mycobacterium phage Noella
2502470	GCA_004148565.1	Mycobacterium phage NoodleTree
2500575	GCA_004016025.1	Mycobacterium phage Norbert
2502424	GCA_004147465.1	Mycobacterium phage Norm
2250383	GCA_003341715.1	Mycobacterium phage NormanBulbieJr
2178921	GCA_003182825.1	Mycobacterium phage NorthStar
1673875	GCA_002606885.1	Mycobacterium phage NoSleep
2250384	GCA_003345085.1	Mycobacterium phage NotAPhaseMom
1089130	GCA_002601805.1	Mycobacterium phage Nova
2099639	GCA_002997655.1	Mycobacterium phage Nozo
2079573	GCA_002997405.1	Mycobacterium phage NuevoMundo
1458713	GCA_002755975.1	Mycobacterium phage Numberten
1445714	GCA_000916375.1	Mycobacterium phage Nyxis
1445727	GCA_000917235.1	Mycobacterium phage Oaker
1445715	GCA_000916475.1	Mycobacterium phage Obama12
1698367	GCA_002756875.1	Mycobacterium phage OBUpride
2502425	GCA_004147485.1	Mycobacterium phage Ochi17

2250363	GCA_003341195.1	Mycobacterium phage OctaviousRex
1340711	GCA_002603745.1	Mycobacterium phage Odin
2099640	GCA_002997535.1	Mycobacterium phage Ogopogo
2040289	GCA_002629825.1	Mycobacterium phage OKCentral2016
1486473	GCA_000923655.1	Mycobacterium phage OkiRoe
2099641	GCA_002997665.1	Mycobacterium phage OlanP
2079284	GCA_002958435.1	Mycobacterium phage OldBen
2315543	GCA_003614375.1	Mycobacterium phage Olga
1052671	GCA_000919295.1	Mycobacterium phage Oline
2163595	GCA_003143135.1	Mycobacterium phage Olive
2099642	GCA_002997675.1	Mycobacterium phage OliverWalter
2250331	GCA_003365875.1	Mycobacterium phage Ollie
2250364	GCA_003341175.1	Mycobacterium phage OlympiaSaint
1541819	GCA_001500415.1	Mycobacterium phage Omnicron
2499050	GCA_004007675.1	Mycobacterium phage Omniscient
2178922	GCA_003182845.1	Mycobacterium phage Oogway
1034141	GCA_003004885.1	Mycobacterium phage Oosterbaan
2079422	GCA_002958805.1	Mycobacterium phage Opia
2530137	GCA_004338915.1	Mycobacterium phage Orange
1647310	GCA_002605805.1	Mycobacterium phage OrangeOswald
373409	GCA_000869205.1	Mycobacterium phage Orion
2419613	GCA_003723135.1	Mycobacterium phage Oscar
1035482	GCA_001505495.1	Mycobacterium phage OSmaximus
1673889	GCA_001501115.1	Mycobacterium phage Ovechkin
2126954	GCA_003051545.1	Mycobacterium phage OwlsT2W
1897542	GCA_002613365.1	Mycobacterium phage PacerPaul
2315544	GCA_003614395.1	Mycobacterium phage Paito
1916117	GCA_002615805.1	Mycobacterium phage Palestino
2126789	GCA_003024095.1	Mycobacterium phage Panamaxus
1821537	GCA_001755245.1	Mycobacterium phage Panchino
2094139	GCA_003014365.1	Mycobacterium phage Paola
2301616	GCA_003601295.1	Mycobacterium phage Paperbeatsrock
1873891	GCA_001745315.1	Mycobacterium phage Papez
1383056	GCA_000911795.1	Mycobacterium phage Papyrus
1718171	GCA_001503295.1	Mycobacterium phage Pari
2234031	GCA_003308895.1	Mycobacterium phage ParkTD
2530138	GCA_004339345.1	Mycobacterium phage Parmesanjohn
2484214	GCA_003722755.1	Mycobacterium phage Pat3
1074308	GCA_000920075.1	Mycobacterium phage Patience
2530139	GCA_004338495.1	Mycobacterium phage Patt
2502462	GCA_004148345.1	Mycobacterium phage Patter
1327773	GCA_000909935.1	Mycobacterium phage PattyP
373410	GCA_000867425.1	Mycobacterium phage PBI1
1640882	GCA_002756575.1	Mycobacterium phage PDRPv
1640883	GCA_002756595.1	Mycobacterium phage PDRPxv
2015848	GCA_002626205.1	Mycobacterium phage Peanam
1325953	GCA_000909975.1	Mycobacterium phage PegLeg
1873950	GCA_002604925.1	Mycobacterium phage Penny1
1735466	GCA_001470075.1	Mycobacterium phage Pepe
2517963	GCA_004520875.1	Mycobacterium phage PeterPeter

2502417	GCA_004147385.1	Mycobacterium phage Petp2012
1927018	GCA_002617485.1	Mycobacterium phage Petruchio
213236	GCA_002758395.1	Mycobacterium phage PG1
2163596	GCA_003094395.1	Mycobacterium phage PGHhamlin
2047842	GCA_002956375.1	Mycobacterium phage Ph8s
2027899	GCA_002629525.1	Mycobacterium phage Phabba
2099447	GCA_002997705.1	Mycobacterium phage Phacado
1897487	GCA_002612665.1	Mycobacterium phage Phaeder
546184	GCA_000874685.1	Mycobacterium phage Phaedrus
2250306	GCA_003365455.1	Mycobacterium phage Phaja
1673885	GCA_002756635.1	Mycobacterium phage Phamished
1897438	GCA_002612425.1	Mycobacterium phage PhancyPhin
1486426	GCA_000920855.1	Mycobacterium phage Phantastic
2301568	GCA_003442395.1	Mycobacterium phage PHappiness
2530140	GCA_004340045.1	Mycobacterium phage Pharaoh
2301711	GCA_003442935.1	Mycobacterium phage Phareon
1897505	GCA_002612945.1	Mycobacterium phage Pharsalus
2041544	GCA_002745355.1	Mycobacterium phage Phasih
1391181	GCA_000913235.1	Mycobacterium phage PhatBacter
1873890	GCA_002604345.1	Mycobacterium phage PhatCats2014
1698356	GCA_001503395.1	Mycobacterium phage Phatniss
1327937	GCA_000908435.1	Mycobacterium phage Phaux
1647302	GCA_001502615.1	Mycobacterium phage Phayonce
1383055	GCA_000913275.1	Mycobacterium phage Phelemich
2015822	GCA_002625705.1	Mycobacterium phage PhelpsODU
2079423	GCA_002958815.1	Mycobacterium phage PhenghisKhan
2079424	GCA_002958825.1	Mycobacterium phage Phergie
2301569	GCA_003442415.1	Mycobacterium phage PherrisBueller
2517964	GCA_004520275.1	Mycobacterium phage Phighter1804
2047843	GCA_002956385.1	Mycobacterium phage Philonius
1034143	GCA_001505255.1	Mycobacterium phage Phipps
2163597	GCA_003143155.1	Mycobacterium phage Phish
2250424	GCA_003364695.1	Mycobacterium phage PhishRPhriends
2301555	GCA_003575785.1	Mycobacterium phage Phlegm
1690684	GCA_001470455.1	Mycobacterium phage Phlei
2234032	GCA_003308335.1	Mycobacterium phage Phleuron
2047844	GCA_002956395.1	Mycobacterium phage Phlorence
591487	GCA_000882015.1	Mycobacterium phage Phlyer
2530141	GCA_004340025.1	Mycobacterium phage Phoebus
2081626	GCA_002990465.1	Mycobacterium phage Phonnegut
2502446	GCA_004148445.1	Mycobacterium phage Phontbonne
2041545	GCA_002744935.1	Mycobacterium phage Phox
1567451	GCA_002605105.1	Mycobacterium phage Phoxy
2015823	GCA_002625725.1	Mycobacterium phage Phrank
2182401	GCA_003183365.1	Mycobacterium phage PhrankReynolds
1821541	GCA_001754805.1	Mycobacterium phage Phrann
2250365	GCA_003341155.1	Mycobacterium phage Phranny
1698359	GCA_002756815.2	Mycobacterium phage Phreak
1698359	GCA_002756815.1	Mycobacterium phage Phreak
2250385	GCA_003341735.1	Mycobacterium phage PhrodoBaggins

1354512	GCA_000912695.1	Mycobacterium phage PhrostyMug
1327774	GCA_000908455.1	Mycobacterium phage Phrux
2041546	GCA_002744955.1	Mycobacterium phage Phunky
2502439	GCA_004147765.1	Mycobacterium phage Phusco
2079572	GCA_002997415.1	Mycobacterium phage Pier
1235693	GCA_002600365.2	Mycobacterium phage Piglet
1235693	GCA_002600365.1	Mycobacterium phage Piglet
2283299	GCA_003366755.1	Mycobacterium phage PinheadLarry
1897426	GCA_002758075.1	Mycobacterium phage Pinkman
2502451	GCA_004148705.1	Mycobacterium phage PinkPlastic
2517965	GCA_004520475.1	Mycobacterium phage Pinnie
1498204	GCA_000921935.1	Mycobacterium phage Pinto
1089131	GCA_002598485.1	Mycobacterium phage Pio
1698417	GCA_001504995.1	Mycobacterium phage Pioneer
2182402	GCA_003183385.1	Mycobacterium phage Pipcraft
373413	GCA_000869225.1	Mycobacterium phage Pipefish
2517966	GCA_004520775.1	Mycobacterium phage Piper2020
2079571	GCA_002959005.1	Mycobacterium phage Pippin
1983576	GCA_002623765.1	Mycobacterium phage Pippy
1567474	GCA_002601665.1	Mycobacterium phage Pipsqueak
1821540	GCA_002757595.1	Mycobacterium phage Pipsqueaks
1527520	GCA_001505815.1	Mycobacterium phage Piro94
2126722	GCA_003034805.1	Mycobacterium phage Pistachio
2283258	GCA_003366255.1	Mycobacterium phage Pita2
2502455	GCA_004147865.1	Mycobacterium phage Pivoine
2250407	GCA_003365055.1	Mycobacterium phage Placalicious
2250425	GCA_003364715.1	Mycobacterium phage Plagueis
1079895	GCA_000917515.1	Mycobacterium phage Pleione
2499051	GCA_004008805.1	Mycobacterium phage Plmatters
373411	GCA_000868285.1	Mycobacterium phage PLot
2301712	GCA_003442975.1	Mycobacterium phage Podrick
2500793	GCA_004147265.1	Mycobacterium phage Poenanya
2502448	GCA_004148505.1	Mycobacterium phage Pollywog
1897543	GCA_002613385.1	Mycobacterium phage Pomar16
2483676	GCA_003722975.1	Mycobacterium phage Popcicle
1675554	GCA_001470755.1	Mycobacterium phage Pops
1698712	GCA_001505615.1	Mycobacterium phage PopTart
2006923	GCA_002625045.1	Mycobacterium phage Porcelain
2499052	GCA_004008615.1	Mycobacterium phage PotatoSplit
1818107	GCA_002600205.1	Mycobacterium phage Potter
1552733	GCA_002605025.1	Mycobacterium phage Power
2077134	GCA_002958325.1	Mycobacterium phage PP
2070497	GCA_002958265.1	Mycobacterium phage PR
1916121	GCA_002615845.1	Mycobacterium phage Prann
543153	GCA_000874645.1	Mycobacterium phage Predator
2182403	GCA_003183425.1	Mycobacterium phage Priamo
2081627	GCA_002997885.1	Mycobacterium phage Priscilla
2484215	GCA_003722775.1	Mycobacterium phage Prithvi
2250366	GCA_003341135.1	Mycobacterium phage Priya
2094140	GCA_003014375.1	Mycobacterium phage ProfessorX

663558	GCA_002600225.1	Mycobacterium phage Puhltonio
2126723	GCA_003034795.1	Mycobacterium phage Puppy
2530142	GCA_004339885.1	Mycobacterium phage Purgamenstris
1983577	GCA_002623785.1	Mycobacterium phage PurpleHaze
2502469	GCA_004148545.1	Mycobacterium phage QBert
1897484	GCA_002612625.1	Mycobacterium phage Qobbit
2499053	GCA_004007815.1	Mycobacterium phage QueenBeane
2301544	GCA_003442135.1	Mycobacterium phage QueenBeesly
2301570	GCA_003442435.1	Mycobacterium phage QuickMath
1675555	GCA_001502395.1	Mycobacterium phage Quico
1354514	GCA_000911035.1	Mycobacterium phage Quink
1551642	GCA_002605005.1	Mycobacterium phage QuinnKiro
373414	GCA_000867445.1	Mycobacterium phage Qyrzula
2530143	GCA_004338645.1	Mycobacterium phage Rabbs
2283291	GCA_003423305.1	Mycobacterium phage Rabinovish
2499054	GCA_004006915.1	Mycobacterium phage Raela
2079593	GCA_002959185.1	Mycobacterium phage RagingRooster
2502472	GCA_004148605.1	Mycobacterium phage Rahel
2502444	GCA_004148205.1	Mycobacterium phage Rajelicia
1035483	GCA_002601105.1	Mycobacterium phage Rakim
2301556	GCA_003442155.1	Mycobacterium phage Rando14
2250426	GCA_003364735.1	Mycobacterium phage Reba
1079894	GCA_000916655.1	Mycobacterium phage Redi
2499055	GCA_004008335.1	Mycobacterium phage RedMaple
1340709	GCA_000910855.1	Mycobacterium phage Redno2
2500794	GCA_004147305.1	Mycobacterium phage RedRaider77
711470	GCA_000926775.1	Mycobacterium phage RedRock
2517967	GCA_004520635.1	Mycobacterium phage Refuge
2182406	GCA_003183465.1	Mycobacterium phage Relief
2079285	GCA_002958445.1	Mycobacterium phage Rem711
2108130	GCA_003013985.1	Mycobacterium phage Remy19
2510512	GCA_004149065.1	Mycobacterium phage Renaissance
2301701	GCA_003442995.1	Mycobacterium phage Renaud18
1340828	GCA_002604045.1	Mycobacterium phage Reprobate
2341078	GCA_003722435.1	Mycobacterium phage Reptar3000
1034115	GCA_002624045.1	Mycobacterium phage Rey
2488788	GCA_003867135.1	Mycobacterium phage Rhynn
1458846	GCA_000914615.1	Mycobacterium phage RhynO
1927021	GCA_002617545.1	Mycobacterium phage Rich
1071506	GCA_000920115.1	Mycobacterium phage RidgeCB
2488976	GCA_003867775.1	Mycobacterium phage Riggan
2530144	GCA_004339945.1	Mycobacterium phage Ringer
2341079	GCA_003722455.1	Mycobacterium phage Riparian
2027900	GCA_002629545.1	Mycobacterium phage RitaG
2108119	GCA_003014215.1	Mycobacterium phage RiverMonster
546806	GCA_000881495.1	Mycobacterium phage Rizal
2530145	GCA_004339965.1	Mycobacterium phage Robyn
1034144	GCA_002633545.1	Mycobacterium phage Rockstar
2250427	GCA_003364775.1	Mycobacterium phage Rohr
2499056	GCA_004008485.1	Mycobacterium phage Roliet

2510576	GCA_004149005.1	Mycobacterium phage RoMag
2502453	GCA_004148765.1	Mycobacterium phage RomaT
1818374	GCA_002597765.1	Mycobacterium phage Romney
1555234	GCA_002605065.1	Mycobacterium phage RonRayGun
1897504	GCA_002612925.1	Mycobacterium phage Roosevelt
1897504	GCA_002612925.2	Mycobacterium phage Roosevelt
2315615	GCA_003613855.1	Mycobacterium phage Roots515
2499057	GCA_004007755.1	Mycobacterium phage Roscoe
205874	GCA_000842345.1	Mycobacterium phage Rosebush
2250307	GCA_003365495.1	Mycobacterium phage Roy17
2250386	GCA_003341755.1	Mycobacterium phage Rubeelu
2301713	GCA_003443015.1	Mycobacterium phage Ruby
1698441	GCA_001504175.1	Mycobacterium phage Rufus
2315714	GCA_003614555.1	Mycobacterium phage Ruin
2099448	GCA_002997755.1	Mycobacterium phage Ruotula
2283292	GCA_003366735.1	Mycobacterium phage Ryadel
1445728	GCA_000915735.1	Mycobacterium phage Saal
2283293	GCA_003366715.1	Mycobacterium phage Sabella
1211284	GCA_002597925.1	Mycobacterium phage Sabertooth
2234078	GCA_003340955.1	Mycobacterium phage Sabia
1913037	GCA_002614735.1	Mycobacterium phage Sabinator
2315616	GCA_003613875.1	Mycobacterium phage Saguaro
2301617	GCA_003614615.1	Mycobacterium phage Salacia
2530147	GCA_004339825.1	Mycobacterium phage Salz
2499058	GCA_004007775.1	Mycobacterium phage Samaymay
2126955	GCA_003051565.1	Mycobacterium phage SamScheppers
2301618	GCA_003601315.1	Mycobacterium phage Samty
1912977	GCA_002614605.1	Mycobacterium phage SamuelLPlaqson
2250367	GCA_003341115.1	Mycobacterium phage Sandalphon
1340827	GCA_000912115.1	Mycobacterium phage SarFire
1354513	GCA_002603845.1	Mycobacterium phage SargentShorty9
2041547	GCA_002744975.1	Mycobacterium phage Sassay
1958910	GCA_002619245.1	Mycobacterium phage SassyB
2499059	GCA_004007835.1	Mycobacterium phage SassyCat97
2419614	GCA_003723155.1	Mycobacterium phage Sauce
1567475	GCA_000954315.1	Mycobacterium phage Sbash
2419615	GCA_003723195.1	Mycobacterium phage Scarlett
2483721	GCA_003722475.1	Mycobacterium phage Schadenfreude
2081628	GCA_002997895.1	Mycobacterium phage Scherzo
2126937	GCA_003034775.1	Mycobacterium phage Schiebel
2126790	GCA_003024075.1	Mycobacterium phage SchoolBus
701455	GCA_002758895.1	Mycobacterium phage Scoot17C
2517968	GCA_004520235.1	Mycobacterium phage Scorpia
546807	GCA_000883055.2	Mycobacterium phage ScottMcG
546807	GCA_000883055.1	Mycobacterium phage ScottMcG
2099449	GCA_002997765.1	Mycobacterium phage Scowl
1327775	GCA_002755615.1	Mycobacterium phage SDcharge11
1458714	GCA_000918335.1	Mycobacterium phage Seabiscuit
1698713	GCA_001504955.1	Mycobacterium phage Seagreen
1052672	GCA_002624925.1	Mycobacterium phage Sebata

1034146	GCA_002633475.1	Mycobacterium phage Send513
2301619	GCA_003601435.1	Mycobacterium phage Serendipitous
1035484	GCA_002755015.1	Mycobacterium phage Serendipity
1701853	GCA_001503315.1	Mycobacterium phage Serenity
1235694	GCA_002600345.1	Mycobacterium phage Serpentine
1327776	GCA_000907435.1	Mycobacterium phage Severus
2126788	GCA_003024065.1	Mycobacterium phage SgtBeansprout
1034124	GCA_002597845.1	Mycobacterium phage Shaka
1983447	GCA_002623505.1	Mycobacterium phage Shandong1
2500807	GCA_004148065.1	Mycobacterium phage Shaobing
2502426	GCA_004147565.1	Mycobacterium phage Shaqnato
1036615	GCA_002624785.1	Mycobacterium phage Shauna1
1036615	GCA_002624785.2	Mycobacterium phage Shauna1
1647313	GCA_001501975.1	Mycobacterium phage ShedlockHolmes
1589274	GCA_001504315.1	Mycobacterium phage Sheen
2041548	GCA_002744995.1	Mycobacterium phage Sheila
2510579	GCA_004147925.1	Mycobacterium phage Shelob
2250308	GCA_003365555.1	Mycobacterium phage ShereKhan
2502467	GCA_004148485.1	Mycobacterium phage ShiaLabeouf
1036616	GCA_002624765.1	Mycobacterium phage ShiLan
1821727	GCA_001755585.1	Mycobacterium phage Shipwreck
1296647	GCA_001470295.1	Mycobacterium phage ShiVal
2502421	GCA_004147505.1	Mycobacterium phage Shnickers
1340835	GCA_002598525.1	Mycobacterium phage Shrimp
2530149	GCA_004340005.1	Mycobacterium phage ShrimpFriedEgg
2283259	GCA_003366555.1	Mycobacterium phage Sibs6
1567476	GCA_002756475.1	Mycobacterium phage Sigman
2234033	GCA_003308375.1	Mycobacterium phage Silvafighter
2517969	GCA_004520255.1	Mycobacterium phage Silverleaf
2250332	GCA_003365915.1	Mycobacterium phage Simpliphy
1933771	GCA_002955015.1	Mycobacterium phage SimranZ1
1034116	GCA_002624745.1	Mycobacterium phage SirDuracell
1034117	GCA_002601065.1	Mycobacterium phage SirHarley
2015824	GCA_002625745.1	Mycobacterium phage SirPhilip
1327777	GCA_000909915.1	Mycobacterium phage SiSi
1821539	GCA_002609585.1	Mycobacterium phage SkinnyPete
2126791	GCA_003024105.1	Mycobacterium phage Skipitt
701456	GCA_000917675.1	Mycobacterium phage SkiPole
1698440	GCA_002604745.2	Mycobacterium phage Slarp
1698440	GCA_002604745.1	Mycobacterium phage Slarp
2015889	GCA_002627005.1	Mycobacterium phage Slimphazie
2027901	GCA_002629565.1	Mycobacterium phage Smairt
2502429	GCA_004147625.1	Mycobacterium phage SmallFry
1673873	GCA_001500475.1	Mycobacterium phage Smeadley
2108131	GCA_003014015.1	Mycobacterium phage Smeagol
2530150	GCA_004339405.1	Mycobacterium phage Smurph
2250333	GCA_003365935.1	Mycobacterium phage Snape
2041549	GCA_002745015.1	Mycobacterium phage SnapTap
1873887	GCA_001744515.1	Mycobacterium phage Sneeze
1698714	GCA_001503335.1	Mycobacterium phage Snenia

555603	GCA_000880495.1	Mycobacterium phage Solon
2499060	GCA_004008675.1	Mycobacterium phage Solosis
2499061	GCA_004008835.1	Mycobacterium phage Sophia
1458725	GCA_000925895.1	Mycobacterium phage Soto
2081629	GCA_002997905.1	Mycobacterium phage Sotrice96
1739966	GCA_001470615.1	Mycobacterium phage Sparkdehlily
1527493	GCA_000955455.1	Mycobacterium phage Sparky
1147141	GCA_002624725.1	Mycobacterium phage Spartacus
2488977	GCA_003867755.1	Mycobacterium phage Spartan300
2502436	GCA_004148985.1	Mycobacterium phage Specks
1567452	GCA_002605125.1	Mycobacterium phage Spike509
2502422	GCA_004147425.1	Mycobacterium phage SpikeBT
2301571	GCA_003442475.1	Mycobacterium phage Spikelee
2530151	GCA_004338985.1	Mycobacterium phage SpongeBob
2023953	GCA_002627365.1	Mycobacterium phage Spoonbill
2502476	GCA_004148725.1	Mycobacterium phage Sprinklers
546808	GCA_000880555.1	Mycobacterium phage Spud
1718175	GCA_002600305.1	Mycobacterium phage Squid
2488960	GCA_003867735.1	Mycobacterium Phage Squiggle
2027902	GCA_002629585.1	Mycobacterium phage Squint
1527512	GCA_000954375.1	Mycobacterium phage Squirty
2015849	GCA_002626225.1	Mycobacterium phage Stagni
1913110	GCA_002615105.1	Mycobacterium phage StarStuff
1897548	GCA_002613485.1	Mycobacterium phage Stasia
2250309	GCA_003365575.1	Mycobacterium phage Steamy
2015850	GCA_002626245.1	Mycobacterium phage StepMih
2015811	GCA_002625505.1	Mycobacterium phage StevieRay
1089137	GCA_000918675.1	Mycobacterium phage Stinger
2499062	GCA_004007875.1	Mycobacterium phage Struggle
2510577	GCA_004147885.1	Mycobacterium phage Stubby
1414748	GCA_000914795.1	Mycobacterium phage Suffolk
2041550	GCA_002956125.1	Mycobacterium phage Sulley
2126817	GCA_003024015.1	Mycobacterium phage SuperAwesome
1913038	GCA_002614765.1	Mycobacterium phage SuperGrey
2041551	GCA_002956135.1	Mycobacterium phage Superphikiman
2499063	GCA_004008075.1	Mycobacterium phage Surely
2517970	GCA_004520575.1	Mycobacterium phage SwagPigglett
1555235	GCA_001517015.1	Mycobacterium phage SweetiePie
1555235	GCA_001517015.2	Mycobacterium phage SweetiePie
2315545	GCA_003613255.1	Mycobacterium phage Sweets
1527534	GCA_001503555.1	Mycobacterium phage Swirley
1458715	GCA_001470715.1	Mycobacterium phage Swish
2250428	GCA_003364835.1	Mycobacterium phage SwissCheese
1034118	GCA_002624705.1	Mycobacterium phage Switzer
1175504	GCA_000898135.1	Mycobacterium phage SWU1
2077150	GCA_002958345.2	Mycobacterium phage SWU2
2077150	GCA_002958345.1	Mycobacterium phage SWU2
2015890	GCA_002627025.1	Mycobacterium phage Tachez
1897549	GCA_002613505.1	Mycobacterium phage Taheera
1204515	GCA_000925915.1	Mycobacterium phage Taj

1089107	GCA_002600265.1	Mycobacterium phage TallGrassMM
2301545	GCA_003442175.1	Mycobacterium phage Tapioca
1920305	GCA_002617065.1	Mycobacterium phage Taptic
1897500	GCA_002612845.1	Mycobacterium phage Taquito
2283260	GCA_003366575.1	Mycobacterium phage Target
2234079	GCA_003340975.1	Mycobacterium phage Tarynearal
1698420	GCA_001503375.1	Mycobacterium phage Tasp14
1567477	GCA_002599945.1	Mycobacterium phage Taurus
1897424	GCA_002612185.1	Mycobacterium phage TBond007
2250334	GCA_003365955.1	Mycobacterium phage TBrady12
2163598	GCA_003143175.1	Mycobacterium phage TChen
2502423	GCA_004147445.1	Mycobacterium phage Teardrop
1815609	GCA_002757455.1	Mycobacterium phage TeardropMSU
2041552	GCA_002745035.1	Mycobacterium phage Terminus
1897550	GCA_002613525.1	Mycobacterium phage Terror
2079425	GCA_002958835.1	Mycobacterium phage Tesla
1698419	GCA_002607245.1	Mycobacterium phage Texage
1927030	GCA_002617725.1	Mycobacterium phage Thanksgivukkah
1718172	GCA_001504095.1	Mycobacterium phage Theia
1718172	GCA_001504095.2	Mycobacterium phage Theia
1701845	GCA_001505595.1	Mycobacterium phage TheloniousMonk
2047874	GCA_002956525.1	Mycobacterium phage Thespis
1052673	GCA_000919335.1	Mycobacterium phage Thibault
2282910	GCA_003423225.1	Mycobacterium phage Thonko
1567478	GCA_002605205.1	Mycobacterium phage Thor
1032894	GCA_002601085.1	Mycobacterium phage Thora
1089133	GCA_002755115.1	Mycobacterium phage ThreeOh3D2
2530152	GCA_004339985.1	Mycobacterium phage ThreeRngTarjay
2094141	GCA_003014385.1	Mycobacterium phage Thumb
2283261	GCA_003366395.1	Mycobacterium phage Thyatira
1527535	GCA_001501075.1	Mycobacterium phage Tiffany
1161934	GCA_002624665.1	Mycobacterium phage Tiger
2499064	GCA_004007915.1	Mycobacterium phage Timmi
1032895	GCA_002624645.1	Mycobacterium phage Timshel
1958911	GCA_002619265.1	Mycobacterium phage TinaFeyge
1955424	GCA_002619105.1	Mycobacterium phage Tinybot
2488978	GCA_003867715.1	Mycobacterium phage TinyTim
2041553	GCA_002745055.1	Mycobacterium phage TipsytheTRex
1074829	GCA_002597885.1	Mycobacterium phage TiroTheta9
88870	GCA_000837465.1	Mycobacterium phage TM4
2126956	GCA_003051585.1	Mycobacterium phage TNguyen7
1897490	GCA_002612705.1	Mycobacterium phage Todacoro
2250310	GCA_003365595.1	Mycobacterium phage Tomathan
2499065	GCA_004007935.1	Mycobacterium phage TomBombadil
1891703	GCA_001746115.1	Mycobacterium phage Tonenili
1873989	GCA_002604065.1	Mycobacterium phage ToneTone
2126811	GCA_003024045.1	Mycobacterium phage TootsiePop
1897497	GCA_002612775.1	Mycobacterium phage Tortellini
2502442	GCA_004147825.1	Mycobacterium phage Tortoise16
1035485	GCA_001504435.1	Mycobacterium phage Toto

1567479	GCA_002605225.1	Mycobacterium phage Treddle
1701803	GCA_002756955.1	Mycobacterium phage Tres
2024298	GCA_002628765.1	Mycobacterium phage TreyKay
1527536	GCA_000955435.1	Mycobacterium phage Trike
2341048	GCA_004338775.1	Mycobacterium phage Tripl3t
1071503	GCA_000917615.1	Mycobacterium phage Trixie
561999	GCA_000883095.1	Mycobacterium phage Troll4
1340825	GCA_000911855.1	Mycobacterium phage Trouble
2099450	GCA_003013885.1	Mycobacterium phage Trypo
1913043	GCA_002614845.1	Mycobacterium phage Tuco
1071504	GCA_000919255.1	Mycobacterium phage Turbido
1701849	GCA_001504935.1	Mycobacterium phage Turj99
439809	GCA_000871965.1	Mycobacterium phage Tweety
1161933	GCA_002624625.1	Mycobacterium phage Twister
2283262	GCA_003366615.1	Mycobacterium phage Tydolla
2126957	GCA_003051625.1	Mycobacterium phage Tyke
2517971	GCA_004520375.1	Mycobacterium phage Typha
1897768	GCA_002758275.1	Mycobacterium phage TyrionL
260120	GCA_000873625.1	Mycobacterium phage U2
2283263	GCA_003366415.1	Mycobacterium phage UAch1
2530153	GCA_004338515.1	Mycobacterium phage Ugenie5
1701847	GCA_002603485.1	Mycobacterium phage Ukulele
663560	GCA_001308715.1	Mycobacterium phage UncleHowie
2108132	GCA_003013915.1	Mycobacterium phage UncleRicky
2015825	GCA_002625765.1	Mycobacterium phage Unicorn
1673876	GCA_001502515.1	Mycobacterium phage UnionJack
2041554	GCA_002745075.1	Mycobacterium phage Updawg
1034147	GCA_002632935.1	Mycobacterium phage UPIE
1912978	GCA_002614635.1	Mycobacterium phage Urkel
1414747	GCA_000914435.1	Mycobacterium phage Validus
2499066	GCA_004007955.1	Mycobacterium phage Valjean
2510580	GCA_004147985.1	Mycobacterium phage ValleyTerrace
2301620	GCA_003601355.1	Mycobacterium phage VasuNzinga
2283300	GCA_003366655.1	Mycobacterium phage Vaticameos
1305710	GCA_000907175.1	Mycobacterium phage vB_MapS_FF47
1340821	GCA_000911875.1	Mycobacterium phage Velveteen
2530154	GCA_004338715.1	Mycobacterium phage Veracruz
2499067	GCA_004007975.1	Mycobacterium phage Veritas
1647301	GCA_001500575.1	Mycobacterium phage Vincenzo
1086800	GCA_000918535.1	Mycobacterium phage Violet
2041555	GCA_002745095.1	Mycobacterium phage Virapocalypse
2250387	GCA_003341775.1	Mycobacterium phage Visconti
1089108	GCA_000919415.1	Mycobacterium phage Vista
1536599	GCA_002756375.1	Mycobacterium phage Vivaldi
1034148	GCA_002599925.1	Mycobacterium phage Vix
1542912	GCA_001551105.1	Mycobacterium phage VohminGhazi
2483722	GCA_003722515.1	Mycobacterium phage Vorrps
1034149	GCA_001503635.1	Mycobacterium phage Vortex
2027903	GCA_002629605.1	Mycobacterium phage Wachhund
2517972	GCA_004520435.1	Mycobacterium phage WaldoWhy

2530155	GCA_004338455.1	Mycobacterium phage Waleliano
2499068	GCA_004008875.1	Mycobacterium phage Wallhey
1088867	GCA_002598465.1	Mycobacterium phage Wally
2315617	GCA_003613155.1	Mycobacterium phage Wamburgrxpress
1340713	GCA_000912495.1	Mycobacterium phage Wanda
2099451	GCA_002997785.1	Mycobacterium phage Wander
2315546	GCA_003613195.1	Mycobacterium phage Waterdiva
1897518	GCA_002613045.1	Mycobacterium phage Waterfoul
1897489	GCA_002612685.1	Mycobacterium phage Watson
938131	GCA_000890475.1	Mycobacterium phage Wee
1784843	GCA_002609145.1	Mycobacterium phage Weiss13
2530156	GCA_004338555.1	Mycobacterium phage Whabigail7
1383054	GCA_000909855.1	Mycobacterium phage Wheeler
1340826	GCA_000910815.1	Mycobacterium phage Whirlwind
2484216	GCA_003722795.1	Mycobacterium phage Whouxphf
2027904	GCA_002629625.1	Mycobacterium phage Wiks
1873449	GCA_002597825.1	Mycobacterium phage Wilbur
373415	GCA_000868305.2	Mycobacterium phage Wildcat
1897507	GCA_002612985.1	Mycobacterium phage Wilder
1056833	GCA_000918615.1	Mycobacterium phage Wile
2047845	GCA_002956405.1	Mycobacterium phage Wilkins
2041556	GCA_002745115.1	Mycobacterium phage Willez
1486404	GCA_002598545.1	Mycobacterium phage Willis
1897769	GCA_002614045.1	Mycobacterium phage WillSterrel
1327778	GCA_002603445.1	Mycobacterium phage Winky
2015891	GCA_002627045.1	Mycobacterium phage Wintermute
1327036	GCA_000907575.1	Mycobacterium phage WIVsmall
2301608	GCA_003601275.1	Mycobacterium phage Wizard007
1701854	GCA_002756975.1	Mycobacterium phage Wooldri
2047846	GCA_002956415.1	Mycobacterium phage WunderPhul
2178923	GCA_003182865.1	Mycobacterium phage Xavia
2099452	GCA_002997795.1	Mycobacterium phage Xavier
1821538	GCA_001755665.1	Mycobacterium phage Xeno
1821279	GCA_002757575.1	Mycobacterium phage Xerxes
1698715	GCA_001504135.1	Mycobacterium phage XFactor
2283301	GCA_003366635.1	Mycobacterium phage xkcd
2072010	GCA_002989785.1	Mycobacterium phage Y10
2072010	GCA_002989795.1	Mycobacterium phage Y10
2072011	GCA_002958275.1	Mycobacterium phage Y2
2126806	GCA_003034625.1	Mycobacterium phage Yahalom
2041557	GCA_002745135.1	Mycobacterium phage YassJohnny
1034119	GCA_002754995.1	Mycobacterium phage Yoshand
2499069	GCA_004008115.1	Mycobacterium phage YouGoGlencoco
2047847	GCA_002956425.1	Mycobacterium phage Youngblood
2510586	GCA_004148045.1	Mycobacterium phage YoureAdopted
1897433	GCA_002612325.1	Mycobacterium phage Yucca
1505226	GCA_002604725.1	Mycobacterium phage YungJamal
2499070	GCA_004008135.1	Mycobacterium phage Zaider
1391433	GCA_000914035.1	Mycobacterium phage Zaka
1897506	GCA_002612965.1	Mycobacterium phage Zakai

1698716	GCA_002603785.1	Mycobacterium phage Zakhe101
2315715	GCA_004138695.1	Mycobacterium phage Zalkecks
1486474	GCA_002604605.1	Mycobacterium phage Zapner
2282911	GCA_003423185.1	Mycobacterium phage Zeeculate
1698253	GCA_002601565.1	Mycobacterium phage Zeenon
2488979	GCA_003867695.1	Mycobacterium phage Zelda
2301572	GCA_003442535.1	Mycobacterium phage Zelink
1034151	GCA_002633525.1	Mycobacterium phage Zemanar
1983573	GCA_002623705.1	Mycobacterium phage Zenon
2006926	GCA_002625085.1	Mycobacterium phage ZenTime222
1930080	GCA_002617765.1	Mycobacterium phage Zephyr
2488980	GCA_003867655.1	Mycobacterium phage Zerg
2499071	GCA_004008305.1	Mycobacterium phage Zetzy
2341047	GCA_004338895.1	Mycobacterium phage Zeuska
2530157	GCA_004338965.1	Mycobacterium phage Zilizebeth
1486427	GCA_000920835.1	Mycobacterium phage ZoeJ
1897498	GCA_002612805.1	Mycobacterium phage Zombie
1541820	GCA_002601285.1	Mycobacterium phage Zonia
2315547	GCA_003613275.1	Mycobacterium phage Zulu
1566994	GCA_002598565.1	Mycobacterium phage ZygoTaiga
373403	GCA_000869145.1	Mycobacterium virus 244
1982901	GCA_000920315.1	Mycobacterium virus Acadian
1089111	GCA_000917775.1	Mycobacterium virus Alma
1034152	GCA_002632985.1	Mycobacterium virus Anaya
861044	GCA_000887555.1	Mycobacterium virus Angelica
685506	GCA_000887155.1	Mycobacterium virus Ardmore
1211281	GCA_002602465.1	Mycobacterium virus Arturo
1195075	GCA_002606865.1	Mycobacterium virus Astro
1168594	GCA_000917635.1	Mycobacterium virus Avani
1089115	GCA_000920275.1	Mycobacterium virus Babsiella
1034129	GCA_002632605.1	Mycobacterium virus Backyardigan
1034099	GCA_002600775.1	Mycobacterium virus Baka
1034100	GCA_002600795.1	Mycobacterium virus Bask21
1034100	GCA_002600795.2	Mycobacterium virus Bask21
1032891	GCA_002600695.1	Mycobacterium virus BBPiebs31
1041807	GCA_002601205.1	Mycobacterium virus Benedict
260121	GCA_000871185.1	Mycobacterium virus Bethlehem
1089116	GCA_000917795.1	Mycobacterium virus Billknuckles
546182	GCA_000880295.1	Mycobacterium virus Boomer
861047	GCA_000890095.1	Mycobacterium virus Bron
561996	GCA_000880575.1	Mycobacterium virus Brujita
1089118	GCA_000919435.1	Mycobacterium virus Bruns
148603	GCA_000837245.1	Mycobacterium virus Bxb1
205870	GCA_000841385.2	Mycobacterium virus Bxz2
205870	GCA_000841385.1	Mycobacterium virus Bxz2
373405	GCA_000869185.1	Mycobacterium virus Che12
205868	GCA_000846225.1	Mycobacterium virus Che8
205872	GCA_000841365.1	Mycobacterium virus Che9c
205876	GCA_000840885.2	Mycobacterium virus Che9d
205869	GCA_000840565.1	Mycobacterium virus CJW1

205875	GCA_000843065.1	Mycobacterium virus Corndog
1089119	GCA_000918795.1	Mycobacterium virus Courthouse
861045	GCA_000889215.2	Mycobacterium virus Crimd
861045	GCA_000889215.1	Mycobacterium virus CrimD
1071502	GCA_002601405.1	Mycobacterium virus Cuco
28369	GCA_000841865.1	Mycobacterium virus D29
28369	GCA_003004865.1	Mycobacterium virus D29
1089120	GCA_000918775.1	Mycobacterium virus Deadp
1034101	GCA_002600815.1	Mycobacterium virus Dlane
1051138	GCA_000919315.1	Mycobacterium virus Doom
1225861	GCA_002602665.1	Mycobacterium virus Dorothy
1097752	GCA_002601945.1	Mycobacterium virus Dotproduct
1074303	GCA_000918595.1	Mycobacterium virus Drago
1041406	GCA_002601185.1	Mycobacterium virus Ericb
1051140	GCA_000918655.1	Mycobacterium virus Euphoria
1074306	GCA_002601485.1	Mycobacterium virus Eureka
1034125	GCA_000892115.1	Mycobacterium virus Faith1
1097753	GCA_001041915.1	Mycobacterium virus Fionnbarth
1089135	GCA_000918735.1	Mycobacterium virus Firecracker
563119	GCA_000880595.1	Mycobacterium virus Fruitloop
1034133	GCA_002632705.1	Mycobacterium virus George
1034135	GCA_002632735.1	Mycobacterium virus Gladiator
1211282	GCA_002602485.1	Mycobacterium virus Goose
1071505	GCA_000920135.1	Mycobacterium virus Gumbie
373407	GCA_000868265.2	Mycobacterium virus Halo
1034103	GCA_002600855.1	Mycobacterium virus Hammer
1032892	GCA_002600715.1	Mycobacterium virus Heldan
1034107	GCA_002600875.1	Mycobacterium virus Ibhubesi
540065	GCA_000880215.1	Mycobacterium virus Jasper
1051143	GCA_002601245.1	Mycobacterium virus Jaws
1034108	GCA_002600905.1	Mycobacterium virus JC27
1089125	GCA_002601725.1	Mycobacterium virus Jeffabunny
1034136	GCA_002709675.1	Mycobacterium virus JHC117
1034137	GCA_002632795.1	Mycobacterium virus Joedirt
540066	GCA_000879595.1	Mycobacterium virus KBG
546183	GCA_000879675.1	Mycobacterium virus Kostya
1034109	GCA_002600935.1	Mycobacterium virus Kssjeb
1089128	GCA_000918755.1	Mycobacterium virus Kugel
31757	GCA_000846545.1	Mycobacterium virus L5
1056831	GCA_000918635.1	Mycobacterium virus Larva
1034110	GCA_002600965.1	Mycobacterium virus Lesedi
1089129	GCA_002601785.1	Mycobacterium virus LHTSCC
1074304	GCA_000918575.1	Mycobacterium virus Liefie
1034111	GCA_002600985.1	Mycobacterium virus Littlee
373408	GCA_000869885.1	Mycobacterium virus Llij
540067	GCA_000874605.1	Mycobacterium virus lockley
1195070	GCA_002602405.1	Mycobacterium virus Macncheese
1211283	GCA_002602505.1	Mycobacterium virus Marcell
1034140	GCA_002866965.1	Mycobacterium virus Microwolf
1034112	GCA_002601005.1	Mycobacterium virus Mozy

1036613	GCA_002601145.1	Mycobacterium virus Mrgordo
1034113	GCA_002601025.1	Mycobacterium virus Museum
1036614	GCA_002601165.1	Mycobacterium virus Mutaforma13
1194642	GCA_002602365.1	Mycobacterium virus Nepal
205879	GCA_000842205.1	Mycobacterium virus Omega
1032893	GCA_002600735.1	Mycobacterium virus Optimus
563122	GCA_000881555.1	Mycobacterium virus Pacc40
1034142	GCA_002632835.1	Mycobacterium virus Packman
663557	GCA_000887055.1	Mycobacterium virus Peaches
1076136	GCA_000919235.1	Mycobacterium virus Perseus
1034114	GCA_002601045.1	Mycobacterium virus Pixie
373412	GCA_000869905.1	Mycobacterium virus PMC
546185	GCA_000875505.1	Mycobacterium virus Porky
540068	GCA_000875425.1	Mycobacterium virus Pukovnik
663559	GCA_002630545.1	Mycobacterium virus Pumpkin
563123	GCA_000883115.1	Mycobacterium virus Ramsey
1225862	GCA_002602685.1	Mycobacterium virus Rebeuca
1034145	GCA_002632885.1	Mycobacterium virus Rockyhorror
1086751	GCA_000917495.1	Mycobacterium virus Rumpelstiltskin
1097754	GCA_002601965.1	Mycobacterium virus Saintus
1089132	GCA_000917715.1	Mycobacterium virus SG4
2560607	GCA_002624685.1	Mycobacterium virus TA17a
927988	GCA_002758935.1	Mycobacterium virus Wonder
1034150	GCA_002632965.1	Mycobacterium virus Yoshi
75590	GCA_000844505.1	Mycoplasma phage MAV1
280702	GCA_000843645.2	Mycoplasma phage phiMFV1
280702	GCA_002921395.1	Mycoplasma phage phiMFV1
280702	GCA_002921385.1	Mycoplasma phage phiMFV1
35238	GCA_000838165.1	Mycoplasma virus P1
311229	GCA_000865205.1	Mycoreovirus 3
2304056	GCA_004133625.1	Myocastor coypus polyomavirus 1
1780507	GCA_001564385.1	Myotis gammaherpesvirus 8
1195367	GCA_003033245.1	Myotis myotis bocavirus 1
563775	GCA_000883135.1	Myotis polyomavirus VM-2008
1195370	GCA_003033165.1	Myotis ricketti papillomavirus 1
2202564	GCA_003657665.1	Myoviridae sp.
2202564	GCA_003652565.1	Myoviridae sp.
2202564	GCA_003657005.1	Myoviridae sp.
2202564	GCA_003657725.1	Myoviridae sp.
2202564	GCA_003651585.1	Myoviridae sp.
2202564	GCA_003655865.1	Myoviridae sp.
2202564	GCA_003659645.1	Myoviridae sp.
2018502	GCA_002270805.1	Myrmica scabrinodis virus 1
185636	GCA_000842545.1	Mythimna loreyi densovirus
1293572	GCA_000427155.1	Mythimna separata entomopoxvirus 'L'
1136024	GCA_002005665.1	Mythimna unipuncta granulovirus
2169746	GCA_002005665.2	Mythimna unipuncta granulovirus B
1692256	GCA_001274385.1	Mytilus sp. clam associated circular virus
49964	GCA_000844485.1	Myxococcus phage Mx8
10273	GCA_000843685.1	Myxoma virus

10273	GCA_004139615.1	Myxoma virus
10273	GCA_004140355.1	Myxoma virus
10273	GCA_004139755.1	Myxoma virus
10273	GCA_004140235.1	Myxoma virus
10273	GCA_004139895.1	Myxoma virus
10273	GCA_004139595.1	Myxoma virus
10273	GCA_004139955.1	Myxoma virus
10273	GCA_004140035.1	Myxoma virus
10273	GCA_004140175.1	Myxoma virus
10273	GCA_004139695.1	Myxoma virus
10273	GCA_004140095.1	Myxoma virus
10273	GCA_004139355.1	Myxoma virus
10273	GCA_004139975.1	Myxoma virus
10273	GCA_004139415.1	Myxoma virus
10273	GCA_004139535.1	Myxoma virus
10273	GCA_004139495.1	Myxoma virus
10273	GCA_004139635.1	Myxoma virus
10273	GCA_004139795.1	Myxoma virus
10273	GCA_004140115.1	Myxoma virus
10273	GCA_004139775.1	Myxoma virus
10273	GCA_004140315.1	Myxoma virus
10273	GCA_004139835.1	Myxoma virus
10273	GCA_004140255.1	Myxoma virus
10273	GCA_004139915.1	Myxoma virus
10273	GCA_004140055.1	Myxoma virus
10273	GCA_004139735.1	Myxoma virus
10273	GCA_004139375.1	Myxoma virus
10273	GCA_004139575.1	Myxoma virus
10273	GCA_004139675.1	Myxoma virus
10273	GCA_004139855.1	Myxoma virus
10273	GCA_004139515.1	Myxoma virus
10273	GCA_004139335.1	Myxoma virus
10273	GCA_004139935.1	Myxoma virus
10273	GCA_004139995.1	Myxoma virus
10273	GCA_004139655.1	Myxoma virus
10273	GCA_004140135.1	Myxoma virus
10273	GCA_004140195.1	Myxoma virus
10273	GCA_004139455.1	Myxoma virus
10273	GCA_004140275.1	Myxoma virus
10273	GCA_004140335.1	Myxoma virus
10273	GCA_004139555.1	Myxoma virus
10273	GCA_004139815.1	Myxoma virus
10273	GCA_004139395.1	Myxoma virus
10273	GCA_004140215.1	Myxoma virus
10273	GCA_004139875.1	Myxoma virus
10273	GCA_004140075.1	Myxoma virus
10273	GCA_004140015.1	Myxoma virus
10273	GCA_004139435.1	Myxoma virus
10273	GCA_004140155.1	Myxoma virus
10273	GCA_004139315.1	Myxoma virus

10273	GCA_004139475.1	Myxoma virus
10273	GCA_004140295.1	Myxoma virus
210016	GCA_000843185.1	Myzus persicae densovirus
194540	GCA_002117695.1	Nairobi sheep disease virus
667516	GCA_001678375.1	Nakiwogo virus
325676	GCA_000893795.1	Nam Dinh virus
1254420	GCA_004130905.1	Nanay virus
96892	GCA_003033975.1	Nanovirus-like particle
96892	GCA_003033985.1	Nanovirus-like particle
96892	GCA_003033995.1	Nanovirus-like particle
160844	GCA_000869965.1	Narcissus common latent virus
394036	GCA_000870425.1	Narcissus degeneration virus
160842	GCA_000917115.1	Narcissus late season yellows virus
12180	GCA_000849125.1	Narcissus mosaic virus
367682	GCA_000867745.1	Narcissus symptomless virus
2025020	GCA_003147465.1	Narcissus virus 1
160843	GCA_000882395.1	Narcissus yellow stripe virus
590647	GCA_000896235.1	Nariva virus
1923780	GCA_001927135.1	narna-like virus 6
114777	GCA_000841885.1	Natrialba phage PhiCh1
114777	GCA_004340325.1	Natrialba phage PhiCh1
1723446	GCA_002829825.1	Natrinema virus SNJ1
59302	GCA_000895975.1	Ndumu virus
1778580	GCA_001550585.1	Nectarine marafivirus M
2560614	GCA_001028925.1	Nectarine stem pitting associated virus
1170424	GCA_001661735.1	Negev virus
312009	GCA_000881735.1	Nemesia ring necrosis virus
204507	GCA_000867485.1	Neodiprion abietis NPV
249151	GCA_000847285.1	Neodiprion lecontei nucleopolyhedrovirus
111874	GCA_000843625.1	Neodiprion sertifer nucleopolyhedrovirus
1985188	GCA_004130495.1	Neofusicoccum luteum fusarivirus 1
1985159	GCA_002210495.1	Neofusicoccum luteum mitovirus 1
1224510	GCA_000917855.1	Nepavirus
2108204	GCA_004131065.1	Nephila clavipes virus 1
2108205	GCA_004131085.1	Nephila clavipes virus 2
2108200	GCA_004131105.1	Nephila clavipes virus 3
2108201	GCA_004131125.1	Nephila clavipes virus 4
2108203	GCA_004117255.1	Nephila clavipes virus 6
797075	GCA_001430135.1	Nerine latent virus
333348	GCA_000866105.1	Nerine virus X
2099521	GCA_004130575.1	Nesidiocoris tenuis iflavirus 1
1930921	GCA_001957715.1	Nesidiocoris tenuis virus
2175246	GCA_003147445.1	Neurachne minor latent virus
1497391	GCA_000922675.1	New Jersey polyomavirus-2013
499854	GCA_000868845.2	New Mapoon virus
1149871	GCA_000895995.1	New World begomovirus associated satellite DNA isolate 1
1149875	GCA_002830425.1	New World begomovirus associated satellite DNA isolate 2
1149880	GCA_002830485.1	New World begomovirus associated satellite DNA isolate 4
1149883	GCA_002830445.1	New World begomovirus associated satellite DNA isolate 4
1150019	GCA_002830405.1	New World begomovirus associated satellite DNA isolate H

331642	GCA_000867125.1	Newbury agent 1
139270	GCA_000862725.1	Newcastle disease virus B1
1972623	GCA_000886315.1	Ngaingan hapavirus
1485186	GCA_000920675.1	Nhumirim virus
1348439	GCA_000926595.1	Niakha virus
1286143	GCA_000923535.2	Nienokoue virus
1159904	GCA_000896035.1	Night heron coronavirus HKU19
1913649	GCA_002366125.1	Nigrospora oryzae fusarivirus 1
1765736	GCA_001654085.1	Nigrospora oryzae victorivirus 1
1267585	GCA_002816495.1	Nilaparvata lugens honeydew virus 1
1345661	GCA_000909415.1	Nilaparvata lugens honeydew virus-2
1345662	GCA_000910275.1	Nilaparvata lugens honeydew virus-3
33724	GCA_000852065.1	Nilaparvata lugens reovirus
1285600	GCA_000869065.1	Nile crocodilepox virus
1229325	GCA_000919535.1	Niminivirus
1229325	GCA_004369725.1	Niminivirus
121791	GCA_000863625.1	Nipah henipavirus
121791	GCA_003147825.1	Nipah henipavirus
121791	GCA_003147845.1	Nipah henipavirus
121791	GCA_003147865.1	Nipah henipavirus
121791	GCA_003147885.1	Nipah henipavirus
121791	GCA_003147905.1	Nipah henipavirus
121791	GCA_003147925.1	Nipah henipavirus
121791	GCA_003147945.1	Nipah henipavirus
121791	GCA_003147965.1	Nipah henipavirus
121791	GCA_003147985.1	Nipah henipavirus
121791	GCA_003148005.1	Nipah henipavirus
121791	GCA_003148025.1	Nipah henipavirus
121791	GCA_003148045.1	Nipah henipavirus
121791	GCA_003148065.1	Nipah henipavirus
1978536	GCA_002815635.1	Nishimuro ledantevirus
1230469	GCA_002602925.1	Nitratiruptor phage NrS-1
1472912	GCA_000921795.1	Nitrincola phage 1M3-16
927392	GCA_003047775.1	Nitrososphaera phage Pro-Nvie1
380442	GCA_002145865.1	Nkolbisson virus
1920748	GCA_001904885.1	NL63-related bat coronavirus
1920748	GCA_003972065.1	NL63-related bat coronavirus
1109711	GCA_000895775.1	Nocardia phage NBR1
1434748	GCA_003330165.1	Nocardia phage NOC1
1434749	GCA_003330465.1	Nocardia phage NOC2
1434750	GCA_003330445.1	Nocardia phage NOC3
12288	GCA_000847805.1	Nodamura virus
1980540	GCA_002834065.1	Nome phantom orthophasmavirus
1168479	GCA_000898415.1	Nonlabens phage P12024L
1168478	GCA_000899015.1	Nonlabens phage P12024S
1605403	GCA_002820765.1	Non-primate hepacivirus NZP1
283876	GCA_000868865.1	Nootka lupine vein clearing virus
363716	GCA_000866325.1	Nora virus
1529909	GCA_003813225.1	Norovirus GI/Hu/JP/2007/GI.P3_GI.3/Shimizu/KK2866
122929	GCA_003638685.1	Norovirus GII

122929	GCA_004193815.1	Norovirus GII
552592	GCA_003638645.1	Norovirus GII.17
490039	GCA_003638665.1	Norovirus GII.2
340017	GCA_001595675.1	Norovirus GIII
1183318	GCA_001595715.1	Norovirus Hu/GIV.1/LakeMacquarie/NSW268O/2010/AU
1985704	GCA_000854665.1	Northern cereal mosaic cytorhabdovirus
11983	GCA_000864005.1	Norwalk virus
95340	GCA_001595695.1	Norwalk-like virus
1562064	GCA_000927675.1	Norway rat hunnivirus
1562065	GCA_000929635.1	Norway rat pegivirus
1775256	GCA_002608925.1	Nostoc phage A1
1775255	GCA_002608905.1	Nostoc phage N1
1955558	GCA_002005685.1	Noumeavirus
486494	GCA_002003995.1	Nounane virus
660955	GCA_002118665.1	Nova virus
1286142	GCA_000907135.1	Nse virus
64292	GCA_000897715.1	Ntaya virus
85652	GCA_000855445.1	Nudaurelia capensis beta virus
2025360	GCA_002270605.1	NY_014 poxvirus
644610	GCA_000882955.1	Nyamanini nyavirus
35316	GCA_002118785.1	Nyando virus
1871153	GCA_001689835.1	Nylanderia fulva virus 1
1871153	GCA_001717295.1	Nylanderia fulva virus 1
318852	GCA_000925635.1	Oak-Vale virus
56879	GCA_000861425.1	Oat blue dwarf virus
146762	GCA_000856785.1	Oat chlorotic stunt virus
497863	GCA_000875245.1	Oat dwarf virus
45103	GCA_000856005.1	Oat golden stripe virus
157837	GCA_000862525.1	Oat mosaic virus
112437	GCA_000852625.1	Oat necrotic mottle virus
380160	GCA_000896135.1	Obodhiang virus
31749	GCA_000852265.1	Obuda pepper virus
1244565	GCA_002116075.1	Ochlerotatus caspius flavivirus
1897640	GCA_002613625.1	Ochrobactrum phage POA1180
1932118	GCA_002617805.1	Ochrobactrum phage POI1126
2020286	GCA_002271125.1	Ocimum basilicum RNA virus 1
2020287	GCA_002270785.1	Ocimum basilicum RNA virus 2
31699	GCA_002889735.1	Ockelbo virus
78522	GCA_002355065.1	Odocoileus adenovirus 1
1592107	GCA_002825725.1	Odonata associated gemycircularvirus-1
1592107	GCA_003849545.1	Odonata associated gemycircularvirus-1
1592108	GCA_002825745.1	Odonata associated gemycircularvirus-2
1592108	GCA_003849525.1	Odonata associated gemycircularvirus-2
1592122	GCA_003033395.1	Odonata-associated circular virus 21
1592125	GCA_003033405.1	Odonata-associated circular virus 5
1592109	GCA_004128935.1	Odonata-associated circular virus-1
1592110	GCA_004129195.1	Odonata-associated circular virus-10
1592111	GCA_004128955.1	Odonata-associated circular virus-11
1592113	GCA_004128975.1	Odonata-associated circular virus-13
1592114	GCA_004128995.1	Odonata-associated circular virus-14

1592115	GCA_004129015.1	Odonata-associated circular virus-15
1592116	GCA_004129175.1	Odonata-associated circular virus-16
1592117	GCA_004129055.1	Odonata-associated circular virus-17
1592118	GCA_004129075.1	Odonata-associated circular virus-18
1592118	GCA_004129095.1	Odonata-associated circular virus-18
1592119	GCA_004129115.1	Odonata-associated circular virus-19
1592120	GCA_004129035.1	Odonata-associated circular virus-2
1592123	GCA_004129135.1	Odonata-associated circular virus-3
1592124	GCA_004129155.1	Odonata-associated circular virus-4
1592127	GCA_004128875.1	Odonata-associated circular virus-7
1592128	GCA_004128895.1	Odonata-associated circular virus-8
1592129	GCA_004128915.1	Odonata-associated circular virus-9
12238	GCA_000859905.1	Odontoglossum ringspot virus
1435411	GCA_000916175.1	Oenococcus phage phi9805
2201414	GCA_003258195.1	Oenococcus phage phiOE33PA
1432847	GCA_000916195.1	Oenococcus phage phiS11
1432848	GCA_000916215.1	Oenococcus phage phiS13
1795442	GCA_003972045.1	Ofaie virus
1272953	GCA_002145845.1	Oita virus
1642458	GCA_001019795.1	Okahandja mammarenavirus
186810	GCA_000841065.1	OkLCV satDNA 10
1249467	GCA_000902995.1	Okra enation leaf curl alphasatellite
908277	GCA_000891415.1	Okra enation leaf curl betasatellite [India:Sonipat:EL10:200
908125	GCA_001866875.1	Okra enation leaf curl virus
908127	GCA_000887915.1	Okra enation leaf curl virus [India:Munthal EL37:2006]
518829	GCA_000844465.1	Okra leaf curl alphasatellite
912034	GCA_000889495.1	Okra leaf curl Cameroon virus
908124	GCA_000890435.1	Okra leaf curl India virus [India:Sonipat EL14A:2006]
454129	GCA_002822125.1	Okra leaf curl Mali virus
454128	GCA_000874105.1	Okra leaf curl Mali virus satellite DNA beta
2010323	GCA_002830125.1	Okra leaf curl Oman betasatellite
1241370	GCA_001504015.1	Okra leaf curl Oman virus
627503	GCA_000885075.1	Okra leaf curl virus-[Cameroon]
70822	GCA_000873885.1	Okra mosaic virus
555460	GCA_000875625.1	Okra mottle virus - [Brazil:okra]
743026	GCA_000890495.1	Okra yellow crinkle Cameroon alphasatellite [CM:Lys1sp2:
743027	GCA_003034045.1	Okra yellow crinkle Cameroon alphasatellite [CM:Lys1sp3:
401040	GCA_000867665.1	Okra yellow crinkle virus
401040	GCA_002986725.1	Okra yellow crinkle virus
627500	GCA_002986715.1	Okra yellow crinkle virus-[Cameroon]
327280	GCA_000889735.1	Okra yellow mosaic Mexico virus
169693	GCA_000846465.1	Okra yellow vein disease associated sequence
223300	GCA_000840545.1	Okra yellow vein mosaic virus-[201]
47669	GCA_000855965.1	Olive latent virus 1
33773	GCA_000851305.3	Olive latent virus 2
33773	GCA_000851305.2	Olive latent virus 2
626962	GCA_000888135.1	Olive latent virus 3
295090	GCA_000858865.1	Olive mild mosaic virus
1250316	GCA_000913975.1	Olive viral satellite RNA
42764	GCA_000872465.1	Oliveros mammarenavirus

2012619	GCA_002210535.1	Olivier's shrew virus 1
82676	GCA_000858225.1	Omikronpapillomavirus 1
753758	GCA_003956505.1	Omono River virus
753758	GCA_003956525.1	Omono River virus
12542	GCA_000855505.1	Omsk hemorrhagic fever virus
43130	GCA_000862605.1	Onion yellow dwarf virus
1917232	GCA_001887825.1	Only Syngen Nebraska virus 5
12153	GCA_000850025.1	Ononis yellow mosaic virus
2169701	GCA_000863005.1	Onyong-nyong virus
2169701	GCA_002888795.1	Onyong-nyong virus
1046267	GCA_004131725.1	Operophtera brumata nucleopolyhedrovirus
352248	GCA_000865965.1	Operophtera brumata reovirus
198597	GCA_000850925.1	Ophiostoma mitovirus 3a
88387	GCA_000852385.1	Ophiostoma mitovirus 4
88388	GCA_000850945.1	Ophiostoma mitovirus 5
88389	GCA_000853205.1	Ophiostoma mitovirus 6
347482	GCA_002987405.1	Ophiostoma partitivirus 1
473784	GCA_001271115.2	Opium poppy mosaic virus
1675864	GCA_001308475.1	Opsiphanes invirae iflavirus 1
2200716	GCA_004131245.1	Opuntia tobamovirus 2
1852702	GCA_900562095.1	Opuntia virus H
253702	GCA_000853845.1	Opuntia virus X
113194	GCA_003033075.1	Orangutan hepadnavirus
1955493	GCA_002005745.1	Orbivirus SX-2017a
152177	GCA_000870945.1	Orchid fleck dichorhavirus
1620895	GCA_002146265.1	Ord River virus
10258	GCA_000844845.1	Orf virus
938083	GCA_003333185.1	Organic Lake phycodnavirus
938080	GCA_002630865.1	Organic Lake virophage
1911434	GCA_001866245.1	Orgi virus
490711	GCA_000879035.1	Orgyia leucostigma nucleopolyhedrovirus
262177	GCA_000837125.1	Orgyia pseudotsugata multiple nucleopolyhedrovirus
192199	GCA_002118565.1	Oriboca virus
1871345	GCA_003673925.1	Orinoco virus
2170052	GCA_000926835.1	Orivirus A
12204	GCA_000901615.1	Ornithogalum mosaic virus
118655	GCA_000853785.1	Oropouche virus
2023057	GCA_002892525.1	Orpheovirus IHUMI-LCC2
977912	GCA_001402145.1	Orsay virus
930075	GCA_003091015.1	Orthobunyavirus BX-2010/Henan/CHN
930075	GCA_003087655.1	Orthobunyavirus BX-2010/Henan/CHN
930075	GCA_003088455.1	Orthobunyavirus BX-2010/Henan/CHN
1678144	GCA_000915995.1	Orthohepevirus B
1678145	GCA_002826465.1	Orthohepevirus C
1678146	GCA_000898475.1	Orthohepevirus D
1678146	GCA_002116135.1	Orthohepevirus D
40058	GCA_002829445.1	Orungo virus
92521	GCA_000880875.1	Oryctes rhinoceros nudivirus
362692	GCA_000866065.1	Oryza rufipogon alphaendornavirus
362693	GCA_000866885.1	Oryza sativa alphaendornavirus

871700	GCA_000887535.1	Oscivirus A1
871701	GCA_000889195.1	Oscivirus A2
2079458	GCA_004128075.1	Osedax japonicus RNA virus 1
261939	GCA_000846065.1	Ostreid herpesvirus 1
880162	GCA_000888835.1	Ostreococcus lucimarinus virus 1
1663208	GCA_001399285.1	Ostreococcus lucimarinus virus 2
1663209	GCA_001399225.1	Ostreococcus lucimarinus virus 7
754062	GCA_003226055.1	Ostreococcus lucimarinus virus OlV3
754063	GCA_003225975.1	Ostreococcus lucimarinus virus OlV4
754064	GCA_000905435.1	Ostreococcus lucimarinus virus OlV5
754065	GCA_003226015.1	Ostreococcus lucimarinus virus OlV6
1663210	GCA_001399265.1	Ostreococcus mediterraneus virus 1
642926	GCA_000885975.1	Ostreococcus tauri virus 1
696472	GCA_000887855.1	Ostreococcus tauri virus 2
1785753	GCA_000872425.2	Ostreococcus tauri virus OtV5
1120767	GCA_003051685.1	Ostreococcus tauri virus RT-2011
1187973	GCA_002366265.1	Otarine picobirnavirus
2035998	GCA_000903955.1	Otomops polyomavirus KY156
2035999	GCA_000903915.1	Otomops polyomavirus KY157
186786	GCA_000874705.1	Ourmia melon virus
114430	GCA_000842705.1	Ovine adenovirus 7
69576	GCA_000861745.1	Ovine enzootic nasal tumor virus
10398	GCA_000866865.1	Ovine gammaherpesvirus 2
1096142	GCA_002827545.1	Ovine hokovirus
11663	GCA_000849165.1	Ovine lentivirus
56145	GCA_003180415.1	Ovine papillomavirus type 2
634772	GCA_002826845.1	Ovis aries papillomavirus 3
1602199	GCA_000928355.1	Oxalis yellow vein virus
218667	GCA_000854505.1	Oyster mushroom spherical virus
2137161	GCA_004117175.1	Oz virus
1795983	GCA_002819825.1	Pacific flying fox associated cyclovirus-1
1795984	GCA_002819845.1	Pacific flying fox associated cyclovirus-2
1795985	GCA_002819865.1	Pacific flying fox associated cyclovirus-3
1796003	GCA_003727055.1	Pacific flying fox associated multicomponent virus
1796003	GCA_003727075.1	Pacific flying fox associated multicomponent virus
1796003	GCA_003727095.1	Pacific flying fox associated multicomponent virus
1796004	GCA_003727155.1	Pacific flying fox faeces associated circular DNA virus-1
1796004	GCA_003727175.1	Pacific flying fox faeces associated circular DNA virus-1
1796005	GCA_003727275.1	Pacific flying fox faeces associated circular DNA virus-10
1796006	GCA_003727295.1	Pacific flying fox faeces associated circular DNA virus-11
1796007	GCA_003727335.1	Pacific flying fox faeces associated circular DNA virus-12
1796008	GCA_003727375.1	Pacific flying fox faeces associated circular DNA virus-13
1796009	GCA_003727395.1	Pacific flying fox faeces associated circular DNA virus-14
1796010	GCA_003727415.1	Pacific flying fox faeces associated circular DNA virus-15
1796011	GCA_003727355.1	Pacific flying fox faeces associated circular DNA virus-2
1796011	GCA_003727315.1	Pacific flying fox faeces associated circular DNA virus-2
1796012	GCA_003727115.1	Pacific flying fox faeces associated circular DNA virus-3
1796013	GCA_003727135.1	Pacific flying fox faeces associated circular DNA virus-4
1796014	GCA_003727195.1	Pacific flying fox faeces associated circular DNA virus-5
1796015	GCA_003727215.1	Pacific flying fox faeces associated circular DNA virus-6

1796016	GCA_003727235.1	Pacific flying fox faeces associated circular DNA virus-7
1796017	GCA_003727255.1	Pacific flying fox faeces associated circular DNA virus-8
1796018	GCA_003727035.1	Pacific flying fox faeces associated circular DNA virus-9
1795988	GCA_002825985.1	Pacific flying fox faeces associated gemycircularvirus-1
1795988	GCA_003849325.1	Pacific flying fox faeces associated gemycircularvirus-1
1795989	GCA_002825765.1	Pacific flying fox faeces associated gemycircularvirus-10
1795989	GCA_003849265.1	Pacific flying fox faeces associated gemycircularvirus-10
1795991	GCA_002826045.1	Pacific flying fox faeces associated gemycircularvirus-12
1795994	GCA_002825785.1	Pacific flying fox faeces associated gemycircularvirus-2
1795994	GCA_003849305.1	Pacific flying fox faeces associated gemycircularvirus-2
1795998	GCA_002826105.1	Pacific flying fox faeces associated gemycircularvirus-6
1795998	GCA_003849285.1	Pacific flying fox faeces associated gemycircularvirus-6
1795999	GCA_002826125.1	Pacific flying fox faeces associated gemycircularvirus-7
1932881	GCA_002114025.1	Pacmanvirus A23
2249772	GCA_003364395.1	Paenibacillus phage Arcticfreeze
2249763	GCA_003368965.1	Paenibacillus phage Ash
2249764	GCA_003368985.1	Paenibacillus phage Bloom
2070189	GCA_002958135.1	Paenibacillus phage BN12
2249773	GCA_003368945.1	Paenibacillus phage C7Cdelta
2249774	GCA_003364415.1	Paenibacillus phage DevRi
1636254	GCA_002756535.1	Paenibacillus phage Diane
1589750	GCA_001505095.1	Paenibacillus phage Diva
2070190	GCA_002958145.1	Paenibacillus phage Dragolir
2249765	GCA_003369005.1	Paenibacillus phage Eltigre
1636255	GCA_001502655.1	Paenibacillus phage Fern
2249766	GCA_003369085.1	Paenibacillus phage Genki
2249775	GCA_003369365.1	Paenibacillus phage Gryphonian
1636256	GCA_003369225.1	Paenibacillus phage Halcyone
1636257	GCA_001501775.1	Paenibacillus phage Harrison
1702260	GCA_002756995.1	Paenibacillus phage Hayley
1589749	GCA_001505755.1	Paenibacillus phage HB10c2
1636258	GCA_003369205.1	Paenibacillus phage Heath
2249776	GCA_003368885.1	Paenibacillus phage Honeybear
2249767	GCA_003369025.1	Paenibacillus phage Jacopo
2249777	GCA_003368905.1	Paenibacillus phage Kawika
2070191	GCA_002958155.1	Paenibacillus phage Kiel007
2249768	GCA_003369045.1	Paenibacillus phage Ley
2070192	GCA_002958165.1	Paenibacillus phage Leyra
2070193	GCA_002958175.1	Paenibacillus phage Likha
2249769	GCA_003369065.1	Paenibacillus phage LincolnB
2249778	GCA_003368925.1	Paenibacillus phage Lucielle
2070195	GCA_002958195.1	Paenibacillus phage Pagassa
1702259	GCA_002605645.1	Paenibacillus phage Paisley
2070194	GCA_002958185.1	Paenibacillus phage PBL1c
754053	GCA_000908775.1	Paenibacillus phage PG1
1337877	GCA_000910595.1	Paenibacillus phage phiIBB_Pl23
1589752	GCA_001551725.1	Paenibacillus phage Rani
2249770	GCA_003369105.1	Paenibacillus phage Saudage
1636259	GCA_003369185.1	Paenibacillus phage Scottie
1589754	GCA_002617305.1	Paenibacillus phage Shelly

1589755	GCA_001504275.1	Paenibacillus phage Sitara
2070196	GCA_002958205.1	Paenibacillus phage Tadhana
2249771	GCA_003369125.1	Paenibacillus phage Toothless
1718161	GCA_001504915.1	Paenibacillus phage Tripp
2282396	GCA_003369405.1	Paenibacillus phage Unity
1636260	GCA_002756555.1	Paenibacillus phage Vadim
1636261	GCA_001502035.1	Paenibacillus phage Vegas
2249779	GCA_003368845.1	Paenibacillus phage Wanderer
1636262	GCA_002605625.1	Paenibacillus phage Willow
1636263	GCA_001501255.1	Paenibacillus phage Xenia
2249780	GCA_003368865.1	Paenibacillus phage Yerffej
1505530	GCA_000923515.1	Pagoda yellow mosaic associated virus
2294094	GCA_004130795.1	Paguma larvata circovirus
1692257	GCA_001274265.1	Palaemonetes intermedius brackish grass shrimp associate
1692258	GCA_001274485.1	Palaemonetes kadiakensis Mississippi grass shrimp associa
1692259	GCA_001275275.1	Palaemonetes sp. common grass shrimp associated circula
1302179	GCA_002003815.1	Palm Creek virus
1891735	GCA_000925455.1	Pan troglodytes polyomavirus 3
1891736	GCA_000902835.1	Pan troglodytes polyomavirus 4
1891737	GCA_000902215.1	Pan troglodytes polyomavirus 5
1891738	GCA_000903735.1	Pan troglodytes polyomavirus 6
1891739	GCA_000901175.1	Pan troglodytes polyomavirus 7
1236400	GCA_001184945.1	Pan troglodytes troglodytes polyomavirus 1
928211	GCA_000926495.1	Pan troglodytes verus polyomavirus 1a
1762023	GCA_001465105.1	Pan troglodytes verus polyomavirus 8
2303411	GCA_004133545.1	Panax ginseng flexivirus 1
1777016	GCA_001550445.1	Panax notoginseng virus A
2248770	GCA_004131705.1	Panax notoginseng virus B
796352	GCA_000887435.1	Panax virus Y
1349409	GCA_000911655.1	Pandoravirus dulcis
1605721	GCA_000928575.1	Pandoravirus inopinatum
2107707	GCA_003233935.1	Pandoravirus macleodensis
2107708	GCA_003233915.1	Pandoravirus neocaledonia
2107709	GCA_003233895.1	Pandoravirus quercus
1349410	GCA_000911955.1	Pandoravirus salinus
2282645	GCA_004134165.1	Panicum ecklonii-associated virus
154834	GCA_000851485.1	Panicum mosaic satellite virus
40279	GCA_000856325.1	Panicum mosaic virus
10826	GCA_002825265.1	Panicum streak virus
10826	GCA_002825285.1	Panicum streak virus
10826	GCA_002825305.1	Panicum streak virus
10826	GCA_002825325.1	Panicum streak virus
10826	GCA_002825365.1	Panicum streak virus
10826	GCA_002825385.1	Panicum streak virus
10826	GCA_002825345.1	Panicum streak virus
268780	GCA_002987265.1	Panicum streak virus - [Kenya]
268779	GCA_000839585.1	Panicum streak virus - Karino
188763	GCA_000843725.1	Panine betaherpesvirus 2 (Chimpanzee cytomegalovirus)
2016467	GCA_002219725.1	Pansavirus 1
2016468	GCA_002219345.1	Pansavirus 2

323366	GCA_003177855.1	Panthera leo persica papillomavirus type 1
2170405	GCA_004132905.1	Panthera leo polyomavirus 1
881915	GCA_000900695.1	Pantoea phage LIMElight
943335	GCA_000893675.1	Pantoea phage LIMEzero
2508071	GCA_004149985.1	Pantoea phage vB_PagM_LIET2
2499073	GCA_004006745.1	Pantoea phage vB_PagS_AAS23
2499074	GCA_004006715.1	Pantoea phage vB_PagS_MED16
2099652	GCA_002997835.1	Pantoea phage vB_PagS_Vid5
913600	GCA_000887775.1	Papaya leaf crumple virus-Panipat 8 [India:Panipat:Papaya
1112202	GCA_000915275.1	Papaya leaf curl alphasatellite
714640	GCA_000844765.1	Papaya leaf curl betasatellite
1036255	GCA_002830165.1	Papaya leaf curl betasatellite-Panipat 6 [India:Panipat:Papa
1507984	GCA_002830145.1	Papaya leaf curl China betasatellite [China:Hainan:2014]
229634	GCA_002822845.1	Papaya leaf curl China virus
269110	GCA_002986735.1	Papaya leaf curl China virus - [G10]
269112	GCA_002986765.1	Papaya leaf curl China virus - [G30]
269109	GCA_000846865.1	Papaya leaf curl China virus - [G8]
631718	GCA_002986755.1	Papaya leaf curl China virus GX4
1504733	GCA_001963075.1	Papaya leaf curl Faisalabad virus
269113	GCA_000845145.1	Papaya leaf curl Guangdong virus - [GD2]
53260	GCA_000841085.1	Papaya leaf curl virus
53260	GCA_002823005.1	Papaya leaf curl virus
53260	GCA_002823025.1	Papaya leaf curl virus
53260	GCA_002822865.1	Papaya leaf curl virus
53260	GCA_002822885.1	Papaya leaf curl virus
53260	GCA_002822905.1	Papaya leaf curl virus
53260	GCA_002822925.1	Papaya leaf curl virus
53260	GCA_002822945.1	Papaya leaf curl virus
53260	GCA_002822965.1	Papaya leaf curl virus
53260	GCA_002986785.1	Papaya leaf curl virus
53260	GCA_002986815.1	Papaya leaf curl virus
1143698	GCA_002822985.1	Papaya leaf curl virus-[soybean: Lucknow]
685899	GCA_000897515.1	Papaya lethal yellowing virus
1497848	GCA_001443785.1	Papaya meleira virus
12181	GCA_000847685.1	Papaya mosaic virus
12205	GCA_000862045.1	Papaya ringspot virus
12207	GCA_002828705.1	Papaya ringspot virus W
340907	GCA_000865345.1	Papiine alphaherpesvirus 2
1221208	GCA_000899955.1	Papilio polyxenes densovirus
2336760	GCA_003589765.1	Papillomaviridae RPVne-OR02-zj
2052558	GCA_003654385.1	Papillomaviridae sp.
2052558	GCA_003656545.1	Papillomaviridae sp.
2052558	GCA_003653385.1	Papillomaviridae sp.
2052558	GCA_003651505.1	Papillomaviridae sp.
990303	GCA_000896995.1	Papio hamadryas papillomavirus 1
1667587	GCA_001008535.1	Papio ursinus cytomegalovirus
35281	GCA_000853745.1	Paprika mild mottle virus
1655644	GCA_001502975.1	Parabacteroides phage YZ-2015a
1655645	GCA_001502355.1	Parabacteroides phage YZ-2015b
1647282	GCA_002605745.1	Paracoccus phage Shpa

2500567	GCA_004015625.1	Paracoccus phage vB_PbeS_Pben1
2500568	GCA_004015645.1	Paracoccus phage vB_PkoS_Pkon1
2494563	GCA_000954255.1	Paracoccus phage vB_PmaS-R3
2500569	GCA_004015665.1	Paracoccus phage vB_PsuS_Psul1
2500570	GCA_004015685.1	Paracoccus phage vB_PthS_Pthi1
2500566	GCA_004015805.1	Paracoccus phage vB_PyeM_Pyei1
2169994	GCA_000880015.1	Paraguayan mammarenavirus
1566298	GCA_001310195.1	Paraiso Escondido virus
10506	GCA_000847045.1	Paramecium bursaria Chlorella virus 1
380598	GCA_000871245.1	Paramecium bursaria Chlorella virus AR158
399781	GCA_000867825.1	Paramecium bursaria Chlorella virus FR483
346932	GCA_003069485.1	Paramecium bursaria chlorella virus MT325
46021	GCA_000873685.1	Paramecium bursaria Chlorella virus NY2A
1692260	GCA_001274125.1	Paramuricea placomus associated circular virus
1579460	GCA_000930695.1	Parapoxvirus red deer/HL953
103782	GCA_000850665.1	Pariacoto virus
64958	GCA_000855245.1	Parietaria mottle virus
1708654	GCA_001292895.1	Parramatta River virus
1548714	GCA_002366025.1	Parrot bornavirus 1
1548714	GCA_002815075.1	Parrot bornavirus 1
1548715	GCA_002366345.1	Parrot bornavirus 2
1548718	GCA_001430055.1	Parrot bornavirus 4
1884879	GCA_001725915.1	Parrot bornavirus 5
1548719	GCA_002366005.1	Parrot bornavirus 7
1128118	GCA_000895735.1	Parrot hepatitis B virus
1128118	GCA_002826145.1	Parrot hepatitis B virus
318845	GCA_002119045.1	Parry Creek virus
12777	GCA_000862945.1	Parsnip yellow fleck virus
1755198	GCA_001654225.1	Parthenium leaf curl alphasatellite
1907771	GCA_001777225.1	Parus major densovirus
1940570	GCA_003389895.1	Parvoviridae sp.
1940570	GCA_003389915.1	Parvoviridae sp.
1940570	GCA_003389935.1	Parvoviridae sp.
1940570	GCA_003389955.1	Parvoviridae sp.
1341019	GCA_000910875.1	Parvovirus NIH-CQV
754189	GCA_002827525.1	Parvovirus YX-2010/CHN
1511783	GCA_900197505.1	Pasivirus A
1511783	GCA_900197175.1	Pasivirus A
1511783	GCA_900197445.1	Pasivirus A
1511784	GCA_000898975.1	Pasivirus A1
1196236	GCA_000898615.1	Paspalum dilatatum striate mosaic virus
1072672	GCA_000899195.1	Paspalum striate mosaic virus
1072672	GCA_002825405.1	Paspalum striate mosaic virus
871699	GCA_000890055.1	Passerivirus A1
2065211	GCA_002890055.1	Passerivirus sp.
379892	GCA_000867525.1	Passiflora latent virus
2162638	GCA_004132865.1	Passion fruit chlorotic mottle virus
1032457	GCA_000892095.1	Passion fruit mosaic virus
31732	GCA_000889535.1	Passion fruit woodiness virus
1737555	GCA_001876915.1	Passionfruit leaf distortion virus

648801	GCA_000883955.1	Passionfruit severe leaf distortion virus
2201988	GCA_003307515.1	Pasteurella phage AFS-2018a
2006930	GCA_002625125.1	Pasteurella phage PHB01
2005054	GCA_002624845.1	Pasteurella phage PHB02
2201742	GCA_003307535.1	Pasteurella phage Pm86
1927015	GCA_002617445.1	Pasteurella phage PMP-GADVASU-IND
342564	GCA_000869825.1	Pasteurella virus F108
1772258	GCA_003029255.1	Pavonia mosaic virus
1772258	GCA_003029255.2	Pavonia mosaic virus
1742596	GCA_001551525.1	Pavonia yellow mosaic virus
1742596	GCA_001551525.2	Pavonia yellow mosaic virus
1737464	GCA_002597305.1	Pbunalikevirus phiFenriz
1737465	GCA_002597285.1	Pbunalikevirus phiHabibi
1737466	GCA_002597265.1	Pbunalikevirus phiMoody
1737467	GCA_002597245.1	Pbunalikevirus phiVader
12294	GCA_000855885.1	Pea early-browning virus
193121	GCA_000852845.1	Pea enation mosaic virus 1
193121	GCA_002826625.1	Pea enation mosaic virus 1
193120	GCA_000850825.1	Pea enation mosaic virus 2
193119	GCA_000844685.1	Pea enation mosaic virus satellite RNA
1931113	GCA_001995595.1	Pea leaf distortion betasatellite
1931111	GCA_001974515.1	Pea leaf distortion virus
1441797	GCA_003028995.1	Pea necrotic yellow dwarf alphasatellite 1
1441798	GCA_003029005.1	Pea necrotic yellow dwarf alphasatellite 3
753670	GCA_000914235.1	Pea necrotic yellow dwarf virus
12208	GCA_000860445.1	Pea seed-borne mosaic virus
199361	GCA_000851825.1	Pea stem necrosis virus
157777	GCA_001184965.1	Pea streak virus
1436892	GCA_000915955.1	Pea yellow stunt virus
400395	GCA_002828225.1	Peace lily mosaic virus
2006498	GCA_002194525.1	Peach associated luteovirus
471498	GCA_000874325.1	Peach chlorotic mottle virus
12894	GCA_000850605.1	Peach latent mosaic viroid
2038685	GCA_004114855.1	Peach leaf pitting-associated virus
183585	GCA_000883375.1	Peach mosaic virus
1955730	GCA_002008435.1	Peach virus D
40687	GCA_000851245.1	Peanut bud necrosis virus
35593	GCA_000845345.1	Peanut chlorotic streak virus
28355	GCA_000850625.1	Peanut clump virus
33763	GCA_000860725.1	Peanut mottle virus
28353	GCA_002828245.1	Peanut stripe virus
12313	GCA_000863785.1	Peanut stunt virus
193122	GCA_000845885.1	Peanut stunt virus satellite RNA
12783	GCA_000855365.1	Pear blister canker viroid
197112	GCA_002830525.1	Pear latent virus
1658615	GCA_001019955.1	Pebjah virus
1856031	GCA_001661875.1	Pecan mosaic-associated virus
2053078	GCA_002957245.1	Pectinobacterium phage PEAT2
1429794	GCA_001503535.1	Pectobacterium bacteriophage PM2
2488835	GCA_003865535.1	Pectobacterium phage Arno160

2489617	GCA_003867155.1	Pectobacterium phage Astalicious
1792242	GCA_002609265.1	Pectobacterium phage CBB
2489618	GCA_003867175.1	Pectobacterium phage Clickz
2489619	GCA_003867195.1	Pectobacterium phage Clickz_B2
2489620	GCA_003867215.1	Pectobacterium phage Clickz_B3
2489621	GCA_003867235.1	Pectobacterium phage Clickz_B4
2489622	GCA_003867255.1	Pectobacterium phage Clickz_B5
2489623	GCA_003867275.1	Pectobacterium phage Clickz_B6
2489624	GCA_003867295.1	Pectobacterium phage Clickz_B7
2489625	GCA_003867315.1	Pectobacterium phage Clickz_B8
2041488	GCA_002956035.1	Pectobacterium phage DU_PP_I
2041489	GCA_002956045.1	Pectobacterium phage DU_PP_II
2041490	GCA_002956055.1	Pectobacterium phage DU_PP_III
2041491	GCA_002956065.1	Pectobacterium phage DU_PP_IV
2041492	GCA_002956075.1	Pectobacterium phage DU_PP_V
2489626	GCA_003867335.1	Pectobacterium phage Ekidair
2320194	GCA_003575365.1	Pectobacterium phage Gaspode
2163634	GCA_003094295.1	Pectobacterium phage Jarilo
2489627	GCA_003867355.1	Pectobacterium phage Khlen
2489628	GCA_003867375.1	Pectobacterium phage Koot
2489629	GCA_003867395.1	Pectobacterium phage Koot_B1
2320195	GCA_003575405.1	Pectobacterium phage Lelidair
2320196	GCA_003575445.1	Pectobacterium phage Momine
1204539	GCA_000899355.1	Pectobacterium phage My1
2163635	GCA_003094315.1	Pectobacterium phage Nepra
2320197	GCA_003575485.1	Pectobacterium phage Nobby
2489630	GCA_003867415.1	Pectobacterium phage Nobby_B1
2489631	GCA_003867435.1	Pectobacterium phage Nobby_B2
2489632	GCA_003867455.1	Pectobacterium phage Nobby_B3
2489633	GCA_003867475.1	Pectobacterium phage Nobby_B4
1654601	GCA_001551225.1	Pectobacterium phage Peat1
1211386	GCA_002602565.1	Pectobacterium phage PhiM1
1116482	GCA_000905095.1	Pectobacterium phage phiTE
2489634	GCA_003867495.1	Pectobacterium phage Phoria
1399915	GCA_000920475.1	Pectobacterium phage PM1
1965269	GCA_003029575.1	Pectobacterium phage POP72
1217810	GCA_000900955.1	Pectobacterium phage PP1
1916414	GCA_002617005.1	Pectobacterium phage PP101
1916414	GCA_002617005.2	Pectobacterium phage PP101
1873958	GCA_001743975.1	Pectobacterium phage PP16
1873958	GCA_001743975.2	Pectobacterium phage PP16
1897743	GCA_002613725.1	Pectobacterium phage PP2
1932882	GCA_002617925.1	Pectobacterium phage PP47
1916101	GCA_002615605.1	Pectobacterium phage PP74
1927014	GCA_002617425.1	Pectobacterium phage PP81
1927014	GCA_002617425.2	Pectobacterium phage PP81
1873959	GCA_001745295.1	Pectobacterium phage PP90
1932883	GCA_002617945.1	Pectobacterium phage PP99
1685500	GCA_002607125.1	Pectobacterium phage PPWS1
2153295	GCA_003764565.1	Pectobacterium phage PPWS2

1961914	GCA_002619725.1	Pectobacterium phage PPWS4
2320198	GCA_003575505.1	Pectobacterium phage Slant
1958916	GCA_002989885.1	Pectobacterium phage vB_PatM_CB7
1958917	GCA_002989895.1	Pectobacterium phage vB_PatP_CB1
1958918	GCA_002989935.1	Pectobacterium phage vB_PatP_CB3
1958919	GCA_002989955.1	Pectobacterium phage vB_PatP_CB4
1983582	GCA_003181195.1	Pectobacterium phage vB_PatP_CB5
2489635	GCA_003867515.1	Pectobacterium phage Zenivior
2489636	GCA_003867535.1	Pectobacterium phage Zenivior_B1
1127516	GCA_000900755.1	Pectobacterium phage ZF40
2169842	GCA_001967255.1	Pedilanthus leaf curl alphasatellite
619631	GCA_000868385.1	Pedilanthus leaf curl virus
619631	GCA_002986825.1	Pedilanthus leaf curl virus
587313	GCA_000881095.1	Pedilanthus leaf curl virus [Pakistan:Multan:2004]
1108070	GCA_000896455.1	Pediococcus virus cIP1
1307800	GCA_000847425.1	Pegivirus A
1307800	GCA_002821105.1	Pegivirus A
1307801	GCA_001661855.1	Pegivirus B
1985359	GCA_002821345.1	Pegivirus F
1985360	GCA_002821365.1	Pegivirus G
1985362	GCA_002821405.1	Pegivirus I
1985363	GCA_000907255.1	Pegivirus J
1985364	GCA_002118605.1	Pegivirus K
1283076	GCA_000905255.1	Pelagibacter phage HTVC008M
1283077	GCA_000906735.1	Pelagibacter phage HTVC010P
1283078	GCA_000904255.1	Pelagibacter phage HTVC011P
1283079	GCA_000905875.1	Pelagibacter phage HTVC019P
167021	GCA_000853545.1	Pelargonium chlorotic ring pattern virus
35291	GCA_000853565.1	Pelargonium flower break virus
35280	GCA_001685265.1	Pelargonium leaf curl virus
167019	GCA_000863485.1	Pelargonium line pattern virus
255587	GCA_000858365.1	Pelargonium necrotic spot virus
167020	GCA_000929215.1	Pelargonium ringspot virus
671126	GCA_000886835.1	Pelargonium vein banding virus
116056	GCA_000851285.1	Pelargonium zonate spot virus
2058161	GCA_004130375.1	Pelodiscus sinensis picornavirus 1
282786	GCA_000866305.1	Penaeid shrimp infectious myonecrosis virus
1419711	GCA_000913915.1	Penaeus monodon circovirus VN11
1513204	GCA_000866565.1	Penaeus monodon hepandensovirus 1
1513208	GCA_000882415.1	Penaeus monodon hepandensovirus 4
1529056	GCA_000922375.1	Penaeus monodon nudivirus
1513225	GCA_003033215.1	Penaeus stylirostris penstyldensovirus 1
430911	GCA_000889715.1	Penaeus vannamei nodavirus
2079598	GCA_003029615.1	Penguin megrivirus
2005055	GCA_001646375.1	Penguin siadenovirus A
648998	GCA_000923135.1	Penguinpox virus
1755754	GCA_001461085.1	Penicillium aurantiogriseum bipartite virus 1
1755753	GCA_001461405.1	Penicillium aurantiogriseum foetidus-like virus
1755752	GCA_001461565.1	Penicillium aurantiogriseum fusarivirus 1
1755781	GCA_001461145.1	Penicillium aurantiogriseum partiti-like virus

1756157	GCA_001461365.1	Penicillium aurantiogriseum partitivirus 1
1755467	GCA_001501435.1	Penicillium aurantiogriseum totivirus 1
158372	GCA_000865945.1	Penicillium chrysogenum virus
2164100	GCA_004131045.1	Penicillium digitatum narna-like virus 1
2164101	GCA_004128095.1	Penicillium digitatum polymycoviruses 1
1833938	GCA_001634095.1	Penicillium digitatum virus 1
1755792	GCA_001461245.1	Penicillium janczewskii chrysovirus 1
1532180	GCA_000924035.1	Penicillium roqueforti ssRNA mycovirus 1
296210	GCA_000864985.2	Penicillium stoloniferum virus F
296210	GCA_000864985.1	Penicillium stoloniferum virus F
216371	GCA_000856185.1	Penicillium stoloniferum virus S
216371	GCA_002987415.1	Penicillium stoloniferum virus S
221262	GCA_000863645.1	Pennisetum mosaic virus
112229	GCA_000856965.1	Pepino mosaic virus
1462681	GCA_001654125.1	Pepo aphid-borne yellows virus
1462681	GCA_003029305.1	Pepo aphid-borne yellows virus
574040	GCA_000881835.1	Pepper chat fruit viroid
1414655	GCA_002003955.1	Pepper chlorotic spot virus
369643	GCA_002868555.1	Pepper cryptic virus 1
1050903	GCA_002024655.1	Pepper cryptic virus 2
1050903	GCA_002868575.1	Pepper cryptic virus 2
2073163	GCA_002937245.1	Pepper enamovirus
223301	GCA_000842765.1	Pepper golden mosaic virus
223301	GCA_002823065.1	Pepper golden mosaic virus
223302	GCA_002823045.1	Pepper golden mosaic virus-[CR]
223303	GCA_000839505.1	Pepper huasteco yellow vein virus
223305	GCA_000842805.1	Pepper leaf curl Bangladesh virus
223305	GCA_002823085.1	Pepper leaf curl Bangladesh virus
223305	GCA_002823105.1	Pepper leaf curl Bangladesh virus
884610	GCA_002823125.1	Pepper leaf curl Lahore virus
884612	GCA_000894415.1	Pepper leaf curl Lahore Virus-[Pakistan:Lahore1:2004]
341713	GCA_002821925.1	Pepper leaf curl Pakistan virus
83839	GCA_000838585.1	Pepper leaf curl virus
436242	GCA_000872545.1	Pepper leaf curl virus satellite DNA beta
223306	GCA_002823145.1	Pepper leaf curl virus-[Malaysia]
519016	GCA_000879295.1	Pepper leaf curl Yunnan virus satellite DNA beta
519015	GCA_000879915.1	Pepper leaf curl Yunnan virus-[YN323]
12239	GCA_000859645.1	Pepper mild mottle virus
12209	GCA_000860525.1	Pepper mottle virus
31750	GCA_000852885.1	Pepper ringspot virus
12210	GCA_000868525.1	Pepper severe mosaic virus
909827	GCA_000891555.1	Pepper vein yellows virus
1965355	GCA_002922445.1	Pepper vein yellows virus 5
255066	GCA_000883555.1	Pepper veinal mottle virus
1803898	GCA_002105405.1	Pepper virus A
1987360	GCA_002158635.1	Pepper yellow dwarf virus - Mexico
554667	GCA_002824925.1	Pepper yellow dwarf virus - New Mexico
292477	GCA_000867505.1	Pepper yellow leaf curl Indonesia virus
1774200	GCA_001502775.2	Pepper yellow leaf curl Thailand virus
1774200	GCA_001502775.1	Pepper yellow leaf curl Thailand virus

1774200	GCA_003029535.1	Pepper yellow leaf curl Thailand virus
881944	GCA_000905155.1	Pepper yellow leaf curl virus
160358	GCA_000889995.1	Pepper yellow mosaic virus
260378	GCA_000840925.1	Pepper yellow vein Mali virus
698400	GCA_900078415.1	Pepper yellows virus
698400	GCA_900078425.1	Pepper yellows virus
1677909	GCA_002605345.1	Peptoclostridium phage phiCDIF1296T
1256869	GCA_000904335.1	Perch perhabdovirus
1346829	GCA_000923335.1	Peridroma alphabaculovirus
1675865	GCA_001308335.1	Perigonia lusca single nucleopolyhedrovirus
170621	GCA_000849405.1	Perina nuda virus
1972569	GCA_000926675.1	Perinet vesiculovirus
97344	GCA_000838785.1	Periplaneta fuliginosa densovirus
1188812	GCA_000901455.1	Peristrophe mosaic virus
1074206	GCA_003033155.1	Peromyscus papillomavirus 1
1131702	GCA_000895195.1	Persea americana alphaendornavirus 1
1587509	GCA_004114775.1	Persea americana chrysovirus
1183241	GCA_000899675.1	Persimmon cryptic virus
1211481	GCA_000919775.1	Persimmon latent virus
1319979	GCA_000909495.1	Persimmon viroid 2
1211480	GCA_000899915.1	Persimmon virus A
1493829	GCA_000928155.1	Persimmon virus B
187978	GCA_000861725.1	Peru tomato mosaic virus
356862	GCA_000867005.1	Peruvian horse sickness virus
2593991	GCA_000866445.1	Peste-des-petits-ruminants virus
119222	GCA_000852085.1	Pestivirus giraffe-1 H138
2170089	GCA_000926015.1	Pestivirus J
1234809	GCA_000899315.1	Pestivirus strain Aydin/04-TR
1692261	GCA_001274365.1	Petrochirus diogenes giant hermit crab associated circular
1935922	GCA_001973875.1	Petunia chlorotic mottle virus
59504	GCA_000839385.1	Petunia vein clearing virus
251749	GCA_000907415.1	Phaeocystis globosa virus
755274	GCA_003225935.1	Phaeocystis globosa virus 14T
1335638	GCA_000909155.1	Phaeocystis globosa virus virophage
2048062	GCA_003308915.1	Phage 203
2048063	GCA_003308935.1	Phage 206
1871314	GCA_002611585.1	Phage MedPE-SWcel-C56
2528528	GCA_004325275.1	Phage NC-A
2528530	GCA_004551555.1	Phage NC-G
1857647	GCA_900092385.1	Phage NCTB
2202648	GCA_003544755.1	Phage sp.
2202648	GCA_003544775.1	Phage sp.
2202648	GCA_003544675.1	Phage sp.
2202648	GCA_003544695.1	Phage sp.
2202648	GCA_003544555.1	Phage sp.
2202648	GCA_003544575.1	Phage sp.
2202648	GCA_003544595.1	Phage sp.
2202648	GCA_003544735.1	Phage sp.
2202648	GCA_003544615.1	Phage sp.
2202648	GCA_003544635.1	Phage sp.

2202648	GCA_003544795.1	Phage sp.
2202648	GCA_003544655.1	Phage sp.
2202648	GCA_003544715.1	Phage sp.
2202648	GCA_003781205.1	Phage sp.
457382	GCA_000872505.1	Phaius virus X
2069327	GCA_004128615.1	Phalaenopsis equestris amalgavirus 1
1188792	GCA_000914895.1	Phaseolus vulgaris alphaendornavirus 1
1188792	GCA_002820445.1	Phaseolus vulgaris alphaendornavirus 1
1188793	GCA_002820465.1	Phaseolus vulgaris alphaendornavirus 2
2268393	GCA_004132405.1	Phaseolus vulgaris endornavirus 3
1756832	GCA_001502815.1	Phasey bean mild yellows virus
1980610	GCA_002814835.1	Phasi Charoen-like phasivirus
2170411	GCA_004132765.1	Philantomba monticola polyomavirus 1
349681	GCA_000888155.1	Phlebiopsis gigantea mycovirus dsRNA 1
1123947	GCA_003088495.1	Phlebovirus JN1/China/2010
1017370	GCA_003088515.1	Phlebovirus JS2007-01
1205899	GCA_003088535.1	Phlebovirus JS2010-014
1205900	GCA_003088575.1	Phlebovirus JS2010-015
1205901	GCA_003088595.1	Phlebovirus JS2010-018
1205902	GCA_003088615.1	Phlebovirus JS2010-019
1010663	GCA_003088675.1	Phlebovirus JS24
1010664	GCA_003088695.1	Phlebovirus JS26
1010665	GCA_003088755.1	Phlebovirus JS6
475777	GCA_000871325.1	Phlox virus B
436066	GCA_000873785.1	Phlox virus S
64894	GCA_002022195.1	Phnom Penh bat virus
11240	GCA_001433625.1	Phocine morbillivirus
706525	GCA_000898875.1	Phocoena phocoena papillomavirus 1
706526	GCA_000898275.1	Phocoena phocoena papillomavirus 2
706527	GCA_000899735.1	Phocoena phocoena papillomavirus 4
1487796	GCA_003182635.1	Phodopus sungorus papillomavirus type 1
1808960	GCA_002029555.1	Phomopsis longicolla circular virus 1
1523858	GCA_000922295.1	Phomopsis longicolla hypovirus
1779340	GCA_002004575.1	Phomopsis longicolla RNA virus 1
1580605	GCA_000930775.1	Phomopsis vexans RNA virus
1708572	GCA_001292955.1	Phopivirus
1391455	GCA_002149665.1	Phormidium phage MIS-PhV1A
1391456	GCA_002149645.1	Phormidium phage MIS-PhV1B
440250	GCA_000873965.1	Phormidium virus WMP3
400567	GCA_000867625.1	Phormidium virus WMP4
192584	GCA_000842725.1	Phthorimaea operculella granulovirus
72539	GCA_000856825.1	Physalis mottle virus
1856627	GCA_001669845.1	Phytomonas serpens narnavirus 1
310750	GCA_000861025.1	Phytophthora alphaendornavirus 1
640897	GCA_000885295.1	Phytophthora infestans RNA virus 1
2303160	GCA_004133505.1	Phytophthora infestans RNA virus 2
1133557	GCA_003726515.1	Phytophthora infestans RNA virus 3
1077832	GCA_001605815.1	Phytophthora infestans RNA virus 4
1608451	GCA_001021275.1	Phytophthora parasitica virus
1224515	GCA_004128835.1	Picalivirus A

1046893	GCA_003985425.1	Picobiliphyte sp. MS584-5 nanovirus
1961162	GCA_002219985.1	Picobirnavirus dog/KNA/2015
1961155	GCA_002118545.1	Picobirnavirus green monkey/KNA/2015
1907787	GCA_004117395.1	Picobirnavirus sp.
1983535	GCA_002145885.1	Picorna-like virus AWando15
1813615	GCA_001706865.1	Picornavirales Bu-1
1813613	GCA_001698355.1	Picornavirales Bu-3
1795648	GCA_001706925.1	Picornavirales Tottori-HG1
1530251	GCA_003389975.1	Picornaviridae sp.
1530251	GCA_003389995.1	Picornaviridae sp.
1530251	GCA_003390015.1	Picornaviridae sp.
1530251	GCA_003390035.1	Picornaviridae sp.
1917412	GCA_003029385.1	Picornaviridae sp. rodent/Ee/PicoV/NX2015
1399966	GCA_003028975.1	Picornavirus HK21
1911436	GCA_003673745.1	Pidgey virus
1249501	GCA_003986445.1	Pig stool associated circular ssDNA virus
1249501	GCA_003986465.1	Pig stool associated circular ssDNA virus
1249501	GCA_003986485.1	Pig stool associated circular ssDNA virus
1249501	GCA_003986505.1	Pig stool associated circular ssDNA virus
1249501	GCA_003986525.1	Pig stool associated circular ssDNA virus
1249501	GCA_003986545.1	Pig stool associated circular ssDNA virus
1249501	GCA_003986565.1	Pig stool associated circular ssDNA virus
1249501	GCA_003986585.1	Pig stool associated circular ssDNA virus
1114942	GCA_000894595.1	Pig stool associated circular ssDNA virus GER2011
764030	GCA_000921315.1	Pigeon adenovirus 1
1907767	GCA_001831345.1	Pigeon adenovirus 2
1414603	GCA_004032355.1	Pigeon circovirus
1414603	GCA_004031875.1	Pigeon circovirus
1414603	GCA_004032015.1	Pigeon circovirus
1414603	GCA_004032395.1	Pigeon circovirus
1414603	GCA_004031715.1	Pigeon circovirus
1414603	GCA_004031695.1	Pigeon circovirus
1414603	GCA_004032055.1	Pigeon circovirus
1414603	GCA_004031615.1	Pigeon circovirus
1414603	GCA_004031815.1	Pigeon circovirus
1414603	GCA_004032095.1	Pigeon circovirus
1414603	GCA_004031835.1	Pigeon circovirus
1414603	GCA_004031755.1	Pigeon circovirus
1414603	GCA_004032315.1	Pigeon circovirus
1414603	GCA_004031895.1	Pigeon circovirus
1414603	GCA_004031775.1	Pigeon circovirus
1414603	GCA_004031975.1	Pigeon circovirus
1414603	GCA_004031675.1	Pigeon circovirus
1414603	GCA_004032375.1	Pigeon circovirus
1414603	GCA_004031795.1	Pigeon circovirus
1414603	GCA_004032075.1	Pigeon circovirus
1414603	GCA_004032115.1	Pigeon circovirus
1414603	GCA_004032235.1	Pigeon circovirus
1414603	GCA_004032515.1	Pigeon circovirus
1414603	GCA_004032155.1	Pigeon circovirus

1414603	GCA_004031955.1	Pigeon circovirus
1414603	GCA_004031635.1	Pigeon circovirus
1414603	GCA_004032415.1	Pigeon circovirus
1414603	GCA_004032195.1	Pigeon circovirus
1414603	GCA_004032435.1	Pigeon circovirus
1414603	GCA_004032455.1	Pigeon circovirus
1414603	GCA_004032475.1	Pigeon circovirus
1414603	GCA_004032495.1	Pigeon circovirus
1414603	GCA_004032255.1	Pigeon circovirus
1414603	GCA_004031915.1	Pigeon circovirus
1414603	GCA_004032535.1	Pigeon circovirus
1414603	GCA_004032035.1	Pigeon circovirus
1414603	GCA_004031735.1	Pigeon circovirus
1414603	GCA_004032215.1	Pigeon circovirus
1414603	GCA_004033275.1	Pigeon circovirus
1414603	GCA_004032335.1	Pigeon circovirus
1414603	GCA_004031855.1	Pigeon circovirus
1414603	GCA_004032275.1	Pigeon circovirus
1414603	GCA_004031995.1	Pigeon circovirus
1414603	GCA_004031595.1	Pigeon circovirus
1414603	GCA_004032135.1	Pigeon circovirus
1414603	GCA_004032295.1	Pigeon circovirus
1414603	GCA_004031935.1	Pigeon circovirus
1414603	GCA_004031655.1	Pigeon circovirus
1414603	GCA_004032175.1	Pigeon circovirus
1414603	GCA_004043795.1	Pigeon circovirus
1414603	GCA_004051335.1	Pigeon circovirus
928289	GCA_000892795.1	Pigeon picornavirus B
1980429	GCA_001580355.1	Pigeonpea sterility mosaic emaravirus 1
1980430	GCA_001695425.1	Pigeonpea sterility mosaic emaravirus 2
10264	GCA_000922075.1	Pigeonpox virus
219584	GCA_000926415.1	Pike fry sprivivirus
1236406	GCA_003033345.1	Piliocolobus badius polyomavirus 2
1236407	GCA_000901195.1	Piliocolobus rufomitratus polyomavirus 1
2293297	GCA_003847285.1	Pimoid spider associated circular virus 1
2293298	GCA_003846765.1	Pimoid spider associated circular virus 2
2033633	GCA_000889415.1	Pineapple bacilliform CO virus
180903	GCA_000872265.1	Pineapple mealybug wilt-associated virus 1
2267679	GCA_004134305.1	Pinus nigra virus 1
262957	GCA_000911095.1	Piper yellow mottle virus
49891	GCA_000856045.1	Pirital mammarenavirus
11274	GCA_002816055.1	Piry virus
912320	GCA_000892155.1	Piscine myocarditis virus AL V-708
1798085	GCA_001567055.1	Piscine myocarditis-like virus
1157337	GCA_002829625.1	Piscine orthoreovirus
1176736	GCA_000923195.1	Pitaya virus X
1450746	GCA_000916835.1	Pithovirus sibericum
1965238	GCA_002037775.1	Pityohyphantes rubrofasciatus iflavirus
1170425	GCA_002024735.1	Piura virus
60876	GCA_002829905.1	Pixuna virus

159252	GCA_000838185.1	Planaria asexual strain-specific virus-like element type 1
2100419	GCA_003972125.1	Planarian secretory cell nidovirus
994601	GCA_000894155.1	Planktothrix phage PaV-LD
159153	GCA_000842825.1	Planococcus citri densovirus
28354	GCA_000856745.1	Plantago asiatica mosaic virus
1830242	GCA_002824765.1	Plantago lanceolata latent virus
312274	GCA_000883295.1	Plantago mottle virus
64698	GCA_000851405.1	Plautia stali intestine virus
1403565	GCA_004134065.1	Pleione flower breaking virus
1755785	GCA_001461545.1	Pleospora typhicola fusarivirus 1
2065204	GCA_002957935.1	Plesiomonas phage phiP4-7
2057942	GCA_002890135.1	Pleurochrysis carterae circular virus
2057944	GCA_003729075.1	Pleurochrysis sp. endemic virus 1a
2057945	GCA_003729095.1	Pleurochrysis sp. endemic virus 1b
2057946	GCA_003729135.1	Pleurochrysis sp. endemic virus 2
2057947	GCA_003729115.1	Pleurochrysis sp. endemic virus unk
2057943	GCA_003764635.1	Pleurochrysis sp. Polinton-like virus
2057943	GCA_003729155.1	Pleurochrysis sp. Polinton-like virus
2057943	GCA_003729175.1	Pleurochrysis sp. Polinton-like virus
2057943	GCA_003729195.1	Pleurochrysis sp. Polinton-like virus
674983	GCA_000859745.1	Pleurotus ostreatus virus 1
262175	GCA_001924155.1	Plodia interpunctella granulovirus
675077	GCA_000872345.1	Plum bark necrosis stem pitting-associated virus
12211	GCA_000862085.1	Plum pox virus
12211	GCA_002828805.1	Plum pox virus
12211	GCA_002828825.1	Plum pox virus
12211	GCA_002828785.1	Plum pox virus
12211	GCA_002987575.1	Plum pox virus
12211	GCA_002987565.1	Plum pox virus
12211	GCA_900093765.1	Plum pox virus
12211	GCA_900093795.1	Plum pox virus
12211	GCA_900093785.1	Plum pox virus
12211	GCA_900093755.1	Plum pox virus
31738	GCA_002987605.1	Plum pox virus strain El Amar
1501716	GCA_000973375.1	Plumeria mosaic virus
98383	GCA_000838005.1	Plutella xylostella granulovirus
379891	GCA_000869385.1	Plutella xylostella multiple nucleopolyhedrovirus
270473	GCA_000857565.1	Pneumonia virus of mice J3666
2482952	GCA_000926375.1	Pneumovirus dog/Bari/100-12/ITA/2012
1708497	GCA_001292915.1	Poaceae-associated gemycircularvirus 1
1708497	GCA_003849385.1	Poaceae-associated gemycircularvirus 1
1708497	GCA_003849365.1	Poaceae-associated gemycircularvirus 1
1105383	GCA_000927735.1	Po-Circo-like virus 21
1105384	GCA_003985445.1	Po-Circo-like virus 22
1105385	GCA_000929675.1	Po-Circo-like virus 41
1105386	GCA_000928635.1	Po-Circo-like virus 51
2202567	GCA_003658425.1	Podoviridae sp.
2202567	GCA_003655805.1	Podoviridae sp.
2202567	GCA_003657645.1	Podoviridae sp.
2202567	GCA_003651605.1	Podoviridae sp.

2202567	GCA_003651625.1	Podoviridae sp.
2202567	GCA_003657565.1	Podoviridae sp.
2202567	GCA_003651765.1	Podoviridae sp.
2202567	GCA_003657585.1	Podoviridae sp.
2202567	GCA_003651785.1	Podoviridae sp.
2202567	GCA_003652065.1	Podoviridae sp.
1465639	GCA_001507515.1	Podovirus Lau218
1465639	GCA_002921805.1	Podovirus Lau218
1465639	GCA_002921815.1	Podovirus Lau218
1699094	GCA_001273685.1	Poecile atricapillus GI tract-associated gemycircularvirus
1871132	GCA_003085835.1	Poecivirus BCCH-449
305785	GCA_000883315.1	Poinsettia latent virus
113553	GCA_000860785.1	Poinsettia mosaic virus
2010279	GCA_002210675.1	Point-Douro narna-like virus
1220025	GCA_000897915.1	Pokeweed mosaic virus
1220025	GCA_002828845.1	Pokeweed mosaic virus
2250215	GCA_004131985.1	Polar bear mastadenovirus 1
1647386	GCA_001500535.1	Polaribacter phage P12002L
1647387	GCA_001502575.1	Polaribacter phage P12002S
2496155	GCA_003958925.1	Polycipiviridae sp.
2093274	GCA_002937355.1	Polygala garcinii associated virus
430606	GCA_002815835.1	Polygonum ringspot tospovirus
1678786	GCA_001745395.1	Polygonum ringspot virus
36362	GCA_002374915.1	Polyomavirus sp.
2049933	GCA_002826165.1	Pomona bat hepatitis B virus
1885565	GCA_002406395.1	Pomona leaf-nosed bat associated polyomavirus
2079347	GCA_002958535.1	Pontimonas phage phiPsal1
1926504	GCA_900199765.1	Poophage MBI-2016a
12166	GCA_000854985.1	Poplar mosaic virus
35265	GCA_000849745.1	Porcine adenovirus 3
35265	GCA_002818095.1	Porcine adenovirus 3
45370	GCA_000846825.1	Porcine adenovirus 5
45370	GCA_000885915.1	Porcine adenovirus 5
1843773	GCA_000897655.1	Porcine associated porprismacovirus 1
2170117	GCA_001646255.1	Porcine associated porprismacovirus 10
2170119	GCA_000906435.1	Porcine associated porprismacovirus 3
2170120	GCA_003033615.1	Porcine associated porprismacovirus 4
2170121	GCA_000926055.1	Porcine associated porprismacovirus 5
2170122	GCA_000922455.1	Porcine associated porprismacovirus 6
2170123	GCA_000921615.1	Porcine associated porprismacovirus 7
2170124	GCA_000924135.1	Porcine associated porprismacovirus 8
2170125	GCA_000926035.1	Porcine associated porprismacovirus 9
1105378	GCA_000914875.1	Porcine astrovirus 2
1254425	GCA_000903455.1	Porcine astrovirus 3
1105379	GCA_000916575.1	Porcine astrovirus 4
1093928	GCA_000916535.1	Porcine astrovirus 5
1165907	GCA_000917435.1	Porcine bocavirus
795694	GCA_000923795.2	Porcine bocavirus 1 pig/ZJD/China/2006
1084715	GCA_000893895.1	Porcine bocavirus 3
1084717	GCA_000891375.1	Porcine bocavirus 4-1

1131622	GCA_000895815.1	Porcine bocavirus 5/JS677
46221	GCA_004041335.1	Porcine circovirus
46221	GCA_004041375.1	Porcine circovirus
46221	GCA_004041395.1	Porcine circovirus
46221	GCA_004041415.1	Porcine circovirus
46221	GCA_004041355.1	Porcine circovirus
46221	GCA_004041435.1	Porcine circovirus
46221	GCA_004041455.1	Porcine circovirus
46221	GCA_004064035.1	Porcine circovirus
46221	GCA_004064015.1	Porcine circovirus
133704	GCA_000837765.1	Porcine circovirus 1
133704	GCA_004035335.1	Porcine circovirus 1
133704	GCA_004033655.1	Porcine circovirus 1
133704	GCA_004035655.1	Porcine circovirus 1
133704	GCA_004034935.1	Porcine circovirus 1
133704	GCA_004049075.1	Porcine circovirus 1
133704	GCA_004049095.1	Porcine circovirus 1
133704	GCA_004049115.1	Porcine circovirus 1
133704	GCA_004049135.1	Porcine circovirus 1
133704	GCA_004053375.1	Porcine circovirus 1
133704	GCA_004048975.1	Porcine circovirus 1
133704	GCA_004049015.1	Porcine circovirus 1
133704	GCA_004049055.1	Porcine circovirus 1
133704	GCA_004048955.1	Porcine circovirus 1
133704	GCA_004049155.1	Porcine circovirus 1
133704	GCA_004049175.1	Porcine circovirus 1
133704	GCA_004049195.1	Porcine circovirus 1
133704	GCA_004048995.1	Porcine circovirus 1
133704	GCA_004045335.1	Porcine circovirus 1
133704	GCA_004049035.1	Porcine circovirus 1
133704	GCA_004077115.1	Porcine circovirus 1
133704	GCA_004075095.1	Porcine circovirus 1
133704	GCA_004075135.1	Porcine circovirus 1
133704	GCA_004075155.1	Porcine circovirus 1
133704	GCA_004073435.1	Porcine circovirus 1
133704	GCA_004077195.1	Porcine circovirus 1
133704	GCA_004069135.1	Porcine circovirus 1
133704	GCA_004069155.1	Porcine circovirus 1
133704	GCA_004069175.1	Porcine circovirus 1
133704	GCA_004073455.1	Porcine circovirus 1
133704	GCA_004073495.1	Porcine circovirus 1
133704	GCA_004073475.1	Porcine circovirus 1
133704	GCA_004084815.1	Porcine circovirus 1
133704	GCA_004085015.1	Porcine circovirus 1
133704	GCA_004082295.1	Porcine circovirus 1
133704	GCA_004075615.1	Porcine circovirus 1
133704	GCA_004076015.1	Porcine circovirus 1
133704	GCA_004086335.1	Porcine circovirus 1
133704	GCA_004070135.1	Porcine circovirus 1
133704	GCA_004070155.1	Porcine circovirus 1

133704	GCA_004095015.1	Porcine circovirus 1
133704	GCA_004095035.1	Porcine circovirus 1
133704	GCA_004095055.1	Porcine circovirus 1
133704	GCA_004095075.1	Porcine circovirus 1
133704	GCA_004095095.1	Porcine circovirus 1
133704	GCA_004095335.1	Porcine circovirus 1
133704	GCA_004095375.1	Porcine circovirus 1
133704	GCA_004095475.1	Porcine circovirus 1
133704	GCA_004089135.1	Porcine circovirus 1
133704	GCA_004089495.1	Porcine circovirus 1
133704	GCA_004093595.1	Porcine circovirus 1
133704	GCA_004089515.1	Porcine circovirus 1
133704	GCA_004093635.1	Porcine circovirus 1
133704	GCA_004099075.1	Porcine circovirus 1
133704	GCA_004095935.1	Porcine circovirus 1
133704	GCA_004096055.1	Porcine circovirus 1
133704	GCA_004088895.1	Porcine circovirus 1
133704	GCA_004098415.1	Porcine circovirus 1
133704	GCA_004098495.1	Porcine circovirus 1
133704	GCA_004096535.1	Porcine circovirus 1
133704	GCA_004096555.1	Porcine circovirus 1
133704	GCA_004096575.1	Porcine circovirus 1
133704	GCA_004096595.1	Porcine circovirus 1
133704	GCA_004096615.1	Porcine circovirus 1
85708	GCA_000862765.1	Porcine circovirus 2
85708	GCA_002819625.1	Porcine circovirus 2
85708	GCA_004037515.1	Porcine circovirus 2
85708	GCA_004032995.1	Porcine circovirus 2
85708	GCA_004033015.1	Porcine circovirus 2
85708	GCA_004037175.1	Porcine circovirus 2
85708	GCA_004035415.1	Porcine circovirus 2
85708	GCA_004035075.1	Porcine circovirus 2
85708	GCA_004034735.1	Porcine circovirus 2
85708	GCA_004037275.1	Porcine circovirus 2
85708	GCA_004037155.1	Porcine circovirus 2
85708	GCA_004037035.1	Porcine circovirus 2
85708	GCA_004033035.1	Porcine circovirus 2
85708	GCA_004033495.1	Porcine circovirus 2
85708	GCA_004036795.1	Porcine circovirus 2
85708	GCA_004034315.1	Porcine circovirus 2
85708	GCA_004035095.1	Porcine circovirus 2
85708	GCA_004034475.1	Porcine circovirus 2
85708	GCA_004036975.1	Porcine circovirus 2
85708	GCA_004034755.1	Porcine circovirus 2
85708	GCA_004036495.1	Porcine circovirus 2
85708	GCA_004033395.1	Porcine circovirus 2
85708	GCA_004033055.1	Porcine circovirus 2
85708	GCA_004034795.1	Porcine circovirus 2
85708	GCA_004037455.1	Porcine circovirus 2
85708	GCA_004037115.1	Porcine circovirus 2

85708	GCA_004035115.1	Porcine circovirus 2
85708	GCA_004034775.1	Porcine circovirus 2
85708	GCA_004033695.1	Porcine circovirus 2
85708	GCA_004033415.1	Porcine circovirus 2
85708	GCA_004033075.1	Porcine circovirus 2
85708	GCA_004036435.1	Porcine circovirus 2
85708	GCA_004037595.1	Porcine circovirus 2
85708	GCA_004033735.1	Porcine circovirus 2
85708	GCA_004033755.1	Porcine circovirus 2
85708	GCA_004033095.1	Porcine circovirus 2
85708	GCA_004033775.1	Porcine circovirus 2
85708	GCA_004035135.1	Porcine circovirus 2
85708	GCA_004037215.1	Porcine circovirus 2
85708	GCA_004034515.1	Porcine circovirus 2
85708	GCA_004032775.1	Porcine circovirus 2
85708	GCA_004036915.1	Porcine circovirus 2
85708	GCA_004033795.1	Porcine circovirus 2
85708	GCA_004033815.1	Porcine circovirus 2
85708	GCA_004033835.1	Porcine circovirus 2
85708	GCA_004037735.1	Porcine circovirus 2
85708	GCA_004033855.1	Porcine circovirus 2
85708	GCA_004037655.1	Porcine circovirus 2
85708	GCA_004034335.1	Porcine circovirus 2
85708	GCA_004036455.1	Porcine circovirus 2
85708	GCA_004033895.1	Porcine circovirus 2
85708	GCA_004035155.1	Porcine circovirus 2
85708	GCA_004034815.1	Porcine circovirus 2
85708	GCA_004033915.1	Porcine circovirus 2
85708	GCA_004030515.1	Porcine circovirus 2
85708	GCA_004033935.1	Porcine circovirus 2
85708	GCA_004033115.1	Porcine circovirus 2
85708	GCA_004033955.1	Porcine circovirus 2
85708	GCA_004037555.1	Porcine circovirus 2
85708	GCA_004033975.1	Porcine circovirus 2
85708	GCA_004033995.1	Porcine circovirus 2
85708	GCA_004037195.1	Porcine circovirus 2
85708	GCA_004033375.1	Porcine circovirus 2
85708	GCA_004035175.1	Porcine circovirus 2
85708	GCA_004034835.1	Porcine circovirus 2
85708	GCA_004034035.1	Porcine circovirus 2
85708	GCA_004034055.1	Porcine circovirus 2
85708	GCA_004033135.1	Porcine circovirus 2
85708	GCA_004037575.1	Porcine circovirus 2
85708	GCA_004033515.1	Porcine circovirus 2
85708	GCA_004037355.1	Porcine circovirus 2
85708	GCA_004036215.1	Porcine circovirus 2
85708	GCA_004035195.1	Porcine circovirus 2
85708	GCA_004036995.1	Porcine circovirus 2
85708	GCA_004034855.1	Porcine circovirus 2
85708	GCA_004037255.1	Porcine circovirus 2

85708	GCA_004037475.1	Porcine circovirus 2
85708	GCA_004036515.1	Porcine circovirus 2
85708	GCA_004034275.1	Porcine circovirus 2
85708	GCA_004035215.1	Porcine circovirus 2
85708	GCA_004034875.1	Porcine circovirus 2
85708	GCA_004032915.1	Porcine circovirus 2
85708	GCA_004030195.1	Porcine circovirus 2
85708	GCA_004034535.1	Porcine circovirus 2
85708	GCA_004035295.1	Porcine circovirus 2
85708	GCA_004033175.1	Porcine circovirus 2
85708	GCA_004037615.1	Porcine circovirus 2
85708	GCA_004030475.1	Porcine circovirus 2
85708	GCA_004037315.1	Porcine circovirus 2
85708	GCA_004035235.1	Porcine circovirus 2
85708	GCA_004034895.1	Porcine circovirus 2
85708	GCA_004032795.1	Porcine circovirus 2
85708	GCA_004036935.1	Porcine circovirus 2
85708	GCA_004034555.1	Porcine circovirus 2
85708	GCA_004033195.1	Porcine circovirus 2
85708	GCA_004037755.1	Porcine circovirus 2
85708	GCA_004034415.1	Porcine circovirus 2
85708	GCA_004035255.1	Porcine circovirus 2
85708	GCA_004034495.1	Porcine circovirus 2
85708	GCA_004035575.1	Porcine circovirus 2
85708	GCA_004034575.1	Porcine circovirus 2
85708	GCA_004033215.1	Porcine circovirus 2
85708	GCA_004036855.1	Porcine circovirus 2
85708	GCA_004030495.1	Porcine circovirus 2
85708	GCA_004037135.1	Porcine circovirus 2
85708	GCA_004036375.1	Porcine circovirus 2
85708	GCA_004030535.1	Porcine circovirus 2
85708	GCA_004035275.1	Porcine circovirus 2
85708	GCA_004034595.1	Porcine circovirus 2
85708	GCA_004036875.1	Porcine circovirus 2
85708	GCA_004034455.1	Porcine circovirus 2
85708	GCA_004033575.1	Porcine circovirus 2
85708	GCA_004033235.1	Porcine circovirus 2
85708	GCA_004037695.1	Porcine circovirus 2
85708	GCA_004032675.1	Porcine circovirus 2
85708	GCA_004036895.1	Porcine circovirus 2
85708	GCA_004037055.1	Porcine circovirus 2
85708	GCA_004034355.1	Porcine circovirus 2
85708	GCA_004035615.1	Porcine circovirus 2
85708	GCA_004034955.1	Porcine circovirus 2
85708	GCA_004035015.1	Porcine circovirus 2
85708	GCA_004032735.1	Porcine circovirus 2
85708	GCA_004032815.1	Porcine circovirus 2
85708	GCA_004032755.1	Porcine circovirus 2
85708	GCA_004033255.1	Porcine circovirus 2
85708	GCA_004032635.1	Porcine circovirus 2

85708	GCA_004036335.1	Porcine circovirus 2
85708	GCA_004037675.1	Porcine circovirus 2
85708	GCA_004037495.1	Porcine circovirus 2
85708	GCA_004037075.1	Porcine circovirus 2
85708	GCA_004037015.1	Porcine circovirus 2
85708	GCA_004036955.1	Porcine circovirus 2
85708	GCA_004035315.1	Porcine circovirus 2
85708	GCA_004034975.1	Porcine circovirus 2
85708	GCA_004036815.1	Porcine circovirus 2
85708	GCA_004036475.1	Porcine circovirus 2
85708	GCA_004037635.1	Porcine circovirus 2
85708	GCA_004030855.1	Porcine circovirus 2
85708	GCA_004037335.1	Porcine circovirus 2
85708	GCA_004037415.1	Porcine circovirus 2
85708	GCA_004035675.1	Porcine circovirus 2
85708	GCA_004034295.1	Porcine circovirus 2
85708	GCA_004034995.1	Porcine circovirus 2
85708	GCA_004034655.1	Porcine circovirus 2
85708	GCA_004031315.1	Porcine circovirus 2
85708	GCA_004033295.1	Porcine circovirus 2
85708	GCA_004037395.1	Porcine circovirus 2
85708	GCA_004037535.1	Porcine circovirus 2
85708	GCA_004037435.1	Porcine circovirus 2
85708	GCA_004034435.1	Porcine circovirus 2
85708	GCA_004035355.1	Porcine circovirus 2
85708	GCA_004037095.1	Porcine circovirus 2
85708	GCA_004037295.1	Porcine circovirus 2
85708	GCA_004035595.1	Porcine circovirus 2
85708	GCA_004034675.1	Porcine circovirus 2
85708	GCA_004036395.1	Porcine circovirus 2
85708	GCA_004035035.1	Porcine circovirus 2
85708	GCA_004032975.1	Porcine circovirus 2
85708	GCA_004036415.1	Porcine circovirus 2
85708	GCA_004032655.1	Porcine circovirus 2
85708	GCA_004033155.1	Porcine circovirus 2
85708	GCA_004037235.1	Porcine circovirus 2
85708	GCA_004035375.1	Porcine circovirus 2
85708	GCA_004034695.1	Porcine circovirus 2
85708	GCA_004034395.1	Porcine circovirus 2
85708	GCA_004035755.1	Porcine circovirus 2
85708	GCA_004037715.1	Porcine circovirus 2
85708	GCA_004037375.1	Porcine circovirus 2
85708	GCA_004035735.1	Porcine circovirus 2
85708	GCA_004034375.1	Porcine circovirus 2
85708	GCA_004035395.1	Porcine circovirus 2
85708	GCA_004035055.1	Porcine circovirus 2
85708	GCA_004034715.1	Porcine circovirus 2
85708	GCA_004033475.1	Porcine circovirus 2
85708	GCA_004033355.1	Porcine circovirus 2
85708	GCA_004036355.1	Porcine circovirus 2

85708	GCA_004047695.1	Porcine circovirus 2
85708	GCA_004052815.1	Porcine circovirus 2
85708	GCA_004054455.1	Porcine circovirus 2
85708	GCA_004061315.1	Porcine circovirus 2
85708	GCA_004045655.1	Porcine circovirus 2
85708	GCA_004052475.1	Porcine circovirus 2
85708	GCA_004039515.1	Porcine circovirus 2
85708	GCA_004056255.1	Porcine circovirus 2
85708	GCA_004055655.1	Porcine circovirus 2
85708	GCA_004043615.1	Porcine circovirus 2
85708	GCA_004052135.1	Porcine circovirus 2
85708	GCA_004057795.1	Porcine circovirus 2
85708	GCA_004047715.1	Porcine circovirus 2
85708	GCA_004041575.1	Porcine circovirus 2
85708	GCA_004051795.1	Porcine circovirus 2
85708	GCA_004062475.1	Porcine circovirus 2
85708	GCA_004045675.1	Porcine circovirus 2
85708	GCA_004039535.1	Porcine circovirus 2
85708	GCA_004041215.1	Porcine circovirus 2
85708	GCA_004056815.1	Porcine circovirus 2
85708	GCA_004043635.1	Porcine circovirus 2
85708	GCA_004058015.1	Porcine circovirus 2
85708	GCA_004047735.1	Porcine circovirus 2
85708	GCA_004051115.1	Porcine circovirus 2
85708	GCA_004041595.1	Porcine circovirus 2
85708	GCA_004045695.1	Porcine circovirus 2
85708	GCA_004050775.1	Porcine circovirus 2
85708	GCA_004039555.1	Porcine circovirus 2
85708	GCA_004053815.1	Porcine circovirus 2
85708	GCA_004057975.1	Porcine circovirus 2
85708	GCA_004043655.1	Porcine circovirus 2
85708	GCA_004047755.1	Porcine circovirus 2
85708	GCA_004061875.1	Porcine circovirus 2
85708	GCA_004050095.1	Porcine circovirus 2
85708	GCA_004060635.1	Porcine circovirus 2
85708	GCA_004045715.1	Porcine circovirus 2
85708	GCA_004059135.1	Porcine circovirus 2
85708	GCA_004043675.1	Porcine circovirus 2
85708	GCA_004047775.1	Porcine circovirus 2
85708	GCA_004039175.1	Porcine circovirus 2
85708	GCA_004061795.1	Porcine circovirus 2
85708	GCA_004045735.1	Porcine circovirus 2
85708	GCA_004056215.1	Porcine circovirus 2
85708	GCA_004039595.1	Porcine circovirus 2
85708	GCA_004056135.1	Porcine circovirus 2
85708	GCA_004060295.1	Porcine circovirus 2
85708	GCA_004043695.1	Porcine circovirus 2
85708	GCA_004047795.1	Porcine circovirus 2
85708	GCA_004054635.1	Porcine circovirus 2
85708	GCA_004045755.1	Porcine circovirus 2

85708	GCA_004060075.1	Porcine circovirus 2
85708	GCA_004039615.1	Porcine circovirus 2
85708	GCA_004053175.1	Porcine circovirus 2
85708	GCA_004043715.1	Porcine circovirus 2
85708	GCA_004055315.1	Porcine circovirus 2
85708	GCA_004047815.1	Porcine circovirus 2
85708	GCA_004052835.1	Porcine circovirus 2
85708	GCA_004059955.1	Porcine circovirus 2
85708	GCA_004045775.1	Porcine circovirus 2
85708	GCA_004052495.1	Porcine circovirus 2
85708	GCA_004039635.1	Porcine circovirus 2
85708	GCA_004054715.1	Porcine circovirus 2
85708	GCA_004043735.1	Porcine circovirus 2
85708	GCA_004052155.1	Porcine circovirus 2
85708	GCA_004059175.1	Porcine circovirus 2
85708	GCA_004047835.1	Porcine circovirus 2
85708	GCA_004051815.1	Porcine circovirus 2
85708	GCA_004045795.1	Porcine circovirus 2
85708	GCA_004059935.1	Porcine circovirus 2
85708	GCA_004041235.1	Porcine circovirus 2
85708	GCA_004043755.1	Porcine circovirus 2
85708	GCA_004047855.1	Porcine circovirus 2
85708	GCA_004040895.1	Porcine circovirus 2
85708	GCA_004058115.1	Porcine circovirus 2
85708	GCA_004045815.1	Porcine circovirus 2
85708	GCA_004050795.1	Porcine circovirus 2
85708	GCA_004039675.1	Porcine circovirus 2
85708	GCA_004060775.1	Porcine circovirus 2
85708	GCA_004043775.1	Porcine circovirus 2
85708	GCA_004047875.1	Porcine circovirus 2
85708	GCA_004055115.1	Porcine circovirus 2
85708	GCA_004059275.1	Porcine circovirus 2
85708	GCA_004050115.1	Porcine circovirus 2
85708	GCA_004045835.1	Porcine circovirus 2
85708	GCA_004061715.1	Porcine circovirus 2
85708	GCA_004062135.1	Porcine circovirus 2
85708	GCA_004053615.1	Porcine circovirus 2
85708	GCA_004039695.1	Porcine circovirus 2
85708	GCA_004061935.1	Porcine circovirus 2
85708	GCA_004047895.1	Porcine circovirus 2
85708	GCA_004056275.1	Porcine circovirus 2
85708	GCA_004041755.1	Porcine circovirus 2
85708	GCA_004045855.1	Porcine circovirus 2
85708	GCA_004054775.1	Porcine circovirus 2
85708	GCA_004039715.1	Porcine circovirus 2
85708	GCA_004058935.1	Porcine circovirus 2
85708	GCA_004056475.1	Porcine circovirus 2
85708	GCA_004047915.1	Porcine circovirus 2
85708	GCA_004041775.1	Porcine circovirus 2
85708	GCA_004048415.1	Porcine circovirus 2

85708	GCA_004045875.1	Porcine circovirus 2
85708	GCA_004039735.1	Porcine circovirus 2
85708	GCA_004053195.1	Porcine circovirus 2
85708	GCA_004054695.1	Porcine circovirus 2
85708	GCA_004047935.1	Porcine circovirus 2
85708	GCA_004052855.1	Porcine circovirus 2
85708	GCA_004041795.1	Porcine circovirus 2
85708	GCA_004055575.1	Porcine circovirus 2
85708	GCA_004045895.1	Porcine circovirus 2
85708	GCA_004052515.1	Porcine circovirus 2
85708	GCA_004049995.1	Porcine circovirus 2
85708	GCA_004061255.1	Porcine circovirus 2
85708	GCA_004052175.1	Porcine circovirus 2
85708	GCA_004047955.1	Porcine circovirus 2
85708	GCA_004059755.1	Porcine circovirus 2
85708	GCA_004054975.1	Porcine circovirus 2
85708	GCA_004041815.1	Porcine circovirus 2
85708	GCA_004051495.1	Porcine circovirus 2
85708	GCA_004062415.1	Porcine circovirus 2
85708	GCA_004047975.1	Porcine circovirus 2
85708	GCA_004040915.1	Porcine circovirus 2
85708	GCA_004041835.1	Porcine circovirus 2
85708	GCA_004055255.1	Porcine circovirus 2
85708	GCA_004050035.1	Porcine circovirus 2
85708	GCA_004058915.1	Porcine circovirus 2
85708	GCA_004053755.1	Porcine circovirus 2
85708	GCA_004047995.1	Porcine circovirus 2
85708	GCA_004057915.1	Porcine circovirus 2
85708	GCA_004062075.1	Porcine circovirus 2
85708	GCA_004041855.1	Porcine circovirus 2
85708	GCA_004039895.1	Porcine circovirus 2
85708	GCA_004061475.1	Porcine circovirus 2
85708	GCA_004056415.1	Porcine circovirus 2
85708	GCA_004062395.1	Porcine circovirus 2
85708	GCA_004048015.1	Porcine circovirus 2
85708	GCA_004039215.1	Porcine circovirus 2
85708	GCA_004041875.1	Porcine circovirus 2
85708	GCA_004060595.1	Porcine circovirus 2
85708	GCA_004048775.1	Porcine circovirus 2
85708	GCA_004056075.1	Porcine circovirus 2
85708	GCA_004048035.1	Porcine circovirus 2
85708	GCA_004060235.1	Porcine circovirus 2
85708	GCA_004041895.1	Porcine circovirus 2
85708	GCA_004056735.1	Porcine circovirus 2
85708	GCA_004054575.1	Porcine circovirus 2
85708	GCA_004039855.1	Porcine circovirus 2
85708	GCA_004053215.1	Porcine circovirus 2
85708	GCA_004048055.1	Porcine circovirus 2
85708	GCA_004052875.1	Porcine circovirus 2
85708	GCA_004061395.1	Porcine circovirus 2

85708	GCA_004041915.1	Porcine circovirus 2
85708	GCA_004046015.1	Porcine circovirus 2
85708	GCA_004052535.1	Porcine circovirus 2
85708	GCA_004059895.1	Porcine circovirus 2
85708	GCA_004039875.1	Porcine circovirus 2
85708	GCA_004052195.1	Porcine circovirus 2
85708	GCA_004048075.1	Porcine circovirus 2
85708	GCA_004053675.1	Porcine circovirus 2
85708	GCA_004062555.1	Porcine circovirus 2
85708	GCA_004041935.1	Porcine circovirus 2
85708	GCA_004046035.1	Porcine circovirus 2
85708	GCA_004055235.1	Porcine circovirus 2
85708	GCA_004050135.1	Porcine circovirus 2
85708	GCA_004048095.1	Porcine circovirus 2
85708	GCA_004051175.1	Porcine circovirus 2
85708	GCA_004046055.1	Porcine circovirus 2
85708	GCA_004039915.1	Porcine circovirus 2
85708	GCA_004056555.1	Porcine circovirus 2
85708	GCA_004048115.1	Porcine circovirus 2
85708	GCA_004058135.1	Porcine circovirus 2
85708	GCA_004059215.1	Porcine circovirus 2
85708	GCA_004050175.1	Porcine circovirus 2
85708	GCA_004039575.1	Porcine circovirus 2
85708	GCA_004044035.1	Porcine circovirus 2
85708	GCA_004053555.1	Porcine circovirus 2
85708	GCA_004057715.1	Porcine circovirus 2
85708	GCA_004048135.1	Porcine circovirus 2
85708	GCA_004053435.1	Porcine circovirus 2
85708	GCA_004062655.1	Porcine circovirus 2
85708	GCA_004060375.1	Porcine circovirus 2
85708	GCA_004057835.1	Porcine circovirus 2
85708	GCA_004060355.1	Porcine circovirus 2
85708	GCA_004044055.1	Porcine circovirus 2
85708	GCA_004048795.1	Porcine circovirus 2
85708	GCA_004058875.1	Porcine circovirus 2
85708	GCA_004048155.1	Porcine circovirus 2
85708	GCA_004048455.1	Porcine circovirus 2
85708	GCA_004039975.1	Porcine circovirus 2
85708	GCA_004053235.1	Porcine circovirus 2
85708	GCA_004044075.1	Porcine circovirus 2
85708	GCA_004060035.1	Porcine circovirus 2
85708	GCA_004052895.1	Porcine circovirus 2
85708	GCA_004057775.1	Porcine circovirus 2
85708	GCA_004052555.1	Porcine circovirus 2
85708	GCA_004062355.1	Porcine circovirus 2
85708	GCA_004044095.1	Porcine circovirus 2
85708	GCA_004052215.1	Porcine circovirus 2
85708	GCA_004061195.1	Porcine circovirus 2
85708	GCA_004048195.1	Porcine circovirus 2
85708	GCA_004056095.1	Porcine circovirus 2

85708	GCA_004051875.1	Porcine circovirus 2
85708	GCA_004055535.1	Porcine circovirus 2
85708	GCA_004041295.1	Porcine circovirus 2
85708	GCA_004062115.1	Porcine circovirus 2
85708	GCA_004044115.1	Porcine circovirus 2
85708	GCA_004060255.1	Porcine circovirus 2
85708	GCA_004058195.1	Porcine circovirus 2
85708	GCA_004055495.1	Porcine circovirus 2
85708	GCA_004048215.1	Porcine circovirus 2
85708	GCA_004051195.1	Porcine circovirus 2
85708	GCA_004056695.1	Porcine circovirus 2
85708	GCA_004055195.1	Porcine circovirus 2
85708	GCA_004044135.1	Porcine circovirus 2
85708	GCA_004048235.1	Porcine circovirus 2
85708	GCA_004039935.1	Porcine circovirus 2
85708	GCA_004057855.1	Porcine circovirus 2
85708	GCA_004046195.1	Porcine circovirus 2
85708	GCA_004059055.1	Porcine circovirus 2
85708	GCA_004044155.1	Porcine circovirus 2
85708	GCA_004060515.1	Porcine circovirus 2
85708	GCA_004039255.1	Porcine circovirus 2
85708	GCA_004054855.1	Porcine circovirus 2
85708	GCA_004059015.1	Porcine circovirus 2
85708	GCA_004046215.1	Porcine circovirus 2
85708	GCA_004038915.1	Porcine circovirus 2
85708	GCA_004053695.1	Porcine circovirus 2
85708	GCA_004044175.1	Porcine circovirus 2
85708	GCA_004048815.1	Porcine circovirus 2
85708	GCA_004053775.1	Porcine circovirus 2
85708	GCA_004046235.1	Porcine circovirus 2
85708	GCA_004053255.1	Porcine circovirus 2
85708	GCA_004054515.1	Porcine circovirus 2
85708	GCA_004044195.1	Porcine circovirus 2
85708	GCA_004052915.1	Porcine circovirus 2
85708	GCA_004052575.1	Porcine circovirus 2
85708	GCA_004055675.1	Porcine circovirus 2
85708	GCA_004044215.1	Porcine circovirus 2
85708	GCA_004052235.1	Porcine circovirus 2
85708	GCA_004048315.1	Porcine circovirus 2
85708	GCA_004051895.1	Porcine circovirus 2
85708	GCA_004056755.1	Porcine circovirus 2
85708	GCA_004056355.1	Porcine circovirus 2
85708	GCA_004051555.1	Porcine circovirus 2
85708	GCA_004056835.1	Porcine circovirus 2
85708	GCA_004044235.1	Porcine circovirus 2
85708	GCA_004060995.1	Porcine circovirus 2
85708	GCA_004060815.1	Porcine circovirus 2
85708	GCA_004048335.1	Porcine circovirus 2
85708	GCA_004051215.1	Porcine circovirus 2
85708	GCA_004040635.1	Porcine circovirus 2

85708	GCA_004056515.1	Porcine circovirus 2
85708	GCA_004057995.1	Porcine circovirus 2
85708	GCA_004044255.1	Porcine circovirus 2
85708	GCA_004061095.1	Porcine circovirus 2
85708	GCA_004039955.1	Porcine circovirus 2
85708	GCA_004060655.1	Porcine circovirus 2
85708	GCA_004054995.1	Porcine circovirus 2
85708	GCA_004044275.1	Porcine circovirus 2
85708	GCA_004048375.1	Porcine circovirus 2
85708	GCA_004049515.1	Porcine circovirus 2
85708	GCA_004054555.1	Porcine circovirus 2
85708	GCA_004061815.1	Porcine circovirus 2
85708	GCA_004046335.1	Porcine circovirus 2
85708	GCA_004038935.1	Porcine circovirus 2
85708	GCA_004060975.1	Porcine circovirus 2
85708	GCA_004056155.1	Porcine circovirus 2
85708	GCA_004044295.1	Porcine circovirus 2
85708	GCA_004048835.1	Porcine circovirus 2
85708	GCA_004048395.1	Porcine circovirus 2
85708	GCA_004053535.1	Porcine circovirus 2
85708	GCA_004054655.1	Porcine circovirus 2
85708	GCA_004046355.1	Porcine circovirus 2
85708	GCA_004060875.1	Porcine circovirus 2
85708	GCA_004044315.1	Porcine circovirus 2
85708	GCA_004060735.1	Porcine circovirus 2
85708	GCA_004052935.1	Porcine circovirus 2
85708	GCA_004059975.1	Porcine circovirus 2
85708	GCA_004046375.1	Porcine circovirus 2
85708	GCA_004052595.1	Porcine circovirus 2
85708	GCA_004044335.1	Porcine circovirus 2
85708	GCA_004052255.1	Porcine circovirus 2
85708	GCA_004048435.1	Porcine circovirus 2
85708	GCA_004051915.1	Porcine circovirus 2
85708	GCA_004061135.1	Porcine circovirus 2
85708	GCA_004046395.1	Porcine circovirus 2
85708	GCA_004044355.1	Porcine circovirus 2
85708	GCA_004061375.1	Porcine circovirus 2
85708	GCA_004056615.1	Porcine circovirus 2
85708	GCA_004040995.1	Porcine circovirus 2
85708	GCA_004062295.1	Porcine circovirus 2
85708	GCA_004046415.1	Porcine circovirus 2
85708	GCA_004056635.1	Porcine circovirus 2
85708	GCA_004060795.1	Porcine circovirus 2
85708	GCA_004044375.1	Porcine circovirus 2
85708	GCA_004048475.1	Porcine circovirus 2
85708	GCA_004055135.1	Porcine circovirus 2
85708	GCA_004042335.1	Porcine circovirus 2
85708	GCA_004046435.1	Porcine circovirus 2
85708	GCA_004053635.1	Porcine circovirus 2
85708	GCA_004060175.1	Porcine circovirus 2

85708	GCA_004044395.1	Porcine circovirus 2
85708	GCA_004048495.1	Porcine circovirus 2
85708	GCA_004039295.1	Porcine circovirus 2
85708	GCA_004056295.1	Porcine circovirus 2
85708	GCA_004042355.1	Porcine circovirus 2
85708	GCA_004046455.1	Porcine circovirus 2
85708	GCA_004038955.1	Porcine circovirus 2
85708	GCA_004054795.1	Porcine circovirus 2
85708	GCA_004050555.1	Porcine circovirus 2
85708	GCA_004054815.1	Porcine circovirus 2
85708	GCA_004048855.1	Porcine circovirus 2
85708	GCA_004048515.1	Porcine circovirus 2
85708	GCA_004042375.1	Porcine circovirus 2
85708	GCA_004038275.1	Porcine circovirus 2
85708	GCA_004046475.1	Porcine circovirus 2
85708	GCA_004050575.1	Porcine circovirus 2
85708	GCA_004060115.1	Porcine circovirus 2
85708	GCA_004048535.1	Porcine circovirus 2
85708	GCA_004052955.1	Porcine circovirus 2
85708	GCA_004042395.1	Porcine circovirus 2
85708	GCA_004046495.1	Porcine circovirus 2
85708	GCA_004052615.1	Porcine circovirus 2
85708	GCA_004040355.1	Porcine circovirus 2
85708	GCA_004061275.1	Porcine circovirus 2
85708	GCA_004052275.1	Porcine circovirus 2
85708	GCA_004062535.1	Porcine circovirus 2
85708	GCA_004038315.1	Porcine circovirus 2
85708	GCA_004055615.1	Porcine circovirus 2
85708	GCA_004061855.1	Porcine circovirus 2
85708	GCA_004042415.1	Porcine circovirus 2
85708	GCA_004051935.1	Porcine circovirus 2
85708	GCA_004046515.1	Porcine circovirus 2
85708	GCA_004062235.1	Porcine circovirus 2
85708	GCA_004050615.1	Porcine circovirus 2
85708	GCA_004051595.1	Porcine circovirus 2
85708	GCA_004062435.1	Porcine circovirus 2
85708	GCA_004038335.1	Porcine circovirus 2
85708	GCA_004051255.1	Porcine circovirus 2
85708	GCA_004060935.1	Porcine circovirus 2
85708	GCA_004042435.1	Porcine circovirus 2
85708	GCA_004056875.1	Porcine circovirus 2
85708	GCA_004046535.1	Porcine circovirus 2
85708	GCA_004055275.1	Porcine circovirus 2
85708	GCA_004050635.1	Porcine circovirus 2
85708	GCA_004044495.1	Porcine circovirus 2
85708	GCA_004038355.1	Porcine circovirus 2
85708	GCA_004057935.1	Porcine circovirus 2
85708	GCA_004042455.1	Porcine circovirus 2
85708	GCA_004039995.1	Porcine circovirus 2
85708	GCA_004046555.1	Porcine circovirus 2

85708	GCA_004056435.1	Porcine circovirus 2
85708	GCA_004050655.1	Porcine circovirus 2
85708	GCA_004039655.1	Porcine circovirus 2
85708	GCA_004044515.1	Porcine circovirus 2
85708	GCA_004038375.1	Porcine circovirus 2
85708	GCA_004039315.1	Porcine circovirus 2
85708	GCA_004059095.1	Porcine circovirus 2
85708	GCA_004060435.1	Porcine circovirus 2
85708	GCA_004042475.1	Porcine circovirus 2
85708	GCA_004057895.1	Porcine circovirus 2
85708	GCA_004046575.1	Porcine circovirus 2
85708	GCA_004038975.1	Porcine circovirus 2
85708	GCA_004040435.1	Porcine circovirus 2
85708	GCA_004061755.1	Porcine circovirus 2
85708	GCA_004044535.1	Porcine circovirus 2
85708	GCA_004059835.1	Porcine circovirus 2
85708	GCA_004048635.1	Porcine circovirus 2
85708	GCA_004055075.1	Porcine circovirus 2
85708	GCA_004042495.1	Porcine circovirus 2
85708	GCA_004038295.1	Porcine circovirus 2
85708	GCA_004046595.1	Porcine circovirus 2
85708	GCA_004054595.1	Porcine circovirus 2
85708	GCA_004050695.1	Porcine circovirus 2
85708	GCA_004053315.1	Porcine circovirus 2
85708	GCA_004044555.1	Porcine circovirus 2
85708	GCA_004054475.1	Porcine circovirus 2
85708	GCA_004048655.1	Porcine circovirus 2
85708	GCA_004052975.1	Porcine circovirus 2
85708	GCA_004061415.1	Porcine circovirus 2
85708	GCA_004042515.1	Porcine circovirus 2
85708	GCA_004046615.1	Porcine circovirus 2
85708	GCA_004052635.1	Porcine circovirus 2
85708	GCA_004059915.1	Porcine circovirus 2
85708	GCA_004050715.1	Porcine circovirus 2
85708	GCA_004044575.1	Porcine circovirus 2
85708	GCA_004052295.1	Porcine circovirus 2
85708	GCA_004054415.1	Porcine circovirus 2
85708	GCA_004048675.1	Porcine circovirus 2
85708	GCA_004062575.1	Porcine circovirus 2
85708	GCA_004042535.1	Porcine circovirus 2
85708	GCA_004051955.1	Porcine circovirus 2
85708	GCA_004046635.1	Porcine circovirus 2
85708	GCA_004058035.1	Porcine circovirus 2
85708	GCA_004044595.1	Porcine circovirus 2
85708	GCA_004062495.1	Porcine circovirus 2
85708	GCA_004038455.1	Porcine circovirus 2
85708	GCA_004051275.1	Porcine circovirus 2
85708	GCA_004042555.1	Porcine circovirus 2
85708	GCA_004046655.1	Porcine circovirus 2
85708	GCA_004058075.1	Porcine circovirus 2

85708	GCA_004061895.1	Porcine circovirus 2
85708	GCA_004044615.1	Porcine circovirus 2
85708	GCA_004050595.1	Porcine circovirus 2
85708	GCA_004056575.1	Porcine circovirus 2
85708	GCA_004048715.1	Porcine circovirus 2
85708	GCA_004057755.1	Porcine circovirus 2
85708	GCA_004042575.1	Porcine circovirus 2
85708	GCA_004046675.1	Porcine circovirus 2
85708	GCA_004059235.1	Porcine circovirus 2
85708	GCA_004040535.1	Porcine circovirus 2
85708	GCA_004049915.1	Porcine circovirus 2
85708	GCA_004044635.1	Porcine circovirus 2
85708	GCA_004053575.1	Porcine circovirus 2
85708	GCA_004057735.1	Porcine circovirus 2
85708	GCA_004038495.1	Porcine circovirus 2
85708	GCA_004049575.1	Porcine circovirus 2
85708	GCA_004056235.1	Porcine circovirus 2
85708	GCA_004042595.1	Porcine circovirus 2
85708	GCA_004060395.1	Porcine circovirus 2
85708	GCA_004060695.1	Porcine circovirus 2
85708	GCA_004040555.1	Porcine circovirus 2
85708	GCA_004044655.1	Porcine circovirus 2
85708	GCA_004048895.1	Porcine circovirus 2
85708	GCA_004054735.1	Porcine circovirus 2
85708	GCA_004038515.1	Porcine circovirus 2
85708	GCA_004042615.1	Porcine circovirus 2
85708	GCA_004048555.1	Porcine circovirus 2
85708	GCA_004055335.1	Porcine circovirus 2
85708	GCA_004040575.1	Porcine circovirus 2
85708	GCA_004053335.1	Porcine circovirus 2
85708	GCA_004044675.1	Porcine circovirus 2
85708	GCA_004038535.1	Porcine circovirus 2
85708	GCA_004052995.1	Porcine circovirus 2
85708	GCA_004042635.1	Porcine circovirus 2
85708	GCA_004061455.1	Porcine circovirus 2
85708	GCA_004054395.1	Porcine circovirus 2
85708	GCA_004052655.1	Porcine circovirus 2
85708	GCA_004058155.1	Porcine circovirus 2
85708	GCA_004040595.1	Porcine circovirus 2
85708	GCA_004044695.1	Porcine circovirus 2
85708	GCA_004052315.1	Porcine circovirus 2
85708	GCA_004038555.1	Porcine circovirus 2
85708	GCA_004042655.1	Porcine circovirus 2
85708	GCA_004051975.1	Porcine circovirus 2
85708	GCA_004055555.1	Porcine circovirus 2
85708	GCA_004040615.1	Porcine circovirus 2
85708	GCA_004044715.1	Porcine circovirus 2
85708	GCA_004058215.1	Porcine circovirus 2
85708	GCA_004041055.1	Porcine circovirus 2
85708	GCA_004042675.1	Porcine circovirus 2

85708	GCA_004053395.1	Porcine circovirus 2
85708	GCA_004056715.1	Porcine circovirus 2
85708	GCA_004062615.1	Porcine circovirus 2
85708	GCA_004044735.1	Porcine circovirus 2
85708	GCA_004040375.1	Porcine circovirus 2
85708	GCA_004062155.1	Porcine circovirus 2
85708	GCA_004042695.1	Porcine circovirus 2
85708	GCA_004053715.1	Porcine circovirus 2
85708	GCA_004062035.1	Porcine circovirus 2
85708	GCA_004049935.1	Porcine circovirus 2
85708	GCA_004062375.1	Porcine circovirus 2
85708	GCA_004044755.1	Porcine circovirus 2
85708	GCA_004056375.1	Porcine circovirus 2
85708	GCA_004060535.1	Porcine circovirus 2
85708	GCA_004049595.1	Porcine circovirus 2
85708	GCA_004042715.1	Porcine circovirus 2
85708	GCA_004046815.1	Porcine circovirus 2
85708	GCA_004039015.1	Porcine circovirus 2
85708	GCA_004044775.1	Porcine circovirus 2
85708	GCA_004048915.1	Porcine circovirus 2
85708	GCA_004060955.1	Porcine circovirus 2
85708	GCA_004048875.1	Porcine circovirus 2
85708	GCA_004042735.1	Porcine circovirus 2
85708	GCA_004048575.1	Porcine circovirus 2
85708	GCA_004046835.1	Porcine circovirus 2
85708	GCA_004053355.1	Porcine circovirus 2
85708	GCA_004054535.1	Porcine circovirus 2
85708	GCA_004044795.1	Porcine circovirus 2
85708	GCA_004055595.1	Porcine circovirus 2
85708	GCA_004038655.1	Porcine circovirus 2
85708	GCA_004053015.1	Porcine circovirus 2
85708	GCA_004042755.1	Porcine circovirus 2
85708	GCA_004061215.1	Porcine circovirus 2
85708	GCA_004060055.1	Porcine circovirus 2
85708	GCA_004046855.1	Porcine circovirus 2
85708	GCA_004052675.1	Porcine circovirus 2
85708	GCA_004044815.1	Porcine circovirus 2
85708	GCA_004052335.1	Porcine circovirus 2
85708	GCA_004059855.1	Porcine circovirus 2
85708	GCA_004038675.1	Porcine circovirus 2
85708	GCA_004051995.1	Porcine circovirus 2
85708	GCA_004058355.1	Porcine circovirus 2
85708	GCA_004046875.1	Porcine circovirus 2
85708	GCA_004060335.1	Porcine circovirus 2
85708	GCA_004059155.1	Porcine circovirus 2
85708	GCA_004044835.1	Porcine circovirus 2
85708	GCA_004061015.1	Porcine circovirus 2
85708	GCA_004048935.1	Porcine circovirus 2
85708	GCA_004051315.1	Porcine circovirus 2
85708	GCA_004055355.1	Porcine circovirus 2

85708	GCA_004046895.1	Porcine circovirus 2
85708	GCA_004044855.1	Porcine circovirus 2
85708	GCA_004040395.1	Porcine circovirus 2
85708	GCA_004062175.1	Porcine circovirus 2
85708	GCA_004038715.1	Porcine circovirus 2
85708	GCA_004060675.1	Porcine circovirus 2
85708	GCA_004046915.1	Porcine circovirus 2
85708	GCA_004049955.1	Porcine circovirus 2
85708	GCA_004055015.1	Porcine circovirus 2
85708	GCA_004044875.1	Porcine circovirus 2
85708	GCA_004049615.1	Porcine circovirus 2
85708	GCA_004060855.1	Porcine circovirus 2
85708	GCA_004053515.1	Porcine circovirus 2
85708	GCA_004057675.1	Porcine circovirus 2
85708	GCA_004061835.1	Porcine circovirus 2
85708	GCA_004046935.1	Porcine circovirus 2
85708	GCA_004056775.1	Porcine circovirus 2
85708	GCA_004056175.1	Porcine circovirus 2
85708	GCA_004044895.1	Porcine circovirus 2
85708	GCA_004038695.1	Porcine circovirus 2
85708	GCA_004038755.1	Porcine circovirus 2
85708	GCA_004061355.1	Porcine circovirus 2
85708	GCA_004048595.1	Porcine circovirus 2
85708	GCA_004058835.1	Porcine circovirus 2
85708	GCA_004051055.1	Porcine circovirus 2
85708	GCA_004048255.1	Porcine circovirus 2
85708	GCA_004044915.1	Porcine circovirus 2
85708	GCA_004061495.1	Porcine circovirus 2
85708	GCA_004062195.1	Porcine circovirus 2
85708	GCA_004053035.1	Porcine circovirus 2
85708	GCA_004059995.1	Porcine circovirus 2
85708	GCA_004052695.1	Porcine circovirus 2
85708	GCA_004044935.1	Porcine circovirus 2
85708	GCA_004052355.1	Porcine circovirus 2
85708	GCA_004038795.1	Porcine circovirus 2
85708	GCA_004052015.1	Porcine circovirus 2
85708	GCA_004061155.1	Porcine circovirus 2
85708	GCA_004060095.1	Porcine circovirus 2
85708	GCA_004061115.1	Porcine circovirus 2
85708	GCA_004051675.1	Porcine circovirus 2
85708	GCA_004054955.1	Porcine circovirus 2
85708	GCA_004044955.1	Porcine circovirus 2
85708	GCA_004041095.1	Porcine circovirus 2
85708	GCA_004062315.1	Porcine circovirus 2
85708	GCA_004050995.1	Porcine circovirus 2
85708	GCA_004040875.1	Porcine circovirus 2
85708	GCA_004056655.1	Porcine circovirus 2
85708	GCA_004044975.1	Porcine circovirus 2
85708	GCA_004040415.1	Porcine circovirus 2
85708	GCA_004038835.1	Porcine circovirus 2

85708	GCA_004055155.1	Porcine circovirus 2
85708	GCA_004042935.1	Porcine circovirus 2
85708	GCA_004051135.1	Porcine circovirus 2
85708	GCA_004049975.1	Porcine circovirus 2
85708	GCA_004057815.1	Porcine circovirus 2
85708	GCA_004044995.1	Porcine circovirus 2
85708	GCA_004038855.1	Porcine circovirus 2
85708	GCA_004056315.1	Porcine circovirus 2
85708	GCA_004042955.1	Porcine circovirus 2
85708	GCA_004060475.1	Porcine circovirus 2
85708	GCA_004039055.1	Porcine circovirus 2
85708	GCA_004051155.1	Porcine circovirus 2
85708	GCA_004058975.1	Porcine circovirus 2
85708	GCA_004045015.1	Porcine circovirus 2
85708	GCA_004038875.1	Porcine circovirus 2
85708	GCA_004042975.1	Porcine circovirus 2
85708	GCA_004048615.1	Porcine circovirus 2
85708	GCA_004040935.1	Porcine circovirus 2
85708	GCA_004048275.1	Porcine circovirus 2
85708	GCA_004060135.1	Porcine circovirus 2
85708	GCA_004045035.1	Porcine circovirus 2
85708	GCA_004038895.1	Porcine circovirus 2
85708	GCA_004053055.1	Porcine circovirus 2
85708	GCA_004042995.1	Porcine circovirus 2
85708	GCA_004056855.1	Porcine circovirus 2
85708	GCA_004052715.1	Porcine circovirus 2
85708	GCA_004040955.1	Porcine circovirus 2
85708	GCA_004061295.1	Porcine circovirus 2
85708	GCA_004045055.1	Porcine circovirus 2
85708	GCA_004052375.1	Porcine circovirus 2
85708	GCA_004061735.1	Porcine circovirus 2
85708	GCA_004055635.1	Porcine circovirus 2
85708	GCA_004060575.1	Porcine circovirus 2
85708	GCA_004043015.1	Porcine circovirus 2
85708	GCA_004052035.1	Porcine circovirus 2
85708	GCA_004040975.1	Porcine circovirus 2
85708	GCA_004062455.1	Porcine circovirus 2
85708	GCA_004045075.1	Porcine circovirus 2
85708	GCA_004051355.1	Porcine circovirus 2
85708	GCA_004056795.1	Porcine circovirus 2
85708	GCA_004043035.1	Porcine circovirus 2
85708	GCA_004060215.1	Porcine circovirus 2
85708	GCA_004055215.1	Porcine circovirus 2
85708	GCA_004051015.1	Porcine circovirus 2
85708	GCA_004055295.1	Porcine circovirus 2
85708	GCA_004051235.1	Porcine circovirus 2
85708	GCA_004045095.1	Porcine circovirus 2
85708	GCA_004050675.1	Porcine circovirus 2
85708	GCA_004057955.1	Porcine circovirus 2
85708	GCA_004043055.1	Porcine circovirus 2

85708	GCA_004059075.1	Porcine circovirus 2
85708	GCA_004056455.1	Porcine circovirus 2
85708	GCA_004041015.1	Porcine circovirus 2
85708	GCA_004039755.1	Porcine circovirus 2
85708	GCA_004045115.1	Porcine circovirus 2
85708	GCA_004049215.1	Porcine circovirus 2
85708	GCA_004039415.1	Porcine circovirus 2
85708	GCA_004059115.1	Porcine circovirus 2
85708	GCA_004043075.1	Porcine circovirus 2
85708	GCA_004039075.1	Porcine circovirus 2
85708	GCA_004053455.1	Porcine circovirus 2
85708	GCA_004041035.1	Porcine circovirus 2
85708	GCA_004061775.1	Porcine circovirus 2
85708	GCA_004045135.1	Porcine circovirus 2
85708	GCA_004038735.1	Porcine circovirus 2
85708	GCA_004038995.1	Porcine circovirus 2
85708	GCA_004056115.1	Porcine circovirus 2
85708	GCA_004060275.1	Porcine circovirus 2
85708	GCA_004038395.1	Porcine circovirus 2
85708	GCA_004054615.1	Porcine circovirus 2
85708	GCA_004051295.1	Porcine circovirus 2
85708	GCA_004048295.1	Porcine circovirus 2
85708	GCA_004053075.1	Porcine circovirus 2
85708	GCA_004061435.1	Porcine circovirus 2
85708	GCA_004043115.1	Porcine circovirus 2
85708	GCA_004052735.1	Porcine circovirus 2
85708	GCA_004041075.1	Porcine circovirus 2
85708	GCA_004052395.1	Porcine circovirus 2
85708	GCA_004039035.1	Porcine circovirus 2
85708	GCA_004062595.1	Porcine circovirus 2
85708	GCA_004052055.1	Porcine circovirus 2
85708	GCA_004060835.1	Porcine circovirus 2
85708	GCA_004051715.1	Porcine circovirus 2
85708	GCA_004041135.1	Porcine circovirus 2
85708	GCA_004060615.1	Porcine circovirus 2
85708	GCA_004051035.1	Porcine circovirus 2
85708	GCA_004058095.1	Porcine circovirus 2
85708	GCA_004041115.1	Porcine circovirus 2
85708	GCA_004062255.1	Porcine circovirus 2
85708	GCA_004040455.1	Porcine circovirus 2
85708	GCA_004054675.1	Porcine circovirus 2
85708	GCA_004060755.1	Porcine circovirus 2
85708	GCA_004043175.1	Porcine circovirus 2
85708	GCA_004055095.1	Porcine circovirus 2
85708	GCA_004061335.1	Porcine circovirus 2
85708	GCA_004059255.1	Porcine circovirus 2
85708	GCA_004051375.1	Porcine circovirus 2
85708	GCA_004050015.1	Porcine circovirus 2
85708	GCA_004053595.1	Porcine circovirus 2
85708	GCA_004054435.1	Porcine circovirus 2

85708	GCA_004043195.1	Porcine circovirus 2
85708	GCA_004059035.1	Porcine circovirus 2
85708	GCA_004039095.1	Porcine circovirus 2
85708	GCA_004060415.1	Porcine circovirus 2
85708	GCA_004051395.1	Porcine circovirus 2
85708	GCA_004054755.1	Porcine circovirus 2
85708	GCA_004039115.1	Porcine circovirus 2
85708	GCA_004043215.1	Porcine circovirus 2
85708	GCA_004038415.1	Porcine circovirus 2
85708	GCA_004051415.1	Porcine circovirus 2
85708	GCA_004039135.1	Porcine circovirus 2
85708	GCA_004053095.1	Porcine circovirus 2
85708	GCA_004058895.1	Porcine circovirus 2
85708	GCA_004052755.1	Porcine circovirus 2
85708	GCA_004051435.1	Porcine circovirus 2
85708	GCA_004045295.1	Porcine circovirus 2
85708	GCA_004052415.1	Porcine circovirus 2
85708	GCA_004061235.1	Porcine circovirus 2
85708	GCA_004052075.1	Porcine circovirus 2
85708	GCA_004059735.1	Porcine circovirus 2
85708	GCA_004051455.1	Porcine circovirus 2
85708	GCA_004051735.1	Porcine circovirus 2
85708	GCA_004045315.1	Porcine circovirus 2
85708	GCA_004058235.1	Porcine circovirus 2
85708	GCA_004041155.1	Porcine circovirus 2
85708	GCA_004056335.1	Porcine circovirus 2
85708	GCA_004040815.1	Porcine circovirus 2
85708	GCA_004060895.1	Porcine circovirus 2
85708	GCA_004051475.1	Porcine circovirus 2
85708	GCA_004040475.1	Porcine circovirus 2
85708	GCA_004039195.1	Porcine circovirus 2
85708	GCA_004053735.1	Porcine circovirus 2
85708	GCA_004060195.1	Porcine circovirus 2
85708	GCA_004062055.1	Porcine circovirus 2
85708	GCA_004041255.1	Porcine circovirus 2
85708	GCA_004045355.1	Porcine circovirus 2
85708	GCA_004056395.1	Porcine circovirus 2
85708	GCA_004060555.1	Porcine circovirus 2
85708	GCA_004053655.1	Porcine circovirus 2
85708	GCA_004047415.1	Porcine circovirus 2
85708	GCA_004051515.1	Porcine circovirus 2
85708	GCA_004045375.1	Porcine circovirus 2
85708	GCA_004038775.1	Porcine circovirus 2
85708	GCA_004039235.1	Porcine circovirus 2
85708	GCA_004038435.1	Porcine circovirus 2
85708	GCA_004047435.1	Porcine circovirus 2
85708	GCA_004053415.1	Porcine circovirus 2
85708	GCA_004051535.1	Porcine circovirus 2
85708	GCA_004062635.1	Porcine circovirus 2
85708	GCA_004045395.1	Porcine circovirus 2

85708	GCA_004053115.1	Porcine circovirus 2
85708	GCA_004047455.1	Porcine circovirus 2
85708	GCA_004052775.1	Porcine circovirus 2
85708	GCA_004041315.1	Porcine circovirus 2
85708	GCA_004045415.1	Porcine circovirus 2
85708	GCA_004052435.1	Porcine circovirus 2
85708	GCA_004059875.1	Porcine circovirus 2
85708	GCA_004039275.1	Porcine circovirus 2
85708	GCA_004043375.1	Porcine circovirus 2
85708	GCA_004052095.1	Porcine circovirus 2
85708	GCA_004047475.1	Porcine circovirus 2
85708	GCA_004057875.1	Porcine circovirus 2
85708	GCA_004061955.1	Porcine circovirus 2
85708	GCA_004051575.1	Porcine circovirus 2
85708	GCA_004051755.1	Porcine circovirus 2
85708	GCA_004045435.1	Porcine circovirus 2
85708	GCA_004061035.1	Porcine circovirus 2
85708	GCA_004049535.1	Porcine circovirus 2
85708	GCA_004041175.1	Porcine circovirus 2
85708	GCA_004043395.1	Porcine circovirus 2
85708	GCA_004055375.1	Porcine circovirus 2
85708	GCA_004047495.1	Porcine circovirus 2
85708	GCA_004051075.1	Porcine circovirus 2
85708	GCA_004053795.1	Porcine circovirus 2
85708	GCA_004056595.1	Porcine circovirus 2
85708	GCA_004045455.1	Porcine circovirus 2
85708	GCA_004040495.1	Porcine circovirus 2
85708	GCA_004061055.1	Porcine circovirus 2
85708	GCA_004049555.1	Porcine circovirus 2
85708	GCA_004062215.1	Porcine circovirus 2
85708	GCA_004043415.1	Porcine circovirus 2
85708	GCA_004056535.1	Porcine circovirus 2
85708	GCA_004047515.1	Porcine circovirus 2
85708	GCA_004051615.1	Porcine circovirus 2
85708	GCA_004050055.1	Porcine circovirus 2
85708	GCA_004055035.1	Porcine circovirus 2
85708	GCA_004045475.1	Porcine circovirus 2
85708	GCA_004059195.1	Porcine circovirus 2
85708	GCA_004062515.1	Porcine circovirus 2
85708	GCA_004039335.1	Porcine circovirus 2
85708	GCA_004039475.1	Porcine circovirus 2
85708	GCA_004055695.1	Porcine circovirus 2
85708	GCA_004043435.1	Porcine circovirus 2
85708	GCA_004057695.1	Porcine circovirus 2
85708	GCA_004047535.1	Porcine circovirus 2
85708	GCA_004051635.1	Porcine circovirus 2
85708	GCA_004056195.1	Porcine circovirus 2
85708	GCA_004045495.1	Porcine circovirus 2
85708	GCA_004062095.1	Porcine circovirus 2
85708	GCA_004039355.1	Porcine circovirus 2

85708	GCA_004043455.1	Porcine circovirus 2
85708	GCA_004048695.1	Porcine circovirus 2
85708	GCA_004058855.1	Porcine circovirus 2
85708	GCA_004047555.1	Porcine circovirus 2
85708	GCA_004051655.1	Porcine circovirus 2
85708	GCA_004048355.1	Porcine circovirus 2
85708	GCA_004045515.1	Porcine circovirus 2
85708	GCA_004058955.1	Porcine circovirus 2
85708	GCA_004039375.1	Porcine circovirus 2
85708	GCA_004053135.1	Porcine circovirus 2
85708	GCA_004043475.1	Porcine circovirus 2
85708	GCA_004060015.1	Porcine circovirus 2
85708	GCA_004047575.1	Porcine circovirus 2
85708	GCA_004052795.1	Porcine circovirus 2
85708	GCA_004045535.1	Porcine circovirus 2
85708	GCA_004052455.1	Porcine circovirus 2
85708	GCA_004039395.1	Porcine circovirus 2
85708	GCA_004043495.1	Porcine circovirus 2
85708	GCA_004052115.1	Porcine circovirus 2
85708	GCA_004058055.1	Porcine circovirus 2
85708	GCA_004061175.1	Porcine circovirus 2
85708	GCA_004047595.1	Porcine circovirus 2
85708	GCA_004060315.1	Porcine circovirus 2
85708	GCA_004051775.1	Porcine circovirus 2
85708	GCA_004055515.1	Porcine circovirus 2
85708	GCA_004045555.1	Porcine circovirus 2
85708	GCA_004041195.1	Porcine circovirus 2
85708	GCA_004043515.1	Porcine circovirus 2
85708	GCA_004058175.1	Porcine circovirus 2
85708	GCA_004062335.1	Porcine circovirus 2
85708	GCA_004047615.1	Porcine circovirus 2
85708	GCA_004051095.1	Porcine circovirus 2
85708	GCA_004061915.1	Porcine circovirus 2
85708	GCA_004056675.1	Porcine circovirus 2
85708	GCA_004045575.1	Porcine circovirus 2
85708	GCA_004040515.1	Porcine circovirus 2
85708	GCA_004039435.1	Porcine circovirus 2
85708	GCA_004055175.1	Porcine circovirus 2
85708	GCA_004043535.1	Porcine circovirus 2
85708	GCA_004047635.1	Porcine circovirus 2
85708	GCA_004050075.1	Porcine circovirus 2
85708	GCA_004045595.1	Porcine circovirus 2
85708	GCA_004060455.1	Porcine circovirus 2
85708	GCA_004039495.1	Porcine circovirus 2
85708	GCA_004060715.1	Porcine circovirus 2
85708	GCA_004043555.1	Porcine circovirus 2
85708	GCA_004060495.1	Porcine circovirus 2
85708	GCA_004047655.1	Porcine circovirus 2
85708	GCA_004039155.1	Porcine circovirus 2
85708	GCA_004054835.1	Porcine circovirus 2

85708	GCA_004058995.1	Porcine circovirus 2
85708	GCA_004045615.1	Porcine circovirus 2
85708	GCA_004038815.1	Porcine circovirus 2
85708	GCA_004049715.1	Porcine circovirus 2
85708	GCA_004043575.1	Porcine circovirus 2
85708	GCA_004038475.1	Porcine circovirus 2
85708	GCA_004047675.1	Porcine circovirus 2
85708	GCA_004060155.1	Porcine circovirus 2
85708	GCA_004045635.1	Porcine circovirus 2
85708	GCA_004056495.1	Porcine circovirus 2
85708	GCA_004055055.1	Porcine circovirus 2
85708	GCA_004053155.1	Porcine circovirus 2
85708	GCA_004054495.1	Porcine circovirus 2
85708	GCA_004043595.1	Porcine circovirus 2
85708	GCA_004061075.1	Porcine circovirus 2
85708	GCA_004073295.1	Porcine circovirus 2
85708	GCA_004066135.1	Porcine circovirus 2
85708	GCA_004072955.1	Porcine circovirus 2
85708	GCA_004084095.1	Porcine circovirus 2
85708	GCA_004070235.1	Porcine circovirus 2
85708	GCA_004077735.1	Porcine circovirus 2
85708	GCA_004086275.1	Porcine circovirus 2
85708	GCA_004066155.1	Porcine circovirus 2
85708	GCA_004070255.1	Porcine circovirus 2
85708	GCA_004077055.1	Porcine circovirus 2
85708	GCA_004083275.1	Porcine circovirus 2
85708	GCA_004066175.1	Porcine circovirus 2
85708	GCA_004070275.1	Porcine circovirus 2
85708	GCA_004076035.1	Porcine circovirus 2
85708	GCA_004084435.1	Porcine circovirus 2
85708	GCA_004080315.1	Porcine circovirus 2
85708	GCA_004075695.1	Porcine circovirus 2
85708	GCA_004082935.1	Porcine circovirus 2
85708	GCA_004066195.1	Porcine circovirus 2
85708	GCA_004070295.1	Porcine circovirus 2
85708	GCA_004075355.1	Porcine circovirus 2
85708	GCA_004080015.1	Porcine circovirus 2
85708	GCA_004085595.1	Porcine circovirus 2
85708	GCA_004068255.1	Porcine circovirus 2
85708	GCA_004075015.1	Porcine circovirus 2
85708	GCA_004079935.1	Porcine circovirus 2
85708	GCA_004083215.1	Porcine circovirus 2
85708	GCA_004066215.1	Porcine circovirus 2
85708	GCA_004064435.1	Porcine circovirus 2
85708	GCA_004083495.1	Porcine circovirus 2
85708	GCA_004070315.1	Porcine circovirus 2
85708	GCA_004078435.1	Porcine circovirus 2
85708	GCA_004086755.1	Porcine circovirus 2
85708	GCA_004073995.1	Porcine circovirus 2
85708	GCA_004085255.1	Porcine circovirus 2

85708	GCA_004066235.1	Porcine circovirus 2
85708	GCA_004070335.1	Porcine circovirus 2
85708	GCA_004073655.1	Porcine circovirus 2
85708	GCA_004079595.1	Porcine circovirus 2
85708	GCA_004078515.1	Porcine circovirus 2
85708	GCA_004068295.1	Porcine circovirus 2
85708	GCA_004073315.1	Porcine circovirus 2
85708	GCA_004072395.1	Porcine circovirus 2
85708	GCA_004082255.1	Porcine circovirus 2
85708	GCA_004066255.1	Porcine circovirus 2
85708	GCA_004072975.1	Porcine circovirus 2
85708	GCA_004070355.1	Porcine circovirus 2
85708	GCA_004064215.1	Porcine circovirus 2
85708	GCA_004077755.1	Porcine circovirus 2
85708	GCA_004068315.1	Porcine circovirus 2
85708	GCA_004072415.1	Porcine circovirus 2
85708	GCA_004083415.1	Porcine circovirus 2
85708	GCA_004066275.1	Porcine circovirus 2
85708	GCA_004070375.1	Porcine circovirus 2
85708	GCA_004077075.1	Porcine circovirus 2
85708	GCA_004078495.1	Porcine circovirus 2
85708	GCA_004064235.1	Porcine circovirus 2
85708	GCA_004083075.1	Porcine circovirus 2
85708	GCA_004081775.1	Porcine circovirus 2
85708	GCA_004068335.1	Porcine circovirus 2
85708	GCA_004078375.1	Porcine circovirus 2
85708	GCA_004072435.1	Porcine circovirus 2
85708	GCA_004084575.1	Porcine circovirus 2
85708	GCA_004066295.1	Porcine circovirus 2
85708	GCA_004085795.1	Porcine circovirus 2
85708	GCA_004070395.1	Porcine circovirus 2
85708	GCA_004081175.1	Porcine circovirus 2
85708	GCA_004064255.1	Porcine circovirus 2
85708	GCA_004068355.1	Porcine circovirus 2
85708	GCA_004072455.1	Porcine circovirus 2
85708	GCA_004075715.1	Porcine circovirus 2
85708	GCA_004085735.1	Porcine circovirus 2
85708	GCA_004066315.1	Porcine circovirus 2
85708	GCA_004070415.1	Porcine circovirus 2
85708	GCA_004075375.1	Porcine circovirus 2
85708	GCA_004084915.1	Porcine circovirus 2
85708	GCA_004084235.1	Porcine circovirus 2
85708	GCA_004068375.1	Porcine circovirus 2
85708	GCA_004064795.1	Porcine circovirus 2
85708	GCA_004072475.1	Porcine circovirus 2
85708	GCA_004082735.1	Porcine circovirus 2
85708	GCA_004086895.1	Porcine circovirus 2
85708	GCA_004066335.1	Porcine circovirus 2
85708	GCA_004064455.1	Porcine circovirus 2
85708	GCA_004070435.1	Porcine circovirus 2

85708	GCA_004081235.1	Porcine circovirus 2
85708	GCA_004064295.1	Porcine circovirus 2
85708	GCA_004082715.1	Porcine circovirus 2
85708	GCA_004068395.1	Porcine circovirus 2
85708	GCA_004084135.1	Porcine circovirus 2
85708	GCA_004079735.1	Porcine circovirus 2
85708	GCA_004072495.1	Porcine circovirus 2
85708	GCA_004066355.1	Porcine circovirus 2
85708	GCA_004070455.1	Porcine circovirus 2
85708	GCA_004082395.1	Porcine circovirus 2
85708	GCA_004082475.1	Porcine circovirus 2
85708	GCA_004068415.1	Porcine circovirus 2
85708	GCA_004073335.1	Porcine circovirus 2
85708	GCA_004072515.1	Porcine circovirus 2
85708	GCA_004078775.1	Porcine circovirus 2
85708	GCA_004066375.1	Porcine circovirus 2
85708	GCA_004070475.1	Porcine circovirus 2
85708	GCA_004083555.1	Porcine circovirus 2
85708	GCA_004064335.1	Porcine circovirus 2
85708	GCA_004077775.1	Porcine circovirus 2
85708	GCA_004072535.1	Porcine circovirus 2
85708	GCA_004077435.1	Porcine circovirus 2
85708	GCA_004082635.1	Porcine circovirus 2
85708	GCA_004066395.1	Porcine circovirus 2
85708	GCA_004070495.1	Porcine circovirus 2
85708	GCA_004077095.1	Porcine circovirus 2
85708	GCA_004084715.1	Porcine circovirus 2
85708	GCA_004082355.1	Porcine circovirus 2
85708	GCA_004072555.1	Porcine circovirus 2
85708	GCA_004066415.1	Porcine circovirus 2
85708	GCA_004070515.1	Porcine circovirus 2
85708	GCA_004081715.1	Porcine circovirus 2
85708	GCA_004080215.1	Porcine circovirus 2
85708	GCA_004081435.1	Porcine circovirus 2
85708	GCA_004072575.1	Porcine circovirus 2
85708	GCA_004075735.1	Porcine circovirus 2
85708	GCA_004086695.1	Porcine circovirus 2
85708	GCA_004066435.1	Porcine circovirus 2
85708	GCA_004078715.1	Porcine circovirus 2
85708	GCA_004070535.1	Porcine circovirus 2
85708	GCA_004075395.1	Porcine circovirus 2
85708	GCA_004064395.1	Porcine circovirus 2
85708	GCA_004075055.1	Porcine circovirus 2
85708	GCA_004081375.1	Porcine circovirus 2
85708	GCA_004072595.1	Porcine circovirus 2
85708	GCA_004066455.1	Porcine circovirus 2
85708	GCA_004079875.1	Porcine circovirus 2
85708	GCA_004070555.1	Porcine circovirus 2
85708	GCA_004084035.1	Porcine circovirus 2
85708	GCA_004081935.1	Porcine circovirus 2

85708	GCA_004072615.1	Porcine circovirus 2
85708	GCA_004063795.1	Porcine circovirus 2
85708	GCA_004066475.1	Porcine circovirus 2
85708	GCA_004084395.1	Porcine circovirus 2
85708	GCA_004070575.1	Porcine circovirus 2
85708	GCA_004081815.1	Porcine circovirus 2
85708	GCA_004079635.1	Porcine circovirus 2
85708	GCA_004063115.1	Porcine circovirus 2
85708	GCA_004083695.1	Porcine circovirus 2
85708	GCA_004072635.1	Porcine circovirus 2
85708	GCA_004081695.1	Porcine circovirus 2
85708	GCA_004066495.1	Porcine circovirus 2
85708	GCA_004062775.1	Porcine circovirus 2
85708	GCA_004078035.1	Porcine circovirus 2
85708	GCA_004070595.1	Porcine circovirus 2
85708	GCA_004086355.1	Porcine circovirus 2
85708	GCA_004077795.1	Porcine circovirus 2
85708	GCA_004081575.1	Porcine circovirus 2
85708	GCA_004080695.1	Porcine circovirus 2
85708	GCA_004084855.1	Porcine circovirus 2
85708	GCA_004072655.1	Porcine circovirus 2
85708	GCA_004078735.1	Porcine circovirus 2
85708	GCA_004066515.1	Porcine circovirus 2
85708	GCA_004064475.1	Porcine circovirus 2
85708	GCA_004072675.1	Porcine circovirus 2
85708	GCA_004082595.1	Porcine circovirus 2
85708	GCA_004066535.1	Porcine circovirus 2
85708	GCA_004080355.1	Porcine circovirus 2
85708	GCA_004064495.1	Porcine circovirus 2
85708	GCA_004078855.1	Porcine circovirus 2
85708	GCA_004083015.1	Porcine circovirus 2
85708	GCA_004081335.1	Porcine circovirus 2
85708	GCA_004072695.1	Porcine circovirus 2
85708	GCA_004075755.1	Porcine circovirus 2
85708	GCA_004086315.1	Porcine circovirus 2
85708	GCA_004066555.1	Porcine circovirus 2
85708	GCA_004081515.1	Porcine circovirus 2
85708	GCA_004075415.1	Porcine circovirus 2
85708	GCA_004064835.1	Porcine circovirus 2
85708	GCA_004084175.1	Porcine circovirus 2
85708	GCA_004072715.1	Porcine circovirus 2
85708	GCA_004081095.1	Porcine circovirus 2
85708	GCA_004066575.1	Porcine circovirus 2
85708	GCA_004085435.1	Porcine circovirus 2
85708	GCA_004082675.1	Porcine circovirus 2
85708	GCA_004086835.1	Porcine circovirus 2
85708	GCA_004085675.1	Porcine circovirus 2
85708	GCA_004064535.1	Porcine circovirus 2
85708	GCA_004085335.1	Porcine circovirus 2
85708	GCA_004072735.1	Porcine circovirus 2

85708	GCA_004063815.1	Porcine circovirus 2
85708	GCA_004066595.1	Porcine circovirus 2
85708	GCA_004083835.1	Porcine circovirus 2
85708	GCA_004064555.1	Porcine circovirus 2
85708	GCA_004078175.1	Porcine circovirus 2
85708	GCA_004073375.1	Porcine circovirus 2
85708	GCA_004072755.1	Porcine circovirus 2
85708	GCA_004079895.1	Porcine circovirus 2
85708	GCA_004066615.1	Porcine circovirus 2
85708	GCA_004073035.1	Porcine circovirus 2
85708	GCA_004084995.1	Porcine circovirus 2
85708	GCA_004064575.1	Porcine circovirus 2
85708	GCA_004077815.1	Porcine circovirus 2
85708	GCA_004072775.1	Porcine circovirus 2
85708	GCA_004083755.1	Porcine circovirus 2
85708	GCA_004066635.1	Porcine circovirus 2
85708	GCA_004081995.1	Porcine circovirus 2
85708	GCA_004085315.1	Porcine circovirus 2
85708	GCA_004077135.1	Porcine circovirus 2
85708	GCA_004064595.1	Porcine circovirus 2
85708	GCA_004085195.1	Porcine circovirus 2
85708	GCA_004072795.1	Porcine circovirus 2
85708	GCA_004066655.1	Porcine circovirus 2
85708	GCA_004083155.1	Porcine circovirus 2
85708	GCA_004082855.1	Porcine circovirus 2
85708	GCA_004081655.1	Porcine circovirus 2
85708	GCA_004085815.1	Porcine circovirus 2
85708	GCA_004072815.1	Porcine circovirus 2
85708	GCA_004065535.1	Porcine circovirus 2
85708	GCA_004066675.1	Porcine circovirus 2
85708	GCA_004084315.1	Porcine circovirus 2
85708	GCA_004075435.1	Porcine circovirus 2
85708	GCA_004064635.1	Porcine circovirus 2
85708	GCA_004086715.1	Porcine circovirus 2
85708	GCA_004082815.1	Porcine circovirus 2
85708	GCA_004064855.1	Porcine circovirus 2
85708	GCA_004072835.1	Porcine circovirus 2
85708	GCA_004066695.1	Porcine circovirus 2
85708	GCA_004064515.1	Porcine circovirus 2
85708	GCA_004085475.1	Porcine circovirus 2
85708	GCA_004074895.1	Porcine circovirus 2
85708	GCA_004083975.1	Porcine circovirus 2
85708	GCA_004072855.1	Porcine circovirus 2
85708	GCA_004074075.1	Porcine circovirus 2
85708	GCA_004078315.1	Porcine circovirus 2
85708	GCA_004066715.1	Porcine circovirus 2
85708	GCA_004084775.1	Porcine circovirus 2
85708	GCA_004083875.1	Porcine circovirus 2
85708	GCA_004074915.1	Porcine circovirus 2
85708	GCA_004080975.1	Porcine circovirus 2

85708	GCA_004073395.1	Porcine circovirus 2
85708	GCA_004072875.1	Porcine circovirus 2
85708	GCA_004066735.1	Porcine circovirus 2
85708	GCA_004062815.1	Porcine circovirus 2
85708	GCA_004080155.1	Porcine circovirus 2
85708	GCA_004074935.1	Porcine circovirus 2
85708	GCA_004077835.1	Porcine circovirus 2
85708	GCA_004072895.1	Porcine circovirus 2
85708	GCA_004084655.1	Porcine circovirus 2
85708	GCA_004066755.1	Porcine circovirus 2
85708	GCA_004084795.1	Porcine circovirus 2
85708	GCA_004074955.1	Porcine circovirus 2
85708	GCA_004083295.1	Porcine circovirus 2
85708	GCA_004084015.1	Porcine circovirus 2
85708	GCA_004072915.1	Porcine circovirus 2
85708	GCA_004084415.1	Porcine circovirus 2
85708	GCA_004081795.1	Porcine circovirus 2
85708	GCA_004066775.1	Porcine circovirus 2
85708	GCA_004074975.1	Porcine circovirus 2
85708	GCA_004084455.1	Porcine circovirus 2
85708	GCA_004083115.1	Porcine circovirus 2
85708	GCA_004065555.1	Porcine circovirus 2
85708	GCA_004082955.1	Porcine circovirus 2
85708	GCA_004066795.1	Porcine circovirus 2
85708	GCA_004075455.1	Porcine circovirus 2
85708	GCA_004074995.1	Porcine circovirus 2
85708	GCA_004081455.1	Porcine circovirus 2
85708	GCA_004085615.1	Porcine circovirus 2
85708	GCA_004064875.1	Porcine circovirus 2
85708	GCA_004062715.1	Porcine circovirus 2
85708	GCA_004079955.1	Porcine circovirus 2
85708	GCA_004066815.1	Porcine circovirus 2
85708	GCA_004078455.1	Porcine circovirus 2
85708	GCA_004064775.1	Porcine circovirus 2
85708	GCA_004064195.1	Porcine circovirus 2
85708	GCA_004081915.1	Porcine circovirus 2
85708	GCA_004062735.1	Porcine circovirus 2
85708	GCA_004074095.1	Porcine circovirus 2
85708	GCA_004081115.1	Porcine circovirus 2
85708	GCA_004066835.1	Porcine circovirus 2
85708	GCA_004079615.1	Porcine circovirus 2
85708	GCA_004075035.1	Porcine circovirus 2
85708	GCA_004083775.1	Porcine circovirus 2
85708	GCA_004063175.1	Porcine circovirus 2
85708	GCA_004062755.1	Porcine circovirus 2
85708	GCA_004082275.1	Porcine circovirus 2
85708	GCA_004066855.1	Porcine circovirus 2
85708	GCA_004064815.1	Porcine circovirus 2
85708	GCA_004077855.1	Porcine circovirus 2
85708	GCA_004080415.1	Porcine circovirus 2

85708	GCA_004077515.1	Porcine circovirus 2
85708	GCA_004083435.1	Porcine circovirus 2
85708	GCA_004066875.1	Porcine circovirus 2
85708	GCA_004075075.1	Porcine circovirus 2
85708	GCA_004079815.1	Porcine circovirus 2
85708	GCA_004062795.1	Porcine circovirus 2
85708	GCA_004080435.1	Porcine circovirus 2
85708	GCA_004084595.1	Porcine circovirus 2
85708	GCA_004066895.1	Porcine circovirus 2
85708	GCA_004078995.1	Porcine circovirus 2
85708	GCA_004083635.1	Porcine circovirus 2
85708	GCA_004068955.1	Porcine circovirus 2
85708	GCA_004073055.1	Porcine circovirus 2
85708	GCA_004065575.1	Porcine circovirus 2
85708	GCA_004085755.1	Porcine circovirus 2
85708	GCA_004066915.1	Porcine circovirus 2
85708	GCA_004075475.1	Porcine circovirus 2
85708	GCA_004080095.1	Porcine circovirus 2
85708	GCA_004078755.1	Porcine circovirus 2
85708	GCA_004075115.1	Porcine circovirus 2
85708	GCA_004068975.1	Porcine circovirus 2
85708	GCA_004064895.1	Porcine circovirus 2
85708	GCA_004062835.1	Porcine circovirus 2
85708	GCA_004082755.1	Porcine circovirus 2
85708	GCA_004086915.1	Porcine circovirus 2
85708	GCA_004066935.1	Porcine circovirus 2
85708	GCA_004081255.1	Porcine circovirus 2
85708	GCA_004068995.1	Porcine circovirus 2
85708	GCA_004079755.1	Porcine circovirus 2
85708	GCA_004063875.1	Porcine circovirus 2
85708	GCA_004066955.1	Porcine circovirus 2
85708	GCA_004063555.1	Porcine circovirus 2
85708	GCA_004082415.1	Porcine circovirus 2
85708	GCA_004069015.1	Porcine circovirus 2
85708	GCA_004085075.1	Porcine circovirus 2
85708	GCA_004066975.1	Porcine circovirus 2
85708	GCA_004062855.1	Porcine circovirus 2
85708	GCA_004078395.1	Porcine circovirus 2
85708	GCA_004083575.1	Porcine circovirus 2
85708	GCA_004075175.1	Porcine circovirus 2
85708	GCA_004077875.1	Porcine circovirus 2
85708	GCA_004082975.1	Porcine circovirus 2
85708	GCA_004069035.1	Porcine circovirus 2
85708	GCA_004077535.1	Porcine circovirus 2
85708	GCA_004066995.1	Porcine circovirus 2
85708	GCA_004084735.1	Porcine circovirus 2
85708	GCA_004075195.1	Porcine circovirus 2
85708	GCA_004085135.1	Porcine circovirus 2
85708	GCA_004069055.1	Porcine circovirus 2
85708	GCA_004084835.1	Porcine circovirus 2

85708	GCA_004080075.1	Porcine circovirus 2
85708	GCA_004067015.1	Porcine circovirus 2
85708	GCA_004081735.1	Porcine circovirus 2
85708	GCA_004075215.1	Porcine circovirus 2
85708	GCA_004079775.1	Porcine circovirus 2
85708	GCA_004069075.1	Porcine circovirus 2
85708	GCA_004080235.1	Porcine circovirus 2
85708	GCA_004062935.1	Porcine circovirus 2
85708	GCA_004075835.1	Porcine circovirus 2
85708	GCA_004078095.1	Porcine circovirus 2
85708	GCA_004067035.1	Porcine circovirus 2
85708	GCA_004075495.1	Porcine circovirus 2
85708	GCA_004082895.1	Porcine circovirus 2
85708	GCA_004075235.1	Porcine circovirus 2
85708	GCA_004064915.1	Porcine circovirus 2
85708	GCA_004081395.1	Porcine circovirus 2
85708	GCA_004062955.1	Porcine circovirus 2
85708	GCA_004078875.1	Porcine circovirus 2
85708	GCA_004067055.1	Porcine circovirus 2
85708	GCA_004084055.1	Porcine circovirus 2
85708	GCA_004075255.1	Porcine circovirus 2
85708	GCA_004082375.1	Porcine circovirus 2
85708	GCA_004069115.1	Porcine circovirus 2
85708	GCA_004078275.1	Porcine circovirus 2
85708	GCA_004082555.1	Porcine circovirus 2
85708	GCA_004063895.1	Porcine circovirus 2
85708	GCA_004067075.1	Porcine circovirus 2
85708	GCA_004078915.1	Porcine circovirus 2
85708	GCA_004085215.1	Porcine circovirus 2
85708	GCA_004082435.1	Porcine circovirus 2
85708	GCA_004075275.1	Porcine circovirus 2
85708	GCA_004063215.1	Porcine circovirus 2
85708	GCA_004083715.1	Porcine circovirus 2
85708	GCA_004062995.1	Porcine circovirus 2
85708	GCA_004067095.1	Porcine circovirus 2
85708	GCA_004078055.1	Porcine circovirus 2
85708	GCA_004086375.1	Porcine circovirus 2
85708	GCA_004075295.1	Porcine circovirus 2
85708	GCA_004077895.1	Porcine circovirus 2
85708	GCA_004080715.1	Porcine circovirus 2
85708	GCA_004084875.1	Porcine circovirus 2
85708	GCA_004063015.1	Porcine circovirus 2
85708	GCA_004077555.1	Porcine circovirus 2
85708	GCA_004067115.1	Porcine circovirus 2
85708	GCA_004086775.1	Porcine circovirus 2
85708	GCA_004071215.1	Porcine circovirus 2
85708	GCA_004077215.1	Porcine circovirus 2
85708	GCA_004075315.1	Porcine circovirus 2
85708	GCA_004085695.1	Porcine circovirus 2
85708	GCA_004081875.1	Porcine circovirus 2

85708	GCA_004073275.1	Porcine circovirus 2
85708	GCA_004081955.1	Porcine circovirus 2
85708	GCA_004085395.1	Porcine circovirus 2
85708	GCA_004067135.1	Porcine circovirus 2
85708	GCA_004080375.1	Porcine circovirus 2
85708	GCA_004071235.1	Porcine circovirus 2
85708	GCA_004084535.1	Porcine circovirus 2
85708	GCA_004083735.1	Porcine circovirus 2
85708	GCA_004075335.1	Porcine circovirus 2
85708	GCA_004081835.1	Porcine circovirus 2
85708	GCA_004069195.1	Porcine circovirus 2
85708	GCA_004083035.1	Porcine circovirus 2
85708	GCA_004063055.1	Porcine circovirus 2
85708	GCA_004065615.1	Porcine circovirus 2
85708	GCA_004080035.1	Porcine circovirus 2
85708	GCA_004067155.1	Porcine circovirus 2
85708	GCA_004081535.1	Porcine circovirus 2
85708	GCA_004071255.1	Porcine circovirus 2
85708	GCA_004075515.1	Porcine circovirus 2
85708	GCA_004084495.1	Porcine circovirus 2
85708	GCA_004065115.1	Porcine circovirus 2
85708	GCA_004069215.1	Porcine circovirus 2
85708	GCA_004064935.1	Porcine circovirus 2
85708	GCA_004084195.1	Porcine circovirus 2
85708	GCA_004067175.1	Porcine circovirus 2
85708	GCA_004082695.1	Porcine circovirus 2
85708	GCA_004071275.1	Porcine circovirus 2
85708	GCA_004086855.1	Porcine circovirus 2
85708	GCA_004065135.1	Porcine circovirus 2
85708	GCA_004086295.1	Porcine circovirus 2
85708	GCA_004069235.1	Porcine circovirus 2
85708	GCA_004085355.1	Porcine circovirus 2
85708	GCA_004063915.1	Porcine circovirus 2
85708	GCA_004081595.1	Porcine circovirus 2
85708	GCA_004067195.1	Porcine circovirus 2
85708	GCA_004079695.1	Porcine circovirus 2
85708	GCA_004071295.1	Porcine circovirus 2
85708	GCA_004078935.1	Porcine circovirus 2
85708	GCA_004065155.1	Porcine circovirus 2
85708	GCA_004078195.1	Porcine circovirus 2
85708	GCA_004063235.1	Porcine circovirus 2
85708	GCA_004073355.1	Porcine circovirus 2
85708	GCA_004081355.1	Porcine circovirus 2
85708	GCA_004067215.1	Porcine circovirus 2
85708	GCA_004071315.1	Porcine circovirus 2
85708	GCA_004065175.1	Porcine circovirus 2
85708	GCA_004077915.1	Porcine circovirus 2
85708	GCA_004083515.1	Porcine circovirus 2
85708	GCA_004063135.1	Porcine circovirus 2
85708	GCA_004077575.1	Porcine circovirus 2

85708	GCA_004067235.1	Porcine circovirus 2
85708	GCA_004082015.1	Porcine circovirus 2
85708	GCA_004071335.1	Porcine circovirus 2
85708	GCA_004077235.1	Porcine circovirus 2
85708	GCA_004065195.1	Porcine circovirus 2
85708	GCA_004063155.1	Porcine circovirus 2
85708	GCA_004081195.1	Porcine circovirus 2
85708	GCA_004067255.1	Porcine circovirus 2
85708	GCA_004083175.1	Porcine circovirus 2
85708	GCA_004071355.1	Porcine circovirus 2
85708	GCA_004065215.1	Porcine circovirus 2
85708	GCA_004081675.1	Porcine circovirus 2
85708	GCA_004073415.1	Porcine circovirus 2
85708	GCA_004065635.1	Porcine circovirus 2
85708	GCA_004067275.1	Porcine circovirus 2
85708	GCA_004080175.1	Porcine circovirus 2
85708	GCA_004084335.1	Porcine circovirus 2
85708	GCA_004071375.1	Porcine circovirus 2
85708	GCA_004065295.1	Porcine circovirus 2
85708	GCA_004080295.1	Porcine circovirus 2
85708	GCA_004065235.1	Porcine circovirus 2
85708	GCA_004084115.1	Porcine circovirus 2
85708	GCA_004064955.1	Porcine circovirus 2
85708	GCA_004084755.1	Porcine circovirus 2
85708	GCA_004063195.1	Porcine circovirus 2
85708	GCA_004067295.1	Porcine circovirus 2
85708	GCA_004064615.1	Porcine circovirus 2
85708	GCA_004085495.1	Porcine circovirus 2
85708	GCA_004071395.1	Porcine circovirus 2
85708	GCA_004065255.1	Porcine circovirus 2
85708	GCA_004064275.1	Porcine circovirus 2
85708	GCA_004079835.1	Porcine circovirus 2
85708	GCA_004084155.1	Porcine circovirus 2
85708	GCA_004074175.1	Porcine circovirus 2
85708	GCA_004078335.1	Porcine circovirus 2
85708	GCA_004067315.1	Porcine circovirus 2
85708	GCA_004082495.1	Porcine circovirus 2
85708	GCA_004071415.1	Porcine circovirus 2
85708	GCA_004063595.1	Porcine circovirus 2
85708	GCA_004065275.1	Porcine circovirus 2
85708	GCA_004078795.1	Porcine circovirus 2
85708	GCA_004067335.1	Porcine circovirus 2
85708	GCA_004071435.1	Porcine circovirus 2
85708	GCA_004075535.1	Porcine circovirus 2
85708	GCA_004077935.1	Porcine circovirus 2
85708	GCA_004085095.1	Porcine circovirus 2
85708	GCA_004063255.1	Porcine circovirus 2
85708	GCA_004077595.1	Porcine circovirus 2
85708	GCA_004067355.1	Porcine circovirus 2
85708	GCA_004085035.1	Porcine circovirus 2

85708	GCA_004071455.1	Porcine circovirus 2
85708	GCA_004077255.1	Porcine circovirus 2
85708	GCA_004075555.1	Porcine circovirus 2
85708	GCA_004083315.1	Porcine circovirus 2
85708	GCA_004076915.1	Porcine circovirus 2
85708	GCA_004063275.1	Porcine circovirus 2
85708	GCA_004067375.1	Porcine circovirus 2
85708	GCA_004071475.1	Porcine circovirus 2
85708	GCA_004065335.1	Porcine circovirus 2
85708	GCA_004084475.1	Porcine circovirus 2
85708	GCA_004073535.1	Porcine circovirus 2
85708	GCA_004075895.1	Porcine circovirus 2
85708	GCA_004078815.1	Porcine circovirus 2
85708	GCA_004067395.1	Porcine circovirus 2
85708	GCA_004084255.1	Porcine circovirus 2
85708	GCA_004071495.1	Porcine circovirus 2
85708	GCA_004065315.1	Porcine circovirus 2
85708	GCA_004065355.1	Porcine circovirus 2
85708	GCA_004081475.1	Porcine circovirus 2
85708	GCA_004085635.1	Porcine circovirus 2
85708	GCA_004064975.1	Porcine circovirus 2
85708	GCA_004079975.1	Porcine circovirus 2
85708	GCA_004067415.1	Porcine circovirus 2
85708	GCA_004084675.1	Porcine circovirus 2
85708	GCA_004071515.1	Porcine circovirus 2
85708	GCA_004078475.1	Porcine circovirus 2
85708	GCA_004065375.1	Porcine circovirus 2
85708	GCA_004086795.1	Porcine circovirus 2
85708	GCA_004074195.1	Porcine circovirus 2
85708	GCA_004081135.1	Porcine circovirus 2
85708	GCA_004067435.1	Porcine circovirus 2
85708	GCA_004085295.1	Porcine circovirus 2
85708	GCA_004079655.1	Porcine circovirus 2
85708	GCA_004071535.1	Porcine circovirus 2
85708	GCA_004073855.1	Porcine circovirus 2
85708	GCA_004065395.1	Porcine circovirus 2
85708	GCA_004083795.1	Porcine circovirus 2
85708	GCA_004073515.1	Porcine circovirus 2
85708	GCA_004073595.1	Porcine circovirus 2
85708	GCA_004078135.1	Porcine circovirus 2
85708	GCA_004067455.1	Porcine circovirus 2
85708	GCA_004071555.1	Porcine circovirus 2
85708	GCA_004086815.1	Porcine circovirus 2
85708	GCA_004065415.1	Porcine circovirus 2
85708	GCA_004077955.1	Porcine circovirus 2
85708	GCA_004084955.1	Porcine circovirus 2
85708	GCA_004073615.1	Porcine circovirus 2
85708	GCA_004077615.1	Porcine circovirus 2
85708	GCA_004083455.1	Porcine circovirus 2
85708	GCA_004067475.1	Porcine circovirus 2

85708	GCA_004071575.1	Porcine circovirus 2
85708	GCA_004077275.1	Porcine circovirus 2
85708	GCA_004076935.1	Porcine circovirus 2
85708	GCA_004082615.1	Porcine circovirus 2
85708	GCA_004073635.1	Porcine circovirus 2
85708	GCA_004080455.1	Porcine circovirus 2
85708	GCA_004084615.1	Porcine circovirus 2
85708	GCA_004067495.1	Porcine circovirus 2
85708	GCA_004084075.1	Porcine circovirus 2
85708	GCA_004071595.1	Porcine circovirus 2
85708	GCA_004078955.1	Porcine circovirus 2
85708	GCA_004065455.1	Porcine circovirus 2
85708	GCA_004066015.1	Porcine circovirus 2
85708	GCA_004065675.1	Porcine circovirus 2
85708	GCA_004081615.1	Porcine circovirus 2
85708	GCA_004067515.1	Porcine circovirus 2
85708	GCA_004071615.1	Porcine circovirus 2
85708	GCA_004075575.1	Porcine circovirus 2
85708	GCA_004080115.1	Porcine circovirus 2
85708	GCA_004065475.1	Porcine circovirus 2
85708	GCA_004084275.1	Porcine circovirus 2
85708	GCA_004064995.1	Porcine circovirus 2
85708	GCA_004082775.1	Porcine circovirus 2
85708	GCA_004067535.1	Porcine circovirus 2
85708	GCA_004064655.1	Porcine circovirus 2
85708	GCA_004071635.1	Porcine circovirus 2
85708	GCA_004083895.1	Porcine circovirus 2
85708	GCA_004081275.1	Porcine circovirus 2
85708	GCA_004065495.1	Porcine circovirus 2
85708	GCA_004064315.1	Porcine circovirus 2
85708	GCA_004085275.1	Porcine circovirus 2
85708	GCA_004063975.1	Porcine circovirus 2
85708	GCA_004083935.1	Porcine circovirus 2
85708	GCA_004067555.1	Porcine circovirus 2
85708	GCA_004071655.1	Porcine circovirus 2
85708	GCA_004073875.1	Porcine circovirus 2
85708	GCA_004079015.1	Porcine circovirus 2
85708	GCA_004065515.1	Porcine circovirus 2
85708	GCA_004079915.1	Porcine circovirus 2
85708	GCA_004063295.1	Porcine circovirus 2
85708	GCA_004084375.1	Porcine circovirus 2
85708	GCA_004067575.1	Porcine circovirus 2
85708	GCA_004071675.1	Porcine circovirus 2
85708	GCA_004083595.1	Porcine circovirus 2
85708	GCA_004075775.1	Porcine circovirus 2
85708	GCA_004077975.1	Porcine circovirus 2
85708	GCA_004077635.1	Porcine circovirus 2
85708	GCA_004067595.1	Porcine circovirus 2
85708	GCA_004071695.1	Porcine circovirus 2
85708	GCA_004077295.1	Porcine circovirus 2

85708	GCA_004083475.1	Porcine circovirus 2
85708	GCA_004075795.1	Porcine circovirus 2
85708	GCA_004076955.1	Porcine circovirus 2
85708	GCA_004083255.1	Porcine circovirus 2
85708	GCA_004067615.1	Porcine circovirus 2
85708	GCA_004082875.1	Porcine circovirus 2
85708	GCA_004071715.1	Porcine circovirus 2
85708	GCA_004081755.1	Porcine circovirus 2
85708	GCA_004075815.1	Porcine circovirus 2
85708	GCA_004066035.1	Porcine circovirus 2
85708	GCA_004083235.1	Porcine circovirus 2
85708	GCA_004082575.1	Porcine circovirus 2
85708	GCA_004075935.1	Porcine circovirus 2
85708	GCA_004067635.1	Porcine circovirus 2
85708	GCA_004071735.1	Porcine circovirus 2
85708	GCA_004075595.1	Porcine circovirus 2
85708	GCA_004082915.1	Porcine circovirus 2
85708	GCA_004086735.1	Porcine circovirus 2
85708	GCA_004065595.1	Porcine circovirus 2
85708	GCA_004065015.1	Porcine circovirus 2
85708	GCA_004081415.1	Porcine circovirus 2
85708	GCA_004085575.1	Porcine circovirus 2
85708	GCA_004067655.1	Porcine circovirus 2
85708	GCA_004064675.1	Porcine circovirus 2
85708	GCA_004071755.1	Porcine circovirus 2
85708	GCA_004063575.1	Porcine circovirus 2
85708	GCA_004075855.1	Porcine circovirus 2
85708	GCA_004083855.1	Porcine circovirus 2
85708	GCA_004078415.1	Porcine circovirus 2
85708	GCA_004073815.1	Porcine circovirus 2
85708	GCA_004074235.1	Porcine circovirus 2
85708	GCA_004078355.1	Porcine circovirus 2
85708	GCA_004085535.1	Porcine circovirus 2
85708	GCA_004067675.1	Porcine circovirus 2
85708	GCA_004071775.1	Porcine circovirus 2
85708	GCA_004063675.1	Porcine circovirus 2
85708	GCA_004085235.1	Porcine circovirus 2
85708	GCA_004075875.1	Porcine circovirus 2
85708	GCA_004078235.1	Porcine circovirus 2
85708	GCA_004073555.1	Porcine circovirus 2
85708	GCA_004079575.1	Porcine circovirus 2
85708	GCA_004073835.1	Porcine circovirus 2
85708	GCA_004067695.1	Porcine circovirus 2
85708	GCA_004062975.1	Porcine circovirus 2
85708	GCA_004078115.1	Porcine circovirus 2
85708	GCA_004078075.1	Porcine circovirus 2
85708	GCA_004071795.1	Porcine circovirus 2
85708	GCA_004084635.1	Porcine circovirus 2
85708	GCA_004065655.1	Porcine circovirus 2
85708	GCA_004077995.1	Porcine circovirus 2

85708	GCA_004080735.1	Porcine circovirus 2
85708	GCA_004063615.1	Porcine circovirus 2
85708	GCA_004077655.1	Porcine circovirus 2
85708	GCA_004067715.1	Porcine circovirus 2
85708	GCA_004071815.1	Porcine circovirus 2
85708	GCA_004077315.1	Porcine circovirus 2
85708	GCA_004075915.1	Porcine circovirus 2
85708	GCA_004076975.1	Porcine circovirus 2
85708	GCA_004081895.1	Porcine circovirus 2
85708	GCA_004063645.1	Porcine circovirus 2
85708	GCA_004067735.1	Porcine circovirus 2
85708	GCA_004080395.1	Porcine circovirus 2
85708	GCA_004071835.1	Porcine circovirus 2
85708	GCA_004084555.1	Porcine circovirus 2
85708	GCA_004083135.1	Porcine circovirus 2
85708	GCA_004078895.1	Porcine circovirus 2
85708	GCA_004082455.1	Porcine circovirus 2
85708	GCA_004083055.1	Porcine circovirus 2
85708	GCA_004073895.1	Porcine circovirus 2
85708	GCA_004075955.1	Porcine circovirus 2
85708	GCA_004067755.1	Porcine circovirus 2
85708	GCA_004082835.1	Porcine circovirus 2
85708	GCA_004071855.1	Porcine circovirus 2
85708	GCA_004082335.1	Porcine circovirus 2
85708	GCA_004082535.1	Porcine circovirus 2
85708	GCA_004065035.1	Porcine circovirus 2
85708	GCA_004084215.1	Porcine circovirus 2
85708	GCA_004073915.1	Porcine circovirus 2
85708	GCA_004067775.1	Porcine circovirus 2
85708	GCA_004064695.1	Porcine circovirus 2
85708	GCA_004071875.1	Porcine circovirus 2
85708	GCA_004086875.1	Porcine circovirus 2
85708	GCA_004064355.1	Porcine circovirus 2
85708	GCA_004086395.1	Porcine circovirus 2
85708	GCA_004081215.1	Porcine circovirus 2
85708	GCA_004085375.1	Porcine circovirus 2
85708	GCA_004073935.1	Porcine circovirus 2
85708	GCA_004074255.1	Porcine circovirus 2
85708	GCA_004067795.1	Porcine circovirus 2
85708	GCA_004079715.1	Porcine circovirus 2
85708	GCA_004071895.1	Porcine circovirus 2
85708	GCA_004075995.1	Porcine circovirus 2
85708	GCA_004078215.1	Porcine circovirus 2
85708	GCA_004073575.1	Porcine circovirus 2
85708	GCA_004073955.1	Porcine circovirus 2
85708	GCA_004067815.1	Porcine circovirus 2
85708	GCA_004071915.1	Porcine circovirus 2
85708	GCA_004078015.1	Porcine circovirus 2
85708	GCA_004083535.1	Porcine circovirus 2
85708	GCA_004084895.1	Porcine circovirus 2

85708	GCA_004073975.1	Porcine circovirus 2
85708	GCA_004077675.1	Porcine circovirus 2
85708	GCA_004081855.1	Porcine circovirus 2
85708	GCA_004067835.1	Porcine circovirus 2
85708	GCA_004071935.1	Porcine circovirus 2
85708	GCA_004077335.1	Porcine circovirus 2
85708	GCA_004076995.1	Porcine circovirus 2
85708	GCA_004084695.1	Porcine circovirus 2
85708	GCA_004063775.1	Porcine circovirus 2
85708	GCA_004067855.1	Porcine circovirus 2
85708	GCA_004083995.1	Porcine circovirus 2
85708	GCA_004083195.1	Porcine circovirus 2
85708	GCA_004071955.1	Porcine circovirus 2
85708	GCA_004074015.1	Porcine circovirus 2
85708	GCA_004075975.1	Porcine circovirus 2
85708	GCA_004083395.1	Porcine circovirus 2
85708	GCA_004067875.1	Porcine circovirus 2
85708	GCA_004080195.1	Porcine circovirus 2
85708	GCA_004084355.1	Porcine circovirus 2
85708	GCA_004071975.1	Porcine circovirus 2
85708	GCA_004075635.1	Porcine circovirus 2
85708	GCA_004083095.1	Porcine circovirus 2
85708	GCA_004065055.1	Porcine circovirus 2
85708	GCA_004081555.1	Porcine circovirus 2
85708	GCA_004074035.1	Porcine circovirus 2
85708	GCA_004067895.1	Porcine circovirus 2
85708	GCA_004064715.1	Porcine circovirus 2
85708	GCA_004085515.1	Porcine circovirus 2
85708	GCA_004064375.1	Porcine circovirus 2
85708	GCA_004079855.1	Porcine circovirus 2
85708	GCA_004081315.1	Porcine circovirus 2
85708	GCA_004074055.1	Porcine circovirus 2
85708	GCA_004067915.1	Porcine circovirus 2
85708	GCA_004082515.1	Porcine circovirus 2
85708	GCA_004063695.1	Porcine circovirus 2
85708	GCA_004063835.1	Porcine circovirus 2
85708	GCA_004067935.1	Porcine circovirus 2
85708	GCA_004073255.1	Porcine circovirus 2
85708	GCA_004083675.1	Porcine circovirus 2
85708	GCA_004081295.1	Porcine circovirus 2
85708	GCA_004062675.1	Porcine circovirus 2
85708	GCA_004063855.1	Porcine circovirus 2
85708	GCA_004077695.1	Porcine circovirus 2
85708	GCA_004085775.1	Porcine circovirus 2
85708	GCA_004067955.1	Porcine circovirus 2
85708	GCA_004085155.1	Porcine circovirus 2
85708	GCA_004077355.1	Porcine circovirus 2
85708	GCA_004085655.1	Porcine circovirus 2
85708	GCA_004083335.1	Porcine circovirus 2
85708	GCA_004077015.1	Porcine circovirus 2

85708	GCA_004074115.1	Porcine circovirus 2
85708	GCA_004067975.1	Porcine circovirus 2
85708	GCA_004079795.1	Porcine circovirus 2
85708	GCA_004066095.1	Porcine circovirus 2
85708	GCA_004080335.1	Porcine circovirus 2
85708	GCA_004074135.1	Porcine circovirus 2
85708	GCA_004078835.1	Porcine circovirus 2
85708	GCA_004067995.1	Porcine circovirus 2
85708	GCA_004082995.1	Porcine circovirus 2
85708	GCA_004083375.1	Porcine circovirus 2
85708	GCA_004083655.1	Porcine circovirus 2
85708	GCA_004081495.1	Porcine circovirus 2
85708	GCA_004065075.1	Porcine circovirus 2
85708	GCA_004074155.1	Porcine circovirus 2
85708	GCA_004083355.1	Porcine circovirus 2
85708	GCA_004079995.1	Porcine circovirus 2
85708	GCA_004068015.1	Porcine circovirus 2
85708	GCA_004064735.1	Porcine circovirus 2
85708	GCA_004074635.1	Porcine circovirus 2
85708	GCA_004082655.1	Porcine circovirus 2
85708	GCA_004063935.1	Porcine circovirus 2
85708	GCA_004085175.1	Porcine circovirus 2
85708	GCA_004081155.1	Porcine circovirus 2
85708	GCA_004068035.1	Porcine circovirus 2
85708	GCA_004063715.1	Porcine circovirus 2
85708	GCA_004083815.1	Porcine circovirus 2
85708	GCA_004063955.1	Porcine circovirus 2
85708	GCA_004078155.1	Porcine circovirus 2
85708	GCA_004082315.1	Porcine circovirus 2
85708	GCA_004063035.1	Porcine circovirus 2
85708	GCA_004084935.1	Porcine circovirus 2
85708	GCA_004072935.1	Porcine circovirus 2
85708	GCA_004084975.1	Porcine circovirus 2
85708	GCA_004074215.1	Porcine circovirus 2
85708	GCA_004077715.1	Porcine circovirus 2
85708	GCA_004085555.1	Porcine circovirus 2
85708	GCA_004077375.1	Porcine circovirus 2
85708	GCA_004085715.1	Porcine circovirus 2
85708	GCA_004081975.1	Porcine circovirus 2
85708	GCA_004085415.1	Porcine circovirus 2
85708	GCA_004077035.1	Porcine circovirus 2
85708	GCA_004063995.1	Porcine circovirus 2
85708	GCA_004078975.1	Porcine circovirus 2
85708	GCA_004066115.1	Porcine circovirus 2
85708	GCA_004080055.1	Porcine circovirus 2
85708	GCA_004081635.1	Porcine circovirus 2
85708	GCA_004065435.1	Porcine circovirus 2
85708	GCA_004080135.1	Porcine circovirus 2
85708	GCA_004084295.1	Porcine circovirus 2
85708	GCA_004070175.1	Porcine circovirus 2

85708	GCA_004065095.1	Porcine circovirus 2
85708	GCA_004083915.1	Porcine circovirus 2
85708	GCA_004074275.1	Porcine circovirus 2
85708	GCA_004082795.1	Porcine circovirus 2
85708	GCA_004064755.1	Porcine circovirus 2
85708	GCA_004083615.1	Porcine circovirus 2
85708	GCA_004064415.1	Porcine circovirus 2
85708	GCA_004085455.1	Porcine circovirus 2
85708	GCA_004070195.1	Porcine circovirus 2
85708	GCA_004074295.1	Porcine circovirus 2
85708	GCA_004083955.1	Porcine circovirus 2
85708	GCA_004063745.1	Porcine circovirus 2
85708	GCA_004078295.1	Porcine circovirus 2
85708	GCA_004078255.1	Porcine circovirus 2
85708	GCA_004070215.1	Porcine circovirus 2
85708	GCA_004085115.1	Porcine circovirus 2
85708	GCA_004088655.1	Porcine circovirus 2
85708	GCA_004098895.1	Porcine circovirus 2
85708	GCA_004096855.1	Porcine circovirus 2
85708	GCA_004090715.1	Porcine circovirus 2
85708	GCA_004094815.1	Porcine circovirus 2
85708	GCA_004098215.1	Porcine circovirus 2
85708	GCA_004088675.1	Porcine circovirus 2
85708	GCA_004092775.1	Porcine circovirus 2
85708	GCA_004096875.1	Porcine circovirus 2
85708	GCA_004090735.1	Porcine circovirus 2
85708	GCA_004094835.1	Porcine circovirus 2
85708	GCA_004087435.1	Porcine circovirus 2
85708	GCA_004086955.1	Porcine circovirus 2
85708	GCA_004092795.1	Porcine circovirus 2
85708	GCA_004096895.1	Porcine circovirus 2
85708	GCA_004090755.1	Porcine circovirus 2
85708	GCA_004094855.1	Porcine circovirus 2
85708	GCA_004088715.1	Porcine circovirus 2
85708	GCA_004092815.1	Porcine circovirus 2
85708	GCA_004091055.1	Porcine circovirus 2
85708	GCA_004096915.1	Porcine circovirus 2
85708	GCA_004090775.1	Porcine circovirus 2
85708	GCA_004094875.1	Porcine circovirus 2
85708	GCA_004088735.1	Porcine circovirus 2
85708	GCA_004092835.1	Porcine circovirus 2
85708	GCA_004096935.1	Porcine circovirus 2
85708	GCA_004090795.1	Porcine circovirus 2
85708	GCA_004094895.1	Porcine circovirus 2
85708	GCA_004088755.1	Porcine circovirus 2
85708	GCA_004092855.1	Porcine circovirus 2
85708	GCA_004096955.1	Porcine circovirus 2
85708	GCA_004094915.1	Porcine circovirus 2
85708	GCA_004088775.1	Porcine circovirus 2
85708	GCA_004098915.1	Porcine circovirus 2

85708	GCA_004092875.1	Porcine circovirus 2
85708	GCA_004096975.1	Porcine circovirus 2
85708	GCA_004098575.1	Porcine circovirus 2
85708	GCA_004094935.1	Porcine circovirus 2
85708	GCA_004098235.1	Porcine circovirus 2
85708	GCA_004088795.1	Porcine circovirus 2
85708	GCA_004092895.1	Porcine circovirus 2
85708	GCA_004096995.1	Porcine circovirus 2
85708	GCA_004090855.1	Porcine circovirus 2
85708	GCA_004087315.1	Porcine circovirus 2
85708	GCA_004088815.1	Porcine circovirus 2
85708	GCA_004097215.1	Porcine circovirus 2
85708	GCA_004098555.1	Porcine circovirus 2
85708	GCA_004092915.1	Porcine circovirus 2
85708	GCA_004097015.1	Porcine circovirus 2
85708	GCA_004090875.1	Porcine circovirus 2
85708	GCA_004094975.1	Porcine circovirus 2
85708	GCA_004087235.1	Porcine circovirus 2
85708	GCA_004088835.1	Porcine circovirus 2
85708	GCA_004092935.1	Porcine circovirus 2
85708	GCA_004091075.1	Porcine circovirus 2
85708	GCA_004097035.1	Porcine circovirus 2
85708	GCA_004094995.1	Porcine circovirus 2
85708	GCA_004088855.1	Porcine circovirus 2
85708	GCA_004092955.1	Porcine circovirus 2
85708	GCA_004097055.1	Porcine circovirus 2
85708	GCA_004090915.1	Porcine circovirus 2
85708	GCA_004092975.1	Porcine circovirus 2
85708	GCA_004099645.1	Porcine circovirus 2
85708	GCA_004097075.1	Porcine circovirus 2
85708	GCA_004090935.1	Porcine circovirus 2
85708	GCA_004089035.1	Porcine circovirus 2
85708	GCA_004098935.1	Porcine circovirus 2
85708	GCA_004092995.1	Porcine circovirus 2
85708	GCA_004097095.1	Porcine circovirus 2
85708	GCA_004088355.1	Porcine circovirus 2
85708	GCA_004090955.1	Porcine circovirus 2
85708	GCA_004098255.1	Porcine circovirus 2
85708	GCA_004093015.1	Porcine circovirus 2
85708	GCA_004097115.1	Porcine circovirus 2
85708	GCA_004087335.1	Porcine circovirus 2
85708	GCA_004088935.1	Porcine circovirus 2
85708	GCA_004093035.1	Porcine circovirus 2
85708	GCA_004087375.1	Porcine circovirus 2
85708	GCA_004097135.1	Porcine circovirus 2
85708	GCA_004090995.1	Porcine circovirus 2
85708	GCA_004088955.1	Porcine circovirus 2
85708	GCA_004093055.1	Porcine circovirus 2
85708	GCA_004097155.1	Porcine circovirus 2
85708	GCA_004095115.1	Porcine circovirus 2

85708	GCA_004088975.1	Porcine circovirus 2
85708	GCA_004093075.1	Porcine circovirus 2
85708	GCA_004097175.1	Porcine circovirus 2
85708	GCA_004091035.1	Porcine circovirus 2
85708	GCA_004095135.1	Porcine circovirus 2
85708	GCA_004088995.1	Porcine circovirus 2
85708	GCA_004093095.1	Porcine circovirus 2
85708	GCA_004097195.1	Porcine circovirus 2
85708	GCA_004095155.1	Porcine circovirus 2
85708	GCA_004089015.1	Porcine circovirus 2
85708	GCA_004093115.1	Porcine circovirus 2
85708	GCA_004086975.1	Porcine circovirus 2
85708	GCA_004098615.1	Porcine circovirus 2
85708	GCA_004095175.1	Porcine circovirus 2
85708	GCA_004093135.1	Porcine circovirus 2
85708	GCA_004086995.1	Porcine circovirus 2
85708	GCA_004095195.1	Porcine circovirus 2
85708	GCA_004089055.1	Porcine circovirus 2
85708	GCA_004087015.1	Porcine circovirus 2
85708	GCA_004093155.1	Porcine circovirus 2
85708	GCA_004095215.1	Porcine circovirus 2
85708	GCA_004089075.1	Porcine circovirus 2
85708	GCA_004093175.1	Porcine circovirus 2
85708	GCA_004087035.1	Porcine circovirus 2
85708	GCA_004095235.1	Porcine circovirus 2
85708	GCA_004093195.1	Porcine circovirus 2
85708	GCA_004087055.1	Porcine circovirus 2
85708	GCA_004095255.1	Porcine circovirus 2
85708	GCA_004093215.1	Porcine circovirus 2
85708	GCA_004087075.1	Porcine circovirus 2
85708	GCA_004095275.1	Porcine circovirus 2
85708	GCA_004098975.1	Porcine circovirus 2
85708	GCA_004093235.1	Porcine circovirus 2
85708	GCA_004095295.1	Porcine circovirus 2
85708	GCA_004098295.1	Porcine circovirus 2
85708	GCA_004093255.1	Porcine circovirus 2
85708	GCA_004087715.1	Porcine circovirus 2
85708	GCA_004091215.1	Porcine circovirus 2
85708	GCA_004097615.1	Porcine circovirus 2
85708	GCA_004095315.1	Porcine circovirus 2
85708	GCA_004097275.1	Porcine circovirus 2
85708	GCA_004093275.1	Porcine circovirus 2
85708	GCA_004087135.1	Porcine circovirus 2
85708	GCA_004091235.1	Porcine circovirus 2
85708	GCA_004093295.1	Porcine circovirus 2
85708	GCA_004097395.1	Porcine circovirus 2
85708	GCA_004091255.1	Porcine circovirus 2
85708	GCA_004095355.1	Porcine circovirus 2
85708	GCA_004093315.1	Porcine circovirus 2
85708	GCA_004087175.1	Porcine circovirus 2

85708	GCA_004091275.1	Porcine circovirus 2
85708	GCA_004093335.1	Porcine circovirus 2
85708	GCA_004087195.1	Porcine circovirus 2
85708	GCA_004091295.1	Porcine circovirus 2
85708	GCA_004089095.1	Porcine circovirus 2
85708	GCA_004095395.1	Porcine circovirus 2
85708	GCA_004089255.1	Porcine circovirus 2
85708	GCA_004098995.1	Porcine circovirus 2
85708	GCA_004093355.1	Porcine circovirus 2
85708	GCA_004097455.1	Porcine circovirus 2
85708	GCA_004091315.1	Porcine circovirus 2
85708	GCA_004095415.1	Porcine circovirus 2
85708	GCA_004088095.1	Porcine circovirus 2
85708	GCA_004093375.1	Porcine circovirus 2
85708	GCA_004097975.1	Porcine circovirus 2
85708	GCA_004097475.1	Porcine circovirus 2
85708	GCA_004091335.1	Porcine circovirus 2
85708	GCA_004095435.1	Porcine circovirus 2
85708	GCA_004089295.1	Porcine circovirus 2
85708	GCA_004097295.1	Porcine circovirus 2
85708	GCA_004093395.1	Porcine circovirus 2
85708	GCA_004097495.1	Porcine circovirus 2
85708	GCA_004091355.1	Porcine circovirus 2
85708	GCA_004095455.1	Porcine circovirus 2
85708	GCA_004093415.1	Porcine circovirus 2
85708	GCA_004087575.1	Porcine circovirus 2
85708	GCA_004091375.1	Porcine circovirus 2
85708	GCA_004090815.1	Porcine circovirus 2
85708	GCA_004099605.1	Porcine circovirus 2
85708	GCA_004093435.1	Porcine circovirus 2
85708	GCA_004097535.1	Porcine circovirus 2
85708	GCA_004091395.1	Porcine circovirus 2
85708	GCA_004095495.1	Porcine circovirus 2
85708	GCA_004099625.1	Porcine circovirus 2
85708	GCA_004093455.1	Porcine circovirus 2
85708	GCA_004091415.1	Porcine circovirus 2
85708	GCA_004089115.1	Porcine circovirus 2
85708	GCA_004095515.1	Porcine circovirus 2
85708	GCA_004099015.1	Porcine circovirus 2
85708	GCA_004093475.1	Porcine circovirus 2
85708	GCA_004097575.1	Porcine circovirus 2
85708	GCA_004088435.1	Porcine circovirus 2
85708	GCA_004091435.1	Porcine circovirus 2
85708	GCA_004095535.1	Porcine circovirus 2
85708	GCA_004089395.1	Porcine circovirus 2
85708	GCA_004093495.1	Porcine circovirus 2
85708	GCA_004097595.1	Porcine circovirus 2
85708	GCA_004091455.1	Porcine circovirus 2
85708	GCA_004097655.1	Porcine circovirus 2
85708	GCA_004095555.1	Porcine circovirus 2

85708	GCA_004089415.1	Porcine circovirus 2
85708	GCA_004097315.1	Porcine circovirus 2
85708	GCA_004093515.1	Porcine circovirus 2
85708	GCA_004091475.1	Porcine circovirus 2
85708	GCA_004095575.1	Porcine circovirus 2
85708	GCA_004089435.1	Porcine circovirus 2
85708	GCA_004097635.1	Porcine circovirus 2
85708	GCA_004091495.1	Porcine circovirus 2
85708	GCA_004090835.1	Porcine circovirus 2
85708	GCA_004095595.1	Porcine circovirus 2
85708	GCA_004089455.1	Porcine circovirus 2
85708	GCA_004087415.1	Porcine circovirus 2
85708	GCA_004091515.1	Porcine circovirus 2
85708	GCA_004095615.1	Porcine circovirus 2
85708	GCA_004089475.1	Porcine circovirus 2
85708	GCA_004097675.1	Porcine circovirus 2
85708	GCA_004091535.1	Porcine circovirus 2
85708	GCA_004095635.1	Porcine circovirus 2
85708	GCA_004099035.1	Porcine circovirus 2
85708	GCA_004088455.1	Porcine circovirus 2
85708	GCA_004097555.1	Porcine circovirus 2
85708	GCA_004095655.1	Porcine circovirus 2
85708	GCA_004093615.1	Porcine circovirus 2
85708	GCA_004091575.1	Porcine circovirus 2
85708	GCA_004095675.1	Porcine circovirus 2
85708	GCA_004089535.1	Porcine circovirus 2
85708	GCA_004087095.1	Porcine circovirus 2
85708	GCA_004087395.1	Porcine circovirus 2
85708	GCA_004091595.1	Porcine circovirus 2
85708	GCA_004095695.1	Porcine circovirus 2
85708	GCA_004089555.1	Porcine circovirus 2
85708	GCA_004091615.1	Porcine circovirus 2
85708	GCA_004095715.1	Porcine circovirus 2
85708	GCA_004089575.1	Porcine circovirus 2
85708	GCA_004097775.1	Porcine circovirus 2
85708	GCA_004091635.1	Porcine circovirus 2
85708	GCA_004095735.1	Porcine circovirus 2
85708	GCA_004089595.1	Porcine circovirus 2
85708	GCA_004087495.1	Porcine circovirus 2
85708	GCA_004087555.1	Porcine circovirus 2
85708	GCA_004099195.1	Porcine circovirus 2
85708	GCA_004091655.1	Porcine circovirus 2
85708	GCA_004095755.1	Porcine circovirus 2
85708	GCA_004089615.1	Porcine circovirus 2
85708	GCA_004099055.1	Porcine circovirus 2
85708	GCA_004097815.1	Porcine circovirus 2
85708	GCA_004088475.1	Porcine circovirus 2
85708	GCA_004091675.1	Porcine circovirus 2
85708	GCA_004095775.1	Porcine circovirus 2
85708	GCA_004089635.1	Porcine circovirus 2

85708	GCA_004098035.1	Porcine circovirus 2
85708	GCA_004091695.1	Porcine circovirus 2
85708	GCA_004097695.1	Porcine circovirus 2
85708	GCA_004095795.1	Porcine circovirus 2
85708	GCA_004089655.1	Porcine circovirus 2
85708	GCA_004087115.1	Porcine circovirus 2
85708	GCA_004098515.1	Porcine circovirus 2
85708	GCA_004091715.1	Porcine circovirus 2
85708	GCA_004095815.1	Porcine circovirus 2
85708	GCA_004091555.1	Porcine circovirus 2
85708	GCA_004089675.1	Porcine circovirus 2
85708	GCA_004087635.1	Porcine circovirus 2
85708	GCA_004091735.1	Porcine circovirus 2
85708	GCA_004089695.1	Porcine circovirus 2
85708	GCA_004090535.1	Porcine circovirus 2
85708	GCA_004087655.1	Porcine circovirus 2
85708	GCA_004091755.1	Porcine circovirus 2
85708	GCA_004089715.1	Porcine circovirus 2
85708	GCA_004087675.1	Porcine circovirus 2
85708	GCA_004091775.1	Porcine circovirus 2
85708	GCA_004089735.1	Porcine circovirus 2
85708	GCA_004087695.1	Porcine circovirus 2
85708	GCA_004091795.1	Porcine circovirus 2
85708	GCA_004089755.1	Porcine circovirus 2
85708	GCA_004098055.1	Porcine circovirus 2
85708	GCA_004097955.1	Porcine circovirus 2
85708	GCA_004091815.1	Porcine circovirus 2
85708	GCA_004087475.1	Porcine circovirus 2
85708	GCA_004089775.1	Porcine circovirus 2
85708	GCA_004087755.1	Porcine circovirus 2
85708	GCA_004091835.1	Porcine circovirus 2
85708	GCA_004089795.1	Porcine circovirus 2
85708	GCA_004093895.1	Porcine circovirus 2
85708	GCA_004097995.1	Porcine circovirus 2
85708	GCA_004091855.1	Porcine circovirus 2
85708	GCA_004090895.1	Porcine circovirus 2
85708	GCA_004095955.1	Porcine circovirus 2
85708	GCA_004087775.1	Porcine circovirus 2
85708	GCA_004089815.1	Porcine circovirus 2
85708	GCA_004093915.1	Porcine circovirus 2
85708	GCA_004098015.1	Porcine circovirus 2
85708	GCA_004095975.1	Porcine circovirus 2
85708	GCA_004089835.1	Porcine circovirus 2
85708	GCA_004093935.1	Porcine circovirus 2
85708	GCA_004135265.1	Porcine circovirus 2
85708	GCA_004087795.1	Porcine circovirus 2
85708	GCA_004091895.1	Porcine circovirus 2
85708	GCA_004095995.1	Porcine circovirus 2
85708	GCA_004089855.1	Porcine circovirus 2
85708	GCA_004099095.1	Porcine circovirus 2

85708	GCA_004093955.1	Porcine circovirus 2
85708	GCA_004087815.1	Porcine circovirus 2
85708	GCA_004091915.1	Porcine circovirus 2
85708	GCA_004096015.1	Porcine circovirus 2
85708	GCA_004089875.1	Porcine circovirus 2
85708	GCA_004093975.1	Porcine circovirus 2
85708	GCA_004087845.1	Porcine circovirus 2
85708	GCA_004098075.1	Porcine circovirus 2
85708	GCA_004091935.1	Porcine circovirus 2
85708	GCA_004097735.1	Porcine circovirus 2
85708	GCA_004096035.1	Porcine circovirus 2
85708	GCA_004089915.1	Porcine circovirus 2
85708	GCA_004087155.1	Porcine circovirus 2
85708	GCA_004098095.1	Porcine circovirus 2
85708	GCA_004091955.1	Porcine circovirus 2
85708	GCA_004087875.1	Porcine circovirus 2
85708	GCA_004094015.1	Porcine circovirus 2
85708	GCA_004098115.1	Porcine circovirus 2
85708	GCA_004091975.1	Porcine circovirus 2
85708	GCA_004096075.1	Porcine circovirus 2
85708	GCA_004089935.1	Porcine circovirus 2
85708	GCA_004090575.1	Porcine circovirus 2
85708	GCA_004094035.1	Porcine circovirus 2
85708	GCA_004087895.1	Porcine circovirus 2
85708	GCA_004088305.1	Porcine circovirus 2
85708	GCA_004091995.1	Porcine circovirus 2
85708	GCA_004096095.1	Porcine circovirus 2
85708	GCA_004089955.1	Porcine circovirus 2
85708	GCA_004094055.1	Porcine circovirus 2
85708	GCA_004087935.1	Porcine circovirus 2
85708	GCA_004092015.1	Porcine circovirus 2
85708	GCA_004096115.1	Porcine circovirus 2
85708	GCA_004089975.1	Porcine circovirus 2
85708	GCA_004088875.1	Porcine circovirus 2
85708	GCA_004098175.1	Porcine circovirus 2
85708	GCA_004092035.1	Porcine circovirus 2
85708	GCA_004096135.1	Porcine circovirus 2
85708	GCA_004098435.1	Porcine circovirus 2
85708	GCA_004089995.1	Porcine circovirus 2
85708	GCA_004094095.1	Porcine circovirus 2
85708	GCA_004087955.1	Porcine circovirus 2
85708	GCA_004092055.1	Porcine circovirus 2
85708	GCA_004097755.1	Porcine circovirus 2
85708	GCA_004096155.1	Porcine circovirus 2
85708	GCA_004090015.1	Porcine circovirus 2
85708	GCA_004097415.1	Porcine circovirus 2
85708	GCA_004094115.1	Porcine circovirus 2
85708	GCA_004087975.1	Porcine circovirus 2
85708	GCA_004092075.1	Porcine circovirus 2
85708	GCA_004096175.1	Porcine circovirus 2

85708	GCA_004090035.1	Porcine circovirus 2
85708	GCA_004094135.1	Porcine circovirus 2
85708	GCA_004088005.1	Porcine circovirus 2
85708	GCA_004092095.1	Porcine circovirus 2
85708	GCA_004096195.1	Porcine circovirus 2
85708	GCA_004090055.1	Porcine circovirus 2
85708	GCA_004094155.1	Porcine circovirus 2
85708	GCA_004088035.1	Porcine circovirus 2
85708	GCA_004092115.1	Porcine circovirus 2
85708	GCA_004096215.1	Porcine circovirus 2
85708	GCA_004090075.1	Porcine circovirus 2
85708	GCA_004094175.1	Porcine circovirus 2
85708	GCA_004098275.1	Porcine circovirus 2
85708	GCA_004092135.1	Porcine circovirus 2
85708	GCA_004096235.1	Porcine circovirus 2
85708	GCA_004090095.1	Porcine circovirus 2
85708	GCA_004094195.1	Porcine circovirus 2
85708	GCA_004088055.1	Porcine circovirus 2
85708	GCA_004092155.1	Porcine circovirus 2
85708	GCA_004096255.1	Porcine circovirus 2
85708	GCA_004090115.1	Porcine circovirus 2
85708	GCA_004094215.1	Porcine circovirus 2
85708	GCA_004098315.1	Porcine circovirus 2
85708	GCA_004092175.1	Porcine circovirus 2
85708	GCA_004087535.1	Porcine circovirus 2
85708	GCA_004096275.1	Porcine circovirus 2
85708	GCA_004090135.1	Porcine circovirus 2
85708	GCA_004097435.1	Porcine circovirus 2
85708	GCA_004094235.1	Porcine circovirus 2
85708	GCA_004098335.1	Porcine circovirus 2
85708	GCA_004092195.1	Porcine circovirus 2
85708	GCA_004090155.1	Porcine circovirus 2
85708	GCA_004094255.1	Porcine circovirus 2
85708	GCA_004092215.1	Porcine circovirus 2
85708	GCA_004096315.1	Porcine circovirus 2
85708	GCA_004090175.1	Porcine circovirus 2
85708	GCA_004090615.1	Porcine circovirus 2
85708	GCA_004094275.1	Porcine circovirus 2
85708	GCA_004143505.1	Porcine circovirus 2
85708	GCA_004092235.1	Porcine circovirus 2
85708	GCA_004096335.1	Porcine circovirus 2
85708	GCA_004090195.1	Porcine circovirus 2
85708	GCA_004094295.1	Porcine circovirus 2
85708	GCA_004098395.1	Porcine circovirus 2
85708	GCA_004092255.1	Porcine circovirus 2
85708	GCA_004096355.1	Porcine circovirus 2
85708	GCA_004090215.1	Porcine circovirus 2
85708	GCA_004097715.1	Porcine circovirus 2
85708	GCA_004087455.1	Porcine circovirus 2
85708	GCA_004094315.1	Porcine circovirus 2

85708	GCA_004098815.1	Porcine circovirus 2
85708	GCA_004092275.1	Porcine circovirus 2
85708	GCA_004096375.1	Porcine circovirus 2
85708	GCA_004088255.1	Porcine circovirus 2
85708	GCA_004090235.1	Porcine circovirus 2
85708	GCA_004094335.1	Porcine circovirus 2
85708	GCA_004098135.1	Porcine circovirus 2
85708	GCA_004092295.1	Porcine circovirus 2
85708	GCA_004097795.1	Porcine circovirus 2
85708	GCA_004096395.1	Porcine circovirus 2
85708	GCA_004090255.1	Porcine circovirus 2
85708	GCA_004087215.1	Porcine circovirus 2
85708	GCA_004094355.1	Porcine circovirus 2
85708	GCA_004098455.1	Porcine circovirus 2
85708	GCA_004092315.1	Porcine circovirus 2
85708	GCA_004096415.1	Porcine circovirus 2
85708	GCA_004090275.1	Porcine circovirus 2
85708	GCA_004094375.1	Porcine circovirus 2
85708	GCA_004098475.1	Porcine circovirus 2
85708	GCA_004092335.1	Porcine circovirus 2
85708	GCA_004090975.1	Porcine circovirus 2
85708	GCA_004096435.1	Porcine circovirus 2
85708	GCA_004090295.1	Porcine circovirus 2
85708	GCA_004090635.1	Porcine circovirus 2
85708	GCA_004094395.1	Porcine circovirus 2
85708	GCA_004092355.1	Porcine circovirus 2
85708	GCA_004096455.1	Porcine circovirus 2
85708	GCA_004090315.1	Porcine circovirus 2
85708	GCA_004088275.1	Porcine circovirus 2
85708	GCA_004092375.1	Porcine circovirus 2
85708	GCA_004089275.1	Porcine circovirus 2
85708	GCA_004096475.1	Porcine circovirus 2
85708	GCA_004090335.1	Porcine circovirus 2
85708	GCA_004099175.1	Porcine circovirus 2
85708	GCA_004094435.1	Porcine circovirus 2
85708	GCA_004092395.1	Porcine circovirus 2
85708	GCA_004096495.1	Porcine circovirus 2
85708	GCA_004097515.1	Porcine circovirus 2
85708	GCA_004090355.1	Porcine circovirus 2
85708	GCA_004094455.1	Porcine circovirus 2
85708	GCA_004098155.1	Porcine circovirus 2
85708	GCA_004088335.1	Porcine circovirus 2
85708	GCA_004096515.1	Porcine circovirus 2
85708	GCA_004090375.1	Porcine circovirus 2
85708	GCA_004094475.1	Porcine circovirus 2
85708	GCA_004087355.1	Porcine circovirus 2
85708	GCA_004090395.1	Porcine circovirus 2
85708	GCA_004094495.1	Porcine circovirus 2
85708	GCA_004098595.1	Porcine circovirus 2
85708	GCA_004090415.1	Porcine circovirus 2

85708	GCA_004090655.1	Porcine circovirus 2
85708	GCA_004094515.1	Porcine circovirus 2
85708	GCA_004088375.1	Porcine circovirus 2
85708	GCA_004092475.1	Porcine circovirus 2
85708	GCA_004090435.1	Porcine circovirus 2
85708	GCA_004094535.1	Porcine circovirus 2
85708	GCA_004088415.1	Porcine circovirus 2
85708	GCA_004092495.1	Porcine circovirus 2
85708	GCA_004094415.1	Porcine circovirus 2
85708	GCA_004090455.1	Porcine circovirus 2
85708	GCA_004094075.1	Porcine circovirus 2
85708	GCA_004094555.1	Porcine circovirus 2
85708	GCA_004098855.1	Porcine circovirus 2
85708	GCA_004092515.1	Porcine circovirus 2
85708	GCA_004090475.1	Porcine circovirus 2
85708	GCA_004094575.1	Porcine circovirus 2
85708	GCA_004092535.1	Porcine circovirus 2
85708	GCA_004087595.1	Porcine circovirus 2
85708	GCA_004096635.1	Porcine circovirus 2
85708	GCA_004090495.1	Porcine circovirus 2
85708	GCA_004087255.1	Porcine circovirus 2
85708	GCA_004094595.1	Porcine circovirus 2
85708	GCA_004092555.1	Porcine circovirus 2
85708	GCA_004096655.1	Porcine circovirus 2
85708	GCA_004090515.1	Porcine circovirus 2
85708	GCA_004094615.1	Porcine circovirus 2
85708	GCA_004091015.1	Porcine circovirus 2
85708	GCA_004096675.1	Porcine circovirus 2
85708	GCA_004090675.1	Porcine circovirus 2
85708	GCA_004094635.1	Porcine circovirus 2
85708	GCA_004088495.1	Porcine circovirus 2
85708	GCA_004096695.1	Porcine circovirus 2
85708	GCA_004090555.1	Porcine circovirus 2
85708	GCA_004094655.1	Porcine circovirus 2
85708	GCA_004096715.1	Porcine circovirus 2
85708	GCA_004099215.1	Porcine circovirus 2
85708	GCA_004094675.1	Porcine circovirus 2
85708	GCA_004088635.1	Porcine circovirus 2
85708	GCA_004135305.1	Porcine circovirus 2
85708	GCA_004096735.1	Porcine circovirus 2
85708	GCA_004098535.1	Porcine circovirus 2
85708	GCA_004090595.1	Porcine circovirus 2
85708	GCA_004094695.1	Porcine circovirus 2
85708	GCA_004098195.1	Porcine circovirus 2
85708	GCA_004098795.1	Porcine circovirus 2
85708	GCA_004087615.1	Porcine circovirus 2
85708	GCA_004096755.1	Porcine circovirus 2
85708	GCA_004087275.1	Porcine circovirus 2
85708	GCA_004094715.1	Porcine circovirus 2
85708	GCA_004086935.1	Porcine circovirus 2

85708	GCA_004096775.1	Porcine circovirus 2
85708	GCA_004087295.1	Porcine circovirus 2
85708	GCA_004094735.1	Porcine circovirus 2
85708	GCA_004098835.1	Porcine circovirus 2
85708	GCA_004096795.1	Porcine circovirus 2
85708	GCA_004090695.1	Porcine circovirus 2
85708	GCA_004094755.1	Porcine circovirus 2
85708	GCA_004096815.1	Porcine circovirus 2
85708	GCA_004094775.1	Porcine circovirus 2
85708	GCA_004098875.1	Porcine circovirus 2
85708	GCA_004096835.1	Porcine circovirus 2
85708	GCA_004099235.1	Porcine circovirus 2
85708	GCA_004094795.1	Porcine circovirus 2
85708	GCA_004326635.1	Porcine circovirus 2
85708	GCA_004326455.1	Porcine circovirus 2
85708	GCA_004325375.1	Porcine circovirus 2
85708	GCA_004326515.1	Porcine circovirus 2
85708	GCA_004326395.1	Porcine circovirus 2
85708	GCA_004327435.1	Porcine circovirus 2
85708	GCA_004325795.1	Porcine circovirus 2
85708	GCA_004325455.1	Porcine circovirus 2
85708	GCA_004325995.1	Porcine circovirus 2
85708	GCA_004326595.1	Porcine circovirus 2
85708	GCA_004325935.1	Porcine circovirus 2
85708	GCA_004327415.1	Porcine circovirus 2
85708	GCA_004325595.1	Porcine circovirus 2
85708	GCA_004326795.1	Porcine circovirus 2
85708	GCA_004326495.1	Porcine circovirus 2
85708	GCA_004325915.1	Porcine circovirus 2
85708	GCA_004325735.1	Porcine circovirus 2
85708	GCA_004326575.1	Porcine circovirus 2
85708	GCA_004326875.1	Porcine circovirus 2
85708	GCA_004326755.1	Porcine circovirus 2
85708	GCA_004326535.1	Porcine circovirus 2
85708	GCA_004325435.1	Porcine circovirus 2
85708	GCA_004325875.1	Porcine circovirus 2
85708	GCA_004326215.1	Porcine circovirus 2
85708	GCA_004325535.1	Porcine circovirus 2
85708	GCA_004325195.1	Porcine circovirus 2
85708	GCA_004326335.1	Porcine circovirus 2
85708	GCA_004327495.1	Porcine circovirus 2
85708	GCA_004326915.1	Porcine circovirus 2
85708	GCA_004325675.1	Porcine circovirus 2
85708	GCA_004326855.1	Porcine circovirus 2
85708	GCA_004326815.1	Porcine circovirus 2
85708	GCA_004326475.1	Porcine circovirus 2
85708	GCA_004326375.1	Porcine circovirus 2
85708	GCA_004326095.1	Porcine circovirus 2
85708	GCA_004325815.1	Porcine circovirus 2
85708	GCA_004326955.1	Porcine circovirus 2

85708	GCA_004325475.1	Porcine circovirus 2
85708	GCA_004325755.1	Porcine circovirus 2
85708	GCA_004325495.1	Porcine circovirus 2
85708	GCA_004326275.1	Porcine circovirus 2
85708	GCA_004326175.1	Porcine circovirus 2
85708	GCA_004326295.1	Porcine circovirus 2
85708	GCA_004326935.1	Porcine circovirus 2
85708	GCA_004326195.1	Porcine circovirus 2
85708	GCA_004325635.1	Porcine circovirus 2
85708	GCA_004326055.1	Porcine circovirus 2
85708	GCA_004327095.1	Porcine circovirus 2
85708	GCA_004325155.1	Porcine circovirus 2
85708	GCA_004325835.1	Porcine circovirus 2
85708	GCA_004325415.1	Porcine circovirus 2
85708	GCA_004326895.1	Porcine circovirus 2
85708	GCA_004326015.1	Porcine circovirus 2
85708	GCA_004325715.1	Porcine circovirus 2
85708	GCA_004325395.1	Porcine circovirus 2
85708	GCA_004326555.1	Porcine circovirus 2
85708	GCA_004326255.1	Porcine circovirus 2
85708	GCA_004327135.1	Porcine circovirus 2
85708	GCA_004166315.1	Porcine circovirus 2
85708	GCA_004326035.1	Porcine circovirus 2
85708	GCA_004326235.1	Porcine circovirus 2
85708	GCA_004325695.1	Porcine circovirus 2
85708	GCA_004326835.1	Porcine circovirus 2
85708	GCA_004327395.1	Porcine circovirus 2
85708	GCA_004326975.1	Porcine circovirus 2
85708	GCA_004326735.1	Porcine circovirus 2
85708	GCA_004327075.1	Porcine circovirus 2
85708	GCA_004327035.1	Porcine circovirus 2
85708	GCA_004325895.1	Porcine circovirus 2
85708	GCA_004327455.1	Porcine circovirus 2
85708	GCA_004325975.1	Porcine circovirus 2
85708	GCA_004326675.1	Porcine circovirus 2
85708	GCA_004327115.1	Porcine circovirus 2
85708	GCA_004326435.1	Porcine circovirus 2
85708	GCA_004326315.1	Porcine circovirus 2
85708	GCA_004326775.1	Porcine circovirus 2
85708	GCA_004326655.1	Porcine circovirus 2
85708	GCA_004326155.1	Porcine circovirus 2
85708	GCA_004295425.1	Porcine circovirus 2
85708	GCA_004325775.1	Porcine circovirus 2
85708	GCA_004327015.1	Porcine circovirus 2
85708	GCA_004325555.1	Porcine circovirus 2
85708	GCA_004326415.1	Porcine circovirus 2
85708	GCA_004326995.1	Porcine circovirus 2
85708	GCA_004325615.1	Porcine circovirus 2
85708	GCA_004326115.1	Porcine circovirus 2
85708	GCA_004295445.1	Porcine circovirus 2

85708	GCA_004327055.1	Porcine circovirus 2
85708	GCA_004325575.1	Porcine circovirus 2
85708	GCA_004326715.1	Porcine circovirus 2
85708	GCA_004326355.1	Porcine circovirus 2
85708	GCA_004326075.1	Porcine circovirus 2
85708	GCA_004295465.1	Porcine circovirus 2
85708	GCA_004194725.1	Porcine circovirus 2
85708	GCA_004326615.1	Porcine circovirus 2
85708	GCA_004194745.1	Porcine circovirus 2
85708	GCA_004325855.1	Porcine circovirus 2
85708	GCA_004194765.1	Porcine circovirus 2
85708	GCA_004325955.1	Porcine circovirus 2
85708	GCA_004325515.1	Porcine circovirus 2
85708	GCA_004338155.1	Porcine circovirus 2
85708	GCA_004194785.1	Porcine circovirus 2
85708	GCA_004325135.1	Porcine circovirus 2
85708	GCA_004338175.1	Porcine circovirus 2
85708	GCA_004194805.1	Porcine circovirus 2
85708	GCA_004325175.1	Porcine circovirus 2
85708	GCA_004338195.1	Porcine circovirus 2
85708	GCA_004194825.1	Porcine circovirus 2
85708	GCA_004194845.1	Porcine circovirus 2
85708	GCA_004327475.1	Porcine circovirus 2
85708	GCA_004194865.1	Porcine circovirus 2
85708	GCA_004338255.1	Porcine circovirus 2
85708	GCA_004194885.1	Porcine circovirus 2
85708	GCA_004325655.1	Porcine circovirus 2
85708	GCA_004326695.1	Porcine circovirus 2
479166	GCA_004091175.1	Porcine circovirus 2 P0404c/03
479167	GCA_004091095.1	Porcine circovirus 2 R0255/209/03
479168	GCA_004091115.1	Porcine circovirus 2 S0255/210/03
348291	GCA_004099155.1	Porcine circovirus 2 strain SD3
348292	GCA_004099135.1	Porcine circovirus 2 strain SD5
348293	GCA_004099115.1	Porcine circovirus 2 strain SD6
479169	GCA_004091155.1	Porcine circovirus 2 T0181c/03
479170	GCA_004091135.1	Porcine circovirus 2 U0168/03
479171	GCA_004091195.1	Porcine circovirus 2 Z0161b/03
1868221	GCA_001866935.1	Porcine circovirus 3
1868221	GCA_004030715.1	Porcine circovirus 3
1868221	GCA_004030995.1	Porcine circovirus 3
1868221	GCA_004029435.1	Porcine circovirus 3
1868221	GCA_004030675.1	Porcine circovirus 3
1868221	GCA_004029455.1	Porcine circovirus 3
1868221	GCA_004031335.1	Porcine circovirus 3
1868221	GCA_004029475.1	Porcine circovirus 3
1868221	GCA_004036635.1	Porcine circovirus 3
1868221	GCA_004029495.1	Porcine circovirus 3
1868221	GCA_004029515.1	Porcine circovirus 3
1868221	GCA_004029535.1	Porcine circovirus 3
1868221	GCA_004036535.1	Porcine circovirus 3

1868221	GCA_004029555.1	Porcine circovirus 3
1868221	GCA_004031195.1	Porcine circovirus 3
1868221	GCA_004030355.1	Porcine circovirus 3
1868221	GCA_004029575.1	Porcine circovirus 3
1868221	GCA_004029595.1	Porcine circovirus 3
1868221	GCA_004029615.1	Porcine circovirus 3
1868221	GCA_004029635.1	Porcine circovirus 3
1868221	GCA_004029655.1	Porcine circovirus 3
1868221	GCA_004029675.1	Porcine circovirus 3
1868221	GCA_004030375.1	Porcine circovirus 3
1868221	GCA_004029695.1	Porcine circovirus 3
1868221	GCA_004029715.1	Porcine circovirus 3
1868221	GCA_004029355.1	Porcine circovirus 3
1868221	GCA_004029735.1	Porcine circovirus 3
1868221	GCA_004029755.1	Porcine circovirus 3
1868221	GCA_004036275.1	Porcine circovirus 3
1868221	GCA_004029775.1	Porcine circovirus 3
1868221	GCA_004029795.1	Porcine circovirus 3
1868221	GCA_004029815.1	Porcine circovirus 3
1868221	GCA_004029835.1	Porcine circovirus 3
1868221	GCA_004029375.1	Porcine circovirus 3
1868221	GCA_004029855.1	Porcine circovirus 3
1868221	GCA_004029875.1	Porcine circovirus 3
1868221	GCA_004030415.1	Porcine circovirus 3
1868221	GCA_004031555.1	Porcine circovirus 3
1868221	GCA_004036715.1	Porcine circovirus 3
1868221	GCA_004029395.1	Porcine circovirus 3
1868221	GCA_004031215.1	Porcine circovirus 3
1868221	GCA_004037775.1	Porcine circovirus 3
1868221	GCA_004030875.1	Porcine circovirus 3
1868221	GCA_004030015.1	Porcine circovirus 3
1868221	GCA_004030035.1	Porcine circovirus 3
1868221	GCA_004030775.1	Porcine circovirus 3
1868221	GCA_004030055.1	Porcine circovirus 3
1868221	GCA_004031355.1	Porcine circovirus 3
1868221	GCA_004030075.1	Porcine circovirus 3
1868221	GCA_004029415.1	Porcine circovirus 3
1868221	GCA_004030095.1	Porcine circovirus 3
1868221	GCA_004036655.1	Porcine circovirus 3
1868221	GCA_004030115.1	Porcine circovirus 3
1868221	GCA_004036315.1	Porcine circovirus 3
1868221	GCA_004036755.1	Porcine circovirus 3
1868221	GCA_004030135.1	Porcine circovirus 3
1868221	GCA_004036575.1	Porcine circovirus 3
1868221	GCA_004031475.1	Porcine circovirus 3
1868221	GCA_004030155.1	Porcine circovirus 3
1868221	GCA_004031135.1	Porcine circovirus 3
1868221	GCA_004030455.1	Porcine circovirus 3
1868221	GCA_004030175.1	Porcine circovirus 3
1868221	GCA_004031495.1	Porcine circovirus 3

1868221	GCA_004031295.1	Porcine circovirus 3
1868221	GCA_004030275.1	Porcine circovirus 3
1868221	GCA_004031155.1	Porcine circovirus 3
1868221	GCA_004030295.1	Porcine circovirus 3
1868221	GCA_004030315.1	Porcine circovirus 3
1868221	GCA_004030335.1	Porcine circovirus 3
1868221	GCA_004036595.1	Porcine circovirus 3
1868221	GCA_004031515.1	Porcine circovirus 3
1868221	GCA_004030395.1	Porcine circovirus 3
1868221	GCA_004031175.1	Porcine circovirus 3
1868221	GCA_004030835.1	Porcine circovirus 3
1868221	GCA_004030435.1	Porcine circovirus 3
1868221	GCA_004036735.1	Porcine circovirus 3
1868221	GCA_004036555.1	Porcine circovirus 3
1868221	GCA_004031535.1	Porcine circovirus 3
1868221	GCA_004030915.1	Porcine circovirus 3
1868221	GCA_004031575.1	Porcine circovirus 3
1868221	GCA_004031235.1	Porcine circovirus 3
1868221	GCA_004030575.1	Porcine circovirus 3
1868221	GCA_004030595.1	Porcine circovirus 3
1868221	GCA_004035775.1	Porcine circovirus 3
1868221	GCA_004030635.1	Porcine circovirus 3
1868221	GCA_004030655.1	Porcine circovirus 3
1868221	GCA_004036675.1	Porcine circovirus 3
1868221	GCA_004030735.1	Porcine circovirus 3
1868221	GCA_004030895.1	Porcine circovirus 3
1868221	GCA_004030555.1	Porcine circovirus 3
1868221	GCA_004036775.1	Porcine circovirus 3
1868221	GCA_004030795.1	Porcine circovirus 3
1868221	GCA_004030815.1	Porcine circovirus 3
1868221	GCA_004031395.1	Porcine circovirus 3
1868221	GCA_004036615.1	Porcine circovirus 3
1868221	GCA_004030935.1	Porcine circovirus 3
1868221	GCA_004031015.1	Porcine circovirus 3
1868221	GCA_004031455.1	Porcine circovirus 3
1868221	GCA_004036295.1	Porcine circovirus 3
1868221	GCA_004031035.1	Porcine circovirus 3
1868221	GCA_004036695.1	Porcine circovirus 3
1868221	GCA_004031055.1	Porcine circovirus 3
1868221	GCA_004031075.1	Porcine circovirus 3
1868221	GCA_004031095.1	Porcine circovirus 3
1868221	GCA_004031115.1	Porcine circovirus 3
1868221	GCA_004030955.1	Porcine circovirus 3
1868221	GCA_004030615.1	Porcine circovirus 3
1868221	GCA_004031255.1	Porcine circovirus 3
1868221	GCA_004030975.1	Porcine circovirus 3
1868221	GCA_004031275.1	Porcine circovirus 3
1868221	GCA_004041555.1	Porcine circovirus 3
1868221	GCA_004041615.1	Porcine circovirus 3
1868221	GCA_004049815.1	Porcine circovirus 3

1868221	GCA_004049755.1	Porcine circovirus 3
1868221	GCA_004053475.1	Porcine circovirus 3
1868221	GCA_004041635.1	Porcine circovirus 3
1868221	GCA_004048735.1	Porcine circovirus 3
1868221	GCA_004041655.1	Porcine circovirus 3
1868221	GCA_004041675.1	Porcine circovirus 3
1868221	GCA_004041695.1	Porcine circovirus 3
1868221	GCA_004049895.1	Porcine circovirus 3
1868221	GCA_004041715.1	Porcine circovirus 3
1868221	GCA_004040215.1	Porcine circovirus 3
1868221	GCA_004041735.1	Porcine circovirus 3
1868221	GCA_004049775.1	Porcine circovirus 3
1868221	GCA_004043815.1	Porcine circovirus 3
1868221	GCA_004048755.1	Porcine circovirus 3
1868221	GCA_004043835.1	Porcine circovirus 3
1868221	GCA_004043855.1	Porcine circovirus 3
1868221	GCA_004051835.1	Porcine circovirus 3
1868221	GCA_004039775.1	Porcine circovirus 3
1868221	GCA_004043875.1	Porcine circovirus 3
1868221	GCA_004050815.1	Porcine circovirus 3
1868221	GCA_004043895.1	Porcine circovirus 3
1868221	GCA_004040235.1	Porcine circovirus 3
1868221	GCA_004039815.1	Porcine circovirus 3
1868221	GCA_004049795.1	Porcine circovirus 3
1868221	GCA_004043915.1	Porcine circovirus 3
1868221	GCA_004043935.1	Porcine circovirus 3
1868221	GCA_004051855.1	Porcine circovirus 3
1868221	GCA_004041275.1	Porcine circovirus 3
1868221	GCA_004041955.1	Porcine circovirus 3
1868221	GCA_004050835.1	Porcine circovirus 3
1868221	GCA_004040255.1	Porcine circovirus 3
1868221	GCA_004041975.1	Porcine circovirus 3
1868221	GCA_004041995.1	Porcine circovirus 3
1868221	GCA_004042015.1	Porcine circovirus 3
1868221	GCA_004048175.1	Porcine circovirus 3
1868221	GCA_004042035.1	Porcine circovirus 3
1868221	GCA_004042055.1	Porcine circovirus 3
1868221	GCA_004040015.1	Porcine circovirus 3
1868221	GCA_004042075.1	Porcine circovirus 3
1868221	GCA_004050855.1	Porcine circovirus 3
1868221	GCA_004040275.1	Porcine circovirus 3
1868221	GCA_004042095.1	Porcine circovirus 3
1868221	GCA_004040055.1	Porcine circovirus 3
1868221	GCA_004049835.1	Porcine circovirus 3
1868221	GCA_004042115.1	Porcine circovirus 3
1868221	GCA_004042135.1	Porcine circovirus 3
1868221	GCA_004042155.1	Porcine circovirus 3
1868221	GCA_004046255.1	Porcine circovirus 3
1868221	GCA_004040115.1	Porcine circovirus 3
1868221	GCA_004042175.1	Porcine circovirus 3

1868221	GCA_004046275.1	Porcine circovirus 3
1868221	GCA_004040135.1	Porcine circovirus 3
1868221	GCA_004046295.1	Porcine circovirus 3
1868221	GCA_004040155.1	Porcine circovirus 3
1868221	GCA_004042215.1	Porcine circovirus 3
1868221	GCA_004046315.1	Porcine circovirus 3
1868221	GCA_004049855.1	Porcine circovirus 3
1868221	GCA_004042235.1	Porcine circovirus 3
1868221	GCA_004040195.1	Porcine circovirus 3
1868221	GCA_004042255.1	Porcine circovirus 3
1868221	GCA_004053275.1	Porcine circovirus 3
1868221	GCA_004042275.1	Porcine circovirus 3
1868221	GCA_004042295.1	Porcine circovirus 3
1868221	GCA_004042315.1	Porcine circovirus 3
1868221	GCA_004050895.1	Porcine circovirus 3
1868221	GCA_004040315.1	Porcine circovirus 3
1868221	GCA_004040295.1	Porcine circovirus 3
1868221	GCA_004049875.1	Porcine circovirus 3
1868221	GCA_004053295.1	Porcine circovirus 3
1868221	GCA_004040335.1	Porcine circovirus 3
1868221	GCA_004038635.1	Porcine circovirus 3
1868221	GCA_004050935.1	Porcine circovirus 3
1868221	GCA_004046695.1	Porcine circovirus 3
1868221	GCA_004046715.1	Porcine circovirus 3
1868221	GCA_004046735.1	Porcine circovirus 3
1868221	GCA_004046755.1	Porcine circovirus 3
1868221	GCA_004038575.1	Porcine circovirus 3
1868221	GCA_004046775.1	Porcine circovirus 3
1868221	GCA_004050875.1	Porcine circovirus 3
1868221	GCA_004038595.1	Porcine circovirus 3
1868221	GCA_004040035.1	Porcine circovirus 3
1868221	GCA_004046795.1	Porcine circovirus 3
1868221	GCA_004038615.1	Porcine circovirus 3
1868221	GCA_004050915.1	Porcine circovirus 3
1868221	GCA_004050955.1	Porcine circovirus 3
1868221	GCA_004050975.1	Porcine circovirus 3
1868221	GCA_004040835.1	Porcine circovirus 3
1868221	GCA_004040855.1	Porcine circovirus 3
1868221	GCA_004040075.1	Porcine circovirus 3
1868221	GCA_004049635.1	Porcine circovirus 3
1868221	GCA_004040095.1	Porcine circovirus 3
1868221	GCA_004043095.1	Porcine circovirus 3
1868221	GCA_004043135.1	Porcine circovirus 3
1868221	GCA_004043155.1	Porcine circovirus 3
1868221	GCA_004049675.1	Porcine circovirus 3
1868221	GCA_004043235.1	Porcine circovirus 3
1868221	GCA_004043255.1	Porcine circovirus 3
1868221	GCA_004043275.1	Porcine circovirus 3
1868221	GCA_004047375.1	Porcine circovirus 3
1868221	GCA_004043295.1	Porcine circovirus 3

1868221	GCA_004047395.1	Porcine circovirus 3
1868221	GCA_004039795.1	Porcine circovirus 3
1868221	GCA_004039455.1	Porcine circovirus 3
1868221	GCA_004043335.1	Porcine circovirus 3
1868221	GCA_004043355.1	Porcine circovirus 3
1868221	GCA_004049655.1	Porcine circovirus 3
1868221	GCA_004040175.1	Porcine circovirus 3
1868221	GCA_004041495.1	Porcine circovirus 3
1868221	GCA_004049695.1	Porcine circovirus 3
1868221	GCA_004041535.1	Porcine circovirus 3
1868221	GCA_004049735.1	Porcine circovirus 3
1868221	GCA_004143545.1	Porcine circovirus 3
1868221	GCA_004143565.1	Porcine circovirus 3
1868221	GCA_004143525.1	Porcine circovirus 3
1868221	GCA_004338325.1	Porcine circovirus 3
1868221	GCA_004194945.1	Porcine circovirus 3
1868221	GCA_004338345.1	Porcine circovirus 3
1868221	GCA_004194965.1	Porcine circovirus 3
1868221	GCA_004194985.1	Porcine circovirus 3
1868221	GCA_004195005.1	Porcine circovirus 3
1868221	GCA_004338115.1	Porcine circovirus 3
1868221	GCA_004338045.1	Porcine circovirus 3
1868221	GCA_004338135.1	Porcine circovirus 3
1868221	GCA_004320425.1	Porcine circovirus 3
1868221	GCA_004320445.1	Porcine circovirus 3
1868221	GCA_004320465.1	Porcine circovirus 3
1868221	GCA_004320485.1	Porcine circovirus 3
1868221	GCA_004338075.1	Porcine circovirus 3
1868221	GCA_004338285.1	Porcine circovirus 3
1868221	GCA_004194905.1	Porcine circovirus 3
1868221	GCA_004338305.1	Porcine circovirus 3
1868221	GCA_004194925.1	Porcine circovirus 3
720569	GCA_000888095.1	Porcine circovirus type 1/2a
720569	GCA_004086635.1	Porcine circovirus type 1/2a
720569	GCA_004086655.1	Porcine circovirus type 1/2a
85709	GCA_004062015.1	Porcine circovirus type 2-B
85709	GCA_004058575.1	Porcine circovirus type 2-B
85709	GCA_004077395.1	Porcine circovirus type 2-B
85709	GCA_004068275.1	Porcine circovirus type 2-B
85709	GCA_004077415.1	Porcine circovirus type 2-B
85709	GCA_004086675.1	Porcine circovirus type 2-B
85543	GCA_004058615.1	Porcine circovirus type 2-C
86385	GCA_004058535.1	Porcine circovirus type 2-D
85544	GCA_004058595.1	Porcine circovirus type 2-E
1506546	GCA_002890155.1	Porcine circovirus-like virus P1
1506546	GCA_004058735.1	Porcine circovirus-like virus P1
1506546	GCA_004058795.1	Porcine circovirus-like virus P1
1506546	GCA_004058675.1	Porcine circovirus-like virus P1
1506546	GCA_004058755.1	Porcine circovirus-like virus P1
1506546	GCA_004058695.1	Porcine circovirus-like virus P1

1506546	GCA_004058815.1	Porcine circovirus-like virus P1
1506546	GCA_004058775.1	Porcine circovirus-like virus P1
1506546	GCA_004058655.1	Porcine circovirus-like virus P1
1506546	GCA_004058715.1	Porcine circovirus-like virus P1
1506546	GCA_004325295.1	Porcine circovirus-like virus P1
1506546	GCA_004325335.1	Porcine circovirus-like virus P1
1506546	GCA_004284835.1	Porcine circovirus-like virus P1
1506546	GCA_004284855.1	Porcine circovirus-like virus P1
1506546	GCA_004325355.1	Porcine circovirus-like virus P1
1506546	GCA_004325315.1	Porcine circovirus-like virus P1
1159905	GCA_000895395.2	Porcine coronavirus HKU15
1159905	GCA_002816235.1	Porcine coronavirus HKU15
168238	GCA_000839825.1	Porcine endogenous retrovirus E
106333	GCA_000849945.1	Porcine enteric sapovirus
64141	GCA_000863405.1	Porcine enterovirus 9
28295	GCA_000848685.1	Porcine epidemic diarrhea virus
28295	GCA_900197235.1	Porcine epidemic diarrhea virus
28295	GCA_900197325.1	Porcine epidemic diarrhea virus
28295	GCA_900197285.1	Porcine epidemic diarrhea virus
28295	GCA_900197535.1	Porcine epidemic diarrhea virus
28295	GCA_900205315.1	Porcine epidemic diarrhea virus
28295	GCA_900197405.1	Porcine epidemic diarrhea virus
28295	GCA_900197355.1	Porcine epidemic diarrhea virus
28295	GCA_900197115.1	Porcine epidemic diarrhea virus
28295	GCA_900197425.1	Porcine epidemic diarrhea virus
28295	GCA_900197165.1	Porcine epidemic diarrhea virus
28295	GCA_900197365.1	Porcine epidemic diarrhea virus
28295	GCA_900197345.1	Porcine epidemic diarrhea virus
28295	GCA_900197105.1	Porcine epidemic diarrhea virus
28295	GCA_900197095.1	Porcine epidemic diarrhea virus
28295	GCA_900197415.1	Porcine epidemic diarrhea virus
28295	GCA_900197135.1	Porcine epidemic diarrhea virus
28295	GCA_900197125.1	Porcine epidemic diarrhea virus
28295	GCA_900197475.1	Porcine epidemic diarrhea virus
28295	GCA_900197485.1	Porcine epidemic diarrhea virus
28295	GCA_900197275.1	Porcine epidemic diarrhea virus
28295	GCA_900197315.1	Porcine epidemic diarrhea virus
28295	GCA_900197335.1	Porcine epidemic diarrhea virus
28295	GCA_900197145.1	Porcine epidemic diarrhea virus
28295	GCA_900197185.1	Porcine epidemic diarrhea virus
28295	GCA_900197225.1	Porcine epidemic diarrhea virus
28295	GCA_900197555.1	Porcine epidemic diarrhea virus
28295	GCA_900197295.1	Porcine epidemic diarrhea virus
28295	GCA_900197155.1	Porcine epidemic diarrhea virus
28295	GCA_900197395.1	Porcine epidemic diarrhea virus
28295	GCA_900197245.1	Porcine epidemic diarrhea virus
28295	GCA_900197455.1	Porcine epidemic diarrhea virus
28295	GCA_900197385.1	Porcine epidemic diarrhea virus
28295	GCA_900197515.1	Porcine epidemic diarrhea virus
28295	GCA_900197305.1	Porcine epidemic diarrhea virus

28295	GCA_900205355.1	Porcine epidemic diarrhea virus
28295	GCA_900205335.1	Porcine epidemic diarrhea virus
28295	GCA_900205345.1	Porcine epidemic diarrhea virus
28295	GCA_900197565.1	Porcine epidemic diarrhea virus
28295	GCA_900205325.1	Porcine epidemic diarrhea virus
28295	GCA_900188515.1	Porcine epidemic diarrhea virus
28295	GCA_900197545.1	Porcine epidemic diarrhea virus
28295	GCA_900197205.1	Porcine epidemic diarrhea virus
28295	GCA_900197375.1	Porcine epidemic diarrhea virus
1843772	GCA_001645995.1	Porcine faeces associated circular DNA molecule-1
2017712	GCA_002271165.1	Porcine feces-associated gemycircularvirus
494652	GCA_002827505.1	Porcine hokovirus
1156769	GCA_001008455.1	Porcine kobuvirus
1156769	GCA_900197265.1	Porcine kobuvirus
1136133	GCA_000895935.1	Porcine kobuvirus SH-W-CHN/2010/China
569195	GCA_000881035.1	Porcine kobuvirus swine/S-1-HUN/2007/Hungary
1208310	GCA_000910935.1	Porcine partetravirus
10796	GCA_000839485.1	Porcine parvovirus
1126383	GCA_000930075.1	Porcine parvovirus 2
707546	GCA_000890295.1	Porcine parvovirus 4
1241957	GCA_000914195.1	Porcine parvovirus 5
1472911	GCA_000920435.1	Porcine parvovirus 6
1820046	GCA_004132625.1	Porcine parvovirus 7
1689785	GCA_003029075.1	Porcine pestivirus 1
1689785	GCA_900086865.1	Porcine pestivirus 1
412969	GCA_000916775.1	Porcine pestivirus isolate Bungowannah
442302	GCA_001611625.1	Porcine picobirnavirus
2126099	GCA_004133485.1	Porcine polyomavirus
1965066	GCA_000862745.1	Porcine reproductive and respiratory syndrome virus 1
1965067	GCA_002816115.1	Porcine reproductive and respiratory syndrome virus 2
1357321	GCA_000925555.1	Porcine respirovirus 1
10913	GCA_003087355.1	Porcine rotavirus
10913	GCA_003155635.1	Porcine rotavirus
10967	GCA_002660055.1	Porcine rotavirus A
10967	GCA_002660015.1	Porcine rotavirus A
10967	GCA_002660075.1	Porcine rotavirus A
10967	GCA_002659855.1	Porcine rotavirus A
10967	GCA_002660035.1	Porcine rotavirus A
10967	GCA_002666835.1	Porcine rotavirus A
10967	GCA_002666855.1	Porcine rotavirus A
10967	GCA_002666475.1	Porcine rotavirus A
10967	GCA_002666495.1	Porcine rotavirus A
10967	GCA_002666875.1	Porcine rotavirus A
10967	GCA_002666515.1	Porcine rotavirus A
10967	GCA_002666535.1	Porcine rotavirus A
10967	GCA_002666615.1	Porcine rotavirus A
10967	GCA_002666655.1	Porcine rotavirus A
10967	GCA_002666715.1	Porcine rotavirus A
10967	GCA_002666735.1	Porcine rotavirus A
10967	GCA_002666915.1	Porcine rotavirus A

10967	GCA_002666755.1	Porcine rotavirus A
10967	GCA_002666795.1	Porcine rotavirus A
10967	GCA_002666815.1	Porcine rotavirus A
10967	GCA_002666935.1	Porcine rotavirus A
10967	GCA_002666595.1	Porcine rotavirus A
10967	GCA_002666955.1	Porcine rotavirus A
10967	GCA_002666635.1	Porcine rotavirus A
10967	GCA_002666695.1	Porcine rotavirus A
10967	GCA_002680155.1	Porcine rotavirus A
10967	GCA_002681495.1	Porcine rotavirus A
10967	GCA_002666775.1	Porcine rotavirus A
10967	GCA_003071105.1	Porcine rotavirus A
10967	GCA_003004905.1	Porcine rotavirus A
10967	GCA_003004895.1	Porcine rotavirus A
10967	GCA_003004915.1	Porcine rotavirus A
10968	GCA_003087295.1	Porcine rotavirus C
10968	GCA_003087275.1	Porcine rotavirus C
10968	GCA_003087315.1	Porcine rotavirus C
53179	GCA_000871825.1	Porcine rubulavirus
1002921	GCA_000863425.1	Porcine sapelovirus 1
1891204	GCA_004133605.1	Porcine serum-associated circular virus
1843773	GCA_001646175.1	Porcine stool-associated circular virus
1843773	GCA_003967995.1	Porcine stool-associated circular virus
1328031	GCA_000908375.1	Porcine stool-associated circular virus 2
1328031	GCA_003964215.1	Porcine stool-associated circular virus 2
1328032	GCA_003963695.1	Porcine stool-associated circular virus 3
1328032	GCA_003963715.1	Porcine stool-associated circular virus 3
1328032	GCA_003963735.1	Porcine stool-associated circular virus 3
1475062	GCA_000919035.1	Porcine stool-associated circular virus 4
1475063	GCA_000919675.1	Porcine stool-associated circular virus 5
1537166	GCA_003967895.1	Porcine stool-associated circular virus 7
1537166	GCA_003967915.1	Porcine stool-associated circular virus 7
1537166	GCA_003967935.1	Porcine stool-associated circular virus 7
2017724	GCA_002270825.1	Porcine stool-associated circular virus/BEL/15V010
85506	GCA_000857805.1	Porcine teschovirus 1
237020	GCA_000913035.1	Porcine torovirus
237020	GCA_900197435.1	Porcine torovirus
1667384	GCA_001045345.1	Portulaca latent viroid
1105380	GCA_000917395.1	Posavirus 1
1105380	GCA_900197525.1	Posavirus 1
1105381	GCA_000916555.1	Posavirus 2
1663127	GCA_001431875.1	Posavirus 3
1930510	GCA_002374955.1	Posavirus sp.
1930510	GCA_003727535.1	Posavirus sp.
1930510	GCA_003727555.1	Posavirus sp.
1930510	GCA_003727575.1	Posavirus sp.
1930510	GCA_003727595.1	Posavirus sp.
1930510	GCA_003727495.1	Posavirus sp.
1930510	GCA_003727515.1	Posavirus sp.
1930510	GCA_003728675.1	Posavirus sp.

1930510	GCA_003728695.1	Posavirus sp.
1930510	GCA_003728715.1	Posavirus sp.
1930510	GCA_003728735.1	Posavirus sp.
1930510	GCA_003728755.1	Posavirus sp.
1930510	GCA_003728255.1	Posavirus sp.
1930510	GCA_003728775.1	Posavirus sp.
1930510	GCA_003728795.1	Posavirus sp.
1930510	GCA_003728815.1	Posavirus sp.
1930510	GCA_003728835.1	Posavirus sp.
1930510	GCA_003728495.1	Posavirus sp.
1930510	GCA_003728395.1	Posavirus sp.
1930510	GCA_003728335.1	Posavirus sp.
1930510	GCA_003728055.1	Posavirus sp.
1930510	GCA_003728095.1	Posavirus sp.
1930510	GCA_003728135.1	Posavirus sp.
1930510	GCA_003728375.1	Posavirus sp.
1930510	GCA_003728075.1	Posavirus sp.
1930510	GCA_003728455.1	Posavirus sp.
1930510	GCA_003728315.1	Posavirus sp.
1930510	GCA_003728155.1	Posavirus sp.
1930510	GCA_003728195.1	Posavirus sp.
1930510	GCA_003728115.1	Posavirus sp.
1930510	GCA_003728215.1	Posavirus sp.
1930510	GCA_003728235.1	Posavirus sp.
1930510	GCA_003728175.1	Posavirus sp.
1930510	GCA_003728295.1	Posavirus sp.
1930510	GCA_003728355.1	Posavirus sp.
1930510	GCA_003728415.1	Posavirus sp.
1930510	GCA_003728435.1	Posavirus sp.
1930510	GCA_003728475.1	Posavirus sp.
1930510	GCA_003728515.1	Posavirus sp.
1930510	GCA_003728535.1	Posavirus sp.
1930510	GCA_003728555.1	Posavirus sp.
1930510	GCA_003728575.1	Posavirus sp.
1930510	GCA_003728595.1	Posavirus sp.
1930510	GCA_003728615.1	Posavirus sp.
1930510	GCA_003728635.1	Posavirus sp.
1930510	GCA_003728275.1	Posavirus sp.
1930510	GCA_003728655.1	Posavirus sp.
1488557	GCA_000919895.1	PoSCV Kor J481
263531	GCA_000869665.1	Possum enterovirus W1
1118369	GCA_000973275.1	Possum nidovirus
2170410	GCA_004132745.1	Potamochoerus porcus polyomavirus 1
410826	GCA_000868685.1	Potato apical leaf curl disease-associated satellite DNA bet
12182	GCA_000852045.1	Potato aucuba mosaic virus
257464	GCA_000913055.1	Potato black ringspot virus
138982	GCA_000883255.1	Potato latent virus
12045	GCA_000856725.1	Potato leafroll virus
37128	GCA_000850685.1	Potato mop-top virus
1725329	GCA_001629905.1	Potato necrosis virus

106118	GCA_000871905.1	Potato rough dwarf virus
12892	GCA_000856265.1	Potato spindle tuber viroid
12215	GCA_000861585.1	Potato virus A
12215	GCA_002987615.1	Potato virus A
2340870	GCA_003033835.1	Potato virus B
1046402	GCA_000899815.1	Potato virus H
12167	GCA_000860585.1	Potato virus M
12169	GCA_000866605.1	Potato virus S
36403	GCA_000879695.1	Potato virus T
2170039	GCA_004117715.1	Potato virus U
40640	GCA_000860845.1	Potato virus V
12183	GCA_000866465.1	Potato virus X
12216	GCA_000862905.1	Potato virus Y
12216	GCA_002828865.1	Potato virus Y
12216	GCA_002987625.1	Potato virus Y
12217	GCA_002987635.1	Potato virus Y strain C
122280	GCA_002828885.1	Potato virus Y strain NTN
317719	GCA_002987655.1	Potato virus Y strain Wilga 156
195060	GCA_000895555.1	Potato yellow dwarf nucleorhabdovirus
223307	GCA_000839305.1	Potato yellow mosaic Panama virus
10827	GCA_000842985.1	Potato yellow mosaic virus
223310	GCA_000838665.1	Potato yellow mosaic virus - [Venezuela]
103881	GCA_000852745.1	Potato yellow vein virus
2283800	GCA_004134325.1	Potexvirus sp.
44562	GCA_000859825.1	Pothos latent virus
64314	GCA_001754105.1	Potiskum virus
1225069	GCA_000918895.1	Pouzolzia golden mosaic virus
1367126	GCA_002823205.1	Pouzolzia mosaic Guangdong virus
1842663	GCA_002924545.1	Powai lake megavirus
11083	GCA_000860485.1	Powassan virus
1198134	GCA_002823225.1	Premna leaf curl virus
2491281	GCA_004379415.1	Prevotella phage Lak-A1
2491281	GCA_004379435.1	Prevotella phage Lak-A1
2491281	GCA_004379455.1	Prevotella phage Lak-A1
2491281	GCA_004379475.1	Prevotella phage Lak-A1
2491282	GCA_004379495.1	Prevotella phage Lak-A2
2491283	GCA_004379515.1	Prevotella phage Lak-B1
2491284	GCA_004379535.1	Prevotella phage Lak-B2
2491285	GCA_004379555.1	Prevotella phage Lak-B3
2491286	GCA_004379575.1	Prevotella phage Lak-B4
2491287	GCA_004379595.1	Prevotella phage Lak-B5
2491288	GCA_004379615.1	Prevotella phage Lak-B6
2491289	GCA_004379635.1	Prevotella phage Lak-B7
2491290	GCA_004379655.1	Prevotella phage Lak-B8
2491291	GCA_004379675.1	Prevotella phage Lak-B9
2491292	GCA_004379695.1	Prevotella phage Lak-C1
1038068	GCA_000864565.1	PreXMRV-1
1511871	GCA_000866725.1	Primate bocaparvovirus 1
1511871	GCA_003033285.1	Primate bocaparvovirus 1
1903318	GCA_001755385.1	Primate norovirus

1511907	GCA_002827465.1	Primate protoparvovirus 1
1692262	GCA_001274245.1	Primnoa pacifica coral associated circular virus
479713	GCA_000885855.1	Primula malacoides virus China/Mar2007
1811408	GCA_001766565.1	Privet leaf blotch-associated virus
2169960	GCA_001308655.1	Privet ringspot virus
889955	GCA_000907015.1	Prochlorococcus phage MED4-184
889956	GCA_000904535.1	Prochlorococcus phage MED4-213
382262	GCA_000906175.1	Prochlorococcus phage P-GSP1
445700	GCA_000892455.1	Prochlorococcus phage P-HM1
445696	GCA_000892475.1	Prochlorococcus phage P-HM2
444862	GCA_000890835.1	Prochlorococcus phage P-RSM4
268746	GCA_000859585.1	Prochlorococcus phage P-SSM2
268746	GCA_002745975.1	Prochlorococcus phage P-SSM2
536453	GCA_000907775.1	Prochlorococcus phage P-SSM3
268747	GCA_000857985.1	Prochlorococcus phage P-SSM4
536454	GCA_002991095.1	Prochlorococcus phage P-SSM5
445688	GCA_000893395.1	Prochlorococcus phage P-SSM7
885867	GCA_000905515.1	Prochlorococcus phage P-SSP10
382273	GCA_000904555.1	Prochlorococcus phage P-SSP3
382275	GCA_002630285.1	Prochlorococcus phage P-SSP6
1542477	GCA_001503075.1	Prochlorococcus phage P-TIM68
444861	GCA_000892495.1	Prochlorococcus phage Syn1
444878	GCA_000891815.1	Prochlorococcus phage Syn33
268748	GCA_000858745.1	Prochlorococcus virus PSSP7
312349	GCA_000862885.1	Procyon lotor papillomavirus 1
188938	GCA_000919835.1	Pronghorn antelope pestivirus
1897531	GCA_002613165.1	Propionibacterium phage Anatole
2041558	GCA_002745155.1	Propionibacterium phage Aquarius
1229794	GCA_000900295.1	Propionibacterium phage ATCC29399B_C
1229793	GCA_000898835.1	Propionibacterium phage ATCC29399B_T
1655012	GCA_001190715.1	Propionibacterium phage Attacne
1897532	GCA_002613185.1	Propionibacterium phage B22
1897533	GCA_002613205.1	Propionibacterium phage B3
189836	GCA_000838285.1	Propionibacterium phage B5
1654740	GCA_001745815.1	Propionibacterium phage BruceLethal
1897534	GCA_002613225.1	Propionibacterium phage Doucette
2041559	GCA_002745175.1	Propionibacterium phage DrParker
1897535	GCA_002613245.1	Propionibacterium phage E1
1897536	GCA_002613265.1	Propionibacterium phage E6
1654780	GCA_001744495.1	Propionibacterium phage Enoki
1897537	GCA_002613285.1	Propionibacterium phage G4
1655013	GCA_002623025.1	Propionibacterium phage Keiki
1655014	GCA_001190295.1	Propionibacterium phage Kubed
1655015	GCA_001190435.1	Propionibacterium phage Lauchelly
2041560	GCA_002745195.1	Propionibacterium phage Leviosa
2041561	GCA_002745215.1	Propionibacterium phage LilBandit
2041562	GCA_002745235.1	Propionibacterium phage MEAK
1654781	GCA_001743815.1	Propionibacterium phage Moyashi
1655016	GCA_001190575.1	Propionibacterium phage MrAK
1655017	GCA_001190695.1	Propionibacterium phage Ouroboros

1229792	GCA_000899415.1	Propionibacterium phage P1.1
1229791	GCA_000897835.1	Propionibacterium phage P100_1
1229790	GCA_000900275.1	Propionibacterium phage P100_A
1229789	GCA_000898815.1	Propionibacterium phage P100D
1229786	GCA_000900255.1	Propionibacterium phage P101A
1229787	GCA_000897815.1	Propionibacterium phage P104A
1229788	GCA_000899395.1	Propionibacterium phage P105
1229784	GCA_000898795.1	Propionibacterium phage P14.4
1229782	GCA_000899375.1	Propionibacterium phage P9.1
1747271	GCA_001470955.1	Propionibacterium phage PA1-14
2079399	GCA_002958555.1	Propionibacterium phage pa15
2079400	GCA_002958565.1	Propionibacterium phage pa27
2079401	GCA_002958575.1	Propionibacterium phage pa28
2079402	GCA_002958585.1	Propionibacterium phage pa29399-1-D_1
2079403	GCA_002958595.1	Propionibacterium phage pa29399-1-D_2
2079405	GCA_002958615.1	Propionibacterium phage pa310
2079406	GCA_002958625.1	Propionibacterium phage pa33
2079407	GCA_002958635.1	Propionibacterium phage pa35
2079404	GCA_002958605.1	Propionibacterium phage pa3-SS3
2079408	GCA_002958645.1	Propionibacterium phage pa59
376758	GCA_000871645.1	Propionibacterium phage PA6
2079409	GCA_002958655.1	Propionibacterium phage pa615
2079410	GCA_002958665.1	Propionibacterium phage pa63
2079411	GCA_002958675.1	Propionibacterium phage pa6919-4
2079412	GCA_002958685.1	Propionibacterium phage pa9-6919-4
1690805	GCA_001505695.1	Propionibacterium phage PAC1
1690814	GCA_002598945.1	Propionibacterium phage PAC10
1690806	GCA_002598785.1	Propionibacterium phage PAC2
1690807	GCA_002598805.1	Propionibacterium phage PAC3
1690808	GCA_002598825.1	Propionibacterium phage PAC4
1690809	GCA_002598845.1	Propionibacterium phage PAC5
1690810	GCA_002598865.1	Propionibacterium phage PAC6
1690811	GCA_002598885.1	Propionibacterium phage PAC7
1690812	GCA_002598905.1	Propionibacterium phage PAC8
1690813	GCA_002598925.1	Propionibacterium phage PAC9
1498188	GCA_001042095.1	Propionibacterium phage Pacnes 2012-15
1983620	GCA_002623925.1	Propionibacterium phage PacnesP1
1983621	GCA_002623945.1	Propionibacterium phage PacnesP2
504501	GCA_000891975.1	Propionibacterium phage PAD20
504553	GCA_000892635.1	Propionibacterium phage PAS50
1838137	GCA_001745255.1	Propionibacterium phage PFR1
1838138	GCA_001745915.1	Propionibacterium phage PFR2
1500798	GCA_001041315.1	Propionibacterium phage PHL009M11
1235645	GCA_000911055.1	Propionibacterium phage PHL010M04
1500799	GCA_001042395.1	Propionibacterium phage PHL025M00
1500800	GCA_001042035.1	Propionibacterium phage PHL030N00
1235646	GCA_002623045.1	Propionibacterium phage PHL037M02
1500801	GCA_001041675.1	Propionibacterium phage PHL041M10
1500802	GCA_001041295.1	Propionibacterium phage PHL055N00
1235647	GCA_000912735.1	Propionibacterium phage PHL060L00

1500803	GCA_002756235.1	Propionibacterium phage PHL064M01
1500804	GCA_002756255.1	Propionibacterium phage PHL064M02
1235648	GCA_002755375.1	Propionibacterium phage PHL066M04
1500805	GCA_002623065.1	Propionibacterium phage PHL067M01
1500806	GCA_002605885.1	Propionibacterium phage PHL067M09
1235649	GCA_000912075.1	Propionibacterium phage PHL067M10
1500807	GCA_001041655.1	Propionibacterium phage PHL070N00
1235650	GCA_000911015.1	Propionibacterium phage PHL071N05
1235651	GCA_002755395.1	Propionibacterium phage PHL073M02
1500808	GCA_001041275.1	Propionibacterium phage PHL082M00
1500809	GCA_002756275.1	Propionibacterium phage PHL082M02
1500810	GCA_002623085.1	Propionibacterium phage PHL082M03
1500811	GCA_002756295.1	Propionibacterium phage PHL082M04
1235652	GCA_002755415.1	Propionibacterium phage PHL085M01
1500812	GCA_001042355.1	Propionibacterium phage PHL085N00
1500813	GCA_001041995.1	Propionibacterium phage PHL092M00
1500814	GCA_001041635.1	Propionibacterium phage PHL095N00
1235653	GCA_000912055.1	Propionibacterium phage PHL111M01
1235654	GCA_000912675.1	Propionibacterium phage PHL112N00
1235655	GCA_000912035.1	Propionibacterium phage PHL113M01
1235656	GCA_000913515.1	Propionibacterium phage PHL114L00
1500815	GCA_002602945.1	Propionibacterium phage PHL114N00
1235657	GCA_002755435.1	Propionibacterium phage PHL115M02
1500816	GCA_001042335.1	Propionibacterium phage PHL116M00
1500817	GCA_002605905.1	Propionibacterium phage PHL116M10
1500818	GCA_002623105.1	Propionibacterium phage PHL117M00
1500819	GCA_002623125.1	Propionibacterium phage PHL117M01
1500820	GCA_001041615.1	Propionibacterium phage PHL132N00
1500821	GCA_001041235.1	Propionibacterium phage PHL141N00
1500822	GCA_001038615.1	Propionibacterium phage PHL150M00
1500823	GCA_002623145.1	Propionibacterium phage PHL151M00
1500824	GCA_002623165.1	Propionibacterium phage PHL151N00
1500825	GCA_001041955.1	Propionibacterium phage PHL152M00
1500826	GCA_002623185.1	Propionibacterium phage PHL163M00
1500827	GCA_001041215.1	Propionibacterium phage PHL171M01
1500828	GCA_001042295.1	Propionibacterium phage PHL179M00
1500829	GCA_002623205.1	Propionibacterium phage PHL194M00
1500830	GCA_001040755.1	Propionibacterium phage PHL199M00
1500831	GCA_001041575.1	Propionibacterium phage PHL301M00
1500832	GCA_002623225.1	Propionibacterium phage PHL308M00
1655018	GCA_001190275.1	Propionibacterium phage Pirate
1655019	GCA_001190415.1	Propionibacterium phage Procrass1
1654782	GCA_001745135.1	Propionibacterium phage QueenBey
1655020	GCA_001190735.1	Propionibacterium phage SKKY
1655021	GCA_001190315.1	Propionibacterium phage Solid
1655022	GCA_001190455.1	Propionibacterium phage Stormborn
2041563	GCA_002745255.1	Propionibacterium phage Supernova
1655023	GCA_001190085.1	Propionibacterium phage Wizzo
1980485	GCA_002994685.1	Prospect Hill orthohantavirus
1912593	GCA_002710055.1	Proteobacteria phage MWH-Nonnen-W8red 1

1912592	GCA_002709985.1	Proteobacteria phage MWH-Nonnen-W8red 2
2483610	GCA_003865475.1	Proteus phage Mydo
2065203	GCA_002957925.1	Proteus phage phiP4-3
1837877	GCA_002743475.1	Proteus phage PM 116
1560282	GCA_001042175.1	Proteus phage PM 75
1560283	GCA_001041815.1	Proteus phage PM 85
1560284	GCA_001041455.1	Proteus phage PM 93
2048008	GCA_002957135.1	Proteus phage PM135
1357704	GCA_001041895.1	Proteus phage PM16
2048007	GCA_002957125.1	Proteus phage PM87
1567485	GCA_001505295.1	Proteus phage pPM_01
2315597	GCA_003668295.1	Proteus phage Stubb
1636250	GCA_001500615.1	Proteus phage vB_PmiM_Pm5461
1636249	GCA_001501275.1	Proteus phage vB_PmiP_Pm5460
1969841	GCA_002621085.1	Proteus phage VB_PmiS-Isfahan
1933093	GCA_002618425.1	Proteus phage vB_PvuS_Pm34
1755694	GCA_003033325.1	Protoparvovirus Zsana/2013/HUN
213633	GCA_000887375.1	Providence virus
1235559	GCA_000897855.1	Providencia phage Redjac
1931407	GCA_002617785.1	Providencia phage vB_PreS_PR1
2508090	GCA_004146665.1	Providencia phage vB_PstP_Stuart
33760	GCA_000869765.1	Prune dwarf virus
37733	GCA_000851045.1	Prunus necrotic ringspot virus
1855510	GCA_003033845.1	Prunus virus F
1472425	GCA_000922315.1	Prunus virus T
2358449	GCA_004134405.1	Psammotettix alienus iflavirus 1
36355	GCA_000886255.1	Pseudalatia unipuncta granulovirus
1506997	GCA_003330585.1	Pseudoalteromonas phage B8b
1874539	GCA_001882195.1	Pseudoalteromonas phage BS5
1916107	GCA_002615645.1	Pseudoalteromonas phage C5a
2510494	GCA_004149965.1	Pseudoalteromonas phage C7
2099852	GCA_002997855.1	Pseudoalteromonas phage Cr39582
2480622	GCA_003691815.1	Pseudoalteromonas phage DW
2510463	GCA_004347065.1	Pseudoalteromonas phage GXT1010
1654919	GCA_001551565.1	Pseudoalteromonas phage H101
1636200	GCA_001503815.1	Pseudoalteromonas phage H103
877240	GCA_000892515.1	Pseudoalteromonas phage H105/1
1357705	GCA_002603865.1	Pseudoalteromonas phage HM1
1357706	GCA_002603885.1	Pseudoalteromonas phage HP1
1357707	GCA_002603905.1	Pseudoalteromonas phage HS1
1357708	GCA_002603925.1	Pseudoalteromonas phage HS2
1357709	GCA_002603945.1	Pseudoalteromonas phage HS5
1357710	GCA_002603965.1	Pseudoalteromonas phage HS6
2041340	GCA_002743895.1	Pseudoalteromonas phage J2-1
2024350	GCA_003329105.1	Pseudoalteromonas phage KB12-38
2065202	GCA_003059675.1	Pseudoalteromonas phage Maelstrom
1874540	GCA_001882135.1	Pseudoalteromonas phage PH1
1913046	GCA_002614885.1	Pseudoalteromonas phage PH357
1955235	GCA_002619045.1	Pseudoalteromonas phage PHS21
1913111	GCA_002615125.1	Pseudoalteromonas phage PHS3

1667322	GCA_001550505.1	Pseudoalteromonas phage Pq0
754052	GCA_000906015.1	Pseudoalteromonas phage pYD6-A
1316739	GCA_000907495.1	Pseudoalteromonas phage RIO-1
2023998	GCA_002627545.1	Pseudoalteromonas phage SL20
2023999	GCA_002627565.1	Pseudoalteromonas phage SL25
1366055	GCA_002604005.1	Pseudoalteromonas phage TW1
1856120	GCA_002610665.1	Pseudoalteromonas phage vB_PspS-H40/1
10661	GCA_000840405.1	Pseudoalteromonas virus PM2
1852626	GCA_002610165.2	Pseudoalteromonas virus vB_PspP-H6/1
1852626	GCA_002610165.1	Pseudoalteromonas virus vB_PspP-H6/1
129726	GCA_000886295.1	Pseudocowpox virus
1685502	GCA_001685305.1	Pseudogymnoascus destructans partitivirus-pa
197783	GCA_000842165.1	Pseudomonad phage gh-1
2419747	GCA_004146485.1	Pseudomonas aeruginosa hage PaZq-1
581037	GCA_000880975.1	Pseudomonas phage 14-1
1729938	GCA_900016745.1	Pseudomonas phage 15b
1729936	GCA_900016765.1	Pseudomonas phage 17A
198110	GCA_000875305.1	Pseudomonas phage 201phi2-1
2163970	GCA_003143215.1	Pseudomonas phage 22PfluR64PP
2163970	GCA_003143215.2	Pseudomonas phage 22PfluR64PP
2163980	GCA_003143435.2	Pseudomonas phage 67PfluR64PP
2163980	GCA_003143435.1	Pseudomonas phage 67PfluR64PP
2163977	GCA_003143255.2	Pseudomonas phage 71PfluR64PP
2163977	GCA_003143255.1	Pseudomonas phage 71PfluR64PP
347325	GCA_000864505.1	Pseudomonas phage 73
2163965	GCA_003143395.1	Pseudomonas phage 98PfluR60PP
1775248	GCA_002608845.1	Pseudomonas phage AAT-1
2163982	GCA_003093995.1	Pseudomonas phage Achelous
1235689	GCA_000902895.1	Pseudomonas phage AF
2163983	GCA_003094015.1	Pseudomonas phage Alpheus
1868597	GCA_002610895.1	Pseudomonas phage AN14
1873949	GCA_001744655.1	Pseudomonas phage Andromeda
151599	GCA_000844345.1	Pseudomonas phage B3
1100790	GCA_000895915.1	Pseudomonas phage Bf7
2079288	GCA_002958465.1	Pseudomonas phage Bjorn
2029635	GCA_003522505.1	Pseudomonas phage BrSP1
1735586	GCA_001470035.1	Pseudomonas phage C11
1327965	GCA_000915815.1	Pseudomonas phage CHA_P1
2053681	GCA_003861655.1	Pseudomonas phage Delta
1640975	GCA_002597225.1	Pseudomonas phage DL52
1640969	GCA_001500955.1	Pseudomonas phage DL54
1640970	GCA_001500995.1	Pseudomonas phage DL60
1640972	GCA_001502995.1	Pseudomonas phage DL62
1640973	GCA_001502375.1	Pseudomonas phage DL64
1640974	GCA_001501595.1	Pseudomonas phage DL68
1542100	GCA_003060145.1	Pseudomonas phage DO4
2483611	GCA_003865495.1	Pseudomonas phage Dobby
2282401	GCA_003369265.1	Pseudomonas phage E79
273133	GCA_000866825.1	Pseudomonas phage EL
2307269	GCA_004015945.1	Pseudomonas phage EPa61

1542103	GCA_003069425.1	Pseudomonas phage F_ET309sp/Pa1651
1542104	GCA_003062225.1	Pseudomonas phage F_ET605sp/Pa1651
1542105	GCA_003060175.1	Pseudomonas phage F_HA1208sp/Pa1651
1542106	GCA_003060205.1	Pseudomonas phage F_HA1961sp/Pa1641
1542107	GCA_003060225.1	Pseudomonas phage F_KK2074sp/Pa1651
1542108	GCA_003060245.1	Pseudomonas phage F_MX1987sp/MX560
1542109	GCA_003060265.1	Pseudomonas phage F_MX560sp/Pa1651
1542110	GCA_003060285.1	Pseudomonas phage F_TK1718sp/PAK
1542111	GCA_003060305.1	Pseudomonas phage F_TK1727sp/MX560
1542112	GCA_003060325.1	Pseudomonas phage F_TK1932sp/Pa1651
1542113	GCA_003114155.1	Pseudomonas phage F_TK432sp/Pa1651
347324	GCA_000865465.1	Pseudomonas phage F10
347329	GCA_000864525.1	Pseudomonas phage F8
1701859	GCA_002597325.1	Pseudomonas phage Gallinipper
2055238	GCA_900241075.1	Pseudomonas phage GP100
2055238	GCA_900291915.2	Pseudomonas phage GP100
1536596	GCA_001041135.1	Pseudomonas phage H70
1755216	GCA_900013285.1	Pseudomonas phage HC15b2
2079287	GCA_002958455.1	Pseudomonas phage Henninger
2499902	GCA_004138875.1	Pseudomonas phage Henu5
2041348	GCA_002743995.1	Pseudomonas phage IME180
2048976	GCA_002957155.1	Pseudomonas phage inbricus
1225791	GCA_002149725.1	Pseudomonas phage JBD18
1542114	GCA_003060345.1	Pseudomonas phage JBD23
1223259	GCA_000906575.1	Pseudomonas phage JBD24
1225792	GCA_002149485.1	Pseudomonas phage JBD25
1093672	GCA_002601925.1	Pseudomonas phage JBD26
1223260	GCA_000904095.1	Pseudomonas phage JBD30
1777052	GCA_001737055.1	Pseudomonas phage JBD44
1223261	GCA_000905735.1	Pseudomonas phage JBD5
1542115	GCA_003060365.1	Pseudomonas phage JBD58a
1542116	GCA_002604985.1	Pseudomonas phage JBD68
1777053	GCA_001736375.1	Pseudomonas phage JBD69
1223262	GCA_000905115.1	Pseudomonas phage JBD88a
1542117	GCA_003060405.1	Pseudomonas phage JBD88b
1542118	GCA_003060425.1	Pseudomonas phage JBD90
1777054	GCA_001736595.1	Pseudomonas phage JBD93
1542119	GCA_003060445.1	Pseudomonas phage JBD94b
1542120	GCA_003060465.1	Pseudomonas phage JBDs5
1481224	GCA_000922915.1	Pseudomonas phage JD024
757342	GCA_000902295.1	Pseudomonas phage JG004
1970799	GCA_002955025.1	Pseudomonas phage JG012
749447	GCA_000900635.1	Pseudomonas phage JG024
1970800	GCA_002955035.1	Pseudomonas phage JG054
1701860	GCA_002597345.1	Pseudomonas phage Jollyroger
1777072	GCA_001736575.1	Pseudomonas phage K5
1716041	GCA_001504115.1	Pseudomonas phage K8
582345	GCA_000892435.1	Pseudomonas phage KPP10
763998	GCA_000904895.1	Pseudomonas phage KPP12
1678082	GCA_001501535.1	Pseudomonas phage KPP21

1772250	GCA_002608315.1	Pseudomonas phage KPP22
1772251	GCA_002608365.1	Pseudomonas phage KPP22M1
1772252	GCA_002608395.1	Pseudomonas phage KPP22M2
1772253	GCA_002608435.1	Pseudomonas phage KPP22M3
1462581	GCA_002633165.1	Pseudomonas phage KPP23
1462608	GCA_000918235.1	Pseudomonas phage KPP25
1701861	GCA_002597365.1	Pseudomonas phage Kraken
1862701	GCA_002607225.1	Pseudomonas phage KTN4
1701862	GCA_002597385.1	Pseudomonas phage Kula
2530172	GCA_004340505.1	Pseudomonas phage Lana
549445	GCA_000875605.1	Pseudomonas phage LBL3
655098	GCA_000884615.1	Pseudomonas phage LIT1
2079289	GCA_002958475.1	Pseudomonas phage Littlefix
386793	GCA_000872125.1	Pseudomonas phage LKA1
1327940	GCA_002603525.1	Pseudomonas phage LKA5
386792	GCA_000871265.1	Pseudomonas phage LKD16
1308899	GCA_002622505.1	Pseudomonas phage LKO4
549446	GCA_000880415.1	Pseudomonas phage LMA2
2234081	GCA_003307395.1	Pseudomonas phage LP14
1161927	GCA_000897175.1	Pseudomonas phage Lu11
484896	GCA_000879115.1	Pseudomonas phage LUZ19
655097	GCA_000886235.1	Pseudomonas phage LUZ7
1327768	GCA_002630385.1	Pseudomonas phage MBL
1868596	GCA_001745515.1	Pseudomonas phage MD8
1204517	GCA_000899055.1	Pseudomonas phage MP1412
1161903	GCA_000898395.1	Pseudomonas phage MP42
1391190	GCA_000922475.1	Pseudomonas phage MP48
1262514	GCA_000911255.1	Pseudomonas phage MPK6
1225790	GCA_000910915.1	Pseudomonas phage MPK7
1158721	GCA_002755155.1	Pseudomonas phage MR299-2
1701863	GCA_002597405.1	Pseudomonas phage Nemo
2163984	GCA_003093975.1	Pseudomonas phage Nerthus
1701864	GCA_002597425.1	Pseudomonas phage Nessie
1158680	GCA_000901655.1	Pseudomonas phage NH-4
2048977	GCA_002957165.1	Pseudomonas phage nickie
2163985	GCA_003093955.1	Pseudomonas phage Njord
2006684	GCA_002625005.1	Pseudomonas phage Noxifer
1844477	GCA_001744715.1	Pseudomonas phage NP1
1775428	GCA_002630505.1	Pseudomonas phage NP3
2079543	GCA_002958925.1	Pseudomonas phage NV1
1784982	GCA_001755545.1	Pseudomonas phage O4
1124849	GCA_000896635.1	Pseudomonas phage OBP
981430	GCA_002630945.1	Pseudomonas phage P3_CHA
2499142	GCA_004208715.1	Pseudomonas phage PA02
1913575	GCA_002615445.1	Pseudomonas phage PA10
347327	GCA_000867045.1	Pseudomonas phage PA11
1530400	GCA_001040995.1	Pseudomonas phage Pa2
1204542	GCA_002617265.1	Pseudomonas phage PA26
1913570	GCA_002615365.1	Pseudomonas phage PA5
1335230	GCA_000912555.1	Pseudomonas phage PaBG

1718273	GCA_001505555.1	Pseudomonas phage PAE1
2419748	GCA_004146445.1	Pseudomonas phage PaGz-1
504346	GCA_000880695.1	Pseudomonas phage PAJU2
743813	GCA_000891855.1	Pseudomonas phage PAK_P1
1348912	GCA_000913255.1	Pseudomonas phage PAK_P2
981431	GCA_000913175.1	Pseudomonas phage PAK_P3
1327966	GCA_000914095.1	Pseudomonas phage PAK_P4
1327964	GCA_000915135.1	Pseudomonas phage PAK_P5
1175657	GCA_001503615.1	Pseudomonas phage PaMx11
1175653	GCA_002597205.1	Pseudomonas phage PaMx13
1175654	GCA_002623005.1	Pseudomonas phage PaMx25
1175659	GCA_001504415.1	Pseudomonas phage PaMx28
1815974	GCA_002757475.1	Pseudomonas phage PaMx33
1815975	GCA_002757495.1	Pseudomonas phage PaMx35
1815976	GCA_002757515.1	Pseudomonas phage PaMx41
1175662	GCA_001505875.1	Pseudomonas phage PaMx42
1815977	GCA_002757535.1	Pseudomonas phage PaMx43
1815978	GCA_002757555.1	Pseudomonas phage PaMx46
1175655	GCA_002601365.1	Pseudomonas phage PaMx73
1175663	GCA_001505215.1	Pseudomonas phage PaMx74
1548421	GCA_003329845.1	Pseudomonas phage PAN70
1716042	GCA_001551785.1	Pseudomonas phage PaoP5
685892	GCA_000903795.1	Pseudomonas phage PaP1
270673	GCA_000843545.1	Pseudomonas phage PaP2
1273709	GCA_002831005.1	Pseudomonas phage PaP4
2419749	GCA_004146505.1	Pseudomonas phage PaSz-4
2419750	GCA_004146525.1	Pseudomonas phage PaSzW-1
2419751	GCA_004146465.1	Pseudomonas phage PaTs-2
1962679	GCA_003059655.1	Pseudomonas phage PAXYB1
2301646	GCA_003575945.1	Pseudomonas phage PaYy-2
538398	GCA_000883535.1	Pseudomonas phage PB1
1837850	GCA_001745375.1	Pseudomonas phage PEV2
1542121	GCA_003060485.1	Pseudomonas phage PEV31
1542122	GCA_003060505.1	Pseudomonas phage PEV32
1562076	GCA_001038595.1	Pseudomonas phage Pf-10
1815630	GCA_002609525.1	Pseudomonas phage pf16
10872	GCA_000837805.1	Pseudomonas phage Pf3
10872	GCA_002601645.1	Pseudomonas phage Pf3
2201462	GCA_003308855.1	Pseudomonas phage PFP1
2006668	GCA_002624965.1	Pseudomonas phage Phabio
2268587	GCA_003341435.1	Pseudomonas phage phCDa
1542123	GCA_003060525.1	Pseudomonas phage Phi05_1400 B
1754216	GCA_002608035.1	Pseudomonas phage phi1
161736	GCA_000851005.1	Pseudomonas phage phi12
134554	GCA_000852445.1	Pseudomonas phage phi13
988656	GCA_000891635.1	Pseudomonas phage phi15
1541891	GCA_002756415.1	Pseudomonas phage phi176
1450169	GCA_001736915.1	Pseudomonas phage phi2
693582	GCA_000886135.1	Pseudomonas phage phi-2
593131	GCA_000882035.1	Pseudomonas phage phi2954

1129145	GCA_000896755.1	Pseudomonas phage phi297
1754217	GCA_001736495.1	Pseudomonas phage phi3
120086	GCA_000848645.1	Pseudomonas phage phi8
1805958	GCA_002609425.1	Pseudomonas phage phiAH14a
1805959	GCA_002609445.1	Pseudomonas phage phiAH14b
1589273	GCA_001503515.1	Pseudomonas phage PhiCHU
1429758	GCA_000911495.1	Pseudomonas phage phiIBB-PAA2
942165	GCA_000892395.1	Pseudomonas phage phiIBB-PF7A
627480	GCA_000882755.1	Pseudomonas phage phikF77
204270	GCA_000858425.1	Pseudomonas phage phiKMV
1597971	GCA_002605425.1	Pseudomonas phage phiKT28
1597972	GCA_002605445.1	Pseudomonas phage phiKTN6
1815957	GCA_001743955.1	Pseudomonas phage phiMK
1815956	GCA_002629845.1	Pseudomonas phage phiNFS
1603039	GCA_002925585.1	Pseudomonas phage phiNN
2079544	GCA_002990265.1	Pseudomonas phage phiNV3
2340713	GCA_003613555.1	Pseudomonas phage phiPA01_302
2340714	GCA_003613575.1	Pseudomonas phage phiPA01_EW
998086	GCA_001502095.1	Pseudomonas phage PhiPA3
1815582	GCA_002609505.1	Pseudomonas phage phiPMW
1500757	GCA_000921055.1	Pseudomonas phage phiPSA1
1629654	GCA_002604705.1	Pseudomonas phage phiPsa17
1500756	GCA_000921095.1	Pseudomonas phage phiPSA2
1458843	GCA_000916255.1	Pseudomonas phage phiPsa374
1080237	GCA_000917575.1	Pseudomonas phage phiPto-bp6g
1752027	GCA_002623365.1	Pseudomonas phage phiR18
1204538	GCA_000906315.1	Pseudomonas phage Phi-S1
1852644	GCA_002925595.1	Pseudomonas phage phiYY
2059855	GCA_002957515.1	Pseudomonas phage PMBT14
2059855	GCA_002957515.2	Pseudomonas phage PMBT14
2059856	GCA_002957525.1	Pseudomonas phage PMBT3
2079290	GCA_002958485.1	Pseudomonas phage PollyC
1718594	GCA_002607505.1	Pseudomonas phage POR1
1701865	GCA_002597445.1	Pseudomonas phage Poseidon
12023	GCA_000855925.1	Pseudomonas phage PP7
1755692	GCA_001470795.1	Pseudomonas phage PPPL-1
1279082	GCA_000915175.1	Pseudomonas phage PPpW-3
1279083	GCA_000916695.1	Pseudomonas phage PPpW-4
2041350	GCA_003029625.1	Pseudomonas phage PPSC2
12024	GCA_000868365.1	Pseudomonas phage PRR1
1573458	GCA_001550905.1	Pseudomonas phage PS-1
2530023	GCA_004340405.1	Pseudomonas phage Psa21
2053693	GCA_002957375.1	Pseudomonas phage Psp6
1983555	GCA_003226615.1	Pseudomonas phage PspYZU01
1983556	GCA_003226635.1	Pseudomonas phage PspYZU05
1983557	GCA_003226655.1	Pseudomonas phage PspYZU08
496396	GCA_000880375.1	Pseudomonas phage PT2
476523	GCA_002630325.1	Pseudomonas phage PT5
2483418	GCA_004367775.1	Pseudomonas phage RLP
1541890	GCA_002756395.1	Pseudomonas phage RWG

1752028	GCA_002607935.1	Pseudomonas phage S12-1
2364194	GCA_004367795.1	Pseudomonas phage SaPL
2249467	GCA_003307475.2	Pseudomonas phage SCYZ1
2249467	GCA_003307475.1	Pseudomonas phage SCYZ1
1729933	GCA_900007805.1	Pseudomonas phage shl2
2006671	GCA_002624985.1	Pseudomonas phage Skulduggery
2041215	GCA_002956005.1	Pseudomonas phage SL1
2041345	GCA_002956015.1	Pseudomonas phage SL2
2041214	GCA_002955995.1	Pseudomonas phage SL4
1772332	GCA_002608785.1	Pseudomonas phage SM1
1701866	GCA_002597465.1	Pseudomonas phage Smee
582382	GCA_000883495.1	Pseudomonas phage SN
2486665	GCA_003865735.1	Pseudomonas phage Spike
1453336	GCA_002630005.1	Pseudomonas phage SPM-1
2268590	GCA_003341055.1	Pseudomonas phage SRT6
2048978	GCA_002957175.1	Pseudomonas phage tabernarius
2060947	GCA_002957795.1	Pseudomonas phage TC6
1114179	GCA_000898175.1	Pseudomonas phage tf
1406974	GCA_000914475.1	Pseudomonas phage TL
1701867	GCA_002597485.1	Pseudomonas phage Triton
1235661	GCA_000900335.1	Pseudomonas phage UFV-P2
2048979	GCA_002957185.1	Pseudomonas phage uligo
2048979	GCA_002957185.2	Pseudomonas phage uligo
1873993	GCA_002757895.1	Pseudomonas phage UNO-SLW1
1873993	GCA_003991805.1	Pseudomonas phage UNO-SLW1
1874529	GCA_002757935.1	Pseudomonas phage UNO-SLW2
1874530	GCA_002757955.1	Pseudomonas phage UNO-SLW3
1874531	GCA_002757975.1	Pseudomonas phage UNO-SLW4
2563491	GCA_004400745.1	Pseudomonas phage vB_Pae_BR123a
2563492	GCA_004400945.1	Pseudomonas phage vB_Pae_BR133a
2563493	GCA_004400965.1	Pseudomonas phage vB_Pae_BR141a
2563494	GCA_004401445.1	Pseudomonas phage vB_Pae_BR141b
2563495	GCA_004400345.1	Pseudomonas phage vB_Pae_BR141c
2563496	GCA_004401865.1	Pseudomonas phage vB_Pae_BR141d
2563497	GCA_004400765.1	Pseudomonas phage vB_Pae_BR143a
2563498	GCA_004400985.1	Pseudomonas phage vB_Pae_BR144a
2563499	GCA_004400045.1	Pseudomonas phage vB_Pae_BR150a
2563500	GCA_004401365.1	Pseudomonas phage vB_Pae_BR153a
2563501	GCA_004401385.1	Pseudomonas phage vB_Pae_BR161a
2563502	GCA_004401765.1	Pseudomonas phage vB_Pae_BR161b
2563503	GCA_004400785.1	Pseudomonas phage vB_Pae_BR161c
2563504	GCA_004400125.1	Pseudomonas phage vB_Pae_BR177a
2563505	GCA_004401085.1	Pseudomonas phage vB_Pae_BR177b
2563506	GCA_004400805.1	Pseudomonas phage vB_Pae_BR178a
2563507	GCA_004400065.1	Pseudomonas phage vB_Pae_BR181a
2563508	GCA_004399925.1	Pseudomonas phage vB_Pae_BR197a
2563509	GCA_004400825.1	Pseudomonas phage vB_Pae_BR200a
2563510	GCA_004401005.1	Pseudomonas phage vB_Pae_BR201a
2563511	GCA_004400845.1	Pseudomonas phage vB_Pae_BR204a
2563512	GCA_004400145.1	Pseudomonas phage vB_Pae_BR204b

2563513	GCA_004401785.1	Pseudomonas phage vB_Pae_BR205a
2563514	GCA_004399945.1	Pseudomonas phage vB_Pae_BR208a
2563515	GCA_004400865.1	Pseudomonas phage vB_Pae_BR213a
2563516	GCA_004399905.1	Pseudomonas phage vB_Pae_BR228a
2563517	GCA_004400885.1	Pseudomonas phage vB_Pae_BR233a
2563518	GCA_004401405.1	Pseudomonas phage vB_Pae_BR243a
2563519	GCA_004401805.1	Pseudomonas phage vB_Pae_BR243b
2563520	GCA_004400165.1	Pseudomonas phage vB_Pae_BR293a
2563521	GCA_004400905.1	Pseudomonas phage vB_Pae_BR299a
2563522	GCA_004399965.1	Pseudomonas phage vB_Pae_BR313a
2563523	GCA_004401825.1	Pseudomonas phage vB_Pae_BR313b
2563524	GCA_004401425.1	Pseudomonas phage vB_Pae_BR313c
2563525	GCA_004400365.1	Pseudomonas phage vB_Pae_BR319a
2563526	GCA_004399985.1	Pseudomonas phage vB_Pae_BR319b
2563527	GCA_004400005.1	Pseudomonas phage vB_Pae_BR320a
2563528	GCA_004400925.1	Pseudomonas phage vB_Pae_BR322a
2563529	GCA_004400025.1	Pseudomonas phage vB_Pae_BR326a
2563530	GCA_004401845.1	Pseudomonas phage vB_Pae_BR327a
2563531	GCA_004400725.1	Pseudomonas phage vB_Pae_BR52a
2563532	GCA_004401745.1	Pseudomonas phage vB_Pae_BR52b
2563533	GCA_004400325.1	Pseudomonas phage vB_Pae_BR58a
2563534	GCA_004401885.1	Pseudomonas phage vB_Pae_BR58b
2563535	GCA_004401105.1	Pseudomonas phage vB_Pae_BR58c
2563536	GCA_004401245.1	Pseudomonas phage vB_Pae_CF118a
2563537	GCA_004401625.1	Pseudomonas phage vB_Pae_CF118b
2563538	GCA_004400245.1	Pseudomonas phage vB_Pae_CF118c
2563539	GCA_004401645.1	Pseudomonas phage vB_Pae_CF121a
2563540	GCA_004401265.1	Pseudomonas phage vB_Pae_CF121b
2563541	GCA_004400585.1	Pseudomonas phage vB_Pae_CF121c
2563542	GCA_004401285.1	Pseudomonas phage vB_Pae_CF124b
2563543	GCA_004400265.1	Pseudomonas phage vB_Pae_CF125a
2563544	GCA_004401965.1	Pseudomonas phage vB_Pae_CF126a
2563545	GCA_004400105.1	Pseudomonas phage vB_Pae_CF126b
2563546	GCA_004400605.1	Pseudomonas phage vB_Pae_CF127a
2563547	GCA_004400285.1	Pseudomonas phage vB_Pae_CF127b
2563548	GCA_004401665.1	Pseudomonas phage vB_Pae_CF136a
2563549	GCA_004401305.1	Pseudomonas phage vB_Pae_CF136b
2563550	GCA_004400625.1	Pseudomonas phage vB_Pae_CF140a
2563551	GCA_004400645.1	Pseudomonas phage vB_Pae_CF145a
2563552	GCA_004400665.1	Pseudomonas phage vB_Pae_CF165a
2563553	GCA_004401125.1	Pseudomonas phage vB_Pae_CF16a
2563554	GCA_004400305.1	Pseudomonas phage vB_Pae_CF177a
2563555	GCA_004401685.1	Pseudomonas phage vB_Pae_CF177b
2563556	GCA_004401325.1	Pseudomonas phage vB_Pae_CF177c
2563557	GCA_004400685.1	Pseudomonas phage vB_Pae_CF183a
2563558	GCA_004401705.1	Pseudomonas phage vB_Pae_CF183b
2563559	GCA_004400705.1	Pseudomonas phage vB_Pae_CF208a
2563560	GCA_004401345.1	Pseudomonas phage vB_Pae_CF213a
2563561	GCA_004401725.1	Pseudomonas phage vB_Pae_CF213b
2563562	GCA_004401145.1	Pseudomonas phage vB_Pae_CF23a

2563563	GCA_004401465.1	Pseudomonas phage vB_Pae_CF23b
2563564	GCA_004401905.1	Pseudomonas phage vB_Pae_CF24a
2563565	GCA_004400405.1	Pseudomonas phage vB_Pae_CF24b
2563566	GCA_004401485.1	Pseudomonas phage vB_Pae_CF28a
2563567	GCA_004400425.1	Pseudomonas phage vB_Pae_CF28b
2563568	GCA_004400445.1	Pseudomonas phage vB_Pae_CF34a
2563569	GCA_004400385.1	Pseudomonas phage vB_Pae_CF3a
2563570	GCA_004401045.1	Pseudomonas phage vB_Pae_CF52a
2563571	GCA_004400085.1	Pseudomonas phage vB_Pae_CF52b
2563572	GCA_004400465.1	Pseudomonas phage vB_Pae_CF53a
2563573	GCA_004400205.1	Pseudomonas phage vB_Pae_CF53b
2563574	GCA_004401505.1	Pseudomonas phage vB_Pae_CF53c
2563575	GCA_004400485.1	Pseudomonas phage vB_Pae_CF54a
2563576	GCA_004401985.1	Pseudomonas phage vB_Pae_CF55a
2563577	GCA_004400505.1	Pseudomonas phage vB_Pae_CF55b
2563578	GCA_004401165.1	Pseudomonas phage vB_Pae_CF57a
2563579	GCA_004401525.1	Pseudomonas phage vB_Pae_CF57b
2563580	GCA_004400185.1	Pseudomonas phage vB_Pae_CF5a
2563581	GCA_004400525.1	Pseudomonas phage vB_Pae_CF60a
2563582	GCA_004401925.1	Pseudomonas phage vB_Pae_CF63a
2563583	GCA_004401185.1	Pseudomonas phage vB_Pae_CF65a
2563584	GCA_004401545.1	Pseudomonas phage vB_Pae_CF65b
2563585	GCA_004400545.1	Pseudomonas phage vB_Pae_CF67a
2563586	GCA_004401945.1	Pseudomonas phage vB_Pae_CF69a
2563587	GCA_004401025.1	Pseudomonas phage vB_Pae_CF6a
2563588	GCA_004401565.1	Pseudomonas phage vB_Pae_CF74a
2563589	GCA_004401205.1	Pseudomonas phage vB_Pae_CF74b
2563590	GCA_004401065.1	Pseudomonas phage vB_Pae_CF77a
2563591	GCA_004401585.1	Pseudomonas phage vB_Pae_CF77b
2563592	GCA_004400225.1	Pseudomonas phage vB_Pae_CF78a
2563593	GCA_004400565.1	Pseudomonas phage vB_Pae_CF79a
2563594	GCA_004401225.1	Pseudomonas phage vB_Pae_CF81a
2563595	GCA_004401605.1	Pseudomonas phage vB_Pae_CF81b
1542090	GCA_001501055.1	Pseudomonas phage vB_Pae_PS44
1542091	GCA_002602085.1	Pseudomonas phage vB_Pae_PS9N
1868842	GCA_002611165.1	Pseudomonas phage vB_Pae1396P-5
1868828	GCA_002611125.1	Pseudomonas phage vB_Pae436M-8
1868829	GCA_002611145.1	Pseudomonas phage vB_Pae575P-3
1141526	GCA_000897095.1	Pseudomonas phage vB_Pae-Kakheti25
1548917	GCA_000955335.1	Pseudomonas phage vB_PaeM_C1-14_Ab28
1548900	GCA_002987395.1	Pseudomonas phage vB_PaeM_C2-10_Ab02
1548903	GCA_003147005.1	Pseudomonas phage vB_PaeM_C2-10_Ab08
1231048	GCA_000902875.1	Pseudomonas phage vB_PaeM_C2-10_Ab1
1548914	GCA_003147145.1	Pseudomonas phage vB_PaeM_C2-10_Ab10
1548915	GCA_003147165.1	Pseudomonas phage vB_PaeM_C2-10_Ab15
1675014	GCA_002606945.1	Pseudomonas phage vB_PaeM_CEB_DP1
2034347	GCA_002955985.1	Pseudomonas phage vB_PaeM_E215
2034346	GCA_002955975.1	Pseudomonas phage vB_PaeM_E217
2500798	GCA_004015925.1	Pseudomonas phage vB_PaeM_fHoPae01
1983539	GCA_002623605.1	Pseudomonas phage vB_PaeM_G1

2488595	GCA_003865855.1	Pseudomonas phage vB_PaeM_LCK69
2099648	GCA_002997435.1	Pseudomonas phage vB_PaeM_LS1
1639815	GCA_001743695.1	Pseudomonas phage vB_PaeM_MAG1
1548901	GCA_000955355.1	Pseudomonas phage vB_PaeM_PAO1_Ab03
1548909	GCA_003147065.1	Pseudomonas phage vB_PaeM_PAO1_Ab04
1548910	GCA_003147085.1	Pseudomonas phage vB_PaeM_PAO1_Ab06
1548911	GCA_003147105.1	Pseudomonas phage vB_PaeM_PAO1_Ab11
1548904	GCA_003147025.1	Pseudomonas phage vB_PaeM_PAO1_Ab17
1548907	GCA_000954955.1	Pseudomonas phage vB_PaeM_PAO1_Ab27
1548916	GCA_003147185.1	Pseudomonas phage vB_PaeM_PAO1_Ab29
1542092	GCA_001500395.1	Pseudomonas phage vB_PaeM_PS24
2508865	GCA_004138895.1	Pseudomonas phage vB_PaeM_SCUT-S1
2508866	GCA_004138915.1	Pseudomonas phage vB_PaeM_SCUT-S2
2161784	GCA_003059785.1	Pseudomonas phage vB_PaeP_130_113
1229675	GCA_003047875.1	Pseudomonas phage vB_PaeP_C1-14_Or
1476391	GCA_000918275.1	Pseudomonas phage vB_PaeP_C2-10_Ab09
1548906	GCA_000954995.1	Pseudomonas phage vB_PaeP_C2-10_Ab22
2034344	GCA_002955955.1	Pseudomonas phage vB_PaeP_DEV
2034343	GCA_002955945.1	Pseudomonas phage vB_PaeP_E220
1639814	GCA_001744375.1	Pseudomonas phage vB_PaeP_MAG4
1234701	GCA_000902175.1	Pseudomonas phage vB_PaeP_p2-10_Or1
1548908	GCA_003147045.1	Pseudomonas phage vB_PaeP_PAO1_1-15pyo
1548902	GCA_000954635.1	Pseudomonas phage vB_PaeP_PAO1_Ab05
1527525	GCA_001041515.1	Pseudomonas phage vB_PaeP_PPA-ABTNL
2034342	GCA_002955935.1	Pseudomonas phage vB_PaeP_PYO2
2530025	GCA_004551535.1	Pseudomonas phage vB_PaeP_TF17
1449437	GCA_000915555.1	Pseudomonas phage vB_PaeP_Tr60_Ab31
2099649	GCA_002997385.1	Pseudomonas phage vB_PaeS_C1
1548905	GCA_000954275.1	Pseudomonas phage vB_PaeS_PAO1_Ab18
1548912	GCA_002988115.1	Pseudomonas phage vB_PaeS_PAO1_Ab19
1548913	GCA_003147125.1	Pseudomonas phage vB_PaeS_PAO1_Ab20
1548918	GCA_000954615.1	Pseudomonas phage vB_PaeS_PAO1_Ab30
1928621	GCA_900327825.1	Pseudomonas phage vB_PaeS_PAO1_HW12
1926503	GCA_900095755.1	Pseudomonas phage vB_PaeS_PcyII-40_PfII40a
1743016	GCA_001470435.1	Pseudomonas phage vB_PaeS_PM105
2034345	GCA_002955965.1	Pseudomonas phage vB_PaeS_S218
1476390	GCA_000923035.1	Pseudomonas phage vB_PaeS_SCH_Ab26
2382122	GCA_003958865.1	Pseudomonas phage vB_PaeS_SCUT-S3
2488702	GCA_003958885.1	Pseudomonas phage vB_PaeS_SCUT-S4
1141525	GCA_000898095.1	Pseudomonas phage vB_Pae-TbilisiM32
1141525	GCA_002632545.1	Pseudomonas phage vB_Pae-TbilisiM32
1777065	GCA_001736355.1	Pseudomonas phage vB_PsyM_KIL1
1777066	GCA_002757195.1	Pseudomonas phage vB_PsyM_KIL2
1777067	GCA_002757215.1	Pseudomonas phage vB_PsyM_KIL3
1777068	GCA_002757235.1	Pseudomonas phage vB_PsyM_KIL3b
1777069	GCA_002757255.1	Pseudomonas phage vB_PsyM_KIL4
1777070	GCA_002757275.1	Pseudomonas phage vB_PsyM_KIL5
1729937	GCA_001551465.1	Pseudomonas phage VCM
2048980	GCA_002957195.1	Pseudomonas phage ventosus
1852562	GCA_002610045.1	Pseudomonas phage VSW-3

2041486	GCA_002744115.1	Pseudomonas phage VW-6B
2041487	GCA_002744135.1	Pseudomonas phage VW-6S
1636189	GCA_001551385.1	Pseudomonas phage YH30
1566995	GCA_001041435.1	Pseudomonas phage YH6
1191380	GCA_002602325.1	Pseudomonas phage YMC/01/01/P52_PAE_BP
1755689	GCA_001470255.1	Pseudomonas phage YMC11/02/R656
1777063	GCA_001736815.1	Pseudomonas phage YMC11/06/C171_PPU_BP
1777064	GCA_001737035.1	Pseudomonas phage YMC11/07/P54_PAE_BP
1755690	GCA_002630845.1	Pseudomonas phage YMC11/11/R1836
2283028	GCA_003423145.1	Pseudomonas phage YMC12/01/R24
2283027	GCA_003423105.1	Pseudomonas phage YMC12/01/R960
2036050	GCA_002743715.1	Pseudomonas phage YS35
1622114	GCA_002605485.1	Pseudomonas phage ZC01
1622115	GCA_002605505.1	Pseudomonas phage ZC03
1622116	GCA_002605525.1	Pseudomonas phage ZC08
1916099	GCA_002615585.1	Pseudomonas phage Zigelbrucke
347326	GCA_000866225.1	Pseudomonas virus 119X
31535	GCA_000845025.2	Pseudomonas virus D3
10708	GCA_000842485.1	Pseudomonas virus D3112
389469	GCA_000867885.1	Pseudomonas virus DMS3
280701	GCA_000859545.1	Pseudomonas virus F116
1093100	GCA_002601905.1	Pseudomonas virus FHA0480
1273707	GCA_002603045.1	Pseudomonas virus H66
1981933	GCA_002622685.1	Pseudomonas virus KNP
1091556	GCA_001040795.1	Pseudomonas virus LPB1
484895	GCA_000879735.1	Pseudomonas virus LUZ24
347328	GCA_000865485.1	Pseudomonas virus M6
397353	GCA_000874305.1	Pseudomonas virus MP22
505291	GCA_000883415.1	Pseudomonas virus MP29
505292	GCA_000882495.1	Pseudomonas virus MP38
937835	GCA_000917475.1	Pseudomonas virus PA1KOR
188350	GCA_000840805.1	Pseudomonas virus PaP3
2011081	GCA_000847025.1	Pseudomonas virus Pf1
2011081	GCA_003719275.1	Pseudomonas virus Pf1
2175295	GCA_002922435.1	Pseudomonas virus Pf1 ERZ-2017
10879	GCA_000852125.1	Pseudomonas virus phi6
10879	GCA_002966165.1	Pseudomonas virus phi6
10879	GCA_002966185.1	Pseudomonas virus phi6
10879	GCA_002966175.1	Pseudomonas virus phi6
35343	GCA_000844605.1	Pseudomonas virus phiCTX
35343	GCA_002630025.1	Pseudomonas virus phiCTX
169683	GCA_000842965.1	Pseudomonas virus phiKZ
1129146	GCA_000895295.1	Pseudomonas virus PMG1
1981932	GCA_002622665.1	Pseudomonas virus WRT
462590	GCA_000871365.1	Pseudomonas virus Yua
185637	GCA_000902575.1	Pseudoplusia includens densovirus
1592335	GCA_000928515.1	Pseudoplusia includens SNPV IE
50294	GCA_000840765.1	Psittacid alphaherpesvirus 1
1580497	GCA_000929035.1	Psittacine adenovirus 3
2169709	GCA_003032855.1	Psittacine aviadenovirus B

754048	GCA_000905395.1	Psychrobacter phage pOW20-A
1071177	GCA_000916615.1	Psychrobacter phage Psymv2
1926633	GCA_002815135.1	Pteromalus puparum negative-strand RNA virus 1
1891773	GCA_000903015.1	Pteronotus davyi polyomavirus 1
1606501	GCA_001430635.1	Pteronotus parnellii polyomavirus 1
1873698	GCA_001695465.1	Pteropox virus
1985404	GCA_002825945.1	Pteropus associated gemycircularvirus 10
1985397	GCA_002825805.1	Pteropus associated gemycircularvirus 3
1985398	GCA_002825825.1	Pteropus associated gemycircularvirus 4
1985399	GCA_002825845.1	Pteropus associated gemycircularvirus 5
1985400	GCA_002825865.1	Pteropus associated gemycircularvirus 6
1985401	GCA_002825885.1	Pteropus associated gemycircularvirus 7
1985402	GCA_002825905.1	Pteropus associated gemycircularvirus 8
1985403	GCA_002825925.1	Pteropus associated gemycircularvirus 9
1747360	GCA_003033135.1	Pudu puda papillomavirus 1
1479613	GCA_000926695.1	Puerto Almendras virus
253454	GCA_002829605.1	Pulau reovirus
323365	GCA_003177835.1	Puma concolor papillomavirus type 1
2169894	GCA_003032735.1	Puma feline foamy virus
32615	GCA_002829785.1	Puma lentivirus 14
420684	GCA_000874505.1	Pumpkin yellow mosaic Malaysia virus
948071	GCA_000911675.1	Puniceispirillum phage HMO-2011
11587	GCA_001021295.1	Punta Toro virus
11587	GCA_002815115.1	Punta Toro virus
1980486	GCA_000854405.1	Puumala orthohantavirus
10576	GCA_003180495.1	Pygmy chimpanzee papillomavirus type 1
1480065	GCA_000920595.1	Pygoscelis adeliae papillomavirus 1
2045113	GCA_003180115.1	Pygoscelis adeliae papillomavirus 2
1805492	GCA_001885445.1	Pyrobaculum filamentous virus 1
270161	GCA_000843585.1	Pyrobaculum spherical virus
425386	GCA_000871785.1	Pyrococcus abyssi virus 1
1891751	GCA_000864605.1	Pyrrhula pyrrhula polyomavirus 1
2083275	GCA_004117215.1	Pythium nunn virus 1
2137352	GCA_003729235.1	Pythium polare bunya-like RNA virus 1
2137353	GCA_004130755.1	Pythium polare RNA virus 1
2137354	GCA_004130775.1	Pythium polare RNA virus 2
1980527	GCA_002145525.1	Qalyub orthonairovirus
1961665	GCA_002008515.1	Qinghai Himalayan marmot astrovirus 1
1961666	GCA_002008655.1	Qinghai Himalayan marmot astrovirus 2
1516115	GCA_003114135.1	Qinghai Lake virophage
1089138	GCA_000895635.1	Quail picornavirus QPV1/HUN/2010
643132	GCA_000882915.1	Quang Binh virus
1841195	GCA_002117655.1	Quezon virus
1161132	GCA_000926475.1	Rabbit astrovirus TN/2208/2010
576948	GCA_000882575.1	Rabbit calicivirus Australia 1 MIC-07
1160968	GCA_000896935.1	Rabbit coronavirus HKU14
10271	GCA_000847965.1	Rabbit fibroma virus
314536	GCA_000861285.1	Rabbit hemorrhagic disease virus-FRG
1756444	GCA_001461125.1	Rabbit picornavirus
303317	GCA_000867805.1	Rabbit vesivirus

11292	GCA_000859625.1	Rabies lyssavirus
11292	GCA_900231335.1	Rabies lyssavirus
11292	GCA_900327645.1	Rabies lyssavirus
11292	GCA_900327715.1	Rabies lyssavirus
11292	GCA_900327735.1	Rabies lyssavirus
11292	GCA_900327725.1	Rabies lyssavirus
11292	GCA_900327665.1	Rabies lyssavirus
11292	GCA_900327655.1	Rabies lyssavirus
11292	GCA_900327685.1	Rabies lyssavirus
11292	GCA_900327615.1	Rabies lyssavirus
11292	GCA_900327635.1	Rabies lyssavirus
11292	GCA_900327695.1	Rabies lyssavirus
11292	GCA_900327755.1	Rabies lyssavirus
11292	GCA_900079035.1	Rabies lyssavirus
11292	GCA_900327625.1	Rabies lyssavirus
11292	GCA_900079025.1	Rabies lyssavirus
11292	GCA_900327675.1	Rabies lyssavirus
11292	GCA_900079055.1	Rabies lyssavirus
11292	GCA_900327705.1	Rabies lyssavirus
11292	GCA_900079015.1	Rabies lyssavirus
11292	GCA_900079005.1	Rabies lyssavirus
11292	GCA_900231215.1	Rabies lyssavirus
11292	GCA_900231345.1	Rabies lyssavirus
11292	GCA_900231235.1	Rabies lyssavirus
11292	GCA_900231365.1	Rabies lyssavirus
11292	GCA_900231295.1	Rabies lyssavirus
11292	GCA_900231355.1	Rabies lyssavirus
11292	GCA_900231375.1	Rabies lyssavirus
11292	GCA_900231315.1	Rabies lyssavirus
11292	GCA_900231395.1	Rabies lyssavirus
11292	GCA_900231195.1	Rabies lyssavirus
11292	GCA_900231325.1	Rabies lyssavirus
11292	GCA_900231385.1	Rabies lyssavirus
11292	GCA_900231185.1	Rabies lyssavirus
11292	GCA_900231175.1	Rabies lyssavirus
11292	GCA_900231285.1	Rabies lyssavirus
11292	GCA_900079065.1	Rabies lyssavirus
11292	GCA_900231305.1	Rabies lyssavirus
11292	GCA_900231255.1	Rabies lyssavirus
11292	GCA_900231245.1	Rabies lyssavirus
11292	GCA_900231425.1	Rabies lyssavirus
11292	GCA_900231265.1	Rabies lyssavirus
11292	GCA_900231225.1	Rabies lyssavirus
11292	GCA_900231415.1	Rabies lyssavirus
11292	GCA_900231275.1	Rabies lyssavirus
11292	GCA_900231405.1	Rabies lyssavirus
11292	GCA_900231205.1	Rabies lyssavirus
11292	GCA_900176675.1	Rabies lyssavirus
11292	GCA_900079045.1	Rabies lyssavirus
1513315	GCA_000929935.1	Raccoon dog amdovirus

1219896	GCA_000920375.1	Raccoon polyomavirus
1978920	GCA_002114005.1	Raccoon-associated polyomavirus 2
10256	GCA_001029045.1	Raccoonpox virus
1972566	GCA_002816075.1	Radi vesiculovirus
662596	GCA_000878995.1	Radish leaf curl betasatellite
435646	GCA_000872445.1	Radish leaf curl virus
435646	GCA_002823245.1	Radish leaf curl virus
328061	GCA_000879335.1	Radish mosaic virus
1911110	GCA_003728895.1	Rainier virus
1460070	GCA_000915515.1	Ralstonia phage 1 NP-2014
2041493	GCA_002956085.1	Ralstonia phage DU_RP_I
2022331	GCA_002627225.1	Ralstonia phage DU_RP_II
2282904	GCA_003354205.1	Ralstonia phage GP4
247080	GCA_000841525.1	Ralstonia phage p12J
926543	GCA_000892535.1	Ralstonia phage PE226
1597967	GCA_002605405.1	Ralstonia phage phiITL-1
1980924	GCA_000879455.1	Ralstonia phage phiRSL1
2201420	GCA_003368625.1	Ralstonia phage phiRSP
1923889	GCA_002617345.1	Ralstonia phage RP12
1923890	GCA_002617365.1	Ralstonia phage RP31
2041351	GCA_003288515.1	Ralstonia phage RPSC1
1483485	GCA_001551265.1	Ralstonia phage RS138
1852598	GCA_002610105.1	Ralstonia phage Rs551
1505528	GCA_000924935.1	Ralstonia phage RS603
1497850	GCA_002602125.1	Ralstonia phage RS611
551790	GCA_000883015.1	Ralstonia phage RSB1
913183	GCA_000918515.1	Ralstonia phage RSB2
1402875	GCA_000911455.1	Ralstonia phage RSB3
1689679	GCA_001500875.1	Ralstonia phage RSF1
1481785	GCA_001503995.1	Ralstonia phage RSJ2
1538364	GCA_001503875.1	Ralstonia phage RSJ5
1417599	GCA_000913935.1	Ralstonia phage RSK1
1585840	GCA_001503055.1	Ralstonia phage RSL2
384359	GCA_000870245.1	Ralstonia phage RSM1
520962	GCA_000883215.1	Ralstonia phage RSM3
1530086	GCA_002630405.1	Ralstonia phage RSMSuper
2070026	GCA_003031025.1	Ralstonia phage RsoP1EGY
2060091	GCA_002990175.1	Ralstonia phage RsoP1IDN
1785960	GCA_001736715.1	Ralstonia phage RSP15
1958965	GCA_002619365.1	Ralstonia phage RS-PI-1
1932892	GCA_002618065.1	Ralstonia phage RS-PII-1
1150992	GCA_000902595.1	Ralstonia phage RSS0
384360	GCA_000868665.1	Ralstonia phage RSS1
1354496	GCA_000910575.1	Ralstonia phage RSS20
1352227	GCA_000908095.1	Ralstonia phage RSS30
1685501	GCA_002607145.1	Ralstonia phage RSS-TH1
1530085	GCA_000924355.1	Ralstonia phage RSY1
1932865	GCA_002617905.1	Ralstonia virus phiAp1
406340	GCA_000873125.1	Ralstonia virus RSA1
2053972	GCA_002957465.1	Ralstonia virus RSIBR1

486809	GCA_000879415.1	Ramie mosaic virus
1874886	GCA_001698395.1	Ramie mosaic Yunnan virus
2185281	GCA_004134005.1	Rana hepevirus
1887314	GCA_001717415.1	Ranavirus maximus
1370094	GCA_000911755.1	Rangifer tarandus papillomavirus 2
85655	GCA_000869925.1	Ranid herpesvirus 1 (Lucke tumor herpesvirus)
389214	GCA_000869245.1	Ranid herpesvirus 2
1987509	GCA_002158775.1	Ranid herpesvirus 3
2053702	GCA_002957425.1	Raoultella phage Ro1
348449	GCA_000868245.1	Raphanus sativus cryptic virus 1
351495	GCA_000872665.1	Raphanus sativus cryptic virus 2
580254	GCA_000881935.1	Raphanus sativus cryptic virus 3
1520002	GCA_000893535.1	Raptor adenovirus 1
1930508	GCA_002374995.1	Rasavirus sp.
1930508	GCA_003727615.1	Rasavirus sp.
1930508	GCA_003727695.1	Rasavirus sp.
1930508	GCA_003727715.1	Rasavirus sp.
1930508	GCA_003727675.1	Rasavirus sp.
1930508	GCA_003727775.1	Rasavirus sp.
1930508	GCA_003727795.1	Rasavirus sp.
1930508	GCA_003727855.1	Rasavirus sp.
1930508	GCA_003727635.1	Rasavirus sp.
1930508	GCA_003727655.1	Rasavirus sp.
12451	GCA_000851365.1	Raspberry bushy dwarf virus
907191	GCA_000889355.1	Raspberry latent virus
1980431	GCA_001580315.1	Raspberry leaf blotch emaravirus
326941	GCA_000870265.1	Raspberry leaf mottle virus
12809	GCA_000854425.1	Raspberry ringspot virus
1734441	GCA_001501455.1	Rat arterivirus 1
1734441	GCA_001963835.1	Rat arterivirus 1
2170126	GCA_001273705.1	Rat associated porprismacovirus 1
1788315	GCA_001560965.1	Rat bocavirus
1763507	GCA_001465505.1	Rat bufavirus SY-2015
502102	GCA_000886515.1	Rat coronavirus Parker
79700	GCA_000844625.1	Rat cytomegalovirus Maastricht (Rat cytomegalovirus)
74581	GCA_002827485.1	Rat parvovirus 1
1699316	GCA_003967955.1	Rat stool-associated circular ssDNA virus
1699316	GCA_003967975.1	Rat stool-associated circular ssDNA virus
1909694	GCA_002614405.1	Rathayibacter phage NCPPB3778
1123754	GCA_000895695.1	Rattail cactus necrosis-associated virus
664730	GCA_003178555.1	Rattus norvegicus papillomavirus 1
1036965	GCA_003179075.1	Rattus norvegicus papillomavirus 2
1756445	GCA_001461525.1	Rattus norvegicus papillomavirus 3
1679933	GCA_001184865.1	Rattus norvegicus polyomavirus 1
1919247	GCA_001891055.1	Rattus norvegicus polyomavirus 2
1919247	GCA_003033355.1	Rattus norvegicus polyomavirus 2
345250	GCA_000869425.1	Raven circovirus
378809	GCA_000943645.1	Ravn virus - Ravn, Kenya, 1987
1405807	GCA_000913675.1	Razdan virus
11834	GCA_000873665.1	RD114 retrovirus

1408894	GCA_000911175.1	Red clover cryptic virus 1
1323524	GCA_000904755.1	Red clover cryptic virus 2
12262	GCA_000860425.1	Red clover mottle virus
12267	GCA_000852165.1	Red clover necrotic mosaic virus
2058094	GCA_004117435.1	Red clover nepovirus A
1714362	GCA_001461445.1	Red clover powdery mildew-associated totivirus 1
1714363	GCA_001461165.1	Red clover powdery mildew-associated totivirus 2
1714364	GCA_001461285.1	Red clover powdery mildew-associated totivirus 3
1714366	GCA_001461585.1	Red clover powdery mildew-associated totivirus 5
1714367	GCA_001461425.1	Red clover powdery mildew-associated totivirus 6
1714368	GCA_001461105.1	Red clover powdery mildew-associated totivirus 7
1714370	GCA_001461265.1	Red clover powdery mildew-associated totivirus 9
590403	GCA_000881135.1	Red clover vein mosaic virus
2079601	GCA_004130255.1	Red-crowned crane parvovirus
2079601	GCA_004130275.1	Red-crowned crane parvovirus
1959287	GCA_002271005.1	Reed chlorotic stripe virus
425279	GCA_000870525.1	Rehmannia mosaic virus
2316740	GCA_004133525.1	Rehmannia virus 1
10569	GCA_003177635.1	Reindeer papillomavirus
122203	GCA_000853985.1	Reptilian ferlavirus
226613	GCA_000919495.1	Reptilian orthoreovirus
12814	GCA_000856445.1	Respiratory syncytial virus
186539	GCA_000854085.1	Reston ebolavirus
11636	GCA_000858025.1	Reticuloendotheliosis virus
103930	GCA_000844865.1	Rhesus cytomegalovirus strain 68-1 (Rhesus cytomegalovir
2170199	GCA_003032795.1	Rhesus macaque simian foamy virus
2218594	GCA_004133465.1	Rhimavirus A
2004965	GCA_002826065.1	Rhinolophus associated gemykibivirus 1
2004966	GCA_002826085.1	Rhinolophus associated gemykibivirus 2
693998	GCA_000875645.1	Rhinolophus bat coronavirus HKU2
2029303	GCA_004130635.1	Rhinolophus blasii polyomavirus 2
1195373	GCA_002819485.1	Rhinolophus ferrumequinum circovirus 1
1464074	GCA_002827105.1	Rhinolophus ferrumequinum papillomavirus 1
2054179	GCA_004130735.1	Rhinolophus gammaherpesvirus 1
1904410	GCA_002037695.1	Rhinolophus hildebrandtii polyomavirus 1
2053079	GCA_004131925.1	Rhinolophus pusillus bocaparvovirus 1
2053080	GCA_004131945.1	Rhinolophus pusillus bocaparvovirus 2
2029304	GCA_004130655.1	Rhinolophus simulator polyomavirus 1
2029305	GCA_004130675.1	Rhinolophus simulator polyomavirus 2
2029306	GCA_004130695.1	Rhinolophus simulator polyomavirus 3
1911555	GCA_001858075.1	Rhinolophus sinicus bocaparvovirus
12131	GCA_000861265.1	Rhinovirus B14
463676	GCA_000872325.1	Rhinovirus C
10704	GCA_000881375.1	Rhizobium phage 16-3
1220601	GCA_002602585.1	Rhizobium phage RHEph01
1220602	GCA_002755275.1	Rhizobium phage RHEph02
1220603	GCA_002755295.1	Rhizobium phage RHEph03
1220604	GCA_002617285.1	Rhizobium phage RHEph04
1220605	GCA_002755315.1	Rhizobium phage RHEph05
1220714	GCA_001040775.1	Rhizobium phage RHEph06

1220715	GCA_002755335.1	Rhizobium phage RHEph08
1220716	GCA_002755355.1	Rhizobium phage RHEph09
1220717	GCA_002080335.1	Rhizobium phage RHEph10
929833	GCA_000910255.1	Rhizobium phage RR1-A
929834	GCA_000908795.1	Rhizobium phage RR1-B
1458697	GCA_000917175.1	Rhizobium phage vB_RglS_P106B
1527770	GCA_000925855.1	Rhizobium phage vB_RleM_P10VF
1498228	GCA_000927395.1	Rhizobium phage vB_RleM_PPF1
1414737	GCA_000916995.1	Rhizobium phage vB_RleS_L338C
1408133	GCA_000912835.1	Rhizoctonia cerealis alphaendornavirus 1
1592828	GCA_000989075.1	Rhizoctonia fumigata mycovirus
2421278	GCA_004132705.1	Rhizoctonia mitovirus 1
1837089	GCA_001634535.1	Rhizoctonia oryzae-sativae mitovirus 1
1411681	GCA_000918495.1	Rhizoctonia solani dsRNA virus 2
1825688	GCA_002158835.1	Rhizoctonia solani dsRNA virus 3
2045504	GCA_004117335.1	Rhizoctonia solani dsRNA virus 4
2162642	GCA_004130855.1	Rhizoctonia solani endornavirus 1
1871631	GCA_001695445.1	Rhizoctonia solani flexivirus 1
1678208	GCA_001184805.1	Rhizoctonia solani RNA virus HN008
46618	GCA_000853025.1	Rhizoctonia solani virus 717
2320183	GCA_004134525.1	Rhizophagus diaphanum mitovirus 1
2320184	GCA_004134505.1	Rhizophagus diaphanum mitovirus 2
2320186	GCA_004134545.1	Rhizophagus irregularis mitovirus 1
758865	GCA_003956665.1	Rhizophagus sp. RF1 medium virus
758866	GCA_004128255.1	Rhizophagus sp. RF1 mitovirus
359987	GCA_000897675.1	Rhizosolenia setigera RNA virus 01
754055	GCA_000905415.1	Rhodobacter phage RC1
1105286	GCA_000896475.1	Rhodobacter phage RcapMu
1131316	GCA_000904295.1	Rhodobacter phage RcapNL
1662333	GCA_002756615.1	Rhodobacter phage RcCronus
1662332	GCA_001501915.1	Rhodobacter phage RcRhea
1698423	GCA_002756895.1	Rhodobacter phage RcSaxon
1662331	GCA_002623345.1	Rhodobacter phage RcSpartan
1662330	GCA_001551025.1	Rhodobacter phage RcTitan
2015826	GCA_002625785.1	Rhodococcus phage Alatin
2201446	GCA_003307615.1	Rhodococcus phage Alpacados
1955425	GCA_002619125.1	Rhodococcus phage AngryOrchard
2015827	GCA_002625805.1	Rhodococcus phage AppleCloud
2530158	GCA_004338695.1	Rhodococcus phage Belenaria
1955426	GCA_002619145.1	Rhodococcus phage BobbyDazzler
2024254	GCA_002628165.1	Rhodococcus phage Bonanza
2201447	GCA_003307655.1	Rhodococcus phage Bradshaw
2201448	GCA_003307675.1	Rhodococcus phage Bryce
1887657	GCA_002612165.1	Rhodococcus phage ChewyVIII
1701851	GCA_001470855.1	Rhodococcus phage CosmicSans
1007869	GCA_000907535.1	Rhodococcus phage E3
2201449	GCA_003307735.1	Rhodococcus phage Erik
2530159	GCA_004338475.1	Rhodococcus phage Espica
2094144	GCA_003014285.1	Rhodococcus phage Finch
2201450	GCA_003307775.1	Rhodococcus phage Gollum

2182359	GCA_003183125.1	Rhodococcus phage Grayson
1897551	GCA_002613545.1	Rhodococcus phage Harlequin
2015828	GCA_002625825.1	Rhodococcus phage Hiro
2182360	GCA_003182985.1	Rhodococcus phage Jace
2015812	GCA_002625525.1	Rhodococcus phage Jester
2015829	GCA_002625845.1	Rhodococcus phage Krishelle
1755671	GCA_002757155.1	Rhodococcus phage Lillie
2015830	GCA_002625865.1	Rhodococcus phage Naiad
2201451	GCA_003307915.1	Rhodococcus phage Nancinator
1897541	GCA_002613345.1	Rhodococcus phage Natosaleda
1897441	GCA_002612485.1	Rhodococcus phage Partridge
2182361	GCA_003183045.1	Rhodococcus phage Peregrin
2201452	GCA_003307975.1	Rhodococcus phage Phrankenstein
2201453	GCA_003308015.1	Rhodococcus phage Rasputin
1109712	GCA_000895855.1	Rhodococcus phage REQ1
1109713	GCA_000895215.1	Rhodococcus phage REQ2
1109714	GCA_000894255.1	Rhodococcus phage REQ3
691966	GCA_000920175.1	Rhodococcus phage ReqiDocB7
691965	GCA_000918715.1	Rhodococcus phage ReqiPepy6
691963	GCA_000918695.1	Rhodococcus phage ReqiPine5
691964	GCA_000920215.1	Rhodococcus phage ReqiPoco6
1109715	GCA_000896695.1	Rhodococcus phage RER2
2015831	GCA_002625885.1	Rhodococcus phage RexFury
1700839	GCA_002756935.1	Rhodococcus phage Rhodalysa
1109717	GCA_000895835.1	Rhodococcus phage RRH1
2234034	GCA_003308355.1	Rhodococcus phage Shuman
2015832	GCA_002625905.1	Rhodococcus phage StCroix
2201454	GCA_003308075.1	Rhodococcus phage Swann
2201455	GCA_003308095.1	Rhodococcus phage Takoda
1975614	GCA_002622305.1	Rhodococcus phage Toil
2027905	GCA_002743655.1	Rhodococcus phage Trina
1747281	GCA_002757035.1	Rhodococcus phage TWAMP
2201445	GCA_003308175.1	Rhodococcus phage UhSalsa
1897437	GCA_002612405.1	Rhodococcus phage Weasels2
1897442	GCA_002612505.1	Rhodococcus phage Yogi
2024280	GCA_002628405.1	Rhodococcus phage Yoncess
1109716	GCA_000894235.1	Rhodococcus virus RGL3
878260	GCA_000887615.1	Rhododendron virus A
1636270	GCA_001551705.1	Rhodoferax phage P26218
148943	GCA_000843805.1	Rhodothermus phage RM378
754056	GCA_000906895.1	Rhodovulum phage RS1
1873452	GCA_001743855.1	Rhodovulum phage vB_RhkS_P1
66834	GCA_000849085.1	Rhopalosiphum padi virus
2175117	GCA_004132125.1	Rhynchobatus djiddensis adomavirus 1
2170102	GCA_000928315.1	Rhynchobatus djiddensis polyomavirus 1
889202	GCA_002867595.1	Rhynchosia golden mosaic Havana virus-[Cuba:Havana:28:
1513519	GCA_002867615.1	Rhynchosia golden mosaic Sinaloa virus
117198	GCA_000840505.1	Rhynchosia golden mosaic virus
117198	GCA_003032355.1	Rhynchosia golden mosaic virus
457286	GCA_000881175.1	Rhynchosia golden mosaic Yucatan virus

714308	GCA_000891055.1	Rhynchosia mild mosaic virus
889203	GCA_002867635.1	Rhynchosia rugose golden mosaic virus-[Cuba:Camaguey:1
2010325	GCA_002830185.1	Rhynchosia yellow mosaic betasatellite
935473	GCA_000887955.1	Rhynchosia yellow mosaic India virus
529680	GCA_002986835.1	Rhynchosia yellow mosaic virus
1569057	GCA_004130965.1	Ribes americanum virus A
51680	GCA_000854645.1	Ribgrass mosaic virus
10990	GCA_000852945.1	Rice black streaked dwarf virus
10991	GCA_000850725.1	Rice dwarf virus
10986	GCA_000870625.1	Rice gall dwarf virus
66266	GCA_000847645.1	Rice grassy stunt tenuivirus
12332	GCA_002890455.1	Rice hoja blanca tenuivirus
2012856	GCA_004130515.1	Rice latent virus 1
2012857	GCA_004130535.1	Rice latent virus 2
59500	GCA_001430675.1	Rice necrosis mosaic virus
42475	GCA_000852965.1	Rice ragged stunt virus
1931356	GCA_004129795.1	Rice stripe mosaic virus
373373	GCA_002867285.1	Rice stripe necrosis virus
12331	GCA_000851385.1	Rice stripe tenuivirus
10654	GCA_000849605.1	Rice tungro bacilliform virus
35287	GCA_000860625.1	Rice tungro spherical virus
2021212	GCA_002271105.1	Rice virus A
31744	GCA_000863085.1	Rice yellow mottle virus
31744	GCA_002834045.1	Rice yellow mottle virus
195056	GCA_000839085.1	Rice yellow mottle virus satellite
59380	GCA_000850705.1	Rice yellow stunt nucleorhabdovirus
936152	GCA_000901735.1	Riemerella phage RAP44
11588	GCA_000847345.1	Rift Valley fever virus
11242	GCA_000856645.1	Rinderpest virus (strain Kabete O)
64285	GCA_000862425.1	Rio Bravo virus
1972687	GCA_002366105.1	Rio Chico almendravirus
332097	GCA_002829925.1	Rio Negro virus
1912234	GCA_001866915.1	Riptortus pedestris virus-1
1803263	GCA_004129675.1	Riverside virus 1
380435	GCA_002146005.1	Rochambeau virus
64315	GCA_004128575.1	Rocio virus
1001080	GCA_002830685.1	Rockport virus
1806636	GCA_004130335.1	Rodent arterivirus
1914442	GCA_002889895.1	Rodent astrovirus
1987141	GCA_002816965.1	Rodent hepatovirus CIV459Lopsik2004
1987142	GCA_002817005.1	Rodent hepatovirus KEF121Sigmas2012
1987140	GCA_001444005.1	Rodent hepatovirus RMU101637Micarv2010
2050020	GCA_004130355.1	Rodent papillomavirus
1074214	GCA_003726175.1	Rodent stool-associated circular genome virus
1074214	GCA_003726195.1	Rodent stool-associated circular genome virus
1074214	GCA_003726215.1	Rodent stool-associated circular genome virus
1074214	GCA_003726235.1	Rodent stool-associated circular genome virus
1074214	GCA_003726275.1	Rodent stool-associated circular genome virus
1074214	GCA_003726295.1	Rodent stool-associated circular genome virus
1074214	GCA_003726315.1	Rodent stool-associated circular genome virus

1074214	GCA_003726335.1	Rodent stool-associated circular genome virus
1074214	GCA_003726355.1	Rodent stool-associated circular genome virus
1074214	GCA_003726375.1	Rodent stool-associated circular genome virus
1074214	GCA_003726395.1	Rodent stool-associated circular genome virus
1074214	GCA_003726415.1	Rodent stool-associated circular genome virus
1074214	GCA_003726435.1	Rodent stool-associated circular genome virus
1074214	GCA_003726455.1	Rodent stool-associated circular genome virus
1074214	GCA_003726475.1	Rodent stool-associated circular genome virus
1074214	GCA_003726495.1	Rodent stool-associated circular genome virus
1074214	GCA_003726255.1	Rodent stool-associated circular genome virus
1514665	GCA_000930715.1	Rodent Torque teno virus 2
2054610	GCA_004131345.1	Rodent Torque teno virus 3
2054610	GCA_004131385.1	Rodent Torque teno virus 3
2054610	GCA_004131325.1	Rodent Torque teno virus 3
2054611	GCA_004131405.1	Rodent Torque teno virus 4
2054611	GCA_004131365.1	Rodent Torque teno virus 4
2054613	GCA_004131425.1	Rodent Torque teno virus 6
2054614	GCA_004131445.1	Rodent Torque teno virus 7
535898	GCA_000906675.1	Rosa rugosa leaf distortion virus
1511807	GCA_000918175.1	Rosavirus A2
1902501	GCA_001744155.1	Rosavirus B
1902502	GCA_001745475.1	Rosavirus C
1268383	GCA_002817335.1	Rosavirus M-7
492502	GCA_000879755.1	Rose cryptic virus 1
875325	GCA_000923375.1	Rose leaf curl betasatellite
875325	GCA_002830205.1	Rose leaf curl betasatellite
875323	GCA_000921455.1	Rose leaf curl virus
1543207	GCA_000924295.1	Rose leaf rosette-associated virus
1980433	GCA_000891875.2	Rose rosette emaravirus
1980433	GCA_000891875.1	Rose rosette emaravirus
474454	GCA_000874445.1	Rose spring dwarf-associated virus
1048434	GCA_000900415.1	Rose yellow mosaic virus
1213588	GCA_000906295.1	Rose yellow vein virus
1708712	GCA_001736275.1	Rosellinia necatrix endornavirus 1
1476202	GCA_000922215.1	Rosellinia necatrix fusarivirus 1
2032989	GCA_002890295.1	Rosellinia necatrix hypovirus 1
658904	GCA_000885395.1	Rosellinia necatrix megabirnavirus 1/W779
1676267	GCA_001551545.1	Rosellinia necatrix megabirnavirus 2-W8
235994	GCA_000864265.1	Rosellinia necatrix partitivirus 1-W8
859651	GCA_001343725.1	Rosellinia necatrix partitivirus 2
1573459	GCA_001433605.1	Rosellinia necatrix partitivirus 6
2025333	GCA_002890595.1	Rosellinia necatrix partitivirus 8
1000373	GCA_000895895.1	Rosellinia necatrix quadrivirus 1
1000373	GCA_003971625.1	Rosellinia necatrix quadrivirus 1
1148491	GCA_000907815.1	Rosellinia necatrix victorivirus 1
1735562	GCA_002607585.1	Roseobacter phage DSS3P8
562742	GCA_000892675.1	Roseobacter phage RDJL Phi 1
1682380	GCA_002623245.1	Roseobacter phage RDJL Phi 2
136084	GCA_000843225.1	Roseobacter virus SIO1
136084	GCA_002991035.1	Roseobacter virus SIO1

136084	GCA_002991075.1	Roseobacter virus SIO1
136084	GCA_002991025.1	Roseobacter virus SIO1
136084	GCA_002991065.1	Roseobacter virus SIO1
136084	GCA_003328505.1	Roseobacter virus SIO1
11029	GCA_000862165.1	Ross River virus
11029	GCA_002889255.1	Ross River virus
259931	GCA_000845305.1	Ross's goose hepatitis B virus
28875	GCA_000880735.1	Rotavirus A
28875	GCA_002636435.1	Rotavirus A
28875	GCA_002635435.1	Rotavirus A
28875	GCA_002635455.1	Rotavirus A
28875	GCA_002639855.1	Rotavirus A
28875	GCA_002635475.1	Rotavirus A
28875	GCA_002635515.1	Rotavirus A
28875	GCA_002635535.1	Rotavirus A
28875	GCA_002636455.1	Rotavirus A
28875	GCA_002635555.1	Rotavirus A
28875	GCA_002639655.1	Rotavirus A
28875	GCA_002640555.1	Rotavirus A
28875	GCA_002640215.1	Rotavirus A
28875	GCA_002635575.1	Rotavirus A
28875	GCA_002639875.1	Rotavirus A
28875	GCA_002639535.1	Rotavirus A
28875	GCA_002635595.1	Rotavirus A
28875	GCA_002635615.1	Rotavirus A
28875	GCA_002635395.1	Rotavirus A
28875	GCA_002635655.1	Rotavirus A
28875	GCA_002635675.1	Rotavirus A
28875	GCA_002640575.1	Rotavirus A
28875	GCA_002635695.1	Rotavirus A
28875	GCA_002639895.1	Rotavirus A
28875	GCA_002639555.1	Rotavirus A
28875	GCA_002635715.1	Rotavirus A
28875	GCA_002635755.1	Rotavirus A
28875	GCA_002635775.1	Rotavirus A
28875	GCA_002635795.1	Rotavirus A
28875	GCA_002640595.1	Rotavirus A
28875	GCA_002635815.1	Rotavirus A
28875	GCA_002639915.1	Rotavirus A
28875	GCA_002639575.1	Rotavirus A
28875	GCA_002635835.1	Rotavirus A
28875	GCA_002635855.1	Rotavirus A
28875	GCA_002635895.1	Rotavirus A
28875	GCA_002639995.1	Rotavirus A
28875	GCA_002635915.1	Rotavirus A
28875	GCA_002635495.1	Rotavirus A
28875	GCA_002635935.1	Rotavirus A
28875	GCA_002639935.1	Rotavirus A
28875	GCA_002639595.1	Rotavirus A
28875	GCA_002635955.1	Rotavirus A

28875	GCA_002635975.1	Rotavirus A
28875	GCA_002635995.1	Rotavirus A
28875	GCA_002636015.1	Rotavirus A
28875	GCA_002636035.1	Rotavirus A
28875	GCA_002636055.1	Rotavirus A
28875	GCA_002634495.1	Rotavirus A
28875	GCA_002636075.1	Rotavirus A
28875	GCA_002639275.1	Rotavirus A
28875	GCA_002636475.1	Rotavirus A
28875	GCA_002640635.1	Rotavirus A
28875	GCA_002636135.1	Rotavirus A
28875	GCA_002636155.1	Rotavirus A
28875	GCA_002640655.1	Rotavirus A
28875	GCA_002640315.1	Rotavirus A
28875	GCA_002639975.1	Rotavirus A
28875	GCA_002639635.1	Rotavirus A
28875	GCA_002636195.1	Rotavirus A
28875	GCA_002639615.1	Rotavirus A
28875	GCA_002636255.1	Rotavirus A
28875	GCA_002636275.1	Rotavirus A
28875	GCA_002640675.1	Rotavirus A
28875	GCA_002640335.1	Rotavirus A
28875	GCA_002636295.1	Rotavirus A
28875	GCA_002636315.1	Rotavirus A
28875	GCA_002639315.1	Rotavirus A
28875	GCA_002636335.1	Rotavirus A
28875	GCA_002639755.1	Rotavirus A
28875	GCA_002636385.1	Rotavirus A
28875	GCA_002640695.1	Rotavirus A
28875	GCA_002640355.1	Rotavirus A
28875	GCA_002636415.1	Rotavirus A
28875	GCA_002640015.1	Rotavirus A
28875	GCA_002639675.1	Rotavirus A
28875	GCA_002639335.1	Rotavirus A
28875	GCA_002636495.1	Rotavirus A
28875	GCA_002640715.1	Rotavirus A
28875	GCA_002640035.1	Rotavirus A
28875	GCA_002639695.1	Rotavirus A
28875	GCA_002639355.1	Rotavirus A
28875	GCA_002634515.1	Rotavirus A
28875	GCA_002634535.1	Rotavirus A
28875	GCA_002634555.1	Rotavirus A
28875	GCA_002634575.1	Rotavirus A
28875	GCA_002634595.1	Rotavirus A
28875	GCA_002640735.1	Rotavirus A
28875	GCA_002640055.1	Rotavirus A
28875	GCA_002634615.1	Rotavirus A
28875	GCA_002639715.1	Rotavirus A
28875	GCA_002639375.1	Rotavirus A
28875	GCA_002634655.1	Rotavirus A

28875	GCA_002634675.1	Rotavirus A
28875	GCA_002634715.1	Rotavirus A
28875	GCA_002640755.1	Rotavirus A
28875	GCA_002640415.1	Rotavirus A
28875	GCA_002640075.1	Rotavirus A
28875	GCA_002634735.1	Rotavirus A
28875	GCA_002639395.1	Rotavirus A
28875	GCA_002634755.1	Rotavirus A
28875	GCA_002636225.1	Rotavirus A
28875	GCA_002634775.1	Rotavirus A
28875	GCA_002638875.1	Rotavirus A
28875	GCA_002636835.1	Rotavirus A
28875	GCA_002634795.1	Rotavirus A
28875	GCA_002634815.1	Rotavirus A
28875	GCA_002634835.1	Rotavirus A
28875	GCA_002640775.1	Rotavirus A
28875	GCA_002640435.1	Rotavirus A
28875	GCA_002634855.1	Rotavirus A
28875	GCA_002634635.1	Rotavirus A
28875	GCA_002639415.1	Rotavirus A
28875	GCA_002634875.1	Rotavirus A
28875	GCA_002634905.1	Rotavirus A
28875	GCA_002634955.1	Rotavirus A
28875	GCA_002639055.1	Rotavirus A
28875	GCA_002634975.1	Rotavirus A
28875	GCA_002639775.1	Rotavirus A
28875	GCA_002639435.1	Rotavirus A
28875	GCA_002634995.1	Rotavirus A
28875	GCA_002635015.1	Rotavirus A
28875	GCA_002635035.1	Rotavirus A
28875	GCA_002635055.1	Rotavirus A
28875	GCA_002639155.1	Rotavirus A
28875	GCA_002635075.1	Rotavirus A
28875	GCA_002639175.1	Rotavirus A
28875	GCA_002640475.1	Rotavirus A
28875	GCA_002635095.1	Rotavirus A
28875	GCA_002639195.1	Rotavirus A
28875	GCA_002639795.1	Rotavirus A
28875	GCA_002639455.1	Rotavirus A
28875	GCA_002635115.1	Rotavirus A
28875	GCA_002639215.1	Rotavirus A
28875	GCA_002639235.1	Rotavirus A
28875	GCA_002639255.1	Rotavirus A
28875	GCA_002640495.1	Rotavirus A
28875	GCA_002639815.1	Rotavirus A
28875	GCA_002639475.1	Rotavirus A
28875	GCA_002635295.1	Rotavirus A
28875	GCA_002635315.1	Rotavirus A
28875	GCA_002640515.1	Rotavirus A
28875	GCA_002635335.1	Rotavirus A

28875	GCA_002639835.1	Rotavirus A
28875	GCA_002639495.1	Rotavirus A
28875	GCA_002635355.1	Rotavirus A
28875	GCA_002652135.1	Rotavirus A
28875	GCA_002645655.1	Rotavirus A
28875	GCA_002662575.1	Rotavirus A
28875	GCA_002651795.1	Rotavirus A
28875	GCA_002643615.1	Rotavirus A
28875	GCA_002658615.1	Rotavirus A
28875	GCA_002647715.1	Rotavirus A
28875	GCA_002657115.1	Rotavirus A
28875	GCA_002645675.1	Rotavirus A
28875	GCA_002656235.1	Rotavirus A
28875	GCA_002649775.1	Rotavirus A
28875	GCA_002653875.1	Rotavirus A
28875	GCA_002655895.1	Rotavirus A
28875	GCA_002651835.1	Rotavirus A
28875	GCA_002658275.1	Rotavirus A
28875	GCA_002655555.1	Rotavirus A
28875	GCA_002645695.1	Rotavirus A
28875	GCA_002656675.1	Rotavirus A
28875	GCA_002649795.1	Rotavirus A
28875	GCA_002643655.1	Rotavirus A
28875	GCA_002647755.1	Rotavirus A
28875	GCA_002654875.1	Rotavirus A
28875	GCA_002660775.1	Rotavirus A
28875	GCA_002645715.1	Rotavirus A
28875	GCA_002644295.1	Rotavirus A
28875	GCA_002649815.1	Rotavirus A
28875	GCA_002657935.1	Rotavirus A
28875	GCA_002643675.1	Rotavirus A
28875	GCA_002647775.1	Rotavirus A
28875	GCA_002656435.1	Rotavirus A
28875	GCA_002653855.1	Rotavirus A
28875	GCA_002649835.1	Rotavirus A
28875	GCA_002659875.1	Rotavirus A
28875	GCA_002643695.1	Rotavirus A
28875	GCA_002647795.1	Rotavirus A
28875	GCA_002653175.1	Rotavirus A
28875	GCA_002657595.1	Rotavirus A
28875	GCA_002661755.1	Rotavirus A
28875	GCA_002645755.1	Rotavirus A
28875	GCA_002652835.1	Rotavirus A
28875	GCA_002649855.1	Rotavirus A
28875	GCA_002660375.1	Rotavirus A
28875	GCA_002643715.1	Rotavirus A
28875	GCA_002647815.1	Rotavirus A
28875	GCA_002658975.1	Rotavirus A
28875	GCA_002658755.1	Rotavirus A
28875	GCA_002641915.1	Rotavirus A

28875	GCA_002645775.1	Rotavirus A
28875	GCA_002649875.1	Rotavirus A
28875	GCA_002657255.1	Rotavirus A
28875	GCA_002651815.1	Rotavirus A
28875	GCA_002643735.1	Rotavirus A
28875	GCA_002647835.1	Rotavirus A
28875	GCA_002641235.1	Rotavirus A
28875	GCA_002651935.1	Rotavirus A
28875	GCA_002659915.1	Rotavirus A
28875	GCA_002656035.1	Rotavirus A
28875	GCA_002658075.1	Rotavirus A
28875	GCA_002656255.1	Rotavirus A
28875	GCA_002649895.1	Rotavirus A
28875	GCA_002643755.1	Rotavirus A
28875	GCA_002655915.1	Rotavirus A
28875	GCA_002647855.1	Rotavirus A
28875	GCA_002661075.1	Rotavirus A
28875	GCA_002655575.1	Rotavirus A
28875	GCA_002645815.1	Rotavirus A
28875	GCA_002649915.1	Rotavirus A
28875	GCA_002654015.1	Rotavirus A
28875	GCA_002647875.1	Rotavirus A
28875	GCA_002654895.1	Rotavirus A
28875	GCA_002651975.1	Rotavirus A
28875	GCA_002645835.1	Rotavirus A
28875	GCA_002654555.1	Rotavirus A
28875	GCA_002649935.1	Rotavirus A
28875	GCA_002656575.1	Rotavirus A
28875	GCA_002660735.1	Rotavirus A
28875	GCA_002643795.1	Rotavirus A
28875	GCA_002643975.1	Rotavirus A
28875	GCA_002647895.1	Rotavirus A
28875	GCA_002655795.1	Rotavirus A
28875	GCA_002643635.1	Rotavirus A
28875	GCA_002645855.1	Rotavirus A
28875	GCA_002657735.1	Rotavirus A
28875	GCA_002643295.1	Rotavirus A
28875	GCA_002658055.1	Rotavirus A
28875	GCA_002647915.1	Rotavirus A
28875	GCA_002653195.1	Rotavirus A
28875	GCA_002645875.1	Rotavirus A
28875	GCA_002649975.1	Rotavirus A
28875	GCA_002658895.1	Rotavirus A
28875	GCA_002660135.1	Rotavirus A
28875	GCA_002654075.1	Rotavirus A
28875	GCA_002647935.1	Rotavirus A
28875	GCA_002657395.1	Rotavirus A
28875	GCA_002652035.1	Rotavirus A
28875	GCA_002645895.1	Rotavirus A
28875	GCA_002649995.1	Rotavirus A

28875	GCA_002647955.1	Rotavirus A
28875	GCA_002657815.1	Rotavirus A
28875	GCA_002652055.1	Rotavirus A
28875	GCA_002645915.1	Rotavirus A
28875	GCA_002656275.1	Rotavirus A
28875	GCA_002650015.1	Rotavirus A
28875	GCA_002657695.1	Rotavirus A
28875	GCA_002658335.1	Rotavirus A
28875	GCA_002655935.1	Rotavirus A
28875	GCA_002657575.1	Rotavirus A
28875	GCA_002655595.1	Rotavirus A
28875	GCA_002645935.1	Rotavirus A
28875	GCA_002650035.1	Rotavirus A
28875	GCA_002657455.1	Rotavirus A
28875	GCA_002647995.1	Rotavirus A
28875	GCA_002656715.1	Rotavirus A
28875	GCA_002654915.1	Rotavirus A
28875	GCA_002644335.1	Rotavirus A
28875	GCA_002650055.1	Rotavirus A
28875	GCA_002663535.1	Rotavirus A
28875	GCA_002654155.1	Rotavirus A
28875	GCA_002643995.1	Rotavirus A
28875	GCA_002648015.1	Rotavirus A
28875	GCA_002657875.1	Rotavirus A
28875	GCA_002652115.1	Rotavirus A
28875	GCA_002653895.1	Rotavirus A
28875	GCA_002645975.1	Rotavirus A
28875	GCA_002656375.1	Rotavirus A
28875	GCA_002650075.1	Rotavirus A
28875	GCA_002660535.1	Rotavirus A
28875	GCA_002653555.1	Rotavirus A
28875	GCA_002648035.1	Rotavirus A
28875	GCA_002659035.1	Rotavirus A
28875	GCA_002653215.1	Rotavirus A
28875	GCA_002645995.1	Rotavirus A
28875	GCA_002652875.1	Rotavirus A
28875	GCA_002654195.1	Rotavirus A
28875	GCA_002652535.1	Rotavirus A
28875	GCA_002648055.1	Rotavirus A
28875	GCA_002660095.1	Rotavirus A
28875	GCA_002652155.1	Rotavirus A
28875	GCA_002659835.1	Rotavirus A
28875	GCA_002652195.1	Rotavirus A
28875	GCA_002646015.1	Rotavirus A
28875	GCA_002660895.1	Rotavirus A
28875	GCA_002658695.1	Rotavirus A
28875	GCA_002650115.1	Rotavirus A
28875	GCA_002651855.1	Rotavirus A
28875	GCA_002654215.1	Rotavirus A
28875	GCA_002648075.1	Rotavirus A

28875	GCA_002641935.1	Rotavirus A
28875	GCA_002646035.1	Rotavirus A
28875	GCA_002656295.1	Rotavirus A
28875	GCA_002650135.1	Rotavirus A
28875	GCA_002660655.1	Rotavirus A
28875	GCA_002654235.1	Rotavirus A
28875	GCA_002648095.1	Rotavirus A
28875	GCA_002656915.1	Rotavirus A
28875	GCA_002662515.1	Rotavirus A
28875	GCA_002658595.1	Rotavirus A
28875	GCA_002655615.1	Rotavirus A
28875	GCA_002646055.1	Rotavirus A
28875	GCA_002650155.1	Rotavirus A
28875	GCA_002644015.1	Rotavirus A
28875	GCA_002648115.1	Rotavirus A
28875	GCA_002652215.1	Rotavirus A
28875	GCA_002646075.1	Rotavirus A
28875	GCA_002658015.1	Rotavirus A
28875	GCA_002644355.1	Rotavirus A
28875	GCA_002650175.1	Rotavirus A
28875	GCA_002644035.1	Rotavirus A
28875	GCA_002654255.1	Rotavirus A
28875	GCA_002648135.1	Rotavirus A
28875	GCA_002660675.1	Rotavirus A
28875	GCA_002653915.1	Rotavirus A
28875	GCA_002646095.1	Rotavirus A
28875	GCA_002643335.1	Rotavirus A
28875	GCA_002644055.1	Rotavirus A
28875	GCA_002656795.1	Rotavirus A
28875	GCA_002648155.1	Rotavirus A
28875	GCA_002642995.1	Rotavirus A
28875	GCA_002652255.1	Rotavirus A
28875	GCA_002646115.1	Rotavirus A
28875	GCA_002660335.1	Rotavirus A
28875	GCA_002652895.1	Rotavirus A
28875	GCA_002650215.1	Rotavirus A
28875	GCA_002644075.1	Rotavirus A
28875	GCA_002648175.1	Rotavirus A
28875	GCA_002652275.1	Rotavirus A
28875	GCA_002646135.1	Rotavirus A
28875	GCA_002657335.1	Rotavirus A
28875	GCA_002660355.1	Rotavirus A
28875	GCA_002650235.1	Rotavirus A
28875	GCA_002651875.1	Rotavirus A
28875	GCA_002644095.1	Rotavirus A
28875	GCA_002652295.1	Rotavirus A
28875	GCA_002646155.1	Rotavirus A
28875	GCA_002658495.1	Rotavirus A
28875	GCA_002656315.1	Rotavirus A
28875	GCA_002650255.1	Rotavirus A

28875	GCA_002644115.1	Rotavirus A
28875	GCA_002656995.1	Rotavirus A
28875	GCA_002655975.1	Rotavirus A
28875	GCA_002648215.1	Rotavirus A
28875	GCA_002652315.1	Rotavirus A
28875	GCA_002655635.1	Rotavirus A
28875	GCA_002650275.1	Rotavirus A
28875	GCA_002644135.1	Rotavirus A
28875	GCA_002658155.1	Rotavirus A
28875	GCA_002648235.1	Rotavirus A
28875	GCA_002652335.1	Rotavirus A
28875	GCA_002657015.1	Rotavirus A
28875	GCA_002656655.1	Rotavirus A
28875	GCA_002646195.1	Rotavirus A
28875	GCA_002654615.1	Rotavirus A
28875	GCA_002650295.1	Rotavirus A
28875	GCA_002654275.1	Rotavirus A
28875	GCA_002648255.1	Rotavirus A
28875	GCA_002657955.1	Rotavirus A
28875	GCA_002652355.1	Rotavirus A
28875	GCA_002653935.1	Rotavirus A
28875	GCA_002646215.1	Rotavirus A
28875	GCA_002650315.1	Rotavirus A
28875	GCA_002653595.1	Rotavirus A
28875	GCA_002654415.1	Rotavirus A
28875	GCA_002660475.1	Rotavirus A
28875	GCA_002648275.1	Rotavirus A
28875	GCA_002653255.1	Rotavirus A
28875	GCA_002652375.1	Rotavirus A
28875	GCA_002646235.1	Rotavirus A
28875	GCA_002652915.1	Rotavirus A
28875	GCA_002650335.1	Rotavirus A
28875	GCA_002654435.1	Rotavirus A
28875	GCA_002657475.1	Rotavirus A
28875	GCA_002656755.1	Rotavirus A
28875	GCA_002648295.1	Rotavirus A
28875	GCA_002652235.1	Rotavirus A
28875	GCA_002646255.1	Rotavirus A
28875	GCA_002650355.1	Rotavirus A
28875	GCA_002651895.1	Rotavirus A
28875	GCA_002644215.1	Rotavirus A
28875	GCA_002660615.1	Rotavirus A
28875	GCA_002648315.1	Rotavirus A
28875	GCA_002657135.1	Rotavirus A
28875	GCA_002646275.1	Rotavirus A
28875	GCA_002656335.1	Rotavirus A
28875	GCA_002650375.1	Rotavirus A
28875	GCA_002654475.1	Rotavirus A
28875	GCA_002655995.1	Rotavirus A
28875	GCA_002648335.1	Rotavirus A

28875	GCA_002655655.1	Rotavirus A
28875	GCA_002646295.1	Rotavirus A
28875	GCA_002650395.1	Rotavirus A
28875	GCA_002654495.1	Rotavirus A
28875	GCA_002648355.1	Rotavirus A
28875	GCA_002659115.1	Rotavirus A
28875	GCA_002652455.1	Rotavirus A
28875	GCA_002654515.1	Rotavirus A
28875	GCA_002654295.1	Rotavirus A
28875	GCA_002648375.1	Rotavirus A
28875	GCA_002652475.1	Rotavirus A
28875	GCA_002656455.1	Rotavirus A
28875	GCA_002658215.1	Rotavirus A
28875	GCA_002650435.1	Rotavirus A
28875	GCA_002643375.1	Rotavirus A
28875	GCA_002654535.1	Rotavirus A
28875	GCA_002657915.1	Rotavirus A
28875	GCA_002648395.1	Rotavirus A
28875	GCA_002643035.1	Rotavirus A
28875	GCA_002652495.1	Rotavirus A
28875	GCA_002657615.1	Rotavirus A
28875	GCA_002652935.1	Rotavirus A
28875	GCA_002650455.1	Rotavirus A
28875	GCA_002644315.1	Rotavirus A
28875	GCA_002660275.1	Rotavirus A
28875	GCA_002652515.1	Rotavirus A
28875	GCA_002658775.1	Rotavirus A
28875	GCA_002650475.1	Rotavirus A
28875	GCA_002651915.1	Rotavirus A
28875	GCA_002654575.1	Rotavirus A
28875	GCA_002648435.1	Rotavirus A
28875	GCA_002656355.1	Rotavirus A
28875	GCA_002650495.1	Rotavirus A
28875	GCA_002658435.1	Rotavirus A
28875	GCA_002654595.1	Rotavirus A
28875	GCA_002656015.1	Rotavirus A
28875	GCA_002648455.1	Rotavirus A
28875	GCA_002652555.1	Rotavirus A
28875	GCA_002655675.1	Rotavirus A
28875	GCA_002650515.1	Rotavirus A
28875	GCA_002644375.1	Rotavirus A
28875	GCA_002663755.1	Rotavirus A
28875	GCA_002648475.1	Rotavirus A
28875	GCA_002652575.1	Rotavirus A
28875	GCA_002654655.1	Rotavirus A
28875	GCA_002650535.1	Rotavirus A
28875	GCA_002656595.1	Rotavirus A
28875	GCA_002654315.1	Rotavirus A
28875	GCA_002648495.1	Rotavirus A
28875	GCA_002659075.1	Rotavirus A

28875	GCA_002652595.1	Rotavirus A
28875	GCA_002653975.1	Rotavirus A
28875	GCA_002650555.1	Rotavirus A
28875	GCA_002643395.1	Rotavirus A
28875	GCA_002644415.1	Rotavirus A
28875	GCA_002658475.1	Rotavirus A
28875	GCA_002648515.1	Rotavirus A
28875	GCA_002652615.1	Rotavirus A
28875	GCA_002642715.1	Rotavirus A
28875	GCA_002650575.1	Rotavirus A
28875	GCA_002658555.1	Rotavirus A
28875	GCA_002658915.1	Rotavirus A
28875	GCA_002654675.1	Rotavirus A
28875	GCA_002648535.1	Rotavirus A
28875	GCA_002657415.1	Rotavirus A
28875	GCA_002650595.1	Rotavirus A
28875	GCA_002644455.1	Rotavirus A
28875	GCA_002658315.1	Rotavirus A
28875	GCA_002648555.1	Rotavirus A
28875	GCA_002650615.1	Rotavirus A
28875	GCA_002657075.1	Rotavirus A
28875	GCA_002644475.1	Rotavirus A
28875	GCA_002648575.1	Rotavirus A
28875	GCA_002656535.1	Rotavirus A
28875	GCA_002655695.1	Rotavirus A
28875	GCA_002658235.1	Rotavirus A
28875	GCA_002644495.1	Rotavirus A
28875	GCA_002648595.1	Rotavirus A
28875	GCA_002656735.1	Rotavirus A
28875	GCA_002652695.1	Rotavirus A
28875	GCA_002644435.1	Rotavirus A
28875	GCA_002650655.1	Rotavirus A
28875	GCA_002644515.1	Rotavirus A
28875	GCA_002654335.1	Rotavirus A
28875	GCA_002648615.1	Rotavirus A
28875	GCA_002657295.1	Rotavirus A
28875	GCA_002657835.1	Rotavirus A
28875	GCA_002653995.1	Rotavirus A
28875	GCA_002650675.1	Rotavirus A
28875	GCA_002660555.1	Rotavirus A
28875	GCA_002643415.1	Rotavirus A
28875	GCA_002644535.1	Rotavirus A
28875	GCA_002648635.1	Rotavirus A
28875	GCA_002659055.1	Rotavirus A
28875	GCA_002653315.1	Rotavirus A
28875	GCA_002658735.1	Rotavirus A
28875	GCA_002656415.1	Rotavirus A
28875	GCA_002660115.1	Rotavirus A
28875	GCA_002642735.1	Rotavirus A
28875	GCA_002650695.1	Rotavirus A

28875	GCA_002644555.1	Rotavirus A
28875	GCA_002648655.1	Rotavirus A
28875	GCA_002658295.1	Rotavirus A
28875	GCA_002652755.1	Rotavirus A
28875	GCA_002656855.1	Rotavirus A
28875	GCA_002650715.1	Rotavirus A
28875	GCA_002644575.1	Rotavirus A
28875	GCA_002648675.1	Rotavirus A
28875	GCA_002657215.1	Rotavirus A
28875	GCA_002650735.1	Rotavirus A
28875	GCA_002644595.1	Rotavirus A
28875	GCA_002656055.1	Rotavirus A
28875	GCA_002648695.1	Rotavirus A
28875	GCA_002658375.1	Rotavirus A
28875	GCA_002652795.1	Rotavirus A
28875	GCA_002655715.1	Rotavirus A
28875	GCA_002650755.1	Rotavirus A
28875	GCA_002655375.1	Rotavirus A
28875	GCA_002648715.1	Rotavirus A
28875	GCA_002652815.1	Rotavirus A
28875	GCA_002654695.1	Rotavirus A
28875	GCA_002650775.1	Rotavirus A
28875	GCA_002644635.1	Rotavirus A
28875	GCA_002654355.1	Rotavirus A
28875	GCA_002648735.1	Rotavirus A
28875	GCA_002657175.1	Rotavirus A
28875	GCA_002660695.1	Rotavirus A
28875	GCA_002642595.1	Rotavirus A
28875	GCA_002643775.1	Rotavirus A
28875	GCA_002650795.1	Rotavirus A
28875	GCA_002657055.1	Rotavirus A
28875	GCA_002643435.1	Rotavirus A
28875	GCA_002644655.1	Rotavirus A
28875	GCA_002648755.1	Rotavirus A
28875	GCA_002643095.1	Rotavirus A
28875	GCA_002656935.1	Rotavirus A
28875	GCA_002652995.1	Rotavirus A
28875	GCA_002650815.1	Rotavirus A
28875	GCA_002644675.1	Rotavirus A
28875	GCA_002652655.1	Rotavirus A
28875	GCA_002648775.1	Rotavirus A
28875	GCA_002642635.1	Rotavirus A
28875	GCA_002657355.1	Rotavirus A
28875	GCA_002650835.1	Rotavirus A
28875	GCA_002644695.1	Rotavirus A
28875	GCA_002648795.1	Rotavirus A
28875	GCA_002658095.1	Rotavirus A
28875	GCA_002651635.1	Rotavirus A
28875	GCA_002642655.1	Rotavirus A
28875	GCA_002662555.1	Rotavirus A

28875	GCA_002658515.1	Rotavirus A
28875	GCA_002644715.1	Rotavirus A
28875	GCA_002656075.1	Rotavirus A
28875	GCA_002648815.1	Rotavirus A
28875	GCA_002642675.1	Rotavirus A
28875	GCA_002650875.1	Rotavirus A
28875	GCA_002655395.1	Rotavirus A
28875	GCA_002644735.1	Rotavirus A
28875	GCA_002648835.1	Rotavirus A
28875	GCA_002642695.1	Rotavirus A
28875	GCA_002660835.1	Rotavirus A
28875	GCA_002654715.1	Rotavirus A
28875	GCA_002644755.1	Rotavirus A
28875	GCA_002654375.1	Rotavirus A
28875	GCA_002660755.1	Rotavirus A
28875	GCA_002652955.1	Rotavirus A
28875	GCA_002654035.1	Rotavirus A
28875	GCA_002642815.1	Rotavirus A
28875	GCA_002650915.1	Rotavirus A
28875	GCA_002643455.1	Rotavirus A
28875	GCA_002644775.1	Rotavirus A
28875	GCA_002660495.1	Rotavirus A
28875	GCA_002648875.1	Rotavirus A
28875	GCA_002643115.1	Rotavirus A
28875	GCA_002652975.1	Rotavirus A
28875	GCA_002658995.1	Rotavirus A
28875	GCA_002653015.1	Rotavirus A
28875	GCA_002650935.1	Rotavirus A
28875	GCA_002644795.1	Rotavirus A
28875	GCA_002657495.1	Rotavirus A
28875	GCA_002652675.1	Rotavirus A
28875	GCA_002648895.1	Rotavirus A
28875	GCA_002642755.1	Rotavirus A
28875	GCA_002660155.1	Rotavirus A
28875	GCA_002650955.1	Rotavirus A
28875	GCA_002651995.1	Rotavirus A
28875	GCA_002644815.1	Rotavirus A
28875	GCA_002658655.1	Rotavirus A
28875	GCA_002658815.1	Rotavirus A
28875	GCA_002648915.1	Rotavirus A
28875	GCA_002642775.1	Rotavirus A
28875	GCA_002657155.1	Rotavirus A
28875	GCA_002661315.1	Rotavirus A
28875	GCA_002651315.1	Rotavirus A
28875	GCA_002650975.1	Rotavirus A
28875	GCA_002658355.1	Rotavirus A
28875	GCA_002656095.1	Rotavirus A
28875	GCA_002648935.1	Rotavirus A
28875	GCA_002642795.1	Rotavirus A
28875	GCA_002655755.1	Rotavirus A

28875	GCA_002662475.1	Rotavirus A
28875	GCA_002650995.1	Rotavirus A
28875	GCA_002655415.1	Rotavirus A
28875	GCA_002644855.1	Rotavirus A
28875	GCA_002648955.1	Rotavirus A
28875	GCA_002653055.1	Rotavirus A
28875	GCA_002660635.1	Rotavirus A
28875	GCA_002654735.1	Rotavirus A
28875	GCA_002651015.1	Rotavirus A
28875	GCA_002644875.1	Rotavirus A
28875	GCA_002649755.1	Rotavirus A
28875	GCA_002644155.1	Rotavirus A
28875	GCA_002648975.1	Rotavirus A
28875	GCA_002656555.1	Rotavirus A
28875	GCA_002656475.1	Rotavirus A
28875	GCA_002654055.1	Rotavirus A
28875	GCA_002651035.1	Rotavirus A
28875	GCA_002643475.1	Rotavirus A
28875	GCA_002644895.1	Rotavirus A
28875	GCA_002659135.1	Rotavirus A
28875	GCA_002663295.1	Rotavirus A
28875	GCA_002648995.1	Rotavirus A
28875	GCA_002643135.1	Rotavirus A
28875	GCA_002642855.1	Rotavirus A
28875	GCA_002657635.1	Rotavirus A
28875	GCA_002646955.1	Rotavirus A
28875	GCA_002653035.1	Rotavirus A
28875	GCA_002644915.1	Rotavirus A
28875	GCA_002660295.1	Rotavirus A
28875	GCA_002649015.1	Rotavirus A
28875	GCA_002642875.1	Rotavirus A
28875	GCA_002658795.1	Rotavirus A
28875	GCA_002646975.1	Rotavirus A
28875	GCA_002652015.1	Rotavirus A
28875	GCA_002644935.1	Rotavirus A
28875	GCA_002663975.1	Rotavirus A
28875	GCA_002649035.1	Rotavirus A
28875	GCA_002651675.1	Rotavirus A
28875	GCA_002653135.1	Rotavirus A
28875	GCA_002645795.1	Rotavirus A
28875	GCA_002646995.1	Rotavirus A
28875	GCA_002651335.1	Rotavirus A
28875	GCA_002651095.1	Rotavirus A
28875	GCA_002644955.1	Rotavirus A
28875	GCA_002656115.1	Rotavirus A
28875	GCA_002649055.1	Rotavirus A
28875	GCA_002642915.1	Rotavirus A
28875	GCA_002656955.1	Rotavirus A
28875	GCA_002655775.1	Rotavirus A
28875	GCA_002647015.1	Rotavirus A

28875	GCA_002651115.1	Rotavirus A
28875	GCA_002655435.1	Rotavirus A
28875	GCA_002644975.1	Rotavirus A
28875	GCA_002642935.1	Rotavirus A
28875	GCA_002658115.1	Rotavirus A
28875	GCA_002660395.1	Rotavirus A
28875	GCA_002647035.1	Rotavirus A
28875	GCA_002654755.1	Rotavirus A
28875	GCA_002651135.1	Rotavirus A
28875	GCA_002644995.1	Rotavirus A
28875	GCA_002649095.1	Rotavirus A
28875	GCA_002642955.1	Rotavirus A
28875	GCA_002643835.1	Rotavirus A
28875	GCA_002647055.1	Rotavirus A
28875	GCA_002656815.1	Rotavirus A
28875	GCA_002651155.1	Rotavirus A
28875	GCA_002657775.1	Rotavirus A
28875	GCA_002643495.1	Rotavirus A
28875	GCA_002645015.1	Rotavirus A
28875	GCA_002649115.1	Rotavirus A
28875	GCA_002656515.1	Rotavirus A
28875	GCA_002653395.1	Rotavirus A
28875	GCA_002642975.1	Rotavirus A
28875	GCA_002660435.1	Rotavirus A
28875	GCA_002658175.1	Rotavirus A
28875	GCA_002651175.1	Rotavirus A
28875	GCA_002658935.1	Rotavirus A
28875	GCA_002645035.1	Rotavirus A
28875	GCA_002652715.1	Rotavirus A
28875	GCA_002649135.1	Rotavirus A
28875	GCA_002653235.1	Rotavirus A
28875	GCA_002647095.1	Rotavirus A
28875	GCA_002651195.1	Rotavirus A
28875	GCA_002649155.1	Rotavirus A
28875	GCA_002651695.1	Rotavirus A
28875	GCA_002643015.1	Rotavirus A
28875	GCA_002647115.1	Rotavirus A
28875	GCA_002651215.1	Rotavirus A
28875	GCA_002645075.1	Rotavirus A
28875	GCA_002656135.1	Rotavirus A
28875	GCA_002649175.1	Rotavirus A
28875	GCA_002653275.1	Rotavirus A
28875	GCA_002660915.1	Rotavirus A
28875	GCA_002647135.1	Rotavirus A
28875	GCA_002658875.1	Rotavirus A
28875	GCA_002651235.1	Rotavirus A
28875	GCA_002658255.1	Rotavirus A
28875	GCA_002655455.1	Rotavirus A
28875	GCA_002645095.1	Rotavirus A
28875	GCA_002658575.1	Rotavirus A

28875	GCA_002649195.1	Rotavirus A
28875	GCA_002647155.1	Rotavirus A
28875	GCA_002654775.1	Rotavirus A
28875	GCA_002651255.1	Rotavirus A
28875	GCA_002645115.1	Rotavirus A
28875	GCA_002644195.1	Rotavirus A
28875	GCA_002649215.1	Rotavirus A
28875	GCA_002657675.1	Rotavirus A
28875	GCA_002643075.1	Rotavirus A
28875	GCA_002654095.1	Rotavirus A
28875	GCA_002647175.1	Rotavirus A
28875	GCA_002660575.1	Rotavirus A
28875	GCA_002643515.1	Rotavirus A
28875	GCA_002645135.1	Rotavirus A
28875	GCA_002649235.1	Rotavirus A
28875	GCA_002643175.1	Rotavirus A
28875	GCA_002653335.1	Rotavirus A
28875	GCA_002647195.1	Rotavirus A
28875	GCA_002656775.1	Rotavirus A
28875	GCA_002645155.1	Rotavirus A
28875	GCA_002652735.1	Rotavirus A
28875	GCA_002649255.1	Rotavirus A
28875	GCA_002660235.1	Rotavirus A
28875	GCA_002653355.1	Rotavirus A
28875	GCA_002652395.1	Rotavirus A
28875	GCA_002647215.1	Rotavirus A
28875	GCA_002645175.1	Rotavirus A
28875	GCA_002649275.1	Rotavirus A
28875	GCA_002657235.1	Rotavirus A
28875	GCA_002653375.1	Rotavirus A
28875	GCA_002647235.1	Rotavirus A
28875	GCA_002659895.1	Rotavirus A
28875	GCA_002645195.1	Rotavirus A
28875	GCA_002656155.1	Rotavirus A
28875	GCA_002658035.1	Rotavirus A
28875	GCA_002658395.1	Rotavirus A
28875	GCA_002643155.1	Rotavirus A
28875	GCA_002664195.1	Rotavirus A
28875	GCA_002655815.1	Rotavirus A
28875	GCA_002647255.1	Rotavirus A
28875	GCA_002656895.1	Rotavirus A
28875	GCA_002651355.1	Rotavirus A
28875	GCA_002655475.1	Rotavirus A
28875	GCA_002645215.1	Rotavirus A
28875	GCA_002649315.1	Rotavirus A
28875	GCA_002653415.1	Rotavirus A
28875	GCA_002647275.1	Rotavirus A
28875	GCA_002658835.1	Rotavirus A
28875	GCA_002654795.1	Rotavirus A
28875	GCA_002651375.1	Rotavirus A

28875	GCA_002645235.1	Rotavirus A
28875	GCA_002654455.1	Rotavirus A
28875	GCA_002649335.1	Rotavirus A
28875	GCA_002660715.1	Rotavirus A
28875	GCA_002643195.1	Rotavirus A
28875	GCA_002654115.1	Rotavirus A
28875	GCA_002651395.1	Rotavirus A
28875	GCA_002643535.1	Rotavirus A
28875	GCA_002645255.1	Rotavirus A
28875	GCA_002649355.1	Rotavirus A
28875	GCA_002657715.1	Rotavirus A
28875	GCA_002653435.1	Rotavirus A
28875	GCA_002643215.1	Rotavirus A
28875	GCA_002647315.1	Rotavirus A
28875	GCA_002657555.1	Rotavirus A
28875	GCA_002653095.1	Rotavirus A
28875	GCA_002651415.1	Rotavirus A
28875	GCA_002649375.1	Rotavirus A
28875	GCA_002657435.1	Rotavirus A
28875	GCA_002653475.1	Rotavirus A
28875	GCA_002647335.1	Rotavirus A
28875	GCA_002659815.1	Rotavirus A
28875	GCA_002657375.1	Rotavirus A
28875	GCA_002651435.1	Rotavirus A
28875	GCA_002652075.1	Rotavirus A
28875	GCA_002645295.1	Rotavirus A
28875	GCA_002657275.1	Rotavirus A
28875	GCA_002649395.1	Rotavirus A
28875	GCA_002643255.1	Rotavirus A
28875	GCA_002657195.1	Rotavirus A
28875	GCA_002647355.1	Rotavirus A
28875	GCA_002658535.1	Rotavirus A
28875	GCA_002651455.1	Rotavirus A
28875	GCA_002660595.1	Rotavirus A
28875	GCA_002645315.1	Rotavirus A
28875	GCA_002649415.1	Rotavirus A
28875	GCA_002655835.1	Rotavirus A
28875	GCA_002647375.1	Rotavirus A
28875	GCA_002651475.1	Rotavirus A
28875	GCA_002655495.1	Rotavirus A
28875	GCA_002645335.1	Rotavirus A
28875	GCA_002649435.1	Rotavirus A
28875	GCA_002653535.1	Rotavirus A
28875	GCA_002647395.1	Rotavirus A
28875	GCA_002656695.1	Rotavirus A
28875	GCA_002654815.1	Rotavirus A
28875	GCA_002651495.1	Rotavirus A
28875	GCA_002645355.1	Rotavirus A
28875	GCA_002661535.1	Rotavirus A
28875	GCA_002644235.1	Rotavirus A

28875	GCA_002649455.1	Rotavirus A
28875	GCA_002663515.1	Rotavirus A
28875	GCA_002643315.1	Rotavirus A
28875	GCA_002654135.1	Rotavirus A
28875	GCA_002647415.1	Rotavirus A
28875	GCA_002657855.1	Rotavirus A
28875	GCA_002641275.1	Rotavirus A
28875	GCA_002643555.1	Rotavirus A
28875	GCA_002645375.1	Rotavirus A
28875	GCA_002649475.1	Rotavirus A
28875	GCA_002661295.1	Rotavirus A
28875	GCA_002653455.1	Rotavirus A
28875	GCA_002653575.1	Rotavirus A
28875	GCA_002647435.1	Rotavirus A
28875	GCA_002659015.1	Rotavirus A
28875	GCA_002657895.1	Rotavirus A
28875	GCA_002653115.1	Rotavirus A
28875	GCA_002651535.1	Rotavirus A
28875	GCA_002645395.1	Rotavirus A
28875	GCA_002657515.1	Rotavirus A
28875	GCA_002652775.1	Rotavirus A
28875	GCA_002649495.1	Rotavirus A
28875	GCA_002643355.1	Rotavirus A
28875	GCA_002652435.1	Rotavirus A
28875	GCA_002647455.1	Rotavirus A
28875	GCA_002660175.1	Rotavirus A
28875	GCA_002651555.1	Rotavirus A
28875	GCA_002652095.1	Rotavirus A
28875	GCA_002645415.1	Rotavirus A
28875	GCA_002657035.1	Rotavirus A
28875	GCA_002658675.1	Rotavirus A
28875	GCA_002651755.1	Rotavirus A
28875	GCA_002653615.1	Rotavirus A
28875	GCA_002647475.1	Rotavirus A
28875	GCA_002651575.1	Rotavirus A
28875	GCA_002645435.1	Rotavirus A
28875	GCA_002656195.1	Rotavirus A
28875	GCA_002649535.1	Rotavirus A
28875	GCA_002660855.1	Rotavirus A
28875	GCA_002660875.1	Rotavirus A
28875	GCA_002653635.1	Rotavirus A
28875	GCA_002655855.1	Rotavirus A
28875	GCA_002647495.1	Rotavirus A
28875	GCA_002651595.1	Rotavirus A
28875	GCA_002645455.1	Rotavirus A
28875	GCA_002660255.1	Rotavirus A
28875	GCA_002649555.1	Rotavirus A
28875	GCA_002653655.1	Rotavirus A
28875	GCA_002654835.1	Rotavirus A
28875	GCA_002651615.1	Rotavirus A

28875	GCA_002645475.1	Rotavirus A
28875	GCA_002657995.1	Rotavirus A
28875	GCA_002644255.1	Rotavirus A
28875	GCA_002649575.1	Rotavirus A
28875	GCA_002653675.1	Rotavirus A
28875	GCA_002656495.1	Rotavirus A
28875	GCA_002647535.1	Rotavirus A
28875	GCA_002643575.1	Rotavirus A
28875	GCA_002659155.1	Rotavirus A
28875	GCA_002649595.1	Rotavirus A
28875	GCA_002643235.1	Rotavirus A
28875	GCA_002653695.1	Rotavirus A
28875	GCA_002657655.1	Rotavirus A
28875	GCA_002647555.1	Rotavirus A
28875	GCA_002642895.1	Rotavirus A
28875	GCA_002651655.1	Rotavirus A
28875	GCA_002658455.1	Rotavirus A
28875	GCA_002645515.1	Rotavirus A
28875	GCA_002660315.1	Rotavirus A
28875	GCA_002649615.1	Rotavirus A
28875	GCA_002653715.1	Rotavirus A
28875	GCA_002647575.1	Rotavirus A
28875	GCA_002645535.1	Rotavirus A
28875	GCA_002649635.1	Rotavirus A
28875	GCA_002647595.1	Rotavirus A
28875	GCA_002657795.1	Rotavirus A
28875	GCA_002645555.1	Rotavirus A
28875	GCA_002656215.1	Rotavirus A
28875	GCA_002649655.1	Rotavirus A
28875	GCA_002653755.1	Rotavirus A
28875	GCA_002656975.1	Rotavirus A
28875	GCA_002655875.1	Rotavirus A
28875	GCA_002647615.1	Rotavirus A
28875	GCA_002651715.1	Rotavirus A
28875	GCA_002655535.1	Rotavirus A
28875	GCA_002645575.1	Rotavirus A
28875	GCA_002649675.1	Rotavirus A
28875	GCA_002653775.1	Rotavirus A
28875	GCA_002658135.1	Rotavirus A
28875	GCA_002660515.1	Rotavirus A
28875	GCA_002647635.1	Rotavirus A
28875	GCA_002656635.1	Rotavirus A
28875	GCA_002645595.1	Rotavirus A
28875	GCA_002660795.1	Rotavirus A
28875	GCA_002660215.1	Rotavirus A
28875	GCA_002644275.1	Rotavirus A
28875	GCA_002649695.1	Rotavirus A
28875	GCA_002653795.1	Rotavirus A
28875	GCA_002647655.1	Rotavirus A
28875	GCA_002643595.1	Rotavirus A

28875	GCA_002645615.1	Rotavirus A
28875	GCA_002649715.1	Rotavirus A
28875	GCA_002653495.1	Rotavirus A
28875	GCA_002653815.1	Rotavirus A
28875	GCA_002660455.1	Rotavirus A
28875	GCA_002647675.1	Rotavirus A
28875	GCA_002653155.1	Rotavirus A
28875	GCA_002651775.1	Rotavirus A
28875	GCA_002658955.1	Rotavirus A
28875	GCA_002645635.1	Rotavirus A
28875	GCA_002651275.1	Rotavirus A
28875	GCA_002642575.1	Rotavirus A
28875	GCA_002658715.1	Rotavirus A
28875	GCA_002653835.1	Rotavirus A
28875	GCA_002647695.1	Rotavirus A
28875	GCA_002672275.1	Rotavirus A
28875	GCA_002674115.1	Rotavirus A
28875	GCA_002672295.1	Rotavirus A
28875	GCA_002676495.1	Rotavirus A
28875	GCA_002674135.1	Rotavirus A
28875	GCA_002681855.1	Rotavirus A
28875	GCA_002676155.1	Rotavirus A
28875	GCA_002672315.1	Rotavirus A
28875	GCA_002675815.1	Rotavirus A
28875	GCA_002675475.1	Rotavirus A
28875	GCA_002674155.1	Rotavirus A
28875	GCA_002675135.1	Rotavirus A
28875	GCA_002672335.1	Rotavirus A
28875	GCA_002674175.1	Rotavirus A
28875	GCA_002671935.1	Rotavirus A
28875	GCA_002672355.1	Rotavirus A
28875	GCA_002678895.1	Rotavirus A
28875	GCA_002674195.1	Rotavirus A
28875	GCA_002672375.1	Rotavirus A
28875	GCA_002666235.1	Rotavirus A
28875	GCA_002674215.1	Rotavirus A
28875	GCA_002672035.1	Rotavirus A
28875	GCA_002677195.1	Rotavirus A
28875	GCA_002674235.1	Rotavirus A
28875	GCA_002672415.1	Rotavirus A
28875	GCA_002666275.1	Rotavirus A
28875	GCA_002676515.1	Rotavirus A
28875	GCA_002674255.1	Rotavirus A
28875	GCA_002676175.1	Rotavirus A
28875	GCA_002672435.1	Rotavirus A
28875	GCA_002666295.1	Rotavirus A
28875	GCA_002675495.1	Rotavirus A
28875	GCA_002674275.1	Rotavirus A
28875	GCA_002675155.1	Rotavirus A
28875	GCA_002672455.1	Rotavirus A

28875	GCA_002676335.1	Rotavirus A
28875	GCA_002679935.1	Rotavirus A
28875	GCA_002670415.1	Rotavirus A
28875	GCA_002674295.1	Rotavirus A
28875	GCA_002672475.1	Rotavirus A
28875	GCA_002666335.1	Rotavirus A
28875	GCA_002678915.1	Rotavirus A
28875	GCA_002674315.1	Rotavirus A
28875	GCA_002678575.1	Rotavirus A
28875	GCA_002672495.1	Rotavirus A
28875	GCA_002676375.1	Rotavirus A
28875	GCA_002674335.1	Rotavirus A
28875	GCA_002672515.1	Rotavirus A
28875	GCA_002666375.1	Rotavirus A
28875	GCA_002677215.1	Rotavirus A
28875	GCA_002674355.1	Rotavirus A
28875	GCA_002672535.1	Rotavirus A
28875	GCA_002666395.1	Rotavirus A
28875	GCA_002676535.1	Rotavirus A
28875	GCA_002674375.1	Rotavirus A
28875	GCA_002668235.1	Rotavirus A
28875	GCA_002676195.1	Rotavirus A
28875	GCA_002668455.1	Rotavirus A
28875	GCA_002672555.1	Rotavirus A
28875	GCA_002666415.1	Rotavirus A
28875	GCA_002675515.1	Rotavirus A
28875	GCA_002674395.1	Rotavirus A
28875	GCA_002675175.1	Rotavirus A
28875	GCA_002672575.1	Rotavirus A
28875	GCA_002666435.1	Rotavirus A
28875	GCA_002672395.1	Rotavirus A
28875	GCA_002674415.1	Rotavirus A
28875	GCA_002672595.1	Rotavirus A
28875	GCA_002679275.1	Rotavirus A
28875	GCA_002676475.1	Rotavirus A
28875	GCA_002674435.1	Rotavirus A
28875	GCA_002672615.1	Rotavirus A
28875	GCA_002674455.1	Rotavirus A
28875	GCA_002672635.1	Rotavirus A
28875	GCA_002667555.1	Rotavirus A
28875	GCA_002677235.1	Rotavirus A
28875	GCA_002674475.1	Rotavirus A
28875	GCA_002672655.1	Rotavirus A
28875	GCA_002676555.1	Rotavirus A
28875	GCA_002674495.1	Rotavirus A
28875	GCA_002676215.1	Rotavirus A
28875	GCA_002678595.1	Rotavirus A
28875	GCA_002672675.1	Rotavirus A
28875	GCA_002675875.1	Rotavirus A
28875	GCA_002675535.1	Rotavirus A

28875	GCA_002674515.1	Rotavirus A
28875	GCA_002678615.1	Rotavirus A
28875	GCA_002675195.1	Rotavirus A
28875	GCA_002672695.1	Rotavirus A
28875	GCA_002676575.1	Rotavirus A
28875	GCA_002674535.1	Rotavirus A
28875	GCA_002682755.1	Rotavirus A
28875	GCA_002680375.1	Rotavirus A
28875	GCA_002672715.1	Rotavirus A
28875	GCA_002674555.1	Rotavirus A
28875	GCA_002678655.1	Rotavirus A
28875	GCA_002672735.1	Rotavirus A
28875	GCA_002674575.1	Rotavirus A
28875	GCA_002672755.1	Rotavirus A
28875	GCA_002667575.1	Rotavirus A
28875	GCA_002677255.1	Rotavirus A
28875	GCA_002674595.1	Rotavirus A
28875	GCA_002668675.1	Rotavirus A
28875	GCA_002666895.1	Rotavirus A
28875	GCA_002672775.1	Rotavirus A
28875	GCA_002676655.1	Rotavirus A
28875	GCA_002674615.1	Rotavirus A
28875	GCA_002676235.1	Rotavirus A
28875	GCA_002672795.1	Rotavirus A
28875	GCA_002681735.1	Rotavirus A
28875	GCA_002681835.1	Rotavirus A
28875	GCA_002674635.1	Rotavirus A
28875	GCA_002672815.1	Rotavirus A
28875	GCA_002666675.1	Rotavirus A
28875	GCA_002682895.1	Rotavirus A
28875	GCA_002674655.1	Rotavirus A
28875	GCA_002672835.1	Rotavirus A
28875	GCA_002676715.1	Rotavirus A
28875	GCA_002674675.1	Rotavirus A
28875	GCA_002672855.1	Rotavirus A
28875	GCA_002674695.1	Rotavirus A
28875	GCA_002672875.1	Rotavirus A
28875	GCA_002667595.1	Rotavirus A
28875	GCA_002670835.1	Rotavirus A
28875	GCA_002677275.1	Rotavirus A
28875	GCA_002674715.1	Rotavirus A
28875	GCA_002672895.1	Rotavirus A
28875	GCA_002676595.1	Rotavirus A
28875	GCA_002670855.1	Rotavirus A
28875	GCA_002674735.1	Rotavirus A
28875	GCA_002681875.1	Rotavirus A
28875	GCA_002676255.1	Rotavirus A
28875	GCA_002672915.1	Rotavirus A
28875	GCA_002682695.1	Rotavirus A
28875	GCA_002676795.1	Rotavirus A

28875	GCA_002682675.1	Rotavirus A
28875	GCA_002670875.1	Rotavirus A
28875	GCA_002674755.1	Rotavirus A
28875	GCA_002675235.1	Rotavirus A
28875	GCA_002672935.1	Rotavirus A
28875	GCA_002674775.1	Rotavirus A
28875	GCA_002672955.1	Rotavirus A
28875	GCA_002670915.1	Rotavirus A
28875	GCA_002674795.1	Rotavirus A
28875	GCA_002672975.1	Rotavirus A
28875	GCA_002670935.1	Rotavirus A
28875	GCA_002674815.1	Rotavirus A
28875	GCA_002668895.1	Rotavirus A
28875	GCA_002672995.1	Rotavirus A
28875	GCA_002667615.1	Rotavirus A
28875	GCA_002677295.1	Rotavirus A
28875	GCA_002674835.1	Rotavirus A
28875	GCA_002673015.1	Rotavirus A
28875	GCA_002674855.1	Rotavirus A
28875	GCA_002676275.1	Rotavirus A
28875	GCA_002673035.1	Rotavirus A
28875	GCA_002674875.1	Rotavirus A
28875	GCA_002675255.1	Rotavirus A
28875	GCA_002673055.1	Rotavirus A
28875	GCA_002674895.1	Rotavirus A
28875	GCA_002673075.1	Rotavirus A
28875	GCA_002674915.1	Rotavirus A
28875	GCA_002681655.1	Rotavirus A
28875	GCA_002678675.1	Rotavirus A
28875	GCA_002673095.1	Rotavirus A
28875	GCA_002674935.1	Rotavirus A
28875	GCA_002673115.1	Rotavirus A
28875	GCA_002677315.1	Rotavirus A
28875	GCA_002674955.1	Rotavirus A
28875	GCA_002669035.1	Rotavirus A
28875	GCA_002673135.1	Rotavirus A
28875	GCA_002676635.1	Rotavirus A
28875	GCA_002674975.1	Rotavirus A
28875	GCA_002676295.1	Rotavirus A
28875	GCA_002669055.1	Rotavirus A
28875	GCA_002673155.1	Rotavirus A
28875	GCA_002681815.1	Rotavirus A
28875	GCA_002675955.1	Rotavirus A
28875	GCA_002674995.1	Rotavirus A
28875	GCA_002669075.1	Rotavirus A
28875	GCA_002675275.1	Rotavirus A
28875	GCA_002673175.1	Rotavirus A
28875	GCA_002677055.1	Rotavirus A
28875	GCA_002671135.1	Rotavirus A
28875	GCA_002675015.1	Rotavirus A

28875	GCA_002679715.1	Rotavirus A
28875	GCA_002669095.1	Rotavirus A
28875	GCA_002673195.1	Rotavirus A
28875	GCA_002671155.1	Rotavirus A
28875	GCA_002675035.1	Rotavirus A
28875	GCA_002669115.1	Rotavirus A
28875	GCA_002678695.1	Rotavirus A
28875	GCA_002673215.1	Rotavirus A
28875	GCA_002678355.1	Rotavirus A
28875	GCA_002671175.1	Rotavirus A
28875	GCA_002673235.1	Rotavirus A
28875	GCA_002667655.1	Rotavirus A
28875	GCA_002671195.1	Rotavirus A
28875	GCA_002677335.1	Rotavirus A
28875	GCA_002675075.1	Rotavirus A
28875	GCA_002673255.1	Rotavirus A
28875	GCA_002671215.1	Rotavirus A
28875	GCA_002675095.1	Rotavirus A
28875	GCA_002676315.1	Rotavirus A
28875	GCA_002673275.1	Rotavirus A
28875	GCA_002671235.1	Rotavirus A
28875	GCA_002675115.1	Rotavirus A
28875	GCA_002667155.1	Rotavirus A
28875	GCA_002671255.1	Rotavirus A
28875	GCA_002682775.1	Rotavirus A
28875	GCA_002673295.1	Rotavirus A
28875	GCA_002667175.1	Rotavirus A
28875	GCA_002671275.1	Rotavirus A
28875	GCA_002679055.1	Rotavirus A
28875	GCA_002678715.1	Rotavirus A
28875	GCA_002673315.1	Rotavirus A
28875	GCA_002671295.1	Rotavirus A
28875	GCA_002668015.1	Rotavirus A
28875	GCA_002667675.1	Rotavirus A
28875	GCA_002671315.1	Rotavirus A
28875	GCA_002666995.1	Rotavirus A
28875	GCA_002673355.1	Rotavirus A
28875	GCA_002678975.1	Rotavirus A
28875	GCA_002680595.1	Rotavirus A
28875	GCA_002676675.1	Rotavirus A
28875	GCA_002675215.1	Rotavirus A
28875	GCA_002666315.1	Rotavirus A
28875	GCA_002675995.1	Rotavirus A
28875	GCA_002681755.1	Rotavirus A
28875	GCA_002671355.1	Rotavirus A
28875	GCA_002675315.1	Rotavirus A
28875	GCA_002673375.1	Rotavirus A
28875	GCA_002671375.1	Rotavirus A
28875	GCA_002669335.1	Rotavirus A
28875	GCA_002673395.1	Rotavirus A

28875	GCA_002671395.1	Rotavirus A
28875	GCA_002678735.1	Rotavirus A
28875	GCA_002673415.1	Rotavirus A
28875	GCA_002678395.1	Rotavirus A
28875	GCA_002671415.1	Rotavirus A
28875	GCA_002675295.1	Rotavirus A
28875	GCA_002673435.1	Rotavirus A
28875	GCA_002667695.1	Rotavirus A
28875	GCA_002677355.1	Rotavirus A
28875	GCA_002671435.1	Rotavirus A
28875	GCA_002667015.1	Rotavirus A
28875	GCA_002673455.1	Rotavirus A
28875	GCA_002676695.1	Rotavirus A
28875	GCA_002671455.1	Rotavirus A
28875	GCA_002676355.1	Rotavirus A
28875	GCA_002676015.1	Rotavirus A
28875	GCA_002671475.1	Rotavirus A
28875	GCA_002675675.1	Rotavirus A
28875	GCA_002675335.1	Rotavirus A
28875	GCA_002673475.1	Rotavirus A
28875	GCA_002678415.1	Rotavirus A
28875	GCA_002671495.1	Rotavirus A
28875	GCA_002669455.1	Rotavirus A
28875	GCA_002673495.1	Rotavirus A
28875	GCA_002682715.1	Rotavirus A
28875	GCA_002671515.1	Rotavirus A
28875	GCA_002679095.1	Rotavirus A
28875	GCA_002669475.1	Rotavirus A
28875	GCA_002678475.1	Rotavirus A
28875	GCA_002673515.1	Rotavirus A
28875	GCA_002678495.1	Rotavirus A
28875	GCA_002671535.1	Rotavirus A
28875	GCA_002675415.1	Rotavirus A
28875	GCA_002678515.1	Rotavirus A
28875	GCA_002667715.1	Rotavirus A
28875	GCA_002667455.1	Rotavirus A
28875	GCA_002678535.1	Rotavirus A
28875	GCA_002678555.1	Rotavirus A
28875	GCA_002667035.1	Rotavirus A
28875	GCA_002673535.1	Rotavirus A
28875	GCA_002677495.1	Rotavirus A
28875	GCA_002671575.1	Rotavirus A
28875	GCA_002675455.1	Rotavirus A
28875	GCA_002666355.1	Rotavirus A
28875	GCA_002669535.1	Rotavirus A
28875	GCA_002667495.1	Rotavirus A
28875	GCA_002671595.1	Rotavirus A
28875	GCA_002675695.1	Rotavirus A
28875	GCA_002681695.1	Rotavirus A
28875	GCA_002678635.1	Rotavirus A

28875	GCA_002675355.1	Rotavirus A
28875	GCA_002673575.1	Rotavirus A
28875	GCA_002667515.1	Rotavirus A
28875	GCA_002671615.1	Rotavirus A
28875	GCA_002667535.1	Rotavirus A
28875	GCA_002671635.1	Rotavirus A
28875	GCA_002679115.1	Rotavirus A
28875	GCA_002669595.1	Rotavirus A
28875	GCA_002678775.1	Rotavirus A
28875	GCA_002673595.1	Rotavirus A
28875	GCA_002678435.1	Rotavirus A
28875	GCA_002671655.1	Rotavirus A
28875	GCA_002678755.1	Rotavirus A
28875	GCA_002673615.1	Rotavirus A
28875	GCA_002671675.1	Rotavirus A
28875	GCA_002675555.1	Rotavirus A
28875	GCA_002681915.1	Rotavirus A
28875	GCA_002667055.1	Rotavirus A
28875	GCA_002673635.1	Rotavirus A
28875	GCA_002676735.1	Rotavirus A
28875	GCA_002681715.1	Rotavirus A
28875	GCA_002681795.1	Rotavirus A
28875	GCA_002676395.1	Rotavirus A
28875	GCA_002673655.1	Rotavirus A
28875	GCA_002681675.1	Rotavirus A
28875	GCA_002676055.1	Rotavirus A
28875	GCA_002678855.1	Rotavirus A
28875	GCA_002671715.1	Rotavirus A
28875	GCA_002675715.1	Rotavirus A
28875	GCA_002675595.1	Rotavirus A
28875	GCA_002675375.1	Rotavirus A
28875	GCA_002673675.1	Rotavirus A
28875	GCA_002667635.1	Rotavirus A
28875	GCA_002671735.1	Rotavirus A
28875	GCA_002675615.1	Rotavirus A
28875	GCA_002678935.1	Rotavirus A
28875	GCA_002671755.1	Rotavirus A
28875	GCA_002675635.1	Rotavirus A
28875	GCA_002678955.1	Rotavirus A
28875	GCA_002678795.1	Rotavirus A
28875	GCA_002673695.1	Rotavirus A
28875	GCA_002678455.1	Rotavirus A
28875	GCA_002668095.1	Rotavirus A
28875	GCA_002678995.1	Rotavirus A
28875	GCA_002673715.1	Rotavirus A
28875	GCA_002667755.1	Rotavirus A
28875	GCA_002677715.1	Rotavirus A
28875	GCA_002679015.1	Rotavirus A
28875	GCA_002679035.1	Rotavirus A
28875	GCA_002667075.1	Rotavirus A

28875	GCA_002673735.1	Rotavirus A
28875	GCA_002676755.1	Rotavirus A
28875	GCA_002671815.1	Rotavirus A
28875	GCA_002681975.1	Rotavirus A
28875	GCA_002676415.1	Rotavirus A
28875	GCA_002679075.1	Rotavirus A
28875	GCA_002673755.1	Rotavirus A
28875	GCA_002667735.1	Rotavirus A
28875	GCA_002671835.1	Rotavirus A
28875	GCA_002675055.1	Rotavirus A
28875	GCA_002671855.1	Rotavirus A
28875	GCA_002675735.1	Rotavirus A
28875	GCA_002682795.1	Rotavirus A
28875	GCA_002673775.1	Rotavirus A
28875	GCA_002679495.1	Rotavirus A
28875	GCA_002671875.1	Rotavirus A
28875	GCA_002665735.1	Rotavirus A
28875	GCA_002678815.1	Rotavirus A
28875	GCA_002673795.1	Rotavirus A
28875	GCA_002671895.1	Rotavirus A
28875	GCA_002668115.1	Rotavirus A
28875	GCA_002667775.1	Rotavirus A
28875	GCA_002667815.1	Rotavirus A
28875	GCA_002671915.1	Rotavirus A
28875	GCA_002667095.1	Rotavirus A
28875	GCA_002673815.1	Rotavirus A
28875	GCA_002667835.1	Rotavirus A
28875	GCA_002676775.1	Rotavirus A
28875	GCA_002681955.1	Rotavirus A
28875	GCA_002676435.1	Rotavirus A
28875	GCA_002673835.1	Rotavirus A
28875	GCA_002667855.1	Rotavirus A
28875	GCA_002681775.1	Rotavirus A
28875	GCA_002671955.1	Rotavirus A
28875	GCA_002675755.1	Rotavirus A
28875	GCA_002675835.1	Rotavirus A
28875	GCA_002673855.1	Rotavirus A
28875	GCA_002667875.1	Rotavirus A
28875	GCA_002671975.1	Rotavirus A
28875	GCA_002675855.1	Rotavirus A
28875	GCA_002673875.1	Rotavirus A
28875	GCA_002671995.1	Rotavirus A
28875	GCA_002678835.1	Rotavirus A
28875	GCA_002677935.1	Rotavirus A
28875	GCA_002675895.1	Rotavirus A
28875	GCA_002668135.1	Rotavirus A
28875	GCA_002673895.1	Rotavirus A
28875	GCA_002667795.1	Rotavirus A
28875	GCA_002675395.1	Rotavirus A
28875	GCA_002681895.1	Rotavirus A

28875	GCA_002675915.1	Rotavirus A
28875	GCA_002667115.1	Rotavirus A
28875	GCA_002673915.1	Rotavirus A
28875	GCA_002672055.1	Rotavirus A
28875	GCA_002675935.1	Rotavirus A
28875	GCA_002676455.1	Rotavirus A
28875	GCA_002673935.1	Rotavirus A
28875	GCA_002672075.1	Rotavirus A
28875	GCA_002675775.1	Rotavirus A
28875	GCA_002675435.1	Rotavirus A
28875	GCA_002673955.1	Rotavirus A
28875	GCA_002672095.1	Rotavirus A
28875	GCA_002675975.1	Rotavirus A
28875	GCA_002673975.1	Rotavirus A
28875	GCA_002667355.1	Rotavirus A
28875	GCA_002682735.1	Rotavirus A
28875	GCA_002672115.1	Rotavirus A
28875	GCA_002675575.1	Rotavirus A
28875	GCA_002672135.1	Rotavirus A
28875	GCA_002668155.1	Rotavirus A
28875	GCA_002668055.1	Rotavirus A
28875	GCA_002672155.1	Rotavirus A
28875	GCA_002676035.1	Rotavirus A
28875	GCA_002675655.1	Rotavirus A
28875	GCA_002667135.1	Rotavirus A
28875	GCA_002674015.1	Rotavirus A
28875	GCA_002676815.1	Rotavirus A
28875	GCA_002672175.1	Rotavirus A
28875	GCA_002666455.1	Rotavirus A
28875	GCA_002674035.1	Rotavirus A
28875	GCA_002672195.1	Rotavirus A
28875	GCA_002675795.1	Rotavirus A
28875	GCA_002676075.1	Rotavirus A
28875	GCA_002672215.1	Rotavirus A
28875	GCA_002676095.1	Rotavirus A
28875	GCA_002682875.1	Rotavirus A
28875	GCA_002674055.1	Rotavirus A
28875	GCA_002671695.1	Rotavirus A
28875	GCA_002678155.1	Rotavirus A
28875	GCA_002676115.1	Rotavirus A
28875	GCA_002678875.1	Rotavirus A
28875	GCA_002674075.1	Rotavirus A
28875	GCA_002672255.1	Rotavirus A
28875	GCA_002676135.1	Rotavirus A
28875	GCA_002674095.1	Rotavirus A
28875	GCA_003005445.1	Rotavirus A
28875	GCA_003005455.1	Rotavirus A
28875	GCA_003005475.1	Rotavirus A
28875	GCA_003005485.1	Rotavirus A
28875	GCA_003005495.1	Rotavirus A

28875	GCA_003005505.1	Rotavirus A
28875	GCA_003004975.1	Rotavirus A
28875	GCA_003005515.1	Rotavirus A
28875	GCA_003005525.1	Rotavirus A
28875	GCA_003005535.1	Rotavirus A
28875	GCA_003005545.1	Rotavirus A
28875	GCA_003005555.1	Rotavirus A
28875	GCA_003005465.1	Rotavirus A
28875	GCA_003005565.1	Rotavirus A
28875	GCA_003005575.1	Rotavirus A
28875	GCA_003005585.1	Rotavirus A
28875	GCA_003005595.1	Rotavirus A
28875	GCA_003005605.1	Rotavirus A
28875	GCA_003005615.1	Rotavirus A
28875	GCA_003071145.1	Rotavirus A
28875	GCA_003005625.1	Rotavirus A
28875	GCA_003005635.1	Rotavirus A
28875	GCA_003005645.1	Rotavirus A
28875	GCA_003005655.1	Rotavirus A
28875	GCA_003005745.1	Rotavirus A
28875	GCA_003005015.1	Rotavirus A
28875	GCA_003005025.1	Rotavirus A
28875	GCA_003071125.1	Rotavirus A
28875	GCA_003004925.1	Rotavirus A
28875	GCA_003004935.1	Rotavirus A
28875	GCA_003004945.1	Rotavirus A
28875	GCA_003004955.1	Rotavirus A
28875	GCA_003004965.1	Rotavirus A
28875	GCA_003071165.1	Rotavirus A
28875	GCA_003005035.1	Rotavirus A
28875	GCA_003005045.1	Rotavirus A
28875	GCA_003005055.1	Rotavirus A
28875	GCA_003005065.1	Rotavirus A
28875	GCA_003005075.1	Rotavirus A
28875	GCA_003005085.1	Rotavirus A
28875	GCA_003005095.1	Rotavirus A
28875	GCA_003005105.1	Rotavirus A
28875	GCA_003005115.1	Rotavirus A
28875	GCA_003005125.1	Rotavirus A
28875	GCA_003071205.1	Rotavirus A
28875	GCA_003005135.1	Rotavirus A
28875	GCA_003005145.1	Rotavirus A
28875	GCA_003005155.1	Rotavirus A
28875	GCA_003005165.1	Rotavirus A
28875	GCA_003005175.1	Rotavirus A
28875	GCA_003005185.1	Rotavirus A
28875	GCA_003005195.1	Rotavirus A
28875	GCA_003005205.1	Rotavirus A
28875	GCA_003005215.1	Rotavirus A
28875	GCA_003005225.1	Rotavirus A

28875	GCA_003005235.1	Rotavirus A
28875	GCA_003071225.1	Rotavirus A
28875	GCA_003005245.1	Rotavirus A
28875	GCA_003005255.1	Rotavirus A
28875	GCA_003005265.1	Rotavirus A
28875	GCA_003005275.1	Rotavirus A
28875	GCA_003005285.1	Rotavirus A
28875	GCA_003005295.1	Rotavirus A
28875	GCA_003005305.1	Rotavirus A
28875	GCA_003005315.1	Rotavirus A
28875	GCA_003005325.1	Rotavirus A
28875	GCA_003005335.1	Rotavirus A
28875	GCA_003005345.1	Rotavirus A
28875	GCA_003005355.1	Rotavirus A
28875	GCA_003005365.1	Rotavirus A
28875	GCA_003005375.1	Rotavirus A
28875	GCA_003005385.1	Rotavirus A
28875	GCA_003005395.1	Rotavirus A
28875	GCA_003005405.1	Rotavirus A
28875	GCA_003005415.1	Rotavirus A
28875	GCA_003005425.1	Rotavirus A
28875	GCA_003005435.1	Rotavirus A
28875	GCA_003258275.1	Rotavirus A
28875	GCA_003259065.1	Rotavirus A
28875	GCA_003259125.1	Rotavirus A
28875	GCA_003259045.1	Rotavirus A
28875	GCA_003258255.1	Rotavirus A
28875	GCA_003259105.1	Rotavirus A
666965	GCA_002634475.1	Rotavirus A 1290xUK reassortant (UKg91290)
666965	GCA_002682595.1	Rotavirus A 1290xUK reassortant (UKg91290)
666966	GCA_002635635.1	Rotavirus A AU32xUK reassortant (UKg9AU32)
666966	GCA_002635875.1	Rotavirus A AU32xUK reassortant (UKg9AU32)
666966	GCA_002636115.1	Rotavirus A AU32xUK reassortant (UKg9AU32)
666967	GCA_002642615.1	Rotavirus A DS-1xUK reassortant (UKg9DS-1)
666967	GCA_002642835.1	Rotavirus A DS-1xUK reassortant (UKg9DS-1)
666967	GCA_002643055.1	Rotavirus A DS-1xUK reassortant (UKg9DS-1)
666968	GCA_002643955.1	Rotavirus A DxUK reassortant (UKg9D)
666968	GCA_002644395.1	Rotavirus A DxUK reassortant (UKg9D)
666968	GCA_002644175.1	Rotavirus A DxUK reassortant (UKg9D)
672377	GCA_002644615.1	Rotavirus A EC2184/ECU/G11P[6]
1056490	GCA_002647295.1	Rotavirus A HC91xUK reassortant (UKg9KC-1)
1182925	GCA_002647735.1	Rotavirus A Hu/BEL/BE2001/2009/G9P[6]
1146934	GCA_002647975.1	Rotavirus A Hu/BEL/F01322/2009/G3P[6]
1146935	GCA_002648195.1	Rotavirus A Hu/BEL/F01498/2009/G3P[6]
1148771	GCA_002637075.1	Rotavirus A Hu/CI-81/2011/KOR
1146936	GCA_002648415.1	Rotavirus A Hu/USA/06-242/2006/G2P[6]
641313	GCA_002662595.1	Rotavirus A human/Bethesda/CH5446/1991/G3P[8]
641314	GCA_002662615.1	Rotavirus A human/Bethesda/CH5455/1991/G3P[8]
641315	GCA_002662655.1	Rotavirus A human/Bethesda/CH5459/1991/G3P[8]
641316	GCA_002662675.1	Rotavirus A human/Bethesda/CH5470/1991/G3P[8]

641317	GCA_002662695.1	Rotavirus A human/Bethesda/CH5475/1991/G3P[8]
641318	GCA_002662715.1	Rotavirus A human/Bethesda/CH5477/1991/G3P[8]
641319	GCA_002662735.1	Rotavirus A human/Bethesda/CH5483/1991/G3P[8]
641320	GCA_002662755.1	Rotavirus A human/Bethesda/CH5484/1991/G3P[8]
641321	GCA_002662775.1	Rotavirus A human/Bethesda/CH5488/1991/G3P[8]
641322	GCA_002662795.1	Rotavirus A human/Bethesda/CH5498/1991/G3P[8]
757016	GCA_002662835.1	Rotavirus A human/Bethesda/DC1208/1980/G4P[8]
641323	GCA_002662875.1	Rotavirus A human/Bethesda/DC1285/1980/G4P[8]
641324	GCA_002662895.1	Rotavirus A human/Bethesda/DC129/1976/G3P[8]
641325	GCA_002662915.1	Rotavirus A human/Bethesda/DC130/1976/G3P[8]
641326	GCA_002662935.1	Rotavirus A human/Bethesda/DC131/1976/G3P[8]
641327	GCA_002662955.1	Rotavirus A human/Bethesda/DC133/1976/G3P[8]
641328	GCA_002662975.1	Rotavirus A human/Bethesda/DC135/1979/G3P[8]
757017	GCA_002662995.1	Rotavirus A human/Bethesda/DC1359/1980/G4P[8]
641329	GCA_002663015.1	Rotavirus A human/Bethesda/DC139/1976/G3P[8]
641330	GCA_002663035.1	Rotavirus A human/Bethesda/DC140/1975/G3P[8]
641331	GCA_002663055.1	Rotavirus A human/Bethesda/DC1455/1975/G3P[8]
641332	GCA_002663095.1	Rotavirus A human/Bethesda/DC1494/1976/G3P[8]
641333	GCA_002663115.1	Rotavirus A human/Bethesda/DC1496/1976/G3P[8]
641334	GCA_002663135.1	Rotavirus A human/Bethesda/DC1497/1976/G3P[8]
641335	GCA_002663155.1	Rotavirus A human/Bethesda/DC1505/1976/G3P[8]
641336	GCA_002663175.1	Rotavirus A human/Bethesda/DC1563/1974/G3P[8]
641337	GCA_002663195.1	Rotavirus A human/Bethesda/DC1600/1980/G3P[8]
641338	GCA_002663215.1	Rotavirus A human/Bethesda/DC168/1976/G3P[8]
641339	GCA_002663235.1	Rotavirus A human/Bethesda/DC1730/1979/G3P[8]
641340	GCA_002663255.1	Rotavirus A human/Bethesda/DC1898/1976/G3P[8]
641341	GCA_002663275.1	Rotavirus A human/Bethesda/DC2069/1976/G3P[8]
641342	GCA_002663315.1	Rotavirus A human/Bethesda/DC2081/1976/G3P[8]
641343	GCA_002663335.1	Rotavirus A human/Bethesda/DC2102/1976/G3P[8]
641344	GCA_002663355.1	Rotavirus A human/Bethesda/DC2106/1976/G3P[8]
641345	GCA_002663375.1	Rotavirus A human/Bethesda/DC2109/1976/G3P[8]
641346	GCA_002663395.1	Rotavirus A human/Bethesda/DC2114/1976/G3P[8]
641347	GCA_002663415.1	Rotavirus A human/Bethesda/DC2119/1976/G3P[8]
641348	GCA_002663435.1	Rotavirus A human/Bethesda/DC2171/1976/G3P[8]
641349	GCA_002663455.1	Rotavirus A human/Bethesda/DC2212/1976/G3P[8]
641350	GCA_002663475.1	Rotavirus A human/Bethesda/DC2238/1976/G3P[8]
641351	GCA_002663495.1	Rotavirus A human/Bethesda/DC2239/1976/G3P[8]
757018	GCA_002663555.1	Rotavirus A human/Bethesda/DC2241/1977/G4P[8]
641352	GCA_002663575.1	Rotavirus A human/Bethesda/DC2262/1976/G3P[8]
641353	GCA_002663595.1	Rotavirus A human/Bethesda/DC2266/1976/G3P[8]
641354	GCA_002663615.1	Rotavirus A human/Bethesda/DC23/1976/G3P[8]
757019	GCA_002663675.1	Rotavirus A human/Bethesda/DC4320/1988/G4P[8]
757020	GCA_002663695.1	Rotavirus A human/Bethesda/DC4608/1980/G4P[8]
757021	GCA_002663715.1	Rotavirus A human/Bethesda/DC4613/1980/G4P[8]
641355	GCA_002663735.1	Rotavirus A human/Bethesda/DC4772/1976/G3P[8]
757022	GCA_002663775.1	Rotavirus A human/Bethesda/DC4996/1977/G4P[8]
757023	GCA_002663795.1	Rotavirus A human/Bethesda/DC5064/1977/G4P[8]
757024	GCA_002663815.1	Rotavirus A human/Bethesda/DC5115/1977/G4P[8]
641356	GCA_002663835.1	Rotavirus A human/Bethesda/DC5142/1975/G3P[8]
641357	GCA_002663855.1	Rotavirus A human/Bethesda/DC5491/1991/G3P[8]

641358	GCA_002663875.1	Rotavirus A human/Bethesda/DC5544/1991/G3P[8]
641359	GCA_002663895.1	Rotavirus A human/Bethesda/DC5549/1991/G3P[8]
641360	GCA_002663915.1	Rotavirus A human/Bethesda/DC5553/1991/G3P[8]
641361	GCA_002663935.1	Rotavirus A human/Bethesda/DC5710/1991/G3P[8]
641362	GCA_002663955.1	Rotavirus A human/Bethesda/DC5728/1991/G3P[8]
641363	GCA_002663995.1	Rotavirus A human/Bethesda/DC5751/1991/G3P[8]
641364	GCA_002664035.1	Rotavirus A human/Bethesda/DC792/1980/G3P[8]
757025	GCA_002664075.1	Rotavirus A human/Bethesda/DC827/1978/G4P[8]
756991	GCA_002664095.1	Rotavirus A human/USA/2009727032/2009/G9P[8]
1004944	GCA_002664115.1	Rotavirus A human/Vanderbilt/VU05-06-72/2005/G12P[8]
1004945	GCA_002664135.1	Rotavirus A human/Vanderbilt/VU05-06-74/2005/G12P[8]
1004941	GCA_002664155.1	Rotavirus A human/Vanderbilt/VU08-09-16/2008/G3P[8]
1004942	GCA_002664175.1	Rotavirus A human/Vanderbilt/VU08-09-20/2008/G3P[8]
1004943	GCA_002664215.1	Rotavirus A human/Vanderbilt/VU08-09-27/2008/G3P[8]
1004946	GCA_002664235.1	Rotavirus A human/Vanderbilt/VU08-09-39/2008/G12P[8]
1004793	GCA_002664255.1	Rotavirus A human/Victoria/CK00001/2004/G1P[8]
1004794	GCA_002664275.1	Rotavirus A human/Victoria/CK00002/2004/G1P[8]
1004795	GCA_002664295.1	Rotavirus A human/Victoria/CK00003/2004/G1P[8]
1004796	GCA_002664315.1	Rotavirus A human/Victoria/CK00004/2004/G1P[8]
1004797	GCA_002664335.1	Rotavirus A human/Victoria/CK00005/2004/G1P[8]
1004798	GCA_002664355.1	Rotavirus A human/Victoria/CK00006/2004/G1P[8]
1004799	GCA_002664375.1	Rotavirus A human/Victoria/CK00007/2004/G1P[8]
1004800	GCA_002664395.1	Rotavirus A human/Victoria/CK00008/2004/G1P[8]
1004801	GCA_002664435.1	Rotavirus A human/Victoria/CK00009/2004/G1P[8]
1004802	GCA_002664455.1	Rotavirus A human/Victoria/CK00011/2004/G1P[8]
1004803	GCA_002664475.1	Rotavirus A human/Victoria/CK00012/2004/G1P[8]
1004804	GCA_002664495.1	Rotavirus A human/Victoria/CK00014/2004/G1P[8]
1004805	GCA_002664515.1	Rotavirus A human/Victoria/CK00015/2005/G1P[8]
1004806	GCA_002664535.1	Rotavirus A human/Victoria/CK00016/2005/G1P[8]
1004807	GCA_002664555.1	Rotavirus A human/Victoria/CK00017/2005/G1P[8]
1004808	GCA_002664575.1	Rotavirus A human/Victoria/CK00018/2005/G1P[8]
1004809	GCA_002664595.1	Rotavirus A human/Victoria/CK00019/2005/G1P[8]
1004810	GCA_002664615.1	Rotavirus A human/Victoria/CK00020/2005/G1P[8]
1004811	GCA_002664655.1	Rotavirus A human/Victoria/CK00021/2005/G1P[8]
1004812	GCA_002664675.1	Rotavirus A human/Victoria/CK00022/2005/G1P[8]
1004813	GCA_002664695.1	Rotavirus A human/Victoria/CK00023/2005/G1P[8]
1004814	GCA_002664715.1	Rotavirus A human/Victoria/CK00024/2005/G1P[8]
1004815	GCA_002664735.1	Rotavirus A human/Victoria/CK00026/2005/G1P[8]
1004816	GCA_002664755.1	Rotavirus A human/Victoria/CK00027/2005/G1P[8]
1004817	GCA_002664775.1	Rotavirus A human/Victoria/CK00028/2005/G1P[8]
1004818	GCA_002664795.1	Rotavirus A human/Victoria/CK00029/2006/G1P[8]
1004819	GCA_002664815.1	Rotavirus A human/Victoria/CK00030/2006/G1P[8]
1004820	GCA_002664835.1	Rotavirus A human/Victoria/CK00032/2006/G1P[8]
1004821	GCA_002664875.1	Rotavirus A human/Victoria/CK00033/2007/G1P[8]
1004822	GCA_002664895.1	Rotavirus A human/Victoria/CK00034/2007/G1P[8]
1004823	GCA_002664915.1	Rotavirus A human/Victoria/CK00035/2005/G1P[8]
1004824	GCA_002664935.1	Rotavirus A human/Victoria/CK00036/2005/G1P[8]
1004825	GCA_002664955.1	Rotavirus A human/Victoria/CK00037/2006/G1P[8]
1004826	GCA_002664975.1	Rotavirus A human/Victoria/CK00039/2006/G1P[8]
1004827	GCA_002664995.1	Rotavirus A human/Victoria/CK00040/2006/G1P[8]

1004828	GCA_002665015.1	Rotavirus A human/Victoria/CK00041/2006/G1P[8]
1004829	GCA_002665035.1	Rotavirus A human/Victoria/CK00043/2006/G1P[8]
1004830	GCA_002665055.1	Rotavirus A human/Victoria/CK00045/2006/G1P[8]
1004831	GCA_002665095.1	Rotavirus A human/Victoria/CK00046/2006/G1P[8]
1004832	GCA_002665115.1	Rotavirus A human/Victoria/CK00047/2006/G1P[8]
1004833	GCA_002665135.1	Rotavirus A human/Victoria/CK00048/2004/G1P[8]
1004834	GCA_002665155.1	Rotavirus A human/Victoria/CK00049/2004/G1P[8]
1004835	GCA_002665175.1	Rotavirus A human/Victoria/CK00050/2005/G1P[8]
1349389	GCA_002662535.1	Rotavirus A human-wt/USA/LB1562/2010/G9P4
1179678	GCA_002648855.1	Rotavirus A IAL28xUK reassortant (UKg9IAL28)
1056489	GCA_002649955.1	Rotavirus A KC-1xUK reassortant (UKg9KC-1)
1795042	GCA_002665415.1	Rotavirus A pheasant-wt/HUN/216/2015/G23P[37]
757426	GCA_002666975.1	Rotavirus A pig/India/RU172/G12P[7]
666969	GCA_002666575.1	Rotavirus A PxUK reassortant (UKg9P)
666969	GCA_002666555.1	Rotavirus A PxUK reassortant (UKg9P)
1835657	GCA_002667275.1	Rotavirus A RVA/Cow-tc/JPN/KK3/1983/G10P[11]
1835655	GCA_002667315.1	Rotavirus A RVA/Cow-tc/THA/61A/1989/G10P[5]
1835658	GCA_002667375.1	Rotavirus A RVA/Cow-tc/THA/A44/1989/G10P[11]
1835659	GCA_002667395.1	Rotavirus A RVA/Cow-tc/THA/A5-10/1988/G8P[1]
1835660	GCA_002667415.1	Rotavirus A RVA/Cow-tc/THA/A5-13/1988/G8P[1]
1835656	GCA_002667435.1	Rotavirus A RVA/Cow-tc/USA/B223/1983/G10P[11]
1193398	GCA_002659475.1	Rotavirus A RVA/Human/NCA/125L/2010/G3P[8]
1193386	GCA_002659495.1	Rotavirus A RVA/Human/NCA/18J/2010/G1P[8]
1193387	GCA_002659515.1	Rotavirus A RVA/Human/NCA/22J/2010/G1P[8]
1193388	GCA_002659555.1	Rotavirus A RVA/Human/NCA/24J/2010/G1P[8]
1193389	GCA_002659575.1	Rotavirus A RVA/Human/NCA/25J/2010/G1P[8]
1193390	GCA_002659595.1	Rotavirus A RVA/Human/NCA/26J/2010/G1P[8]
1193391	GCA_002659615.1	Rotavirus A RVA/Human/NCA/28J/2010/G1P[8]
1193392	GCA_002659635.1	Rotavirus A RVA/Human/NCA/41J/2010/G1P[8]
1193393	GCA_002659655.1	Rotavirus A RVA/Human/NCA/45J/2010/G1P[8]
1193395	GCA_002659695.1	Rotavirus A RVA/Human/NCA/72J/2010/G1P[8]
1193396	GCA_002659715.1	Rotavirus A RVA/Human/NCA/7J/2010/G1P[8]
1193397	GCA_002659735.1	Rotavirus A RVA/Human/NCA/9J/2010/G1P[8]
1193399	GCA_002659775.1	Rotavirus A RVA/Human/NCA/OL/2010/G4P[6]
1193401	GCA_002659675.1	Rotavirus A RVA/Human/NCA64J/2010/G3P[8]
1307161	GCA_002640235.1	Rotavirus A RVA/Human-wt/ITA/ASTI23/2007/G9P8
1307162	GCA_002640255.1	Rotavirus A RVA/Human-wt/ITA/AV21/2010/G9P8
1307163	GCA_002640275.1	Rotavirus A RVA/Human-wt/ITA/AV28/2010/G9P8
1307165	GCA_002640295.1	Rotavirus A RVA/Human-wt/ITA/JES11/2010/G9P8
1973203	GCA_002641195.1	Rotavirus A RVA/Human-wt/JPN/CH1020/2016/G2P[4]
1973207	GCA_002641215.1	Rotavirus A RVA/Human-wt/JPN/CH1023/2016/G9P[8]
1973200	GCA_002641435.1	Rotavirus A RVA/Human-wt/JPN/IS1078/2015/G3P[8]
1973208	GCA_002641455.1	Rotavirus A RVA/Human-wt/JPN/IS1080/2016/G9P[8]
1973201	GCA_002641475.1	Rotavirus A RVA/Human-wt/JPN/IS1090/2016/G3P[8]
1973205	GCA_002641495.1	Rotavirus A RVA/Human-wt/JPN/K-21-16/2016/G2P[4]
1973204	GCA_002641535.1	Rotavirus A RVA/Human-wt/JPN/K-3-16/2016/G2P[4]
1973202	GCA_002641555.1	Rotavirus A RVA/Human-wt/JPN/MI1125/2016/G3P[8]
1973209	GCA_002641575.1	Rotavirus A RVA/Human-wt/JPN/MI1128/2016/G9P[8]
1973206	GCA_002641595.1	Rotavirus A RVA/Human-wt/JPN/MI1132/2016/G2P[4]
1933901	GCA_002643815.1	Rotavirus A RVA/Human-wt/NPL/07N1760/2007/G26P[19

1907161	GCA_002646335.1	Rotavirus A RVA/Human-wt/THA/KKL-117/2014/G9P[23]
1882325	GCA_002646355.1	Rotavirus A RVA/Human-wt/THA/KKL-17/2013/G8P[8]
1882337	GCA_002646415.1	Rotavirus A RVA/Human-wt/THA/LS-202/2014/G2P[4]
1882338	GCA_002646435.1	Rotavirus A RVA/Human-wt/THA/LS-L7/2014/G2P[4]
1882332	GCA_002646455.1	Rotavirus A RVA/Human-wt/THA/NP-130/2014/G8P[8]
1882329	GCA_002646495.1	Rotavirus A RVA/Human-wt/THA/PCB-103/2013/G8P[8]
1882333	GCA_002646555.1	Rotavirus A RVA/Human-wt/THA/PCB-656/2014/G8P[8]
1882326	GCA_002646575.1	Rotavirus A RVA/Human-wt/THA/PCB-79/2013/G8P[8]
1882327	GCA_002646605.1	Rotavirus A RVA/Human-wt/THA/PCB-84/2013/G8P[8]
1882328	GCA_002646655.1	Rotavirus A RVA/Human-wt/THA/PCB-85/2013/G8P[8]
1882330	GCA_002646675.1	Rotavirus A RVA/Human-wt/THA/SKT-107/2013/G8P[8]
1882334	GCA_002646795.1	Rotavirus A RVA/Human-wt/THA/SKT-457/2014/G8P[8]
1882335	GCA_002646875.1	Rotavirus A RVA/Human-wt/THA/SSKT-269/2014/G8P[8]
1882336	GCA_002646915.1	Rotavirus A RVA/Human-wt/THA/SSL-55/2014/G8P[8]
1882331	GCA_002646935.1	Rotavirus A RVA/Human-wt/THA/SWL-12/2013/G8P[8]
1954144	GCA_002655315.1	Rotavirus A RVA/Human-wt/VNM/SP127/2013/G1P[4]
1954145	GCA_002655335.1	Rotavirus A RVA/Human-wt/VNM/SP193/2013/G1P[4]
1307168	GCA_002659935.1	Rotavirus A RVA/Pig-wt/ITA/2CR/2009/G9P[23]
1307169	GCA_002659955.1	Rotavirus A RVA/Pig-wt/ITA/3BS/2009/G9P[23]
1307170	GCA_002659995.1	Rotavirus A RVA/Pig-wt/ITA/7RE/2009/G9P[23]
666970	GCA_002660955.1	Rotavirus A ST3xUK reassortant (UKg9ST3)
666970	GCA_002660975.1	Rotavirus A ST3xUK reassortant (UKg9ST3)
638299	GCA_002682375.1	Rotavirus A strain 116E/AG
1179680	GCA_002661195.1	Rotavirus A WaxUK reassortant (UKg4Wa)
1537146	GCA_002634935.1	Rotavirus A/Rat-wt/GER/KS-11-573/2011/G3P[3]
28876	GCA_003159135.1	Rotavirus B
28876	GCA_003159195.1	Rotavirus B
28876	GCA_003159235.1	Rotavirus B
28876	GCA_003159255.1	Rotavirus B
28876	GCA_003159215.1	Rotavirus B
28876	GCA_003156315.1	Rotavirus B
36427	GCA_003086975.1	Rotavirus C
36427	GCA_003087055.1	Rotavirus C
36427	GCA_003087075.1	Rotavirus C
36427	GCA_003156195.1	Rotavirus C
335100	GCA_003156295.1	Rotavirus D
884200	GCA_000890155.1	Rotavirus D chicken/05V0049/DEU/2005
994994	GCA_000910335.1	Rotavirus F chicken/03V0568/DEU/2003
994995	GCA_001343825.1	Rotavirus G chicken/03V0567/DEU/2003
72132	GCA_002659255.1	Rotavirus G1
416557	GCA_002661095.1	Rotavirus G10P[8]
73034	GCA_002659435.1	Rotavirus G2
73034	GCA_002659235.1	Rotavirus G2
73034	GCA_003005675.1	Rotavirus G2
1842895	GCA_003005725.1	Rotavirus G28
1842895	GCA_003005735.1	Rotavirus G28
73036	GCA_002640095.1	Rotavirus G3
73036	GCA_002659175.1	Rotavirus G3
73036	GCA_002659355.1	Rotavirus G3
73036	GCA_002659395.1	Rotavirus G3

73036	GCA_002659335.1	Rotavirus G3
73036	GCA_002659195.1	Rotavirus G3
73036	GCA_002659275.1	Rotavirus G3
73036	GCA_002661815.1	Rotavirus G3
73036	GCA_002659295.1	Rotavirus G3
73036	GCA_003005685.1	Rotavirus G3
73036	GCA_003005695.1	Rotavirus G3
73036	GCA_003005705.1	Rotavirus G3
73036	GCA_003005715.1	Rotavirus G3
71031	GCA_002632475.1	Rotavirus G8
71031	GCA_002656835.1	Rotavirus G8
71031	GCA_002678375.1	Rotavirus G8
71031	GCA_002667335.1	Rotavirus G8
71031	GCA_002669555.1	Rotavirus G8
71031	GCA_002671775.1	Rotavirus G8
71031	GCA_002673995.1	Rotavirus G8
42567	GCA_002659375.1	Rotavirus G9
42567	GCA_002659415.1	Rotavirus G9
42567	GCA_002659215.1	Rotavirus G9
478084	GCA_002645955.1	Rotavirus G9P[6]
1637496	GCA_000973395.2	Rotavirus I
1460005	GCA_002676835.1	Rotavirus RVA/Human-wt/USA/12US1134/2012/G3P[9]
1432563	GCA_001021255.1	Rottboellia yellow mottle virus
1508710	GCA_000921235.1	Roundleaf bat hepatitis B virus
1508710	GCA_002826185.1	Roundleaf bat hepatitis B virus
11886	GCA_000855425.1	Rous sarcoma virus
369584	GCA_000870025.1	Rousettus aegyptiacus papillomavirus 1
1904411	GCA_002037715.1	Rousettus aegyptiacus polyomavirus 1
1892416	GCA_001725835.1	Rousettus bat coronavirus
1241933	GCA_000899495.1	Rousettus bat coronavirus HKU10
2053082	GCA_004131965.1	Rousettus leschenaultii bocaparvovirus 1
1303019	GCA_000918835.1	ROUT virus
64288	GCA_002820685.1	Royal Farm virus
11041	GCA_000863025.1	Rubella virus
1243178	GCA_000900395.1	Rubus canadensis virus 1
198310	GCA_000928335.1	Rubus yellow net virus
587370	GCA_000881055.1	Rudbeckia flower distortion virus
2480188	GCA_004132365.1	Rudphi virus 5
1851646	GCA_002610025.1	Ruegeria phage 45A6
1555208	GCA_000925815.1	Ruegeria phage DSS3-P1
2006924	GCA_003186085.1	Ruegeria phage DSS3-P22
2218610	GCA_003308635.1	Ruegeria phage vB_RpoMi-V15
2218611	GCA_003308615.1	Ruegeria phage vB_RpoP-V12
2218612	GCA_003308735.1	Ruegeria phage vB_RpoP-V13
2218613	GCA_003369325.1	Ruegeria phage vB_RpoP-V14
2218614	GCA_003308775.1	Ruegeria phage vB_RpoP-V17
2218615	GCA_003308675.1	Ruegeria phage vB_RpoP-V21
2218616	GCA_003308715.1	Ruegeria phage vB_RpoS-V10
2218617	GCA_003308695.1	Ruegeria phage vB_RpoS-V11
2218618	GCA_003308755.1	Ruegeria phage vB_RpoS-V16

2218619	GCA_003308655.1	Ruegeria phage vB_RpoS-V18
2218620	GCA_003308595.1	Ruegeria phage vB_RpoS-V7
1163708	GCA_000919755.1	Rupicapra rupicapra papillomavirus 1
1776868	GCA_003033175.1	Rusa timorensis papillomavirus type 1
1905556	GCA_004129495.1	Rusa timorensis papillomavirus type 2
40666	GCA_000860685.1	Ryegrass mosaic virus
119910	GCA_000860825.1	Ryegrass mottle virus
119910	GCA_002830885.1	Ryegrass mottle virus
2169995	GCA_003032625.1	Ryukyu mammarenavirus
64284	GCA_002004615.1	Saboya virus
89463	GCA_000847625.1	Sacbrood virus
182851	GCA_002609685.1	Saccharomonospora phage PIS 136
186772	GCA_000851605.1	Saccharomyces 20S RNA narnavirus
198599	GCA_000853185.1	Saccharomyces 23S RNA narnavirus
12450	GCA_000848385.1	Saccharomyces cerevisiae killer virus M1
11008	GCA_000852145.1	Saccharomyces cerevisiae virus L-A
42478	GCA_000847405.1	Saccharomyces cerevisiae virus L-BC (La)
1921825	GCA_001904905.1	Saccharomyces kudriavzevii virus L-A1
683179	GCA_000886895.1	Saccharum streak virus
2320522	GCA_004128115.1	Saesbyeol virus
434309	GCA_000871545.1	Saffold virus
2070152	GCA_002922455.1	Saffron latent virus
59303	GCA_002888735.1	Sagiyama virus
52274	GCA_000853965.1	Saguaro cactus virus
990304	GCA_000916955.1	Saimiri sciureus papillomavirus 1
990305	GCA_003179115.1	Saimiri sciureus papillomavirus 2
990306	GCA_003179135.1	Saimiri sciureus papillomavirus 3
1236410	GCA_002827925.1	Saimiri sciureus polyomavirus 1
10353	GCA_000890195.1	Saimiriine alphaherpesvirus 1
1535247	GCA_000894115.1	Saimiriine betaherpesvirus 4
10381	GCA_000845465.1	Saimiriine gammaherpesvirus 2 (Herpesvirus saimiri)
11080	GCA_000866785.1	Saint Louis encephalitis virus
120499	GCA_000927255.1	Salem virus
754067	GCA_000904435.1	Salicola phage CGphi29
2015814	GCA_002625545.1	Salicola phage SCTP-2
2041854	GCA_002990115.1	Salinibacter virus M8CC-19
2041855	GCA_002990125.1	Salinibacter virus M8CR30-2
1161935	GCA_000900975.1	Salinivibrio phage CW02
1897436	GCA_002612385.1	Salinivibrio phage SMHB1
1183239	GCA_000897135.1	Salisaeta icosahedral phage 1
1330524	GCA_000885035.1	Salivirus A
1547495	GCA_000926235.1	Salivirus FHB
651733	GCA_000886535.1	Salivirus NG-J1
381543	GCA_000926395.1	Salmon aquaparamyxovirus
1680908	GCA_001271235.1	Salmon gill poxvirus
55987	GCA_000854145.2	Salmon isavirus
84589	GCA_000857265.1	Salmon pancreas disease virus
1813783	GCA_001884575.1	Salmonella phage 100268_sal2
1813773	GCA_002757355.1	Salmonella phage 101962B_sal5
1813780	GCA_002757435.1	Salmonella phage 103203_sal4

1813779	GCA_001881795.1	Salmonella phage 103203_sal5
1813781	GCA_001881935.1	Salmonella phage 118970_sal1
1813782	GCA_001882075.1	Salmonella phage 118970_sal2
1813771	GCA_001882095.1	Salmonella phage 118970_sal3
1810705	GCA_001736255.1	Salmonella phage 118970_sal4
2508061	GCA_004146685.1	Salmonella phage 1-23
1813777	GCA_002757395.1	Salmonella phage 146851_sal4
1813774	GCA_002757375.1	Salmonella phage 146851_sal5
1647402	GCA_002605845.1	Salmonella phage 18-India
1654883	GCA_001550665.1	Salmonella phage 19
1654884	GCA_001547705.1	Salmonella phage 21
1654885	GCA_002599425.1	Salmonella phage 22
1654886	GCA_002599405.1	Salmonella phage 25
1970747	GCA_002621145.1	Salmonella phage 29485
2508069	GCA_004138835.1	Salmonella phage 3-29
1654887	GCA_002599385.1	Salmonella phage 34
1654888	GCA_002599365.1	Salmonella phage 35
1654890	GCA_001517035.1	Salmonella phage 37
1654891	GCA_001516975.1	Salmonella phage 38
1654892	GCA_002606625.1	Salmonella phage 39
2268591	GCA_003341475.1	Salmonella phage 3A_8767
1654894	GCA_002606665.1	Salmonella phage 41
1813769	GCA_001881875.1	Salmonella phage 64795_sal3
1813778	GCA_002757415.1	Salmonella phage 64795_sal4
1054968	GCA_000892955.1	Salmonella phage 7-11
1113547	GCA_000927575.1	Salmonella phage 9NA
1965386	GCA_002620085.1	Salmonella phage alphaalpha
2483851	GCA_003865595.1	Salmonella phage Astrid
1543199	GCA_001755025.1	Salmonella phage BP12A
1543201	GCA_001754725.1	Salmonella phage BP12B
1543203	GCA_001754285.1	Salmonella phage BP12C
1543205	GCA_001755145.1	Salmonella phage BP63
1857099	GCA_001881975.1	Salmonella phage BPS11Q3
2060120	GCA_002957685.1	Salmonella phage BPS11T2
1857100	GCA_001881995.1	Salmonella phage BPS15Q2
2060121	GCA_002957695.1	Salmonella phage BPS15S6
2060122	GCA_002957705.1	Salmonella phage BPS17L1
2060123	GCA_002957715.1	Salmonella phage BPS17S6
2060124	GCA_002957725.1	Salmonella phage BPS17W1
1958914	GCA_002990035.1	Salmonella phage BSP101
2053015	GCA_003861675.1	Salmonella phage BSP161
1960312	GCA_002990025.1	Salmonella phage BSP22A
1958913	GCA_002989985.1	Salmonella phage BSPM4
454798	GCA_001015285.1	Salmonella phage Det7
1163482	GCA_000902635.1	Salmonella phage Ent1
215158	GCA_000840625.2	Salmonella phage epsilon15
215158	GCA_000840625.1	Salmonella phage epsilon15
348058	GCA_000882655.1	Salmonella phage epsilon34
1611545	GCA_001470275.1	Salmonella phage f18SE
1788345	GCA_002757295.1	Salmonella phage f2SE

1788346	GCA_002757315.1	Salmonella phage f3SE
77775	GCA_000841605.1	Salmonella phage FelixO1
1087480	GCA_002630765.1	Salmonella phage FO1a
1747352	GCA_002757095.1	Salmonella phage fSE1C
1695221	GCA_002756775.1	Salmonella phage fSE4S
1173769	GCA_000907915.1	Salmonella phage FSL SP-004
1173770	GCA_003441755.1	Salmonella phage FSL SP-010
1173771	GCA_003441775.1	Salmonella phage FSL SP-012
1173753	GCA_003441695.1	Salmonella phage FSL SP-019
1173767	GCA_003441715.1	Salmonella phage FSL SP-029
1173749	GCA_000910415.1	Salmonella phage FSL SP-031
1173750	GCA_003441815.1	Salmonella phage FSL SP-038
1173755	GCA_002602265.1	Salmonella phage FSL SP-039
1173751	GCA_003441835.1	Salmonella phage FSL SP-049
1173761	GCA_000908895.1	Salmonella phage FSL SP-058
1173759	GCA_003441655.1	Salmonella phage FSL SP-062
1173768	GCA_003441735.1	Salmonella phage FSL SP-063
1173762	GCA_000909555.1	Salmonella phage FSL SP-076
1173756	GCA_003441675.1	Salmonella phage FSL SP-099
1173772	GCA_003441795.1	Salmonella phage FSL SP-107
1173758	GCA_002602285.1	Salmonella phage FSL SP-124
590739	GCA_000884195.1	Salmonella phage g341c
2108163	GCA_003014045.1	Salmonella phage GE_vB_7A
2108164	GCA_003014055.1	Salmonella phage GE_vB_B1
2108165	GCA_003014065.1	Salmonella phage GE_vB_B3
2108166	GCA_003014075.1	Salmonella phage GE_vB_BS
2108167	GCA_003014085.1	Salmonella phage GE_vB_HIL
2108168	GCA_003014095.1	Salmonella phage GE_vB_M4
2108169	GCA_003014105.1	Salmonella phage GE_vB_M5
2108170	GCA_003014115.1	Salmonella phage GE_vB_MG
2108171	GCA_003014125.1	Salmonella phage GE_vB_N5
2108173	GCA_003014145.1	Salmonella phage GE_vB_NS7
2108174	GCA_003014155.1	Salmonella phage GE_vB_TR
1868169	GCA_001885505.1	Salmonella phage GG32
1965464	GCA_002620325.1	Salmonella phage GJL01
1873985	GCA_001882315.1	Salmonella phage IME207
1340534	GCA_000910395.1	Salmonella phage Jersey
2048975	GCA_003850205.1	Salmonella phage KFS-SE1
1434751	GCA_003330385.1	Salmonella phage KK-2013
1871711	GCA_002991175.1	Salmonella phage KNP6
1929963	GCA_002617745.1	Salmonella phage LPSE1
1973454	GCA_002622285.1	Salmonella phage LPST10
2528247	GCA_004340225.1	Salmonella phage LSE7621
1540823	GCA_000929095.1	Salmonella phage LSPA1
2488859	GCA_003865695.1	Salmonella phage Lumpael
1868170	GCA_001885525.1	Salmonella phage MA12
1406794	GCA_000912955.1	Salmonella phage Marshall
1406795	GCA_000911335.1	Salmonella phage Maynard
2283282	GCA_003575885.1	Salmonella phage Meda
2041413	GCA_002744075.1	Salmonella phage Melville

2301531	GCA_003575685.1	Salmonella phage Mooltan
2500169	GCA_004015485.1	Salmonella phage Munch
1572716	GCA_002620065.1	Salmonella phage Mushroom
2054274	GCA_002957495.1	Salmonella phage Mutine
1647411	GCA_001745155.1	Salmonella phage NR01
200913	GCA_002599345.1	Salmonella phage P22-pbi
529929	GCA_000875285.1	Salmonella phage phiSG-JL2
1837218	GCA_001744735.1	Salmonella phage phSE-2
1837219	GCA_002609705.1	Salmonella phage phSE-5
2081904	GCA_002959195.1	Salmonella phage PMBT28
2420341	GCA_003668335.1	Salmonella phage PS5
889338	GCA_000893935.1	Salmonella phage PVP-SE1
929814	GCA_000903195.1	Salmonella phage RE-2010
2231334	GCA_003341835.1	Salmonella phage S100
2231335	GCA_003342515.1	Salmonella phage S101
2231336	GCA_003341855.1	Salmonella phage S102
2231337	GCA_003341875.1	Salmonella phage S103
2231338	GCA_003341895.1	Salmonella phage S104
2231339	GCA_003341915.1	Salmonella phage S106
2231340	GCA_003341935.1	Salmonella phage S107
2231341	GCA_003341955.1	Salmonella phage S111
2231342	GCA_003341975.1	Salmonella phage S113
2231343	GCA_003341995.1	Salmonella phage S114
2231344	GCA_003342015.1	Salmonella phage S115
2231345	GCA_003342035.1	Salmonella phage S116
2231346	GCA_003342055.1	Salmonella phage S117
2231347	GCA_003342535.1	Salmonella phage S118
2231348	GCA_003342075.1	Salmonella phage S119
2231349	GCA_003342095.1	Salmonella phage S120
2231350	GCA_003342115.1	Salmonella phage S123
2231351	GCA_003342415.1	Salmonella phage S124
2231352	GCA_003342135.1	Salmonella phage S126
2231354	GCA_003342175.1	Salmonella phage S131
2231355	GCA_003342195.1	Salmonella phage S132
2231356	GCA_003342215.1	Salmonella phage S133
2231357	GCA_003342555.1	Salmonella phage S134
2231358	GCA_003342235.1	Salmonella phage S135
2231359	GCA_003342255.1	Salmonella phage S137
2231360	GCA_003342275.1	Salmonella phage S138
2231361	GCA_003342295.1	Salmonella phage S142
2231362	GCA_003342315.1	Salmonella phage S147
2267250	GCA_003342335.1	Salmonella phage S149
1913039	GCA_002614785.1	Salmonella phage S8
2115848	GCA_002629925.1	Salmonella phage SE1 (in:Nonagvirus)
329864	GCA_000881955.1	Salmonella phage SE1 (in:P22virus)
2081631	GCA_002997395.1	Salmonella phage SE131
1115478	GCA_000894315.1	Salmonella phage SE2
1965375	GCA_002620045.1	Salmonella phage SE40
2483612	GCA_003865515.1	Salmonella phage Seafire
2500556	GCA_004016005.1	Salmonella phage Season12

2530161	GCA_004340445.1	Salmonella phage SEE-1
2419756	GCA_004146745.1	Salmonella phage Segz_1
1647455	GCA_001502555.1	Salmonella phage SEN1
1647458	GCA_001470915.1	Salmonella phage SEN22
1647463	GCA_001470135.1	Salmonella phage SEN34
1647465	GCA_001501935.1	Salmonella phage SEN4
1647466	GCA_001470675.1	Salmonella phage SEN5
1647468	GCA_002605925.1	Salmonella phage SEN8
2301647	GCA_003575925.1	Salmonella phage SenALZ1
2301648	GCA_003575905.1	Salmonella phage SenASZ3
2419752	GCA_004146765.1	Salmonella phage SeSz-1
2419753	GCA_004146805.1	Salmonella phage SeSz-2
2479482	GCA_004146785.1	Salmonella phage Seszw_1
424949	GCA_000913715.1	Salmonella phage SETP13
424944	GCA_000870645.1	Salmonella phage SETP3
424947	GCA_000912875.1	Salmonella phage SETP7
2419754	GCA_004146705.1	Salmonella phage SeWh-1
2419755	GCA_004146725.1	Salmonella phage SeZq-1
1080800	GCA_000893035.1	Salmonella phage SFP10
1108865	GCA_000900855.1	Salmonella phage Sh19
2025819	GCA_003328965.1	Salmonella phage SH9
1572715	GCA_001500355.1	Salmonella phage Shivani
1965374	GCA_002620025.1	Salmonella phage Si3
2316013	GCA_003575645.1	Salmonella phage Siskin
1815968	GCA_001744215.1	Salmonella phage SJ46
2234035	GCA_003342395.1	Salmonella phage Skate
1204528	GCA_000904875.1	Salmonella phage SKML-39
2283281	GCA_003575865.1	Salmonella phage Solent
1920294	GCA_002743495.1	Salmonella phage SP01
1173760	GCA_002831045.2	Salmonella phage SP069
2109652	GCA_002924565.1	Salmonella phage SP1a
2136797	GCA_003183805.1	Salmonella phage SPAsTU
1327941	GCA_002755655.1	Salmonella phage SPC32H
1327942	GCA_002755675.1	Salmonella phage SPC32N
1125653	GCA_000895275.1	Salmonella phage SPN1S
1147140	GCA_000900935.1	Salmonella phage SPN3UB
1090134	GCA_001042275.1	Salmonella phage SPN3US
1127357	GCA_000896335.1	Salmonella phage SPN9CC
1162289	GCA_002602165.1	Salmonella phage SPN9TCW
1198117	GCA_003329225.1	Salmonella phage SPT-1
293644	GCA_000857125.1	Salmonella phage SS3e
1204529	GCA_001041715.1	Salmonella phage SSE121
1177632	GCA_000899335.1	Salmonella phage SSU5
2023990	GCA_002627425.1	Salmonella phage ST11
714583	GCA_000891435.1	Salmonella phage ST160
2024311	GCA_002628825.1	Salmonella phage St161
2024312	GCA_002628845.1	Salmonella phage St162
1204544	GCA_003330505.1	Salmonella phage ST4
175950	GCA_000841985.1	Salmonella phage ST64B
2480623	GCA_003691795.1	Salmonella phage STG2

1204531	GCA_001041775.1	Salmonella phage STML-198
1914788	GCA_002615545.1	Salmonella phage STP03
1908549	GCA_002614305.1	Salmonella phage STP07
1971233	GCA_002924585.1	Salmonella phage Stp1
1445860	GCA_000954975.2	Salmonella phage STP4-a
2136699	GCA_003183785.1	Salmonella phage STsAS
2316014	GCA_003575665.1	Salmonella phage Sw2
1912492	GCA_002954915.1	Salmonella phage UPF_BP1
1913047	GCA_002614905.1	Salmonella phage UPF_BP2
1868654	GCA_001744855.1	Salmonella phage vB_SalM_PM10
1458849	GCA_000917915.1	Salmonella phage vB_SalM_SJ2
1446492	GCA_000922695.1	Salmonella phage vB_SalM_SJ3
2018029	GCA_002627185.1	Salmonella phage vB_SalP_PM43
1168548	GCA_000899835.1	Salmonella phage vB_SemP_Emek
2029636	GCA_003260735.1	Salmonella phage vB_SenM_PA13076
1868843	GCA_002611185.1	Salmonella phage vB_SenM-2
1087482	GCA_000905195.1	Salmonella phage vB_SenMS16
1211279	GCA_002624865.1	Salmonella phage vB_SenS_AG11
2136179	GCA_003031035.1	Salmonella phage vB_SenS_PHB07
2420239	GCA_003719075.1	Salmonella phage vB_SenS_phi135
2024214	GCA_002627765.1	Salmonella phage vB_SenS_PVP-SE2
1913114	GCA_002615185.1	Salmonella phage vB_SenS_Sasha
1913117	GCA_002615205.1	Salmonella phage vB_SenS_Sergei
1465618	GCA_000917095.1	Salmonella phage vB_SenS-Ent2
1465613	GCA_000920035.1	Salmonella phage vB_SenS-Ent3
1815631	GCA_001745955.1	Salmonella phage vB_SnwM_CGG4-1
1168549	GCA_000899875.1	Salmonella phage vB_SosS_Oslo
1567025	GCA_001041335.1	Salmonella phage vB_SPuM_SP116
2081603	GCA_002997375.1	Salmonella phage vB_SpuP_Spp16
2081603	GCA_002997375.2	Salmonella phage vB_SpuP_Spp16
2234083	GCA_003307455.1	Salmonella phage vB_SpuS_Sp4
424716	GCA_000875025.1	Salmonella phage Vi II-E1
866889	GCA_000890795.1	Salmonella phage Vi06
1204541	GCA_002624885.1	Salmonella phage wksl3
2053686	GCA_002957345.1	Salmonella phage YSP2
1934252	GCA_900156925.2	Salmonella virus BTP1
1541887	GCA_000924975.1	Salmonella virus Chi
1541887	GCA_002604965.1	Salmonella virus Chi
1173754	GCA_000907935.1	Salmonella virus FSLSP030
1173757	GCA_000909515.1	Salmonella virus FSLSP088
155148	GCA_000838905.1	Salmonella virus HK620
1005056	GCA_000907975.1	Salmonella virus iEPS5
10754	GCA_000845765.1	Salmonella virus P22
10754	GCA_002599325.1	Salmonella virus P22
10754	GCA_002599445.1	Salmonella virus P22
10754	GCA_002599305.1	Salmonella virus P22
12407	GCA_000843405.1	Salmonella virus PsP3
194966	GCA_000843145.1	Salmonella virus SP6
194966	GCA_002618985.1	Salmonella virus SP6
977927	GCA_000891755.1	Salmonella virus SPC35

1141134	GCA_000901515.1	Salmonella virus SPN19
173443	GCA_000841165.1	Salmonella virus ST64T
1540099	GCA_002149405.1	Salmonella virus Stitch
1987993	GCA_000890895.1	Salmonella virus ViI
2053700	GCA_002957405.1	Salmonella virus VSe102
2301720	GCA_003443415.1	Salmonella virus VSe103
2053699	GCA_002957395.1	Salmonella virus VSe11
2301721	GCA_003443435.1	Salmonella virus VSiP
2301722	GCA_003443455.1	Salmonella virus VSt10
2301723	GCA_003443475.1	Salmonella virus VSt472
508442	GCA_003181315.1	Salmonid herpesvirus 3
1981916	GCA_002118465.1	Salmovirus WFRC1
2419804	GCA_004134665.1	Salvia divinorum RNA virus 1
2419805	GCA_004134685.1	Salvia hispanica RNA virus 1
1461100	GCA_002966455.1	Samba virus
2052851	GCA_004117315.1	Sambucus virus S
1955199	GCA_002024715.1	San Bernardo virus
1564182	GCA_000925155.1	San Miguel sea lion virus 8
688699	GCA_000891955.1	Sandfly fever Turkey virus
1980489	GCA_002145925.1	Sangassou orthohantavirus
1552661	GCA_001432055.1	Santa barbara virus
977913	GCA_000890595.1	Santeuil nodavirus
1923355	GCA_001962915.1	Sanxia atyid shrimp virus 1
1923356	GCA_001961235.1	Sanxia atyid shrimp virus 2
1923357	GCA_001961975.1	Sanxia atyid shrimp virus 3
1923358	GCA_001961095.1	Sanxia atyid shrimp virus 4
1923360	GCA_001962895.1	Sanxia narna-like virus 1
1923365	GCA_001961215.1	Sanxia permutotetra-like virus 1
1923366	GCA_001961955.1	Sanxia picorna-like virus 1
1923367	GCA_001961075.1	Sanxia picorna-like virus 10
1923368	GCA_001962875.1	Sanxia picorna-like virus 11
1923369	GCA_001925255.1	Sanxia picorna-like virus 12
1923370	GCA_001961195.1	Sanxia picorna-like virus 13
1923371	GCA_001959555.1	Sanxia picorna-like virus 2
1923372	GCA_001961055.1	Sanxia picorna-like virus 3
1923373	GCA_001962855.1	Sanxia picorna-like virus 4
1923374	GCA_001961175.1	Sanxia picorna-like virus 5
1923377	GCA_001959535.1	Sanxia picorna-like virus 8
1923378	GCA_001961035.1	Sanxia picorna-like virus 9
1923354	GCA_001961115.1	Sanxia Qinvirus-like virus 1
1923380	GCA_001962835.1	Sanxia sobemo-like virus 1
1923381	GCA_001961155.1	Sanxia sobemo-like virus 2
1923382	GCA_001959515.1	Sanxia sobemo-like virus 3
1923383	GCA_001961015.1	Sanxia sobemo-like virus 4
1923384	GCA_001962815.1	Sanxia sobemo-like virus 5
1923385	GCA_001958755.1	Sanxia tombus-like virus 1
1923386	GCA_001959495.1	Sanxia tombus-like virus 2
1923387	GCA_001960995.1	Sanxia tombus-like virus 3
1923388	GCA_001962795.1	Sanxia tombus-like virus 4
1923389	GCA_001958735.1	Sanxia tombus-like virus 5

1923390	GCA_001959475.1	Sanxia tombus-like virus 6
1923391	GCA_001927215.1	Sanxia tombus-like virus 7
1923392	GCA_001960975.1	Sanxia tombus-like virus 8
1923393	GCA_001962775.1	Sanxia tombus-like virus 9
1608060	GCA_001746075.1	Sanxia Water Strider Virus 1
1923394	GCA_001958715.1	Sanxia water strider virus 10
1923397	GCA_001959455.1	Sanxia water strider virus 13
1923398	GCA_001960955.1	Sanxia water strider virus 14
1923399	GCA_001926375.1	Sanxia water strider virus 15
1923400	GCA_001962755.1	Sanxia water strider virus 16
1923401	GCA_001958695.1	Sanxia water strider virus 17
1923403	GCA_001959435.1	Sanxia water strider virus 19
1923404	GCA_001960935.1	Sanxia water strider virus 20
1923405	GCA_001960335.1	Sanxia water strider virus 21
1608063	GCA_001744755.1	Sanxia water strider virus 4
1608064	GCA_001744075.1	Sanxia Water Strider Virus 5
1746060	GCA_001443965.1	Sanxia water strider virus 6
1923406	GCA_001958675.1	Sanxia water strider virus 7
1923407	GCA_001959415.1	Sanxia water strider virus 8
1923408	GCA_001960915.1	Sanxia water strider virus 9
686984	GCA_900197465.1	Sapelovirus A
1815512	GCA_004114835.1	Saphire II virus
290314	GCA_000855765.1	Sapovirus C12
291175	GCA_000854265.1	Sapovirus Hu/Dresden/pJG-Sap01/DE
1280920	GCA_001008475.1	Sapovirus Hu/Nagoya/NGY-1/2012/JPN
234601	GCA_000853825.1	Sapovirus Mc10
2109380	GCA_004130835.1	Sarawak virus
31503	GCA_000851265.1	Satellite maize white line mosaic virus
12881	GCA_000849185.1	Satellite tobacco mosaic virus
2025096	GCA_002988085.1	Satellite tobacco necrosis virus C
198608	GCA_000841025.1	Satellites of Trichomonas vaginalis T1 virus
159141	GCA_000899155.1	Sathuperi orthobunyavirus
47416	GCA_000860985.1	Satsuma dwarf virus
40012	GCA_002004315.1	Saumarez Reef virus
1234171	GCA_000899295.1	Sauropus leaf curl disease associated DNA beta
1130981	GCA_002823285.1	Sauropus leaf curl virus
1577997	GCA_000929975.1	Sauropus yellowing virus
1697349	GCA_001274405.1	Scale drop disease virus
157018	GCA_000858245.1	Scallion mosaic virus
1300323	GCA_002830705.1	Scheffersomyces segobiensis virus L
253700	GCA_000881915.1	Schlumbergera virus X
191289	GCA_000820355.1	Sclerophthora macrospora virus A
75914	GCA_000853645.1	Sclerophthora macrospora virus B
2316144	GCA_004132445.1	Sclerotinia minor endornavirus 1
1859161	GCA_001678415.1	Sclerotinia nivalis victorivirus 1
2003398	GCA_000920615.1	Sclerotinia sclerotiorum betaendornavirus 1
1654339	GCA_001019975.1	Sclerotinia sclerotiorum botybirnavirus 1
213034	GCA_000865105.1	Sclerotinia sclerotiorum debilitation-associated RNA virus
1502101	GCA_000920995.1	Sclerotinia sclerotiorum debilitation-associated RNA virus 2
1788309	GCA_003029215.1	Sclerotinia sclerotiorum deltaflexivirus 1

2219092	GCA_004133965.1	Sclerotinia sclerotiorum deltaflexivirus 2
1181310	GCA_000897075.1	Sclerotinia sclerotiorum dsRNA mycovirus-L
1353016	GCA_000907855.1	Sclerotinia sclerotiorum endornavirus 1
1661062	GCA_001027375.1	Sclerotinia sclerotiorum fusarivirus 1
664785	GCA_000886735.1	Sclerotinia sclerotiorum hypovirulence associated DNA viru
664785	GCA_003849565.1	Sclerotinia sclerotiorum hypovirulence associated DNA viru
664785	GCA_003849585.1	Sclerotinia sclerotiorum hypovirulence associated DNA viru
664785	GCA_003850305.1	Sclerotinia sclerotiorum hypovirulence associated DNA viru
664785	GCA_003850325.1	Sclerotinia sclerotiorum hypovirulence associated DNA viru
664785	GCA_003849625.1	Sclerotinia sclerotiorum hypovirulence associated DNA viru
664785	GCA_003849605.1	Sclerotinia sclerotiorum hypovirulence associated DNA viru
664785	GCA_003850285.1	Sclerotinia sclerotiorum hypovirulence associated DNA viru
2231776	GCA_003848365.1	Sclerotinia sclerotiorum hypovirulence associated DNA viru
1074325	GCA_000894815.1	Sclerotinia sclerotiorum hypovirus 1
1423347	GCA_000911435.1	Sclerotinia sclerotiorum hypovirus 2
1661257	GCA_001030085.1	Sclerotinia sclerotiorum megabirnavirus 1
1494074	GCA_000943685.1	Sclerotinia sclerotiorum mitovirus 1 HC025
1133728	GCA_004128795.1	Sclerotinia sclerotiorum mitovirus 2
1279099	GCA_001461325.1	Sclerotinia sclerotiorum mitovirus 3
1435446	GCA_000915595.1	Sclerotinia sclerotiorum mitovirus 6
1840528	GCA_001646235.1	Sclerotinia sclerotiorum mycoreovirus 4
1483724	GCA_000925535.1	Sclerotinia sclerotiorum negative-stranded RNA virus 1
1435457	GCA_000960965.1	Sclerotinia sclerotiorum negative-stranded RNA virus 3
1708391	GCA_003673785.1	Sclerotinia sclerotiorum negative-stranded RNA virus 4
659497	GCA_000884095.1	Sclerotinia sclerotiorum partitivirus S
1435450	GCA_001651105.1	Sclerotinia sclerotiorum umbra-like virus 1
1895000	GCA_001725935.1	Sclerotium hydrophilum virus 1
994485	GCA_002829525.1	Scophthalmus maximus reovirus
936149	GCA_000925595.1	Scophthalmus maximus rhabdovirus
693999	GCA_000870985.1	Scotophilus bat coronavirus 512
312273	GCA_000882375.1	Scrophularia mottle virus
1882382	GCA_001717395.1	Sea otter parvovirus 1
1552409	GCA_000926975.1	Sea otter polyomavirus 1
1416741	GCA_003260795.1	Sea otter poxvirus
1979755	GCA_002827165.1	Sea star-associated densovirus
575913	GCA_000883595.1	Sea turtle tornovirus 1
1427157	GCA_000924275.1	Seal anellovirus 2
1427156	GCA_000921755.1	Seal anellovirus 3
1566011	GCA_000930795.1	Seal anellovirus 4
991022	GCA_000891655.1	Seal anellovirus TFFN/USA/2006
187984	GCA_002219465.1	Seal parapoxvirus
471728	GCA_000874345.1	Seal picornavirus type 1
1294177	GCA_000909315.1	Sebokele virus 1
11033	GCA_000860285.1	Semliki Forest virus
1272957	GCA_002145605.1	Sena Madureira virus
390157	GCA_000881615.1	Senecavirus A
310103	GCA_000860065.1	Senecio yellow mosaic virus
1884991	GCA_001706945.1	Senna leaf curl virus
1881013	GCA_001707025.1	Senna mosaic virus
12557	GCA_000855645.1	Seoul virus 80-39

44026	GCA_000868725.1	Sepik virus
2094713	GCA_004131485.1	Serinus canaria papillomavirus 1
2169996	GCA_000879015.1	Serra do Navio mammarenavirus
2024250	GCA_002628085.1	Serratia phage 2050H1
2024251	GCA_002628105.1	Serratia phage 2050H2
2024252	GCA_002628125.1	Serratia phage 2050HW
1962671	GCA_002619745.1	Serratia phage BF
2006940	GCA_002625145.1	Serratia phage CBH8
2006941	GCA_002625165.1	Serratia phage CHI14
1282995	GCA_000909435.1	Serratia phage Eta
1871709	GCA_002709865.1	Serratia phage KNP4
1262513	GCA_000905035.1	Serratia phage phiMAM1
1481112	GCA_000920775.1	Serratia phage PS2
2282409	GCA_003365975.1	Serratia phage Scapp
1897434	GCA_002612345.2	Serratia phage SM9-3Y
1897434	GCA_002612345.1	Serratia phage SM9-3Y
2419930	GCA_003718775.1	Serratia phage vB_SmaA_3M
1852640	GCA_002745835.1	Serratia phage vB_Sru_IME250
1852640	GCA_002745855.1	Serratia phage vB_Sru_IME250
2006942	GCA_002625185.1	Serratia phage X20
1519097	GCA_000928375.1	Sesavirus CSL10538
12558	GCA_000862445.1	Sesbania mosaic virus
247507	GCA_000869085.1	Sevenband grouper nervous necrosis virus
694009	GCA_000864885.1	Severe acute respiratory syndrome-related coronavirus
1003835	GCA_003088895.1	Severe fever with thrombocytopenia virus
1003835	GCA_003088915.1	Severe fever with thrombocytopenia virus
1003835	GCA_003088955.1	Severe fever with thrombocytopenia virus
1003835	GCA_003088975.1	Severe fever with thrombocytopenia virus
1003835	GCA_003091035.1	Severe fever with thrombocytopenia virus
1003835	GCA_003091055.1	Severe fever with thrombocytopenia virus
1003835	GCA_003089015.1	Severe fever with thrombocytopenia virus
1003835	GCA_003091075.1	Severe fever with thrombocytopenia virus
1003835	GCA_003089035.1	Severe fever with thrombocytopenia virus
1003835	GCA_003091095.1	Severe fever with thrombocytopenia virus
1003835	GCA_003089055.1	Severe fever with thrombocytopenia virus
1003835	GCA_003091115.1	Severe fever with thrombocytopenia virus
1003835	GCA_003089075.1	Severe fever with thrombocytopenia virus
1003835	GCA_003091135.1	Severe fever with thrombocytopenia virus
1003835	GCA_003089095.1	Severe fever with thrombocytopenia virus
1003835	GCA_003091155.1	Severe fever with thrombocytopenia virus
1003835	GCA_003089115.1	Severe fever with thrombocytopenia virus
1003835	GCA_003091175.1	Severe fever with thrombocytopenia virus
1003835	GCA_003089135.1	Severe fever with thrombocytopenia virus
1003835	GCA_003089155.1	Severe fever with thrombocytopenia virus
1003835	GCA_003091215.1	Severe fever with thrombocytopenia virus
1003835	GCA_003091235.1	Severe fever with thrombocytopenia virus
1003835	GCA_003091255.1	Severe fever with thrombocytopenia virus
1003835	GCA_003091275.1	Severe fever with thrombocytopenia virus
1003835	GCA_003089235.1	Severe fever with thrombocytopenia virus
1003835	GCA_003091295.1	Severe fever with thrombocytopenia virus

1003835	GCA_003089255.1	Severe fever with thrombocytopenia virus
1003835	GCA_003091315.1	Severe fever with thrombocytopenia virus
1003835	GCA_003089275.1	Severe fever with thrombocytopenia virus
1003835	GCA_003091335.1	Severe fever with thrombocytopenia virus
1003835	GCA_003089295.1	Severe fever with thrombocytopenia virus
1003835	GCA_003091355.1	Severe fever with thrombocytopenia virus
1003835	GCA_003089315.1	Severe fever with thrombocytopenia virus
1003835	GCA_003091375.1	Severe fever with thrombocytopenia virus
1003835	GCA_003089335.1	Severe fever with thrombocytopenia virus
1003835	GCA_003091395.1	Severe fever with thrombocytopenia virus
1003835	GCA_003089355.1	Severe fever with thrombocytopenia virus
1003835	GCA_003089375.1	Severe fever with thrombocytopenia virus
1003835	GCA_003091435.1	Severe fever with thrombocytopenia virus
1003835	GCA_003089395.1	Severe fever with thrombocytopenia virus
1003835	GCA_003091455.1	Severe fever with thrombocytopenia virus
1003835	GCA_003089415.1	Severe fever with thrombocytopenia virus
1003835	GCA_003091475.1	Severe fever with thrombocytopenia virus
1003835	GCA_003091495.1	Severe fever with thrombocytopenia virus
1003835	GCA_003089455.1	Severe fever with thrombocytopenia virus
1003835	GCA_003091515.1	Severe fever with thrombocytopenia virus
1003835	GCA_003089475.1	Severe fever with thrombocytopenia virus
1003835	GCA_003091535.1	Severe fever with thrombocytopenia virus
1003835	GCA_003089495.1	Severe fever with thrombocytopenia virus
1003835	GCA_003091555.1	Severe fever with thrombocytopenia virus
1003835	GCA_003089515.1	Severe fever with thrombocytopenia virus
1003835	GCA_003091575.1	Severe fever with thrombocytopenia virus
1003835	GCA_003089535.1	Severe fever with thrombocytopenia virus
1003835	GCA_003091595.1	Severe fever with thrombocytopenia virus
1003835	GCA_003089555.1	Severe fever with thrombocytopenia virus
1003835	GCA_003091615.1	Severe fever with thrombocytopenia virus
1003835	GCA_003089575.1	Severe fever with thrombocytopenia virus
1003835	GCA_003089595.1	Severe fever with thrombocytopenia virus
1003835	GCA_003091655.1	Severe fever with thrombocytopenia virus
1003835	GCA_003089615.1	Severe fever with thrombocytopenia virus
1003835	GCA_003091675.1	Severe fever with thrombocytopenia virus
1003835	GCA_003089635.1	Severe fever with thrombocytopenia virus
1003835	GCA_003091695.1	Severe fever with thrombocytopenia virus
1003835	GCA_003091715.1	Severe fever with thrombocytopenia virus
1003835	GCA_003089675.1	Severe fever with thrombocytopenia virus
1003835	GCA_003091735.1	Severe fever with thrombocytopenia virus
1003835	GCA_003089695.1	Severe fever with thrombocytopenia virus
1003835	GCA_003091755.1	Severe fever with thrombocytopenia virus
1003835	GCA_003089715.1	Severe fever with thrombocytopenia virus
1003835	GCA_003091775.1	Severe fever with thrombocytopenia virus
1003835	GCA_003089735.1	Severe fever with thrombocytopenia virus
1003835	GCA_003087695.1	Severe fever with thrombocytopenia virus
1003835	GCA_003091795.1	Severe fever with thrombocytopenia virus
1003835	GCA_003089755.1	Severe fever with thrombocytopenia virus
1003835	GCA_003091815.1	Severe fever with thrombocytopenia virus
1003835	GCA_003089775.1	Severe fever with thrombocytopenia virus

1003835	GCA_003091835.1	Severe fever with thrombocytopenia virus
1003835	GCA_003089795.1	Severe fever with thrombocytopenia virus
1003835	GCA_003089815.1	Severe fever with thrombocytopenia virus
1003835	GCA_003087775.1	Severe fever with thrombocytopenia virus
1003835	GCA_003091875.1	Severe fever with thrombocytopenia virus
1003835	GCA_003089835.1	Severe fever with thrombocytopenia virus
1003835	GCA_003091895.1	Severe fever with thrombocytopenia virus
1003835	GCA_003089855.1	Severe fever with thrombocytopenia virus
1003835	GCA_003091915.1	Severe fever with thrombocytopenia virus
1003835	GCA_003091935.1	Severe fever with thrombocytopenia virus
1003835	GCA_003091955.1	Severe fever with thrombocytopenia virus
1003835	GCA_003089915.1	Severe fever with thrombocytopenia virus
1003835	GCA_003091975.1	Severe fever with thrombocytopenia virus
1003835	GCA_003089935.1	Severe fever with thrombocytopenia virus
1003835	GCA_003089955.1	Severe fever with thrombocytopenia virus
1003835	GCA_003089975.1	Severe fever with thrombocytopenia virus
1003835	GCA_003089995.1	Severe fever with thrombocytopenia virus
1003835	GCA_003090015.1	Severe fever with thrombocytopenia virus
1003835	GCA_003090035.1	Severe fever with thrombocytopenia virus
1003835	GCA_003090055.1	Severe fever with thrombocytopenia virus
1003835	GCA_003087715.1	Severe fever with thrombocytopenia virus
1003835	GCA_003090075.1	Severe fever with thrombocytopenia virus
1003835	GCA_003090095.1	Severe fever with thrombocytopenia virus
1003835	GCA_003090135.1	Severe fever with thrombocytopenia virus
1003835	GCA_003090155.1	Severe fever with thrombocytopenia virus
1003835	GCA_003090175.1	Severe fever with thrombocytopenia virus
1003835	GCA_003087735.1	Severe fever with thrombocytopenia virus
1003835	GCA_003090195.1	Severe fever with thrombocytopenia virus
1003835	GCA_003092255.1	Severe fever with thrombocytopenia virus
1003835	GCA_003090215.1	Severe fever with thrombocytopenia virus
1003835	GCA_003092275.1	Severe fever with thrombocytopenia virus
1003835	GCA_003090235.1	Severe fever with thrombocytopenia virus
1003835	GCA_003090255.1	Severe fever with thrombocytopenia virus
1003835	GCA_003090275.1	Severe fever with thrombocytopenia virus
1003835	GCA_003092335.1	Severe fever with thrombocytopenia virus
1003835	GCA_003090295.1	Severe fever with thrombocytopenia virus
1003835	GCA_003092355.1	Severe fever with thrombocytopenia virus
1003835	GCA_003087755.1	Severe fever with thrombocytopenia virus
1003835	GCA_003090315.1	Severe fever with thrombocytopenia virus
1003835	GCA_003092375.1	Severe fever with thrombocytopenia virus
1003835	GCA_003092395.1	Severe fever with thrombocytopenia virus
1003835	GCA_003090355.1	Severe fever with thrombocytopenia virus
1003835	GCA_003092415.1	Severe fever with thrombocytopenia virus
1003835	GCA_003090375.1	Severe fever with thrombocytopenia virus
1003835	GCA_003092435.1	Severe fever with thrombocytopenia virus
1003835	GCA_003090395.1	Severe fever with thrombocytopenia virus
1003835	GCA_003092455.1	Severe fever with thrombocytopenia virus
1003835	GCA_003090415.1	Severe fever with thrombocytopenia virus
1003835	GCA_003092475.1	Severe fever with thrombocytopenia virus
1003835	GCA_003090435.1	Severe fever with thrombocytopenia virus

1003835	GCA_003092495.1	Severe fever with thrombocytopenia virus
1003835	GCA_003090455.1	Severe fever with thrombocytopenia virus
1003835	GCA_003090475.1	Severe fever with thrombocytopenia virus
1003835	GCA_003090495.1	Severe fever with thrombocytopenia virus
1003835	GCA_003090515.1	Severe fever with thrombocytopenia virus
1003835	GCA_003088475.1	Severe fever with thrombocytopenia virus
1003835	GCA_003090535.1	Severe fever with thrombocytopenia virus
1003835	GCA_003087795.1	Severe fever with thrombocytopenia virus
1003835	GCA_003090575.1	Severe fever with thrombocytopenia virus
1003835	GCA_003090595.1	Severe fever with thrombocytopenia virus
1003835	GCA_003090615.1	Severe fever with thrombocytopenia virus
1003835	GCA_003090635.1	Severe fever with thrombocytopenia virus
1003835	GCA_003090655.1	Severe fever with thrombocytopenia virus
1003835	GCA_003090675.1	Severe fever with thrombocytopenia virus
1003835	GCA_003088635.1	Severe fever with thrombocytopenia virus
1003835	GCA_003090695.1	Severe fever with thrombocytopenia virus
1003835	GCA_003088655.1	Severe fever with thrombocytopenia virus
1003835	GCA_003090715.1	Severe fever with thrombocytopenia virus
1003835	GCA_003090735.1	Severe fever with thrombocytopenia virus
1003835	GCA_003090775.1	Severe fever with thrombocytopenia virus
1003835	GCA_003090795.1	Severe fever with thrombocytopenia virus
1003835	GCA_003090815.1	Severe fever with thrombocytopenia virus
1003835	GCA_003088795.1	Severe fever with thrombocytopenia virus
1003835	GCA_003090855.1	Severe fever with thrombocytopenia virus
1003835	GCA_003088835.1	Severe fever with thrombocytopenia virus
1003835	GCA_003088855.1	Severe fever with thrombocytopenia virus
1003835	GCA_003087855.1	Severe fever with thrombocytopenia virus
1003835	GCA_003088875.1	Severe fever with thrombocytopenia virus
1843761	GCA_000928295.1	Sewage associated gemycircularvirus 3
1843761	GCA_001646055.1	Sewage associated gemycircularvirus 3
1843761	GCA_003849105.1	Sewage associated gemycircularvirus 3
1843761	GCA_003849125.1	Sewage associated gemycircularvirus 3
1985407	GCA_000928275.1	Sewage derived gemycircularvirus 1
1985408	GCA_000930215.1	Sewage derived gemycircularvirus 2
1592207	GCA_000930235.1	Sewage-associated circular DNA molecule
1592211	GCA_002149465.1	Sewage-associated circular DNA molecule-1
1595961	GCA_002149805.1	Sewage-associated circular DNA molecule-2
1592212	GCA_000930175.1	Sewage-associated circular DNA molecule-3
1519385	GCA_003726635.1	Sewage-associated circular DNA virus-1
1519386	GCA_003726655.1	Sewage-associated circular DNA virus-10
1519387	GCA_003726675.1	Sewage-associated circular DNA virus-11
1519388	GCA_003726695.1	Sewage-associated circular DNA virus-12
1519389	GCA_003726715.1	Sewage-associated circular DNA virus-13
1519390	GCA_003726735.1	Sewage-associated circular DNA virus-14
1592082	GCA_000931055.1	Sewage-associated circular DNA virus-15
1592083	GCA_000931255.1	Sewage-associated circular DNA virus-16
1592084	GCA_000929355.1	Sewage-associated circular DNA virus-17
1592085	GCA_000928475.1	Sewage-associated circular DNA virus-18
1592086	GCA_000931035.1	Sewage-associated circular DNA virus-19
1519391	GCA_001441035.1	Sewage-associated circular DNA virus-2

1592087	GCA_000931235.1	Sewage-associated circular DNA virus-20
1592088	GCA_000929335.1	Sewage-associated circular DNA virus-21
1592089	GCA_000928455.1	Sewage-associated circular DNA virus-22
1592090	GCA_000931015.1	Sewage-associated circular DNA virus-23
1592091	GCA_000931215.1	Sewage-associated circular DNA virus-24
1592092	GCA_000929315.1	Sewage-associated circular DNA virus-25
1592093	GCA_000928435.1	Sewage-associated circular DNA virus-26
1592094	GCA_000930995.1	Sewage-associated circular DNA virus-27
1592095	GCA_000931195.1	Sewage-associated circular DNA virus-28
1592096	GCA_000929295.1	Sewage-associated circular DNA virus-29
1519392	GCA_003726755.1	Sewage-associated circular DNA virus-3
1592097	GCA_000928415.1	Sewage-associated circular DNA virus-30
1592098	GCA_000930975.1	Sewage-associated circular DNA virus-31
1592099	GCA_000931175.1	Sewage-associated circular DNA virus-32
1592100	GCA_000929275.1	Sewage-associated circular DNA virus-33
1592101	GCA_000928395.1	Sewage-associated circular DNA virus-34
1592102	GCA_000930955.1	Sewage-associated circular DNA virus-35
1592103	GCA_000930315.1	Sewage-associated circular DNA virus-36
1592104	GCA_000929255.1	Sewage-associated circular DNA virus-37
1519393	GCA_003726775.1	Sewage-associated circular DNA virus-4
1519394	GCA_003726795.1	Sewage-associated circular DNA virus-5
1519395	GCA_003726815.1	Sewage-associated circular DNA virus-6
1519396	GCA_003726835.1	Sewage-associated circular DNA virus-7
1519397	GCA_003726855.1	Sewage-associated circular DNA virus-8
1519398	GCA_003726875.1	Sewage-associated circular DNA virus-9
1592081	GCA_000928495.1	Sewage-associated gemycircularvirus 1
1519407	GCA_000929175.1	Sewage-associated gemycircularvirus 2
1519399	GCA_000928235.1	Sewage-associated gemycircularvirus 4
1519400	GCA_000928215.1	Sewage-associated gemycircularvirus 5
1519401	GCA_000930835.1	Sewage-associated gemycircularvirus 6
1519404	GCA_000929155.1	Sewage-associated gemycircularvirus 9
1519409	GCA_000930855.1	Sewage-associated gemycircularvirus-10a
1519410	GCA_003849505.1	Sewage-associated gemycircularvirus-10b
1519402	GCA_000930195.1	Sewage-associated gemycircularvirus-7a
1519405	GCA_002825965.1	Sewage-associated gemycircularvirus-7b
992211	GCA_003092315.1	SFTS virus AH15
992212	GCA_000897355.1	SFTS virus HB29
992213	GCA_003087815.1	SFTS virus HN13
992214	GCA_003087835.1	SFTS virus HN6
1316165	GCA_003087875.1	SFTS virus HNXY_115
1316166	GCA_003087915.1	SFTS virus HNXY_130
1316167	GCA_003087935.1	SFTS virus HNXY_144
1316169	GCA_003087955.1	SFTS virus HNXY_164
1316170	GCA_003087975.1	SFTS virus HNXY_170
1316171	GCA_003087995.1	SFTS virus HNXY_174
1316172	GCA_003088015.1	SFTS virus HNXY_182
1316173	GCA_003088035.1	SFTS virus HNXY_186
1316174	GCA_003088055.1	SFTS virus HNXY_188
1316175	GCA_003088075.1	SFTS virus HNXY_191
1316176	GCA_003088095.1	SFTS virus HNXY_195

1316177	GCA_003088135.1	SFTS virus HNXY_2
1316178	GCA_003088155.1	SFTS virus HNXY_202
1316179	GCA_003088175.1	SFTS virus HNXY_206
1316180	GCA_003088195.1	SFTS virus HNXY_207
1316181	GCA_003088215.1	SFTS virus HNXY_212
1316182	GCA_003088235.1	SFTS virus HNXY_224
1316183	GCA_003088255.1	SFTS virus HNXY_231
1316184	GCA_003088275.1	SFTS virus HNXY_245
1316185	GCA_003088295.1	SFTS virus HNXY_262
1316186	GCA_003088315.1	SFTS virus HNXY_278
1316187	GCA_003088355.1	SFTS virus HNXY_293
1316188	GCA_003088375.1	SFTS virus HNXY_31
1316189	GCA_003088395.1	SFTS virus HNXY_319
1316190	GCA_003088415.1	SFTS virus HNXY_327
1316191	GCA_003088435.1	SFTS virus HNXY_93
992215	GCA_003088715.1	SFTS virus JS3
992216	GCA_003088735.1	SFTS virus JS4
992217	GCA_003088815.1	SFTS virus LN2
992218	GCA_003088935.1	SFTS virus LN3
1115693	GCA_003090835.1	SFTS virus Mao
992219	GCA_003089175.1	SFTS virus SD24
992220	GCA_003089195.1	SFTS virus SD4
1923409	GCA_001925935.1	Shahe arthropod virus 1
1923414	GCA_001960315.1	Shahe endorna-like virus 1
1923415	GCA_001921575.1	Shahe hepe-like virus 1
1923416	GCA_001921335.1	Shahe hepe-like virus 2
1923417	GCA_001958655.1	Shahe heteroptera virus 1
1923418	GCA_001926695.1	Shahe heteroptera virus 2
1923419	GCA_001921415.1	Shahe heteroptera virus 3
1923420	GCA_001959395.1	Shahe heteroptera virus 4
1923421	GCA_001960895.1	Shahe isopoda virus 1
1923422	GCA_001960295.1	Shahe isopoda virus 2
1923423	GCA_001958635.1	Shahe isopoda virus 3
1923425	GCA_001927195.1	Shahe isopoda virus 5
1923429	GCA_001959375.1	Shahe narna-like virus 1
1923430	GCA_001960875.1	Shahe narna-like virus 2
1923431	GCA_001960275.1	Shahe narna-like virus 3
1923433	GCA_001958615.1	Shahe narna-like virus 5
1923435	GCA_001959355.1	Shahe narna-like virus 7
1923439	GCA_001960855.1	Shahe picorna-like virus 1
1923440	GCA_001960255.1	Shahe picorna-like virus 10
1923441	GCA_001958595.1	Shahe picorna-like virus 11
1923442	GCA_001959335.1	Shahe picorna-like virus 12
1923443	GCA_001960835.1	Shahe picorna-like virus 13
1923444	GCA_001960235.1	Shahe picorna-like virus 14
1923445	GCA_001958575.1	Shahe picorna-like virus 2
1923446	GCA_001959315.1	Shahe picorna-like virus 3
1923447	GCA_001960815.1	Shahe picorna-like virus 4
1923448	GCA_001960215.1	Shahe picorna-like virus 5
1923449	GCA_001958555.1	Shahe picorna-like virus 6

1923450	GCA_001959295.1	Shahe picorna-like virus 7
1923451	GCA_001926355.1	Shahe picorna-like virus 8
1923452	GCA_001960795.1	Shahe picorna-like virus 9
1923453	GCA_001960195.1	Shahe qinvirus-like virus 1
1923454	GCA_001958535.1	Shahe sobemo-like virus 1
1923455	GCA_001959275.1	Shahe tombus-like virus 1
1923456	GCA_001960775.1	Shahe tombus-like virus 2
1923457	GCA_001960175.1	Shahe yuevirus-like virus 1
31770	GCA_000850765.1	Shallot virus X
62059	GCA_000864145.1	Shallot yellow stripe virus
159150	GCA_000900015.1	Shamonda orthobunyavirus
2170191	GCA_003029045.1	Shanbavirus A
1923460	GCA_001958515.1	Shayang ascaridia galli virus 2
1608065	GCA_001755425.1	Shayang Fly Virus 1
1608066	GCA_001754565.1	Shayang Fly Virus 2
1608067	GCA_001754125.1	Shayang Fly Virus 3
1746061	GCA_001444145.1	Shayang fly virus 4
1608068	GCA_001754985.1	Shayang Spider Virus 1
1746062	GCA_001443825.1	Shayang spider virus 4
1923463	GCA_001925915.1	Shayang virga-like virus 1
1843756	GCA_001646275.1	Sheep faeces associated smacovirus 1
1843757	GCA_001646455.1	Sheep faeces associated smacovirus 2
1843758	GCA_001645875.1	Sheep faeces associated smacovirus 3
1634381	GCA_000989015.1	Sheep polyomavirus 1
376848	GCA_000840205.1	Sheeppox virus 17077-99
259898	GCA_000848945.1	Sheldgoose hepatitis B virus
1852599	GCA_002610125.1	Shewanella phage SFCi1
1445859	GCA_000917215.2	Shewanella phage Spp001
1965372	GCA_002620005.1	Shewanella phage SppYZU01
1970795	GCA_002621245.1	Shewanella phage SppYZU05
1458859	GCA_000925035.1	Shewanella sp. phage 1/4
1458860	GCA_000927595.1	Shewanella sp. phage 1/40
1458861	GCA_000927535.1	Shewanella sp. phage 1/41
1458862	GCA_000929495.1	Shewanella sp. phage 1/44
1458863	GCA_000925875.1	Shewanella sp. phage 3/49
1416032	GCA_001551625.1	Shigella phage 75/02 Stx
637730	GCA_000888035.1	Shigella phage Ag3
1109721	GCA_000895135.1	Shigella phage EP23
1871710	GCA_002709885.1	Shigella phage KNP5
2419683	GCA_003719035.1	Shigella phage phi2457T
2340715	GCA_003613115.1	Shigella phage phi25-307
1498227	GCA_000925775.1	Shigella phage POCJ13
1414738	GCA_000915775.1	Shigella phage pSb-1
2496551	GCA_000908595.1	Shigella phage pSf-1
1572702	GCA_000929055.1	Shigella phage pSf-2
1551641	GCA_000930595.1	Shigella phage pSs-1
2024313	GCA_002628865.1	Shigella phage Sd1
2282196	GCA_002628885.1	Shigella phage Sf11 SMD-2017
2024315	GCA_002628905.1	Shigella phage Sf12
2024316	GCA_002628925.1	Shigella phage Sf13

2024304	GCA_002955345.1	Shigella phage Sf14
2024317	GCA_002628945.1	Shigella phage Sf15
2024318	GCA_002628965.1	Shigella phage Sf16
2024305	GCA_002955355.1	Shigella phage Sf17
2024319	GCA_002628985.1	Shigella phage Sf18
2024306	GCA_002955365.1	Shigella phage Sf19
2024307	GCA_002955375.1	Shigella phage Sf20
2024308	GCA_002955385.1	Shigella phage Sf21
2024320	GCA_002743575.1	Shigella phage Sf22
2024321	GCA_002629005.1	Shigella phage Sf23
2024309	GCA_002955395.1	Shigella phage Sf24
2024310	GCA_002955405.1	Shigella phage Sf25
66284	GCA_000909715.1	Shigella phage SfII
2024314	GCA_003323775.1	Shigella phage Sfin-1
1407493	GCA_000913735.1	Shigella phage SfIV
1567022	GCA_001041695.1	Shigella phage SfMu
1785176	GCA_002989995.1	Shigella phage SFN6B
2269380	GCA_003369165.1	Shigella phage SFPH2
2518961	GCA_004521055.1	Shigella phage SfPhi01
1913048	GCA_002614925.1	Shigella phage SH6
1913049	GCA_002614945.1	Shigella phage SH7
1863007	GCA_001745335.1	Shigella phage SHBML-50-1
1541825	GCA_000925755.1	Shigella phage Shf125875
1002725	GCA_000892655.1	Shigella phage Shfl2
1863008	GCA_001743555.1	Shigella phage SHFML-11
1863009	GCA_001745995.1	Shigella phage SHFML-26
1863010	GCA_001744675.1	Shigella phage SHSML-45
1863011	GCA_001746195.1	Shigella phage SHSML-52-1
645664	GCA_000888655.1	Shigella phage SP18
1983588	GCA_002955055.1	Shigella phage SSP1
1675531	GCA_001470215.1	Shigella phage Ss-VASD
2500762	GCA_004015885.1	Shigella phage vB_SdyM_006
2500763	GCA_004015905.1	Shigella phage vB_SflM_004
2048005	GCA_002745295.1	Shigella phage vB_SflS-ISF001
2507846	GCA_004146825.1	Shigella phage vB_SsoS_008
2006922	GCA_002625025.1	Shigella phage vB_SsoS-ISF002
10761	GCA_000845865.1	Shigella virus Sf6
1002724	GCA_000891015.1	Shigella virus Shfl1
746543	GCA_000927155.1	Shimoni bat lyssavirus
2006394	GCA_004130715.1	Shinobi tetravirus
638660	GCA_001678255.1	Short-finned eel ranavirus
1987145	GCA_001443885.1	Shrew hepatovirus KS121232Sorara2012
92652	GCA_003024735.1	Shrimp white spot syndrome virus
92652	GCA_003972705.1	Shrimp white spot syndrome virus
1923464	GCA_001959255.1	Shuangao alphatetra-like virus 1
1608072	GCA_001755405.1	Shuangao Bedbug Virus 2
1608074	GCA_003673765.1	Shuangao Fly Virus 2
1608075	GCA_001754545.1	Shuangao Insect Virus 1
1923466	GCA_001960755.1	Shuangao insect virus 10
1923467	GCA_001960155.1	Shuangao insect virus 11

1923468	GCA_001958495.1	Shuangao insect virus 12
1608076	GCA_002814495.1	Shuangao Insect Virus 2
1746063	GCA_001446205.1	Shuangao insect virus 7
1923469	GCA_001959235.1	Shuangao insect virus 8
1923470	GCA_001960735.1	Shuangao insect virus 9
1608081	GCA_003673585.1	Shuangao lacewing virus
1746064	GCA_001444045.1	Shuangao lacewing virus 2
1923474	GCA_001960135.1	Shuangao sobemo-like virus 1
1923475	GCA_001958475.1	Shuangao sobemo-like virus 2
1923476	GCA_001959215.1	Shuangao sobemo-like virus 3
1923477	GCA_001960715.1	Shuangao toti-like virus
1217821	GCA_000899935.1	Sibine fusca densovirus
2220003	GCA_003260775.1	Sichuan takin astrovirus
2220002	GCA_003260755.1	Sichuan takin enterovirus
1755588	GCA_000919575.1	Sicinivirus A
1692263	GCA_001274465.1	Sicyonia brevirostris associated circular virus
1904882	GCA_001777325.1	Sida angular mosaic virus
1949196	GCA_003029405.1	Sida bright yellow mosaic virus
1949196	GCA_003029405.2	Sida bright yellow mosaic virus
1949197	GCA_003029395.1	Sida chlorotic mottle virus
1904883	GCA_001777265.1	Sida chlorotic vein virus
1264880	GCA_002823305.1	Sida ciliaris golden mosaic virus
536082	GCA_002823325.1	Sida common mosaic virus
929767	GCA_002823345.1	Sida golden mosaic Braco virus-[Jamaica:Liguanea:2008]
2080282	GCA_002986845.1	Sida golden mosaic Brazil virus
929769	GCA_000889555.1	Sida golden mosaic Buckup virus-[Jamaica:St. Elizabeth:20[illegible]
223313	GCA_000840525.1	Sida golden mosaic Costa Rica virus
871521	GCA_000888515.1	Sida golden mosaic Florida virus-Malvastrum
223315	GCA_002823365.1	Sida golden mosaic Florida virus-USA
223316	GCA_000841325.1	Sida golden mosaic Honduras virus
1002681	GCA_002823385.1	Sida golden mosaic Lara virus
51034	GCA_000837905.1	Sida golden mosaic virus
759390	GCA_000888355.1	Sida golden mottle virus
1949198	GCA_003029415.1	Sida golden yellow spot virus
223318	GCA_002823405.1	Sida golden yellow vein virus-[A11]
929766	GCA_000840485.1	Sida golden yellow vein virus-[Jamaica:Liguanea2:2008]
2169843	GCA_000864325.1	Sida leaf curl alphasatellite
2169843	GCA_003034085.1	Sida leaf curl alphasatellite
337823	GCA_000866845.1	Sida leaf curl virus
337824	GCA_000865285.1	Sida leaf curl virus-associated DNA beta
228897	GCA_000840905.1	Sida micrantha mosaic virus
228897	GCA_002986855.1	Sida micrantha mosaic virus
1046571	GCA_000895795.1	Sida mosaic Alagoas virus
932069	GCA_000893095.1	Sida mosaic Bolivia virus 1
932070	GCA_000891535.1	Sida mosaic Bolivia virus 2
367850	GCA_000867305.1	Sida mosaic Sinaloa virus
1266995	GCA_000904175.1	Sida mottle Alagoas virus
223319	GCA_000841285.1	Sida mottle virus-[Brazil]
1266993	GCA_000905175.1	Sida yellow blotch virus
536083	GCA_002823465.1	Sida yellow leaf curl virus

1266994	GCA_000906655.1	Sida yellow mosaic Alagoas virus
291270	GCA_000865705.1	Sida yellow mosaic China satellite DNA beta
290090	GCA_000897235.1	Sida yellow mosaic China virus - [Hainan 8]
223320	GCA_000864865.1	Sida yellow mosaic virus-[Brazil]
408719	GCA_000867925.1	Sida yellow mosaic Yucatan virus
1093958	GCA_000896395.1	Sida yellow mottle virus
1266977	GCA_000905775.1	Sida yellow net virus
1506184	GCA_000923555.1	Sida yellow vein alphasatellite
1293961	GCA_000914395.1	Sida yellow vein China alphasatellite
420255	GCA_000871525.1	Sida yellow vein Madurai virus
1535266	GCA_001430315.1	Sida yellow vein mosaic alphasatellite
447597	GCA_000870865.1	Sida yellow vein Vietnam alphasatellite
390441	GCA_000871685.1	Sida yellow vein Vietnam virus
447602	GCA_000871745.1	Sida yellow vein Vietnam virus satellite DNA beta
223321	GCA_000843025.1	Sida yellow vein virus
326489	GCA_000864065.1	Sida yellow vein virus satellite DNA beta
858513	GCA_002823485.1	Sidastrum golden leaf spot virus
1418029	GCA_002830225.1	Siegesbeckia yellow vein betasatellite
379728	GCA_000867585.1	Siegesbeckia yellow vein Guangxi virus
371463	GCA_000867465.1	Siegesbeckia yellow vein virus-[GD13]
371411	GCA_000868325.1	Siegesbeckia yellow vein virus-[GD13]-associated DNA beta
2293299	GCA_003847245.1	Sierra dome spider associated circular virus 1
2293300	GCA_003846745.1	Sierra dome spider associated circular virus 2
1424280	GCA_000921215.1	Sierra Nevada virus
490912	GCA_000882935.1	Silicibacter phage DSS3phi2
1126411	GCA_000926295.1	Silurus glanis circovirus
1126411	GCA_004080955.1	Silurus glanis circovirus
35306	GCA_000897575.1	Simbu orthobunyavirus
310540	GCA_000847325.1	Simian adenovirus 1
38432	GCA_001430155.1	Simian adenovirus 13
1715778	GCA_001430595.1	Simian adenovirus 16
909210	GCA_000913475.1	Simian adenovirus 18
38416	GCA_001430375.1	Simian adenovirus 19
585059	GCA_000905275.1	Simian adenovirus 20
198503	GCA_000847245.1	Simian adenovirus 21
175567	GCA_000848905.1	Simian adenovirus 25
38420	GCA_000846705.1	Simian adenovirus 3
995022	GCA_000890695.1	Simian adenovirus 49
413258	GCA_001430555.1	Simian adenovirus 8
1560346	GCA_000928615.1	Simian adenovirus DM-2014
929831	GCA_002815255.1	Simian Agent 10
442851	GCA_002816765.1	Simian enterovirus SV4
221703	GCA_003047575.1	Simian foamy virus Pongo pygmaeus pygmaeus
1965068	GCA_002816155.1	Simian hemorrhagic encephalitis virus
38143	GCA_000848625.1	Simian hemorrhagic fever virus
11723	GCA_000863925.1	Simian immunodeficiency virus
11723	GCA_003108785.1	Simian immunodeficiency virus
11723	GCA_003099055.1	Simian immunodeficiency virus
11723	GCA_003109325.1	Simian immunodeficiency virus
11723	GCA_003101955.1	Simian immunodeficiency virus

11723	GCA_003100935.1	Simian immunodeficiency virus
11723	GCA_003101975.1	Simian immunodeficiency virus
11723	GCA_003104695.1	Simian immunodeficiency virus
11723	GCA_003098275.1	Simian immunodeficiency virus
11723	GCA_003101935.1	Simian immunodeficiency virus
11723	GCA_003101995.1	Simian immunodeficiency virus
11723	GCA_003098215.1	Simian immunodeficiency virus
11723	GCA_003192405.1	Simian immunodeficiency virus
11723	GCA_003191705.1	Simian immunodeficiency virus
11723	GCA_003191755.1	Simian immunodeficiency virus
11723	GCA_003191795.1	Simian immunodeficiency virus
11723	GCA_003191815.1	Simian immunodeficiency virus
11723	GCA_003191835.1	Simian immunodeficiency virus
11723	GCA_003191855.1	Simian immunodeficiency virus
11723	GCA_003191875.1	Simian immunodeficiency virus
11723	GCA_003191895.1	Simian immunodeficiency virus
11723	GCA_003191915.1	Simian immunodeficiency virus
11723	GCA_003191935.1	Simian immunodeficiency virus
11723	GCA_003191955.1	Simian immunodeficiency virus
11723	GCA_003191975.1	Simian immunodeficiency virus
11723	GCA_003192005.1	Simian immunodeficiency virus
11723	GCA_003192025.1	Simian immunodeficiency virus
11723	GCA_003192045.1	Simian immunodeficiency virus
11723	GCA_003192065.1	Simian immunodeficiency virus
11723	GCA_003192085.1	Simian immunodeficiency virus
11723	GCA_003192105.1	Simian immunodeficiency virus
11723	GCA_003192125.1	Simian immunodeficiency virus
11723	GCA_003192145.1	Simian immunodeficiency virus
11723	GCA_003192165.1	Simian immunodeficiency virus
11723	GCA_003192185.1	Simian immunodeficiency virus
11723	GCA_003192205.1	Simian immunodeficiency virus
11723	GCA_003192345.1	Simian immunodeficiency virus
11723	GCA_003192365.1	Simian immunodeficiency virus
11723	GCA_003192385.1	Simian immunodeficiency virus
11723	GCA_003192425.1	Simian immunodeficiency virus
11723	GCA_003192445.1	Simian immunodeficiency virus
11723	GCA_003192465.1	Simian immunodeficiency virus
11723	GCA_003192485.1	Simian immunodeficiency virus
11723	GCA_003190905.1	Simian immunodeficiency virus
11723	GCA_003192505.1	Simian immunodeficiency virus
11723	GCA_003192525.1	Simian immunodeficiency virus
11723	GCA_003192545.1	Simian immunodeficiency virus
11723	GCA_003193445.1	Simian immunodeficiency virus
11723	GCA_003192565.1	Simian immunodeficiency virus
11723	GCA_003192585.1	Simian immunodeficiency virus
11723	GCA_003192605.1	Simian immunodeficiency virus
11723	GCA_003191605.1	Simian immunodeficiency virus
11723	GCA_003192685.1	Simian immunodeficiency virus
11723	GCA_003196755.1	Simian immunodeficiency virus
11723	GCA_003192705.1	Simian immunodeficiency virus

11723	GCA_003192725.1	Simian immunodeficiency virus
11723	GCA_003192745.1	Simian immunodeficiency virus
11723	GCA_003192765.1	Simian immunodeficiency virus
11723	GCA_003192785.1	Simian immunodeficiency virus
11723	GCA_003192805.1	Simian immunodeficiency virus
11723	GCA_003192825.1	Simian immunodeficiency virus
11723	GCA_003192845.1	Simian immunodeficiency virus
11723	GCA_003191775.1	Simian immunodeficiency virus
11723	GCA_003192885.1	Simian immunodeficiency virus
11723	GCA_003192905.1	Simian immunodeficiency virus
11723	GCA_003192925.1	Simian immunodeficiency virus
11723	GCA_003192945.1	Simian immunodeficiency virus
11723	GCA_003192985.1	Simian immunodeficiency virus
11723	GCA_003193005.1	Simian immunodeficiency virus
11723	GCA_003193025.1	Simian immunodeficiency virus
11723	GCA_003193045.1	Simian immunodeficiency virus
11723	GCA_003193065.1	Simian immunodeficiency virus
11723	GCA_003193085.1	Simian immunodeficiency virus
11723	GCA_003202515.1	Simian immunodeficiency virus
11723	GCA_003193105.1	Simian immunodeficiency virus
11723	GCA_003193125.1	Simian immunodeficiency virus
11723	GCA_003192865.1	Simian immunodeficiency virus
11723	GCA_003193165.1	Simian immunodeficiency virus
11723	GCA_003193185.1	Simian immunodeficiency virus
11723	GCA_003193205.1	Simian immunodeficiency virus
11723	GCA_003193245.1	Simian immunodeficiency virus
11723	GCA_003193265.1	Simian immunodeficiency virus
11723	GCA_003193285.1	Simian immunodeficiency virus
11723	GCA_003193305.1	Simian immunodeficiency virus
11723	GCA_003193325.1	Simian immunodeficiency virus
11723	GCA_003193345.1	Simian immunodeficiency virus
11723	GCA_003191665.1	Simian immunodeficiency virus
11723	GCA_003193365.1	Simian immunodeficiency virus
11723	GCA_003191725.1	Simian immunodeficiency virus
11723	GCA_003193385.1	Simian immunodeficiency virus
11723	GCA_003193405.1	Simian immunodeficiency virus
11723	GCA_003193425.1	Simian immunodeficiency virus
11723	GCA_003193465.1	Simian immunodeficiency virus
11723	GCA_003192625.1	Simian immunodeficiency virus
11723	GCA_003193505.1	Simian immunodeficiency virus
11723	GCA_003193525.1	Simian immunodeficiency virus
11723	GCA_003193545.1	Simian immunodeficiency virus
11723	GCA_003191585.1	Simian immunodeficiency virus
11723	GCA_003193565.1	Simian immunodeficiency virus
11723	GCA_003191645.1	Simian immunodeficiency virus
11726	GCA_003192965.1	Simian immunodeficiency virus - agm
349974	GCA_003193225.1	Simian immunodeficiency virus - agm.sab-1
349975	GCA_003193485.1	Simian immunodeficiency virus - agm.tan-1
538563	GCA_003193145.1	Simian immunodeficiency virus - olc
160754	GCA_003191565.1	Simian immunodeficiency virus 17E-Cl

159122	GCA_000851745.1	Simian immunodeficiency virus SIV-mnd 2
1962300	GCA_000906395.1	Simian mastadenovirus C
1923976	GCA_001904845.1	Simian mastadenovirus WIV19
31598	GCA_002827365.1	Simian parvovirus
1454041	GCA_000921975.1	Simian pegivirus
723802	GCA_000888575.1	Simian retrovirus 4
1904439	GCA_001754525.1	Simian retrovirus 8
1979166	GCA_000859165.1	Simian rubulavirus
1002918	GCA_000856165.1	Simian sapelovirus 1
481147	GCA_000881775.1	Simian T-cell lymphotropic virus 6
33747	GCA_000861125.1	Simian T-lymphotropic virus 1
33748	GCA_000860005.1	Simian T-lymphotropic virus 2
39101	GCA_000847845.1	Simian T-lymphotropic virus 3
1619218	GCA_000959655.1	Simian torque teno virus 30
1619219	GCA_000954935.1	Simian torque teno virus 31
1619220	GCA_000959695.1	Simian torque teno virus 32
1629656	GCA_000969135.1	Simian torque teno virus 33
1629657	GCA_000969075.1	Simian torque teno virus 34
46771	GCA_000866005.1	Simian virus 12
1980491	GCA_000854765.1	Sin Nombre orthohantavirus
1980491	GCA_002830985.1	Sin Nombre orthohantavirus
1768874	GCA_001654145.1	Sinapis alba cryptic virus 1
11034	GCA_000860545.1	Sindbis virus
11034	GCA_002888835.1	Sindbis virus
11034	GCA_002888895.1	Sindbis virus
262968	GCA_000846905.1	Singapore grouper iridovirus
373862	GCA_000870185.1	Siniperca chuatsi rhabdovirus
2049306	GCA_002957205.2	Sinorhizobium phage HMSP1-Susan
2049306	GCA_002957205.1	Sinorhizobium phage HMSP1-Susan
179237	GCA_000837425.1	Sinorhizobium phage PBC5
2029632	GCA_002629645.1	Sinorhizobium phage phi2LM21
2029634	GCA_002629665.1	Sinorhizobium phage phi3LM21
1524882	GCA_001534635.1	Sinorhizobium phage phiLM21
1357423	GCA_001023565.1	Sinorhizobium phage phiM12
1647404	GCA_002605865.1	Sinorhizobium phage phiM19
2005789	GCA_002624905.1	Sinorhizobium phage phiM5
2301527	GCA_003429165.1	Sinorhizobium phage phiM6
1647403	GCA_002622405.1	Sinorhizobium phage phiM7
1636182	GCA_001470155.1	Sinorhizobium phage phiM9
1647405	GCA_001501175.1	Sinorhizobium phage phiN3
2170413	GCA_003654285.1	Siphoviridae sp.
2170413	GCA_003657685.1	Siphoviridae sp.
2170413	GCA_003659865.1	Siphoviridae sp.
2170413	GCA_003653225.1	Siphoviridae sp.
2170413	GCA_003657505.1	Siphoviridae sp.
2170413	GCA_003651665.1	Siphoviridae sp.
2170413	GCA_003651685.1	Siphoviridae sp.
2170413	GCA_003651845.1	Siphoviridae sp.
2170413	GCA_003651885.1	Siphoviridae sp.
2170413	GCA_003651905.1	Siphoviridae sp.

2170413	GCA_003651965.1	Siphoviridae sp.
2170413	GCA_003656085.1	Siphoviridae sp.
2170413	GCA_003658225.1	Siphoviridae sp.
2170413	GCA_003652085.1	Siphoviridae sp.
2170413	GCA_003652105.1	Siphoviridae sp.
498715	GCA_003193585.1	SIV-wrc Pbt-05GM-X02
1636544	GCA_002605685.1	Skermania phage SPI1
1520004	GCA_000895055.1	Skua adenovirus 1
1641642	GCA_001271155.1	Skunk adenovirus PB1
1961072	GCA_002118725.1	Skunk amdoparvovirus
160796	GCA_001745695.1	Skunkpox virus
78540	GCA_000862545.1	Sleeping disease virus
458132	GCA_000887395.1	Slow bee paralysis virus
1581151	GCA_000930035.1	Slow loris parvovirus 1
289366	GCA_000859305.1	Small anellovirus 1
289367	GCA_000860145.1	Small anellovirus 2
1538101	GCA_000922435.1	Small begomovirus-associated satellite
252602	GCA_000844085.1	Snake adeno-associated virus
189830	GCA_000872165.1	Snake adenovirus 1
2419666	GCA_004134705.1	Snake deltavirus F18-5
40270	GCA_000849325.1	Snakehead retrovirus
103603	GCA_000849265.1	Snakehead virus
89623	GCA_000844545.1	Snow goose hepatitis B virus
89623	GCA_003033025.1	Snow goose hepatitis B virus
373126	GCA_000867065.1	Sodalis phage phiSG1
373126	GCA_002630045.1	Sodalis phage phiSG1
673375	GCA_000886975.1	Sodalis phage SO1
1917080	GCA_004129515.1	Sodiomyces alkalinus fusarivirus 1
2293301	GCA_003847125.1	Soft spider associated circular virus 1
2152923	GCA_004133265.1	Sogatella furcifera hepe-like virus
2082585	GCA_004132645.1	Sogatella furcifera totivirus 1
2082586	GCA_004132665.1	Sogatella furcifera totivirus 2
100887	GCA_000849285.1	Soil-borne cereal mosaic virus
28375	GCA_000849985.1	Soil-borne wheat mosaic virus
64317	GCA_000955255.1	Sokoluk virus
932073	GCA_000921815.1	Solanum mosaic Bolivia virus
12471	GCA_002004135.1	Solanum nodiflorum mottle virus
195061	GCA_000837585.1	Solanum nodiflorum mottle virus satellite RNA
1414671	GCA_000912895.1	Solenopsis invicta densovirus
294369	GCA_000854925.1	Solenopsis invicta virus 1
439491	GCA_000870805.1	Solenopsis invicta virus 2
439491	GCA_003029605.1	Solenopsis invicta virus 2
631345	GCA_000881215.1	Solenopsis invicta virus 3
2018500	GCA_002271145.1	Solenopsis invicta virus 4
11307	GCA_000848205.1	Sonchus yellow net nucleorhabdovirus
1980161	GCA_003029465.1	Sophora alopecuroides yellow stunt alphasatellite 1a
1980165	GCA_003029485.1	Sophora alopecuroides yellow stunt alphasatellite 4
1980168	GCA_003029475.1	Sophora alopecuroides yellow stunt alphasatellite 7a
1980173	GCA_003029455.1	Sophora alopecuroides yellow stunt alphasatellite 8
1980174	GCA_003029505.1	Sophora alopecuroides yellow stunt alphasatellite 9

1891704	GCA_001722805.1	Sophora japonica powdery mildew-associated partitivirus
107804	GCA_000850905.1	Sorghum chlorotic spot virus
32619	GCA_000856865.1	Sorghum mosaic virus
1452514	GCA_000924495.1	Sosuga virus
2010246	GCA_003032635.1	Souris virus
63723	GCA_000838365.1	South African cassava mosaic virus
12139	GCA_000860745.1	Southern bean mosaic virus
12139	GCA_002830905.1	Southern bean mosaic virus
196398	GCA_000863105.1	Southern cowpea mosaic virus
1159195	GCA_000895355.1	Southern elephant seal virus
519497	GCA_000889455.1	Southern rice black-streaked dwarf virus
591166	GCA_000883395.1	Southern tomato virus
1546178	GCA_000924335.1	Southwest baboon virus 1
2016402	GCA_003032655.1	Southwest carpet python virus
378833	GCA_000881455.1	Sowbane mosaic virus
408136	GCA_002823505.1	Soybean blistering mosaic virus
761702	GCA_000889935.1	Soybean chlorotic blotch virus
10651	GCA_000847985.1	Soybean chlorotic mottle virus
1221206	GCA_000897535.1	Soybean chlorotic spot virus
1034377	GCA_000923415.1	Soybean cyst nematode socyvirus
1495316	GCA_000918195.1	Soybean cyst nematode virus 5
12049	GCA_000848565.1	Soybean dwarf virus
1927714	GCA_001923735.1	Soybean latent spherical virus
1719040	GCA_001448415.1	Soybean leaf-associated gemycircularvirus 1
1719040	GCA_001448415.2	Soybean leaf-associated gemycircularvirus 1
1719048	GCA_003673825.1	Soybean leaf-associated negative-stranded RNA virus 1
1719049	GCA_003673845.1	Soybean leaf-associated negative-stranded RNA virus 2
1719050	GCA_003673865.1	Soybean leaf-associated negative-stranded RNA virus 3
1719051	GCA_003673885.1	Soybean leaf-associated negative-stranded RNA virus 4
761701	GCA_000888375.1	Soybean mild mottle virus
12222	GCA_002987665.1	Soybean mosaic virus
12223	GCA_000862465.1	Soybean mosaic virus N
1221449	GCA_000899175.1	Soybean Putnam virus
1080798	GCA_000893015.1	Soybean yellow common mosaic virus
578361	GCA_000881895.1	Soybean yellow mottle mosaic virus
1691889	GCA_001271035.1	Spanish goat encephalitis virus
1159906	GCA_000868165.1	Sparrow coronavirus HKU17
1885928	GCA_001717215.1	Sparus aurata papillomavirus 1
1885927	GCA_001717195.1	Sparus aurata polyomavirus 1
73497	GCA_000850285.1	Sphaeropsis sapinea RNA virus 1
73498	GCA_000848425.1	Sphaeropsis sapinea RNA virus 2
2169402	GCA_001974335.1	Sphenicid alphaherpesvirus 1
2169402	GCA_900095425.1	Sphenicid alphaherpesvirus 1
1980139	GCA_002622365.1	Sphingobium phage Lacusarx
1150991	GCA_000902455.1	Sphingomonas phage PAU
2282912	GCA_003423285.1	Sphingomonas phage Scott
2293302	GCA_003847345.1	Spider associated circular virus 1
2293302	GCA_003847365.1	Spider associated circular virus 1
2293302	GCA_003847385.1	Spider associated circular virus 1
2293303	GCA_003847325.1	Spider associated circular virus 2

2293304	GCA_003846685.1	Spider associated circular virus 3
2170200	GCA_003032805.1	Spider monkey simian foamy virus
390439	GCA_000873265.1	Spilanthes yellow vein virus
1985164	GCA_002204065.1	Spinach amalgavirus 1
1937817	GCA_002004375.1	Spinach cryptic virus 1
945225	GCA_000893115.1	Spinach curly top Arizona virus
42680	GCA_000850785.1	Spinach latent virus
873160	GCA_000888695.1	Spinach severe curly top virus
1432565	GCA_000915875.1	Spinach yellow vein Sikar virus
2293305	GCA_003847085.1	Spinybacked orbweaver circular virus 1
2293305	GCA_003847065.1	Spinybacked orbweaver circular virus 1
2293306	GCA_003847145.1	Spinybacked orbweaver circular virus 2
185959	GCA_000839205.1	Spiroplasma phage 1-C74
10854	GCA_000847925.1	Spiroplasma phage 1-R8A2B
745349	GCA_002630705.1	Spiroplasma phage SVGII3
93224	GCA_000838525.1	Spiroplasma phage SVTS2
455649	GCA_000872145.1	Spiroplasma virus SkV1CR23x
10855	GCA_000839985.1	Spiroplasma virus SpV4
1004049	GCA_000896795.1	Spissistilus festinus reovirus
862943	GCA_000888455.1	Spissistilus festinus virus 1
1111710	GCA_000895015.1	Spodoptera exigua iflavirus 1
1228012	GCA_000917415.1	Spodoptera exigua iflavirus 2
10454	GCA_000529585.1	Spodoptera exigua multiple nucleopolyhedrovirus
10454	GCA_000530255.2	Spodoptera exigua multiple nucleopolyhedrovirus
10454	GCA_000689295.1	Spodoptera exigua multiple nucleopolyhedrovirus
10454	GCA_000530085.1	Spodoptera exigua multiple nucleopolyhedrovirus
10454	GCA_000530135.1	Spodoptera exigua multiple nucleopolyhedrovirus
10454	GCA_000839865.1	Spodoptera exigua multiple nucleopolyhedrovirus
10454	GCA_000530235.2	Spodoptera exigua multiple nucleopolyhedrovirus
10454	GCA_000529295.1	Spodoptera exigua multiple nucleopolyhedrovirus
113370	GCA_000867605.1	Spodoptera frugiperda ascovirus 1a
307454	GCA_000943625.1	Spodoptera frugiperda granulovirus
10455	GCA_000868825.1	Spodoptera frugiperda multiple nucleopolyhedrovirus
1481139	GCA_000927195.1	Spodoptera frugiperda rhabdovirus
10456	GCA_002819145.1	Spodoptera littoralis nucleopolyhedrovirus
359919	GCA_000871585.1	Spodoptera litura granulovirus
46242	GCA_000839885.1	Spodoptera litura nucleopolyhedrovirus
566270	GCA_000881875.1	Spodoptera litura nucleopolyhedrovirus II
64318	GCA_001610725.1	Spondweni virus
1302849	GCA_000897615.1	Sporobolus striate mosaic virus 1
1302850	GCA_000899215.1	Sporobolus striate mosaic virus 2
188141	GCA_000854785.1	Spring beauty latent virus
543939	GCA_000880395.1	Sputnik virophage
1133031	GCA_000919615.1	Sputnik virophage 2
1133031	GCA_002954995.1	Sputnik virophage 2
1133032	GCA_000920455.1	Sputnik virophage 3
1810396	GCA_002219665.1	Squash chlorotic leaf spot virus
269275	GCA_000865805.1	Squash leaf curl China virus - [B]
269277	GCA_000843565.1	Squash leaf curl Philippines virus
10829	GCA_000837705.1	Squash leaf curl virus

222474	GCA_000858265.1	Squash leaf curl Yunnan virus
223325	GCA_000844565.1	Squash mild leaf curl virus-[Imperial Valley]
12263	GCA_000861625.1	Squash mosaic virus
397544	GCA_000879215.1	Squash vein yellowing virus
1968887	GCA_002219645.1	Squirrel adenovirus 1
452475	GCA_000873725.1	Squirrel monkey polyomavirus
11857	GCA_000851925.1	Squirrel monkey retrovirus-H
2170201	GCA_003032815.1	Squirrel monkey simian foamy virus
240426	GCA_000913615.1	Squirrelpox virus
2029293	GCA_002354965.1	Squirrelpox virus Berlin_2015
475454	GCA_002986875.1	Sri Lankan cassava mosaic virus - [Ker20]
223328	GCA_000858725.1	Sri Lankan cassava mosaic virus-[Colombo]
1620897	GCA_002146185.1	Sripur virus
104581	GCA_000856145.1	St Croix River virus
223329	GCA_000843765.1	Stachytarpheta leaf curl virus - [Hn5]
2496589	GCA_004355635.1	Staphylococcal phage MR003
1505027	GCA_001504695.1	Staphylococcus phage 23MRA
320836	GCA_000858605.1	Staphylococcus phage 2638A
1934428	GCA_002618805.1	Staphylococcus phage 3 AJ-2017
1505026	GCA_001503095.1	Staphylococcus phage 3MRA
1675528	GCA_002756655.1	Staphylococcus phage 55-2
1675529	GCA_002756675.1	Staphylococcus phage 55-3
320832	GCA_000858585.1	Staphylococcus phage 66
1195081	GCA_002597585.1	Staphylococcus phage 676Z
1500386	GCA_000921075.1	Staphylococcus phage 6ec
307898	GCA_001551285.1	Staphylococcus phage 812
307898	GCA_002921835.1	Staphylococcus phage 812
307898	GCA_002921855.1	Staphylococcus phage 812
307898	GCA_002921845.1	Staphylococcus phage 812
307898	GCA_002921825.1	Staphylococcus phage 812
307898	GCA_003862095.1	Staphylococcus phage 812
2419617	GCA_003862115.1	Staphylococcus phage 812h1
1195077	GCA_002597565.1	Staphylococcus phage A3R
516541	GCA_002597665.1	Staphylococcus phage A5W
1958907	GCA_002619185.1	Staphylococcus phage Andhra
2059854	GCA_003085775.1	Staphylococcus phage B1
1636204	GCA_001503015.1	Staphylococcus phage B166
1636205	GCA_001504615.1	Staphylococcus phage B236
1543206	GCA_001745035.1	Staphylococcus phage BP39
2510150	GCA_004147065.1	Staphylococcus phage CH1
1792269	GCA_001755305.1	Staphylococcus phage CNPx
2108359	GCA_004284875.1	Staphylococcus phage CSA13
1464127	GCA_000923675.1	Staphylococcus phage DW2
1195078	GCA_002597605.1	Staphylococcus phage Fi200W
760530	GCA_000901675.1	Staphylococcus phage GH15
1453367	GCA_000914715.1	Staphylococcus phage GRCS
2492948	GCA_004138715.1	Staphylococcus phage Henu2
2054877	GCA_003861715.1	Staphylococcus phage HSA30
2059858	GCA_003861735.1	Staphylococcus phage HSA84
2163024	GCA_003059805.1	Staphylococcus phage HYZ21

1965486	GCA_002620745.1	Staphylococcus phage IME1318_01
1965487	GCA_002620765.1	Staphylococcus phage IME1323_01
1965492	GCA_002620865.1	Staphylococcus phage IME1346_01
1965489	GCA_002620805.1	Staphylococcus phage IME1348_01
1965493	GCA_002620885.1	Staphylococcus phage IME1354_01
1965490	GCA_002620825.1	Staphylococcus phage IME1361_01
1965536	GCA_002621005.1	Staphylococcus phage IME1364_01
1965494	GCA_002620905.1	Staphylococcus phage IME1365_01
1965491	GCA_002620845.1	Staphylococcus phage IME1367_01
1965533	GCA_002620945.1	Staphylococcus phage IME1367_02
1610830	GCA_002597685.1	Staphylococcus phage IME-SA1
1673884	GCA_002597725.1	Staphylococcus phage IME-SA118
1673874	GCA_002597745.1	Staphylococcus phage IME-SA119
1610831	GCA_002597705.1	Staphylococcus phage IME-SA2
1610872	GCA_001502695.1	Staphylococcus phage IME-SA4
1028375	GCA_002597505.1	Staphylococcus phage ISP
2024236	GCA_003085795.1	Staphylococcus phage JA1
2483712	GCA_003864235.1	Staphylococcus phage JBug18
1239383	GCA_000903675.1	Staphylococcus phage JD007
1273712	GCA_000907895.1	Staphylococcus phage JS01
1296641	GCA_002603325.1	Staphylococcus phage LH1
2234038	GCA_003342375.1	Staphylococcus phage Lorac
1524910	GCA_000924855.1	Staphylococcus phage MCE-2014
1916103	GCA_002615625.1	Staphylococcus phage MRSA107
1195079	GCA_002597625.1	Staphylococcus phage MSA6
1526408	GCA_000926755.1	Staphylococcus phage P108
1840236	GCA_002609985.1	Staphylococcus phage P1105
1920379	GCA_002617225.1	Staphylococcus phage P240
1735645	GCA_002607605.1	Staphylococcus phage P282
1195080	GCA_002597645.1	Staphylococcus phage P4W
1735646	GCA_002607625.1	Staphylococcus phage P630
204090	GCA_000842185.1	Staphylococcus phage P68
668618	GCA_000886755.1	Staphylococcus phage P954
2483713	GCA_003864175.1	Staphylococcus phage Pabna
1542124	GCA_003060665.1	Staphylococcus phage Phi05_1554 UV
1542125	GCA_003060685.1	Staphylococcus phage Phi05_2289 M, S, UV
1542126	GCA_003060095.1	Staphylococcus phage Phi06_2986 B
1542127	GCA_003060005.1	Staphylococcus phage Phi06_3106 B
1542128	GCA_003059915.1	Staphylococcus phage Phi06_3244 B
430796	GCA_000880655.1	Staphylococcus phage phi2958PVL
1955176	GCA_002619005.1	Staphylococcus phage phi575
874267	GCA_000903855.1	Staphylococcus phage phi5967PVL
874268	GCA_002630785.1	Staphylococcus phage phi7247PVL
1165702	GCA_000906595.1	Staphylococcus phage phi7401PVL
1955181	GCA_002619025.1	Staphylococcus phage phi879
1519999	GCA_000930755.1	Staphylococcus phage phiBU01
1340769	GCA_002622385.1	Staphylococcus phage phiIBB-SEP1
1572704	GCA_001501675.1	Staphylococcus phage phiIPLA-C1C
1572703	GCA_001504675.1	Staphylococcus phage phiIPLA-RODI
1698421	GCA_001470875.1	Staphylococcus phage phiJB

387909	GCA_000870305.1	Staphylococcus phage phiNM3
1589269	GCA_002630085.1	Staphylococcus phage phiNM4-gamma4
648017	GCA_000886435.1	Staphylococcus phage phiPVL-CN125
1403390	GCA_000913115.1	Staphylococcus phage phiRS7
2126734	GCA_003023925.1	Staphylococcus phage phiSA_BS1
2126724	GCA_003034595.1	Staphylococcus phage phiSA_BS2
2028134	GCA_002989755.1	Staphylococcus phage phiSA039
1498220	GCA_000926815.1	Staphylococcus phage phiSa119
1450142	GCA_000917015.1	Staphylococcus phage phiSA12
2029064	GCA_002743675.1	Staphylococcus phage phiSa2wa_st1
2060948	GCA_002957805.1	Staphylococcus phage phiSa2wa_st121mssa
2060949	GCA_002957815.1	Staphylococcus phage phiSa2wa_st22
2060950	GCA_002957825.1	Staphylococcus phage phiSa2wa_st30
2060951	GCA_002957835.1	Staphylococcus phage phiSa2wa_st5
2060952	GCA_002957845.1	Staphylococcus phage phiSa2wa_st59
2060953	GCA_002957855.1	Staphylococcus phage phiSa2wa_st72
2060954	GCA_002957865.1	Staphylococcus phage phiSa2wa_st78
2060955	GCA_002957875.1	Staphylococcus phage phiSa2wa_st80
2060956	GCA_002957885.1	Staphylococcus phage phiSa2wa_st93
2060957	GCA_002957895.1	Staphylococcus phage phiSa2wa_st93mssa
2483714	GCA_003864195.1	Staphylococcus phage Pike
2483715	GCA_003864215.1	Staphylococcus phage Pontiff
1319980	GCA_002633585.1	Staphylococcus phage PSa3
1961797	GCA_002619705.1	Staphylococcus phage pSa-3
320831	GCA_000860185.1	Staphylococcus phage PT1028
71366	GCA_000843665.1	Staphylococcus phage PVL
1970744	GCA_002621105.1	Staphylococcus phage qdsa001
1970746	GCA_002621125.1	Staphylococcus phage qdsa002
2234036	GCA_003368645.1	Staphylococcus phage Quidividi
320843	GCA_000860245.1	Staphylococcus phage ROSA
1010615	GCA_002600655.1	Staphylococcus phage S13'
1010614	GCA_000895755.1	Staphylococcus phage S24-1
1041526	GCA_000913135.1	Staphylococcus phage S25-3
1041527	GCA_000911475.1	Staphylococcus phage S25-4
694060	GCA_001308695.1	Staphylococcus phage SA1
2268607	GCA_003368685.1	Staphylococcus phage SA1014ruMSSAST7
1347760	GCA_000908995.1	Staphylococcus phage SA12
1195086	GCA_000908135.1	Staphylococcus phage SA13
2268608	GCA_003368705.1	Staphylococcus phage SA137ruMSSAST121PVL
2510151	GCA_004147085.1	Staphylococcus phage Sa30
2268621	GCA_003368785.1	Staphylococcus phage SA345ruMSSAST8
2268622	GCA_003368745.1	Staphylococcus phage SA45ruMSSAST97
1239385	GCA_002597525.1	Staphylococcus phage SA5
2268623	GCA_003368765.1	Staphylococcus phage SA537ruMSSAST97
1969736	GCA_002621065.1	Staphylococcus phage SA7
2268609	GCA_003575805.1	Staphylococcus phage SA780ruMSSAST101
1498171	GCA_001504335.1	Staphylococcus phage SA97
470865	GCA_000872005.1	Staphylococcus phage SAP-2
1007127	GCA_000914175.1	Staphylococcus phage Sb1
1913581	GCA_002615465.1	Staphylococcus phage SCH1

1913582	GCA_002615485.1	Staphylococcus phage SCH111
2066503	GCA_002957945.1	Staphylococcus phage SH-St 15644
1821228	GCA_001746055.1	Staphylococcus phage SLPW
1168612	GCA_000900875.1	Staphylococcus phage SMSAP5
2024210	GCA_002627685.1	Staphylococcus phage SN10
2024211	GCA_002627705.1	Staphylococcus phage SN11
2024212	GCA_002627725.1	Staphylococcus phage SN13
2024213	GCA_002627745.1	Staphylococcus phage SN8
1211285	GCA_002602525.1	Staphylococcus phage SP5
1211286	GCA_002602545.1	Staphylococcus phage SP6
1118063	GCA_000898375.1	Staphylococcus phage SpaA1
1732063	GCA_001551345.1	Staphylococcus phage SPbeta-like
1913446	GCA_002615285.1	Staphylococcus phage SpT152
1913447	GCA_002615305.1	Staphylococcus phage SpT252
1913448	GCA_002615325.1	Staphylococcus phage SpT5
1913449	GCA_002615345.1	Staphylococcus phage SpT99F3
1958922	GCA_002619325.1	Staphylococcus phage St 134
1913667	GCA_002615525.1	Staphylococcus phage StAP1
1195076	GCA_002597545.1	Staphylococcus phage Staph1N
1200862	GCA_001737115.1	Staphylococcus phage Stau2
1195068	GCA_000910015.1	Staphylococcus phage StauST398-1
1277672	GCA_000910095.1	Staphylococcus phage StauST398-2
1195071	GCA_000908535.1	Staphylococcus phage StauST398-3
1308896	GCA_000914455.1	Staphylococcus phage StauST398-4
1308897	GCA_000916135.1	Staphylococcus phage StauST398-5
1147042	GCA_000905915.2	Staphylococcus phage StB12
1147043	GCA_000904855.1	Staphylococcus phage StB20
1732064	GCA_001504075.1	Staphylococcus phage StB20-like
1147044	GCA_000903815.1	Staphylococcus phage StB27
1262512	GCA_000924875.1	Staphylococcus phage Team1
1168613	GCA_000896255.1	Staphylococcus phage TEM123
947066	GCA_002633665.1	Staphylococcus phage TEM126
2282407	GCA_003575625.1	Staphylococcus phage Terranova
445515	GCA_000874165.2	Staphylococcus phage tp310-1
445516	GCA_000873505.2	Staphylococcus phage tp310-2
445517	GCA_000871065.2	Staphylococcus phage tp310-3
2234037	GCA_003368665.1	Staphylococcus phage Twillingate
2029631	GCA_002955925.1	Staphylococcus phage UPMK_2
1981514	GCA_002622585.1	Staphylococcus phage vB_Sau_CG
1981552	GCA_002622605.1	Staphylococcus phage vB_Sau_Clo6
1981553	GCA_002622625.1	Staphylococcus phage vB_Sau_S24
2161785	GCA_003059745.1	Staphylococcus phage vB_SauM_0414_108
2070179	GCA_002958045.1	Staphylococcus phage vB_SauM_LM12
1235659	GCA_000910895.1	Staphylococcus phage vB_SauM_Remus
1235660	GCA_000907055.1	Staphylococcus phage vB_SauM_Romulus
2024543	GCA_002629025.1	Staphylococcus phage vB_SauM-fRuSau02
2074138	GCA_002958295.1	Staphylococcus phage vB_SauP_phiAGO1.3
2074139	GCA_002958305.1	Staphylococcus phage vB_SauP_phiAGO1.9
2510149	GCA_004147105.1	Staphylococcus phage vB_SauS_fPfSau02
1852565	GCA_002610065.1	Staphylococcus phage vB_SauS_IMEP5

1674930	GCA_001502335.1	Staphylococcus phage vB_SauS_phi2
2305035	GCA_003693745.1	Staphylococcus phage VB_SavM_JYL01
1434319	GCA_000915755.1	Staphylococcus phage vB_SepS_SEP9
1868844	GCA_002611205.1	Staphylococcus phage vB_SscM-1
1868845	GCA_002611225.1	Staphylococcus phage vB_SscM-2
2234082	GCA_003307435.1	Staphylococcus phage VB-SauS-SA2
2500148	GCA_004015985.1	Staphylococcus phage VB-SavM-JYL02
1399949	GCA_000912915.1	Staphylococcus phage YMC/09/04/R1988
129009	GCA_000839005.1	Staphylococcus prophage phiPV83
360398	GCA_000867845.1	Staphylococcus virus 108PVL
12360	GCA_000841245.1	Staphylococcus virus 11
186153	GCA_000842085.1	Staphylococcus virus 13
55511	GCA_000857625.1	Staphylococcus virus 187
320846	GCA_000859425.1	Staphylococcus virus 29
320840	GCA_000860325.1	Staphylococcus virus 37
215167	GCA_000859385.1	Staphylococcus virus 3a
320837	GCA_000857645.1	Staphylococcus virus 42e
204086	GCA_000843045.1	Staphylococcus virus 44AHJD
320839	GCA_000860225.1	Staphylococcus virus 47
320847	GCA_000860265.1	Staphylococcus virus 52a
320835	GCA_000860205.1	Staphylococcus virus 53
320845	GCA_000857685.1	Staphylococcus virus 55
320834	GCA_000859365.1	Staphylococcus virus 69
320844	GCA_000860345.1	Staphylococcus virus 71
259901	GCA_000840945.1	Staphylococcus virus 77
405947	GCA_001689815.1	Staphylococcus virus 80
53369	GCA_000871605.1	Staphylococcus virus 80alpha
59506	GCA_000857605.1	Staphylococcus virus 85
320848	GCA_000860365.1	Staphylococcus virus 88
320849	GCA_000857705.1	Staphylococcus virus 92
320842	GCA_000859405.1	Staphylococcus virus 96
2500557	GCA_004016065.1	Staphylococcus virus B122
398839	GCA_000868745.1	Staphylococcus virus CNPH82
320841	GCA_000857665.1	Staphylococcus virus EW
292029	GCA_000859345.1	Staphylococcus virus G1
575610	GCA_000881855.1	Staphylococcus virus IPLA35
1197952	GCA_000899895.1	Staphylococcus virus IPLA5
1197953	GCA_000897455.1	Staphylococcus virus IPLA7
575608	GCA_000880915.1	Staphylococcus virus IPLA88
2508849	GCA_004139295.1	Staphylococcus virus J-Sa36
221915	GCA_000846645.1	Staphylococcus virus K
221915	GCA_003051665.1	Staphylococcus virus K
399185	GCA_000870365.1	Staphylococcus virus PH15
186152	GCA_000842945.1	Staphylococcus virus phi12
106284	GCA_000837405.1	Staphylococcus virus phiETA
326036	GCA_000867945.1	Staphylococcus virus phiETA2
326037	GCA_000868805.1	Staphylococcus virus phiETA3
379501	GCA_000872225.1	Staphylococcus virus phiMR11
487152	GCA_000874465.1	Staphylococcus virus phiMR25
387907	GCA_000870285.1	Staphylococcus virus phiNM1

387908	GCA_001501355.1	Staphylococcus virus phiNM2
387910	GCA_001500695.1	Staphylococcus virus phiNM4
130478	GCA_000837385.1	Staphylococcus virus phiSLT
1204543	GCA_000901915.1	Staphylococcus virus SA11
2508850	GCA_004139255.1	Staphylococcus virus Sa83
2508851	GCA_004139275.1	Staphylococcus virus Sa87
872294	GCA_000888535.1	Staphylococcus virus SAP26
55510	GCA_000858505.1	Staphylococcus virus Twort
320850	GCA_000859445.1	Staphylococcus virus X2
349370	GCA_000868125.1	Starling circovirus
349370	GCA_004075675.1	Starling circovirus
349370	GCA_004075655.1	Starling circovirus
436911	GCA_000879655.1	Steller sea lion vesivirus
2499625	GCA_004128135.1	Stemphylium lycopersici mycovirus
2047758	GCA_002956155.1	Stenotrophomonas phage DLP4
1211280	GCA_001503215.1	Stenotrophomonas phage IME13
1239382	GCA_000903135.1	Stenotrophomonas phage IME15
1654717	GCA_002606605.1	Stenotrophomonas phage IME-SM1
1027253	GCA_000891155.1	Stenotrophomonas phage phiSHP2
1346893	GCA_002625225.1	Stenotrophomonas phage phiSMA6
2011087	GCA_002826545.1	Stenotrophomonas phage PSH1
573591	GCA_000882475.1	Stenotrophomonas phage S1
1343494	GCA_000908815.1	Stenotrophomonas phage SMA7
334856	GCA_000863885.1	Stenotrophomonas phage SMA9
1168563	GCA_000917355.1	Stenotrophomonas phage Smp131
2044561	GCA_002956145.1	Stenotrophomonas phage vB_SmaS_DLP_5
1642588	GCA_002606745.1	Stenotrophomonas phage vB_SmaS-DLP_1
1642663	GCA_001501575.1	Stenotrophomonas phage vB_SmaS-DLP_2
1651816	GCA_002606585.1	Stenotrophomonas phage vB_SmaS-DLP_6
2555548	GCA_004521575.1	Stenotrophomonas phage YB07
2282646	GCA_004134145.1	Stipagrostis associaed virus
1277649	GCA_000904055.1	STL polyomavirus
110944	GCA_003047495.1	Stork hepatitis B virus
399314	GCA_000869405.1	Strawberry chlorotic fleck-associated virus
28351	GCA_000857165.1	Strawberry latent ringspot virus
195062	GCA_000858845.1	Strawberry latent ringspot virus satellite RNA
12187	GCA_000855525.1	Strawberry mild yellow edge virus
167161	GCA_000850445.1	Strawberry mottle virus
243563	GCA_000869645.1	Strawberry necrotic shock virus
227507	GCA_000855845.1	Strawberry pallidosis-associated virus
1564903	GCA_000927455.1	Strawberry polerovirus 1
47903	GCA_000857365.1	Strawberry vein banding virus
335187	GCA_000870125.1	Streptocarpus flower break virus
2484225	GCA_003865835.1	Streptococcus phage 109751
1718281	GCA_002607485.1	Streptococcus phage 128
2041494	GCA_003012575.1	Streptococcus phage 16B8
1392231	GCA_000916975.1	Streptococcus phage 20617
2041495	GCA_003012585.1	Streptococcus phage 31B4
2484226	GCA_003865815.1	Streptococcus phage 33888
646413	GCA_000884895.1	Streptococcus phage 5093

1718280	GCA_002607465.1	Streptococcus phage 53
1718279	GCA_002607445.1	Streptococcus phage 73
2041496	GCA_003012545.1	Streptococcus phage 7A5
2041497	GCA_003012525.1	Streptococcus phage 7T
2041498	GCA_003012555.1	Streptococcus phage 9A
2041499	GCA_003012565.1	Streptococcus phage 9B4
2041500	GCA_003012535.1	Streptococcus phage A0
1701850	GCA_001470335.1	Streptococcus phage A25
1647391	GCA_001501955.1	Streptococcus phage APCM01
2041501	GCA_003012595.1	Streptococcus phage B0
2041502	GCA_003012605.1	Streptococcus phage B5
2041503	GCA_003012615.1	Streptococcus phage C0
2365005	GCA_003866255.1	Streptococcus phage CHPC1005
2365006	GCA_003866835.1	Streptococcus phage CHPC1008
2365007	GCA_003866275.1	Streptococcus phage CHPC1014
2365008	GCA_003866295.1	Streptococcus phage CHPC1027
2365009	GCA_003866315.1	Streptococcus phage CHPC1029
2365010	GCA_003866855.1	Streptococcus phage CHPC1033
2365011	GCA_003933115.1	Streptococcus phage CHPC1034
2365012	GCA_003866335.1	Streptococcus phage CHPC1036
2365013	GCA_003933135.1	Streptococcus phage CHPC1037
2365014	GCA_003866355.1	Streptococcus phage CHPC1040
2365015	GCA_003866375.1	Streptococcus phage CHPC1041
2365016	GCA_003866395.1	Streptococcus phage CHPC1042
2365017	GCA_003866875.1	Streptococcus phage CHPC1045
2365018	GCA_003866415.1	Streptococcus phage CHPC1046
2365019	GCA_003866435.1	Streptococcus phage CHPC1048
2365020	GCA_003866455.1	Streptococcus phage CHPC1057
2365021	GCA_003866495.1	Streptococcus phage CHPC1062
2365022	GCA_003866515.1	Streptococcus phage CHPC1067
2365023	GCA_003866535.1	Streptococcus phage CHPC1073
2365024	GCA_003866555.1	Streptococcus phage CHPC1083
2365025	GCA_003866575.1	Streptococcus phage CHPC1084
2365026	GCA_003866595.1	Streptococcus phage CHPC1091
2365028	GCA_003866615.1	Streptococcus phage CHPC1148
1913083	GCA_002615045.1	Streptococcus phage CHPC1151
2365029	GCA_003866915.1	Streptococcus phage CHPC1152
2365030	GCA_003866635.1	Streptococcus phage CHPC1156
2365031	GCA_003866655.1	Streptococcus phage CHPC1230
2365032	GCA_003866675.1	Streptococcus phage CHPC1246
2365033	GCA_003866695.1	Streptococcus phage CHPC1247
2365034	GCA_003866715.1	Streptococcus phage CHPC1248
2365035	GCA_003865895.1	Streptococcus phage CHPC572
1913081	GCA_002615005.1	Streptococcus phage CHPC577
2365036	GCA_003866735.1	Streptococcus phage CHPC595
2365037	GCA_003865915.1	Streptococcus phage CHPC640
2365038	GCA_003865775.1	Streptococcus phage CHPC642
2365039	GCA_003865935.1	Streptococcus phage CHPC663
2365040	GCA_003865955.1	Streptococcus phage CHPC676
2365041	GCA_003865975.1	Streptococcus phage CHPC869

2365042	GCA_003865995.1	Streptococcus phage CHPC873
2365043	GCA_003866025.1	Streptococcus phage CHPC875
2365044	GCA_003866055.1	Streptococcus phage CHPC877
2365045	GCA_003866755.1	Streptococcus phage CHPC879
2365046	GCA_003866075.1	Streptococcus phage CHPC919
2365047	GCA_003866095.1	Streptococcus phage CHPC925
1913082	GCA_002615025.1	Streptococcus phage CHPC926
2365048	GCA_003866115.1	Streptococcus phage CHPC927
2365049	GCA_003866135.1	Streptococcus phage CHPC928
2365050	GCA_003866155.1	Streptococcus phage CHPC929
2365051	GCA_003866775.1	Streptococcus phage CHPC930
2365052	GCA_003866175.1	Streptococcus phage CHPC931
2365053	GCA_003933155.1	Streptococcus phage CHPC933
2365054	GCA_003866195.1	Streptococcus phage CHPC950
2365055	GCA_003866215.1	Streptococcus phage CHPC951
2365056	GCA_003866795.1	Streptococcus phage CHPC952
2365057	GCA_003866235.1	Streptococcus phage CHPC954
2365058	GCA_003866815.1	Streptococcus phage CHPC979
2365059	GCA_003933095.1	Streptococcus phage CHPC982
10747	GCA_000849625.1	Streptococcus phage Cp-1
10748	GCA_002988105.1	Streptococcus phage CP-7
2108109	GCA_003182725.1	Streptococcus phage D1024
2108111	GCA_003182745.1	Streptococcus phage D1811
2006928	GCA_002625105.1	Streptococcus phage D4276
2108112	GCA_003182705.1	Streptococcus phage D5842
1448273	GCA_000921875.1	Streptococcus phage DCC1738
59241	GCA_000893335.1	Streptococcus phage Dp-1
12402	GCA_000848885.1	Streptococcus phage EJ-1
1448276	GCA_000922935.1	Streptococcus phage IC1
1916151	GCA_002615885.1	Streptococcus phage IPP10
1916152	GCA_002615905.1	Streptococcus phage IPP11
1916153	GCA_002615925.1	Streptococcus phage IPP12
1916154	GCA_002615945.1	Streptococcus phage IPP14
1916155	GCA_002615965.1	Streptococcus phage IPP15
1916156	GCA_002615985.1	Streptococcus phage IPP16
1916157	GCA_002616005.1	Streptococcus phage IPP17
1916158	GCA_002616025.1	Streptococcus phage IPP18
1916159	GCA_002616045.1	Streptococcus phage IPP19
1916160	GCA_002616065.1	Streptococcus phage IPP20
1916161	GCA_002616085.1	Streptococcus phage IPP21
1916162	GCA_002616105.1	Streptococcus phage IPP22
1916163	GCA_002616125.1	Streptococcus phage IPP23
1916164	GCA_002616145.1	Streptococcus phage IPP24
1916165	GCA_002616165.1	Streptococcus phage IPP25
1916166	GCA_002616185.1	Streptococcus phage IPP26
1916167	GCA_002616205.1	Streptococcus phage IPP27
1916168	GCA_002616225.1	Streptococcus phage IPP28
1916169	GCA_002616245.1	Streptococcus phage IPP29
1916170	GCA_002616265.1	Streptococcus phage IPP30
1916171	GCA_002616285.1	Streptococcus phage IPP31

1916172	GCA_002616305.1	Streptococcus phage IPP32
1916173	GCA_002616325.1	Streptococcus phage IPP34
1916174	GCA_002616345.1	Streptococcus phage IPP35
1916175	GCA_002616365.1	Streptococcus phage IPP36
1916177	GCA_002616385.1	Streptococcus phage IPP38
1916178	GCA_002616405.1	Streptococcus phage IPP39
1916179	GCA_002616425.1	Streptococcus phage IPP40
1916180	GCA_002616445.1	Streptococcus phage IPP41
1916181	GCA_002616465.1	Streptococcus phage IPP42
1916182	GCA_002616485.1	Streptococcus phage IPP43
1916183	GCA_002616505.1	Streptococcus phage IPP44
1916184	GCA_002616525.1	Streptococcus phage IPP45
1916185	GCA_002616545.1	Streptococcus phage IPP46
1916186	GCA_002616565.1	Streptococcus phage IPP48
1916187	GCA_002616585.1	Streptococcus phage IPP5
1916188	GCA_002616605.1	Streptococcus phage IPP50
1916189	GCA_002616625.1	Streptococcus phage IPP51
1916190	GCA_002616645.1	Streptococcus phage IPP52
1916191	GCA_002616665.1	Streptococcus phage IPP53
1916192	GCA_002616685.1	Streptococcus phage IPP54
1916193	GCA_002616705.1	Streptococcus phage IPP55
1916194	GCA_002616725.1	Streptococcus phage IPP57
1916195	GCA_002616745.1	Streptococcus phage IPP58
1916196	GCA_002616765.1	Streptococcus phage IPP60
1916197	GCA_002616785.1	Streptococcus phage IPP61
1916198	GCA_002616805.1	Streptococcus phage IPP62
1916199	GCA_002616825.1	Streptococcus phage IPP63
1916200	GCA_002616845.1	Streptococcus phage IPP64
1916201	GCA_002616865.1	Streptococcus phage IPP65
1916202	GCA_002616885.1	Streptococcus phage IPP66
1916203	GCA_002616905.1	Streptococcus phage IPP67
1916204	GCA_002616925.1	Streptococcus phage IPP68
1916205	GCA_002616945.1	Streptococcus phage IPP69
1916206	GCA_002616965.1	Streptococcus phage IPP8
1916207	GCA_002616985.1	Streptococcus phage IPP9
1225806	GCA_002602645.1	Streptococcus phage JX01
1448274	GCA_000921895.1	Streptococcus phage K13
2041504	GCA_003012625.1	Streptococcus phage L5A1
2419523	GCA_003668435.1	Streptococcus phage LF1
2419524	GCA_003668455.1	Streptococcus phage LF2
2419525	GCA_003668475.1	Streptococcus phage LF3
2419526	GCA_003668495.1	Streptococcus phage LF4
1225805	GCA_002602625.1	Streptococcus phage LYGO9
372457	GCA_000884015.1	Streptococcus phage M102
1587907	GCA_001504655.1	Streptococcus phage M102AD
2041505	GCA_003012635.1	Streptococcus phage M19
120574	GCA_000849705.1	Streptococcus phage MM1
341698	GCA_002602445.1	Streptococcus phage MM1 1998
2041506	GCA_003012645.1	Streptococcus phage MM25
1971410	GCA_002621325.1	Streptococcus phage P0091

1971411	GCA_002621345.1	Streptococcus phage P0092
1971412	GCA_002621365.1	Streptococcus phage P0093
1971413	GCA_002621385.1	Streptococcus phage P0094
1971414	GCA_002621405.1	Streptococcus phage P0095
1971415	GCA_002621425.1	Streptococcus phage P3681
1971416	GCA_002621445.1	Streptococcus phage P3684
1971417	GCA_002621465.1	Streptococcus phage P4761
1971418	GCA_002621485.1	Streptococcus phage P5641
1971419	GCA_002621505.1	Streptococcus phage P5651
1971420	GCA_002621525.1	Streptococcus phage P5652
1971421	GCA_002621545.1	Streptococcus phage P7132
1971422	GCA_002621565.1	Streptococcus phage P7133
1971423	GCA_002621585.1	Streptococcus phage P7134
1971424	GCA_002621605.1	Streptococcus phage P7151
1971425	GCA_002621625.1	Streptococcus phage P7152
1971426	GCA_002621645.1	Streptococcus phage P7154
1971427	GCA_002621665.1	Streptococcus phage P7571
1971428	GCA_002621685.1	Streptococcus phage P7572
1971429	GCA_002621705.1	Streptococcus phage P7573
1971430	GCA_002621725.1	Streptococcus phage P7574
1971431	GCA_002621745.1	Streptococcus phage P7601
1971432	GCA_002621765.1	Streptococcus phage P7602
1971433	GCA_002621785.1	Streptococcus phage P7631
1971434	GCA_002621805.1	Streptococcus phage P7632
1971435	GCA_002621825.1	Streptococcus phage P7633
1971436	GCA_002621845.1	Streptococcus phage P7951
1971437	GCA_002621865.1	Streptococcus phage P7952
1971438	GCA_002621885.1	Streptococcus phage P7953
1971439	GCA_002621905.1	Streptococcus phage P7954
1971440	GCA_002621925.1	Streptococcus phage P7955
1971441	GCA_002621945.1	Streptococcus phage P8921
1971442	GCA_002621965.1	Streptococcus phage P8922
403905	GCA_000871105.1	Streptococcus phage P9
1971443	GCA_002621985.1	Streptococcus phage P9851
1971444	GCA_002622005.1	Streptococcus phage P9852
1971445	GCA_002622025.1	Streptococcus phage P9853
1971446	GCA_002622045.1	Streptococcus phage P9854
1971447	GCA_002622065.1	Streptococcus phage P9901
1971448	GCA_002622085.1	Streptococcus phage P9902
1971449	GCA_002622105.1	Streptococcus phage P9903
644007	GCA_000886415.1	Streptococcus phage PH10
537874	GCA_000875325.1	Streptococcus phage PH15
1582354	GCA_002605365.1	Streptococcus phage phi1207.3
1289595	GCA_002603185.1	Streptococcus phage phi20c
1289596	GCA_002603205.1	Streptococcus phage phi30c
423476	GCA_000868025.1	Streptococcus phage phi3396
1289597	GCA_002603225.1	Streptococcus phage phi5218
1289598	GCA_002603245.1	Streptococcus phage phi7917
1289599	GCA_002603265.1	Streptococcus phage phi891591
1701811	GCA_001882175.1	Streptococcus phage phiARI0004

1701812	GCA_001882335.1	Streptococcus phage phiARI0031
1701813	GCA_001885385.1	Streptococcus phage phiARI0131-1
1701814	GCA_001884675.1	Streptococcus phage phiARI0131-2
1701823	GCA_003330285.1	Streptococcus phage phiARI0455b
1701824	GCA_001881755.1	Streptococcus phage phiARI0460-1
1701826	GCA_001881835.1	Streptococcus phage phiARI0462
1701827	GCA_001882055.1	Streptococcus phage phiARI0468-1
1701828	GCA_001881695.1	Streptococcus phage phiARI0468-2
1701830	GCA_001885405.1	Streptococcus phage phiARI0468-4
1701836	GCA_003330265.1	Streptococcus phage phiARI0639b
1701837	GCA_001884655.1	Streptococcus phage phiARI0746
1701839	GCA_003330185.1	Streptococcus phage phiARI0826b
1701841	GCA_003330345.1	Streptococcus phage phiARI0831b
1701842	GCA_001736395.1	Streptococcus phage phiARI0923
1701844	GCA_003330365.1	Streptococcus phage phiARI0995b
1267169	GCA_000911375.1	Streptococcus phage phiBHN167
1289600	GCA_002745635.1	Streptococcus phage phiD12
1289600	GCA_002745655.1	Streptococcus phage phiD12
1860179	GCA_002610705.1	Streptococcus phage phiJH1301-1
1860194	GCA_002610805.1	Streptococcus phage phiJH1301-2
1860190	GCA_002610725.1	Streptococcus phage phiJH1301-3
1860178	GCA_002610685.1	Streptococcus phage phiLP081102
571950	GCA_004284815.1	Streptococcus phage phi-m46.1
1239381	GCA_000901595.1	Streptococcus phage phiNJ2
1701259	GCA_002607325.1	Streptococcus phage phiNJ3
1289601	GCA_002603285.1	Streptococcus phage phiS10
1701260	GCA_002607345.1	Streptococcus phage phiSC070807
1277891	GCA_002603125.1	Streptococcus phage phiSS12
861448	GCA_002633645.1	Streptococcus phage phi-SsUD.1
1277892	GCA_002603145.1	Streptococcus phage phiST1
1860195	GCA_002610825.1	Streptococcus phage phiZJ20091101-1
1860191	GCA_002610745.1	Streptococcus phage phiZJ20091101-2
1860192	GCA_002610765.1	Streptococcus phage phiZJ20091101-3
1860193	GCA_002610785.1	Streptococcus phage phiZJ20091101-4
2041507	GCA_003012655.1	Streptococcus phage R1
157924	GCA_000843165.1	Streptococcus phage SM1
413066	GCA_000867905.1	Streptococcus phage SMP
1498213	GCA_002601525.1	Streptococcus phage SOCP
1897555	GCA_002613605.1	Streptococcus phage SpGS-1
1461246	GCA_002604445.1	Streptococcus phage Spn1
1208587	GCA_000910555.1	Streptococcus phage SPQS1
1566990	GCA_001041795.1	Streptococcus phage SpSL1
2041508	GCA_003012665.1	Streptococcus phage STP1
2041509	GCA_003012675.1	Streptococcus phage STP2
1933695	GCA_002618725.1	Streptococcus phage Str01
1933693	GCA_002618705.1	Streptococcus phage Str03
1589270	GCA_001470775.1	Streptococcus phage Str-PAP-1
2419628	GCA_003925045.1	Streptococcus phage SW1
2419629	GCA_003925185.1	Streptococcus phage SW10
2419630	GCA_003925205.1	Streptococcus phage SW11

2419631	GCA_003925525.1	Streptococcus phage SW1151
2419632	GCA_003925225.1	Streptococcus phage SW12
2419633	GCA_003925245.1	Streptococcus phage SW13
2419634	GCA_003925265.1	Streptococcus phage SW14
2419635	GCA_003925285.1	Streptococcus phage SW15
2419636	GCA_003925005.1	Streptococcus phage SW16
2419637	GCA_003925305.1	Streptococcus phage SW17
2419638	GCA_003925325.1	Streptococcus phage SW18
2419639	GCA_003925345.1	Streptococcus phage SW19
2419640	GCA_003925065.1	Streptococcus phage SW2
2419641	GCA_003925365.1	Streptococcus phage SW20
2419642	GCA_003925125.1	Streptococcus phage SW21
2419643	GCA_003925385.1	Streptococcus phage SW22
2419644	GCA_003925405.1	Streptococcus phage SW23
2483877	GCA_003925765.1	Streptococcus phage SW24
2419645	GCA_003925425.1	Streptococcus phage SW25
2419646	GCA_003925445.1	Streptococcus phage SW26
2419647	GCA_003925465.1	Streptococcus phage SW27
2419648	GCA_003925485.1	Streptococcus phage SW28
2419649	GCA_003925085.1	Streptococcus phage SW3
2419650	GCA_003925505.1	Streptococcus phage SW30
2419651	GCA_003925665.1	Streptococcus phage SW31
2419652	GCA_003925685.1	Streptococcus phage SW32
2419653	GCA_003925705.1	Streptococcus phage SW33
2419654	GCA_003925105.1	Streptococcus phage SW4
2483878	GCA_003925725.1	Streptococcus phage SW5
2419655	GCA_003925025.1	Streptococcus phage SW6
2483879	GCA_003925745.1	Streptococcus phage SW7
2419656	GCA_003925145.1	Streptococcus phage SW8
2419657	GCA_003925165.1	Streptococcus phage SW9
2419658	GCA_003925545.1	Streptococcus phage SWK1
2419659	GCA_003925565.1	Streptococcus phage SWK2
2419660	GCA_003925585.1	Streptococcus phage SWK3
2419661	GCA_003925605.1	Streptococcus phage SWK4
2419662	GCA_003925625.1	Streptococcus phage SWK5
2419663	GCA_003925645.1	Streptococcus phage SWK6
35344	GCA_001470495.1	Streptococcus phage T12
1385385	GCA_000911295.1	Streptococcus phage TP-778L
73422	GCA_000904075.1	Streptococcus phage TP-J34
2041510	GCA_003012685.1	Streptococcus phage V2
2069608	GCA_002957985.1	Streptococcus phage vB_SthS_VA214
2069609	GCA_002957995.1	Streptococcus phage vB_SthS_VA460
2184051	GCA_003814495.1	Streptococcus phage VS-2018a
1051631	GCA_001275515.1	Streptococcus phage YMC-2011
1028788	GCA_004319705.1	Streptococcus phi-m46.1-like phage MB56Spyo045
306323	GCA_000858545.1	Streptococcus virus 2972
112023	GCA_000839465.1	Streptococcus virus 7201
459845	GCA_000874965.1	Streptococcus virus 858
1814957	GCA_001745715.1	Streptococcus virus 9871
1814958	GCA_001744395.1	Streptococcus virus 9872

1814959	GCA_002609485.1	Streptococcus virus 9873
1814960	GCA_001743715.1	Streptococcus virus 9874
682370	GCA_000886115.1	Streptococcus virus ALQ132
230871	GCA_000842265.1	Streptococcus virus C1
90410	GCA_000857925.1	Streptococcus virus DT1
1962672	GCA_002619765.1	Streptococcus virus MS1
85154	GCA_000841145.1	Streptococcus virus O1205
633135	GCA_000885535.1	Streptococcus virus phiAbc2
78541	GCA_000836985.1	Streptococcus virus Sfi11
72638	GCA_000839445.1	Streptococcus virus Sfi19
64186	GCA_000837365.1	Streptococcus virus Sfi21
1690426	GCA_002756735.1	Streptomyces phage Aaronocolus
2059880	GCA_002957555.1	Streptomyces phage AbbeyMikolon
2041353	GCA_002744015.1	Streptomyces phage Abt2graduatex2
2053672	GCA_002957305.1	Streptomyces phage Alsaber
1673877	GCA_001501895.1	Streptomyces phage Amela
2041205	GCA_002743735.1	Streptomyces phage Amethyst
2250335	GCA_003354185.1	Streptomyces phage Annadreamy
2510513	GCA_004149525.1	Streptomyces phage Asten
2059881	GCA_002957565.1	Streptomyces phage Attoomi
2500795	GCA_004149105.1	Streptomyces phage Austintatious
1933692	GCA_002618685.1	Streptomyces phage BabyGotBac
2510587	GCA_004149245.1	Streptomyces phage BartholomewSD
2182342	GCA_003183705.1	Streptomyces phage BayC
2024286	GCA_002628525.1	Streptomyces phage BeardedLady
2079426	GCA_002958845.1	Streptomyces phage BillNye
2079427	GCA_002958855.1	Streptomyces phage Bing
1920307	GCA_002617085.1	Streptomyces phage Bioscum
2250336	GCA_003365655.1	Streptomyces phage Blueeyedbeauty
2502449	GCA_004149305.1	Streptomyces phage BoomerJR
2510514	GCA_004149605.1	Streptomyces phage Bovely
2510524	GCA_004149325.1	Streptomyces phage Bowden
1873994	GCA_002611825.1	Streptomyces phage Brataylor
1913591	GCA_002615505.1	Streptomyces phage BRock
2024287	GCA_002628545.1	Streptomyces phage BryanRecycles
2530160	GCA_004339925.1	Streptomyces phage Caelum
1690425	GCA_001502475.1	Streptomyces phage Caliburn
2024288	GCA_002628565.1	Streptomyces phage Celeste
1821728	GCA_002609665.1	Streptomyces phage Chymera
2301714	GCA_003442695.1	Streptomyces phage Comrade
2250337	GCA_003365675.1	Streptomyces phage Crosby
1690427	GCA_002603425.1	Streptomyces phage Danzina
2315716	GCA_003613215.1	Streptomyces phage Darolandstone
2024289	GCA_002628585.1	Streptomyces phage Dattran
2041206	GCA_002743755.1	Streptomyces phage Daudau
2041207	GCA_002743775.1	Streptomyces phage Diane
2024284	GCA_002628485.1	Streptomyces phage DrGrey
2182318	GCA_003183565.1	Streptomyces phage Eddasa
2517973	GCA_004520615.1	Streptomyces phage EGole
2024290	GCA_002628605.1	Streptomyces phage Esperer

2510569	GCA_004149185.1	Streptomyces phage Euratis
2182408	GCA_003183245.1	Streptomyces phage FlowerPower
2502445	GCA_004149285.1	Streptomyces phage Genie2
2500796	GCA_004149145.1	Streptomyces phage Gibson
2488789	GCA_004015365.1	Streptomyces phage Gilson
2182319	GCA_003183585.1	Streptomyces phage Goby
1873995	GCA_002611845.1	Streptomyces phage Godpower
2283302	GCA_003366895.1	Streptomyces phage Haizum
2301573	GCA_003442275.1	Streptomyces phage Hank144
2060086	GCA_002957595.1	Streptomyces phage HaugeAnator
2201346	GCA_003308415.1	Streptomyces phage Henoccus
2499072	GCA_004006885.1	Streptomyces phage Hiyaa
2182396	GCA_003183265.1	Streptomyces phage HotFries
1690428	GCA_002756755.1	Streptomyces phage Hydra
2182397	GCA_003183285.1	Streptomyces phage Ibantik
2510515	GCA_004149425.1	Streptomyces phage IceWarrior
1920308	GCA_002617105.1	Streptomyces phage Ididsumtinwong
2053813	GCA_002957455.1	Streptomyces phage Immanuel3
2510516	GCA_004149505.1	Streptomyces phage Indigo
1674926	GCA_001500455.1	Streptomyces phage Izzy
2201347	GCA_003308435.1	Streptomyces phage JackieB
2510525	GCA_004149385.1	Streptomyces phage Janus
2024291	GCA_002628625.1	Streptomyces phage Jash
1556290	GCA_001550725.1	Streptomyces phage Jay2Jay
1913034	GCA_002614665.1	Streptomyces phage Joe
2315618	GCA_003613775.1	Streptomyces phage JustBecause
2283303	GCA_003366855.1	Streptomyces phage Karimac
2315619	GCA_003613815.1	Streptomyces phage Kromp
1674927	GCA_001502495.1	Streptomyces phage Lannister
2201348	GCA_003308475.1	Streptomyces phage LazerLemon
2301574	GCA_003442315.1	Streptomyces phage LibertyBell
1327758	GCA_000908495.1	Streptomyces phage Lika
2510571	GCA_004149205.1	Streptomyces phage Lilbooboo
1873996	GCA_002611865.1	Streptomyces phage Lorelei
2283304	GCA_003366815.1	Streptomyces phage LukeCage
2510517	GCA_004149585.1	Streptomyces phage Madamato
1775283	GCA_002608945.1	Streptomyces phage Maih
2182320	GCA_003183545.1	Streptomyces phage Maneekul
2053812	GCA_002957445.1	Streptomyces phage Manuel
2315717	GCA_003614475.1	Streptomyces phage Microdon
2023959	GCA_002627405.1	Streptomyces phage Mildred21
1920309	GCA_002617125.1	Streptomyces phage Mojorita
2182399	GCA_003183325.1	Streptomyces phage Moozy
370623	GCA_000869005.1	Streptomyces phage mu1/6
2182321	GCA_003183525.1	Streptomyces phage Nabi
2510518	GCA_004149485.1	Streptomyces phage Namo
1873777	GCA_001745835.1	Streptomyces phage Nanodon
2510519	GCA_004149545.1	Streptomyces phage Nerdos
2108133	GCA_003014005.1	Streptomyces phage Nesbitt
2510526	GCA_004149345.1	Streptomyces phage Nishikigoi

2023992	GCA_002627445.1	Streptomyces phage NootNoot
2488981	GCA_003867865.1	Streptomyces phage Olicious
2024292	GCA_002628645.1	Streptomyces phage Oliynyk
1897524	GCA_002613085.1	Streptomyces phage OlympicHelado
2059882	GCA_002957575.1	Streptomyces phage Omar
2024293	GCA_002628665.1	Streptomyces phage Ozzie
2079428	GCA_002958865.1	Streptomyces phage OzzyJ
2108134	GCA_003014035.1	Streptomyces phage Paedore
1920310	GCA_002617145.1	Streptomyces phage PapayaSalad
2023993	GCA_002627465.1	Streptomyces phage Paradiddles
2023994	GCA_002627485.1	Streptomyces phage Peebs
2060087	GCA_002957605.1	Streptomyces phage Percastrophe
1239386	GCA_002602965.1	Streptomyces phage phiCAM
1211278	GCA_002601425.1	Streptomyces phage phiELB20
1204524	GCA_000898755.1	Streptomyces phage phiHau3
1755682	GCA_001500775.1	Streptomyces phage phiSAJS1
1755682	GCA_001500775.2	Streptomyces phage phiSAJS1
747763	GCA_000888395.1	Streptomyces phage phiSASD1
1920311	GCA_002617165.1	Streptomyces phage Picard
2510520	GCA_004149565.1	Streptomyces phage Popy
10732	GCA_000899575.1	Streptomyces phage R4
2182404	GCA_003183445.1	Streptomyces phage Rainydai
1920312	GCA_002617185.1	Streptomyces phage Raleigh
2182322	GCA_003183505.1	Streptomyces phage Rana
2182405	GCA_003183405.1	Streptomyces phage RavenPuff
1897525	GCA_002613105.1	Streptomyces phage Rima
1897525	GCA_002613105.2	Streptomyces phage Rima
2060088	GCA_002957615.1	Streptomyces phage Romero
2059883	GCA_002957585.1	Streptomyces phage Rowa
2182343	GCA_003183725.1	Streptomyces phage Salete
2023995	GCA_002627505.1	Streptomyces phage Samisti12
2283264	GCA_003366315.1	Streptomyces phage Satis
2041354	GCA_002744035.1	Streptomyces phage Scap1
2510572	GCA_004149225.1	Streptomyces phage Sebastisaurus
2182407	GCA_003183485.1	Streptomyces phage SendItCS
1690817	GCA_001505035.1	Streptomyces phage SF1
1690818	GCA_001503415.1	Streptomyces phage SF3
2510521	GCA_004149405.1	Streptomyces phage Shawty
2283305	GCA_003366675.1	Streptomyces phage SparkleGoddess
2024285	GCA_002628505.1	Streptomyces phage Spectropatronm
2041208	GCA_002743795.1	Streptomyces phage SqueakyClean
2283266	GCA_003366355.1	Streptomyces phage Starbow
2283265	GCA_003366375.1	Streptomyces phage StarPlatinum
1327759	GCA_000909095.1	Streptomyces phage Sujidade
2015806	GCA_002625405.1	Streptomyces phage Sushi23
1204525	GCA_000900215.1	Streptomyces phage SV1
2041209	GCA_002743815.1	Streptomyces phage Tefunt
2510588	GCA_004149265.1	Streptomyces phage Teutsch
2301715	GCA_003443055.1	Streptomyces phage Thestral
2510527	GCA_004149365.1	Streptomyces phage TinaBelcher

1965452	GCA_002620105.1	Streptomyces phage ToastyFinz
2182323	GCA_003183605.1	Streptomyces phage Toma
2060089	GCA_002957625.1	Streptomyces phage ToriToki
1636184	GCA_001502675.1	Streptomyces phage TP1604
2301575	GCA_003442515.1	Streptomyces phage TrvxScott
2201349	GCA_003308455.1	Streptomyces phage UNTPL
2510568	GCA_004149165.1	Streptomyces phage Vash
1673878	GCA_002606905.1	Streptomyces phage Verse
10702	GCA_000844125.1	Streptomyces phage VWB
2015805	GCA_002625385.1	Streptomyces phage Warpy
2126735	GCA_003023955.1	Streptomyces phage Wentworth
2500797	GCA_004149125.1	Streptomyces phage WheeHeim
2283267	GCA_003366435.1	Streptomyces phage Wofford
2053723	GCA_002957435.1	Streptomyces phage WRightOn
1784947	GCA_002609185.1	Streptomyces phage Xkcd426
2301621	GCA_003601375.1	Streptomyces phage Yaboi
2126736	GCA_003023945.1	Streptomyces phage Yara
1636183	GCA_001500635.1	Streptomyces phage YDN12
2201421	GCA_003308495.1	Streptomyces phage Yosif
1327760	GCA_000910055.1	Streptomyces phage Zemlya
2060090	GCA_002957635.1	Streptomyces phage ZooBear
225588	GCA_000843085.1	Streptomyces virus phiBT1
10719	GCA_000848045.2	Streptomyces virus phiC31
374422	GCA_000897775.1	Streptomyces virus TG1
35297	GCA_000849525.1	Striped jack nervous necrosis virus
1606500	GCA_002827865.1	Sturnira lilium polyomavirus 1
1792286	GCA_002609305.1	Stx converting phage vB_EcoS_P22
1792287	GCA_002609325.1	Stx converting phage vB_EcoS_P27
1792288	GCA_002609345.1	Stx converting phage vB_EcoS_P32
1792289	GCA_002609365.1	Stx converting phage vB_EcoS_ST2-8624
1855815	GCA_002594585.1	Stx1 converting phage AU5Stx1
1855816	GCA_002594605.1	Stx1 converting phage AU6Stx1
180816	GCA_002594625.1	Stx2 converting phage I
563769	GCA_000880675.1	Stx2-converting phage 1717
379329	GCA_000870145.1	Stx2-converting phage 86
1226259	GCA_002602805.1	Stx2-converting phage Stx2a_1447
1226253	GCA_002602705.1	Stx2-converting phage Stx2a_F349
1226252	GCA_002595065.1	Stx2-converting phage Stx2a_F403
1226254	GCA_002595085.1	Stx2-converting phage Stx2a_F422
1226255	GCA_002602725.1	Stx2-converting phage Stx2a_F451
1226256	GCA_002602745.1	Stx2-converting phage Stx2a_F723
1226257	GCA_002602765.1	Stx2-converting phage Stx2a_F765
1226260	GCA_002602825.1	Stx2-converting phage Stx2a_WGPS2
1226261	GCA_002602845.1	Stx2-converting phage Stx2a_WGPS4
1226262	GCA_002602865.1	Stx2-converting phage Stx2a_WGPS6
1226263	GCA_002602885.1	Stx2-converting phage Stx2a_WGPS8
1226258	GCA_002602785.1	Stx2-converting phage Stx2a_WGPS9
537009	GCA_000927175.1	Stygiolobus rod-shaped virus
646010	GCA_000900095.2	Suakwa aphid-borne yellows virus
12472	GCA_000862585.1	Subterranean clover mottle virus

195063	GCA_000846765.1	Subterranean clover mottle virus satellite RNA
1458458	GCA_002149165.1	Subterranean clover stunt C2 alphasatellite
1458459	GCA_002149265.1	Subterranean clover stunt C6 alphasatellite
36772	GCA_002994705.1	Subterranean clover stunt virus
1563660	GCA_001465085.1	Sucra jujuba nucleopolyhedrovirus
186540	GCA_000855585.1	Sudan ebolavirus
2018312	GCA_002270985.1	Sudan watermelon mosaic virus
1577137	GCA_001432035.1	Suffolk virus
1965225	GCA_004130415.1	Sugar beet cyst nematode virus 1
1960252	GCA_002819425.1	Sugarcane bacilliform Guadeloupe A virus
1960253	GCA_000886875.1	Sugarcane bacilliform Guadeloupe D virus
362398	GCA_000838945.1	Sugarcane bacilliform IM virus
362399	GCA_000868105.1	Sugarcane bacilliform MO virus
1919062	GCA_001891015.1	Sugarcane chlorotic streak virus
12224	GCA_000854025.1	Sugarcane mosaic virus
268785	GCA_000841765.1	Sugarcane streak Egypt virus - [Giza]
53954	GCA_000887195.1	Sugarcane streak mosaic virus
78395	GCA_000843205.1	Sugarcane streak Reunion virus
78395	GCA_002825425.1	Sugarcane streak Reunion virus
10836	GCA_002825445.1	Sugarcane streak virus
268781	GCA_000840065.1	Sugarcane streak virus - [Natal]
167927	GCA_000853085.1	Sugarcane striate mosaic-associated virus
1868659	GCA_002237255.1	Sugarcane striate virus
1868659	GCA_003029085.1	Sugarcane striate virus
1492296	GCA_000919135.1	Sugarcane white streak virus
94290	GCA_000861525.1	Sugarcane yellow leaf virus
10345	GCA_000843825.1	Suid alphaherpesvirus 1
10345	GCA_900230325.1	Suid alphaherpesvirus 1
1608255	GCA_000913455.1	Suid betaherpesvirus 2
490913	GCA_000881335.1	Sulfitobacter phage EE36phi1
1526550	GCA_001040815.1	Sulfitobacter phage NYA-2014a
754045	GCA_000904375.1	Sulfitobacter phage pCB2047-A
754043	GCA_000906855.1	Sulfitobacter phage pCB2047-C
754046	GCA_000905995.1	Sulfitobacter phage phiCB2047-B
2493122	GCA_003950195.1	Sulfolobales Beppu filamentous virus 1
2493123	GCA_003950175.1	Sulfolobales Beppu filamentous virus 2
2493124	GCA_003950155.1	Sulfolobales Beppu filamentous virus 3
2493121	GCA_003950135.1	Sulfolobales Beppu rod-shaped virus 1
2493126	GCA_003950115.1	Sulfolobales Beppu virus 1
1298531	GCA_000904595.1	Sulfolobales Mexican fusellovirus 1
1245909	GCA_000902255.1	Sulfolobales Mexican rudivirus 1
1732179	GCA_001502855.1	Sulfolobales virus YNP1
1732180	GCA_001502235.1	Sulfolobales Virus YNP2
2056194	GCA_002957505.1	Sulfolobus ellipsoid virus 1
2304198	GCA_003441495.1	Sulfolobus filamentous virus 1
176106	GCA_000838145.1	Sulfolobus islandicus filamentous virus
1902331	GCA_002608185.1	Sulfolobus islandicus filamentous virus 2
157898	GCA_000845065.1	Sulfolobus islandicus rod-shaped virus 1
1983545	GCA_002158755.1	Sulfolobus islandicus rod-shaped virus 10
1983546	GCA_002158615.1	Sulfolobus islandicus rod-shaped virus 11

157899	GCA_000857285.1	Sulfolobus islandicus rod-shaped virus 2
1983547	GCA_002158815.1	Sulfolobus islandicus rod-shaped virus 4
1983548	GCA_002158735.1	Sulfolobus islandicus rod-shaped virus 5
1983549	GCA_002158675.1	Sulfolobus islandicus rod-shaped virus 6
1983550	GCA_002158595.1	Sulfolobus islandicus rod-shaped virus 7
1983551	GCA_002158795.1	Sulfolobus islandicus rod-shaped virus 8
1983552	GCA_002158715.1	Sulfolobus islandicus rod-shaped virus 9
282066	GCA_002605325.1	Sulfolobus islandicus rudivirus 1 variant XX
1895333	GCA_001725895.1	Sulfolobus islandicus rudivirus 3
1351702	GCA_000917075.1	Sulfolobus monocaudavirus SMV1
1580591	GCA_001503855.1	Sulfolobus monocaudavirus SMV2
1732177	GCA_001551185.1	Sulfolobus monocaudavirus SMV3
1732178	GCA_001500815.1	Sulfolobus monocaudavirus SMV4
1982658	GCA_002623385.1	Sulfolobus polyhedral virus 1
2493125	GCA_003950075.1	Sulfolobus polyhedral virus 2
2491899	GCA_003868535.1	Sulfolobus spindle-shaped virus
2491899	GCA_003868555.1	Sulfolobus spindle-shaped virus
2491899	GCA_003868575.1	Sulfolobus spindle-shaped virus
2491899	GCA_003868595.1	Sulfolobus spindle-shaped virus
2491899	GCA_003868615.1	Sulfolobus spindle-shaped virus
2491899	GCA_003868635.1	Sulfolobus spindle-shaped virus
2491899	GCA_003868655.1	Sulfolobus spindle-shaped virus
2491899	GCA_003868695.1	Sulfolobus spindle-shaped virus
2491899	GCA_003868715.1	Sulfolobus spindle-shaped virus
2491899	GCA_003868735.1	Sulfolobus spindle-shaped virus
2491899	GCA_003868755.1	Sulfolobus spindle-shaped virus
2491899	GCA_003869515.1	Sulfolobus spindle-shaped virus
2491899	GCA_003869575.1	Sulfolobus spindle-shaped virus
2491899	GCA_003869595.1	Sulfolobus spindle-shaped virus
2491899	GCA_003869775.1	Sulfolobus spindle-shaped virus
2491899	GCA_003869795.1	Sulfolobus spindle-shaped virus
2491899	GCA_003869815.1	Sulfolobus spindle-shaped virus
2491899	GCA_003869875.1	Sulfolobus spindle-shaped virus
2491899	GCA_003869895.1	Sulfolobus spindle-shaped virus
2491899	GCA_003869915.1	Sulfolobus spindle-shaped virus
2491899	GCA_003869935.1	Sulfolobus spindle-shaped virus
2491899	GCA_003869955.1	Sulfolobus spindle-shaped virus
2491899	GCA_003869975.1	Sulfolobus spindle-shaped virus
2491899	GCA_003869995.1	Sulfolobus spindle-shaped virus
2491899	GCA_003870015.1	Sulfolobus spindle-shaped virus
2491899	GCA_003870035.1	Sulfolobus spindle-shaped virus
2491899	GCA_003870055.1	Sulfolobus spindle-shaped virus
2491899	GCA_003868475.1	Sulfolobus spindle-shaped virus
2491899	GCA_003868395.1	Sulfolobus spindle-shaped virus
2491899	GCA_003868415.1	Sulfolobus spindle-shaped virus
2491899	GCA_003868435.1	Sulfolobus spindle-shaped virus
2491899	GCA_003868455.1	Sulfolobus spindle-shaped virus
2491899	GCA_003868495.1	Sulfolobus spindle-shaped virus
2491899	GCA_003868515.1	Sulfolobus spindle-shaped virus
244589	GCA_000838445.1	Sulfolobus spindle-shaped virus 1

244590	GCA_000842405.1	Sulfolobus spindle-shaped virus 2
1959009	GCA_002619405.1	Sulfolobus spindle-shaped virus 3
459290	GCA_000872305.1	Sulfolobus spindle-shaped virus 4
459291	GCA_000880455.1	Sulfolobus spindle-shaped virus 5
693627	GCA_000884475.1	Sulfolobus spindle-shaped virus 6
693628	GCA_000886095.1	Sulfolobus spindle-shaped virus 7
1959008	GCA_002619385.1	Sulfolobus spindle-shaped virus Lassen
269145	GCA_000845405.1	Sulfolobus turreted icosahedral virus 1
754004	GCA_000889015.1	Sulfolobus turreted icosahedral virus 2
248496	GCA_000842585.1	Sulfolobus virus Kamchatka 1
256994	GCA_000843445.1	Sulfolobus virus Ragged Hills
285013	GCA_000846105.1	Sulfolobus virus STSV1
1123964	GCA_000903055.1	Sulfolobus virus STSV2
1604875	GCA_001430235.1	Sumatran orang-utan polyomavirus
129732	GCA_000886335.1	Sunflower chlorotic mottle virus
1250303	GCA_000901935.1	Sunflower leaf curl Karnataka alphasatellite
715480	GCA_000904735.1	Sunflower mild mosaic virus
1963260	GCA_002029655.1	Sunflower ring blotch virus
1491491	GCA_000925655.1	Sunguru virus
579113	GCA_000884115.1	Sunn hemp leaf distortion virus
1195087	GCA_000925355.1	Sunshine Coast virus
446138	GCA_000883075.1	Sus scrofa papillomavirus 1
2025338	GCA_002270625.1	Sus scrofa papillomavirus 2
1680894	GCA_004129235.1	Sus scrofa polyomavirus 1
1929030	GCA_002149865.1	Swagivirus HG
459957	GCA_000925255.1	Swan circovirus
459957	GCA_004091875.1	Swan circovirus
28348	GCA_000852225.1	Sweet clover necrotic mosaic virus
647294	GCA_000884855.1	Sweet potato badnavirus B
1307958	GCA_000898495.1	Sweet potato C6 virus
263004	GCA_000854905.1	Sweet potato chlorotic fleck virus
81931	GCA_000853225.1	Sweet potato chlorotic stunt virus
930168	GCA_000892575.1	Sweet potato collusive virus
12844	GCA_000863185.1	Sweet potato feathery mottle virus
688540	GCA_000892555.1	Sweet potato golden vein associated virus
2169736	GCA_003029275.1	Sweet potato golden vein Korea virus
31743	GCA_000905635.1	Sweet potato latent virus
693893	GCA_000886995.1	Sweet potato leaf curl Bengal virus - [India:West Bengal:20
652718	GCA_000884435.1	Sweet potato leaf curl Canary virus
652718	GCA_001698315.1	Sweet potato leaf curl Canary virus
1198219	GCA_002823565.1	Sweet potato leaf curl China virus
1993647	GCA_000894135.1	Sweet potato leaf curl deltasatellite 1
1993648	GCA_000925215.2	Sweet potato leaf curl deltasatellite 2
223330	GCA_000842125.1	Sweet potato leaf curl Georgia virus-[16]
1489312	GCA_000921495.1	Sweet potato leaf curl Guangxi virus
1489311	GCA_000907875.1	Sweet potato leaf curl Henan virus
1489311	GCA_000920735.1	Sweet potato leaf curl Henan virus
574890	GCA_000886055.1	Sweet potato leaf curl Lanzarote virus
994388	GCA_000927695.1	Sweet potato leaf curl Sao Paulo virus
1392388	GCA_000913435.1	Sweet potato leaf curl Shanghai virus

2026196	GCA_002823585.1	Sweet potato leaf curl Sichuan virus 1
2026195	GCA_000912175.1	Sweet potato leaf curl Sichuan virus 2
999288	GCA_000893435.1	Sweet potato leaf curl South Carolina virus
652717	GCA_000875485.1	Sweet potato leaf curl Spain virus
940846	GCA_000893075.1	Sweet potato leaf curl Uganda virus-[Uganda:Kampala:200
100755	GCA_000864705.1	Sweet potato leaf curl virus
100755	GCA_002823685.1	Sweet potato leaf curl virus
100755	GCA_002823705.1	Sweet potato leaf curl virus
100755	GCA_002823605.1	Sweet potato leaf curl virus
100755	GCA_002823625.1	Sweet potato leaf curl virus
100755	GCA_002823645.1	Sweet potato leaf curl virus
100755	GCA_002823665.1	Sweet potato leaf curl virus
100755	GCA_002823725.1	Sweet potato leaf curl virus
100755	GCA_002823745.1	Sweet potato leaf curl virus
100755	GCA_002823765.1	Sweet potato leaf curl virus
100755	GCA_002823785.1	Sweet potato leaf curl virus
100755	GCA_002986895.1	Sweet potato leaf curl virus
100755	GCA_002986885.1	Sweet potato leaf curl virus
652719	GCA_002823805.1	Sweet potato leaf curl virus-Spain
41459	GCA_000860665.1	Sweet potato mild mottle virus
1513528	GCA_001967215.1	Sweet potato mosaic virus
1513529	GCA_002823825.1	Sweet potato mosaic virus - [Brazil:Brasilia1:2007]
2034762	GCA_000893715.1	Sweet potato pakakuy virus
603333	GCA_002158695.1	Sweet potato symptomless virus 1
603333	GCA_003029565.1	Sweet potato symptomless virus 1
995049	GCA_000891675.1	Sweet potato vein clearing virus
453050	GCA_000896295.1	Sweet potato virus 2
930881	GCA_000888795.1	Sweet potato virus C
46619	GCA_000898935.1	Sweet potato virus G
1272958	GCA_002145685.1	Sweetwater Branch virus
1766554	GCA_001501415.1	Swine enteric coronavirus
1766554	GCA_900078225.1	Swine enteric coronavirus
63421	GCA_002826265.1	Swine hepatitis E virus
10276	GCA_000839965.1	Swinepox virus
1027869	GCA_000893635.1	Switchgrass mosaic virus
1571533	GCA_000928935.1	Switchgrass mosaic-associated virus 1
2175118	GCA_004132145.1	Symphysodon discus adomavirus 1
1493507	GCA_002595605.1	Synechococcus phage ACG-2014a
1493507	GCA_002595625.1	Synechococcus phage ACG-2014a
1493507	GCA_002595645.1	Synechococcus phage ACG-2014a
1493507	GCA_002595665.1	Synechococcus phage ACG-2014a
1493507	GCA_002595685.1	Synechococcus phage ACG-2014a
1493507	GCA_002595705.1	Synechococcus phage ACG-2014a
1493507	GCA_002595725.1	Synechococcus phage ACG-2014a
1493507	GCA_002595745.1	Synechococcus phage ACG-2014a
1493507	GCA_002595765.1	Synechococcus phage ACG-2014a
1493507	GCA_002595785.1	Synechococcus phage ACG-2014a
1493507	GCA_002595805.1	Synechococcus phage ACG-2014a
1493507	GCA_002595825.1	Synechococcus phage ACG-2014a
1493507	GCA_002595845.1	Synechococcus phage ACG-2014a

1493507	GCA_002595865.1	Synechococcus phage ACG-2014a
1493507	GCA_002595885.1	Synechococcus phage ACG-2014a
1493507	GCA_002595905.1	Synechococcus phage ACG-2014a
1493507	GCA_002603765.1	Synechococcus phage ACG-2014a
1493507	GCA_002595925.1	Synechococcus phage ACG-2014a
1493507	GCA_002595945.1	Synechococcus phage ACG-2014a
1493507	GCA_002595965.1	Synechococcus phage ACG-2014a
1493507	GCA_002595985.1	Synechococcus phage ACG-2014a
1493507	GCA_002596005.1	Synechococcus phage ACG-2014a
1493507	GCA_002596025.1	Synechococcus phage ACG-2014a
1493507	GCA_002596045.1	Synechococcus phage ACG-2014a
1493508	GCA_001019915.1	Synechococcus phage ACG-2014b
1493508	GCA_002596925.1	Synechococcus phage ACG-2014b
1493508	GCA_002596945.1	Synechococcus phage ACG-2014b
1493508	GCA_002596965.1	Synechococcus phage ACG-2014b
1493508	GCA_002596985.1	Synechococcus phage ACG-2014b
1493508	GCA_002597005.1	Synechococcus phage ACG-2014b
1493508	GCA_002597025.1	Synechococcus phage ACG-2014b
1493508	GCA_002597045.1	Synechococcus phage ACG-2014b
1493508	GCA_002597065.1	Synechococcus phage ACG-2014b
1493508	GCA_002597085.1	Synechococcus phage ACG-2014b
1493508	GCA_002597105.1	Synechococcus phage ACG-2014b
1493508	GCA_002597125.1	Synechococcus phage ACG-2014b
1493508	GCA_002597145.1	Synechococcus phage ACG-2014b
1493508	GCA_002597165.1	Synechococcus phage ACG-2014b
1493508	GCA_002597185.1	Synechococcus phage ACG-2014b
1493508	GCA_002596865.1	Synechococcus phage ACG-2014b
1493508	GCA_002596885.1	Synechococcus phage ACG-2014b
1493508	GCA_002596905.1	Synechococcus phage ACG-2014b
1079998	GCA_000902315.1	Synechococcus phage ACG-2014c
1079998	GCA_002921795.1	Synechococcus phage ACG-2014c
1079998	GCA_002921765.1	Synechococcus phage ACG-2014c
1079998	GCA_002921745.1	Synechococcus phage ACG-2014c
1079998	GCA_002921775.1	Synechococcus phage ACG-2014c
1493509	GCA_000982365.1	Synechococcus phage ACG-2014d
1493509	GCA_002548725.1	Synechococcus phage ACG-2014d
1493509	GCA_002548365.1	Synechococcus phage ACG-2014d
1493509	GCA_002549035.1	Synechococcus phage ACG-2014d
1493509	GCA_002549445.1	Synechococcus phage ACG-2014d
1493509	GCA_002548855.1	Synechococcus phage ACG-2014d
1493509	GCA_002549405.1	Synechococcus phage ACG-2014d
1493509	GCA_002548405.1	Synechococcus phage ACG-2014d
1493509	GCA_002548215.1	Synechococcus phage ACG-2014d
1493509	GCA_002534635.1	Synechococcus phage ACG-2014d
1493509	GCA_002548235.1	Synechococcus phage ACG-2014d
1493509	GCA_002548275.1	Synechococcus phage ACG-2014d
1493509	GCA_002534655.1	Synechococcus phage ACG-2014d
1493509	GCA_002548435.1	Synechococcus phage ACG-2014d
1493509	GCA_002548815.1	Synechococcus phage ACG-2014d
1493509	GCA_002548475.1	Synechococcus phage ACG-2014d

1493509	GCA_002548135.1	Synechococcus phage ACG-2014d
1493509	GCA_002549215.1	Synechococcus phage ACG-2014d
1493509	GCA_002548555.1	Synechococcus phage ACG-2014d
1493509	GCA_002548575.1	Synechococcus phage ACG-2014d
1493509	GCA_002549535.1	Synechococcus phage ACG-2014d
1493509	GCA_002548155.1	Synechococcus phage ACG-2014d
1493509	GCA_002548655.1	Synechococcus phage ACG-2014d
1493509	GCA_002549515.1	Synechococcus phage ACG-2014d
1493509	GCA_002548515.1	Synechococcus phage ACG-2014d
1493509	GCA_002548185.1	Synechococcus phage ACG-2014d
1493509	GCA_002548785.1	Synechococcus phage ACG-2014d
1493509	GCA_002548835.1	Synechococcus phage ACG-2014d
1493509	GCA_002548875.1	Synechococcus phage ACG-2014d
1493509	GCA_002548905.1	Synechococcus phage ACG-2014d
1493509	GCA_002549095.1	Synechococcus phage ACG-2014d
1493509	GCA_002549015.1	Synechococcus phage ACG-2014d
1493509	GCA_002549055.1	Synechococcus phage ACG-2014d
1493509	GCA_002549075.1	Synechococcus phage ACG-2014d
1493509	GCA_002548965.1	Synechococcus phage ACG-2014d
1493509	GCA_002549235.1	Synechococcus phage ACG-2014d
1493509	GCA_002548615.1	Synechococcus phage ACG-2014d
1493509	GCA_002549375.1	Synechococcus phage ACG-2014d
1493509	GCA_002549335.1	Synechococcus phage ACG-2014d
1493509	GCA_002549465.1	Synechococcus phage ACG-2014d
1493509	GCA_002549255.1	Synechococcus phage ACG-2014d
1493509	GCA_002549155.1	Synechococcus phage ACG-2014d
1493509	GCA_002548325.1	Synechococcus phage ACG-2014d
1493509	GCA_002548765.1	Synechococcus phage ACG-2014d
1493509	GCA_002548685.1	Synechococcus phage ACG-2014d
1493509	GCA_002549295.1	Synechococcus phage ACG-2014d
1493510	GCA_000982325.1	Synechococcus phage ACG-2014e
1493510	GCA_002921755.1	Synechococcus phage ACG-2014e
1493510	GCA_002921785.1	Synechococcus phage ACG-2014e
1493511	GCA_000982385.1	Synechococcus phage ACG-2014f
1493511	GCA_002593785.1	Synechococcus phage ACG-2014f
1493511	GCA_002593805.1	Synechococcus phage ACG-2014f
1493511	GCA_002593825.1	Synechococcus phage ACG-2014f
1493511	GCA_002593845.1	Synechococcus phage ACG-2014f
1493511	GCA_002593865.1	Synechococcus phage ACG-2014f
1493511	GCA_002593885.1	Synechococcus phage ACG-2014f
1493511	GCA_002593905.1	Synechococcus phage ACG-2014f
1493511	GCA_002593925.1	Synechococcus phage ACG-2014f
1493511	GCA_002593945.1	Synechococcus phage ACG-2014f
1493511	GCA_002593965.1	Synechococcus phage ACG-2014f
1493511	GCA_002593985.1	Synechococcus phage ACG-2014f
1493511	GCA_002594005.1	Synechococcus phage ACG-2014f
1493511	GCA_002594025.1	Synechococcus phage ACG-2014f
1493511	GCA_002594045.1	Synechococcus phage ACG-2014f
1493511	GCA_002594065.1	Synechococcus phage ACG-2014f
1493511	GCA_002594085.1	Synechococcus phage ACG-2014f

1493511	GCA_002594105.1	Synechococcus phage ACG-2014f
1493511	GCA_002594125.1	Synechococcus phage ACG-2014f
1493511	GCA_002594145.1	Synechococcus phage ACG-2014f
1493511	GCA_002594165.1	Synechococcus phage ACG-2014f
1493511	GCA_002594185.1	Synechococcus phage ACG-2014f
1493511	GCA_002594205.1	Synechococcus phage ACG-2014f
1493511	GCA_002594225.1	Synechococcus phage ACG-2014f
1493511	GCA_002594245.1	Synechococcus phage ACG-2014f
1493511	GCA_002594265.1	Synechococcus phage ACG-2014f
1493511	GCA_002594285.1	Synechococcus phage ACG-2014f
1493511	GCA_002594305.1	Synechococcus phage ACG-2014f
1493511	GCA_002594325.1	Synechococcus phage ACG-2014f
1493511	GCA_002594345.1	Synechococcus phage ACG-2014f
1493511	GCA_002594365.1	Synechococcus phage ACG-2014f
1493511	GCA_002594385.1	Synechococcus phage ACG-2014f
1493511	GCA_002594405.1	Synechococcus phage ACG-2014f
1493511	GCA_002594425.1	Synechococcus phage ACG-2014f
1493511	GCA_002594445.1	Synechococcus phage ACG-2014f
1493511	GCA_002594465.1	Synechococcus phage ACG-2014f
1493511	GCA_002594485.1	Synechococcus phage ACG-2014f
1493511	GCA_002594505.1	Synechococcus phage ACG-2014f
1493511	GCA_002594525.1	Synechococcus phage ACG-2014f
1493511	GCA_002594545.1	Synechococcus phage ACG-2014f
1493511	GCA_002594565.1	Synechococcus phage ACG-2014f
1493512	GCA_000982345.1	Synechococcus phage ACG-2014g
1340810	GCA_000918375.1	Synechococcus phage ACG-2014h
1493513	GCA_001019995.1	Synechococcus phage ACG-2014i
1493514	GCA_000982305.1	Synechococcus phage ACG-2014j
1493514	GCA_002604645.1	Synechococcus phage ACG-2014j
2023996	GCA_002627525.1	Synechococcus phage Bellamy
889950	GCA_002598405.1	Synechococcus phage KBS-M-1A
1079999	GCA_000901695.1	Synechococcus phage metaG-MbCM1
2484637	GCA_003723235.1	Synechococcus phage S-B05
2545435	GCA_004521515.1	Synechococcus phage S-B28
2484638	GCA_003723215.1	Synechococcus phage S-B43
2163901	GCA_003093875.1	Synechococcus phage S-B64
2545437	GCA_004521555.1	Synechococcus phage S-B68
754037	GCA_000906915.1	Synechococcus phage S-CAM1
754037	GCA_002922075.1	Synechococcus phage S-CAM1
754037	GCA_002922085.1	Synechococcus phage S-CAM1
754037	GCA_002922095.1	Synechococcus phage S-CAM1
754037	GCA_002922115.1	Synechococcus phage S-CAM1
754037	GCA_002922105.1	Synechococcus phage S-CAM1
1883365	GCA_001881915.1	Synechococcus phage S-CAM22
1883365	GCA_002922185.1	Synechococcus phage S-CAM22
1883365	GCA_002922195.1	Synechococcus phage S-CAM22
1883366	GCA_001882155.1	Synechococcus phage S-CAM3
1883366	GCA_002922135.1	Synechococcus phage S-CAM3
1883366	GCA_002922125.1	Synechococcus phage S-CAM3
1883367	GCA_002366065.1	Synechococcus phage S-CAM4

1883367	GCA_002922145.1	Synechococcus phage S-CAM4
1883367	GCA_002922155.1	Synechococcus phage S-CAM4
1883368	GCA_001882035.1	Synechococcus phage S-CAM7
1883368	GCA_002612085.1	Synechococcus phage S-CAM7
754038	GCA_000907715.1	Synechococcus phage S-CAM8
754038	GCA_002630745.1	Synechococcus phage S-CAM8
1883369	GCA_001881955.1	Synechococcus phage S-CAM9
1883369	GCA_002922165.1	Synechococcus phage S-CAM9
1883369	GCA_002922175.1	Synechococcus phage S-CAM9
1273711	GCA_000929515.1	Synechococcus phage S-CBP1
756277	GCA_000925075.1	Synechococcus phage S-CBP2
756276	GCA_000925935.1	Synechococcus phage S-CBP3
754059	GCA_000929555.1	Synechococcus phage S-CBP4
461711	GCA_001503235.1	Synechococcus phage S-CBP42
909297	GCA_000894995.1	Synechococcus phage S-CBS1
753084	GCA_000891995.1	Synechococcus phage S-CBS2
753085	GCA_000891035.1	Synechococcus phage S-CBS3
756275	GCA_000896775.1	Synechococcus phage S-CBS4
2053653	GCA_003861695.1	Synechococcus phage S-CBWM1
1026955	GCA_000892775.1	Synechococcus phage S-CRM01
2484639	GCA_003723255.1	Synechococcus phage S-E7
1468179	GCA_002604485.1	Synechococcus phage S-EIVl
2488632	GCA_003862135.1	Synechococcus phage S-H1
1983572	GCA_002623685.1	Synechococcus phage S-H35
754039	GCA_000908735.1	Synechococcus phage S-IOM18
2008320	GCA_002955335.1	Synechococcus phage S-LBS1
1340812	GCA_000917315.1	Synechococcus phage S-MbCM100
1340811	GCA_002755835.1	Synechococcus phage S-MbCM25
2484640	GCA_003723455.1	Synechococcus phage S-P4
238854	GCA_000857445.1	Synechococcus phage S-PM2
238854	GCA_003147225.1	Synechococcus phage S-PM2
687800	GCA_002547705.1	Synechococcus phage S-RIM2
687800	GCA_002546705.1	Synechococcus phage S-RIM2
687800	GCA_002547745.1	Synechococcus phage S-RIM2
687800	GCA_002546415.1	Synechococcus phage S-RIM2
687800	GCA_002547775.1	Synechococcus phage S-RIM2
687800	GCA_002547875.1	Synechococcus phage S-RIM2
687800	GCA_002547045.1	Synechococcus phage S-RIM2
687800	GCA_002548055.1	Synechococcus phage S-RIM2
687800	GCA_002546735.1	Synechococcus phage S-RIM2
687800	GCA_002547995.1	Synechococcus phage S-RIM2
687800	GCA_002548035.1	Synechococcus phage S-RIM2
687800	GCA_002546055.1	Synechococcus phage S-RIM2
687800	GCA_002546075.1	Synechococcus phage S-RIM2
687800	GCA_002546445.1	Synechococcus phage S-RIM2
687800	GCA_002546105.1	Synechococcus phage S-RIM2
687800	GCA_002546145.1	Synechococcus phage S-RIM2
687800	GCA_002548105.1	Synechococcus phage S-RIM2
687800	GCA_002547435.1	Synechococcus phage S-RIM2
687800	GCA_002546775.1	Synechococcus phage S-RIM2

687800	GCA_002547255.1	Synechococcus phage S-RIM2
687800	GCA_002547125.1	Synechococcus phage S-RIM2
687800	GCA_002546825.1	Synechococcus phage S-RIM2
687800	GCA_002546175.1	Synechococcus phage S-RIM2
687800	GCA_002546485.1	Synechococcus phage S-RIM2
687800	GCA_002547795.1	Synechococcus phage S-RIM2
687800	GCA_002547465.1	Synechococcus phage S-RIM2
687800	GCA_002547175.1	Synechococcus phage S-RIM2
687800	GCA_002546205.1	Synechococcus phage S-RIM2
687800	GCA_002546255.1	Synechococcus phage S-RIM2
687800	GCA_002546645.1	Synechococcus phage S-RIM2
687800	GCA_002546885.1	Synechococcus phage S-RIM2
687800	GCA_002547815.1	Synechococcus phage S-RIM2
687800	GCA_002547515.1	Synechococcus phage S-RIM2
687800	GCA_002546675.1	Synechococcus phage S-RIM2
687800	GCA_002546755.1	Synechococcus phage S-RIM2
687800	GCA_002547225.1	Synechococcus phage S-RIM2
687800	GCA_002546555.1	Synechococcus phage S-RIM2
687800	GCA_002547895.1	Synechococcus phage S-RIM2
687800	GCA_002546595.1	Synechococcus phage S-RIM2
687800	GCA_002547405.1	Synechococcus phage S-RIM2
687800	GCA_002546285.1	Synechococcus phage S-RIM2
687800	GCA_002547575.1	Synechococcus phage S-RIM2
687800	GCA_002547095.1	Synechococcus phage S-RIM2
687800	GCA_002547275.1	Synechococcus phage S-RIM2
687800	GCA_002547955.1	Synechococcus phage S-RIM2
687800	GCA_002546525.1	Synechococcus phage S-RIM2
687800	GCA_002546915.1	Synechococcus phage S-RIM2
687800	GCA_002546945.1	Synechococcus phage S-RIM2
687800	GCA_002547595.1	Synechococcus phage S-RIM2
687800	GCA_002546985.1	Synechococcus phage S-RIM2
687800	GCA_002546315.1	Synechococcus phage S-RIM2
687800	GCA_002547625.1	Synechococcus phage S-RIM2
687800	GCA_002546355.1	Synechococcus phage S-RIM2
687800	GCA_002547015.1	Synechococcus phage S-RIM2
687800	GCA_002547975.1	Synechococcus phage S-RIM2
687800	GCA_002547325.1	Synechococcus phage S-RIM2
687800	GCA_002547385.1	Synechococcus phage S-RIM2
687800	GCA_002547655.1	Synechococcus phage S-RIM2
687800	GCA_002745335.1	Synechococcus phage S-RIM2
869662	GCA_000906935.1	Synechococcus phage S-RIM2 R1_1999
869661	GCA_002412125.1	Synechococcus phage S-RIM2 R21_2007
869663	GCA_002723435.1	Synechococcus phage S-RIM2 R9_2006
756278	GCA_002598245.1	Synechococcus phage S-RIM8
756278	GCA_002598265.1	Synechococcus phage S-RIM8
756278	GCA_002598285.1	Synechococcus phage S-RIM8
756278	GCA_002598305.1	Synechococcus phage S-RIM8
756278	GCA_002598325.1	Synechococcus phage S-RIM8
756278	GCA_002598345.1	Synechococcus phage S-RIM8
869724	GCA_000904235.1	Synechococcus phage S-RIM8 A.HR1

869725	GCA_002598385.1	Synechococcus phage S-RIM8 A.HR3
869726	GCA_002598365.1	Synechococcus phage S-RIM8 A.HR5
754041	GCA_000905455.1	Synechococcus phage S-RIP1
754040	GCA_000906055.1	Synechococcus phage S-RIP2
555387	GCA_000886695.1	Synechococcus phage S-RSM4
445683	GCA_000891795.1	Synechococcus phage S-ShM2
754042	GCA_000904455.1	Synechococcus phage S-SKS1
444859	GCA_000893355.1	Synechococcus phage S-SM1
444860	GCA_000890815.1	Synechococcus phage S-SM2
536466	GCA_000905555.1	Synechococcus phage S-SSM4
445685	GCA_000891835.1	Synechococcus phage S-SSM5
445686	GCA_000890855.1	Synechococcus phage S-SSM7
2268578	GCA_003443335.1	Synechococcus phage S-T4
1815521	GCA_001885345.1	Synechococcus phage S-WAM1
1815522	GCA_001882115.1	Synechococcus phage S-WAM2
445684	GCA_000893375.1	Synechococcus phage Syn19
382359	GCA_000870005.1	Synechococcus phage syn9
1332145	GCA_003369305.1	Synechococcus T7-like virus S-TIP37
151528	GCA_000849825.2	Synechococcus virus P60
1964565	GCA_002619805.1	Synechococcus virus S-ESS1
2100130	GCA_003428315.1	Synechococcus virus S-PRM1
438482	GCA_000873225.1	Synechococcus virus Syn5
1544378	GCA_000925235.1	Synedrella leaf curl alphasatellite
1899219	GCA_001755365.1	Synedrella leaf curl virus
1914900	GCA_003029435.1	Synedrella yellow vein clearing virus
11631	GCA_000853285.1	Tacaribe mammarenavirus
1608083	GCA_001755105.1	Tacheng Tick Virus 1
1608085	GCA_001755525.1	Tacheng Tick Virus 3
1608086	GCA_001441015.1	Tacheng Tick Virus 4
1608087	GCA_001441095.1	Tacheng Tick Virus 5
1608088	GCA_001754665.1	Tacheng Tick Virus 6
1608089	GCA_001754225.1	Tacheng Tick Virus 7
1746065	GCA_001443925.1	Tacheng tick virus 8
1732201	GCA_001316415.1	Tadarida brasiliensis circovirus 1
1588048	GCA_000929075.1	Tadarida brasiliensis polyomavirus 1
1588049	GCA_000928175.1	Tadarida brasiliensis polyomavirus 2
1930825	GCA_001939235.1	Tai Forest alphavirus
186541	GCA_000888475.1	Tai Forest ebolavirus
1406343	GCA_002118925.1	Tai virus
1117633	GCA_000927115.1	Tailam virus
1608090	GCA_001755085.1	Taishun Tick Virus
1414644	GCA_000911235.1	Tall oatgrass mosaic virus
1338506	GCA_002826885.1	Talpa europaea papillomavirus 1
1338506	GCA_003033145.1	Talpa europaea papillomavirus 1
161675	GCA_000861685.1	Tamana bat virus
945960	GCA_000955115.1	Tamarillo leaf malformation virus
45223	GCA_000879315.1	Tamiami mammarenavirus
1081702	GCA_000891355.1	Tamus red mosaic virus
1489714	GCA_000920695.1	Tanay virus
1489714	GCA_003726595.1	Tanay virus

1489714	GCA_003726555.1	Tanay virus
1489714	GCA_003726575.1	Tanay virus
1489714	GCA_003729835.1	Tanay virus
1926501	GCA_002008735.1	Tapara virus
1634914	GCA_000973315.1	Taro bacilliform CH virus
178354	GCA_000840385.1	Taro bacilliform virus
303300	GCA_000858885.1	Taro vein chlorosis nucleorhabdovirus
1721090	GCA_001433565.1	Tasmanian aquabirnavirus
28871	GCA_000869985.1	Taterapox virus
142102	GCA_000849385.1	Taura syndrome virus
1672384	GCA_003032605.1	Tavallinen suomalainen mies virus
2419940	GCA_004117535.1	Tea plant line pattern virus
2419939	GCA_004117515.1	Tea plant necrotic ring blotch virus
2169737	GCA_001678335.2	Telfairia golden mosaic virus
321302	GCA_002986275.1	Tellina virus 1
400394	GCA_000873465.1	Telosma mosaic virus
64293	GCA_000894755.1	Tembusu virus
1628899	GCA_001190495.1	Temperate fruit decay-associated virus
173707	GCA_000838205.2	Temperate phage phiNIH1.1
1880590	GCA_002611925.1	Tenacibaculum phage pT24
1288358	GCA_000925415.1	Tench rhabdovirus
1508712	GCA_000923755.1	Tent-making bat hepatitis B virus
1508712	GCA_002826205.1	Tent-making bat hepatitis B virus
2293307	GCA_003846785.1	Tentweb spider associated circular virus 1
2108549	GCA_003848425.1	Termite associated circular virus 1
2108550	GCA_003848545.1	Termite associated circular virus 2
2108550	GCA_003847405.1	Termite associated circular virus 2
2108551	GCA_003848405.1	Termite associated circular virus 3
2108552	GCA_003848525.1	Termite associated circular virus 4
2486188	GCA_004141815.1	Termite gut associated microvirus 1
2486189	GCA_004141845.1	Termite gut associated microvirus 2
1561226	GCA_001308455.2	Testudinid herpesvirus 3
1561226	GCA_003024885.1	Testudinid herpesvirus 3
35319	GCA_002814395.1	Tete orthobunyavirus
1908804	GCA_002374895.1	Tetraparvovirus sp.
756284	GCA_003225995.1	Tetraselmis viridis virus N1
756285	GCA_000906975.1	Tetraselmis viridis virus S1
754070	GCA_000906075.1	Tetraselmis viridis virus S20
754071	GCA_000905475.1	Tetraselmis viridis virus SI1
2060617	GCA_003057795.1	Tetraselmis virus 1
981335	GCA_000902975.1	Tetrasphaera phage TJE1
1712389	GCA_002867385.1	Tetterwort vein chlorosis virus
1554501	GCA_001431975.1	Teviot virus
1554501	GCA_002815315.1	Teviot virus
469660	GCA_000873765.1	Thalassomonas phage BA3
1591444	GCA_000930295.1	Thaumetopoea pityocampa iflavirus 1
1307119	GCA_002821325.1	Theiler's disease-associated virus
12127	GCA_000861185.1	Theiler's murine encephalomyeltits virus GDVII
1770613	GCA_001500735.1	Thelephora terrestris virus 1
1126885	GCA_001275535.1	Thermoanaerobacterium phage THSA-485A

1661715	GCA_002606785.1	Thermobifida phage P1312
2500138	GCA_004015415.1	Thermobifida phage P318
1115696	GCA_000896815.1	Thermococcus prieurii virus 1
1115696	GCA_002989825.1	Thermococcus prieurii virus 1
292639	GCA_000846885.1	Thermoproteus tenax spherical virus 1
1406341	GCA_002604085.1	Thermus phage G20c
1352230	GCA_000909595.1	Thermus phage phi OH2
1400796	GCA_003991745.1	Thermus phage phiFa
2301535	GCA_003991725.1	Thermus phage phiKo
2301536	GCA_003991705.1	Thermus phage phiLo
2301537	GCA_003991765.1	Thermus phage phiMa
1968340	GCA_002621045.1	Thermus phage phiOH16
1968339	GCA_002621025.1	Thermus phage phiOH3
407392	GCA_000869585.1	Thermus phage phiYS40
407392	GCA_002630225.1	Thermus phage phiYS40
699370	GCA_000891315.1	Thermus phage TMA
1298530	GCA_004146425.1	Thermus phage TSP4
2494333	GCA_004015445.1	Thermus phage YS40_Isch
1714273	GCA_000842885.1	Thermus virus IN93
466051	GCA_000871085.1	Thermus virus P23-45
1714272	GCA_000884275.1	Thermus virus P23-77
466052	GCA_000871945.1	Thermus virus P74-26
197772	GCA_000841825.1	Thetapapillomavirus 1
1980529	GCA_002831145.1	Thiafora orthonairovirus
274584	GCA_000883775.1	Thielaviopsis basicola mitovirus
1654356	GCA_001020055.1	Thika virus
1352511	GCA_000910355.1	Thin paspalum asymptomatic virus
1698523	GCA_002607265.1	Thiobacimonas phage vB_ThpS-P1
577122	GCA_002117755.1	T'Ho virus
11569	GCA_000854245.1	Thogoto thogotovirus
1980493	GCA_000879955.1	Thottopalayam virus
1941233	GCA_002004355.1	Thrips-associated genomovirus 1
1941234	GCA_002004035.1	Thrips-associated genomovirus 2
1941237	GCA_002004655.1	Thrips-associated genomovirus 3
1941239	GCA_002004995.1	Thrips-associated genomovirus 4
572290	GCA_000883335.1	Thrush coronavirus HKU12-600
299200	GCA_000866365.1	Thunberg fritillary mosaic virus
101850	GCA_000901335.1	Thysanoplusia orichalcea nucleopolyhedrovirus
1163537	GCA_000894495.1	Tianjin totivirus
2169919	GCA_001679895.1	Tibetan frog hepatitis B virus
318844	GCA_000904355.1	Tibrogargan virus
1983779	GCA_004050735.1	Tick circovirus
1983779	GCA_004050755.1	Tick circovirus
2025477	GCA_003848685.1	Tick-associated genomovirus 1
2025478	GCA_003848665.1	Tick-associated genomovirus 2
2025479	GCA_003848645.1	Tick-associated genomovirus 3
11084	GCA_000863125.1	Tick-borne encephalitis virus
43764	GCA_000886035.1	Tiger puffer nervous necrosis virus
1549864	GCA_001630085.1	Tilapia lake virus
2018685	GCA_002219325.1	Tinamou hepatitis B virus

2029771	GCA_002355025.1	Tioga picorna-like virus 1
162013	GCA_000852405.1	Tioman virus
1546257	GCA_000930875.1	Tipula oleracea nudivirus
994672	GCA_000905855.1	Titi monkey adenovirus ECC-2011
2053587	GCA_002957265.1	TM7 phage DolZOral124_53_65
184020	GCA_000851725.1	Tobacco bushy top virus
298974	GCA_000844225.1	Tobacco bushy top virus satellite-like RNA
926844	GCA_000864785.1	Tobacco curly shoot alphasatellite
926844	GCA_003028935.1	Tobacco curly shoot alphasatellite
219596	GCA_000845605.1	Tobacco curly shoot betasatellite
223250	GCA_000859325.1	Tobacco curly shoot virus - [Y41]
12227	GCA_000861345.1	Tobacco etch virus
1213187	GCA_000897935.1	Tobacco leaf chlorosis betasatellite
587314	GCA_002987885.1	Tobacco leaf curl betasatellite-[Pakistan:Multan:Pedilanthu
439423	GCA_002986905.1	Tobacco leaf curl Comoros virus
336987	GCA_001629945.1	Tobacco leaf curl Cuba virus
336987	GCA_002310935.1	Tobacco leaf curl Cuba virus
169691	GCA_000845585.1	Tobacco leaf curl disease associated sequence
2010326	GCA_000871565.1	Tobacco leaf curl Japan betasatellite
223332	GCA_000842145.1	Tobacco leaf curl Japan virus
2010327	GCA_002830245.1	Tobacco leaf curl Patna betasatellite
905055	GCA_000887695.1	Tobacco leaf curl PUSA alphasatellite
905054	GCA_000890215.1	Tobacco leaf curl Pusa virus
409287	GCA_000871705.1	Tobacco leaf curl Thailand virus
67762	GCA_001634495.1	Tobacco leaf curl virus
218845	GCA_000846005.2	Tobacco leaf curl Yunnan betasatellite
218845	GCA_003052185.1	Tobacco leaf curl Yunnan betasatellite
269282	GCA_000860165.1	Tobacco leaf curl Yunnan virus - [Y136]
223337	GCA_000838925.1	Tobacco leaf curl Zimbabwe virus
196691	GCA_002986915.1	Tobacco leaf rugose virus
12241	GCA_000847545.1	Tobacco mild green mosaic virus
12242	GCA_000854365.1	Tobacco mosaic virus
1792583	GCA_001646355.1	Tobacco mosqueado virus
530536	GCA_002986925.1	Tobacco mottle leaf curl virus
12055	GCA_000857065.1	Tobacco necrosis virus A
12056	GCA_000848725.1	Tobacco necrosis virus D
12295	GCA_000851445.1	Tobacco rattle virus
12282	GCA_000856105.1	Tobacco ringspot virus
31504	GCA_000840125.1	Tobacco ringspot virus satellite RNA
12317	GCA_000865505.1	Tobacco streak virus
33765	GCA_000873745.1	Tobacco vein banding mosaic virus
107324	GCA_000837445.1	Tobacco vein clearing virus
192203	GCA_000879975.1	Tobacco vein distorting virus
12228	GCA_000860705.1	Tobacco vein mottling virus
1692045	GCA_001271275.1	Tobacco virus 1
1972995	GCA_002087205.1	Tobacco virus 2
565052	GCA_000892135.1	Tobacco yellow crinkle virus
10830	GCA_000840085.1	Tobacco yellow dwarf virus
1615758	GCA_001550785.1	Tofla virus
1826170	GCA_001654305.1	Tokyovirus A1

939923	GCA_000887895.1	Tolypocladium cylindrosporum virus 1
2060142	GCA_002890115.1	Tomato apical leaf curl virus
12885	GCA_000848345.1	Tomato apical stunt viroid
12315	GCA_000853065.1	Tomato aspermy virus
2027645	GCA_002285045.1	Tomato associated geminivirus 1
234829	GCA_000843945.1	Tomato begomovirus satellite DNA beta
12275	GCA_000853325.1	Tomato black ring virus
196089	GCA_000855605.1	Tomato black ring virus satellite RNA
1010616	GCA_000911635.1	Tomato blistering mosaic virus
1340746	GCA_002823845.1	Tomato bright yellow mosaic virus
858512	GCA_002823865.1	Tomato bright yellow mottle virus
1761477	GCA_001461485.1	Tomato brown rugose fruit virus
12145	GCA_000858805.1	Tomato bushy stunt virus
135749	GCA_000846405.1	Tomato bushy stunt virus satellite RNA B1
240492	GCA_000842645.1	Tomato chino La Paz virus
269300	GCA_002823905.1	Tomato chino La Paz virus - [Baja California Sur]
1127768	GCA_002823885.1	Tomato Chino La Paz virus -SLP 4
67754	GCA_000864085.1	Tomato chlorosis virus
100785	GCA_000855405.1	Tomato chlorotic dwarf viroid
983437	GCA_000894835.2	Tomato chlorotic leaf distortion virus-[Venezuela:Zulia:200
1701549	GCA_003029095.1	Tomato chlorotic mottle Guyane virus
1701549	GCA_003029095.2	Tomato chlorotic mottle Guyane virus
172391	GCA_002823925.1	Tomato chlorotic mottle virus
223338	GCA_000838345.1	Tomato chlorotic mottle virus-[Brazil]
223339	GCA_002823945.1	Tomato chlorotic mottle virus-Crumple
12851	GCA_002270845.1	Tomato chlorotic spot virus
661101	GCA_000885175.1	Tomato chocolate spot virus
536084	GCA_000879555.1	Tomato common mosaic virus
269301	GCA_000841345.1	Tomato curly stunt virus - [South Africa]
1195378	GCA_000894215.1	Tomato dwarf leaf virus
1632184	GCA_000969195.1	Tomato enation leaf curl virus
858511	GCA_002823965.1	Tomato golden leaf distortion virus
1336597	GCA_000909455.1	Tomato golden leaf spot virus
223341	GCA_000839565.1	Tomato golden mosaic virus-Yellow vein
223342	GCA_000839225.1	Tomato golden mottle virus
296190	GCA_002867655.1	Tomato golden vein virus
52135	GCA_000885955.1	Tomato infectious chlorosis virus
1089387	GCA_002823985.1	Tomato interveinal chlorosis virus
1608257	GCA_003029065.1	Tomato latent virus
1407057	GCA_001961415.1	Tomato leaf curl alphasatellite
439422	GCA_002986935.1	Tomato leaf curl Anjouan virus
392761	GCA_000868885.1	Tomato leaf curl Arusha virus
2010331	GCA_002830265.1	Tomato leaf curl Bangalore betasatellite
220608	GCA_000839265.1	Tomato leaf curl Bangalore virus
220608	GCA_002824005.1	Tomato leaf curl Bangalore virus
407832	GCA_002824065.1	Tomato leaf curl Bangalore virus - [India:Kerala II:2005]
220935	GCA_002824045.1	Tomato leaf curl Bangalore virus-[Ban4]
220933	GCA_002824025.1	Tomato leaf curl Bangalore virus-[Ban5]
486898	GCA_000872385.1	Tomato leaf curl Bangalore virus-[Ban5] satellite DNA beta
885379	GCA_000889335.1	Tomato leaf curl Bangladesh betasatellite

885379	GCA_002987895.1	Tomato leaf curl Bangladesh betasatellite
223344	GCA_000840445.1	Tomato leaf curl Bangladesh virus
1498504	GCA_000922655.1	Tomato leaf curl Barka virus
220812	GCA_000848965.1	Tomato leaf curl betasatellite
220812	GCA_002987905.1	Tomato leaf curl betasatellite
220812	GCA_002987995.1	Tomato leaf curl betasatellite
1955054	GCA_002005645.1	Tomato leaf curl Burkina Faso virus
743040	GCA_000890355.1	Tomato leaf curl Cameroon alphasatellite
743041	GCA_003034055.1	Tomato leaf curl Cameroon alphasatellite [CM:OMHD3:Ok
743042	GCA_003034065.1	Tomato leaf curl Cameroon alphasatellite [CM:TOS2D1:To:
693895	GCA_000885495.1	Tomato leaf curl Cameroon virus - [Cameroon:Buea:Okra:2
507474	GCA_000875005.1	Tomato leaf curl Cebu virus
278069	GCA_000862785.1	Tomato leaf curl China betasatellite
278069	GCA_002987915.1	Tomato leaf curl China betasatellite
278069	GCA_002988005.1	Tomato leaf curl China betasatellite
229636	GCA_002824085.1	Tomato leaf curl China virus
229636	GCA_002986955.1	Tomato leaf curl China virus
269302	GCA_002986945.1	Tomato leaf curl China virus - [G18]
269303	GCA_000842525.1	Tomato leaf curl China virus - [G32]
1329381	GCA_000907295.1	Tomato leaf curl China virus - OX2
997078	GCA_001430535.1	Tomato leaf curl Comoros virus
507475	GCA_000879815.1	Tomato leaf curl Cotabato virus
1993650	GCA_000843845.1	Tomato leaf curl deltasatellite
439426	GCA_002986995.1	Tomato leaf curl Diana virus
1384632	GCA_000915235.1	Tomato leaf curl Gandhinagar betasatellite
1384631	GCA_000914215.1	Tomato leaf curl Gandhinagar virus
501328	GCA_000874905.1	Tomato leaf curl Ghana virus
400835	GCA_000869445.1	Tomato leaf curl Guangdong virus
377609	GCA_000868445.1	Tomato leaf curl Guangxi virus
269306	GCA_000841205.1	Tomato leaf curl Gujarat virus - [Varanasi]
664980	GCA_000885835.1	Tomato leaf curl Hainan virus
664980	GCA_002824105.1	Tomato leaf curl Hainan virus
671275	GCA_002986745.1	Tomato leaf curl Hainan virus HaNHK7
1231334	GCA_000899255.1	Tomato leaf curl Hajipur betasatellite
971271	GCA_000892255.1	Tomato leaf curl Hanoi virus
270140	GCA_000845745.1	Tomato leaf curl Iran virus
53991	GCA_002830285.1	Tomato leaf curl Java betasatellite
263001	GCA_000842305.1	Tomato leaf curl Java virus
263577	GCA_002824125.1	Tomato leaf curl Java virus-[Ageratum]
263578	GCA_000844785.1	Tomato leaf curl Java virus-[Ageratum] satellite DNA
436243	GCA_000871425.1	Tomato leaf curl Joydebpur betasatellite
436243	GCA_002987935.1	Tomato leaf curl Joydebpur betasatellite
1000690	GCA_002830005.1	Tomato leaf curl Joydebpur betasatellite [India/Jaunpur/Ch
309390	GCA_000864425.1	Tomato leaf curl Joydebpur virus
309390	GCA_002824145.1	Tomato leaf curl Joydebpur virus
1397670	GCA_000869485.1	Tomato leaf curl Karnataka betasatellite
220932	GCA_000840185.1	Tomato leaf curl Karnataka virus
220932	GCA_900079205.1	Tomato leaf curl Karnataka virus
220932	GCA_900079195.1	Tomato leaf curl Karnataka virus
220932	GCA_900079235.1	Tomato leaf curl Karnataka virus

220932	GCA_900079245.1	Tomato leaf curl Karnataka virus
551531	GCA_000881415.1	Tomato leaf curl Kerala virus
551531	GCA_900079225.1	Tomato leaf curl Kerala virus
548915	GCA_000880355.1	Tomato leaf curl Kumasi virus
2010332	GCA_002830305.1	Tomato leaf curl Laguna betasatellite
2010333	GCA_002987955.1	Tomato leaf curl Laos betasatellite
223345	GCA_000842065.1	Tomato leaf curl Laos virus
1303385	GCA_000915295.1	Tomato leaf curl Liwa virus
996312	GCA_002987025.1	Tomato leaf curl Madagascar virus-Androy [Madagascar:To
996311	GCA_000857385.1	Tomato leaf curl Madagascar virus-Menabe [Madagascar:N
2049892	GCA_002830325.1	Tomato leaf curl Malaysia betasatellite
223346	GCA_000843005.1	Tomato leaf curl Malaysia virus
260379	GCA_000841705.1	Tomato leaf curl Mali virus
302100	GCA_000859125.1	Tomato leaf curl Mayotte virus
507476	GCA_000872745.1	Tomato leaf curl Mindanao virus
439425	GCA_002987035.1	Tomato leaf curl Moheli virus
439421	GCA_002986975.1	Tomato leaf curl Namakely virus
439421	GCA_002987045.1	Tomato leaf curl Namakely virus
2031200	GCA_002987965.1	Tomato leaf curl Nepal betasatellite
478812	GCA_000846505.1	Tomato leaf curl New Delhi betasatellite
223347	GCA_900079285.1	Tomato leaf curl New Delhi virus
223347	GCA_900079275.1	Tomato leaf curl New Delhi virus
223347	GCA_900079305.1	Tomato leaf curl New Delhi virus
223347	GCA_900079295.1	Tomato leaf curl New Delhi virus
223347	GCA_900079215.1	Tomato leaf curl New Delhi virus
223347	GCA_900079325.1	Tomato leaf curl New Delhi virus
2050583	GCA_002824165.1	Tomato leaf curl New Delhi virus 2
2049944	GCA_002824185.1	Tomato leaf curl New Delhi virus 4
223351	GCA_000842925.1	Tomato leaf curl New Delhi virus-Severe
614034	GCA_000883635.1	Tomato leaf curl Nigeria virus-[Nigeria:2006]
713949	GCA_000888615.1	Tomato leaf curl Oman virus
538004	GCA_000884935.1	Tomato leaf curl Pakistan alphasatellite
1609290	GCA_002080175.1	Tomato leaf curl Pakistan betasatellite
526476	GCA_000875365.1	Tomato leaf curl Palampur virus
526476	GCA_900079265.1	Tomato leaf curl Palampur virus
526476	GCA_900079315.1	Tomato leaf curl Palampur virus
526476	GCA_900079255.1	Tomato leaf curl Palampur virus
575918	GCA_000882795.1	Tomato leaf curl Patna betasatellite
575917	GCA_000881195.1	Tomato leaf curl Patna virus
2173168	GCA_000870925.1	Tomato leaf curl Philippine betasatellite
96054	GCA_000840685.1	Tomato leaf curl Philippines virus
499612	GCA_002824225.1	Tomato leaf curl Philippines virus - Laguna
499613	GCA_002824205.1	Tomato leaf curl Philippines virus - Los Banos:L3
407838	GCA_000868585.1	Tomato leaf curl Pune virus - [India:Pune:2005]
2021667	GCA_002270925.1	Tomato leaf curl purple vein virus
420254	GCA_002824245.1	Tomato leaf curl Rajasthan virus - [India:Rajasthan:2005]
938276	GCA_000896855.1	Tomato leaf curl Ranchi virus
425303	GCA_000871445.1	Tomato leaf curl Seychelles virus
71186	GCA_000871805.1	Tomato leaf curl Sinaloa virus
223352	GCA_000841305.1	Tomato leaf curl Sri Lanka virus

270146	GCA_002824265.1	Tomato leaf curl Sudan virus
270146	GCA_002824305.1	Tomato leaf curl Sudan virus
270147	GCA_000842665.1	Tomato leaf curl Sudan virus - [Gezira]
270148	GCA_002824285.1	Tomato leaf curl Sudan virus - [Shambat]
634441	GCA_000886855.1	Tomato leaf curl Sulawesi virus
196093	GCA_000840145.1	Tomato leaf curl Taiwan virus
196093	GCA_002824325.1	Tomato leaf curl Taiwan virus
196093	GCA_002824345.1	Tomato leaf curl Taiwan virus
196093	GCA_002824365.1	Tomato leaf curl Taiwan virus
929051	GCA_000889475.1	Tomato leaf curl Togo betasatellite-[Togo:2006]
439427	GCA_002987065.1	Tomato leaf curl Toliara virus
341035	GCA_002824385.1	Tomato leaf curl Uganda virus - [Iganga]
208177	GCA_000842785.1	Tomato leaf curl Vietnam virus
28350	GCA_002824425.1	Tomato leaf curl virus
28350	GCA_002824445.1	Tomato leaf curl virus
28350	GCA_002824465.1	Tomato leaf curl virus
223353	GCA_000838425.1	Tomato leaf curl virus-[AU]
223354	GCA_002824405.1	Tomato leaf curl virus-[Solanum species D1]
315392	GCA_000867705.1	Tomato leaf curl virus-Pune-associated DNA beta
1176156	GCA_000901415.1	Tomato leaf curl Yemen betasatellite
2203894	GCA_004131285.1	Tomato leaf curl Yunnan betasatellite
681518	GCA_000888595.1	Tomato leaf deformation virus
536085	GCA_002824485.1	Tomato leaf distortion virus
470166	GCA_000879575.1	Tomato marchitez virus
536086	GCA_000874565.1	Tomato mild mosaic virus
178599	GCA_002987495.1	Tomato mild mottle virus
1124577	GCA_000870725.1	Tomato mild yellow leaf curl Aragua virus
223357	GCA_000837605.1	Tomato mosaic Havana virus-[Quivican]
12253	GCA_000853705.1	Tomato mosaic virus
172394	GCA_001891035.1	Tomato mottle leaf curl virus
172394	GCA_002867675.1	Tomato mottle leaf curl virus
1391702	GCA_000911995.1	Tomato mottle mosaic virus
223358	GCA_000838745.1	Tomato mottle Taino virus
223359	GCA_000837105.1	Tomato mottle virus-[Florida]
1266458	GCA_000926355.1	Tomato mottle wrinkle virus
1481465	GCA_001308355.1	Tomato necrotic dwarf virus
1637492	GCA_003033825.1	Tomato necrotic streak virus
1176153	GCA_000898035.1	Tomato necrotic stunt virus
12888	GCA_000856425.1	Tomato planta macho viroid
49267	GCA_000848785.1	Tomato pseudo-curly top virus
12280	GCA_000860465.1	Tomato ringspot virus
223360	GCA_000837205.1	Tomato rugose mosaic virus-[Ube]
1162280	GCA_000904155.2	Tomato rugose yellow leaf curl virus
1162280	GCA_000904155.1	Tomato rugose yellow leaf curl virus
92963	GCA_002987075.1	Tomato severe leaf curl virus
1086071	GCA_002824505.1	Tomato severe leaf curl virus - Huasteca
223361	GCA_000845785.1	Tomato severe leaf curl virus-[Guatemala 96-1]
158463	GCA_000874065.1	Tomato severe rugose virus
1933298	GCA_000854725.1	Tomato spotted wilt tospovirus
370833	GCA_000872965.1	Tomato torrado virus

427315	GCA_000872085.1	Tomato yellow dwarf disease associated satellite DNA beta
232380	GCA_000927655.1	Tomato yellow leaf curl Axarquia virus
391090	GCA_000875665.1	Tomato yellow leaf curl betasatellite
240557	GCA_000863825.1	Tomato yellow leaf curl China alphasatellite
240557	GCA_003034015.1	Tomato yellow leaf curl China alphasatellite
1255765	GCA_000900915.1	Tomato yellow leaf curl China betasatellite
185793	GCA_000862185.1	Tomato yellow leaf curl China virus
185793	GCA_002824525.1	Tomato yellow leaf curl China virus
185793	GCA_002987125.1	Tomato yellow leaf curl China virus
408092	GCA_002824545.1	Tomato yellow leaf curl China virus-[YN72]
221527	GCA_002987095.1	Tomato yellow leaf curl China virus-Tb[Y10]
221526	GCA_002987135.1	Tomato yellow leaf curl China virus-Tb[Y5]
221532	GCA_002987105.1	Tomato yellow leaf curl China virus-To[Y25]
297249	GCA_000868505.1	Tomato yellow leaf curl Guangdong virus
395349	GCA_000869285.1	Tomato yellow leaf curl Indonesia virus-[Lembang]
266800	GCA_000840985.1	Tomato yellow leaf curl Kanchanaburi virus-[Thailand Kan1
219744	GCA_000842905.1	Tomato yellow leaf curl Malaga virus
260377	GCA_001028945.1	Tomato yellow leaf curl Mali virus
260377	GCA_002824585.1	Tomato yellow leaf curl Mali virus
391337	GCA_002824565.1	Tomato yellow leaf curl Mali virus-[Ethiopia]
2083207	GCA_002830345.1	Tomato yellow leaf curl Rajasthan betasatellite
123735	GCA_000844945.1	Tomato yellow leaf curl Sardinia virus
1391746	GCA_000910955.1	Tomato yellow leaf curl Saudi virus
2040662	GCA_002830365.1	Tomato yellow leaf curl Shandong betasatellite
1825931	GCA_001634615.1	Tomato yellow leaf curl Shuangbai virus - [Y4536]
2169845	GCA_000842325.1	Tomato yellow leaf curl Thailand alphasatellite
693897	GCA_000845625.1	Tomato yellow leaf curl Thailand betasatellite
221540	GCA_000857225.1	Tomato yellow leaf curl Thailand virus-[2]
447604	GCA_000873345.1	Tomato yellow leaf curl Vietnam betasatellite
390442	GCA_000873945.1	Tomato yellow leaf curl Vietnam virus
10832	GCA_002824685.1	Tomato yellow leaf curl virus
220944	GCA_002987165.1	Tomato yellow leaf curl virus - Mild
220938	GCA_000858205.1	Tomato yellow leaf curl virus-[Almeria]
761705	GCA_002824665.1	Tomato yellow leaf curl virus-[Genaveh:Iran]
761708	GCA_002824705.1	Tomato yellow leaf curl virus-[Minab:Iran]
220943	GCA_002987155.1	Tomato yellow leaf curl virus-Iran
2169846	GCA_003029525.1	Tomato yellow leaf curl Yunnan alphasatellite
2033721	GCA_002830385.1	Tomato yellow leaf curl Yunnan betasatellite
1335768	GCA_000909115.1	Tomato yellow leaf curl Yunnan virus
649996	GCA_000896215.1	Tomato yellow leaf distortion virus
260909	GCA_000843525.1	Tomato yellow margin leaf curl virus
86502	GCA_000904935.1	Tomato yellow mottle virus
1967841	GCA_002116055.1	Tomato yellow mottle-associated virus
2169827	GCA_003029425.1	Tomato yellow spot alphasatellite
365523	GCA_000865405.1	Tomato yellow spot virus
55216	GCA_000874525.1	Tomato yellow vein streak virus
460926	GCA_000879835.1	Tomato zonate spot virus
2053981	GCA_004130395.1	Tomelloso virus
60877	GCA_002829945.1	Tonate virus
1758878	GCA_001461505.1	Tongilchon virus 1

1764085	GCA_001629845.1	Toros virus
1764085	GCA_001629845.2	Toros virus
687385	GCA_000889755.1	Torque teno canis virus
687383	GCA_000889835.1	Torque teno douroucouli virus
1673633	GCA_004129255.1	Torque teno equus virus 1
687384	GCA_000887235.1	Torque teno felis virus
2065043	GCA_002818525.1	Torque teno felis virus 2
2010248	GCA_002194485.1	Torque teno indri virus 1
2012676	GCA_002210695.1	Torque teno Leptonychotes weddellii virus-1
2012677	GCA_002210895.1	Torque teno Leptonychotes weddellii virus-2
687379	GCA_000870545.1	Torque teno midi virus 1
2065051	GCA_002818685.1	Torque teno midi virus 10
2065052	GCA_002818705.1	Torque teno midi virus 11
2065053	GCA_002818725.1	Torque teno midi virus 12
2065054	GCA_002818745.1	Torque teno midi virus 13
2065055	GCA_002818765.1	Torque teno midi virus 14
2065056	GCA_002818785.1	Torque teno midi virus 15
687380	GCA_000887335.1	Torque teno midi virus 2
2065044	GCA_002818545.1	Torque teno midi virus 3
2065045	GCA_002818565.1	Torque teno midi virus 4
2065046	GCA_002818585.1	Torque teno midi virus 5
2065047	GCA_002818605.1	Torque teno midi virus 6
2065048	GCA_002818625.1	Torque teno midi virus 7
2065049	GCA_002818645.1	Torque teno midi virus 8
2065050	GCA_002818665.1	Torque teno midi virus 9
687369	GCA_000887355.1	Torque teno mini virus 1
2065036	GCA_002818445.1	Torque teno mini virus 10
2065037	GCA_002818465.1	Torque teno mini virus 11
2065038	GCA_002818485.1	Torque teno mini virus 12
1859149	GCA_001661815.1	Torque teno mini virus 18
687370	GCA_000888275.1	Torque teno mini virus 2
687371	GCA_000887315.1	Torque teno mini virus 3
687372	GCA_000888295.1	Torque teno mini virus 4
687373	GCA_000888975.1	Torque teno mini virus 5
687374	GCA_000888315.1	Torque teno mini virus 6
687375	GCA_000888255.1	Torque teno mini virus 7
687376	GCA_000887215.1	Torque teno mini virus 8
687377	GCA_000837005.1	Torque teno mini virus 9
1535290	GCA_000930495.1	Torque teno mini virus ALA22
1535291	GCA_000929855.1	Torque teno mini virus ALH8
687386	GCA_000888195.1	Torque teno sus virus 1a
687387	GCA_001008435.1	Torque teno sus virus 1b
1968861	GCA_000888335.1	Torque teno sus virus k2a
1239832	GCA_002818805.1	Torque teno sus virus k2b
1543419	GCA_000921775.1	Torque teno Tadarida brasiliensis virus
687382	GCA_000888955.1	Torque teno tamarin virus
687381	GCA_002818505.1	Torque teno tupaia virus
68887	GCA_000893775.1	Torque teno virus
687340	GCA_000857545.1	Torque teno virus 1
687349	GCA_000887255.1	Torque teno virus 10

687350	GCA_002818275.1	Torque teno virus 11
687351	GCA_000889775.1	Torque teno virus 12
687352	GCA_002818305.1	Torque teno virus 13
687353	GCA_000888915.1	Torque teno virus 14
687354	GCA_000889875.1	Torque teno virus 15
687355	GCA_000889855.1	Torque teno virus 16
687356	GCA_002986165.1	Torque teno virus 17
687357	GCA_002986195.1	Torque teno virus 18
687358	GCA_000888235.1	Torque teno virus 19
687341	GCA_000890135.1	Torque teno virus 2
687359	GCA_002818335.1	Torque teno virus 20
687360	GCA_002818355.1	Torque teno virus 21
687361	GCA_002986205.1	Torque teno virus 22
687362	GCA_002818385.1	Torque teno virus 23
687363	GCA_002818405.1	Torque teno virus 24
687364	GCA_000889815.1	Torque teno virus 25
687365	GCA_000889795.1	Torque teno virus 26
687366	GCA_000888215.1	Torque teno virus 27
687367	GCA_000888895.1	Torque teno virus 28
687368	GCA_002818425.1	Torque teno virus 29
687342	GCA_000888935.1	Torque teno virus 3
687343	GCA_000886355.1	Torque teno virus 4
687344	GCA_002818195.1	Torque teno virus 5
687345	GCA_000888995.1	Torque teno virus 6
687346	GCA_000887275.1	Torque teno virus 7
687347	GCA_000887295.1	Torque teno virus 8
687348	GCA_002818245.1	Torque teno virus 9
1218490	GCA_000882055.1	Torque teno zalophus virus 1
1578826	GCA_000930675.1	Tortoise picornavirus
1483681	GCA_000920635.1	Tortoise rafivirus A
1736634	GCA_001401365.1	Torulaspora delbrueckii dsRNA Mbarr-1 killer virus
11590	GCA_000854285.1	Toscana virus
1857323	GCA_001675505.1	Tospovirus kiwifruit/YXW/2014
2169782	GCA_003181335.1	Toxoneuron nigriceps bracovirus
944580	GCA_000892335.1	Trailing lespedeza virus 1
11149	GCA_002985995.1	Transmissible gastroenteritis virus
47680	GCA_000848065.1	Tree shrew adenovirus 1
47680	GCA_002818135.1	Tree shrew adenovirus 1
2218588	GCA_004133145.1	Trematomus bernacchii polyomavirus 1
2170103	GCA_000989095.1	Trematomus pennellii polyomavirus 1
70796	GCA_000863245.1	Tremovirus A
103442	GCA_000853005.1	Triatoma virus
1144379	GCA_000895335.1	Trichechus manatus latirostris papillomavirus 2
291589	GCA_000845185.1	Trichechus manatus papillomavirus 1
1429799	GCA_002827085.1	Trichechus manatus papillomavirus 3
1435497	GCA_001440955.1	Trichechus manatus papillomavirus 4
2305250	GCA_004133305.1	Trichoderma asperellum dsRNA virus 1
1934322	GCA_001973855.1	Trichoderma atroviride mycovirus
2315392	GCA_004128155.1	Trichoderma harzianum bipartite mycovirus 1
862909	GCA_000887495.1	Trichodysplasia spinulosa-associated polyomavirus

674953	GCA_000852245.1	Trichomonas vaginalis virus 1
674953	GCA_001271095.1	Trichomonas vaginalis virus 1
674954	GCA_000850845.1	Trichomonas vaginalis virus 2
170965	GCA_000851585.1	Trichomonas vaginalis virus 3
1008292	GCA_002830765.1	Trichomonas vaginalis virus 4
1646350	GCA_001271215.1	Trichomonas vaginalis virus dsRNA satellite S1
1646351	GCA_001271055.1	Trichomonas vaginalis virus dsRNA satellite S1prime
328615	GCA_000868565.1	Trichoplusia ni ascovirus 2c
134606	GCA_000839705.1	Trichoplusia ni cypovirus 15
1916701	GCA_002819285.1	Trichoplusia ni granulovirus LBIV-12
332054	GCA_000864125.1	Trichoplusia ni single nucleopolyhedrovirus
2083181	GCA_002826745.1	Trichoplusia ni TED virus
1590173	GCA_000931275.1	Trifolium-associated circular DNA virus 1
431317	GCA_000883975.1	Triticum mosaic virus
1825950	GCA_003029285.1	Triumfetta yellow mosaic virus
1825950	GCA_003029285.2	Triumfetta yellow mosaic virus
327387	GCA_001654245.1	Tropical soda apple mosaic virus
1636545	GCA_001500595.1	Tsukamurella phage TIN2
1636546	GCA_001502635.1	Tsukamurella phage TIN3
1636547	GCA_002623325.1	Tsukamurella phage TIN4
981330	GCA_000890675.1	Tsukamurella phage TPA2
1647476	GCA_001737075.1	Tsukamurella phage TPA4
93678	GCA_000905335.1	TTV-like mini virus
941765	GCA_000889635.1	Tuber aestivum betaendornavirus
1037523	GCA_000893695.1	Tuber aestivum mitovirus
927810	GCA_002830725.1	Tuber aestivum virus 1
2293308	GCA_003847425.1	Tubeweb spider associated circular virus 1
1138489	GCA_002029635.1	Tucunduba virus
798072	GCA_000927275.1	Tuhoko virus 1
798073	GCA_000926515.1	Tuhoko virus 2
798074	GCA_000925475.1	Tuhoko virus 3
1980494	GCA_000855225.1	Tula orthohantavirus
151043	GCA_000850805.1	Tulare apple mosaic virus
167132	GCA_000852485.1	Tulip virus X
1810944	GCA_004128175.1	Tunis virus
1421067	GCA_002826725.1	Tunisvirus fontaine2
2170402	GCA_004132925.1	Tupaia glis polyomavirus 1
1768064	GCA_001502175.1	Tupaia hepatovirus A
92129	GCA_000848605.1	Tupaia paramyxovirus
318558	GCA_000861765.1	Tupaia virus
10397	GCA_000849685.1	Tupaiid betaherpesvirus 1
2126985	GCA_002966485.1	Tupanvirus soda lake
878329	GCA_000888635.1	Turkey adenovirus 1
41678	GCA_000845965.1	Turkey adenovirus 3
41678	GCA_000856905.1	Turkey adenovirus 3
2170130	GCA_000918075.1	Turkey associated porprismacovirus 1
1408257	GCA_000912195.1	Turkey aviadenovirus 4
1408258	GCA_000913655.1	Turkey aviadenovirus 5
1293536	GCA_002816595.1	Turkey avisivirus
11152	GCA_000880055.1	Turkey coronavirus

1214230	GCA_000897475.1	Turkey gallivirus
980964	GCA_000906415.1	Turkey hepatitis virus 2993D
740933	GCA_000921275.1	Turkey parvovirus 1078
740934	GCA_002827205.1	Turkey parvovirus 260
336486	GCA_001431935.1	Turkeypox virus
11988	GCA_000853045.1	Turnip crinkle virus
196094	GCA_000843865.1	Turnip crinkle virus satellite RNA
298975	GCA_000845045.1	Turnip crinkle virus virulent satellite RNA C
859650	GCA_000887455.1	Turnip curly top virus
859650	GCA_002825605.1	Turnip curly top virus
859650	GCA_002825625.1	Turnip curly top virus
859650	GCA_002825645.1	Turnip curly top virus
1766828	GCA_001550845.1	Turnip leaf roll virus
12230	GCA_000863465.1	Turnip mosaic virus
392504	GCA_000885935.1	Turnip ringspot virus
218923	GCA_000916675.1	Turnip rosette virus
218923	GCA_002830925.1	Turnip rosette virus
29272	GCA_000849245.1	Turnip vein-clearing virus
12154	GCA_000862065.1	Turnip yellow mosaic virus
131083	GCA_000855905.1	Turnip yellows virus
936059	GCA_000874765.1	Tursiops truncatus papillomavirus 1
936060	GCA_000867365.1	Tursiops truncatus papillomavirus 2
1144380	GCA_003179235.1	Tursiops truncatus papillomavirus 4
1144381	GCA_003179255.1	Tursiops truncatus papillomavirus 5
1144382	GCA_003179275.1	Tursiops truncatus papillomavirus 6
1144383	GCA_003179295.1	Tursiops truncatus papillomavirus 7
496865	GCA_003178195.1	Tursiops truncatus papillomavirus type 3
2292642	GCA_004133565.1	Turtle grass virus X
604276	GCA_000880235.1	TYLCAxV-Sic1-[IT:Sic2/2:04]
366686	GCA_000865445.1	TYLCCNV-[Y322] satellite DNA beta
40004	GCA_000914295.1	Tyuleniy virus
1923781	GCA_002004695.1	Ubei picorna-like virus 3
64297	GCA_002004295.1	Uganda S virus
946046	GCA_000888855.1	Ugandan cassava brown streak virus
946046	GCA_002827965.1	Ugandan cassava brown streak virus
40060	GCA_000923315.1	Umatilla virus
552554	GCA_002814435.1	Umbre virus
348686	GCA_003178015.1	Uncia uncia papillomavirus type 1
2100421	GCA_003715865.1	uncultured Caudovirales phage
2100421	GCA_003714425.1	uncultured Caudovirales phage
2100421	GCA_003714345.1	uncultured Caudovirales phage
2100421	GCA_003716245.1	uncultured Caudovirales phage
2100421	GCA_003716185.1	uncultured Caudovirales phage
2100421	GCA_003714025.1	uncultured Caudovirales phage
2100421	GCA_003716605.1	uncultured Caudovirales phage
2100421	GCA_003714165.1	uncultured Caudovirales phage
2100421	GCA_003713805.1	uncultured Caudovirales phage
2100421	GCA_003714805.1	uncultured Caudovirales phage
2100421	GCA_003715745.1	uncultured Caudovirales phage
2100421	GCA_003714005.1	uncultured Caudovirales phage

2100421	GCA_003715405.1	uncultured Caudovirales phage
2100421	GCA_003713925.1	uncultured Caudovirales phage
2100421	GCA_003715145.1	uncultured Caudovirales phage
2100421	GCA_003713845.1	uncultured Caudovirales phage
2100421	GCA_003714545.1	uncultured Caudovirales phage
2100421	GCA_003715185.1	uncultured Caudovirales phage
2100421	GCA_003714905.1	uncultured Caudovirales phage
2100421	GCA_003715845.1	uncultured Caudovirales phage
2100421	GCA_003714645.1	uncultured Caudovirales phage
2100421	GCA_003713465.1	uncultured Caudovirales phage
2100421	GCA_003714245.1	uncultured Caudovirales phage
2100421	GCA_003714725.1	uncultured Caudovirales phage
2100421	GCA_003715025.1	uncultured Caudovirales phage
2100421	GCA_003715925.1	uncultured Caudovirales phage
2100421	GCA_003715705.1	uncultured Caudovirales phage
2100421	GCA_003715445.1	uncultured Caudovirales phage
2100421	GCA_003713545.1	uncultured Caudovirales phage
2100421	GCA_003714485.1	uncultured Caudovirales phage
2100421	GCA_003713365.1	uncultured Caudovirales phage
2100421	GCA_003713485.1	uncultured Caudovirales phage
2100421	GCA_003715085.1	uncultured Caudovirales phage
2100421	GCA_003714945.1	uncultured Caudovirales phage
2100421	GCA_003713865.1	uncultured Caudovirales phage
2100421	GCA_003713625.1	uncultured Caudovirales phage
2100421	GCA_003714745.1	uncultured Caudovirales phage
2100421	GCA_003714585.1	uncultured Caudovirales phage
2100421	GCA_003715545.1	uncultured Caudovirales phage
2100421	GCA_003715425.1	uncultured Caudovirales phage
2100421	GCA_003713425.1	uncultured Caudovirales phage
2100421	GCA_003714185.1	uncultured Caudovirales phage
2100421	GCA_003715245.1	uncultured Caudovirales phage
2100421	GCA_003716145.1	uncultured Caudovirales phage
2100421	GCA_003713965.1	uncultured Caudovirales phage
2100421	GCA_003716205.1	uncultured Caudovirales phage
2100421	GCA_003714985.1	uncultured Caudovirales phage
2100421	GCA_003715885.1	uncultured Caudovirales phage
2100421	GCA_003713725.1	uncultured Caudovirales phage
2100421	GCA_003716625.1	uncultured Caudovirales phage
2100421	GCA_003716285.1	uncultured Caudovirales phage
2100421	GCA_003715645.1	uncultured Caudovirales phage
2100421	GCA_003714445.1	uncultured Caudovirales phage
2100421	GCA_003715605.1	uncultured Caudovirales phage
2100421	GCA_003715385.1	uncultured Caudovirales phage
2100421	GCA_003716665.1	uncultured Caudovirales phage
2100421	GCA_003716265.1	uncultured Caudovirales phage
2100421	GCA_003714045.1	uncultured Caudovirales phage
2100421	GCA_003716005.1	uncultured Caudovirales phage
2100421	GCA_003716065.1	uncultured Caudovirales phage
2100421	GCA_003716025.1	uncultured Caudovirales phage
2100421	GCA_003714825.1	uncultured Caudovirales phage

2100421	GCA_003716645.1	uncultured Caudovirales phage
2100421	GCA_003716705.1	uncultured Caudovirales phage
2100421	GCA_003715765.1	uncultured Caudovirales phage
2100421	GCA_003716725.1	uncultured Caudovirales phage
2100421	GCA_003715485.1	uncultured Caudovirales phage
2100421	GCA_003715805.1	uncultured Caudovirales phage
2100421	GCA_003714125.1	uncultured Caudovirales phage
2100421	GCA_003716225.1	uncultured Caudovirales phage
2100421	GCA_003715585.1	uncultured Caudovirales phage
2100421	GCA_003715505.1	uncultured Caudovirales phage
2100421	GCA_003713705.1	uncultured Caudovirales phage
2100421	GCA_003716815.1	uncultured Caudovirales phage
2100421	GCA_003714765.1	uncultured Caudovirales phage
2100421	GCA_003716835.1	uncultured Caudovirales phage
2100421	GCA_003713665.1	uncultured Caudovirales phage
2100421	GCA_003715345.1	uncultured Caudovirales phage
2100421	GCA_003715045.1	uncultured Caudovirales phage
2100421	GCA_003715945.1	uncultured Caudovirales phage
2100421	GCA_003713785.1	uncultured Caudovirales phage
2100421	GCA_003715065.1	uncultured Caudovirales phage
2100421	GCA_003714665.1	uncultured Caudovirales phage
2100421	GCA_003714365.1	uncultured Caudovirales phage
2100421	GCA_003715965.1	uncultured Caudovirales phage
2100421	GCA_003715525.1	uncultured Caudovirales phage
2100421	GCA_003713885.1	uncultured Caudovirales phage
2100421	GCA_003714865.1	uncultured Caudovirales phage
2100421	GCA_003714605.1	uncultured Caudovirales phage
2100421	GCA_003715265.1	uncultured Caudovirales phage
2100421	GCA_003715565.1	uncultured Caudovirales phage
2100421	GCA_003713445.1	uncultured Caudovirales phage
2100421	GCA_003714205.1	uncultured Caudovirales phage
2100421	GCA_003715305.1	uncultured Caudovirales phage
2100421	GCA_003716165.1	uncultured Caudovirales phage
2100421	GCA_003713985.1	uncultured Caudovirales phage
2100421	GCA_003713745.1	uncultured Caudovirales phage
2100421	GCA_003714705.1	uncultured Caudovirales phage
2100421	GCA_003713645.1	uncultured Caudovirales phage
2100421	GCA_003715665.1	uncultured Caudovirales phage
2100421	GCA_003714465.1	uncultured Caudovirales phage
2100421	GCA_003714065.1	uncultured Caudovirales phage
2100421	GCA_003715105.1	uncultured Caudovirales phage
2100421	GCA_003715465.1	uncultured Caudovirales phage
2100421	GCA_003713585.1	uncultured Caudovirales phage
2100421	GCA_003713385.1	uncultured Caudovirales phage
2100421	GCA_003714305.1	uncultured Caudovirales phage
2100421	GCA_003716045.1	uncultured Caudovirales phage
2100421	GCA_003715205.1	uncultured Caudovirales phage
2100421	GCA_003716105.1	uncultured Caudovirales phage
2100421	GCA_003715785.1	uncultured Caudovirales phage
2100421	GCA_003715725.1	uncultured Caudovirales phage

2100421	GCA_003714685.1	uncultured Caudovirales phage
2100421	GCA_003713505.1	uncultured Caudovirales phage
2100421	GCA_003714405.1	uncultured Caudovirales phage
2100421	GCA_003714145.1	uncultured Caudovirales phage
2100421	GCA_003715365.1	uncultured Caudovirales phage
2100421	GCA_003713345.1	uncultured Caudovirales phage
2100421	GCA_003716745.1	uncultured Caudovirales phage
2100421	GCA_003714925.1	uncultured Caudovirales phage
2100421	GCA_003714785.1	uncultured Caudovirales phage
2100421	GCA_003713765.1	uncultured Caudovirales phage
2100421	GCA_003713685.1	uncultured Caudovirales phage
2100421	GCA_003713565.1	uncultured Caudovirales phage
2100421	GCA_003714525.1	uncultured Caudovirales phage
2100421	GCA_003715625.1	uncultured Caudovirales phage
2100421	GCA_003714085.1	uncultured Caudovirales phage
2100421	GCA_003714105.1	uncultured Caudovirales phage
2100421	GCA_003716685.1	uncultured Caudovirales phage
2100421	GCA_003713905.1	uncultured Caudovirales phage
2100421	GCA_003714885.1	uncultured Caudovirales phage
2100421	GCA_003713825.1	uncultured Caudovirales phage
2100421	GCA_003714625.1	uncultured Caudovirales phage
2100421	GCA_003715825.1	uncultured Caudovirales phage
2100421	GCA_003714285.1	uncultured Caudovirales phage
2100421	GCA_003714225.1	uncultured Caudovirales phage
2100421	GCA_003715325.1	uncultured Caudovirales phage
2100421	GCA_003715005.1	uncultured Caudovirales phage
2100421	GCA_003715905.1	uncultured Caudovirales phage
2100421	GCA_003714845.1	uncultured Caudovirales phage
2100421	GCA_003715685.1	uncultured Caudovirales phage
2100421	GCA_003713525.1	uncultured Caudovirales phage
2100421	GCA_003715285.1	uncultured Caudovirales phage
2100421	GCA_003715125.1	uncultured Caudovirales phage
2100421	GCA_003714505.1	uncultured Caudovirales phage
2100421	GCA_003713605.1	uncultured Caudovirales phage
2100421	GCA_003714565.1	uncultured Caudovirales phage
2100421	GCA_003714265.1	uncultured Caudovirales phage
2100421	GCA_003714385.1	uncultured Caudovirales phage
2100421	GCA_003715165.1	uncultured Caudovirales phage
2100421	GCA_003713405.1	uncultured Caudovirales phage
2100421	GCA_003714325.1	uncultured Caudovirales phage
2100421	GCA_003715985.1	uncultured Caudovirales phage
2100421	GCA_003715225.1	uncultured Caudovirales phage
2100421	GCA_003716125.1	uncultured Caudovirales phage
2100421	GCA_003713945.1	uncultured Caudovirales phage
186617	GCA_003985905.1	uncultured marine virus
186617	GCA_003985925.1	uncultured marine virus
186617	GCA_003985945.1	uncultured marine virus
186617	GCA_003985965.1	uncultured marine virus
186617	GCA_003985985.1	uncultured marine virus
186617	GCA_003986005.1	uncultured marine virus

186617	GCA_003985545.1	uncultured marine virus
186617	GCA_003985565.1	uncultured marine virus
186617	GCA_003985585.1	uncultured marine virus
186617	GCA_003985605.1	uncultured marine virus
186617	GCA_003985625.1	uncultured marine virus
186617	GCA_003985645.1	uncultured marine virus
186617	GCA_003985665.1	uncultured marine virus
186617	GCA_003985685.1	uncultured marine virus
186617	GCA_003985705.1	uncultured marine virus
186617	GCA_003985725.1	uncultured marine virus
186617	GCA_003985745.1	uncultured marine virus
186617	GCA_003985765.1	uncultured marine virus
186617	GCA_003985785.1	uncultured marine virus
186617	GCA_003985805.1	uncultured marine virus
186617	GCA_003985825.1	uncultured marine virus
186617	GCA_003985845.1	uncultured marine virus
186617	GCA_003985865.1	uncultured marine virus
186617	GCA_003985885.1	uncultured marine virus
1868660	GCA_003505095.1	uncultured Mediterranean phage
1868660	GCA_003502305.1	uncultured Mediterranean phage
1868660	GCA_003502325.1	uncultured Mediterranean phage
1868660	GCA_003502345.1	uncultured Mediterranean phage
1868660	GCA_003502365.1	uncultured Mediterranean phage
1868660	GCA_003502385.1	uncultured Mediterranean phage
1868660	GCA_003502405.1	uncultured Mediterranean phage
1868660	GCA_003502425.1	uncultured Mediterranean phage
1868660	GCA_003502475.1	uncultured Mediterranean phage
1868660	GCA_003502515.1	uncultured Mediterranean phage
1868660	GCA_003502555.1	uncultured Mediterranean phage
1868660	GCA_003502575.1	uncultured Mediterranean phage
1868660	GCA_003502595.1	uncultured Mediterranean phage
1868660	GCA_003502455.1	uncultured Mediterranean phage
1868660	GCA_003502615.1	uncultured Mediterranean phage
1868660	GCA_003502645.1	uncultured Mediterranean phage
1868660	GCA_003502665.1	uncultured Mediterranean phage
1868660	GCA_003502685.1	uncultured Mediterranean phage
1868660	GCA_003502715.1	uncultured Mediterranean phage
1868660	GCA_003505195.1	uncultured Mediterranean phage
1868660	GCA_003502735.1	uncultured Mediterranean phage
1868660	GCA_003502755.1	uncultured Mediterranean phage
1868660	GCA_003502775.1	uncultured Mediterranean phage
1868660	GCA_003502795.1	uncultured Mediterranean phage
1868660	GCA_003502835.1	uncultured Mediterranean phage
1868660	GCA_003502865.1	uncultured Mediterranean phage
1868660	GCA_003502885.1	uncultured Mediterranean phage
1868660	GCA_003502925.1	uncultured Mediterranean phage
1868660	GCA_003502945.1	uncultured Mediterranean phage
1868660	GCA_003502965.1	uncultured Mediterranean phage
1868660	GCA_003502985.1	uncultured Mediterranean phage
1868660	GCA_003503005.1	uncultured Mediterranean phage

1868660	GCA_003503025.1	uncultured Mediterranean phage
1868660	GCA_003505035.1	uncultured Mediterranean phage
1868660	GCA_003502535.1	uncultured Mediterranean phage
1868660	GCA_003503045.1	uncultured Mediterranean phage
1868660	GCA_003505135.1	uncultured Mediterranean phage
1868660	GCA_003505165.1	uncultured Mediterranean phage
1868660	GCA_003505225.1	uncultured Mediterranean phage
1868660	GCA_003505275.1	uncultured Mediterranean phage
1868660	GCA_003505365.1	uncultured Mediterranean phage
1868660	GCA_003505435.1	uncultured Mediterranean phage
1868660	GCA_003505455.1	uncultured Mediterranean phage
1868660	GCA_003505575.1	uncultured Mediterranean phage
1868660	GCA_003505595.1	uncultured Mediterranean phage
1868660	GCA_003505615.1	uncultured Mediterranean phage
1868660	GCA_003505645.1	uncultured Mediterranean phage
1868660	GCA_003505685.1	uncultured Mediterranean phage
1868660	GCA_003505725.1	uncultured Mediterranean phage
1868660	GCA_003505775.1	uncultured Mediterranean phage
1868660	GCA_003505755.1	uncultured Mediterranean phage
1407671	GCA_002581645.1	uncultured Mediterranean phage uvMED
1407671	GCA_002582365.1	uncultured Mediterranean phage uvMED
1407671	GCA_002581695.1	uncultured Mediterranean phage uvMED
1407671	GCA_002581745.1	uncultured Mediterranean phage uvMED
1407671	GCA_002581785.1	uncultured Mediterranean phage uvMED
1407671	GCA_002581855.1	uncultured Mediterranean phage uvMED
1407671	GCA_002581875.1	uncultured Mediterranean phage uvMED
1407671	GCA_002577775.1	uncultured Mediterranean phage uvMED
1407671	GCA_002583645.1	uncultured Mediterranean phage uvMED
1407671	GCA_002581975.1	uncultured Mediterranean phage uvMED
1407671	GCA_002577815.1	uncultured Mediterranean phage uvMED
1407671	GCA_002577855.1	uncultured Mediterranean phage uvMED
1407671	GCA_002577885.1	uncultured Mediterranean phage uvMED
1407671	GCA_002582005.1	uncultured Mediterranean phage uvMED
1407671	GCA_002583765.1	uncultured Mediterranean phage uvMED
1407671	GCA_002582045.1	uncultured Mediterranean phage uvMED
1407671	GCA_002582105.1	uncultured Mediterranean phage uvMED
1407671	GCA_002577965.1	uncultured Mediterranean phage uvMED
1407671	GCA_002582135.1	uncultured Mediterranean phage uvMED
1407671	GCA_002577985.1	uncultured Mediterranean phage uvMED
1407671	GCA_002582175.1	uncultured Mediterranean phage uvMED
1407671	GCA_002578005.1	uncultured Mediterranean phage uvMED
1407671	GCA_002578025.1	uncultured Mediterranean phage uvMED
1407671	GCA_002582215.1	uncultured Mediterranean phage uvMED
1407671	GCA_002578055.1	uncultured Mediterranean phage uvMED
1407671	GCA_002584445.1	uncultured Mediterranean phage uvMED
1407671	GCA_002582295.1	uncultured Mediterranean phage uvMED
1407671	GCA_002578125.1	uncultured Mediterranean phage uvMED
1407671	GCA_002578145.1	uncultured Mediterranean phage uvMED
1407671	GCA_002578175.1	uncultured Mediterranean phage uvMED
1407671	GCA_002580375.1	uncultured Mediterranean phage uvMED

1407671	GCA_002578215.1	uncultured Mediterranean phage uvMED
1407671	GCA_002579685.1	uncultured Mediterranean phage uvMED
1407671	GCA_002579015.1	uncultured Mediterranean phage uvMED
1407671	GCA_002578235.1	uncultured Mediterranean phage uvMED
1407671	GCA_002582405.1	uncultured Mediterranean phage uvMED
1407671	GCA_002578265.1	uncultured Mediterranean phage uvMED
1407671	GCA_002578295.1	uncultured Mediterranean phage uvMED
1407671	GCA_002578345.1	uncultured Mediterranean phage uvMED
1407671	GCA_002582495.1	uncultured Mediterranean phage uvMED
1407671	GCA_002584495.1	uncultured Mediterranean phage uvMED
1407671	GCA_002578385.1	uncultured Mediterranean phage uvMED
1407671	GCA_002583155.1	uncultured Mediterranean phage uvMED
1407671	GCA_002582525.1	uncultured Mediterranean phage uvMED
1407671	GCA_002578415.1	uncultured Mediterranean phage uvMED
1407671	GCA_002582565.1	uncultured Mediterranean phage uvMED
1407671	GCA_002578465.1	uncultured Mediterranean phage uvMED
1407671	GCA_002578525.1	uncultured Mediterranean phage uvMED
1407671	GCA_002582615.1	uncultured Mediterranean phage uvMED
1407671	GCA_002582645.1	uncultured Mediterranean phage uvMED
1407671	GCA_002578555.1	uncultured Mediterranean phage uvMED
1407671	GCA_002582675.1	uncultured Mediterranean phage uvMED
1407671	GCA_002578605.1	uncultured Mediterranean phage uvMED
1407671	GCA_002582735.1	uncultured Mediterranean phage uvMED
1407671	GCA_002578645.1	uncultured Mediterranean phage uvMED
1407671	GCA_002578675.1	uncultured Mediterranean phage uvMED
1407671	GCA_002582805.1	uncultured Mediterranean phage uvMED
1407671	GCA_002578725.1	uncultured Mediterranean phage uvMED
1407671	GCA_002582845.1	uncultured Mediterranean phage uvMED
1407671	GCA_002582885.1	uncultured Mediterranean phage uvMED
1407671	GCA_002578785.1	uncultured Mediterranean phage uvMED
1407671	GCA_002582905.1	uncultured Mediterranean phage uvMED
1407671	GCA_002584585.1	uncultured Mediterranean phage uvMED
1407671	GCA_002578805.1	uncultured Mediterranean phage uvMED
1407671	GCA_002582945.1	uncultured Mediterranean phage uvMED
1407671	GCA_002578865.1	uncultured Mediterranean phage uvMED
1407671	GCA_002583005.1	uncultured Mediterranean phage uvMED
1407671	GCA_002581915.1	uncultured Mediterranean phage uvMED
1407671	GCA_002578905.1	uncultured Mediterranean phage uvMED
1407671	GCA_002578945.1	uncultured Mediterranean phage uvMED
1407671	GCA_002583045.1	uncultured Mediterranean phage uvMED
1407671	GCA_002583075.1	uncultured Mediterranean phage uvMED
1407671	GCA_002578985.1	uncultured Mediterranean phage uvMED
1407671	GCA_002583115.1	uncultured Mediterranean phage uvMED
1407671	GCA_002579045.1	uncultured Mediterranean phage uvMED
1407671	GCA_002579065.1	uncultured Mediterranean phage uvMED
1407671	GCA_002579085.1	uncultured Mediterranean phage uvMED
1407671	GCA_002583205.1	uncultured Mediterranean phage uvMED
1407671	GCA_002583225.1	uncultured Mediterranean phage uvMED
1407671	GCA_002584745.1	uncultured Mediterranean phage uvMED
1407671	GCA_002579125.1	uncultured Mediterranean phage uvMED

1407671	GCA_002579185.1	uncultured Mediterranean phage uvMED
1407671	GCA_002583295.1	uncultured Mediterranean phage uvMED
1407671	GCA_002579225.1	uncultured Mediterranean phage uvMED
1407671	GCA_002583365.1	uncultured Mediterranean phage uvMED
1407671	GCA_002583385.1	uncultured Mediterranean phage uvMED
1407671	GCA_002584655.1	uncultured Mediterranean phage uvMED
1407671	GCA_002579315.1	uncultured Mediterranean phage uvMED
1407671	GCA_002583425.1	uncultured Mediterranean phage uvMED
1407671	GCA_002583455.1	uncultured Mediterranean phage uvMED
1407671	GCA_002579345.1	uncultured Mediterranean phage uvMED
1407671	GCA_002579405.1	uncultured Mediterranean phage uvMED
1407671	GCA_002579875.1	uncultured Mediterranean phage uvMED
1407671	GCA_002579435.1	uncultured Mediterranean phage uvMED
1407671	GCA_002583585.1	uncultured Mediterranean phage uvMED
1407671	GCA_002579495.1	uncultured Mediterranean phage uvMED
1407671	GCA_002579525.1	uncultured Mediterranean phage uvMED
1407671	GCA_002579555.1	uncultured Mediterranean phage uvMED
1407671	GCA_002583665.1	uncultured Mediterranean phage uvMED
1407671	GCA_002584005.1	uncultured Mediterranean phage uvMED
1407671	GCA_002579595.1	uncultured Mediterranean phage uvMED
1407671	GCA_002583685.1	uncultured Mediterranean phage uvMED
1407671	GCA_002579645.1	uncultured Mediterranean phage uvMED
1407671	GCA_002579255.1	uncultured Mediterranean phage uvMED
1407671	GCA_002583825.1	uncultured Mediterranean phage uvMED
1407671	GCA_002579705.1	uncultured Mediterranean phage uvMED
1407671	GCA_002583845.1	uncultured Mediterranean phage uvMED
1407671	GCA_002581335.1	uncultured Mediterranean phage uvMED
1407671	GCA_002579745.1	uncultured Mediterranean phage uvMED
1407671	GCA_002583875.1	uncultured Mediterranean phage uvMED
1407671	GCA_002579805.1	uncultured Mediterranean phage uvMED
1407671	GCA_002583925.1	uncultured Mediterranean phage uvMED
1407671	GCA_002584085.1	uncultured Mediterranean phage uvMED
1407671	GCA_002583985.1	uncultured Mediterranean phage uvMED
1407671	GCA_002579845.1	uncultured Mediterranean phage uvMED
1407671	GCA_002579275.1	uncultured Mediterranean phage uvMED
1407671	GCA_002579925.1	uncultured Mediterranean phage uvMED
1407671	GCA_002584025.1	uncultured Mediterranean phage uvMED
1407671	GCA_002577945.1	uncultured Mediterranean phage uvMED
1407671	GCA_002579955.1	uncultured Mediterranean phage uvMED
1407671	GCA_002579995.1	uncultured Mediterranean phage uvMED
1407671	GCA_002584165.1	uncultured Mediterranean phage uvMED
1407671	GCA_002584135.1	uncultured Mediterranean phage uvMED
1407671	GCA_002580065.1	uncultured Mediterranean phage uvMED
1407671	GCA_002584195.1	uncultured Mediterranean phage uvMED
1407671	GCA_002580105.1	uncultured Mediterranean phage uvMED
1407671	GCA_002584235.1	uncultured Mediterranean phage uvMED
1407671	GCA_002580035.1	uncultured Mediterranean phage uvMED
1407671	GCA_002580205.1	uncultured Mediterranean phage uvMED
1407671	GCA_002584285.1	uncultured Mediterranean phage uvMED
1407671	GCA_002582785.1	uncultured Mediterranean phage uvMED

1407671	GCA_002584335.1	uncultured Mediterranean phage uvMED
1407671	GCA_002580225.1	uncultured Mediterranean phage uvMED
1407671	GCA_002584385.1	uncultured Mediterranean phage uvMED
1407671	GCA_002584825.1	uncultured Mediterranean phage uvMED
1407671	GCA_002580255.1	uncultured Mediterranean phage uvMED
1407671	GCA_002584415.1	uncultured Mediterranean phage uvMED
1407671	GCA_002580285.1	uncultured Mediterranean phage uvMED
1407671	GCA_002580315.1	uncultured Mediterranean phage uvMED
1407671	GCA_002580425.1	uncultured Mediterranean phage uvMED
1407671	GCA_002584555.1	uncultured Mediterranean phage uvMED
1407671	GCA_002580445.1	uncultured Mediterranean phage uvMED
1407671	GCA_002580495.1	uncultured Mediterranean phage uvMED
1407671	GCA_002584615.1	uncultured Mediterranean phage uvMED
1407671	GCA_002583485.1	uncultured Mediterranean phage uvMED
1407671	GCA_002580525.1	uncultured Mediterranean phage uvMED
1407671	GCA_002580585.1	uncultured Mediterranean phage uvMED
1407671	GCA_002581435.1	uncultured Mediterranean phage uvMED
1407671	GCA_002580545.1	uncultured Mediterranean phage uvMED
1407671	GCA_002580635.1	uncultured Mediterranean phage uvMED
1407671	GCA_002584705.1	uncultured Mediterranean phage uvMED
1407671	GCA_002583525.1	uncultured Mediterranean phage uvMED
1407671	GCA_002580685.1	uncultured Mediterranean phage uvMED
1407671	GCA_002584785.1	uncultured Mediterranean phage uvMED
1407671	GCA_002580705.1	uncultured Mediterranean phage uvMED
1407671	GCA_002584895.1	uncultured Mediterranean phage uvMED
1407671	GCA_002580735.1	uncultured Mediterranean phage uvMED
1407671	GCA_002580795.1	uncultured Mediterranean phage uvMED
1407671	GCA_002584865.1	uncultured Mediterranean phage uvMED
1407671	GCA_002582245.1	uncultured Mediterranean phage uvMED
1407671	GCA_002580845.1	uncultured Mediterranean phage uvMED
1407671	GCA_002584945.1	uncultured Mediterranean phage uvMED
1407671	GCA_002580155.1	uncultured Mediterranean phage uvMED
1407671	GCA_002584965.1	uncultured Mediterranean phage uvMED
1407671	GCA_002584265.1	uncultured Mediterranean phage uvMED
1407671	GCA_002580875.1	uncultured Mediterranean phage uvMED
1407671	GCA_002584995.1	uncultured Mediterranean phage uvMED
1407671	GCA_002578085.1	uncultured Mediterranean phage uvMED
1407671	GCA_002580905.1	uncultured Mediterranean phage uvMED
1407671	GCA_002585065.1	uncultured Mediterranean phage uvMED
1407671	GCA_002580985.1	uncultured Mediterranean phage uvMED
1407671	GCA_002585085.1	uncultured Mediterranean phage uvMED
1407671	GCA_002581025.1	uncultured Mediterranean phage uvMED
1407671	GCA_002585105.1	uncultured Mediterranean phage uvMED
1407671	GCA_002585145.1	uncultured Mediterranean phage uvMED
1407671	GCA_002581045.1	uncultured Mediterranean phage uvMED
1407671	GCA_002582455.1	uncultured Mediterranean phage uvMED
1407671	GCA_002581095.1	uncultured Mediterranean phage uvMED
1407671	GCA_002581145.1	uncultured Mediterranean phage uvMED
1407671	GCA_002578105.1	uncultured Mediterranean phage uvMED
1407671	GCA_002581175.1	uncultured Mediterranean phage uvMED

1407671	GCA_002581215.1	uncultured Mediterranean phage uvMED
1407671	GCA_002581255.1	uncultured Mediterranean phage uvMED
1407671	GCA_002581295.1	uncultured Mediterranean phage uvMED
1407671	GCA_002581375.1	uncultured Mediterranean phage uvMED
1407671	GCA_002581405.1	uncultured Mediterranean phage uvMED
1407671	GCA_002581475.1	uncultured Mediterranean phage uvMED
1407671	GCA_002581535.1	uncultured Mediterranean phage uvMED
1407671	GCA_002582325.1	uncultured Mediterranean phage uvMED
1407671	GCA_002581565.1	uncultured Mediterranean phage uvMED
1407671	GCA_002580925.1	uncultured Mediterranean phage uvMED
1407671	GCA_002581595.1	uncultured Mediterranean phage uvMED
926697	GCA_002921635.1	uncultured Microviridae
926697	GCA_002921625.1	uncultured Microviridae
743531	GCA_003439975.1	uncultured phage MedDCM-OCT-S01-C1
743535	GCA_003439995.1	uncultured phage MedDCM-OCT-S04-C1035
743536	GCA_003440015.1	uncultured phage MedDCM-OCT-S04-C1161
743537	GCA_003440035.1	uncultured phage MedDCM-OCT-S04-C1201
743539	GCA_003440075.1	uncultured phage MedDCM-OCT-S04-C1227
743540	GCA_003440095.1	uncultured phage MedDCM-OCT-S04-C136
743543	GCA_003440755.1	uncultured phage MedDCM-OCT-S04-C24
743544	GCA_003440775.1	uncultured phage MedDCM-OCT-S04-C26
743547	GCA_003440835.1	uncultured phage MedDCM-OCT-S04-C507
743548	GCA_003440135.1	uncultured phage MedDCM-OCT-S04-C64
743549	GCA_003440855.1	uncultured phage MedDCM-OCT-S04-C650
743550	GCA_003440155.1	uncultured phage MedDCM-OCT-S04-C695
743553	GCA_003440175.1	uncultured phage MedDCM-OCT-S04-C890
743554	GCA_003440915.1	uncultured phage MedDCM-OCT-S04-C93
743555	GCA_003440195.1	uncultured phage MedDCM-OCT-S05-C113
743563	GCA_003440255.1	uncultured phage MedDCM-OCT-S05-C64
743564	GCA_003440275.1	uncultured phage MedDCM-OCT-S05-C767
743566	GCA_003440295.1	uncultured phage MedDCM-OCT-S06-C1041
743567	GCA_003441055.1	uncultured phage MedDCM-OCT-S08-C1115
743568	GCA_003441075.1	uncultured phage MedDCM-OCT-S08-C1281
743570	GCA_003440335.1	uncultured phage MedDCM-OCT-S08-C151
743573	GCA_003440395.1	uncultured phage MedDCM-OCT-S08-C1731
743575	GCA_003440435.1	uncultured phage MedDCM-OCT-S08-C233
743576	GCA_003441095.1	uncultured phage MedDCM-OCT-S08-C239
743577	GCA_003441115.1	uncultured phage MedDCM-OCT-S08-C304
743578	GCA_003441135.1	uncultured phage MedDCM-OCT-S08-C41
743579	GCA_003440455.1	uncultured phage MedDCM-OCT-S08-C495
743580	GCA_003440475.1	uncultured phage MedDCM-OCT-S08-C582
743582	GCA_003441155.1	uncultured phage MedDCM-OCT-S08-C964
743584	GCA_003440515.1	uncultured phage MedDCM-OCT-S09-C23
743586	GCA_003440555.1	uncultured phage MedDCM-OCT-S09-C299
743588	GCA_003440575.1	uncultured phage MedDCM-OCT-S09-C399
743590	GCA_003441235.1	uncultured phage MedDCM-OCT-S09-C7
743596	GCA_003440615.1	uncultured phage MedDCM-OCT-S12-C102
1074044	GCA_000954235.1	uncultured phage WW-nAnB
1449896	GCA_000955375.1	Uncultured phage WW-nAnB strain 2
1449897	GCA_000954655.1	Uncultured phage WW-nAnB strain 3

340016	GCA_002922265.1	uncultured virus
340016	GCA_002922245.1	uncultured virus
340016	GCA_002922215.1	uncultured virus
340016	GCA_002922275.1	uncultured virus
340016	GCA_002922285.1	uncultured virus
340016	GCA_002922255.1	uncultured virus
340016	GCA_002922295.1	uncultured virus
340016	GCA_002922225.1	uncultured virus
340016	GCA_002922235.1	uncultured virus
340016	GCA_002922205.1	uncultured virus
340016	GCA_003533105.1	uncultured virus
340016	GCA_003544355.1	uncultured virus
340016	GCA_003502045.1	uncultured virus
340016	GCA_003536935.1	uncultured virus
340016	GCA_003533145.1	uncultured virus
340016	GCA_003540925.1	uncultured virus
340016	GCA_003536685.1	uncultured virus
340016	GCA_003502065.1	uncultured virus
340016	GCA_003544075.1	uncultured virus
340016	GCA_003541005.1	uncultured virus
340016	GCA_003502085.1	uncultured virus
340016	GCA_003533165.1	uncultured virus
340016	GCA_003536135.1	uncultured virus
340016	GCA_003502105.1	uncultured virus
340016	GCA_003536305.1	uncultured virus
340016	GCA_003533185.1	uncultured virus
340016	GCA_003502125.1	uncultured virus
340016	GCA_003536415.1	uncultured virus
340016	GCA_003541035.1	uncultured virus
340016	GCA_003502145.1	uncultured virus
340016	GCA_003544475.1	uncultured virus
340016	GCA_003541055.1	uncultured virus
340016	GCA_003533215.1	uncultured virus
340016	GCA_003537295.1	uncultured virus
340016	GCA_003502165.1	uncultured virus
340016	GCA_003536975.1	uncultured virus
340016	GCA_003544495.1	uncultured virus
340016	GCA_003502185.1	uncultured virus
340016	GCA_003541075.1	uncultured virus
340016	GCA_003528285.1	uncultured virus
340016	GCA_003502225.1	uncultured virus
340016	GCA_003541105.1	uncultured virus
340016	GCA_003528765.1	uncultured virus
340016	GCA_003502245.1	uncultured virus
340016	GCA_003543975.1	uncultured virus
340016	GCA_003541155.1	uncultured virus
340016	GCA_003528165.1	uncultured virus
340016	GCA_003502265.1	uncultured virus
340016	GCA_003528345.1	uncultured virus
340016	GCA_003541195.1	uncultured virus

340016	GCA_003528465.1	uncultured virus
340016	GCA_003544035.1	uncultured virus
340016	GCA_003527865.1	uncultured virus
340016	GCA_003541215.1	uncultured virus
340016	GCA_003544515.1	uncultured virus
340016	GCA_003498225.1	uncultured virus
340016	GCA_003528395.1	uncultured virus
340016	GCA_003544235.1	uncultured virus
340016	GCA_003543955.1	uncultured virus
340016	GCA_003498245.1	uncultured virus
340016	GCA_003541235.1	uncultured virus
340016	GCA_003528435.1	uncultured virus
340016	GCA_003539995.1	uncultured virus
340016	GCA_003498265.1	uncultured virus
340016	GCA_003541255.1	uncultured virus
340016	GCA_003498285.1	uncultured virus
340016	GCA_003532255.1	uncultured virus
340016	GCA_003498305.1	uncultured virus
340016	GCA_003498325.1	uncultured virus
340016	GCA_003541305.1	uncultured virus
340016	GCA_003528505.1	uncultured virus
340016	GCA_003531705.1	uncultured virus
340016	GCA_003540535.1	uncultured virus
340016	GCA_003498345.1	uncultured virus
340016	GCA_003498365.1	uncultured virus
340016	GCA_003531735.1	uncultured virus
340016	GCA_003498385.1	uncultured virus
340016	GCA_003537355.1	uncultured virus
340016	GCA_003528525.1	uncultured virus
340016	GCA_003537035.1	uncultured virus
340016	GCA_003544255.1	uncultured virus
340016	GCA_003536745.1	uncultured virus
340016	GCA_003498405.1	uncultured virus
340016	GCA_003544335.1	uncultured virus
340016	GCA_003536435.1	uncultured virus
340016	GCA_003531755.1	uncultured virus
340016	GCA_003541395.1	uncultured virus
340016	GCA_003528545.1	uncultured virus
340016	GCA_003498425.1	uncultured virus
340016	GCA_003527905.1	uncultured virus
340016	GCA_003536525.1	uncultured virus
340016	GCA_003528565.1	uncultured virus
340016	GCA_003498445.1	uncultured virus
340016	GCA_003540965.1	uncultured virus
340016	GCA_003531805.1	uncultured virus
340016	GCA_003498465.1	uncultured virus
340016	GCA_003528185.1	uncultured virus
340016	GCA_003531855.1	uncultured virus
340016	GCA_003498505.1	uncultured virus
340016	GCA_003544375.1	uncultured virus

340016	GCA_003541355.1	uncultured virus
340016	GCA_003540075.1	uncultured virus
340016	GCA_003537085.1	uncultured virus
340016	GCA_003528645.1	uncultured virus
340016	GCA_003498525.1	uncultured virus
340016	GCA_003544275.1	uncultured virus
340016	GCA_003544095.1	uncultured virus
340016	GCA_003531885.1	uncultured virus
340016	GCA_003498545.1	uncultured virus
340016	GCA_003544435.1	uncultured virus
340016	GCA_003540355.1	uncultured virus
340016	GCA_003540795.1	uncultured virus
340016	GCA_003498565.1	uncultured virus
340016	GCA_003531915.1	uncultured virus
340016	GCA_003498585.1	uncultured virus
340016	GCA_003544155.1	uncultured virus
340016	GCA_003536715.1	uncultured virus
340016	GCA_003528205.1	uncultured virus
340016	GCA_003498605.1	uncultured virus
340016	GCA_003531945.1	uncultured virus
340016	GCA_003498625.1	uncultured virus
340016	GCA_003544455.1	uncultured virus
340016	GCA_003498645.1	uncultured virus
340016	GCA_003536455.1	uncultured virus
340016	GCA_003536105.1	uncultured virus
340016	GCA_003544015.1	uncultured virus
340016	GCA_003531985.1	uncultured virus
340016	GCA_003498665.1	uncultured virus
340016	GCA_003528785.1	uncultured virus
340016	GCA_003499045.1	uncultured virus
340016	GCA_003543935.1	uncultured virus
340016	GCA_003498685.1	uncultured virus
340016	GCA_003528805.1	uncultured virus
340016	GCA_003532025.1	uncultured virus
340016	GCA_003498705.1	uncultured virus
340016	GCA_003536205.1	uncultured virus
340016	GCA_003498725.1	uncultured virus
340016	GCA_003532055.1	uncultured virus
340016	GCA_003498745.1	uncultured virus
340016	GCA_003536835.1	uncultured virus
340016	GCA_003528885.1	uncultured virus
340016	GCA_003540015.1	uncultured virus
340016	GCA_003532075.1	uncultured virus
340016	GCA_003536795.1	uncultured virus
340016	GCA_003498765.1	uncultured virus
340016	GCA_003536485.1	uncultured virus
340016	GCA_003498785.1	uncultured virus
340016	GCA_003544175.1	uncultured virus
340016	GCA_003528905.1	uncultured virus
340016	GCA_003498805.1	uncultured virus

340016	GCA_003541375.1	uncultured virus
340016	GCA_003532105.1	uncultured virus
340016	GCA_003539955.1	uncultured virus
340016	GCA_003498825.1	uncultured virus
340016	GCA_003544315.1	uncultured virus
340016	GCA_003528935.1	uncultured virus
340016	GCA_003536635.1	uncultured virus
340016	GCA_003498845.1	uncultured virus
340016	GCA_003532135.1	uncultured virus
340016	GCA_003528965.1	uncultured virus
340016	GCA_003498865.1	uncultured virus
340016	GCA_003537395.1	uncultured virus
340016	GCA_003532165.1	uncultured virus
340016	GCA_003528835.1	uncultured virus
340016	GCA_003498885.1	uncultured virus
340016	GCA_003540195.1	uncultured virus
340016	GCA_003498905.1	uncultured virus
340016	GCA_003532215.1	uncultured virus
340016	GCA_003544395.1	uncultured virus
340016	GCA_003498925.1	uncultured virus
340016	GCA_003532235.1	uncultured virus
340016	GCA_003498945.1	uncultured virus
340016	GCA_003536175.1	uncultured virus
340016	GCA_003502205.1	uncultured virus
340016	GCA_003498965.1	uncultured virus
340016	GCA_003536995.1	uncultured virus
340016	GCA_003540045.1	uncultured virus
340016	GCA_003498985.1	uncultured virus
340016	GCA_003537415.1	uncultured virus
340016	GCA_003540095.1	uncultured virus
340016	GCA_003537135.1	uncultured virus
340016	GCA_003499005.1	uncultured virus
340016	GCA_003532295.1	uncultured virus
340016	GCA_003499025.1	uncultured virus
340016	GCA_003540115.1	uncultured virus
340016	GCA_003537115.1	uncultured virus
340016	GCA_003540165.1	uncultured virus
340016	GCA_003536235.1	uncultured virus
340016	GCA_003532315.1	uncultured virus
340016	GCA_003499065.1	uncultured virus
340016	GCA_003540515.1	uncultured virus
340016	GCA_003544055.1	uncultured virus
340016	GCA_003499085.1	uncultured virus
340016	GCA_003532355.1	uncultured virus
340016	GCA_003499105.1	uncultured virus
340016	GCA_003537435.1	uncultured virus
340016	GCA_003536955.1	uncultured virus
340016	GCA_003532395.1	uncultured virus
340016	GCA_003499125.1	uncultured virus
340016	GCA_003532425.1	uncultured virus

340016	GCA_003544115.1	uncultured virus
340016	GCA_003540215.1	uncultured virus
340016	GCA_003499145.1	uncultured virus
340016	GCA_003537155.1	uncultured virus
340016	GCA_003540255.1	uncultured virus
340016	GCA_003531775.1	uncultured virus
340016	GCA_003499165.1	uncultured virus
340016	GCA_003537185.1	uncultured virus
340016	GCA_003536875.1	uncultured virus
340016	GCA_003540275.1	uncultured virus
340016	GCA_003528315.1	uncultured virus
340016	GCA_003499205.1	uncultured virus
340016	GCA_003543995.1	uncultured virus
340016	GCA_003540295.1	uncultured virus
340016	GCA_003532475.1	uncultured virus
340016	GCA_003537255.1	uncultured virus
340016	GCA_003536775.1	uncultured virus
340016	GCA_003532695.1	uncultured virus
340016	GCA_003540315.1	uncultured virus
340016	GCA_003532455.1	uncultured virus
340016	GCA_003536255.1	uncultured virus
340016	GCA_003532495.1	uncultured virus
340016	GCA_003532535.1	uncultured virus
340016	GCA_003537335.1	uncultured virus
340016	GCA_003536595.1	uncultured virus
340016	GCA_003544535.1	uncultured virus
340016	GCA_003540385.1	uncultured virus
340016	GCA_003544295.1	uncultured virus
340016	GCA_003532585.1	uncultured virus
340016	GCA_003540435.1	uncultured virus
340016	GCA_003532615.1	uncultured virus
340016	GCA_003528605.1	uncultured virus
340016	GCA_003540475.1	uncultured virus
340016	GCA_003537375.1	uncultured virus
340016	GCA_003532635.1	uncultured virus
340016	GCA_003536275.1	uncultured virus
340016	GCA_003540495.1	uncultured virus
340016	GCA_003536895.1	uncultured virus
340016	GCA_003532655.1	uncultured virus
340016	GCA_003528625.1	uncultured virus
340016	GCA_003532725.1	uncultured virus
340016	GCA_003543895.1	uncultured virus
340016	GCA_003540875.1	uncultured virus
340016	GCA_003540555.1	uncultured virus
340016	GCA_003540605.1	uncultured virus
340016	GCA_003536015.1	uncultured virus
340016	GCA_003528225.1	uncultured virus
340016	GCA_003532795.1	uncultured virus
340016	GCA_003537215.1	uncultured virus
340016	GCA_003527845.1	uncultured virus

340016	GCA_003536565.1	uncultured virus
340016	GCA_003532825.1	uncultured virus
340016	GCA_003540695.1	uncultured virus
340016	GCA_003536615.1	uncultured virus
340016	GCA_003532855.1	uncultured virus
340016	GCA_003528365.1	uncultured virus
340016	GCA_003532875.1	uncultured virus
340016	GCA_003544415.1	uncultured virus
340016	GCA_003540715.1	uncultured virus
340016	GCA_003537235.1	uncultured virus
340016	GCA_003527945.1	uncultured virus
340016	GCA_003532905.1	uncultured virus
340016	GCA_003544135.1	uncultured virus
340016	GCA_003544195.1	uncultured virus
340016	GCA_003536355.1	uncultured virus
340016	GCA_003540745.1	uncultured virus
340016	GCA_003527985.1	uncultured virus
340016	GCA_003532945.1	uncultured virus
340016	GCA_003540835.1	uncultured virus
340016	GCA_003540895.1	uncultured virus
340016	GCA_003528685.1	uncultured virus
340016	GCA_003532975.1	uncultured virus
340016	GCA_003528005.1	uncultured virus
340016	GCA_003540855.1	uncultured virus
340016	GCA_003532775.1	uncultured virus
340016	GCA_003533005.1	uncultured virus
340016	GCA_003528985.1	uncultured virus
340016	GCA_003528025.1	uncultured virus
340016	GCA_003544215.1	uncultured virus
340016	GCA_003540655.1	uncultured virus
340016	GCA_003533045.1	uncultured virus
340016	GCA_003528055.1	uncultured virus
340016	GCA_003543915.1	uncultured virus
340016	GCA_003540335.1	uncultured virus
340016	GCA_003528085.1	uncultured virus
340016	GCA_003540675.1	uncultured virus
340016	GCA_003533065.1	uncultured virus
340016	GCA_003536045.1	uncultured virus
340016	GCA_003528735.1	uncultured virus
340016	GCA_003528585.1	uncultured virus
340016	GCA_003501995.1	uncultured virus
340016	GCA_003536395.1	uncultured virus
340016	GCA_003528145.1	uncultured virus
340016	GCA_003502025.1	uncultured virus
340016	GCA_003536075.1	uncultured virus
1864484	GCA_001678295.1	Ungulate bocaparvovirus 6
1511914	GCA_001430475.1	Ungulate tetraparvovirus 1
1511916	GCA_002219485.1	Ungulate tetraparvovirus 3
1862826	GCA_001678815.1	unidentified circular ssDNA virus
1862826	GCA_003673105.1	unidentified circular ssDNA virus

1862826	GCA_003673525.1	unidentified circular ssDNA virus
1862826	GCA_003673125.1	unidentified circular ssDNA virus
1862826	GCA_003673325.1	unidentified circular ssDNA virus
1862826	GCA_003673465.1	unidentified circular ssDNA virus
1862826	GCA_003673085.1	unidentified circular ssDNA virus
1862826	GCA_003673265.1	unidentified circular ssDNA virus
1862826	GCA_003673065.1	unidentified circular ssDNA virus
1862826	GCA_003673545.1	unidentified circular ssDNA virus
1862826	GCA_003673345.1	unidentified circular ssDNA virus
1862826	GCA_003673485.1	unidentified circular ssDNA virus
1862826	GCA_003673565.1	unidentified circular ssDNA virus
1862826	GCA_003673165.1	unidentified circular ssDNA virus
1862826	GCA_003673305.1	unidentified circular ssDNA virus
1862826	GCA_003673245.1	unidentified circular ssDNA virus
694448	GCA_002889935.1	unidentified human coronavirus
38018	GCA_900291425.1	unidentified phage
38018	GCA_900327785.1	unidentified phage
38018	GCA_900327795.1	unidentified phage
38018	GCA_900327505.1	unidentified phage
38018	GCA_900291435.1	unidentified phage
38018	GCA_900291295.1	unidentified phage
38018	GCA_900291395.1	unidentified phage
38018	GCA_900291235.1	unidentified phage
38018	GCA_900327465.1	unidentified phage
38018	GCA_900327485.1	unidentified phage
38018	GCA_900291445.1	unidentified phage
38018	GCA_900291135.1	unidentified phage
38018	GCA_900291385.1	unidentified phage
38018	GCA_900327765.1	unidentified phage
38018	GCA_900327445.1	unidentified phage
38018	GCA_900291305.1	unidentified phage
38018	GCA_900327515.1	unidentified phage
38018	GCA_900604365.1	unidentified phage
38018	GCA_900291245.1	unidentified phage
38018	GCA_900327455.1	unidentified phage
38018	GCA_900291355.1	unidentified phage
38018	GCA_900291325.1	unidentified phage
38018	GCA_900291315.1	unidentified phage
38018	GCA_900291285.1	unidentified phage
38018	GCA_900291045.1	unidentified phage
38018	GCA_900291055.1	unidentified phage
38018	GCA_900291335.1	unidentified phage
38018	GCA_900327745.1	unidentified phage
38018	GCA_900327475.1	unidentified phage
38018	GCA_900291145.1	unidentified phage
38018	GCA_900327525.1	unidentified phage
38018	GCA_900291195.1	unidentified phage
38018	GCA_900291215.1	unidentified phage
38018	GCA_900291125.1	unidentified phage
38018	GCA_900291175.1	unidentified phage

38018	GCA_900291375.1	unidentified phage
38018	GCA_900327595.1	unidentified phage
38018	GCA_900327565.1	unidentified phage
38018	GCA_900014825.1	unidentified phage
38018	GCA_900291165.1	unidentified phage
38018	GCA_900291185.1	unidentified phage
38018	GCA_900291255.1	unidentified phage
38018	GCA_900327545.1	unidentified phage
1214906	GCA_900243475.1	unidentified virus
1672385	GCA_003032595.1	University of Giessen virus
1382279	GCA_000918855.1	University of Helsinki virus
354090	GCA_000862205.1	UR2 sarcoma virus
1675866	GCA_002157455.1	Urbanus proteus nucleopolyhedrovirus
1055750	GCA_002008775.1	Uriurana virus
520848	GCA_000874425.1	Urochloa streak virus
461322	GCA_000874405.1	Ursus maritimus papillomavirus 1
1926502	GCA_002008595.1	Urucuri virus
2305465	GCA_004134725.1	Ustilaginoidea virens nonsegmented virus 2
1415665	GCA_000908155.1	Ustilaginoidea virens partitivirus 2
1312445	GCA_000904675.1	Ustilaginoidea virens RNA virus 1
1460374	GCA_000916155.1	Ustilaginoidea virens RNA virus 3
1756615	GCA_001461665.1	Ustilaginoidea virens RNA virus 5
1561171	GCA_000925395.1	Ustilaginoidea virens RNA virus L
1561172	GCA_000924555.1	Ustilaginoidea virens RNA virus M
1670975	GCA_001045365.1	Ustilaginoidea virens unassigned RNA virus HNND-1
28882	GCA_000851465.1	Ustilago maydis virus H1
64286	GCA_000854945.1	Usutu virus
11591	GCA_000851865.1	Uukuniemi virus
10245	GCA_003703355.1	Vaccinia virus
10245	GCA_003971425.1	Vaccinia virus
10254	GCA_000860085.1	Vaccinia virus WR
10254	GCA_900236015.1	Vaccinia virus WR
1129873	GCA_000894535.1	Valsa ceratosperma hypovirus 1
326202	GCA_000926935.1	Vanilla distortion mosaic virus
2016426	GCA_002219605.1	Vanilla latent virus
186373	GCA_002828445.1	Vanilla mosaic virus
2016427	GCA_002219785.1	Vanilla virus X
1885248	GCA_001698295.1	Variegated squirrel bornavirus 1
1885248	GCA_002153825.1	Variegated squirrel bornavirus 1
1885248	GCA_002985965.1	Variegated squirrel bornavirus 1
1885248	GCA_900089145.1	Variegated squirrel bornavirus 1
1885248	GCA_900089215.1	Variegated squirrel bornavirus 1
1885248	GCA_900089365.1	Variegated squirrel bornavirus 1
1885248	GCA_900089205.1	Variegated squirrel bornavirus 1
1885248	GCA_900089185.1	Variegated squirrel bornavirus 1
1885248	GCA_900089425.1	Variegated squirrel bornavirus 1
1885248	GCA_900089305.1	Variegated squirrel bornavirus 1
1885248	GCA_900089435.1	Variegated squirrel bornavirus 1
1885248	GCA_900089335.1	Variegated squirrel bornavirus 1
1885248	GCA_900089395.1	Variegated squirrel bornavirus 1

10255	GCA_000859885.1	Variola virus (smallpox virus)
10255	GCA_900155315.1	Variola virus (smallpox virus)
10255	GCA_900155325.1	Variola virus (smallpox virus)
232800	GCA_000856945.1	Varroa destructor virus 1
2056384	GCA_004129835.1	Varroa destructor virus 2
2056385	GCA_003727455.1	Varroa destructor virus 3
2077299	GCA_002937255.1	Varroa mite associated genomovirus 1
2077300	GCA_002937265.1	Varroa mite associated virus 1
1682187	GCA_001188625.1	Varroa Tymo-like virus
1881630	GCA_003029325.1	Velvet bean golden mosaic virus
667119	GCA_000884375.1	Velvet bean severe mosaic virus
12473	GCA_000889275.1	Velvet tobacco mottle virus
196095	GCA_000839285.1	Velvet tobacco mottle virus Satellite RNA
11036	GCA_000862105.1	Venezuelan equine encephalitis virus
11036	GCA_002889775.1	Venezuelan equine encephalitis virus
11036	GCA_002888875.1	Venezuelan equine encephalitis virus
11036	GCA_002888755.1	Venezuelan equine encephalitis virus
11036	GCA_002889655.1	Venezuelan equine encephalitis virus
11036	GCA_002889695.1	Venezuelan equine encephalitis virus
515446	GCA_000875185.1	Verbena virus Y
945250	GCA_000892815.1	Verbesina encelioides leaf curl alphasatellite
1925152	GCA_001907845.1	Vernonia crinkle betasatellite
1925153	GCA_001907745.1	Vernonia crinkle virus
666635	GCA_000885995.1	Vernonia yellow vein betasatellite
2169908	GCA_000894775.1	Vernonia yellow vein Fujian alphasatellite
2169908	GCA_003028985.1	Vernonia yellow vein Fujian alphasatellite
2050589	GCA_000892915.1	Vernonia yellow vein Fujian betasatellite
367061	GCA_000864465.1	Vernonia yellow vein virus
1636271	GCA_001534595.1	Verrucomicrobia phage P8625
759389	GCA_002867345.1	Verticillium dahliae chrysovirus 1
1310424	GCA_001292935.1	Verticillium dahliae partitivirus 1
35612	GCA_000847705.1	Vesicular exanthema of swine virus
198833	GCA_000924515.1	Vesicular stomatitis Alagoas virus
11277	GCA_000850045.1	Vesicular stomatitis Indiana virus
11277	GCA_002815995.1	Vesicular stomatitis Indiana virus
11280	GCA_000923855.1	Vesicular stomatitis New Jersey virus
11280	GCA_002816035.1	Vesicular stomatitis New Jersey virus
1615583	GCA_001019935.1	Vesivirus ferret badger/JX12/China/2012
187944	GCA_002611905.1	Vibrio phage 1
1881287	GCA_003926115.1	Vibrio phage 1.003.O._10N.286.48.A2
1881288	GCA_003926135.1	Vibrio phage 1.004.O._10N.261.54.A2
1881414	GCA_003926155.1	Vibrio phage 1.005.O._10N.286.48.F2
1881241	GCA_003926175.1	Vibrio phage 1.007.O._10N.261.55.F9
1881242	GCA_003926195.1	Vibrio phage 1.008.O._10N.286.54.E5
1881289	GCA_003926215.1	Vibrio phage 1.009.O._10N.261.51.C9
1881243	GCA_003926235.1	Vibrio phage 1.011.O._10N.286.49.B11
1881290	GCA_003926255.1	Vibrio phage 1.012.O._10N.261.48.C12
1881244	GCA_003926275.1	Vibrio phage 1.013.O._10N.286.54.F9
1881188	GCA_003926295.1	Vibrio phage 1.015.O._10N.222.51.E5
1881291	GCA_003926315.1	Vibrio phage 1.016.O._10N.286.46.A11

1881397	GCA_003926335.1	Vibrio phage 1.017.O._10N.286.55.C11
1881450	GCA_003926355.1	Vibrio phage 1.020.O._10N.222.48.A2
1881415	GCA_003926375.1	Vibrio phage 1.021.A._10N.222.51.F9
1881416	GCA_003926395.1	Vibrio phage 1.021.B._10N.222.51.F9
1881417	GCA_003926415.1	Vibrio phage 1.021.C._10N.222.51.F9
1881418	GCA_003926435.1	Vibrio phage 1.022.O._10N.286.45.A10
1881419	GCA_003926455.1	Vibrio phage 1.023.O._10N.222.51.B4
1881292	GCA_003926475.1	Vibrio phage 1.024.O._10N.261.45.F8
1881420	GCA_003926495.1	Vibrio phage 1.025.O._10N.222.46.B6
1881421	GCA_003926515.1	Vibrio phage 1.026.O._10N.222.49.C7
1881293	GCA_003926535.1	Vibrio phage 1.027.O._10N.286.54.B8
1881380	GCA_003926555.1	Vibrio phage 1.028.O._10N.286.45.B6
1881381	GCA_003926575.1	Vibrio phage 1.029.O._10N.261.55.A7
1881407	GCA_003926595.1	Vibrio phage 1.030.O._10N.222.55.F9
1881294	GCA_003926615.1	Vibrio phage 1.031.O._10N.261.46.F8
1881422	GCA_003926635.1	Vibrio phage 1.032.O._10N.261.54.F5
1881295	GCA_003926655.1	Vibrio phage 1.033.O._10N.222.49.B8
1881189	GCA_003926675.1	Vibrio phage 1.034.O._10N.261.46.B7
1881190	GCA_003926695.1	Vibrio phage 1.034.X._10N.261.46.B7
1881296	GCA_003926715.1	Vibrio phage 1.036.O._10N.286.45.C3
1881297	GCA_003926735.1	Vibrio phage 1.037.O._10N.261.52.F7
1881298	GCA_003926755.1	Vibrio phage 1.038.O._10N.286.51.C2
1881299	GCA_003926775.1	Vibrio phage 1.039.O._10N.286.55.A2
1881300	GCA_003926795.1	Vibrio phage 1.040.O._10N.286.45.B9
1881301	GCA_003926815.1	Vibrio phage 1.042.O._10N.286.45.B8
1881302	GCA_003926835.1	Vibrio phage 1.043.O._10N.261.52.C7
1881303	GCA_003926855.1	Vibrio phage 1.044.O._10N.261.51.B8
1881423	GCA_003926875.1	Vibrio phage 1.046.O._10N.286.52.E3
1881245	GCA_003926895.1	Vibrio phage 1.047.O._10N.286.55.F2
1881304	GCA_003926915.1	Vibrio phage 1.048.O._10N.286.46.A10
2070715	GCA_003344225.1	Vibrio phage 1.049.O._10N.286.54.B5
2070716	GCA_003344245.1	Vibrio phage 1.050.O._10N.286.48.A6
2070717	GCA_003926935.1	Vibrio phage 1.052.A._10N.286.46.C3
1881382	GCA_003926955.1	Vibrio phage 1.054.O._10N.261.52.A1
1881305	GCA_003926975.1	Vibrio phage 1.056.O._10N.261.48.C11
1881306	GCA_003926995.1	Vibrio phage 1.057.O._10N.261.46.B12
1881449	GCA_003927015.1	Vibrio phage 1.060.A._10N.261.48.B5
1881307	GCA_003927035.1	Vibrio phage 1.061.O._10N.286.55.C2
1881246	GCA_003927055.1	Vibrio phage 1.062.O._10N.286.55.C3
1881308	GCA_003927075.1	Vibrio phage 1.063.O._10N.261.45.C7
1881455	GCA_003927095.1	Vibrio phage 1.064.O._10N.261.52.E2
1881309	GCA_003852975.1	Vibrio phage 1.066.O._10N.286.46.E8
1881310	GCA_003927115.1	Vibrio phage 1.067.O._10N.261.52.C9
1881311	GCA_003852955.1	Vibrio phage 1.068.O._10N.261.51.F8
1881312	GCA_003927135.1	Vibrio phage 1.069.O._10N.286.49.F11
2070719	GCA_003927155.1	Vibrio phage 1.070.O._10N.261.45.B2
1881313	GCA_003927175.1	Vibrio phage 1.071.A._10N.286.46.A12
1881456	GCA_003852935.1	Vibrio phage 1.072.O._10N.286.48.A12
1881191	GCA_003927195.1	Vibrio phage 1.074.O._10N.222.49.B7
1881398	GCA_003927215.1	Vibrio phage 1.075.O._10N.286.55.B10

2070720	GCA_003927235.1	Vibrio phage 1.076.O._10N.286.51.B7
2070721	GCA_003927255.1	Vibrio phage 1.077.O._10N.261.45.A10
1881314	GCA_003927275.1	Vibrio phage 1.079.O._10N.286.45.E9
1881315	GCA_003927295.1	Vibrio phage 1.080.O._10N.286.48.A4
2070722	GCA_003927315.1	Vibrio phage 1.081.O._10N.286.52.C2
1881192	GCA_003927335.1	Vibrio phage 1.082.O._10N.261.49.E4
2070723	GCA_003344305.1	Vibrio phage 1.083.O._10N.286.52.B9
2070724	GCA_003927355.1	Vibrio phage 1.084.O._10N.261.49.F5
1881193	GCA_003927375.1	Vibrio phage 1.085.O._10N.222.51.E3
1881424	GCA_003927395.1	Vibrio phage 1.086.O._10N.222.51.F8
1881316	GCA_003927415.1	Vibrio phage 1.087.A._10N.261.45.F9
1881317	GCA_003927435.1	Vibrio phage 1.088.O._10N.261.46.A1
1881318	GCA_003927455.1	Vibrio phage 1.089.O._10N.261.51.F9
1881319	GCA_003927475.1	Vibrio phage 1.090.B._10N.286.48.F1
1881320	GCA_003927495.1	Vibrio phage 1.091.O._10N.286.52.B12
1881399	GCA_003927515.1	Vibrio phage 1.093.O._10N.286.55.E10
1881400	GCA_003927535.1	Vibrio phage 1.094.O._10N.286.55.E12
1881401	GCA_003927555.1	Vibrio phage 1.095.O._10N.286.46.E10
1881321	GCA_003927575.1	Vibrio phage 1.098.O._10N.286.51.B9
1881322	GCA_003927595.1	Vibrio phage 1.100.O._10N.261.45.C3
2070725	GCA_003927615.1	Vibrio phage 1.101.O._10N.261.45.C6
1881323	GCA_003927635.1	Vibrio phage 1.102.O._10N.261.45.E3
1881425	GCA_003927655.1	Vibrio phage 1.103.O._10N.261.52.F2
1881324	GCA_003927675.1	Vibrio phage 1.104.O._10N.286.49.A12
1881247	GCA_003927695.1	Vibrio phage 1.105.O._10N.286.49.B4
1881248	GCA_003927715.1	Vibrio phage 1.106.O._10N.286.51.F7
1881325	GCA_003927735.1	Vibrio phage 1.107.A._10N.286.52.E10
1881326	GCA_003927755.1	Vibrio phage 1.107.B._10N.286.52.E10
1881327	GCA_003927775.1	Vibrio phage 1.107.C._10N.286.52.E10
1881194	GCA_003927795.1	Vibrio phage 1.108.O._10N.222.51.A4
1881195	GCA_003927815.1	Vibrio phage 1.110.O._10N.261.52.C1
1881328	GCA_003927835.1	Vibrio phage 1.111.A._10N.286.45.E6
1881329	GCA_003927855.1	Vibrio phage 1.111.B._10N.286.45.E6
1881330	GCA_003927875.1	Vibrio phage 1.112.O._10N.286.46.B11
1881426	GCA_003927895.1	Vibrio phage 1.113.A._10N.286.51.E7
1881196	GCA_003927915.1	Vibrio phage 1.115.A._10N.222.49.B11
1881197	GCA_003927935.1	Vibrio phage 1.115.B._10N.222.49.B11
1881198	GCA_003927955.1	Vibrio phage 1.116.O._10N.222.52.C10
1881199	GCA_003927975.1	Vibrio phage 1.117.O._10N.261.45.E9
1881331	GCA_003927995.1	Vibrio phage 1.118.A._10N.261.49.F6
1881332	GCA_003928015.1	Vibrio phage 1.118.B._10N.261.49.F6
1881333	GCA_003928035.1	Vibrio phage 1.119.O._10N.261.51.A9
2070726	GCA_003928055.1	Vibrio phage 1.121.O._10N.286.46.C4
1881249	GCA_003928075.1	Vibrio phage 1.122.A._10N.286.46.F8
1881250	GCA_003928095.1	Vibrio phage 1.122.B._10N.286.46.F8
2070727	GCA_003928115.1	Vibrio phage 1.123.O._10N.286.48.F3
1881384	GCA_003928135.1	Vibrio phage 1.124.O._10N.286.49.B1
1881427	GCA_003928155.1	Vibrio phage 1.125.O._10N.286.49.F5
1881334	GCA_003928175.1	Vibrio phage 1.127.O._10N.286.52.E12
1881200	GCA_003928195.1	Vibrio phage 1.131.O._10N.222.49.A8

1881201	GCA_003928215.1	Vibrio phage 1.132.O._10N.222.49.F8
1881202	GCA_003928235.1	Vibrio phage 1.133.O._10N.222.51.E4
1881203	GCA_003928255.1	Vibrio phage 1.134.O._10N.222.52.B8
1881408	GCA_003928275.1	Vibrio phage 1.135.O._10N.222.54.B6
1881385	GCA_003928295.1	Vibrio phage 1.136.O._10N.261.45.E11
1881335	GCA_003928315.1	Vibrio phage 1.137.O._10N.261.46.B5
1881336	GCA_003928335.1	Vibrio phage 1.138.O._10N.261.48.A1
1881204	GCA_003928355.1	Vibrio phage 1.139.A._10N.261.48.C6
1881205	GCA_003928375.1	Vibrio phage 1.139.B._10N.261.48.C6
1881286	GCA_003928395.1	Vibrio phage 1.141.A._10N.261.49.B3
1881386	GCA_003928415.1	Vibrio phage 1.142.O._10N.261.49.E11
1881337	GCA_003928435.1	Vibrio phage 1.143.O._10N.261.55.C8
1881428	GCA_003928455.1	Vibrio phage 1.144.O._10N.286.45.B3
1881206	GCA_003928475.1	Vibrio phage 1.147.O._10N.286.49.E9
1881207	GCA_003928495.1	Vibrio phage 1.148.O._10N.286.54.A10
1881251	GCA_003928515.1	Vibrio phage 1.149.O._10N.286.55.A12
1881429	GCA_003928535.1	Vibrio phage 1.150.O._10N.222.46.A6
1881430	GCA_003928555.1	Vibrio phage 1.151.O._10N.222.46.B1
1881431	GCA_003928575.1	Vibrio phage 1.152.O._10N.222.46.E1
1881405	GCA_003928595.1	Vibrio phage 1.155.O._10N.222.55.B3
1881388	GCA_003928615.1	Vibrio phage 1.156.O._10N.261.45.A6
1881208	GCA_003928635.1	Vibrio phage 1.157.O._10N.261.45.B7
1881338	GCA_003928655.1	Vibrio phage 1.158.O._10N.261.45.E12
1881389	GCA_003928675.1	Vibrio phage 1.159.O._10N.261.46.F12
1881339	GCA_003928695.1	Vibrio phage 1.160.O._10N.261.48.B11
1881209	GCA_003928715.1	Vibrio phage 1.162.O._10N.261.48.E3
1881409	GCA_003928735.1	Vibrio phage 1.164.O._10N.261.51.A7
1881210	GCA_003928755.1	Vibrio phage 1.165.O._10N.261.51.B7
1881341	GCA_003928775.1	Vibrio phage 1.166.O._10N.261.51.C7
1881211	GCA_003928795.1	Vibrio phage 1.167.O._10N.261.51.F2
1881212	GCA_003928815.1	Vibrio phage 1.168.O._10N.261.52.A10
1881213	GCA_003928835.1	Vibrio phage 1.169.O._10N.261.52.B1
1881390	GCA_003928855.1	Vibrio phage 1.170.O._10N.261.52.C3
1881342	GCA_003928875.1	Vibrio phage 1.171.O._10N.261.52.F12
1881214	GCA_003928895.1	Vibrio phage 1.172.O._10N.261.52.F5
1881252	GCA_003928915.1	Vibrio phage 1.173.O._10N.261.55.A11
1881343	GCA_003928935.1	Vibrio phage 1.174.O._10N.261.55.A8
1881344	GCA_003928955.1	Vibrio phage 1.175.O._10N.261.55.B3
1881215	GCA_003928975.1	Vibrio phage 1.176.O._10N.261.55.F5
1881402	GCA_003928995.1	Vibrio phage 1.177.O._10N.286.45.E10
1881253	GCA_003929015.1	Vibrio phage 1.178.O._10N.286.45.E12
1881432	GCA_003929035.1	Vibrio phage 1.179.O._10N.286.45.F12
1881433	GCA_003929055.1	Vibrio phage 1.181.O._10N.286.46.C9
1881216	GCA_003929075.1	Vibrio phage 1.182.O._10N.286.46.E1
1881391	GCA_003929095.1	Vibrio phage 1.183.O._10N.286.48.B7
1881254	GCA_003929115.1	Vibrio phage 1.184.A._10N.286.49.A5
1881392	GCA_003929135.1	Vibrio phage 1.185.O._10N.286.49.C2
1881255	GCA_003929155.1	Vibrio phage 1.186.O._10N.286.49.E3
1881434	GCA_003344105.1	Vibrio phage 1.187.O._10N.286.49.F1
1881217	GCA_003929175.1	Vibrio phage 1.188.A._10N.286.51.A6

1881218	GCA_003929195.1	Vibrio phage 1.188.B._10N.286.51.A6
1881219	GCA_003929215.1	Vibrio phage 1.188.C._10N.286.51.A6
1881220	GCA_003929235.1	Vibrio phage 1.189.B._10N.286.51.B5
1881221	GCA_003929255.1	Vibrio phage 1.189.C._10N.286.51.B5
1881222	GCA_003929275.1	Vibrio phage 1.189.O._10N.286.51.B5
1881345	GCA_003929295.1	Vibrio phage 1.190.O._10N.286.51.F12
1881435	GCA_003929315.1	Vibrio phage 1.191.O._10N.286.52.B4
1881436	GCA_003344145.1	Vibrio phage 1.193.O._10N.286.52.C6
1881346	GCA_003929335.1	Vibrio phage 1.194.O._10N.286.54.B1
1881347	GCA_003929355.1	Vibrio phage 1.195.O._10N.286.54.C8
1881348	GCA_003929375.1	Vibrio phage 1.196.O._10N.286.54.E12
1881256	GCA_003929395.1	Vibrio phage 1.197.A._10N.286.54.F2
1881257	GCA_003929415.1	Vibrio phage 1.198.A._10N.286.54.F4
1881258	GCA_003929435.1	Vibrio phage 1.198.B._10N.286.54.F4
1881259	GCA_003929455.1	Vibrio phage 1.199.A._10N.286.55.C10
1881260	GCA_003929475.1	Vibrio phage 1.199.B._10N.286.55.C10
1881349	GCA_003929495.1	Vibrio phage 1.200.O._10N.286.55.E1
1881261	GCA_003929515.1	Vibrio phage 1.201.B._10N.286.55.F1
1881262	GCA_003597575.1	Vibrio phage 1.202.O._10N.222.45.E8
1881263	GCA_003929535.1	Vibrio phage 1.204.O._10N.222.46.F12
1881451	GCA_003929555.1	Vibrio phage 1.205.O._10N.222.51.A7
1881223	GCA_003929575.1	Vibrio phage 1.206.O._10N.222.51.B10
1881224	GCA_003929595.1	Vibrio phage 1.207.B._10N.222.51.C2
1881437	GCA_003929615.1	Vibrio phage 1.208.B._10N.222.52.A7
1881225	GCA_003929635.1	Vibrio phage 1.209.O._10N.222.52.B2
1881406	GCA_003929655.1	Vibrio phage 1.210.O._10N.222.52.C2
1881452	GCA_003929675.1	Vibrio phage 1.211.A._10N.222.52.F11
1881453	GCA_003929695.1	Vibrio phage 1.211.B._10N.222.52.F11
1881410	GCA_003929715.1	Vibrio phage 1.213.O._10N.222.54.F10
1881264	GCA_003929735.1	Vibrio phage 1.214.O._10N.222.54.F11
1881438	GCA_003929755.1	Vibrio phage 1.215.A._10N.222.54.F7
1881439	GCA_003929775.1	Vibrio phage 1.215.B._10N.222.54.F7
1881411	GCA_003929795.1	Vibrio phage 1.216.O._10N.222.55.C12
1881393	GCA_003929815.1	Vibrio phage 1.217.O._10N.261.45.A1
1881394	GCA_003929835.1	Vibrio phage 1.219.O._10N.261.45.E2
1881350	GCA_003929855.1	Vibrio phage 1.223.O._10N.261.48.A9
1881226	GCA_003929875.1	Vibrio phage 1.224.A._10N.261.48.B1
1881440	GCA_003929895.1	Vibrio phage 1.225.O._10N.261.48.B7
1881227	GCA_003929915.1	Vibrio phage 1.226.O._10N.261.48.E5
1881228	GCA_003929935.1	Vibrio phage 1.228.O._10N.261.49.C1
1881351	GCA_003929955.1	Vibrio phage 1.231.O._10N.261.49.F8
1881229	GCA_003929995.1	Vibrio phage 1.233.A._10N.261.51.E6
1881230	GCA_003930015.1	Vibrio phage 1.233.B._10N.261.51.E6
1881352	GCA_003930035.1	Vibrio phage 1.235.O._10N.261.52.B2
1881265	GCA_003930055.1	Vibrio phage 1.236.O._10N.261.52.C4
1881353	GCA_003930075.1	Vibrio phage 1.237.A._10N.261.52.C5
1881354	GCA_003930095.1	Vibrio phage 1.237.B._10N.261.52.C5
1881231	GCA_003930115.1	Vibrio phage 1.238.A._10N.261.52.F10
1881232	GCA_003930135.1	Vibrio phage 1.238.B._10N.261.52.F10
1881233	GCA_003930155.1	Vibrio phage 1.239.O._10N.261.52.F6

1881234	GCA_003930175.1	Vibrio phage 1.240.O._10N.261.52.F8
1881235	GCA_003930195.1	Vibrio phage 1.242.O._10N.261.54.B2
1881266	GCA_003930215.1	Vibrio phage 1.243.O._10N.261.54.B5
1881412	GCA_003930235.1	Vibrio phage 1.244.A._10N.261.54.C3
1881236	GCA_003930255.1	Vibrio phage 1.245.O._10N.261.54.C7
1881413	GCA_003930275.1	Vibrio phage 1.246.O._10N.261.54.E10
1881441	GCA_003930295.1	Vibrio phage 1.247.A._10N.261.54.E12
1881442	GCA_003930315.1	Vibrio phage 1.247.B._10N.261.54.E12
1881267	GCA_003930335.1	Vibrio phage 1.248.O._10N.261.54.F1
1881268	GCA_003930355.1	Vibrio phage 1.249.A._10N.261.55.B9
1881269	GCA_003930375.1	Vibrio phage 1.249.B._10N.261.55.B9
1881443	GCA_003930395.1	Vibrio phage 1.250.O._10N.261.55.E11
1881355	GCA_003930415.1	Vibrio phage 1.251.O._10N.261.55.E5
1881270	GCA_003930435.1	Vibrio phage 1.253.O._10N.286.45.B12
1881356	GCA_003930455.1	Vibrio phage 1.254.O._10N.286.45.C8
1881444	GCA_003930475.1	Vibrio phage 1.255.O._10N.286.45.F1
1881271	GCA_003930495.1	Vibrio phage 1.256.O._10N.286.45.F8
1881445	GCA_003930515.1	Vibrio phage 1.257.O._10N.286.46.A4
1881446	GCA_003930535.1	Vibrio phage 1.259.O._10N.286.48.F4
1881237	GCA_003930555.1	Vibrio phage 1.261.O._10N.286.51.A7
1881238	GCA_003930575.1	Vibrio phage 1.262.O._10N.286.51.A9
1881272	GCA_003930595.1	Vibrio phage 1.263.A._10N.286.51.B1
1881273	GCA_003930615.1	Vibrio phage 1.263.B._10N.286.51.B1
1881447	GCA_003930635.1	Vibrio phage 1.264.O._10N.286.51.F2
1881274	GCA_003930655.1	Vibrio phage 1.265.O._10N.286.52.F6
1881239	GCA_003930675.1	Vibrio phage 1.266.O._10N.286.52.F9
1881357	GCA_003930695.1	Vibrio phage 1.267.O._10N.286.54.A1
1881358	GCA_003930715.1	Vibrio phage 1.268.A._10N.286.54.A11
1881359	GCA_003930735.1	Vibrio phage 1.268.B._10N.286.54.A11
1881360	GCA_003930755.1	Vibrio phage 1.269.O._10N.286.54.A6
1881361	GCA_003930775.1	Vibrio phage 1.270.A._10N.286.54.A8
1881362	GCA_003930795.1	Vibrio phage 1.270.B._10N.286.54.A8
1881363	GCA_003930815.1	Vibrio phage 1.271.A._10N.286.54.B4
1881364	GCA_003930835.1	Vibrio phage 1.271.B._10N.286.54.B4
1881365	GCA_003930855.1	Vibrio phage 1.272.O._10N.286.54.C4
1881275	GCA_003930875.1	Vibrio phage 1.273.O._10N.286.54.C7
1881366	GCA_003930895.1	Vibrio phage 1.274.O._10N.286.54.E1
1881276	GCA_003930915.1	Vibrio phage 1.275.O._10N.286.54.E11
1881277	GCA_003930935.1	Vibrio phage 1.276.O._10N.286.54.E4
1881367	GCA_003930955.1	Vibrio phage 1.277.A._10N.286.54.E7
1881368	GCA_003930975.1	Vibrio phage 1.277.B._10N.286.54.E7
1881278	GCA_003930995.1	Vibrio phage 1.278.O._10N.286.54.E8
1881369	GCA_003931015.1	Vibrio phage 1.280.O._10N.286.54.F6
1881370	GCA_003931035.1	Vibrio phage 1.281.O._10N.286.54.F7
1881371	GCA_003931055.1	Vibrio phage 1.282.A._10N.286.54.F8
1881372	GCA_003931075.1	Vibrio phage 1.283.A._10N.286.55.A1
1881373	GCA_003931095.1	Vibrio phage 1.283.B._10N.286.55.A1
1881374	GCA_003931115.1	Vibrio phage 1.283.C._10N.286.55.A1
1881279	GCA_003931135.1	Vibrio phage 1.284.A._10N.286.55.A5
1881375	GCA_003931155.1	Vibrio phage 1.285.O._10N.286.55.C12

1881280	GCA_003931175.1	Vibrio phage 1.286.O._10N.286.55.C4
1881281	GCA_003931195.1	Vibrio phage 1.287.O._10N.286.55.C7
1881282	GCA_003931215.1	Vibrio phage 1.289.A._10N.286.55.E8
1881283	GCA_003931235.1	Vibrio phage 1.291.O._10N.286.55.F6
1881284	GCA_003931255.1	Vibrio phage 1.293.O._10N.261.52.E1
754075	GCA_000905495.1	Vibrio phage 11895-B1
1881376	GCA_003852915.1	Vibrio phage 2.044.O._10N.261.51.B8
1881378	GCA_003931275.1	Vibrio phage 2.092.O._10N.286.52.B7
1881403	GCA_003931295.1	Vibrio phage 2.095.A._10N.286.46.E10
1881404	GCA_003931315.1	Vibrio phage 2.095.B._10N.286.46.E10
2070728	GCA_003931335.1	Vibrio phage 2.096.O._10N.286.48.B5
1881240	GCA_003931355.1	Vibrio phage 2.117.O._10N.261.45.E9
1881448	GCA_003931375.1	Vibrio phage 2.130.O._10N.222.46.C2
1881395	GCA_003931395.1	Vibrio phage 2.159.A._10N.261.46.F12
1881396	GCA_003931415.1	Vibrio phage 2.159.B._10N.261.46.F12
1881285	GCA_003931435.1	Vibrio phage 2.275.O._10N.286.54.E11
1500712	GCA_002601345.1	Vibrio phage 24
1873904	GCA_002611765.1	Vibrio phage 2E1
1881379	GCA_003931455.1	Vibrio phage 3.058.O._10N.286.46.B8
1777046	GCA_002608965.1	Vibrio phage 919TP
2070057	GCA_002958015.1	Vibrio phage Aphrodite1
2070058	GCA_002958025.1	Vibrio phage Ares1
1434127	GCA_002602145.1	Vibrio phage AS51
2060111	GCA_002957665.1	Vibrio phage Athena1
2283011	GCA_003441475.1	Vibrio phage BONAISHI
2570300	GCA_002957645.1	Vibrio phage Ceto
1458716	GCA_000918975.1	Vibrio phage CHOED
1558463	GCA_002599745.1	Vibrio phage CJY
1903259	GCA_002633255.1	Vibrio phage CKB-S1
1903260	GCA_002633285.1	Vibrio phage CKB-S2
1897745	GCA_002613745.1	Vibrio phage Cla
10689	GCA_000903155.1	Vibrio phage CP-T1
573171	GCA_000908255.1	Vibrio phage douglas 12A4
573172	GCA_001550745.1	Vibrio phage eugene 12A10
2315335	GCA_003832995.1	Vibrio phage fNo16
83201	GCA_000837925.1	Vibrio phage fs2
1558464	GCA_002599765.1	Vibrio phage H1
1673892	GCA_002606925.1	Vibrio phage H188
2268914	GCA_002599725.1	Vibrio phage H2 PGK-2017
2268915	GCA_002599785.1	Vibrio phage H2 SGB-2014
1965457	GCA_002620185.1	Vibrio phage H20
1558466	GCA_002599805.1	Vibrio phage H3
1965458	GCA_002620205.1	Vibrio phage H8
573173	GCA_000907195.1	Vibrio phage helene 12B3
573174	GCA_000906335.1	Vibrio phage henriette 12B8
1897746	GCA_002613765.1	Vibrio phage Her
979525	GCA_000893175.1	Vibrio phage ICP1
979526	GCA_002599565.1	Vibrio phage ICP1_2001_A
979527	GCA_002599585.1	Vibrio phage ICP1_2004_A
979528	GCA_002599545.1	Vibrio phage ICP1_2005_A

979529	GCA_002599525.1	Vibrio phage ICP1_2006_A
979530	GCA_002599505.1	Vibrio phage ICP1_2006_B
979531	GCA_002599485.1	Vibrio phage ICP1_2006_C
979532	GCA_002599465.1	Vibrio phage ICP1_2006_D
1296591	GCA_003443355.1	Vibrio phage ICP1_2006_E
1296592	GCA_003575525.1	Vibrio phage ICP1_2011_A
2302418	GCA_003575545.1	Vibrio phage ICP1_2011_B
2302419	GCA_003443375.1	Vibrio phage ICP1_2012_A
979533	GCA_000890655.1	Vibrio phage ICP2
979534	GCA_002758955.1	Vibrio phage ICP2_2006_A
1529057	GCA_002756355.1	Vibrio phage ICP2_2011_A
1529058	GCA_000921695.1	Vibrio phage ICP2_2013_A_Haiti
979535	GCA_000892295.1	Vibrio phage ICP3
979536	GCA_002758975.1	Vibrio phage ICP3_2007_A
979537	GCA_002758995.1	Vibrio phage ICP3_2008_A
979539	GCA_002759015.1	Vibrio phage ICP3_2009_B
1916097	GCA_002710095.1	Vibrio phage J14
1558467	GCA_001041475.1	Vibrio phage J2
1558468	GCA_002599825.1	Vibrio phage J3
1283071	GCA_000908755.1	Vibrio phage JA-1
573176	GCA_002710485.1	Vibrio phage jenny 12G5
1983592	GCA_002955065.1	Vibrio phage JSF1
1983593	GCA_002955075.1	Vibrio phage JSF10
1983594	GCA_002955085.1	Vibrio phage JSF11
1983595	GCA_002955095.1	Vibrio phage JSF12
1983596	GCA_002955105.1	Vibrio phage JSF13
1983597	GCA_002955115.1	Vibrio phage JSF14
1983598	GCA_002955125.1	Vibrio phage JSF15
1983599	GCA_002955135.1	Vibrio phage JSF17
1983600	GCA_002955145.1	Vibrio phage JSF18
1983601	GCA_002955155.1	Vibrio phage JSF2
1983602	GCA_002955165.1	Vibrio phage JSF20
1983603	GCA_002955175.1	Vibrio phage JSF23
1983604	GCA_002955185.1	Vibrio phage JSF24
2026085	GCA_002955545.1	Vibrio phage JSF25
1983605	GCA_002955195.1	Vibrio phage JSF27
1983606	GCA_002955205.1	Vibrio phage JSF28
1916111	GCA_002615685.1	Vibrio phage JSF3
1983607	GCA_002955215.1	Vibrio phage JSF30
1983608	GCA_002955225.1	Vibrio phage JSF31
1983609	GCA_002955235.1	Vibrio phage JSF32
1983610	GCA_002955245.1	Vibrio phage JSF33
1983611	GCA_002955255.1	Vibrio phage JSF34
1983612	GCA_002955265.1	Vibrio phage JSF35
1983613	GCA_002955275.1	Vibrio phage JSF36
1916110	GCA_002615665.1	Vibrio phage JSF4
1983614	GCA_002955285.1	Vibrio phage JSF5
1983615	GCA_002955295.1	Vibrio phage JSF6
1292086	GCA_002603305.1	Vibrio phage JSF7
1983616	GCA_002955305.1	Vibrio phage JSF9

1912325	GCA_002633335.1	Vibrio phage K04M1_VK04M1
1912326	GCA_002633375.1	Vibrio phage K05K4_VK05K4_1
1912327	GCA_002633405.1	Vibrio phage K05K4_VK05K4_2
292443	GCA_000846985.1	Vibrio phage KSF1
75320	GCA_000843785.1	Vibrio phage KVP40
1897747	GCA_002613785.1	Vibrio phage Len
2500150	GCA_004015505.1	Vibrio phage LP.1
2500151	GCA_004015465.1	Vibrio phage LP.2
573175	GCA_000904715.1	Vibrio phage martha 12B12
644563	GCA_000887015.1	Vibrio phage N4
867695	GCA_002630525.1	Vibrio phage ND1-fs1
115992	GCA_000910195.2	Vibrio phage nt-1
1965459	GCA_002620225.1	Vibrio phage P2
2488664	GCA_003865655.1	Vibrio phage P23
1965460	GCA_002620245.1	Vibrio phage P3
1897748	GCA_002613805.1	Vibrio phage Pel
1589297	GCA_001505775.1	Vibrio phage phi 1
1476902	GCA_002604685.1	Vibrio phage phi 2
1589298	GCA_001505115.1	Vibrio phage phi 3
1151014	GCA_000928875.1	Vibrio phage phi-A318
1747713	GCA_002757115.1	Vibrio phage phi-Grn1
1747714	GCA_002757135.1	Vibrio phage phi-ST2
1076759	GCA_001015265.1	Vibrio phage phiVC8
689414	GCA_003051825.1	Vibrio phage pre-CTX
689414	GCA_003051745.1	Vibrio phage pre-CTX
689414	GCA_003051785.1	Vibrio phage pre-CTX
689414	GCA_003051765.1	Vibrio phage pre-CTX
689414	GCA_003051725.1	Vibrio phage pre-CTX
689414	GCA_003051805.1	Vibrio phage pre-CTX
689414	GCA_003926015.1	Vibrio phage pre-CTX
689414	GCA_003926035.1	Vibrio phage pre-CTX
689414	GCA_003926055.1	Vibrio phage pre-CTX
689414	GCA_003926075.1	Vibrio phage pre-CTX
689414	GCA_003926095.1	Vibrio phage pre-CTX
1938577	GCA_002618825.1	Vibrio phage pTD1
1451050	GCA_001041395.1	Vibrio phage PV94
1461743	GCA_000915635.1	Vibrio phage PVA1
1897751	GCA_002613865.1	Vibrio phage pVa-1
1897752	GCA_002613885.1	Vibrio phage pVa-2
1958921	GCA_002619305.1	Vibrio phage pVa-21
1965461	GCA_002620265.1	Vibrio phage pVa-3
1965462	GCA_002620285.1	Vibrio phage pVa-4
1897753	GCA_002613905.1	Vibrio phage pVa-5
1897754	GCA_002613925.1	Vibrio phage pVa-6
1897755	GCA_002613945.1	Vibrio phage pVa-7
1965463	GCA_002620305.1	Vibrio phage pVa-8
1965485	GCA_002620725.1	Vibrio phage pVco-5
1150989	GCA_000900795.1	Vibrio phage pVp-1
754058	GCA_000904415.1	Vibrio phage PWH3a-P1
754049	GCA_000904395.1	Vibrio phage pYD21-A

754051	GCA_000908715.1	Vibrio phage pYD38-A
929835	GCA_000909395.1	Vibrio phage pYD38-B
1003177	GCA_001551505.1	Vibrio phage qdvp001
1558469	GCA_001041095.1	Vibrio phage QH
2283098	GCA_003367015.1	Vibrio phage R01
2116688	GCA_003094035.1	Vibrio phage Rostov 6
2544946	GCA_004340245.1	Vibrio phage Rostov 7
2086639	GCA_002990475.1	Vibrio phage Rostov-1
1653734	GCA_002633235.1	Vibrio phage RYC
1873905	GCA_002611785.1	Vibrio phage S4-7
1414739	GCA_000915795.1	Vibrio phage SHOU24
700512	GCA_000896615.1	Vibrio phage SIO-2
1161928	GCA_002617325.1	Vibrio phage SSP002
1897749	GCA_002613825.1	Vibrio phage Strym
2570301	GCA_002957655.1	Vibrio phage Thalassa
1897750	GCA_002613845.1	Vibrio phage VaK
2508852	GCA_004138815.1	Vibrio phage ValB1MD
1610855	GCA_001501635.1	Vibrio phage ValKK3
2484244	GCA_004146965.1	Vibrio phage ValLY_3
2484246	GCA_004146985.1	Vibrio phage ValSw4_1
2024229	GCA_002627785.1	Vibrio phage vB_ValP_IME271
1127518	GCA_000902415.1	Vibrio phage vB_VchM-138
1868610	GCA_003344125.1	Vibrio phage vB_VhaS-a
1868611	GCA_003604955.1	Vibrio phage vB_VhaS-tm
1775142	GCA_002608825.1	Vibrio phage vB_VmeM-32
1689660	GCA_002607165.1	Vibrio phage vB_VorS-PVo5
1229754	GCA_000902755.1	Vibrio phage vB_VpaM_MAR
2041472	GCA_003441355.1	Vibrio phage vB_VpaP_KF1
2041473	GCA_003441375.1	Vibrio phage vB_VpaP_KF2
2041474	GCA_003441395.1	Vibrio phage vB_VpaS_KF3
2041475	GCA_003441415.1	Vibrio phage vB_VpaS_KF4
2041476	GCA_003441435.1	Vibrio phage vB_VpaS_KF5
2041477	GCA_003441455.1	Vibrio phage vB_VpaS_KF6
1229755	GCA_000902135.1	Vibrio phage vB_VpaS_MAR10
2419783	GCA_004340605.1	Vibrio phage vB_VpaS_OWB
2301664	GCA_003443075.1	Vibrio phage vB_VpS_PG07
1913109	GCA_002615085.1	Vibrio phage vB_VspP_pVa5
2026084	GCA_002743615.1	Vibrio phage vB_VspS_VS-ABTNL-3
754074	GCA_000906095.1	Vibrio phage VBM1
754072	GCA_000906955.1	Vibrio phage VBP32
754073	GCA_000904475.1	Vibrio phage VBP47
1480731	GCA_002604545.1	Vibrio phage Vc1
1283073	GCA_002603165.1	Vibrio phage VCO139
1841196	GCA_002757675.1	Vibrio phage VcP032
1105327	GCA_000894015.1	Vibrio phage VCY-phi
642111	GCA_000883935.1	Vibrio phage VEJphi
2059879	GCA_002957545.1	Vibrio phage VEN
127511	GCA_002603105.1	Vibrio phage Vf12
1340745	GCA_000907795.1	Vibrio phage VFJ
127507	GCA_000837165.1	Vibrio phage VfO3K6

127508	GCA_000839665.1	Vibrio phage VfO4K68
223524	GCA_000840605.1	Vibrio phage VGJphi
1262539	GCA_000914495.1	Vibrio phage VH7D
207597	GCA_000841185.1	Vibrio phage VHML
1434659	GCA_003931495.1	Vibrio phage VP_EVC_2013
2059867	GCA_002957535.1	Vibrio phage Vp_R1
2082229	GCA_003368605.1	Vibrio phage VP06
238893	GCA_004216435.1	Vibrio phage VP16C
238892	GCA_004216455.1	Vibrio phage VP16T
260372	GCA_000844065.1	Vibrio phage VP2
588068	GCA_002629945.1	Vibrio phage VP3
1262540	GCA_003931475.1	Vibrio phage VP4B
260827	GCA_000844445.1	Vibrio phage VP5
631719	GCA_001308515.1	Vibrio phage VP585
1932890	GCA_002618025.1	Vibrio phage Vp670
641832	GCA_000881295.1	Vibrio phage VP93
2484247	GCA_004147005.1	Vibrio phage VpaJT_1
1538804	GCA_000955015.2	Vibrio phage VpKK5
1233488	GCA_000910375.1	Vibrio phage VPMS1
1524880	GCA_002604885.1	Vibrio phage VPp1
1341101	GCA_000912935.1	Vibrio phage VPUSM 8
181604	GCA_000843365.1	Vibrio phage VSK
180503	GCA_002609565.1	Vibrio phage VSKK
2484248	GCA_004147025.1	Vibrio phage VspDsh_1
2484249	GCA_004147045.1	Vibrio phage VspSw_1
1168281	GCA_000906755.1	Vibrio phage VvAW1
2059877	GCA_003979555.1	Vibrio phage VVP001
1500713	GCA_000922995.1	Vibrio phage X29
2267403	GCA_003441855.1	Vibrio phage YC
141904	GCA_000893195.1	Vibrio virus CTXphi
141904	GCA_002921865.1	Vibrio virus CTXphi
141904	GCA_003069445.1	Vibrio virus CTXphi
141904	GCA_003094455.1	Vibrio virus CTXphi
141904	GCA_003931515.1	Vibrio virus CTXphi
141904	GCA_004319725.1	Vibrio virus CTXphi
70203	GCA_000842845.1	Vibrio virus fs1
70734	GCA_000839045.1	Vibrio virus K139
493906	GCA_000872585.1	Vibrio virus Kappa
2500581	GCA_004194215.1	Vibrio virus vB_VspP_SBP1
127509	GCA_000845325.1	Vibrio virus Vf33
261726	GCA_000868005.1	Vibrio virus VP882
194802	GCA_000843265.1	Vibrio virus VpV262
1204514	GCA_002630725.1	Vibriophage phi-pp2
329886	GCA_000864645.1	Vibriophage VP4
54289	GCA_000865745.1	Vicia cryptic virus
54289	GCA_002868075.1	Vicia cryptic virus
54289	GCA_002868095.1	Vicia cryptic virus
54289	GCA_002868055.1	Vicia cryptic virus
54289	GCA_002868035.1	Vicia cryptic virus
310749	GCA_000864345.1	Vicia faba alphaendornavirus

1661392	GCA_001654265.1	Victorian trout aquabirnavirus
2169757	GCA_002080195.1	Vicugna pacos polyomavirus 1
1312724	GCA_002824725.1	Vigna yellow mosaic virus
1170656	GCA_001430335.1	Vinca leaf curl virus
1147160	GCA_000856505.1	Viral hemorrhagic septicemia virus Fil3
2530052	GCA_004521595.1	Virgibacillus phage Mimir87
1924547	GCA_003728995.1	Virus Rctr16k
1924548	GCA_003728915.1	Virus Rctr197k
1924549	GCA_003728975.1	Virus Rctr41k
1924550	GCA_003728955.1	Virus Rctr71
1924551	GCA_003728935.1	Virus Rctr85
2202649	GCA_003652305.1	Virus sp.
2202649	GCA_003658745.1	Virus sp.
2202649	GCA_003655325.1	Virus sp.
2202649	GCA_003656905.1	Virus sp.
2202649	GCA_003659125.1	Virus sp.
2202649	GCA_003657245.1	Virus sp.
2202649	GCA_003657225.1	Virus sp.
2202649	GCA_003651745.1	Virus sp.
2202649	GCA_003655985.1	Virus sp.
2202649	GCA_003659325.1	Virus sp.
2202649	GCA_003729915.1	Virus sp.
2169971	GCA_000849025.1	Visna-maedi virus
28874	GCA_001744115.1	Volepox virus
1163709	GCA_002827145.1	Vulpes vulpes papillomavirus 1
1888308	GCA_001717255.1	Wabat virus
40067	GCA_001184905.1	Wad Medani virus
1569258	GCA_001432115.1	Walkabout Creek virus
40061	GCA_000912775.1	Wallal virus
1457165	GCA_000916075.1	Wallerfield virus
39720	GCA_000850265.1	Walleye dermal sarcoma virus
146073	GCA_000852525.1	Walrus calicivirus
40062	GCA_002829465.1	Warrego virus
1169032	GCA_000849485.1	Wasabi mottle virus
1888309	GCA_001717315.1	Washington bat picornavirus
2293309	GCA_003846885.1	Water beetle associated circular virus 1
2293309	GCA_003846905.1	Water beetle associated circular virus 1
1848040	GCA_001651145.1	Water chestnut soymovirus 1
76052	GCA_002816095.1	Watermelon bud necrosis virus
35341	GCA_000837565.1	Watermelon chlorotic stunt virus
146500	GCA_000856625.1	Watermelon mosaic virus
1933300	GCA_000858945.1	Watermelon silver mottle tospovirus
1978413	GCA_002105425.1	Watermelon virus A
1161906	GCA_000899855.1	Weissella phage phiYS61
1970788	GCA_003693725.1	Weissella phage PWc
1837862	GCA_001746035.1	Weissella phage WCP30
1566309	GCA_000927955.1	Wellfleet Bay virus
1508228	GCA_002219865.1	Wencheng Sm shrew coronavirus
1922377	GCA_001926115.1	Wenling chuvirus-like virus 1
1922378	GCA_001925675.1	Wenling chuvirus-like virus 2

1923478	GCA_001960115.1	Wenling crustacean virus 1
1923479	GCA_001958455.1	Wenling crustacean virus 10
1923480	GCA_001959195.1	Wenling crustacean virus 11
1923481	GCA_001960695.1	Wenling crustacean virus 12
1923482	GCA_001960095.1	Wenling crustacean virus 13
1923483	GCA_001958435.1	Wenling crustacean virus 14
1923484	GCA_001959175.1	Wenling crustacean virus 15
1923485	GCA_001960675.1	Wenling crustacean virus 2
1923486	GCA_001960075.1	Wenling crustacean virus 3
1923487	GCA_001958415.1	Wenling crustacean virus 4
1923488	GCA_001959155.1	Wenling crustacean virus 5
1923489	GCA_001960655.1	Wenling crustacean virus 6
1923492	GCA_001921515.1	Wenling crustacean virus 9
2116466	GCA_004128195.1	Wenling frogfish arenavirus 1
2116467	GCA_004128215.1	Wenling frogfish arenavirus 2
1923493	GCA_001960055.1	Wenling hepe-like virus 1
1923494	GCA_001958395.1	Wenling hepe-like virus 2
1923495	GCA_001959135.1	Wenling hepe-like virus 3
1923496	GCA_001960635.1	Wenling hepe-like virus 4
1923501	GCA_001960035.1	Wenling narna-like virus 1
1923502	GCA_001958375.1	Wenling narna-like virus 2
1923503	GCA_001959115.1	Wenling narna-like virus 3
1923504	GCA_001960615.1	Wenling narna-like virus 4
1923505	GCA_001960015.1	Wenling narna-like virus 5
1923507	GCA_001958355.1	Wenling narna-like virus 7
1923509	GCA_001959095.1	Wenling narna-like virus 9
1923510	GCA_001960595.1	Wenling nido-like virus 1
1923529	GCA_001959995.1	Wenling picorna-like virus 1
1923530	GCA_001926675.1	Wenling picorna-like virus 2
1923531	GCA_001958335.1	Wenling picorna-like virus 3
1923532	GCA_001959075.1	Wenling picorna-like virus 4
1923533	GCA_001960575.1	Wenling picorna-like virus 5
1923534	GCA_001959975.1	Wenling picorna-like virus 6
1923535	GCA_001958315.1	Wenling picorna-like virus 7
1923536	GCA_001959055.1	Wenling picorna-like virus 8
1923537	GCA_001960555.1	Wenling picorna-like virus 9
1746066	GCA_001444125.1	Wenling shark virus
1923540	GCA_001959955.1	Wenling sobemo-like virus 1
1923541	GCA_001958295.1	Wenling sobemo-like virus 2
1923542	GCA_001959035.1	Wenling toga-like virus
1923543	GCA_001960535.1	Wenling tombus-like virus 1
1923544	GCA_001959935.1	Wenling tombus-like virus 2
1923545	GCA_001958275.1	Wenling tombus-like virus 3
1923546	GCA_001959015.1	Wenling tombus-like virus 4
1923547	GCA_001960515.1	Wenling tombus-like virus 5
1923549	GCA_001959915.1	Wenling toti-like virus 2
1923551	GCA_001958255.1	Wenling zhaovirus-like virus 1
1923553	GCA_001958995.1	Wenzhou bivalvia virus 1
1923554	GCA_001960495.1	Wenzhou bivalvia virus 2
1923555	GCA_001959895.1	Wenzhou bivalvia virus 3

1923557	GCA_001958235.1	Wenzhou channeled applesnail virus 1
1923558	GCA_001958975.1	Wenzhou channeled applesnail virus 2
1923559	GCA_001960475.1	Wenzhou channeled applesnail virus 3
1608091	GCA_001755505.1	Wenzhou Crab Virus 1
1608092	GCA_003673645.1	Wenzhou crab virus 2
1608093	GCA_001754645.1	Wenzhou Crab Virus 3
1923561	GCA_001959875.1	Wenzhou crab virus 4
1923562	GCA_001958215.1	Wenzhou crab virus 5
1923563	GCA_001958955.1	Wenzhou gastropodes virus 1
1923564	GCA_001960455.1	Wenzhou gastropodes virus 2
1923566	GCA_001958195.1	Wenzhou hepe-like virus 1
1923565	GCA_001959855.1	Wenzhou hepe-like virus 2
1588685	GCA_000930735.1	Wenzhou mammarenavirus
1923577	GCA_001958935.1	Wenzhou narna-like virus 2
1923578	GCA_001960435.1	Wenzhou narna-like virus 3
1923579	GCA_001927175.1	Wenzhou narna-like virus 4
1923580	GCA_001959835.1	Wenzhou narna-like virus 5
1923582	GCA_001958175.1	Wenzhou narna-like virus 7
1923583	GCA_001958915.1	Wenzhou narna-like virus 8
1923584	GCA_001960415.1	Wenzhou narna-like virus 9
1923593	GCA_001959815.1	Wenzhou picorna-like virus 1
1923594	GCA_001958155.1	Wenzhou picorna-like virus 10
1923595	GCA_001958895.1	Wenzhou picorna-like virus 11
1923596	GCA_001960395.1	Wenzhou picorna-like virus 12
1923597	GCA_001959795.1	Wenzhou picorna-like virus 13
1923598	GCA_001958135.1	Wenzhou picorna-like virus 14
1923599	GCA_001958875.1	Wenzhou picorna-like virus 15
1923600	GCA_001960375.1	Wenzhou picorna-like virus 16
1923601	GCA_001959775.1	Wenzhou picorna-like virus 17
1923602	GCA_001958115.1	Wenzhou picorna-like virus 18
1923603	GCA_001958855.1	Wenzhou picorna-like virus 19
1923604	GCA_001960355.1	Wenzhou picorna-like virus 2
1923605	GCA_001959755.1	Wenzhou picorna-like virus 20
1923606	GCA_001958095.1	Wenzhou picorna-like virus 21
1923607	GCA_001958835.1	Wenzhou picorna-like virus 22
1923608	GCA_001957955.1	Wenzhou picorna-like virus 23
1923609	GCA_001959735.1	Wenzhou picorna-like virus 24
1923610	GCA_001958075.1	Wenzhou picorna-like virus 25
1923611	GCA_001958815.1	Wenzhou picorna-like virus 26
1923612	GCA_001957935.1	Wenzhou picorna-like virus 27
1923613	GCA_001959715.1	Wenzhou picorna-like virus 28
1923614	GCA_001958055.1	Wenzhou picorna-like virus 29
1923615	GCA_001926335.1	Wenzhou picorna-like virus 3
1923617	GCA_001958795.1	Wenzhou picorna-like virus 31
1923618	GCA_001957915.1	Wenzhou picorna-like virus 32
1923619	GCA_001959695.1	Wenzhou picorna-like virus 33
1923621	GCA_001958035.1	Wenzhou picorna-like virus 35
1923622	GCA_001958775.1	Wenzhou picorna-like virus 36
1923623	GCA_001957895.1	Wenzhou picorna-like virus 37
1923625	GCA_001959675.1	Wenzhou picorna-like virus 39

1923626	GCA_001958015.1	Wenzhou picorna-like virus 4
1923627	GCA_001968335.1	Wenzhou picorna-like virus 40
1923628	GCA_001957875.1	Wenzhou picorna-like virus 41
1923629	GCA_001959655.1	Wenzhou picorna-like virus 42
1923630	GCA_001957995.1	Wenzhou picorna-like virus 43
1923631	GCA_001968315.1	Wenzhou picorna-like virus 44
1923632	GCA_001957855.1	Wenzhou picorna-like virus 45
1923633	GCA_001959635.1	Wenzhou picorna-like virus 46
1923634	GCA_001957975.1	Wenzhou picorna-like virus 47
1923635	GCA_001968295.1	Wenzhou picorna-like virus 48
1923636	GCA_001957835.1	Wenzhou picorna-like virus 49
1923637	GCA_001959615.1	Wenzhou picorna-like virus 5
1923639	GCA_001967775.1	Wenzhou picorna-like virus 51
1923640	GCA_001968275.1	Wenzhou picorna-like virus 52
1923641	GCA_001957815.1	Wenzhou picorna-like virus 53
1923642	GCA_001959595.1	Wenzhou picorna-like virus 54
1923643	GCA_001967755.1	Wenzhou picorna-like virus 6
1923644	GCA_001968255.1	Wenzhou picorna-like virus 7
1923646	GCA_001957795.1	Wenzhou picorna-like virus 9
1923647	GCA_001959575.1	Wenzhou qinvirus-like virus 1
1923648	GCA_001967735.1	Wenzhou qinvirus-like virus 2
1608095	GCA_001755065.1	Wenzhou Shrimp Virus 1
1923649	GCA_001968235.1	Wenzhou shrimp virus 10
1923650	GCA_001957775.1	Wenzhou shrimp virus 3
1923651	GCA_001957315.1	Wenzhou shrimp virus 4
1923652	GCA_001967715.1	Wenzhou shrimp virus 5
1923653	GCA_001968215.1	Wenzhou shrimp virus 6
1923654	GCA_001925895.1	Wenzhou shrimp virus 7
1923655	GCA_001957755.1	Wenzhou shrimp virus 8
1923656	GCA_001957295.1	Wenzhou shrimp virus 9
1923657	GCA_001967695.1	Wenzhou sobemo-like virus 1
1923658	GCA_001968195.1	Wenzhou sobemo-like virus 2
1923659	GCA_001957735.1	Wenzhou sobemo-like virus 3
1923660	GCA_001957275.1	Wenzhou sobemo-like virus 4
1923661	GCA_001970265.1	Wenzhou tapeworm virus 1
1608094	GCA_001754205.1	Wenzhou Tick Virus
1923662	GCA_001970565.1	Wenzhou tombus-like virus 1
1923663	GCA_001970725.1	Wenzhou tombus-like virus 10
1923664	GCA_001970405.1	Wenzhou tombus-like virus 11
1923665	GCA_001970705.1	Wenzhou tombus-like virus 12
1923667	GCA_001970545.1	Wenzhou tombus-like virus 14
1923668	GCA_001970245.1	Wenzhou tombus-like virus 15
1923669	GCA_001970225.1	Wenzhou tombus-like virus 16
1923670	GCA_001970685.1	Wenzhou tombus-like virus 17
1923671	GCA_001970385.1	Wenzhou tombus-like virus 18
1923672	GCA_001970525.1	Wenzhou tombus-like virus 2
1923673	GCA_001970825.1	Wenzhou tombus-like virus 3
1923674	GCA_001970665.1	Wenzhou tombus-like virus 4
1923675	GCA_001970365.1	Wenzhou tombus-like virus 5
1923676	GCA_001970505.1	Wenzhou tombus-like virus 6

1923677	GCA_001970805.1	Wenzhou tombus-like virus 7
1923678	GCA_001970645.1	Wenzhou tombus-like virus 8
1923679	GCA_001970345.1	Wenzhou tombus-like virus 9
1923680	GCA_001926655.1	Wenzhou toti-like virus 1
1923682	GCA_001970485.1	Wenzhou weivirus-like virus 1
1923683	GCA_001970785.1	Wenzhou yanvirus-like virus 1
1923684	GCA_001970625.1	Wenzhou yanvirus-like virus 2
164416	GCA_000883915.1	Wesselsbron virus
1046573	GCA_000896655.1	West African Asystasia virus 1
1046574	GCA_002824745.1	West African Asystasia virus 2
249584	GCA_000924535.1	West Caucasian bat lyssavirus
11082	GCA_000875385.1	West Nile virus
11082	GCA_000861085.1	West Nile virus
2170203	GCA_000848485.1	Western chimpanzee simian foamy virus
11039	GCA_000850885.1	Western equine encephalitis virus
11039	GCA_002889215.1	Western equine encephalitis virus
2170204	GCA_003032825.1	Western lowland gorilla simian foamy virus
48543	GCA_000896835.1	Whataroa virus
1174526	GCA_000894555.1	Wheat dwarf India virus
10834	GCA_002825495.1	Wheat dwarf virus
10834	GCA_002825545.1	Wheat dwarf virus
10834	GCA_002825575.1	Wheat dwarf virus
10834	GCA_002987285.1	Wheat dwarf virus
10834	GCA_002987295.1	Wheat dwarf virus
290587	GCA_000865525.1	Wheat dwarf virus - [Enkoping1]
460364	GCA_000873525.1	Wheat eqlid mosaic virus
2019445	GCA_002270665.1	Wheat leaf yellowing-associated virus
35285	GCA_004117675.1	Wheat spindle streak mosaic virus
31741	GCA_000862365.1	Wheat streak mosaic virus
2364281	GCA_004117795.1	Wheat stripe mosaic virus
572239	GCA_000884995.1	Wheat yellow dwarf virus-GPV
75746	GCA_000862385.1	Wheat yellow mosaic virus
405554	GCA_000867725.1	White bream virus
292052	GCA_000854325.1	White clover cryptic virus 1
1323525	GCA_000907235.1	White clover cryptic virus 2
12188	GCA_000855045.1	White clover mosaic virus
1913024	GCA_001866835.1	White clover mottle virus
390237	GCA_002828285.1	White lupin mosaic virus
342409	GCA_000848085.2	White spot syndrome virus
342409	GCA_003972365.1	White spot syndrome virus
342409	GCA_003972385.1	White spot syndrome virus
342409	GCA_003972465.1	White spot syndrome virus
342409	GCA_003972525.1	White spot syndrome virus
342409	GCA_003972545.1	White spot syndrome virus
342409	GCA_003972345.1	White spot syndrome virus
342409	GCA_003972405.1	White spot syndrome virus
342409	GCA_003972425.1	White spot syndrome virus
342409	GCA_003972445.1	White spot syndrome virus
342409	GCA_003972485.1	White spot syndrome virus
342409	GCA_003972505.1	White spot syndrome virus

1690672	GCA_001308495.1	White sucker hepatitis B virus
1159907	GCA_000896875.1	White-eye coronavirus HKU16
2560838	GCA_003029185.1	Whitefly associated Guatemala alphasatellite 1
2169828	GCA_003029175.1	Whitefly associated Guatemala alphasatellite 2
2169829	GCA_003029195.1	Whitefly associated Puerto Rico alphasatellite 1
2169739	GCA_003029115.1	Whitefly-associated begomovirus 1
2169740	GCA_003029125.1	Whitefly-associated begomovirus 2
2169741	GCA_003029135.1	Whitefly-associated begomovirus 3
2169742	GCA_003029145.1	Whitefly-associated begomovirus 4
2169743	GCA_003029155.1	Whitefly-associated begomovirus 6
2169744	GCA_003029165.1	Whitefly-associated begomovirus 7
2170205	GCA_003032835.1	White-tufted-ear marmoset simian foamy virus
46919	GCA_000872865.1	Whitewater Arroyo mammarenavirus
1159908	GCA_000895415.1	Wigeon coronavirus HKU20
2018313	GCA_002270685.1	Wild melon vein banding virus
1862127	GCA_001678235.1	Wild onion symptomless virus
187977	GCA_000863445.1	Wild potato mosaic virus
400396	GCA_000871025.1	Wild tomato mosaic virus
2025352	GCA_002270745.1	Wild vitis virus 1
2010280	GCA_002210555.1	Wilkie narna-like virus 1
2010280	GCA_003729375.1	Wilkie narna-like virus 1
2010281	GCA_002210915.1	Wilkie narna-like virus 2
2010283	GCA_002210755.1	Wilkie partiti-like virus 1
2010284	GCA_002210655.1	Wilkie partiti-like virus 2
2010284	GCA_003729315.1	Wilkie partiti-like virus 2
2169693	GCA_001755825.1	Winged bean alphaendornavirus 1
68347	GCA_000891235.1	Wiseana iridescent virus
373593	GCA_000880135.1	Wissadula golden mosaic St Thomas Virus
1904884	GCA_001777245.1	Wissadula yellow mosaic virus
1973265	GCA_002080255.1	Wisteria badnavirus 1
201862	GCA_000866545.1	Wisteria vein mosaic virus
1118369	GCA_000973275.2	Wobbly possum disease virus
949124	GCA_002633685.1	Wolbachia endosymbiont wVitA of Nasonia vitripennis pha
949125	GCA_002633705.1	Wolbachia endosymbiont wVitA of Nasonia vitripennis pha
949126	GCA_002633725.1	Wolbachia endosymbiont wVitA of Nasonia vitripennis pha
1969991	GCA_002990955.1	Wolbachia phage sr1WOdamA
112596	GCA_002602005.1	Wolbachia phage WO
1867943	GCA_002158555.1	Wolkberg virus
1972626	GCA_000882535.1	Wongabel hapavirus
35269	GCA_000840285.1	Woodchuck hepatitis virus
2293310	GCA_003846665.1	Woodlouse hunter spider associated circular virus 1
68416	GCA_001429935.1	Woolly monkey hepatitis B virus
68416	GCA_003033095.1	Woolly monkey hepatitis B virus
11970	GCA_000870685.1	Woolly monkey sarcoma virus
10987	GCA_002994715.1	Wound tumor virus
440266	GCA_000870785.1	WU Polyomavirus
1923685	GCA_001970325.1	Wuchan romanomermis nematode virus 1
1923686	GCA_001970465.1	Wuchan romanomermis nematode virus 2
1923687	GCA_001970765.1	Wuchan romanomermis nematode virus 3
1608097	GCA_001755485.1	Wuchang Cockroach Virus 1

1608099	GCA_003673625.1	Wuchang Cockroach Virus 3
1923688	GCA_001970605.1	Wuchang cockroach Virus 4
1608100	GCA_001754625.1	Wuhan Ant Virus
1746067	GCA_001444165.1	Wuhan aphid virus 1
1746068	GCA_001443945.1	Wuhan aphid virus 2
1923690	GCA_001970445.1	Wuhan arthropod virus 1
1923691	GCA_001970745.1	Wuhan arthropod virus 2
1923692	GCA_001970585.1	Wuhan arthropod virus 3
1923693	GCA_001970285.1	Wuhan arthropod virus 4
1746069	GCA_001654325.1	Wuhan centipede virus
1923695	GCA_001970425.1	Wuhan coneheads virus 1
1923696	GCA_001973835.1	Wuhan coneheads virus 2
1746070	GCA_001446225.1	Wuhan cricket virus
1923697	GCA_002004895.1	Wuhan cricket virus 2
1746071	GCA_001446245.1	Wuhan flea virus
1608101	GCA_001754185.1	Wuhan Fly Virus 1
1608102	GCA_001755045.1	Wuhan Fly Virus 2
1923698	GCA_001974315.1	Wuhan fly virus 4
1923699	GCA_001973995.1	Wuhan fly virus 5
1923701	GCA_001974135.1	Wuhan heteroptera virus 1
1923702	GCA_001974455.1	Wuhan heteroptera virus 2
1923703	GCA_001927155.1	Wuhan heteroptera virus 3
1608139	GCA_001754905.1	Wuhan horsefly Virus
1923704	GCA_001974295.1	Wuhan horsefly Virus 3
1923705	GCA_001973975.1	Wuhan house centipede virus 1
1923706	GCA_001974115.1	Wuhan house centipede virus 2
1923707	GCA_001974435.1	Wuhan house centipede virus 3
1923708	GCA_001974275.1	Wuhan house centipede virus 4
1923709	GCA_001973955.1	Wuhan house centipede virus 5
1923710	GCA_001974095.1	Wuhan house centipede virus 6
1923713	GCA_001974415.1	Wuhan house centipede virus 9
1608104	GCA_001755465.1	Wuhan House Fly Virus 1
1608105	GCA_001754605.1	Wuhan House Fly Virus 2
1923714	GCA_001974255.1	Wuhan insect virus 10
1923715	GCA_001973935.1	Wuhan insect virus 11
1923716	GCA_002008615.1	Wuhan insect virus 12
1923717	GCA_001974155.1	Wuhan insect virus 13
1923718	GCA_001974075.1	Wuhan insect virus 14
1923719	GCA_001974395.1	Wuhan insect virus 15
1923721	GCA_001974235.1	Wuhan insect virus 17
1923722	GCA_001973915.1	Wuhan insect virus 18
1923723	GCA_001974055.1	Wuhan insect virus 19
1608107	GCA_001706905.1	Wuhan Insect virus 2
1923724	GCA_001974375.1	Wuhan insect virus 20
1923725	GCA_001974215.1	Wuhan insect virus 21
1923726	GCA_002008815.1	Wuhan insect virus 22
1923727	GCA_001921615.1	Wuhan insect virus 23
1923730	GCA_001973895.1	Wuhan insect virus 26
1923731	GCA_001974035.1	Wuhan insect virus 27
1923732	GCA_001974355.1	Wuhan insect virus 28

1923735	GCA_001974195.1	Wuhan insect virus 31
1923736	GCA_002004235.1	Wuhan insect virus 33
1923737	GCA_002003915.1	Wuhan insect virus 34
1923738	GCA_002004535.1	Wuhan insect virus 35
1608109	GCA_001755445.1	Wuhan Insect virus 4
1608110	GCA_001755325.1	Wuhan Insect virus 5
1608111	GCA_001755745.1	Wuhan Insect virus 6
1608112	GCA_001754885.1	Wuhan Insect virus 7
1923739	GCA_002004875.1	Wuhan insect virus 8
1923740	GCA_002004215.1	Wuhan insect virus 9
1923741	GCA_001921315.1	Wuhan large pig roundworm virus 1
1608114	GCA_001754445.1	Wuhan Louse Fly Virus 10
1608119	GCA_001754465.1	Wuhan Louse Fly Virus 5
1608120	GCA_003673705.1	Wuhan louse fly virus 6
1608121	GCA_003673605.1	Wuhan Louse Fly Virus 7
1608123	GCA_001755785.1	Wuhan Louse Fly Virus 9
1608125	GCA_003972765.1	Wuhan Millipede Virus 2
1923742	GCA_002003895.1	Wuhan millipede virus 3
1923689	GCA_001970305.1	Wuhan Millipede virus 4
1608126	GCA_001754925.1	Wuhan mosquito virus 1
1608127	GCA_001754485.1	Wuhan Mosquito Virus 2
1608133	GCA_001440835.1	Wuhan Mosquito Virus 8
1608134	GCA_001755345.1	Wuhan Mosquito Virus 9
1923743	GCA_002004515.1	Wuhan nido-like virus 1
1923744	GCA_001926315.1	Wuhan pillworm virus 1
1923745	GCA_002004855.1	Wuhan pillworm virus 2
1923746	GCA_002004195.1	Wuhan pillworm virus 3
1923748	GCA_002003875.1	Wuhan poty-like virus 1
1923751	GCA_001921395.1	Wuhan spider virus 2
1923752	GCA_002004495.1	Wuhan spider virus 3
1923753	GCA_002004835.1	Wuhan spider virus 4
1923754	GCA_002004175.1	Wuhan spider virus 5
1923755	GCA_002003855.1	Wuhan spider virus 6
1923756	GCA_002004475.1	Wuhan spider virus 7
1923757	GCA_002004815.1	Wuhan spider virus 8
1923758	GCA_002004155.1	Wuhan spider virus 9
1923759	GCA_002003835.1	Wuhan spirurian nematodes virus 1
1608137	GCA_001755765.1	Wuhan Tick Virus 1
1608138	GCA_001440995.1	Wuhan tick virus 2
1245561	GCA_003033305.1	Wuharv parvovirus 1
1980612	GCA_001755805.1	Wutai mosquito phasivirus
273350	GCA_002814455.1	Wyeomyia orthobunyavirus
1188795	GCA_000904115.1	Xanthomonas citri phage CP2
2163636	GCA_003094275.1	Xanthomonas phage Carpasina
1784979	GCA_002624525.1	Xanthomonas phage f20-Xaj
1784980	GCA_002609225.1	Xanthomonas phage f29-Xaj
1784981	GCA_002624545.1	Xanthomonas phage f30-Xaj
1927017	GCA_003861635.1	Xanthomonas phage KPhi1
331627	GCA_000865365.1	Xanthomonas phage OP2
2024237	GCA_002627925.1	Xanthomonas phage phi Xc10

126989	GCA_003308835.1	Xanthomonas phage phiLF
2201344	GCA_003308795.1	Xanthomonas phage phiLf2
2201472	GCA_003308815.1	Xanthomonas phage phiXv2
2108116	GCA_003014195.1	Xanthomonas phage RiverRider
2108116	GCA_003014195.2	Xanthomonas phage RiverRider
1129194	GCA_000896315.1	Xanthomonas phage vB_XveM_DIBBI
1463607	GCA_002601625.1	Xanthomonas phage XacF1
2042251	GCA_003173815.1	Xanthomonas phage XacN1
1775249	GCA_002608865.1	Xanthomonas phage XAJ2
1775250	GCA_002608885.1	Xanthomonas phage XAJ24
1852597	GCA_002610085.1	Xanthomonas phage Xf109
1979540	GCA_002622345.1	Xanthomonas phage Xf409
1852622	GCA_002610145.1	Xanthomonas phage Xoo-sp2
322855	GCA_000857585.1	Xanthomonas phage Xp15
2099853	GCA_004193995.1	Xanthomonas phage XPP1
2099854	GCA_004194015.1	Xanthomonas phage XPP2
2099855	GCA_004194035.1	Xanthomonas phage XPP3
2099856	GCA_004194055.1	Xanthomonas phage XPP4
2099857	GCA_004194075.1	Xanthomonas phage XPP6
2099858	GCA_004194095.1	Xanthomonas phage XPP8
2099859	GCA_004194115.1	Xanthomonas phage XPP9
2099860	GCA_004194135.1	Xanthomonas phage XPV1
2099861	GCA_004194155.1	Xanthomonas phage XPV2
2099862	GCA_004194175.1	Xanthomonas phage XPV3
10862	GCA_000842465.1	Xanthomonas virus Cf1c
1188794	GCA_000901275.1	Xanthomonas virus CP1
329254	GCA_000866925.1	Xanthomonas virus OP1
538979	GCA_000884875.1	Xanthomonas virus phil7
2182344	GCA_004139235.1	Xanthomonas virus XcP1
470314	GCA_000873245.1	Xanthomonas virus Xop411
232237	GCA_000842285.1	Xanthomonas virus Xp10
1167690	GCA_000904635.1	Xanthophyllomyces dendrorhous virus L1A
1167691	GCA_002830745.1	Xanthophyllomyces dendrorhous virus L1B
1933103	GCA_002618445.1	Xenohaliotis phage pCXc-HC2016
1933104	GCA_002618465.1	Xenohaliotis phage pCXc-HR2015
204873	GCA_000875345.1	Xenopus laevis endogenous retrovirus Xen1
51677	GCA_000837945.1	Xestia c-nigrum granulovirus
1272959	GCA_000926455.1	Xiburema virus
1608141	GCA_001755005.1	Xincheng Mosquito Virus
1746072	GCA_001443805.1	Xingshan cricket virus
1923760	GCA_001925875.1	Xingshan nematode virus 1
1923761	GCA_002004455.1	Xingshan nematode virus 2
1923763	GCA_002004795.1	Xingshan nematode virus 4
1923764	GCA_002005095.1	Xingshan nematode virus 5
1923765	GCA_002004775.1	Xingshan nematode virus 6
1923768	GCA_002004115.1	Xinzhou dimarhabdovirus virus 1
1923769	GCA_002004735.1	Xinzhou nematode virus 1
1923770	GCA_002005075.1	Xinzhou nematode virus 2
1923771	GCA_002004755.1	Xinzhou nematode virus 3
1923772	GCA_002004095.1	Xinzhou nematode virus 4

1923773	GCA_002004715.1	Xinzhou nematode virus 5
1923775	GCA_002005055.1	Xinzhou nematode virus 7
1923776	GCA_001921495.1	Xinzhou partiti-like virus 1
1746073	GCA_001444025.1	Xinzhou spider virus 2
1746074	GCA_001654425.1	Xinzhou spider virus 3
1923777	GCA_002004395.1	Xinzhou toro-like virus
10561	GCA_000863665.1	Xipapillomavirus 1
1821227	GCA_002022215.1	Xishuangbanna aedes flavivirus
1415145	GCA_000914055.1	Xylella phage Paz
1415146	GCA_000913195.1	Xylella phage Prado
1415147	GCA_002604145.1	Xylella phage Salvo
1415148	GCA_002604165.1	Xylella phage Sano
670252	GCA_000885475.1	Xylella phage Xfas53
38804	GCA_000845705.1	Yaba monkey tumor virus
132475	GCA_000847185.1	Yaba-like disease virus
1561150	GCA_000928595.1	Yacon necrotic mottle virus
1868472	GCA_001693545.1	Yacon virus A
1148496	GCA_003956565.1	Yado-kari virus 1
2094187	GCA_003956545.1	Yado-nushi virus 1-A
1604871	GCA_001618995.1	Yak enterovirus
596957	GCA_002828105.1	Yam chlorotic necrotic mosaic virus
1592930	GCA_000988995.1	Yam latent virus
87097	GCA_000901635.1	Yam mild mosaic virus
41460	GCA_000851785.1	Yam mosaic virus
1421055	GCA_000913095.1	Yam spherical virus
1503864	GCA_000926955.1	Yam virus X
418827	GCA_000894095.1	Yambean mosaic virus
64319	GCA_002022175.1	Yaounde virus
1272960	GCA_001431995.1	Yata virus
1286213	GCA_000930015.1	Yellow baboon polyomavirus 1
1286214	GCA_000928975.1	Yellow baboon polyomavirus 2
11089	GCA_000857725.1	Yellow fever virus
96029	GCA_003972805.1	Yellow head virus
2170240	GCA_000922155.1	Yellow oat grass mosaic virus
1416026	GCA_000912435.1	Yellow tailflower mild mottle virus
2170206	GCA_003032845.1	Yellow-breasted capuchin simian foamy virus
1586712	GCA_001430255.1	Yellowstone lake mimivirus
1586713	GCA_001430655.1	Yellowstone lake phycodnavirus 1
1586714	GCA_001430455.1	Yellowstone lake phycodnavirus 2
1586715	GCA_001430035.1	Yellowstone lake phycodnavirus 3
1557033	GCA_001440895.1	Yellowstone Lake virophage 5
1557034	GCA_001440975.1	Yellowstone Lake virophage 6
1557035	GCA_001441055.1	Yellowstone Lake virophage 7
360000	GCA_000853245.1	Yellowtail ascites virus - Y-6
1514896	GCA_000923175.1	Yerba mate alphaendornavirus
2219873	GCA_004133065.1	Yerba mate-associated circular DNA virus 1
369257	GCA_000868705.1	Yersinia phage Berlin
2077277	GCA_900323715.1	Yersinia phage fEV-1
1932893	GCA_002618085.1	Yersinia phage fHe-Yen3-01
1965363	GCA_002619865.1	Yersinia phage fHe-Yen9-01

2052743	GCA_002990515.1	Yersinia phage fHe-Yen9-03
2052742	GCA_002990485.1	Yersinia phage fHe-Yen9-04
2052933	GCA_002990615.1	Yersinia phage fPS-10
2052933	GCA_002990835.1	Yersinia phage fPS-10
2052934	GCA_002990815.1	Yersinia phage fPS16
2052747	GCA_002990595.1	Yersinia phage fPS-19
2052748	GCA_002990715.1	Yersinia phage fPS-21
2052749	GCA_002990555.1	Yersinia phage fPS-26
2052750	GCA_002990695.1	Yersinia phage fPS-50
2052751	GCA_002990585.1	Yersinia phage fPS-52
2052752	GCA_002990765.1	Yersinia phage fPS-53
2052753	GCA_002990805.1	Yersinia phage fPS-54-ocr
2052754	GCA_002990725.1	Yersinia phage fPS-59
2052755	GCA_002990735.1	Yersinia phage fPS-64
2052745	GCA_002990635.1	Yersinia phage fPS-7
2052756	GCA_002990775.1	Yersinia phage fPS-85
2052757	GCA_002990675.1	Yersinia phage fPS-86
2052744	GCA_002990645.1	Yersinia phage fPS-89
2052746	GCA_002990525.1	Yersinia phage fPS-9
1206559	GCA_000901215.1	Yersinia phage phi80-18
1206559	GCA_000901215.2	Yersinia phage phi80-18
227720	GCA_000843345.1	Yersinia phage phiA1122
1206560	GCA_001042055.1	Yersinia phage phiD1
331278	GCA_000895615.1	Yersinia phage phiR1-37
1206558	GCA_000904795.1	Yersinia phage phiR1-RT
1206557	GCA_000903755.2	Yersinia phage phiR201
1206556	GCA_003047835.1	Yersinia phage phiR8-01
1662463	GCA_002606805.1	Yersinia phage phiYe-F10
110457	GCA_000847165.1	Yersinia phage phiYeO3-12
1351740	GCA_001042115.1	Yersinia phage PST
172667	GCA_000857465.1	Yersinia phage PY54
2321390	GCA_003668415.1	Yersinia phage PYPS2T
2321391	GCA_003865455.1	Yersinia phage PYPS50
1195074	GCA_002755215.1	Yersinia phage R
1589265	GCA_001505135.1	Yersinia phage vB_YenM_TG1
1735591	GCA_001470735.1	Yersinia phage vB_YenP_AP10
1536611	GCA_000926875.1	Yersinia phage vB_YenP_AP5
1675027	GCA_001501555.1	Yersinia phage vB_YenP_ISAO8
1176434	GCA_002755175.1	Yersinia phage Y
532078	GCA_000892275.1	Yersinia phage Yepe2
928293	GCA_000919455.1	Yersinia phage Yep-phi
1176764	GCA_002633605.1	Yersinia phage YpP-G
1176765	GCA_002991145.1	Yersinia phage YpP-R
1176766	GCA_002991125.1	Yersinia phage YpP-Y
1176767	GCA_002991165.1	Yersinia phage YpsP-G
227940	GCA_000841405.1	Yersinia virus L413C
1608144	GCA_001754945.1	Yichang Insect virus
2053026	GCA_004130315.1	Yichang virus
2479372	GCA_004132945.1	Ying Kou virus
1712572	GCA_001630045.1	Yogue virus

1076255	GCA_000892975.1	Yokapox virus
64294	GCA_000858665.1	Yokose virus
1608146	GCA_001754505.1	Yongjia Tick Virus 2
2315803	GCA_004117695.1	Yongsan bunyavirus 1
2315809	GCA_004134745.1	Yongsan iflavirus 1
2315806	GCA_004134445.1	Yongsan picorna-like virus 3
2315807	GCA_004134465.1	Yongsan picorna-like virus 4
2315813	GCA_004134485.1	Yongsan tombus-like virus 1
228578	GCA_000852505.1	Youcai mosaic virus
1972567	GCA_000925435.1	Yug Bogdanovac vesiculovirus
306276	GCA_000864365.1	Yunnan orbivirus
1620507	GCA_004128855.1	Zahedan rhabdovirus
186538	GCA_900007085.1	Zaire ebolavirus
998829	GCA_000891895.1	Zalophus californianus papillomavirus 1
1411887	GCA_000915035.1	Zamilon virus
1411887	GCA_003006235.1	Zamilon virus
1411887	GCA_003006235.2	Zamilon virus
270478	GCA_000882455.1	Zantedeschia mild mosaic virus
2382121	GCA_004117775.1	Zea mays chrysovirus 1
1642515	GCA_000989035.1	Zebra finch circovirus
1642515	GCA_004058315.1	Zebra finch circovirus
1642515	GCA_004058275.1	Zebra finch circovirus
1642515	GCA_004058335.1	Zebra finch circovirus
1642515	GCA_004058295.1	Zebra finch circovirus
1642515	GCA_004058255.1	Zebra finch circovirus
1764086	GCA_001629925.2	Zerdali virus
1764086	GCA_001629925.1	Zerdali virus
1923778	GCA_002004075.1	Zhejiang mosquito virus 1
1923779	GCA_001926635.1	Zhejiang mosquito virus 3
64320	GCA_000882815.1	Zika virus
64320	GCA_002366285.1	Zika virus
220813	GCA_000844325.1	Zinnia leaf curl virus-associated DNA beta
1810947	GCA_004128235.1	Zirqa virus
1985202	GCA_002184155.1	Zostera marina amalgavirus 1
1985203	GCA_002153845.1	Zostera marina amalgavirus 2
111418	GCA_000859845.1	Zucchini green mottle mosaic virus
83872	GCA_001876895.1	Zucchini lethal chlorosis virus
1679239	GCA_002829205.1	Zucchini shoestring virus
1406816	GCA_000915255.1	Zucchini tigre mosaic virus
12232	GCA_000861645.1	Zucchini yellow mosaic virus
253701	GCA_000854525.1	Zygocactus virus X
114871	GCA_000853105.1	Zygosaccharomyces bailii virus Z
