# Supplementary material for: Mosquito genomes are frequently invaded by transposable elements through horizontal transfer
Source: PLoS Genet. 2020 Nov 30;16(11):e1008946. doi: 10.1371/journal.pgen.1008946 (PMC7728395; doi:10.1371/journal.pgen.1008946)

TEdna analysis

To ensure that few TEs are present in the genome of *An. punctulatus* e *An. koliensis*, we download fastq files SRR1302211 (for *An. koliensis*) and SRR1302209 (for *An. punctulatus*) from SRA database. These to files were trimmed using trimmomatic-0.38 (AVGQUAL:20 MAXINFO: 50:0.5 MINLEN:20). We tested different k-mers for assembly using TEdna: 47, 51, and 57 for *An. koliensis* reads, and 61, 71 and 81 for *An. punctulatus* reads. We also tested the different threshold parameters of TEdna, which vary from 1 to 12. Using this method, we found 83 and 338 repetitive sequences in the genome of *An. punctulatus* and *An. koliensis* respectively. Only 26 and 127 sequences are putative transposable elements in *An. punctulatus* and *An. koliensis* respectively, values similar to those found in the search for homology search in the assembled genome. Most sequences found by TEdna are unknown repeats, as can be seen in figures below.

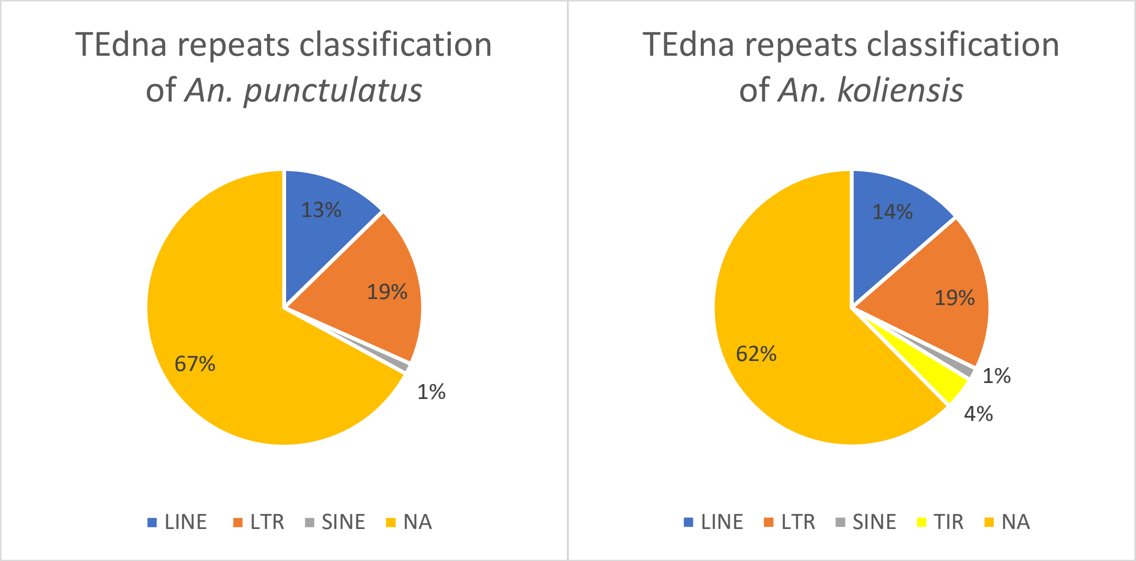

Supplement: S5 File — (PDF) [file pgen.1008946.s005.pdf]
