# Supplementary material for: Mosquito genomes are frequently invaded by transposable elements through horizontal transfer
Source: PLoS Genet. 2020 Nov 30;16(11):e1008946. doi: 10.1371/journal.pgen.1008946 (PMC7728395; doi:10.1371/journal.pgen.1008946)
Supplement: S6 File — The file shows HTT cases when we cannot differentiate HTT from an introgression, or HTT that represent removed in different genome assemblies of the same species. (PDF) [file pgen.1008946.s006.pdf]

Five low copy TE families presented HTT significant signals in VHICA analysis. However, its copies were not found in one of the new assembly version of species *An. funestus* or *An. stephensi*. Although it could be also a result from ancestral polymorphism and/or unequal TE loss in some population, we decided to remove them from the final HTT count. They are two families of Bel-Pao, two families of Gypsy, and one family of Copia. Their VHICA reports are presented below:

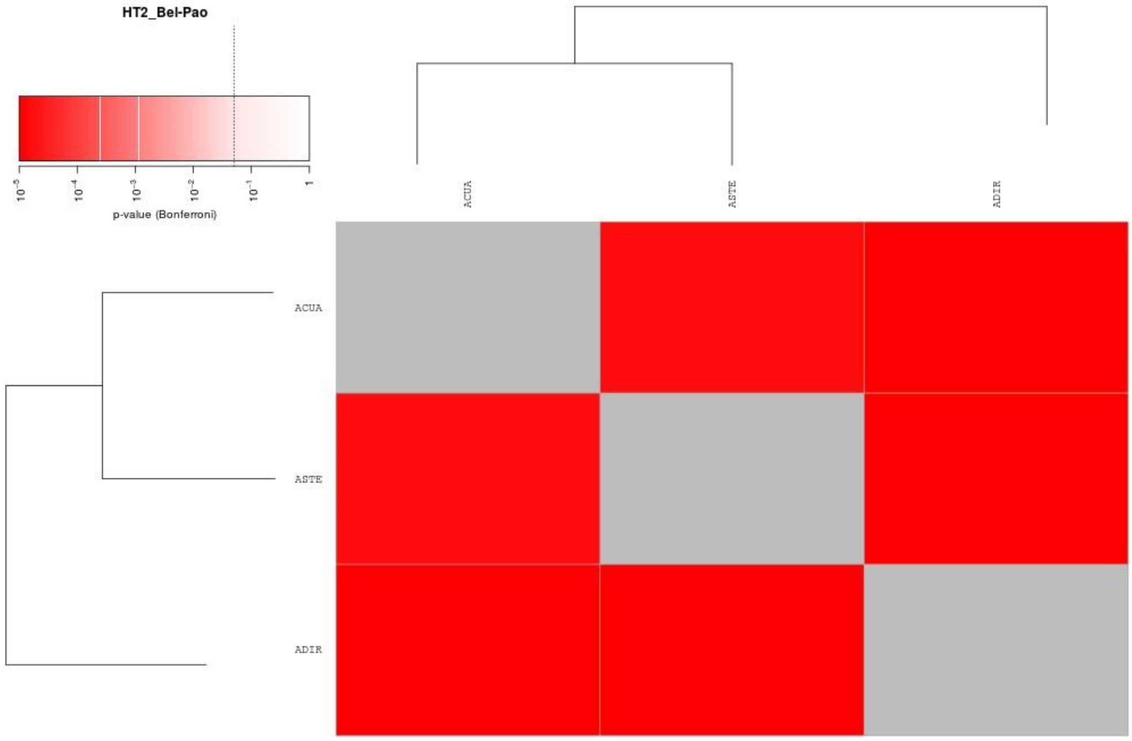

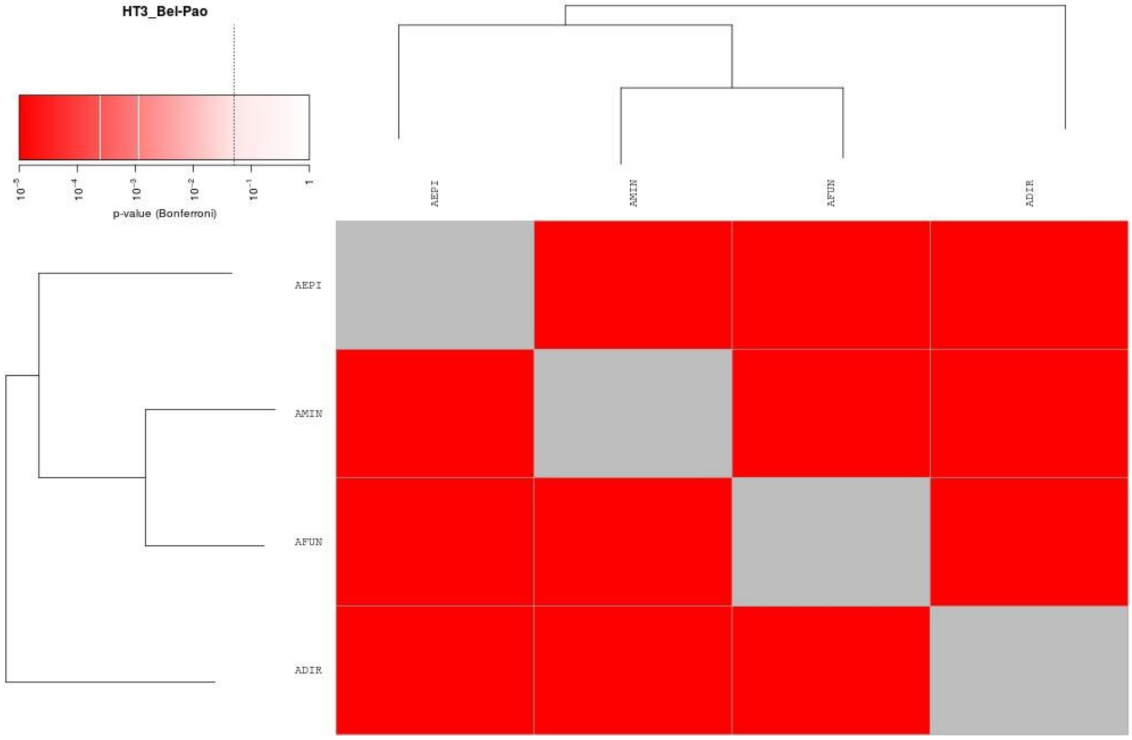

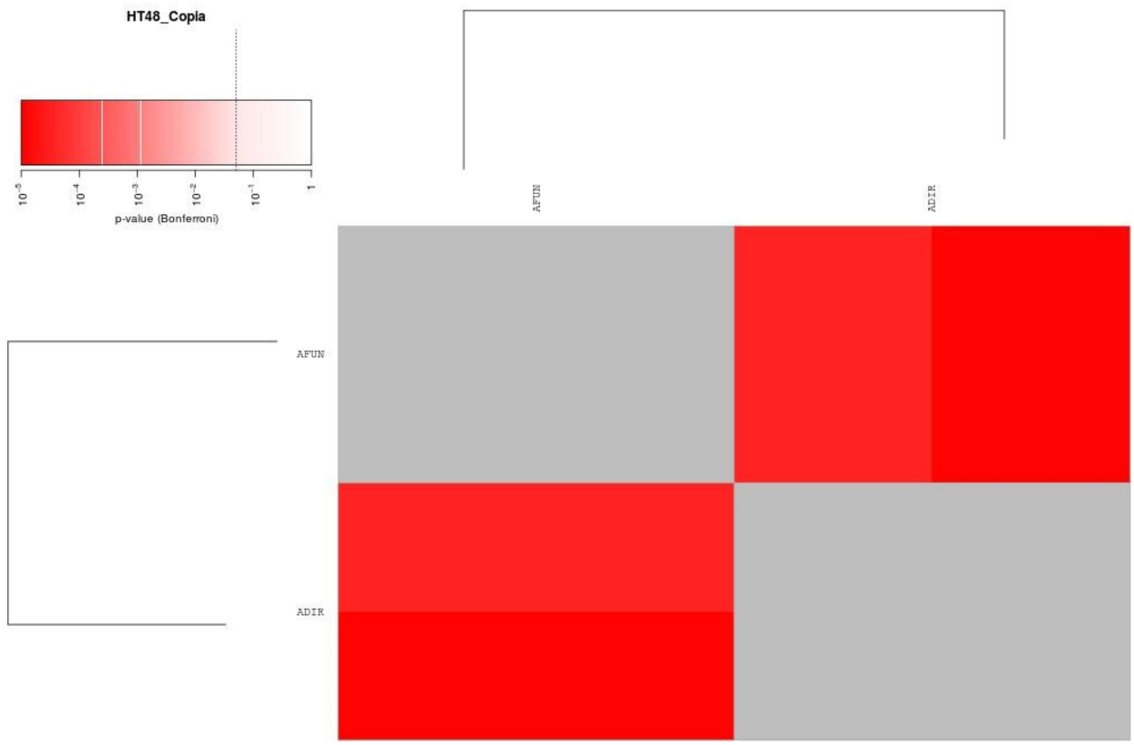
HT48_Copia
p-value (Bonferroni)
AFUN
ADIR
AFUN
ADIR

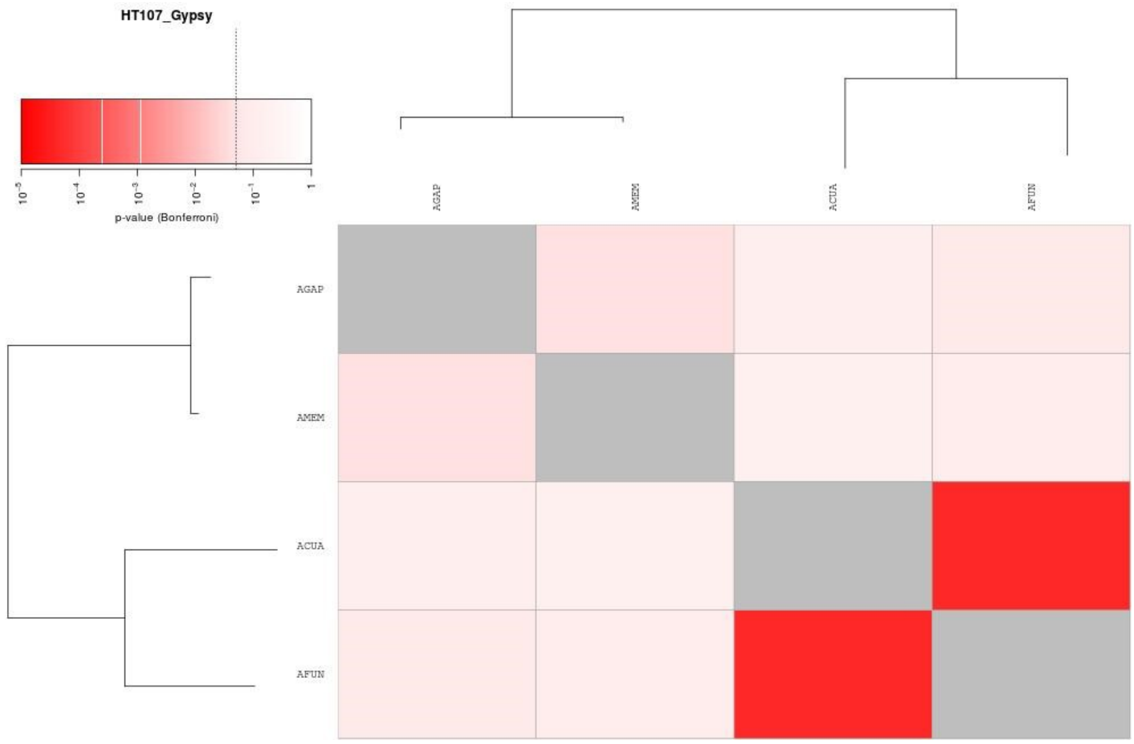
HT107_Gypsy
p-value (Bonferroni)
AGAP
AMEM
ACUA
AFUN
AGAP
AMEM
ACUA
AFUN

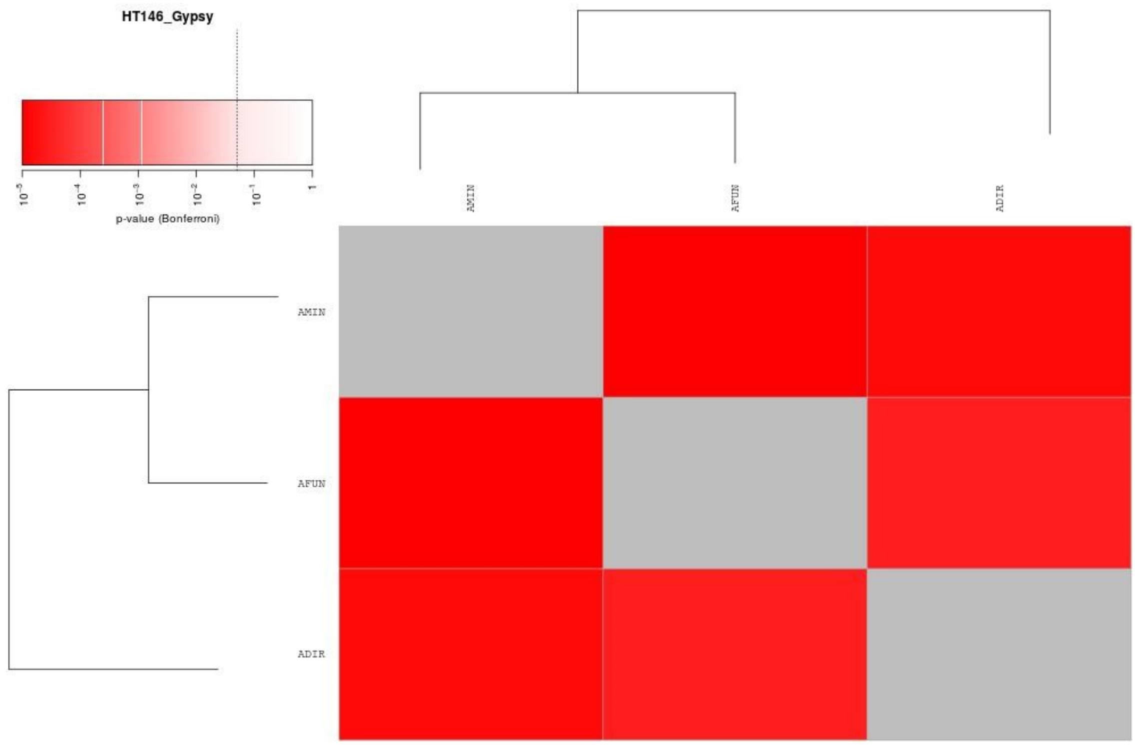

We used the comparison of the flanking region of the transposable element to differentiate a horizontal transfer event from an introgression event in species with a history of introgression. However, in nine cases, we cannot differentiate HTT from introgression, due to the small size of the flanking region (element close to the start or end of the contig) or due to the presence of NNNs within this region. Because of this, we decided to remove them from the final HTT count. Two of these families are from Bel-Pao, two from Gypsy, two from Tc1-mariner, one from CACTA, one from Helitron, and one from P superfamily. Their VHICA reports are presented below:

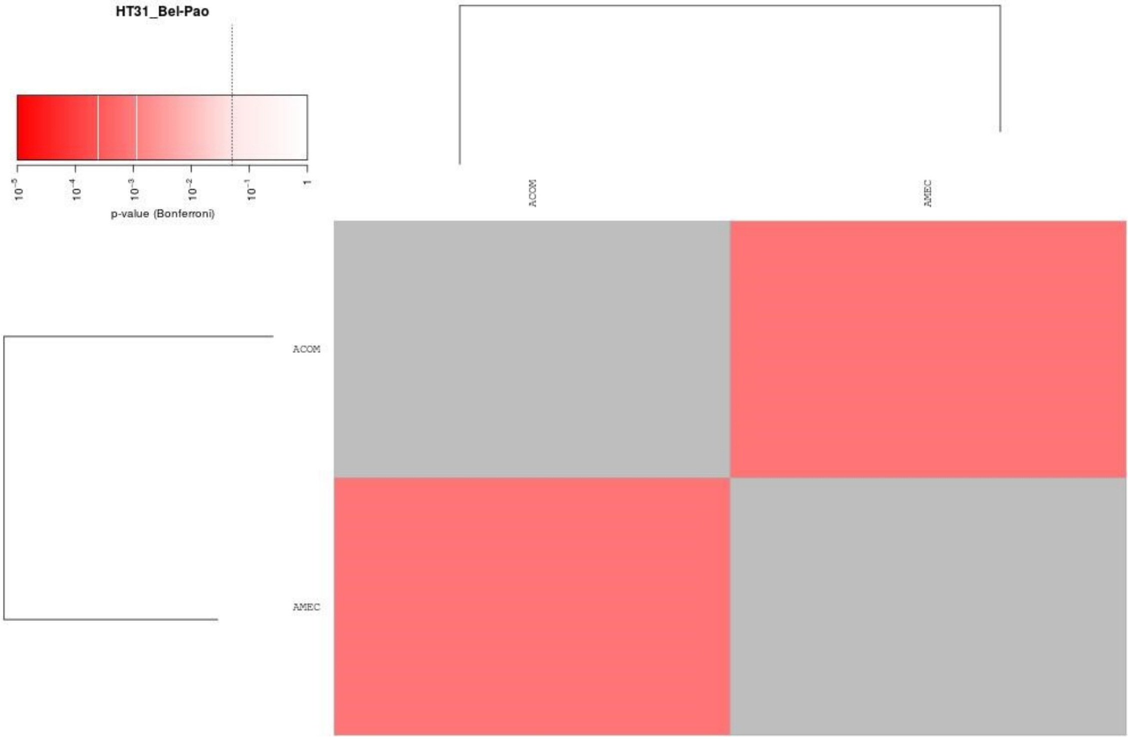
HT31_Bel-Pao
p-value (Bonferroni)
ACOM
AMEC
ACOM
AMEC

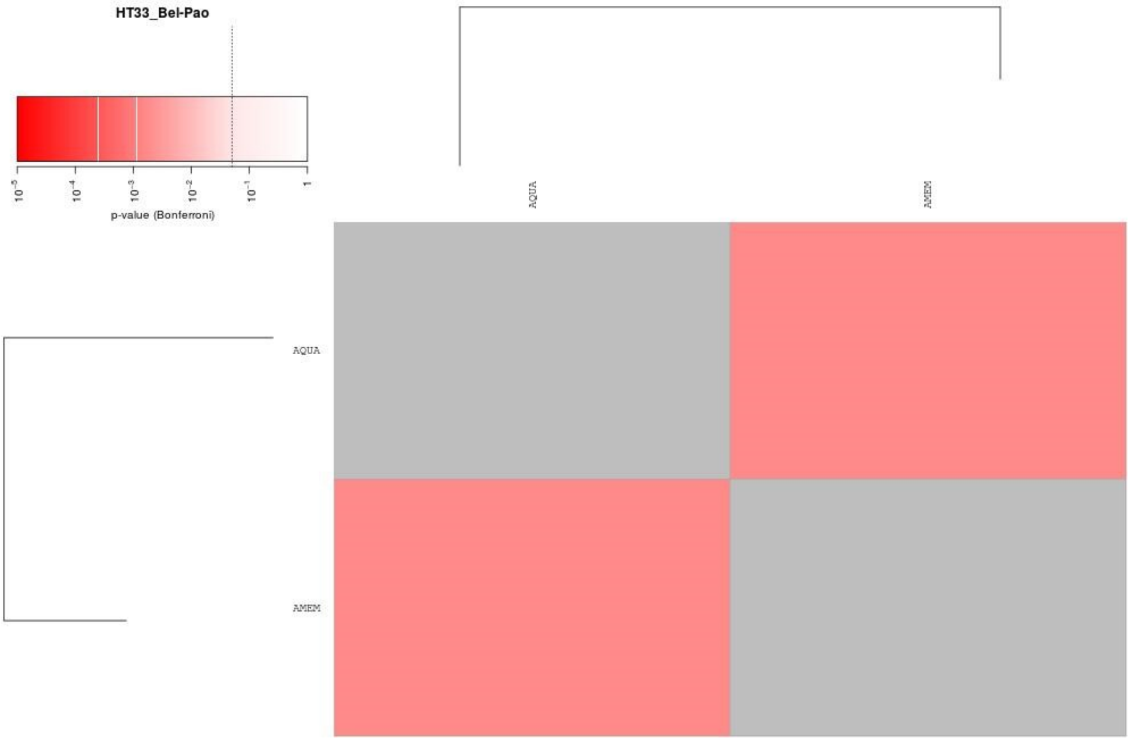
HT33_Bel-Pao
p-value (Bonferroni)
AQUA
AMEM
AQUA
AMEM

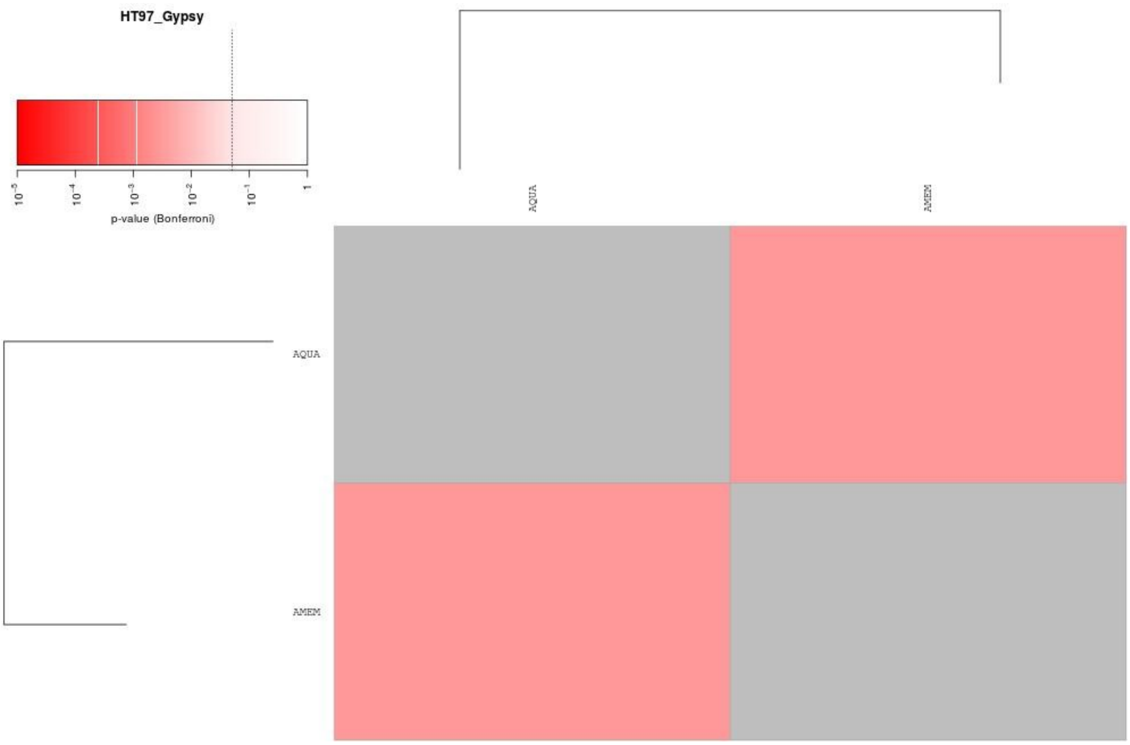
HT97_Gypsy
p-value (Bonferroni)
AQUA
AMEM
AQUA
AMEM

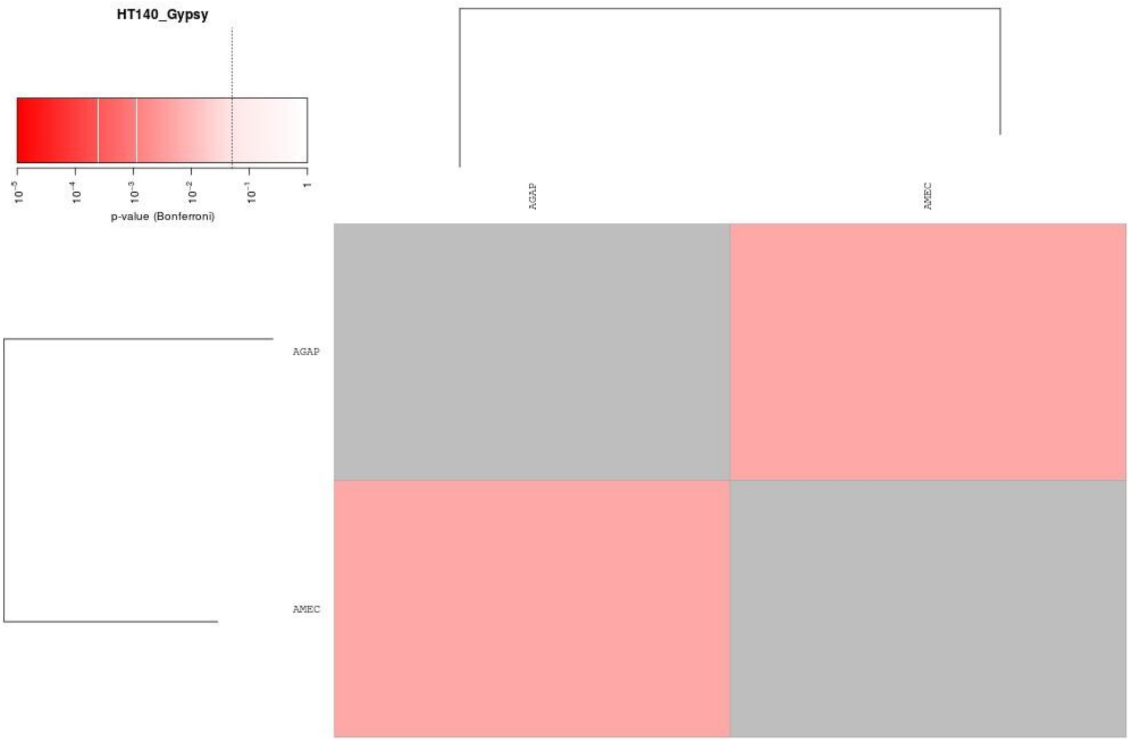
HT140_Gypsy
p-value (Bonferroni)
AGAP
AMEC
AGAP
AMEC

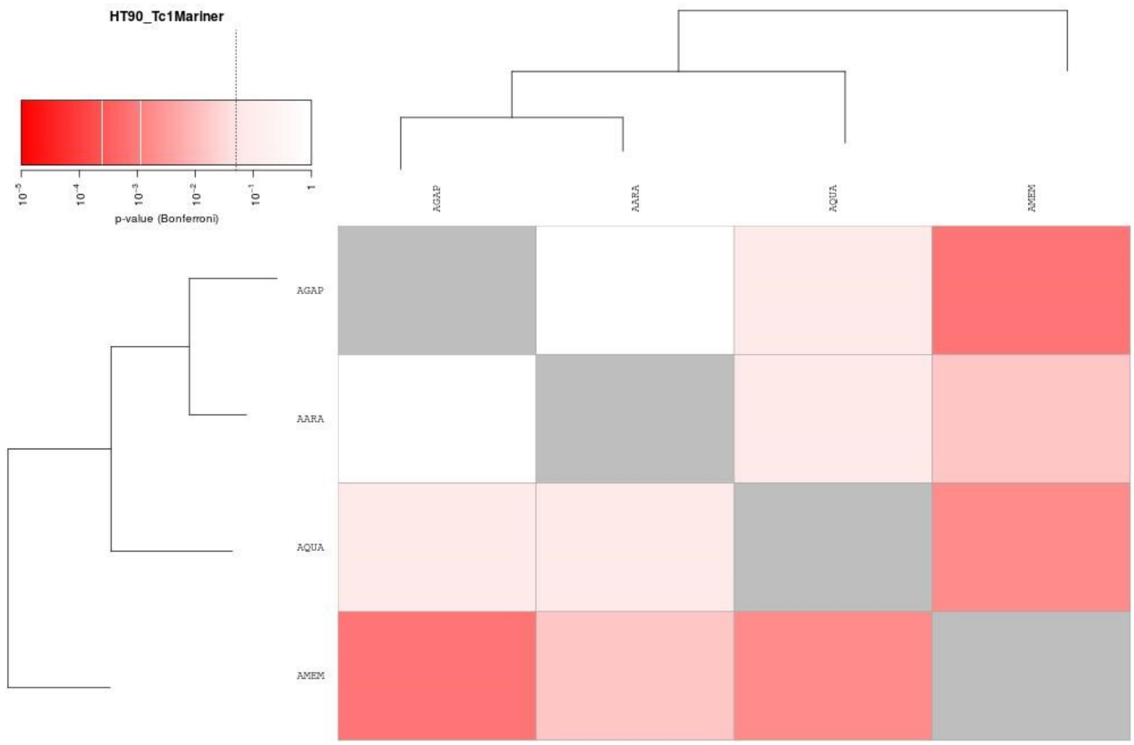
HT90_Tc1Mariner
10^{-5} 10^{-4} 10^{-3} 10^{-2} 10^{-1} 1
p-value (Bonferroni)
AGAP
AARA
AQUA
AMEM

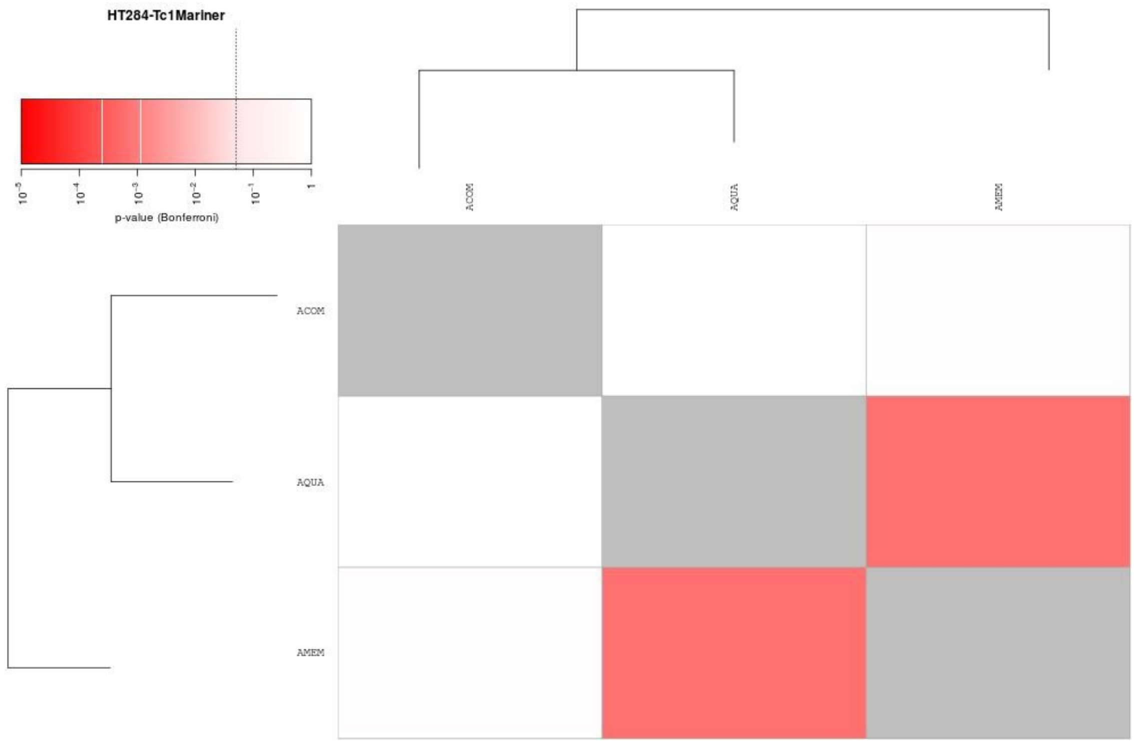
HT284-Tc1Mariner
10^{-5} 10^{-4} 10^{-3} 10^{-2} 10^{-1} 1
p-value (Bonferroni)
ACOM
AQUA
AMEM

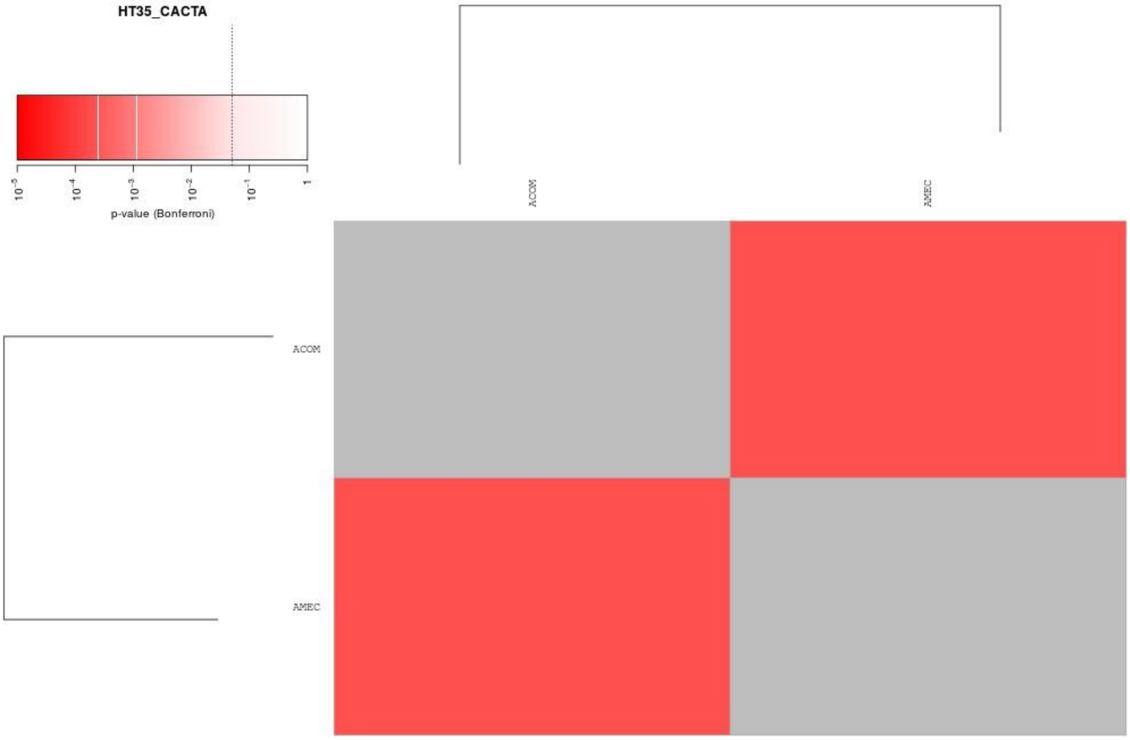
HT35_CACTA
p-value (Bonferroni)
ACOM
AMEC
ACOM
AMEC

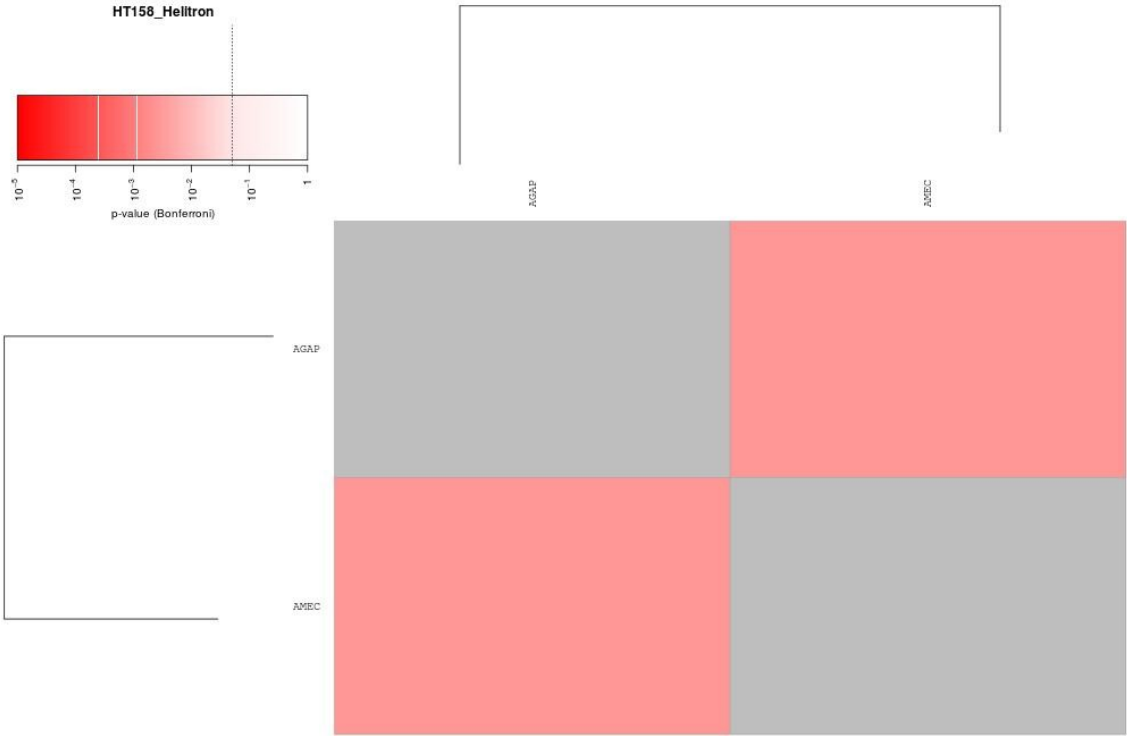
HT158_Helitron
p-value (Bonferroni)
AGAP
AMEC
AGAP
AMEC

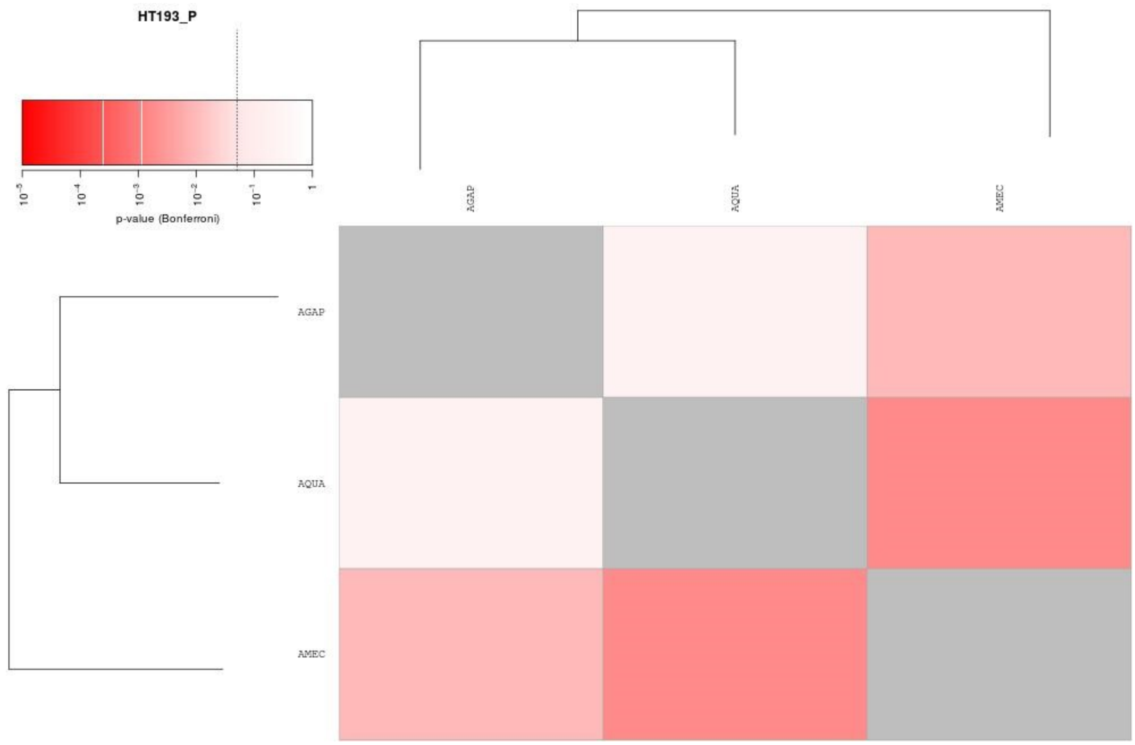

HT193_P
10⁻⁵ 10⁻⁴ 10⁻³ 10⁻² 10⁻¹ 1
p-value (Bonferroni)
AGAP
AQUA
AMEC
AGAP
AQUA
AMEC
