# Supplementary material for: Mosquito genomes are frequently invaded by transposable elements through horizontal transfer
Source: PLoS Genet. 2020 Nov 30;16(11):e1008946. doi: 10.1371/journal.pgen.1008946 (PMC7728395; doi:10.1371/journal.pgen.1008946)
Supplement: S1 Table — (DOCX) [file pgen.1008946.s008.docx]

| **S1 Table** – List of mosquito genome assemblies used in this study. | | | |  |  |
| --- | --- | --- | --- | --- | --- |
| **Species name** | **GenBank assembly accession** | **Number of contigs/scaffolds** | **Contigs/Scaffold N50 score** | **Source/Assembly** | **Coverage** |
| *Anopheles gambiae* | GCF_000005575.2 | 8 | 49,364,325 | Vectorbase/AgamP4 | - |
| *Anopheles albimanus* | GCA_000349125.2 | 201 | 37,976,048 | Vectorbase/AalbS2 | 95.0x |
| *Anopheles koliensis* | GCA_000956275.1 | *41,925* | 4,659 | NCBI/AKwgs3 | 39.0x |
| *Anopheles dirus* | GCA_000349145.1 | *1,266* | 6,906,475 | Vectorbase/AdirW1 | 184.0x |
| *Anopheles atroparvus* | GCA_000473505.1 | 1,371 | 9,206,694 | Vectorbase/AatrE1 | 98.0x |
| *Anopheles arabiensis* | GCA_000349185.1 | 1,214 | 5,604,218 | Vectorbase/AaraD1 | 100.0x |
| *Anopheles christyi* | GCA_000349165.1 | 30,369 | 9,057 | Vectorbase/AchrA1 | 35.0x |
| *Anopheles coluzzii* | GCA_000150765.1 | 10,521 | 4,437,438 | Vectorbase/AcolM1 | 8x |
| *Anopheles culicifacies* | GCA_000473375.1 | 16,162 | 22,320 | Vectorbase/AculA1 | 31.0x |
| *Anopheles darlingi* | GCA_000211455.3 | 2,220 | 115,072 | Vectorbase/AdarC3 | 20.0x |
| *Anopheles epiroticus* | GCA_000349105.1 | 2,673 | 366,526 | Vectorbase/AepiE1 | 49.0x |
| *Anopheles farauti* | GCA_000473445.2 | 310 | 12,895,223 | Vectorbase/AfarF2 | 233.0x |
| *Anopheles funestus* | GCA_000349085.1 | 1,392 | 671,960 | Vectorbase/AfunF1 | 135.0x |
| *Anopheles maculatus* | GCA_000473185.1 | 47,797 | 3,841 | Vectorbase/AmacM1 | 25.0x |
| *Anopheles melas* | GCA_000473525.2 | 20,229 | 18,103 | Vectorbase/AmelC2 | 147.0x |
| *Anopheles merus* | GCA_000473845.2 | 2,027 | 1,489,982 | Vectorbase/AmerM2 | 147.0x |
| *Anopheles minimus* | GCA_000349025.1 | 678 | 10,313,149 | Vectorbase/AminM1 | 211.0x |
| *Anopheles punctulatus* | GCA_000956255.1 | 20,774 | 10,256 | NCBI/APwgs2 | 50.0x |
| *Anopheles quadriannulatus* | GCA_000349065.1 | 2,823 | 1,641,272 | Vectorbase/AquaS1 | 93.0x |
| *Anopheles sinensis* | GCA_000441895.2 | 9,592 | 814,231 | Vectorbase/AsinC2 | 30.0x |
| *Anopheles stephensi* | GCA_000349045.1 | 1,110 | 837,295 | Vectorbase/AsteS1 | 97.0x |
| *Culex quinquefasciatus* | GCF_000209185.1 | 3,172 | 486,756 | Vectorbase/CpipJ2 | 6.14x |
| *Aedes aegypti* | GCA_000004015.3 | 4,757 | 1,547,048 | Vectorbase/AaegL3 | 7.6X |
| *Aedes albopictus* | GCA_001444175.2 | 154,782 | 201,017 | Vectorbase/AaloF1 | 229x |
