# Supplementary figures and images for: Mosquito genomes are frequently invaded by transposable elements through horizontal transfer
Source: PLoS Genet. 2020 Nov 30;16(11):e1008946. doi: 10.1371/journal.pgen.1008946 (PMC7728395; doi:10.1371/journal.pgen.1008946)

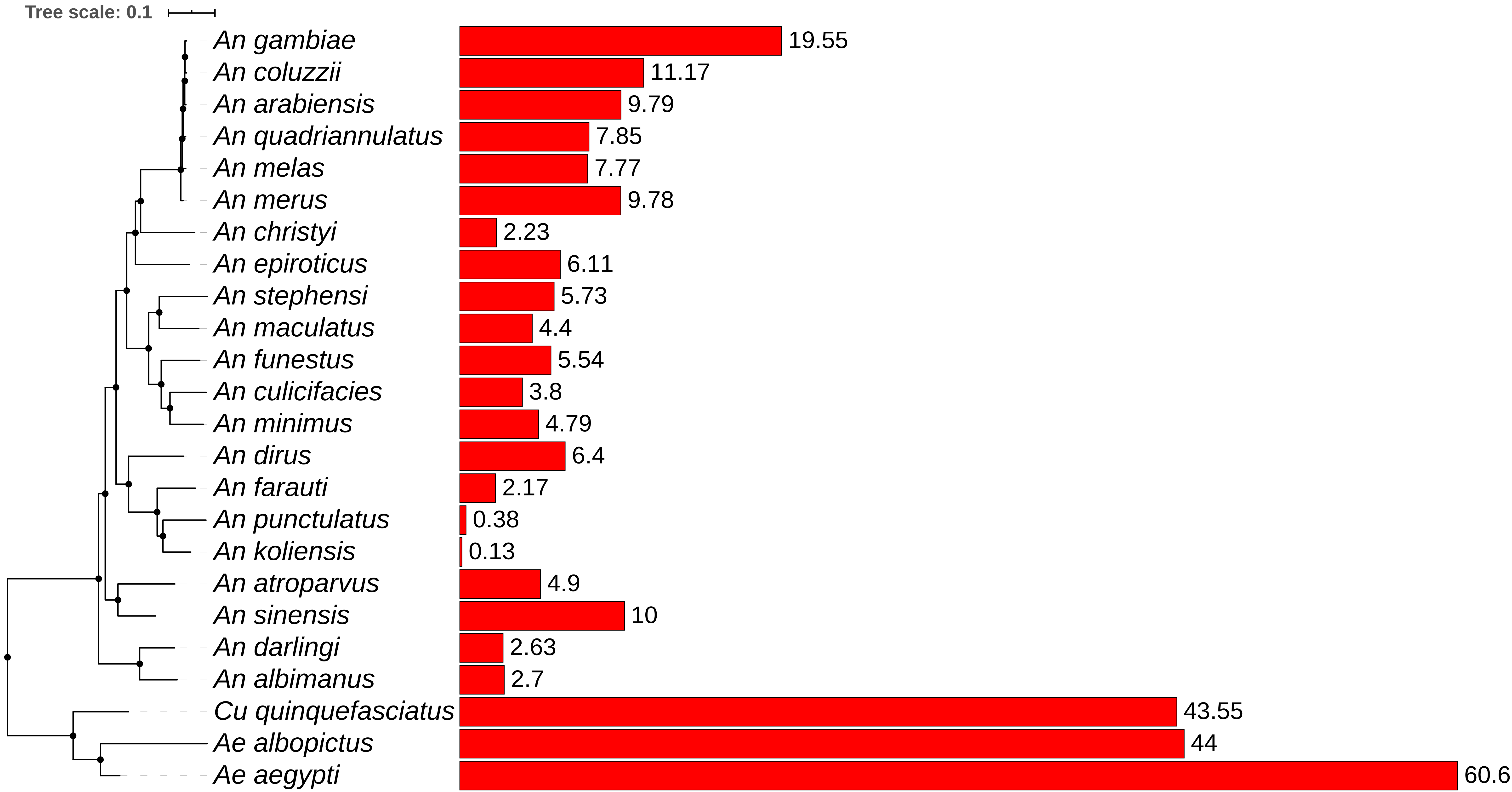

Supplement: S1 Fig — (PNG) [file pgen.1008946.s009.png]

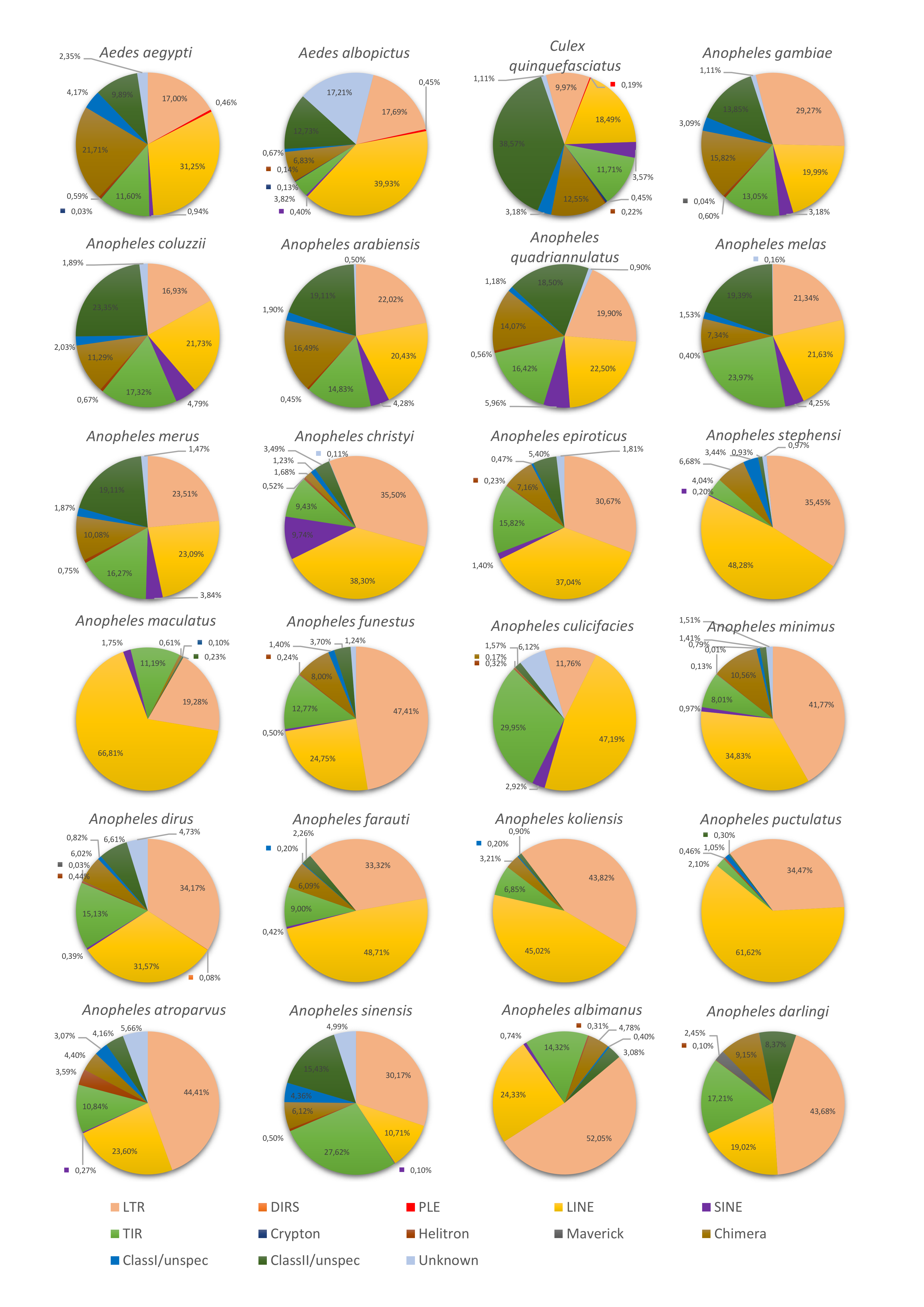

Supplement: S2 Fig — (PNG) [file pgen.1008946.s010.png]

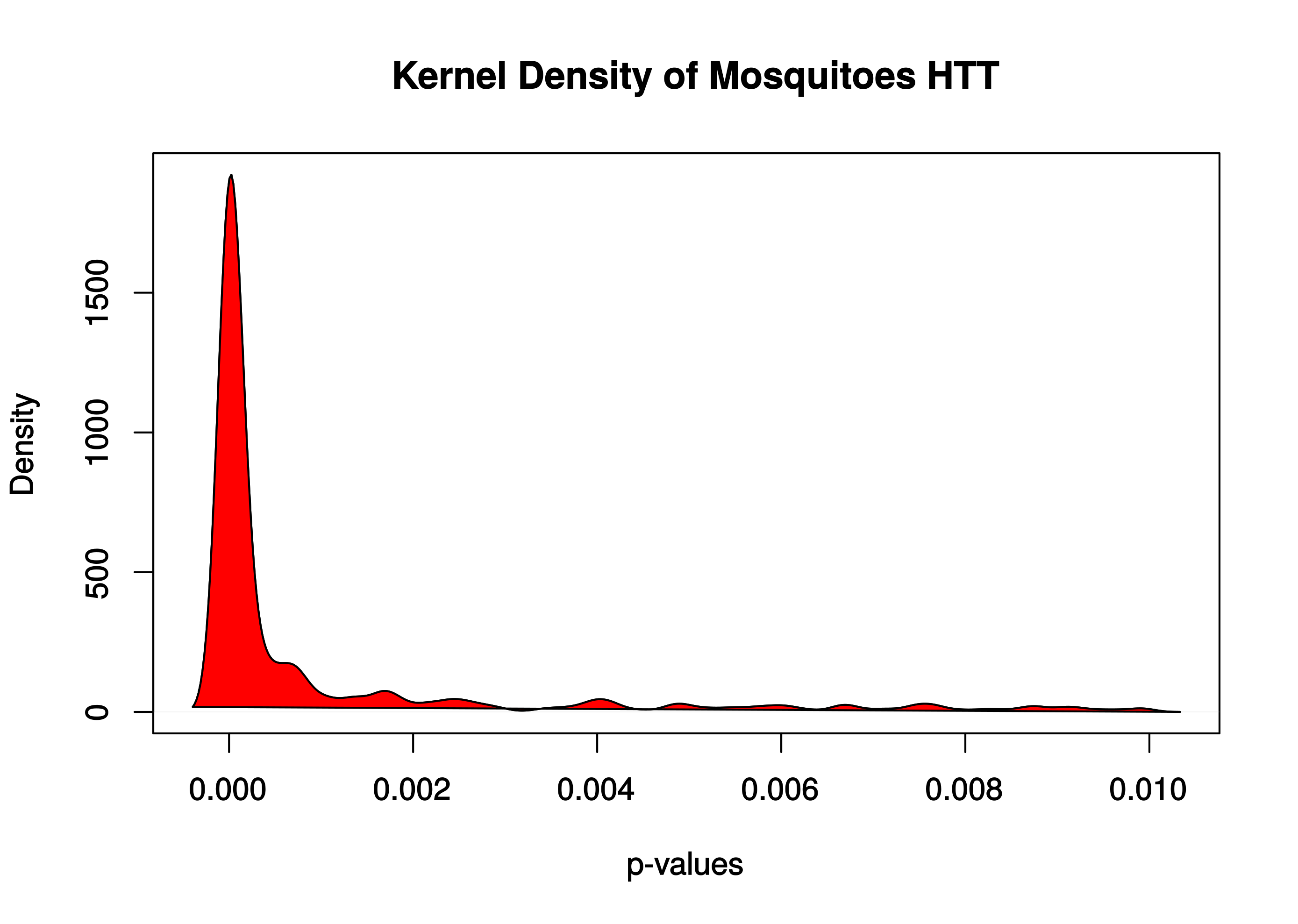

Supplement: S4 Fig — The majority of detected HTT signals are very significant. (PNG) [file pgen.1008946.s012.png]

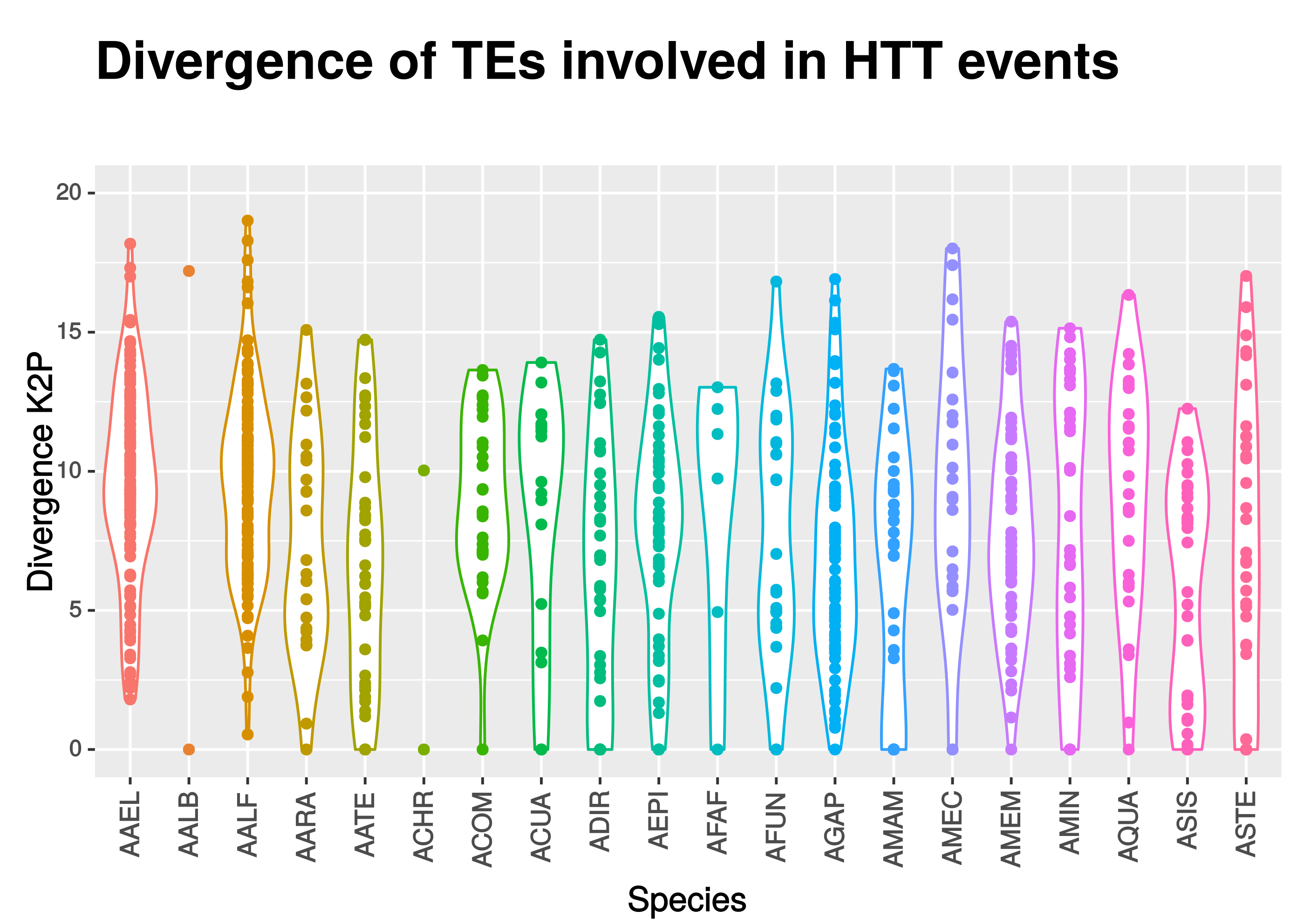

Supplement: S5 Fig — Each dot represents the relative age of each family in a mosquito genome. (PNG) [file pgen.1008946.s013.png]

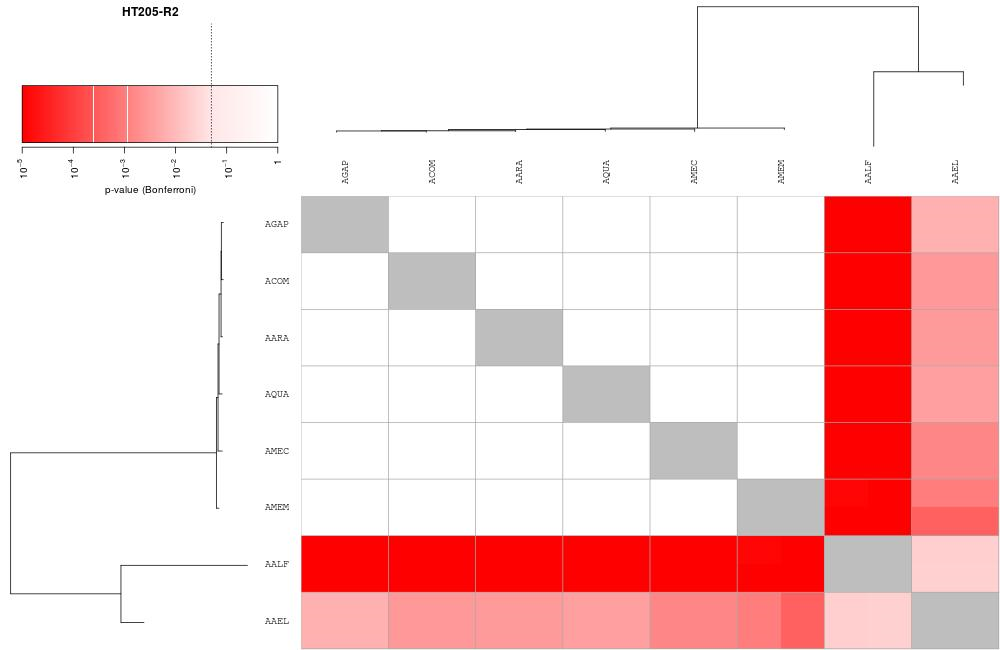

Supplement: S6 Fig — Each red square represents a significant HTT pairwise comparison. (PNG) [file pgen.1008946.s014.png]

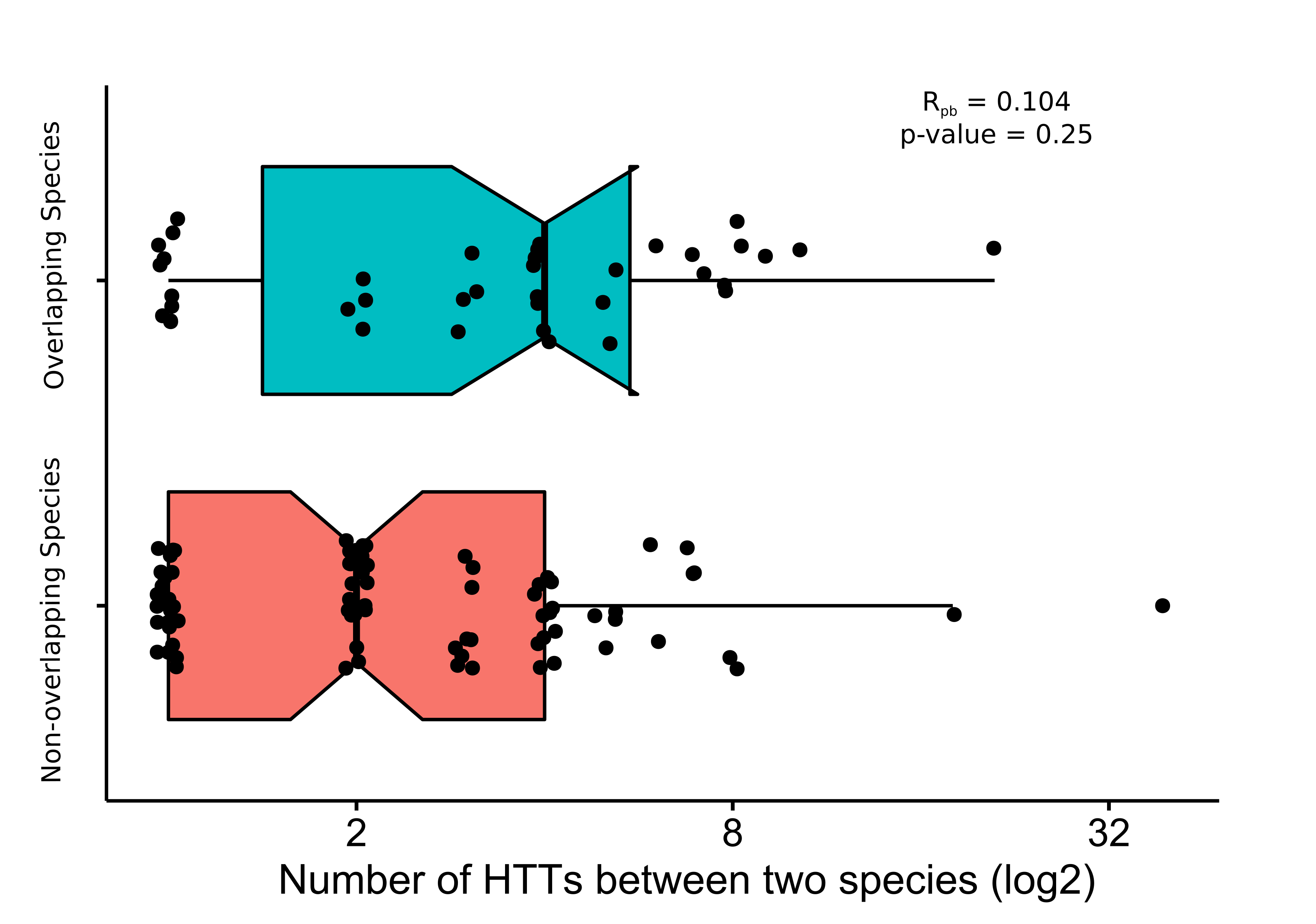

Supplement: S7 Fig — Each dot represents the number of horizontal transfers between to species. (PNG) [file pgen.1008946.s015.png]

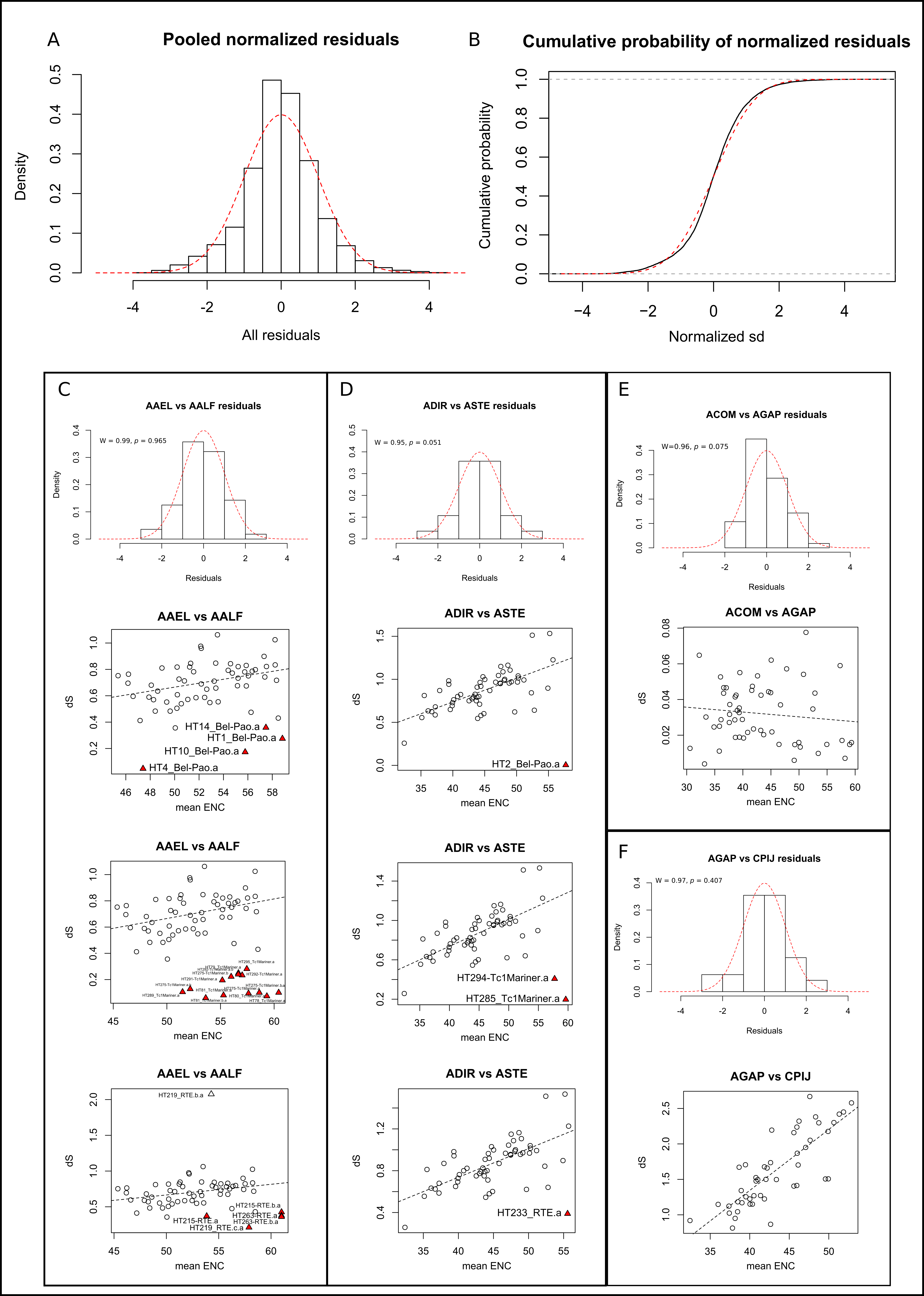

Supplement: S8 Fig — Histogram (A) and cumulative probability curve (B) showing that the distribution of pooled residuals of linear regressions are Gaussian. Histograms (C, D, E, F) of some pairwise comparison were also showed, p-values of Kolmogorov–Smirnov test of normality confirm that we cannot reject normality. Relation between codon bias and dS can also be seen. (PNG) [file pgen.1008946.s016.png]

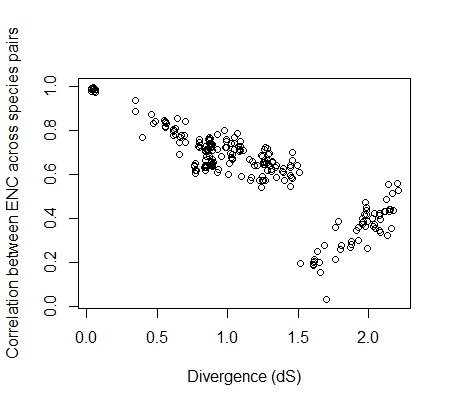

Supplement: S9 Fig — The plot shows the correlation of CUB of each pairwise comparison (Y axis) and synonymous substitution rate (X axis). (JPEG) [file pgen.1008946.s017.jpeg]
